# Gene expression analysis reveals important pathways for drought response in leaves and roots of a wheat cultivar adapted to rainfed cropping in the Cerrado biome

**DOI:** 10.1590/1678-4685-GMB-2015-0327

**Published:** 2016-10-20

**Authors:** Liane Balvedi Poersch-Bortolon, Jorge Fernando Pereira, Antonio Nhani, Hebert Hernán Soto Gonzáles, Gisele Abigail Montan Torres, Luciano Consoli, Rafael Augusto Arenhart, Maria Helena Bodanese-Zanettini, Márcia Margis-Pinheiro

**Affiliations:** 1 Departamento de Genética, Instituto de Biociências, Universidade Federal do Rio Grande do Sul, Porto Alegre, RS, Brazil; 2 Embrapa Trigo, Passo Fundo, RS, Brazil; 3 Programa de Pós-Graduação em Recursos Naturais, Universidade Federal de Roraima, Boa Vista, RR, Brazil

**Keywords:** 454 sequencing, candidate genes, RT-qPCR, Triticum aestivum, water deficit

## Abstract

Drought limits wheat production in the Brazilian Cerrado biome. In order to search for candidate genes associated to the response to water deficit, we analyzed the gene expression profiles, under severe drought stress, in roots and leaves of the cultivar MGS1 Aliança, a well-adapted cultivar to the Cerrado. A set of 4,422 candidate genes was found in roots and leaves. The number of down-regulated transcripts in roots was higher than the up-regulated transcripts, while the opposite occurred in leaves. The number of common transcripts between the two tissues was 1,249, while 2,124 were specific to roots and 1,049 specific to leaves. Quantitative RT-PCR analysis revealed a 0.78 correlation with the expression data. The candidate genes were distributed across all chromosomes and component genomes, but a greater number was mapped on the B genome, particularly on chromosomes 3B, 5B and 2B. When considering both tissues, 116 different pathways were induced. One common pathway, among the top three activated pathways in both tissues, was starch and sucrose metabolism. These results pave the way for future marker development and selection of important genes and are useful for understanding the metabolic pathways involved in wheat drought response.

## Introduction

The central part of Brazil, consisting mostly of the Cerrado biome, is considered to be the new frontier for increasing Brazilian wheat production. Although the wheat harvest in that region can reach more than 4,000 kg.ha^−1^ on average in irrigated areas (De Mori and Só e Silva, 2013), rainfed cropping has great potential to improve production once it requires little investment and has a large area for expansion. To follow this path, rainfed wheat production in the Brazilian Cerrado must cope with three major abiotic stresses: soil acidity, heat and drought (Scheeren *et al*., 2008).

Drought is broadly accepted as the most important environmental stress in agriculture and is a major constraint on plant survival, productivity and quality (Nezhadahmadi *et al*., 2013). Drought is forecast to be exacerbated by incremental increases in temperature and changes in precipitation profiles. For instance, each degree °C of increase in global mean temperature is projected to reduce global wheat grain production by approximately 6% (Asseng *et al*., 2015). In Brazil, wheat yield is theorized to be reduced up to 31% with temperature increases of 3–4 °C, offsetting the positive effects of increased CO_2_ levels on wheat grain yield (Siqueira *et al*., 2000; Streck and Alberto, 2006). Because water is largely used in irrigated agriculture (70–90% of global water use), this sector will be heavily affected by climate change (Bär *et al*., 2015). In this context, improving drought tolerance of wheat cultivars is essential for yield increases in rainfed farming.

Plants have developed several mechanisms to address drought stress, and drought tolerance is a quantitative trait with a complex response at molecular, metabolic and physiological levels (Nezhadahmadi *et al*., 2013). In wheat, several traits, such as the timing of phenological stages, carbohydrate metabolism, stomatal conductance, osmotic adjustment, late senescence of the flag leaf, flowering time, increased root:shoot ratio, high values of soluble stem carbohydrate shortly after anthesis, and increased early ground cover, among others, have been linked to the drought tolerance response (Fischer *et al*., 1998; Foulkes *et al*., 2007; Reynolds *et al*., 2007; Nezhadahmadi *et al*., 2013). To understand the mechanisms underlying this response, gene expression analysis using subtractive cDNA libraries and microarrays have been performed in wheat (Zhang *et al*., 2004; Way *et al*., 2005; Xue *et al*., 2006, 2008; Mohammadi *et al*., 2008; Ergen *et al*., 2009; Li *et al*., 2012; Reddy *et al*., 2014). However, nowadays, the most preferred technique to evaluate gene expression is high-throughput cDNA sequencing (RNA-Seq) based on next-generation sequencing technology. Up till now, the use of RNA-seq, which is not limited to the number of transcripts pre-defined in probes, to study the drought response in bread wheat (*Triticum aestivum*) has been rare (Okay *et al*., 2014; Liu *et al*., 2015; Budak *et al*., 2015). One obstacle to that type of study in bread wheat is the complexity of its hexaploid genome, which is estimated to be 17 gigabases in size and encoding more than 124,000 genes, of which approximately 76% of the assembled sequences contain repeats (IWGSC-International Wheat Genome Sequencing Consortium, 2014).

In the present study, a gene expression analysis was performed aiming at the identification of candidate genes involved in the drought responses in a wheat cultivar adapted to the Brazilian Cerrado region. A set of 4,422 candidate genes was obtained, with 2,124 specific to roots, 1,049 specific to leaves, and 1,249 sequences that were common between both tissues. A strong correlation between RNA-seq and RT-qPCR (quantitative reverse transcription polymerase chain reaction) data was observed. The importance of specific chromosome regions and genomes, as well as the most activated pathways, are reported. These results are also applied to the understanding of the metabolic pathways involved in wheat drought response.

## Materials and Methods

### Plant material, drought stress and RNA extraction

The Brazilian wheat cultivar MGS1 Aliança (*Triticum aestivum*) was used in this study due its good productivity in rainfed farming in the Brazilian Cerrado. This cultivar showed the highest yield across different sowing dates among 152 wheat genotypes tested under drought conditions in the Cerrado (Ribeiro Júnior *et al*., 2006). MGS1 Aliança was released in 1990 by EPAMIG (Empresa de Pesquisa Agropecuária de Minas Gerais) and it is still recommended for wheat production in the Cerrado (Comissão Brasileira de Pesquisa de Trigo e Triticale (2016). Seeds of MGS1 Aliança were surface-sterilized in NaClO (0.2% of active chlorine) for 1 min, washed three times with sterile distilled water (1 min each) and germinated at 23 °C in the dark for two days. Germinated seeds were transferred to pots (3 seeds per pot) containing 6.5 kg of a mixture of soil, sand and vermiculite (2:1:1) and incubated in a glasshouse with natural light at 22 ± 4 °C. Plants were watered daily. Control plants were grown for five weeks at 100% of field capacity while, in the stress treatment, plants were watered for 2 weeks at 75% of field capacity followed by 3 weeks of water deprivation. The water status of the plants was monitored by measurement of the leaf relative water content (RWC) (Barrs and Weatherley, 1962) and the water potential (Scholander pump). All three plants from one pot were pooled and the leaves and roots were collected separately, immediately frozen in liquid nitrogen, and stored at −80 °C. Total RNA was extracted with TRIzol® reagent (Invitrogen) according to the manufacturer's instructions, and purified using an RNeasy Mini Kit (Qiagen). During the purification, a DNase digestion step was performed with an RNase-free DNase Set (Qiagen). RNA quality was assessed using a Bioanalyzer (Agilent) and samples with an RIN (RNA integrity number) > 7.5 and rRNA ratio > 1.5 were used in subsequent analyses.

### 454 Sequencing

Total RNA was sent to Macrogen Inc. (South Korea) for sequencing of four libraries (control root, treated root, control leaf, and treated leaf) on a Genome Sequencer FLX Titanium instrument (Roche) according to standard protocols.

### Sequence data analysis, *de novo* assembly and functional annotation

The sequence data analysis, assembly and annotation followed the protocol available from Macrogen. Briefly, raw data were processed using the Roche GS FLX software v 2.8. The reads were assembled using GS De Novo Assembler software v 2.6. The assembly parameters were kept at default values for both the assembly and cDNA option. Singleton cleaning (elimination of contaminants, low quality, low-complexity and vectors) was performed in SeqClean (http://sourceforge.net/projects/seqclean/), with a minimum length of 100 bp and Lucy (http://lucy.sourceforge.net/). Similarity analysis was performed using BLAST (1.0e-3 cutoff) and the Gene Ontology (GO) (http://www.geneontology.org/) database to obtain sequence annotations. The data discussed in this study have been deposited in NCBI Gene Expression Omnibus (Edgar *et al*., 2002) and are accessible through GEO Series accession number GSE81833 (www.ncbi.nlm.nih.gov/geo).

### Statistical analysis

Statistical analyses of differentially expressed transcripts between the control and stressed treatments were performed with the DEGseq v 2.6 R package (http://www.bioconductor.org/packages/2.6/bioc/html/DEGseq.html), using the MARS model. Isotigs with a p-value < 0.001 were considered significantly different. The compared samples were: control leaf assembled sequences (isotigs) versus drought-stressed leaf assembled sequences (isotigs), and control root assembled sequences (isotigs) versus drought-stressed root assembled sequences (isotigs).

### CAP3 assembly, Blast2GO, genome assembly and functional annotation

A second round of assembly was performed with two aims: (1) to group sequences lacking previous significant identity that could belong to the same transcript but may have come from different genomic regions; and (2) to compare the expression of transcripts in each tissue (leaf and root). All the isotigs and singleton sequences from roots and leaves, as well as the quality sequence files, were used as input. The analysis was performed with CAP3 (Huang and Madan, 1999) software using default parameters, except for a 40 overlap length cutoff and a 90 overlap percent identity cutoff. Assembled sequences that contained one or more differentially expressed transcript in their composition and had previously been determined (by DEGseq) were considered as differentially expressed (DE) as well. These DE sequences (contigs and singletons) from CAP3 assembly were annotated using Blast2GO software (Götz *et al*., 2008) with default parameters. Blast2GO performs searches against the Gene Ontology (GO), the Kyoto Encyclopedia of Genes and Genomes (KEGG) and Interpro databases in order to determine the metabolic pathways they belong to. After annotation, the sequences obtained from the Cap3 assembly were mapped against the available Ensembl genomic sequences of *Triticum aestivum* (v. 1.26; http://plants.ensembl.org/Triticum_aestivum/Info/Index) using BWA (Li and Durbin, 2010) and SAMtools (Li *et al*., 2009) to analyze the distribution of these sequences over the wheat chromosomes and genomes. Mapping was carried out using BWA-SW "-t 6" or 6 threads. A chi-square test was used to determine if the distribution among the *T. aestivum* component genomes was statistically different. To identify transcriptions factors (TFs) encoding transcripts among the genes differentially expressed under drought, the sequences were compared by similarity search (BlastP cutoff 1e-100) against the Plant Transcription Factor Database version 3.0 (PlantTFDB) (http://www.bmicc.org/web/english/search/planttfdb) (Jin *et al*., 2014).

### RT-qPCR

Drought stress treatment was similar to the procedure described previously, except that five plants were cultivated per pot and incubated in a growth cabinet with controlled conditions (22 °C with 16/8 hours light/dark and humidity at 60%). Root and leaf samples, from a pool of five plants belonging to the same pot, were collected at two time points: after two weeks of growth and after five weeks of growth. The experiment was performed in triplicate biological samples. RNA extraction and purification were performed as described above. RNA quality and quantity were assessed using a NanoDrop 2000 Spectrophotometer (Thermo Scientific) and 1.5% agarose gels. Synthesis of cDNA was done with the Thermo Script^TM^ RT-PCR System (Invitrogen) using 2 μg of DNA-free RNA and Oligo (dT)_20_ primers. Gene-specific primers were designed using Primer3Plus (http://www.bioinformatics.nl/cgi-bin/primer3plus/primer3plus.cgi/). RT-qPCR assays were conducted in technical triplicates using a 7500 Real Time PCR System (Applied Biosystems) with 7500 Software v2.0.6. The cycles and reactions were as follows: 10 min at 95 °C, followed by 40 cycles for 15 s at 95 °C, 30 s at 60 °C, 30 s at 72 °C, and a final melting curve analysis protocol consisting of heating to 95 °C for 15 s, 60 °C for 1 min and heating to 95 °C. Reactions were performed in a final volume of 25 μL, containing 12.5 μL of SYBR^®^ Green PCR Master Mix (Applied Biosystems), 10 μL of diluted cDNA (1:100), 0.25 μL of primers (10 μM each) and 2.25 μL of water. Relative expression data analyses were performed by comparative quantification of the amplified products using the 2^−ΔΔCT^ method (Schmittgen and Livak, 2008). The reference genes used for normalization of expression were those encoding ATPase, Ribosylation Factor, RNAseL (Paolacci *et al*., 2009), Ta10105, Ta14126 and Ta27922 (Long *et al*., 2010). The geNorm v3.5 software (http://medgen.ugent.be/~jvdesomp/genorm/) was used to select the two best reference genes for the respective experimental condition.

## Results

### Sequencing analysis

In order to search for candidate genes and metabolic pathways associated to drought stress in wheat, high-throughput sequencing was done using 454 sequencing technology with cDNA originating from drought-stressed and control roots and leaves. When harvested, the mean values for leaf water potential and for RWC were, respectively, −0.38 MPa and 98% in the control plants and −2.12 MPa and 50.1% in the stressed plants, indicating that, based on the parameters detailed by Hsiao (1973), the treated plants were severely drought-stressed. The sequencing analyses yielded 1,225,527 reads from the four libraries (control and treated roots, control and treated leaves). Among these, 305,731 reads were obtained for the root control sample and 300,665 for roots under drought stress. From the total 606,396 reads, 453,218 reads (74.7%) were fully assembled and 32,085 isotigs were identified, with an average size of 1,085 bases and an N50 of 1,299. Fifteen percent (90,933 reads) were partially assembled, and 6.6% (40,377 reads) remained as singletons, with 37,457 reads considered valid. Additionally, 17,257 reads were anchored to repeat regions, 4,179 were considered outliers and 345 were too short to be used in the computational analysis (Table 1).

**Table 1:** Analyses of the reads obtained from the four libraries (root control, root stressed, leaf control, and leaf stressed)

Reads	Root	Leaf
Number of reads	606,396	619,131
Number of bases	366,686,703	345,617,595
Reads in control condition	305,731	319,997
Number of bases in control condition	184,485,642	177,132,266
Average read length	603,425	553,544
Reads in drought condition	300,665	299,134
Number of bases in drought condition	182,201,061	168,485,369
Average read length	605,994	563,244
Fully assembled	453,218	519,150
Partially assembled	90,933	65,475
Singletons	40,377	31,374
Repeat	17,257	189
Outlier	4,179	2,806
Too short	345	135
Number of isogroups	24,900	16,585
Average contig count	1.5	1.4
Number of isogroups with one isotig	20,969	14,592
Number of isotigs	32,085	19,899
Average isotig size	1085,508	952.03
N50 isotig size	1,299	1,115
Valid singletons	37,457	28,880

Isogroup is the collection of contigs containing reads that imply connections between them. Isotig is analogous to an individual transcript.

Regarding the leaf-derived sequences, 619,131 reads were used in the assembly computation (319,997 from leaves in control sample and 299,134 from leaves under drought stress). From the total, 519,150 reads (83.8%) were fully assembled and 19,899 isotigs were identified, with an average size of 952 bases and an N50 isotig size of 1,115. Approximately 10% of the reads (65,475 reads) were partially assembled and 5% (31,374 reads) were singletons, with 28,880 reads considered valid. Furthermore, 189 reads anchored to repeat regions, 2,806 were considered outliers and 135 were too short to be used in the computational analysis (Table 1).

### Search for candidate genes

After assembly and annotation, we searched for candidate genes differentially expressed between control and treated samples. The homogeneous distribution of the four libraries is presented in Figure S1. A total of 4,422 candidate genes was identified in both tissues (p < 0.001) (Table S1). Among those, 2,808 isotigs were obtained from roots, with 1,100 up-regulated and 1,708 down-regulated isotigs under stress conditions. Statistical analysis showed that 1,614 isotigs in leaves were significantly different (p-value < 0.001). Up-regulation occurred in 1,017 isotigs, while down-regulation was observed in 597.

**Figure 1:**
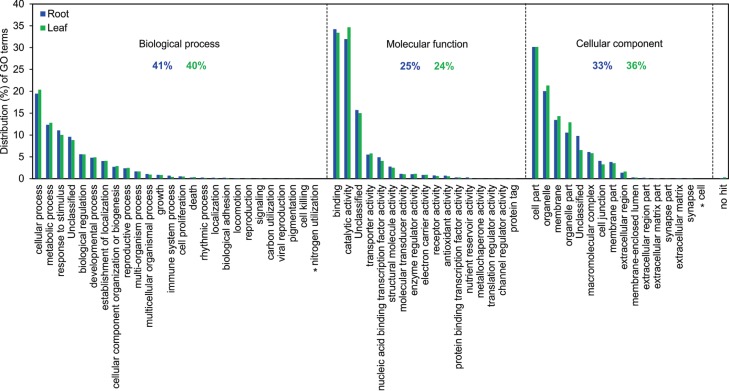
Functional annotation of the 4,422 candidate genes detected in root and leaf tissues of the wheat cultivar MGS1 Aliança under drought stress. Gene Ontology (GO) analysis was performed for three main categories (biological process, molecular function and cellular component). Asterisk indicates GO terms present in root tissue only. No-hit sequences correspond to 0.1% and 0.3% of the leaf and root sequences, respectively. Note that a sequence may have multiples terms associated to it.

Gene Ontology (GO) categories of the candidate genes are shown in Figure 1. The functional annotation of the root and leaf isotigs revealed that 41% and 40% of the sequences were, respectively, involved in biological process, 25% and 24% in molecular function, 33% and 36% were cellular components, while the remaining sequences were no-hits. The comparison of GO terms among the four main categories revealed that the distribution of candidate genes was similar between root and leaf. Among the sequences annotated in biological processes, cellular and metabolic processes were highly represented. Among molecular functions, sequences related to binding and catalytic activity were the most represented GO terms. Regarding cellular component, the most represented category was cell part.

### Expression profile validation

For validation of the gene expression analysis, a second and independent experiment was performed where plant samples were collected after two and five weeks of growth. For the two-week-old plants, mean leaf water potential and RWC values were −0.37 MPa and 96.7% for the control plants and −0.39 MPa and 95.8% for the treatment. After five weeks of growth, the mean values for leaf water potential and RWC were −0.42 MPa and 95.6% for the control plants and −2.04 MPa and 54.6% for the drought-stressed plants. This indicates that the plants had a similar water status before water was withheld but a different status after five weeks of growth. The analyses of the expression profile in two-week-old control (just before the irrigation withholding) as well as in five-week-old control and treated plants, allowed for the comparison of candidate gene expression not only after the drought period but also before the stress.

**Figure 2:**
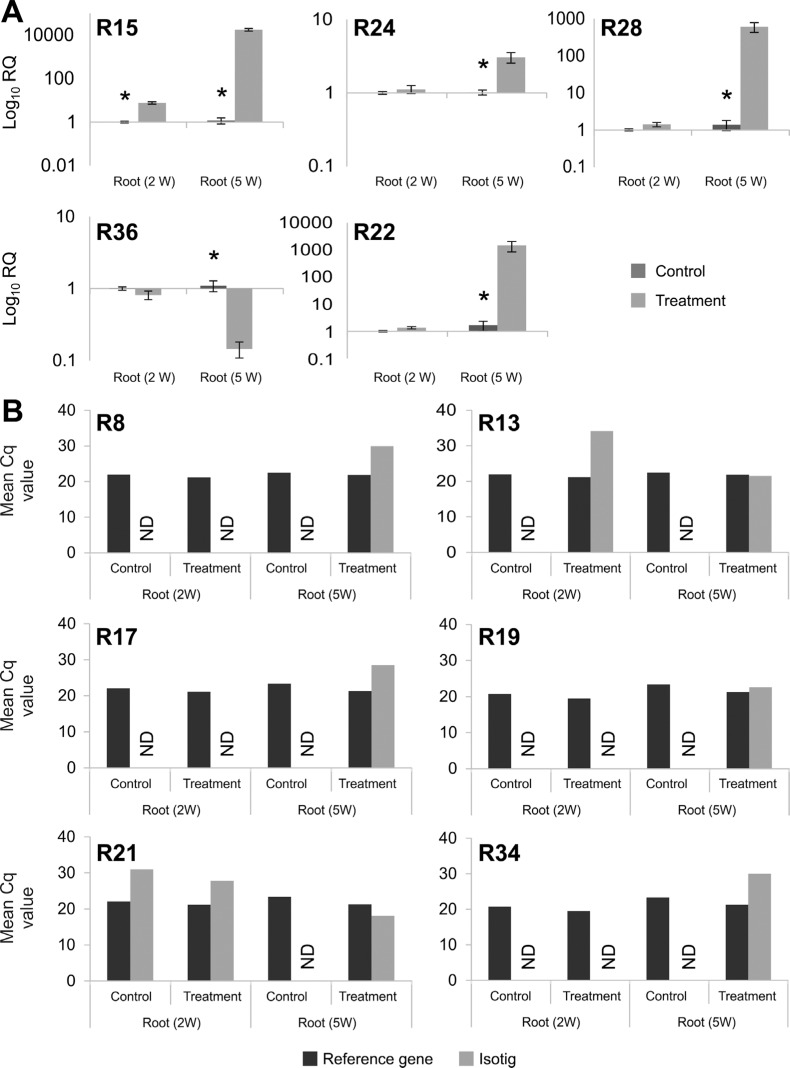
RT-qPCR analysis of selected root transcripts. Expression is shown for 11 transcripts that were validated by RT-qPCR. Wheat roots were sampled from two-week-old (2 W) plants, where control plants were irrigated at 100% of field capacity (FC) and treated plants were irrigated at 75% FC. Five-week-old (5 W) plants are represented by control plants (irrigated at 100% FC) and treated plants, where irrigation was withdrawn for three weeks. The experiment was performed in triplicate biological samples and in technical triplicates. (A) Relative expression was calculated by the 2^−ΔΔct^ method. Asterisks represent significantly different (Student's *t*-test, p ≤ 0.05) transcript levels. (B) Mean of the Cq value. "ND" means not detected. For more details on primers see Table 2 and Table S2.

**Figure 3:**
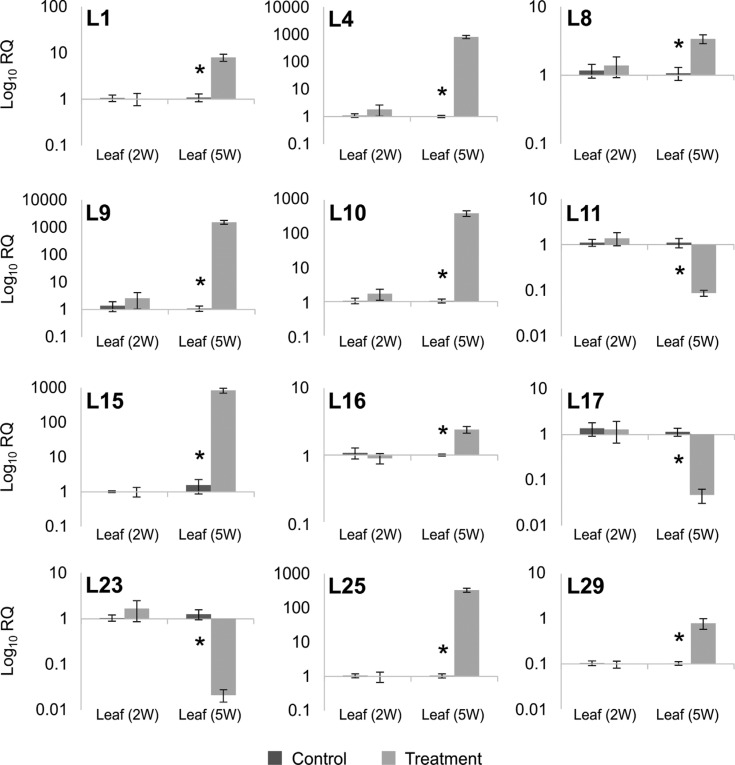
RT-qPCR analysis of selected leaf transcripts. Expression is shown for the 12 transcripts that were validated by RT-qPCR. Wheat leaves were sampled from two week-old (2 W) plants, where control plants were irrigated at 100% of field capacity (FC) and treated plants were irrigated at 75% FC. Five-week-old (5 W) plants are represented by control plants irrigated at 100% FC, and treated plants, where irrigation was withdrawn for three weeks. The experiment was performed in triplicate biological samples, with technical triplicates for each. Relative expression was calculated by the 2^−ΔΔct^ method. Asterisks represent significantly different (Student's *t*-test, *p* ≤ 0.05) transcript levels. For more details on primers see Table 2 and Table S2.

The relative expression of 15 root- and 20 leaf-derived transcripts (Table S2) was measured by RT-qPCR for experimental validation of the RNA-seq data. These 35 transcripts were chosen for validation because they showed different levels of expression (up- or down-regulated), are associated to different enzymes from the same pathway or belong to different metabolic pathways (Table S1). The expression of four root isotigs was not validated because the control and the drought-stressed samples after five weeks of growth were statistically similar. On the other hand, the expression of five root isotigs (R15, R24, R28, R36 and R22) were significantly different between treated and control plants after five weeks of growth (Figure 2A). When comparing the samples collected from two-week-old plants, only the R15 sequence was significantly different between treatments. For the other six isotigs, expression in control samples was not detected, but was detected in the drought-treated samples, indicating that their expression changed in response to water deprivation. Because of this change, the mean Cq values are presented (Figure 2B). A set of 20 transcripts from leaves was also evaluated by RT-qPCR. The expression of 12 isotigs (L1, L4, L8, L9, L10, L11, L15, L16, L17, L23, L25 and L29) were significantly different between the control and treated samples of five-week-old plants (Figure 3). Excluding the six root sequences with non-detected Cq values, the Pearson's correlation between the RNA-seq and RT-qPCR data for the other 29 transcripts was 0.78 (Figure S2).

### Genome localization and tissue-specificity of the candidate genes

**Figure 4:**
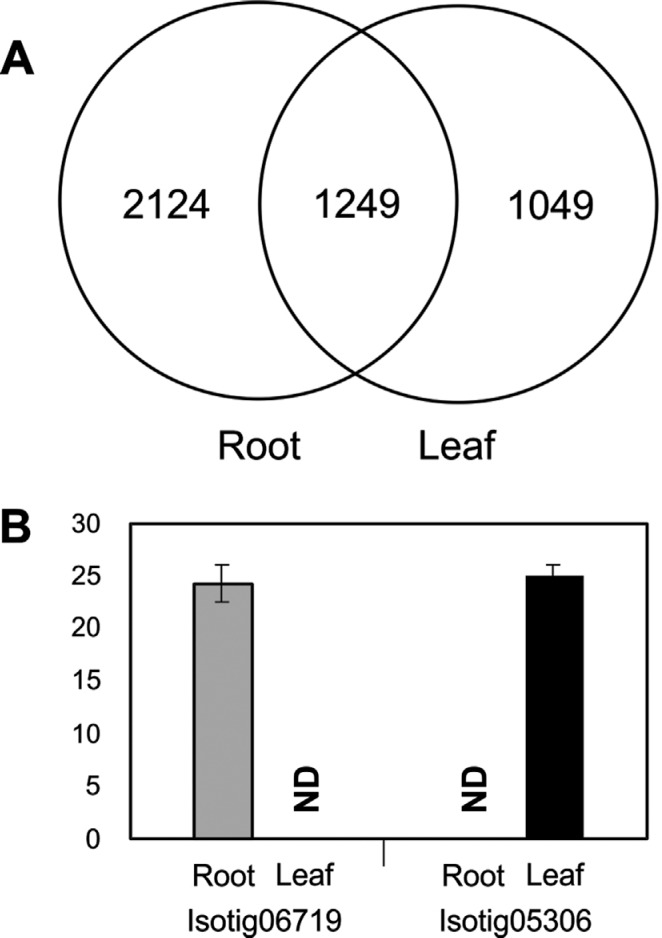
Root or leaf specific candidate genes identified in response to drought stress in the wheat cultivar MGS1 Aliança. (A) Venn diagram representing the number of candidate genes obtained after DEGseq analysis. The candidate genes are distributed in root and leaf according to CAP3 assembly. (B) RT-qPCR analysis of two randomly selected specific transcripts, showing that isotig06719 (see primer sequences on Table S2) is specifically expressed in root tissue and the isotig05306 (primers CCGTGTCACTTCCCTTGATT and GGAGAGGTTGAGATGGGTGA) is expressed in leaves only. "ND" means not detected.

After comparing the candidate genes between root and leaf samples (2,808 and 1,614, respectively), 2,124 sequences were found to be specifically expressed in roots, 1,049 specifically in leaves, and 1,249 sequences were common to both tissues (Figure 4A). One sequence specific for each tissue (isotig06719, the same as the one used to design the primer R33 listed on Table S2), and isotig05306 (annotated as "AT1G47890 - defense response - kinase activity") for root and leaf, respectively, were used for RT-qPCR analyses. The positive amplification of these sequences in specific tissues (Figure 4B) corroborates our *in silico* analysis. Searches against KEGG failed to detect pathways for these specific sequences.

**Figure 5:**
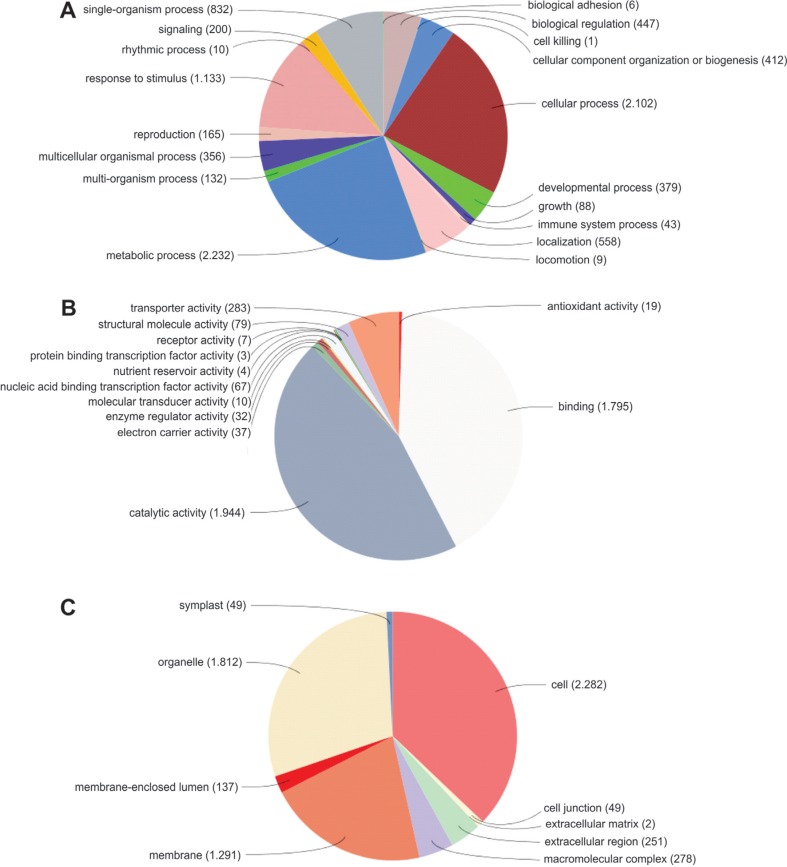
Pie diagrams demonstrating the percentage of contigs and singlets generated in CAP3 within the functional categories of Gene Ontology. Results are based on Blast2GO data mining. (A) Biological process, (B) molecular function, and (C) cellular component.

An additional assembly (performed with the CAP3 software) allowed for the comparison of transcript expression between the two tissues. A total of 118,321 sequences were used (32,085 isotigs and 37,457 singletons from roots and 19,899 isotigs and 28,880 singletons from leaves). After the assembly, 11,746 contigs and 69,407 singlets were obtained with 1,393 and 2,594, respectively, considered as differentially expressed and, consequently, as candidate genes. The 3,987 candidate genes (1,393 contigs and 2,594 singlets) were analyzed for functional annotation, with 96.4% of sequences annotated and 4.5% showed mapping results (Figure S3).The highest similarity rate corresponded to sequences from *Aegilops tauschii* (30%), followed by *Hordeum vulgare* (29.5%), *Triticum urartu* (15%), *T. aestivum* (13%) and *Brachypodium distachyon* (5.5%) (Figure S3). With regards to the GO distribution of the sequences assembled by CAP3 (Figure 5), the categories with the most abundant sequences in biological processes were metabolic processes, cellular processes, response to stimulus, single-organism processes, localization and biological regulation; for molecular function the most prevalent categories were catalytic activity and binding; and for cellular components the categories were cell, organelle and membrane.

**Figure 6:**
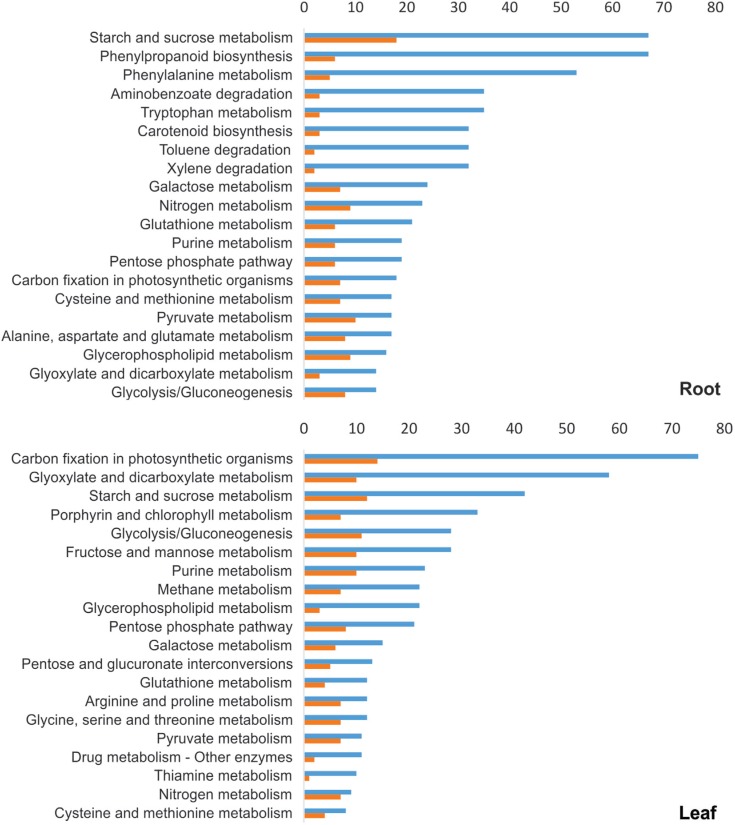
Top 20 biological pathways of root and leaf tissues activated in the wheat cultivar MGS1 Aliança under drought conditions. Sequences were analyzed by Blast2GO against the Kyoto Encyclopedia of Genes and Genomes (KEGG). Blue bars represent the number of sequences and orange bars represent the number of enzymes.

To identify biological pathways that are active in wheat drought response, the 3,987 candidate genes described above were analyzed using Blast2GO software against KEGG pathways. The results revealed 116 different pathways (Table S3) involved in wheat drought response. The top 20 pathways (with the highest number of sequences) for root and leaf tissues are presented in Figure 6. Among the top 20 pathways in both tissues, 28 different pathways were detected, with 12 pathways in common but ranked in different positions. Starch and sucrose metabolism pathway-related transcripts had the highest ranking in roots but were the third most commonly identified ones in leaves. In addition, the number of sequences for the fructose and mannose pathways was 3.5 times higher in leaves, while the arginine and proline metabolism pathway presented a similar number of sequences for both tissues. We also analyzed putative pathways related to the 22 up-regulated sequences with annotation that were tested by RT-qPCR (Table S2). Among the 22 transcripts, 10 generated results when searched against KEGG, revealing 13 different pathways (Table 2). With the exception of L4, whose function was not linked to a specific pathway, all isotigs remained in the same enzyme classes and pathways as identified before the assembling with CAP3.

**Table 2:** Biological pathways related to 10 sequences validated by RT-qPCR and the respective contigs where they assemble in CAP3.

Primer*^1^	Pathway*^2^	#Seqs	#Enzs
R8	Fructose and mannose metabolism	Contig607LR	ec:1.1.1.21 - reductase
R8	Galactose metabolism	Contig607LR	ec:1.1.1.21 - reductase
R8	Glycerolipid metabolism	Contig607LR	ec:1.1.1.21 - reductase
R8	Glycosphingolipid biosynthesis - globoseries	Contig607LR	ec:3.2.1.22 - melibiase
R8	Pentose and glucoronate interconversions	Contig607LR	ec:1.1.1.21 - reductase
R8	Pyruvate metabolism	Contig607LR	ec:1.1.1.21 - reductase
R24	Arginine and proline metabolism	Contig3704LR	ec:2.7.2.11 - 5-kinase; ec: 1.2.1.41 - dehydrogenase
R28	Galactose metabolism	Contig575LR	ec:2.4.1.82 - galactosyltransferase, ec:3.2.1.22 - melibiase
R28	Glycerolipid metabolism	Contig575LR	ec:3.2.1.22 - melibiase
R28	Sphingolipid metabolism	Contig575LR	ec:3.2.1.22 - melibiase
L4	Purine metabolism	Contig506LR	ec:3.6.1.3 - adenylpyrophosphatase
L8	Arginine and proline metabolism	Contig5346LR	ec:1.2.1.19 - dehydrogenase
L8	Beta-Alanine metabolism	Contig5346LR	ec:1.2.1.19 - dehydrogenase
L8	Glycine, serine and threonine metabolism	Contig5346LR	ec:1.2.1.8 - dehydrogenase
L9	Pyruvate metabolism	Contig178LR	ec:1.1.1.38 - dehydrogenase (oxaloacetate-decarboxylating)
L10	Starch and sucrose metabolism	Contig472LR	ec:2.4.1.12 - synthase (UDP-forming)
L15	T cell receptor signaling pathway	Contig149LR	ec:3.1.3.16 - phosphatase
L25	Arginine and proline metabolism	Contig132L	ec:2.7.2.11 - 5-kinase; ec: 1.2.1.41 - dehydrogenase
L29	Arginine and proline metabolism	Singlet	ec:2.7.2.11 - 5-kinase; ec: 1.2.1.41 - dehydrogenase

Analysis was performed with Blast2GO against the Kyoto Encyclopedia of genes and Genomes (KEGG). #Seqs means the number of sequences in that pathway; #Enzs indicates the number of enzymes corresponding to the sequences.

*1 One primer can correspond to more than one pathway.

*2 Alphabetical order based on the primer name. See more detail of the primers in Table S2.

**Figure 7:**
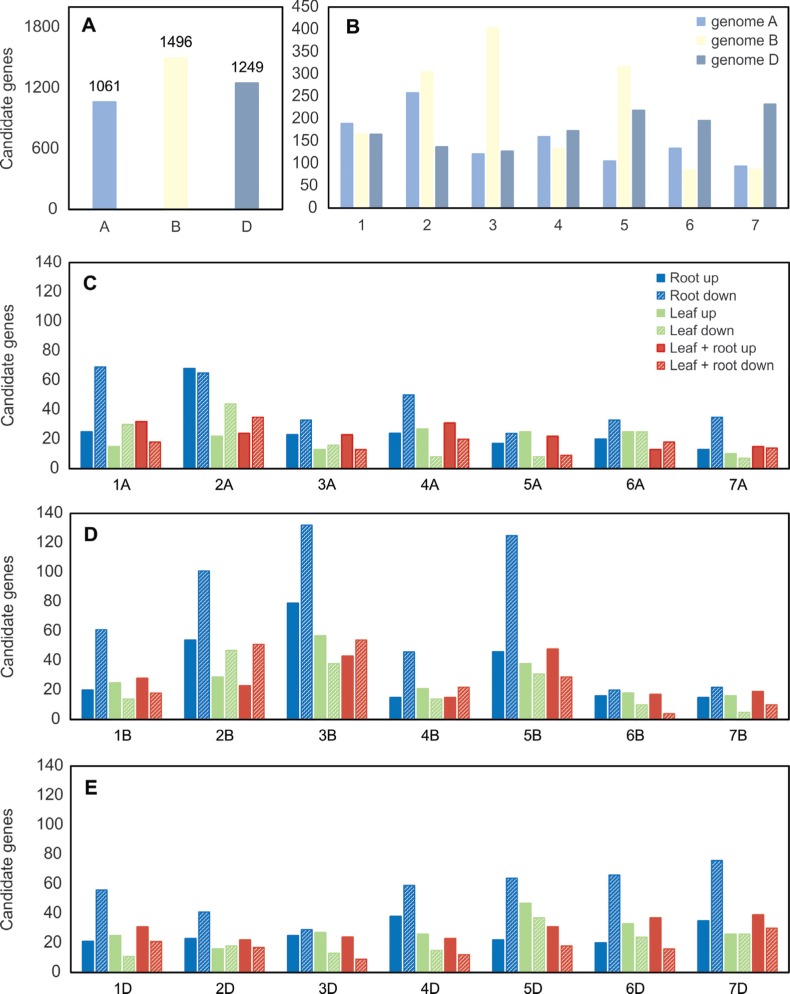
Genome distribution of the candidate genes associated to drought response in the wheat cultivar MGS1 Aliança. Sequences were retrieved by BLAST against the *Triticum aestivum* cv. Chinese Spring genome at an E-value cutoff 1e-100. (A) Number of candidate genes per genome. (B) Total number of candidate genes per chromosome. (C), (D) and (E) Number of candidate genes that are up- or down-regulated and that are specific to roots, leaves, in common between both tissues for genome A, B and D, respectively.

**Figure 8:**
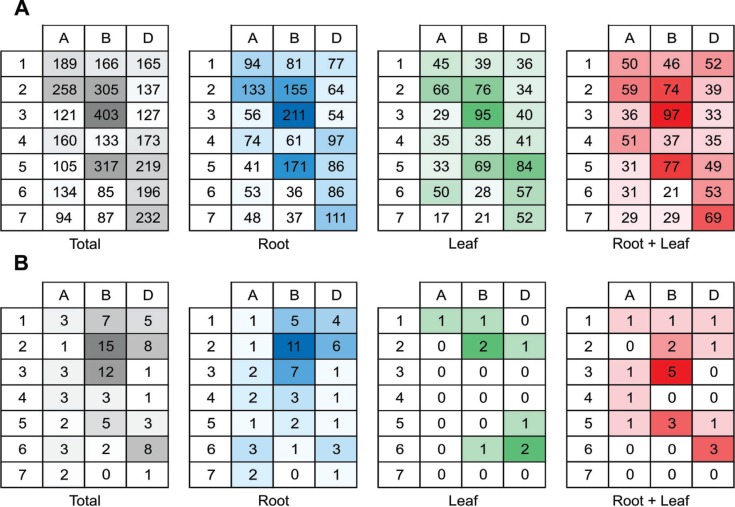
Heat map showing the localization of the candidate genes associated to drought response in the wheat cultivar MGS1 Aliança. Hit distribution by tissue was obtained by BLAST against the genome of *Triticum aestivum* cv. Chinese spring at an E-value cutoff 1e-100. Numbers represent the hit distribution for each chromosome. "Total" means hit distribution of all sequences. "Root" means hit distribution of transcripts specifically expressed in roots. "Leaf" means hit distribution of transcripts specifically expressed in leaf. "Root + Leaf" means hit distribution of transcripts expressed in common between root and leaf. Darker colors mean higher number of reads. (A) All the candidate genes. (B) Only the candidate genes classified as no-hit.

An analysis of the distribution of candidate genes across the wheat genome was done by BLAST searches against the sequenced *T. aestivum* cv. Chinese Spring genome (Figure 7, Figure 8). Among the 3,987 candidate genes assembled by CAP3, 158 transcripts could not be mapped. More candidate genes were located in the B genome (p < 0.001 by the chi-square test) compared to the A and D genomes (Figure 7A). In addition, chromosomes 3B, 5B and 2B had more sequences related to drought response (Figure 7B). Candidate genes specific to roots or leaves and in common between the two tissues were detected in all genomes and chromosomes (Figure 7C-E). Although chromosomes 3B, 5B and 2B showed the highest number of candidate genes, most of the transcripts mapping to these chromosomes were down-regulated. In fact, only chromosomes 5A, 6B, 7B and 3D presented at least 10% more up-regulated transcripts than down-regulated ones. The chromosomes with more up-regulated sequences were 3B, 5B and 2A. The CAP3 assembly also allowed for the identification of no-hits candidate genes per chromosome (expression only in root or leaf and expression in both tissues), where 88 no-hit sequences were detected (Figure 8). The two chromosomes with the highest numbers of no-hit sequences were chromosomes 2B and 3B. In these chromosomes, most of the no-hit sequences was specific to roots (Figure 8B).

### Transcription factors

Transcription factors (TFs) play a central role in the plant response to drought (Tuberosa and Salvi, 2006). Thus, we searched for TFs among the differentially expressed sequences. To achieve this, the similarity of the sequences was evaluated against a plant transcription factor database (PlantTFDB) with an E-value cutoff of e-100. Several TFs, such as E2F/DP, SRS, WOX, M-type, NF-YB, GRF, LBD, CPP, GeBP, STAT, BBR-BPC, Whirly, BES1, NF-YA, NF-YC, HB-PHD, GATA, DBB, NF-X1, VOZ, CO-like, AP2, B3, SBP, Dof, ARR-B, HB-other, MIKC, EIL, Nin-like, Trihelix, G2-like, HD-ZIP, CAMTA, MYB, HSF, ERF, TALE, WRKY, C2H2, FAR1, bHLH, NAC, bZIP, MYB related, C3H, ARF, GRAS and DREB were found (Figure S4). The species with the greatest numbers of hits were *Oryza sativa japonica*, *Sorghum bicolor*, *T. aestivum* and *B. distachyon* (data not shown).

## Discussion

Rainfed wheat plants growing in the Cerrado biome need to cope with different abiotic stresses, with drought being one of the most important factors. In this context, a wheat cultivar adapted to that region represents an excellent model to study drought response mechanisms. Here, we identified 4,422 candidate genes associated to severe drought response in both root and leaf tissues during the tillering stage of the wheat cultivar MGS1 Aliança. Although the early stages of pollen development are the most vulnerable to drought in cereals (Fischer, 1973), seed germination and early seedling growth are also considered critical stages for wheat establishment (Zhang *et al*., 2014). Therefore, the early phase of wheat development is an important stage to evaluate the effect of drought. Moreover, for wheat farming in the Cerrado, dry spells can occur during the tillering stage (Ribeiro Júnior *et al*., 2006).

The functional annotation of the transcripts reported here (Figure 1) is in agreement with other reports (Deokar *et al*., 2011; Li *et al*., 2012; Zhou *et al*., 2012). However, one important difference is the technique used here (454 sequencing technology) in comparison to the one used to evaluate the gene expression in previous studies. The 454 technology is an ‘open’ system in which gene expression can be accurately measured by counting the detected identical transcripts, potentially capturing all the transcripts in a sample (Coram *et al*., 2008). Although the Blast2GO analysis showed similarity of the bread wheat expressed sequences with *A. tauschii* and *H. vulgare* (Figure S3), sequences with unknown function or no-hits were also found (Figure 8B). The no-hit sequences are an important contribution of high-throughput sequencing techniques because they represent a more complete description of gene expression and should be important to understand drought stress response in wheat. In our survey, the distribution of the no-hit sequences was higher on chromosome 2B (38, considering both chromosome arms). Regarding the three wheat genome components, the B genome harbored the highest number of no-hit sequences (44) when compared to the D genome (27) and A genome (17) (Figure 8B).

The total number of identified repressed transcripts in response to drought (2,305 for roots and leaves) was higher than the number of induced transcripts (2,117 for both tissues). However, when considering each tissue separately, the number of repressed transcripts was lower than the induced transcripts in leaves (597 repressed and 1,017 induced) but higher in roots (1,708 repressed and 1,100 induced). A higher number of repressed transcripts under drought conditions in hexaploid wheat was also reported by other authors (Mohammadi *et al*., 2008; Li *et al*., 2012). To validate the repression/induction detected by the RNA-seq experiment, we used RT-qPCR to confirm the expression profile of 35 candidate genes (15 from roots and 20 from leaves). These candidate genes are representative of different pathways or code for different enzymes in the same pathway (Table S2), providing a broad validation of the RNA-seq experiment. The RT-qPCR assays revealed statistically significant differences for 73.3% and 60% sequences from root and leaf, respectively (Figure 2, Figure 3). Significant differences were not detected for the remaining sequences, but the direction of the expression profile was generally the same, and the Pearson's correlation between the RNA-seq and RT-qPCR data was 0.78 (Figure S2). It is important to note that the RT-qPCR assays were done as a second and independent experiment for confirmation of the gene expression. For RNA-seq experiments reported previously by others, the RT-qPCR correlation coefficients varied from 0.58 to 0.98 (Nagalakshmi *et al*., 2008; Kogenaru *et al*., 2012). The RT-qPCR technique was also used to confirm the expression of two tissue-specific sequences found among the candidate genes (Figure 4).

An important mechanism used by plants to tolerate drought is osmotic adjustment (Nezhadahmadi *et al*., 2013). In this process, accumulation of solutes in cells allows to decrease the osmotic potential and to maintain the cell turgor as drought stress develops. Osmoprotectants synthesized in response to drought stress include low molecular weight and highly soluble compounds, such as sugars, proline, polyols, and quaternary ammonium (Pintó-Marijuan and Munné-Bosch, 2013). In wheat, osmotic adjustment is positively associated with higher yield under drought stress and could partly explain the genotypic variation in stomatal response of wheat cultivars that differ in their responses to drought (Morgan and Condon, 1986; Izanloo *et al*., 2008). Here, we identified the sucrose metabolism as an important pathway for drought response in the cultivar MGS1 Aliança (Figure 6). When considering both tissues separately, the sucrose metabolism pathway was still found to be among the three most important ones. There are four enzymes that play a key role in starch metabolism: EC 2.4.1.13 - Susase, EC 2.7.7.27 - AGPase, EC 2.4.1.21 - STSase and EC, 2.4.1.18 - SBE (Yang *et al*., 2004), and all these enzymes, except for AGPase, were activated during the water stress evaluated in this study. These enzymes also played an important role when previously evaluated in wheat plants grown under water stress conditions (Ahmadi and Baker, 2001). In addition, starch and sucrose metabolism, phenylpropanoid biosynthesis, and glyoxylate and dicarboxylate metabolism were also the most frequently detected KEGG pathways in a transcriptome analysis of *Paulownia australis* grown under drought conditions (Dong *et al*., 2014). Furthermore, proline is a solute that plays a role as a protective agent for cells under osmotic stress, performing an important function in the drought stress response (Nezhadahmadi *et al*., 2013). In our study, the P5CS1 and DELTA-OAT transcripts, related to proline biosynthesis, were up-regulated in leaf and root tissues (Table S4). In contrast, the ALDH12A1 and P5CS2 transcripts were up-regulated only in leaves, and the ALDH1 transcript was up-regulated in roots only. In fact, the arginine and proline metabolism pathway is among the top 20 pathways found to be induced in leaves (Figure 6).

Other transcripts already linked to the drought response were found among the candidate genes (Table S4). These transcripts include glutathione S-transferase and others related to glutathione biosynthesis and catabolism (GGT1, GSTU25, AT1G65820 and GSTU18 down-regulated in roots; GSH1 and OXP1 up-regulated in roots; GR1 up-regulated in leaves; GSTL3 up-regulated in leaves and roots, and ERD9 down-regulated in roots and up-regulated in leaves); dehydrins (DHN1 up-regulated in leaves and roots); and other late embryogenesis abundant (LEA) proteins (LEA7 was down- and up-regulated in leaves, while up-regulation was observed for AtLEA4-1, LEA14, LEA and LEA4-5; in roots, LEA14, AT2G46140, AtLEA4-1 and LEA7 were up-regulated, while ACC1 was down-regulated). These proteins are important enzymes involved in stress responses, helping to cope with detoxification and reducing cellular damage by recovering denatured proteins and stabilizing membranes (Koag *et al*., 2003; Umezawa *et al*., 2006). For example, the wheat LEA genes PMA1959 and PMA80 improved water deficit resistance in rice (Cheng *et al*., 2002), and the wheat dehydrin, DHN-5, improved drought tolerance when overexpressed in *Arabidopsis thaliana* (Brini *et al*., 2007). Moreover, these proteins are among the differentially expressed transcripts detected in hard red winter wheat cultivars submitted to water-deficit conditions (Reddy *et al*., 2014).

Another strategy to decrease the effects of drought is to retard leaf senescence (a process that is accelerated in drought-sensitive genotypes). In practical terms, leaf senescence leads to reduced yield, meaning that the suppression of drought-induced leaf senescence is desirable (Jewell *et al*., 2010). In the MGS1 Aliança genotype analyzed here, candidate genes with GO terms related to leaf senescence were found in both tissues (RCA, HAI1 and LTI65 in leaf and SAG12, SAG29, LTI65, ARF1, WRYK70 and OPR1 in root) (Table S4). Moreover, many candidate genes related to the biosynthesis of the hormone abscisic acid (ABA) were also found in our study; for example, the AAO3 and NCED3 (9-cis-epoxycarotenoid dioxygenase) transcripts, which code for important enzymes in the ABA biosynthesis pathway (Table S4). Overexpression of the NCED3 transcript in Arabidopsis leads to tolerance of drought (Iuchi *et al*., 2001). ABA synthesis increases in plants under water stress, inducing stomatal closure, reducing water loss via transpiration, and shaping transcript expression, which is also important for response to salinity and cold (Shinozaki and Yamaguchi-Shinozaki, 1997; Mahajan and Tuteja, 2005; Guóth *et al*., 2009). In addition, transcripts related to ABA transduction signaling were also identified; for example, the OST1 transcript, which responds to ABA stimulus controlling stomatal closure (Mustilli *et al*., 2002; Yoshida *et al*., 2002).

The candidate genes induced by drought stress and classified as "transcription factors" were less numerous than reported by Li *et al*. (2012). In this category, TFs such as bZIP, CBF, EREBP, WRKY, MADS, NAC and Myb were found (Figure S4). Some of these TFs have been analyzed by others, being up-regulated in roots of a drought-tolerant genotype (Okay *et al*., 2014) and induced by drought stress in different species of *Triticum* (Baloglu *et al*., 2014). A large number of TFs has also been found to be differentially regulated in response to heat, drought and their combination (Liu *et al*., 2015). In addition, an increase in drought tolerance has been demonstrated in transgenic plants over-expressing some of those TFs, such as transgenic Arabidopsis expressing NAC TF or TaMYB2A (Mao *et al*., 2011; Li *et al*., 2014), rice expressing the DREB1A from Arabidopsis (Ravikumar *et al*., 2014), wheat plants overexpressing MYB-TF (TaPIMP1) or TaERF3 (Zhang *et al*., 2012; Rong *et al*., 2014) and tobacco expressing TaABP1 (bZIP-TF) or TaWRKY10 (Cao *et al*., 2012; Wang *et al*., 2013).

One of the practical applications of the isolation of drought-related genes is the development of transgenic plants that are more tolerant to drought stress. So far, several papers have reported on that approach, using transcripts belonging to some of the functional groups discussed above. Examples in transgenic wheat include osmoprotectant genes (Abebe *et al*., 2003; Vendruscolo *et al*., 2007), LEA proteins (Sivamani *et al*., 2000; Bahieldin *et al*., 2005), a gene from the C_4_ pathway (Qin *et al*., 2015) and TFs (Morran *et al*., 2011; Xue *et al*., 2011; Saint Pierre *et al*., 2012; Zhang *et al*., 2012). In these reports, the experiments were performed with genes obtained from *A. thaliana* (*DREB*), *Atriplex hortensis* (*BADH*), *Escherichia coli* (*mtlD* or *betA*), barley (HVA1), cotton (*GhDREB*), rice (*SNAC1*), or *Vigna aconitifolia* (*P5CS*). Only a few studies have been performed with genes isolated from wheat, such as *TaDREB2*, *TaDREB3*, *TaNAC69-1*, or *TaPIMP1* (Morran *et al*., 2011; Xue *et al*., 2011; Zhang *et al*., 2012). So far, field data regarding the performance of these transgenic plants have not been conclusive, with the transgenic lines not outperforming the controls or showing unstable performance along the years (Bahieldin *et al*., 2005; Saint Pierre *et al*., 2012). Nonetheless, it should be interesting to evaluate the production of these plants in the Cerrado region.

The candidate genes found here are distributed across all component genomes and chromosomes of the wheat genome (Figure 7, Figure 8). The number of sequences belonging to the B genome was higher in comparison with the A and D genomes. During evolution, the diploid genomes A and B (from wild species related to *T. urartu* and *Aegilops speltoides*, respectively) underwent an alloploydization event to form the tetraploid wheat *T. turgidum*, followed by another alloploydization with the D genome (*Aegilops tauschii*) (Leach *et al*., 2014). It has been shown that there is a tendency of B genome homoeoloci to contribute more to gene expression in wheat than A or D genome homoeoloci (Leach *et al*., 2014). Moreover, the wild *T. turgidum* spp. *dicoccoides* (AABB genome), which is the ancestor of cultivated *T. turgidum* ssp. *durum* and *T. aestivum* (Budak *et al*., 2013), contains a gene pool enriched for various agronomic traits, including drought tolerance (Peleg *et al*., 2008; Ergen *et al*., 2009). That information could encourage investigations on drought response in tetraploid wheat, and that tolerance could be incorporated into synthetic lines. However, it is important to note that the interaction among the A, B and D genomes could activate or silence homeologous genes (Wang *et al*., 2011), making the introduction of genes from the B genome into the hexaploid genome a laborious task. Figures 7 and 8 also show that chromosomes 3B, 5B and 2B contribute with a greater number of drought-related transcripts in both roots and leaves. In wheat, quantitative trait loci (QTL) identified under different water regimes have been reported for traits like, for example, canopy temperature, carbon isotope discrimination, photosynthetic parameters and yield or yield components (Sheoran *et al*., 2016). Virtually all wheat chromosomes and component genomes contain QTL for drought tolerance, most of them explaining a small fraction of the observed phenotypic variation. When focusing on chromosomes 3B, 5B and 2B, the major regions identified in our study, QTL for a number of traits correlated to drought tolerance have been described like, for instance, abscisic acid, canopy temperature, carbon isotope discrimination, chlorophyll content, coleoptile length, flag leaf rolling index, flag leaf senescence, grain number, grain size, grain weight, normalized difference vegetation index, water soluble carbohydrates, phenology (anthesis, heading, maturity), plant height, and yield (Sheoran *et al*., 2016). The co-localization of the QTL with some of the candidate genes obtained in this survey could be an interesting target for future work.

In conclusion, the present study allowed for the identification of genes related to important pathways for drought response in the wheat cultivar MGS1 Aliança, a well-adapted cultivar for rainfed farming in the Cerrado biome. Clearly, our results showed that the main pathways activated under water deprivation differ for roots and leaves. Increments in drought tolerance through conventional and biotechnological approaches should take this difference into consideration. The drought stress-related transcripts described here will be further characterized to provide targets of interest for breeders. They are also important to elucidate the complex regulatory network(s) of the drought response. The characterization of candidate genes that are differentially expressed among drought-tolerant and -sensitive genotypes can help identify useful molecular markers and candidate genes. In the long run, the interesting targets and molecular markers can be used to achieve more sustainable wheat production.

## Supplementary Material

The following online material is available for this article:

Figure S1 - Gene expression distribution between control and drought-stressed (treated) roots and leaves of the wheat cultivar MGS1 Aliança.

Figure S2 - Correlation of transcript levels between RNA-seq and RT-qPCR data.

Figure S3 - Results of the Blast2GO analysis with the 3,987 candidate genes.

Figure S4 - Transcription factor distribution by family.

Table S1 - Set of 4,422 candidate genes identified in root and leaf tissues of the wheat cultivar MGS1 Aliança under drought.

Table S2 - Group of transcripts used in validation by RT-qPCR.

Table S3 - Biological pathways activated in wheat under drought (Blast2GO and KEGG analysis)

Table S4 - Details of selected candidate genes.
