# Supplementary material for: Gene expression analysis reveals important pathways for drought response in leaves and roots of a wheat cultivar adapted to rainfed cropping in the Cerrado biome
Source: Genet Mol Biol. 2016 Oct 20;39(4):629–45. doi: 10.1590/1678-4685-GMB-2015-0327 (PMC5127152; doi:10.1590/1678-4685-GMB-2015-0327)
Supplement: Supplementary file 1 [file 1415-4757-gmb-1678-4685-GMB-2015-0327-Suppl01.pdf]

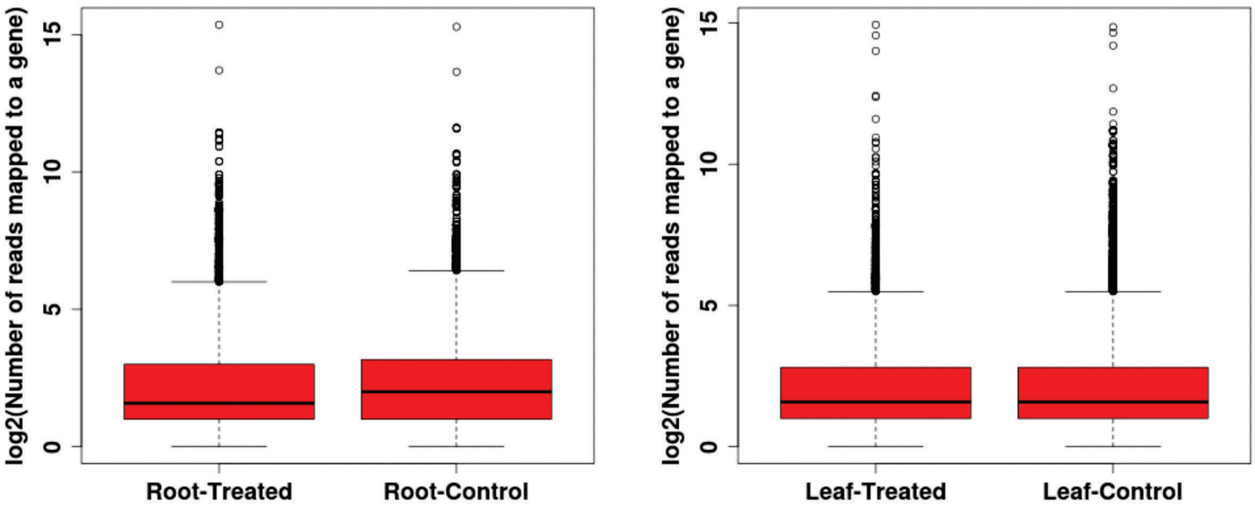

Figure S1 - Gene expression distribution between control and drought-stressed (treated) roots and leaves of the wheat cultivar MGS1 Aliança. Transcripts were obtained by 454 pyrosequencing of four libraries (control and treated roots, control and treated leaves) performed on a Genome Sequencer FLX Titanium instrument (Roche). Calculation was performed in DEGseq software for analysis of the statically differentially expressed transcripts in order to identify candidate genes associated to drought.
