# Supplementary material for: Gene expression analysis reveals important pathways for drought response in leaves and roots of a wheat cultivar adapted to rainfed cropping in the Cerrado biome
Source: Genet Mol Biol. 2016 Oct 20;39(4):629–45. doi: 10.1590/1678-4685-GMB-2015-0327 (PMC5127152; doi:10.1590/1678-4685-GMB-2015-0327)
Supplement: Supplementary file 3 [file 1415-4757-gmb-1678-4685-GMB-2015-0327-Suppl06.pdf]

Table S2 - Group of transcripts used in validation by RT-qPCR.

Primer	Isotig	Primer F	Primer R	Expression	Annotation
R1	isotig 15618	GCGCGAGAAACTAAAACGAC	CTCCCCACCACCGACTACTA	down	AT2G36830 - gamma-tip - gamma tonoplast intrinsic protein 1– "water channel activity" - "water transport" - "urea transport"
R3	isotig 08533	GAAAGCACAGCAACAAACAGG	TCGCGTGTATTAAGACTCCAGTG	down	AT4G23400 - PIP1;5 - plasma membrane intrinsic protein 1;5 - "water channel activity"
R6	isotig 12220	AGCTACAAGGAGGCCTGACA	CAAATTTTTCCCGCAGTCAT	down	AT5G14420 - RGLG2 - ring domain ligase2 - "ubiquitin-protein ligase activity" - "negative regulation of response to water deprivation"
R8	isotig 04932	ATGCTACGGTTCATGTGCAA	ACATGGGAGGAACAGAATGC	up	AT2G37770 - ChlAKR - "aldo-keto reductase (NADP) activity" - "response to water deprivation"
R13	isotigs 08385/6	GGCCAACTGAGTCGTAGTTCA	ACACAACCCTTGGACACACA	up	AT1G01470 - LEA14 - "response to desiccation"
R15	isotig 08654	CTTGCTCTTGCACCCAATC	GGTCGAGATCGAGATGGAGA	up	AT1G04560 - molecular function unknown
R17	isotig 09327	TTCTTTTTGGCAGGGACACT	CTTCGTCCAAGCAGACATTG	up	AT1G04560 - molecular function unknown
R19	isotig 09284	GTCTGACCGTCTTTGAGCTTTTA	GCAGCACATGCACAGGAA	up	AT1G04560 - molecular function unknown
R21	isotig 19917	TAGCGATCGACCTGTGTCTG	CGCCGATTATTTATCCCGTA	up	Dehydrin DHN1 - "cellular response to water deprivation"
R22	isotig 07281	TGCATGAAGGCAGTGTACGA	TGCCGACGTCTGTTGATATTT	up	AT3G21000 - "molecular function"
R24	isotig 11306	GAGACAAGTCCCGTGTTGGT	CCCCACGGAGAACTTTAACA	up	AT2G39800 - P5CS1 Delta1-Pyrroline-5-Carboxylate Synthase 1 - "proline biosynthetic process" - "response to water deprivation"
R28	isotig 04456	GCGGTGGTCGTGGTCA	CTCTCATCTCTCGATCAATGTCTC	up	AT3G57520 - SIP2 - "response to oxidative stress" - "galactinol-sucrose galactosyltransferase activity"
R33	isotig 06719	TTTTCGCGTACGGGACTATC	CCATGGACGTGTGATACCTG	up	No hit
R34	isotig 15379	AGCGGGATCATGTATGGAAG	CGCCATATGCGGTAATAGGT	up	No hit
R36	isotig 16364	GACGACGTACACTTGCTGGA	CGCCGACTACTGAAAACCTC	down	AT4G01470 - TIP1;3Tonoplast intrinsic protein 1;3 - "water transport" - "urea transport"
L1	isotig 09384	GTCCACAGCCTTGTTGAGGT	AAGAGCTCGAGGAGGAGGAG	up	No hit
L2	isotig 02373	GCTTCATGCAGGAAAGACAA	ACATTGCGAGTGCTGAACTG	up	AT4G02280 - SUS3 - "sucrose biosynthetic process"
L3	isotig 02886/7	ATGGCAGGGACCTTAGTCCT	TCTCAGACCGACAAATGCTG	up	AT5G50920 - CLPC1 - " regulation of chlorophyll biosynthetic process"
L4	isotig 05427	GATTCTGCGCTCAAACCTTC	CTGGGACGGTCTCCTATCAA	up	DAA1 "DUO1-activated ATPase 1" - "nucleotide binding"
L6	isotig 19351	GATTCGGGTTCAGTTGGCTA	ACGGAGGTGAGAGTCGAATG	up	No hit
L7	isotig 02194	AATTTGTTTGTGTAGTCCTTGAGC	GCAGCGACCTTCACCATT	down	AT1G73010 - PS2 - "phosphate starvation-induced gene 2"
L8	isotigs 03192/3	GCTGAAGCCTTGGACAAAAG	TGTACAACCAGCTGCCAGAG	up	AT1G74920 - ALDH10A8 - "response to water deprivation"
L9	isotig 03016	GGCTCGTATGCTTGTTTCGT	TTACACAAACCTGAACCCTGTG	up	AT2G19900 - NADP-ME1
L10	isotig 13697	CGCGTATCAGTGTTGCAGTC	TGTCACGGTCGGGAGATAAT	up	AT5G06760 - LEA4-5 - "response to osmotic stress" - "response to water deprivation"
L11	isotig 03896	CCTGGATTCTGCTGGTCTTT	CTAACGAAGACGCACACTCG	down	AT5G60660 - PIP2;4 - "water channel activity" - "hydrogen peroxide transmembrane transport"
L13	isotig 00436/7/8	TTGGCGAATACCACATACGA	CTGGCTTTCAAGGAATGGAA	up	AT3G18000 - XPL1 - "post-embryonic root development"
L14	isotig 03490/1	GTCCCTCCAGTCGAAGTCCT	TCACCAAGTTCTCCGACCTC	up	AT4G39090 - RD19 - "response to desiccation"
L15	isotigs 02086/7/8	GTCGGTGTGTGTCCCTTTTT	GCAGTCCTGGCAAATTAAGC	up	AT2G29380 - HAI3 "highly ABA-induced PP2C gene 3" - AT3G11410 - PP2CA - "response to water deprivation" - "regulation of stomatal movement"
L16	isoitg 11547	CCGTGTATGTGTGGTCCTTG	TTTATGCACACGCCCCTAAT	up	No hit
L17	isotig 07408	AGTCAGAGGTCGCTCTTCGT	GCGTTCAACCATACAAGCAA	down	AT2G34420 - LHB1B2 - "photosynthesis, light harvesting in photosystem II"
L19	isotig 00436/7/8	ATGATGAGCCCAAGTTACGG	TTCTTCTTTGGCACCTACGG	up	AT3G18000 – XPL1 - "phosphoethanolamine N-methyltransferase activity"
L23	isotig 02266	GTAGGGGATTCGCCTTCTTC	GGAACGGGAACTTGACAAAC	down	AT4G25480 - DREB1A - "sequence-specific DNA binding transcription factor activity" - "response to water deprivation"
L25	isotig 01879	ACTGTAGAATTGCATCGGTACATC	TTCAGGTGCTCTTTGCCATA	up	AT2G39800 - P5CS1 - "proline biosynthetic process" - "response to water deprivation"
L28	isotig 01300/1/2	ATGTTCCTCTTGCCATCACC	AGACAGTCCTGCTGCCAAGT	up	AT2G05710 - ACO3 - "response to oxidative stress" - "citrate metabolic process"
L29	isotig 02920	CATTGGCAATTGGTCGTTCT	CGGGAGTACGAGTGAAGGAT	up	AT2G39800 - P5CS1 - "proline biosynthetic process" - "response to water deprivation"

Primers starting with "R" indicates sequences from roots and starting with "L" indicates sequences from leaf tissue.
"Expression" refers to the increase (up) or decrease (down) in the number of transcripts detected in the drought stressed treatment in comparison to the control, as obtained by the 454 sequencing.
