# Supplementary material for: Gene expression analysis reveals important pathways for drought response in leaves and roots of a wheat cultivar adapted to rainfed cropping in the Cerrado biome
Source: Genet Mol Biol. 2016 Oct 20;39(4):629–45. doi: 10.1590/1678-4685-GMB-2015-0327 (PMC5127152; doi:10.1590/1678-4685-GMB-2015-0327)
Supplement: Supplementary file 4 [file 1415-4757-gmb-1678-4685-GMB-2015-0327-Suppl02.pdf]

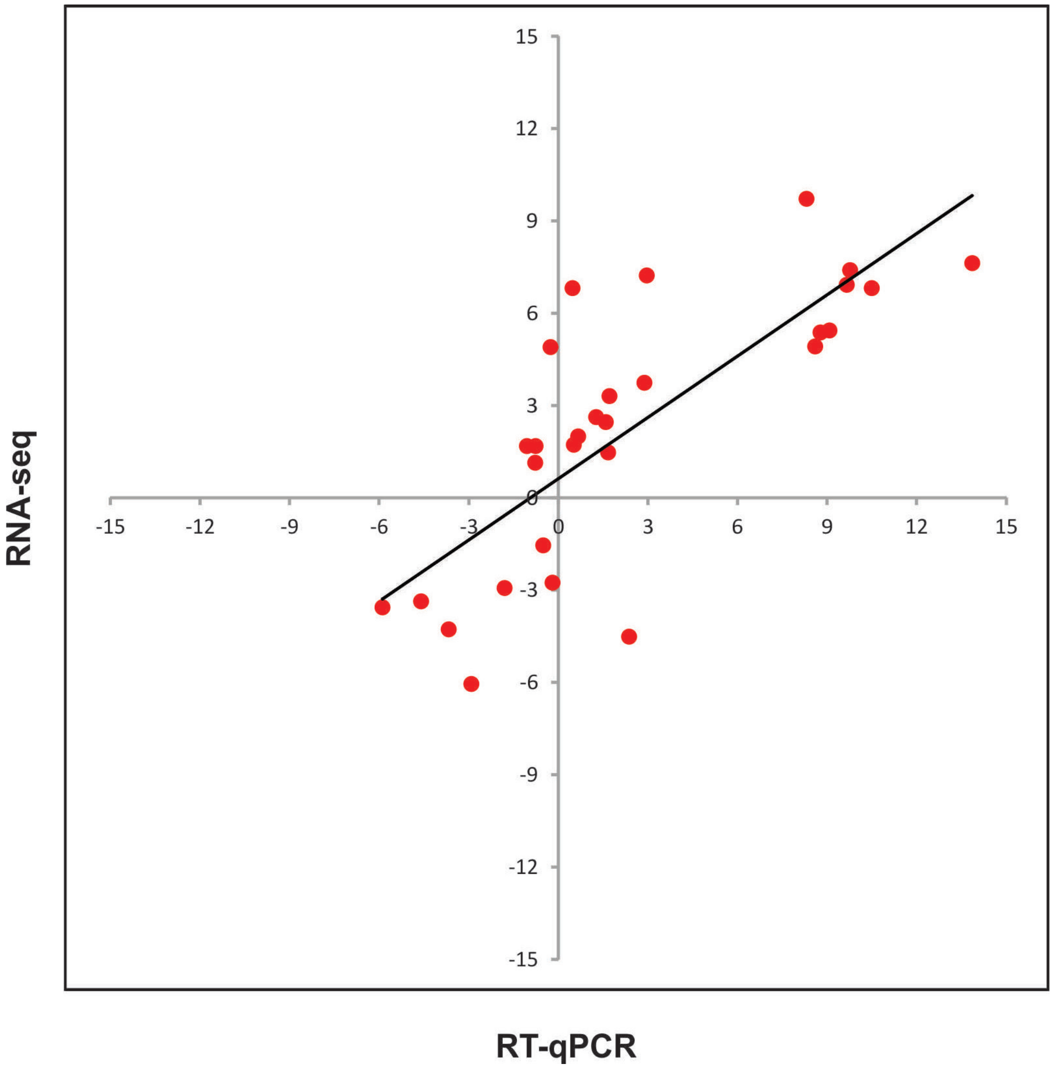

Figure S2 - Correlation of transcript levels between RNA-seq and RT-qPCR data. The analysis was performed for nine root- and 20 leaf-derived transcripts. Individual gene expression was calculated by fold change (log_2) of control and drought-stressed samples. The Pearson correlation coefficient is 0.7804.
