# Supplementary material for: Gene expression analysis reveals important pathways for drought response in leaves and roots of a wheat cultivar adapted to rainfed cropping in the Cerrado biome
Source: Genet Mol Biol. 2016 Oct 20;39(4):629–45. doi: 10.1590/1678-4685-GMB-2015-0327 (PMC5127152; doi:10.1590/1678-4685-GMB-2015-0327)
Supplement: Supplementary file 5 [file 1415-4757-gmb-1678-4685-GMB-2015-0327-Suppl03.pdf]

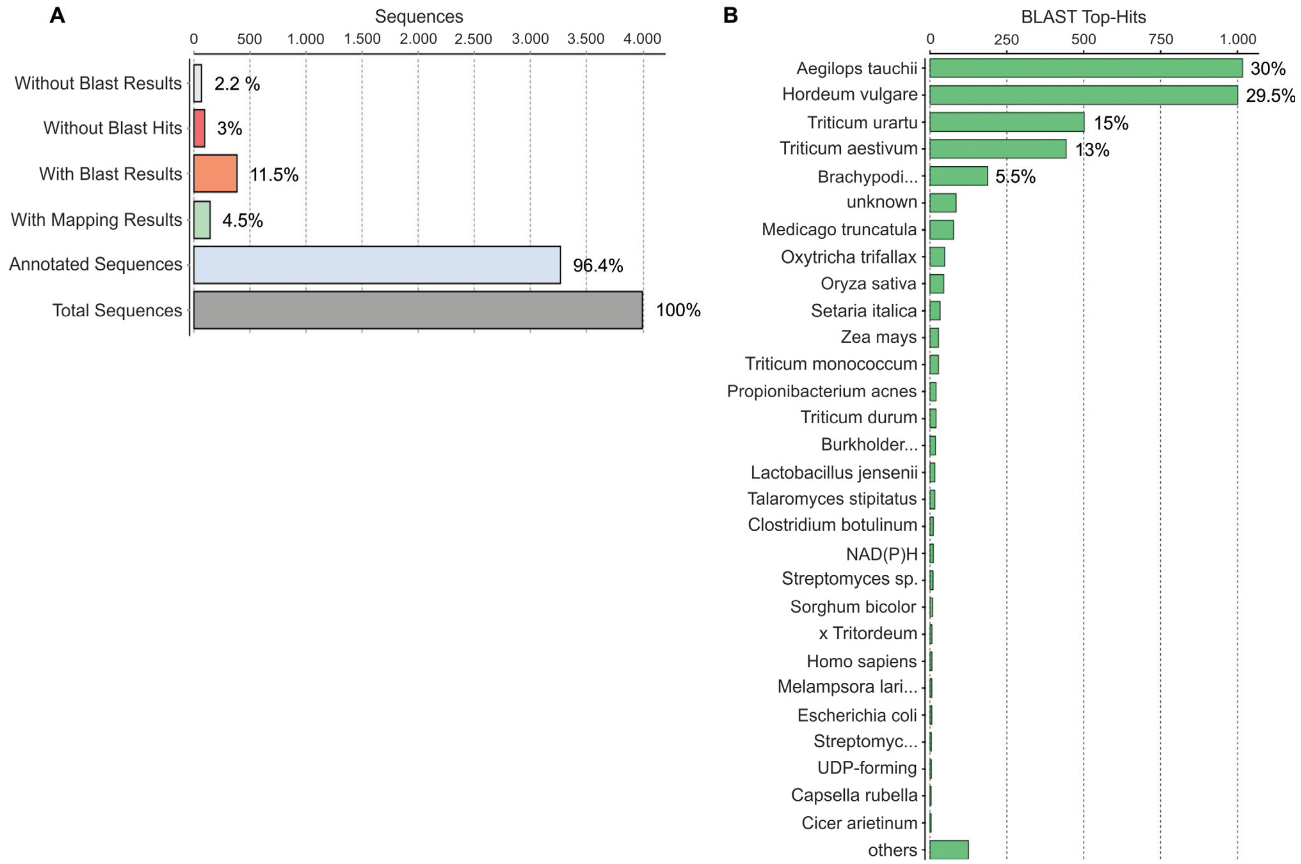


**Figure S3** - Results of the Blast2GO analysis with the 3,987 candidate genes obtained after an additional assembly performed with CAP3 software. **(A)** Data distribution according to BLAST, mapping and annotation results. **(B)** Distribution of similarity in different species.
