# Supplementary material for: Gene expression analysis reveals important pathways for drought response in leaves and roots of a wheat cultivar adapted to rainfed cropping in the Cerrado biome
Source: Genet Mol Biol. 2016 Oct 20;39(4):629–45. doi: 10.1590/1678-4685-GMB-2015-0327 (PMC5127152; doi:10.1590/1678-4685-GMB-2015-0327)
Supplement: Supplementary file 6 [file 1415-4757-gmb-1678-4685-GMB-2015-0327-Suppl07.pdf]

Table S3 - Biological pathways activated in wheat under drought resulted from Blast2GO analysis against the Kyoto Encyclopedia of genes and Genomes (KEGG). #Seqs: number of sequences in that pathway; #Enzs: number of enzymes corresponding to the sequences.

#	Pathways	Total		Root		Leaf		Root and Leaf	
		#Seqs	#Enzs	#Seqs	#Enzs	#Seqs	#Enzs	#Seqs	#Enzs
1	Starch and sucrose metabolism	152	30	67	18	42	12	43	25
2	Carbon fixation in photosynthetic organisms	113	18	18	7	75	14	20	12
3	Glyoxylate and dicarboxylate metabolism	86	12	14	3	58	10	14	7
4	Phenylpropanoid biosynthesis	85	9	67	6	7	6	11	6
5	Glycolysis/Gluconeogenesis	71	21	14	8	28	11	29	17
6	Galactose metabolism	67	13	24	7	15	6	28	12
7	Phenylalanine metabolism	67	9	53	5	3	4	11	7
8	Purine metabolism	66	16	19	6	23	10	24	13
9	Pentose phosphate pathway	59	11	19	6	21	8	19	9
10	Glutathione metabolism	56	14	21	6	12	4	23	12
11	Fructose and mannose metabolism	53	14	8	5	28	10	17	13
12	Methane metabolism	51	11	12	5	22	7	17	9
13	Glycerophospholipid metabolism	51	10	16	9	22	3	13	6
14	Aminobenzoate degradation	50	5	35	3	6	2	9	2
15	Cysteine and methionine metabolism	44	16	17	7	8	4	19	15
16	Nitrogen metabolism	43	11	23	9	9	7	11	6
17	Pyruvate metabolism	41	15	17	10	11	7	13	10
18	Porphyrin and chlorophyll metabolism	41	11	1	1	33	7	7	8
19	Tryptophan metabolism	41	5	35	3	3	2	3	2
20	Carotenoid biosynthesis	41	4	32	3	6	3	3	2
21	Arginine and proline metabolism	38	14	11	5	12	7	15	9
22	Glycine, serine and threonine metabolism	34	11	7	4	12	7	15	8
23	Pentose and glucuronate interconversions	34	8	14	4	13	5	7	6
24	Alanine, aspartate and glutamate metabolism	33	10	17	8	5	5	11	8
25	Toluene degradation	33	2	32	2	-	-	1	1
26	Xylene degradation	33	2	32	2	-	-	1	1
27	Amino sugar and nucleotide sugar metabolism	32	16	6	5	8	5	18	15
28	Glycerolipid metabolism	28	9	10	6	5	3	13	7
29	Carbon fixation pathways in prokaryotes	27	8	14	5	5	3	8	5
30	Cyanoamino acid metabolism	27	4	14	2	8	2	5	4
31	Thiamine metabolism	27	3	10	3	10	1	7	3
32	Ascorbate and aldarate metabolism	24	7	11	3	6	3	7	5
33	Citrate cycle (TCA cycle)	22	9	8	4	7	3	7	5
34	Oxidative phosphorylation	22	5	9	3	6	3	7	4
35	One carbon pool by folate	20	5	7	4	5	2	8	4
36	Drug metabolism - cytochrome P450	20	3	8	1	3	1	9	3

#	Pathways	Total		Root		Leaf		Root and Leaf	
		#Seqs	#Enzs	#Seqs	#Enzs	#Seqs	#Enzs	#Seqs	#Enzs
37	Metabolism of xenobiotics by cytochrome P450	20	3	8	1	3	1	9	3
38	Alpha-linolenic acid metabolism	19	6	9	4	6	1	4	3
39	Inositol phosphate metabolism	18	7	7	2	6	5	5	3
40	Sulfur metabolism	18	6	5	2	8	4	5	6
41	Riboflavin metabolism	17	3	5	1	4	1	8	3
42	Linoleic acid metabolism	17	3	7	2	6	1	4	2
43	Drug metabolism - Other enzymes	17	2	-	-	11	2	6	2
44	Ether lipid metabolism	16	2	4	2	8	1	4	2
45	Beta-alanine metabolism	15	6	6	2	4	3	5	4
46	Selenocompound metabolism	15	4	5	3	6	2	4	3
47	Valine, leucine and isoleucine degradation	14	8	5	3	5	3	4	3
48	Terpenoid backcone biosynthesis	14	6	9	4	2	1	3	3
49	Tyrosine metabolism	14	6	7	3	2	1	5	6
50	Other glycan degradation	13	3	11	3	-	-	2	1
51	Phosphatidyinositol signaling system	12	6	3	2	2	2	7	4
52	Butanoate metabolism	12	4	6	3	3	3	3	4
53	Propanoate metabolism	9	6	3	2	2	1	4	4
54	Streptomycin biosynthesis	9	4	-	-	3	2	6	3
55	Fatty acid degradation	8	6	1	1	2	1	5	4
56	Fatty acid biosynthesis	8	5	3	2	1	1	4	4
57	Taurine and hypotaurine metabolism	8	3	5	2	1	1	2	2
58	Photosynthesis	8	2	1	1	5	2	3	2
59	Glycosphingolipid biosynthesis - globo series	8	2	2	1	1	1	5	2
60	Sphingolipid metabolism	8	2	3	2	1	1	4	1
61	Biosynthesis of ansamycins	8	1	3	1	1	1	4	1
62	Ubiquinone and other therpenoid-quinone biosynthesis	7	3	6	2	-	-	1	1
63	Flavonoid biosynthesis	6	3	4	1	2	2	-	-
64	Lysine degradation	6	3	3	1	2	1	1	1
65	Pyrimidine metabolism	6	3	-	-	3	1	3	2
66	Arachidonic acid metabolism	6	3	1	1	-	-	5	3
67	Phenylalanine, tyrosine and tryptophan biosynthesis	5	6	2	1	-	-	3	6
68	Biosynthesis of unsaturated fatty acids	5	4	3	2	-	-	2	2
69	Caffeine metabolism	5	3	-	-	1	1	4	3
70	Retinol metabolism	5	3	2	1	-	-	3	2
71	Steroid hormone biosynthesis	5	2	2	1	-	-	3	2
72	Nicotinate and nicotinamide metabolism	5	2	2	2	-	-	3	1
73	Biosynthesis of terpenoids and steroids	5	1	2	1	3	1	-	-
74	Biotin metabolism	4	2	2	1	-	-	2	2
75	Steroid biosynthesis	4	2	-	-	2	2	2	2
76	Caprolactan degradation	4	1	3	1	-	-	1	1

#	Pathways	Total		Root		Leaf		Root and Leaf	
		#Seqs	#Enzs	#Seqs	#Enzs	#Seqs	#Enzs	#Seqs	#Enzs
77	T cell receptor signaling pathway	4	1	2	1	-	-	2	1
78	Chloroalkane and chloroalkene degradation	3	2	-	-	2	1	1	1
79	Aminoacyl-tRNA biosynthesis	3	2	2	1	-	-	1	1
80	N-Glycan biosynthesis	3	2	-	-	2	2	1	1
81	Histidine metabolism	3	2	-	-	2	1	1	1
82	Betalain biosynthesis	3	1	2	1	-	-	1	1
83	D-glutamine and D-glutamate metabolism	3	1	3	1	-	-	-	-
84	Valine, leucine and isoleucine biosynthesis	2	2	1	1	-	-	1	1
85	mTOR signaling pathway	2	1	1	1	1	1	-	-
86	Limonene and pinene degradation	2	1	-	-	2	1	-	-
87	Other types of O-glycan biosynthesis	2	1	2	1	-	-	-	-
88	Glycosaminoglycan biosynthesis - heparan sulfate/heparin	2	1	2	1	-	-	-	-
89	Glycosaminoglycan biosynthesis - chondroitin sulfate/dermatan sulfate	2	1	2	1	-	-	-	-
90	Zeatin biosynthesis	1	2	1	2	-	-	-	-
91	Tropane, piperidine and pyridine alkaloid biosynthesis	1	2	-	-	-	-	1	2
92	Isoquinoline alkaloid bioynthesis	1	2	-	-	-	-	1	2
93	Novobiocin biosynthesis	1	2	-	-	-	-	1	2
94	Naphthalene degradation	1	1	-	-	-	-	1	1
95	Stilbenoid, diarylheptanoid and gingerol biosynthesis	1	1	-	-	1	1	-	-
96	Lysine biosynthesis	1	1	-	-	-	-	1	1
97	Benzoate degradation	1	1	1	1	-	-	-	-
98	Synthesis and degradation of ketone bodies	1	1	-	-	-	-	1	1
99	Biosynthesis of vancomycin group antibiotics	1	1	-	-	-	-	1	1
100	Butirosin and neomycin biosynthesis	1	1	-	-	-	-	1	1
101	Polyketide sugar unit biosynthesis	1	1	-	-	-	-	1	1
102	Pantothenate and CoA biosynthesis	1	1	-	-	-	-	1	1
103	Diterpenoid biosynthesis	1	1	1	1	-	-	-	-
104	Indole alkaloid biosynthesis	1	1	-	-	-	-	1	1
105	Styrene degradation	1	1	1	1	-	-	-	-
106	Ethylbenzene degradation	1	1	1	1	-	-	-	-
107	Fatty acid elongation	1	1	1	1	-	-	-	-
108	Various types of N-glycan biosynthesis	1	1	-	-	1	1	-	-
109	Aflotoxin bioshyntesis	1	1	-	-	-	-	1	1
110	Tetracycline bioshynthesis	1	1	-	-	-	-	1	1
111	Geraniol degradation	1	1	1	1	-	-	-	-
112	Primary bile acid biosynthesis	1	1	-	-	1	1	-	-
113	Glycosphingolipid biosynthesis - ganglio series	1	1	1	1	-	-	-	-
114	Glycosphingolipid biosynthesis - lacto and neo-lactoseries	1	1	-	-	-	-	1	1
115	C-5branched dibasic acid metabolism	1	1	-	-	-	-	1	1
116	Glycosaminoglycan degradation	1	1	1	1	-	-	-	-
