# Supplementary material for: Gene expression analysis reveals important pathways for drought response in leaves and roots of a wheat cultivar adapted to rainfed cropping in the Cerrado biome
Source: Genet Mol Biol. 2016 Oct 20;39(4):629–45. doi: 10.1590/1678-4685-GMB-2015-0327 (PMC5127152; doi:10.1590/1678-4685-GMB-2015-0327)
Supplement: Supplementary file 7 [file 1415-4757-gmb-1678-4685-GMB-2015-0327-Suppl04.pdf]

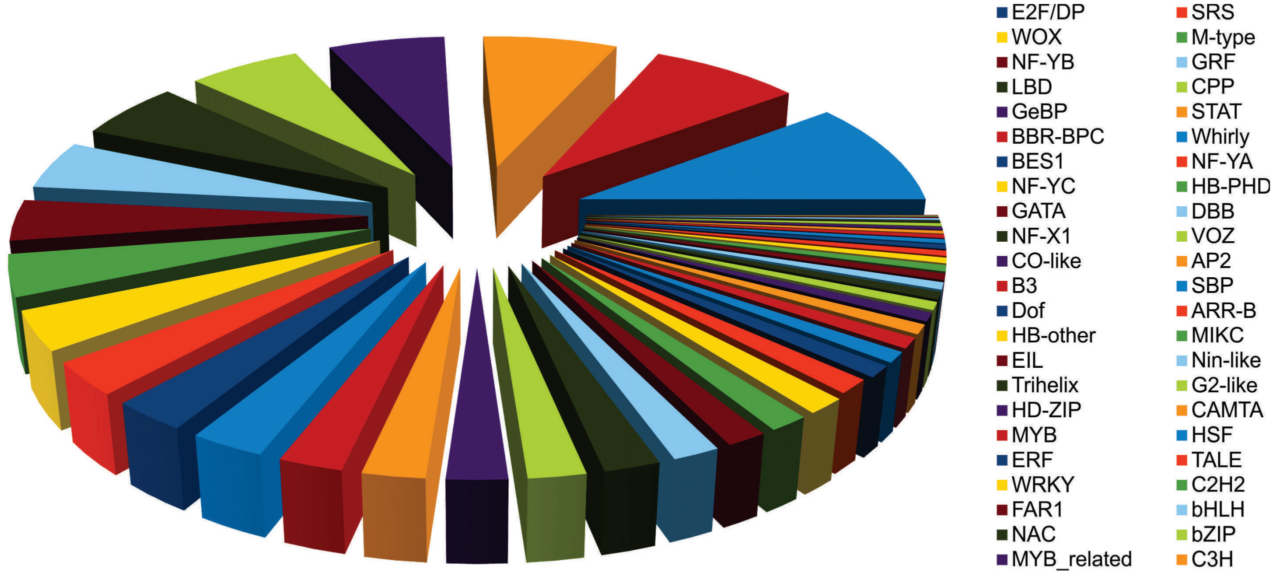

Figure S4 - Transcription factor distribution by family. Candidate genes were compared by similarity searches (cutoff 1e-100) against the Transcription Factor Database (TFDB). Among the TF families, GRAS, ARF, C3H, and MYB proteins were the most abundant ones.
