# Supplementary material for: Gene expression analysis reveals important pathways for drought response in leaves and roots of a wheat cultivar adapted to rainfed cropping in the Cerrado biome
Source: Genet Mol Biol. 2016 Oct 20;39(4):629–45. doi: 10.1590/1678-4685-GMB-2015-0327 (PMC5127152; doi:10.1590/1678-4685-GMB-2015-0327)
Supplement: Supplementary file 2 [file 1415-4757-gmb-1678-4685-GMB-2015-0327-Suppl05.pdf]

Table S1 - Set of 4,422 candidate genes identified in root and leaf tissues of the wheat cultivar MGS1 Aliança under drought.

Tissue	Identification	# of reads (stress)	# of reads (control)	Fold Change	p-value	Description
Root	Contig00003	73	13	2.360	1.32E-10	No hit
Root	Contig00045	41987	39888	-0.055	7.57E-09	SGD|S000028567 - symbol:RRT15 "Putative protein of unknown function" species:4932 "Saccharomyces cerevisiae" [GO:0006351 "transcription, DNA-dependent" evidence=IEA] [GO:0005575 "cellular_component" evidence=ND] [GO:0008150 "biological_process" evidence=ND] [GO:0006355 "regulation of transcription, DNA-dependent" evidence=IEA] [GO:0003674 "molecular_function" evidence=ND] SGD:S000028567 GO:GO:0006355 GO:GO:0006351 EMBL:BK006945 EMBL:U51921 RefSeq:NP_878128.1 ProteinModelPortal:Q3E811 STRING:Q3E811 EnsemblFungi:YLR162W-A GeneID:1466416 KEGG:sce:YLR162W-A CYGD:YLR162w-a GeneTree:ENSGT00530000064745 OMA:FYINCIM OrthoDB:EOG45TGXP NextBio:983475 ArrayExpress:Q3E811 Genevestigator:Q3E811 Uniprot:Q3E811
Root	Contig00046	62	3	4.240	1.60E-14	No hit
Root	Contig00054	764	863	-0.305	2.02E-05	CGD|CAL0002846 - symbol:CSA1 species:5476 "Candida albicans" [GO:0009277 "fungal-type cell wall" evidence=ISS;IDA] [GO:0009986 "cell surface" evidence=ISS;IDA] [GO:0020037 "heme binding" evidence=IGI] [GO:0006879 "cellular iron ion homeostasis" evidence=IMP] [GO:0005933 "cellular bud" evidence=IDA] [GO:0046658 "anchored to plasma membrane" evidence=ISS;IMP] [GO:0030446 "hyphal cell wall" evidence=IDA] [GO:0044011 "single-species biofilm formation on inanimate substrate" evidence=IMP] CGD:CAL0002846 EMBL:AACQ01000024 InterPro:IPR008427 Pfam:PF05730 RefSeq:XP_720404.1 RefSeq:XP_888643.1 GeneID:3638009 GeneID:3704110 KEGG:cal:CaO19.7114 KEGG:cal:CaO19_7114 InterPro:IPR014005 SMART:SM00747 Uniprot:G1UB63
Root	Contig00082	13319	12813	-0.073	3.05E-05	SGD|S000028422 - symbol:TAR1 "Mitochondrial protein potentially involved in respiratory metabolism" species:4932 "Saccharomyces cerevisiae" [GO:0003674 "molecular_function" evidence=ND] [GO:0043457 "regulation of cellular respiration" evidence=IGI] [GO:0005739 "mitochondrion" evidence=IEA;IMP] SGD:S000028422 GO:GO:0005739 EMBL:BK006945 EMBL:U53879 GO:GO:0043457 EMBL:Z73326 EMBL:AF479964 RefSeq:NP_690845.1 ProteinModelPortal:Q8TGM6 STRING:Q8TGM6 EnsemblFungi:YLR154W-C GeneID:1466405 KEGG:sce:YLR154W-C CYGD:YLR154w-c eggNOG:NOG79861 OMA:SCSKEHR OrthoDB:EOG466ZX8 NextBio:967146 Genevestigator:Q8TGM6 GermOnline:YLR154W-C Uniprot:Q8TGM6
Root	Contig00147	634	25	4.536	1.69E-137	No hit
Root	Contig00175	1927	101	4.125	0	No hit
Root	Contig00293	562	24	4.420	9.88E-121	No hit
Root	Contig00426	13	0	4.571	0.000344357	No hit
Root	Contig00436	40	2	4.193	7.78E-10	No hit
Root	Contig00447	34	7	2.151	3.42E-05	UNIPROTKB|P17931 - symbol:LGALS3 "Galectin-3" species:9606 "Homo sapiens" [GO:0001501 "skeletal system development" evidence=IEA] [GO:0005515 "protein binding" evidence=IPI] [GO:0005578 "proteinaceous extracellular matrix" evidence=IEA] [GO:0005634 "nucleus" evidence=IDA] [GO:0005681 "spliceosomal complex" evidence=IEA] [GO:0005737 "cytoplasm" evidence=IDA] [GO:0005743 "mitochondrial inner membrane" evidence=IDA] [GO:0005886 "plasma membrane" evidence=TAS] [GO:0006397 "mRNA processing" evidence=IEA] [GO:0008380 "RNA splicing" evidence=IEA] [GO:0019863 "IgE binding" evidence=IEA] [GO:0030154 "cell differentiation" evidence=IEA] [GO:0030198 "extracellular matrix organization" evidence=IEA] [GO:0031012 "extracellular matrix" evidence=IDA] [GO:0045087 "innate immune response" evidence=TAS] GO:GO:0005886 Reactome:REACT_6900 GO:GO:0030154 EMBL:CH471061 GO:GO:0008380 GO:GO:0005743 GO:GO:0005529 GO:GO:0006397 GO:GO:0045087 InterPro:IPR008985 InterPro:IPR013320 Gene3D:G3DSA:2.60.120.200 SUPFAM:SSF49899 HOVERGEN:HBG006255 InterPro:IPR001079 Pfam:PF00337 SMART:SM00908 SMART:SM00276 PROSITE:PS51304 GO:GO:0005681 Pathway_Interaction_DB:hedgehog_glipathway GO:GO:0019863 eggNOG:NOG312075 OrthoDB:EOG4C87TX InterPro:IPR015534 PANTHER:PTHR11346:SF26 EMBL:M57710 EMBL:M35368 EMBL:M36682 EMBL:M64303 EMBL:S59012 EMBL:AF031425 EMBL:AF031422 EMBL:AF031423 EMBL:AF031424 EMBL:AB006780 EMBL:AK314929 EMBL:CR456897 EMBL:AL139316 EMBL:BC001120 EMBL:BC053667 IPI:IPI00465431 PIR:A35820 RefSeq:NP_002297.2 UniGene:Hs.531081 PDB:1A3K PDB:1KJL PDB:1KJR PDB:2NMN PDB:2NMO PDB:2NN8 PDB:2XG3 PDB:3AYA PDB:3AYC PDB:3AYD PDB:3AYE PDB:3T1L PDB:3T1M PDB:3ZSJ PDB:3ZSK PDB:3ZSL PDB:3ZSM PDBsum:1A3K PDBsum:1KJL PDBsum:1KJR PDBsum:2NMN PDBsum:2NMO PDBsum:2NN8 PDBsum:2XG3 PDBsum:3AYA PDBsum:3AYC PDBsum:3AYD PDBsum:3AYE PDBsum:3T1L PDBsum:3T1M PDBsum:3ZSJ PDBsum:3ZSK PDBsum:3ZSL

Tissue	Identification	# of reads (stress)	# of reads (control)	Fold Change	p-value	Description
						PDBsum:3ZSM ProteinModelPortal:P17931 SMR:P17931 DIP:DIP-45623N IntAct:P17931 STRING:P17931 PhosphoSite:P17931 DMDM:215274262 DOSAC-COBS-2DPAGE:P17931 REPRODUCTION-2DPAGE:IPI00465431 UCD-2DPAGE:P17931 PRIDE:P17931 DNASU:3958 Ensembl:ENST00000254301 GeneID:3958 KEGG:hsa:3958 CTD:3958 GeneCards:GC14P055595 HGNC:HGNC:6563 HPA:CAB005191 HPA:HPA003162 MIM:153619 neXtProt:NX_P17931 PharmGKB:PA30340 GeneTree:ENSGT00630000089626 InParanoid:P17931 KO:K06831 OMA:QAPPGAY PhylomeDB:P17931 BindingDB:P17931 NextBio:15531 PMAP-CutDB:Q6IBA7 ArrayExpress:P17931 Bgee:P17931 CleanEx:HS_LGALS3 Genevestigator:P17931 GermOnline:ENSG00000131981 Uniprot:P17931
Root	Contig00455	36	0	6.041	1.05E-09	No hit
Root	Contig00496	31	3	3.240	8.04E-07	No hit
Root	Contig00498	60	2	4.778	8.85E-15	UNIPROTKB|P17931 - symbol:LGALS3 "Galectin-3" species:9606 "Homo sapiens" [GO:0001501 "skeletal system development" evidence=IEA] [GO:0005515 "protein binding" evidence=IPI] [GO:0005578 "proteinaceous extracellular matrix" evidence=IEA] [GO:0005634 "nucleus" evidence=IDA] [GO:0005681 "spliceosomal complex" evidence=IEA] [GO:0005737 "cytoplasm" evidence=IDA] [GO:0005743 "mitochondrial inner membrane" evidence=IDA] [GO:0005886 "plasma membrane" evidence=TAS] [GO:0006397 "mRNA processing" evidence=IEA] [GO:0008380 "RNA splicing" evidence=IEA] [GO:0019863 "IgE binding" evidence=IEA] [GO:0030154 "cell differentiation" evidence=IEA] [GO:0030198 "extracellular matrix organization" evidence=IEA] [GO:0031012 "extracellular matrix" evidence=IDA] [GO:0045087 "innate immune response" evidence=TAS] GO:GO:0005886 Reactome:REACT_6900 GO:GO:0030154 EMBL:CH471061 GO:GO:0008380 GO:GO:0005743 GO:GO:0005529 GO:GO:0006397 GO:GO:0045087 InterPro:IPR008985 InterPro:IPR013320 Gene3D:G3DSA:2.60.120.200 SUPFAM:SSF49899 HOVERGEN:HBG006255 InterPro:IPR001079 Pfam:PF00337 SMART:SM00908 SMART:SM00276 PROSITE:PS51304 GO:GO:0005681 Pathway_Interaction_DB:hedgehog_glipathway GO:GO:0019863 eggNOG:NOG312075 OrthoDB:EOG4C87TX InterPro:IPR015534 PANTHER:PTHR11346:SF26 EMBL:M57710 EMBL:M35368 EMBL:M36682 EMBL:M64303 EMBL:S59012 EMBL:AF031425 EMBL:AF031422 EMBL:AF031423 EMBL:AF031424 EMBL:AB006780 EMBL:AK314929 EMBL:CR456897 EMBL:AL139316 EMBL:BC001120 EMBL:BC053667 IPI:IPI00465431 PIR:A35820 RefSeq:NP_002297.2 UniGene:Hs.531081 PDB:1A3K PDB:1KJL PDB:1KJR PDB:2NMN PDB:2NMO PDB:2NN8 PDB:2XG3 PDB:3AYA PDB:3AYC PDB:3AYD PDB:3AYE PDB:3T1L PDB:3T1M PDB:3ZSJ PDB:3ZSK PDB:3ZSL PDB:3ZSM PDBsum:1A3K PDBsum:1KJL PDBsum:1KJR PDBsum:2NMN PDBsum:2NMO PDBsum:2NN8 PDBsum:2XG3 PDBsum:3AYA PDBsum:3AYC PDBsum:3AYD PDBsum:3AYE PDBsum:3T1L PDBsum:3T1M PDBsum:3ZSJ PDBsum:3ZSK PDBsum:3ZSL PDBsum:3ZSM ProteinModelPortal:P17931 SMR:P17931 DIP:DIP-45623N IntAct:P17931 STRING:P17931 PhosphoSite:P17931 DMDM:215274262 DOSAC-COBS-2DPAGE:P17931 REPRODUCTION-2DPAGE:IPI00465431 UCD-2DPAGE:P17931 PRIDE:P17931 DNASU:3958 Ensembl:ENST00000254301 GeneID:3958 KEGG:hsa:3958 CTD:3958 GeneCards:GC14P055595 HGNC:HGNC:6563 HPA:CAB005191 HPA:HPA003162 MIM:153619 neXtProt:NX_P17931 PharmGKB:PA30340 GeneTree:ENSGT00630000089626 InParanoid:P17931 KO:K06831 OMA:QAPPGAY PhylomeDB:P17931 BindingDB:P17931 NextBio:15531 PMAP-CutDB:Q6IBA7 ArrayExpress:P17931 Bgee:P17931 CleanEx:HS_LGALS3 Genevestigator:P17931 GermOnline:ENSG00000131981 Uniprot:P17931
Root	Contig00507	108	12	3.041	3.63E-19	UNIPROTKB|Q8NEZ4 - symbol:MLL3 "Histone-lysine N-methyltransferase MLL3" species:9606 "Homo sapiens" [GO:0003677 "DNA binding" evidence=IEA] [GO:0005515 "protein binding" evidence=IPI] [GO:0006351 "transcription, DNA-dependent" evidence=IEA] [GO:0006355 "regulation of transcription, DNA-dependent" evidence=IEA] [GO:0008270 "zinc ion binding" evidence=IEA] [GO:0035097 "histone methyltransferase complex" evidence=IEA] [GO:0035556 "intracellular signal transduction" evidence=IEA] [GO:0042800 "histone methyltransferase activity (H3-K4 specific)" evidence=IEA] InterPro:IPR001841 Pfam:PF00856 InterPro:IPR009071 InterPro:IPR000637 InterPro:IPR001214 InterPro:IPR001594 InterPro:IPR001965 InterPro:IPR002219 InterPro:IPR003888 InterPro:IPR003889 InterPro:IPR017956 InterPro:IPR019787 Pfam:PF00628 Pfam:PF05964 Pfam:PF05965 PROSITE:PS00354 PROSITE:PS50016 PROSITE:PS50089 PROSITE:PS50216 PROSITE:PS50280 PROSITE:PS51542 PROSITE:PS51543 SMART:SM00109 SMART:SM00184 SMART:SM00249 SMART:SM00317 SMART:SM00384 SMART:SM00398 SMART:SM00541 SMART:SM00542 InterPro:IPR011011 InterPro:IPR018516 InterPro:IPR018518 GO:GO:0035556 GO:GO:0005515 GO:GO:0006355 GO:GO:0003677 GO:GO:0008270 GO:GO:0006351 InterPro:IPR013083 Gene3D:G3DSA:3.30.40.10 SUPFAM:SSF57903 PROSITE:PS01359 eggNOG:COG2940 InterPro:IPR003616 SMART:SM00508

Tissue	Identification	# of reads (stress)	# of reads (control)	Fold Change	p-value	Description
						PROSITE:PS50868 EMBL:AC005631 EMBL:AC006017 GeneTree:ENSGT00650000093319 EMBL:AY024361 EMBL:AF264750 EMBL:AC104692 EMBL:AB040939 EMBL:AK022687 EMBL:AK075113 EMBL:AL833924 IPI:IPI00168806 IPI:IPI00375729 IPI:IPI00916332 RefSeq:NP_733751.2 UniGene:Hs.647120 PDB:2YSM PDB:2YUK PDB:3UVL PDBsum:2YSM PDBsum:2YUK PDBsum:3UVL ProteinModelPortal:Q8NEZ4 SMR:Q8NEZ4 DIP:DIP-48649N IntAct:Q8NEZ4 STRING:Q8NEZ4 PhosphoSite:Q8NEZ4 DMDM:221222521 PRIDE:Q8NEZ4 Ensembl:ENST00000262189 GeneID:58508 KEGG:hsa:58508 UCSC:uc003wla.1 CTD:58508 GeneCards:GC07M151832 H-InvDB:HIX0007238 HGNC:HGNC:13726 MIM:606833 neXtProt:NX_Q8NEZ4 PharmGKB:PA30847 HOVERGEN:HBG045586 InParanoid:Q8NEZ4 KO:K09188 OMA:DTSRQNK PhylomeDB:Q8NEZ4 NextBio:65023 ArrayExpress:Q8NEZ4 Bgee:Q8NEZ4 CleanEx:HS_MLL3 Genevestigator:Q8NEZ4 GermOnline:ENSG00000055609 Uniprot:Q8NEZ4
Root	Isotig00001	302	169	0.709	1.75E-07	TAIR|locus:2061310 - symbol:GUN1 "AT2G31400" species:3702 "Arabidopsis thaliana" [GO:0003677 "DNA binding" evidence=IDA] [GO:0009507 "chloroplast" evidence=IDA] [GO:0031930 "mitochondria-nucleus signaling pathway" evidence=IMP] [GO:0010019 "chloroplast-nucleus signaling pathway" evidence=IMP] InterPro:IPR011990 InterPro:IPR002885 INTERPRO:IPR002625 GO:GO:0009507 EMBL:CP002685 GenomeReviews:CT485783_GR GO:GO:0003677 GO:GO:0031930 Gene3D:G3DSA:1.25.40.10 EMBL:AC007169 PROSITE:PS51375 Pfam:PF01535 Pfam:PF13812 TIGRFAMs:TIGR00756 SMART:SM00463 PROSITE:PS50828 GO:GO:0010019 Pfam:PF13041 EMBL:BX819946 IPI:IPI00538422 PIR:C84720 RefSeq:NP_180698.1 UniGene:At.20815 ProteinModelPortal:Q9SIC9 SMR:Q9SIC9 PRIDE:Q9SIC9 EnsemblPlants:AT2G31400.1 GeneID:817698 KEGG:ath:AT2G31400 GeneFarm:4937 TAIR:At2g31400 eggNOG:NOG313285 HOGENOM:HBG319251 InParanoid:Q9SIC9 OMA:GNTVYAF PhylomeDB:Q9SIC9 ProtClustDB:CLSN2683142 ArrayExpress:Q9SIC9 Genevestigator:Q9SIC9 Uniprot:Q9SIC9
Root	Isotig00002	706	384	0.750	4.73E-17	TAIR|locus:2061310 - symbol:GUN1 "AT2G31400" species:3702 "Arabidopsis thaliana" [GO:0003677 "DNA binding" evidence=IDA] [GO:0009507 "chloroplast" evidence=IDA] [GO:0031930 "mitochondria-nucleus signaling pathway" evidence=IMP] [GO:0010019 "chloroplast-nucleus signaling pathway" evidence=IMP] InterPro:IPR011990 InterPro:IPR002885 INTERPRO:IPR002625 GO:GO:0009507 EMBL:CP002685 GenomeReviews:CT485783_GR GO:GO:0003677 GO:GO:0031930 Gene3D:G3DSA:1.25.40.10 EMBL:AC007169 PROSITE:PS51375 Pfam:PF01535 Pfam:PF13812 TIGRFAMs:TIGR00756 SMART:SM00463 PROSITE:PS50828 GO:GO:0010019 Pfam:PF13041 EMBL:BX819946 IPI:IPI00538422 PIR:C84720 RefSeq:NP_180698.1 UniGene:At.20815 ProteinModelPortal:Q9SIC9 SMR:Q9SIC9 PRIDE:Q9SIC9 EnsemblPlants:AT2G31400.1 GeneID:817698 KEGG:ath:AT2G31400 GeneFarm:4937 TAIR:At2g31400 eggNOG:NOG313285 HOGENOM:HBG319251 InParanoid:Q9SIC9 OMA:GNTVYAF PhylomeDB:Q9SIC9 ProtClustDB:CLSN2683142 ArrayExpress:Q9SIC9 Genevestigator:Q9SIC9 Uniprot:Q9SIC9
Root	Isotig00003	767	92	2.931	2.11E-121	TAIR|locus:2061310 - symbol:GUN1 "AT2G31400" species:3702 "Arabidopsis thaliana" [GO:0003677 "DNA binding" evidence=IDA] [GO:0009507 "chloroplast" evidence=IDA] [GO:0031930 "mitochondria-nucleus signaling pathway" evidence=IMP] [GO:0010019 "chloroplast-nucleus signaling pathway" evidence=IMP] InterPro:IPR011990 InterPro:IPR002885 INTERPRO:IPR002625 GO:GO:0009507 EMBL:CP002685 GenomeReviews:CT485783_GR GO:GO:0003677 GO:GO:0031930 Gene3D:G3DSA:1.25.40.10 EMBL:AC007169 PROSITE:PS51375 Pfam:PF01535 Pfam:PF13812 TIGRFAMs:TIGR00756 SMART:SM00463 PROSITE:PS50828 GO:GO:0010019 Pfam:PF13041 EMBL:BX819946 IPI:IPI00538422 PIR:C84720 RefSeq:NP_180698.1 UniGene:At.20815 ProteinModelPortal:Q9SIC9 SMR:Q9SIC9 PRIDE:Q9SIC9 EnsemblPlants:AT2G31400.1 GeneID:817698 KEGG:ath:AT2G31400 GeneFarm:4937 TAIR:At2g31400 eggNOG:NOG313285 HOGENOM:HBG319251 InParanoid:Q9SIC9 OMA:GNTVYAF PhylomeDB:Q9SIC9 ProtClustDB:CLSN2683142 ArrayExpress:Q9SIC9 Genevestigator:Q9SIC9 Uniprot:Q9SIC9
Root	Isotig00004	767	92	2.931	2.11E-121	TAIR|locus:2061310 - symbol:GUN1 "AT2G31400" species:3702 "Arabidopsis thaliana" [GO:0003677 "DNA binding" evidence=IDA] [GO:0009507 "chloroplast" evidence=IDA] [GO:0031930 "mitochondria-nucleus signaling pathway" evidence=IMP] [GO:0010019 "chloroplast-nucleus signaling pathway" evidence=IMP] InterPro:IPR011990 InterPro:IPR002885 INTERPRO:IPR002625 GO:GO:0009507 EMBL:CP002685 GenomeReviews:CT485783_GR GO:GO:0003677 GO:GO:0031930 Gene3D:G3DSA:1.25.40.10 EMBL:AC007169 PROSITE:PS51375 Pfam:PF01535 Pfam:PF13812 TIGRFAMs:TIGR00756 SMART:SM00463 PROSITE:PS50828 GO:GO:0010019 Pfam:PF13041 EMBL:BX819946 IPI:IPI00538422 PIR:C84720 RefSeq:NP_180698.1 UniGene:At.20815 ProteinModelPortal:Q9SIC9 SMR:Q9SIC9 PRIDE:Q9SIC9 EnsemblPlants:AT2G31400.1 GeneID:817698 KEGG:ath:AT2G31400 GeneFarm:4937 TAIR:At2g31400 eggNOG:NOG313285 HOGENOM:HBG319251

Tissue	Identification	# of reads (stress)	# of reads (control)	Fold Change	p-value	Description
						InParanoid:Q9SIC9 OMA:GNTVYAF PhylomeDB:Q9SIC9 ProtClustDB:CLSN2683142 ArrayExpress:Q9SIC9 Genevestigator:Q9SIC9 Uniprot:Q9SIC9
Root	Isotig00005	767	92	2.931	2.11E-121	TAIR|locus:2061310 - symbol:GUN1 "AT2G31400" species:3702 "Arabidopsis thaliana" [GO:0003677 "DNA binding" evidence=IDA] [GO:0009507 "chloroplast" evidence=IDA] [GO:0031930 "mitochondria-nucleus signaling pathway" evidence=IMP] [GO:0010019 "chloroplast-nucleus signaling pathway" evidence=IMP] InterPro:IPR011990 InterPro:IPR002885 INTERPRO:IPR002625 GO:GO:0009507 EMBL:CP002685 GenomeReviews:CT485783_GR GO:GO:0003677 GO:GO:0031930 Gene3D:G3DSA:1.25.40.10 EMBL:AC007169 PROSITE:PS51375 Pfam:PF01535 Pfam:PF13812 TIGRFAMs:TIGR00756 SMART:SM00463 PROSITE:PS50828 GO:GO:0010019 Pfam:PF13041 EMBL:BX819946 IPI:IPI00538422 PIR:C84720 RefSeq:NP_180698.1 UniGene:At.20815 ProteinModelPortal:Q9SIC9 SMR:Q9SIC9 PRIDE:Q9SIC9 EnsemblPlants:AT2G31400.1 GeneID:817698 KEGG:ath:AT2G31400 GeneFarm:4937 TAIR:At2g31400 eggNOG:NOG313285 HOGENOM:HBG319251 InParanoid:Q9SIC9 OMA:GNTVYAF PhylomeDB:Q9SIC9 ProtClustDB:CLSN2683142 ArrayExpress:Q9SIC9 Genevestigator:Q9SIC9 Uniprot:Q9SIC9
Root	Isotig00006	766	92	2.929	3.59E-121	TAIR|locus:2061310 - symbol:GUN1 "AT2G31400" species:3702 "Arabidopsis thaliana" [GO:0003677 "DNA binding" evidence=IDA] [GO:0009507 "chloroplast" evidence=IDA] [GO:0031930 "mitochondria-nucleus signaling pathway" evidence=IMP] [GO:0010019 "chloroplast-nucleus signaling pathway" evidence=IMP] InterPro:IPR011990 InterPro:IPR002885 INTERPRO:IPR002625 GO:GO:0009507 EMBL:CP002685 GenomeReviews:CT485783_GR GO:GO:0003677 GO:GO:0031930 Gene3D:G3DSA:1.25.40.10 EMBL:AC007169 PROSITE:PS51375 Pfam:PF01535 Pfam:PF13812 TIGRFAMs:TIGR00756 SMART:SM00463 PROSITE:PS50828 GO:GO:0010019 Pfam:PF13041 EMBL:BX819946 IPI:IPI00538422 PIR:C84720 RefSeq:NP_180698.1 UniGene:At.20815 ProteinModelPortal:Q9SIC9 SMR:Q9SIC9 PRIDE:Q9SIC9 EnsemblPlants:AT2G31400.1 GeneID:817698 KEGG:ath:AT2G31400 GeneFarm:4937 TAIR:At2g31400 eggNOG:NOG313285 HOGENOM:HBG319251 InParanoid:Q9SIC9 OMA:GNTVYAF PhylomeDB:Q9SIC9 ProtClustDB:CLSN2683142 ArrayExpress:Q9SIC9 Genevestigator:Q9SIC9 Uniprot:Q9SIC9
Root	Isotig00007	766	92	2.929	3.59E-121	TAIR|locus:2061310 - symbol:GUN1 "AT2G31400" species:3702 "Arabidopsis thaliana" [GO:0003677 "DNA binding" evidence=IDA] [GO:0009507 "chloroplast" evidence=IDA] [GO:0031930 "mitochondria-nucleus signaling pathway" evidence=IMP] [GO:0010019 "chloroplast-nucleus signaling pathway" evidence=IMP] InterPro:IPR011990 InterPro:IPR002885 INTERPRO:IPR002625 GO:GO:0009507 EMBL:CP002685 GenomeReviews:CT485783_GR GO:GO:0003677 GO:GO:0031930 Gene3D:G3DSA:1.25.40.10 EMBL:AC007169 PROSITE:PS51375 Pfam:PF01535 Pfam:PF13812 TIGRFAMs:TIGR00756 SMART:SM00463 PROSITE:PS50828 GO:GO:0010019 Pfam:PF13041 EMBL:BX819946 IPI:IPI00538422 PIR:C84720 RefSeq:NP_180698.1 UniGene:At.20815 ProteinModelPortal:Q9SIC9 SMR:Q9SIC9 PRIDE:Q9SIC9 EnsemblPlants:AT2G31400.1 GeneID:817698 KEGG:ath:AT2G31400 GeneFarm:4937 TAIR:At2g31400 eggNOG:NOG313285 HOGENOM:HBG319251 InParanoid:Q9SIC9 OMA:GNTVYAF PhylomeDB:Q9SIC9 ProtClustDB:CLSN2683142 ArrayExpress:Q9SIC9 Genevestigator:Q9SIC9 Uniprot:Q9SIC9
Root	Isotig00008	591	44	3.619	1.75E-112	TAIR|locus:2061310 - symbol:GUN1 "AT2G31400" species:3702 "Arabidopsis thaliana" [GO:0003677 "DNA binding" evidence=IDA] [GO:0009507 "chloroplast" evidence=IDA] [GO:0031930 "mitochondria-nucleus signaling pathway" evidence=IMP] [GO:0010019 "chloroplast-nucleus signaling pathway" evidence=IMP] InterPro:IPR011990 InterPro:IPR002885 INTERPRO:IPR002625 GO:GO:0009507 EMBL:CP002685 GenomeReviews:CT485783_GR GO:GO:0003677 GO:GO:0031930 Gene3D:G3DSA:1.25.40.10 EMBL:AC007169 PROSITE:PS51375 Pfam:PF01535 Pfam:PF13812 TIGRFAMs:TIGR00756 SMART:SM00463 PROSITE:PS50828 GO:GO:0010019 Pfam:PF13041 EMBL:BX819946 IPI:IPI00538422 PIR:C84720 RefSeq:NP_180698.1 UniGene:At.20815 ProteinModelPortal:Q9SIC9 SMR:Q9SIC9 PRIDE:Q9SIC9 EnsemblPlants:AT2G31400.1 GeneID:817698 KEGG:ath:AT2G31400 GeneFarm:4937 TAIR:At2g31400 eggNOG:NOG313285 HOGENOM:HBG319251 InParanoid:Q9SIC9 OMA:GNTVYAF PhylomeDB:Q9SIC9 ProtClustDB:CLSN2683142 ArrayExpress:Q9SIC9 Genevestigator:Q9SIC9 Uniprot:Q9SIC9
Root	Isotig00009	766	92	2.929	3.59E-121	TAIR|locus:2061310 - symbol:GUN1 "AT2G31400" species:3702 "Arabidopsis thaliana" [GO:0003677 "DNA binding" evidence=IDA] [GO:0009507 "chloroplast" evidence=IDA] [GO:0031930 "mitochondria-nucleus signaling pathway" evidence=IMP] [GO:0010019 "chloroplast-nucleus signaling pathway" evidence=IMP] InterPro:IPR011990 InterPro:IPR002885 INTERPRO:IPR002625 GO:GO:0009507 EMBL:CP002685 GenomeReviews:CT485783_GR GO:GO:0003677 GO:GO:0031930 Gene3D:G3DSA:1.25.40.10 EMBL:AC007169 PROSITE:PS51375 Pfam:PF01535 Pfam:PF13812 TIGRFAMs:TIGR00756 SMART:SM00463 PROSITE:PS50828 GO:GO:0010019

Tissue	Identification	# of reads (stress)	# of reads (control)	Fold Change	p-value	Description
						Pfam:PF13041 EMBL:BX819946 IPI:IPI00538422 PIR:C84720 RefSeq:NP_180698.1 UniGene:At.20815 ProteinModelPortal:Q9SIC9 SMR:Q9SIC9 PRIDE:Q9SIC9 EnsemblPlants:AT2G31400.1 GeneID:817698 KEGG:ath:AT2G31400 GeneFarm:4937 TAIR:At2g31400 eggNOG:NOG313285 HOGENOM:HBG319251 InParanoid:Q9SIC9 OMA:GNTVYAF PhylomeDB:Q9SIC9 ProtClustDB:CLSN2683142 ArrayExpress:Q9SIC9 Genevestigator:Q9SIC9 Uniprot:Q9SIC9
Root	Isotig00010	181	240	-0.536	0.000147189	TAIR|locus:2061310 - symbol:GUN1 "AT2G31400" species:3702 "Arabidopsis thaliana" [GO:0003677 "DNA binding" evidence=IDA] [GO:0009507 "chloroplast" evidence=IDA] [GO:0031930 "mitochondria-nucleus signaling pathway" evidence=IMP] [GO:0010019 "chloroplast-nucleus signaling pathway" evidence=IMP] InterPro:IPR011990 InterPro:IPR002885 INTERPRO:IPR002625 GO:GO:0009507 EMBL:CP002685 GenomeReviews:CT485783_GR GO:GO:0003677 GO:GO:0031930 Gene3D:G3DSA:1.25.40.10 EMBL:AC007169 PROSITE:PS51375 Pfam:PF01535 Pfam:PF13812 TIGRFAMs:TIGR00756 SMART:SM00463 PROSITE:PS50828 GO:GO:0010019 Pfam:PF13041 EMBL:BX819946 IPI:IPI00538422 PIR:C84720 RefSeq:NP_180698.1 UniGene:At.20815 ProteinModelPortal:Q9SIC9 SMR:Q9SIC9 PRIDE:Q9SIC9 EnsemblPlants:AT2G31400.1 GeneID:817698 KEGG:ath:AT2G31400 GeneFarm:4937 TAIR:At2g31400 eggNOG:NOG313285 HOGENOM:HBG319251 InParanoid:Q9SIC9 OMA:GNTVYAF PhylomeDB:Q9SIC9 ProtClustDB:CLSN2683142 ArrayExpress:Q9SIC9 Genevestigator:Q9SIC9 Uniprot:Q9SIC9
Root	Isotig00011	181	240	-0.536	0.000147189	TAIR|locus:2061310 - symbol:GUN1 "AT2G31400" species:3702 "Arabidopsis thaliana" [GO:0003677 "DNA binding" evidence=IDA] [GO:0009507 "chloroplast" evidence=IDA] [GO:0031930 "mitochondria-nucleus signaling pathway" evidence=IMP] [GO:0010019 "chloroplast-nucleus signaling pathway" evidence=IMP] InterPro:IPR011990 InterPro:IPR002885 INTERPRO:IPR002625 GO:GO:0009507 EMBL:CP002685 GenomeReviews:CT485783_GR GO:GO:0003677 GO:GO:0031930 Gene3D:G3DSA:1.25.40.10 EMBL:AC007169 PROSITE:PS51375 Pfam:PF01535 Pfam:PF13812 TIGRFAMs:TIGR00756 SMART:SM00463 PROSITE:PS50828 GO:GO:0010019 Pfam:PF13041 EMBL:BX819946 IPI:IPI00538422 PIR:C84720 RefSeq:NP_180698.1 UniGene:At.20815 ProteinModelPortal:Q9SIC9 SMR:Q9SIC9 PRIDE:Q9SIC9 EnsemblPlants:AT2G31400.1 GeneID:817698 KEGG:ath:AT2G31400 GeneFarm:4937 TAIR:At2g31400 eggNOG:NOG313285 HOGENOM:HBG319251 InParanoid:Q9SIC9 OMA:GNTVYAF PhylomeDB:Q9SIC9 ProtClustDB:CLSN2683142 ArrayExpress:Q9SIC9 Genevestigator:Q9SIC9 Uniprot:Q9SIC9
Root	Isotig00012	616	79	2.834	1.03E-94	TAIR|locus:2061310 - symbol:GUN1 "AT2G31400" species:3702 "Arabidopsis thaliana" [GO:0003677 "DNA binding" evidence=IDA] [GO:0009507 "chloroplast" evidence=IDA] [GO:0031930 "mitochondria-nucleus signaling pathway" evidence=IMP] [GO:0010019 "chloroplast-nucleus signaling pathway" evidence=IMP] InterPro:IPR011990 InterPro:IPR002885 INTERPRO:IPR002625 GO:GO:0009507 EMBL:CP002685 GenomeReviews:CT485783_GR GO:GO:0003677 GO:GO:0031930 Gene3D:G3DSA:1.25.40.10 EMBL:AC007169 PROSITE:PS51375 Pfam:PF01535 Pfam:PF13812 TIGRFAMs:TIGR00756 SMART:SM00463 PROSITE:PS50828 GO:GO:0010019 Pfam:PF13041 EMBL:BX819946 IPI:IPI00538422 PIR:C84720 RefSeq:NP_180698.1 UniGene:At.20815 ProteinModelPortal:Q9SIC9 SMR:Q9SIC9 PRIDE:Q9SIC9 EnsemblPlants:AT2G31400.1 GeneID:817698 KEGG:ath:AT2G31400 GeneFarm:4937 TAIR:At2g31400 eggNOG:NOG313285 HOGENOM:HBG319251 InParanoid:Q9SIC9 OMA:GNTVYAF PhylomeDB:Q9SIC9 ProtClustDB:CLSN2683142 ArrayExpress:Q9SIC9 Genevestigator:Q9SIC9 Uniprot:Q9SIC9
Root	Isotig00013	616	79	2.834	1.03E-94	TAIR|locus:2061310 - symbol:GUN1 "AT2G31400" species:3702 "Arabidopsis thaliana" [GO:0003677 "DNA binding" evidence=IDA] [GO:0009507 "chloroplast" evidence=IDA] [GO:0031930 "mitochondria-nucleus signaling pathway" evidence=IMP] [GO:0010019 "chloroplast-nucleus signaling pathway" evidence=IMP] InterPro:IPR011990 InterPro:IPR002885 INTERPRO:IPR002625 GO:GO:0009507 EMBL:CP002685 GenomeReviews:CT485783_GR GO:GO:0003677 GO:GO:0031930 Gene3D:G3DSA:1.25.40.10 EMBL:AC007169 PROSITE:PS51375 Pfam:PF01535 Pfam:PF13812 TIGRFAMs:TIGR00756 SMART:SM00463 PROSITE:PS50828 GO:GO:0010019 Pfam:PF13041 EMBL:BX819946 IPI:IPI00538422 PIR:C84720 RefSeq:NP_180698.1 UniGene:At.20815 ProteinModelPortal:Q9SIC9 SMR:Q9SIC9 PRIDE:Q9SIC9 EnsemblPlants:AT2G31400.1 GeneID:817698 KEGG:ath:AT2G31400 GeneFarm:4937 TAIR:At2g31400 eggNOG:NOG313285 HOGENOM:HBG319251 InParanoid:Q9SIC9 OMA:GNTVYAF PhylomeDB:Q9SIC9 ProtClustDB:CLSN2683142 ArrayExpress:Q9SIC9 Genevestigator:Q9SIC9 Uniprot:Q9SIC9
Root	Isotig00014	616	79	2.834	1.03E-94	TAIR|locus:2061310 - symbol:GUN1 "AT2G31400" species:3702 "Arabidopsis thaliana" [GO:0003677 "DNA binding" evidence=IDA] [GO:0009507 "chloroplast" evidence=IDA] [GO:0031930 "mitochondria-nucleus signaling pathway" evidence=IMP] [GO:0010019 "chloroplast-nucleus signaling pathway" evidence=IMP] InterPro:IPR011990

Tissue	Identification	# of reads (stress)	# of reads (control)	Fold Change	p-value	Description
						InterPro:IPR002885 INTERPRO:IPR002625 GO:GO:0009507 EMBL:CP002685 GenomeReviews:CT485783_GR GO:GO:0003677 GO:GO:0031930 Gene3D:G3DSA:1.25.40.10 EMBL:AC007169 PROSITE:PS51375 Pfam:PF01535 Pfam:PF13812 TIGRFAMs:TIGR00756 SMART:SM00463 PROSITE:PS50828 GO:GO:0010019 Pfam:PF13041 EMBL:BX819946 IPI:IPI00538422 PIR:C84720 RefSeq:NP_180698.1 UniGene:At.20815 ProteinModelPortal:Q9SIC9 SMR:Q9SIC9 PRIDE:Q9SIC9 EnsemblPlants:AT2G31400.1 GeneID:817698 KEGG:ath:AT2G31400 GeneFarm:4937 TAIR:At2g31400 eggNOG:NOG313285 HOGENOM:HBG319251 InParanoid:Q9SIC9 OMA:GNTVYAF PhylomeDB:Q9SIC9 ProtClustDB:CLSN2683142 ArrayExpress:Q9SIC9 Genevestigator:Q9SIC9 Uniprot:Q9SIC9
Root	Isotig00015	182	60	1.472	1.59E-13	TAIR|locus:2061310 - symbol:GUN1 "AT2G31400" species:3702 "Arabidopsis thaliana" [GO:0003677 "DNA binding" evidence=IDA] [GO:0009507 "chloroplast" evidence=IDA] [GO:0031930 "mitochondria-nucleus signaling pathway" evidence=IMP] [GO:0010019 "chloroplast-nucleus signaling pathway" evidence=IMP] InterPro:IPR011990 InterPro:IPR002885 INTERPRO:IPR002625 GO:GO:0009507 EMBL:CP002685 GenomeReviews:CT485783_GR GO:GO:0003677 GO:GO:0031930 Gene3D:G3DSA:1.25.40.10 EMBL:AC007169 PROSITE:PS51375 Pfam:PF01535 Pfam:PF13812 TIGRFAMs:TIGR00756 SMART:SM00463 PROSITE:PS50828 GO:GO:0010019 Pfam:PF13041 EMBL:BX819946 IPI:IPI00538422 PIR:C84720 RefSeq:NP_180698.1 UniGene:At.20815 ProteinModelPortal:Q9SIC9 SMR:Q9SIC9 PRIDE:Q9SIC9 EnsemblPlants:AT2G31400.1 GeneID:817698 KEGG:ath:AT2G31400 GeneFarm:4937 TAIR:At2g31400 eggNOG:NOG313285 HOGENOM:HBG319251 InParanoid:Q9SIC9 OMA:GNTVYAF PhylomeDB:Q9SIC9 ProtClustDB:CLSN2683142 ArrayExpress:Q9SIC9 Genevestigator:Q9SIC9 Uniprot:Q9SIC9
Root	Isotig00016	295	160	0.754	5.06E-08	SGD|S000028422 - symbol:TAR1 "Mitochondrial protein potentially involved in respiratory metabolism" species:4932 "Saccharomyces cerevisiae" [GO:0003674 "molecular_function" evidence=ND] [GO:0043457 "regulation of cellular respiration" evidence=IGI] [GO:0005739 "mitochondrion" evidence=IEA;IMP] SGD:S000028422 GO:GO:0005739 EMBL:BK006945 EMBL:U53879 GO:GO:0043457 EMBL:Z73326 EMBL:AF479964 RefSeq:NP_690845.1 ProteinModelPortal:Q8TGM6 STRING:Q8TGM6 EnsemblFungi:YLR154W-C GeneID:1466405 KEGG:sce:YLR154W-C CYGD:YLR154w-c eggNOG:NOG79861 OMA:SCSKEHR OrthoDB:EOG466ZX8 NextBio:967146 Genevestigator:Q8TGM6 GermOnline:YLR154W-C Uniprot:Q8TGM6
Root	Isotig00017	699	375	0.769	1.31E-17	SGD|S000028422 - symbol:TAR1 "Mitochondrial protein potentially involved in respiratory metabolism" species:4932 "Saccharomyces cerevisiae" [GO:0003674 "molecular_function" evidence=ND] [GO:0043457 "regulation of cellular respiration" evidence=IGI] [GO:0005739 "mitochondrion" evidence=IEA;IMP] SGD:S000028422 GO:GO:0005739 EMBL:BK006945 EMBL:U53879 GO:GO:0043457 EMBL:Z73326 EMBL:AF479964 RefSeq:NP_690845.1 ProteinModelPortal:Q8TGM6 STRING:Q8TGM6 EnsemblFungi:YLR154W-C GeneID:1466405 KEGG:sce:YLR154W-C CYGD:YLR154w-c eggNOG:NOG79861 OMA:SCSKEHR OrthoDB:EOG466ZX8 NextBio:967146 Genevestigator:Q8TGM6 GermOnline:YLR154W-C Uniprot:Q8TGM6
Root	Isotig00018	760	83	3.066	1.49E-125	SGD|S000028422 - symbol:TAR1 "Mitochondrial protein potentially involved in respiratory metabolism" species:4932 "Saccharomyces cerevisiae" [GO:0003674 "molecular_function" evidence=ND] [GO:0043457 "regulation of cellular respiration" evidence=IGI] [GO:0005739 "mitochondrion" evidence=IEA;IMP] SGD:S000028422 GO:GO:0005739 EMBL:BK006945 EMBL:U53879 GO:GO:0043457 EMBL:Z73326 EMBL:AF479964 RefSeq:NP_690845.1 ProteinModelPortal:Q8TGM6 STRING:Q8TGM6 EnsemblFungi:YLR154W-C GeneID:1466405 KEGG:sce:YLR154W-C CYGD:YLR154w-c eggNOG:NOG79861 OMA:SCSKEHR OrthoDB:EOG466ZX8 NextBio:967146 Genevestigator:Q8TGM6 GermOnline:YLR154W-C Uniprot:Q8TGM6
Root	Isotig00019	760	83	3.066	1.49E-125	SGD|S000028422 - symbol:TAR1 "Mitochondrial protein potentially involved in respiratory metabolism" species:4932 "Saccharomyces cerevisiae" [GO:0003674 "molecular_function" evidence=ND] [GO:0043457 "regulation of cellular respiration" evidence=IGI] [GO:0005739 "mitochondrion" evidence=IEA;IMP] SGD:S000028422 GO:GO:0005739 EMBL:BK006945 EMBL:U53879 GO:GO:0043457 EMBL:Z73326 EMBL:AF479964 RefSeq:NP_690845.1 ProteinModelPortal:Q8TGM6 STRING:Q8TGM6 EnsemblFungi:YLR154W-C GeneID:1466405 KEGG:sce:YLR154W-C CYGD:YLR154w-c eggNOG:NOG79861 OMA:SCSKEHR OrthoDB:EOG466ZX8 NextBio:967146 Genevestigator:Q8TGM6 GermOnline:YLR154W-C Uniprot:Q8TGM6
Root	Isotig00020	760	83	3.066	1.49E-125	SGD|S000028422 - symbol:TAR1 "Mitochondrial protein potentially involved in respiratory metabolism" species:4932 "Saccharomyces cerevisiae" [GO:0003674 "molecular_function" evidence=ND] [GO:0043457 "regulation of cellular respiration" evidence=IGI] [GO:0005739 "mitochondrion" evidence=IEA;IMP] SGD:S000028422 GO:GO:0005739 EMBL:BK006945 EMBL:U53879 GO:GO:0043457 EMBL:Z73326 EMBL:AF479964 RefSeq:NP_690845.1 ProteinModelPortal:Q8TGM6 STRING:Q8TGM6 EnsemblFungi:YLR154W-

Tissue	Identification	# of reads (stress)	# of reads (control)	Fold Change	p-value	Description
						C GeneID:1466405 KEGG:sce:YLR154W-C CYGD:YLR154w-c eggNOG:NOG79861 OMA:SCSKEHR OrthoDB:EOG466ZX8 NextBio:967146 Genevestigator:Q8TGM6 GermOnline:YLR154W-C Uniprot:Q8TGM6
Root	Isotig00021	759	83	3.064	2.55E-125	SGD|S000028422 - symbol:TAR1 "Mitochondrial protein potentially involved in respiratory metabolism" species:4932 "Saccharomyces cerevisiae" [GO:0003674 "molecular_function" evidence=ND] [GO:0043457 "regulation of cellular respiration" evidence=IGI] [GO:0005739 "mitochondrion" evidence=IEA;IMP] SGD:S000028422 GO:GO:0005739 EMBL:BK006945 EMBL:U53879 GO:GO:0043457 EMBL:Z73326 EMBL:AF479964 RefSeq:NP_690845.1 ProteinModelPortal:Q8TGM6 STRING:Q8TGM6 EnsemblFungi:YLR154W-C GeneID:1466405 KEGG:sce:YLR154W-C CYGD:YLR154w-c eggNOG:NOG79861 OMA:SCSKEHR OrthoDB:EOG466ZX8 NextBio:967146 Genevestigator:Q8TGM6 GermOnline:YLR154W-C Uniprot:Q8TGM6
Root	Isotig00022	759	83	3.064	2.55E-125	SGD|S000028422 - symbol:TAR1 "Mitochondrial protein potentially involved in respiratory metabolism" species:4932 "Saccharomyces cerevisiae" [GO:0003674 "molecular_function" evidence=ND] [GO:0043457 "regulation of cellular respiration" evidence=IGI] [GO:0005739 "mitochondrion" evidence=IEA;IMP] SGD:S000028422 GO:GO:0005739 EMBL:BK006945 EMBL:U53879 GO:GO:0043457 EMBL:Z73326 EMBL:AF479964 RefSeq:NP_690845.1 ProteinModelPortal:Q8TGM6 STRING:Q8TGM6 EnsemblFungi:YLR154W-C GeneID:1466405 KEGG:sce:YLR154W-C CYGD:YLR154w-c eggNOG:NOG79861 OMA:SCSKEHR OrthoDB:EOG466ZX8 NextBio:967146 Genevestigator:Q8TGM6 GermOnline:YLR154W-C Uniprot:Q8TGM6
Root	Isotig00023	584	35	3.932	1.26E-117	SGD|S000028567 - symbol:RRT15 "Putative protein of unknown function" species:4932 "Saccharomyces cerevisiae" [GO:0006351 "transcription, DNA-dependent" evidence=IEA] [GO:0005575 "cellular_component" evidence=ND] [GO:0008150 "biological_process" evidence=ND] [GO:0006355 "regulation of transcription, DNA-dependent" evidence=IEA] [GO:0003674 "molecular_function" evidence=ND] SGD:S000028567 GO:GO:0006355 GO:GO:0006351 EMBL:BK006945 EMBL:U51921 RefSeq:NP_878128.1 ProteinModelPortal:Q3E811 STRING:Q3E811 EnsemblFungi:YLR162W-A GeneID:1466416 KEGG:sce:YLR162W-A CYGD:YLR162w-a GeneTree:ENSGT00530000064745 OMA:FYINCIM OrthoDB:EOG45TGXP NextBio:983475 ArrayExpress:Q3E811 Genevestigator:Q3E811 Uniprot:Q3E811
Root	Isotig00024	759	83	3.064	2.55E-125	SGD|S000028422 - symbol:TAR1 "Mitochondrial protein potentially involved in respiratory metabolism" species:4932 "Saccharomyces cerevisiae" [GO:0003674 "molecular_function" evidence=ND] [GO:0043457 "regulation of cellular respiration" evidence=IGI] [GO:0005739 "mitochondrion" evidence=IEA;IMP] SGD:S000028422 GO:GO:0005739 EMBL:BK006945 EMBL:U53879 GO:GO:0043457 EMBL:Z73326 EMBL:AF479964 RefSeq:NP_690845.1 ProteinModelPortal:Q8TGM6 STRING:Q8TGM6 EnsemblFungi:YLR154W-C GeneID:1466405 KEGG:sce:YLR154W-C CYGD:YLR154w-c eggNOG:NOG79861 OMA:SCSKEHR OrthoDB:EOG466ZX8 NextBio:967146 Genevestigator:Q8TGM6 GermOnline:YLR154W-C Uniprot:Q8TGM6
Root	Isotig00025	92	40	1.073	4.18E-05	TAIR|locus:2061310 - symbol:GUN1 "AT2G31400" species:3702 "Arabidopsis thaliana" [GO:0003677 "DNA binding" evidence=IDA] [GO:0009507 "chloroplast" evidence=IDA] [GO:0031930 "mitochondria-nucleus signaling pathway" evidence=IMP] [GO:0010019 "chloroplast-nucleus signaling pathway" evidence=IMP] InterPro:IPR011990 InterPro:IPR002885 INTERPRO:IPR002625 GO:GO:0009507 EMBL:CP002685 GenomeReviews:CT485783_GR GO:GO:0003677 GO:GO:0031930 Gene3D:G3DSA:1.25.40.10 EMBL:AC007169 PROSITE:PS51375 Pfam:PF01535 Pfam:PF13812 TIGRFAMs:TIGR00756 SMART:SM00463 PROSITE:PS50828 GO:GO:0010019 Pfam:PF13041 EMBL:BX819946 IPI:IPI00538422 PIR:C84720 RefSeq:NP_180698.1 UniGene:At.20815 ProteinModelPortal:Q9SIC9 SMR:Q9SIC9 PRIDE:Q9SIC9 EnsemblPlants:AT2G31400.1 GeneID:817698 KEGG:ath:AT2G31400 GeneFarm:4937 TAIR:At2g31400 eggNOG:NOG313285 HOGENOM:HBG319251 InParanoid:Q9SIC9 OMA:GNTVYAF PhylomeDB:Q9SIC9 ProtClustDB:CLSN2683142 ArrayExpress:Q9SIC9 Genevestigator:Q9SIC9 Uniprot:Q9SIC9
Root	Isotig00026	172	45	1.805	9.97E-17	TAIR|locus:2061310 - symbol:GUN1 "AT2G31400" species:3702 "Arabidopsis thaliana" [GO:0003677 "DNA binding" evidence=IDA] [GO:0009507 "chloroplast" evidence=IDA] [GO:0031930 "mitochondria-nucleus signaling pathway" evidence=IMP] [GO:0010019 "chloroplast-nucleus signaling pathway" evidence=IMP] InterPro:IPR011990 InterPro:IPR002885 INTERPRO:IPR002625 GO:GO:0009507 EMBL:CP002685 GenomeReviews:CT485783_GR GO:GO:0003677 GO:GO:0031930 Gene3D:G3DSA:1.25.40.10 EMBL:AC007169 PROSITE:PS51375 Pfam:PF01535 Pfam:PF13812 TIGRFAMs:TIGR00756 SMART:SM00463 PROSITE:PS50828 GO:GO:0010019 Pfam:PF13041 EMBL:BX819946 IPI:IPI00538422 PIR:C84720 RefSeq:NP_180698.1 UniGene:At.20815 ProteinModelPortal:Q9SIC9 SMR:Q9SIC9 PRIDE:Q9SIC9 EnsemblPlants:AT2G31400.1 GeneID:817698 KEGG:ath:AT2G31400 GeneFarm:4937 TAIR:At2g31400 eggNOG:NOG313285 HOGENOM:HBG319251

Tissue	Identification	# of reads (stress)	# of reads (control)	Fold Change	p-value	Description
						InParanoid:Q9SIC9 OMA:GNTVYAF PhylomeDB:Q9SIC9 ProtClustDB:CLSN2683142 ArrayExpress:Q9SIC9 Genevestigator:Q9SIC9 Uniprot:Q9SIC9
Root	Isotig00027	174	231	-0.538	0.000187739	SGD|S000028422 - symbol:TAR1 "Mitochondrial protein potentially involved in respiratory metabolism" species:4932 "Saccharomyces cerevisiae" [GO:0003674 "molecular_function" evidence=ND] [GO:0043457 "regulation of cellular respiration" evidence=IGI] [GO:0005739 "mitochondrion" evidence=IEA;IMP] SGD:S000028422 GO:GO:0005739 EMBL:BK006945 EMBL:U53879 GO:GO:0043457 EMBL:Z73326 EMBL:AF479964 RefSeq:NP_690845.1 ProteinModelPortal:Q8TGM6 STRING:Q8TGM6 EnsemblFungi:YLR154W-C GeneID:1466405 KEGG:sce:YLR154W-C CYGD:YLR154w-c eggNOG:NOG79861 OMA:SCSKEHR OrthoDB:EOG466ZX8 NextBio:967146 Genevestigator:Q8TGM6 GermOnline:YLR154W-C Uniprot:Q8TGM6
Root	Isotig00028	174	231	-0.538	0.000187739	SGD|S000028422 - symbol:TAR1 "Mitochondrial protein potentially involved in respiratory metabolism" species:4932 "Saccharomyces cerevisiae" [GO:0003674 "molecular_function" evidence=ND] [GO:0043457 "regulation of cellular respiration" evidence=IGI] [GO:0005739 "mitochondrion" evidence=IEA;IMP] SGD:S000028422 GO:GO:0005739 EMBL:BK006945 EMBL:U53879 GO:GO:0043457 EMBL:Z73326 EMBL:AF479964 RefSeq:NP_690845.1 ProteinModelPortal:Q8TGM6 STRING:Q8TGM6 EnsemblFungi:YLR154W-C GeneID:1466405 KEGG:sce:YLR154W-C CYGD:YLR154w-c eggNOG:NOG79861 OMA:SCSKEHR OrthoDB:EOG466ZX8 NextBio:967146 Genevestigator:Q8TGM6 GermOnline:YLR154W-C Uniprot:Q8TGM6
Root	Isotig00029	609	70	2.992	1.02E-98	SGD|S000028422 - symbol:TAR1 "Mitochondrial protein potentially involved in respiratory metabolism" species:4932 "Saccharomyces cerevisiae" [GO:0003674 "molecular_function" evidence=ND] [GO:0043457 "regulation of cellular respiration" evidence=IGI] [GO:0005739 "mitochondrion" evidence=IEA;IMP] SGD:S000028422 GO:GO:0005739 EMBL:BK006945 EMBL:U53879 GO:GO:0043457 EMBL:Z73326 EMBL:AF479964 RefSeq:NP_690845.1 ProteinModelPortal:Q8TGM6 STRING:Q8TGM6 EnsemblFungi:YLR154W-C GeneID:1466405 KEGG:sce:YLR154W-C CYGD:YLR154w-c eggNOG:NOG79861 OMA:SCSKEHR OrthoDB:EOG466ZX8 NextBio:967146 Genevestigator:Q8TGM6 GermOnline:YLR154W-C Uniprot:Q8TGM6
Root	Isotig00030	609	70	2.992	1.02E-98	SGD|S000028422 - symbol:TAR1 "Mitochondrial protein potentially involved in respiratory metabolism" species:4932 "Saccharomyces cerevisiae" [GO:0003674 "molecular_function" evidence=ND] [GO:0043457 "regulation of cellular respiration" evidence=IGI] [GO:0005739 "mitochondrion" evidence=IEA;IMP] SGD:S000028422 GO:GO:0005739 EMBL:BK006945 EMBL:U53879 GO:GO:0043457 EMBL:Z73326 EMBL:AF479964 RefSeq:NP_690845.1 ProteinModelPortal:Q8TGM6 STRING:Q8TGM6 EnsemblFungi:YLR154W-C GeneID:1466405 KEGG:sce:YLR154W-C CYGD:YLR154w-c eggNOG:NOG79861 OMA:SCSKEHR OrthoDB:EOG466ZX8 NextBio:967146 Genevestigator:Q8TGM6 GermOnline:YLR154W-C Uniprot:Q8TGM6
Root	Isotig00031	609	70	2.992	1.02E-98	SGD|S000028422 - symbol:TAR1 "Mitochondrial protein potentially involved in respiratory metabolism" species:4932 "Saccharomyces cerevisiae" [GO:0003674 "molecular_function" evidence=ND] [GO:0043457 "regulation of cellular respiration" evidence=IGI] [GO:0005739 "mitochondrion" evidence=IEA;IMP] SGD:S000028422 GO:GO:0005739 EMBL:BK006945 EMBL:U53879 GO:GO:0043457 EMBL:Z73326 EMBL:AF479964 RefSeq:NP_690845.1 ProteinModelPortal:Q8TGM6 STRING:Q8TGM6 EnsemblFungi:YLR154W-C GeneID:1466405 KEGG:sce:YLR154W-C CYGD:YLR154w-c eggNOG:NOG79861 OMA:SCSKEHR OrthoDB:EOG466ZX8 NextBio:967146 Genevestigator:Q8TGM6 GermOnline:YLR154W-C Uniprot:Q8TGM6
Root	Isotig00032	100	41	1.157	5.58E-06	TAIR|locus:2061310 - symbol:GUN1 "AT2G31400" species:3702 "Arabidopsis thaliana" [GO:0003677 "DNA binding" evidence=IDA] [GO:0009507 "chloroplast" evidence=IDA] [GO:0031930 "mitochondria-nucleus signaling pathway" evidence=IMP] [GO:0010019 "chloroplast-nucleus signaling pathway" evidence=IMP] InterPro:IPR011990 InterPro:IPR002885 INTERPRO:IPR002625 GO:GO:0009507 EMBL:CP002685 GenomeReviews:CT485783_GR GO:GO:0003677 GO:GO:0031930 Gene3D:G3DSA:1.25.40.10 EMBL:AC007169 PROSITE:PS51375 Pfam:PF01535 Pfam:PF13812 TIGRFAMs:TIGR00756 SMART:SM00463 PROSITE:PS50828 GO:GO:0010019 Pfam:PF13041 EMBL:BX819946 IPI:IPI00538422 PIR:C84720 RefSeq:NP_180698.1 UniGene:At.20815 ProteinModelPortal:Q9SIC9 SMR:Q9SIC9 PRIDE:Q9SIC9 EnsemblPlants:AT2G31400.1 GeneID:817698 KEGG:ath:AT2G31400 GeneFarm:4937 TAIR:At2g31400 eggNOG:NOG313285 HOGENOM:HBG319251 InParanoid:Q9SIC9 OMA:GNTVYAF PhylomeDB:Q9SIC9 ProtClustDB:CLSN2683142 ArrayExpress:Q9SIC9 Genevestigator:Q9SIC9 Uniprot:Q9SIC9
Root	Isotig00033	175	51	1.650	3.71E-15	SGD|S000028422 - symbol:TAR1 "Mitochondrial protein potentially involved in respiratory metabolism" species:4932 "Saccharomyces cerevisiae" [GO:0003674 "molecular_function" evidence=ND] [GO:0043457 "regulation of cellular respiration" evidence=IGI] [GO:0005739 "mitochondrion" evidence=IEA;IMP] SGD:S000028422 GO:GO:0005739 EMBL:BK006945 EMBL:U53879 GO:GO:0043457 EMBL:Z73326

Tissue	Identification	# of reads (stress)	# of reads (control)	Fold Change	p-value	Description
						EMBL:AF479964 RefSeq:NP_690845.1 ProteinModelPortal:Q8TGM6 STRING:Q8TGM6 EnsemblFungi:YLR154W-C GeneID:1466405 KEGG:sce:YLR154W-C CYGD:YLR154w-c eggNOG:NOG79861 OMA:SCSKEHR OrthoDB:EOG466ZX8 NextBio:967146 Genevestigator:Q8TGM6 GermOnline:YLR154W-C Uniprot:Q8TGM6
Root	Isotig00034	85	31	1.326	3.17E-06	SGD|S000028422 - symbol:TAR1 "Mitochondrial protein potentially involved in respiratory metabolism" species:4932 "Saccharomyces cerevisiae" [GO:0003674 "molecular_function" evidence=ND] [GO:0043457 "regulation of cellular respiration" evidence=IGI] [GO:0005739 "mitochondrion" evidence=IEA;IMP] SGD:S000028422 GO:GO:0005739 EMBL:BK006945 EMBL:U53879 GO:GO:0043457 EMBL:Z73326 EMBL:AF479964 RefSeq:NP_690845.1 ProteinModelPortal:Q8TGM6 STRING:Q8TGM6 EnsemblFungi:YLR154W-C GeneID:1466405 KEGG:sce:YLR154W-C CYGD:YLR154w-c eggNOG:NOG79861 OMA:SCSKEHR OrthoDB:EOG466ZX8 NextBio:967146 Genevestigator:Q8TGM6 GermOnline:YLR154W-C Uniprot:Q8TGM6
Root	Isotig00035	165	36	2.067	5.53E-19	SGD|S000028422 - symbol:TAR1 "Mitochondrial protein potentially involved in respiratory metabolism" species:4932 "Saccharomyces cerevisiae" [GO:0003674 "molecular_function" evidence=ND] [GO:0043457 "regulation of cellular respiration" evidence=IGI] [GO:0005739 "mitochondrion" evidence=IEA;IMP] SGD:S000028422 GO:GO:0005739 EMBL:BK006945 EMBL:U53879 GO:GO:0043457 EMBL:Z73326 EMBL:AF479964 RefSeq:NP_690845.1 ProteinModelPortal:Q8TGM6 STRING:Q8TGM6 EnsemblFungi:YLR154W-C GeneID:1466405 KEGG:sce:YLR154W-C CYGD:YLR154w-c eggNOG:NOG79861 OMA:SCSKEHR OrthoDB:EOG466ZX8 NextBio:967146 Genevestigator:Q8TGM6 GermOnline:YLR154W-C Uniprot:Q8TGM6
Root	Isotig00037	93	32	1.410	3.26E-07	SGD|S000028422 - symbol:TAR1 "Mitochondrial protein potentially involved in respiratory metabolism" species:4932 "Saccharomyces cerevisiae" [GO:0003674 "molecular_function" evidence=ND] [GO:0043457 "regulation of cellular respiration" evidence=IGI] [GO:0005739 "mitochondrion" evidence=IEA;IMP] SGD:S000028422 GO:GO:0005739 EMBL:BK006945 EMBL:U53879 GO:GO:0043457 EMBL:Z73326 EMBL:AF479964 RefSeq:NP_690845.1 ProteinModelPortal:Q8TGM6 STRING:Q8TGM6 EnsemblFungi:YLR154W-C GeneID:1466405 KEGG:sce:YLR154W-C CYGD:YLR154w-c eggNOG:NOG79861 OMA:SCSKEHR OrthoDB:EOG466ZX8 NextBio:967146 Genevestigator:Q8TGM6 GermOnline:YLR154W-C Uniprot:Q8TGM6
Root	Isotig00038	409	168	1.155	4.71E-20	No hit
Root	Isotig00039	419	177	1.114	1.88E-19	No hit
Root	Isotig00040	406	168	1.144	1.20E-19	No hit
Root	Isotig00041	410	168	1.158	3.45E-20	No hit
Root	Isotig00042	407	165	1.174	1.91E-20	No hit
Root	Isotig00043	416	177	1.104	4.66E-19	No hit
Root	Isotig00044	420	177	1.118	1.39E-19	No hit
Root	Isotig00045	417	174	1.132	7.96E-20	No hit
Root	Isotig00046	407	168	1.148	8.77E-20	No hit
Root	Isotig00047	404	165	1.163	4.90E-20	No hit
Root	Isotig00048	417	177	1.107	3.45E-19	No hit
Root	Isotig00049	414	174	1.122	1.99E-19	No hit
Root	Isotig00050	387	161	1.136	1.29E-18	SGD|S000028422 - symbol:TAR1 "Mitochondrial protein potentially involved in respiratory metabolism" species:4932 "Saccharomyces cerevisiae" [GO:0003674 "molecular_function" evidence=ND] [GO:0043457 "regulation of cellular respiration" evidence=IGI] [GO:0005739 "mitochondrion" evidence=IEA;IMP] SGD:S000028422 GO:GO:0005739 EMBL:BK006945 EMBL:U53879 GO:GO:0043457 EMBL:Z73326 EMBL:AF479964 RefSeq:NP_690845.1 ProteinModelPortal:Q8TGM6 STRING:Q8TGM6 EnsemblFungi:YLR154W-C GeneID:1466405 KEGG:sce:YLR154W-C CYGD:YLR154w-c eggNOG:NOG79861 OMA:SCSKEHR OrthoDB:EOG466ZX8 NextBio:967146 Genevestigator:Q8TGM6 GermOnline:YLR154W-C Uniprot:Q8TGM6
Root	Isotig00051	386	159	1.151	6.47E-19	SGD|S000028422 - symbol:TAR1 "Mitochondrial protein potentially involved in respiratory metabolism" species:4932 "Saccharomyces cerevisiae" [GO:0003674 "molecular_function" evidence=ND] [GO:0043457 "regulation of cellular respiration" evidence=IGI] [GO:0005739 "mitochondrion" evidence=IEA;IMP] SGD:S000028422 GO:GO:0005739 EMBL:BK006945 EMBL:U53879 GO:GO:0043457 EMBL:Z73326 EMBL:AF479964 RefSeq:NP_690845.1 ProteinModelPortal:Q8TGM6 STRING:Q8TGM6 EnsemblFungi:YLR154W-C GeneID:1466405 KEGG:sce:YLR154W-C CYGD:YLR154w-c eggNOG:NOG79861 OMA:SCSKEHR OrthoDB:EOG466ZX8 NextBio:967146 Genevestigator:Q8TGM6 GermOnline:YLR154W-C Uniprot:Q8TGM6
Root	Isotig00052	387	161	1.136	1.29E-18	No hit

Tissue	Identification	# of reads (stress)	# of reads (control)	Fold Change	p-value	Description
Root	Isotig00053	386	159	1.151	6.47E-19	No hit
Root	Isotig00054	397	170	1.095	4.94E-18	SGD|S000028422 - symbol:TAR1 "Mitochondrial protein potentially involved in respiratory metabolism" species:4932 "Saccharomyces cerevisiae" [GO:0003674 "molecular_function" evidence=ND] [GO:0043457 "regulation of cellular respiration" evidence=IGI] [GO:0005739 "mitochondrion" evidence=IEA;IMP] SGD:S000028422 GO:GO:0005739 EMBL:BK006945 EMBL:U53879 GO:GO:0043457 EMBL:Z73326 EMBL:AF479964 RefSeq:NP_690845.1 ProteinModelPortal:Q8TGM6 STRING:Q8TGM6 EnsemblFungi:YLR154W-C GeneID:1466405 KEGG:sce:YLR154W-C CYGD:YLR154w-c eggNOG:NOG79861 OMA:SCSKEHR OrthoDB:EOG466ZX8 NextBio:967146 Genevestigator:Q8TGM6 GermOnline:YLR154W-C Uniprot:Q8TGM6
Root	Isotig00055	396	168	1.108	2.56E-18	SGD|S000028422 - symbol:TAR1 "Mitochondrial protein potentially involved in respiratory metabolism" species:4932 "Saccharomyces cerevisiae" [GO:0003674 "molecular_function" evidence=ND] [GO:0043457 "regulation of cellular respiration" evidence=IGI] [GO:0005739 "mitochondrion" evidence=IEA;IMP] SGD:S000028422 GO:GO:0005739 EMBL:BK006945 EMBL:U53879 GO:GO:0043457 EMBL:Z73326 EMBL:AF479964 RefSeq:NP_690845.1 ProteinModelPortal:Q8TGM6 STRING:Q8TGM6 EnsemblFungi:YLR154W-C GeneID:1466405 KEGG:sce:YLR154W-C CYGD:YLR154w-c eggNOG:NOG79861 OMA:SCSKEHR OrthoDB:EOG466ZX8 NextBio:967146 Genevestigator:Q8TGM6 GermOnline:YLR154W-C Uniprot:Q8TGM6
Root	Isotig00056	397	170	1.095	4.94E-18	No hit
Root	Isotig00057	396	168	1.108	2.56E-18	No hit
Root	Isotig00058	388	161	1.140	9.49E-19	SGD|S000028422 - symbol:TAR1 "Mitochondrial protein potentially involved in respiratory metabolism" species:4932 "Saccharomyces cerevisiae" [GO:0003674 "molecular_function" evidence=ND] [GO:0043457 "regulation of cellular respiration" evidence=IGI] [GO:0005739 "mitochondrion" evidence=IEA;IMP] SGD:S000028422 GO:GO:0005739 EMBL:BK006945 EMBL:U53879 GO:GO:0043457 EMBL:Z73326 EMBL:AF479964 RefSeq:NP_690845.1 ProteinModelPortal:Q8TGM6 STRING:Q8TGM6 EnsemblFungi:YLR154W-C GeneID:1466405 KEGG:sce:YLR154W-C CYGD:YLR154w-c eggNOG:NOG79861 OMA:SCSKEHR OrthoDB:EOG466ZX8 NextBio:967146 Genevestigator:Q8TGM6 GermOnline:YLR154W-C Uniprot:Q8TGM6
Root	Isotig00059	385	158	1.156	5.33E-19	SGD|S000028422 - symbol:TAR1 "Mitochondrial protein potentially involved in respiratory metabolism" species:4932 "Saccharomyces cerevisiae" [GO:0003674 "molecular_function" evidence=ND] [GO:0043457 "regulation of cellular respiration" evidence=IGI] [GO:0005739 "mitochondrion" evidence=IEA;IMP] SGD:S000028422 GO:GO:0005739 EMBL:BK006945 EMBL:U53879 GO:GO:0043457 EMBL:Z73326 EMBL:AF479964 RefSeq:NP_690845.1 ProteinModelPortal:Q8TGM6 STRING:Q8TGM6 EnsemblFungi:YLR154W-C GeneID:1466405 KEGG:sce:YLR154W-C CYGD:YLR154w-c eggNOG:NOG79861 OMA:SCSKEHR OrthoDB:EOG466ZX8 NextBio:967146 Genevestigator:Q8TGM6 GermOnline:YLR154W-C Uniprot:Q8TGM6
Root	Isotig00060	387	159	1.154	4.74E-19	SGD|S000028422 - symbol:TAR1 "Mitochondrial protein potentially involved in respiratory metabolism" species:4932 "Saccharomyces cerevisiae" [GO:0003674 "molecular_function" evidence=ND] [GO:0043457 "regulation of cellular respiration" evidence=IGI] [GO:0005739 "mitochondrion" evidence=IEA;IMP] SGD:S000028422 GO:GO:0005739 EMBL:BK006945 EMBL:U53879 GO:GO:0043457 EMBL:Z73326 EMBL:AF479964 RefSeq:NP_690845.1 ProteinModelPortal:Q8TGM6 STRING:Q8TGM6 EnsemblFungi:YLR154W-C GeneID:1466405 KEGG:sce:YLR154W-C CYGD:YLR154w-c eggNOG:NOG79861 OMA:SCSKEHR OrthoDB:EOG466ZX8 NextBio:967146 Genevestigator:Q8TGM6 GermOnline:YLR154W-C Uniprot:Q8TGM6
Root	Isotig00061	384	156	1.171	2.63E-19	SGD|S000028422 - symbol:TAR1 "Mitochondrial protein potentially involved in respiratory metabolism" species:4932 "Saccharomyces cerevisiae" [GO:0003674 "molecular_function" evidence=ND] [GO:0043457 "regulation of cellular respiration" evidence=IGI] [GO:0005739 "mitochondrion" evidence=IEA;IMP] SGD:S000028422 GO:GO:0005739 EMBL:BK006945 EMBL:U53879 GO:GO:0043457 EMBL:Z73326 EMBL:AF479964 RefSeq:NP_690845.1 ProteinModelPortal:Q8TGM6 STRING:Q8TGM6 EnsemblFungi:YLR154W-C GeneID:1466405 KEGG:sce:YLR154W-C CYGD:YLR154w-c eggNOG:NOG79861 OMA:SCSKEHR OrthoDB:EOG466ZX8 NextBio:967146 Genevestigator:Q8TGM6 GermOnline:YLR154W-C Uniprot:Q8TGM6
Root	Isotig00062	388	161	1.140	9.49E-19	No hit
Root	Isotig00063	385	158	1.156	5.33E-19	No hit
Root	Isotig00064	387	159	1.154	4.74E-19	No hit
Root	Isotig00065	384	156	1.171	2.63E-19	No hit
Root	Isotig00066	398	170	1.098	3.66E-18	SGD|S000028422 - symbol:TAR1 "Mitochondrial protein potentially involved in respiratory metabolism" species:4932 "Saccharomyces cerevisiae" [GO:0003674 "molecular_function" evidence=ND] [GO:0043457 "regulation of cellular respiration" evidence=IGI] [GO:0005739 "mitochondrion" evidence=IEA;IMP]

Tissue	Identification	# of reads (stress)	# of reads (control)	Fold Change	p-value	Description
						SGD:S000028422 GO:GO:0005739 EMBL:BK006945 EMBL:U53879 GO:GO:0043457 EMBL:Z73326 EMBL:AF479964 RefSeq:NP_690845.1 ProteinModelPortal:Q8TGM6 STRING:Q8TGM6 EnsemblFungi:YLR154W-C GeneID:1466405 KEGG:sce:YLR154W-C CYGD:YLR154w-c eggNOG:NOG79861 OMA:SCSKEHR OrthoDB:EOG466ZX8 NextBio:967146 Genevestigator:Q8TGM6 GermOnline:YLR154W-C Uniprot:Q8TGM6
Root	Isotig00067	395	167	1.113	2.13E-18	SGD|S000028422 - symbol:TAR1 "Mitochondrial protein potentially involved in respiratory metabolism" species:4932 "Saccharomyces cerevisiae" [GO:0003674 "molecular_function" evidence=ND] [GO:0043457 "regulation of cellular respiration" evidence=IGI] [GO:0005739 "mitochondrion" evidence=IEA;IMP] SGD:S000028422 GO:GO:0005739 EMBL:BK006945 EMBL:U53879 GO:GO:0043457 EMBL:Z73326 EMBL:AF479964 RefSeq:NP_690845.1 ProteinModelPortal:Q8TGM6 STRING:Q8TGM6 EnsemblFungi:YLR154W-C GeneID:1466405 KEGG:sce:YLR154W-C CYGD:YLR154w-c eggNOG:NOG79861 OMA:SCSKEHR OrthoDB:EOG466ZX8 NextBio:967146 Genevestigator:Q8TGM6 GermOnline:YLR154W-C Uniprot:Q8TGM6
Root	Isotig00068	397	168	1.112	1.89E-18	SGD|S000028422 - symbol:TAR1 "Mitochondrial protein potentially involved in respiratory metabolism" species:4932 "Saccharomyces cerevisiae" [GO:0003674 "molecular_function" evidence=ND] [GO:0043457 "regulation of cellular respiration" evidence=IGI] [GO:0005739 "mitochondrion" evidence=IEA;IMP] SGD:S000028422 GO:GO:0005739 EMBL:BK006945 EMBL:U53879 GO:GO:0043457 EMBL:Z73326 EMBL:AF479964 RefSeq:NP_690845.1 ProteinModelPortal:Q8TGM6 STRING:Q8TGM6 EnsemblFungi:YLR154W-C GeneID:1466405 KEGG:sce:YLR154W-C CYGD:YLR154w-c eggNOG:NOG79861 OMA:SCSKEHR OrthoDB:EOG466ZX8 NextBio:967146 Genevestigator:Q8TGM6 GermOnline:YLR154W-C Uniprot:Q8TGM6
Root	Isotig00069	394	165	1.127	1.09E-18	SGD|S000028422 - symbol:TAR1 "Mitochondrial protein potentially involved in respiratory metabolism" species:4932 "Saccharomyces cerevisiae" [GO:0003674 "molecular_function" evidence=ND] [GO:0043457 "regulation of cellular respiration" evidence=IGI] [GO:0005739 "mitochondrion" evidence=IEA;IMP] SGD:S000028422 GO:GO:0005739 EMBL:BK006945 EMBL:U53879 GO:GO:0043457 EMBL:Z73326 EMBL:AF479964 RefSeq:NP_690845.1 ProteinModelPortal:Q8TGM6 STRING:Q8TGM6 EnsemblFungi:YLR154W-C GeneID:1466405 KEGG:sce:YLR154W-C CYGD:YLR154w-c eggNOG:NOG79861 OMA:SCSKEHR OrthoDB:EOG466ZX8 NextBio:967146 Genevestigator:Q8TGM6 GermOnline:YLR154W-C Uniprot:Q8TGM6
Root	Isotig00070	398	170	1.098	3.66E-18	No hit
Root	Isotig00071	395	167	1.113	2.13E-18	No hit
Root	Isotig00072	397	168	1.112	1.89E-18	No hit
Root	Isotig00073	394	165	1.127	1.09E-18	No hit
Root	Isotig00078	180	75	1.134	2.03E-09	No hit
Root	Isotig00079	190	84	1.049	7.45E-09	No hit
Root	Isotig00082	202	80	1.207	2.45E-11	SGD|S000028422 - symbol:TAR1 "Mitochondrial protein potentially involved in respiratory metabolism" species:4932 "Saccharomyces cerevisiae" [GO:0003674 "molecular_function" evidence=ND] [GO:0043457 "regulation of cellular respiration" evidence=IGI] [GO:0005739 "mitochondrion" evidence=IEA;IMP] SGD:S000028422 GO:GO:0005739 EMBL:BK006945 EMBL:U53879 GO:GO:0043457 EMBL:Z73326 EMBL:AF479964 RefSeq:NP_690845.1 ProteinModelPortal:Q8TGM6 STRING:Q8TGM6 EnsemblFungi:YLR154W-C GeneID:1466405 KEGG:sce:YLR154W-C CYGD:YLR154w-c eggNOG:NOG79861 OMA:SCSKEHR OrthoDB:EOG466ZX8 NextBio:967146 Genevestigator:Q8TGM6 GermOnline:YLR154W-C Uniprot:Q8TGM6
Root	Isotig00083	199	80	1.186	6.47E-11	No hit
Root	Isotig00084	180	73	1.173	7.29E-10	SGD|S000028422 - symbol:TAR1 "Mitochondrial protein potentially involved in respiratory metabolism" species:4932 "Saccharomyces cerevisiae" [GO:0003674 "molecular_function" evidence=ND] [GO:0043457 "regulation of cellular respiration" evidence=IGI] [GO:0005739 "mitochondrion" evidence=IEA;IMP] SGD:S000028422 GO:GO:0005739 EMBL:BK006945 EMBL:U53879 GO:GO:0043457 EMBL:Z73326 EMBL:AF479964 RefSeq:NP_690845.1 ProteinModelPortal:Q8TGM6 STRING:Q8TGM6 EnsemblFungi:YLR154W-C GeneID:1466405 KEGG:sce:YLR154W-C CYGD:YLR154w-c eggNOG:NOG79861 OMA:SCSKEHR OrthoDB:EOG466ZX8 NextBio:967146 Genevestigator:Q8TGM6 GermOnline:YLR154W-C Uniprot:Q8TGM6
Root	Isotig00085	179	71	1.205	3.49E-10	SGD|S000028422 - symbol:TAR1 "Mitochondrial protein potentially involved in respiratory metabolism" species:4932 "Saccharomyces cerevisiae" [GO:0003674 "molecular_function" evidence=ND] [GO:0043457 "regulation of cellular respiration" evidence=IGI] [GO:0005739 "mitochondrion" evidence=IEA;IMP] SGD:S000028422 GO:GO:0005739 EMBL:BK006945 EMBL:U53879 GO:GO:0043457 EMBL:Z73326 EMBL:AF479964 RefSeq:NP_690845.1 ProteinModelPortal:Q8TGM6 STRING:Q8TGM6 EnsemblFungi:YLR154W-

Tissue	Identification	# of reads (stress)	# of reads (control)	Fold Change	p-value	Description
						C GeneID:1466405 KEGG:sce:YLR154W-C CYGD:YLR154w-c eggNOG:NOG79861 OMA:SCSKEHR OrthoDB:EOG466ZX8 NextBio:967146 Genevestigator:Q8TGM6 GermOnline:YLR154W-C Uniprot:Q8TGM6
Root	Isotig00086	180	73	1.173	7.29E-10	No hit
Root	Isotig00087	179	71	1.205	3.49E-10	No hit
Root	Isotig00088	2770	3117	-0.299	1.40E-15	SGD|S000028422 - symbol:TAR1 "Mitochondrial protein potentially involved in respiratory metabolism" species:4932 "Saccharomyces cerevisiae" [GO:0003674 "molecular_function" evidence=ND] [GO:0043457 "regulation of cellular respiration" evidence=IGI] [GO:0005739 "mitochondrion" evidence=IEA;IMP] SGD:S000028422 GO:GO:0005739 EMBL:BK006945 EMBL:U53879 GO:GO:0043457 EMBL:Z73326 EMBL:AF479964 RefSeq:NP_690845.1 ProteinModelPortal:Q8TGM6 STRING:Q8TGM6 EnsemblFungi:YLR154W-C GeneID:1466405 KEGG:sce:YLR154W-C CYGD:YLR154w-c eggNOG:NOG79861 OMA:SCSKEHR OrthoDB:EOG466ZX8 NextBio:967146 Genevestigator:Q8TGM6 GermOnline:YLR154W-C Uniprot:Q8TGM6
Root	Isotig00089	2770	3116	-0.299	1.55E-15	SGD|S000028422 - symbol:TAR1 "Mitochondrial protein potentially involved in respiratory metabolism" species:4932 "Saccharomyces cerevisiae" [GO:0003674 "molecular_function" evidence=ND] [GO:0043457 "regulation of cellular respiration" evidence=IGI] [GO:0005739 "mitochondrion" evidence=IEA;IMP] SGD:S000028422 GO:GO:0005739 EMBL:BK006945 EMBL:U53879 GO:GO:0043457 EMBL:Z73326 EMBL:AF479964 RefSeq:NP_690845.1 ProteinModelPortal:Q8TGM6 STRING:Q8TGM6 EnsemblFungi:YLR154W-C GeneID:1466405 KEGG:sce:YLR154W-C CYGD:YLR154w-c eggNOG:NOG79861 OMA:SCSKEHR OrthoDB:EOG466ZX8 NextBio:967146 Genevestigator:Q8TGM6 GermOnline:YLR154W-C Uniprot:Q8TGM6
Root	Isotig00090	2770	3117	-0.299	1.40E-15	SGD|S000028422 - symbol:TAR1 "Mitochondrial protein potentially involved in respiratory metabolism" species:4932 "Saccharomyces cerevisiae" [GO:0003674 "molecular_function" evidence=ND] [GO:0043457 "regulation of cellular respiration" evidence=IGI] [GO:0005739 "mitochondrion" evidence=IEA;IMP] SGD:S000028422 GO:GO:0005739 EMBL:BK006945 EMBL:U53879 GO:GO:0043457 EMBL:Z73326 EMBL:AF479964 RefSeq:NP_690845.1 ProteinModelPortal:Q8TGM6 STRING:Q8TGM6 EnsemblFungi:YLR154W-C GeneID:1466405 KEGG:sce:YLR154W-C CYGD:YLR154w-c eggNOG:NOG79861 OMA:SCSKEHR OrthoDB:EOG466ZX8 NextBio:967146 Genevestigator:Q8TGM6 GermOnline:YLR154W-C Uniprot:Q8TGM6
Root	Isotig00091	2689	3059	-0.315	1.01E-16	TAIR|locus:2825935 - symbol:AT1G28135 "AT1G28135" species:3702 "Arabidopsis thaliana" [GO:0003674 "molecular_function" evidence=ND] [GO:0008150 "biological_process" evidence=ND] EMBL:CP002684 GenomeReviews:CT485782_GR EMBL:BT010779 EMBL:BT014675 IPI:IPI00538576 RefSeq:NP_174135.1 UniGene:At.48243 IntAct:Q6IDD0 PRIDE:Q6IDD0 EnsemblPlants:AT1G28135.1 GeneID:839707 KEGG:ath:AT1G28135 TAIR:At1g28135 Genevestigator:Q6IDD0 Uniprot:Q6IDD0
Root	Isotig00092	2688	3060	-0.316	8.11E-17	TAIR|locus:2825935 - symbol:AT1G28135 "AT1G28135" species:3702 "Arabidopsis thaliana" [GO:0003674 "molecular_function" evidence=ND] [GO:0008150 "biological_process" evidence=ND] EMBL:CP002684 GenomeReviews:CT485782_GR EMBL:BT010779 EMBL:BT014675 IPI:IPI00538576 RefSeq:NP_174135.1 UniGene:At.48243 IntAct:Q6IDD0 PRIDE:Q6IDD0 EnsemblPlants:AT1G28135.1 GeneID:839707 KEGG:ath:AT1G28135 TAIR:At1g28135 Genevestigator:Q6IDD0 Uniprot:Q6IDD0
Root	Isotig00093	2338	1619	0.401	3.31E-18	SGD|S000028422 - symbol:TAR1 "Mitochondrial protein potentially involved in respiratory metabolism" species:4932 "Saccharomyces cerevisiae" [GO:0003674 "molecular_function" evidence=ND] [GO:0043457 "regulation of cellular respiration" evidence=IGI] [GO:0005739 "mitochondrion" evidence=IEA;IMP] SGD:S000028422 GO:GO:0005739 EMBL:BK006945 EMBL:U53879 GO:GO:0043457 EMBL:Z73326 EMBL:AF479964 RefSeq:NP_690845.1 ProteinModelPortal:Q8TGM6 STRING:Q8TGM6 EnsemblFungi:YLR154W-C GeneID:1466405 KEGG:sce:YLR154W-C CYGD:YLR154w-c eggNOG:NOG79861 OMA:SCSKEHR OrthoDB:EOG466ZX8 NextBio:967146 Genevestigator:Q8TGM6 GermOnline:YLR154W-C Uniprot:Q8TGM6
Root	Isotig00094	2338	1618	0.402	2.82E-18	SGD|S000028422 - symbol:TAR1 "Mitochondrial protein potentially involved in respiratory metabolism" species:4932 "Saccharomyces cerevisiae" [GO:0003674 "molecular_function" evidence=ND] [GO:0043457 "regulation of cellular respiration" evidence=IGI] [GO:0005739 "mitochondrion" evidence=IEA;IMP] SGD:S000028422 GO:GO:0005739 EMBL:BK006945 EMBL:U53879 GO:GO:0043457 EMBL:Z73326 EMBL:AF479964 RefSeq:NP_690845.1 ProteinModelPortal:Q8TGM6 STRING:Q8TGM6 EnsemblFungi:YLR154W-C GeneID:1466405 KEGG:sce:YLR154W-C CYGD:YLR154w-c eggNOG:NOG79861 OMA:SCSKEHR OrthoDB:EOG466ZX8 NextBio:967146 Genevestigator:Q8TGM6 GermOnline:YLR154W-C Uniprot:Q8TGM6
Root	Isotig00095	2338	1619	0.401	3.31E-18	SGD|S000028422 - symbol:TAR1 "Mitochondrial protein potentially involved in respiratory metabolism" species:4932 "Saccharomyces cerevisiae" [GO:0003674 "molecular_function" evidence=ND] [GO:0043457 "regulation of cellular respiration" evidence=IGI] [GO:0005739 "mitochondrion" evidence=IEA;IMP]

Tissue	Identification	# of reads (stress)	# of reads (control)	Fold Change	p-value	Description
						SGD:S000028422 GO:GO:0005739 EMBL:BK006945 EMBL:U53879 GO:GO:0043457 EMBL:Z73326 EMBL:AF479964 RefSeq:NP_690845.1 ProteinModelPortal:Q8TGM6 STRING:Q8TGM6 EnsemblFungi:YLR154W-C GeneID:1466405 KEGG:sce:YLR154W-C CYGD:YLR154w-c eggNOG:NOG79861 OMA:SCSKEHR OrthoDB:EOG466ZX8 NextBio:967146 Genevestigator:Q8TGM6 GermOnline:YLR154W-C Uniprot:Q8TGM6
Root	Isotig00096	2257	1561	0.403	9.29E-18	SGD|S000028567 - symbol:RRT15 "Putative protein of unknown function" species:4932 "Saccharomyces cerevisiae" [GO:0006351 "transcription, DNA-dependent" evidence=IEA] [GO:0005575 "cellular_component" evidence=ND] [GO:0008150 "biological_process" evidence=ND] [GO:0006355 "regulation of transcription, DNA-dependent" evidence=IEA] [GO:0003674 "molecular_function" evidence=ND] SGD:S000028567 GO:GO:0006355 GO:GO:0006351 EMBL:BK006945 EMBL:U51921 RefSeq:NP_878128.1 ProteinModelPortal:Q3E811 STRING:Q3E811 EnsemblFungi:YLR162W-A GeneID:1466416 KEGG:sce:YLR162W-A CYGD:YLR162w-a GeneTree:ENSGT00530000064745 OMA:FYINCIM OrthoDB:EOG45TGXP NextBio:983475 ArrayExpress:Q3E811 Genevestigator:Q3E811 Uniprot:Q3E811
Root	Isotig00097	2256	1562	0.401	1.24E-17	SGD|S000028567 - symbol:RRT15 "Putative protein of unknown function" species:4932 "Saccharomyces cerevisiae" [GO:0006351 "transcription, DNA-dependent" evidence=IEA] [GO:0005575 "cellular_component" evidence=ND] [GO:0008150 "biological_process" evidence=ND] [GO:0006355 "regulation of transcription, DNA-dependent" evidence=IEA] [GO:0003674 "molecular_function" evidence=ND] SGD:S000028567 GO:GO:0006355 GO:GO:0006351 EMBL:BK006945 EMBL:U51921 RefSeq:NP_878128.1 ProteinModelPortal:Q3E811 STRING:Q3E811 EnsemblFungi:YLR162W-A GeneID:1466416 KEGG:sce:YLR162W-A CYGD:YLR162w-a GeneTree:ENSGT00530000064745 OMA:FYINCIM OrthoDB:EOG45TGXP NextBio:983475 ArrayExpress:Q3E811 Genevestigator:Q3E811 Uniprot:Q3E811
Root	Isotig00098	1982	1295	0.485	1.42E-21	SGD|S000028567 - symbol:RRT15 "Putative protein of unknown function" species:4932 "Saccharomyces cerevisiae" [GO:0006351 "transcription, DNA-dependent" evidence=IEA] [GO:0005575 "cellular_component" evidence=ND] [GO:0008150 "biological_process" evidence=ND] [GO:0006355 "regulation of transcription, DNA-dependent" evidence=IEA] [GO:0003674 "molecular_function" evidence=ND] SGD:S000028567 GO:GO:0006355 GO:GO:0006351 EMBL:BK006945 EMBL:U51921 RefSeq:NP_878128.1 ProteinModelPortal:Q3E811 STRING:Q3E811 EnsemblFungi:YLR162W-A GeneID:1466416 KEGG:sce:YLR162W-A CYGD:YLR162w-a GeneTree:ENSGT00530000064745 OMA:FYINCIM OrthoDB:EOG45TGXP NextBio:983475 ArrayExpress:Q3E811 Genevestigator:Q3E811 Uniprot:Q3E811
Root	Isotig00106	659	438	0.460	1.64E-07	SGD|S000028567 - symbol:RRT15 "Putative protein of unknown function" species:4932 "Saccharomyces cerevisiae" [GO:0006351 "transcription, DNA-dependent" evidence=IEA] [GO:0005575 "cellular_component" evidence=ND] [GO:0008150 "biological_process" evidence=ND] [GO:0006355 "regulation of transcription, DNA-dependent" evidence=IEA] [GO:0003674 "molecular_function" evidence=ND] SGD:S000028567 GO:GO:0006355 GO:GO:0006351 EMBL:BK006945 EMBL:U51921 RefSeq:NP_878128.1 ProteinModelPortal:Q3E811 STRING:Q3E811 EnsemblFungi:YLR162W-A GeneID:1466416 KEGG:sce:YLR162W-A CYGD:YLR162w-a GeneTree:ENSGT00530000064745 OMA:FYINCIM OrthoDB:EOG45TGXP NextBio:983475 ArrayExpress:Q3E811 Genevestigator:Q3E811 Uniprot:Q3E811
Root	Isotig00109	414	157	1.270	2.61E-23	SGD|S000028567 - symbol:RRT15 "Putative protein of unknown function" species:4932 "Saccharomyces cerevisiae" [GO:0006351 "transcription, DNA-dependent" evidence=IEA] [GO:0005575 "cellular_component" evidence=ND] [GO:0008150 "biological_process" evidence=ND] [GO:0006355 "regulation of transcription, DNA-dependent" evidence=IEA] [GO:0003674 "molecular_function" evidence=ND] SGD:S000028567 GO:GO:0006355 GO:GO:0006351 EMBL:BK006945 EMBL:U51921 RefSeq:NP_878128.1 ProteinModelPortal:Q3E811 STRING:Q3E811 EnsemblFungi:YLR162W-A GeneID:1466416 KEGG:sce:YLR162W-A CYGD:YLR162w-a GeneTree:ENSGT00530000064745 OMA:FYINCIM OrthoDB:EOG45TGXP NextBio:983475 ArrayExpress:Q3E811 Genevestigator:Q3E811 Uniprot:Q3E811
Root	Isotig00110	415	157	1.273	1.87E-23	SGD|S000028567 - symbol:RRT15 "Putative protein of unknown function" species:4932 "Saccharomyces cerevisiae" [GO:0006351 "transcription, DNA-dependent" evidence=IEA] [GO:0005575 "cellular_component" evidence=ND] [GO:0008150 "biological_process" evidence=ND] [GO:0006355 "regulation of transcription, DNA-dependent" evidence=IEA] [GO:0003674 "molecular_function" evidence=ND] SGD:S000028567 GO:GO:0006355 GO:GO:0006351 EMBL:BK006945 EMBL:U51921 RefSeq:NP_878128.1 ProteinModelPortal:Q3E811 STRING:Q3E811 EnsemblFungi:YLR162W-A GeneID:1466416 KEGG:sce:YLR162W-A CYGD:YLR162w-a GeneTree:ENSGT00530000064745 OMA:FYINCIM OrthoDB:EOG45TGXP NextBio:983475 ArrayExpress:Q3E811 Genevestigator:Q3E811 Uniprot:Q3E811

Tissue	Identification	# of reads (stress)	# of reads (control)	Fold Change	p-value	Description
Root	Isotig00111	392	148	1.276	2.57E-22	SGD|S000028567 - symbol:RRT15 "Putative protein of unknown function" species:4932 "Saccharomyces cerevisiae" [GO:0006351 "transcription, DNA-dependent" evidence=IEA] [GO:0005575 "cellular_component" evidence=ND] [GO:0008150 "biological_process" evidence=ND] [GO:0006355 "regulation of transcription, DNA-dependent" evidence=IEA] [GO:0003674 "molecular_function" evidence=ND] SGD:S000028567 GO:GO:0006355 GO:GO:0006351 EMBL:BK006945 EMBL:U51921 RefSeq:NP_878128.1 ProteinModelPortal:Q3E811 STRING:Q3E811 EnsemblFungi:YLR162W-A GeneID:1466416 KEGG:sce:YLR162W-A CYGD:YLR162w-a GeneTree:ENSGT00530000064745 OMA:FYINCIM OrthoDB:EOG45TGXP NextBio:983475 ArrayExpress:Q3E811 Genevestigator:Q3E811 Uniprot:Q3E811
Root	Isotig00112	393	148	1.280	1.84E-22	SGD|S000028567 - symbol:RRT15 "Putative protein of unknown function" species:4932 "Saccharomyces cerevisiae" [GO:0006351 "transcription, DNA-dependent" evidence=IEA] [GO:0005575 "cellular_component" evidence=ND] [GO:0008150 "biological_process" evidence=ND] [GO:0006355 "regulation of transcription, DNA-dependent" evidence=IEA] [GO:0003674 "molecular_function" evidence=ND] SGD:S000028567 GO:GO:0006355 GO:GO:0006351 EMBL:BK006945 EMBL:U51921 RefSeq:NP_878128.1 ProteinModelPortal:Q3E811 STRING:Q3E811 EnsemblFungi:YLR162W-A GeneID:1466416 KEGG:sce:YLR162W-A CYGD:YLR162w-a GeneTree:ENSGT00530000064745 OMA:FYINCIM OrthoDB:EOG45TGXP NextBio:983475 ArrayExpress:Q3E811 Genevestigator:Q3E811 Uniprot:Q3E811
Root	Isotig00113	401	155	1.242	6.42E-22	SGD|S000028567 - symbol:RRT15 "Putative protein of unknown function" species:4932 "Saccharomyces cerevisiae" [GO:0006351 "transcription, DNA-dependent" evidence=IEA] [GO:0005575 "cellular_component" evidence=ND] [GO:0008150 "biological_process" evidence=ND] [GO:0006355 "regulation of transcription, DNA-dependent" evidence=IEA] [GO:0003674 "molecular_function" evidence=ND] SGD:S000028567 GO:GO:0006355 GO:GO:0006351 EMBL:BK006945 EMBL:U51921 RefSeq:NP_878128.1 ProteinModelPortal:Q3E811 STRING:Q3E811 EnsemblFungi:YLR162W-A GeneID:1466416 KEGG:sce:YLR162W-A CYGD:YLR162w-a GeneTree:ENSGT00530000064745 OMA:FYINCIM OrthoDB:EOG45TGXP NextBio:983475 ArrayExpress:Q3E811 Genevestigator:Q3E811 Uniprot:Q3E811
Root	Isotig00114	404	156	1.244	4.15E-22	SGD|S000028567 - symbol:RRT15 "Putative protein of unknown function" species:4932 "Saccharomyces cerevisiae" [GO:0006351 "transcription, DNA-dependent" evidence=IEA] [GO:0005575 "cellular_component" evidence=ND] [GO:0008150 "biological_process" evidence=ND] [GO:0006355 "regulation of transcription, DNA-dependent" evidence=IEA] [GO:0003674 "molecular_function" evidence=ND] SGD:S000028567 GO:GO:0006355 GO:GO:0006351 EMBL:BK006945 EMBL:U51921 RefSeq:NP_878128.1 ProteinModelPortal:Q3E811 STRING:Q3E811 EnsemblFungi:YLR162W-A GeneID:1466416 KEGG:sce:YLR162W-A CYGD:YLR162w-a GeneTree:ENSGT00530000064745 OMA:FYINCIM OrthoDB:EOG45TGXP NextBio:983475 ArrayExpress:Q3E811 Genevestigator:Q3E811 Uniprot:Q3E811
Root	Isotig00115	377	149	1.210	6.26E-20	SGD|S000028567 - symbol:RRT15 "Putative protein of unknown function" species:4932 "Saccharomyces cerevisiae" [GO:0006351 "transcription, DNA-dependent" evidence=IEA] [GO:0005575 "cellular_component" evidence=ND] [GO:0008150 "biological_process" evidence=ND] [GO:0006355 "regulation of transcription, DNA-dependent" evidence=IEA] [GO:0003674 "molecular_function" evidence=ND] SGD:S000028567 GO:GO:0006355 GO:GO:0006351 EMBL:BK006945 EMBL:U51921 RefSeq:NP_878128.1 ProteinModelPortal:Q3E811 STRING:Q3E811 EnsemblFungi:YLR162W-A GeneID:1466416 KEGG:sce:YLR162W-A CYGD:YLR162w-a GeneTree:ENSGT00530000064745 OMA:FYINCIM OrthoDB:EOG45TGXP NextBio:983475 ArrayExpress:Q3E811 Genevestigator:Q3E811 Uniprot:Q3E811
Root	Isotig00116	414	157	1.270	2.61E-23	SGD|S000028567 - symbol:RRT15 "Putative protein of unknown function" species:4932 "Saccharomyces cerevisiae" [GO:0006351 "transcription, DNA-dependent" evidence=IEA] [GO:0005575 "cellular_component" evidence=ND] [GO:0008150 "biological_process" evidence=ND] [GO:0006355 "regulation of transcription, DNA-dependent" evidence=IEA] [GO:0003674 "molecular_function" evidence=ND] SGD:S000028567 GO:GO:0006355 GO:GO:0006351 EMBL:BK006945 EMBL:U51921 RefSeq:NP_878128.1 ProteinModelPortal:Q3E811 STRING:Q3E811 EnsemblFungi:YLR162W-A GeneID:1466416 KEGG:sce:YLR162W-A CYGD:YLR162w-a GeneTree:ENSGT00530000064745 OMA:FYINCIM OrthoDB:EOG45TGXP NextBio:983475 ArrayExpress:Q3E811 Genevestigator:Q3E811 Uniprot:Q3E811
Root	Isotig00117	414	157	1.270	2.61E-23	SGD|S000028567 - symbol:RRT15 "Putative protein of unknown function" species:4932 "Saccharomyces cerevisiae" [GO:0006351 "transcription, DNA-dependent" evidence=IEA] [GO:0005575 "cellular_component" evidence=ND] [GO:0008150 "biological_process" evidence=ND] [GO:0006355 "regulation of transcription, DNA-dependent" evidence=IEA] [GO:0003674 "molecular_function" evidence=ND] SGD:S000028567 GO:GO:0006355

Tissue	Identification	# of reads (stress)	# of reads (control)	Fold Change	p-value	Description
						GO:GO:0006351 EMBL:BK006945 EMBL:U51921 RefSeq:NP_878128.1 ProteinModelPortal:Q3E811 STRING:Q3E811 EnsemblFungi:YLR162W-A GeneID:1466416 KEGG:sce:YLR162W-A CYGD:YLR162w-a GeneTree:ENSGT00530000064745 OMA:FYINCIM OrthoDB:EOG45TGXP NextBio:983475 ArrayExpress:Q3E811 Genevestigator:Q3E811 Uniprot:Q3E811
Root	Isotig00118	379	146	1.247	6.38E-21	SGD|S000028567 - symbol:RRT15 "Putative protein of unknown function" species:4932 "Saccharomyces cerevisiae" [GO:0006351 "transcription, DNA-dependent" evidence=IEA] [GO:0005575 "cellular_component" evidence=ND] [GO:0008150 "biological_process" evidence=ND] [GO:0006355 "regulation of transcription, DNA-dependent" evidence=IEA] [GO:0003674 "molecular_function" evidence=ND] SGD:S000028567 GO:GO:0006355 GO:GO:0006351 EMBL:BK006945 EMBL:U51921 RefSeq:NP_878128.1 ProteinModelPortal:Q3E811 STRING:Q3E811 EnsemblFungi:YLR162W-A GeneID:1466416 KEGG:sce:YLR162W-A CYGD:YLR162w-a GeneTree:ENSGT00530000064745 OMA:FYINCIM OrthoDB:EOG45TGXP NextBio:983475 ArrayExpress:Q3E811 Genevestigator:Q3E811 Uniprot:Q3E811
Root	Isotig00119	382	147	1.249	4.11E-21	SGD|S000028567 - symbol:RRT15 "Putative protein of unknown function" species:4932 "Saccharomyces cerevisiae" [GO:0006351 "transcription, DNA-dependent" evidence=IEA] [GO:0005575 "cellular_component" evidence=ND] [GO:0008150 "biological_process" evidence=ND] [GO:0006355 "regulation of transcription, DNA-dependent" evidence=IEA] [GO:0003674 "molecular_function" evidence=ND] SGD:S000028567 GO:GO:0006355 GO:GO:0006351 EMBL:BK006945 EMBL:U51921 RefSeq:NP_878128.1 ProteinModelPortal:Q3E811 STRING:Q3E811 EnsemblFungi:YLR162W-A GeneID:1466416 KEGG:sce:YLR162W-A CYGD:YLR162w-a GeneTree:ENSGT00530000064745 OMA:FYINCIM OrthoDB:EOG45TGXP NextBio:983475 ArrayExpress:Q3E811 Genevestigator:Q3E811 Uniprot:Q3E811
Root	Isotig00120	378	149	1.214	4.53E-20	SGD|S000028567 - symbol:RRT15 "Putative protein of unknown function" species:4932 "Saccharomyces cerevisiae" [GO:0006351 "transcription, DNA-dependent" evidence=IEA] [GO:0005575 "cellular_component" evidence=ND] [GO:0008150 "biological_process" evidence=ND] [GO:0006355 "regulation of transcription, DNA-dependent" evidence=IEA] [GO:0003674 "molecular_function" evidence=ND] SGD:S000028567 GO:GO:0006355 GO:GO:0006351 EMBL:BK006945 EMBL:U51921 RefSeq:NP_878128.1 ProteinModelPortal:Q3E811 STRING:Q3E811 EnsemblFungi:YLR162W-A GeneID:1466416 KEGG:sce:YLR162W-A CYGD:YLR162w-a GeneTree:ENSGT00530000064745 OMA:FYINCIM OrthoDB:EOG45TGXP NextBio:983475 ArrayExpress:Q3E811 Genevestigator:Q3E811 Uniprot:Q3E811
Root	Isotig00121	415	157	1.273	1.87E-23	SGD|S000028567 - symbol:RRT15 "Putative protein of unknown function" species:4932 "Saccharomyces cerevisiae" [GO:0006351 "transcription, DNA-dependent" evidence=IEA] [GO:0005575 "cellular_component" evidence=ND] [GO:0008150 "biological_process" evidence=ND] [GO:0006355 "regulation of transcription, DNA-dependent" evidence=IEA] [GO:0003674 "molecular_function" evidence=ND] SGD:S000028567 GO:GO:0006355 GO:GO:0006351 EMBL:BK006945 EMBL:U51921 RefSeq:NP_878128.1 ProteinModelPortal:Q3E811 STRING:Q3E811 EnsemblFungi:YLR162W-A GeneID:1466416 KEGG:sce:YLR162W-A CYGD:YLR162w-a GeneTree:ENSGT00530000064745 OMA:FYINCIM OrthoDB:EOG45TGXP NextBio:983475 ArrayExpress:Q3E811 Genevestigator:Q3E811 Uniprot:Q3E811
Root	Isotig00122	355	140	1.213	6.28E-19	SGD|S000028567 - symbol:RRT15 "Putative protein of unknown function" species:4932 "Saccharomyces cerevisiae" [GO:0006351 "transcription, DNA-dependent" evidence=IEA] [GO:0005575 "cellular_component" evidence=ND] [GO:0008150 "biological_process" evidence=ND] [GO:0006355 "regulation of transcription, DNA-dependent" evidence=IEA] [GO:0003674 "molecular_function" evidence=ND] SGD:S000028567 GO:GO:0006355 GO:GO:0006351 EMBL:BK006945 EMBL:U51921 RefSeq:NP_878128.1 ProteinModelPortal:Q3E811 STRING:Q3E811 EnsemblFungi:YLR162W-A GeneID:1466416 KEGG:sce:YLR162W-A CYGD:YLR162w-a GeneTree:ENSGT00530000064745 OMA:FYINCIM OrthoDB:EOG45TGXP NextBio:983475 ArrayExpress:Q3E811 Genevestigator:Q3E811 Uniprot:Q3E811
Root	Isotig00123	415	157	1.273	1.87E-23	SGD|S000028567 - symbol:RRT15 "Putative protein of unknown function" species:4932 "Saccharomyces cerevisiae" [GO:0006351 "transcription, DNA-dependent" evidence=IEA] [GO:0005575 "cellular_component" evidence=ND] [GO:0008150 "biological_process" evidence=ND] [GO:0006355 "regulation of transcription, DNA-dependent" evidence=IEA] [GO:0003674 "molecular_function" evidence=ND] SGD:S000028567 GO:GO:0006355 GO:GO:0006351 EMBL:BK006945 EMBL:U51921 RefSeq:NP_878128.1 ProteinModelPortal:Q3E811 STRING:Q3E811 EnsemblFungi:YLR162W-A GeneID:1466416 KEGG:sce:YLR162W-A CYGD:YLR162w-a GeneTree:ENSGT00530000064745 OMA:FYINCIM OrthoDB:EOG45TGXP NextBio:983475 ArrayExpress:Q3E811 Genevestigator:Q3E811 Uniprot:Q3E811

Tissue	Identification	# of reads (stress)	# of reads (control)	Fold Change	p-value	Description
Root	Isotig00124	392	148	1.276	2.57E-22	SGD|S000028567 - symbol:RRT15 "Putative protein of unknown function" species:4932 "Saccharomyces cerevisiae" [GO:0006351 "transcription, DNA-dependent" evidence=IEA] [GO:0005575 "cellular_component" evidence=ND] [GO:0008150 "biological_process" evidence=ND] [GO:0006355 "regulation of transcription, DNA-dependent" evidence=IEA] [GO:0003674 "molecular_function" evidence=ND] SGD:S000028567 GO:GO:0006355 GO:GO:0006351 EMBL:BK006945 EMBL:U51921 RefSeq:NP_878128.1 ProteinModelPortal:Q3E811 STRING:Q3E811 EnsemblFungi:YLR162W-A GeneID:1466416 KEGG:sce:YLR162W-A CYGD:YLR162w-a GeneTree:ENSGT00530000064745 OMA:FYINCIM OrthoDB:EOG45TGXP NextBio:983475 ArrayExpress:Q3E811 Genevestigator:Q3E811 Uniprot:Q3E811
Root	Isotig00125	392	148	1.276	2.57E-22	SGD|S000028567 - symbol:RRT15 "Putative protein of unknown function" species:4932 "Saccharomyces cerevisiae" [GO:0006351 "transcription, DNA-dependent" evidence=IEA] [GO:0005575 "cellular_component" evidence=ND] [GO:0008150 "biological_process" evidence=ND] [GO:0006355 "regulation of transcription, DNA-dependent" evidence=IEA] [GO:0003674 "molecular_function" evidence=ND] SGD:S000028567 GO:GO:0006355 GO:GO:0006351 EMBL:BK006945 EMBL:U51921 RefSeq:NP_878128.1 ProteinModelPortal:Q3E811 STRING:Q3E811 EnsemblFungi:YLR162W-A GeneID:1466416 KEGG:sce:YLR162W-A CYGD:YLR162w-a GeneTree:ENSGT00530000064745 OMA:FYINCIM OrthoDB:EOG45TGXP NextBio:983475 ArrayExpress:Q3E811 Genevestigator:Q3E811 Uniprot:Q3E811
Root	Isotig00126	356	140	1.217	4.54E-19	SGD|S000028567 - symbol:RRT15 "Putative protein of unknown function" species:4932 "Saccharomyces cerevisiae" [GO:0006351 "transcription, DNA-dependent" evidence=IEA] [GO:0005575 "cellular_component" evidence=ND] [GO:0008150 "biological_process" evidence=ND] [GO:0006355 "regulation of transcription, DNA-dependent" evidence=IEA] [GO:0003674 "molecular_function" evidence=ND] SGD:S000028567 GO:GO:0006355 GO:GO:0006351 EMBL:BK006945 EMBL:U51921 RefSeq:NP_878128.1 ProteinModelPortal:Q3E811 STRING:Q3E811 EnsemblFungi:YLR162W-A GeneID:1466416 KEGG:sce:YLR162W-A CYGD:YLR162w-a GeneTree:ENSGT00530000064745 OMA:FYINCIM OrthoDB:EOG45TGXP NextBio:983475 ArrayExpress:Q3E811 Genevestigator:Q3E811 Uniprot:Q3E811
Root	Isotig00127	364	147	1.179	1.41E-18	SGD|S000028567 - symbol:RRT15 "Putative protein of unknown function" species:4932 "Saccharomyces cerevisiae" [GO:0006351 "transcription, DNA-dependent" evidence=IEA] [GO:0005575 "cellular_component" evidence=ND] [GO:0008150 "biological_process" evidence=ND] [GO:0006355 "regulation of transcription, DNA-dependent" evidence=IEA] [GO:0003674 "molecular_function" evidence=ND] SGD:S000028567 GO:GO:0006355 GO:GO:0006351 EMBL:BK006945 EMBL:U51921 RefSeq:NP_878128.1 ProteinModelPortal:Q3E811 STRING:Q3E811 EnsemblFungi:YLR162W-A GeneID:1466416 KEGG:sce:YLR162W-A CYGD:YLR162w-a GeneTree:ENSGT00530000064745 OMA:FYINCIM OrthoDB:EOG45TGXP NextBio:983475 ArrayExpress:Q3E811 Genevestigator:Q3E811 Uniprot:Q3E811
Root	Isotig00128	367	148	1.181	9.11E-19	SGD|S000028567 - symbol:RRT15 "Putative protein of unknown function" species:4932 "Saccharomyces cerevisiae" [GO:0006351 "transcription, DNA-dependent" evidence=IEA] [GO:0005575 "cellular_component" evidence=ND] [GO:0008150 "biological_process" evidence=ND] [GO:0006355 "regulation of transcription, DNA-dependent" evidence=IEA] [GO:0003674 "molecular_function" evidence=ND] SGD:S000028567 GO:GO:0006355 GO:GO:0006351 EMBL:BK006945 EMBL:U51921 RefSeq:NP_878128.1 ProteinModelPortal:Q3E811 STRING:Q3E811 EnsemblFungi:YLR162W-A GeneID:1466416 KEGG:sce:YLR162W-A CYGD:YLR162w-a GeneTree:ENSGT00530000064745 OMA:FYINCIM OrthoDB:EOG45TGXP NextBio:983475 ArrayExpress:Q3E811 Genevestigator:Q3E811 Uniprot:Q3E811
Root	Isotig00129	393	148	1.280	1.84E-22	SGD|S000028567 - symbol:RRT15 "Putative protein of unknown function" species:4932 "Saccharomyces cerevisiae" [GO:0006351 "transcription, DNA-dependent" evidence=IEA] [GO:0005575 "cellular_component" evidence=ND] [GO:0008150 "biological_process" evidence=ND] [GO:0006355 "regulation of transcription, DNA-dependent" evidence=IEA] [GO:0003674 "molecular_function" evidence=ND] SGD:S000028567 GO:GO:0006355 GO:GO:0006351 EMBL:BK006945 EMBL:U51921 RefSeq:NP_878128.1 ProteinModelPortal:Q3E811 STRING:Q3E811 EnsemblFungi:YLR162W-A GeneID:1466416 KEGG:sce:YLR162W-A CYGD:YLR162w-a GeneTree:ENSGT00530000064745 OMA:FYINCIM OrthoDB:EOG45TGXP NextBio:983475 ArrayExpress:Q3E811 Genevestigator:Q3E811 Uniprot:Q3E811
Root	Isotig00130	401	155	1.242	6.42E-22	SGD|S000028567 - symbol:RRT15 "Putative protein of unknown function" species:4932 "Saccharomyces cerevisiae" [GO:0006351 "transcription, DNA-dependent" evidence=IEA] [GO:0005575 "cellular_component" evidence=ND] [GO:0008150 "biological_process" evidence=ND] [GO:0006355 "regulation of transcription, DNA-dependent" evidence=IEA] [GO:0003674 "molecular_function" evidence=ND] SGD:S000028567 GO:GO:0006355

Tissue	Identification	# of reads (stress)	# of reads (control)	Fold Change	p-value	Description
						GO:GO:0006351 EMBL:BK006945 EMBL:U51921 RefSeq:NP_878128.1 ProteinModelPortal:Q3E811 STRING:Q3E811 EnsemblFungi:YLR162W-A GeneID:1466416 KEGG:sce:YLR162W-A CYGD:YLR162w-a GeneTree:ENSGT00530000064745 OMA:FYINCIM OrthoDB:EOG45TGXP NextBio:983475 ArrayExpress:Q3E811 Genevestigator:Q3E811 Uniprot:Q3E811
Root	Isotig00131	404	156	1.244	4.15E-22	SGD|S000028567 - symbol:RRT15 "Putative protein of unknown function" species:4932 "Saccharomyces cerevisiae" [GO:0006351 "transcription, DNA-dependent" evidence=IEA] [GO:0005575 "cellular_component" evidence=ND] [GO:0008150 "biological_process" evidence=ND] [GO:0006355 "regulation of transcription, DNA-dependent" evidence=IEA] [GO:0003674 "molecular_function" evidence=ND] SGD:S000028567 GO:GO:0006355 GO:GO:0006351 EMBL:BK006945 EMBL:U51921 RefSeq:NP_878128.1 ProteinModelPortal:Q3E811 STRING:Q3E811 EnsemblFungi:YLR162W-A GeneID:1466416 KEGG:sce:YLR162W-A CYGD:YLR162w-a GeneTree:ENSGT00530000064745 OMA:FYINCIM OrthoDB:EOG45TGXP NextBio:983475 ArrayExpress:Q3E811 Genevestigator:Q3E811 Uniprot:Q3E811
Root	Isotig00132	393	148	1.280	1.84E-22	SGD|S000028567 - symbol:RRT15 "Putative protein of unknown function" species:4932 "Saccharomyces cerevisiae" [GO:0006351 "transcription, DNA-dependent" evidence=IEA] [GO:0005575 "cellular_component" evidence=ND] [GO:0008150 "biological_process" evidence=ND] [GO:0006355 "regulation of transcription, DNA-dependent" evidence=IEA] [GO:0003674 "molecular_function" evidence=ND] SGD:S000028567 GO:GO:0006355 GO:GO:0006351 EMBL:BK006945 EMBL:U51921 RefSeq:NP_878128.1 ProteinModelPortal:Q3E811 STRING:Q3E811 EnsemblFungi:YLR162W-A GeneID:1466416 KEGG:sce:YLR162W-A CYGD:YLR162w-a GeneTree:ENSGT00530000064745 OMA:FYINCIM OrthoDB:EOG45TGXP NextBio:983475 ArrayExpress:Q3E811 Genevestigator:Q3E811 Uniprot:Q3E811
Root	Isotig00133	401	155	1.242	6.42E-22	SGD|S000028567 - symbol:RRT15 "Putative protein of unknown function" species:4932 "Saccharomyces cerevisiae" [GO:0006351 "transcription, DNA-dependent" evidence=IEA] [GO:0005575 "cellular_component" evidence=ND] [GO:0008150 "biological_process" evidence=ND] [GO:0006355 "regulation of transcription, DNA-dependent" evidence=IEA] [GO:0003674 "molecular_function" evidence=ND] SGD:S000028567 GO:GO:0006355 GO:GO:0006351 EMBL:BK006945 EMBL:U51921 RefSeq:NP_878128.1 ProteinModelPortal:Q3E811 STRING:Q3E811 EnsemblFungi:YLR162W-A GeneID:1466416 KEGG:sce:YLR162W-A CYGD:YLR162w-a GeneTree:ENSGT00530000064745 OMA:FYINCIM OrthoDB:EOG45TGXP NextBio:983475 ArrayExpress:Q3E811 Genevestigator:Q3E811 Uniprot:Q3E811
Root	Isotig00134	404	156	1.244	4.15E-22	SGD|S000028567 - symbol:RRT15 "Putative protein of unknown function" species:4932 "Saccharomyces cerevisiae" [GO:0006351 "transcription, DNA-dependent" evidence=IEA] [GO:0005575 "cellular_component" evidence=ND] [GO:0008150 "biological_process" evidence=ND] [GO:0006355 "regulation of transcription, DNA-dependent" evidence=IEA] [GO:0003674 "molecular_function" evidence=ND] SGD:S000028567 GO:GO:0006355 GO:GO:0006351 EMBL:BK006945 EMBL:U51921 RefSeq:NP_878128.1 ProteinModelPortal:Q3E811 STRING:Q3E811 EnsemblFungi:YLR162W-A GeneID:1466416 KEGG:sce:YLR162W-A CYGD:YLR162w-a GeneTree:ENSGT00530000064745 OMA:FYINCIM OrthoDB:EOG45TGXP NextBio:983475 ArrayExpress:Q3E811 Genevestigator:Q3E811 Uniprot:Q3E811
Root	Isotig00135	342	138	1.180	1.42E-17	SGD|S000028567 - symbol:RRT15 "Putative protein of unknown function" species:4932 "Saccharomyces cerevisiae" [GO:0006351 "transcription, DNA-dependent" evidence=IEA] [GO:0005575 "cellular_component" evidence=ND] [GO:0008150 "biological_process" evidence=ND] [GO:0006355 "regulation of transcription, DNA-dependent" evidence=IEA] [GO:0003674 "molecular_function" evidence=ND] SGD:S000028567 GO:GO:0006355 GO:GO:0006351 EMBL:BK006945 EMBL:U51921 RefSeq:NP_878128.1 ProteinModelPortal:Q3E811 STRING:Q3E811 EnsemblFungi:YLR162W-A GeneID:1466416 KEGG:sce:YLR162W-A CYGD:YLR162w-a GeneTree:ENSGT00530000064745 OMA:FYINCIM OrthoDB:EOG45TGXP NextBio:983475 ArrayExpress:Q3E811 Genevestigator:Q3E811 Uniprot:Q3E811
Root	Isotig00136	345	139	1.183	9.18E-18	SGD|S000028567 - symbol:RRT15 "Putative protein of unknown function" species:4932 "Saccharomyces cerevisiae" [GO:0006351 "transcription, DNA-dependent" evidence=IEA] [GO:0005575 "cellular_component" evidence=ND] [GO:0008150 "biological_process" evidence=ND] [GO:0006355 "regulation of transcription, DNA-dependent" evidence=IEA] [GO:0003674 "molecular_function" evidence=ND] SGD:S000028567 GO:GO:0006355 GO:GO:0006351 EMBL:BK006945 EMBL:U51921 RefSeq:NP_878128.1 ProteinModelPortal:Q3E811 STRING:Q3E811 EnsemblFungi:YLR162W-A GeneID:1466416 KEGG:sce:YLR162W-A CYGD:YLR162w-a GeneTree:ENSGT00530000064745 OMA:FYINCIM OrthoDB:EOG45TGXP NextBio:983475 ArrayExpress:Q3E811 Genevestigator:Q3E811 Uniprot:Q3E811

Tissue	Identification	# of reads (stress)	# of reads (control)	Fold Change	p-value	Description
Root	Isotig00137	379	146	1.247	6.38E-21	SGD|S000028567 - symbol:RRT15 "Putative protein of unknown function" species:4932 "Saccharomyces cerevisiae" [GO:0006351 "transcription, DNA-dependent" evidence=IEA] [GO:0005575 "cellular_component" evidence=ND] [GO:0008150 "biological_process" evidence=ND] [GO:0006355 "regulation of transcription, DNA-dependent" evidence=IEA] [GO:0003674 "molecular_function" evidence=ND] SGD:S000028567 GO:GO:0006355 GO:GO:0006351 EMBL:BK006945 EMBL:U51921 RefSeq:NP_878128.1 ProteinModelPortal:Q3E811 STRING:Q3E811 EnsemblFungi:YLR162W-A GeneID:1466416 KEGG:sce:YLR162W-A CYGD:YLR162w-a GeneTree:ENSGT00530000064745 OMA:FYINCIM OrthoDB:EOG45TGXP NextBio:983475 ArrayExpress:Q3E811 Genevestigator:Q3E811 Uniprot:Q3E811
Root	Isotig00138	382	147	1.249	4.11E-21	SGD|S000028567 - symbol:RRT15 "Putative protein of unknown function" species:4932 "Saccharomyces cerevisiae" [GO:0006351 "transcription, DNA-dependent" evidence=IEA] [GO:0005575 "cellular_component" evidence=ND] [GO:0008150 "biological_process" evidence=ND] [GO:0006355 "regulation of transcription, DNA-dependent" evidence=IEA] [GO:0003674 "molecular_function" evidence=ND] SGD:S000028567 GO:GO:0006355 GO:GO:0006351 EMBL:BK006945 EMBL:U51921 RefSeq:NP_878128.1 ProteinModelPortal:Q3E811 STRING:Q3E811 EnsemblFungi:YLR162W-A GeneID:1466416 KEGG:sce:YLR162W-A CYGD:YLR162w-a GeneTree:ENSGT00530000064745 OMA:FYINCIM OrthoDB:EOG45TGXP NextBio:983475 ArrayExpress:Q3E811 Genevestigator:Q3E811 Uniprot:Q3E811
Root	Isotig00139	379	146	1.247	6.38E-21	SGD|S000028567 - symbol:RRT15 "Putative protein of unknown function" species:4932 "Saccharomyces cerevisiae" [GO:0006351 "transcription, DNA-dependent" evidence=IEA] [GO:0005575 "cellular_component" evidence=ND] [GO:0008150 "biological_process" evidence=ND] [GO:0006355 "regulation of transcription, DNA-dependent" evidence=IEA] [GO:0003674 "molecular_function" evidence=ND] SGD:S000028567 GO:GO:0006355 GO:GO:0006351 EMBL:BK006945 EMBL:U51921 RefSeq:NP_878128.1 ProteinModelPortal:Q3E811 STRING:Q3E811 EnsemblFungi:YLR162W-A GeneID:1466416 KEGG:sce:YLR162W-A CYGD:YLR162w-a GeneTree:ENSGT00530000064745 OMA:FYINCIM OrthoDB:EOG45TGXP NextBio:983475 ArrayExpress:Q3E811 Genevestigator:Q3E811 Uniprot:Q3E811
Root	Isotig00140	382	147	1.249	4.11E-21	SGD|S000028567 - symbol:RRT15 "Putative protein of unknown function" species:4932 "Saccharomyces cerevisiae" [GO:0006351 "transcription, DNA-dependent" evidence=IEA] [GO:0005575 "cellular_component" evidence=ND] [GO:0008150 "biological_process" evidence=ND] [GO:0006355 "regulation of transcription, DNA-dependent" evidence=IEA] [GO:0003674 "molecular_function" evidence=ND] SGD:S000028567 GO:GO:0006355 GO:GO:0006351 EMBL:BK006945 EMBL:U51921 RefSeq:NP_878128.1 ProteinModelPortal:Q3E811 STRING:Q3E811 EnsemblFungi:YLR162W-A GeneID:1466416 KEGG:sce:YLR162W-A CYGD:YLR162w-a GeneTree:ENSGT00530000064745 OMA:FYINCIM OrthoDB:EOG45TGXP NextBio:983475 ArrayExpress:Q3E811 Genevestigator:Q3E811 Uniprot:Q3E811
Root	Isotig00141	317	126	1.202	7.71E-17	SGD|S000028567 - symbol:RRT15 "Putative protein of unknown function" species:4932 "Saccharomyces cerevisiae" [GO:0006351 "transcription, DNA-dependent" evidence=IEA] [GO:0005575 "cellular_component" evidence=ND] [GO:0008150 "biological_process" evidence=ND] [GO:0006355 "regulation of transcription, DNA-dependent" evidence=IEA] [GO:0003674 "molecular_function" evidence=ND] SGD:S000028567 GO:GO:0006355 GO:GO:0006351 EMBL:BK006945 EMBL:U51921 RefSeq:NP_878128.1 ProteinModelPortal:Q3E811 STRING:Q3E811 EnsemblFungi:YLR162W-A GeneID:1466416 KEGG:sce:YLR162W-A CYGD:YLR162w-a GeneTree:ENSGT00530000064745 OMA:FYINCIM OrthoDB:EOG45TGXP NextBio:983475 ArrayExpress:Q3E811 Genevestigator:Q3E811 Uniprot:Q3E811
Root	Isotig00142	318	126	1.207	5.58E-17	SGD|S000028567 - symbol:RRT15 "Putative protein of unknown function" species:4932 "Saccharomyces cerevisiae" [GO:0006351 "transcription, DNA-dependent" evidence=IEA] [GO:0005575 "cellular_component" evidence=ND] [GO:0008150 "biological_process" evidence=ND] [GO:0006355 "regulation of transcription, DNA-dependent" evidence=IEA] [GO:0003674 "molecular_function" evidence=ND] SGD:S000028567 GO:GO:0006355 GO:GO:0006351 EMBL:BK006945 EMBL:U51921 RefSeq:NP_878128.1 ProteinModelPortal:Q3E811 STRING:Q3E811 EnsemblFungi:YLR162W-A GeneID:1466416 KEGG:sce:YLR162W-A CYGD:YLR162w-a GeneTree:ENSGT00530000064745 OMA:FYINCIM OrthoDB:EOG45TGXP NextBio:983475 ArrayExpress:Q3E811 Genevestigator:Q3E811 Uniprot:Q3E811
Root	Isotig00143	295	117	1.205	7.78E-16	SGD|S000028567 - symbol:RRT15 "Putative protein of unknown function" species:4932 "Saccharomyces cerevisiae" [GO:0006351 "transcription, DNA-dependent" evidence=IEA] [GO:0005575 "cellular_component" evidence=ND] [GO:0008150 "biological_process" evidence=ND] [GO:0006355 "regulation of transcription, DNA-dependent" evidence=IEA] [GO:0003674 "molecular_function" evidence=ND] SGD:S000028567 GO:GO:0006355

Tissue	Identification	# of reads (stress)	# of reads (control)	Fold Change	p-value	Description
						GO:GO:0006351 EMBL:BK006945 EMBL:U51921 RefSeq:NP_878128.1 ProteinModelPortal:Q3E811 STRING:Q3E811 EnsemblFungi:YLR162W-A GeneID:1466416 KEGG:sce:YLR162W-A CYGD:YLR162w-a GeneTree:ENSGT00530000064745 OMA:FYINCIM OrthoDB:EOG45TGXP NextBio:983475 ArrayExpress:Q3E811 Genevestigator:Q3E811 Uniprot:Q3E811
Root	Isotig00144	296	117	1.210	5.63E-16	SGD|S000028567 - symbol:RRT15 "Putative protein of unknown function" species:4932 "Saccharomyces cerevisiae" [GO:0006351 "transcription, DNA-dependent" evidence=IEA] [GO:0005575 "cellular_component" evidence=ND] [GO:0008150 "biological_process" evidence=ND] [GO:0006355 "regulation of transcription, DNA-dependent" evidence=IEA] [GO:0003674 "molecular_function" evidence=ND] SGD:S000028567 GO:GO:0006355 GO:GO:0006351 EMBL:BK006945 EMBL:U51921 RefSeq:NP_878128.1 ProteinModelPortal:Q3E811 STRING:Q3E811 EnsemblFungi:YLR162W-A GeneID:1466416 KEGG:sce:YLR162W-A CYGD:YLR162w-a GeneTree:ENSGT00530000064745 OMA:FYINCIM OrthoDB:EOG45TGXP NextBio:983475 ArrayExpress:Q3E811 Genevestigator:Q3E811 Uniprot:Q3E811
Root	Isotig00145	304	124	1.165	1.71E-15	SGD|S000028567 - symbol:RRT15 "Putative protein of unknown function" species:4932 "Saccharomyces cerevisiae" [GO:0006351 "transcription, DNA-dependent" evidence=IEA] [GO:0005575 "cellular_component" evidence=ND] [GO:0008150 "biological_process" evidence=ND] [GO:0006355 "regulation of transcription, DNA-dependent" evidence=IEA] [GO:0003674 "molecular_function" evidence=ND] SGD:S000028567 GO:GO:0006355 GO:GO:0006351 EMBL:BK006945 EMBL:U51921 RefSeq:NP_878128.1 ProteinModelPortal:Q3E811 STRING:Q3E811 EnsemblFungi:YLR162W-A GeneID:1466416 KEGG:sce:YLR162W-A CYGD:YLR162w-a GeneTree:ENSGT00530000064745 OMA:FYINCIM OrthoDB:EOG45TGXP NextBio:983475 ArrayExpress:Q3E811 Genevestigator:Q3E811 Uniprot:Q3E811
Root	Isotig00146	307	125	1.167	1.11E-15	SGD|S000028567 - symbol:RRT15 "Putative protein of unknown function" species:4932 "Saccharomyces cerevisiae" [GO:0006351 "transcription, DNA-dependent" evidence=IEA] [GO:0005575 "cellular_component" evidence=ND] [GO:0008150 "biological_process" evidence=ND] [GO:0006355 "regulation of transcription, DNA-dependent" evidence=IEA] [GO:0003674 "molecular_function" evidence=ND] SGD:S000028567 GO:GO:0006355 GO:GO:0006351 EMBL:BK006945 EMBL:U51921 RefSeq:NP_878128.1 ProteinModelPortal:Q3E811 STRING:Q3E811 EnsemblFungi:YLR162W-A GeneID:1466416 KEGG:sce:YLR162W-A CYGD:YLR162w-a GeneTree:ENSGT00530000064745 OMA:FYINCIM OrthoDB:EOG45TGXP NextBio:983475 ArrayExpress:Q3E811 Genevestigator:Q3E811 Uniprot:Q3E811
Root	Isotig00147	386	147	1.264	1.09E-21	SGD|S000028567 - symbol:RRT15 "Putative protein of unknown function" species:4932 "Saccharomyces cerevisiae" [GO:0006351 "transcription, DNA-dependent" evidence=IEA] [GO:0005575 "cellular_component" evidence=ND] [GO:0008150 "biological_process" evidence=ND] [GO:0006355 "regulation of transcription, DNA-dependent" evidence=IEA] [GO:0003674 "molecular_function" evidence=ND] SGD:S000028567 GO:GO:0006355 GO:GO:0006351 EMBL:BK006945 EMBL:U51921 RefSeq:NP_878128.1 ProteinModelPortal:Q3E811 STRING:Q3E811 EnsemblFungi:YLR162W-A GeneID:1466416 KEGG:sce:YLR162W-A CYGD:YLR162w-a GeneTree:ENSGT00530000064745 OMA:FYINCIM OrthoDB:EOG45TGXP NextBio:983475 ArrayExpress:Q3E811 Genevestigator:Q3E811 Uniprot:Q3E811
Root	Isotig00148	282	115	1.165	1.74E-14	SGD|S000028567 - symbol:RRT15 "Putative protein of unknown function" species:4932 "Saccharomyces cerevisiae" [GO:0006351 "transcription, DNA-dependent" evidence=IEA] [GO:0005575 "cellular_component" evidence=ND] [GO:0008150 "biological_process" evidence=ND] [GO:0006355 "regulation of transcription, DNA-dependent" evidence=IEA] [GO:0003674 "molecular_function" evidence=ND] SGD:S000028567 GO:GO:0006355 GO:GO:0006351 EMBL:BK006945 EMBL:U51921 RefSeq:NP_878128.1 ProteinModelPortal:Q3E811 STRING:Q3E811 EnsemblFungi:YLR162W-A GeneID:1466416 KEGG:sce:YLR162W-A CYGD:YLR162w-a GeneTree:ENSGT00530000064745 OMA:FYINCIM OrthoDB:EOG45TGXP NextBio:983475 ArrayExpress:Q3E811 Genevestigator:Q3E811 Uniprot:Q3E811
Root	Isotig00149	285	116	1.168	1.13E-14	SGD|S000028567 - symbol:RRT15 "Putative protein of unknown function" species:4932 "Saccharomyces cerevisiae" [GO:0006351 "transcription, DNA-dependent" evidence=IEA] [GO:0005575 "cellular_component" evidence=ND] [GO:0008150 "biological_process" evidence=ND] [GO:0006355 "regulation of transcription, DNA-dependent" evidence=IEA] [GO:0003674 "molecular_function" evidence=ND] SGD:S000028567 GO:GO:0006355 GO:GO:0006351 EMBL:BK006945 EMBL:U51921 RefSeq:NP_878128.1 ProteinModelPortal:Q3E811 STRING:Q3E811 EnsemblFungi:YLR162W-A GeneID:1466416 KEGG:sce:YLR162W-A CYGD:YLR162w-a GeneTree:ENSGT00530000064745 OMA:FYINCIM OrthoDB:EOG45TGXP NextBio:983475 ArrayExpress:Q3E811 Genevestigator:Q3E811 Uniprot:Q3E811

Tissue	Identification	# of reads (stress)	# of reads (control)	Fold Change	p-value	Description
Root	Isotig00150	387	147	1.268	7.80E-22	SGD|S000028567 - symbol:RRT15 "Putative protein of unknown function" species:4932 "Saccharomyces cerevisiae" [GO:0006351 "transcription, DNA-dependent" evidence=IEA] [GO:0005575 "cellular_component" evidence=ND] [GO:0008150 "biological_process" evidence=ND] [GO:0006355 "regulation of transcription, DNA-dependent" evidence=IEA] [GO:0003674 "molecular_function" evidence=ND] SGD:S000028567 GO:GO:0006355 GO:GO:0006351 EMBL:BK006945 EMBL:U51921 RefSeq:NP_878128.1 ProteinModelPortal:Q3E811 STRING:Q3E811 EnsemblFungi:YLR162W-A GeneID:1466416 KEGG:sce:YLR162W-A CYGD:YLR162w-a GeneTree:ENSGT00530000064745 OMA:FYINCIM OrthoDB:EOG45TGXP NextBio:983475 ArrayExpress:Q3E811 Genevestigator:Q3E811 Uniprot:Q3E811
Root	Isotig00151	373	145	1.234	2.65E-20	SGD|S000028567 - symbol:RRT15 "Putative protein of unknown function" species:4932 "Saccharomyces cerevisiae" [GO:0006351 "transcription, DNA-dependent" evidence=IEA] [GO:0005575 "cellular_component" evidence=ND] [GO:0008150 "biological_process" evidence=ND] [GO:0006355 "regulation of transcription, DNA-dependent" evidence=IEA] [GO:0003674 "molecular_function" evidence=ND] SGD:S000028567 GO:GO:0006355 GO:GO:0006351 EMBL:BK006945 EMBL:U51921 RefSeq:NP_878128.1 ProteinModelPortal:Q3E811 STRING:Q3E811 EnsemblFungi:YLR162W-A GeneID:1466416 KEGG:sce:YLR162W-A CYGD:YLR162w-a GeneTree:ENSGT00530000064745 OMA:FYINCIM OrthoDB:EOG45TGXP NextBio:983475 ArrayExpress:Q3E811 Genevestigator:Q3E811 Uniprot:Q3E811
Root	Isotig00152	376	146	1.236	1.71E-20	SGD|S000028567 - symbol:RRT15 "Putative protein of unknown function" species:4932 "Saccharomyces cerevisiae" [GO:0006351 "transcription, DNA-dependent" evidence=IEA] [GO:0005575 "cellular_component" evidence=ND] [GO:0008150 "biological_process" evidence=ND] [GO:0006355 "regulation of transcription, DNA-dependent" evidence=IEA] [GO:0003674 "molecular_function" evidence=ND] SGD:S000028567 GO:GO:0006355 GO:GO:0006351 EMBL:BK006945 EMBL:U51921 RefSeq:NP_878128.1 ProteinModelPortal:Q3E811 STRING:Q3E811 EnsemblFungi:YLR162W-A GeneID:1466416 KEGG:sce:YLR162W-A CYGD:YLR162w-a GeneTree:ENSGT00530000064745 OMA:FYINCIM OrthoDB:EOG45TGXP NextBio:983475 ArrayExpress:Q3E811 Genevestigator:Q3E811 Uniprot:Q3E811
Root	Isotig00153	349	139	1.199	2.56E-18	SGD|S000028567 - symbol:RRT15 "Putative protein of unknown function" species:4932 "Saccharomyces cerevisiae" [GO:0006351 "transcription, DNA-dependent" evidence=IEA] [GO:0005575 "cellular_component" evidence=ND] [GO:0008150 "biological_process" evidence=ND] [GO:0006355 "regulation of transcription, DNA-dependent" evidence=IEA] [GO:0003674 "molecular_function" evidence=ND] SGD:S000028567 GO:GO:0006355 GO:GO:0006351 EMBL:BK006945 EMBL:U51921 RefSeq:NP_878128.1 ProteinModelPortal:Q3E811 STRING:Q3E811 EnsemblFungi:YLR162W-A GeneID:1466416 KEGG:sce:YLR162W-A CYGD:YLR162w-a GeneTree:ENSGT00530000064745 OMA:FYINCIM OrthoDB:EOG45TGXP NextBio:983475 ArrayExpress:Q3E811 Genevestigator:Q3E811 Uniprot:Q3E811
Root	Isotig00154	386	147	1.264	1.09E-21	SGD|S000028567 - symbol:RRT15 "Putative protein of unknown function" species:4932 "Saccharomyces cerevisiae" [GO:0006351 "transcription, DNA-dependent" evidence=IEA] [GO:0005575 "cellular_component" evidence=ND] [GO:0008150 "biological_process" evidence=ND] [GO:0006355 "regulation of transcription, DNA-dependent" evidence=IEA] [GO:0003674 "molecular_function" evidence=ND] SGD:S000028567 GO:GO:0006355 GO:GO:0006351 EMBL:BK006945 EMBL:U51921 RefSeq:NP_878128.1 ProteinModelPortal:Q3E811 STRING:Q3E811 EnsemblFungi:YLR162W-A GeneID:1466416 KEGG:sce:YLR162W-A CYGD:YLR162w-a GeneTree:ENSGT00530000064745 OMA:FYINCIM OrthoDB:EOG45TGXP NextBio:983475 ArrayExpress:Q3E811 Genevestigator:Q3E811 Uniprot:Q3E811
Root	Isotig00155	386	147	1.264	1.09E-21	SGD|S000028567 - symbol:RRT15 "Putative protein of unknown function" species:4932 "Saccharomyces cerevisiae" [GO:0006351 "transcription, DNA-dependent" evidence=IEA] [GO:0005575 "cellular_component" evidence=ND] [GO:0008150 "biological_process" evidence=ND] [GO:0006355 "regulation of transcription, DNA-dependent" evidence=IEA] [GO:0003674 "molecular_function" evidence=ND] SGD:S000028567 GO:GO:0006355 GO:GO:0006351 EMBL:BK006945 EMBL:U51921 RefSeq:NP_878128.1 ProteinModelPortal:Q3E811 STRING:Q3E811 EnsemblFungi:YLR162W-A GeneID:1466416 KEGG:sce:YLR162W-A CYGD:YLR162w-a GeneTree:ENSGT00530000064745 OMA:FYINCIM OrthoDB:EOG45TGXP NextBio:983475 ArrayExpress:Q3E811 Genevestigator:Q3E811 Uniprot:Q3E811
Root	Isotig00156	350	139	1.203	1.85E-18	SGD|S000028567 - symbol:RRT15 "Putative protein of unknown function" species:4932 "Saccharomyces cerevisiae" [GO:0006351 "transcription, DNA-dependent" evidence=IEA] [GO:0005575 "cellular_component" evidence=ND] [GO:0008150 "biological_process" evidence=ND] [GO:0006355 "regulation of transcription, DNA-dependent" evidence=IEA] [GO:0003674 "molecular_function" evidence=ND] SGD:S000028567 GO:GO:0006355

Tissue	Identification	# of reads (stress)	# of reads (control)	Fold Change	p-value	Description
						GO:GO:0006351 EMBL:BK006945 EMBL:U51921 RefSeq:NP_878128.1 ProteinModelPortal:Q3E811 STRING:Q3E811 EnsemblFungi:YLR162W-A GeneID:1466416 KEGG:sce:YLR162W-A CYGD:YLR162w-a GeneTree:ENSGT00530000064745 OMA:FYINCIM OrthoDB:EOG45TGXP NextBio:983475 ArrayExpress:Q3E811 Genevestigator:Q3E811 Uniprot:Q3E811
Root	Isotig00157	387	147	1.268	7.80E-22	SGD|S000028567 - symbol:RRT15 "Putative protein of unknown function" species:4932 "Saccharomyces cerevisiae" [GO:0006351 "transcription, DNA-dependent" evidence=IEA] [GO:0005575 "cellular_component" evidence=ND] [GO:0008150 "biological_process" evidence=ND] [GO:0006355 "regulation of transcription, DNA-dependent" evidence=IEA] [GO:0003674 "molecular_function" evidence=ND] SGD:S000028567 GO:GO:0006355 GO:GO:0006351 EMBL:BK006945 EMBL:U51921 RefSeq:NP_878128.1 ProteinModelPortal:Q3E811 STRING:Q3E811 EnsemblFungi:YLR162W-A GeneID:1466416 KEGG:sce:YLR162W-A CYGD:YLR162w-a GeneTree:ENSGT00530000064745 OMA:FYINCIM OrthoDB:EOG45TGXP NextBio:983475 ArrayExpress:Q3E811 Genevestigator:Q3E811 Uniprot:Q3E811
Root	Isotig00158	387	147	1.268	7.80E-22	SGD|S000028567 - symbol:RRT15 "Putative protein of unknown function" species:4932 "Saccharomyces cerevisiae" [GO:0006351 "transcription, DNA-dependent" evidence=IEA] [GO:0005575 "cellular_component" evidence=ND] [GO:0008150 "biological_process" evidence=ND] [GO:0006355 "regulation of transcription, DNA-dependent" evidence=IEA] [GO:0003674 "molecular_function" evidence=ND] SGD:S000028567 GO:GO:0006355 GO:GO:0006351 EMBL:BK006945 EMBL:U51921 RefSeq:NP_878128.1 ProteinModelPortal:Q3E811 STRING:Q3E811 EnsemblFungi:YLR162W-A GeneID:1466416 KEGG:sce:YLR162W-A CYGD:YLR162w-a GeneTree:ENSGT00530000064745 OMA:FYINCIM OrthoDB:EOG45TGXP NextBio:983475 ArrayExpress:Q3E811 Genevestigator:Q3E811 Uniprot:Q3E811
Root	Isotig00159	336	137	1.165	5.64E-17	SGD|S000028567 - symbol:RRT15 "Putative protein of unknown function" species:4932 "Saccharomyces cerevisiae" [GO:0006351 "transcription, DNA-dependent" evidence=IEA] [GO:0005575 "cellular_component" evidence=ND] [GO:0008150 "biological_process" evidence=ND] [GO:0006355 "regulation of transcription, DNA-dependent" evidence=IEA] [GO:0003674 "molecular_function" evidence=ND] SGD:S000028567 GO:GO:0006355 GO:GO:0006351 EMBL:BK006945 EMBL:U51921 RefSeq:NP_878128.1 ProteinModelPortal:Q3E811 STRING:Q3E811 EnsemblFungi:YLR162W-A GeneID:1466416 KEGG:sce:YLR162W-A CYGD:YLR162w-a GeneTree:ENSGT00530000064745 OMA:FYINCIM OrthoDB:EOG45TGXP NextBio:983475 ArrayExpress:Q3E811 Genevestigator:Q3E811 Uniprot:Q3E811
Root	Isotig00160	339	138	1.168	3.66E-17	SGD|S000028567 - symbol:RRT15 "Putative protein of unknown function" species:4932 "Saccharomyces cerevisiae" [GO:0006351 "transcription, DNA-dependent" evidence=IEA] [GO:0005575 "cellular_component" evidence=ND] [GO:0008150 "biological_process" evidence=ND] [GO:0006355 "regulation of transcription, DNA-dependent" evidence=IEA] [GO:0003674 "molecular_function" evidence=ND] SGD:S000028567 GO:GO:0006355 GO:GO:0006351 EMBL:BK006945 EMBL:U51921 RefSeq:NP_878128.1 ProteinModelPortal:Q3E811 STRING:Q3E811 EnsemblFungi:YLR162W-A GeneID:1466416 KEGG:sce:YLR162W-A CYGD:YLR162w-a GeneTree:ENSGT00530000064745 OMA:FYINCIM OrthoDB:EOG45TGXP NextBio:983475 ArrayExpress:Q3E811 Genevestigator:Q3E811 Uniprot:Q3E811
Root	Isotig00161	373	145	1.234	2.65E-20	SGD|S000028567 - symbol:RRT15 "Putative protein of unknown function" species:4932 "Saccharomyces cerevisiae" [GO:0006351 "transcription, DNA-dependent" evidence=IEA] [GO:0005575 "cellular_component" evidence=ND] [GO:0008150 "biological_process" evidence=ND] [GO:0006355 "regulation of transcription, DNA-dependent" evidence=IEA] [GO:0003674 "molecular_function" evidence=ND] SGD:S000028567 GO:GO:0006355 GO:GO:0006351 EMBL:BK006945 EMBL:U51921 RefSeq:NP_878128.1 ProteinModelPortal:Q3E811 STRING:Q3E811 EnsemblFungi:YLR162W-A GeneID:1466416 KEGG:sce:YLR162W-A CYGD:YLR162w-a GeneTree:ENSGT00530000064745 OMA:FYINCIM OrthoDB:EOG45TGXP NextBio:983475 ArrayExpress:Q3E811 Genevestigator:Q3E811 Uniprot:Q3E811
Root	Isotig00162	376	146	1.236	1.71E-20	SGD|S000028567 - symbol:RRT15 "Putative protein of unknown function" species:4932 "Saccharomyces cerevisiae" [GO:0006351 "transcription, DNA-dependent" evidence=IEA] [GO:0005575 "cellular_component" evidence=ND] [GO:0008150 "biological_process" evidence=ND] [GO:0006355 "regulation of transcription, DNA-dependent" evidence=IEA] [GO:0003674 "molecular_function" evidence=ND] SGD:S000028567 GO:GO:0006355 GO:GO:0006351 EMBL:BK006945 EMBL:U51921 RefSeq:NP_878128.1 ProteinModelPortal:Q3E811 STRING:Q3E811 EnsemblFungi:YLR162W-A GeneID:1466416 KEGG:sce:YLR162W-A CYGD:YLR162w-a GeneTree:ENSGT00530000064745 OMA:FYINCIM OrthoDB:EOG45TGXP NextBio:983475 ArrayExpress:Q3E811 Genevestigator:Q3E811 Uniprot:Q3E811

Tissue	Identification	# of reads (stress)	# of reads (control)	Fold Change	p-value	Description
Root	Isotig00163	373	145	1.234	2.65E-20	SGD|S000028567 - symbol:RRT15 "Putative protein of unknown function" species:4932 "Saccharomyces cerevisiae" [GO:0006351 "transcription, DNA-dependent" evidence=IEA] [GO:0005575 "cellular_component" evidence=ND] [GO:0008150 "biological_process" evidence=ND] [GO:0006355 "regulation of transcription, DNA-dependent" evidence=IEA] [GO:0003674 "molecular_function" evidence=ND] SGD:S000028567 GO:GO:0006355 GO:GO:0006351 EMBL:BK006945 EMBL:U51921 RefSeq:NP_878128.1 ProteinModelPortal:Q3E811 STRING:Q3E811 EnsemblFungi:YLR162W-A GeneID:1466416 KEGG:sce:YLR162W-A CYGD:YLR162w-a GeneTree:ENSGT00530000064745 OMA:FYINCIM OrthoDB:EOG45TGXP NextBio:983475 ArrayExpress:Q3E811 Genevestigator:Q3E811 Uniprot:Q3E811
Root	Isotig00164	376	146	1.236	1.71E-20	SGD|S000028567 - symbol:RRT15 "Putative protein of unknown function" species:4932 "Saccharomyces cerevisiae" [GO:0006351 "transcription, DNA-dependent" evidence=IEA] [GO:0005575 "cellular_component" evidence=ND] [GO:0008150 "biological_process" evidence=ND] [GO:0006355 "regulation of transcription, DNA-dependent" evidence=IEA] [GO:0003674 "molecular_function" evidence=ND] SGD:S000028567 GO:GO:0006355 GO:GO:0006351 EMBL:BK006945 EMBL:U51921 RefSeq:NP_878128.1 ProteinModelPortal:Q3E811 STRING:Q3E811 EnsemblFungi:YLR162W-A GeneID:1466416 KEGG:sce:YLR162W-A CYGD:YLR162w-a GeneTree:ENSGT00530000064745 OMA:FYINCIM OrthoDB:EOG45TGXP NextBio:983475 ArrayExpress:Q3E811 Genevestigator:Q3E811 Uniprot:Q3E811
Root	Isotig00165	289	116	1.188	3.16E-15	SGD|S000028567 - symbol:RRT15 "Putative protein of unknown function" species:4932 "Saccharomyces cerevisiae" [GO:0006351 "transcription, DNA-dependent" evidence=IEA] [GO:0005575 "cellular_component" evidence=ND] [GO:0008150 "biological_process" evidence=ND] [GO:0006355 "regulation of transcription, DNA-dependent" evidence=IEA] [GO:0003674 "molecular_function" evidence=ND] SGD:S000028567 GO:GO:0006355 GO:GO:0006351 EMBL:BK006945 EMBL:U51921 RefSeq:NP_878128.1 ProteinModelPortal:Q3E811 STRING:Q3E811 EnsemblFungi:YLR162W-A GeneID:1466416 KEGG:sce:YLR162W-A CYGD:YLR162w-a GeneTree:ENSGT00530000064745 OMA:FYINCIM OrthoDB:EOG45TGXP NextBio:983475 ArrayExpress:Q3E811 Genevestigator:Q3E811 Uniprot:Q3E811
Root	Isotig00166	290	116	1.193	2.29E-15	SGD|S000028567 - symbol:RRT15 "Putative protein of unknown function" species:4932 "Saccharomyces cerevisiae" [GO:0006351 "transcription, DNA-dependent" evidence=IEA] [GO:0005575 "cellular_component" evidence=ND] [GO:0008150 "biological_process" evidence=ND] [GO:0006355 "regulation of transcription, DNA-dependent" evidence=IEA] [GO:0003674 "molecular_function" evidence=ND] SGD:S000028567 GO:GO:0006355 GO:GO:0006351 EMBL:BK006945 EMBL:U51921 RefSeq:NP_878128.1 ProteinModelPortal:Q3E811 STRING:Q3E811 EnsemblFungi:YLR162W-A GeneID:1466416 KEGG:sce:YLR162W-A CYGD:YLR162w-a GeneTree:ENSGT00530000064745 OMA:FYINCIM OrthoDB:EOG45TGXP NextBio:983475 ArrayExpress:Q3E811 Genevestigator:Q3E811 Uniprot:Q3E811
Root	Isotig00167	229	83	1.335	1.48E-14	SGD|S000028422 - symbol:TAR1 "Mitochondrial protein potentially involved in respiratory metabolism" species:4932 "Saccharomyces cerevisiae" [GO:0003674 "molecular_function" evidence=ND] [GO:0043457 "regulation of cellular respiration" evidence=IGI] [GO:0005739 "mitochondrion" evidence=IEA;IMP] SGD:S000028422 GO:GO:0005739 EMBL:BK006945 EMBL:U53879 GO:GO:0043457 EMBL:Z73326 EMBL:AF479964 RefSeq:NP_690845.1 ProteinModelPortal:Q8TGM6 STRING:Q8TGM6 EnsemblFungi:YLR154W-C GeneID:1466405 KEGG:sce:YLR154W-C CYGD:YLR154w-c eggNOG:NOG79861 OMA:SCSKEHR OrthoDB:EOG466ZX8 NextBio:967146 Genevestigator:Q8TGM6 GermOnline:YLR154W-C Uniprot:Q8TGM6
Root	Isotig00168	276	114	1.147	6.85E-14	SGD|S000028567 - symbol:RRT15 "Putative protein of unknown function" species:4932 "Saccharomyces cerevisiae" [GO:0006351 "transcription, DNA-dependent" evidence=IEA] [GO:0005575 "cellular_component" evidence=ND] [GO:0008150 "biological_process" evidence=ND] [GO:0006355 "regulation of transcription, DNA-dependent" evidence=IEA] [GO:0003674 "molecular_function" evidence=ND] SGD:S000028567 GO:GO:0006355 GO:GO:0006351 EMBL:BK006945 EMBL:U51921 RefSeq:NP_878128.1 ProteinModelPortal:Q3E811 STRING:Q3E811 EnsemblFungi:YLR162W-A GeneID:1466416 KEGG:sce:YLR162W-A CYGD:YLR162w-a GeneTree:ENSGT00530000064745 OMA:FYINCIM OrthoDB:EOG45TGXP NextBio:983475 ArrayExpress:Q3E811 Genevestigator:Q3E811 Uniprot:Q3E811
Root	Isotig00169	279	115	1.150	4.45E-14	SGD|S000028567 - symbol:RRT15 "Putative protein of unknown function" species:4932 "Saccharomyces cerevisiae" [GO:0006351 "transcription, DNA-dependent" evidence=IEA] [GO:0005575 "cellular_component" evidence=ND] [GO:0008150 "biological_process" evidence=ND] [GO:0006355 "regulation of transcription, DNA-dependent" evidence=IEA] [GO:0003674 "molecular_function" evidence=ND] SGD:S000028567 GO:GO:0006355 GO:GO:0006351 EMBL:BK006945 EMBL:U51921 RefSeq:NP_878128.1 ProteinModelPortal:Q3E811

Tissue	Identification	# of reads (stress)	# of reads (control)	Fold Change	p-value	Description
						STRING:Q3E811 EnsemblFungi:YLR162W-A GeneID:1466416 KEGG:sce:YLR162W-A CYGD:YLR162w-a GeneTree:ENSGT00530000064745 OMA:FYINCIM OrthoDB:EOG45TGXP NextBio:983475 ArrayExpress:Q3E811 Genevestigator:Q3E811 Uniprot:Q3E811
Root	Isotig00170	175	73	1.132	3.54E-09	SGD|S000028567 - symbol:RRT15 "Putative protein of unknown function" species:4932 "Saccharomyces cerevisiae" [GO:0006351 "transcription, DNA-dependent" evidence=IEA] [GO:0005575 "cellular_component" evidence=ND] [GO:0008150 "biological_process" evidence=ND] [GO:0006355 "regulation of transcription, DNA-dependent" evidence=IEA] [GO:0003674 "molecular_function" evidence=ND] SGD:S000028567 GO:GO:0006355 GO:GO:0006351 EMBL:BK006945 EMBL:U51921 RefSeq:NP_878128.1 ProteinModelPortal:Q3E811 STRING:Q3E811 EnsemblFungi:YLR162W-A GeneID:1466416 KEGG:sce:YLR162W-A CYGD:YLR162w-a GeneTree:ENSGT00530000064745 OMA:FYINCIM OrthoDB:EOG45TGXP NextBio:983475 ArrayExpress:Q3E811 Genevestigator:Q3E811 Uniprot:Q3E811
Root	Isotig00171	207	74	1.355	1.43E-13	SGD|S000028422 - symbol:TAR1 "Mitochondrial protein potentially involved in respiratory metabolism" species:4932 "Saccharomyces cerevisiae" [GO:0003674 "molecular_function" evidence=ND] [GO:0043457 "regulation of cellular respiration" evidence=IGI] [GO:0005739 "mitochondrion" evidence=IEA;IMP] SGD:S000028422 GO:GO:0005739 EMBL:BK006945 EMBL:U53879 GO:GO:0043457 EMBL:Z73326 EMBL:AF479964 RefSeq:NP_690845.1 ProteinModelPortal:Q8TGM6 STRING:Q8TGM6 EnsemblFungi:YLR154W-C GeneID:1466405 KEGG:sce:YLR154W-C CYGD:YLR154w-c eggNOG:NOG79861 OMA:SCSKEHR OrthoDB:EOG466ZX8 NextBio:967146 Genevestigator:Q8TGM6 GermOnline:YLR154W-C Uniprot:Q8TGM6
Root	Isotig00172	138	65	0.957	4.91E-06	SGD|S000028567 - symbol:RRT15 "Putative protein of unknown function" species:4932 "Saccharomyces cerevisiae" [GO:0006351 "transcription, DNA-dependent" evidence=IEA] [GO:0005575 "cellular_component" evidence=ND] [GO:0008150 "biological_process" evidence=ND] [GO:0006355 "regulation of transcription, DNA-dependent" evidence=IEA] [GO:0003674 "molecular_function" evidence=ND] SGD:S000028567 GO:GO:0006355 GO:GO:0006351 EMBL:BK006945 EMBL:U51921 RefSeq:NP_878128.1 ProteinModelPortal:Q3E811 STRING:Q3E811 EnsemblFungi:YLR162W-A GeneID:1466416 KEGG:sce:YLR162W-A CYGD:YLR162w-a GeneTree:ENSGT00530000064745 OMA:FYINCIM OrthoDB:EOG45TGXP NextBio:983475 ArrayExpress:Q3E811 Genevestigator:Q3E811 Uniprot:Q3E811
Root	Isotig00173	175	73	1.132	3.54E-09	SGD|S000028567 - symbol:RRT15 "Putative protein of unknown function" species:4932 "Saccharomyces cerevisiae" [GO:0006351 "transcription, DNA-dependent" evidence=IEA] [GO:0005575 "cellular_component" evidence=ND] [GO:0008150 "biological_process" evidence=ND] [GO:0006355 "regulation of transcription, DNA-dependent" evidence=IEA] [GO:0003674 "molecular_function" evidence=ND] SGD:S000028567 GO:GO:0006355 GO:GO:0006351 EMBL:BK006945 EMBL:U51921 RefSeq:NP_878128.1 ProteinModelPortal:Q3E811 STRING:Q3E811 EnsemblFungi:YLR162W-A GeneID:1466416 KEGG:sce:YLR162W-A CYGD:YLR162w-a GeneTree:ENSGT00530000064745 OMA:FYINCIM OrthoDB:EOG45TGXP NextBio:983475 ArrayExpress:Q3E811 Genevestigator:Q3E811 Uniprot:Q3E811
Root	Isotig00174	175	73	1.132	3.54E-09	SGD|S000028567 - symbol:RRT15 "Putative protein of unknown function" species:4932 "Saccharomyces cerevisiae" [GO:0006351 "transcription, DNA-dependent" evidence=IEA] [GO:0005575 "cellular_component" evidence=ND] [GO:0008150 "biological_process" evidence=ND] [GO:0006355 "regulation of transcription, DNA-dependent" evidence=IEA] [GO:0003674 "molecular_function" evidence=ND] SGD:S000028567 GO:GO:0006355 GO:GO:0006351 EMBL:BK006945 EMBL:U51921 RefSeq:NP_878128.1 ProteinModelPortal:Q3E811 STRING:Q3E811 EnsemblFungi:YLR162W-A GeneID:1466416 KEGG:sce:YLR162W-A CYGD:YLR162w-a GeneTree:ENSGT00530000064745 OMA:FYINCIM OrthoDB:EOG45TGXP NextBio:983475 ArrayExpress:Q3E811 Genevestigator:Q3E811 Uniprot:Q3E811
Root	Isotig00176	201	73	1.332	6.41E-13	RGD|727924 - symbol:LOC257642 "rRNA promoter binding protein" species:10116 "Rattus norvegicus" [GO:0003690 "double-stranded DNA binding" evidence=IDA] [GO:0003700 "sequence-specific DNA binding transcription factor activity" evidence=IDA] [GO:0008134 "transcription factor binding" evidence=IDA] [GO:0008283 "cell proliferation" evidence=IDA] [GO:0009303 "rRNA transcription" evidence=IDA] RGD:727924 GO:GO:0008134 GO:GO:0008283 GO:GO:0003700 GO:GO:0003690 GO:GO:0009303 EMBL:U77931 IPI:IPI00195015 RefSeq:NP_671477.1 GeneID:257642 KEGG:rno:257642 NextBio:624170 Genevestigator:Q99JC0 Uniprot:Q99JC0
Root	Isotig00177	92	26	1.694	6.24E-09	SGD|S000028567 - symbol:RRT15 "Putative protein of unknown function" species:4932 "Saccharomyces cerevisiae" [GO:0006351 "transcription, DNA-dependent" evidence=IEA] [GO:0005575 "cellular_component" evidence=ND] [GO:0008150 "biological_process" evidence=ND] [GO:0006355 "regulation of transcription, DNA-dependent" evidence=IEA] [GO:0003674 "molecular_function" evidence=ND] SGD:S000028567 GO:GO:0006355

Tissue	Identification	# of reads (stress)	# of reads (control)	Fold Change	p-value	Description
						GO:GO:0006351 EMBL:BK006945 EMBL:U51921 RefSeq:NP_878128.1 ProteinModelPortal:Q3E811 STRING:Q3E811 EnsemblFungi:YLR162W-A GeneID:1466416 KEGG:sce:YLR162W-A CYGD:YLR162w-a GeneTree:ENSGT00530000064745 OMA:FYINCIM OrthoDB:EOG45TGXP NextBio:983475 ArrayExpress:Q3E811 Genevestigator:Q3E811 Uniprot:Q3E811
Root	Isotig00178	92	26	1.694	6.24E-09	SGD|S000028567 - symbol:RRT15 "Putative protein of unknown function" species:4932 "Saccharomyces cerevisiae" [GO:0006351 "transcription, DNA-dependent" evidence=IEA] [GO:0005575 "cellular_component" evidence=ND] [GO:0008150 "biological_process" evidence=ND] [GO:0006355 "regulation of transcription, DNA-dependent" evidence=IEA] [GO:0003674 "molecular_function" evidence=ND] SGD:S000028567 GO:GO:0006355 GO:GO:0006351 EMBL:BK006945 EMBL:U51921 RefSeq:NP_878128.1 ProteinModelPortal:Q3E811 STRING:Q3E811 EnsemblFungi:YLR162W-A GeneID:1466416 KEGG:sce:YLR162W-A CYGD:YLR162w-a GeneTree:ENSGT00530000064745 OMA:FYINCIM OrthoDB:EOG45TGXP NextBio:983475 ArrayExpress:Q3E811 Genevestigator:Q3E811 Uniprot:Q3E811
Root	Isotig00179	92	26	1.694	6.24E-09	SGD|S000028567 - symbol:RRT15 "Putative protein of unknown function" species:4932 "Saccharomyces cerevisiae" [GO:0006351 "transcription, DNA-dependent" evidence=IEA] [GO:0005575 "cellular_component" evidence=ND] [GO:0008150 "biological_process" evidence=ND] [GO:0006355 "regulation of transcription, DNA-dependent" evidence=IEA] [GO:0003674 "molecular_function" evidence=ND] SGD:S000028567 GO:GO:0006355 GO:GO:0006351 EMBL:BK006945 EMBL:U51921 RefSeq:NP_878128.1 ProteinModelPortal:Q3E811 STRING:Q3E811 EnsemblFungi:YLR162W-A GeneID:1466416 KEGG:sce:YLR162W-A CYGD:YLR162w-a GeneTree:ENSGT00530000064745 OMA:FYINCIM OrthoDB:EOG45TGXP NextBio:983475 ArrayExpress:Q3E811 Genevestigator:Q3E811 Uniprot:Q3E811
Root	Isotig00180	94	188	-1.129	1.72E-10	TAIR|locus:2120532 - symbol:CSLC12 "AT4G07960" species:3702 "Arabidopsis thaliana" [GO:0016757 "transferase activity, transferring glycosyl groups" evidence=ISS] [GO:0016759 "cellulose synthase activity" evidence=ISS] [GO:0009506 "plasmodesma" evidence=IDA] GO:GO:0016021 GO:GO:0009506 EMBL:CP002687 GenomeReviews:CT486007_GR GO:GO:0000139 GO:GO:0007047 GO:GO:0016757 CAZy:GT2 eggNOG:COG1215 HOGENOM:HBG602904 EMBL:AC006266 EMBL:AL161508 EMBL:AK118480 EMBL:AY087066 EMBL:BT008770 IPI:IPI00545815 PIR:B85078 RefSeq:NP_192536.1 UniGene:At.33785 ProteinModelPortal:Q9ZQB9 STRING:Q9ZQB9 PRIDE:Q9ZQB9 EnsemblPlants:AT4G07960.1 GeneID:826301 KEGG:ath:AT4G07960 TAIR:At4g07960 InParanoid:Q9ZQB9 OMA:FEIAAYF PhylomeDB:Q9ZQB9 ProtClustDB:CLSN2685849 ArrayExpress:Q9ZQB9 Genevestigator:Q9ZQB9 Uniprot:Q9ZQB9
Root	Isotig00181	94	188	-1.129	1.72E-10	TAIR|locus:2120532 - symbol:CSLC12 "AT4G07960" species:3702 "Arabidopsis thaliana" [GO:0016757 "transferase activity, transferring glycosyl groups" evidence=ISS] [GO:0016759 "cellulose synthase activity" evidence=ISS] [GO:0009506 "plasmodesma" evidence=IDA] GO:GO:0016021 GO:GO:0009506 EMBL:CP002687 GenomeReviews:CT486007_GR GO:GO:0000139 GO:GO:0007047 GO:GO:0016757 CAZy:GT2 eggNOG:COG1215 HOGENOM:HBG602904 EMBL:AC006266 EMBL:AL161508 EMBL:AK118480 EMBL:AY087066 EMBL:BT008770 IPI:IPI00545815 PIR:B85078 RefSeq:NP_192536.1 UniGene:At.33785 ProteinModelPortal:Q9ZQB9 STRING:Q9ZQB9 PRIDE:Q9ZQB9 EnsemblPlants:AT4G07960.1 GeneID:826301 KEGG:ath:AT4G07960 TAIR:At4g07960 InParanoid:Q9ZQB9 OMA:FEIAAYF PhylomeDB:Q9ZQB9 ProtClustDB:CLSN2685849 ArrayExpress:Q9ZQB9 Genevestigator:Q9ZQB9 Uniprot:Q9ZQB9
Root	Isotig00182	94	188	-1.129	1.72E-10	TAIR|locus:2120532 - symbol:CSLC12 "AT4G07960" species:3702 "Arabidopsis thaliana" [GO:0016757 "transferase activity, transferring glycosyl groups" evidence=ISS] [GO:0016759 "cellulose synthase activity" evidence=ISS] [GO:0009506 "plasmodesma" evidence=IDA] GO:GO:0016021 GO:GO:0009506 EMBL:CP002687 GenomeReviews:CT486007_GR GO:GO:0000139 GO:GO:0007047 GO:GO:0016757 CAZy:GT2 eggNOG:COG1215 HOGENOM:HBG602904 EMBL:AC006266 EMBL:AL161508 EMBL:AK118480 EMBL:AY087066 EMBL:BT008770 IPI:IPI00545815 PIR:B85078 RefSeq:NP_192536.1 UniGene:At.33785 ProteinModelPortal:Q9ZQB9 STRING:Q9ZQB9 PRIDE:Q9ZQB9 EnsemblPlants:AT4G07960.1 GeneID:826301 KEGG:ath:AT4G07960 TAIR:At4g07960 InParanoid:Q9ZQB9 OMA:FEIAAYF PhylomeDB:Q9ZQB9 ProtClustDB:CLSN2685849 ArrayExpress:Q9ZQB9 Genevestigator:Q9ZQB9 Uniprot:Q9ZQB9
Root	Isotig00183	94	189	-1.137	1.23E-10	TAIR|locus:2120532 - symbol:CSLC12 "AT4G07960" species:3702 "Arabidopsis thaliana" [GO:0016757 "transferase activity, transferring glycosyl groups" evidence=ISS] [GO:0016759 "cellulose synthase activity" evidence=ISS] [GO:0009506 "plasmodesma" evidence=IDA] GO:GO:0016021 GO:GO:0009506 EMBL:CP002687 GenomeReviews:CT486007_GR GO:GO:0000139 GO:GO:0007047 GO:GO:0016757 CAZy:GT2 eggNOG:COG1215 HOGENOM:HBG602904 EMBL:AC006266 EMBL:AL161508 EMBL:AK118480

Tissue	Identification	# of reads (stress)	# of reads (control)	Fold Change	p-value	Description
						EMBL:AY087066 EMBL:BT008770 IPI:IPI00545815 PIR:B85078 RefSeq:NP_192536.1 UniGene:At.33785 ProteinModelPortal:Q9ZQB9 STRING:Q9ZQB9 PRIDE:Q9ZQB9 EnsemblPlants:AT4G07960.1 GeneID:826301 KEGG:ath:AT4G07960 TAIR:At4g07960 InParanoid:Q9ZQB9 OMA:FEIAAYF PhylomeDB:Q9ZQB9 ProtClustDB:CLSN2685849 ArrayExpress:Q9ZQB9 Genevestigator:Q9ZQB9 Uniprot:Q9ZQB9
Root	Isotig00184	94	189	-1.137	1.23E-10	TAIR|locus:2120532 - symbol:CSLC12 "AT4G07960" species:3702 "Arabidopsis thaliana" [GO:0016757 "transferase activity, transferring glycosyl groups" evidence=ISS] [GO:0016759 "cellulose synthase activity" evidence=ISS] [GO:0009506 "plasmodesma" evidence=IDA] GO:GO:0016021 GO:GO:0009506 EMBL:CP002687 GenomeReviews:CT486007_GR GO:GO:0000139 GO:GO:0007047 GO:GO:0016757 CAZy:GT2 eggNOG:COG1215 HOGENOM:HBG602904 EMBL:AC006266 EMBL:AL161508 EMBL:AK118480 EMBL:AY087066 EMBL:BT008770 IPI:IPI00545815 PIR:B85078 RefSeq:NP_192536.1 UniGene:At.33785 ProteinModelPortal:Q9ZQB9 STRING:Q9ZQB9 PRIDE:Q9ZQB9 EnsemblPlants:AT4G07960.1 GeneID:826301 KEGG:ath:AT4G07960 TAIR:At4g07960 InParanoid:Q9ZQB9 OMA:FEIAAYF PhylomeDB:Q9ZQB9 ProtClustDB:CLSN2685849 ArrayExpress:Q9ZQB9 Genevestigator:Q9ZQB9 Uniprot:Q9ZQB9
Root	Isotig00185	90	179	-1.121	5.87E-10	TAIR|locus:2120532 - symbol:CSLC12 "AT4G07960" species:3702 "Arabidopsis thaliana" [GO:0016757 "transferase activity, transferring glycosyl groups" evidence=ISS] [GO:0016759 "cellulose synthase activity" evidence=ISS] [GO:0009506 "plasmodesma" evidence=IDA] GO:GO:0016021 GO:GO:0009506 EMBL:CP002687 GenomeReviews:CT486007_GR GO:GO:0000139 GO:GO:0007047 GO:GO:0016757 CAZy:GT2 eggNOG:COG1215 HOGENOM:HBG602904 EMBL:AC006266 EMBL:AL161508 EMBL:AK118480 EMBL:AY087066 EMBL:BT008770 IPI:IPI00545815 PIR:B85078 RefSeq:NP_192536.1 UniGene:At.33785 ProteinModelPortal:Q9ZQB9 STRING:Q9ZQB9 PRIDE:Q9ZQB9 EnsemblPlants:AT4G07960.1 GeneID:826301 KEGG:ath:AT4G07960 TAIR:At4g07960 InParanoid:Q9ZQB9 OMA:FEIAAYF PhylomeDB:Q9ZQB9 ProtClustDB:CLSN2685849 ArrayExpress:Q9ZQB9 Genevestigator:Q9ZQB9 Uniprot:Q9ZQB9
Root	Isotig00186	90	179	-1.121	5.87E-10	TAIR|locus:2120532 - symbol:CSLC12 "AT4G07960" species:3702 "Arabidopsis thaliana" [GO:0016757 "transferase activity, transferring glycosyl groups" evidence=ISS] [GO:0016759 "cellulose synthase activity" evidence=ISS] [GO:0009506 "plasmodesma" evidence=IDA] GO:GO:0016021 GO:GO:0009506 EMBL:CP002687 GenomeReviews:CT486007_GR GO:GO:0000139 GO:GO:0007047 GO:GO:0016757 CAZy:GT2 eggNOG:COG1215 HOGENOM:HBG602904 EMBL:AC006266 EMBL:AL161508 EMBL:AK118480 EMBL:AY087066 EMBL:BT008770 IPI:IPI00545815 PIR:B85078 RefSeq:NP_192536.1 UniGene:At.33785 ProteinModelPortal:Q9ZQB9 STRING:Q9ZQB9 PRIDE:Q9ZQB9 EnsemblPlants:AT4G07960.1 GeneID:826301 KEGG:ath:AT4G07960 TAIR:At4g07960 InParanoid:Q9ZQB9 OMA:FEIAAYF PhylomeDB:Q9ZQB9 ProtClustDB:CLSN2685849 ArrayExpress:Q9ZQB9 Genevestigator:Q9ZQB9 Uniprot:Q9ZQB9
Root	Isotig00187	90	179	-1.121	5.87E-10	TAIR|locus:2120532 - symbol:CSLC12 "AT4G07960" species:3702 "Arabidopsis thaliana" [GO:0016757 "transferase activity, transferring glycosyl groups" evidence=ISS] [GO:0016759 "cellulose synthase activity" evidence=ISS] [GO:0009506 "plasmodesma" evidence=IDA] GO:GO:0016021 GO:GO:0009506 EMBL:CP002687 GenomeReviews:CT486007_GR GO:GO:0000139 GO:GO:0007047 GO:GO:0016757 CAZy:GT2 eggNOG:COG1215 HOGENOM:HBG602904 EMBL:AC006266 EMBL:AL161508 EMBL:AK118480 EMBL:AY087066 EMBL:BT008770 IPI:IPI00545815 PIR:B85078 RefSeq:NP_192536.1 UniGene:At.33785 ProteinModelPortal:Q9ZQB9 STRING:Q9ZQB9 PRIDE:Q9ZQB9 EnsemblPlants:AT4G07960.1 GeneID:826301 KEGG:ath:AT4G07960 TAIR:At4g07960 InParanoid:Q9ZQB9 OMA:FEIAAYF PhylomeDB:Q9ZQB9 ProtClustDB:CLSN2685849 ArrayExpress:Q9ZQB9 Genevestigator:Q9ZQB9 Uniprot:Q9ZQB9
Root	Isotig00188	90	180	-1.129	4.19E-10	TAIR|locus:2120532 - symbol:CSLC12 "AT4G07960" species:3702 "Arabidopsis thaliana" [GO:0016757 "transferase activity, transferring glycosyl groups" evidence=ISS] [GO:0016759 "cellulose synthase activity" evidence=ISS] [GO:0009506 "plasmodesma" evidence=IDA] GO:GO:0016021 GO:GO:0009506 EMBL:CP002687 GenomeReviews:CT486007_GR GO:GO:0000139 GO:GO:0007047 GO:GO:0016757 CAZy:GT2 eggNOG:COG1215 HOGENOM:HBG602904 EMBL:AC006266 EMBL:AL161508 EMBL:AK118480 EMBL:AY087066 EMBL:BT008770 IPI:IPI00545815 PIR:B85078 RefSeq:NP_192536.1 UniGene:At.33785 ProteinModelPortal:Q9ZQB9 STRING:Q9ZQB9 PRIDE:Q9ZQB9 EnsemblPlants:AT4G07960.1 GeneID:826301 KEGG:ath:AT4G07960 TAIR:At4g07960 InParanoid:Q9ZQB9 OMA:FEIAAYF PhylomeDB:Q9ZQB9 ProtClustDB:CLSN2685849 ArrayExpress:Q9ZQB9 Genevestigator:Q9ZQB9 Uniprot:Q9ZQB9
Root	Isotig00189	91	185	-1.153	1.20E-10	TAIR|locus:2120532 - symbol:CSLC12 "AT4G07960" species:3702 "Arabidopsis thaliana" [GO:0016757 "transferase activity, transferring glycosyl groups" evidence=ISS] [GO:0016759 "cellulose synthase activity" evidence=ISS] [GO:0009506 "plasmodesma" evidence=IDA] GO:GO:0016021 GO:GO:0009506 EMBL:CP002687

Tissue	Identification	# of reads (stress)	# of reads (control)	Fold Change	p-value	Description
						GenomeReviews:CT486007_GR GO:GO:0000139 GO:GO:0007047 GO:GO:0016757 CAZy:GT2 eggNOG:COG1215 HOGENOM:HBG602904 EMBL:AC006266 EMBL:AL161508 EMBL:AK118480 EMBL:AY087066 EMBL:BT008770 IPI:IPI00545815 PIR:B85078 RefSeq:NP_192536.1 UniGene:At.33785 ProteinModelPortal:Q9ZQB9 STRING:Q9ZQB9 PRIDE:Q9ZQB9 EnsemblPlants:AT4G07960.1 GeneID:826301 KEGG:ath:AT4G07960 TAIR:At4g07960 InParanoid:Q9ZQB9 OMA:FEIAAYF PhylomeDB:Q9ZQB9 ProtClustDB:CLSN2685849 ArrayExpress:Q9ZQB9 Genevestigator:Q9ZQB9 Uniprot:Q9ZQB9
Root	Isotig00190	90	180	-1.129	4.19E-10	TAIR|locus:2120532 - symbol:CSLC12 "AT4G07960" species:3702 "Arabidopsis thaliana" [GO:0016757 "transferase activity, transferring glycosyl groups" evidence=ISS] [GO:0016759 "cellulose synthase activity" evidence=ISS] [GO:0009506 "plasmodesma" evidence=IDA] GO:GO:0016021 GO:GO:0009506 EMBL:CP002687 GenomeReviews:CT486007_GR GO:GO:0000139 GO:GO:0007047 GO:GO:0016757 CAZy:GT2 eggNOG:COG1215 HOGENOM:HBG602904 EMBL:AC006266 EMBL:AL161508 EMBL:AK118480 EMBL:AY087066 EMBL:BT008770 IPI:IPI00545815 PIR:B85078 RefSeq:NP_192536.1 UniGene:At.33785 ProteinModelPortal:Q9ZQB9 STRING:Q9ZQB9 PRIDE:Q9ZQB9 EnsemblPlants:AT4G07960.1 GeneID:826301 KEGG:ath:AT4G07960 TAIR:At4g07960 InParanoid:Q9ZQB9 OMA:FEIAAYF PhylomeDB:Q9ZQB9 ProtClustDB:CLSN2685849 ArrayExpress:Q9ZQB9 Genevestigator:Q9ZQB9 Uniprot:Q9ZQB9
Root	Isotig00191	87	176	-1.145	4.12E-10	TAIR|locus:2120532 - symbol:CSLC12 "AT4G07960" species:3702 "Arabidopsis thaliana" [GO:0016757 "transferase activity, transferring glycosyl groups" evidence=ISS] [GO:0016759 "cellulose synthase activity" evidence=ISS] [GO:0009506 "plasmodesma" evidence=IDA] GO:GO:0016021 GO:GO:0009506 EMBL:CP002687 GenomeReviews:CT486007_GR GO:GO:0000139 GO:GO:0007047 GO:GO:0016757 CAZy:GT2 eggNOG:COG1215 HOGENOM:HBG602904 EMBL:AC006266 EMBL:AL161508 EMBL:AK118480 EMBL:AY087066 EMBL:BT008770 IPI:IPI00545815 PIR:B85078 RefSeq:NP_192536.1 UniGene:At.33785 ProteinModelPortal:Q9ZQB9 STRING:Q9ZQB9 PRIDE:Q9ZQB9 EnsemblPlants:AT4G07960.1 GeneID:826301 KEGG:ath:AT4G07960 TAIR:At4g07960 InParanoid:Q9ZQB9 OMA:FEIAAYF PhylomeDB:Q9ZQB9 ProtClustDB:CLSN2685849 ArrayExpress:Q9ZQB9 Genevestigator:Q9ZQB9 Uniprot:Q9ZQB9
Root	Isotig00192	94	187	-1.121	2.42E-10	TAIR|locus:2120532 - symbol:CSLC12 "AT4G07960" species:3702 "Arabidopsis thaliana" [GO:0016757 "transferase activity, transferring glycosyl groups" evidence=ISS] [GO:0016759 "cellulose synthase activity" evidence=ISS] [GO:0009506 "plasmodesma" evidence=IDA] GO:GO:0016021 GO:GO:0009506 EMBL:CP002687 GenomeReviews:CT486007_GR GO:GO:0000139 GO:GO:0007047 GO:GO:0016757 CAZy:GT2 eggNOG:COG1215 HOGENOM:HBG602904 EMBL:AC006266 EMBL:AL161508 EMBL:AK118480 EMBL:AY087066 EMBL:BT008770 IPI:IPI00545815 PIR:B85078 RefSeq:NP_192536.1 UniGene:At.33785 ProteinModelPortal:Q9ZQB9 STRING:Q9ZQB9 PRIDE:Q9ZQB9 EnsemblPlants:AT4G07960.1 GeneID:826301 KEGG:ath:AT4G07960 TAIR:At4g07960 InParanoid:Q9ZQB9 OMA:FEIAAYF PhylomeDB:Q9ZQB9 ProtClustDB:CLSN2685849 ArrayExpress:Q9ZQB9 Genevestigator:Q9ZQB9 Uniprot:Q9ZQB9
Root	Isotig00193	94	187	-1.121	2.42E-10	TAIR|locus:2120532 - symbol:CSLC12 "AT4G07960" species:3702 "Arabidopsis thaliana" [GO:0016757 "transferase activity, transferring glycosyl groups" evidence=ISS] [GO:0016759 "cellulose synthase activity" evidence=ISS] [GO:0009506 "plasmodesma" evidence=IDA] GO:GO:0016021 GO:GO:0009506 EMBL:CP002687 GenomeReviews:CT486007_GR GO:GO:0000139 GO:GO:0007047 GO:GO:0016757 CAZy:GT2 eggNOG:COG1215 HOGENOM:HBG602904 EMBL:AC006266 EMBL:AL161508 EMBL:AK118480 EMBL:AY087066 EMBL:BT008770 IPI:IPI00545815 PIR:B85078 RefSeq:NP_192536.1 UniGene:At.33785 ProteinModelPortal:Q9ZQB9 STRING:Q9ZQB9 PRIDE:Q9ZQB9 EnsemblPlants:AT4G07960.1 GeneID:826301 KEGG:ath:AT4G07960 TAIR:At4g07960 InParanoid:Q9ZQB9 OMA:FEIAAYF PhylomeDB:Q9ZQB9 ProtClustDB:CLSN2685849 ArrayExpress:Q9ZQB9 Genevestigator:Q9ZQB9 Uniprot:Q9ZQB9
Root	Isotig00194	94	187	-1.121	2.42E-10	TAIR|locus:2120532 - symbol:CSLC12 "AT4G07960" species:3702 "Arabidopsis thaliana" [GO:0016757 "transferase activity, transferring glycosyl groups" evidence=ISS] [GO:0016759 "cellulose synthase activity" evidence=ISS] [GO:0009506 "plasmodesma" evidence=IDA] GO:GO:0016021 GO:GO:0009506 EMBL:CP002687 GenomeReviews:CT486007_GR GO:GO:0000139 GO:GO:0007047 GO:GO:0016757 CAZy:GT2 eggNOG:COG1215 HOGENOM:HBG602904 EMBL:AC006266 EMBL:AL161508 EMBL:AK118480 EMBL:AY087066 EMBL:BT008770 IPI:IPI00545815 PIR:B85078 RefSeq:NP_192536.1 UniGene:At.33785 ProteinModelPortal:Q9ZQB9 STRING:Q9ZQB9 PRIDE:Q9ZQB9 EnsemblPlants:AT4G07960.1 GeneID:826301 KEGG:ath:AT4G07960 TAIR:At4g07960 InParanoid:Q9ZQB9 OMA:FEIAAYF PhylomeDB:Q9ZQB9 ProtClustDB:CLSN2685849 ArrayExpress:Q9ZQB9 Genevestigator:Q9ZQB9 Uniprot:Q9ZQB9

Tissue	Identification	# of reads (stress)	# of reads (control)	Fold Change	p-value	Description
Root	Isotig00195	94	188	-1.129	1.72E-10	TAIR|locus:2120532 - symbol:CSLC12 "AT4G07960" species:3702 "Arabidopsis thaliana" [GO:0016757 "transferase activity, transferring glycosyl groups" evidence=ISS] [GO:0016759 "cellulose synthase activity" evidence=ISS] [GO:0009506 "plasmodesma" evidence=IDA] GO:GO:0016021 GO:GO:0009506 EMBL:CP002687 GenomeReviews:CT486007_GR GO:GO:0000139 GO:GO:0007047 GO:GO:0016757 CAZy:GT2 eggNOG:COG1215 HOGENOM:HBG602904 EMBL:AC006266 EMBL:AL161508 EMBL:AK118480 EMBL:AY087066 EMBL:BT008770 IPI:IPI00545815 PIR:B85078 RefSeq:NP_192536.1 UniGene:At.33785 ProteinModelPortal:Q9ZQB9 STRING:Q9ZQB9 PRIDE:Q9ZQB9 EnsemblPlants:AT4G07960.1 GeneID:826301 KEGG:ath:AT4G07960 TAIR:At4g07960 InParanoid:Q9ZQB9 OMA:FEIAAYF PhylomeDB:Q9ZQB9 ProtClustDB:CLSN2685849 ArrayExpress:Q9ZQB9 Genevestigator:Q9ZQB9 Uniprot:Q9ZQB9
Root	Isotig00196	94	188	-1.129	1.72E-10	TAIR|locus:2120532 - symbol:CSLC12 "AT4G07960" species:3702 "Arabidopsis thaliana" [GO:0016757 "transferase activity, transferring glycosyl groups" evidence=ISS] [GO:0016759 "cellulose synthase activity" evidence=ISS] [GO:0009506 "plasmodesma" evidence=IDA] GO:GO:0016021 GO:GO:0009506 EMBL:CP002687 GenomeReviews:CT486007_GR GO:GO:0000139 GO:GO:0007047 GO:GO:0016757 CAZy:GT2 eggNOG:COG1215 HOGENOM:HBG602904 EMBL:AC006266 EMBL:AL161508 EMBL:AK118480 EMBL:AY087066 EMBL:BT008770 IPI:IPI00545815 PIR:B85078 RefSeq:NP_192536.1 UniGene:At.33785 ProteinModelPortal:Q9ZQB9 STRING:Q9ZQB9 PRIDE:Q9ZQB9 EnsemblPlants:AT4G07960.1 GeneID:826301 KEGG:ath:AT4G07960 TAIR:At4g07960 InParanoid:Q9ZQB9 OMA:FEIAAYF PhylomeDB:Q9ZQB9 ProtClustDB:CLSN2685849 ArrayExpress:Q9ZQB9 Genevestigator:Q9ZQB9 Uniprot:Q9ZQB9
Root	Isotig00197	91	184	-1.145	1.70E-10	TAIR|locus:2120532 - symbol:CSLC12 "AT4G07960" species:3702 "Arabidopsis thaliana" [GO:0016757 "transferase activity, transferring glycosyl groups" evidence=ISS] [GO:0016759 "cellulose synthase activity" evidence=ISS] [GO:0009506 "plasmodesma" evidence=IDA] GO:GO:0016021 GO:GO:0009506 EMBL:CP002687 GenomeReviews:CT486007_GR GO:GO:0000139 GO:GO:0007047 GO:GO:0016757 CAZy:GT2 eggNOG:COG1215 HOGENOM:HBG602904 EMBL:AC006266 EMBL:AL161508 EMBL:AK118480 EMBL:AY087066 EMBL:BT008770 IPI:IPI00545815 PIR:B85078 RefSeq:NP_192536.1 UniGene:At.33785 ProteinModelPortal:Q9ZQB9 STRING:Q9ZQB9 PRIDE:Q9ZQB9 EnsemblPlants:AT4G07960.1 GeneID:826301 KEGG:ath:AT4G07960 TAIR:At4g07960 InParanoid:Q9ZQB9 OMA:FEIAAYF PhylomeDB:Q9ZQB9 ProtClustDB:CLSN2685849 ArrayExpress:Q9ZQB9 Genevestigator:Q9ZQB9 Uniprot:Q9ZQB9
Root	Isotig00198	94	187	-1.121	2.42E-10	TAIR|locus:2120532 - symbol:CSLC12 "AT4G07960" species:3702 "Arabidopsis thaliana" [GO:0016757 "transferase activity, transferring glycosyl groups" evidence=ISS] [GO:0016759 "cellulose synthase activity" evidence=ISS] [GO:0009506 "plasmodesma" evidence=IDA] GO:GO:0016021 GO:GO:0009506 EMBL:CP002687 GenomeReviews:CT486007_GR GO:GO:0000139 GO:GO:0007047 GO:GO:0016757 CAZy:GT2 eggNOG:COG1215 HOGENOM:HBG602904 EMBL:AC006266 EMBL:AL161508 EMBL:AK118480 EMBL:AY087066 EMBL:BT008770 IPI:IPI00545815 PIR:B85078 RefSeq:NP_192536.1 UniGene:At.33785 ProteinModelPortal:Q9ZQB9 STRING:Q9ZQB9 PRIDE:Q9ZQB9 EnsemblPlants:AT4G07960.1 GeneID:826301 KEGG:ath:AT4G07960 TAIR:At4g07960 InParanoid:Q9ZQB9 OMA:FEIAAYF PhylomeDB:Q9ZQB9 ProtClustDB:CLSN2685849 ArrayExpress:Q9ZQB9 Genevestigator:Q9ZQB9 Uniprot:Q9ZQB9
Root	Isotig00199	94	187	-1.121	2.42E-10	TAIR|locus:2120532 - symbol:CSLC12 "AT4G07960" species:3702 "Arabidopsis thaliana" [GO:0016757 "transferase activity, transferring glycosyl groups" evidence=ISS] [GO:0016759 "cellulose synthase activity" evidence=ISS] [GO:0009506 "plasmodesma" evidence=IDA] GO:GO:0016021 GO:GO:0009506 EMBL:CP002687 GenomeReviews:CT486007_GR GO:GO:0000139 GO:GO:0007047 GO:GO:0016757 CAZy:GT2 eggNOG:COG1215 HOGENOM:HBG602904 EMBL:AC006266 EMBL:AL161508 EMBL:AK118480 EMBL:AY087066 EMBL:BT008770 IPI:IPI00545815 PIR:B85078 RefSeq:NP_192536.1 UniGene:At.33785 ProteinModelPortal:Q9ZQB9 STRING:Q9ZQB9 PRIDE:Q9ZQB9 EnsemblPlants:AT4G07960.1 GeneID:826301 KEGG:ath:AT4G07960 TAIR:At4g07960 InParanoid:Q9ZQB9 OMA:FEIAAYF PhylomeDB:Q9ZQB9 ProtClustDB:CLSN2685849 ArrayExpress:Q9ZQB9 Genevestigator:Q9ZQB9 Uniprot:Q9ZQB9
Root	Isotig00200	94	187	-1.121	2.42E-10	TAIR|locus:2120532 - symbol:CSLC12 "AT4G07960" species:3702 "Arabidopsis thaliana" [GO:0016757 "transferase activity, transferring glycosyl groups" evidence=ISS] [GO:0016759 "cellulose synthase activity" evidence=ISS] [GO:0009506 "plasmodesma" evidence=IDA] GO:GO:0016021 GO:GO:0009506 EMBL:CP002687 GenomeReviews:CT486007_GR GO:GO:0000139 GO:GO:0007047 GO:GO:0016757 CAZy:GT2 eggNOG:COG1215 HOGENOM:HBG602904 EMBL:AC006266 EMBL:AL161508 EMBL:AK118480 EMBL:AY087066 EMBL:BT008770 IPI:IPI00545815 PIR:B85078 RefSeq:NP_192536.1 UniGene:At.33785 ProteinModelPortal:Q9ZQB9 STRING:Q9ZQB9 PRIDE:Q9ZQB9 EnsemblPlants:AT4G07960.1 GeneID:826301

Tissue	Identification	# of reads (stress)	# of reads (control)	Fold Change	p-value	Description
						KEGG:ath:AT4G07960 TAIR:At4g07960 InParanoid:Q9ZQB9 OMA:FEIAAYF PhylomeDB:Q9ZQB9 ProtClustDB:CLSN2685849 ArrayExpress:Q9ZQB9 Genevestigator:Q9ZQB9 Uniprot:Q9ZQB9
Root	Isotig00201	94	188	-1.129	1.72E-10	TAIR|locus:2120532 - symbol:CSLC12 "AT4G07960" species:3702 "Arabidopsis thaliana" [GO:0016757 "transferase activity, transferring glycosyl groups" evidence=ISS] [GO:0016759 "cellulose synthase activity" evidence=ISS] [GO:0009506 "plasmodesma" evidence=IDA] GO:GO:0016021 GO:GO:0009506 EMBL:CP002687 GenomeReviews:CT486007_GR GO:GO:0000139 GO:GO:0007047 GO:GO:0016757 CAZy:GT2 eggNOG:COG1215 HOGENOM:HBG602904 EMBL:AC006266 EMBL:AL161508 EMBL:AK118480 EMBL:AY087066 EMBL:BT008770 IPI:IPI00545815 PIR:B85078 RefSeq:NP_192536.1 UniGene:At.33785 ProteinModelPortal:Q9ZQB9 STRING:Q9ZQB9 PRIDE:Q9ZQB9 EnsemblPlants:AT4G07960.1 GeneID:826301 KEGG:ath:AT4G07960 TAIR:At4g07960 InParanoid:Q9ZQB9 OMA:FEIAAYF PhylomeDB:Q9ZQB9 ProtClustDB:CLSN2685849 ArrayExpress:Q9ZQB9 Genevestigator:Q9ZQB9 Uniprot:Q9ZQB9
Root	Isotig00202	94	188	-1.129	1.72E-10	TAIR|locus:2120532 - symbol:CSLC12 "AT4G07960" species:3702 "Arabidopsis thaliana" [GO:0016757 "transferase activity, transferring glycosyl groups" evidence=ISS] [GO:0016759 "cellulose synthase activity" evidence=ISS] [GO:0009506 "plasmodesma" evidence=IDA] GO:GO:0016021 GO:GO:0009506 EMBL:CP002687 GenomeReviews:CT486007_GR GO:GO:0000139 GO:GO:0007047 GO:GO:0016757 CAZy:GT2 eggNOG:COG1215 HOGENOM:HBG602904 EMBL:AC006266 EMBL:AL161508 EMBL:AK118480 EMBL:AY087066 EMBL:BT008770 IPI:IPI00545815 PIR:B85078 RefSeq:NP_192536.1 UniGene:At.33785 ProteinModelPortal:Q9ZQB9 STRING:Q9ZQB9 PRIDE:Q9ZQB9 EnsemblPlants:AT4G07960.1 GeneID:826301 KEGG:ath:AT4G07960 TAIR:At4g07960 InParanoid:Q9ZQB9 OMA:FEIAAYF PhylomeDB:Q9ZQB9 ProtClustDB:CLSN2685849 ArrayExpress:Q9ZQB9 Genevestigator:Q9ZQB9 Uniprot:Q9ZQB9
Root	Isotig00203	91	184	-1.145	1.70E-10	TAIR|locus:2120532 - symbol:CSLC12 "AT4G07960" species:3702 "Arabidopsis thaliana" [GO:0016757 "transferase activity, transferring glycosyl groups" evidence=ISS] [GO:0016759 "cellulose synthase activity" evidence=ISS] [GO:0009506 "plasmodesma" evidence=IDA] GO:GO:0016021 GO:GO:0009506 EMBL:CP002687 GenomeReviews:CT486007_GR GO:GO:0000139 GO:GO:0007047 GO:GO:0016757 CAZy:GT2 eggNOG:COG1215 HOGENOM:HBG602904 EMBL:AC006266 EMBL:AL161508 EMBL:AK118480 EMBL:AY087066 EMBL:BT008770 IPI:IPI00545815 PIR:B85078 RefSeq:NP_192536.1 UniGene:At.33785 ProteinModelPortal:Q9ZQB9 STRING:Q9ZQB9 PRIDE:Q9ZQB9 EnsemblPlants:AT4G07960.1 GeneID:826301 KEGG:ath:AT4G07960 TAIR:At4g07960 InParanoid:Q9ZQB9 OMA:FEIAAYF PhylomeDB:Q9ZQB9 ProtClustDB:CLSN2685849 ArrayExpress:Q9ZQB9 Genevestigator:Q9ZQB9 Uniprot:Q9ZQB9
Root	Isotig00204	91	185	-1.153	1.20E-10	TAIR|locus:2120532 - symbol:CSLC12 "AT4G07960" species:3702 "Arabidopsis thaliana" [GO:0016757 "transferase activity, transferring glycosyl groups" evidence=ISS] [GO:0016759 "cellulose synthase activity" evidence=ISS] [GO:0009506 "plasmodesma" evidence=IDA] GO:GO:0016021 GO:GO:0009506 EMBL:CP002687 GenomeReviews:CT486007_GR GO:GO:0000139 GO:GO:0007047 GO:GO:0016757 CAZy:GT2 eggNOG:COG1215 HOGENOM:HBG602904 EMBL:AC006266 EMBL:AL161508 EMBL:AK118480 EMBL:AY087066 EMBL:BT008770 IPI:IPI00545815 PIR:B85078 RefSeq:NP_192536.1 UniGene:At.33785 ProteinModelPortal:Q9ZQB9 STRING:Q9ZQB9 PRIDE:Q9ZQB9 EnsemblPlants:AT4G07960.1 GeneID:826301 KEGG:ath:AT4G07960 TAIR:At4g07960 InParanoid:Q9ZQB9 OMA:FEIAAYF PhylomeDB:Q9ZQB9 ProtClustDB:CLSN2685849 ArrayExpress:Q9ZQB9 Genevestigator:Q9ZQB9 Uniprot:Q9ZQB9
Root	Isotig00205	87	176	-1.145	4.12E-10	TAIR|locus:2120532 - symbol:CSLC12 "AT4G07960" species:3702 "Arabidopsis thaliana" [GO:0016757 "transferase activity, transferring glycosyl groups" evidence=ISS] [GO:0016759 "cellulose synthase activity" evidence=ISS] [GO:0009506 "plasmodesma" evidence=IDA] GO:GO:0016021 GO:GO:0009506 EMBL:CP002687 GenomeReviews:CT486007_GR GO:GO:0000139 GO:GO:0007047 GO:GO:0016757 CAZy:GT2 eggNOG:COG1215 HOGENOM:HBG602904 EMBL:AC006266 EMBL:AL161508 EMBL:AK118480 EMBL:AY087066 EMBL:BT008770 IPI:IPI00545815 PIR:B85078 RefSeq:NP_192536.1 UniGene:At.33785 ProteinModelPortal:Q9ZQB9 STRING:Q9ZQB9 PRIDE:Q9ZQB9 EnsemblPlants:AT4G07960.1 GeneID:826301 KEGG:ath:AT4G07960 TAIR:At4g07960 InParanoid:Q9ZQB9 OMA:FEIAAYF PhylomeDB:Q9ZQB9 ProtClustDB:CLSN2685849 ArrayExpress:Q9ZQB9 Genevestigator:Q9ZQB9 Uniprot:Q9ZQB9
Root	Isotig00206	91	184	-1.145	1.70E-10	TAIR|locus:2120532 - symbol:CSLC12 "AT4G07960" species:3702 "Arabidopsis thaliana" [GO:0016757 "transferase activity, transferring glycosyl groups" evidence=ISS] [GO:0016759 "cellulose synthase activity" evidence=ISS] [GO:0009506 "plasmodesma" evidence=IDA] GO:GO:0016021 GO:GO:0009506 EMBL:CP002687 GenomeReviews:CT486007_GR GO:GO:0000139 GO:GO:0007047 GO:GO:0016757 CAZy:GT2 eggNOG:COG1215 HOGENOM:HBG602904 EMBL:AC006266 EMBL:AL161508 EMBL:AK118480

Tissue	Identification	# of reads (stress)	# of reads (control)	Fold Change	p-value	Description
						EMBL:AY087066 EMBL:BT008770 IPI:IPI00545815 PIR:B85078 RefSeq:NP_192536.1 UniGene:At.33785 ProteinModelPortal:Q9ZQB9 STRING:Q9ZQB9 PRIDE:Q9ZQB9 EnsemblPlants:AT4G07960.1 GeneID:826301 KEGG:ath:AT4G07960 TAIR:At4g07960 InParanoid:Q9ZQB9 OMA:FEIAAYF PhylomeDB:Q9ZQB9 ProtClustDB:CLSN2685849 ArrayExpress:Q9ZQB9 Genevestigator:Q9ZQB9 Uniprot:Q9ZQB9
Root	Isotig00207	91	184	-1.145	1.70E-10	TAIR|locus:2120532 - symbol:CSLC12 "AT4G07960" species:3702 "Arabidopsis thaliana" [GO:0016757 "transferase activity, transferring glycosyl groups" evidence=ISS] [GO:0016759 "cellulose synthase activity" evidence=ISS] [GO:0009506 "plasmodesma" evidence=IDA] GO:GO:0016021 GO:GO:0009506 EMBL:CP002687 GenomeReviews:CT486007_GR GO:GO:0000139 GO:GO:0007047 GO:GO:0016757 CAZy:GT2 eggNOG:COG1215 HOGENOM:HBG602904 EMBL:AC006266 EMBL:AL161508 EMBL:AK118480 EMBL:AY087066 EMBL:BT008770 IPI:IPI00545815 PIR:B85078 RefSeq:NP_192536.1 UniGene:At.33785 ProteinModelPortal:Q9ZQB9 STRING:Q9ZQB9 PRIDE:Q9ZQB9 EnsemblPlants:AT4G07960.1 GeneID:826301 KEGG:ath:AT4G07960 TAIR:At4g07960 InParanoid:Q9ZQB9 OMA:FEIAAYF PhylomeDB:Q9ZQB9 ProtClustDB:CLSN2685849 ArrayExpress:Q9ZQB9 Genevestigator:Q9ZQB9 Uniprot:Q9ZQB9
Root	Isotig00219	21	62	-1.691	6.33E-07	TAIR|locus:2159143 - symbol:AT5G13980 species:3702 "Arabidopsis thaliana" [GO:0003824 "catalytic activity" evidence=IEA] [GO:0004553 "hydrolase activity, hydrolyzing O-glycosyl compounds" evidence=IEA] [GO:0004559 "alpha-mannosidase activity" evidence=IEA;ISS] [GO:0005975 "carbohydrate metabolic process" evidence=IEA] [GO:0006013 "mannose metabolic process" evidence=IEA] [GO:0008270 "zinc ion binding" evidence=IEA] [GO:0015923 "mannosidase activity" evidence=IEA] [GO:0030246 "carbohydrate binding" evidence=IEA] [GO:0005773 "vacuole" evidence=IDA] [GO:0005618 "cell wall" evidence=IDA] [GO:0005774 "vacuolar membrane" evidence=IDA] [GO:0009505 "plant-type cell wall" evidence=IDA] [GO:0048046 "apoplast" evidence=IDA] InterPro:IPR000602 InterPro:IPR011013 InterPro:IPR011330 InterPro:IPR011682 InterPro:IPR013780 InterPro:IPR015341 Pfam:PF01074 Pfam:PF07748 Pfam:PF09261 SMART:SM00872 GO:GO:0048046 GO:GO:0005774 EMBL:CP002688 Gene3D:G3DSA:2.60.40.1180 GO:GO:0008270 GO:GO:0009505 GO:GO:0030246 SUPFAM:SSF74650 SUPFAM:SSF88713 GO:GO:0006013 CAZy:GH38 GO:GO:0004559 Gene3D:G3DSA:3.20.110.10 HSSP:Q29451 OMA:SPVVQDD ProtClustDB:CLSN2684873 EMBL:AY099704 EMBL:BT000301 IPI:IPI00529830 RefSeq:NP_196902.2 RefSeq:NP_851037.2 UniGene:At.19885 ProteinModelPortal:Q8LPJ3 SMR:Q8LPJ3 PRIDE:Q8LPJ3 EnsemblPlants:AT5G13980.1 EnsemblPlants:AT5G13980.2 GeneID:831246 KEGG:ath:AT5G13980 TAIR:At5g13980 InParanoid:Q8LPJ3 PhylomeDB:Q8LPJ3 ArrayExpress:Q8LPJ3 Genevestigator:Q8LPJ3 Uniprot:Q8LPJ3
Root	Isotig00220	21	62	-1.691	6.33E-07	TAIR|locus:2159143 - symbol:AT5G13980 species:3702 "Arabidopsis thaliana" [GO:0003824 "catalytic activity" evidence=IEA] [GO:0004553 "hydrolase activity, hydrolyzing O-glycosyl compounds" evidence=IEA] [GO:0004559 "alpha-mannosidase activity" evidence=IEA;ISS] [GO:0005975 "carbohydrate metabolic process" evidence=IEA] [GO:0006013 "mannose metabolic process" evidence=IEA] [GO:0008270 "zinc ion binding" evidence=IEA] [GO:0015923 "mannosidase activity" evidence=IEA] [GO:0030246 "carbohydrate binding" evidence=IEA] [GO:0005773 "vacuole" evidence=IDA] [GO:0005618 "cell wall" evidence=IDA] [GO:0005774 "vacuolar membrane" evidence=IDA] [GO:0009505 "plant-type cell wall" evidence=IDA] [GO:0048046 "apoplast" evidence=IDA] InterPro:IPR000602 InterPro:IPR011013 InterPro:IPR011330 InterPro:IPR011682 InterPro:IPR013780 InterPro:IPR015341 Pfam:PF01074 Pfam:PF07748 Pfam:PF09261 SMART:SM00872 GO:GO:0048046 GO:GO:0005774 EMBL:CP002688 Gene3D:G3DSA:2.60.40.1180 GO:GO:0008270 GO:GO:0009505 GO:GO:0030246 SUPFAM:SSF74650 SUPFAM:SSF88713 GO:GO:0006013 CAZy:GH38 GO:GO:0004559 Gene3D:G3DSA:3.20.110.10 HSSP:Q29451 OMA:SPVVQDD ProtClustDB:CLSN2684873 EMBL:AY099704 EMBL:BT000301 IPI:IPI00529830 RefSeq:NP_196902.2 RefSeq:NP_851037.2 UniGene:At.19885 ProteinModelPortal:Q8LPJ3 SMR:Q8LPJ3 PRIDE:Q8LPJ3 EnsemblPlants:AT5G13980.1 EnsemblPlants:AT5G13980.2 GeneID:831246 KEGG:ath:AT5G13980 TAIR:At5g13980 InParanoid:Q8LPJ3 PhylomeDB:Q8LPJ3 ArrayExpress:Q8LPJ3 Genevestigator:Q8LPJ3 Uniprot:Q8LPJ3
Root	Isotig00221	21	61	-1.667	9.99E-07	TAIR|locus:2159143 - symbol:AT5G13980 species:3702 "Arabidopsis thaliana" [GO:0003824 "catalytic activity" evidence=IEA] [GO:0004553 "hydrolase activity, hydrolyzing O-glycosyl compounds" evidence=IEA] [GO:0004559 "alpha-mannosidase activity" evidence=IEA;ISS] [GO:0005975 "carbohydrate metabolic process" evidence=IEA] [GO:0006013 "mannose metabolic process" evidence=IEA] [GO:0008270 "zinc ion binding" evidence=IEA] [GO:0015923 "mannosidase activity" evidence=IEA] [GO:0030246 "carbohydrate binding" evidence=IEA] [GO:0005773 "vacuole" evidence=IDA] [GO:0005618 "cell wall" evidence=IDA] [GO:0005774 "vacuolar membrane" evidence=IDA] [GO:0009505 "plant-type cell wall" evidence=IDA] [GO:0048046 "apoplast" evidence=IDA]

Tissue	Identification	# of reads (stress)	# of reads (control)	Fold Change	p-value	Description
						InterPro:IPR000602 InterPro:IPR011013 InterPro:IPR011330 InterPro:IPR011682 InterPro:IPR013780 InterPro:IPR015341 Pfam:PF01074 Pfam:PF07748 Pfam:PF09261 SMART:SM00872 GO:GO:0048046 GO:GO:0005774 EMBL:CP002688 Gene3D:G3DSA:2.60.40.1180 GO:GO:0008270 GO:GO:0009505 GO:GO:0030246 SUPFAM:SSF74650 SUPFAM:SSF88713 GO:GO:0006013 CAZy:GH38 GO:GO:0004559 Gene3D:G3DSA:3.20.110.10 HSSP:Q29451 OMA:SPVVQDD ProtClustDB:CLSN2684873 EMBL:AY099704 EMBL:BT000301 IPI:IPI00529830 RefSeq:NP_196902.2 RefSeq:NP_851037.2 UniGene:At.19885 ProteinModelPortal:Q8LPJ3 SMR:Q8LPJ3 PRIDE:Q8LPJ3 EnsemblPlants:AT5G13980.1 EnsemblPlants:AT5G13980.2 GeneID:831246 KEGG:ath:AT5G13980 TAIR:At5g13980 InParanoid:Q8LPJ3 PhylomeDB:Q8LPJ3 ArrayExpress:Q8LPJ3 Genevestigator:Q8LPJ3 Uniprot:Q8LPJ3
Root	Isotig00222	21	61	-1.667	9.99E-07	TAIR|locus:2159143 - symbol:AT5G13980 species:3702 "Arabidopsis thaliana" [GO:0003824 "catalytic activity" evidence=IEA] [GO:0004553 "hydrolase activity, hydrolyzing O-glycosyl compounds" evidence=IEA] [GO:0004559 "alpha-mannosidase activity" evidence=IEA;ISS] [GO:0005975 "carbohydrate metabolic process" evidence=IEA] [GO:0006013 "mannose metabolic process" evidence=IEA] [GO:0008270 "zinc ion binding" evidence=IEA] [GO:0015923 "mannosidase activity" evidence=IEA] [GO:0030246 "carbohydrate binding" evidence=IEA] [GO:0005773 "vacuole" evidence=IDA] [GO:0005618 "cell wall" evidence=IDA] [GO:0005774 "vacuolar membrane" evidence=IDA] [GO:0009505 "plant-type cell wall" evidence=IDA] [GO:0048046 "apoplast" evidence=IDA] InterPro:IPR000602 InterPro:IPR011013 InterPro:IPR011330 InterPro:IPR011682 InterPro:IPR013780 InterPro:IPR015341 Pfam:PF01074 Pfam:PF07748 Pfam:PF09261 SMART:SM00872 GO:GO:0048046 GO:GO:0005774 EMBL:CP002688 Gene3D:G3DSA:2.60.40.1180 GO:GO:0008270 GO:GO:0009505 GO:GO:0030246 SUPFAM:SSF74650 SUPFAM:SSF88713 GO:GO:0006013 CAZy:GH38 GO:GO:0004559 Gene3D:G3DSA:3.20.110.10 HSSP:Q29451 OMA:SPVVQDD ProtClustDB:CLSN2684873 EMBL:AY099704 EMBL:BT000301 IPI:IPI00529830 RefSeq:NP_196902.2 RefSeq:NP_851037.2 UniGene:At.19885 ProteinModelPortal:Q8LPJ3 SMR:Q8LPJ3 PRIDE:Q8LPJ3 EnsemblPlants:AT5G13980.1 EnsemblPlants:AT5G13980.2 GeneID:831246 KEGG:ath:AT5G13980 TAIR:At5g13980 InParanoid:Q8LPJ3 PhylomeDB:Q8LPJ3 ArrayExpress:Q8LPJ3 Genevestigator:Q8LPJ3 Uniprot:Q8LPJ3
Root	Isotig00223	20	59	-1.690	1.19E-06	TAIR|locus:2159143 - symbol:AT5G13980 species:3702 "Arabidopsis thaliana" [GO:0003824 "catalytic activity" evidence=IEA] [GO:0004553 "hydrolase activity, hydrolyzing O-glycosyl compounds" evidence=IEA] [GO:0004559 "alpha-mannosidase activity" evidence=IEA;ISS] [GO:0005975 "carbohydrate metabolic process" evidence=IEA] [GO:0006013 "mannose metabolic process" evidence=IEA] [GO:0008270 "zinc ion binding" evidence=IEA] [GO:0015923 "mannosidase activity" evidence=IEA] [GO:0030246 "carbohydrate binding" evidence=IEA] [GO:0005773 "vacuole" evidence=IDA] [GO:0005618 "cell wall" evidence=IDA] [GO:0005774 "vacuolar membrane" evidence=IDA] [GO:0009505 "plant-type cell wall" evidence=IDA] [GO:0048046 "apoplast" evidence=IDA] InterPro:IPR000602 InterPro:IPR011013 InterPro:IPR011330 InterPro:IPR011682 InterPro:IPR013780 InterPro:IPR015341 Pfam:PF01074 Pfam:PF07748 Pfam:PF09261 SMART:SM00872 GO:GO:0048046 GO:GO:0005774 EMBL:CP002688 Gene3D:G3DSA:2.60.40.1180 GO:GO:0008270 GO:GO:0009505 GO:GO:0030246 SUPFAM:SSF74650 SUPFAM:SSF88713 GO:GO:0006013 CAZy:GH38 GO:GO:0004559 Gene3D:G3DSA:3.20.110.10 HSSP:Q29451 OMA:SPVVQDD ProtClustDB:CLSN2684873 EMBL:AY099704 EMBL:BT000301 IPI:IPI00529830 RefSeq:NP_196902.2 RefSeq:NP_851037.2 UniGene:At.19885 ProteinModelPortal:Q8LPJ3 SMR:Q8LPJ3 PRIDE:Q8LPJ3 EnsemblPlants:AT5G13980.1 EnsemblPlants:AT5G13980.2 GeneID:831246 KEGG:ath:AT5G13980 TAIR:At5g13980 InParanoid:Q8LPJ3 PhylomeDB:Q8LPJ3 ArrayExpress:Q8LPJ3 Genevestigator:Q8LPJ3 Uniprot:Q8LPJ3
Root	Isotig00224	20	59	-1.690	1.19E-06	TAIR|locus:2159143 - symbol:AT5G13980 species:3702 "Arabidopsis thaliana" [GO:0003824 "catalytic activity" evidence=IEA] [GO:0004553 "hydrolase activity, hydrolyzing O-glycosyl compounds" evidence=IEA] [GO:0004559 "alpha-mannosidase activity" evidence=IEA;ISS] [GO:0005975 "carbohydrate metabolic process" evidence=IEA] [GO:0006013 "mannose metabolic process" evidence=IEA] [GO:0008270 "zinc ion binding" evidence=IEA] [GO:0015923 "mannosidase activity" evidence=IEA] [GO:0030246 "carbohydrate binding" evidence=IEA] [GO:0005773 "vacuole" evidence=IDA] [GO:0005618 "cell wall" evidence=IDA] [GO:0005774 "vacuolar membrane" evidence=IDA] [GO:0009505 "plant-type cell wall" evidence=IDA] [GO:0048046 "apoplast" evidence=IDA] InterPro:IPR000602 InterPro:IPR011013 InterPro:IPR011330 InterPro:IPR011682 InterPro:IPR013780 InterPro:IPR015341 Pfam:PF01074 Pfam:PF07748 Pfam:PF09261 SMART:SM00872 GO:GO:0048046 GO:GO:0005774 EMBL:CP002688 Gene3D:G3DSA:2.60.40.1180 GO:GO:0008270 GO:GO:0009505 GO:GO:0030246 SUPFAM:SSF74650 SUPFAM:SSF88713 GO:GO:0006013 CAZy:GH38 GO:GO:0004559

Tissue	Identification	# of reads (stress)	# of reads (control)	Fold Change	p-value	Description
						Gene3D:G3DSA:3.20.110.10 HSSP:Q29451 OMA:SPVVQDD ProtClustDB:CLSN2684873 EMBL:AY099704 EMBL:BT000301 IPI:IPI00529830 RefSeq:NP_196902.2 RefSeq:NP_851037.2 UniGene:At.19885 ProteinModelPortal:Q8LPJ3 SMR:Q8LPJ3 PRIDE:Q8LPJ3 EnsemblPlants:AT5G13980.1 EnsemblPlants:AT5G13980.2 GeneID:831246 KEGG:ath:AT5G13980 TAIR:At5g13980 InParanoid:Q8LPJ3 PhylomeDB:Q8LPJ3 ArrayExpress:Q8LPJ3 Genevestigator:Q8LPJ3 Uniprot:Q8LPJ3
Root	Isotig00225	20	59	-1.690	1.19E-06	TAIR|locus:2159143 - symbol:AT5G13980 species:3702 "Arabidopsis thaliana" [GO:0003824 "catalytic activity" evidence=IEA] [GO:0004553 "hydrolase activity, hydrolyzing O-glycosyl compounds" evidence=IEA] [GO:0004559 "alpha-mannosidase activity" evidence=IEA;ISS] [GO:0005975 "carbohydrate metabolic process" evidence=IEA] [GO:0006013 "mannose metabolic process" evidence=IEA] [GO:0008270 "zinc ion binding" evidence=IEA] [GO:0015923 "mannosidase activity" evidence=IEA] [GO:0030246 "carbohydrate binding" evidence=IEA] [GO:0005773 "vacuole" evidence=IDA] [GO:0005618 "cell wall" evidence=IDA] [GO:0005774 "vacuolar membrane" evidence=IDA] [GO:0009505 "plant-type cell wall" evidence=IDA] [GO:0048046 "apoplast" evidence=IDA] InterPro:IPR000602 InterPro:IPR011013 InterPro:IPR011330 InterPro:IPR011682 InterPro:IPR013780 InterPro:IPR015341 Pfam:PF01074 Pfam:PF07748 Pfam:PF09261 SMART:SM00872 GO:GO:0048046 GO:GO:0005774 EMBL:CP002688 Gene3D:G3DSA:2.60.40.1180 GO:GO:0008270 GO:GO:0009505 GO:GO:0030246 SUPFAM:SSF74650 SUPFAM:SSF88713 GO:GO:0006013 CAZy:GH38 GO:GO:0004559 Gene3D:G3DSA:3.20.110.10 HSSP:Q29451 OMA:SPVVQDD ProtClustDB:CLSN2684873 EMBL:AY099704 EMBL:BT000301 IPI:IPI00529830 RefSeq:NP_196902.2 RefSeq:NP_851037.2 UniGene:At.19885 ProteinModelPortal:Q8LPJ3 SMR:Q8LPJ3 PRIDE:Q8LPJ3 EnsemblPlants:AT5G13980.1 EnsemblPlants:AT5G13980.2 GeneID:831246 KEGG:ath:AT5G13980 TAIR:At5g13980 InParanoid:Q8LPJ3 PhylomeDB:Q8LPJ3 ArrayExpress:Q8LPJ3 Genevestigator:Q8LPJ3 Uniprot:Q8LPJ3
Root	Isotig00226	20	59	-1.690	1.19E-06	TAIR|locus:2159143 - symbol:AT5G13980 species:3702 "Arabidopsis thaliana" [GO:0003824 "catalytic activity" evidence=IEA] [GO:0004553 "hydrolase activity, hydrolyzing O-glycosyl compounds" evidence=IEA] [GO:0004559 "alpha-mannosidase activity" evidence=IEA;ISS] [GO:0005975 "carbohydrate metabolic process" evidence=IEA] [GO:0006013 "mannose metabolic process" evidence=IEA] [GO:0008270 "zinc ion binding" evidence=IEA] [GO:0015923 "mannosidase activity" evidence=IEA] [GO:0030246 "carbohydrate binding" evidence=IEA] [GO:0005773 "vacuole" evidence=IDA] [GO:0005618 "cell wall" evidence=IDA] [GO:0005774 "vacuolar membrane" evidence=IDA] [GO:0009505 "plant-type cell wall" evidence=IDA] [GO:0048046 "apoplast" evidence=IDA] InterPro:IPR000602 InterPro:IPR011013 InterPro:IPR011330 InterPro:IPR011682 InterPro:IPR013780 InterPro:IPR015341 Pfam:PF01074 Pfam:PF07748 Pfam:PF09261 SMART:SM00872 GO:GO:0048046 GO:GO:0005774 EMBL:CP002688 Gene3D:G3DSA:2.60.40.1180 GO:GO:0008270 GO:GO:0009505 GO:GO:0030246 SUPFAM:SSF74650 SUPFAM:SSF88713 GO:GO:0006013 CAZy:GH38 GO:GO:0004559 Gene3D:G3DSA:3.20.110.10 HSSP:Q29451 OMA:SPVVQDD ProtClustDB:CLSN2684873 EMBL:AY099704 EMBL:BT000301 IPI:IPI00529830 RefSeq:NP_196902.2 RefSeq:NP_851037.2 UniGene:At.19885 ProteinModelPortal:Q8LPJ3 SMR:Q8LPJ3 PRIDE:Q8LPJ3 EnsemblPlants:AT5G13980.1 EnsemblPlants:AT5G13980.2 GeneID:831246 KEGG:ath:AT5G13980 TAIR:At5g13980 InParanoid:Q8LPJ3 PhylomeDB:Q8LPJ3 ArrayExpress:Q8LPJ3 Genevestigator:Q8LPJ3 Uniprot:Q8LPJ3
Root	Isotig00227	12	48	-2.129	3.14E-07	TAIR|locus:2159143 - symbol:AT5G13980 species:3702 "Arabidopsis thaliana" [GO:0003824 "catalytic activity" evidence=IEA] [GO:0004553 "hydrolase activity, hydrolyzing O-glycosyl compounds" evidence=IEA] [GO:0004559 "alpha-mannosidase activity" evidence=IEA;ISS] [GO:0005975 "carbohydrate metabolic process" evidence=IEA] [GO:0006013 "mannose metabolic process" evidence=IEA] [GO:0008270 "zinc ion binding" evidence=IEA] [GO:0015923 "mannosidase activity" evidence=IEA] [GO:0030246 "carbohydrate binding" evidence=IEA] [GO:0005773 "vacuole" evidence=IDA] [GO:0005618 "cell wall" evidence=IDA] [GO:0005774 "vacuolar membrane" evidence=IDA] [GO:0009505 "plant-type cell wall" evidence=IDA] [GO:0048046 "apoplast" evidence=IDA] InterPro:IPR000602 InterPro:IPR011013 InterPro:IPR011330 InterPro:IPR011682 InterPro:IPR013780 InterPro:IPR015341 Pfam:PF01074 Pfam:PF07748 Pfam:PF09261 SMART:SM00872 GO:GO:0048046 GO:GO:0005774 EMBL:CP002688 Gene3D:G3DSA:2.60.40.1180 GO:GO:0008270 GO:GO:0009505 GO:GO:0030246 SUPFAM:SSF74650 SUPFAM:SSF88713 GO:GO:0006013 CAZy:GH38 GO:GO:0004559 Gene3D:G3DSA:3.20.110.10 HSSP:Q29451 OMA:SPVVQDD ProtClustDB:CLSN2684873 EMBL:AY099704 EMBL:BT000301 IPI:IPI00529830 RefSeq:NP_196902.2 RefSeq:NP_851037.2 UniGene:At.19885 ProteinModelPortal:Q8LPJ3 SMR:Q8LPJ3 PRIDE:Q8LPJ3 EnsemblPlants:AT5G13980.1

Tissue	Identification	# of reads (stress)	# of reads (control)	Fold Change	p-value	Description
						EnsemblPlants:AT5G13980.2 GeneID:831246 KEGG:ath:AT5G13980 TAIR:At5g13980 InParanoid:Q8LPJ3 PhylomeDB:Q8LPJ3 ArrayExpress:Q8LPJ3 Genevestigator:Q8LPJ3 Uniprot:Q8LPJ3
Root	Isotig00228	12	48	-2.129	3.14E-07	TAIR|locus:2159143 - symbol:AT5G13980 species:3702 "Arabidopsis thaliana" [GO:0003824 "catalytic activity" evidence=IEA] [GO:0004553 "hydrolase activity, hydrolyzing O-glycosyl compounds" evidence=IEA] [GO:0004559 "alpha-mannosidase activity" evidence=IEA;ISS] [GO:0005975 "carbohydrate metabolic process" evidence=IEA] [GO:0006013 "mannose metabolic process" evidence=IEA] [GO:0008270 "zinc ion binding" evidence=IEA] [GO:0015923 "mannosidase activity" evidence=IEA] [GO:0030246 "carbohydrate binding" evidence=IEA] [GO:0005773 "vacuole" evidence=IDA] [GO:0005618 "cell wall" evidence=IDA] [GO:0005774 "vacuolar membrane" evidence=IDA] [GO:0009505 "plant-type cell wall" evidence=IDA] [GO:0048046 "apoplast" evidence=IDA] InterPro:IPR000602 InterPro:IPR011013 InterPro:IPR011330 InterPro:IPR011682 InterPro:IPR013780 InterPro:IPR015341 Pfam:PF01074 Pfam:PF07748 Pfam:PF09261 SMART:SM00872 GO:GO:0048046 GO:GO:0005774 EMBL:CP002688 Gene3D:G3DSA:2.60.40.1180 GO:GO:0008270 GO:GO:0009505 GO:GO:0030246 SUPFAM:SSF74650 SUPFAM:SSF88713 GO:GO:0006013 CAZy:GH38 GO:GO:0004559 Gene3D:G3DSA:3.20.110.10 HSSP:Q29451 OMA:SPVVQDD ProtClustDB:CLSN2684873 EMBL:AY099704 EMBL:BT000301 IPI:IPI00529830 RefSeq:NP_196902.2 RefSeq:NP_851037.2 UniGene:At.19885 ProteinModelPortal:Q8LPJ3 SMR:Q8LPJ3 PRIDE:Q8LPJ3 EnsemblPlants:AT5G13980.1 EnsemblPlants:AT5G13980.2 GeneID:831246 KEGG:ath:AT5G13980 TAIR:At5g13980 InParanoid:Q8LPJ3 PhylomeDB:Q8LPJ3 ArrayExpress:Q8LPJ3 Genevestigator:Q8LPJ3 Uniprot:Q8LPJ3
Root	Isotig00229	1	34	-5.216	6.54E-10	TAIR|locus:2090817 - symbol:AT3G26720 species:3702 "Arabidopsis thaliana" [GO:0003824 "catalytic activity" evidence=IEA] [GO:0004553 "hydrolase activity, hydrolyzing O-glycosyl compounds" evidence=IEA] [GO:0004559 "alpha-mannosidase activity" evidence=IEA;ISS] [GO:0005975 "carbohydrate metabolic process" evidence=IEA] [GO:0006013 "mannose metabolic process" evidence=IEA] [GO:0008270 "zinc ion binding" evidence=IEA] [GO:0015923 "mannosidase activity" evidence=IEA] [GO:0030246 "carbohydrate binding" evidence=IEA] [GO:0005773 "vacuole" evidence=IDA] [GO:0005774 "vacuolar membrane" evidence=IDA] [GO:0009505 "plant-type cell wall" evidence=IDA] [GO:0005829 "cytosol" evidence=RCA] InterPro:IPR000602 InterPro:IPR011013 InterPro:IPR011330 InterPro:IPR011682 InterPro:IPR013780 InterPro:IPR015341 Pfam:PF01074 Pfam:PF07748 Pfam:PF09261 SMART:SM00872 GO:GO:0005774 EMBL:CP002686 Gene3D:G3DSA:2.60.40.1180 GO:GO:0008270 GO:GO:0009505 GO:GO:0030246 SUPFAM:SSF74650 SUPFAM:SSF88713 EMBL:X98130 EMBL:AB026648 GO:GO:0006013 CAZy:GH38 GO:GO:0004559 Gene3D:G3DSA:3.20.110.10 HOGENOM:HBG718615 OMA:DSAPLNE KO:K01191 HSSP:Q29451 EMBL:AY039536 EMBL:AY113006 EMBL:Y11767 IPI:IPI00531647 RefSeq:NP_189306.1 UniGene:At.21651 ProteinModelPortal:P94078 SMR:P94078 PRIDE:P94078 EnsemblPlants:AT3G26720.1 GeneID:822284 KEGG:ath:AT3G26720 TAIR:At3g26720 InParanoid:P94078 PhylomeDB:P94078 ProtClustDB:CLSN2684873 Genevestigator:P94078 Uniprot:P94078
Root	Isotig00230	1	34	-5.216	6.54E-10	TAIR|locus:2090817 - symbol:AT3G26720 species:3702 "Arabidopsis thaliana" [GO:0003824 "catalytic activity" evidence=IEA] [GO:0004553 "hydrolase activity, hydrolyzing O-glycosyl compounds" evidence=IEA] [GO:0004559 "alpha-mannosidase activity" evidence=IEA;ISS] [GO:0005975 "carbohydrate metabolic process" evidence=IEA] [GO:0006013 "mannose metabolic process" evidence=IEA] [GO:0008270 "zinc ion binding" evidence=IEA] [GO:0015923 "mannosidase activity" evidence=IEA] [GO:0030246 "carbohydrate binding" evidence=IEA] [GO:0005773 "vacuole" evidence=IDA] [GO:0005774 "vacuolar membrane" evidence=IDA] [GO:0009505 "plant-type cell wall" evidence=IDA] [GO:0005829 "cytosol" evidence=RCA] InterPro:IPR000602 InterPro:IPR011013 InterPro:IPR011330 InterPro:IPR011682 InterPro:IPR013780 InterPro:IPR015341 Pfam:PF01074 Pfam:PF07748 Pfam:PF09261 SMART:SM00872 GO:GO:0005774 EMBL:CP002686 Gene3D:G3DSA:2.60.40.1180 GO:GO:0008270 GO:GO:0009505 GO:GO:0030246 SUPFAM:SSF74650 SUPFAM:SSF88713 EMBL:X98130 EMBL:AB026648 GO:GO:0006013 CAZy:GH38 GO:GO:0004559 Gene3D:G3DSA:3.20.110.10 HOGENOM:HBG718615 OMA:DSAPLNE KO:K01191 HSSP:Q29451 EMBL:AY039536 EMBL:AY113006 EMBL:Y11767 IPI:IPI00531647 RefSeq:NP_189306.1 UniGene:At.21651 ProteinModelPortal:P94078 SMR:P94078 PRIDE:P94078 EnsemblPlants:AT3G26720.1 GeneID:822284 KEGG:ath:AT3G26720 TAIR:At3g26720 InParanoid:P94078 PhylomeDB:P94078 ProtClustDB:CLSN2684873 Genevestigator:P94078 Uniprot:P94078
Root	Isotig00312	95	137	-0.657	0.000576456	ZFIN|ZDB-GENE-030131-8278 - symbol:eef1a1a "eukaryotic translation elongation factor 1 alpha 1a" species:7955 "Danio rerio" [GO:0003924 "GTPase activity" evidence=IEA] [GO:0005525 "GTP binding" evidence=IEA] [GO:0006414 "translational elongation" evidence=IEA] [GO:0003746 "translation elongation factor activity" evidence=IEA] [GO:0005737 "cytoplasm" evidence=IEA] [GO:0000166 "nucleotide binding" evidence=IEA]

Tissue	Identification	# of reads (stress)	# of reads (control)	Fold Change	p-value	Description
						InterPro:IPR000795 InterPro:IPR004160 InterPro:IPR004161 InterPro:IPR004539 Pfam:PF00009 Pfam:PF03143 Pfam:PF03144 PRINTS:PR00315 PROSITE:PS00301 ZFIN:ZDB-GENE-030131-8278 GO:GO:0005525 GO:GO:0005737 GO:GO:0003924 GO:GO:0003746 GeneTree:ENSGT00570000078778 InterPro:IPR009001 InterPro:IPR009000 SUPFAM:SSF50465 SUPFAM:SSF50447 HOGENOM:HBG307581 HOVERGEN:HBG000179 KO:K03231 OMA:SIETHHM OrthoDB:EOG40K7ZP TIGRFAMs:TIGR00483 HSSP:P02994 HAMAP:MF_00118_A EMBL:BX323884 EMBL:BC060907 IPI:IPI00485902 RefSeq:NP_956303.1 UniGene:Dr.159839 UniGene:Dr.80241 ProteinModelPortal:Q6P969 SMR:Q6P969 STRING:Q6P969 PRIDE:Q6P969 Ensembl:ENSDART00000128726 GeneID:336334 KEGG:dre:336334 CTD:336334 InParanoid:Q6P969 PhylomeDB:Q6P969 ArrayExpress:Q6P969 Bgee:Q6P969 Uniprot:Q6P969
Root	Isotig00313	94	144	-0.744	8.23E-05	TAIR|locus:2175118 - symbol:AT5G60390 "AT5G60390" species:3702 "Arabidopsis thaliana" [GO:0003746 "translation elongation factor activity" evidence=ISS] [GO:0006414 "translational elongation" evidence=IEA] [GO:0005516 "calmodulin binding" evidence=ISS] [GO:0005886 "plasma membrane" evidence=IDA] [GO:0005739 "mitochondrion" evidence=IDA] [GO:0005773 "vacuole" evidence=IDA] [GO:0005634 "nucleus" evidence=IDA] [GO:0005737 "cytoplasm" evidence=IDA] [GO:0005829 "cytosol" evidence=RCA] [GO:0009506 "plasmodesma" evidence=IDA] [GO:0005794 "Golgi apparatus" evidence=RCA] PROSITE:PS00301 EMBL:CP002684 EMBL:U63815 EMBL:AC026875 EMBL:X16430 EMBL:AY039583 EMBL:BT000595 IPI:IPI00548618 PIR:S06724 UniGene:At.69737 UniGene:At.74830 EnsemblPlants:AT1G07940.1 EnsemblPlants:AT1G07940.2 TAIR:At1g07940 OMA:FLKAGDA GermOnline:AT1G07940 Uniprot:P0DH99
Root	Isotig00321	186	13	3.710	2.83E-37	TAIR|locus:2163153 - symbol:NAC100 "AT5G61430" species:3702 "Arabidopsis thaliana" [GO:0005575 "cellular_component" evidence=ND] [GO:0006355 "regulation of transcription, DNA-dependent" evidence=IEA] [GO:0007275 "multicellular organismal development" evidence=ISS] [GO:0003700 "sequence-specific DNA binding transcription factor activity" evidence=ISS] InterPro:IPR003441 Pfam:PF02365 PROSITE:PS51005 GO:GO:0005634 EMBL:CP002688 GenomeReviews:BA000015_GR GO:GO:0006355 GO:GO:0003677 GO:GO:0006351 EMBL:AB010073 HSSP:Q9C932 SUPFAM:SSF101941 EMBL:AY054644 EMBL:AY081496 EMBL:AY085102 IPI:IPI00529416 RefSeq:NP_200951.1 UniGene:At.7461 ProteinModelPortal:Q9FLJ2 SMR:Q9FLJ2 PRIDE:Q9FLJ2 DNASU:836264 EnsemblPlants:AT5G61430.1 GeneID:836264 KEGG:ath:AT5G61430 TAIR:At5g61430 eggNOG:NOG255211 InParanoid:Q9FLJ2 OMA:HAMIENN PhylomeDB:Q9FLJ2 ProtClustDB:CLSN2686918 Genevestigator:Q9FLJ2 Uniprot:Q9FLJ2
Root	Isotig00322	185	13	3.702	4.97E-37	TAIR|locus:2163153 - symbol:NAC100 "AT5G61430" species:3702 "Arabidopsis thaliana" [GO:0005575 "cellular_component" evidence=ND] [GO:0006355 "regulation of transcription, DNA-dependent" evidence=IEA] [GO:0007275 "multicellular organismal development" evidence=ISS] [GO:0003700 "sequence-specific DNA binding transcription factor activity" evidence=ISS] InterPro:IPR003441 Pfam:PF02365 PROSITE:PS51005 GO:GO:0005634 EMBL:CP002688 GenomeReviews:BA000015_GR GO:GO:0006355 GO:GO:0003677 GO:GO:0006351 EMBL:AB010073 HSSP:Q9C932 SUPFAM:SSF101941 EMBL:AY054644 EMBL:AY081496 EMBL:AY085102 IPI:IPI00529416 RefSeq:NP_200951.1 UniGene:At.7461 ProteinModelPortal:Q9FLJ2 SMR:Q9FLJ2 PRIDE:Q9FLJ2 DNASU:836264 EnsemblPlants:AT5G61430.1 GeneID:836264 KEGG:ath:AT5G61430 TAIR:At5g61430 eggNOG:NOG255211 InParanoid:Q9FLJ2 OMA:HAMIENN PhylomeDB:Q9FLJ2 ProtClustDB:CLSN2686918 Genevestigator:Q9FLJ2 Uniprot:Q9FLJ2
Root	Isotig00323	186	13	3.710	2.83E-37	TAIR|locus:2163153 - symbol:NAC100 "AT5G61430" species:3702 "Arabidopsis thaliana" [GO:0005575 "cellular_component" evidence=ND] [GO:0006355 "regulation of transcription, DNA-dependent" evidence=IEA] [GO:0007275 "multicellular organismal development" evidence=ISS] [GO:0003700 "sequence-specific DNA binding transcription factor activity" evidence=ISS] InterPro:IPR003441 Pfam:PF02365 PROSITE:PS51005 GO:GO:0005634 EMBL:CP002688 GenomeReviews:BA000015_GR GO:GO:0006355 GO:GO:0003677 GO:GO:0006351 EMBL:AB010073 HSSP:Q9C932 SUPFAM:SSF101941 EMBL:AY054644 EMBL:AY081496 EMBL:AY085102 IPI:IPI00529416 RefSeq:NP_200951.1 UniGene:At.7461 ProteinModelPortal:Q9FLJ2 SMR:Q9FLJ2 PRIDE:Q9FLJ2 DNASU:836264 EnsemblPlants:AT5G61430.1 GeneID:836264 KEGG:ath:AT5G61430 TAIR:At5g61430 eggNOG:NOG255211 InParanoid:Q9FLJ2 OMA:HAMIENN PhylomeDB:Q9FLJ2 ProtClustDB:CLSN2686918 Genevestigator:Q9FLJ2 Uniprot:Q9FLJ2
Root	Isotig00324	185	13	3.702	4.97E-37	TAIR|locus:2163153 - symbol:NAC100 "AT5G61430" species:3702 "Arabidopsis thaliana" [GO:0005575 "cellular_component" evidence=ND] [GO:0006355 "regulation of transcription, DNA-dependent" evidence=IEA] [GO:0007275 "multicellular organismal development" evidence=ISS] [GO:0003700 "sequence-specific DNA binding transcription factor activity" evidence=ISS] InterPro:IPR003441 Pfam:PF02365 PROSITE:PS51005

Tissue	Identification	# of reads (stress)	# of reads (control)	Fold Change	p-value	Description
						GO:GO:0005634 EMBL:CP002688 GenomeReviews:BA000015_GR GO:GO:0006355 GO:GO:0003677 GO:GO:0006351 EMBL:AB010073 HSSP:Q9C932 SUPFAM:SSF101941 EMBL:AY054644 EMBL:AY081496 EMBL:AY085102 IPI:IPI00529416 RefSeq:NP_200951.1 UniGene:At.7461 ProteinModelPortal:Q9FLJ2 SMR:Q9FLJ2 PRIDE:Q9FLJ2 DNASU:836264 EnsemblPlants:AT5G61430.1 GeneID:836264 KEGG:ath:AT5G61430 TAIR:At5g61430 eggNOG:NOG255211 InParanoid:Q9FLJ2 OMA:HAMIENN PhylomeDB:Q9FLJ2 ProtClustDB:CLSN2686918 Genevestigator:Q9FLJ2 Uniprot:Q9FLJ2
Root	Isotig00325	176	12	3.745	1.37E-35	TAIR|locus:2163153 - symbol:NAC100 "AT5G61430" species:3702 "Arabidopsis thaliana" [GO:0005575 "cellular_component" evidence=ND] [GO:0006355 "regulation of transcription, DNA-dependent" evidence=IEA] [GO:0007275 "multicellular organismal development" evidence=ISS] [GO:0003700 "sequence-specific DNA binding transcription factor activity" evidence=ISS] InterPro:IPR003441 Pfam:PF02365 PROSITE:PS51005 GO:GO:0005634 EMBL:CP002688 GenomeReviews:BA000015_GR GO:GO:0006355 GO:GO:0003677 GO:GO:0006351 EMBL:AB010073 HSSP:Q9C932 SUPFAM:SSF101941 EMBL:AY054644 EMBL:AY081496 EMBL:AY085102 IPI:IPI00529416 RefSeq:NP_200951.1 UniGene:At.7461 ProteinModelPortal:Q9FLJ2 SMR:Q9FLJ2 PRIDE:Q9FLJ2 DNASU:836264 EnsemblPlants:AT5G61430.1 GeneID:836264 KEGG:ath:AT5G61430 TAIR:At5g61430 eggNOG:NOG255211 InParanoid:Q9FLJ2 OMA:HAMIENN PhylomeDB:Q9FLJ2 ProtClustDB:CLSN2686918 Genevestigator:Q9FLJ2 Uniprot:Q9FLJ2
Root	Isotig00326	176	12	3.745	1.37E-35	TAIR|locus:2163153 - symbol:NAC100 "AT5G61430" species:3702 "Arabidopsis thaliana" [GO:0005575 "cellular_component" evidence=ND] [GO:0006355 "regulation of transcription, DNA-dependent" evidence=IEA] [GO:0007275 "multicellular organismal development" evidence=ISS] [GO:0003700 "sequence-specific DNA binding transcription factor activity" evidence=ISS] InterPro:IPR003441 Pfam:PF02365 PROSITE:PS51005 GO:GO:0005634 EMBL:CP002688 GenomeReviews:BA000015_GR GO:GO:0006355 GO:GO:0003677 GO:GO:0006351 EMBL:AB010073 HSSP:Q9C932 SUPFAM:SSF101941 EMBL:AY054644 EMBL:AY081496 EMBL:AY085102 IPI:IPI00529416 RefSeq:NP_200951.1 UniGene:At.7461 ProteinModelPortal:Q9FLJ2 SMR:Q9FLJ2 PRIDE:Q9FLJ2 DNASU:836264 EnsemblPlants:AT5G61430.1 GeneID:836264 KEGG:ath:AT5G61430 TAIR:At5g61430 eggNOG:NOG255211 InParanoid:Q9FLJ2 OMA:HAMIENN PhylomeDB:Q9FLJ2 ProtClustDB:CLSN2686918 Genevestigator:Q9FLJ2 Uniprot:Q9FLJ2
Root	Isotig00327	186	8	4.410	3.90E-41	TAIR|locus:2163153 - symbol:NAC100 "AT5G61430" species:3702 "Arabidopsis thaliana" [GO:0005575 "cellular_component" evidence=ND] [GO:0006355 "regulation of transcription, DNA-dependent" evidence=IEA] [GO:0007275 "multicellular organismal development" evidence=ISS] [GO:0003700 "sequence-specific DNA binding transcription factor activity" evidence=ISS] InterPro:IPR003441 Pfam:PF02365 PROSITE:PS51005 GO:GO:0005634 EMBL:CP002688 GenomeReviews:BA000015_GR GO:GO:0006355 GO:GO:0003677 GO:GO:0006351 EMBL:AB010073 HSSP:Q9C932 SUPFAM:SSF101941 EMBL:AY054644 EMBL:AY081496 EMBL:AY085102 IPI:IPI00529416 RefSeq:NP_200951.1 UniGene:At.7461 ProteinModelPortal:Q9FLJ2 SMR:Q9FLJ2 PRIDE:Q9FLJ2 DNASU:836264 EnsemblPlants:AT5G61430.1 GeneID:836264 KEGG:ath:AT5G61430 TAIR:At5g61430 eggNOG:NOG255211 InParanoid:Q9FLJ2 OMA:HAMIENN PhylomeDB:Q9FLJ2 ProtClustDB:CLSN2686918 Genevestigator:Q9FLJ2 Uniprot:Q9FLJ2
Root	Isotig00328	185	8	4.402	6.85E-41	TAIR|locus:2163153 - symbol:NAC100 "AT5G61430" species:3702 "Arabidopsis thaliana" [GO:0005575 "cellular_component" evidence=ND] [GO:0006355 "regulation of transcription, DNA-dependent" evidence=IEA] [GO:0007275 "multicellular organismal development" evidence=ISS] [GO:0003700 "sequence-specific DNA binding transcription factor activity" evidence=ISS] InterPro:IPR003441 Pfam:PF02365 PROSITE:PS51005 GO:GO:0005634 EMBL:CP002688 GenomeReviews:BA000015_GR GO:GO:0006355 GO:GO:0003677 GO:GO:0006351 EMBL:AB010073 HSSP:Q9C932 SUPFAM:SSF101941 EMBL:AY054644 EMBL:AY081496 EMBL:AY085102 IPI:IPI00529416 RefSeq:NP_200951.1 UniGene:At.7461 ProteinModelPortal:Q9FLJ2 SMR:Q9FLJ2 PRIDE:Q9FLJ2 DNASU:836264 EnsemblPlants:AT5G61430.1 GeneID:836264 KEGG:ath:AT5G61430 TAIR:At5g61430 eggNOG:NOG255211 InParanoid:Q9FLJ2 OMA:HAMIENN PhylomeDB:Q9FLJ2 ProtClustDB:CLSN2686918 Genevestigator:Q9FLJ2 Uniprot:Q9FLJ2
Root	Isotig00329	176	7	4.523	1.91E-39	TAIR|locus:2163153 - symbol:NAC100 "AT5G61430" species:3702 "Arabidopsis thaliana" [GO:0005575 "cellular_component" evidence=ND] [GO:0006355 "regulation of transcription, DNA-dependent" evidence=IEA] [GO:0007275 "multicellular organismal development" evidence=ISS] [GO:0003700 "sequence-specific DNA binding transcription factor activity" evidence=ISS] InterPro:IPR003441 Pfam:PF02365 PROSITE:PS51005 GO:GO:0005634 EMBL:CP002688 GenomeReviews:BA000015_GR GO:GO:0006355 GO:GO:0003677 GO:GO:0006351 EMBL:AB010073 HSSP:Q9C932 SUPFAM:SSF101941 EMBL:AY054644 EMBL:AY081496

Tissue	Identification	# of reads (stress)	# of reads (control)	Fold Change	p-value	Description
						EMBL:AY085102 IPI:IPI00529416 RefSeq:NP_200951.1 UniGene:At.7461 ProteinModelPortal:Q9FLJ2 SMR:Q9FLJ2 PRIDE:Q9FLJ2 DNASU:836264 EnsemblPlants:AT5G61430.1 GeneID:836264 KEGG:ath:AT5G61430 TAIR:At5g61430 eggNOG:NOG255211 InParanoid:Q9FLJ2 OMA:HAMIENN PhylomeDB:Q9FLJ2 ProtClustDB:CLSN2686918 Genevestigator:Q9FLJ2 Uniprot:Q9FLJ2
Root	Isotig00330	187	2	6.418	1.45E-43	TAIR|locus:2034640 - symbol:ARK3 "armadillo repeat kinesin 3" species:3702 "Arabidopsis thaliana" [GO:0003777 "microtubule motor activity" evidence=IEA;ISS] [GO:0005524 "ATP binding" evidence=IEA] [GO:0007018 "microtubule-based movement" evidence=IEA] [GO:0009574 "preprophase band" evidence=IDA] InterPro:IPR000225 InterPro:IPR001752 InterPro:IPR011989 InterPro:IPR016024 InterPro:IPR019821 Pfam:PF00225 Pfam:PF00514 PRINTS:PR00380 PROSITE:PS00411 PROSITE:PS50067 PROSITE:PS50176 SMART:SM00129 SMART:SM00185 EMBL:CP002684 GenomeReviews:CT485782_GR GO:GO:0005524 SUPFAM:SSF48371 Gene3D:G3DSA:1.25.10.10 eggNOG:COG5059 GO:GO:0005874 GO:GO:0003777 HSSP:P17119 HOGENOM:HBG319705 GO:GO:0007018 Gene3D:G3DSA:3.40.850.10 EMBL:AB290930 EMBL:AF159052 EMBL:AC025416 IPI:IPI00543027 RefSeq:NP_563908.1 UniGene:At.16100 ProteinModelPortal:Q9FZ06 SMR:Q9FZ06 PRIDE:Q9FZ06 EnsemblPlants:AT1G12430.1 GeneID:837799 KEGG:ath:AT1G12430 GeneFarm:2953 TAIR:At1g12430 InParanoid:Q9FZ06 OMA:HAILMVN PhylomeDB:Q9FZ06 Genevestigator:Q9FZ06 GO:GO:0009574 Uniprot:Q9FZ06
Root	Isotig00331	186	2	6.410	2.37E-43	TAIR|locus:2034640 - symbol:ARK3 "armadillo repeat kinesin 3" species:3702 "Arabidopsis thaliana" [GO:0003777 "microtubule motor activity" evidence=IEA;ISS] [GO:0005524 "ATP binding" evidence=IEA] [GO:0007018 "microtubule-based movement" evidence=IEA] [GO:0009574 "preprophase band" evidence=IDA] InterPro:IPR000225 InterPro:IPR001752 InterPro:IPR011989 InterPro:IPR016024 InterPro:IPR019821 Pfam:PF00225 Pfam:PF00514 PRINTS:PR00380 PROSITE:PS00411 PROSITE:PS50067 PROSITE:PS50176 SMART:SM00129 SMART:SM00185 EMBL:CP002684 GenomeReviews:CT485782_GR GO:GO:0005524 SUPFAM:SSF48371 Gene3D:G3DSA:1.25.10.10 eggNOG:COG5059 GO:GO:0005874 GO:GO:0003777 HSSP:P17119 HOGENOM:HBG319705 GO:GO:0007018 Gene3D:G3DSA:3.40.850.10 EMBL:AB290930 EMBL:AF159052 EMBL:AC025416 IPI:IPI00543027 RefSeq:NP_563908.1 UniGene:At.16100 ProteinModelPortal:Q9FZ06 SMR:Q9FZ06 PRIDE:Q9FZ06 EnsemblPlants:AT1G12430.1 GeneID:837799 KEGG:ath:AT1G12430 GeneFarm:2953 TAIR:At1g12430 InParanoid:Q9FZ06 OMA:HAILMVN PhylomeDB:Q9FZ06 Genevestigator:Q9FZ06 GO:GO:0009574 Uniprot:Q9FZ06
Root	Isotig00332	186	2	6.410	2.37E-43	TAIR|locus:2034640 - symbol:ARK3 "armadillo repeat kinesin 3" species:3702 "Arabidopsis thaliana" [GO:0003777 "microtubule motor activity" evidence=IEA;ISS] [GO:0005524 "ATP binding" evidence=IEA] [GO:0007018 "microtubule-based movement" evidence=IEA] [GO:0009574 "preprophase band" evidence=IDA] InterPro:IPR000225 InterPro:IPR001752 InterPro:IPR011989 InterPro:IPR016024 InterPro:IPR019821 Pfam:PF00225 Pfam:PF00514 PRINTS:PR00380 PROSITE:PS00411 PROSITE:PS50067 PROSITE:PS50176 SMART:SM00129 SMART:SM00185 EMBL:CP002684 GenomeReviews:CT485782_GR GO:GO:0005524 SUPFAM:SSF48371 Gene3D:G3DSA:1.25.10.10 eggNOG:COG5059 GO:GO:0005874 GO:GO:0003777 HSSP:P17119 HOGENOM:HBG319705 GO:GO:0007018 Gene3D:G3DSA:3.40.850.10 EMBL:AB290930 EMBL:AF159052 EMBL:AC025416 IPI:IPI00543027 RefSeq:NP_563908.1 UniGene:At.16100 ProteinModelPortal:Q9FZ06 SMR:Q9FZ06 PRIDE:Q9FZ06 EnsemblPlants:AT1G12430.1 GeneID:837799 KEGG:ath:AT1G12430 GeneFarm:2953 TAIR:At1g12430 InParanoid:Q9FZ06 OMA:HAILMVN PhylomeDB:Q9FZ06 Genevestigator:Q9FZ06 GO:GO:0009574 Uniprot:Q9FZ06
Root	Isotig00333	185	2	6.402	3.87E-43	TAIR|locus:2034640 - symbol:ARK3 "armadillo repeat kinesin 3" species:3702 "Arabidopsis thaliana" [GO:0003777 "microtubule motor activity" evidence=IEA;ISS] [GO:0005524 "ATP binding" evidence=IEA] [GO:0007018 "microtubule-based movement" evidence=IEA] [GO:0009574 "preprophase band" evidence=IDA] InterPro:IPR000225 InterPro:IPR001752 InterPro:IPR011989 InterPro:IPR016024 InterPro:IPR019821 Pfam:PF00225 Pfam:PF00514 PRINTS:PR00380 PROSITE:PS00411 PROSITE:PS50067 PROSITE:PS50176 SMART:SM00129 SMART:SM00185 EMBL:CP002684 GenomeReviews:CT485782_GR GO:GO:0005524 SUPFAM:SSF48371 Gene3D:G3DSA:1.25.10.10 eggNOG:COG5059 GO:GO:0005874 GO:GO:0003777 HSSP:P17119 HOGENOM:HBG319705 GO:GO:0007018 Gene3D:G3DSA:3.40.850.10 EMBL:AB290930 EMBL:AF159052 EMBL:AC025416 IPI:IPI00543027 RefSeq:NP_563908.1 UniGene:At.16100 ProteinModelPortal:Q9FZ06 SMR:Q9FZ06 PRIDE:Q9FZ06 EnsemblPlants:AT1G12430.1 GeneID:837799 KEGG:ath:AT1G12430 GeneFarm:2953 TAIR:At1g12430 InParanoid:Q9FZ06 OMA:HAILMVN PhylomeDB:Q9FZ06 Genevestigator:Q9FZ06 GO:GO:0009574 Uniprot:Q9FZ06

Tissue	Identification	# of reads (stress)	# of reads (control)	Fold Change	p-value	Description
Root	Isotig00334	177	1	7.339	2.33E-39	UNIPROTKB|F1MI03 - symbol:SRRM2 "Uncharacterized protein" species:9913 "Bos taurus" [GO:0015030 "Cajal body" evidence=IEA] [GO:0016607 "nuclear speck" evidence=IEA] [GO:0047485 "protein N-terminus binding" evidence=IEA] [GO:0070742 "C2H2 zinc finger domain binding" evidence=IEA] [GO:0071013 "catalytic step 2 spliceosome" evidence=IEA] InterPro:IPR024945 SMART:SM01104 InterPro:IPR013170 Pfam:PF08312 GeneTree:ENSGT00650000093061 OMA:TSPAPWK EMBL:DAAA02057317 EMBL:DAAA02057318 IPI:IPI00688395 Ensembl:ENSBTAT00000037527 Uniprot:F1MI03
Root	Isotig00336	176	1	7.330	3.60E-39	UNIPROTKB|F1MI03 - symbol:SRRM2 "Uncharacterized protein" species:9913 "Bos taurus" [GO:0015030 "Cajal body" evidence=IEA] [GO:0016607 "nuclear speck" evidence=IEA] [GO:0047485 "protein N-terminus binding" evidence=IEA] [GO:0070742 "C2H2 zinc finger domain binding" evidence=IEA] [GO:0071013 "catalytic step 2 spliceosome" evidence=IEA] InterPro:IPR024945 SMART:SM01104 InterPro:IPR013170 Pfam:PF08312 GeneTree:ENSGT00650000093061 OMA:TSPAPWK EMBL:DAAA02057317 EMBL:DAAA02057318 IPI:IPI00688395 Ensembl:ENSBTAT00000037527 Uniprot:F1MI03
Root	Isotig00342	193	10	4.142	2.62E-41	No hit
Root	Isotig00343	185	9	4.232	4.02E-40	No hit
Root	Isotig00344	190	9	4.271	2.39E-41	UNIPROTKB|E1BE02 - symbol:E2F7 "Uncharacterized protein" species:9913 "Bos taurus" [GO:0003677 "DNA binding" evidence=IEA] [GO:0003700 "sequence-specific DNA binding transcription factor activity" evidence=IEA] [GO:0005667 "transcription factor complex" evidence=IEA] [GO:0006351 "transcription, DNA-dependent" evidence=IEA] [GO:0008285 "negative regulation of cell proliferation" evidence=IEA] [GO:0042802 "identical protein binding" evidence=IEA] InterPro:IPR003316 Pfam:PF02319 GO:GO:0003677 GO:GO:0006351 InterPro:IPR011991 Gene3D:G3DSA:1.10.10.10 GO:GO:0005667 InterPro:IPR015633 PANTHER:PTHR12081 GeneTree:ENSGT00530000063616 OMA:QRKVNSE EMBL:DAAA02012389 EMBL:DAAA02012390 IPI:IPI00691572 Ensembl:ENSBTAT00000022518 PhylomeDB:E1BE02 Uniprot:E1BE02
Root	Isotig00345	94	5	4.104	7.25E-21	No hit
Root	Isotig00346	92	5	4.073	2.26E-20	ZFIN|ZDB-GENE-990415-15 - symbol:arx "aristaless related homeobox" species:7955 "Danio rerio" [GO:0043565 "sequence-specific DNA binding" evidence=IEA] [GO:0005634 "nucleus" evidence=IEA] [GO:0003677 "DNA binding" evidence=IEA] [GO:0006355 "regulation of transcription, DNA-dependent" evidence=IEA] [GO:0003700 "sequence-specific DNA binding transcription factor activity" evidence=IEA] [GO:0007275 "multicellular organismal development" evidence=IEA] [GO:0006351 "transcription, DNA-dependent" evidence=IEA] InterPro:IPR001356 InterPro:IPR009057 InterPro:IPR017970 Pfam:PF00046 PROSITE:PS00027 PROSITE:PS50071 SMART:SM00389 InterPro:IPR003654 ZFIN:ZDB-GENE-990415-15 GO:GO:0007275 GO:GO:0005634 GO:GO:0043565 GO:GO:0003700 GO:GO:0006351 Gene3D:G3DSA:1.10.10.60 SUPFAM:SSF46689 Pfam:PF03826 PROSITE:PS50803 eggNOG:NOG313402 GeneTree:ENSGT00650000092984 KO:K09452 EMBL:AB006104 IPI:IPI00502004 RefSeq:NP_571459.1 UniGene:Dr.9011 ProteinModelPortal:O42115 SMR:O42115 STRING:O42115 PRIDE:O42115 Ensembl:ENSDART00000080810 GeneID:30657 KEGG:dre:30657 CTD:170302 HOGENOM:HBG715732 HOVERGEN:HBG004285 InParanoid:O42115 OMA:RRSPCKV OrthoDB:EOG43TZW9 PhylomeDB:O42115 ArrayExpress:O42115 Bgee:O42115 Uniprot:O42115
Root	Isotig00347	133	8	3.926	3.96E-28	MGI|MGI:2144837 - symbol:Ptpn23 "protein tyrosine phosphatase, non-receptor type 23" species:10090 "Mus musculus" [GO:0005634 "nucleus" evidence=ISO] [GO:0005737 "cytoplasm" evidence=ISO] [GO:0005768 "endosome" evidence=ISO] [GO:0016311 "dephosphorylation" evidence=IEA] [GO:0016791 "phosphatase activity" evidence=IEA] [GO:0004725 "protein tyrosine phosphatase activity" evidence=IEA] [GO:0005932 "microtubule basal body" evidence=ISO] [GO:0005856 "cytoskeleton" evidence=IEA] [GO:0016787 "hydrolase activity" evidence=IEA] [GO:0030030 "cell projection organization" evidence=IEA] [GO:0004721 "phosphoprotein phosphatase activity" evidence=IEA] [GO:0005929 "cilium" evidence=IEA] [GO:0031410 "cytoplasmic vesicle" evidence=IEA] [GO:0042995 "cell projection" evidence=IEA] [GO:0006470 "protein dephosphorylation" evidence=IEA] [GO:0060271 "cilium morphogenesis" evidence=ISO] InterPro:IPR000242 InterPro:IPR000387 InterPro:IPR016130 InterPro:IPR025304 Pfam:PF00102 Pfam:PF13949 PRINTS:PR00700 PROSITE:PS00383 PROSITE:PS50005 PROSITE:PS50055 PROSITE:PS50056 PROSITE:PS50293 SMART:SM00194 MGI:MGI:2144837 GO:GO:0005634 GO:GO:0005768 GO:GO:0016023 GO:GO:0005929 GO:GO:0005932 InterPro:IPR004328 Gene3D:G3DSA:1.25.40.280 Pfam:PF03097 SMART:SM01041 PROSITE:PS51180 GeneTree:ENSGT00650000093161 GO:GO:0060271 GO:GO:0004725 eggNOG:COG5599 KO:K01104 PDB:2W10 PDBsum:2W10 CTD:25930 HOVERGEN:HBG082231 OMA:GPGPHYL EMBL:BY750106 EMBL:CF734421 EMBL:CB248963 EMBL:BC006582 EMBL:BC022721 EMBL:BC059902 EMBL:AK173178 IPI:IPI00464166

Tissue	Identification	# of reads (stress)	# of reads (control)	Fold Change	p-value	Description
						IPI:IPI00606716 RefSeq:NP_001074512.1 UniGene:Mm.335477 ProteinModelPortal:Q6PB44 SMR:Q6PB44 DIP:DIP-48351N IntAct:Q6PB44 STRING:Q6PB44 PhosphoSite:Q6PB44 PRIDE:Q6PB44 Ensembl:ENSMUST00000040021 GeneID:104831 KEGG:mmu:104831 UCSC:uc009rty.1 UCSC:uc012hbi.1 HOGENOM:HBG444522 InParanoid:Q6PB44 OrthoDB:EOG4NCMBX PhylomeDB:Q6PB44 NextBio:357310 ArrayExpress:Q6PB44 Bgee:Q6PB44 Genevestigator:Q6PB44 GermOnline:ENSMUSG00000036057 Uniprot:Q6PB44
Root	Isotig00348	123	6	4.229	3.15E-27	No hit
Root	Isotig00349	114	6	4.119	5.16E-25	No hit
Root	Isotig00351	30	3	3.193	1.42E-06	MGI|MGI:2144837 - symbol:Ptpn23 "protein tyrosine phosphatase, non-receptor type 23" species:10090 "Mus musculus" [GO:0005634 "nucleus" evidence=ISO] [GO:0005737 "cytoplasm" evidence=ISO] [GO:0005768 "endosome" evidence=ISO] [GO:0016311 "dephosphorylation" evidence=IEA] [GO:0016791 "phosphatase activity" evidence=IEA] [GO:0004725 "protein tyrosine phosphatase activity" evidence=IEA] [GO:0005932 "microtubule basal body" evidence=ISO] [GO:0005856 "cytoskeleton" evidence=IEA] [GO:0016787 "hydrolase activity" evidence=IEA] [GO:0030030 "cell projection organization" evidence=IEA] [GO:0004721 "phosphoprotein phosphatase activity" evidence=IEA] [GO:0005929 "cilium" evidence=IEA] [GO:0031410 "cytoplasmic vesicle" evidence=IEA] [GO:0042995 "cell projection" evidence=IEA] [GO:0006470 "protein dephosphorylation" evidence=IEA] [GO:0060271 "cilium morphogenesis" evidence=ISO] InterPro:IPR000242 InterPro:IPR000387 InterPro:IPR016130 InterPro:IPR025304 Pfam:PF00102 Pfam:PF13949 PRINTS:PR00700 PROSITE:PS00383 PROSITE:PS50005 PROSITE:PS50055 PROSITE:PS50056 PROSITE:PS50293 SMART:SM00194 MGI:MGI:2144837 GO:GO:0005634 GO:GO:0005768 GO:GO:0016023 GO:GO:0005929 GO:GO:0005932 InterPro:IPR004328 Gene3D:G3DSA:1.25.40.280 Pfam:PF03097 SMART:SM01041 PROSITE:PS51180 GeneTree:ENSGT00650000093161 GO:GO:0060271 GO:GO:0004725 eggNOG:COG5599 KO:K01104 PDB:2W10 PDBsum:2W10 CTD:25930 HOVERGEN:HBG082231 OMA:GPGPHYL EMBL:BY750106 EMBL:CF734421 EMBL:CB248963 EMBL:BC006582 EMBL:BC022721 EMBL:BC059902 EMBL:AK173178 IPI:IPI00464166 IPI:IPI00606716 RefSeq:NP_001074512.1 UniGene:Mm.335477 ProteinModelPortal:Q6PB44 SMR:Q6PB44 DIP:DIP-48351N IntAct:Q6PB44 STRING:Q6PB44 PhosphoSite:Q6PB44 PRIDE:Q6PB44 Ensembl:ENSMUST00000040021 GeneID:104831 KEGG:mmu:104831 UCSC:uc009rty.1 UCSC:uc012hbi.1 HOGENOM:HBG444522 InParanoid:Q6PB44 OrthoDB:EOG4NCMBX PhylomeDB:Q6PB44 NextBio:357310 ArrayExpress:Q6PB44 Bgee:Q6PB44 Genevestigator:Q6PB44 GermOnline:ENSMUSG00000036057 Uniprot:Q6PB44
Root	Isotig00352	13	1	3.571	0.000882483	UNIPROTKB|E2R9J1 - symbol:HEYL "Uncharacterized protein" species:9615 "Canis lupus familiaris" [GO:0003677 "DNA binding" evidence=IEA] [GO:0005634 "nucleus" evidence=IEA] [GO:0006351 "transcription, DNA-dependent" evidence=IEA] [GO:0006355 "regulation of transcription, DNA-dependent" evidence=IEA] InterPro:IPR003650 Pfam:PF07527 PROSITE:PS51054 InterPro:IPR011598 Pfam:PF00010 GO:GO:0005634 GO:GO:0006355 GO:GO:0003677 GO:GO:0006351 Gene3D:G3DSA:4.10.280.10 SMART:SM00353 SUPFAM:SSF47459 PROSITE:PS50888 InterPro:IPR018352 SMART:SM00511 GeneTree:ENSGT00510000046466 KO:K09091 CTD:26508 OMA:WVSEITE RefSeq:XP_849702.1 Ensembl:ENSCAFT00000004941 GeneID:607804 KEGG:cfa:607804 Uniprot:E2R9J1
Root	Isotig00353	28	3	3.093	4.38E-06	MGI|MGI:2144837 - symbol:Ptpn23 "protein tyrosine phosphatase, non-receptor type 23" species:10090 "Mus musculus" [GO:0005634 "nucleus" evidence=ISO] [GO:0005737 "cytoplasm" evidence=ISO] [GO:0005768 "endosome" evidence=ISO] [GO:0016311 "dephosphorylation" evidence=IEA] [GO:0016791 "phosphatase activity" evidence=IEA] [GO:0004725 "protein tyrosine phosphatase activity" evidence=IEA] [GO:0005932 "microtubule basal body" evidence=ISO] [GO:0005856 "cytoskeleton" evidence=IEA] [GO:0016787 "hydrolase activity" evidence=IEA] [GO:0030030 "cell projection organization" evidence=IEA] [GO:0004721 "phosphoprotein phosphatase activity" evidence=IEA] [GO:0005929 "cilium" evidence=IEA] [GO:0031410 "cytoplasmic vesicle" evidence=IEA] [GO:0042995 "cell projection" evidence=IEA] [GO:0006470 "protein dephosphorylation" evidence=IEA] [GO:0060271 "cilium morphogenesis" evidence=ISO] InterPro:IPR000242 InterPro:IPR000387 InterPro:IPR016130 InterPro:IPR025304 Pfam:PF00102 Pfam:PF13949 PRINTS:PR00700 PROSITE:PS00383 PROSITE:PS50005 PROSITE:PS50055 PROSITE:PS50056 PROSITE:PS50293 SMART:SM00194 MGI:MGI:2144837 GO:GO:0005634 GO:GO:0005768 GO:GO:0016023 GO:GO:0005929 GO:GO:0005932 InterPro:IPR004328 Gene3D:G3DSA:1.25.40.280 Pfam:PF03097 SMART:SM01041 PROSITE:PS51180 GeneTree:ENSGT00650000093161 GO:GO:0060271 GO:GO:0004725 eggNOG:COG5599 KO:K01104 PDB:2W10

Tissue	Identification	# of reads (stress)	# of reads (control)	Fold Change	p-value	Description
						PDBsum:2W10 CTD:25930 HOVERGEN:HBG082231 OMA:GPGPHYL EMBL:BY750106 EMBL:CF734421 EMBL:CB248963 EMBL:BC006582 EMBL:BC022721 EMBL:BC059902 EMBL:AK173178 IPI:IPI00464166 IPI:IPI00606716 RefSeq:NP_001074512.1 UniGene:Mm.335477 ProteinModelPortal:Q6PB44 SMR:Q6PB44 DIP:DIP-48351N IntAct:Q6PB44 STRING:Q6PB44 PhosphoSite:Q6PB44 PRIDE:Q6PB44 Ensembl:ENSMUST00000040021 GeneID:104831 KEGG:mmu:104831 UCSC:uc009rty.1 UCSC:uc012hbi.1 HOGENOM:HBG444522 InParanoid:Q6PB44 OrthoDB:EOG4NCMBX PhylomeDB:Q6PB44 NextBio:357310 ArrayExpress:Q6PB44 Bgee:Q6PB44 Genevestigator:Q6PB44 GermOnline:ENSMUSG00000036057 Uniprot:Q6PB44
Root	Isotig00354	13	1	3.571	0.000882483	UNIPROTKB|E2R9J1 - symbol:HEYL "Uncharacterized protein" species:9615 "Canis lupus familiaris" [GO:0003677 "DNA binding" evidence=IEA] [GO:0005634 "nucleus" evidence=IEA] [GO:0006351 "transcription, DNA-dependent" evidence=IEA] [GO:0006355 "regulation of transcription, DNA-dependent" evidence=IEA] InterPro:IPR003650 Pfam:PF07527 PROSITE:PS51054 InterPro:IPR011598 Pfam:PF00010 GO:GO:0005634 GO:GO:0006355 GO:GO:0003677 GO:GO:0006351 Gene3D:G3DSA:4.10.280.10 SMART:SM00353 SUPFAM:SSF47459 PROSITE:PS50888 InterPro:IPR018352 SMART:SM00511 GeneTree:ENSGT00510000046466 KO:K09091 CTD:26508 OMA:WVSEITE RefSeq:XP_849702.1 Ensembl:ENSCAFT00000004941 GeneID:607804 KEGG:cfa:607804 Uniprot:E2R9J1
Root	Isotig00366	24	78	-1.829	3.61E-09	TAIR|locus:2079834 - symbol:AAO2 "aldehyde oxidase 2" species:3702 "Arabidopsis thaliana" [GO:0055114 "oxidation-reduction process" evidence=IEA] [GO:0004031 "aldehyde oxidase activity" evidence=IDA] [GO:0005829 "cytosol" evidence=IDA] InterPro:IPR001041 InterPro:IPR002346 InterPro:IPR002888 InterPro:IPR006058 InterPro:IPR008274 InterPro:IPR016166 InterPro:IPR016169 InterPro:IPR016208 Pfam:PF00111 Pfam:PF00941 Pfam:PF01799 Pfam:PF02738 PIRSF:PIRSF000127 PROSITE:PS00197 PROSITE:PS00559 PROSITE:PS51085 PROSITE:PS51387 InterPro:IPR000674 Pfam:PF01315 GO:GO:0005829 EMBL:CP002686 GenomeReviews:BA000014_GR GO:GO:0005506 GO:GO:0009055 GO:GO:0050660 SUPFAM:SSF56176 GO:GO:0004031 eggNOG:COG4630 GO:GO:0051537 InterPro:IPR012675 InterPro:IPR005107 Gene3D:G3DSA:1.10.150.120 Gene3D:G3DSA:3.30.365.10 Gene3D:G3DSA:3.90.1170.50 Gene3D:G3DSA:3.30.390.50 Gene3D:G3DSA:3.30.465.10 Gene3D:G3DSA:3.10.20.30 Pfam:PF03450 SMART:SM01008 SMART:SM01092 SUPFAM:SSF47741 SUPFAM:SSF56003 SUPFAM:SSF54665 SUPFAM:SSF55447 SUPFAM:SSF54292 HOGENOM:HBG746468 ProtClustDB:PLN00192 EMBL:AB005805 EMBL:AL391734 EMBL:AF039896 IPI:IPI00529435 PIR:T51623 PIR:T52050 RefSeq:NP_189946.1 UniGene:At.462 ProteinModelPortal:Q7G192 SMR:Q7G192 STRING:Q7G192 PRIDE:Q7G192 EnsemblPlants:AT3G43600.1 GeneID:823457 KEGG:ath:AT3G43600 GeneFarm:4893 TAIR:At3g43600 InParanoid:Q7G192 OMA:ILYDCGK PhylomeDB:Q7G192 BioCyc:ARA:AT3G43600-MONOMER BioCyc:MetaCyc:AT3G43600-MONOMER Genevestigator:Q7G192 GermOnline:AT3G43600 GO:GO:0016614 Uniprot:Q7G192
Root	Isotig00367	24	78	-1.829	3.61E-09	TAIR|locus:2079834 - symbol:AAO2 "aldehyde oxidase 2" species:3702 "Arabidopsis thaliana" [GO:0055114 "oxidation-reduction process" evidence=IEA] [GO:0004031 "aldehyde oxidase activity" evidence=IDA] [GO:0005829 "cytosol" evidence=IDA] InterPro:IPR001041 InterPro:IPR002346 InterPro:IPR002888 InterPro:IPR006058 InterPro:IPR008274 InterPro:IPR016166 InterPro:IPR016169 InterPro:IPR016208 Pfam:PF00111 Pfam:PF00941 Pfam:PF01799 Pfam:PF02738 PIRSF:PIRSF000127 PROSITE:PS00197 PROSITE:PS00559 PROSITE:PS51085 PROSITE:PS51387 InterPro:IPR000674 Pfam:PF01315 GO:GO:0005829 EMBL:CP002686 GenomeReviews:BA000014_GR GO:GO:0005506 GO:GO:0009055 GO:GO:0050660 SUPFAM:SSF56176 GO:GO:0004031 eggNOG:COG4630 GO:GO:0051537 InterPro:IPR012675 InterPro:IPR005107 Gene3D:G3DSA:1.10.150.120 Gene3D:G3DSA:3.30.365.10 Gene3D:G3DSA:3.90.1170.50 Gene3D:G3DSA:3.30.390.50 Gene3D:G3DSA:3.30.465.10 Gene3D:G3DSA:3.10.20.30 Pfam:PF03450 SMART:SM01008 SMART:SM01092 SUPFAM:SSF47741 SUPFAM:SSF56003 SUPFAM:SSF54665 SUPFAM:SSF55447 SUPFAM:SSF54292 HOGENOM:HBG746468 ProtClustDB:PLN00192 EMBL:AB005805 EMBL:AL391734 EMBL:AF039896 IPI:IPI00529435 PIR:T51623 PIR:T52050 RefSeq:NP_189946.1 UniGene:At.462 ProteinModelPortal:Q7G192 SMR:Q7G192 STRING:Q7G192 PRIDE:Q7G192 EnsemblPlants:AT3G43600.1 GeneID:823457 KEGG:ath:AT3G43600 GeneFarm:4893 TAIR:At3g43600 InParanoid:Q7G192 OMA:ILYDCGK PhylomeDB:Q7G192 BioCyc:ARA:AT3G43600-MONOMER BioCyc:MetaCyc:AT3G43600-MONOMER Genevestigator:Q7G192 GermOnline:AT3G43600 GO:GO:0016614 Uniprot:Q7G192
Root	Isotig00368	19	84	-2.273	1.83E-12	TAIR|locus:2079834 - symbol:AAO2 "aldehyde oxidase 2" species:3702 "Arabidopsis thaliana" [GO:0055114 "oxidation-reduction process" evidence=IEA] [GO:0004031 "aldehyde oxidase activity" evidence=IDA] [GO:0005829

Tissue	Identification	# of reads (stress)	# of reads (control)	Fold Change	p-value	Description
						"cytosol" evidence=IDA] InterPro:IPR001041 InterPro:IPR002346 InterPro:IPR002888 InterPro:IPR006058 InterPro:IPR008274 InterPro:IPR016166 InterPro:IPR016169 InterPro:IPR016208 Pfam:PF00111 Pfam:PF00941 Pfam:PF01799 Pfam:PF02738 PIRSF:PIRSF000127 PROSITE:PS00197 PROSITE:PS00559 PROSITE:PS51085 PROSITE:PS51387 InterPro:IPR000674 Pfam:PF01315 GO:GO:0005829 EMBL:CP002686 GenomeReviews:BA000014_GR GO:GO:0005506 GO:GO:0009055 GO:GO:0050660 SUPFAM:SSF56176 GO:GO:0004031 eggNOG:COG4630 GO:GO:0051537 InterPro:IPR012675 InterPro:IPR005107 Gene3D:G3DSA:1.10.150.120 Gene3D:G3DSA:3.30.365.10 Gene3D:G3DSA:3.90.1170.50 Gene3D:G3DSA:3.30.390.50 Gene3D:G3DSA:3.30.465.10 Gene3D:G3DSA:3.10.20.30 Pfam:PF03450 SMART:SM01008 SMART:SM01092 SUPFAM:SSF47741 SUPFAM:SSF56003 SUPFAM:SSF54665 SUPFAM:SSF55447 SUPFAM:SSF54292 HOGENOM:HBG746468 ProtClustDB:PLN00192 EMBL:AB005805 EMBL:AL391734 EMBL:AF039896 IPI:IPI00529435 PIR:T51623 PIR:T52050 RefSeq:NP_189946.1 UniGene:At.462 ProteinModelPortal:Q7G192 SMR:Q7G192 STRING:Q7G192 PRIDE:Q7G192 EnsemblPlants:AT3G43600.1 GeneID:823457 KEGG:ath:AT3G43600 GeneFarm:4893 TAIR:At3g43600 InParanoid:Q7G192 OMA:ILYDCGK PhylomeDB:Q7G192 BioCyc:ARA:AT3G43600-MONOMER BioCyc:MetaCyc:AT3G43600-MONOMER Genevestigator:Q7G192 GermOnline:AT3G43600 GO:GO:0016614 Uniprot:Q7G192
Root	Isotig00369	24	78	-1.829	3.61E-09	TAIR|locus:2079834 - symbol:AAO2 "aldehyde oxidase 2" species:3702 "Arabidopsis thaliana" [GO:0055114 "oxidation-reduction process" evidence=IEA] [GO:0004031 "aldehyde oxidase activity" evidence=IDA] [GO:0005829 "cytosol" evidence=IDA] InterPro:IPR001041 InterPro:IPR002346 InterPro:IPR002888 InterPro:IPR006058 InterPro:IPR008274 InterPro:IPR016166 InterPro:IPR016169 InterPro:IPR016208 Pfam:PF00111 Pfam:PF00941 Pfam:PF01799 Pfam:PF02738 PIRSF:PIRSF000127 PROSITE:PS00197 PROSITE:PS00559 PROSITE:PS51085 PROSITE:PS51387 InterPro:IPR000674 Pfam:PF01315 GO:GO:0005829 EMBL:CP002686 GenomeReviews:BA000014_GR GO:GO:0005506 GO:GO:0009055 GO:GO:0050660 SUPFAM:SSF56176 GO:GO:0004031 eggNOG:COG4630 GO:GO:0051537 InterPro:IPR012675 InterPro:IPR005107 Gene3D:G3DSA:1.10.150.120 Gene3D:G3DSA:3.30.365.10 Gene3D:G3DSA:3.90.1170.50 Gene3D:G3DSA:3.30.390.50 Gene3D:G3DSA:3.30.465.10 Gene3D:G3DSA:3.10.20.30 Pfam:PF03450 SMART:SM01008 SMART:SM01092 SUPFAM:SSF47741 SUPFAM:SSF56003 SUPFAM:SSF54665 SUPFAM:SSF55447 SUPFAM:SSF54292 HOGENOM:HBG746468 ProtClustDB:PLN00192 EMBL:AB005805 EMBL:AL391734 EMBL:AF039896 IPI:IPI00529435 PIR:T51623 PIR:T52050 RefSeq:NP_189946.1 UniGene:At.462 ProteinModelPortal:Q7G192 SMR:Q7G192 STRING:Q7G192 PRIDE:Q7G192 EnsemblPlants:AT3G43600.1 GeneID:823457 KEGG:ath:AT3G43600 GeneFarm:4893 TAIR:At3g43600 InParanoid:Q7G192 OMA:ILYDCGK PhylomeDB:Q7G192 BioCyc:ARA:AT3G43600-MONOMER BioCyc:MetaCyc:AT3G43600-MONOMER Genevestigator:Q7G192 GermOnline:AT3G43600 GO:GO:0016614 Uniprot:Q7G192
Root	Isotig00370	24	78	-1.829	3.61E-09	TAIR|locus:2079834 - symbol:AAO2 "aldehyde oxidase 2" species:3702 "Arabidopsis thaliana" [GO:0055114 "oxidation-reduction process" evidence=IEA] [GO:0004031 "aldehyde oxidase activity" evidence=IDA] [GO:0005829 "cytosol" evidence=IDA] InterPro:IPR001041 InterPro:IPR002346 InterPro:IPR002888 InterPro:IPR006058 InterPro:IPR008274 InterPro:IPR016166 InterPro:IPR016169 InterPro:IPR016208 Pfam:PF00111 Pfam:PF00941 Pfam:PF01799 Pfam:PF02738 PIRSF:PIRSF000127 PROSITE:PS00197 PROSITE:PS00559 PROSITE:PS51085 PROSITE:PS51387 InterPro:IPR000674 Pfam:PF01315 GO:GO:0005829 EMBL:CP002686 GenomeReviews:BA000014_GR GO:GO:0005506 GO:GO:0009055 GO:GO:0050660 SUPFAM:SSF56176 GO:GO:0004031 eggNOG:COG4630 GO:GO:0051537 InterPro:IPR012675 InterPro:IPR005107 Gene3D:G3DSA:1.10.150.120 Gene3D:G3DSA:3.30.365.10 Gene3D:G3DSA:3.90.1170.50 Gene3D:G3DSA:3.30.390.50 Gene3D:G3DSA:3.30.465.10 Gene3D:G3DSA:3.10.20.30 Pfam:PF03450 SMART:SM01008 SMART:SM01092 SUPFAM:SSF47741 SUPFAM:SSF56003 SUPFAM:SSF54665 SUPFAM:SSF55447 SUPFAM:SSF54292 HOGENOM:HBG746468 ProtClustDB:PLN00192 EMBL:AB005805 EMBL:AL391734 EMBL:AF039896 IPI:IPI00529435 PIR:T51623 PIR:T52050 RefSeq:NP_189946.1 UniGene:At.462 ProteinModelPortal:Q7G192 SMR:Q7G192 STRING:Q7G192 PRIDE:Q7G192 EnsemblPlants:AT3G43600.1 GeneID:823457 KEGG:ath:AT3G43600 GeneFarm:4893 TAIR:At3g43600 InParanoid:Q7G192 OMA:ILYDCGK PhylomeDB:Q7G192 BioCyc:ARA:AT3G43600-MONOMER BioCyc:MetaCyc:AT3G43600-MONOMER Genevestigator:Q7G192 GermOnline:AT3G43600 GO:GO:0016614 Uniprot:Q7G192
Root	Isotig00371	19	84	-2.273	1.83E-12	TAIR|locus:2079834 - symbol:AAO2 "aldehyde oxidase 2" species:3702 "Arabidopsis thaliana" [GO:0055114 "oxidation-reduction process" evidence=IEA] [GO:0004031 "aldehyde oxidase activity" evidence=IDA] [GO:0005829 "cytosol" evidence=IDA] InterPro:IPR001041 InterPro:IPR002346 InterPro:IPR002888 InterPro:IPR006058

Tissue	Identification	# of reads (stress)	# of reads (control)	Fold Change	p-value	Description
						InterPro:IPR008274 InterPro:IPR016166 InterPro:IPR016169 InterPro:IPR016208 Pfam:PF00111 Pfam:PF00941 Pfam:PF01799 Pfam:PF02738 PIRSF:PIRSF000127 PROSITE:PS00197 PROSITE:PS00559 PROSITE:PS51085 PROSITE:PS51387 InterPro:IPR000674 Pfam:PF01315 GO:GO:0005829 EMBL:CP002686 GenomeReviews:BA000014_GR GO:GO:0005506 GO:GO:0009055 GO:GO:0050660 SUPFAM:SSF56176 GO:GO:0004031 eggNOG:COG4630 GO:GO:0051537 InterPro:IPR012675 InterPro:IPR005107 Gene3D:G3DSA:1.10.150.120 Gene3D:G3DSA:3.30.365.10 Gene3D:G3DSA:3.90.1170.50 Gene3D:G3DSA:3.30.390.50 Gene3D:G3DSA:3.30.465.10 Gene3D:G3DSA:3.10.20.30 Pfam:PF03450 SMART:SM01008 SMART:SM01092 SUPFAM:SSF47741 SUPFAM:SSF56003 SUPFAM:SSF54665 SUPFAM:SSF55447 SUPFAM:SSF54292 HOGENOM:HBG746468 ProtClustDB:PLN00192 EMBL:AB005805 EMBL:AL391734 EMBL:AF039896 IPI:IPI00529435 PIR:T51623 PIR:T52050 RefSeq:NP_189946.1 UniGene:At.462 ProteinModelPortal:Q7G192 SMR:Q7G192 STRING:Q7G192 PRIDE:Q7G192 EnsemblPlants:AT3G43600.1 GeneID:823457 KEGG:ath:AT3G43600 GeneFarm:4893 TAIR:At3g43600 InParanoid:Q7G192 OMA:ILYDCGK PhylomeDB:Q7G192 BioCyc:ARA:AT3G43600-MONOMER BioCyc:MetaCyc:AT3G43600-MONOMER Genevestigator:Q7G192 GermOnline:AT3G43600 GO:GO:0016614 Uniprot:Q7G192
Root	Isotig00372	19	84	-2.273	1.83E-12	TAIR|locus:2079834 - symbol:AAO2 "aldehyde oxidase 2" species:3702 "Arabidopsis thaliana" [GO:0055114 "oxidation-reduction process" evidence=IEA] [GO:0004031 "aldehyde oxidase activity" evidence=IDA] [GO:0005829 "cytosol" evidence=IDA] InterPro:IPR001041 InterPro:IPR002346 InterPro:IPR002888 InterPro:IPR006058 InterPro:IPR008274 InterPro:IPR016166 InterPro:IPR016169 InterPro:IPR016208 Pfam:PF00111 Pfam:PF00941 Pfam:PF01799 Pfam:PF02738 PIRSF:PIRSF000127 PROSITE:PS00197 PROSITE:PS00559 PROSITE:PS51085 PROSITE:PS51387 InterPro:IPR000674 Pfam:PF01315 GO:GO:0005829 EMBL:CP002686 GenomeReviews:BA000014_GR GO:GO:0005506 GO:GO:0009055 GO:GO:0050660 SUPFAM:SSF56176 GO:GO:0004031 eggNOG:COG4630 GO:GO:0051537 InterPro:IPR012675 InterPro:IPR005107 Gene3D:G3DSA:1.10.150.120 Gene3D:G3DSA:3.30.365.10 Gene3D:G3DSA:3.90.1170.50 Gene3D:G3DSA:3.30.390.50 Gene3D:G3DSA:3.30.465.10 Gene3D:G3DSA:3.10.20.30 Pfam:PF03450 SMART:SM01008 SMART:SM01092 SUPFAM:SSF47741 SUPFAM:SSF56003 SUPFAM:SSF54665 SUPFAM:SSF55447 SUPFAM:SSF54292 HOGENOM:HBG746468 ProtClustDB:PLN00192 EMBL:AB005805 EMBL:AL391734 EMBL:AF039896 IPI:IPI00529435 PIR:T51623 PIR:T52050 RefSeq:NP_189946.1 UniGene:At.462 ProteinModelPortal:Q7G192 SMR:Q7G192 STRING:Q7G192 PRIDE:Q7G192 EnsemblPlants:AT3G43600.1 GeneID:823457 KEGG:ath:AT3G43600 GeneFarm:4893 TAIR:At3g43600 InParanoid:Q7G192 OMA:ILYDCGK PhylomeDB:Q7G192 BioCyc:ARA:AT3G43600-MONOMER BioCyc:MetaCyc:AT3G43600-MONOMER Genevestigator:Q7G192 GermOnline:AT3G43600 GO:GO:0016614 Uniprot:Q7G192
Root	Isotig00373	19	84	-2.273	1.83E-12	TAIR|locus:2079834 - symbol:AAO2 "aldehyde oxidase 2" species:3702 "Arabidopsis thaliana" [GO:0055114 "oxidation-reduction process" evidence=IEA] [GO:0004031 "aldehyde oxidase activity" evidence=IDA] [GO:0005829 "cytosol" evidence=IDA] InterPro:IPR001041 InterPro:IPR002346 InterPro:IPR002888 InterPro:IPR006058 InterPro:IPR008274 InterPro:IPR016166 InterPro:IPR016169 InterPro:IPR016208 Pfam:PF00111 Pfam:PF00941 Pfam:PF01799 Pfam:PF02738 PIRSF:PIRSF000127 PROSITE:PS00197 PROSITE:PS00559 PROSITE:PS51085 PROSITE:PS51387 InterPro:IPR000674 Pfam:PF01315 GO:GO:0005829 EMBL:CP002686 GenomeReviews:BA000014_GR GO:GO:0005506 GO:GO:0009055 GO:GO:0050660 SUPFAM:SSF56176 GO:GO:0004031 eggNOG:COG4630 GO:GO:0051537 InterPro:IPR012675 InterPro:IPR005107 Gene3D:G3DSA:1.10.150.120 Gene3D:G3DSA:3.30.365.10 Gene3D:G3DSA:3.90.1170.50 Gene3D:G3DSA:3.30.390.50 Gene3D:G3DSA:3.30.465.10 Gene3D:G3DSA:3.10.20.30 Pfam:PF03450 SMART:SM01008 SMART:SM01092 SUPFAM:SSF47741 SUPFAM:SSF56003 SUPFAM:SSF54665 SUPFAM:SSF55447 SUPFAM:SSF54292 HOGENOM:HBG746468 ProtClustDB:PLN00192 EMBL:AB005805 EMBL:AL391734 EMBL:AF039896 IPI:IPI00529435 PIR:T51623 PIR:T52050 RefSeq:NP_189946.1 UniGene:At.462 ProteinModelPortal:Q7G192 SMR:Q7G192 STRING:Q7G192 PRIDE:Q7G192 EnsemblPlants:AT3G43600.1 GeneID:823457 KEGG:ath:AT3G43600 GeneFarm:4893 TAIR:At3g43600 InParanoid:Q7G192 OMA:ILYDCGK PhylomeDB:Q7G192 BioCyc:ARA:AT3G43600-MONOMER BioCyc:MetaCyc:AT3G43600-MONOMER Genevestigator:Q7G192 GermOnline:AT3G43600 GO:GO:0016614 Uniprot:Q7G192
Root	Isotig00374	24	73	-1.734	3.78E-08	TAIR|locus:2079834 - symbol:AAO2 "aldehyde oxidase 2" species:3702 "Arabidopsis thaliana" [GO:0055114 "oxidation-reduction process" evidence=IEA] [GO:0004031 "aldehyde oxidase activity" evidence=IDA] [GO:0005829 "cytosol" evidence=IDA] InterPro:IPR001041 InterPro:IPR002346 InterPro:IPR002888 InterPro:IPR006058 InterPro:IPR008274 InterPro:IPR016166 InterPro:IPR016169 InterPro:IPR016208 Pfam:PF00111 Pfam:PF00941

Tissue	Identification	# of reads (stress)	# of reads (control)	Fold Change	p-value	Description
						Pfam:PF01799 Pfam:PF02738 PIRSF:PIRSF000127 PROSITE:PS00197 PROSITE:PS00559 PROSITE:PS51085 PROSITE:PS51387 InterPro:IPR000674 Pfam:PF01315 GO:GO:0005829 EMBL:CP002686 GenomeReviews:BA000014_GR GO:GO:0005506 GO:GO:0009055 GO:GO:0050660 SUPFAM:SSF56176 GO:GO:0004031 eggNOG:COG4630 GO:GO:0051537 InterPro:IPR012675 InterPro:IPR005107 Gene3D:G3DSA:1.10.150.120 Gene3D:G3DSA:3.30.365.10 Gene3D:G3DSA:3.90.1170.50 Gene3D:G3DSA:3.30.390.50 Gene3D:G3DSA:3.30.465.10 Gene3D:G3DSA:3.10.20.30 Pfam:PF03450 SMART:SM01008 SMART:SM01092 SUPFAM:SSF47741 SUPFAM:SSF56003 SUPFAM:SSF54665 SUPFAM:SSF55447 SUPFAM:SSF54292 HOGENOM:HBG746468 ProtClustDB:PLN00192 EMBL:AB005805 EMBL:AL391734 EMBL:AF039896 IPI:IPI00529435 PIR:T51623 PIR:T52050 RefSeq:NP_189946.1 UniGene:At.462 ProteinModelPortal:Q7G192 SMR:Q7G192 STRING:Q7G192 PRIDE:Q7G192 EnsemblPlants:AT3G43600.1 GeneID:823457 KEGG:ath:AT3G43600 GeneFarm:4893 TAIR:At3g43600 InParanoid:Q7G192 OMA:ILYDCGK PhylomeDB:Q7G192 BioCyc:ARA:AT3G43600-MONOMER BioCyc:MetaCyc:AT3G43600-MONOMER Genevestigator:Q7G192 GermOnline:AT3G43600 GO:GO:0016614 Uniprot:Q7G192
Root	Isotig00375	24	73	-1.734	3.78E-08	TAIR|locus:2079834 - symbol:AAO2 "aldehyde oxidase 2" species:3702 "Arabidopsis thaliana" [GO:0055114 "oxidation-reduction process" evidence=IEA] [GO:0004031 "aldehyde oxidase activity" evidence=IDA] [GO:0005829 "cytosol" evidence=IDA] InterPro:IPR001041 InterPro:IPR002346 InterPro:IPR002888 InterPro:IPR006058 InterPro:IPR008274 InterPro:IPR016166 InterPro:IPR016169 InterPro:IPR016208 Pfam:PF00111 Pfam:PF00941 Pfam:PF01799 Pfam:PF02738 PIRSF:PIRSF000127 PROSITE:PS00197 PROSITE:PS00559 PROSITE:PS51085 PROSITE:PS51387 InterPro:IPR000674 Pfam:PF01315 GO:GO:0005829 EMBL:CP002686 GenomeReviews:BA000014_GR GO:GO:0005506 GO:GO:0009055 GO:GO:0050660 SUPFAM:SSF56176 GO:GO:0004031 eggNOG:COG4630 GO:GO:0051537 InterPro:IPR012675 InterPro:IPR005107 Gene3D:G3DSA:1.10.150.120 Gene3D:G3DSA:3.30.365.10 Gene3D:G3DSA:3.90.1170.50 Gene3D:G3DSA:3.30.390.50 Gene3D:G3DSA:3.30.465.10 Gene3D:G3DSA:3.10.20.30 Pfam:PF03450 SMART:SM01008 SMART:SM01092 SUPFAM:SSF47741 SUPFAM:SSF56003 SUPFAM:SSF54665 SUPFAM:SSF55447 SUPFAM:SSF54292 HOGENOM:HBG746468 ProtClustDB:PLN00192 EMBL:AB005805 EMBL:AL391734 EMBL:AF039896 IPI:IPI00529435 PIR:T51623 PIR:T52050 RefSeq:NP_189946.1 UniGene:At.462 ProteinModelPortal:Q7G192 SMR:Q7G192 STRING:Q7G192 PRIDE:Q7G192 EnsemblPlants:AT3G43600.1 GeneID:823457 KEGG:ath:AT3G43600 GeneFarm:4893 TAIR:At3g43600 InParanoid:Q7G192 OMA:ILYDCGK PhylomeDB:Q7G192 BioCyc:ARA:AT3G43600-MONOMER BioCyc:MetaCyc:AT3G43600-MONOMER Genevestigator:Q7G192 GermOnline:AT3G43600 GO:GO:0016614 Uniprot:Q7G192
Root	Isotig00376	19	79	-2.185	2.58E-11	TAIR|locus:2079834 - symbol:AAO2 "aldehyde oxidase 2" species:3702 "Arabidopsis thaliana" [GO:0055114 "oxidation-reduction process" evidence=IEA] [GO:0004031 "aldehyde oxidase activity" evidence=IDA] [GO:0005829 "cytosol" evidence=IDA] InterPro:IPR001041 InterPro:IPR002346 InterPro:IPR002888 InterPro:IPR006058 InterPro:IPR008274 InterPro:IPR016166 InterPro:IPR016169 InterPro:IPR016208 Pfam:PF00111 Pfam:PF00941 Pfam:PF01799 Pfam:PF02738 PIRSF:PIRSF000127 PROSITE:PS00197 PROSITE:PS00559 PROSITE:PS51085 PROSITE:PS51387 InterPro:IPR000674 Pfam:PF01315 GO:GO:0005829 EMBL:CP002686 GenomeReviews:BA000014_GR GO:GO:0005506 GO:GO:0009055 GO:GO:0050660 SUPFAM:SSF56176 GO:GO:0004031 eggNOG:COG4630 GO:GO:0051537 InterPro:IPR012675 InterPro:IPR005107 Gene3D:G3DSA:1.10.150.120 Gene3D:G3DSA:3.30.365.10 Gene3D:G3DSA:3.90.1170.50 Gene3D:G3DSA:3.30.390.50 Gene3D:G3DSA:3.30.465.10 Gene3D:G3DSA:3.10.20.30 Pfam:PF03450 SMART:SM01008 SMART:SM01092 SUPFAM:SSF47741 SUPFAM:SSF56003 SUPFAM:SSF54665 SUPFAM:SSF55447 SUPFAM:SSF54292 HOGENOM:HBG746468 ProtClustDB:PLN00192 EMBL:AB005805 EMBL:AL391734 EMBL:AF039896 IPI:IPI00529435 PIR:T51623 PIR:T52050 RefSeq:NP_189946.1 UniGene:At.462 ProteinModelPortal:Q7G192 SMR:Q7G192 STRING:Q7G192 PRIDE:Q7G192 EnsemblPlants:AT3G43600.1 GeneID:823457 KEGG:ath:AT3G43600 GeneFarm:4893 TAIR:At3g43600 InParanoid:Q7G192 OMA:ILYDCGK PhylomeDB:Q7G192 BioCyc:ARA:AT3G43600-MONOMER BioCyc:MetaCyc:AT3G43600-MONOMER Genevestigator:Q7G192 GermOnline:AT3G43600 GO:GO:0016614 Uniprot:Q7G192
Root	Isotig00377	24	73	-1.734	3.78E-08	TAIR|locus:2079834 - symbol:AAO2 "aldehyde oxidase 2" species:3702 "Arabidopsis thaliana" [GO:0055114 "oxidation-reduction process" evidence=IEA] [GO:0004031 "aldehyde oxidase activity" evidence=IDA] [GO:0005829 "cytosol" evidence=IDA] InterPro:IPR001041 InterPro:IPR002346 InterPro:IPR002888 InterPro:IPR006058 InterPro:IPR008274 InterPro:IPR016166 InterPro:IPR016169 InterPro:IPR016208 Pfam:PF00111 Pfam:PF00941 Pfam:PF01799 Pfam:PF02738 PIRSF:PIRSF000127 PROSITE:PS00197 PROSITE:PS00559 PROSITE:PS51085

Tissue	Identification	# of reads (stress)	# of reads (control)	Fold Change	p-value	Description
						PROSITE:PS51387 InterPro:IPR000674 Pfam:PF01315 GO:GO:0005829 EMBL:CP002686 GenomeReviews:BA000014_GR GO:GO:0005506 GO:GO:0009055 GO:GO:0050660 SUPFAM:SSF56176 GO:GO:0004031 eggNOG:COG4630 GO:GO:0051537 InterPro:IPR012675 InterPro:IPR005107 Gene3D:G3DSA:1.10.150.120 Gene3D:G3DSA:3.30.365.10 Gene3D:G3DSA:3.90.1170.50 Gene3D:G3DSA:3.30.390.50 Gene3D:G3DSA:3.30.465.10 Gene3D:G3DSA:3.10.20.30 Pfam:PF03450 SMART:SM01008 SMART:SM01092 SUPFAM:SSF47741 SUPFAM:SSF56003 SUPFAM:SSF54665 SUPFAM:SSF55447 SUPFAM:SSF54292 HOGENOM:HBG746468 ProtClustDB:PLN00192 EMBL:AB005805 EMBL:AL391734 EMBL:AF039896 IPI:IPI00529435 PIR:T51623 PIR:T52050 RefSeq:NP_189946.1 UniGene:At.462 ProteinModelPortal:Q7G192 SMR:Q7G192 STRING:Q7G192 PRIDE:Q7G192 EnsemblPlants:AT3G43600.1 GeneID:823457 KEGG:ath:AT3G43600 GeneFarm:4893 TAIR:At3g43600 InParanoid:Q7G192 OMA:ILYDCGK PhylomeDB:Q7G192 BioCyc:ARA:AT3G43600-MONOMER BioCyc:MetaCyc:AT3G43600-MONOMER Genevestigator:Q7G192 GermOnline:AT3G43600 GO:GO:0016614 Uniprot:Q7G192
Root	Isotig00378	24	73	-1.734	3.78E-08	TAIR|locus:2079834 - symbol:AAO2 "aldehyde oxidase 2" species:3702 "Arabidopsis thaliana" [GO:0055114 "oxidation-reduction process" evidence=IEA] [GO:0004031 "aldehyde oxidase activity" evidence=IDA] [GO:0005829 "cytosol" evidence=IDA] InterPro:IPR001041 InterPro:IPR002346 InterPro:IPR002888 InterPro:IPR006058 InterPro:IPR008274 InterPro:IPR016166 InterPro:IPR016169 InterPro:IPR016208 Pfam:PF00111 Pfam:PF00941 Pfam:PF01799 Pfam:PF02738 PIRSF:PIRSF000127 PROSITE:PS00197 PROSITE:PS00559 PROSITE:PS51085 PROSITE:PS51387 InterPro:IPR000674 Pfam:PF01315 GO:GO:0005829 EMBL:CP002686 GenomeReviews:BA000014_GR GO:GO:0005506 GO:GO:0009055 GO:GO:0050660 SUPFAM:SSF56176 GO:GO:0004031 eggNOG:COG4630 GO:GO:0051537 InterPro:IPR012675 InterPro:IPR005107 Gene3D:G3DSA:1.10.150.120 Gene3D:G3DSA:3.30.365.10 Gene3D:G3DSA:3.90.1170.50 Gene3D:G3DSA:3.30.390.50 Gene3D:G3DSA:3.30.465.10 Gene3D:G3DSA:3.10.20.30 Pfam:PF03450 SMART:SM01008 SMART:SM01092 SUPFAM:SSF47741 SUPFAM:SSF56003 SUPFAM:SSF54665 SUPFAM:SSF55447 SUPFAM:SSF54292 HOGENOM:HBG746468 ProtClustDB:PLN00192 EMBL:AB005805 EMBL:AL391734 EMBL:AF039896 IPI:IPI00529435 PIR:T51623 PIR:T52050 RefSeq:NP_189946.1 UniGene:At.462 ProteinModelPortal:Q7G192 SMR:Q7G192 STRING:Q7G192 PRIDE:Q7G192 EnsemblPlants:AT3G43600.1 GeneID:823457 KEGG:ath:AT3G43600 GeneFarm:4893 TAIR:At3g43600 InParanoid:Q7G192 OMA:ILYDCGK PhylomeDB:Q7G192 BioCyc:ARA:AT3G43600-MONOMER BioCyc:MetaCyc:AT3G43600-MONOMER Genevestigator:Q7G192 GermOnline:AT3G43600 GO:GO:0016614 Uniprot:Q7G192
Root	Isotig00379	19	79	-2.185	2.58E-11	TAIR|locus:2079834 - symbol:AAO2 "aldehyde oxidase 2" species:3702 "Arabidopsis thaliana" [GO:0055114 "oxidation-reduction process" evidence=IEA] [GO:0004031 "aldehyde oxidase activity" evidence=IDA] [GO:0005829 "cytosol" evidence=IDA] InterPro:IPR001041 InterPro:IPR002346 InterPro:IPR002888 InterPro:IPR006058 InterPro:IPR008274 InterPro:IPR016166 InterPro:IPR016169 InterPro:IPR016208 Pfam:PF00111 Pfam:PF00941 Pfam:PF01799 Pfam:PF02738 PIRSF:PIRSF000127 PROSITE:PS00197 PROSITE:PS00559 PROSITE:PS51085 PROSITE:PS51387 InterPro:IPR000674 Pfam:PF01315 GO:GO:0005829 EMBL:CP002686 GenomeReviews:BA000014_GR GO:GO:0005506 GO:GO:0009055 GO:GO:0050660 SUPFAM:SSF56176 GO:GO:0004031 eggNOG:COG4630 GO:GO:0051537 InterPro:IPR012675 InterPro:IPR005107 Gene3D:G3DSA:1.10.150.120 Gene3D:G3DSA:3.30.365.10 Gene3D:G3DSA:3.90.1170.50 Gene3D:G3DSA:3.30.390.50 Gene3D:G3DSA:3.30.465.10 Gene3D:G3DSA:3.10.20.30 Pfam:PF03450 SMART:SM01008 SMART:SM01092 SUPFAM:SSF47741 SUPFAM:SSF56003 SUPFAM:SSF54665 SUPFAM:SSF55447 SUPFAM:SSF54292 HOGENOM:HBG746468 ProtClustDB:PLN00192 EMBL:AB005805 EMBL:AL391734 EMBL:AF039896 IPI:IPI00529435 PIR:T51623 PIR:T52050 RefSeq:NP_189946.1 UniGene:At.462 ProteinModelPortal:Q7G192 SMR:Q7G192 STRING:Q7G192 PRIDE:Q7G192 EnsemblPlants:AT3G43600.1 GeneID:823457 KEGG:ath:AT3G43600 GeneFarm:4893 TAIR:At3g43600 InParanoid:Q7G192 OMA:ILYDCGK PhylomeDB:Q7G192 BioCyc:ARA:AT3G43600-MONOMER BioCyc:MetaCyc:AT3G43600-MONOMER Genevestigator:Q7G192 GermOnline:AT3G43600 GO:GO:0016614 Uniprot:Q7G192
Root	Isotig00380	19	79	-2.185	2.58E-11	TAIR|locus:2079834 - symbol:AAO2 "aldehyde oxidase 2" species:3702 "Arabidopsis thaliana" [GO:0055114 "oxidation-reduction process" evidence=IEA] [GO:0004031 "aldehyde oxidase activity" evidence=IDA] [GO:0005829 "cytosol" evidence=IDA] InterPro:IPR001041 InterPro:IPR002346 InterPro:IPR002888 InterPro:IPR006058 InterPro:IPR008274 InterPro:IPR016166 InterPro:IPR016169 InterPro:IPR016208 Pfam:PF00111 Pfam:PF00941 Pfam:PF01799 Pfam:PF02738 PIRSF:PIRSF000127 PROSITE:PS00197 PROSITE:PS00559 PROSITE:PS51085 PROSITE:PS51387 InterPro:IPR000674 Pfam:PF01315 GO:GO:0005829 EMBL:CP002686

Tissue	Identification	# of reads (stress)	# of reads (control)	Fold Change	p-value	Description
						GenomeReviews:BA000014_GR GO:GO:0005506 GO:GO:0009055 GO:GO:0050660 SUPFAM:SSF56176 GO:GO:0004031 eggNOG:COG4630 GO:GO:0051537 InterPro:IPR012675 InterPro:IPR005107 Gene3D:G3DSA:1.10.150.120 Gene3D:G3DSA:3.30.365.10 Gene3D:G3DSA:3.90.1170.50 Gene3D:G3DSA:3.30.390.50 Gene3D:G3DSA:3.30.465.10 Gene3D:G3DSA:3.10.20.30 Pfam:PF03450 SMART:SM01008 SMART:SM01092 SUPFAM:SSF47741 SUPFAM:SSF56003 SUPFAM:SSF54665 SUPFAM:SSF55447 SUPFAM:SSF54292 HOGENOM:HBG746468 ProtClustDB:PLN00192 EMBL:AB005805 EMBL:AL391734 EMBL:AF039896 IPI:IPI00529435 PIR:T51623 PIR:T52050 RefSeq:NP_189946.1 UniGene:At.462 ProteinModelPortal:Q7G192 SMR:Q7G192 STRING:Q7G192 PRIDE:Q7G192 EnsemblPlants:AT3G43600.1 GeneID:823457 KEGG:ath:AT3G43600 GeneFarm:4893 TAIR:At3g43600 InParanoid:Q7G192 OMA:ILYDCGK PhylomeDB:Q7G192 BioCyc:ARA:AT3G43600-MONOMER BioCyc:MetaCyc:AT3G43600-MONOMER Genevestigator:Q7G192 GermOnline:AT3G43600 GO:GO:0016614 Uniprot:Q7G192
Root	Isotig00381	19	79	-2.185	2.58E-11	TAIR|locus:2079834 - symbol:AAO2 "aldehyde oxidase 2" species:3702 "Arabidopsis thaliana" [GO:0055114 "oxidation-reduction process" evidence=IEA] [GO:0004031 "aldehyde oxidase activity" evidence=IDA] [GO:0005829 "cytosol" evidence=IDA] InterPro:IPR001041 InterPro:IPR002346 InterPro:IPR002888 InterPro:IPR006058 InterPro:IPR008274 InterPro:IPR016166 InterPro:IPR016169 InterPro:IPR016208 Pfam:PF00111 Pfam:PF00941 Pfam:PF01799 Pfam:PF02738 PIRSF:PIRSF000127 PROSITE:PS00197 PROSITE:PS00559 PROSITE:PS51085 PROSITE:PS51387 InterPro:IPR000674 Pfam:PF01315 GO:GO:0005829 EMBL:CP002686 GenomeReviews:BA000014_GR GO:GO:0005506 GO:GO:0009055 GO:GO:0050660 SUPFAM:SSF56176 GO:GO:0004031 eggNOG:COG4630 GO:GO:0051537 InterPro:IPR012675 InterPro:IPR005107 Gene3D:G3DSA:1.10.150.120 Gene3D:G3DSA:3.30.365.10 Gene3D:G3DSA:3.90.1170.50 Gene3D:G3DSA:3.30.390.50 Gene3D:G3DSA:3.30.465.10 Gene3D:G3DSA:3.10.20.30 Pfam:PF03450 SMART:SM01008 SMART:SM01092 SUPFAM:SSF47741 SUPFAM:SSF56003 SUPFAM:SSF54665 SUPFAM:SSF55447 SUPFAM:SSF54292 HOGENOM:HBG746468 ProtClustDB:PLN00192 EMBL:AB005805 EMBL:AL391734 EMBL:AF039896 IPI:IPI00529435 PIR:T51623 PIR:T52050 RefSeq:NP_189946.1 UniGene:At.462 ProteinModelPortal:Q7G192 SMR:Q7G192 STRING:Q7G192 PRIDE:Q7G192 EnsemblPlants:AT3G43600.1 GeneID:823457 KEGG:ath:AT3G43600 GeneFarm:4893 TAIR:At3g43600 InParanoid:Q7G192 OMA:ILYDCGK PhylomeDB:Q7G192 BioCyc:ARA:AT3G43600-MONOMER BioCyc:MetaCyc:AT3G43600-MONOMER Genevestigator:Q7G192 GermOnline:AT3G43600 GO:GO:0016614 Uniprot:Q7G192
Root	Isotig00382	19	77	-2.148	7.31E-11	TAIR|locus:2079834 - symbol:AAO2 "aldehyde oxidase 2" species:3702 "Arabidopsis thaliana" [GO:0055114 "oxidation-reduction process" evidence=IEA] [GO:0004031 "aldehyde oxidase activity" evidence=IDA] [GO:0005829 "cytosol" evidence=IDA] InterPro:IPR001041 InterPro:IPR002346 InterPro:IPR002888 InterPro:IPR006058 InterPro:IPR008274 InterPro:IPR016166 InterPro:IPR016169 InterPro:IPR016208 Pfam:PF00111 Pfam:PF00941 Pfam:PF01799 Pfam:PF02738 PIRSF:PIRSF000127 PROSITE:PS00197 PROSITE:PS00559 PROSITE:PS51085 PROSITE:PS51387 InterPro:IPR000674 Pfam:PF01315 GO:GO:0005829 EMBL:CP002686 GenomeReviews:BA000014_GR GO:GO:0005506 GO:GO:0009055 GO:GO:0050660 SUPFAM:SSF56176 GO:GO:0004031 eggNOG:COG4630 GO:GO:0051537 InterPro:IPR012675 InterPro:IPR005107 Gene3D:G3DSA:1.10.150.120 Gene3D:G3DSA:3.30.365.10 Gene3D:G3DSA:3.90.1170.50 Gene3D:G3DSA:3.30.390.50 Gene3D:G3DSA:3.30.465.10 Gene3D:G3DSA:3.10.20.30 Pfam:PF03450 SMART:SM01008 SMART:SM01092 SUPFAM:SSF47741 SUPFAM:SSF56003 SUPFAM:SSF54665 SUPFAM:SSF55447 SUPFAM:SSF54292 HOGENOM:HBG746468 ProtClustDB:PLN00192 EMBL:AB005805 EMBL:AL391734 EMBL:AF039896 IPI:IPI00529435 PIR:T51623 PIR:T52050 RefSeq:NP_189946.1 UniGene:At.462 ProteinModelPortal:Q7G192 SMR:Q7G192 STRING:Q7G192 PRIDE:Q7G192 EnsemblPlants:AT3G43600.1 GeneID:823457 KEGG:ath:AT3G43600 GeneFarm:4893 TAIR:At3g43600 InParanoid:Q7G192 OMA:ILYDCGK PhylomeDB:Q7G192 BioCyc:ARA:AT3G43600-MONOMER BioCyc:MetaCyc:AT3G43600-MONOMER Genevestigator:Q7G192 GermOnline:AT3G43600 GO:GO:0016614 Uniprot:Q7G192
Root	Isotig00383	19	77	-2.148	7.31E-11	TAIR|locus:2079834 - symbol:AAO2 "aldehyde oxidase 2" species:3702 "Arabidopsis thaliana" [GO:0055114 "oxidation-reduction process" evidence=IEA] [GO:0004031 "aldehyde oxidase activity" evidence=IDA] [GO:0005829 "cytosol" evidence=IDA] InterPro:IPR001041 InterPro:IPR002346 InterPro:IPR002888 InterPro:IPR006058 InterPro:IPR008274 InterPro:IPR016166 InterPro:IPR016169 InterPro:IPR016208 Pfam:PF00111 Pfam:PF00941 Pfam:PF01799 Pfam:PF02738 PIRSF:PIRSF000127 PROSITE:PS00197 PROSITE:PS00559 PROSITE:PS51085 PROSITE:PS51387 InterPro:IPR000674 Pfam:PF01315 GO:GO:0005829 EMBL:CP002686 GenomeReviews:BA000014_GR GO:GO:0005506 GO:GO:0009055 GO:GO:0050660 SUPFAM:SSF56176

Tissue	Identification	# of reads (stress)	# of reads (control)	Fold Change	p-value	Description
						GO:GO:0004031 eggNOG:COG4630 GO:GO:0051537 InterPro:IPR012675 InterPro:IPR005107 Gene3D:G3DSA:1.10.150.120 Gene3D:G3DSA:3.30.365.10 Gene3D:G3DSA:3.90.1170.50 Gene3D:G3DSA:3.30.390.50 Gene3D:G3DSA:3.30.465.10 Gene3D:G3DSA:3.10.20.30 Pfam:PF03450 SMART:SM01008 SMART:SM01092 SUPFAM:SSF47741 SUPFAM:SSF56003 SUPFAM:SSF54665 SUPFAM:SSF55447 SUPFAM:SSF54292 HOGENOM:HBG746468 ProtClustDB:PLN00192 EMBL:AB005805 EMBL:AL391734 EMBL:AF039896 IPI:IPI00529435 PIR:T51623 PIR:T52050 RefSeq:NP_189946.1 UniGene:At.462 ProteinModelPortal:Q7G192 SMR:Q7G192 STRING:Q7G192 PRIDE:Q7G192 EnsemblPlants:AT3G43600.1 GeneID:823457 KEGG:ath:AT3G43600 GeneFarm:4893 TAIR:At3g43600 InParanoid:Q7G192 OMA:ILYDCGK PhylomeDB:Q7G192 BioCyc:ARA:AT3G43600-MONOMER BioCyc:MetaCyc:AT3G43600-MONOMER Genevestigator:Q7G192 GermOnline:AT3G43600 GO:GO:0016614 Uniprot:Q7G192
Root	Isotig00384	19	64	-1.881	5.11E-08	TAIR|locus:2045149 - symbol:AAO3 "AT2G27150" species:3702 "Arabidopsis thaliana" [GO:0004031 "aldehyde oxidase activity" evidence=IDA] [GO:0050302 "indole-3-acetaldehyde oxidase activity" evidence=IDA] [GO:0009688 "abscisic acid biosynthetic process" evidence=IMP] [GO:0010293 "abscisic aldehyde oxidase activity" evidence=IDA] [GO:0031625 "ubiquitin protein ligase binding" evidence=IPI] [GO:0005829 "cytosol" evidence=IDA] InterPro:IPR001041 InterPro:IPR002346 InterPro:IPR002888 InterPro:IPR006058 InterPro:IPR008274 InterPro:IPR016166 InterPro:IPR016169 InterPro:IPR016208 Pfam:PF00111 Pfam:PF00941 Pfam:PF01799 Pfam:PF02738 PIRSF:PIRSF000127 PROSITE:PS00197 PROSITE:PS00559 PROSITE:PS51085 PROSITE:PS51387 InterPro:IPR000674 Pfam:PF01315 GO:GO:0005829 EMBL:CP002685 GenomeReviews:CT485783_GR GO:GO:0005506 GO:GO:0009055 GO:GO:0050660 GO:GO:0009688 GO:GO:0031625 SUPFAM:SSF56176 eggNOG:COG4630 GO:GO:0051537 InterPro:IPR012675 InterPro:IPR005107 Gene3D:G3DSA:1.10.150.120 Gene3D:G3DSA:3.30.365.10 Gene3D:G3DSA:3.90.1170.50 Gene3D:G3DSA:3.30.390.50 Gene3D:G3DSA:3.30.465.10 Gene3D:G3DSA:3.10.20.30 Pfam:PF03450 SMART:SM01008 SMART:SM01092 SUPFAM:SSF47741 SUPFAM:SSF56003 SUPFAM:SSF54665 SUPFAM:SSF55447 SUPFAM:SSF54292 HOGENOM:HBG746468 ProtClustDB:PLN00192 GO:GO:0050302 GO:GO:0009851 GO:GO:0016614 EMBL:AB016622 EMBL:AC007154 EMBL:AB010080 IPI:IPI00517261 PIR:D84669 PIR:T52176 RefSeq:NP_001077966.1 RefSeq:NP_180283.1 UniGene:At.20239 ProteinModelPortal:Q7G9P4 SMR:Q7G9P4 STRING:Q7G9P4 PRIDE:Q7G9P4 EnsemblPlants:AT2G27150.1 EnsemblPlants:AT2G27150.2 GeneID:817257 KEGG:ath:AT2G27150 GeneFarm:4894 TAIR:At2g27150 InParanoid:Q7G9P4 KO:K09842 OMA:ADELTRC PhylomeDB:Q7G9P4 BioCyc:ARA:AT2G27150-MONOMER BioCyc:MetaCyc:AT2G27150-MONOMER BRENDA:1.2.3.14 ArrayExpress:Q7G9P4 Genevestigator:Q7G9P4 GermOnline:AT2G27150 GO:GO:0010293 Uniprot:Q7G9P4
Root	Isotig00385	19	64	-1.881	5.11E-08	TAIR|locus:2045149 - symbol:AAO3 "AT2G27150" species:3702 "Arabidopsis thaliana" [GO:0004031 "aldehyde oxidase activity" evidence=IDA] [GO:0050302 "indole-3-acetaldehyde oxidase activity" evidence=IDA] [GO:0009688 "abscisic acid biosynthetic process" evidence=IMP] [GO:0010293 "abscisic aldehyde oxidase activity" evidence=IDA] [GO:0031625 "ubiquitin protein ligase binding" evidence=IPI] [GO:0005829 "cytosol" evidence=IDA] InterPro:IPR001041 InterPro:IPR002346 InterPro:IPR002888 InterPro:IPR006058 InterPro:IPR008274 InterPro:IPR016166 InterPro:IPR016169 InterPro:IPR016208 Pfam:PF00111 Pfam:PF00941 Pfam:PF01799 Pfam:PF02738 PIRSF:PIRSF000127 PROSITE:PS00197 PROSITE:PS00559 PROSITE:PS51085 PROSITE:PS51387 InterPro:IPR000674 Pfam:PF01315 GO:GO:0005829 EMBL:CP002685 GenomeReviews:CT485783_GR GO:GO:0005506 GO:GO:0009055 GO:GO:0050660 GO:GO:0009688 GO:GO:0031625 SUPFAM:SSF56176 eggNOG:COG4630 GO:GO:0051537 InterPro:IPR012675 InterPro:IPR005107 Gene3D:G3DSA:1.10.150.120 Gene3D:G3DSA:3.30.365.10 Gene3D:G3DSA:3.90.1170.50 Gene3D:G3DSA:3.30.390.50 Gene3D:G3DSA:3.30.465.10 Gene3D:G3DSA:3.10.20.30 Pfam:PF03450 SMART:SM01008 SMART:SM01092 SUPFAM:SSF47741 SUPFAM:SSF56003 SUPFAM:SSF54665 SUPFAM:SSF55447 SUPFAM:SSF54292 HOGENOM:HBG746468 ProtClustDB:PLN00192 GO:GO:0050302 GO:GO:0009851 GO:GO:0016614 EMBL:AB016622 EMBL:AC007154 EMBL:AB010080 IPI:IPI00517261 PIR:D84669 PIR:T52176 RefSeq:NP_001077966.1 RefSeq:NP_180283.1 UniGene:At.20239 ProteinModelPortal:Q7G9P4 SMR:Q7G9P4 STRING:Q7G9P4 PRIDE:Q7G9P4 EnsemblPlants:AT2G27150.1 EnsemblPlants:AT2G27150.2 GeneID:817257 KEGG:ath:AT2G27150 GeneFarm:4894 TAIR:At2g27150 InParanoid:Q7G9P4 KO:K09842 OMA:ADELTRC PhylomeDB:Q7G9P4 BioCyc:ARA:AT2G27150-MONOMER BioCyc:MetaCyc:AT2G27150-MONOMER BRENDA:1.2.3.14 ArrayExpress:Q7G9P4 Genevestigator:Q7G9P4 GermOnline:AT2G27150 GO:GO:0010293 Uniprot:Q7G9P4

Tissue	Identification	# of reads (stress)	# of reads (control)	Fold Change	p-value	Description
Root	Isotig00386	14	70	-2.451	1.76E-11	TAIR|locus:2079834 - symbol:AAO2 "aldehyde oxidase 2" species:3702 "Arabidopsis thaliana" [GO:0055114 "oxidation-reduction process" evidence=IEA] [GO:0004031 "aldehyde oxidase activity" evidence=IDA] [GO:0005829 "cytosol" evidence=IDA] InterPro:IPR001041 InterPro:IPR002346 InterPro:IPR002888 InterPro:IPR006058 InterPro:IPR008274 InterPro:IPR016166 InterPro:IPR016169 InterPro:IPR016208 Pfam:PF00111 Pfam:PF00941 Pfam:PF01799 Pfam:PF02738 PIRSF:PIRSF000127 PROSITE:PS00197 PROSITE:PS00559 PROSITE:PS51085 PROSITE:PS51387 InterPro:IPR000674 Pfam:PF01315 GO:GO:0005829 EMBL:CP002686 GenomeReviews:BA000014_GR GO:GO:0005506 GO:GO:0009055 GO:GO:0050660 SUPFAM:SSF56176 GO:GO:0004031 eggNOG:COG4630 GO:GO:0051537 InterPro:IPR012675 InterPro:IPR005107 Gene3D:G3DSA:1.10.150.120 Gene3D:G3DSA:3.30.365.10 Gene3D:G3DSA:3.90.1170.50 Gene3D:G3DSA:3.30.390.50 Gene3D:G3DSA:3.30.465.10 Gene3D:G3DSA:3.10.20.30 Pfam:PF03450 SMART:SM01008 SMART:SM01092 SUPFAM:SSF47741 SUPFAM:SSF56003 SUPFAM:SSF54665 SUPFAM:SSF55447 SUPFAM:SSF54292 HOGENOM:HBG746468 ProtClustDB:PLN00192 EMBL:AB005805 EMBL:AL391734 EMBL:AF039896 IPI:IPI00529435 PIR:T51623 PIR:T52050 RefSeq:NP_189946.1 UniGene:At.462 ProteinModelPortal:Q7G192 SMR:Q7G192 STRING:Q7G192 PRIDE:Q7G192 EnsemblPlants:AT3G43600.1 GeneID:823457 KEGG:ath:AT3G43600 GeneFarm:4893 TAIR:At3g43600 InParanoid:Q7G192 OMA:ILYDCGK PhylomeDB:Q7G192 BioCyc:ARA:AT3G43600-MONOMER BioCyc:MetaCyc:AT3G43600-MONOMER Genevestigator:Q7G192 GermOnline:AT3G43600 GO:GO:0016614 Uniprot:Q7G192
Root	Isotig00387	19	64	-1.881	5.11E-08	TAIR|locus:2045149 - symbol:AAO3 "AT2G27150" species:3702 "Arabidopsis thaliana" [GO:0004031 "aldehyde oxidase activity" evidence=IDA] [GO:0050302 "indole-3-acetaldehyde oxidase activity" evidence=IDA] [GO:0009688 "abscisic acid biosynthetic process" evidence=IMP] [GO:0010293 "abscisic aldehyde oxidase activity" evidence=IDA] [GO:0031625 "ubiquitin protein ligase binding" evidence=IPI] [GO:0005829 "cytosol" evidence=IDA] InterPro:IPR001041 InterPro:IPR002346 InterPro:IPR002888 InterPro:IPR006058 InterPro:IPR008274 InterPro:IPR016166 InterPro:IPR016169 InterPro:IPR016208 Pfam:PF00111 Pfam:PF00941 Pfam:PF01799 Pfam:PF02738 PIRSF:PIRSF000127 PROSITE:PS00197 PROSITE:PS00559 PROSITE:PS51085 PROSITE:PS51387 InterPro:IPR000674 Pfam:PF01315 GO:GO:0005829 EMBL:CP002685 GenomeReviews:CT485783_GR GO:GO:0005506 GO:GO:0009055 GO:GO:0050660 GO:GO:0009688 GO:GO:0031625 SUPFAM:SSF56176 eggNOG:COG4630 GO:GO:0051537 InterPro:IPR012675 InterPro:IPR005107 Gene3D:G3DSA:1.10.150.120 Gene3D:G3DSA:3.30.365.10 Gene3D:G3DSA:3.90.1170.50 Gene3D:G3DSA:3.30.390.50 Gene3D:G3DSA:3.30.465.10 Gene3D:G3DSA:3.10.20.30 Pfam:PF03450 SMART:SM01008 SMART:SM01092 SUPFAM:SSF47741 SUPFAM:SSF56003 SUPFAM:SSF54665 SUPFAM:SSF55447 SUPFAM:SSF54292 HOGENOM:HBG746468 ProtClustDB:PLN00192 GO:GO:0050302 GO:GO:0009851 GO:GO:0016614 EMBL:AB016622 EMBL:AC007154 EMBL:AB010080 IPI:IPI00517261 PIR:D84669 PIR:T52176 RefSeq:NP_001077966.1 RefSeq:NP_180283.1 UniGene:At.20239 ProteinModelPortal:Q7G9P4 SMR:Q7G9P4 STRING:Q7G9P4 PRIDE:Q7G9P4 EnsemblPlants:AT2G27150.1 EnsemblPlants:AT2G27150.2 GeneID:817257 KEGG:ath:AT2G27150 GeneFarm:4894 TAIR:At2g27150 InParanoid:Q7G9P4 KO:K09842 OMA:ADELTRC PhylomeDB:Q7G9P4 BioCyc:ARA:AT2G27150-MONOMER BioCyc:MetaCyc:AT2G27150-MONOMER BRENDA:1.2.3.14 ArrayExpress:Q7G9P4 Genevestigator:Q7G9P4 GermOnline:AT2G27150 GO:GO:0010293 Uniprot:Q7G9P4
Root	Isotig00388	19	64	-1.881	5.11E-08	TAIR|locus:2045149 - symbol:AAO3 "AT2G27150" species:3702 "Arabidopsis thaliana" [GO:0004031 "aldehyde oxidase activity" evidence=IDA] [GO:0050302 "indole-3-acetaldehyde oxidase activity" evidence=IDA] [GO:0009688 "abscisic acid biosynthetic process" evidence=IMP] [GO:0010293 "abscisic aldehyde oxidase activity" evidence=IDA] [GO:0031625 "ubiquitin protein ligase binding" evidence=IPI] [GO:0005829 "cytosol" evidence=IDA] InterPro:IPR001041 InterPro:IPR002346 InterPro:IPR002888 InterPro:IPR006058 InterPro:IPR008274 InterPro:IPR016166 InterPro:IPR016169 InterPro:IPR016208 Pfam:PF00111 Pfam:PF00941 Pfam:PF01799 Pfam:PF02738 PIRSF:PIRSF000127 PROSITE:PS00197 PROSITE:PS00559 PROSITE:PS51085 PROSITE:PS51387 InterPro:IPR000674 Pfam:PF01315 GO:GO:0005829 EMBL:CP002685 GenomeReviews:CT485783_GR GO:GO:0005506 GO:GO:0009055 GO:GO:0050660 GO:GO:0009688 GO:GO:0031625 SUPFAM:SSF56176 eggNOG:COG4630 GO:GO:0051537 InterPro:IPR012675 InterPro:IPR005107 Gene3D:G3DSA:1.10.150.120 Gene3D:G3DSA:3.30.365.10 Gene3D:G3DSA:3.90.1170.50 Gene3D:G3DSA:3.30.390.50 Gene3D:G3DSA:3.30.465.10 Gene3D:G3DSA:3.10.20.30 Pfam:PF03450 SMART:SM01008 SMART:SM01092 SUPFAM:SSF47741 SUPFAM:SSF56003 SUPFAM:SSF54665 SUPFAM:SSF55447 SUPFAM:SSF54292 HOGENOM:HBG746468 ProtClustDB:PLN00192 GO:GO:0050302 GO:GO:0009851 GO:GO:0016614 EMBL:AB016622 EMBL:AC007154 EMBL:AB010080 IPI:IPI00517261

Tissue	Identification	# of reads (stress)	# of reads (control)	Fold Change	p-value	Description
						PIR:D84669 PIR:T52176 RefSeq:NP_001077966.1 RefSeq:NP_180283.1 UniGene:At.20239 ProteinModelPortal:Q7G9P4 SMR:Q7G9P4 STRING:Q7G9P4 PRIDE:Q7G9P4 EnsemblPlants:AT2G27150.1 EnsemblPlants:AT2G27150.2 GeneID:817257 KEGG:ath:AT2G27150 GeneFarm:4894 TAIR:At2g27150 InParanoid:Q7G9P4 KO:K09842 OMA:ADELTRC PhylomeDB:Q7G9P4 BioCyc:ARA:AT2G27150-MONOMER BioCyc:MetaCyc:AT2G27150-MONOMER BRENDA:1.2.3.14 ArrayExpress:Q7G9P4 Genevestigator:Q7G9P4 GermOnline:AT2G27150 GO:GO:0010293 Uniprot:Q7G9P4
Root	Isotig00389	14	70	-2.451	1.76E-11	TAIR|locus:2079834 - symbol:AAO2 "aldehyde oxidase 2" species:3702 "Arabidopsis thaliana" [GO:0055114 "oxidation-reduction process" evidence=IEA] [GO:0004031 "aldehyde oxidase activity" evidence=IDA] [GO:0005829 "cytosol" evidence=IDA] InterPro:IPR001041 InterPro:IPR002346 InterPro:IPR002888 InterPro:IPR006058 InterPro:IPR008274 InterPro:IPR016166 InterPro:IPR016169 InterPro:IPR016208 Pfam:PF00111 Pfam:PF00941 Pfam:PF01799 Pfam:PF02738 PIRSF:PIRSF000127 PROSITE:PS00197 PROSITE:PS00559 PROSITE:PS51085 PROSITE:PS51387 InterPro:IPR000674 Pfam:PF01315 GO:GO:0005829 EMBL:CP002686 GenomeReviews:BA000014_GR GO:GO:0005506 GO:GO:0009055 GO:GO:0050660 SUPFAM:SSF56176 GO:GO:0004031 eggNOG:COG4630 GO:GO:0051537 InterPro:IPR012675 InterPro:IPR005107 Gene3D:G3DSA:1.10.150.120 Gene3D:G3DSA:3.30.365.10 Gene3D:G3DSA:3.90.1170.50 Gene3D:G3DSA:3.30.390.50 Gene3D:G3DSA:3.30.465.10 Gene3D:G3DSA:3.10.20.30 Pfam:PF03450 SMART:SM01008 SMART:SM01092 SUPFAM:SSF47741 SUPFAM:SSF56003 SUPFAM:SSF54665 SUPFAM:SSF55447 SUPFAM:SSF54292 HOGENOM:HBG746468 ProtClustDB:PLN00192 EMBL:AB005805 EMBL:AL391734 EMBL:AF039896 IPI:IPI00529435 PIR:T51623 PIR:T52050 RefSeq:NP_189946.1 UniGene:At.462 ProteinModelPortal:Q7G192 SMR:Q7G192 STRING:Q7G192 PRIDE:Q7G192 EnsemblPlants:AT3G43600.1 GeneID:823457 KEGG:ath:AT3G43600 GeneFarm:4893 TAIR:At3g43600 InParanoid:Q7G192 OMA:ILYDCGK PhylomeDB:Q7G192 BioCyc:ARA:AT3G43600-MONOMER BioCyc:MetaCyc:AT3G43600-MONOMER Genevestigator:Q7G192 GermOnline:AT3G43600 GO:GO:0016614 Uniprot:Q7G192
Root	Isotig00390	14	70	-2.451	1.76E-11	TAIR|locus:2147127 - symbol:AO1 "AT5G20960" species:3702 "Arabidopsis thaliana" [GO:0004031 "aldehyde oxidase activity" evidence=IDA] [GO:0050302 "indole-3-acetaldehyde oxidase activity" evidence=IDA] [GO:0009851 "auxin biosynthetic process" evidence=IMP] InterPro:IPR001041 InterPro:IPR002346 InterPro:IPR002888 InterPro:IPR006058 InterPro:IPR008274 InterPro:IPR016166 InterPro:IPR016167 InterPro:IPR016169 InterPro:IPR016208 Pfam:PF00111 Pfam:PF00941 Pfam:PF01799 Pfam:PF02738 PIRSF:PIRSF000127 PROSITE:PS00197 PROSITE:PS00559 PROSITE:PS51085 PROSITE:PS51387 InterPro:IPR000674 Pfam:PF01315 EMBL:CP002688 GenomeReviews:BA000015_GR GO:GO:0005506 GO:GO:0009055 GO:GO:0050660 GO:GO:0009688 GO:GO:0008762 Gene3D:G3DSA:3.30.43.10 SUPFAM:SSF56176 eggNOG:COG4630 GO:GO:0051537 InterPro:IPR012675 InterPro:IPR005107 Gene3D:G3DSA:1.10.150.120 Gene3D:G3DSA:3.30.365.10 Gene3D:G3DSA:3.90.1170.50 Gene3D:G3DSA:3.30.390.50 Gene3D:G3DSA:3.30.465.10 Gene3D:G3DSA:3.10.20.30 Pfam:PF03450 SMART:SM01008 SMART:SM01092 SUPFAM:SSF47741 SUPFAM:SSF56003 SUPFAM:SSF54665 SUPFAM:SSF55447 SUPFAM:SSF54292 EMBL:AF296834 EMBL:AF039895 EMBL:AB005804 IPI:IPI00532267 PIR:T51622 PIR:T52049 RefSeq:NP_568407.2 RefSeq:NP_851049.1 UniGene:At.19954 ProteinModelPortal:Q7G193 SMR:Q7G193 IntAct:Q7G193 STRING:Q7G193 PRIDE:Q7G193 EnsemblPlants:AT5G20960.1 EnsemblPlants:AT5G20960.2 GeneID:832221 KEGG:ath:AT5G20960 GeneFarm:4892 TAIR:At5g20960 HOGENOM:HBG746468 InParanoid:Q7G193 KO:K11817 OMA:ADSQKHA PhylomeDB:Q7G193 ProtClustDB:PLN00192 Genevestigator:Q7G193 GermOnline:AT5G20960 GO:GO:0050302 GO:GO:0009851 Uniprot:Q7G193
Root	Isotig00391	14	70	-2.451	1.76E-11	TAIR|locus:2147127 - symbol:AO1 "AT5G20960" species:3702 "Arabidopsis thaliana" [GO:0004031 "aldehyde oxidase activity" evidence=IDA] [GO:0050302 "indole-3-acetaldehyde oxidase activity" evidence=IDA] [GO:0009851 "auxin biosynthetic process" evidence=IMP] InterPro:IPR001041 InterPro:IPR002346 InterPro:IPR002888 InterPro:IPR006058 InterPro:IPR008274 InterPro:IPR016166 InterPro:IPR016167 InterPro:IPR016169 InterPro:IPR016208 Pfam:PF00111 Pfam:PF00941 Pfam:PF01799 Pfam:PF02738 PIRSF:PIRSF000127 PROSITE:PS00197 PROSITE:PS00559 PROSITE:PS51085 PROSITE:PS51387 InterPro:IPR000674 Pfam:PF01315 EMBL:CP002688 GenomeReviews:BA000015_GR GO:GO:0005506 GO:GO:0009055 GO:GO:0050660 GO:GO:0009688 GO:GO:0008762 Gene3D:G3DSA:3.30.43.10 SUPFAM:SSF56176 eggNOG:COG4630 GO:GO:0051537 InterPro:IPR012675 InterPro:IPR005107 Gene3D:G3DSA:1.10.150.120 Gene3D:G3DSA:3.30.365.10 Gene3D:G3DSA:3.90.1170.50 Gene3D:G3DSA:3.30.390.50 Gene3D:G3DSA:3.30.465.10 Gene3D:G3DSA:3.10.20.30 Pfam:PF03450 SMART:SM01008 SMART:SM01092

Tissue	Identification	# of reads (stress)	# of reads (control)	Fold Change	p-value	Description
						SUPFAM:SSF47741 SUPFAM:SSF56003 SUPFAM:SSF54665 SUPFAM:SSF55447 SUPFAM:SSF54292 EMBL:AF296834 EMBL:AF039895 EMBL:AB005804 IPI:IPI00532267 PIR:T51622 PIR:T52049 RefSeq:NP_568407.2 RefSeq:NP_851049.1 UniGene:At.19954 ProteinModelPortal:Q7G193 SMR:Q7G193 IntAct:Q7G193 STRING:Q7G193 PRIDE:Q7G193 EnsemblPlants:AT5G20960.1 EnsemblPlants:AT5G20960.2 GeneID:832221 KEGG:ath:AT5G20960 GeneFarm:4892 TAIR:At5g20960 HOGENOM:HBG746468 InParanoid:Q7G193 KO:K11817 OMA:ADSQKHA PhylomeDB:Q7G193 ProtClustDB:PLN00192 Genevestigator:Q7G193 GermOnline:AT5G20960 GO:GO:0050302 GO:GO:0009851 Uniprot:Q7G193
Root	Isotig00392	19	72	-2.051	9.54E-10	TAIR|locus:2147127 - symbol:AO1 "AT5G20960" species:3702 "Arabidopsis thaliana" [GO:0004031 "aldehyde oxidase activity" evidence=IDA] [GO:0050302 "indole-3-acetaldehyde oxidase activity" evidence=IDA] [GO:0009851 "auxin biosynthetic process" evidence=IMP] InterPro:IPR001041 InterPro:IPR002346 InterPro:IPR002888 InterPro:IPR006058 InterPro:IPR008274 InterPro:IPR016166 InterPro:IPR016167 InterPro:IPR016169 InterPro:IPR016208 Pfam:PF00111 Pfam:PF00941 Pfam:PF01799 Pfam:PF02738 PIRSF:PIRSF000127 PROSITE:PS00197 PROSITE:PS00559 PROSITE:PS51085 PROSITE:PS51387 InterPro:IPR000674 Pfam:PF01315 EMBL:CP002688 GenomeReviews:BA000015_GR GO:GO:0005506 GO:GO:0009055 GO:GO:0050660 GO:GO:0009688 GO:GO:0008762 Gene3D:G3DSA:3.30.43.10 SUPFAM:SSF56176 eggNOG:COG4630 GO:GO:0051537 InterPro:IPR012675 InterPro:IPR005107 Gene3D:G3DSA:1.10.150.120 Gene3D:G3DSA:3.30.365.10 Gene3D:G3DSA:3.90.1170.50 Gene3D:G3DSA:3.30.390.50 Gene3D:G3DSA:3.30.465.10 Gene3D:G3DSA:3.10.20.30 Pfam:PF03450 SMART:SM01008 SMART:SM01092 SUPFAM:SSF47741 SUPFAM:SSF56003 SUPFAM:SSF54665 SUPFAM:SSF55447 SUPFAM:SSF54292 EMBL:AF296834 EMBL:AF039895 EMBL:AB005804 IPI:IPI00532267 PIR:T51622 PIR:T52049 RefSeq:NP_568407.2 RefSeq:NP_851049.1 UniGene:At.19954 ProteinModelPortal:Q7G193 SMR:Q7G193 IntAct:Q7G193 STRING:Q7G193 PRIDE:Q7G193 EnsemblPlants:AT5G20960.1 EnsemblPlants:AT5G20960.2 GeneID:832221 KEGG:ath:AT5G20960 GeneFarm:4892 TAIR:At5g20960 HOGENOM:HBG746468 InParanoid:Q7G193 KO:K11817 OMA:ADSQKHA PhylomeDB:Q7G193 ProtClustDB:PLN00192 Genevestigator:Q7G193 GermOnline:AT5G20960 GO:GO:0050302 GO:GO:0009851 Uniprot:Q7G193
Root	Isotig00393	19	72	-2.051	9.54E-10	TAIR|locus:2147127 - symbol:AO1 "AT5G20960" species:3702 "Arabidopsis thaliana" [GO:0004031 "aldehyde oxidase activity" evidence=IDA] [GO:0050302 "indole-3-acetaldehyde oxidase activity" evidence=IDA] [GO:0009851 "auxin biosynthetic process" evidence=IMP] InterPro:IPR001041 InterPro:IPR002346 InterPro:IPR002888 InterPro:IPR006058 InterPro:IPR008274 InterPro:IPR016166 InterPro:IPR016167 InterPro:IPR016169 InterPro:IPR016208 Pfam:PF00111 Pfam:PF00941 Pfam:PF01799 Pfam:PF02738 PIRSF:PIRSF000127 PROSITE:PS00197 PROSITE:PS00559 PROSITE:PS51085 PROSITE:PS51387 InterPro:IPR000674 Pfam:PF01315 EMBL:CP002688 GenomeReviews:BA000015_GR GO:GO:0005506 GO:GO:0009055 GO:GO:0050660 GO:GO:0009688 GO:GO:0008762 Gene3D:G3DSA:3.30.43.10 SUPFAM:SSF56176 eggNOG:COG4630 GO:GO:0051537 InterPro:IPR012675 InterPro:IPR005107 Gene3D:G3DSA:1.10.150.120 Gene3D:G3DSA:3.30.365.10 Gene3D:G3DSA:3.90.1170.50 Gene3D:G3DSA:3.30.390.50 Gene3D:G3DSA:3.30.465.10 Gene3D:G3DSA:3.10.20.30 Pfam:PF03450 SMART:SM01008 SMART:SM01092 SUPFAM:SSF47741 SUPFAM:SSF56003 SUPFAM:SSF54665 SUPFAM:SSF55447 SUPFAM:SSF54292 EMBL:AF296834 EMBL:AF039895 EMBL:AB005804 IPI:IPI00532267 PIR:T51622 PIR:T52049 RefSeq:NP_568407.2 RefSeq:NP_851049.1 UniGene:At.19954 ProteinModelPortal:Q7G193 SMR:Q7G193 IntAct:Q7G193 STRING:Q7G193 PRIDE:Q7G193 EnsemblPlants:AT5G20960.1 EnsemblPlants:AT5G20960.2 GeneID:832221 KEGG:ath:AT5G20960 GeneFarm:4892 TAIR:At5g20960 HOGENOM:HBG746468 InParanoid:Q7G193 KO:K11817 OMA:ADSQKHA PhylomeDB:Q7G193 ProtClustDB:PLN00192 Genevestigator:Q7G193 GermOnline:AT5G20960 GO:GO:0050302 GO:GO:0009851 Uniprot:Q7G193
Root	Isotig00394	14	63	-2.299	8.06E-10	TAIR|locus:2079834 - symbol:AAO2 "aldehyde oxidase 2" species:3702 "Arabidopsis thaliana" [GO:0055114 "oxidation-reduction process" evidence=IEA] [GO:0004031 "aldehyde oxidase activity" evidence=IDA] [GO:0005829 "cytosol" evidence=IDA] InterPro:IPR001041 InterPro:IPR002346 InterPro:IPR002888 InterPro:IPR006058 InterPro:IPR008274 InterPro:IPR016166 InterPro:IPR016169 InterPro:IPR016208 Pfam:PF00111 Pfam:PF00941 Pfam:PF01799 Pfam:PF02738 PIRSF:PIRSF000127 PROSITE:PS00197 PROSITE:PS00559 PROSITE:PS51085 PROSITE:PS51387 InterPro:IPR000674 Pfam:PF01315 GO:GO:0005829 EMBL:CP002686 GenomeReviews:BA000014_GR GO:GO:0005506 GO:GO:0009055 GO:GO:0050660 SUPFAM:SSF56176 GO:GO:0004031 eggNOG:COG4630 GO:GO:0051537 InterPro:IPR012675 InterPro:IPR005107 Gene3D:G3DSA:1.10.150.120 Gene3D:G3DSA:3.30.365.10 Gene3D:G3DSA:3.90.1170.50

Tissue	Identification	# of reads (stress)	# of reads (control)	Fold Change	p-value	Description
						Gene3D:G3DSA:3.30.390.50 Gene3D:G3DSA:3.30.465.10 Gene3D:G3DSA:3.10.20.30 Pfam:PF03450 SMART:SM01008 SMART:SM01092 SUPFAM:SSF47741 SUPFAM:SSF56003 SUPFAM:SSF54665 SUPFAM:SSF55447 SUPFAM:SSF54292 HOGENOM:HBG746468 ProtClustDB:PLN00192 EMBL:AB005805 EMBL:AL391734 EMBL:AF039896 IPI:IPI00529435 PIR:T51623 PIR:T52050 RefSeq:NP_189946.1 UniGene:At.462 ProteinModelPortal:Q7G192 SMR:Q7G192 STRING:Q7G192 PRIDE:Q7G192 EnsemblPlants:AT3G43600.1 GeneID:823457 KEGG:ath:AT3G43600 GeneFarm:4893 TAIR:At3g43600 InParanoid:Q7G192 OMA:ILYDCGK PhylomeDB:Q7G192 BioCyc:ARA:AT3G43600-MONOMER BioCyc:MetaCyc:AT3G43600-MONOMER Genevestigator:Q7G192 GermOnline:AT3G43600 GO:GO:0016614 Uniprot:Q7G192
Root	Isotig00395	14	63	-2.299	8.06E-10	TAIR|locus:2079834 - symbol:AAO2 "aldehyde oxidase 2" species:3702 "Arabidopsis thaliana" [GO:0055114 "oxidation-reduction process" evidence=IEA] [GO:0004031 "aldehyde oxidase activity" evidence=IDA] [GO:0005829 "cytosol" evidence=IDA] InterPro:IPR001041 InterPro:IPR002346 InterPro:IPR002888 InterPro:IPR006058 InterPro:IPR008274 InterPro:IPR016166 InterPro:IPR016169 InterPro:IPR016208 Pfam:PF00111 Pfam:PF00941 Pfam:PF01799 Pfam:PF02738 PIRSF:PIRSF000127 PROSITE:PS00197 PROSITE:PS00559 PROSITE:PS51085 PROSITE:PS51387 InterPro:IPR000674 Pfam:PF01315 GO:GO:0005829 EMBL:CP002686 GenomeReviews:BA000014_GR GO:GO:0005506 GO:GO:0009055 GO:GO:0050660 SUPFAM:SSF56176 GO:GO:0004031 eggNOG:COG4630 GO:GO:0051537 InterPro:IPR012675 InterPro:IPR005107 Gene3D:G3DSA:1.10.150.120 Gene3D:G3DSA:3.30.365.10 Gene3D:G3DSA:3.90.1170.50 Gene3D:G3DSA:3.30.390.50 Gene3D:G3DSA:3.30.465.10 Gene3D:G3DSA:3.10.20.30 Pfam:PF03450 SMART:SM01008 SMART:SM01092 SUPFAM:SSF47741 SUPFAM:SSF56003 SUPFAM:SSF54665 SUPFAM:SSF55447 SUPFAM:SSF54292 HOGENOM:HBG746468 ProtClustDB:PLN00192 EMBL:AB005805 EMBL:AL391734 EMBL:AF039896 IPI:IPI00529435 PIR:T51623 PIR:T52050 RefSeq:NP_189946.1 UniGene:At.462 ProteinModelPortal:Q7G192 SMR:Q7G192 STRING:Q7G192 PRIDE:Q7G192 EnsemblPlants:AT3G43600.1 GeneID:823457 KEGG:ath:AT3G43600 GeneFarm:4893 TAIR:At3g43600 InParanoid:Q7G192 OMA:ILYDCGK PhylomeDB:Q7G192 BioCyc:ARA:AT3G43600-MONOMER BioCyc:MetaCyc:AT3G43600-MONOMER Genevestigator:Q7G192 GermOnline:AT3G43600 GO:GO:0016614 Uniprot:Q7G192
Root	Isotig00411	191	13	3.748	1.70E-38	No hit
Root	Isotig00412	193	13	3.763	5.51E-39	No hit
Root	Isotig00413	191	13	3.748	1.70E-38	No hit
Root	Isotig00414	193	13	3.763	5.51E-39	FB|FBgn0003372 - symbol:Sgs1 "Salivary gland secretion 1" species:7227 "Drosophila melanogaster" [GO:0005198 "structural molecule activity" evidence=TAS] [GO:0005576 "extracellular region" evidence=TAS] [GO:0007594 "puparial adhesion" evidence=TAS] EMBL:AE014134 GeneTree:ENSGT00650000092999 RefSeq:NP_523475.3 UniGene:Dm.23846 ProteinModelPortal:Q9VR49 STRING:Q9VR49 EnsemblMetazoa:FBtr0077393 GeneID:33701 KEGG:dme:Dmel_CG3047 UCSC:CG3047-RA CTD:33701 FlyBase:FBgn0003372 eggNOG:NOG124330 OMA:RSTTTTC OrthoDB:EOG41VHM9 NextBio:784856 ArrayExpress:Q9VR49 Bgee:Q9VR49 Uniprot:Q9VR49
Root	Isotig00415	191	13	3.748	1.70E-38	No hit
Root	Isotig00416	191	13	3.748	1.70E-38	No hit
Root	Isotig00417	191	13	3.748	1.70E-38	No hit
Root	Isotig00418	193	13	3.763	5.51E-39	No hit
Root	Isotig00419	191	13	3.748	1.70E-38	No hit
Root	Isotig00420	196	11	4.026	2.89E-41	No hit
Root	Isotig00421	193	13	3.763	5.51E-39	No hit
Root	Isotig00422	198	11	4.041	9.34E-42	No hit
Root	Isotig00423	191	13	3.748	1.70E-38	No hit
Root	Isotig00424	193	13	3.763	5.51E-39	No hit
Root	Isotig00425	191	13	3.748	1.70E-38	No hit
Root	Isotig00426	191	13	3.748	1.70E-38	No hit
Root	Isotig00427	193	13	3.763	5.51E-39	No hit
Root	Isotig00428	191	13	3.748	1.70E-38	No hit
Root	Isotig00429	196	11	4.026	2.89E-41	No hit
Root	Isotig00430	191	13	3.748	1.70E-38	No hit

Tissue	Identification	# of reads (stress)	# of reads (control)	Fold Change	p-value	Description
Root	Isotig00431	191	13	3.748	1.70E-38	No hit
Root	Isotig00432	163	9	4.050	1.01E-34	No hit
Root	Isotig00433	165	9	4.067	3.26E-35	FB|FBgn0003372 - symbol:Sgs1 "Salivary gland secretion 1" species:7227 "Drosophila melanogaster" [GO:0005198 "structural molecule activity" evidence=TAS] [GO:0005576 "extracellular region" evidence=TAS] [GO:0007594 "puparial adhesion" evidence=TAS] EMBL:AE014134 GeneTree:ENSGT00650000092999 RefSeq:NP_523475.3 UniGene:Dm.23846 ProteinModelPortal:Q9VR49 STRING:Q9VR49 EnsemblMetazoa:FBtr0077393 GeneID:33701 KEGG:dme:Dmel_CG3047 UCSC:CG3047-RA CTD:33701 FlyBase:FBgn0003372 eggNOG:NOG124330 OMA:RSTTTC OrthoDB:EOG41VHM9 NextBio:784856 ArrayExpress:Q9VR49 Bgee:Q9VR49 Uniprot:Q9VR49
Root	Isotig00434	163	9	4.050	1.01E-34	No hit
Root	Isotig00435	114	8	3.704	1.83E-23	No hit
Root	Isotig00442	19	3	2.534	0.000639389	No hit
Root	Isotig00443	19	3	2.534	0.000639389	No hit
Root	Isotig00450	28	55	-1.103	0.00070297	TAIR|locus:2051789 - symbol:MTHFR2 "methylenetetrahydrofolate reductase 2" species:3702 "Arabidopsis thaliana" [GO:0004489 "methylenetetrahydrofolate reductase (NADPH) activity" evidence=IEA;ISS] [GO:0006555 "methionine metabolic process" evidence=IEA;ISS] [GO:0055114 "oxidation-reduction process" evidence=IEA] [GO:0005829 "cytosol" evidence=IDA] InterPro:IPR003171 InterPro:IPR004621 Pfam:PF02219 GO:GO:0005829 EMBL:CP002685 GenomeReviews:CT485783_GR GO:GO:0006555 EMBL:AC004005 eggNOG:COG0685 HOGENOM:HBG649727 KO:K00297 GO:GO:0004489 UniGene:At.23509 ProtClustDB:PLN02540 TIGRFAMs:TIGR00677 EMBL:AJ245414 EMBL:AF181967 EMBL:AY050434 EMBL:AF370515 EMBL:AK220952 IPI:IPI00541304 PIR:T00696 RefSeq:NP_566011.1 UniGene:At.11693 ProteinModelPortal:O80585 SMR:O80585 IntAct:O80585 STRING:O80585 PRIDE:O80585 ProMEX:O80585 EnsemblPlants:AT2G44160.1 GeneID:819022 KEGG:ath:AT2G44160 GeneFarm:5158 TAIR:At2g44160 InParanoid:O80585 OMA:MIDESKI PhylomeDB:O80585 ArrayExpress:O80585 Genevestigator:O80585 GermOnline:AT2G44160 Uniprot:O80585
Root	Isotig00451	13	40	-1.750	4.15E-05	TAIR|locus:2051789 - symbol:MTHFR2 "methylenetetrahydrofolate reductase 2" species:3702 "Arabidopsis thaliana" [GO:0004489 "methylenetetrahydrofolate reductase (NADPH) activity" evidence=IEA;ISS] [GO:0006555 "methionine metabolic process" evidence=IEA;ISS] [GO:0055114 "oxidation-reduction process" evidence=IEA] [GO:0005829 "cytosol" evidence=IDA] InterPro:IPR003171 InterPro:IPR004621 Pfam:PF02219 GO:GO:0005829 EMBL:CP002685 GenomeReviews:CT485783_GR GO:GO:0006555 EMBL:AC004005 eggNOG:COG0685 HOGENOM:HBG649727 KO:K00297 GO:GO:0004489 UniGene:At.23509 ProtClustDB:PLN02540 TIGRFAMs:TIGR00677 EMBL:AJ245414 EMBL:AF181967 EMBL:AY050434 EMBL:AF370515 EMBL:AK220952 IPI:IPI00541304 PIR:T00696 RefSeq:NP_566011.1 UniGene:At.11693 ProteinModelPortal:O80585 SMR:O80585 IntAct:O80585 STRING:O80585 PRIDE:O80585 ProMEX:O80585 EnsemblPlants:AT2G44160.1 GeneID:819022 KEGG:ath:AT2G44160 GeneFarm:5158 TAIR:At2g44160 InParanoid:O80585 OMA:MIDESKI PhylomeDB:O80585 ArrayExpress:O80585 Genevestigator:O80585 GermOnline:AT2G44160 Uniprot:O80585
Root	Isotig00453	18	43	-1.385	0.000339352	TAIR|locus:2051789 - symbol:MTHFR2 "methylenetetrahydrofolate reductase 2" species:3702 "Arabidopsis thaliana" [GO:0004489 "methylenetetrahydrofolate reductase (NADPH) activity" evidence=IEA;ISS] [GO:0006555 "methionine metabolic process" evidence=IEA;ISS] [GO:0055114 "oxidation-reduction process" evidence=IEA] [GO:0005829 "cytosol" evidence=IDA] InterPro:IPR003171 InterPro:IPR004621 Pfam:PF02219 GO:GO:0005829 EMBL:CP002685 GenomeReviews:CT485783_GR GO:GO:0006555 EMBL:AC004005 eggNOG:COG0685 HOGENOM:HBG649727 KO:K00297 GO:GO:0004489 UniGene:At.23509 ProtClustDB:PLN02540 TIGRFAMs:TIGR00677 EMBL:AJ245414 EMBL:AF181967 EMBL:AY050434 EMBL:AF370515 EMBL:AK220952 IPI:IPI00541304 PIR:T00696 RefSeq:NP_566011.1 UniGene:At.11693 ProteinModelPortal:O80585 SMR:O80585 IntAct:O80585 STRING:O80585 PRIDE:O80585 ProMEX:O80585 EnsemblPlants:AT2G44160.1 GeneID:819022 KEGG:ath:AT2G44160 GeneFarm:5158 TAIR:At2g44160 InParanoid:O80585 OMA:MIDESKI PhylomeDB:O80585 ArrayExpress:O80585 Genevestigator:O80585 GermOnline:AT2G44160 Uniprot:O80585
Root	Isotig00454	25	113	-2.305	1.69E-16	TAIR|locus:2130419 - symbol:OASA1 "AT4G14880" species:3702 "Arabidopsis thaliana" [GO:0004124 "cysteine synthase activity" evidence=IGI;IDA] [GO:0005829 "cytosol" evidence=ISS;IDA] [GO:0006535 "cysteine biosynthetic process from serine" evidence=TAS] [GO:0046686 "response to cadmium ion" evidence=IEP;IMP;IDA] [GO:0007568 "aging" evidence=IMP] [GO:0005634 "nucleus" evidence=IDA] [GO:0005774 "vacuolar membrane" evidence=IDA] [GO:0016020 "membrane" evidence=IDA] [GO:0005886 "plasma membrane" evidence=IDA] [GO:0005777 "peroxisome" evidence=IDA] [GO:0009507 "chloroplast" evidence=IDA] [GO:0019344 "cysteine

Tissue	Identification	# of reads (stress)	# of reads (control)	Fold Change	p-value	Description
						biosynthetic process" evidence=IMP] [GO:0048046 "apoplast" evidence=IDA] [GO:0005515 "protein binding" evidence=IPI] [GO:0009570 "chloroplast stroma" evidence=IDA] InterPro:IPR001216 InterPro:IPR001926 InterPro:IPR005856 InterPro:IPR005859 Pfam:PF00291 PROSITE:PS00901 GO:GO:0005829 GO:GO:0005886 EMBL:CP002687 GO:GO:0048046 GO:GO:0009570 GO:GO:0005634 GO:GO:0046686 GO:GO:0005774 GO:GO:0005515 GO:GO:0030170 SUPFAM:SSF53686 GO:GO:0005777 GO:GO:0007568 GO:GO:0016740 EMBL:AL161540 EMBL:Z97337 eggNOG:COG0031 GO:GO:0006535 UniGene:At.34389 GO:GO:0004124 EMBL:X80376 EMBL:X84097 EMBL:AJ272027 EMBL:AY045825 EMBL:BT025878 IPI:IPI00519731 PIR:A71412 PIR:S48694 RefSeq:NP_001190732.1 RefSeq:NP_001190733.1 RefSeq:NP_193224.1 RefSeq:NP_849386.1 UniGene:At.30 PDB:1Z7W PDB:1Z7Y PDB:2ISQ PDBsum:1Z7W PDBsum:1Z7Y PDBsum:2ISQ ProteinModelPortal:P47998 SMR:P47998 IntAct:P47998 STRING:P47998 PRIDE:P47998 EnsemblPlants:AT4G14880.1 EnsemblPlants:AT4G14880.2 EnsemblPlants:AT4G14880.3 EnsemblPlants:AT4G14880.4 GeneID:827145 KEGG:ath:AT4G14880 TAIR:At4g14880 InParanoid:P47998 KO:K01738 OMA:SCGERYM PhylomeDB:P47998 ProtClustDB:PLN02565 ArrayExpress:P47998 Genevestigator:P47998 GermOnline:AT4G14880 TIGRFAMs:TIGR01139 TIGRFAMs:TIGR01136 Uniprot:P47998
Root	Isotig00456	12	69	-2.653	3.00E-12	TAIR|locus:2183139 - symbol:SULTR3;5 "sulfate transporter 3;5" species:3702 "Arabidopsis thaliana" [GO:0006810 "transport" evidence=IEA] [GO:0008272 "sulfate transport" evidence=IEA] [GO:0015116 "sulfate transmembrane transporter activity" evidence=ISS] [GO:0016020 "membrane" evidence=IEA] [GO:0016021 "integral to membrane" evidence=IEA] [GO:0055085 "transmembrane transport" evidence=IEA] InterPro:IPR001902 InterPro:IPR011547 InterPro:IPR018045 Pfam:PF00916 PROSITE:PS01130 GO:GO:0016021 EMBL:CP002688 GenomeReviews:BA000015_GR GO:GO:0015293 GO:GO:0008271 InterPro:IPR002645 Gene3D:G3DSA:3.30.750.24 SUPFAM:SSF52091 PROSITE:PS50801 EMBL:AF296838 eggNOG:COG0659 Pfam:PF01740 TIGRFAMs:TIGR00815 HOGENOM:HBG554309 ProtClustDB:CLSN2685981 EMBL:AB061739 IPI:IPI00538924 RefSeq:NP_568377.1 UniGene:At.10546 ProteinModelPortal:Q94LW6 SMR:Q94LW6 PRIDE:Q94LW6 EnsemblPlants:AT5G19600.1 GeneID:832080 KEGG:ath:AT5G19600 GeneFarm:2700 TAIR:At5g19600 InParanoid:Q94LW6 OMA:CKETFFP PhylomeDB:Q94LW6 ArrayExpress:Q94LW6 Genevestigator:Q94LW6 GermOnline:AT5G19600 Uniprot:Q94LW6
Root	Isotig00457	12	69	-2.653	3.00E-12	TAIR|locus:2183139 - symbol:SULTR3;5 "sulfate transporter 3;5" species:3702 "Arabidopsis thaliana" [GO:0006810 "transport" evidence=IEA] [GO:0008272 "sulfate transport" evidence=IEA] [GO:0015116 "sulfate transmembrane transporter activity" evidence=ISS] [GO:0016020 "membrane" evidence=IEA] [GO:0016021 "integral to membrane" evidence=IEA] [GO:0055085 "transmembrane transport" evidence=IEA] InterPro:IPR001902 InterPro:IPR011547 InterPro:IPR018045 Pfam:PF00916 PROSITE:PS01130 GO:GO:0016021 EMBL:CP002688 GenomeReviews:BA000015_GR GO:GO:0015293 GO:GO:0008271 InterPro:IPR002645 Gene3D:G3DSA:3.30.750.24 SUPFAM:SSF52091 PROSITE:PS50801 EMBL:AF296838 eggNOG:COG0659 Pfam:PF01740 TIGRFAMs:TIGR00815 HOGENOM:HBG554309 ProtClustDB:CLSN2685981 EMBL:AB061739 IPI:IPI00538924 RefSeq:NP_568377.1 UniGene:At.10546 ProteinModelPortal:Q94LW6 SMR:Q94LW6 PRIDE:Q94LW6 EnsemblPlants:AT5G19600.1 GeneID:832080 KEGG:ath:AT5G19600 GeneFarm:2700 TAIR:At5g19600 InParanoid:Q94LW6 OMA:CKETFFP PhylomeDB:Q94LW6 ArrayExpress:Q94LW6 Genevestigator:Q94LW6 GermOnline:AT5G19600 Uniprot:Q94LW6
Root	Isotig00458	12	67	-2.610	9.38E-12	TAIR|locus:2183139 - symbol:SULTR3;5 "sulfate transporter 3;5" species:3702 "Arabidopsis thaliana" [GO:0006810 "transport" evidence=IEA] [GO:0008272 "sulfate transport" evidence=IEA] [GO:0015116 "sulfate transmembrane transporter activity" evidence=ISS] [GO:0016020 "membrane" evidence=IEA] [GO:0016021 "integral to membrane" evidence=IEA] [GO:0055085 "transmembrane transport" evidence=IEA] InterPro:IPR001902 InterPro:IPR011547 InterPro:IPR018045 Pfam:PF00916 PROSITE:PS01130 GO:GO:0016021 EMBL:CP002688 GenomeReviews:BA000015_GR GO:GO:0015293 GO:GO:0008271 InterPro:IPR002645 Gene3D:G3DSA:3.30.750.24 SUPFAM:SSF52091 PROSITE:PS50801 EMBL:AF296838 eggNOG:COG0659 Pfam:PF01740 TIGRFAMs:TIGR00815 HOGENOM:HBG554309 ProtClustDB:CLSN2685981 EMBL:AB061739 IPI:IPI00538924 RefSeq:NP_568377.1 UniGene:At.10546 ProteinModelPortal:Q94LW6 SMR:Q94LW6 PRIDE:Q94LW6 EnsemblPlants:AT5G19600.1 GeneID:832080 KEGG:ath:AT5G19600 GeneFarm:2700 TAIR:At5g19600 InParanoid:Q94LW6 OMA:CKETFFP PhylomeDB:Q94LW6 ArrayExpress:Q94LW6 Genevestigator:Q94LW6 GermOnline:AT5G19600 Uniprot:Q94LW6
Root	Isotig00459	12	67	-2.610	9.38E-12	TAIR|locus:2183139 - symbol:SULTR3;5 "sulfate transporter 3;5" species:3702 "Arabidopsis thaliana" [GO:0006810 "transport" evidence=IEA] [GO:0008272 "sulfate transport" evidence=IEA] [GO:0015116 "sulfate transmembrane

Tissue	Identification	# of reads (stress)	# of reads (control)	Fold Change	p-value	Description
						transporter activity" evidence=ISS] [GO:0016020 "membrane" evidence=IEA] [GO:0016021 "integral to membrane" evidence=IEA] [GO:0055085 "transmembrane transport" evidence=IEA] InterPro:IPR001902 InterPro:IPR011547 InterPro:IPR018045 Pfam:PF00916 PROSITE:PS01130 GO:GO:0016021 EMBL:CP002688 GenomeReviews:BA000015_GR GO:GO:0015293 GO:GO:0008271 InterPro:IPR002645 Gene3D:G3DSA:3.30.750.24 SUPFAM:SSF52091 PROSITE:PS50801 EMBL:AF296838 eggNOG:COG0659 Pfam:PF01740 TIGRFAMs:TIGR00815 HOGENOM:HBG554309 ProtClustDB:CLSN2685981 EMBL:AB061739 IPI:IPI00538924 RefSeq:NP_568377.1 UniGene:At.10546 ProteinModelPortal:Q94LW6 SMR:Q94LW6 PRIDE:Q94LW6 EnsemblPlants:AT5G19600.1 GeneID:832080 KEGG:ath:AT5G19600 GeneFarm:2700 TAIR:At5g19600 InParanoid:Q94LW6 OMA:CKETFFP PhylomeDB:Q94LW6 ArrayExpress:Q94LW6 Genevestigator:Q94LW6 GermOnline:AT5G19600 Uniprot:Q94LW6
Root	Isotig00460	12	67	-2.610	9.38E-12	TAIR|locus:2183139 - symbol:SULTR3;5 "sulfate transporter 3;5" species:3702 "Arabidopsis thaliana" [GO:0006810 "transport" evidence=IEA] [GO:0008272 "sulfate transport" evidence=IEA] [GO:0015116 "sulfate transmembrane transporter activity" evidence=ISS] [GO:0016020 "membrane" evidence=IEA] [GO:0016021 "integral to membrane" evidence=IEA] [GO:0055085 "transmembrane transport" evidence=IEA] InterPro:IPR001902 InterPro:IPR011547 InterPro:IPR018045 Pfam:PF00916 PROSITE:PS01130 GO:GO:0016021 EMBL:CP002688 GenomeReviews:BA000015_GR GO:GO:0015293 GO:GO:0008271 InterPro:IPR002645 Gene3D:G3DSA:3.30.750.24 SUPFAM:SSF52091 PROSITE:PS50801 EMBL:AF296838 eggNOG:COG0659 Pfam:PF01740 TIGRFAMs:TIGR00815 HOGENOM:HBG554309 ProtClustDB:CLSN2685981 EMBL:AB061739 IPI:IPI00538924 RefSeq:NP_568377.1 UniGene:At.10546 ProteinModelPortal:Q94LW6 SMR:Q94LW6 PRIDE:Q94LW6 EnsemblPlants:AT5G19600.1 GeneID:832080 KEGG:ath:AT5G19600 GeneFarm:2700 TAIR:At5g19600 InParanoid:Q94LW6 OMA:CKETFFP PhylomeDB:Q94LW6 ArrayExpress:Q94LW6 Genevestigator:Q94LW6 GermOnline:AT5G19600 Uniprot:Q94LW6
Root	Isotig00461	12	67	-2.610	9.38E-12	TAIR|locus:2183139 - symbol:SULTR3;5 "sulfate transporter 3;5" species:3702 "Arabidopsis thaliana" [GO:0006810 "transport" evidence=IEA] [GO:0008272 "sulfate transport" evidence=IEA] [GO:0015116 "sulfate transmembrane transporter activity" evidence=ISS] [GO:0016020 "membrane" evidence=IEA] [GO:0016021 "integral to membrane" evidence=IEA] [GO:0055085 "transmembrane transport" evidence=IEA] InterPro:IPR001902 InterPro:IPR011547 InterPro:IPR018045 Pfam:PF00916 PROSITE:PS01130 GO:GO:0016021 EMBL:CP002688 GenomeReviews:BA000015_GR GO:GO:0015293 GO:GO:0008271 InterPro:IPR002645 Gene3D:G3DSA:3.30.750.24 SUPFAM:SSF52091 PROSITE:PS50801 EMBL:AF296838 eggNOG:COG0659 Pfam:PF01740 TIGRFAMs:TIGR00815 HOGENOM:HBG554309 ProtClustDB:CLSN2685981 EMBL:AB061739 IPI:IPI00538924 RefSeq:NP_568377.1 UniGene:At.10546 ProteinModelPortal:Q94LW6 SMR:Q94LW6 PRIDE:Q94LW6 EnsemblPlants:AT5G19600.1 GeneID:832080 KEGG:ath:AT5G19600 GeneFarm:2700 TAIR:At5g19600 InParanoid:Q94LW6 OMA:CKETFFP PhylomeDB:Q94LW6 ArrayExpress:Q94LW6 Genevestigator:Q94LW6 GermOnline:AT5G19600 Uniprot:Q94LW6
Root	Isotig00462	3	52	-4.244	1.90E-13	TAIR|locus:2183139 - symbol:SULTR3;5 "sulfate transporter 3;5" species:3702 "Arabidopsis thaliana" [GO:0006810 "transport" evidence=IEA] [GO:0008272 "sulfate transport" evidence=IEA] [GO:0015116 "sulfate transmembrane transporter activity" evidence=ISS] [GO:0016020 "membrane" evidence=IEA] [GO:0016021 "integral to membrane" evidence=IEA] [GO:0055085 "transmembrane transport" evidence=IEA] InterPro:IPR001902 InterPro:IPR011547 InterPro:IPR018045 Pfam:PF00916 PROSITE:PS01130 GO:GO:0016021 EMBL:CP002688 GenomeReviews:BA000015_GR GO:GO:0015293 GO:GO:0008271 InterPro:IPR002645 Gene3D:G3DSA:3.30.750.24 SUPFAM:SSF52091 PROSITE:PS50801 EMBL:AF296838 eggNOG:COG0659 Pfam:PF01740 TIGRFAMs:TIGR00815 HOGENOM:HBG554309 ProtClustDB:CLSN2685981 EMBL:AB061739 IPI:IPI00538924 RefSeq:NP_568377.1 UniGene:At.10546 ProteinModelPortal:Q94LW6 SMR:Q94LW6 PRIDE:Q94LW6 EnsemblPlants:AT5G19600.1 GeneID:832080 KEGG:ath:AT5G19600 GeneFarm:2700 TAIR:At5g19600 InParanoid:Q94LW6 OMA:CKETFFP PhylomeDB:Q94LW6 ArrayExpress:Q94LW6 Genevestigator:Q94LW6 GermOnline:AT5G19600 Uniprot:Q94LW6
Root	Isotig00463	3	42	-3.936	9.98E-11	TAIR|locus:2183139 - symbol:SULTR3;5 "sulfate transporter 3;5" species:3702 "Arabidopsis thaliana" [GO:0006810 "transport" evidence=IEA] [GO:0008272 "sulfate transport" evidence=IEA] [GO:0015116 "sulfate transmembrane transporter activity" evidence=ISS] [GO:0016020 "membrane" evidence=IEA] [GO:0016021 "integral to membrane" evidence=IEA] [GO:0055085 "transmembrane transport" evidence=IEA] InterPro:IPR001902 InterPro:IPR011547 InterPro:IPR018045 Pfam:PF00916 PROSITE:PS01130 GO:GO:0016021 EMBL:CP002688 GenomeReviews:BA000015_GR GO:GO:0015293 GO:GO:0008271 InterPro:IPR002645

Tissue	Identification	# of reads (stress)	# of reads (control)	Fold Change	p-value	Description
						Gene3D:G3DSA:3.30.750.24 SUPFAM:SSF52091 PROSITE:PS50801 EMBL:AF296838 eggNOG:COG0659 Pfam:PF01740 TIGRFAMs:TIGR00815 HOGENOM:HBG554309 ProtClustDB:CLSN2685981 EMBL:AB061739 IPI:IPI00538924 RefSeq:NP_568377.1 UniGene:At.10546 ProteinModelPortal:Q94LW6 SMR:Q94LW6 PRIDE:Q94LW6 EnsemblPlants:AT5G19600.1 GeneID:832080 KEGG:ath:AT5G19600 GeneFarm:2700 TAIR:At5g19600 InParanoid:Q94LW6 OMA:CKETFFP PhylomeDB:Q94LW6 ArrayExpress:Q94LW6 Genevestigator:Q94LW6 GermOnline:AT5G19600 Uniprot:Q94LW6
Root	Isotig00464	3	50	-4.188	6.62E-13	TAIR|locus:2183139 - symbol:SULTR3;5 "sulfate transporter 3;5" species:3702 "Arabidopsis thaliana" [GO:0006810 "transport" evidence=IEA] [GO:0008272 "sulfate transport" evidence=IEA] [GO:0015116 "sulfate transmembrane transporter activity" evidence=ISS] [GO:0016020 "membrane" evidence=IEA] [GO:0016021 "integral to membrane" evidence=IEA] [GO:0055085 "transmembrane transport" evidence=IEA] InterPro:IPR001902 InterPro:IPR011547 InterPro:IPR018045 Pfam:PF00916 PROSITE:PS01130 GO:GO:0016021 EMBL:CP002688 GenomeReviews:BA000015_GR GO:GO:0015293 GO:GO:0008271 InterPro:IPR002645 Gene3D:G3DSA:3.30.750.24 SUPFAM:SSF52091 PROSITE:PS50801 EMBL:AF296838 eggNOG:COG0659 Pfam:PF01740 TIGRFAMs:TIGR00815 HOGENOM:HBG554309 ProtClustDB:CLSN2685981 EMBL:AB061739 IPI:IPI00538924 RefSeq:NP_568377.1 UniGene:At.10546 ProteinModelPortal:Q94LW6 SMR:Q94LW6 PRIDE:Q94LW6 EnsemblPlants:AT5G19600.1 GeneID:832080 KEGG:ath:AT5G19600 GeneFarm:2700 TAIR:At5g19600 InParanoid:Q94LW6 OMA:CKETFFP PhylomeDB:Q94LW6 ArrayExpress:Q94LW6 Genevestigator:Q94LW6 GermOnline:AT5G19600 Uniprot:Q94LW6
Root	Isotig00465	9	31	-1.913	0.000125821	TAIR|locus:3437527 - symbol:SULTR3;1 "sulfate transporter 3;1" species:3702 "Arabidopsis thaliana" [GO:0005215 "transporter activity" evidence=IEA] [GO:0006810 "transport" evidence=IEA] [GO:0008271 "secondary active sulfate transmembrane transporter activity" evidence=IEA] [GO:0008272 "sulfate transport" evidence=IEA;ISS] [GO:0015116 "sulfate transmembrane transporter activity" evidence=ISS] [GO:0055085 "transmembrane transport" evidence=IEA] InterPro:IPR001902 InterPro:IPR011547 InterPro:IPR018045 Pfam:PF00916 PROSITE:PS01130 GO:GO:0016021 EMBL:CP002686 GenomeReviews:BA000014_GR GO:GO:0006950 GO:GO:0015293 EMBL:AF049236 EMBL:AL049711 GO:GO:0008271 InterPro:IPR002645 Gene3D:G3DSA:3.30.750.24 SUPFAM:SSF52091 PROSITE:PS50801 eggNOG:COG0659 Pfam:PF01740 TIGRFAMs:TIGR00815 HOGENOM:HBG554309 ProtClustDB:CLSN2685981 EMBL:D89631 EMBL:AB012048 EMBL:AY099873 IPI:IPI00533966 PIR:T48901 PIR:T48902 PIR:T49069 PIR:T51161 RefSeq:NP_190758.2 UniGene:At.11928 ProteinModelPortal:Q9SV13 SMR:Q9SV13 PRIDE:Q9SV13 EnsemblPlants:AT3G51895.1 GeneID:824353 KEGG:ath:AT3G51895 GeneFarm:2359 TAIR:At3g51895 InParanoid:Q9SV13 PhylomeDB:Q9SV13 ArrayExpress:Q9SV13 Genevestigator:Q9SV13 GermOnline:AT3G51895 Uniprot:Q9SV13
Root	Isotig00466	3	50	-4.188	6.62E-13	TAIR|locus:2183139 - symbol:SULTR3;5 "sulfate transporter 3;5" species:3702 "Arabidopsis thaliana" [GO:0006810 "transport" evidence=IEA] [GO:0008272 "sulfate transport" evidence=IEA] [GO:0015116 "sulfate transmembrane transporter activity" evidence=ISS] [GO:0016020 "membrane" evidence=IEA] [GO:0016021 "integral to membrane" evidence=IEA] [GO:0055085 "transmembrane transport" evidence=IEA] InterPro:IPR001902 InterPro:IPR011547 InterPro:IPR018045 Pfam:PF00916 PROSITE:PS01130 GO:GO:0016021 EMBL:CP002688 GenomeReviews:BA000015_GR GO:GO:0015293 GO:GO:0008271 InterPro:IPR002645 Gene3D:G3DSA:3.30.750.24 SUPFAM:SSF52091 PROSITE:PS50801 EMBL:AF296838 eggNOG:COG0659 Pfam:PF01740 TIGRFAMs:TIGR00815 HOGENOM:HBG554309 ProtClustDB:CLSN2685981 EMBL:AB061739 IPI:IPI00538924 RefSeq:NP_568377.1 UniGene:At.10546 ProteinModelPortal:Q94LW6 SMR:Q94LW6 PRIDE:Q94LW6 EnsemblPlants:AT5G19600.1 GeneID:832080 KEGG:ath:AT5G19600 GeneFarm:2700 TAIR:At5g19600 InParanoid:Q94LW6 OMA:CKETFFP PhylomeDB:Q94LW6 ArrayExpress:Q94LW6 Genevestigator:Q94LW6 GermOnline:AT5G19600 Uniprot:Q94LW6
Root	Isotig00467	9	31	-1.913	0.000125821	TAIR|locus:3437527 - symbol:SULTR3;1 "sulfate transporter 3;1" species:3702 "Arabidopsis thaliana" [GO:0005215 "transporter activity" evidence=IEA] [GO:0006810 "transport" evidence=IEA] [GO:0008271 "secondary active sulfate transmembrane transporter activity" evidence=IEA] [GO:0008272 "sulfate transport" evidence=IEA;ISS] [GO:0015116 "sulfate transmembrane transporter activity" evidence=ISS] [GO:0055085 "transmembrane transport" evidence=IEA] InterPro:IPR001902 InterPro:IPR011547 InterPro:IPR018045 Pfam:PF00916 PROSITE:PS01130 GO:GO:0016021 EMBL:CP002686 GenomeReviews:BA000014_GR GO:GO:0006950 GO:GO:0015293 EMBL:AF049236 EMBL:AL049711 GO:GO:0008271 InterPro:IPR002645 Gene3D:G3DSA:3.30.750.24 SUPFAM:SSF52091 PROSITE:PS50801 eggNOG:COG0659 Pfam:PF01740 TIGRFAMs:TIGR00815 HOGENOM:HBG554309 ProtClustDB:CLSN2685981 EMBL:D89631 EMBL:AB012048 EMBL:AY099873

Tissue	Identification	# of reads (stress)	# of reads (control)	Fold Change	p-value	Description
						IPI:IPI00533966 PIR:T48901 PIR:T48902 PIR:T49069 PIR:T51161 RefSeq:NP_190758.2 UniGene:At.11928 ProteinModelPortal:Q9SV13 SMR:Q9SV13 PRIDE:Q9SV13 EnsemblPlants:AT3G51895.1 GeneID:824353 KEGG:ath:AT3G51895 GeneFarm:2359 TAIR:At3g51895 InParanoid:Q9SV13 PhylomeDB:Q9SV13 ArrayExpress:Q9SV13 Genevestigator:Q9SV13 GermOnline:AT3G51895 Uniprot:Q9SV13
Root	Isotig00468	3	40	-3.866	3.51E-10	TAIR|locus:2183139 - symbol:SULTR3;5 "sulfate transporter 3;5" species:3702 "Arabidopsis thaliana" [GO:0006810 "transport" evidence=IEA] [GO:0008272 "sulfate transport" evidence=IEA] [GO:0015116 "sulfate transmembrane transporter activity" evidence=ISS] [GO:0016020 "membrane" evidence=IEA] [GO:0016021 "integral to membrane" evidence=IEA] [GO:0055085 "transmembrane transport" evidence=IEA] InterPro:IPR001902 InterPro:IPR011547 InterPro:IPR018045 Pfam:PF00916 PROSITE:PS01130 GO:GO:0016021 EMBL:CP002688 GenomeReviews:BA000015_GR GO:GO:0015293 GO:GO:0008271 InterPro:IPR002645 Gene3D:G3DSA:3.30.750.24 SUPFAM:SSF52091 PROSITE:PS50801 EMBL:AF296838 eggNOG:COG0659 Pfam:PF01740 TIGRFAMs:TIGR00815 HOGENOM:HBG554309 ProtClustDB:CLSN2685981 EMBL:AB061739 IPI:IPI00538924 RefSeq:NP_568377.1 UniGene:At.10546 ProteinModelPortal:Q94LW6 SMR:Q94LW6 PRIDE:Q94LW6 EnsemblPlants:AT5G19600.1 GeneID:832080 KEGG:ath:AT5G19600 GeneFarm:2700 TAIR:At5g19600 InParanoid:Q94LW6 OMA:CKETFFP PhylomeDB:Q94LW6 ArrayExpress:Q94LW6 Genevestigator:Q94LW6 GermOnline:AT5G19600 Uniprot:Q94LW6
Root	Isotig00469	3	40	-3.866	3.51E-10	TAIR|locus:2183139 - symbol:SULTR3;5 "sulfate transporter 3;5" species:3702 "Arabidopsis thaliana" [GO:0006810 "transport" evidence=IEA] [GO:0008272 "sulfate transport" evidence=IEA] [GO:0015116 "sulfate transmembrane transporter activity" evidence=ISS] [GO:0016020 "membrane" evidence=IEA] [GO:0016021 "integral to membrane" evidence=IEA] [GO:0055085 "transmembrane transport" evidence=IEA] InterPro:IPR001902 InterPro:IPR011547 InterPro:IPR018045 Pfam:PF00916 PROSITE:PS01130 GO:GO:0016021 EMBL:CP002688 GenomeReviews:BA000015_GR GO:GO:0015293 GO:GO:0008271 InterPro:IPR002645 Gene3D:G3DSA:3.30.750.24 SUPFAM:SSF52091 PROSITE:PS50801 EMBL:AF296838 eggNOG:COG0659 Pfam:PF01740 TIGRFAMs:TIGR00815 HOGENOM:HBG554309 ProtClustDB:CLSN2685981 EMBL:AB061739 IPI:IPI00538924 RefSeq:NP_568377.1 UniGene:At.10546 ProteinModelPortal:Q94LW6 SMR:Q94LW6 PRIDE:Q94LW6 EnsemblPlants:AT5G19600.1 GeneID:832080 KEGG:ath:AT5G19600 GeneFarm:2700 TAIR:At5g19600 InParanoid:Q94LW6 OMA:CKETFFP PhylomeDB:Q94LW6 ArrayExpress:Q94LW6 Genevestigator:Q94LW6 GermOnline:AT5G19600 Uniprot:Q94LW6
Root	Isotig00471	0	14	-4.936	8.24E-05	TAIR|locus:2183139 - symbol:SULTR3;5 "sulfate transporter 3;5" species:3702 "Arabidopsis thaliana" [GO:0006810 "transport" evidence=IEA] [GO:0008272 "sulfate transport" evidence=IEA] [GO:0015116 "sulfate transmembrane transporter activity" evidence=ISS] [GO:0016020 "membrane" evidence=IEA] [GO:0016021 "integral to membrane" evidence=IEA] [GO:0055085 "transmembrane transport" evidence=IEA] InterPro:IPR001902 InterPro:IPR011547 InterPro:IPR018045 Pfam:PF00916 PROSITE:PS01130 GO:GO:0016021 EMBL:CP002688 GenomeReviews:BA000015_GR GO:GO:0015293 GO:GO:0008271 InterPro:IPR002645 Gene3D:G3DSA:3.30.750.24 SUPFAM:SSF52091 PROSITE:PS50801 EMBL:AF296838 eggNOG:COG0659 Pfam:PF01740 TIGRFAMs:TIGR00815 HOGENOM:HBG554309 ProtClustDB:CLSN2685981 EMBL:AB061739 IPI:IPI00538924 RefSeq:NP_568377.1 UniGene:At.10546 ProteinModelPortal:Q94LW6 SMR:Q94LW6 PRIDE:Q94LW6 EnsemblPlants:AT5G19600.1 GeneID:832080 KEGG:ath:AT5G19600 GeneFarm:2700 TAIR:At5g19600 InParanoid:Q94LW6 OMA:CKETFFP PhylomeDB:Q94LW6 ArrayExpress:Q94LW6 Genevestigator:Q94LW6 GermOnline:AT5G19600 Uniprot:Q94LW6
Root	Isotig00473	5	74	-4.016	5.70E-18	TAIR|locus:2203221 - symbol:NIA1 "AT1G77760" species:3702 "Arabidopsis thaliana" [GO:0006809 "nitric oxide biosynthetic process" evidence=IMP] [GO:0005829 "cytosol" evidence=TAS] [GO:0009416 "response to light stimulus" evidence=IMP] [GO:0005515 "protein binding" evidence=IPI] [GO:0008940 "nitrate reductase activity" evidence=IMP] [GO:0042128 "nitrate assimilation" evidence=IMP] InterPro:IPR000572 InterPro:IPR001199 InterPro:IPR001433 InterPro:IPR001709 InterPro:IPR001834 InterPro:IPR005066 InterPro:IPR008335 InterPro:IPR012137 InterPro:IPR017927 InterPro:IPR017938 InterPro:IPR018506 InterPro:IPR022407 Pfam:PF00173 Pfam:PF00174 Pfam:PF00175 Pfam:PF03404 PIRSF:PIRSF000233 PRINTS:PR00363 PRINTS:PR00371 PRINTS:PR00406 PRINTS:PR00407 PROSITE:PS00191 PROSITE:PS00559 PROSITE:PS50255 PROSITE:PS51384 InterPro:IPR008333 Pfam:PF00970 EMBL:CP002684 GenomeReviews:CT485782_GR GO:GO:0005829 GO:GO:0005515 GO:GO:0009635 GO:GO:0009055 GO:GO:0050660 GO:GO:0020037 GO:GO:0009416 Gene3D:G3DSA:3.10.120.10 SUPFAM:SSF55856 InterPro:IPR014756 SUPFAM:SSF81296 GO:GO:0043546 eggNOG:COG0543 SUPFAM:SSF63380

Tissue	Identification	# of reads (stress)	# of reads (control)	Fold Change	p-value	Description
						GO:GO:0006809 GO:GO:0042128 EMBL:AC012193 GO:GO:0030151 KO:K00360 EMBL:Z19050 EMBL:AY090950 EMBL:AF424624 EMBL:BT000989 EMBL:X13434 EMBL:X13436 IPI:IPI00518959 PIR:E96807 PIR:S35228 RefSeq:NP_177899.1 UniGene:At.17771 ProteinModelPortal:P11832 SMR:P11832 IntAct:P11832 STRING:P11832 PRIDE:P11832 EnsemblPlants:AT1G77760.1 GeneID:844112 KEGG:ath:AT1G77760 TAIR:At1g77760 HOGENOM:HBG326850 InParanoid:P11832 OMA:HGAVPKA PhylomeDB:P11832 ProtClustDB:PLN02252 ArrayExpress:P11832 Genevestigator:P11832 GermOnline:AT1G77760 GO:GO:0009703 Gene3D:G3DSA:2.60.40.650 Gene3D:G3DSA:3.90.420.10 SUPFAM:SSF56524 Uniprot:P11832
Root	Isotig00474	5	74	-4.016	5.70E-18	TAIR|locus:2203221 - symbol:NIA1 "AT1G77760" species:3702 "Arabidopsis thaliana" [GO:0006809 "nitric oxide biosynthetic process" evidence=IMP] [GO:0005829 "cytosol" evidence=TAS] [GO:0009416 "response to light stimulus" evidence=IMP] [GO:0005515 "protein binding" evidence=IPI] [GO:0008940 "nitrate reductase activity" evidence=IMP] [GO:0042128 "nitrate assimilation" evidence=IMP] InterPro:IPR000572 InterPro:IPR001199 InterPro:IPR001433 InterPro:IPR001709 InterPro:IPR001834 InterPro:IPR005066 InterPro:IPR008335 InterPro:IPR012137 InterPro:IPR017927 InterPro:IPR017938 InterPro:IPR018506 InterPro:IPR022407 Pfam:PF00173 Pfam:PF00174 Pfam:PF00175 Pfam:PF03404 PIRSF:PIRSF000233 PRINTS:PR00363 PRINTS:PR00371 PRINTS:PR00406 PRINTS:PR00407 PROSITE:PS00191 PROSITE:PS00559 PROSITE:PS50255 PROSITE:PS51384 InterPro:IPR008333 Pfam:PF00970 EMBL:CP002684 GenomeReviews:CT485782_GR GO:GO:0005829 GO:GO:0005515 GO:GO:0009635 GO:GO:0009055 GO:GO:0050660 GO:GO:0020037 GO:GO:0009416 Gene3D:G3DSA:3.10.120.10 SUPFAM:SSF55856 InterPro:IPR014756 SUPFAM:SSF81296 GO:GO:0043546 eggNOG:COG0543 SUPFAM:SSF63380 GO:GO:0006809 GO:GO:0042128 EMBL:AC012193 GO:GO:0030151 KO:K00360 EMBL:Z19050 EMBL:AY090950 EMBL:AF424624 EMBL:BT000989 EMBL:X13434 EMBL:X13436 IPI:IPI00518959 PIR:E96807 PIR:S35228 RefSeq:NP_177899.1 UniGene:At.17771 ProteinModelPortal:P11832 SMR:P11832 IntAct:P11832 STRING:P11832 PRIDE:P11832 EnsemblPlants:AT1G77760.1 GeneID:844112 KEGG:ath:AT1G77760 TAIR:At1g77760 HOGENOM:HBG326850 InParanoid:P11832 OMA:HGAVPKA PhylomeDB:P11832 ProtClustDB:PLN02252 ArrayExpress:P11832 Genevestigator:P11832 GermOnline:AT1G77760 GO:GO:0009703 Gene3D:G3DSA:2.60.40.650 Gene3D:G3DSA:3.90.420.10 SUPFAM:SSF56524 Uniprot:P11832
Root	Isotig00475	4	75	-4.358	5.91E-19	TAIR|locus:2203221 - symbol:NIA1 "AT1G77760" species:3702 "Arabidopsis thaliana" [GO:0006809 "nitric oxide biosynthetic process" evidence=IMP] [GO:0005829 "cytosol" evidence=TAS] [GO:0009416 "response to light stimulus" evidence=IMP] [GO:0005515 "protein binding" evidence=IPI] [GO:0008940 "nitrate reductase activity" evidence=IMP] [GO:0042128 "nitrate assimilation" evidence=IMP] InterPro:IPR000572 InterPro:IPR001199 InterPro:IPR001433 InterPro:IPR001709 InterPro:IPR001834 InterPro:IPR005066 InterPro:IPR008335 InterPro:IPR012137 InterPro:IPR017927 InterPro:IPR017938 InterPro:IPR018506 InterPro:IPR022407 Pfam:PF00173 Pfam:PF00174 Pfam:PF00175 Pfam:PF03404 PIRSF:PIRSF000233 PRINTS:PR00363 PRINTS:PR00371 PRINTS:PR00406 PRINTS:PR00407 PROSITE:PS00191 PROSITE:PS00559 PROSITE:PS50255 PROSITE:PS51384 InterPro:IPR008333 Pfam:PF00970 EMBL:CP002684 GenomeReviews:CT485782_GR GO:GO:0005829 GO:GO:0005515 GO:GO:0009635 GO:GO:0009055 GO:GO:0050660 GO:GO:0020037 GO:GO:0009416 Gene3D:G3DSA:3.10.120.10 SUPFAM:SSF55856 InterPro:IPR014756 SUPFAM:SSF81296 GO:GO:0043546 eggNOG:COG0543 SUPFAM:SSF63380 GO:GO:0006809 GO:GO:0042128 EMBL:AC012193 GO:GO:0030151 KO:K00360 EMBL:Z19050 EMBL:AY090950 EMBL:AF424624 EMBL:BT000989 EMBL:X13434 EMBL:X13436 IPI:IPI00518959 PIR:E96807 PIR:S35228 RefSeq:NP_177899.1 UniGene:At.17771 ProteinModelPortal:P11832 SMR:P11832 IntAct:P11832 STRING:P11832 PRIDE:P11832 EnsemblPlants:AT1G77760.1 GeneID:844112 KEGG:ath:AT1G77760 TAIR:At1g77760 HOGENOM:HBG326850 InParanoid:P11832 OMA:HGAVPKA PhylomeDB:P11832 ProtClustDB:PLN02252 ArrayExpress:P11832 Genevestigator:P11832 GermOnline:AT1G77760 GO:GO:0009703 Gene3D:G3DSA:2.60.40.650 Gene3D:G3DSA:3.90.420.10 SUPFAM:SSF56524 Uniprot:P11832
Root	Isotig00476	5	74	-4.016	5.70E-18	TAIR|locus:2203221 - symbol:NIA1 "AT1G77760" species:3702 "Arabidopsis thaliana" [GO:0006809 "nitric oxide biosynthetic process" evidence=IMP] [GO:0005829 "cytosol" evidence=TAS] [GO:0009416 "response to light stimulus" evidence=IMP] [GO:0005515 "protein binding" evidence=IPI] [GO:0008940 "nitrate reductase activity" evidence=IMP] [GO:0042128 "nitrate assimilation" evidence=IMP] InterPro:IPR000572 InterPro:IPR001199 InterPro:IPR001433 InterPro:IPR001709 InterPro:IPR001834 InterPro:IPR005066 InterPro:IPR008335 InterPro:IPR012137 InterPro:IPR017927 InterPro:IPR017938 InterPro:IPR018506 InterPro:IPR022407 Pfam:PF00173 Pfam:PF00174 Pfam:PF00175 Pfam:PF03404 PIRSF:PIRSF000233 PRINTS:PR00363

Tissue	Identification	# of reads (stress)	# of reads (control)	Fold Change	p-value	Description
						PRINTS:PR00371 PRINTS:PR00406 PRINTS:PR00407 PROSITE:PS00191 PROSITE:PS00559 PROSITE:PS50255 PROSITE:PS51384 InterPro:IPR008333 Pfam:PF00970 EMBL:CP002684 GenomeReviews:CT485782_GR GO:GO:0005829 GO:GO:0005515 GO:GO:0009635 GO:GO:0009055 GO:GO:0050660 GO:GO:0020037 GO:GO:0009416 Gene3D:G3DSA:3.10.120.10 SUPFAM:SSF55856 InterPro:IPR014756 SUPFAM:SSF81296 GO:GO:0043546 eggNOG:COG0543 SUPFAM:SSF63380 GO:GO:0006809 GO:GO:0042128 EMBL:AC012193 GO:GO:0030151 KO:K00360 EMBL:Z19050 EMBL:AY090950 EMBL:AF424624 EMBL:BT000989 EMBL:X13434 EMBL:X13436 IPI:IPI00518959 PIR:E96807 PIR:S35228 RefSeq:NP_177899.1 UniGene:At.17771 ProteinModelPortal:P11832 SMR:P11832 IntAct:P11832 STRING:P11832 PRIDE:P11832 EnsemblPlants:AT1G77760.1 GeneID:844112 KEGG:ath:AT1G77760 TAIR:At1g77760 HOGENOM:HBG326850 InParanoid:P11832 OMA:HGAVPKA PhylomeDB:P11832 ProtClustDB:PLN02252 ArrayExpress:P11832 Genevestigator:P11832 GermOnline:AT1G77760 GO:GO:0009703 Gene3D:G3DSA:2.60.40.650 Gene3D:G3DSA:3.90.420.10 SUPFAM:SSF56524 Uniprot:P11832
Root	Isotig00477	5	74	-4.016	5.70E-18	TAIR|locus:2203221 - symbol:NIA1 "AT1G77760" species:3702 "Arabidopsis thaliana" [GO:0006809 "nitric oxide biosynthetic process" evidence=IMP] [GO:0005829 "cytosol" evidence=TAS] [GO:0009416 "response to light stimulus" evidence=IMP] [GO:0005515 "protein binding" evidence=IPI] [GO:0008940 "nitrate reductase activity" evidence=IMP] [GO:0042128 "nitrate assimilation" evidence=IMP] InterPro:IPR000572 InterPro:IPR001199 InterPro:IPR001433 InterPro:IPR001709 InterPro:IPR001834 InterPro:IPR005066 InterPro:IPR008335 InterPro:IPR012137 InterPro:IPR017927 InterPro:IPR017938 InterPro:IPR018506 InterPro:IPR022407 Pfam:PF00173 Pfam:PF00174 Pfam:PF00175 Pfam:PF03404 PIRSF:PIRSF000233 PRINTS:PR00363 PRINTS:PR00371 PRINTS:PR00406 PRINTS:PR00407 PROSITE:PS00191 PROSITE:PS00559 PROSITE:PS50255 PROSITE:PS51384 InterPro:IPR008333 Pfam:PF00970 EMBL:CP002684 GenomeReviews:CT485782_GR GO:GO:0005829 GO:GO:0005515 GO:GO:0009635 GO:GO:0009055 GO:GO:0050660 GO:GO:0020037 GO:GO:0009416 Gene3D:G3DSA:3.10.120.10 SUPFAM:SSF55856 InterPro:IPR014756 SUPFAM:SSF81296 GO:GO:0043546 eggNOG:COG0543 SUPFAM:SSF63380 GO:GO:0006809 GO:GO:0042128 EMBL:AC012193 GO:GO:0030151 KO:K00360 EMBL:Z19050 EMBL:AY090950 EMBL:AF424624 EMBL:BT000989 EMBL:X13434 EMBL:X13436 IPI:IPI00518959 PIR:E96807 PIR:S35228 RefSeq:NP_177899.1 UniGene:At.17771 ProteinModelPortal:P11832 SMR:P11832 IntAct:P11832 STRING:P11832 PRIDE:P11832 EnsemblPlants:AT1G77760.1 GeneID:844112 KEGG:ath:AT1G77760 TAIR:At1g77760 HOGENOM:HBG326850 InParanoid:P11832 OMA:HGAVPKA PhylomeDB:P11832 ProtClustDB:PLN02252 ArrayExpress:P11832 Genevestigator:P11832 GermOnline:AT1G77760 GO:GO:0009703 Gene3D:G3DSA:2.60.40.650 Gene3D:G3DSA:3.90.420.10 SUPFAM:SSF56524 Uniprot:P11832
Root	Isotig00478	4	75	-4.358	5.91E-19	TAIR|locus:2203221 - symbol:NIA1 "AT1G77760" species:3702 "Arabidopsis thaliana" [GO:0006809 "nitric oxide biosynthetic process" evidence=IMP] [GO:0005829 "cytosol" evidence=TAS] [GO:0009416 "response to light stimulus" evidence=IMP] [GO:0005515 "protein binding" evidence=IPI] [GO:0008940 "nitrate reductase activity" evidence=IMP] [GO:0042128 "nitrate assimilation" evidence=IMP] InterPro:IPR000572 InterPro:IPR001199 InterPro:IPR001433 InterPro:IPR001709 InterPro:IPR001834 InterPro:IPR005066 InterPro:IPR008335 InterPro:IPR012137 InterPro:IPR017927 InterPro:IPR017938 InterPro:IPR018506 InterPro:IPR022407 Pfam:PF00173 Pfam:PF00174 Pfam:PF00175 Pfam:PF03404 PIRSF:PIRSF000233 PRINTS:PR00363 PRINTS:PR00371 PRINTS:PR00406 PRINTS:PR00407 PROSITE:PS00191 PROSITE:PS00559 PROSITE:PS50255 PROSITE:PS51384 InterPro:IPR008333 Pfam:PF00970 EMBL:CP002684 GenomeReviews:CT485782_GR GO:GO:0005829 GO:GO:0005515 GO:GO:0009635 GO:GO:0009055 GO:GO:0050660 GO:GO:0020037 GO:GO:0009416 Gene3D:G3DSA:3.10.120.10 SUPFAM:SSF55856 InterPro:IPR014756 SUPFAM:SSF81296 GO:GO:0043546 eggNOG:COG0543 SUPFAM:SSF63380 GO:GO:0006809 GO:GO:0042128 EMBL:AC012193 GO:GO:0030151 KO:K00360 EMBL:Z19050 EMBL:AY090950 EMBL:AF424624 EMBL:BT000989 EMBL:X13434 EMBL:X13436 IPI:IPI00518959 PIR:E96807 PIR:S35228 RefSeq:NP_177899.1 UniGene:At.17771 ProteinModelPortal:P11832 SMR:P11832 IntAct:P11832 STRING:P11832 PRIDE:P11832 EnsemblPlants:AT1G77760.1 GeneID:844112 KEGG:ath:AT1G77760 TAIR:At1g77760 HOGENOM:HBG326850 InParanoid:P11832 OMA:HGAVPKA PhylomeDB:P11832 ProtClustDB:PLN02252 ArrayExpress:P11832 Genevestigator:P11832 GermOnline:AT1G77760 GO:GO:0009703 Gene3D:G3DSA:2.60.40.650 Gene3D:G3DSA:3.90.420.10 SUPFAM:SSF56524 Uniprot:P11832
Root	Isotig00479	5	72	-3.977	1.98E-17	TAIR|locus:2203221 - symbol:NIA1 "AT1G77760" species:3702 "Arabidopsis thaliana" [GO:0006809 "nitric oxide biosynthetic process" evidence=IMP] [GO:0005829 "cytosol" evidence=TAS] [GO:0009416 "response to light

Tissue	Identification	# of reads (stress)	# of reads (control)	Fold Change	p-value	Description
						stimulus" evidence=IMP] [GO:0005515 "protein binding" evidence=IPI] [GO:0008940 "nitrate reductase activity" evidence=IMP] [GO:0042128 "nitrate assimilation" evidence=IMP] InterPro:IPR000572 InterPro:IPR001199 InterPro:IPR001433 InterPro:IPR001709 InterPro:IPR001834 InterPro:IPR005066 InterPro:IPR008335 InterPro:IPR012137 InterPro:IPR017927 InterPro:IPR017938 InterPro:IPR018506 InterPro:IPR022407 Pfam:PF00173 Pfam:PF00174 Pfam:PF00175 Pfam:PF03404 PIRSF:PIRSF000233 PRINTS:PR00363 PRINTS:PR00371 PRINTS:PR00406 PRINTS:PR00407 PROSITE:PS00191 PROSITE:PS00559 PROSITE:PS50255 PROSITE:PS51384 InterPro:IPR008333 Pfam:PF00970 EMBL:CP002684 GenomeReviews:CT485782_GR GO:GO:0005829 GO:GO:0005515 GO:GO:0009635 GO:GO:0009055 GO:GO:0050660 GO:GO:0020037 GO:GO:0009416 Gene3D:G3DSA:3.10.120.10 SUPFAM:SSF55856 InterPro:IPR014756 SUPFAM:SSF81296 GO:GO:0043546 eggNOG:COG0543 SUPFAM:SSF63380 GO:GO:0006809 GO:GO:0042128 EMBL:AC012193 GO:GO:0030151 KO:K00360 EMBL:Z19050 EMBL:AY090950 EMBL:AF424624 EMBL:BT000989 EMBL:X13434 EMBL:X13436 IPI:IPI00518959 PIR:E96807 PIR:S35228 RefSeq:NP_177899.1 UniGene:At.17771 ProteinModelPortal:P11832 SMR:P11832 IntAct:P11832 STRING:P11832 PRIDE:P11832 EnsemblPlants:AT1G77760.1 GeneID:844112 KEGG:ath:AT1G77760 TAIR:At1g77760 HOGENOM:HBG326850 InParanoid:P11832 OMA:HGAVPKA PhylomeDB:P11832 ProtClustDB:PLN02252 ArrayExpress:P11832 Genevestigator:P11832 GermOnline:AT1G77760 GO:GO:0009703 Gene3D:G3DSA:2.60.40.650 Gene3D:G3DSA:3.90.420.10 SUPFAM:SSF56524 Uniprot:P11832
Root	Isotig00480	5	72	-3.977	1.98E-17	TAIR|locus:2203221 - symbol:NIA1 "AT1G77760" species:3702 "Arabidopsis thaliana" [GO:0006809 "nitric oxide biosynthetic process" evidence=IMP] [GO:0005829 "cytosol" evidence=TAS] [GO:0009416 "response to light stimulus" evidence=IMP] [GO:0005515 "protein binding" evidence=IPI] [GO:0008940 "nitrate reductase activity" evidence=IMP] [GO:0042128 "nitrate assimilation" evidence=IMP] InterPro:IPR000572 InterPro:IPR001199 InterPro:IPR001433 InterPro:IPR001709 InterPro:IPR001834 InterPro:IPR005066 InterPro:IPR008335 InterPro:IPR012137 InterPro:IPR017927 InterPro:IPR017938 InterPro:IPR018506 InterPro:IPR022407 Pfam:PF00173 Pfam:PF00174 Pfam:PF00175 Pfam:PF03404 PIRSF:PIRSF000233 PRINTS:PR00363 PRINTS:PR00371 PRINTS:PR00406 PRINTS:PR00407 PROSITE:PS00191 PROSITE:PS00559 PROSITE:PS50255 PROSITE:PS51384 InterPro:IPR008333 Pfam:PF00970 EMBL:CP002684 GenomeReviews:CT485782_GR GO:GO:0005829 GO:GO:0005515 GO:GO:0009635 GO:GO:0009055 GO:GO:0050660 GO:GO:0020037 GO:GO:0009416 Gene3D:G3DSA:3.10.120.10 SUPFAM:SSF55856 InterPro:IPR014756 SUPFAM:SSF81296 GO:GO:0043546 eggNOG:COG0543 SUPFAM:SSF63380 GO:GO:0006809 GO:GO:0042128 EMBL:AC012193 GO:GO:0030151 KO:K00360 EMBL:Z19050 EMBL:AY090950 EMBL:AF424624 EMBL:BT000989 EMBL:X13434 EMBL:X13436 IPI:IPI00518959 PIR:E96807 PIR:S35228 RefSeq:NP_177899.1 UniGene:At.17771 ProteinModelPortal:P11832 SMR:P11832 IntAct:P11832 STRING:P11832 PRIDE:P11832 EnsemblPlants:AT1G77760.1 GeneID:844112 KEGG:ath:AT1G77760 TAIR:At1g77760 HOGENOM:HBG326850 InParanoid:P11832 OMA:HGAVPKA PhylomeDB:P11832 ProtClustDB:PLN02252 ArrayExpress:P11832 Genevestigator:P11832 GermOnline:AT1G77760 GO:GO:0009703 Gene3D:G3DSA:2.60.40.650 Gene3D:G3DSA:3.90.420.10 SUPFAM:SSF56524 Uniprot:P11832
Root	Isotig00481	4	73	-4.319	2.04E-18	TAIR|locus:2203221 - symbol:NIA1 "AT1G77760" species:3702 "Arabidopsis thaliana" [GO:0006809 "nitric oxide biosynthetic process" evidence=IMP] [GO:0005829 "cytosol" evidence=TAS] [GO:0009416 "response to light stimulus" evidence=IMP] [GO:0005515 "protein binding" evidence=IPI] [GO:0008940 "nitrate reductase activity" evidence=IMP] [GO:0042128 "nitrate assimilation" evidence=IMP] InterPro:IPR000572 InterPro:IPR001199 InterPro:IPR001433 InterPro:IPR001709 InterPro:IPR001834 InterPro:IPR005066 InterPro:IPR008335 InterPro:IPR012137 InterPro:IPR017927 InterPro:IPR017938 InterPro:IPR018506 InterPro:IPR022407 Pfam:PF00173 Pfam:PF00174 Pfam:PF00175 Pfam:PF03404 PIRSF:PIRSF000233 PRINTS:PR00363 PRINTS:PR00371 PRINTS:PR00406 PRINTS:PR00407 PROSITE:PS00191 PROSITE:PS00559 PROSITE:PS50255 PROSITE:PS51384 InterPro:IPR008333 Pfam:PF00970 EMBL:CP002684 GenomeReviews:CT485782_GR GO:GO:0005829 GO:GO:0005515 GO:GO:0009635 GO:GO:0009055 GO:GO:0050660 GO:GO:0020037 GO:GO:0009416 Gene3D:G3DSA:3.10.120.10 SUPFAM:SSF55856 InterPro:IPR014756 SUPFAM:SSF81296 GO:GO:0043546 eggNOG:COG0543 SUPFAM:SSF63380 GO:GO:0006809 GO:GO:0042128 EMBL:AC012193 GO:GO:0030151 KO:K00360 EMBL:Z19050 EMBL:AY090950 EMBL:AF424624 EMBL:BT000989 EMBL:X13434 EMBL:X13436 IPI:IPI00518959 PIR:E96807 PIR:S35228 RefSeq:NP_177899.1 UniGene:At.17771 ProteinModelPortal:P11832 SMR:P11832 IntAct:P11832 STRING:P11832 PRIDE:P11832 EnsemblPlants:AT1G77760.1 GeneID:844112 KEGG:ath:AT1G77760 TAIR:At1g77760 HOGENOM:HBG326850 InParanoid:P11832 OMA:HGAVPKA PhylomeDB:P11832 ProtClustDB:PLN02252

Tissue	Identification	# of reads (stress)	# of reads (control)	Fold Change	p-value	Description
						ArrayExpress:P11832 Genevestigator:P11832 GermOnline:AT1G77760 GO:GO:0009703 Gene3D:G3DSA:2.60.40.650 Gene3D:G3DSA:3.90.420.10 SUPFAM:SSF56524 Uniprot:P11832
Root	Isotig00482	5	72	-3.977	1.98E-17	TAIR|locus:2203221 - symbol:NIA1 "AT1G77760" species:3702 "Arabidopsis thaliana" [GO:0006809 "nitric oxide biosynthetic process" evidence=IMP] [GO:0005829 "cytosol" evidence=TAS] [GO:0009416 "response to light stimulus" evidence=IMP] [GO:0005515 "protein binding" evidence=IPI] [GO:0008940 "nitrate reductase activity" evidence=IMP] [GO:0042128 "nitrate assimilation" evidence=IMP] InterPro:IPR000572 InterPro:IPR001199 InterPro:IPR001433 InterPro:IPR001709 InterPro:IPR001834 InterPro:IPR005066 InterPro:IPR008335 InterPro:IPR012137 InterPro:IPR017927 InterPro:IPR017938 InterPro:IPR018506 InterPro:IPR022407 Pfam:PF00173 Pfam:PF00174 Pfam:PF00175 Pfam:PF03404 PIRSF:PIRSF000233 PRINTS:PR00363 PRINTS:PR00371 PRINTS:PR00406 PRINTS:PR00407 PROSITE:PS00191 PROSITE:PS00559 PROSITE:PS50255 PROSITE:PS51384 InterPro:IPR008333 Pfam:PF00970 EMBL:CP002684 GenomeReviews:CT485782_GR GO:GO:0005829 GO:GO:0005515 GO:GO:0009635 GO:GO:0009055 GO:GO:0050660 GO:GO:0020037 GO:GO:0009416 Gene3D:G3DSA:3.10.120.10 SUPFAM:SSF55856 InterPro:IPR014756 SUPFAM:SSF81296 GO:GO:0043546 eggNOG:COG0543 SUPFAM:SSF63380 GO:GO:0006809 GO:GO:0042128 EMBL:AC012193 GO:GO:0030151 KO:K00360 EMBL:Z19050 EMBL:AY090950 EMBL:AF424624 EMBL:BT000989 EMBL:X13434 EMBL:X13436 IPI:IPI00518959 PIR:E96807 PIR:S35228 RefSeq:NP_177899.1 UniGene:At.17771 ProteinModelPortal:P11832 SMR:P11832 IntAct:P11832 STRING:P11832 PRIDE:P11832 EnsemblPlants:AT1G77760.1 GeneID:844112 KEGG:ath:AT1G77760 TAIR:At1g77760 HOGENOM:HBG326850 InParanoid:P11832 OMA:HGAVPKA PhylomeDB:P11832 ProtClustDB:PLN02252 ArrayExpress:P11832 Genevestigator:P11832 GermOnline:AT1G77760 GO:GO:0009703 Gene3D:G3DSA:2.60.40.650 Gene3D:G3DSA:3.90.420.10 SUPFAM:SSF56524 Uniprot:P11832
Root	Isotig00483	5	72	-3.977	1.98E-17	TAIR|locus:2203221 - symbol:NIA1 "AT1G77760" species:3702 "Arabidopsis thaliana" [GO:0006809 "nitric oxide biosynthetic process" evidence=IMP] [GO:0005829 "cytosol" evidence=TAS] [GO:0009416 "response to light stimulus" evidence=IMP] [GO:0005515 "protein binding" evidence=IPI] [GO:0008940 "nitrate reductase activity" evidence=IMP] [GO:0042128 "nitrate assimilation" evidence=IMP] InterPro:IPR000572 InterPro:IPR001199 InterPro:IPR001433 InterPro:IPR001709 InterPro:IPR001834 InterPro:IPR005066 InterPro:IPR008335 InterPro:IPR012137 InterPro:IPR017927 InterPro:IPR017938 InterPro:IPR018506 InterPro:IPR022407 Pfam:PF00173 Pfam:PF00174 Pfam:PF00175 Pfam:PF03404 PIRSF:PIRSF000233 PRINTS:PR00363 PRINTS:PR00371 PRINTS:PR00406 PRINTS:PR00407 PROSITE:PS00191 PROSITE:PS00559 PROSITE:PS50255 PROSITE:PS51384 InterPro:IPR008333 Pfam:PF00970 EMBL:CP002684 GenomeReviews:CT485782_GR GO:GO:0005829 GO:GO:0005515 GO:GO:0009635 GO:GO:0009055 GO:GO:0050660 GO:GO:0020037 GO:GO:0009416 Gene3D:G3DSA:3.10.120.10 SUPFAM:SSF55856 InterPro:IPR014756 SUPFAM:SSF81296 GO:GO:0043546 eggNOG:COG0543 SUPFAM:SSF63380 GO:GO:0006809 GO:GO:0042128 EMBL:AC012193 GO:GO:0030151 KO:K00360 EMBL:Z19050 EMBL:AY090950 EMBL:AF424624 EMBL:BT000989 EMBL:X13434 EMBL:X13436 IPI:IPI00518959 PIR:E96807 PIR:S35228 RefSeq:NP_177899.1 UniGene:At.17771 ProteinModelPortal:P11832 SMR:P11832 IntAct:P11832 STRING:P11832 PRIDE:P11832 EnsemblPlants:AT1G77760.1 GeneID:844112 KEGG:ath:AT1G77760 TAIR:At1g77760 HOGENOM:HBG326850 InParanoid:P11832 OMA:HGAVPKA PhylomeDB:P11832 ProtClustDB:PLN02252 ArrayExpress:P11832 Genevestigator:P11832 GermOnline:AT1G77760 GO:GO:0009703 Gene3D:G3DSA:2.60.40.650 Gene3D:G3DSA:3.90.420.10 SUPFAM:SSF56524 Uniprot:P11832
Root	Isotig00484	4	73	-4.319	2.04E-18	TAIR|locus:2203221 - symbol:NIA1 "AT1G77760" species:3702 "Arabidopsis thaliana" [GO:0006809 "nitric oxide biosynthetic process" evidence=IMP] [GO:0005829 "cytosol" evidence=TAS] [GO:0009416 "response to light stimulus" evidence=IMP] [GO:0005515 "protein binding" evidence=IPI] [GO:0008940 "nitrate reductase activity" evidence=IMP] [GO:0042128 "nitrate assimilation" evidence=IMP] InterPro:IPR000572 InterPro:IPR001199 InterPro:IPR001433 InterPro:IPR001709 InterPro:IPR001834 InterPro:IPR005066 InterPro:IPR008335 InterPro:IPR012137 InterPro:IPR017927 InterPro:IPR017938 InterPro:IPR018506 InterPro:IPR022407 Pfam:PF00173 Pfam:PF00174 Pfam:PF00175 Pfam:PF03404 PIRSF:PIRSF000233 PRINTS:PR00363 PRINTS:PR00371 PRINTS:PR00406 PRINTS:PR00407 PROSITE:PS00191 PROSITE:PS00559 PROSITE:PS50255 PROSITE:PS51384 InterPro:IPR008333 Pfam:PF00970 EMBL:CP002684 GenomeReviews:CT485782_GR GO:GO:0005829 GO:GO:0005515 GO:GO:0009635 GO:GO:0009055 GO:GO:0050660 GO:GO:0020037 GO:GO:0009416 Gene3D:G3DSA:3.10.120.10 SUPFAM:SSF55856 InterPro:IPR014756 SUPFAM:SSF81296 GO:GO:0043546 eggNOG:COG0543 SUPFAM:SSF63380

Tissue	Identification	# of reads (stress)	# of reads (control)	Fold Change	p-value	Description
						GO:GO:0006809 GO:GO:0042128 EMBL:AC012193 GO:GO:0030151 KO:K00360 EMBL:Z19050 EMBL:AY090950 EMBL:AF424624 EMBL:BT000989 EMBL:X13434 EMBL:X13436 IPI:IPI00518959 PIR:E96807 PIR:S35228 RefSeq:NP_177899.1 UniGene:At.17771 ProteinModelPortal:P11832 SMR:P11832 IntAct:P11832 STRING:P11832 PRIDE:P11832 EnsemblPlants:AT1G77760.1 GeneID:844112 KEGG:ath:AT1G77760 TAIR:At1g77760 HOGENOM:HBG326850 InParanoid:P11832 OMA:HGAVPKA PhylomeDB:P11832 ProtClustDB:PLN02252 ArrayExpress:P11832 Genevestigator:P11832 GermOnline:AT1G77760 GO:GO:0009703 Gene3D:G3DSA:2.60.40.650 Gene3D:G3DSA:3.90.420.10 SUPFAM:SSF56524 Uniprot:P11832
Root	Isotig00485	1	27	-4.884	4.78E-08	TAIR|locus:2203221 - symbol:NIA1 "AT1G77760" species:3702 "Arabidopsis thaliana" [GO:0006809 "nitric oxide biosynthetic process" evidence=IMP] [GO:0005829 "cytosol" evidence=TAS] [GO:0009416 "response to light stimulus" evidence=IMP] [GO:0005515 "protein binding" evidence=IPI] [GO:0008940 "nitrate reductase activity" evidence=IMP] [GO:0042128 "nitrate assimilation" evidence=IMP] InterPro:IPR000572 InterPro:IPR001199 InterPro:IPR001433 InterPro:IPR001709 InterPro:IPR001834 InterPro:IPR005066 InterPro:IPR008335 InterPro:IPR012137 InterPro:IPR017927 InterPro:IPR017938 InterPro:IPR018506 InterPro:IPR022407 Pfam:PF00173 Pfam:PF00174 Pfam:PF00175 Pfam:PF03404 PIRSF:PIRSF000233 PRINTS:PR00363 PRINTS:PR00371 PRINTS:PR00406 PRINTS:PR00407 PROSITE:PS00191 PROSITE:PS00559 PROSITE:PS50255 PROSITE:PS51384 InterPro:IPR008333 Pfam:PF00970 EMBL:CP002684 GenomeReviews:CT485782_GR GO:GO:0005829 GO:GO:0005515 GO:GO:0009635 GO:GO:0009055 GO:GO:0050660 GO:GO:0020037 GO:GO:0009416 Gene3D:G3DSA:3.10.120.10 SUPFAM:SSF55856 InterPro:IPR014756 SUPFAM:SSF81296 GO:GO:0043546 eggNOG:COG0543 SUPFAM:SSF63380 GO:GO:0006809 GO:GO:0042128 EMBL:AC012193 GO:GO:0030151 KO:K00360 EMBL:Z19050 EMBL:AY090950 EMBL:AF424624 EMBL:BT000989 EMBL:X13434 EMBL:X13436 IPI:IPI00518959 PIR:E96807 PIR:S35228 RefSeq:NP_177899.1 UniGene:At.17771 ProteinModelPortal:P11832 SMR:P11832 IntAct:P11832 STRING:P11832 PRIDE:P11832 EnsemblPlants:AT1G77760.1 GeneID:844112 KEGG:ath:AT1G77760 TAIR:At1g77760 HOGENOM:HBG326850 InParanoid:P11832 OMA:HGAVPKA PhylomeDB:P11832 ProtClustDB:PLN02252 ArrayExpress:P11832 Genevestigator:P11832 GermOnline:AT1G77760 GO:GO:0009703 Gene3D:G3DSA:2.60.40.650 Gene3D:G3DSA:3.90.420.10 SUPFAM:SSF56524 Uniprot:P11832
Root	Isotig00486	1	25	-4.773	1.66E-07	TAIR|locus:2203221 - symbol:NIA1 "AT1G77760" species:3702 "Arabidopsis thaliana" [GO:0006809 "nitric oxide biosynthetic process" evidence=IMP] [GO:0005829 "cytosol" evidence=TAS] [GO:0009416 "response to light stimulus" evidence=IMP] [GO:0005515 "protein binding" evidence=IPI] [GO:0008940 "nitrate reductase activity" evidence=IMP] [GO:0042128 "nitrate assimilation" evidence=IMP] InterPro:IPR000572 InterPro:IPR001199 InterPro:IPR001433 InterPro:IPR001709 InterPro:IPR001834 InterPro:IPR005066 InterPro:IPR008335 InterPro:IPR012137 InterPro:IPR017927 InterPro:IPR017938 InterPro:IPR018506 InterPro:IPR022407 Pfam:PF00173 Pfam:PF00174 Pfam:PF00175 Pfam:PF03404 PIRSF:PIRSF000233 PRINTS:PR00363 PRINTS:PR00371 PRINTS:PR00406 PRINTS:PR00407 PROSITE:PS00191 PROSITE:PS00559 PROSITE:PS50255 PROSITE:PS51384 InterPro:IPR008333 Pfam:PF00970 EMBL:CP002684 GenomeReviews:CT485782_GR GO:GO:0005829 GO:GO:0005515 GO:GO:0009635 GO:GO:0009055 GO:GO:0050660 GO:GO:0020037 GO:GO:0009416 Gene3D:G3DSA:3.10.120.10 SUPFAM:SSF55856 InterPro:IPR014756 SUPFAM:SSF81296 GO:GO:0043546 eggNOG:COG0543 SUPFAM:SSF63380 GO:GO:0006809 GO:GO:0042128 EMBL:AC012193 GO:GO:0030151 KO:K00360 EMBL:Z19050 EMBL:AY090950 EMBL:AF424624 EMBL:BT000989 EMBL:X13434 EMBL:X13436 IPI:IPI00518959 PIR:E96807 PIR:S35228 RefSeq:NP_177899.1 UniGene:At.17771 ProteinModelPortal:P11832 SMR:P11832 IntAct:P11832 STRING:P11832 PRIDE:P11832 EnsemblPlants:AT1G77760.1 GeneID:844112 KEGG:ath:AT1G77760 TAIR:At1g77760 HOGENOM:HBG326850 InParanoid:P11832 OMA:HGAVPKA PhylomeDB:P11832 ProtClustDB:PLN02252 ArrayExpress:P11832 Genevestigator:P11832 GermOnline:AT1G77760 GO:GO:0009703 Gene3D:G3DSA:2.60.40.650 Gene3D:G3DSA:3.90.420.10 SUPFAM:SSF56524 Uniprot:P11832
Root	Isotig00490	34	8	1.958	9.61E-05	TAIR|locus:2011656 - symbol:HMG1 "AT1G76490" species:3702 "Arabidopsis thaliana" [GO:0016126 "sterol biosynthetic process" evidence=IMP] [GO:0005777 "peroxisome" evidence=IDA] [GO:0009536 "plastid" evidence=IDA] [GO:0005515 "protein binding" evidence=IPI] [GO:0005789 "endoplasmic reticulum membrane" evidence=IDA] [GO:0019287 "isopentenyl diphosphate biosynthetic process, mevalonate pathway" evidence=IDA] [GO:0042282 "hydroxymethylglutaryl-CoA reductase activity" evidence=IGI;ISS;IDA] [GO:0060964 "regulation of gene silencing by miRNA" evidence=IMP] [GO:0008299 "isoprenoid biosynthetic process" evidence=ISS;TAS] [GO:0016020 "membrane" evidence=TAS] [GO:0005783 "endoplasmic reticulum" evidence=IDA]

Tissue	Identification	# of reads (stress)	# of reads (control)	Fold Change	p-value	Description
						InterPro:IPR002202 InterPro:IPR004554 InterPro:IPR009023 InterPro:IPR009029 InterPro:IPR023074 InterPro:IPR023076 InterPro:IPR023282 Pfam:PF00368 PRINTS:PR00071 PROSITE:PS00066 PROSITE:PS00318 PROSITE:PS01192 PROSITE:PS50065 GO:GO:0016021 EMBL:CP002684 GenomeReviews:CT485782_GR GO:GO:0005515 GO:GO:0005789 GO:GO:0050661 GO:GO:0016126 GO:GO:0015936 EMBL:AC012394 EMBL:AC015450 GO:GO:0019287 EMBL:X15032 EMBL:J04537 EMBL:L19261 EMBL:AY488113 EMBL:AF385690 EMBL:BT000703 EMBL:BT010468 IPI:IPI00545411 IPI:IPI01018461 PIR:A32107 RefSeq:NP_177775.2 UniGene:At.22772 ProteinModelPortal:P14891 SMR:P14891 STRING:P14891 PRIDE:P14891 GeneID:843982 KEGG:ath:AT1G76490 TAIR:At1g76490 eggNOG:COG1257 InParanoid:P14891 KO:K00021 PhylomeDB:P14891 ProtClustDB:CLSN2683887 BioCyc:ARA:AT1G76490-MONOMER BioCyc:MetaCyc:AT1G76490-MONOMER Genevestigator:P14891 GO:GO:0004420 GO:GO:0042282 Gene3D:G3DSA:3.30.70.420 Gene3D:G3DSA:3.90.770.10 Gene3D:G3DSA:1.10.3270.10 PANTHER:PTHR10572 SUPFAM:SSF55035 SUPFAM:SSF56542 TIGRFAMs:TIGR00533 Uniprot:P14891
Root	Isotig00491	40	11	1.734	9.88E-05	TAIR|locus:2011656 - symbol:HMG1 "AT1G76490" species:3702 "Arabidopsis thaliana" [GO:0016126 "sterol biosynthetic process" evidence=IMP] [GO:0005777 "peroxisome" evidence=IDA] [GO:0009536 "plastid" evidence=IDA] [GO:0005515 "protein binding" evidence=IPI] [GO:0005789 "endoplasmic reticulum membrane" evidence=IDA] [GO:0019287 "isopentenyl diphosphate biosynthetic process, mevalonate pathway" evidence=IDA] [GO:0042282 "hydroxymethylglutaryl-CoA reductase activity" evidence=IGI;ISS;IDA] [GO:0060964 "regulation of gene silencing by miRNA" evidence=IMP] [GO:0008299 "isoprenoid biosynthetic process" evidence=ISS;TAS] [GO:0016020 "membrane" evidence=TAS] [GO:0005783 "endoplasmic reticulum" evidence=IDA] InterPro:IPR002202 InterPro:IPR004554 InterPro:IPR009023 InterPro:IPR009029 InterPro:IPR023074 InterPro:IPR023076 InterPro:IPR023282 Pfam:PF00368 PRINTS:PR00071 PROSITE:PS00066 PROSITE:PS00318 PROSITE:PS01192 PROSITE:PS50065 GO:GO:0016021 EMBL:CP002684 GenomeReviews:CT485782_GR GO:GO:0005515 GO:GO:0005789 GO:GO:0050661 GO:GO:0016126 GO:GO:0015936 EMBL:AC012394 EMBL:AC015450 GO:GO:0019287 EMBL:X15032 EMBL:J04537 EMBL:L19261 EMBL:AY488113 EMBL:AF385690 EMBL:BT000703 EMBL:BT010468 IPI:IPI00545411 IPI:IPI01018461 PIR:A32107 RefSeq:NP_177775.2 UniGene:At.22772 ProteinModelPortal:P14891 SMR:P14891 STRING:P14891 PRIDE:P14891 GeneID:843982 KEGG:ath:AT1G76490 TAIR:At1g76490 eggNOG:COG1257 InParanoid:P14891 KO:K00021 PhylomeDB:P14891 ProtClustDB:CLSN2683887 BioCyc:ARA:AT1G76490-MONOMER BioCyc:MetaCyc:AT1G76490-MONOMER Genevestigator:P14891 GO:GO:0004420 GO:GO:0042282 Gene3D:G3DSA:3.30.70.420 Gene3D:G3DSA:3.90.770.10 Gene3D:G3DSA:1.10.3270.10 PANTHER:PTHR10572 SUPFAM:SSF55035 SUPFAM:SSF56542 TIGRFAMs:TIGR00533 Uniprot:P14891
Root	Isotig00493	40	11	1.734	9.88E-05	TAIR|locus:2011656 - symbol:HMG1 "AT1G76490" species:3702 "Arabidopsis thaliana" [GO:0016126 "sterol biosynthetic process" evidence=IMP] [GO:0005777 "peroxisome" evidence=IDA] [GO:0009536 "plastid" evidence=IDA] [GO:0005515 "protein binding" evidence=IPI] [GO:0005789 "endoplasmic reticulum membrane" evidence=IDA] [GO:0019287 "isopentenyl diphosphate biosynthetic process, mevalonate pathway" evidence=IDA] [GO:0042282 "hydroxymethylglutaryl-CoA reductase activity" evidence=IGI;ISS;IDA] [GO:0060964 "regulation of gene silencing by miRNA" evidence=IMP] [GO:0008299 "isoprenoid biosynthetic process" evidence=ISS;TAS] [GO:0016020 "membrane" evidence=TAS] [GO:0005783 "endoplasmic reticulum" evidence=IDA] InterPro:IPR002202 InterPro:IPR004554 InterPro:IPR009023 InterPro:IPR009029 InterPro:IPR023074 InterPro:IPR023076 InterPro:IPR023282 Pfam:PF00368 PRINTS:PR00071 PROSITE:PS00066 PROSITE:PS00318 PROSITE:PS01192 PROSITE:PS50065 GO:GO:0016021 EMBL:CP002684 GenomeReviews:CT485782_GR GO:GO:0005515 GO:GO:0005789 GO:GO:0050661 GO:GO:0016126 GO:GO:0015936 EMBL:AC012394 EMBL:AC015450 GO:GO:0019287 EMBL:X15032 EMBL:J04537 EMBL:L19261 EMBL:AY488113 EMBL:AF385690 EMBL:BT000703 EMBL:BT010468 IPI:IPI00545411 IPI:IPI01018461 PIR:A32107 RefSeq:NP_177775.2 UniGene:At.22772 ProteinModelPortal:P14891 SMR:P14891 STRING:P14891 PRIDE:P14891 GeneID:843982 KEGG:ath:AT1G76490 TAIR:At1g76490 eggNOG:COG1257 InParanoid:P14891 KO:K00021 PhylomeDB:P14891 ProtClustDB:CLSN2683887 BioCyc:ARA:AT1G76490-MONOMER BioCyc:MetaCyc:AT1G76490-MONOMER Genevestigator:P14891 GO:GO:0004420 GO:GO:0042282 Gene3D:G3DSA:3.30.70.420 Gene3D:G3DSA:3.90.770.10 Gene3D:G3DSA:1.10.3270.10 PANTHER:PTHR10572 SUPFAM:SSF55035 SUPFAM:SSF56542 TIGRFAMs:TIGR00533 Uniprot:P14891
Root	Isotig00494	33	7	2.108	5.57E-05	TAIR|locus:2011656 - symbol:HMG1 "AT1G76490" species:3702 "Arabidopsis thaliana" [GO:0016126 "sterol biosynthetic process" evidence=IMP] [GO:0005777 "peroxisome" evidence=IDA] [GO:0009536 "plastid"

Tissue	Identification	# of reads (stress)	# of reads (control)	Fold Change	p-value	Description
						evidence=IDA] [GO:0005515 "protein binding" evidence=IPI] [GO:0005789 "endoplasmic reticulum membrane" evidence=IDA] [GO:0019287 "isopentenyl diphosphate biosynthetic process, mevalonate pathway" evidence=IDA] [GO:0042282 "hydroxymethylglutaryl-CoA reductase activity" evidence=IGI;ISS;IDA] [GO:0060964 "regulation of gene silencing by miRNA" evidence=IMP] [GO:0008299 "isoprenoid biosynthetic process" evidence=ISS;TAS] [GO:0016020 "membrane" evidence=TAS] [GO:0005783 "endoplasmic reticulum" evidence=IDA] InterPro:IPR002202 InterPro:IPR004554 InterPro:IPR009023 InterPro:IPR009029 InterPro:IPR023074 InterPro:IPR023076 InterPro:IPR023282 Pfam:PF00368 PRINTS:PR00071 PROSITE:PS00066 PROSITE:PS00318 PROSITE:PS01192 PROSITE:PS50065 GO:GO:0016021 EMBL:CP002684 GenomeReviews:CT485782_GR GO:GO:0005515 GO:GO:0005789 GO:GO:0050661 GO:GO:0016126 GO:GO:0015936 EMBL:AC012394 EMBL:AC015450 GO:GO:0019287 EMBL:X15032 EMBL:J04537 EMBL:L19261 EMBL:AY488113 EMBL:AF385690 EMBL:BT000703 EMBL:BT010468 IPI:IPI00545411 IPI:IPI01018461 PIR:A32107 RefSeq:NP_177775.2 UniGene:At.22772 ProteinModelPortal:P14891 SMR:P14891 STRING:P14891 PRIDE:P14891 GeneID:843982 KEGG:ath:AT1G76490 TAIR:At1g76490 eggNOG:COG1257 InParanoid:P14891 KO:K00021 PhylomeDB:P14891 ProtClustDB:CLSN2683887 BioCyc:ARA:AT1G76490-MONOMER BioCyc:MetaCyc:AT1G76490-MONOMER Genevestigator:P14891 GO:GO:0004420 GO:GO:0042282 Gene3D:G3DSA:3.30.70.420 Gene3D:G3DSA:3.90.770.10 Gene3D:G3DSA:1.10.3270.10 PANTHER:PTHR10572 SUPFAM:SSF55035 SUPFAM:SSF56542 TIGRFAMs:TIGR00533 Uniprot:P14891
Root	Isotig00495	39	10	1.835	6.39E-05	TAIR|locus:2011656 - symbol:HMG1 "AT1G76490" species:3702 "Arabidopsis thaliana" [GO:0016126 "sterol biosynthetic process" evidence=IMP] [GO:0005777 "peroxisome" evidence=IDA] [GO:0009536 "plastid" evidence=IDA] [GO:0005515 "protein binding" evidence=IPI] [GO:0005789 "endoplasmic reticulum membrane" evidence=IDA] [GO:0019287 "isopentenyl diphosphate biosynthetic process, mevalonate pathway" evidence=IDA] [GO:0042282 "hydroxymethylglutaryl-CoA reductase activity" evidence=IGI;ISS;IDA] [GO:0060964 "regulation of gene silencing by miRNA" evidence=IMP] [GO:0008299 "isoprenoid biosynthetic process" evidence=ISS;TAS] [GO:0016020 "membrane" evidence=TAS] [GO:0005783 "endoplasmic reticulum" evidence=IDA] InterPro:IPR002202 InterPro:IPR004554 InterPro:IPR009023 InterPro:IPR009029 InterPro:IPR023074 InterPro:IPR023076 InterPro:IPR023282 Pfam:PF00368 PRINTS:PR00071 PROSITE:PS00066 PROSITE:PS00318 PROSITE:PS01192 PROSITE:PS50065 GO:GO:0016021 EMBL:CP002684 GenomeReviews:CT485782_GR GO:GO:0005515 GO:GO:0005789 GO:GO:0050661 GO:GO:0016126 GO:GO:0015936 EMBL:AC012394 EMBL:AC015450 GO:GO:0019287 EMBL:X15032 EMBL:J04537 EMBL:L19261 EMBL:AY488113 EMBL:AF385690 EMBL:BT000703 EMBL:BT010468 IPI:IPI00545411 IPI:IPI01018461 PIR:A32107 RefSeq:NP_177775.2 UniGene:At.22772 ProteinModelPortal:P14891 SMR:P14891 STRING:P14891 PRIDE:P14891 GeneID:843982 KEGG:ath:AT1G76490 TAIR:At1g76490 eggNOG:COG1257 InParanoid:P14891 KO:K00021 PhylomeDB:P14891 ProtClustDB:CLSN2683887 BioCyc:ARA:AT1G76490-MONOMER BioCyc:MetaCyc:AT1G76490-MONOMER Genevestigator:P14891 GO:GO:0004420 GO:GO:0042282 Gene3D:G3DSA:3.30.70.420 Gene3D:G3DSA:3.90.770.10 Gene3D:G3DSA:1.10.3270.10 PANTHER:PTHR10572 SUPFAM:SSF55035 SUPFAM:SSF56542 TIGRFAMs:TIGR00533 Uniprot:P14891
Root	Isotig00496	39	10	1.835	6.39E-05	TAIR|locus:2011656 - symbol:HMG1 "AT1G76490" species:3702 "Arabidopsis thaliana" [GO:0016126 "sterol biosynthetic process" evidence=IMP] [GO:0005777 "peroxisome" evidence=IDA] [GO:0009536 "plastid" evidence=IDA] [GO:0005515 "protein binding" evidence=IPI] [GO:0005789 "endoplasmic reticulum membrane" evidence=IDA] [GO:0019287 "isopentenyl diphosphate biosynthetic process, mevalonate pathway" evidence=IDA] [GO:0042282 "hydroxymethylglutaryl-CoA reductase activity" evidence=IGI;ISS;IDA] [GO:0060964 "regulation of gene silencing by miRNA" evidence=IMP] [GO:0008299 "isoprenoid biosynthetic process" evidence=ISS;TAS] [GO:0016020 "membrane" evidence=TAS] [GO:0005783 "endoplasmic reticulum" evidence=IDA] InterPro:IPR002202 InterPro:IPR004554 InterPro:IPR009023 InterPro:IPR009029 InterPro:IPR023074 InterPro:IPR023076 InterPro:IPR023282 Pfam:PF00368 PRINTS:PR00071 PROSITE:PS00066 PROSITE:PS00318 PROSITE:PS01192 PROSITE:PS50065 GO:GO:0016021 EMBL:CP002684 GenomeReviews:CT485782_GR GO:GO:0005515 GO:GO:0005789 GO:GO:0050661 GO:GO:0016126 GO:GO:0015936 EMBL:AC012394 EMBL:AC015450 GO:GO:0019287 EMBL:X15032 EMBL:J04537 EMBL:L19261 EMBL:AY488113 EMBL:AF385690 EMBL:BT000703 EMBL:BT010468 IPI:IPI00545411 IPI:IPI01018461 PIR:A32107 RefSeq:NP_177775.2 UniGene:At.22772 ProteinModelPortal:P14891 SMR:P14891 STRING:P14891 PRIDE:P14891 GeneID:843982 KEGG:ath:AT1G76490 TAIR:At1g76490 eggNOG:COG1257 InParanoid:P14891 KO:K00021 PhylomeDB:P14891 ProtClustDB:CLSN2683887 BioCyc:ARA:AT1G76490-MONOMER BioCyc:MetaCyc:AT1G76490-MONOMER Genevestigator:P14891 GO:GO:0004420 GO:GO:0042282 Gene3D:G3DSA:3.30.70.420

Tissue	Identification	# of reads (stress)	# of reads (control)	Fold Change	p-value	Description
						Gene3D:G3DSA:3.90.770.10 Gene3D:G3DSA:1.10.3270.10 PANTHER:PTHR10572 SUPFAM:SSF55035 SUPFAM:SSF56542 TIGRFAMs:TIGR00533 Uniprot:P14891
Root	Isotig00520	19	3	2.534	0.000639389	No hit
Root	Isotig00521	16	2	2.871	0.000812389	No hit
Root	Isotig00522	19	2	3.119	0.000146225	No hit
Root	Isotig00524	19	3	2.534	0.000639389	No hit
Root	Isotig00526	16	2	2.871	0.000812389	No hit
Root	Isotig00527	19	2	3.119	0.000146225	No hit
Root	Isotig00529	18	2	3.041	0.000259481	No hit
Root	Isotig00530	17	2	2.958	0.000459627	No hit
Root	Isotig00532	15	1	3.778	0.000266457	No hit
Root	Isotig00533	18	1	4.041	4.47E-05	No hit
Root	Isotig00534	15	1	3.778	0.000266457	No hit
Root	Isotig00537	48	5	3.134	1.46E-09	TAIR|locus:2027549 - symbol:GolS2 "AT1G56600" species:3702 "Arabidopsis thaliana" [GO:0005575 "cellular_component" evidence=ND] [GO:0016051 "carbohydrate biosynthetic process" evidence=ISS] [GO:0016757 "transferase activity, transferring glycosyl groups" evidence=ISS] [GO:0016758 "transferase activity, transferring hexosyl groups" evidence=ISS] [GO:0006012 "galactose metabolic process" evidence=IDA] [GO:0006979 "response to oxidative stress" evidence=IEP] [GO:0009409 "response to cold" evidence=IMP] [GO:0009651 "response to salt stress" evidence=IMP] [GO:0047216 "inositol 3-alpha-galactosyltransferase activity" evidence=IDA] [GO:0009414 "response to water deprivation" evidence=IEP] InterPro:IPR002495 Pfam:PF01501 EMBL:CP002684 GO:GO:0006979 GO:GO:0009409 GO:GO:0009651 GO:GO:0009414 GO:GO:0006012 EMBL:AC009323 CAZy:GT8 OMA:DSQDAAN ProtClustDB:PLN00176 GO:GO:0047216 EMBL:AY050410 EMBL:AY058238 EMBL:AB062849 IPI:IPI00517274 PIR:G96607 RefSeq:NP_176053.1 UniGene:At.19795 ProteinModelPortal:Q9FXB2 SMR:Q9FXB2 STRING:Q9FXB2 PRIDE:Q9FXB2 EnsemblPlants:AT1G56600.1 GeneID:842114 KEGG:ath:AT1G56600 TAIR:At1g56600 InParanoid:Q9FXB2 PhylomeDB:Q9FXB2 Genevestigator:Q9FXB2 Uniprot:Q9FXB2
Root	Isotig00538	47	4	3.426	4.96E-10	TAIR|locus:2027549 - symbol:GolS2 "AT1G56600" species:3702 "Arabidopsis thaliana" [GO:0005575 "cellular_component" evidence=ND] [GO:0016051 "carbohydrate biosynthetic process" evidence=ISS] [GO:0016757 "transferase activity, transferring glycosyl groups" evidence=ISS] [GO:0016758 "transferase activity, transferring hexosyl groups" evidence=ISS] [GO:0006012 "galactose metabolic process" evidence=IDA] [GO:0006979 "response to oxidative stress" evidence=IEP] [GO:0009409 "response to cold" evidence=IMP] [GO:0009651 "response to salt stress" evidence=IMP] [GO:0047216 "inositol 3-alpha-galactosyltransferase activity" evidence=IDA] [GO:0009414 "response to water deprivation" evidence=IEP] InterPro:IPR002495 Pfam:PF01501 EMBL:CP002684 GO:GO:0006979 GO:GO:0009409 GO:GO:0009651 GO:GO:0009414 GO:GO:0006012 EMBL:AC009323 CAZy:GT8 OMA:DSQDAAN ProtClustDB:PLN00176 GO:GO:0047216 EMBL:AY050410 EMBL:AY058238 EMBL:AB062849 IPI:IPI00517274 PIR:G96607 RefSeq:NP_176053.1 UniGene:At.19795 ProteinModelPortal:Q9FXB2 SMR:Q9FXB2 STRING:Q9FXB2 PRIDE:Q9FXB2 EnsemblPlants:AT1G56600.1 GeneID:842114 KEGG:ath:AT1G56600 TAIR:At1g56600 InParanoid:Q9FXB2 PhylomeDB:Q9FXB2 Genevestigator:Q9FXB2 Uniprot:Q9FXB2
Root	Isotig00539	46	4	3.395	8.74E-10	TAIR|locus:2027549 - symbol:GolS2 "AT1G56600" species:3702 "Arabidopsis thaliana" [GO:0005575 "cellular_component" evidence=ND] [GO:0016051 "carbohydrate biosynthetic process" evidence=ISS] [GO:0016757 "transferase activity, transferring glycosyl groups" evidence=ISS] [GO:0016758 "transferase activity, transferring hexosyl groups" evidence=ISS] [GO:0006012 "galactose metabolic process" evidence=IDA] [GO:0006979 "response to oxidative stress" evidence=IEP] [GO:0009409 "response to cold" evidence=IMP] [GO:0009651 "response to salt stress" evidence=IMP] [GO:0047216 "inositol 3-alpha-galactosyltransferase activity" evidence=IDA] [GO:0009414 "response to water deprivation" evidence=IEP] InterPro:IPR002495 Pfam:PF01501 EMBL:CP002684 GO:GO:0006979 GO:GO:0009409 GO:GO:0009651 GO:GO:0009414 GO:GO:0006012 EMBL:AC009323 CAZy:GT8 OMA:DSQDAAN ProtClustDB:PLN00176 GO:GO:0047216 EMBL:AY050410 EMBL:AY058238 EMBL:AB062849 IPI:IPI00517274 PIR:G96607 RefSeq:NP_176053.1 UniGene:At.19795 ProteinModelPortal:Q9FXB2 SMR:Q9FXB2 STRING:Q9FXB2 PRIDE:Q9FXB2 EnsemblPlants:AT1G56600.1 GeneID:842114 KEGG:ath:AT1G56600 TAIR:At1g56600 InParanoid:Q9FXB2 PhylomeDB:Q9FXB2 Genevestigator:Q9FXB2 Uniprot:Q9FXB2

Tissue	Identification	# of reads (stress)	# of reads (control)	Fold Change	p-value	Description
Root	Isotig00540	47	4	3.426	4.96E-10	TAIR|locus:2027549 - symbol:GolS2 "AT1G56600" species:3702 "Arabidopsis thaliana" [GO:0005575 "cellular_component" evidence=ND] [GO:0016051 "carbohydrate biosynthetic process" evidence=ISS] [GO:0016757 "transferase activity, transferring glycosyl groups" evidence=ISS] [GO:0016758 "transferase activity, transferring hexosyl groups" evidence=ISS] [GO:0006012 "galactose metabolic process" evidence=IDA] [GO:0006979 "response to oxidative stress" evidence=IEP] [GO:0009409 "response to cold" evidence=IMP] [GO:0009651 "response to salt stress" evidence=IMP] [GO:0047216 "inositol 3-alpha-galactosyltransferase activity" evidence=IDA] [GO:0009414 "response to water deprivation" evidence=IEP] InterPro:IPR002495 Pfam:PF01501 EMBL:CP002684 GO:GO:0006979 GO:GO:0009409 GO:GO:0009651 GO:GO:0009414 GO:GO:0006012 EMBL:AC009323 CAZy:GT8 OMA:DSQDAAN ProtClustDB:PLN00176 GO:GO:0047216 EMBL:AY050410 EMBL:AY058238 EMBL:AB062849 IPI:IPI00517274 PIR:G96607 RefSeq:NP_176053.1 UniGene:At.19795 ProteinModelPortal:Q9FXB2 SMR:Q9FXB2 STRING:Q9FXB2 PRIDE:Q9FXB2 EnsemblPlants:AT1G56600.1 GeneID:842114 KEGG:ath:AT1G56600 TAIR:At1g56600 InParanoid:Q9FXB2 PhylomeDB:Q9FXB2 Genevestigator:Q9FXB2 Uniprot:Q9FXB2
Root	Isotig00541	46	4	3.395	8.74E-10	TAIR|locus:2027549 - symbol:GolS2 "AT1G56600" species:3702 "Arabidopsis thaliana" [GO:0005575 "cellular_component" evidence=ND] [GO:0016051 "carbohydrate biosynthetic process" evidence=ISS] [GO:0016757 "transferase activity, transferring glycosyl groups" evidence=ISS] [GO:0016758 "transferase activity, transferring hexosyl groups" evidence=ISS] [GO:0006012 "galactose metabolic process" evidence=IDA] [GO:0006979 "response to oxidative stress" evidence=IEP] [GO:0009409 "response to cold" evidence=IMP] [GO:0009651 "response to salt stress" evidence=IMP] [GO:0047216 "inositol 3-alpha-galactosyltransferase activity" evidence=IDA] [GO:0009414 "response to water deprivation" evidence=IEP] InterPro:IPR002495 Pfam:PF01501 EMBL:CP002684 GO:GO:0006979 GO:GO:0009409 GO:GO:0009651 GO:GO:0009414 GO:GO:0006012 EMBL:AC009323 CAZy:GT8 OMA:DSQDAAN ProtClustDB:PLN00176 GO:GO:0047216 EMBL:AY050410 EMBL:AY058238 EMBL:AB062849 IPI:IPI00517274 PIR:G96607 RefSeq:NP_176053.1 UniGene:At.19795 ProteinModelPortal:Q9FXB2 SMR:Q9FXB2 STRING:Q9FXB2 PRIDE:Q9FXB2 EnsemblPlants:AT1G56600.1 GeneID:842114 KEGG:ath:AT1G56600 TAIR:At1g56600 InParanoid:Q9FXB2 PhylomeDB:Q9FXB2 Genevestigator:Q9FXB2 Uniprot:Q9FXB2
Root	Isotig00542	42	1	5.263	5.01E-11	TAIR|locus:2027549 - symbol:GolS2 "AT1G56600" species:3702 "Arabidopsis thaliana" [GO:0005575 "cellular_component" evidence=ND] [GO:0016051 "carbohydrate biosynthetic process" evidence=ISS] [GO:0016757 "transferase activity, transferring glycosyl groups" evidence=ISS] [GO:0016758 "transferase activity, transferring hexosyl groups" evidence=ISS] [GO:0006012 "galactose metabolic process" evidence=IDA] [GO:0006979 "response to oxidative stress" evidence=IEP] [GO:0009409 "response to cold" evidence=IMP] [GO:0009651 "response to salt stress" evidence=IMP] [GO:0047216 "inositol 3-alpha-galactosyltransferase activity" evidence=IDA] [GO:0009414 "response to water deprivation" evidence=IEP] InterPro:IPR002495 Pfam:PF01501 EMBL:CP002684 GO:GO:0006979 GO:GO:0009409 GO:GO:0009651 GO:GO:0009414 GO:GO:0006012 EMBL:AC009323 CAZy:GT8 OMA:DSQDAAN ProtClustDB:PLN00176 GO:GO:0047216 EMBL:AY050410 EMBL:AY058238 EMBL:AB062849 IPI:IPI00517274 PIR:G96607 RefSeq:NP_176053.1 UniGene:At.19795 ProteinModelPortal:Q9FXB2 SMR:Q9FXB2 STRING:Q9FXB2 PRIDE:Q9FXB2 EnsemblPlants:AT1G56600.1 GeneID:842114 KEGG:ath:AT1G56600 TAIR:At1g56600 InParanoid:Q9FXB2 PhylomeDB:Q9FXB2 Genevestigator:Q9FXB2 Uniprot:Q9FXB2
Root	Isotig00543	41	0	6.229	8.08E-11	TAIR|locus:2027549 - symbol:GolS2 "AT1G56600" species:3702 "Arabidopsis thaliana" [GO:0005575 "cellular_component" evidence=ND] [GO:0016051 "carbohydrate biosynthetic process" evidence=ISS] [GO:0016757 "transferase activity, transferring glycosyl groups" evidence=ISS] [GO:0016758 "transferase activity, transferring hexosyl groups" evidence=ISS] [GO:0006012 "galactose metabolic process" evidence=IDA] [GO:0006979 "response to oxidative stress" evidence=IEP] [GO:0009409 "response to cold" evidence=IMP] [GO:0009651 "response to salt stress" evidence=IMP] [GO:0047216 "inositol 3-alpha-galactosyltransferase activity" evidence=IDA] [GO:0009414 "response to water deprivation" evidence=IEP] InterPro:IPR002495 Pfam:PF01501 EMBL:CP002684 GO:GO:0006979 GO:GO:0009409 GO:GO:0009651 GO:GO:0009414 GO:GO:0006012 EMBL:AC009323 CAZy:GT8 OMA:DSQDAAN ProtClustDB:PLN00176 GO:GO:0047216 EMBL:AY050410 EMBL:AY058238 EMBL:AB062849 IPI:IPI00517274 PIR:G96607 RefSeq:NP_176053.1 UniGene:At.19795 ProteinModelPortal:Q9FXB2 SMR:Q9FXB2 STRING:Q9FXB2 PRIDE:Q9FXB2 EnsemblPlants:AT1G56600.1 GeneID:842114 KEGG:ath:AT1G56600 TAIR:At1g56600 InParanoid:Q9FXB2 PhylomeDB:Q9FXB2 Genevestigator:Q9FXB2 Uniprot:Q9FXB2

Tissue	Identification	# of reads (stress)	# of reads (control)	Fold Change	p-value	Description
Root	Isotig00544	40	0	6.193	1.34E-10	TAIR|locus:2027549 - symbol:GolS2 "AT1G56600" species:3702 "Arabidopsis thaliana" [GO:0005575 "cellular_component" evidence=ND] [GO:0016051 "carbohydrate biosynthetic process" evidence=ISS] [GO:0016757 "transferase activity, transferring glycosyl groups" evidence=ISS] [GO:0016758 "transferase activity, transferring hexosyl groups" evidence=ISS] [GO:0006012 "galactose metabolic process" evidence=IDA] [GO:0006979 "response to oxidative stress" evidence=IEP] [GO:0009409 "response to cold" evidence=IMP] [GO:0009651 "response to salt stress" evidence=IMP] [GO:0047216 "inositol 3-alpha-galactosyltransferase activity" evidence=IDA] [GO:0009414 "response to water deprivation" evidence=IEP] InterPro:IPR002495 Pfam:PF01501 EMBL:CP002684 GO:GO:0006979 GO:GO:0009409 GO:GO:0009651 GO:GO:0009414 GO:GO:0006012 EMBL:AC009323 CAZy:GT8 OMA:DSQDAAN ProtClustDB:PLN00176 GO:GO:0047216 EMBL:AY050410 EMBL:AY058238 EMBL:AB062849 IPI:IPI00517274 PIR:G96607 RefSeq:NP_176053.1 UniGene:At.19795 ProteinModelPortal:Q9FXB2 SMR:Q9FXB2 STRING:Q9FXB2 PRIDE:Q9FXB2 EnsemblPlants:AT1G56600.1 GeneID:842114 KEGG:ath:AT1G56600 TAIR:At1g56600 InParanoid:Q9FXB2 PhylomeDB:Q9FXB2 Genevestigator:Q9FXB2 Uniprot:Q9FXB2
Root	Isotig00545	41	0	6.229	8.08E-11	TAIR|locus:2027549 - symbol:GolS2 "AT1G56600" species:3702 "Arabidopsis thaliana" [GO:0005575 "cellular_component" evidence=ND] [GO:0016051 "carbohydrate biosynthetic process" evidence=ISS] [GO:0016757 "transferase activity, transferring glycosyl groups" evidence=ISS] [GO:0016758 "transferase activity, transferring hexosyl groups" evidence=ISS] [GO:0006012 "galactose metabolic process" evidence=IDA] [GO:0006979 "response to oxidative stress" evidence=IEP] [GO:0009409 "response to cold" evidence=IMP] [GO:0009651 "response to salt stress" evidence=IMP] [GO:0047216 "inositol 3-alpha-galactosyltransferase activity" evidence=IDA] [GO:0009414 "response to water deprivation" evidence=IEP] InterPro:IPR002495 Pfam:PF01501 EMBL:CP002684 GO:GO:0006979 GO:GO:0009409 GO:GO:0009651 GO:GO:0009414 GO:GO:0006012 EMBL:AC009323 CAZy:GT8 OMA:DSQDAAN ProtClustDB:PLN00176 GO:GO:0047216 EMBL:AY050410 EMBL:AY058238 EMBL:AB062849 IPI:IPI00517274 PIR:G96607 RefSeq:NP_176053.1 UniGene:At.19795 ProteinModelPortal:Q9FXB2 SMR:Q9FXB2 STRING:Q9FXB2 PRIDE:Q9FXB2 EnsemblPlants:AT1G56600.1 GeneID:842114 KEGG:ath:AT1G56600 TAIR:At1g56600 InParanoid:Q9FXB2 PhylomeDB:Q9FXB2 Genevestigator:Q9FXB2 Uniprot:Q9FXB2
Root	Isotig00546	40	0	6.193	1.34E-10	TAIR|locus:2027549 - symbol:GolS2 "AT1G56600" species:3702 "Arabidopsis thaliana" [GO:0005575 "cellular_component" evidence=ND] [GO:0016051 "carbohydrate biosynthetic process" evidence=ISS] [GO:0016757 "transferase activity, transferring glycosyl groups" evidence=ISS] [GO:0016758 "transferase activity, transferring hexosyl groups" evidence=ISS] [GO:0006012 "galactose metabolic process" evidence=IDA] [GO:0006979 "response to oxidative stress" evidence=IEP] [GO:0009409 "response to cold" evidence=IMP] [GO:0009651 "response to salt stress" evidence=IMP] [GO:0047216 "inositol 3-alpha-galactosyltransferase activity" evidence=IDA] [GO:0009414 "response to water deprivation" evidence=IEP] InterPro:IPR002495 Pfam:PF01501 EMBL:CP002684 GO:GO:0006979 GO:GO:0009409 GO:GO:0009651 GO:GO:0009414 GO:GO:0006012 EMBL:AC009323 CAZy:GT8 OMA:DSQDAAN ProtClustDB:PLN00176 GO:GO:0047216 EMBL:AY050410 EMBL:AY058238 EMBL:AB062849 IPI:IPI00517274 PIR:G96607 RefSeq:NP_176053.1 UniGene:At.19795 ProteinModelPortal:Q9FXB2 SMR:Q9FXB2 STRING:Q9FXB2 PRIDE:Q9FXB2 EnsemblPlants:AT1G56600.1 GeneID:842114 KEGG:ath:AT1G56600 TAIR:At1g56600 InParanoid:Q9FXB2 PhylomeDB:Q9FXB2 Genevestigator:Q9FXB2 Uniprot:Q9FXB2
Root	Isotig00570	31	110	-1.956	2.29E-13	TAIR|locus:2031855 - symbol:PBE1 "20S proteasome beta subunit E1" species:3702 "Arabidopsis thaliana" [GO:0004175 "endopeptidase activity" evidence=IEA] [GO:0004298 "threonine-type endopeptidase activity" evidence=IEA] [GO:0008233 "peptidase activity" evidence=ISS] [GO:0006511 "ubiquitin-dependent protein catabolic process" evidence=TAS] [GO:0046686 "response to cadmium ion" evidence=IEP] [GO:0000502 "proteasome complex" evidence=IDA] [GO:0005839 "proteasome core complex" evidence=TAS] InterPro:IPR000243 InterPro:IPR001353 InterPro:IPR016050 InterPro:IPR023333 Pfam:PF00227 PRINTS:PR00141 PROSITE:PS00854 PROSITE:PS51476 EMBL:CP002684 GenomeReviews:CT485782_GR GO:GO:0005634 GO:GO:0046686 GO:GO:0005737 EMBL:AC007357 GO:GO:0004298 GO:GO:0051603 GO:GO:0005839 eggNOG:COG0638 HOGENOM:HBG499923 EMBL:Y13695 EMBL:AF043536 EMBL:AF348579 EMBL:AY050871 EMBL:AY150473 IPI:IPI00544396 PIR:F86264 RefSeq:NP_172765.1 UniGene:At.22436 ProteinModelPortal:O23717 SMR:O23717 STRING:O23717 PRIDE:O23717 EnsemblPlants:AT1G13060.1 GeneID:837863 KEGG:ath:AT1G13060 TAIR:At1g13060 InParanoid:O23717 KO:K02737 OMA:QTFAYGV PhylomeDB:O23717 ProtClustDB:CLSN2682489 Genevestigator:O23717 Uniprot:O23717

Tissue	Identification	# of reads (stress)	# of reads (control)	Fold Change	p-value	Description
Root	Isotig00571	35	112	-1.807	2.35E-12	TAIR|locus:2031855 - symbol:PBE1 "20S proteasome beta subunit E1" species:3702 "Arabidopsis thaliana" [GO:0004175 "endopeptidase activity" evidence=IEA] [GO:0004298 "threonine-type endopeptidase activity" evidence=IEA] [GO:0008233 "peptidase activity" evidence=ISS] [GO:0006511 "ubiquitin-dependent protein catabolic process" evidence=TAS] [GO:0046686 "response to cadmium ion" evidence=IEP] [GO:0000502 "proteasome complex" evidence=IDA] [GO:0005839 "proteasome core complex" evidence=TAS] InterPro:IPR000243 InterPro:IPR001353 InterPro:IPR016050 InterPro:IPR023333 Pfam:PF00227 PRINTS:PR00141 PROSITE:PS00854 PROSITE:PS51476 EMBL:CP002684 GenomeReviews:CT485782_GR GO:GO:0005634 GO:GO:0046686 GO:GO:0005737 EMBL:AC007357 GO:GO:0004298 GO:GO:0051603 GO:GO:0005839 eggNOG:COG0638 HOGENOM:HBG499923 EMBL:Y13695 EMBL:AF043536 EMBL:AF348579 EMBL:AY050871 EMBL:AY150473 IPI:IPI00544396 PIR:F86264 RefSeq:NP_172765.1 UniGene:At.22436 ProteinModelPortal:O23717 SMR:O23717 STRING:O23717 PRIDE:O23717 EnsemblPlants:AT1G13060.1 GeneID:837863 KEGG:ath:AT1G13060 TAIR:At1g13060 InParanoid:O23717 KO:K02737 OMA:QTFAYGV PhylomeDB:O23717 ProtClustDB:CLSN2682489 Genevestigator:O23717 Uniprot:O23717
Root	Isotig00574	9	53	-2.687	7.37E-10	TAIR|locus:2177003 - symbol:TUB6 "AT5G12250" species:3702 "Arabidopsis thaliana" [GO:0009409 "response to cold" evidence=IEP] [GO:0005200 "structural constituent of cytoskeleton" evidence=ISS] [GO:0007017 "microtubule-based process" evidence=ISS] [GO:0015630 "microtubule cytoskeleton" evidence=ISS] [GO:0005886 "plasma membrane" evidence=IDA] [GO:0009651 "response to salt stress" evidence=IEP] InterPro:IPR000217 InterPro:IPR002453 InterPro:IPR003008 InterPro:IPR008280 InterPro:IPR017975 InterPro:IPR018316 Pfam:PF00091 Pfam:PF03953 PRINTS:PR01161 PRINTS:PR01163 PROSITE:PS00227 SMART:SM00864 SMART:SM00865 GO:GO:0005525 GO:GO:0005886 GO:GO:0005737 EMBL:CP002688 GenomeReviews:BA000015_GR GO:GO:0005198 GO:GO:0003924 GO:GO:0009409 GO:GO:0009651 GO:GO:0005874 GO:GO:0051258 Gene3D:G3DSA:3.40.50.1440 SUPFAM:SSF52490 GO:GO:0007018 EMBL:AB007727 Gene3D:G3DSA:3.30.1330.20 SUPFAM:SSF55307 HOGENOM:HBG750007 InterPro:IPR023123 Gene3D:G3DSA:1.10.287.600 PANTHER:PTHR11588 InterPro:IPR013838 KO:K07375 ProtClustDB:PLN00220 PROSITE:PS00228 OMA:EVINDEH EMBL:M84703 EMBL:AF360260 EMBL:AY040074 IPI:IPI00545934 PIR:JQ1590 RefSeq:NP_196786.1 UniGene:At.23425 UniGene:At.72672 UniGene:At.74848 ProteinModelPortal:P29514 SMR:P29514 STRING:P29514 PRIDE:P29514 EnsemblPlants:AT5G12250.1 GeneID:831100 KEGG:ath:AT5G12250 TAIR:At5g12250 InParanoid:P29514 PhylomeDB:P29514 ArrayExpress:P29514 Genevestigator:P29514 GermOnline:AT5G12250 Uniprot:P29514
Root	Isotig00605	2	15	-3.036	0.000493759	No hit
Root	Isotig00607	2	14	-2.936	0.000925454	No hit
Root	Isotig00608	2	15	-3.036	0.000493759	No hit
Root	Isotig00609	51	10	2.222	2.25E-07	No hit
Root	Isotig00610	46	11	1.935	6.77E-06	No hit
Root	Isotig00611	52	11	2.112	4.08E-07	No hit
Root	Isotig00615	37	9	1.911	6.27E-05	No hit
Root	Isotig00617	38	10	1.797	9.99E-05	No hit
Root	Isotig00618	29	8	1.729	0.000936087	No hit
Root	Isotig00621	49	18	1.316	0.000437347	TAIR|locus:2123782 - symbol:GAMMA-VPE "AT4G32940" species:3702 "Arabidopsis thaliana" [GO:0004197 "cysteine-type endopeptidase activity" evidence=ISS;IDA] [GO:0006508 "proteolysis" evidence=ISS] [GO:0000323 "lytic vacuole" evidence=IDA] [GO:0006624 "vacuolar protein processing" evidence=ISS] InterPro:IPR001096 Pfam:PF01650 PIRSF:PIRSF019663 PRINTS:PR00776 EMBL:CP002687 GenomeReviews:CT486007_GR GO:GO:0004197 GO:GO:0006508 EMBL:AL031804 EMBL:AL161582 eggNOG:COG5206 PANTHER:PTHR12000 KO:K01369 HOGENOM:HBG318720 GO:GO:0000323 ProtClustDB:CLSN2682340 EMBL:D61395 EMBL:AF370160 EMBL:AF424619 EMBL:AY059104 EMBL:AY133531 IPI:IPI00537536 PIR:T05302 RefSeq:NP_195020.1 UniGene:At.21567 UniGene:At.48929 UniGene:At.75206 ProteinModelPortal:Q39119 STRING:Q39119 MEROPS:C13.006 PRIDE:Q39119 EnsemblPlants:AT4G32940.1 GeneID:829431 KEGG:ath:AT4G32940 TAIR:At4g32940 InParanoid:Q39119 OMA:ICHAYQL PhylomeDB:Q39119 Genevestigator:Q39119 Uniprot:Q39119
Root	Isotig00622	49	18	1.316	0.000437347	TAIR|locus:2123782 - symbol:GAMMA-VPE "AT4G32940" species:3702 "Arabidopsis thaliana" [GO:0004197 "cysteine-type endopeptidase activity" evidence=ISS;IDA] [GO:0006508 "proteolysis" evidence=ISS] [GO:0000323 "lytic vacuole" evidence=IDA] [GO:0006624 "vacuolar protein processing" evidence=ISS] InterPro:IPR001096

Tissue	Identification	# of reads (stress)	# of reads (control)	Fold Change	p-value	Description
						Pfam:PF01650 PIRSF:PIRSF019663 PRINTS:PR00776 EMBL:CP002687 GenomeReviews:CT486007_GR GO:GO:0004197 GO:GO:0006508 EMBL:AL031804 EMBL:AL161582 eggNOG:COG5206 PANTHER:PTHR12000 KO:K01369 HOGENOM:HBG318720 GO:GO:0000323 ProtClustDB:CLSN2682340 EMBL:D61395 EMBL:AF370160 EMBL:AF424619 EMBL:AY059104 EMBL:AY133531 IPI:IPI00537536 PIR:T05302 RefSeq:NP_195020.1 UniGene:At.21567 UniGene:At.48929 UniGene:At.75206 ProteinModelPortal:Q39119 STRING:Q39119 MEROPS:C13.006 PRIDE:Q39119 EnsemblPlants:AT4G32940.1 GeneID:829431 KEGG:ath:AT4G32940 TAIR:At4g32940 InParanoid:Q39119 OMA:ICHAYQL PhylomeDB:Q39119 Genevestigator:Q39119 Uniprot:Q39119
Root	Isotig00625	49	16	1.486	0.000115562	TAIR|locus:2123782 - symbol:GAMMA-VPE "AT4G32940" species:3702 "Arabidopsis thaliana" [GO:0004197 "cysteine-type endopeptidase activity" evidence=ISS;IDA] [GO:0006508 "proteolysis" evidence=ISS] [GO:0000323 "lytic vacuole" evidence=IDA] [GO:0006624 "vacuolar protein processing" evidence=ISS] InterPro:IPR001096 Pfam:PF01650 PIRSF:PIRSF019663 PRINTS:PR00776 EMBL:CP002687 GenomeReviews:CT486007_GR GO:GO:0004197 GO:GO:0006508 EMBL:AL031804 EMBL:AL161582 eggNOG:COG5206 PANTHER:PTHR12000 KO:K01369 HOGENOM:HBG318720 GO:GO:0000323 ProtClustDB:CLSN2682340 EMBL:D61395 EMBL:AF370160 EMBL:AF424619 EMBL:AY059104 EMBL:AY133531 IPI:IPI00537536 PIR:T05302 RefSeq:NP_195020.1 UniGene:At.21567 UniGene:At.48929 UniGene:At.75206 ProteinModelPortal:Q39119 STRING:Q39119 MEROPS:C13.006 PRIDE:Q39119 EnsemblPlants:AT4G32940.1 GeneID:829431 KEGG:ath:AT4G32940 TAIR:At4g32940 InParanoid:Q39119 OMA:ICHAYQL PhylomeDB:Q39119 Genevestigator:Q39119 Uniprot:Q39119
Root	Isotig00626	49	16	1.486	0.000115562	TAIR|locus:2123782 - symbol:GAMMA-VPE "AT4G32940" species:3702 "Arabidopsis thaliana" [GO:0004197 "cysteine-type endopeptidase activity" evidence=ISS;IDA] [GO:0006508 "proteolysis" evidence=ISS] [GO:0000323 "lytic vacuole" evidence=IDA] [GO:0006624 "vacuolar protein processing" evidence=ISS] InterPro:IPR001096 Pfam:PF01650 PIRSF:PIRSF019663 PRINTS:PR00776 EMBL:CP002687 GenomeReviews:CT486007_GR GO:GO:0004197 GO:GO:0006508 EMBL:AL031804 EMBL:AL161582 eggNOG:COG5206 PANTHER:PTHR12000 KO:K01369 HOGENOM:HBG318720 GO:GO:0000323 ProtClustDB:CLSN2682340 EMBL:D61395 EMBL:AF370160 EMBL:AF424619 EMBL:AY059104 EMBL:AY133531 IPI:IPI00537536 PIR:T05302 RefSeq:NP_195020.1 UniGene:At.21567 UniGene:At.48929 UniGene:At.75206 ProteinModelPortal:Q39119 STRING:Q39119 MEROPS:C13.006 PRIDE:Q39119 EnsemblPlants:AT4G32940.1 GeneID:829431 KEGG:ath:AT4G32940 TAIR:At4g32940 InParanoid:Q39119 OMA:ICHAYQL PhylomeDB:Q39119 Genevestigator:Q39119 Uniprot:Q39119
Root	Isotig00645	54	8	2.626	4.34E-09	TAIR|locus:2051008 - symbol:AT2G33590 species:3702 "Arabidopsis thaliana" [GO:0003824 "catalytic activity" evidence=IEA] [GO:0005575 "cellular_component" evidence=ND] [GO:0009809 "lignin biosynthetic process" evidence=ISS] [GO:0016621 "cinnamoyl-CoA reductase activity" evidence=ISS] [GO:0050662 "coenzyme binding" evidence=IEA] [GO:0046686 "response to cadmium ion" evidence=IEP] InterPro:IPR001509 InterPro:IPR016040 Pfam:PF01370 GO:GO:0046686 EMBL:CP002685 GenomeReviews:CT485783_GR eggNOG:COG0451 GO:GO:0000166 Gene3D:G3DSA:3.40.50.720 HOGENOM:HBG755066 GO:GO:0003824 GO:GO:0044237 GO:GO:0050662 EMBL:AC002332 EMBL:AY120714 EMBL:AY084584 EMBL:BT000055 IPI:IPI00536249 PIR:D84747 RefSeq:NP_180917.1 UniGene:At.19951 ProteinModelPortal:O22809 SMR:O22809 STRING:O22809 PRIDE:O22809 EnsemblPlants:AT2G33590.1 GeneID:817925 KEGG:ath:AT2G33590 TAIR:At2g33590 InParanoid:O22809 OMA:CLAKTRA PhylomeDB:O22809 ProtClustDB:CLSN2683499 ArrayExpress:O22809 Genevestigator:O22809 Uniprot:O22809
Root	Isotig00646	55	8	2.652	2.57E-09	TAIR|locus:2051008 - symbol:AT2G33590 species:3702 "Arabidopsis thaliana" [GO:0003824 "catalytic activity" evidence=IEA] [GO:0005575 "cellular_component" evidence=ND] [GO:0009809 "lignin biosynthetic process" evidence=ISS] [GO:0016621 "cinnamoyl-CoA reductase activity" evidence=ISS] [GO:0050662 "coenzyme binding" evidence=IEA] [GO:0046686 "response to cadmium ion" evidence=IEP] InterPro:IPR001509 InterPro:IPR016040 Pfam:PF01370 GO:GO:0046686 EMBL:CP002685 GenomeReviews:CT485783_GR eggNOG:COG0451 GO:GO:0000166 Gene3D:G3DSA:3.40.50.720 HOGENOM:HBG755066 GO:GO:0003824 GO:GO:0044237 GO:GO:0050662 EMBL:AC002332 EMBL:AY120714 EMBL:AY084584 EMBL:BT000055 IPI:IPI00536249 PIR:D84747 RefSeq:NP_180917.1 UniGene:At.19951 ProteinModelPortal:O22809 SMR:O22809 STRING:O22809 PRIDE:O22809 EnsemblPlants:AT2G33590.1 GeneID:817925 KEGG:ath:AT2G33590 TAIR:At2g33590 InParanoid:O22809 OMA:CLAKTRA PhylomeDB:O22809 ProtClustDB:CLSN2683499 ArrayExpress:O22809 Genevestigator:O22809 Uniprot:O22809

Tissue	Identification	# of reads (stress)	# of reads (control)	Fold Change	p-value	Description
Root	Isotig00647	53	4	3.599	1.65E-11	SGD|S000028422 - symbol:TAR1 "Mitochondrial protein potentially involved in respiratory metabolism" species:4932 "Saccharomyces cerevisiae" [GO:0003674 "molecular_function" evidence=ND] [GO:0043457 "regulation of cellular respiration" evidence=IGI] [GO:0005739 "mitochondrion" evidence=IEA;IMP] SGD:S000028422 GO:GO:0005739 EMBL:BK006945 EMBL:U53879 GO:GO:0043457 EMBL:Z73326 EMBL:AF479964 RefSeq:NP_690845.1 ProteinModelPortal:Q8TGM6 STRING:Q8TGM6 EnsemblFungi:YLR154W-C GeneID:1466405 KEGG:sce:YLR154W-C CYGD:YLR154w-c eggNOG:NOG79861 OMA:SCSKEHR OrthoDB:EOG466ZX8 NextBio:967146 Genevestigator:Q8TGM6 GermOnline:YLR154W-C Uniprot:Q8TGM6
Root	Isotig00648	49	2	4.486	4.48E-12	SGD|S000028422 - symbol:TAR1 "Mitochondrial protein potentially involved in respiratory metabolism" species:4932 "Saccharomyces cerevisiae" [GO:0003674 "molecular_function" evidence=ND] [GO:0043457 "regulation of cellular respiration" evidence=IGI] [GO:0005739 "mitochondrion" evidence=IEA;IMP] SGD:S000028422 GO:GO:0005739 EMBL:BK006945 EMBL:U53879 GO:GO:0043457 EMBL:Z73326 EMBL:AF479964 RefSeq:NP_690845.1 ProteinModelPortal:Q8TGM6 STRING:Q8TGM6 EnsemblFungi:YLR154W-C GeneID:1466405 KEGG:sce:YLR154W-C CYGD:YLR154w-c eggNOG:NOG79861 OMA:SCSKEHR OrthoDB:EOG466ZX8 NextBio:967146 Genevestigator:Q8TGM6 GermOnline:YLR154W-C Uniprot:Q8TGM6
Root	Isotig00649	30	8	1.778	0.000599962	TAIR|locus:2051008 - symbol:AT2G33590 species:3702 "Arabidopsis thaliana" [GO:0003824 "catalytic activity" evidence=IEA] [GO:0005575 "cellular_component" evidence=ND] [GO:0009809 "lignin biosynthetic process" evidence=ISS] [GO:0016621 "cinnamoyl-CoA reductase activity" evidence=ISS] [GO:0050662 "coenzyme binding" evidence=IEA] [GO:0046686 "response to cadmium ion" evidence=IEP] InterPro:IPR001509 InterPro:IPR016040 Pfam:PF01370 GO:GO:0046686 EMBL:CP002685 GenomeReviews:CT485783_GR eggNOG:COG0451 GO:GO:0000166 Gene3D:G3DSA:3.40.50.720 HOGENOM:HBG755066 GO:GO:0003824 GO:GO:0044237 GO:GO:0050662 EMBL:AC002332 EMBL:AY120714 EMBL:AY084584 EMBL:BT000055 IPI:IPI00536249 PIR:D84747 RefSeq:NP_180917.1 UniGene:At.19951 ProteinModelPortal:O22809 SMR:O22809 STRING:O22809 PRIDE:O22809 EnsemblPlants:AT2G33590.1 GeneID:817925 KEGG:ath:AT2G33590 TAIR:At2g33590 InParanoid:O22809 OMA:CLAKTRA PhylomeDB:O22809 ProtClustDB:CLSN2683499 ArrayExpress:O22809 Genevestigator:O22809 Uniprot:O22809
Root	Isotig00651	31	8	1.825	0.000382406	TAIR|locus:2051008 - symbol:AT2G33590 species:3702 "Arabidopsis thaliana" [GO:0003824 "catalytic activity" evidence=IEA] [GO:0005575 "cellular_component" evidence=ND] [GO:0009809 "lignin biosynthetic process" evidence=ISS] [GO:0016621 "cinnamoyl-CoA reductase activity" evidence=ISS] [GO:0050662 "coenzyme binding" evidence=IEA] [GO:0046686 "response to cadmium ion" evidence=IEP] InterPro:IPR001509 InterPro:IPR016040 Pfam:PF01370 GO:GO:0046686 EMBL:CP002685 GenomeReviews:CT485783_GR eggNOG:COG0451 GO:GO:0000166 Gene3D:G3DSA:3.40.50.720 HOGENOM:HBG755066 GO:GO:0003824 GO:GO:0044237 GO:GO:0050662 EMBL:AC002332 EMBL:AY120714 EMBL:AY084584 EMBL:BT000055 IPI:IPI00536249 PIR:D84747 RefSeq:NP_180917.1 UniGene:At.19951 ProteinModelPortal:O22809 SMR:O22809 STRING:O22809 PRIDE:O22809 EnsemblPlants:AT2G33590.1 GeneID:817925 KEGG:ath:AT2G33590 TAIR:At2g33590 InParanoid:O22809 OMA:CLAKTRA PhylomeDB:O22809 ProtClustDB:CLSN2683499 ArrayExpress:O22809 Genevestigator:O22809 Uniprot:O22809
Root	Isotig00654	29	4	2.729	1.12E-05	RGD|727924 - symbol:LOC257642 "rRNA promoter binding protein" species:10116 "Rattus norvegicus" [GO:0003690 "double-stranded DNA binding" evidence=IDA] [GO:0003700 "sequence-specific DNA binding transcription factor activity" evidence=IDA] [GO:0008134 "transcription factor binding" evidence=IDA] [GO:0008283 "cell proliferation" evidence=IDA] [GO:0009303 "rRNA transcription" evidence=IDA] RGD:727924 GO:GO:0008134 GO:GO:0008283 GO:GO:0003700 GO:GO:0003690 GO:GO:0009303 EMBL:U77931 IPI:IPI00195015 RefSeq:NP_671477.1 GeneID:257642 KEGG:rno:257642 NextBio:624170 Genevestigator:Q99JC0 Uniprot:Q99JC0
Root	Isotig00655	25	2	3.515	4.58E-06	RGD|727924 - symbol:LOC257642 "rRNA promoter binding protein" species:10116 "Rattus norvegicus" [GO:0003690 "double-stranded DNA binding" evidence=IDA] [GO:0003700 "sequence-specific DNA binding transcription factor activity" evidence=IDA] [GO:0008134 "transcription factor binding" evidence=IDA] [GO:0008283 "cell proliferation" evidence=IDA] [GO:0009303 "rRNA transcription" evidence=IDA] RGD:727924 GO:GO:0008134 GO:GO:0008283 GO:GO:0003700 GO:GO:0003690 GO:GO:0009303 EMBL:U77931 IPI:IPI00195015 RefSeq:NP_671477.1 GeneID:257642 KEGG:rno:257642 NextBio:624170 Genevestigator:Q99JC0 Uniprot:Q99JC0
Root	Isotig00656	21	54	-1.492	2.19E-05	TAIR|locus:2159223 - symbol:CNX1 "calnexin 1" species:3702 "Arabidopsis thaliana" [GO:0005509 "calcium ion binding" evidence=IEA] [GO:0006457 "protein folding" evidence=IEA] [GO:0051082 "unfolded protein binding" evidence=IEA] [GO:0005739 "mitochondrion" evidence=IDA] [GO:0005773 "vacuole" evidence=IDA] [GO:0009505 "plant-type cell wall" evidence=IDA] [GO:0005783 "endoplasmic reticulum" evidence=IDA] [GO:0005774 "vacuolar

Tissue	Identification	# of reads (stress)	# of reads (control)	Fold Change	p-value	Description
						membrane" evidence=IDA] [GO:0005886 "plasma membrane" evidence=IDA] [GO:0016020 "membrane" evidence=IDA] [GO:0009507 "chloroplast" evidence=IDA] [GO:0009506 "plasmodesma" evidence=IDA] [GO:0005794 "Golgi apparatus" evidence=RCA] [GO:0005792 "microsome" evidence=IDA] InterPro:IPR001580 InterPro:IPR009033 InterPro:IPR018124 Pfam:PF00262 PRINTS:PR00626 PROSITE:PS00803 PROSITE:PS00804 PROSITE:PS00805 GO:GO:0016021 GO:GO:0005739 GO:GO:0005886 GO:GO:0009506 GO:GO:0005774 EMBL:CP002688 GenomeReviews:BA000015_GR GO:GO:0009507 GO:GO:0005509 GO:GO:0006457 GO:GO:0005789 GO:GO:0005529 GO:GO:0051082 EMBL:AB010069 InterPro:IPR008985 InterPro:IPR013320 Gene3D:G3DSA:2.60.120.200 SUPFAM:SSF49899 GO:GO:0009505 eggNOG:NOG305105 HOGENOM:HBG444251 PANTHER:PTHR11073 SUPFAM:SSF63887 EMBL:Z18242 EMBL:AY059880 EMBL:AY114669 EMBL:AY086864 IPI:IPI00539561 PIR:JN0597 RefSeq:NP_200987.1 UniGene:At.21770 ProteinModelPortal:P29402 SMR:P29402 STRING:P29402 PRIDE:P29402 EnsemblPlants:AT5G61790.1 GeneID:836301 KEGG:ath:AT5G61790 TAIR:At5g61790 InParanoid:P29402 KO:K08054 OMA:DYKGIWK PhylomeDB:P29402 ProtClustDB:CLSN2686945 ArrayExpress:P29402 Genevestigator:P29402 GermOnline:AT5G61790 Gene3D:G3DSA:2.10.250.10 Uniprot:P29402
Root	Isotig00657	21	54	-1.492	2.19E-05	TAIR|locus:2159223 - symbol:CNX1 "calnexin 1" species:3702 "Arabidopsis thaliana" [GO:0005509 "calcium ion binding" evidence=IEA] [GO:0006457 "protein folding" evidence=IEA] [GO:0051082 "unfolded protein binding" evidence=IEA] [GO:0005739 "mitochondrion" evidence=IDA] [GO:0005773 "vacuole" evidence=IDA] [GO:0009505 "plant-type cell wall" evidence=IDA] [GO:0005783 "endoplasmic reticulum" evidence=IDA] [GO:0005774 "vacuolar membrane" evidence=IDA] [GO:0005886 "plasma membrane" evidence=IDA] [GO:0016020 "membrane" evidence=IDA] [GO:0009507 "chloroplast" evidence=IDA] [GO:0009506 "plasmodesma" evidence=IDA] [GO:0005794 "Golgi apparatus" evidence=RCA] [GO:0005792 "microsome" evidence=IDA] InterPro:IPR001580 InterPro:IPR009033 InterPro:IPR018124 Pfam:PF00262 PRINTS:PR00626 PROSITE:PS00803 PROSITE:PS00804 PROSITE:PS00805 GO:GO:0016021 GO:GO:0005739 GO:GO:0005886 GO:GO:0009506 GO:GO:0005774 EMBL:CP002688 GenomeReviews:BA000015_GR GO:GO:0009507 GO:GO:0005509 GO:GO:0006457 GO:GO:0005789 GO:GO:0005529 GO:GO:0051082 EMBL:AB010069 InterPro:IPR008985 InterPro:IPR013320 Gene3D:G3DSA:2.60.120.200 SUPFAM:SSF49899 GO:GO:0009505 eggNOG:NOG305105 HOGENOM:HBG444251 PANTHER:PTHR11073 SUPFAM:SSF63887 EMBL:Z18242 EMBL:AY059880 EMBL:AY114669 EMBL:AY086864 IPI:IPI00539561 PIR:JN0597 RefSeq:NP_200987.1 UniGene:At.21770 ProteinModelPortal:P29402 SMR:P29402 STRING:P29402 PRIDE:P29402 EnsemblPlants:AT5G61790.1 GeneID:836301 KEGG:ath:AT5G61790 TAIR:At5g61790 InParanoid:P29402 KO:K08054 OMA:DYKGIWK PhylomeDB:P29402 ProtClustDB:CLSN2686945 ArrayExpress:P29402 Genevestigator:P29402 GermOnline:AT5G61790 Gene3D:G3DSA:2.10.250.10 Uniprot:P29402
Root	Isotig00658	23	52	-1.306	0.000165922	TAIR|locus:2159223 - symbol:CNX1 "calnexin 1" species:3702 "Arabidopsis thaliana" [GO:0005509 "calcium ion binding" evidence=IEA] [GO:0006457 "protein folding" evidence=IEA] [GO:0051082 "unfolded protein binding" evidence=IEA] [GO:0005739 "mitochondrion" evidence=IDA] [GO:0005773 "vacuole" evidence=IDA] [GO:0009505 "plant-type cell wall" evidence=IDA] [GO:0005783 "endoplasmic reticulum" evidence=IDA] [GO:0005774 "vacuolar membrane" evidence=IDA] [GO:0005886 "plasma membrane" evidence=IDA] [GO:0016020 "membrane" evidence=IDA] [GO:0009507 "chloroplast" evidence=IDA] [GO:0009506 "plasmodesma" evidence=IDA] [GO:0005794 "Golgi apparatus" evidence=RCA] [GO:0005792 "microsome" evidence=IDA] InterPro:IPR001580 InterPro:IPR009033 InterPro:IPR018124 Pfam:PF00262 PRINTS:PR00626 PROSITE:PS00803 PROSITE:PS00804 PROSITE:PS00805 GO:GO:0016021 GO:GO:0005739 GO:GO:0005886 GO:GO:0009506 GO:GO:0005774 EMBL:CP002688 GenomeReviews:BA000015_GR GO:GO:0009507 GO:GO:0005509 GO:GO:0006457 GO:GO:0005789 GO:GO:0005529 GO:GO:0051082 EMBL:AB010069 InterPro:IPR008985 InterPro:IPR013320 Gene3D:G3DSA:2.60.120.200 SUPFAM:SSF49899 GO:GO:0009505 eggNOG:NOG305105 HOGENOM:HBG444251 PANTHER:PTHR11073 SUPFAM:SSF63887 EMBL:Z18242 EMBL:AY059880 EMBL:AY114669 EMBL:AY086864 IPI:IPI00539561 PIR:JN0597 RefSeq:NP_200987.1 UniGene:At.21770 ProteinModelPortal:P29402 SMR:P29402 STRING:P29402 PRIDE:P29402 EnsemblPlants:AT5G61790.1 GeneID:836301 KEGG:ath:AT5G61790 TAIR:At5g61790 InParanoid:P29402 KO:K08054 OMA:DYKGIWK PhylomeDB:P29402 ProtClustDB:CLSN2686945 ArrayExpress:P29402 Genevestigator:P29402 GermOnline:AT5G61790 Gene3D:G3DSA:2.10.250.10 Uniprot:P29402
Root	Isotig00659	23	52	-1.306	0.000165922	TAIR|locus:2159223 - symbol:CNX1 "calnexin 1" species:3702 "Arabidopsis thaliana" [GO:0005509 "calcium ion binding" evidence=IEA] [GO:0006457 "protein folding" evidence=IEA] [GO:0051082 "unfolded protein binding"

Tissue	Identification	# of reads (stress)	# of reads (control)	Fold Change	p-value	Description
						evidence=IEA] [GO:0005739 "mitochondrion" evidence=IDA] [GO:0005773 "vacuole" evidence=IDA] [GO:0009505 "plant-type cell wall" evidence=IDA] [GO:0005783 "endoplasmic reticulum" evidence=IDA] [GO:0005774 "vacuolar membrane" evidence=IDA] [GO:0005886 "plasma membrane" evidence=IDA] [GO:0016020 "membrane" evidence=IDA] [GO:0009507 "chloroplast" evidence=IDA] [GO:0009506 "plasmodesma" evidence=IDA] [GO:0005794 "Golgi apparatus" evidence=RCA] [GO:0005792 "microsome" evidence=IDA] InterPro:IPR001580 InterPro:IPR009033 InterPro:IPR018124 Pfam:PF00262 PRINTS:PR00626 PROSITE:PS00803 PROSITE:PS00804 PROSITE:PS00805 GO:GO:0016021 GO:GO:0005739 GO:GO:0005886 GO:GO:0009506 GO:GO:0005774 EMBL:CP002688 GenomeReviews:BA000015_GR GO:GO:0009507 GO:GO:0005509 GO:GO:0006457 GO:GO:0005789 GO:GO:0005529 GO:GO:0051082 EMBL:AB010069 InterPro:IPR008985 InterPro:IPR013320 Gene3D:G3DSA:2.60.120.200 SUPFAM:SSF49899 GO:GO:0009505 eggNOG:NOG305105 HOGENOM:HBG444251 PANTHER:PTHR11073 SUPFAM:SSF63887 EMBL:Z18242 EMBL:AY059880 EMBL:AY114669 EMBL:AY086864 IPI:IPI00539561 PIR:JN0597 RefSeq:NP_200987.1 UniGene:At.21770 ProteinModelPortal:P29402 SMR:P29402 STRING:P29402 PRIDE:P29402 EnsemblPlants:AT5G61790.1 GeneID:836301 KEGG:ath:AT5G61790 TAIR:At5g61790 InParanoid:P29402 KO:K08054 OMA:DYKGIWK PhylomeDB:P29402 ProtClustDB:CLSN2686945 ArrayExpress:P29402 Genevestigator:P29402 GermOnline:AT5G61790 Gene3D:G3DSA:2.10.250.10 Uniprot:P29402
Root	Isotig00660	23	52	-1.306	0.000165922	TAIR|locus:2159223 - symbol:CNX1 "calnexin 1" species:3702 "Arabidopsis thaliana" [GO:0005509 "calcium ion binding" evidence=IEA] [GO:0006457 "protein folding" evidence=IEA] [GO:0051082 "unfolded protein binding" evidence=IEA] [GO:0005739 "mitochondrion" evidence=IDA] [GO:0005773 "vacuole" evidence=IDA] [GO:0009505 "plant-type cell wall" evidence=IDA] [GO:0005783 "endoplasmic reticulum" evidence=IDA] [GO:0005774 "vacuolar membrane" evidence=IDA] [GO:0005886 "plasma membrane" evidence=IDA] [GO:0016020 "membrane" evidence=IDA] [GO:0009507 "chloroplast" evidence=IDA] [GO:0009506 "plasmodesma" evidence=IDA] [GO:0005794 "Golgi apparatus" evidence=RCA] [GO:0005792 "microsome" evidence=IDA] InterPro:IPR001580 InterPro:IPR009033 InterPro:IPR018124 Pfam:PF00262 PRINTS:PR00626 PROSITE:PS00803 PROSITE:PS00804 PROSITE:PS00805 GO:GO:0016021 GO:GO:0005739 GO:GO:0005886 GO:GO:0009506 GO:GO:0005774 EMBL:CP002688 GenomeReviews:BA000015_GR GO:GO:0009507 GO:GO:0005509 GO:GO:0006457 GO:GO:0005789 GO:GO:0005529 GO:GO:0051082 EMBL:AB010069 InterPro:IPR008985 InterPro:IPR013320 Gene3D:G3DSA:2.60.120.200 SUPFAM:SSF49899 GO:GO:0009505 eggNOG:NOG305105 HOGENOM:HBG444251 PANTHER:PTHR11073 SUPFAM:SSF63887 EMBL:Z18242 EMBL:AY059880 EMBL:AY114669 EMBL:AY086864 IPI:IPI00539561 PIR:JN0597 RefSeq:NP_200987.1 UniGene:At.21770 ProteinModelPortal:P29402 SMR:P29402 STRING:P29402 PRIDE:P29402 EnsemblPlants:AT5G61790.1 GeneID:836301 KEGG:ath:AT5G61790 TAIR:At5g61790 InParanoid:P29402 KO:K08054 OMA:DYKGIWK PhylomeDB:P29402 ProtClustDB:CLSN2686945 ArrayExpress:P29402 Genevestigator:P29402 GermOnline:AT5G61790 Gene3D:G3DSA:2.10.250.10 Uniprot:P29402
Root	Isotig00661	23	52	-1.306	0.000165922	TAIR|locus:2159223 - symbol:CNX1 "calnexin 1" species:3702 "Arabidopsis thaliana" [GO:0005509 "calcium ion binding" evidence=IEA] [GO:0006457 "protein folding" evidence=IEA] [GO:0051082 "unfolded protein binding" evidence=IEA] [GO:0005739 "mitochondrion" evidence=IDA] [GO:0005773 "vacuole" evidence=IDA] [GO:0009505 "plant-type cell wall" evidence=IDA] [GO:0005783 "endoplasmic reticulum" evidence=IDA] [GO:0005774 "vacuolar membrane" evidence=IDA] [GO:0005886 "plasma membrane" evidence=IDA] [GO:0016020 "membrane" evidence=IDA] [GO:0009507 "chloroplast" evidence=IDA] [GO:0009506 "plasmodesma" evidence=IDA] [GO:0005794 "Golgi apparatus" evidence=RCA] [GO:0005792 "microsome" evidence=IDA] InterPro:IPR001580 InterPro:IPR009033 InterPro:IPR018124 Pfam:PF00262 PRINTS:PR00626 PROSITE:PS00803 PROSITE:PS00804 PROSITE:PS00805 GO:GO:0016021 GO:GO:0005739 GO:GO:0005886 GO:GO:0009506 GO:GO:0005774 EMBL:CP002688 GenomeReviews:BA000015_GR GO:GO:0009507 GO:GO:0005509 GO:GO:0006457 GO:GO:0005789 GO:GO:0005529 GO:GO:0051082 EMBL:AB010069 InterPro:IPR008985 InterPro:IPR013320 Gene3D:G3DSA:2.60.120.200 SUPFAM:SSF49899 GO:GO:0009505 eggNOG:NOG305105 HOGENOM:HBG444251 PANTHER:PTHR11073 SUPFAM:SSF63887 EMBL:Z18242 EMBL:AY059880 EMBL:AY114669 EMBL:AY086864 IPI:IPI00539561 PIR:JN0597 RefSeq:NP_200987.1 UniGene:At.21770 ProteinModelPortal:P29402 SMR:P29402 STRING:P29402 PRIDE:P29402 EnsemblPlants:AT5G61790.1 GeneID:836301 KEGG:ath:AT5G61790 TAIR:At5g61790 InParanoid:P29402 KO:K08054 OMA:DYKGIWK PhylomeDB:P29402 ProtClustDB:CLSN2686945 ArrayExpress:P29402 Genevestigator:P29402 GermOnline:AT5G61790 Gene3D:G3DSA:2.10.250.10 Uniprot:P29402

Tissue	Identification	# of reads (stress)	# of reads (control)	Fold Change	p-value	Description
Root	Isotig00674	438	101	1.988	2.18E-45	TAIR|locus:505006460 - symbol:AHL20 "AT4G14465" species:3702 "Arabidopsis thaliana" [GO:0003674 "molecular_function" evidence=ND] [GO:0005575 "cellular_component" evidence=ND] [GO:0042742 "defense response to bacterium" evidence=IMP] EMBL:CP002687 GenomeReviews:CT486007_GR GO:GO:0042742 HOGENOM:HBG748911 InterPro:IPR005175 InterPro:IPR014476 Pfam:PF03479 PIRSF:PIRSF016021 EMBL:BT006423 EMBL:AK118702 EMBL:AK227935 EMBL:BR000356 IPI:IPI00521749 RefSeq:NP_567432.1 UniGene:At.33280 UniGene:At.67625 ProteinModelPortal:Q8GWQ2 SMR:Q8GWQ2 IntAct:Q8GWQ2 PRIDE:Q8GWQ2 EnsemblPlants:AT4G14465.1 GeneID:827093 KEGG:ath:AT4G14465 TAIR:At4g14465 eggNOG:NOG328801 InParanoid:Q8GWQ2 OMA:YAWAHAR PhylomeDB:Q8GWQ2 ProtClustDB:CLSN2917544 Genevestigator:Q8GWQ2 Uniprot:Q8GWQ2
Root	Isotig00675	438	101	1.988	2.18E-45	TAIR|locus:505006460 - symbol:AHL20 "AT4G14465" species:3702 "Arabidopsis thaliana" [GO:0003674 "molecular_function" evidence=ND] [GO:0005575 "cellular_component" evidence=ND] [GO:0042742 "defense response to bacterium" evidence=IMP] EMBL:CP002687 GenomeReviews:CT486007_GR GO:GO:0042742 HOGENOM:HBG748911 InterPro:IPR005175 InterPro:IPR014476 Pfam:PF03479 PIRSF:PIRSF016021 EMBL:BT006423 EMBL:AK118702 EMBL:AK227935 EMBL:BR000356 IPI:IPI00521749 RefSeq:NP_567432.1 UniGene:At.33280 UniGene:At.67625 ProteinModelPortal:Q8GWQ2 SMR:Q8GWQ2 IntAct:Q8GWQ2 PRIDE:Q8GWQ2 EnsemblPlants:AT4G14465.1 GeneID:827093 KEGG:ath:AT4G14465 TAIR:At4g14465 eggNOG:NOG328801 InParanoid:Q8GWQ2 OMA:YAWAHAR PhylomeDB:Q8GWQ2 ProtClustDB:CLSN2917544 Genevestigator:Q8GWQ2 Uniprot:Q8GWQ2
Root	Isotig00676	469	144	1.575	1.37E-35	UNIPROTKB|Q70KG5 - symbol:RAFTIN1A "Protein RAFTIN 1A" species:4565 "Triticum aestivum" [GO:0009555 "pollen development" evidence=ISS] [GO:0043668 "exine" evidence=IDA] GO:GO:0009555 InterPro:IPR004873 Pfam:PF03181 PROSITE:PS51277 EMBL:AJ575662 EMBL:AJ575663 UniGene:Ta.56095 Gramene:Q70KG5 GO:GO:0043668 Uniprot:Q70KG5
Root	Isotig00677	55	11	2.193	9.64E-08	TAIR|locus:2156997 - symbol:AT5G49690 species:3702 "Arabidopsis thaliana" [GO:0008152 "metabolic process" evidence=IEA] [GO:0016757 "transferase activity, transferring glycosyl groups" evidence=ISS] [GO:0005829 "cytosol" evidence=IDA] InterPro:IPR002213 Pfam:PF00201 PROSITE:PS00375 GO:GO:0005829 EMBL:CP002688 GenomeReviews:BA000015_GR GO:GO:0016758 CAZy:GT1 PANTHER:PTHR11926 EMBL:AB025613 HOGENOM:HBG749942 eggNOG:NOG271171 EMBL:AK117150 EMBL:BT005390 IPI:IPI00525933 RefSeq:NP_199780.1 UniGene:At.29773 UniGene:At.75314 ProteinModelPortal:Q9LTA3 SMR:Q9LTA3 IntAct:Q9LTA3 PRIDE:Q9LTA3 EnsemblPlants:AT5G49690.1 GeneID:835032 KEGG:ath:AT5G49690 TAIR:At5g49690 InParanoid:Q9LTA3 OMA:YDYASHW PhylomeDB:Q9LTA3 ProtClustDB:PLN02670 Genevestigator:Q9LTA3 Uniprot:Q9LTA3
Root	Isotig00678	40	0	6.193	1.34E-10	TAIR|locus:2156997 - symbol:AT5G49690 species:3702 "Arabidopsis thaliana" [GO:0008152 "metabolic process" evidence=IEA] [GO:0016757 "transferase activity, transferring glycosyl groups" evidence=ISS] [GO:0005829 "cytosol" evidence=IDA] InterPro:IPR002213 Pfam:PF00201 PROSITE:PS00375 GO:GO:0005829 EMBL:CP002688 GenomeReviews:BA000015_GR GO:GO:0016758 CAZy:GT1 PANTHER:PTHR11926 EMBL:AB025613 HOGENOM:HBG749942 eggNOG:NOG271171 EMBL:AK117150 EMBL:BT005390 IPI:IPI00525933 RefSeq:NP_199780.1 UniGene:At.29773 UniGene:At.75314 ProteinModelPortal:Q9LTA3 SMR:Q9LTA3 IntAct:Q9LTA3 PRIDE:Q9LTA3 EnsemblPlants:AT5G49690.1 GeneID:835032 KEGG:ath:AT5G49690 TAIR:At5g49690 InParanoid:Q9LTA3 OMA:YDYASHW PhylomeDB:Q9LTA3 ProtClustDB:PLN02670 Genevestigator:Q9LTA3 Uniprot:Q9LTA3
Root	Isotig00683	44	16	1.330	0.000777292	TAIR|locus:2222607 - symbol:AT5G14520 "AT5G14520" species:3702 "Arabidopsis thaliana" [GO:0003713 "transcription coactivator activity" evidence=ISS] [GO:0005622 "intracellular" evidence=IEA;ISS] [GO:0005730 "nucleolus" evidence=IEA] [GO:0008283 "cell proliferation" evidence=IEA] InterPro:IPR001357 InterPro:IPR010613 Pfam:PF00533 Pfam:PF06732 PROSITE:PS50172 SMART:SM00292 EMBL:CP002688 GenomeReviews:BA000015_GR GO:GO:0005730 SUPFAM:SSF52113 GO:GO:0006364 EMBL:AL163792 eggNOG:COG5163 HOGENOM:HBG410102 KO:K14843 OMA:SIKGVYY HSSP:P18887 IPI:IPI00522738 PIR:T48625 RefSeq:NP_196956.1 UniGene:At.50457 ProteinModelPortal:Q9LYK7 SMR:Q9LYK7 STRING:Q9LYK7 PRIDE:Q9LYK7 EnsemblPlants:AT5G14520.1 GeneID:831303 KEGG:ath:AT5G14520 TAIR:At5g14520 InParanoid:Q9LYK7 PhylomeDB:Q9LYK7 ProtClustDB:CLSN2687226 Genevestigator:Q9LYK7 Uniprot:Q9LYK7
Root	Isotig00684	44	16	1.330	0.000777292	TAIR|locus:2222607 - symbol:AT5G14520 "AT5G14520" species:3702 "Arabidopsis thaliana" [GO:0003713 "transcription coactivator activity" evidence=ISS] [GO:0005622 "intracellular" evidence=IEA;ISS] [GO:0005730 "nucleolus" evidence=IEA] [GO:0008283 "cell proliferation" evidence=IEA] InterPro:IPR001357 InterPro:IPR010613

Tissue	Identification	# of reads (stress)	# of reads (control)	Fold Change	p-value	Description
						Pfam:PF00533 Pfam:PF06732 PROSITE:PS50172 SMART:SM00292 EMBL:CP002688 GenomeReviews:BA000015_GR GO:GO:0005730 SUPFAM:SSF52113 GO:GO:0006364 EMBL:AL163792 eggNOG:COG5163 HOGENOM:HBG410102 KO:K14843 OMA:SIKGVYY HSSP:P18887 IPI:IPI00522738 PIR:T48625 RefSeq:NP_196956.1 UniGene:At.50457 ProteinModelPortal:Q9LYK7 SMR:Q9LYK7 STRING:Q9LYK7 PRIDE:Q9LYK7 EnsemblPlants:AT5G14520.1 GeneID:831303 KEGG:ath:AT5G14520 TAIR:At5g14520 InParanoid:Q9LYK7 PhylomeDB:Q9LYK7 ProtClustDB:CLSN2687226 Genevestigator:Q9LYK7 Uniprot:Q9LYK7
Root	Isotig00688	20	3	2.608	0.000371713	TAIR|locus:2119971 - symbol:AT4G28990 "AT4G28990" species:3702 "Arabidopsis thaliana" [GO:0008150 "biological_process" evidence=ND] [GO:0008270 "zinc ion binding" evidence=IEA] InterPro:IPR001876 PROSITE:PS01358 PROSITE:PS50199 SMART:SM00547 EMBL:CP002687 GO:GO:0008270 GO:GO:0005622 EMBL:AL078470 EMBL:AL161574 EMBL:BT029205 IPI:IPI00536508 PIR:T08954 RefSeq:NP_194628.1 UniGene:At.31966 ProteinModelPortal:Q9SZD0 SMR:Q9SZD0 PRIDE:Q9SZD0 EnsemblPlants:AT4G28990.1 GeneID:829020 KEGG:ath:AT4G28990 TAIR:At4g28990 InParanoid:Q9SZD0 OMA:FARREYC PhylomeDB:Q9SZD0 ProtClustDB:CLSN2916031 ArrayExpress:Q9SZD0 Genevestigator:Q9SZD0 Uniprot:Q9SZD0
Root	Isotig00693	33	4	2.915	1.26E-06	No hit
Root	Isotig00694	33	4	2.915	1.26E-06	TAIR|locus:2036467 - symbol:ETFALPHA "AT1G50940" species:3702 "Arabidopsis thaliana" [GO:0005739 "mitochondrion" evidence=IDA] [GO:0005507 "copper ion binding" evidence=IDA] InterPro:IPR001308 PIRSF:PIRSF000089 Pfam:PF01012 Pfam:PF00766 EMBL:CP002684 GenomeReviews:CT485782_GR GO:GO:0009055 GO:GO:0050660 GO:GO:0005759 GO:GO:0006810 GO:GO:0005507 GO:GO:0022900 InterPro:IPR014729 Gene3D:G3DSA:3.40.50.620 EMBL:AC079284 EMBL:BT004989 EMBL:AK117515 IPI:IPI00542927 PIR:E96546 RefSeq:NP_175507.1 UniGene:At.21003 UniGene:At.66968 HSSP:P13804 ProteinModelPortal:Q9C6I6 SMR:Q9C6I6 IntAct:Q9C6I6 STRING:Q9C6I6 PRIDE:Q9C6I6 EnsemblPlants:AT1G50940.1 GeneID:841516 KEGG:ath:AT1G50940 GeneFarm:2340 TAIR:At1g50940 eggNOG:COG2025 HOGENOM:HBG615986 InParanoid:Q9C6I6 KO:K03522 OMA:TIRSTSF PhylomeDB:Q9C6I6 ProtClustDB:PLN00022 ArrayExpress:Q9C6I6 Genevestigator:Q9C6I6 InterPro:IPR014730 InterPro:IPR014731 InterPro:IPR018206 SMART:SM00893 PROSITE:PS00696 Uniprot:Q9C6I6
Root	Isotig00695	28	4	2.678	1.93E-05	No hit
Root	Isotig00696	17	1	3.958	8.09E-05	No hit
Root	Isotig00697	17	1	3.958	8.09E-05	TAIR|locus:2036467 - symbol:ETFALPHA "AT1G50940" species:3702 "Arabidopsis thaliana" [GO:0005739 "mitochondrion" evidence=IDA] [GO:0005507 "copper ion binding" evidence=IDA] InterPro:IPR001308 PIRSF:PIRSF000089 Pfam:PF01012 Pfam:PF00766 EMBL:CP002684 GenomeReviews:CT485782_GR GO:GO:0009055 GO:GO:0050660 GO:GO:0005759 GO:GO:0006810 GO:GO:0005507 GO:GO:0022900 InterPro:IPR014729 Gene3D:G3DSA:3.40.50.620 EMBL:AC079284 EMBL:BT004989 EMBL:AK117515 IPI:IPI00542927 PIR:E96546 RefSeq:NP_175507.1 UniGene:At.21003 UniGene:At.66968 HSSP:P13804 ProteinModelPortal:Q9C6I6 SMR:Q9C6I6 IntAct:Q9C6I6 STRING:Q9C6I6 PRIDE:Q9C6I6 EnsemblPlants:AT1G50940.1 GeneID:841516 KEGG:ath:AT1G50940 GeneFarm:2340 TAIR:At1g50940 eggNOG:COG2025 HOGENOM:HBG615986 InParanoid:Q9C6I6 KO:K03522 OMA:TIRSTSF PhylomeDB:Q9C6I6 ProtClustDB:PLN00022 ArrayExpress:Q9C6I6 Genevestigator:Q9C6I6 InterPro:IPR014730 InterPro:IPR014731 InterPro:IPR018206 SMART:SM00893 PROSITE:PS00696 Uniprot:Q9C6I6
Root	Isotig00698	274	118	1.086	8.96E-13	TAIR|locus:2165512 - symbol:PAF1 "proteasome alpha subunit F1" species:3702 "Arabidopsis thaliana" [GO:0004175 "endopeptidase activity" evidence=IEA] [GO:0004298 "threonine-type endopeptidase activity" evidence=IEA] [GO:0008233 "peptidase activity" evidence=ISS] [GO:0006511 "ubiquitin-dependent protein catabolic process" evidence=TAS] [GO:0005886 "plasma membrane" evidence=IDA] [GO:0046685 "response to arsenic-containing substance" evidence=IMP] [GO:0000502 "proteasome complex" evidence=ISS;IDA] [GO:0005829 "cytosol" evidence=IDA] [GO:0030163 "protein catabolic process" evidence=ISS] [GO:0005839 "proteasome core complex" evidence=TAS] InterPro:IPR000426 InterPro:IPR001353 InterPro:IPR023332 Pfam:PF00227 Pfam:PF10584 PROSITE:PS00388 PROSITE:PS51475 SMART:SM00948 GO:GO:0005829 GO:GO:0005886 GO:GO:0005634 EMBL:CP002688 GenomeReviews:BA000015_GR GO:GO:0046685 GO:GO:0006511 GO:GO:0004298 EMBL:AB007647 EMBL:M98495 EMBL:AF043526 EMBL:AF375447 EMBL:AY058128 EMBL:AY113047 EMBL:AY085017 IPI:IPI00525551 PIR:S39900 PIR:T51974 RefSeq:NP_199093.1 UniGene:At.23724 ProteinModelPortal:P34066 SMR:P34066 IntAct:P34066 STRING:P34066 PRIDE:P34066 EnsemblPlants:AT5G42790.1 GeneID:834290 KEGG:ath:AT5G42790 TAIR:At5g42790 eggNOG:COG0638 HOGENOM:HBG499923 InParanoid:P34066 KO:K02725 OMA:DVAPMEM PhylomeDB:P34066

Tissue	Identification	# of reads (stress)	# of reads (control)	Fold Change	p-value	Description
						ProtClustDB:CLSN2681914 ArrayExpress:P34066 Genevestigator:P34066 GermOnline:AT5G42790 GO:GO:0019773 Uniprot:P34066
Root	Isotig00699	255	105	1.151	5.11E-13	TAIR|locus:2157869 - symbol:PNG1 "AT5G49570" species:3702 "Arabidopsis thaliana" [GO:0003824 "catalytic activity" evidence=ISS] [GO:0000224 "peptide-N4-(N-acetyl-beta-glucosaminyl)asparagine amidase activity" evidence=IGI;IDA] [GO:0005829 "cytosol" evidence=IDA] [GO:0009751 "response to salicylic acid stimulus" evidence=IEP] [GO:0010188 "response to microbial phytotoxin" evidence=IEP] [GO:0010193 "response to ozone" evidence=IEP] InterPro:IPR018325 Pfam:PF03835 GO:GO:0005829 GO:GO:0005634 EMBL:CP002688 GenomeReviews:BA000015_GR GO:GO:0046872 GO:GO:0003684 GO:GO:0010193 GO:GO:0009751 InterPro:IPR008979 SUPFAM:SSF49785 GO:GO:0006289 EMBL:AB023033 InterPro:IPR002931 GO:GO:0010188 SMART:SM00460 KO:K01456 GO:GO:0000224 HSSP:Q8K113 eggNOG:NOG307426 EMBL:AY140065 EMBL:BT003161 EMBL:BT003398 EMBL:AK228156 IPI:IPI00533409 RefSeq:NP_199768.1 UniGene:At.27656 UniGene:At.29778 ProteinModelPortal:Q9FGY9 SMR:Q9FGY9 STRING:Q9FGY9 PRIDE:Q9FGY9 EnsemblPlants:AT5G49570.1 GeneID:835019 KEGG:ath:AT5G49570 TAIR:At5g49570 HOGENOM:HBG316986 InParanoid:Q9FGY9 OMA:QLLFWFK PhylomeDB:Q9FGY9 ProtClustDB:CLSN2686981 Genevestigator:Q9FGY9 GermOnline:AT5G49570 Uniprot:Q9FGY9
Root	Isotig00700	250	101	1.179	3.17E-13	No hit
Root	Isotig00701	247	98	1.205	1.71E-13	No hit
Root	Isotig00702	242	97	1.190	5.03E-13	No hit
Root	Isotig00703	242	101	1.132	3.91E-12	No hit
Root	Isotig00718	55	17	1.565	2.20E-05	No hit
Root	Isotig00719	56	17	1.591	1.47E-05	No hit
Root	Isotig00720	59	17	1.666	4.26E-06	No hit
Root	Isotig00721	55	17	1.565	2.20E-05	TIGR_CMR|GSU_1062 - symbol:GSU_1062 "cytochrome c family protein, putative" species:243231 "Geobacter sulfurreducens PCA" [GO:0009055 "electron carrier activity" evidence=ISS] EMBL:AE017180 GenomeReviews:AE017180_GR RefSeq:NP_952115.1 GeneID:2688672 KEGG:gsu:GSU1062 PATRIC:22024890 TIGR:GSU1062 HOGENOM:HBG090252 OMA:RIPELND ProtClustDB:CLSK743107 Uniprot:Q74EA1
Root	Isotig00722	60	17	1.690	2.80E-06	No hit
Root	Isotig00723	55	17	1.565	2.20E-05	No hit
Root	Isotig00724	60	17	1.690	2.80E-06	No hit
Root	Isotig00725	59	17	1.666	4.26E-06	TIGR_CMR|GSU_1062 - symbol:GSU_1062 "cytochrome c family protein, putative" species:243231 "Geobacter sulfurreducens PCA" [GO:0009055 "electron carrier activity" evidence=ISS] EMBL:AE017180 GenomeReviews:AE017180_GR RefSeq:NP_952115.1 GeneID:2688672 KEGG:gsu:GSU1062 PATRIC:22024890 TIGR:GSU1062 HOGENOM:HBG090252 OMA:RIPELND ProtClustDB:CLSK743107 Uniprot:Q74EA1
Root	Isotig00726	61	17	1.714	1.84E-06	No hit
Root	Isotig00727	59	17	1.666	4.26E-06	No hit
Root	Isotig00728	60	17	1.690	2.80E-06	TIGR_CMR|GSU_1062 - symbol:GSU_1062 "cytochrome c family protein, putative" species:243231 "Geobacter sulfurreducens PCA" [GO:0009055 "electron carrier activity" evidence=ISS] EMBL:AE017180 GenomeReviews:AE017180_GR RefSeq:NP_952115.1 GeneID:2688672 KEGG:gsu:GSU1062 PATRIC:22024890 TIGR:GSU1062 HOGENOM:HBG090252 OMA:RIPELND ProtClustDB:CLSK743107 Uniprot:Q74EA1
Root	Isotig00729	60	17	1.690	2.80E-06	No hit
Root	Isotig00730	55	17	1.565	2.20E-05	No hit
Root	Isotig00731	59	17	1.666	4.26E-06	No hit
Root	Isotig00732	60	17	1.690	2.80E-06	No hit
Root	Isotig00733	317	824	-1.507	9.46E-63	SGD|S000028422 - symbol:TAR1 "Mitochondrial protein potentially involved in respiratory metabolism" species:4932 "Saccharomyces cerevisiae" [GO:0003674 "molecular_function" evidence=ND] [GO:0043457 "regulation of cellular respiration" evidence=IGI] [GO:0005739 "mitochondrion" evidence=IEA;IMP] SGD:S000028422 GO:GO:0005739 EMBL:BK006945 EMBL:U53879 GO:GO:0043457 EMBL:Z73326 EMBL:AF479964 RefSeq:NP_690845.1 ProteinModelPortal:Q8TGM6 STRING:Q8TGM6 EnsemblFungi:YLR154W-C GeneID:1466405 KEGG:sce:YLR154W-C CYGD:YLR154w-c eggNOG:NOG79861 OMA:SCSKEHR OrthoDB:EOG466ZX8 NextBio:967146 Genevestigator:Q8TGM6 GermOnline:YLR154W-C Uniprot:Q8TGM6

Tissue	Identification	# of reads (stress)	# of reads (control)	Fold Change	p-value	Description
Root	Isotig00734	353	959	-1.571	3.54E-77	SGD|S000028422 - symbol:TAR1 "Mitochondrial protein potentially involved in respiratory metabolism" species:4932 "Saccharomyces cerevisiae" [GO:0003674 "molecular_function" evidence=ND] [GO:0043457 "regulation of cellular respiration" evidence=IGI] [GO:0005739 "mitochondrion" evidence=IEA;IMP] SGD:S000028422 GO:GO:0005739 EMBL:BK006945 EMBL:U53879 GO:GO:0043457 EMBL:Z73326 EMBL:AF479964 RefSeq:NP_690845.1 ProteinModelPortal:Q8TGM6 STRING:Q8TGM6 EnsemblFungi:YLR154W-C GeneID:1466405 KEGG:sce:YLR154W-C CYGD:YLR154w-c eggNOG:NOG79861 OMA:SCSKEHR OrthoDB:EOG466ZX8 NextBio:967146 Genevestigator:Q8TGM6 GermOnline:YLR154W-C Uniprot:Q8TGM6
Root	Isotig00735	353	959	-1.571	3.54E-77	SGD|S000028422 - symbol:TAR1 "Mitochondrial protein potentially involved in respiratory metabolism" species:4932 "Saccharomyces cerevisiae" [GO:0003674 "molecular_function" evidence=ND] [GO:0043457 "regulation of cellular respiration" evidence=IGI] [GO:0005739 "mitochondrion" evidence=IEA;IMP] SGD:S000028422 GO:GO:0005739 EMBL:BK006945 EMBL:U53879 GO:GO:0043457 EMBL:Z73326 EMBL:AF479964 RefSeq:NP_690845.1 ProteinModelPortal:Q8TGM6 STRING:Q8TGM6 EnsemblFungi:YLR154W-C GeneID:1466405 KEGG:sce:YLR154W-C CYGD:YLR154w-c eggNOG:NOG79861 OMA:SCSKEHR OrthoDB:EOG466ZX8 NextBio:967146 Genevestigator:Q8TGM6 GermOnline:YLR154W-C Uniprot:Q8TGM6
Root	Isotig00736	353	959	-1.571	3.54E-77	SGD|S000028422 - symbol:TAR1 "Mitochondrial protein potentially involved in respiratory metabolism" species:4932 "Saccharomyces cerevisiae" [GO:0003674 "molecular_function" evidence=ND] [GO:0043457 "regulation of cellular respiration" evidence=IGI] [GO:0005739 "mitochondrion" evidence=IEA;IMP] SGD:S000028422 GO:GO:0005739 EMBL:BK006945 EMBL:U53879 GO:GO:0043457 EMBL:Z73326 EMBL:AF479964 RefSeq:NP_690845.1 ProteinModelPortal:Q8TGM6 STRING:Q8TGM6 EnsemblFungi:YLR154W-C GeneID:1466405 KEGG:sce:YLR154W-C CYGD:YLR154w-c eggNOG:NOG79861 OMA:SCSKEHR OrthoDB:EOG466ZX8 NextBio:967146 Genevestigator:Q8TGM6 GermOnline:YLR154W-C Uniprot:Q8TGM6
Root	Isotig00737	252	576	-1.322	1.03E-36	SGD|S000028567 - symbol:RRT15 "Putative protein of unknown function" species:4932 "Saccharomyces cerevisiae" [GO:0006351 "transcription, DNA-dependent" evidence=IEA] [GO:0005575 "cellular_component" evidence=ND] [GO:0008150 "biological_process" evidence=ND] [GO:0006355 "regulation of transcription, DNA-dependent" evidence=IEA] [GO:0003674 "molecular_function" evidence=ND] SGD:S000028567 GO:GO:0006355 GO:GO:0006351 EMBL:BK006945 EMBL:U51921 RefSeq:NP_878128.1 ProteinModelPortal:Q3E811 STRING:Q3E811 EnsemblFungi:YLR162W-A GeneID:1466416 KEGG:sce:YLR162W-A CYGD:YLR162w-a GeneTree:ENSGT00530000064745 OMA:FYINCIM OrthoDB:EOG45TGXP NextBio:983475 ArrayExpress:Q3E811 Genevestigator:Q3E811 Uniprot:Q3E811
Root	Isotig00738	200	434	-1.247	8.89E-26	SGD|S000028567 - symbol:RRT15 "Putative protein of unknown function" species:4932 "Saccharomyces cerevisiae" [GO:0006351 "transcription, DNA-dependent" evidence=IEA] [GO:0005575 "cellular_component" evidence=ND] [GO:0008150 "biological_process" evidence=ND] [GO:0006355 "regulation of transcription, DNA-dependent" evidence=IEA] [GO:0003674 "molecular_function" evidence=ND] SGD:S000028567 GO:GO:0006355 GO:GO:0006351 EMBL:BK006945 EMBL:U51921 RefSeq:NP_878128.1 ProteinModelPortal:Q3E811 STRING:Q3E811 EnsemblFungi:YLR162W-A GeneID:1466416 KEGG:sce:YLR162W-A CYGD:YLR162w-a GeneTree:ENSGT00530000064745 OMA:FYINCIM OrthoDB:EOG45TGXP NextBio:983475 ArrayExpress:Q3E811 Genevestigator:Q3E811 Uniprot:Q3E811
Root	Isotig00739	200	434	-1.247	8.89E-26	SGD|S000028567 - symbol:RRT15 "Putative protein of unknown function" species:4932 "Saccharomyces cerevisiae" [GO:0006351 "transcription, DNA-dependent" evidence=IEA] [GO:0005575 "cellular_component" evidence=ND] [GO:0008150 "biological_process" evidence=ND] [GO:0006355 "regulation of transcription, DNA-dependent" evidence=IEA] [GO:0003674 "molecular_function" evidence=ND] SGD:S000028567 GO:GO:0006355 GO:GO:0006351 EMBL:BK006945 EMBL:U51921 RefSeq:NP_878128.1 ProteinModelPortal:Q3E811 STRING:Q3E811 EnsemblFungi:YLR162W-A GeneID:1466416 KEGG:sce:YLR162W-A CYGD:YLR162w-a GeneTree:ENSGT00530000064745 OMA:FYINCIM OrthoDB:EOG45TGXP NextBio:983475 ArrayExpress:Q3E811 Genevestigator:Q3E811 Uniprot:Q3E811
Root	Isotig00740	200	434	-1.247	8.89E-26	SGD|S000028567 - symbol:RRT15 "Putative protein of unknown function" species:4932 "Saccharomyces cerevisiae" [GO:0006351 "transcription, DNA-dependent" evidence=IEA] [GO:0005575 "cellular_component" evidence=ND] [GO:0008150 "biological_process" evidence=ND] [GO:0006355 "regulation of transcription, DNA-dependent" evidence=IEA] [GO:0003674 "molecular_function" evidence=ND] SGD:S000028567 GO:GO:0006355 GO:GO:0006351 EMBL:BK006945 EMBL:U51921 RefSeq:NP_878128.1 ProteinModelPortal:Q3E811 STRING:Q3E811 EnsemblFungi:YLR162W-A GeneID:1466416 KEGG:sce:YLR162W-A CYGD:YLR162w-a

Tissue	Identification	# of reads (stress)	# of reads (control)	Fold Change	p-value	Description
						GeneTree:ENSGT00530000064745 OMA:FYINCIM OrthoDB:EOG45TGXP NextBio:983475 ArrayExpress:Q3E811 Genevestigator:Q3E811 Uniprot:Q3E811
Root	Isotig00741	242	562	-1.345	8.92E-37	SGD|S000028567 - symbol:RRT15 "Putative protein of unknown function" species:4932 "Saccharomyces cerevisiae" [GO:0006351 "transcription, DNA-dependent" evidence=IEA] [GO:0005575 "cellular_component" evidence=ND] [GO:0008150 "biological_process" evidence=ND] [GO:0006355 "regulation of transcription, DNA-dependent" evidence=IEA] [GO:0003674 "molecular_function" evidence=ND] SGD:S000028567 GO:GO:0006355 GO:GO:0006351 EMBL:BK006945 EMBL:U51921 RefSeq:NP_878128.1 ProteinModelPortal:Q3E811 STRING:Q3E811 EnsemblFungi:YLR162W-A GeneID:1466416 KEGG:sce:YLR162W-A CYGD:YLR162w-a GeneTree:ENSGT00530000064745 OMA:FYINCIM OrthoDB:EOG45TGXP NextBio:983475 ArrayExpress:Q3E811 Genevestigator:Q3E811 Uniprot:Q3E811
Root	Isotig00742	278	697	-1.455	1.01E-50	SGD|S000028567 - symbol:RRT15 "Putative protein of unknown function" species:4932 "Saccharomyces cerevisiae" [GO:0006351 "transcription, DNA-dependent" evidence=IEA] [GO:0005575 "cellular_component" evidence=ND] [GO:0008150 "biological_process" evidence=ND] [GO:0006355 "regulation of transcription, DNA-dependent" evidence=IEA] [GO:0003674 "molecular_function" evidence=ND] SGD:S000028567 GO:GO:0006355 GO:GO:0006351 EMBL:BK006945 EMBL:U51921 RefSeq:NP_878128.1 ProteinModelPortal:Q3E811 STRING:Q3E811 EnsemblFungi:YLR162W-A GeneID:1466416 KEGG:sce:YLR162W-A CYGD:YLR162w-a GeneTree:ENSGT00530000064745 OMA:FYINCIM OrthoDB:EOG45TGXP NextBio:983475 ArrayExpress:Q3E811 Genevestigator:Q3E811 Uniprot:Q3E811
Root	Isotig00743	278	697	-1.455	1.01E-50	SGD|S000028567 - symbol:RRT15 "Putative protein of unknown function" species:4932 "Saccharomyces cerevisiae" [GO:0006351 "transcription, DNA-dependent" evidence=IEA] [GO:0005575 "cellular_component" evidence=ND] [GO:0008150 "biological_process" evidence=ND] [GO:0006355 "regulation of transcription, DNA-dependent" evidence=IEA] [GO:0003674 "molecular_function" evidence=ND] SGD:S000028567 GO:GO:0006355 GO:GO:0006351 EMBL:BK006945 EMBL:U51921 RefSeq:NP_878128.1 ProteinModelPortal:Q3E811 STRING:Q3E811 EnsemblFungi:YLR162W-A GeneID:1466416 KEGG:sce:YLR162W-A CYGD:YLR162w-a GeneTree:ENSGT00530000064745 OMA:FYINCIM OrthoDB:EOG45TGXP NextBio:983475 ArrayExpress:Q3E811 Genevestigator:Q3E811 Uniprot:Q3E811
Root	Isotig00744	278	697	-1.455	1.01E-50	SGD|S000028567 - symbol:RRT15 "Putative protein of unknown function" species:4932 "Saccharomyces cerevisiae" [GO:0006351 "transcription, DNA-dependent" evidence=IEA] [GO:0005575 "cellular_component" evidence=ND] [GO:0008150 "biological_process" evidence=ND] [GO:0006355 "regulation of transcription, DNA-dependent" evidence=IEA] [GO:0003674 "molecular_function" evidence=ND] SGD:S000028567 GO:GO:0006355 GO:GO:0006351 EMBL:BK006945 EMBL:U51921 RefSeq:NP_878128.1 ProteinModelPortal:Q3E811 STRING:Q3E811 EnsemblFungi:YLR162W-A GeneID:1466416 KEGG:sce:YLR162W-A CYGD:YLR162w-a GeneTree:ENSGT00530000064745 OMA:FYINCIM OrthoDB:EOG45TGXP NextBio:983475 ArrayExpress:Q3E811 Genevestigator:Q3E811 Uniprot:Q3E811
Root	Isotig00745	188	535	-1.638	1.94E-46	SGD|S000028422 - symbol:TAR1 "Mitochondrial protein potentially involved in respiratory metabolism" species:4932 "Saccharomyces cerevisiae" [GO:0003674 "molecular_function" evidence=ND] [GO:0043457 "regulation of cellular respiration" evidence=IGI] [GO:0005739 "mitochondrion" evidence=IEA;IMP] SGD:S000028422 GO:GO:0005739 EMBL:BK006945 EMBL:U53879 GO:GO:0043457 EMBL:Z73326 EMBL:AF479964 RefSeq:NP_690845.1 ProteinModelPortal:Q8TGM6 STRING:Q8TGM6 EnsemblFungi:YLR154W-C GeneID:1466405 KEGG:sce:YLR154W-C CYGD:YLR154w-c eggNOG:NOG79861 OMA:SCSKEHR OrthoDB:EOG466ZX8 NextBio:967146 Genevestigator:Q8TGM6 GermOnline:YLR154W-C Uniprot:Q8TGM6
Root	Isotig00746	113	273	-1.402	8.19E-20	No hit
Root	Isotig00747	4	53	-3.857	5.31E-13	TAIR|locus:2152160 - symbol:BGLU13 "beta glucosidase 13" species:3702 "Arabidopsis thaliana" [GO:0003824 "catalytic activity" evidence=IEA] [GO:0004553 "hydrolase activity, hydrolyzing O-glycosyl compounds" evidence=IEA;ISS] [GO:0005975 "carbohydrate metabolic process" evidence=IEA] [GO:0043169 "cation binding" evidence=IEA] [GO:0005794 "Golgi apparatus" evidence=IDA] InterPro:IPR001360 InterPro:IPR013781 InterPro:IPR018120 Pfam:PF00232 PRINTS:PR00131 PROSITE:PS00572 PROSITE:PS00653 EMBL:CP002688 GenomeReviews:BA000015_GR GO:GO:0043169 InterPro:IPR017853 Gene3D:G3DSA:3.20.20.80 SUPFAM:SSF51445 GO:GO:0005975 GO:GO:0008422 CAZy:GH1 eggNOG:COG2723 HOGENOM:HBG316462 PANTHER:PTHR10353 EMBL:AB024024 HSSP:P26205 KO:K01188 ProtClustDB:CLSN2683204 EMBL:BT033043 IPI:IPI00536489 RefSeq:NP_199277.1 UniGene:At.50504 UniGene:At.75350 ProteinModelPortal:Q9LU02 SMR:Q9LU02 STRING:Q9LU02 PRIDE:Q9LU02 EnsemblPlants:AT5G44640.1 GeneID:834493

Tissue	Identification	# of reads (stress)	# of reads (control)	Fold Change	p-value	Description
						KEGG:ath:AT5G44640 TAIR:At5g44640 InParanoid:Q9LU02 OMA:EVIAKKH PhylomeDB:Q9LU02 Genevestigator:Q9LU02 Uniprot:Q9LU02
Root	Isotig00748	4	53	-3.857	5.31E-13	TAIR|locus:2152160 - symbol:BGLU13 "beta glucosidase 13" species:3702 "Arabidopsis thaliana" [GO:0003824 "catalytic activity" evidence=IEA] [GO:0004553 "hydrolase activity, hydrolyzing O-glycosyl compounds" evidence=IEA;ISS] [GO:0005975 "carbohydrate metabolic process" evidence=IEA] [GO:0043169 "cation binding" evidence=IEA] [GO:0005794 "Golgi apparatus" evidence=IDA] InterPro:IPR001360 InterPro:IPR013781 InterPro:IPR018120 Pfam:PF00232 PRINTS:PR00131 PROSITE:PS00572 PROSITE:PS00653 EMBL:CP002688 GenomeReviews:BA000015_GR GO:GO:0043169 InterPro:IPR017853 Gene3D:G3DSA:3.20.20.80 SUPFAM:SSF51445 GO:GO:0005975 GO:GO:0008422 CAZy:GH1 eggNOG:COG2723 HOGENOM:HBG316462 PANTHER:PTHR10353 EMBL:AB024024 HSSP:P26205 KO:K01188 ProtClustDB:CLSN2683204 EMBL:BT033043 IPI:IPI00536489 RefSeq:NP_199277.1 UniGene:At.50504 UniGene:At.75350 ProteinModelPortal:Q9LU02 SMR:Q9LU02 STRING:Q9LU02 PRIDE:Q9LU02 EnsemblPlants:AT5G44640.1 GeneID:834493 KEGG:ath:AT5G44640 TAIR:At5g44640 InParanoid:Q9LU02 OMA:EVIAKKH PhylomeDB:Q9LU02 Genevestigator:Q9LU02 Uniprot:Q9LU02
Root	Isotig00749	4	44	-3.588	1.46E-10	TAIR|locus:2197960 - symbol:BGLU40 "beta glucosidase 40" species:3702 "Arabidopsis thaliana" [GO:0003824 "catalytic activity" evidence=IEA] [GO:0004553 "hydrolase activity, hydrolyzing O-glycosyl compounds" evidence=IEA;ISS] [GO:0005975 "carbohydrate metabolic process" evidence=IEA] [GO:0043169 "cation binding" evidence=IEA] [GO:0009507 "chloroplast" evidence=IDA] [GO:0048046 "apoplast" evidence=IDA] InterPro:IPR001360 InterPro:IPR013781 InterPro:IPR018120 Pfam:PF00232 PRINTS:PR00131 PROSITE:PS00572 PROSITE:PS00653 EMBL:CP002684 GenomeReviews:CT485782_GR EMBL:AC013427 GO:GO:0048046 GO:GO:0009507 GO:GO:0043169 InterPro:IPR017853 Gene3D:G3DSA:3.20.20.80 SUPFAM:SSF51445 GO:GO:0005975 GO:GO:0008422 CAZy:GH1 eggNOG:COG2723 PANTHER:PTHR10353 HSSP:P26205 KO:K01188 EMBL:AY045927 EMBL:AY142610 EMBL:AY085043 EMBL:AK221011 IPI:IPI00537698 PIR:F86392 RefSeq:NP_173978.1 UniGene:At.15959 ProteinModelPortal:Q9FZE0 SMR:Q9FZE0 STRING:Q9FZE0 PRIDE:Q9FZE0 EnsemblPlants:AT1G26560.1 GeneID:839196 KEGG:ath:AT1G26560 TAIR:At1g26560 InParanoid:Q9FZE0 OMA:NDFAAYA PhylomeDB:Q9FZE0 ProtClustDB:CLSN2682658 ArrayExpress:Q9FZE0 Uniprot:Q9FZE0
Root	Isotig00750	4	44	-3.588	1.46E-10	TAIR|locus:2197960 - symbol:BGLU40 "beta glucosidase 40" species:3702 "Arabidopsis thaliana" [GO:0003824 "catalytic activity" evidence=IEA] [GO:0004553 "hydrolase activity, hydrolyzing O-glycosyl compounds" evidence=IEA;ISS] [GO:0005975 "carbohydrate metabolic process" evidence=IEA] [GO:0043169 "cation binding" evidence=IEA] [GO:0009507 "chloroplast" evidence=IDA] [GO:0048046 "apoplast" evidence=IDA] InterPro:IPR001360 InterPro:IPR013781 InterPro:IPR018120 Pfam:PF00232 PRINTS:PR00131 PROSITE:PS00572 PROSITE:PS00653 EMBL:CP002684 GenomeReviews:CT485782_GR EMBL:AC013427 GO:GO:0048046 GO:GO:0009507 GO:GO:0043169 InterPro:IPR017853 Gene3D:G3DSA:3.20.20.80 SUPFAM:SSF51445 GO:GO:0005975 GO:GO:0008422 CAZy:GH1 eggNOG:COG2723 PANTHER:PTHR10353 HSSP:P26205 KO:K01188 EMBL:AY045927 EMBL:AY142610 EMBL:AY085043 EMBL:AK221011 IPI:IPI00537698 PIR:F86392 RefSeq:NP_173978.1 UniGene:At.15959 ProteinModelPortal:Q9FZE0 SMR:Q9FZE0 STRING:Q9FZE0 PRIDE:Q9FZE0 EnsemblPlants:AT1G26560.1 GeneID:839196 KEGG:ath:AT1G26560 TAIR:At1g26560 InParanoid:Q9FZE0 OMA:NDFAAYA PhylomeDB:Q9FZE0 ProtClustDB:CLSN2682658 ArrayExpress:Q9FZE0 Uniprot:Q9FZE0
Root	Isotig00751	1	43	-5.555	3.04E-12	TAIR|locus:2152160 - symbol:BGLU13 "beta glucosidase 13" species:3702 "Arabidopsis thaliana" [GO:0003824 "catalytic activity" evidence=IEA] [GO:0004553 "hydrolase activity, hydrolyzing O-glycosyl compounds" evidence=IEA;ISS] [GO:0005975 "carbohydrate metabolic process" evidence=IEA] [GO:0043169 "cation binding" evidence=IEA] [GO:0005794 "Golgi apparatus" evidence=IDA] InterPro:IPR001360 InterPro:IPR013781 InterPro:IPR018120 Pfam:PF00232 PRINTS:PR00131 PROSITE:PS00572 PROSITE:PS00653 EMBL:CP002688 GenomeReviews:BA000015_GR GO:GO:0043169 InterPro:IPR017853 Gene3D:G3DSA:3.20.20.80 SUPFAM:SSF51445 GO:GO:0005975 GO:GO:0008422 CAZy:GH1 eggNOG:COG2723 HOGENOM:HBG316462 PANTHER:PTHR10353 EMBL:AB024024 HSSP:P26205 KO:K01188 ProtClustDB:CLSN2683204 EMBL:BT033043 IPI:IPI00536489 RefSeq:NP_199277.1 UniGene:At.50504 UniGene:At.75350 ProteinModelPortal:Q9LU02 SMR:Q9LU02 STRING:Q9LU02 PRIDE:Q9LU02 EnsemblPlants:AT5G44640.1 GeneID:834493 KEGG:ath:AT5G44640 TAIR:At5g44640 InParanoid:Q9LU02 OMA:EVIAKKH PhylomeDB:Q9LU02 Genevestigator:Q9LU02 Uniprot:Q9LU02

Tissue	Identification	# of reads (stress)	# of reads (control)	Fold Change	p-value	Description
Root	Isotig00752	1	43	-5.555	3.04E-12	TAIR|locus:2152160 - symbol:BGLU13 "beta glucosidase 13" species:3702 "Arabidopsis thaliana" [GO:0003824 "catalytic activity" evidence=IEA] [GO:0004553 "hydrolase activity, hydrolyzing O-glycosyl compounds" evidence=IEA;ISS] [GO:0005975 "carbohydrate metabolic process" evidence=IEA] [GO:0043169 "cation binding" evidence=IEA] [GO:0005794 "Golgi apparatus" evidence=IDA] InterPro:IPR001360 InterPro:IPR013781 InterPro:IPR018120 Pfam:PF00232 PRINTS:PR00131 PROSITE:PS00572 PROSITE:PS00653 EMBL:CP002688 GenomeReviews:BA000015_GR GO:GO:0043169 InterPro:IPR017853 Gene3D:G3DSA:3.20.20.80 SUPFAM:SSF51445 GO:GO:0005975 GO:GO:0008422 CAZy:GH1 eggNOG:COG2723 HOGENOM:HBG316462 PANTHER:PTHR10353 EMBL:AB024024 HSSP:P26205 KO:K01188 ProtClustDB:CLSN2683204 EMBL:BT033043 IPI:IPI00536489 RefSeq:NP_199277.1 UniGene:At.50504 UniGene:At.75350 ProteinModelPortal:Q9LU02 SMR:Q9LU02 STRING:Q9LU02 PRIDE:Q9LU02 EnsemblPlants:AT5G44640.1 GeneID:834493 KEGG:ath:AT5G44640 TAIR:At5g44640 InParanoid:Q9LU02 OMA:EVIAKKH PhylomeDB:Q9LU02 Genevestigator:Q9LU02 Uniprot:Q9LU02
Root	Isotig00753	1	44	-5.588	1.69E-12	TAIR|locus:2152160 - symbol:BGLU13 "beta glucosidase 13" species:3702 "Arabidopsis thaliana" [GO:0003824 "catalytic activity" evidence=IEA] [GO:0004553 "hydrolase activity, hydrolyzing O-glycosyl compounds" evidence=IEA;ISS] [GO:0005975 "carbohydrate metabolic process" evidence=IEA] [GO:0043169 "cation binding" evidence=IEA] [GO:0005794 "Golgi apparatus" evidence=IDA] InterPro:IPR001360 InterPro:IPR013781 InterPro:IPR018120 Pfam:PF00232 PRINTS:PR00131 PROSITE:PS00572 PROSITE:PS00653 EMBL:CP002688 GenomeReviews:BA000015_GR GO:GO:0043169 InterPro:IPR017853 Gene3D:G3DSA:3.20.20.80 SUPFAM:SSF51445 GO:GO:0005975 GO:GO:0008422 CAZy:GH1 eggNOG:COG2723 HOGENOM:HBG316462 PANTHER:PTHR10353 EMBL:AB024024 HSSP:P26205 KO:K01188 ProtClustDB:CLSN2683204 EMBL:BT033043 IPI:IPI00536489 RefSeq:NP_199277.1 UniGene:At.50504 UniGene:At.75350 ProteinModelPortal:Q9LU02 SMR:Q9LU02 STRING:Q9LU02 PRIDE:Q9LU02 EnsemblPlants:AT5G44640.1 GeneID:834493 KEGG:ath:AT5G44640 TAIR:At5g44640 InParanoid:Q9LU02 OMA:EVIAKKH PhylomeDB:Q9LU02 Genevestigator:Q9LU02 Uniprot:Q9LU02
Root	Isotig00754	1	44	-5.588	1.69E-12	TAIR|locus:2152160 - symbol:BGLU13 "beta glucosidase 13" species:3702 "Arabidopsis thaliana" [GO:0003824 "catalytic activity" evidence=IEA] [GO:0004553 "hydrolase activity, hydrolyzing O-glycosyl compounds" evidence=IEA;ISS] [GO:0005975 "carbohydrate metabolic process" evidence=IEA] [GO:0043169 "cation binding" evidence=IEA] [GO:0005794 "Golgi apparatus" evidence=IDA] InterPro:IPR001360 InterPro:IPR013781 InterPro:IPR018120 Pfam:PF00232 PRINTS:PR00131 PROSITE:PS00572 PROSITE:PS00653 EMBL:CP002688 GenomeReviews:BA000015_GR GO:GO:0043169 InterPro:IPR017853 Gene3D:G3DSA:3.20.20.80 SUPFAM:SSF51445 GO:GO:0005975 GO:GO:0008422 CAZy:GH1 eggNOG:COG2723 HOGENOM:HBG316462 PANTHER:PTHR10353 EMBL:AB024024 HSSP:P26205 KO:K01188 ProtClustDB:CLSN2683204 EMBL:BT033043 IPI:IPI00536489 RefSeq:NP_199277.1 UniGene:At.50504 UniGene:At.75350 ProteinModelPortal:Q9LU02 SMR:Q9LU02 STRING:Q9LU02 PRIDE:Q9LU02 EnsemblPlants:AT5G44640.1 GeneID:834493 KEGG:ath:AT5G44640 TAIR:At5g44640 InParanoid:Q9LU02 OMA:EVIAKKH PhylomeDB:Q9LU02 Genevestigator:Q9LU02 Uniprot:Q9LU02
Root	Isotig00755	1	34	-5.216	6.54E-10	TAIR|locus:2197960 - symbol:BGLU40 "beta glucosidase 40" species:3702 "Arabidopsis thaliana" [GO:0003824 "catalytic activity" evidence=IEA] [GO:0004553 "hydrolase activity, hydrolyzing O-glycosyl compounds" evidence=IEA;ISS] [GO:0005975 "carbohydrate metabolic process" evidence=IEA] [GO:0043169 "cation binding" evidence=IEA] [GO:0009507 "chloroplast" evidence=IDA] [GO:0048046 "apoplast" evidence=IDA] InterPro:IPR001360 InterPro:IPR013781 InterPro:IPR018120 Pfam:PF00232 PRINTS:PR00131 PROSITE:PS00572 PROSITE:PS00653 EMBL:CP002684 GenomeReviews:CT485782_GR EMBL:AC013427 GO:GO:0048046 GO:GO:0009507 GO:GO:0043169 InterPro:IPR017853 Gene3D:G3DSA:3.20.20.80 SUPFAM:SSF51445 GO:GO:0005975 GO:GO:0008422 CAZy:GH1 eggNOG:COG2723 PANTHER:PTHR10353 HSSP:P26205 KO:K01188 EMBL:AY045927 EMBL:AY142610 EMBL:AY085043 EMBL:AK221011 IPI:IPI00537698 PIR:F86392 RefSeq:NP_173978.1 UniGene:At.15959 ProteinModelPortal:Q9FZE0 SMR:Q9FZE0 STRING:Q9FZE0 PRIDE:Q9FZE0 EnsemblPlants:AT1G26560.1 GeneID:839196 KEGG:ath:AT1G26560 TAIR:At1g26560 InParanoid:Q9FZE0 OMA:NDFAAYA PhylomeDB:Q9FZE0 ProtClustDB:CLSN2682658 ArrayExpress:Q9FZE0 Uniprot:Q9FZE0
Root	Isotig00756	1	34	-5.216	6.54E-10	TAIR|locus:2197960 - symbol:BGLU40 "beta glucosidase 40" species:3702 "Arabidopsis thaliana" [GO:0003824 "catalytic activity" evidence=IEA] [GO:0004553 "hydrolase activity, hydrolyzing O-glycosyl compounds" evidence=IEA;ISS] [GO:0005975 "carbohydrate metabolic process" evidence=IEA] [GO:0043169 "cation binding"

Tissue	Identification	# of reads (stress)	# of reads (control)	Fold Change	p-value	Description
						evidence=IEA] [GO:0009507 "chloroplast" evidence=IDA] [GO:0048046 "apoplast" evidence=IDA] InterPro:IPR001360 InterPro:IPR013781 InterPro:IPR018120 Pfam:PF00232 PRINTS:PR00131 PROSITE:PS00572 PROSITE:PS00653 EMBL:CP002684 GenomeReviews:CT485782_GR EMBL:AC013427 GO:GO:0048046 GO:GO:0009507 GO:GO:0043169 InterPro:IPR017853 Gene3D:G3DSA:3.20.20.80 SUPFAM:SSF51445 GO:GO:0005975 GO:GO:0008422 CAZy:GH1 eggNOG:COG2723 PANTHER:PTHR10353 HSSP:P26205 KO:K01188 EMBL:AY045927 EMBL:AY142610 EMBL:AY085043 EMBL:AK221011 IPI:IPI00537698 PIR:F86392 RefSeq:NP_173978.1 UniGene:At.15959 ProteinModelPortal:Q9FZE0 SMR:Q9FZE0 STRING:Q9FZE0 PRIDE:Q9FZE0 EnsemblPlants:AT1G26560.1 GeneID:839196 KEGG:ath:AT1G26560 TAIR:At1g26560 InParanoid:Q9FZE0 OMA:NDFAAYA PhylomeDB:Q9FZE0 ProtClustDB:CLSN2682658 ArrayExpress:Q9FZE0 Uniprot:Q9FZE0
Root	Isotig00757	1	35	-5.258	3.57E-10	TAIR|locus:2197960 - symbol:BGLU40 "beta glucosidase 40" species:3702 "Arabidopsis thaliana" [GO:0003824 "catalytic activity" evidence=IEA] [GO:0004553 "hydrolase activity, hydrolyzing O-glycosyl compounds" evidence=IEA;ISS] [GO:0005975 "carbohydrate metabolic process" evidence=IEA] [GO:0043169 "cation binding" evidence=IEA] [GO:0009507 "chloroplast" evidence=IDA] [GO:0048046 "apoplast" evidence=IDA] InterPro:IPR001360 InterPro:IPR013781 InterPro:IPR018120 Pfam:PF00232 PRINTS:PR00131 PROSITE:PS00572 PROSITE:PS00653 EMBL:CP002684 GenomeReviews:CT485782_GR EMBL:AC013427 GO:GO:0048046 GO:GO:0009507 GO:GO:0043169 InterPro:IPR017853 Gene3D:G3DSA:3.20.20.80 SUPFAM:SSF51445 GO:GO:0005975 GO:GO:0008422 CAZy:GH1 eggNOG:COG2723 PANTHER:PTHR10353 HSSP:P26205 KO:K01188 EMBL:AY045927 EMBL:AY142610 EMBL:AY085043 EMBL:AK221011 IPI:IPI00537698 PIR:F86392 RefSeq:NP_173978.1 UniGene:At.15959 ProteinModelPortal:Q9FZE0 SMR:Q9FZE0 STRING:Q9FZE0 PRIDE:Q9FZE0 EnsemblPlants:AT1G26560.1 GeneID:839196 KEGG:ath:AT1G26560 TAIR:At1g26560 InParanoid:Q9FZE0 OMA:NDFAAYA PhylomeDB:Q9FZE0 ProtClustDB:CLSN2682658 ArrayExpress:Q9FZE0 Uniprot:Q9FZE0
Root	Isotig00759	1	35	-5.258	3.57E-10	TAIR|locus:2197960 - symbol:BGLU40 "beta glucosidase 40" species:3702 "Arabidopsis thaliana" [GO:0003824 "catalytic activity" evidence=IEA] [GO:0004553 "hydrolase activity, hydrolyzing O-glycosyl compounds" evidence=IEA;ISS] [GO:0005975 "carbohydrate metabolic process" evidence=IEA] [GO:0043169 "cation binding" evidence=IEA] [GO:0009507 "chloroplast" evidence=IDA] [GO:0048046 "apoplast" evidence=IDA] InterPro:IPR001360 InterPro:IPR013781 InterPro:IPR018120 Pfam:PF00232 PRINTS:PR00131 PROSITE:PS00572 PROSITE:PS00653 EMBL:CP002684 GenomeReviews:CT485782_GR EMBL:AC013427 GO:GO:0048046 GO:GO:0009507 GO:GO:0043169 InterPro:IPR017853 Gene3D:G3DSA:3.20.20.80 SUPFAM:SSF51445 GO:GO:0005975 GO:GO:0008422 CAZy:GH1 eggNOG:COG2723 PANTHER:PTHR10353 HSSP:P26205 KO:K01188 EMBL:AY045927 EMBL:AY142610 EMBL:AY085043 EMBL:AK221011 IPI:IPI00537698 PIR:F86392 RefSeq:NP_173978.1 UniGene:At.15959 ProteinModelPortal:Q9FZE0 SMR:Q9FZE0 STRING:Q9FZE0 PRIDE:Q9FZE0 EnsemblPlants:AT1G26560.1 GeneID:839196 KEGG:ath:AT1G26560 TAIR:At1g26560 InParanoid:Q9FZE0 OMA:NDFAAYA PhylomeDB:Q9FZE0 ProtClustDB:CLSN2682658 ArrayExpress:Q9FZE0 Uniprot:Q9FZE0
Root	Isotig00773	15	58	-2.080	3.03E-08	TAIR|locus:2151581 - symbol:AT5G61510 species:3702 "Arabidopsis thaliana" [GO:0003824 "catalytic activity" evidence=IEA] [GO:0008270 "zinc ion binding" evidence=IEA] [GO:0016491 "oxidoreductase activity" evidence=IEA] [GO:0009644 "response to high light intensity" evidence=IEP] [GO:0005829 "cytosol" evidence=IDA] [GO:0030154 "cell differentiation" evidence=IEP] InterPro:IPR002085 InterPro:IPR013149 InterPro:IPR013154 InterPro:IPR016040 Pfam:PF00107 Pfam:PF08240 GO:GO:0005829 EMBL:CP002688 GenomeReviews:BA000015_GR GO:GO:0000166 Gene3D:G3DSA:3.40.50.720 GO:GO:0008270 GO:GO:0016491 HOGENOM:HBG753318 InterPro:IPR011032 PANTHER:PTHR11695 SUPFAM:SSF50129 GO:GO:0009644 eggNOG:COG0604 KO:K00344 EMBL:BT029771 IPI:IPI00528545 RefSeq:NP_200959.2 UniGene:At.29101 ProteinModelPortal:A1L4Y4 SMR:A1L4Y4 PRIDE:A1L4Y4 EnsemblPlants:AT5G61510.1 GeneID:836272 KEGG:ath:AT5G61510 TAIR:At5g61510 InParanoid:A1L4Y4 OMA:AEEQILP PhylomeDB:A1L4Y4 ProtClustDB:CLSN2681116 Genevestigator:A1L4Y4 Uniprot:A1L4Y4
Root	Isotig00774	15	57	-2.055	5.06E-08	TAIR|locus:2151581 - symbol:AT5G61510 species:3702 "Arabidopsis thaliana" [GO:0003824 "catalytic activity" evidence=IEA] [GO:0008270 "zinc ion binding" evidence=IEA] [GO:0016491 "oxidoreductase activity" evidence=IEA] [GO:0009644 "response to high light intensity" evidence=IEP] [GO:0005829 "cytosol" evidence=IDA] [GO:0030154 "cell differentiation" evidence=IEP] InterPro:IPR002085 InterPro:IPR013149 InterPro:IPR013154 InterPro:IPR016040 Pfam:PF00107 Pfam:PF08240 GO:GO:0005829 EMBL:CP002688

Tissue	Identification	# of reads (stress)	# of reads (control)	Fold Change	p-value	Description
						GenomeReviews:BA000015_GR GO:GO:0000166 Gene3D:G3DSA:3.40.50.720 GO:GO:0008270 GO:GO:0016491 HOGENOM:HBG753318 InterPro:IPR011032 PANTHER:PTHR11695 SUPFAM:SSF50129 GO:GO:0009644 eggNOG:COG0604 KO:K00344 EMBL:BT029771 IPI:IPI00528545 RefSeq:NP_200959.2 UniGene:At.29101 ProteinModelPortal:A1L4Y4 SMR:A1L4Y4 PRIDE:A1L4Y4 EnsemblPlants:AT5G61510.1 GeneID:836272 KEGG:ath:AT5G61510 TAIR:At5g61510 InParanoid:A1L4Y4 OMA:AEEQILP PhylomeDB:A1L4Y4 ProtClustDB:CLSN2681116 Genevestigator:A1L4Y4 Uniprot:A1L4Y4
Root	Isotig00775	15	57	-2.055	5.06E-08	TAIR|locus:2151581 - symbol:AT5G61510 species:3702 "Arabidopsis thaliana" [GO:0003824 "catalytic activity" evidence=IEA] [GO:0008270 "zinc ion binding" evidence=IEA] [GO:0016491 "oxidoreductase activity" evidence=IEA] [GO:0009644 "response to high light intensity" evidence=IEP] [GO:0005829 "cytosol" evidence=IDA] [GO:0030154 "cell differentiation" evidence=IEP] InterPro:IPR002085 InterPro:IPR013149 InterPro:IPR013154 InterPro:IPR016040 Pfam:PF00107 Pfam:PF08240 GO:GO:0005829 EMBL:CP002688 GenomeReviews:BA000015_GR GO:GO:0000166 Gene3D:G3DSA:3.40.50.720 GO:GO:0008270 GO:GO:0016491 HOGENOM:HBG753318 InterPro:IPR011032 PANTHER:PTHR11695 SUPFAM:SSF50129 GO:GO:0009644 eggNOG:COG0604 KO:K00344 EMBL:BT029771 IPI:IPI00528545 RefSeq:NP_200959.2 UniGene:At.29101 ProteinModelPortal:A1L4Y4 SMR:A1L4Y4 PRIDE:A1L4Y4 EnsemblPlants:AT5G61510.1 GeneID:836272 KEGG:ath:AT5G61510 TAIR:At5g61510 InParanoid:A1L4Y4 OMA:AEEQILP PhylomeDB:A1L4Y4 ProtClustDB:CLSN2681116 Genevestigator:A1L4Y4 Uniprot:A1L4Y4
Root	Isotig00776	15	56	-2.029	8.42E-08	TAIR|locus:2151581 - symbol:AT5G61510 species:3702 "Arabidopsis thaliana" [GO:0003824 "catalytic activity" evidence=IEA] [GO:0008270 "zinc ion binding" evidence=IEA] [GO:0016491 "oxidoreductase activity" evidence=IEA] [GO:0009644 "response to high light intensity" evidence=IEP] [GO:0005829 "cytosol" evidence=IDA] [GO:0030154 "cell differentiation" evidence=IEP] InterPro:IPR002085 InterPro:IPR013149 InterPro:IPR013154 InterPro:IPR016040 Pfam:PF00107 Pfam:PF08240 GO:GO:0005829 EMBL:CP002688 GenomeReviews:BA000015_GR GO:GO:0000166 Gene3D:G3DSA:3.40.50.720 GO:GO:0008270 GO:GO:0016491 HOGENOM:HBG753318 InterPro:IPR011032 PANTHER:PTHR11695 SUPFAM:SSF50129 GO:GO:0009644 eggNOG:COG0604 KO:K00344 EMBL:BT029771 IPI:IPI00528545 RefSeq:NP_200959.2 UniGene:At.29101 ProteinModelPortal:A1L4Y4 SMR:A1L4Y4 PRIDE:A1L4Y4 EnsemblPlants:AT5G61510.1 GeneID:836272 KEGG:ath:AT5G61510 TAIR:At5g61510 InParanoid:A1L4Y4 OMA:AEEQILP PhylomeDB:A1L4Y4 ProtClustDB:CLSN2681116 Genevestigator:A1L4Y4 Uniprot:A1L4Y4
Root	Isotig00777	0	48	-6.714	3.16E-13	TAIR|locus:2089428 - symbol:LBO1 "AT3G21420" species:3702 "Arabidopsis thaliana" [GO:0005575 "cellular_component" evidence=ND] [GO:0008152 "metabolic process" evidence=ISS] [GO:0016491 "oxidoreductase activity" evidence=IEA;ISS] InterPro:IPR005123 Pfam:PF03171 PROSITE:PS51471 EMBL:CP002686 GenomeReviews:BA000014_GR eggNOG:COG3491 HOGENOM:HBG749762 GO:GO:0016706 HSSP:Q96323 EMBL:AP001305 EMBL:AY084795 EMBL:BT005988 EMBL:AK227427 IPI:IPI00545861 RefSeq:NP_566685.1 UniGene:At.38063 ProteinModelPortal:Q9LIF4 SMR:Q9LIF4 PRIDE:Q9LIF4 EnsemblPlants:AT3G21420.1 GeneID:821696 KEGG:ath:AT3G21420 TAIR:At3g21420 InParanoid:Q9LIF4 OMA:FIREEYE PhylomeDB:Q9LIF4 ProtClustDB:PLN02758 Genevestigator:Q9LIF4 Uniprot:Q9LIF4
Root	Isotig00778	0	47	-6.684	5.35E-13	TAIR|locus:2089428 - symbol:LBO1 "AT3G21420" species:3702 "Arabidopsis thaliana" [GO:0005575 "cellular_component" evidence=ND] [GO:0008152 "metabolic process" evidence=ISS] [GO:0016491 "oxidoreductase activity" evidence=IEA;ISS] InterPro:IPR005123 Pfam:PF03171 PROSITE:PS51471 EMBL:CP002686 GenomeReviews:BA000014_GR eggNOG:COG3491 HOGENOM:HBG749762 GO:GO:0016706 HSSP:Q96323 EMBL:AP001305 EMBL:AY084795 EMBL:BT005988 EMBL:AK227427 IPI:IPI00545861 RefSeq:NP_566685.1 UniGene:At.38063 ProteinModelPortal:Q9LIF4 SMR:Q9LIF4 PRIDE:Q9LIF4 EnsemblPlants:AT3G21420.1 GeneID:821696 KEGG:ath:AT3G21420 TAIR:At3g21420 InParanoid:Q9LIF4 OMA:FIREEYE PhylomeDB:Q9LIF4 ProtClustDB:PLN02758 Genevestigator:Q9LIF4 Uniprot:Q9LIF4
Root	Isotig00779	0	47	-6.684	5.35E-13	TAIR|locus:2089428 - symbol:LBO1 "AT3G21420" species:3702 "Arabidopsis thaliana" [GO:0005575 "cellular_component" evidence=ND] [GO:0008152 "metabolic process" evidence=ISS] [GO:0016491 "oxidoreductase activity" evidence=IEA;ISS] InterPro:IPR005123 Pfam:PF03171 PROSITE:PS51471 EMBL:CP002686 GenomeReviews:BA000014_GR eggNOG:COG3491 HOGENOM:HBG749762 GO:GO:0016706 HSSP:Q96323 EMBL:AP001305 EMBL:AY084795 EMBL:BT005988 EMBL:AK227427 IPI:IPI00545861 RefSeq:NP_566685.1 UniGene:At.38063 ProteinModelPortal:Q9LIF4 SMR:Q9LIF4 PRIDE:Q9LIF4 EnsemblPlants:AT3G21420.1 GeneID:821696 KEGG:ath:AT3G21420 TAIR:At3g21420 InParanoid:Q9LIF4 OMA:FIREEYE PhylomeDB:Q9LIF4 ProtClustDB:PLN02758 Genevestigator:Q9LIF4 Uniprot:Q9LIF4

Tissue	Identification	# of reads (stress)	# of reads (control)	Fold Change	p-value	Description
Root	Isotig00780	0	46	-6.653	9.06E-13	TAIR|locus:2089428 - symbol:LBO1 "AT3G21420" species:3702 "Arabidopsis thaliana" [GO:0005575 "cellular_component" evidence=ND] [GO:0008152 "metabolic process" evidence=ISS] [GO:0016491 "oxidoreductase activity" evidence=IEA;ISS] InterPro:IPR005123 Pfam:PF03171 PROSITE:PS51471 EMBL:CP002686 GenomeReviews:BA000014_GR eggNOG:COG3491 HOGENOM:HBG749762 GO:GO:0016706 HSSP:Q96323 EMBL:AP001305 EMBL:AY084795 EMBL:BT005988 EMBL:AK227427 IPI:IPI00545861 RefSeq:NP_566685.1 UniGene:At.38063 ProteinModelPortal:Q9LIF4 SMR:Q9LIF4 PRIDE:Q9LIF4 EnsemblPlants:AT3G21420.1 GeneID:821696 KEGG:ath:AT3G21420 TAIR:At3g21420 InParanoid:Q9LIF4 OMA:FIREEYE PhylomeDB:Q9LIF4 ProtClustDB:PLN02758 Genevestigator:Q9LIF4 Uniprot:Q9LIF4
Root	Isotig00781	0	47	-6.684	5.35E-13	TAIR|locus:2089428 - symbol:LBO1 "AT3G21420" species:3702 "Arabidopsis thaliana" [GO:0005575 "cellular_component" evidence=ND] [GO:0008152 "metabolic process" evidence=ISS] [GO:0016491 "oxidoreductase activity" evidence=IEA;ISS] InterPro:IPR005123 Pfam:PF03171 PROSITE:PS51471 EMBL:CP002686 GenomeReviews:BA000014_GR eggNOG:COG3491 HOGENOM:HBG749762 GO:GO:0016706 HSSP:Q96323 EMBL:AP001305 EMBL:AY084795 EMBL:BT005988 EMBL:AK227427 IPI:IPI00545861 RefSeq:NP_566685.1 UniGene:At.38063 ProteinModelPortal:Q9LIF4 SMR:Q9LIF4 PRIDE:Q9LIF4 EnsemblPlants:AT3G21420.1 GeneID:821696 KEGG:ath:AT3G21420 TAIR:At3g21420 InParanoid:Q9LIF4 OMA:FIREEYE PhylomeDB:Q9LIF4 ProtClustDB:PLN02758 Genevestigator:Q9LIF4 Uniprot:Q9LIF4
Root	Isotig00782	0	46	-6.653	9.06E-13	TAIR|locus:2089428 - symbol:LBO1 "AT3G21420" species:3702 "Arabidopsis thaliana" [GO:0005575 "cellular_component" evidence=ND] [GO:0008152 "metabolic process" evidence=ISS] [GO:0016491 "oxidoreductase activity" evidence=IEA;ISS] InterPro:IPR005123 Pfam:PF03171 PROSITE:PS51471 EMBL:CP002686 GenomeReviews:BA000014_GR eggNOG:COG3491 HOGENOM:HBG749762 GO:GO:0016706 HSSP:Q96323 EMBL:AP001305 EMBL:AY084795 EMBL:BT005988 EMBL:AK227427 IPI:IPI00545861 RefSeq:NP_566685.1 UniGene:At.38063 ProteinModelPortal:Q9LIF4 SMR:Q9LIF4 PRIDE:Q9LIF4 EnsemblPlants:AT3G21420.1 GeneID:821696 KEGG:ath:AT3G21420 TAIR:At3g21420 InParanoid:Q9LIF4 OMA:FIREEYE PhylomeDB:Q9LIF4 ProtClustDB:PLN02758 Genevestigator:Q9LIF4 Uniprot:Q9LIF4
Root	Isotig00783	0	46	-6.653	9.06E-13	TAIR|locus:2089428 - symbol:LBO1 "AT3G21420" species:3702 "Arabidopsis thaliana" [GO:0005575 "cellular_component" evidence=ND] [GO:0008152 "metabolic process" evidence=ISS] [GO:0016491 "oxidoreductase activity" evidence=IEA;ISS] InterPro:IPR005123 Pfam:PF03171 PROSITE:PS51471 EMBL:CP002686 GenomeReviews:BA000014_GR eggNOG:COG3491 HOGENOM:HBG749762 GO:GO:0016706 HSSP:Q96323 EMBL:AP001305 EMBL:AY084795 EMBL:BT005988 EMBL:AK227427 IPI:IPI00545861 RefSeq:NP_566685.1 UniGene:At.38063 ProteinModelPortal:Q9LIF4 SMR:Q9LIF4 PRIDE:Q9LIF4 EnsemblPlants:AT3G21420.1 GeneID:821696 KEGG:ath:AT3G21420 TAIR:At3g21420 InParanoid:Q9LIF4 OMA:FIREEYE PhylomeDB:Q9LIF4 ProtClustDB:PLN02758 Genevestigator:Q9LIF4 Uniprot:Q9LIF4
Root	Isotig00784	0	45	-6.621	1.54E-12	TAIR|locus:2089428 - symbol:LBO1 "AT3G21420" species:3702 "Arabidopsis thaliana" [GO:0005575 "cellular_component" evidence=ND] [GO:0008152 "metabolic process" evidence=ISS] [GO:0016491 "oxidoreductase activity" evidence=IEA;ISS] InterPro:IPR005123 Pfam:PF03171 PROSITE:PS51471 EMBL:CP002686 GenomeReviews:BA000014_GR eggNOG:COG3491 HOGENOM:HBG749762 GO:GO:0016706 HSSP:Q96323 EMBL:AP001305 EMBL:AY084795 EMBL:BT005988 EMBL:AK227427 IPI:IPI00545861 RefSeq:NP_566685.1 UniGene:At.38063 ProteinModelPortal:Q9LIF4 SMR:Q9LIF4 PRIDE:Q9LIF4 EnsemblPlants:AT3G21420.1 GeneID:821696 KEGG:ath:AT3G21420 TAIR:At3g21420 InParanoid:Q9LIF4 OMA:FIREEYE PhylomeDB:Q9LIF4 ProtClustDB:PLN02758 Genevestigator:Q9LIF4 Uniprot:Q9LIF4
Root	Isotig00786	131	270	-1.172	3.12E-15	ZFIN|ZDB-GENE-070410-9 - symbol:zgc:158463 "zgc:158463" species:7955 "Danio rerio" [GO:0003674 "molecular_function" evidence=ND] [GO:0005575 "cellular_component" evidence=ND] [GO:0008150 "biological_process" evidence=ND] ZFIN:ZDB-GENE-070410-9 EMBL:BX296557 EMBL:BX537263 EMBL:BC129240 IPI:IPI00837201 RefSeq:NP_001091866.1 RefSeq:XP_003200836.1 UniGene:Dr.155369 Ensembl:ENSDART00000102863 Ensembl:ENSDART00000126982 GeneID:100037361 GeneID:100151188 KEGG:dre:100037361 KEGG:dre:100151188 eggNOG:NOG298153 GeneTree:ENSGT00390000016898 InParanoid:A2VD29 OMA:ACHQHAL OrthoDB:EOG4FJ8BF Bgee:A2VD29 Uniprot:A2VD29
Root	Isotig00787	131	269	-1.167	4.41E-15	ZFIN|ZDB-GENE-070410-9 - symbol:zgc:158463 "zgc:158463" species:7955 "Danio rerio" [GO:0003674 "molecular_function" evidence=ND] [GO:0005575 "cellular_component" evidence=ND] [GO:0008150 "biological_process" evidence=ND] ZFIN:ZDB-GENE-070410-9 EMBL:BX296557 EMBL:BX537263 EMBL:BC129240 IPI:IPI00837201 RefSeq:NP_001091866.1 RefSeq:XP_003200836.1 UniGene:Dr.155369 Ensembl:ENSDART00000102863 Ensembl:ENSDART00000126982 GeneID:100037361 GeneID:100151188

Tissue	Identification	# of reads (stress)	# of reads (control)	Fold Change	p-value	Description
						KEGG:dre:100037361 KEGG:dre:100151188 eggNOG:NOG298153 GeneTree:ENSGT00390000016898 InParanoid:A2VD29 OMA:ACHQHAL OrthoDB:EOG4FJ8BF Bgee:A2VD29 Uniprot:A2VD29
Root	Isotig00788	218	456	-1.194	2.53E-25	ZFIN|ZDB-GENE-070410-9 - symbol:zgc:158463 "zgc:158463" species:7955 "Danio rerio" [GO:0003674 "molecular_function" evidence=ND] [GO:0005575 "cellular_component" evidence=ND] [GO:0008150 "biological_process" evidence=ND] ZFIN:ZDB-GENE-070410-9 EMBL:BX296557 EMBL:BX537263 EMBL:BC129240 IPI:IPI00837201 RefSeq:NP_001091866.1 RefSeq:XP_003200836.1 UniGene:Dr.155369 Ensembl:ENSDART00000102863 Ensembl:ENSDART00000126982 GeneID:100037361 GeneID:100151188 KEGG:dre:100037361 KEGG:dre:100151188 eggNOG:NOG298153 GeneTree:ENSGT00390000016898 InParanoid:A2VD29 OMA:ACHQHAL OrthoDB:EOG4FJ8BF Bgee:A2VD29 Uniprot:A2VD29
Root	Isotig00789	218	455	-1.191	3.59E-25	ZFIN|ZDB-GENE-070410-9 - symbol:zgc:158463 "zgc:158463" species:7955 "Danio rerio" [GO:0003674 "molecular_function" evidence=ND] [GO:0005575 "cellular_component" evidence=ND] [GO:0008150 "biological_process" evidence=ND] ZFIN:ZDB-GENE-070410-9 EMBL:BX296557 EMBL:BX537263 EMBL:BC129240 IPI:IPI00837201 RefSeq:NP_001091866.1 RefSeq:XP_003200836.1 UniGene:Dr.155369 Ensembl:ENSDART00000102863 Ensembl:ENSDART00000126982 GeneID:100037361 GeneID:100151188 KEGG:dre:100037361 KEGG:dre:100151188 eggNOG:NOG298153 GeneTree:ENSGT00390000016898 InParanoid:A2VD29 OMA:ACHQHAL OrthoDB:EOG4FJ8BF Bgee:A2VD29 Uniprot:A2VD29
Root	Isotig00790	130	270	-1.183	1.94E-15	ZFIN|ZDB-GENE-070410-9 - symbol:zgc:158463 "zgc:158463" species:7955 "Danio rerio" [GO:0003674 "molecular_function" evidence=ND] [GO:0005575 "cellular_component" evidence=ND] [GO:0008150 "biological_process" evidence=ND] ZFIN:ZDB-GENE-070410-9 EMBL:BX296557 EMBL:BX537263 EMBL:BC129240 IPI:IPI00837201 RefSeq:NP_001091866.1 RefSeq:XP_003200836.1 UniGene:Dr.155369 Ensembl:ENSDART00000102863 Ensembl:ENSDART00000126982 GeneID:100037361 GeneID:100151188 KEGG:dre:100037361 KEGG:dre:100151188 eggNOG:NOG298153 GeneTree:ENSGT00390000016898 InParanoid:A2VD29 OMA:ACHQHAL OrthoDB:EOG4FJ8BF Bgee:A2VD29 Uniprot:A2VD29
Root	Isotig00791	130	269	-1.178	2.75E-15	ZFIN|ZDB-GENE-070410-9 - symbol:zgc:158463 "zgc:158463" species:7955 "Danio rerio" [GO:0003674 "molecular_function" evidence=ND] [GO:0005575 "cellular_component" evidence=ND] [GO:0008150 "biological_process" evidence=ND] ZFIN:ZDB-GENE-070410-9 EMBL:BX296557 EMBL:BX537263 EMBL:BC129240 IPI:IPI00837201 RefSeq:NP_001091866.1 RefSeq:XP_003200836.1 UniGene:Dr.155369 Ensembl:ENSDART00000102863 Ensembl:ENSDART00000126982 GeneID:100037361 GeneID:100151188 KEGG:dre:100037361 KEGG:dre:100151188 eggNOG:NOG298153 GeneTree:ENSGT00390000016898 InParanoid:A2VD29 OMA:ACHQHAL OrthoDB:EOG4FJ8BF Bgee:A2VD29 Uniprot:A2VD29
Root	Isotig00792	217	456	-1.200	1.56E-25	ZFIN|ZDB-GENE-070410-9 - symbol:zgc:158463 "zgc:158463" species:7955 "Danio rerio" [GO:0003674 "molecular_function" evidence=ND] [GO:0005575 "cellular_component" evidence=ND] [GO:0008150 "biological_process" evidence=ND] ZFIN:ZDB-GENE-070410-9 EMBL:BX296557 EMBL:BX537263 EMBL:BC129240 IPI:IPI00837201 RefSeq:NP_001091866.1 RefSeq:XP_003200836.1 UniGene:Dr.155369 Ensembl:ENSDART00000102863 Ensembl:ENSDART00000126982 GeneID:100037361 GeneID:100151188 KEGG:dre:100037361 KEGG:dre:100151188 eggNOG:NOG298153 GeneTree:ENSGT00390000016898 InParanoid:A2VD29 OMA:ACHQHAL OrthoDB:EOG4FJ8BF Bgee:A2VD29 Uniprot:A2VD29
Root	Isotig00793	217	455	-1.197	2.22E-25	ZFIN|ZDB-GENE-070410-9 - symbol:zgc:158463 "zgc:158463" species:7955 "Danio rerio" [GO:0003674 "molecular_function" evidence=ND] [GO:0005575 "cellular_component" evidence=ND] [GO:0008150 "biological_process" evidence=ND] ZFIN:ZDB-GENE-070410-9 EMBL:BX296557 EMBL:BX537263 EMBL:BC129240 IPI:IPI00837201 RefSeq:NP_001091866.1 RefSeq:XP_003200836.1 UniGene:Dr.155369 Ensembl:ENSDART00000102863 Ensembl:ENSDART00000126982 GeneID:100037361 GeneID:100151188 KEGG:dre:100037361 KEGG:dre:100151188 eggNOG:NOG298153 GeneTree:ENSGT00390000016898 InParanoid:A2VD29 OMA:ACHQHAL OrthoDB:EOG4FJ8BF Bgee:A2VD29 Uniprot:A2VD29
Root	Isotig00794	100	206	-1.172	5.81E-12	ZFIN|ZDB-GENE-070410-9 - symbol:zgc:158463 "zgc:158463" species:7955 "Danio rerio" [GO:0003674 "molecular_function" evidence=ND] [GO:0005575 "cellular_component" evidence=ND] [GO:0008150 "biological_process" evidence=ND] ZFIN:ZDB-GENE-070410-9 EMBL:BX296557 EMBL:BX537263 EMBL:BC129240 IPI:IPI00837201 RefSeq:NP_001091866.1 RefSeq:XP_003200836.1 UniGene:Dr.155369 Ensembl:ENSDART00000102863 Ensembl:ENSDART00000126982 GeneID:100037361 GeneID:100151188 KEGG:dre:100037361 KEGG:dre:100151188 eggNOG:NOG298153 GeneTree:ENSGT00390000016898 InParanoid:A2VD29 OMA:ACHQHAL OrthoDB:EOG4FJ8BF Bgee:A2VD29 Uniprot:A2VD29

Tissue	Identification	# of reads (stress)	# of reads (control)	Fold Change	p-value	Description
Root	Isotig00795	100	205	-1.165	8.21E-12	ZFIN|ZDB-GENE-070410-9 - symbol:zgc:158463 "zgc:158463" species:7955 "Danio rerio" [GO:0003674 "molecular_function" evidence=ND] [GO:0005575 "cellular_component" evidence=ND] [GO:0008150 "biological_process" evidence=ND] ZFIN:ZDB-GENE-070410-9 EMBL:BX296557 EMBL:BX537263 EMBL:BC129240 IPI:IPI00837201 RefSeq:NP_001091866.1 RefSeq:XP_003200836.1 UniGene:Dr.155369 Ensembl:ENSDART00000102863 Ensembl:ENSDART00000126982 GeneID:100037361 GeneID:100151188 KEGG:dre:100037361 KEGG:dre:100151188 eggNOG:NOG298153 GeneTree:ENSGT00390000016898 InParanoid:A2VD29 OMA:ACHQHAL OrthoDB:EOG4FJ8BF Bgee:A2VD29 Uniprot:A2VD29
Root	Isotig00796	132	268	-1.151	9.91E-15	No hit
Root	Isotig00797	99	206	-1.186	3.60E-12	ZFIN|ZDB-GENE-070410-9 - symbol:zgc:158463 "zgc:158463" species:7955 "Danio rerio" [GO:0003674 "molecular_function" evidence=ND] [GO:0005575 "cellular_component" evidence=ND] [GO:0008150 "biological_process" evidence=ND] ZFIN:ZDB-GENE-070410-9 EMBL:BX296557 EMBL:BX537263 EMBL:BC129240 IPI:IPI00837201 RefSeq:NP_001091866.1 RefSeq:XP_003200836.1 UniGene:Dr.155369 Ensembl:ENSDART00000102863 Ensembl:ENSDART00000126982 GeneID:100037361 GeneID:100151188 KEGG:dre:100037361 KEGG:dre:100151188 eggNOG:NOG298153 GeneTree:ENSGT00390000016898 InParanoid:A2VD29 OMA:ACHQHAL OrthoDB:EOG4FJ8BF Bgee:A2VD29 Uniprot:A2VD29
Root	Isotig00798	99	205	-1.179	5.11E-12	ZFIN|ZDB-GENE-070410-9 - symbol:zgc:158463 "zgc:158463" species:7955 "Danio rerio" [GO:0003674 "molecular_function" evidence=ND] [GO:0005575 "cellular_component" evidence=ND] [GO:0008150 "biological_process" evidence=ND] ZFIN:ZDB-GENE-070410-9 EMBL:BX296557 EMBL:BX537263 EMBL:BC129240 IPI:IPI00837201 RefSeq:NP_001091866.1 RefSeq:XP_003200836.1 UniGene:Dr.155369 Ensembl:ENSDART00000102863 Ensembl:ENSDART00000126982 GeneID:100037361 GeneID:100151188 KEGG:dre:100037361 KEGG:dre:100151188 eggNOG:NOG298153 GeneTree:ENSGT00390000016898 InParanoid:A2VD29 OMA:ACHQHAL OrthoDB:EOG4FJ8BF Bgee:A2VD29 Uniprot:A2VD29
Root	Isotig00799	1	22	-4.588	1.10E-06	TAIR|locus:2097993 - symbol:AT3G62160 "AT3G62160" species:3702 "Arabidopsis thaliana" [GO:0005575 "cellular_component" evidence=ND] [GO:0008150 "biological_process" evidence=ND] [GO:0016740 "transferase activity" evidence=ISS] [GO:0016747 "transferase activity, transferring acyl groups other than amino-acyl groups" evidence=IEA] InterPro:IPR003480 Pfam:PF02458 EMBL:CP002686 GenomeReviews:BA000014_GR InterPro:IPR023213 Gene3D:G3DSA:3.30.559.10 GO:GO:0016747 EMBL:AL138651 HOGENOM:HBG599319 EMBL:BT020224 EMBL:BT020438 IPI:IPI00527765 PIR:T48008 RefSeq:NP_191775.1 UniGene:At.34171 ProteinModelPortal:Q9M1Q8 PRIDE:Q9M1Q8 EnsemblPlants:AT3G62160.1 GeneID:825389 KEGG:ath:AT3G62160 TAIR:At3g62160 eggNOG:NOG284877 InParanoid:Q9M1Q8 OMA:GFVMGLR PhylomeDB:Q9M1Q8 ProtClustDB:CLSN2684119 Genevestigator:Q9M1Q8 Uniprot:Q9M1Q8
Root	Isotig00800	1	22	-4.588	1.10E-06	TAIR|locus:2097993 - symbol:AT3G62160 "AT3G62160" species:3702 "Arabidopsis thaliana" [GO:0005575 "cellular_component" evidence=ND] [GO:0008150 "biological_process" evidence=ND] [GO:0016740 "transferase activity" evidence=ISS] [GO:0016747 "transferase activity, transferring acyl groups other than amino-acyl groups" evidence=IEA] InterPro:IPR003480 Pfam:PF02458 EMBL:CP002686 GenomeReviews:BA000014_GR InterPro:IPR023213 Gene3D:G3DSA:3.30.559.10 GO:GO:0016747 EMBL:AL138651 HOGENOM:HBG599319 EMBL:BT020224 EMBL:BT020438 IPI:IPI00527765 PIR:T48008 RefSeq:NP_191775.1 UniGene:At.34171 ProteinModelPortal:Q9M1Q8 PRIDE:Q9M1Q8 EnsemblPlants:AT3G62160.1 GeneID:825389 KEGG:ath:AT3G62160 TAIR:At3g62160 eggNOG:NOG284877 InParanoid:Q9M1Q8 OMA:GFVMGLR PhylomeDB:Q9M1Q8 ProtClustDB:CLSN2684119 Genevestigator:Q9M1Q8 Uniprot:Q9M1Q8
Root	Isotig00801	0	23	-5.653	3.32E-07	TAIR|locus:2097993 - symbol:AT3G62160 "AT3G62160" species:3702 "Arabidopsis thaliana" [GO:0005575 "cellular_component" evidence=ND] [GO:0008150 "biological_process" evidence=ND] [GO:0016740 "transferase activity" evidence=ISS] [GO:0016747 "transferase activity, transferring acyl groups other than amino-acyl groups" evidence=IEA] InterPro:IPR003480 Pfam:PF02458 EMBL:CP002686 GenomeReviews:BA000014_GR InterPro:IPR023213 Gene3D:G3DSA:3.30.559.10 GO:GO:0016747 EMBL:AL138651 HOGENOM:HBG599319 EMBL:BT020224 EMBL:BT020438 IPI:IPI00527765 PIR:T48008 RefSeq:NP_191775.1 UniGene:At.34171 ProteinModelPortal:Q9M1Q8 PRIDE:Q9M1Q8 EnsemblPlants:AT3G62160.1 GeneID:825389 KEGG:ath:AT3G62160 TAIR:At3g62160 eggNOG:NOG284877 InParanoid:Q9M1Q8 OMA:GFVMGLR PhylomeDB:Q9M1Q8 ProtClustDB:CLSN2684119 Genevestigator:Q9M1Q8 Uniprot:Q9M1Q8
Root	Isotig00802	0	23	-5.653	3.32E-07	TAIR|locus:2097993 - symbol:AT3G62160 "AT3G62160" species:3702 "Arabidopsis thaliana" [GO:0005575 "cellular_component" evidence=ND] [GO:0008150 "biological_process" evidence=ND] [GO:0016740 "transferase activity" evidence=ISS] [GO:0016747 "transferase activity, transferring acyl groups other than amino-acyl groups"

Tissue	Identification	# of reads (stress)	# of reads (control)	Fold Change	p-value	Description
						evidence=IEA] InterPro:IPR003480 Pfam:PF02458 EMBL:CP002686 GenomeReviews:BA000014_GR InterPro:IPR023213 Gene3D:G3DSA:3.30.559.10 GO:GO:0016747 EMBL:AL138651 HOGENOM:HBG599319 EMBL:BT020224 EMBL:BT020438 IPI:IPI00527765 PIR:T48008 RefSeq:NP_191775.1 UniGene:At.34171 ProteinModelPortal:Q9M1Q8 PRIDE:Q9M1Q8 EnsemblPlants:AT3G62160.1 GeneID:825389 KEGG:ath:AT3G62160 TAIR:At3g62160 eggNOG:NOG284877 InParanoid:Q9M1Q8 OMA:GFVMGLR PhylomeDB:Q9M1Q8 ProtClustDB:CLSN2684119 Genevestigator:Q9M1Q8 Uniprot:Q9M1Q8
Root	Isotig00803	0	21	-5.521	1.09E-06	TAIR|locus:2097993 - symbol:AT3G62160 "AT3G62160" species:3702 "Arabidopsis thaliana" [GO:0005575 "cellular_component" evidence=ND] [GO:0008150 "biological_process" evidence=ND] [GO:0016740 "transferase activity" evidence=ISS] [GO:0016747 "transferase activity, transferring acyl groups other than amino-acyl groups" evidence=IEA] InterPro:IPR003480 Pfam:PF02458 EMBL:CP002686 GenomeReviews:BA000014_GR InterPro:IPR023213 Gene3D:G3DSA:3.30.559.10 GO:GO:0016747 EMBL:AL138651 HOGENOM:HBG599319 EMBL:BT020224 EMBL:BT020438 IPI:IPI00527765 PIR:T48008 RefSeq:NP_191775.1 UniGene:At.34171 ProteinModelPortal:Q9M1Q8 PRIDE:Q9M1Q8 EnsemblPlants:AT3G62160.1 GeneID:825389 KEGG:ath:AT3G62160 TAIR:At3g62160 eggNOG:NOG284877 InParanoid:Q9M1Q8 OMA:GFVMGLR PhylomeDB:Q9M1Q8 ProtClustDB:CLSN2684119 Genevestigator:Q9M1Q8 Uniprot:Q9M1Q8
Root	Isotig00804	0	21	-5.521	1.09E-06	TAIR|locus:2097993 - symbol:AT3G62160 "AT3G62160" species:3702 "Arabidopsis thaliana" [GO:0005575 "cellular_component" evidence=ND] [GO:0008150 "biological_process" evidence=ND] [GO:0016740 "transferase activity" evidence=ISS] [GO:0016747 "transferase activity, transferring acyl groups other than amino-acyl groups" evidence=IEA] InterPro:IPR003480 Pfam:PF02458 EMBL:CP002686 GenomeReviews:BA000014_GR InterPro:IPR023213 Gene3D:G3DSA:3.30.559.10 GO:GO:0016747 EMBL:AL138651 HOGENOM:HBG599319 EMBL:BT020224 EMBL:BT020438 IPI:IPI00527765 PIR:T48008 RefSeq:NP_191775.1 UniGene:At.34171 ProteinModelPortal:Q9M1Q8 PRIDE:Q9M1Q8 EnsemblPlants:AT3G62160.1 GeneID:825389 KEGG:ath:AT3G62160 TAIR:At3g62160 eggNOG:NOG284877 InParanoid:Q9M1Q8 OMA:GFVMGLR PhylomeDB:Q9M1Q8 ProtClustDB:CLSN2684119 Genevestigator:Q9M1Q8 Uniprot:Q9M1Q8
Root	Isotig00805	0	22	-5.588	6.01E-07	TAIR|locus:2097993 - symbol:AT3G62160 "AT3G62160" species:3702 "Arabidopsis thaliana" [GO:0005575 "cellular_component" evidence=ND] [GO:0008150 "biological_process" evidence=ND] [GO:0016740 "transferase activity" evidence=ISS] [GO:0016747 "transferase activity, transferring acyl groups other than amino-acyl groups" evidence=IEA] InterPro:IPR003480 Pfam:PF02458 EMBL:CP002686 GenomeReviews:BA000014_GR InterPro:IPR023213 Gene3D:G3DSA:3.30.559.10 GO:GO:0016747 EMBL:AL138651 HOGENOM:HBG599319 EMBL:BT020224 EMBL:BT020438 IPI:IPI00527765 PIR:T48008 RefSeq:NP_191775.1 UniGene:At.34171 ProteinModelPortal:Q9M1Q8 PRIDE:Q9M1Q8 EnsemblPlants:AT3G62160.1 GeneID:825389 KEGG:ath:AT3G62160 TAIR:At3g62160 eggNOG:NOG284877 InParanoid:Q9M1Q8 OMA:GFVMGLR PhylomeDB:Q9M1Q8 ProtClustDB:CLSN2684119 Genevestigator:Q9M1Q8 Uniprot:Q9M1Q8
Root	Isotig00806	0	22	-5.588	6.01E-07	TAIR|locus:2097993 - symbol:AT3G62160 "AT3G62160" species:3702 "Arabidopsis thaliana" [GO:0005575 "cellular_component" evidence=ND] [GO:0008150 "biological_process" evidence=ND] [GO:0016740 "transferase activity" evidence=ISS] [GO:0016747 "transferase activity, transferring acyl groups other than amino-acyl groups" evidence=IEA] InterPro:IPR003480 Pfam:PF02458 EMBL:CP002686 GenomeReviews:BA000014_GR InterPro:IPR023213 Gene3D:G3DSA:3.30.559.10 GO:GO:0016747 EMBL:AL138651 HOGENOM:HBG599319 EMBL:BT020224 EMBL:BT020438 IPI:IPI00527765 PIR:T48008 RefSeq:NP_191775.1 UniGene:At.34171 ProteinModelPortal:Q9M1Q8 PRIDE:Q9M1Q8 EnsemblPlants:AT3G62160.1 GeneID:825389 KEGG:ath:AT3G62160 TAIR:At3g62160 eggNOG:NOG284877 InParanoid:Q9M1Q8 OMA:GFVMGLR PhylomeDB:Q9M1Q8 ProtClustDB:CLSN2684119 Genevestigator:Q9M1Q8 Uniprot:Q9M1Q8
Root	Isotig00808	0	11	-4.588	0.000569522	TAIR|locus:2097993 - symbol:AT3G62160 "AT3G62160" species:3702 "Arabidopsis thaliana" [GO:0005575 "cellular_component" evidence=ND] [GO:0008150 "biological_process" evidence=ND] [GO:0016740 "transferase activity" evidence=ISS] [GO:0016747 "transferase activity, transferring acyl groups other than amino-acyl groups" evidence=IEA] InterPro:IPR003480 Pfam:PF02458 EMBL:CP002686 GenomeReviews:BA000014_GR InterPro:IPR023213 Gene3D:G3DSA:3.30.559.10 GO:GO:0016747 EMBL:AL138651 HOGENOM:HBG599319 EMBL:BT020224 EMBL:BT020438 IPI:IPI00527765 PIR:T48008 RefSeq:NP_191775.1 UniGene:At.34171 ProteinModelPortal:Q9M1Q8 PRIDE:Q9M1Q8 EnsemblPlants:AT3G62160.1 GeneID:825389 KEGG:ath:AT3G62160 TAIR:At3g62160 eggNOG:NOG284877 InParanoid:Q9M1Q8 OMA:GFVMGLR PhylomeDB:Q9M1Q8 ProtClustDB:CLSN2684119 Genevestigator:Q9M1Q8 Uniprot:Q9M1Q8

Tissue	Identification	# of reads (stress)	# of reads (control)	Fold Change	p-value	Description
Root	Isotig00811	15	72	-2.392	1.76E-11	TAIR|locus:2057589 - symbol:PHO2 "AT2G33770" species:3702 "Arabidopsis thaliana" [GO:0004842 "ubiquitin-protein ligase activity" evidence=ISS] [GO:0005575 "cellular_component" evidence=ND] [GO:0016036 "cellular response to phosphate starvation" evidence=IMP] [GO:0055062 "phosphate ion homeostasis" evidence=IMP] [GO:0006817 "phosphate ion transport" evidence=IMP] InterPro:IPR000608 Pfam:PF00179 PROSITE:PS50127 GO:GO:0005524 EMBL:CP002685 GenomeReviews:CT485783_GR GO:GO:0016036 GO:GO:0004842 InterPro:IPR016135 Gene3D:G3DSA:3.10.110.10 SUPFAM:SSF54495 GO:GO:0055062 EMBL:U78721 PROSITE:PS00183 GO:GO:0006817 HOGENOM:HBG319025 EMBL:DQ027037 EMBL:AY074292 EMBL:AY091326 EMBL:AK229934 EMBL:AK230162 IPI:IPI00518927 IPI:IPI00900502 PIR:D84749 RefSeq:NP_850218.1 UniGene:At.14693 ProteinModelPortal:Q8VY10 SMR:Q8VY10 STRING:Q8VY10 PRIDE:Q8VY10 EnsemblPlants:AT2G33770.1 GeneID:817943 KEGG:ath:AT2G33770 TAIR:At2g33770 eggNOG:NOG315712 InParanoid:Q8VY10 OMA:RVYEERM PhylomeDB:Q8VY10 ProtClustDB:CLSN2918162 Genevestigator:Q8VY10 Uniprot:Q8VY10
Root	Isotig00812	15	69	-2.331	9.01E-11	TAIR|locus:2057589 - symbol:PHO2 "AT2G33770" species:3702 "Arabidopsis thaliana" [GO:0004842 "ubiquitin-protein ligase activity" evidence=ISS] [GO:0005575 "cellular_component" evidence=ND] [GO:0016036 "cellular response to phosphate starvation" evidence=IMP] [GO:0055062 "phosphate ion homeostasis" evidence=IMP] [GO:0006817 "phosphate ion transport" evidence=IMP] InterPro:IPR000608 Pfam:PF00179 PROSITE:PS50127 GO:GO:0005524 EMBL:CP002685 GenomeReviews:CT485783_GR GO:GO:0016036 GO:GO:0004842 InterPro:IPR016135 Gene3D:G3DSA:3.10.110.10 SUPFAM:SSF54495 GO:GO:0055062 EMBL:U78721 PROSITE:PS00183 GO:GO:0006817 HOGENOM:HBG319025 EMBL:DQ027037 EMBL:AY074292 EMBL:AY091326 EMBL:AK229934 EMBL:AK230162 IPI:IPI00518927 IPI:IPI00900502 PIR:D84749 RefSeq:NP_850218.1 UniGene:At.14693 ProteinModelPortal:Q8VY10 SMR:Q8VY10 STRING:Q8VY10 PRIDE:Q8VY10 EnsemblPlants:AT2G33770.1 GeneID:817943 KEGG:ath:AT2G33770 TAIR:At2g33770 eggNOG:NOG315712 InParanoid:Q8VY10 OMA:RVYEERM PhylomeDB:Q8VY10 ProtClustDB:CLSN2918162 Genevestigator:Q8VY10 Uniprot:Q8VY10
Root	Isotig00813	15	72	-2.392	1.76E-11	TAIR|locus:2057589 - symbol:PHO2 "AT2G33770" species:3702 "Arabidopsis thaliana" [GO:0004842 "ubiquitin-protein ligase activity" evidence=ISS] [GO:0005575 "cellular_component" evidence=ND] [GO:0016036 "cellular response to phosphate starvation" evidence=IMP] [GO:0055062 "phosphate ion homeostasis" evidence=IMP] [GO:0006817 "phosphate ion transport" evidence=IMP] InterPro:IPR000608 Pfam:PF00179 PROSITE:PS50127 GO:GO:0005524 EMBL:CP002685 GenomeReviews:CT485783_GR GO:GO:0016036 GO:GO:0004842 InterPro:IPR016135 Gene3D:G3DSA:3.10.110.10 SUPFAM:SSF54495 GO:GO:0055062 EMBL:U78721 PROSITE:PS00183 GO:GO:0006817 HOGENOM:HBG319025 EMBL:DQ027037 EMBL:AY074292 EMBL:AY091326 EMBL:AK229934 EMBL:AK230162 IPI:IPI00518927 IPI:IPI00900502 PIR:D84749 RefSeq:NP_850218.1 UniGene:At.14693 ProteinModelPortal:Q8VY10 SMR:Q8VY10 STRING:Q8VY10 PRIDE:Q8VY10 EnsemblPlants:AT2G33770.1 GeneID:817943 KEGG:ath:AT2G33770 TAIR:At2g33770 eggNOG:NOG315712 InParanoid:Q8VY10 OMA:RVYEERM PhylomeDB:Q8VY10 ProtClustDB:CLSN2918162 Genevestigator:Q8VY10 Uniprot:Q8VY10
Root	Isotig00814	15	69	-2.331	9.01E-11	TAIR|locus:2057589 - symbol:PHO2 "AT2G33770" species:3702 "Arabidopsis thaliana" [GO:0004842 "ubiquitin-protein ligase activity" evidence=ISS] [GO:0005575 "cellular_component" evidence=ND] [GO:0016036 "cellular response to phosphate starvation" evidence=IMP] [GO:0055062 "phosphate ion homeostasis" evidence=IMP] [GO:0006817 "phosphate ion transport" evidence=IMP] InterPro:IPR000608 Pfam:PF00179 PROSITE:PS50127 GO:GO:0005524 EMBL:CP002685 GenomeReviews:CT485783_GR GO:GO:0016036 GO:GO:0004842 InterPro:IPR016135 Gene3D:G3DSA:3.10.110.10 SUPFAM:SSF54495 GO:GO:0055062 EMBL:U78721 PROSITE:PS00183 GO:GO:0006817 HOGENOM:HBG319025 EMBL:DQ027037 EMBL:AY074292 EMBL:AY091326 EMBL:AK229934 EMBL:AK230162 IPI:IPI00518927 IPI:IPI00900502 PIR:D84749 RefSeq:NP_850218.1 UniGene:At.14693 ProteinModelPortal:Q8VY10 SMR:Q8VY10 STRING:Q8VY10 PRIDE:Q8VY10 EnsemblPlants:AT2G33770.1 GeneID:817943 KEGG:ath:AT2G33770 TAIR:At2g33770 eggNOG:NOG315712 InParanoid:Q8VY10 OMA:RVYEERM PhylomeDB:Q8VY10 ProtClustDB:CLSN2918162 Genevestigator:Q8VY10 Uniprot:Q8VY10
Root	Isotig00815	16	72	-2.299	5.10E-11	TAIR|locus:2057589 - symbol:PHO2 "AT2G33770" species:3702 "Arabidopsis thaliana" [GO:0004842 "ubiquitin-protein ligase activity" evidence=ISS] [GO:0005575 "cellular_component" evidence=ND] [GO:0016036 "cellular response to phosphate starvation" evidence=IMP] [GO:0055062 "phosphate ion homeostasis" evidence=IMP] [GO:0006817 "phosphate ion transport" evidence=IMP] InterPro:IPR000608 Pfam:PF00179 PROSITE:PS50127

Tissue	Identification	# of reads (stress)	# of reads (control)	Fold Change	p-value	Description
						GO:GO:0005524 EMBL:CP002685 GenomeReviews:CT485783_GR GO:GO:0016036 GO:GO:0004842 InterPro:IPR016135 Gene3D:G3DSA:3.10.110.10 SUPFAM:SSF54495 GO:GO:0055062 EMBL:U78721 PROSITE:PS00183 GO:GO:0006817 HOGENOM:HBG319025 EMBL:DQ027037 EMBL:AY074292 EMBL:AY091326 EMBL:AK229934 EMBL:AK230162 IPI:IPI00518927 IPI:IPI00900502 PIR:D84749 RefSeq:NP_850218.1 UniGene:At.14693 ProteinModelPortal:Q8VY10 SMR:Q8VY10 STRING:Q8VY10 PRIDE:Q8VY10 EnsemblPlants:AT2G33770.1 GeneID:817943 KEGG:ath:AT2G33770 TAIR:At2g33770 eggNOG:NOG315712 InParanoid:Q8VY10 OMA:RVYEERM PhylomeDB:Q8VY10 ProtClustDB:CLSN2918162 Genevestigator:Q8VY10 Uniprot:Q8VY10
Root	Isotig00816	16	69	-2.237	2.53E-10	TAIR|locus:2057589 - symbol:PHO2 "AT2G33770" species:3702 "Arabidopsis thaliana" [GO:0004842 "ubiquitin-protein ligase activity" evidence=ISS] [GO:0005575 "cellular_component" evidence=ND] [GO:0016036 "cellular response to phosphate starvation" evidence=IMP] [GO:0055062 "phosphate ion homeostasis" evidence=IMP] [GO:0006817 "phosphate ion transport" evidence=IMP] InterPro:IPR000608 Pfam:PF00179 PROSITE:PS50127 GO:GO:0005524 EMBL:CP002685 GenomeReviews:CT485783_GR GO:GO:0016036 GO:GO:0004842 InterPro:IPR016135 Gene3D:G3DSA:3.10.110.10 SUPFAM:SSF54495 GO:GO:0055062 EMBL:U78721 PROSITE:PS00183 GO:GO:0006817 HOGENOM:HBG319025 EMBL:DQ027037 EMBL:AY074292 EMBL:AY091326 EMBL:AK229934 EMBL:AK230162 IPI:IPI00518927 IPI:IPI00900502 PIR:D84749 RefSeq:NP_850218.1 UniGene:At.14693 ProteinModelPortal:Q8VY10 SMR:Q8VY10 STRING:Q8VY10 PRIDE:Q8VY10 EnsemblPlants:AT2G33770.1 GeneID:817943 KEGG:ath:AT2G33770 TAIR:At2g33770 eggNOG:NOG315712 InParanoid:Q8VY10 OMA:RVYEERM PhylomeDB:Q8VY10 ProtClustDB:CLSN2918162 Genevestigator:Q8VY10 Uniprot:Q8VY10
Root	Isotig00817	16	72	-2.299	5.10E-11	TAIR|locus:2057589 - symbol:PHO2 "AT2G33770" species:3702 "Arabidopsis thaliana" [GO:0004842 "ubiquitin-protein ligase activity" evidence=ISS] [GO:0005575 "cellular_component" evidence=ND] [GO:0016036 "cellular response to phosphate starvation" evidence=IMP] [GO:0055062 "phosphate ion homeostasis" evidence=IMP] [GO:0006817 "phosphate ion transport" evidence=IMP] InterPro:IPR000608 Pfam:PF00179 PROSITE:PS50127 GO:GO:0005524 EMBL:CP002685 GenomeReviews:CT485783_GR GO:GO:0016036 GO:GO:0004842 InterPro:IPR016135 Gene3D:G3DSA:3.10.110.10 SUPFAM:SSF54495 GO:GO:0055062 EMBL:U78721 PROSITE:PS00183 GO:GO:0006817 HOGENOM:HBG319025 EMBL:DQ027037 EMBL:AY074292 EMBL:AY091326 EMBL:AK229934 EMBL:AK230162 IPI:IPI00518927 IPI:IPI00900502 PIR:D84749 RefSeq:NP_850218.1 UniGene:At.14693 ProteinModelPortal:Q8VY10 SMR:Q8VY10 STRING:Q8VY10 PRIDE:Q8VY10 EnsemblPlants:AT2G33770.1 GeneID:817943 KEGG:ath:AT2G33770 TAIR:At2g33770 eggNOG:NOG315712 InParanoid:Q8VY10 OMA:RVYEERM PhylomeDB:Q8VY10 ProtClustDB:CLSN2918162 Genevestigator:Q8VY10 Uniprot:Q8VY10
Root	Isotig00818	16	69	-2.237	2.53E-10	TAIR|locus:2057589 - symbol:PHO2 "AT2G33770" species:3702 "Arabidopsis thaliana" [GO:0004842 "ubiquitin-protein ligase activity" evidence=ISS] [GO:0005575 "cellular_component" evidence=ND] [GO:0016036 "cellular response to phosphate starvation" evidence=IMP] [GO:0055062 "phosphate ion homeostasis" evidence=IMP] [GO:0006817 "phosphate ion transport" evidence=IMP] InterPro:IPR000608 Pfam:PF00179 PROSITE:PS50127 GO:GO:0005524 EMBL:CP002685 GenomeReviews:CT485783_GR GO:GO:0016036 GO:GO:0004842 InterPro:IPR016135 Gene3D:G3DSA:3.10.110.10 SUPFAM:SSF54495 GO:GO:0055062 EMBL:U78721 PROSITE:PS00183 GO:GO:0006817 HOGENOM:HBG319025 EMBL:DQ027037 EMBL:AY074292 EMBL:AY091326 EMBL:AK229934 EMBL:AK230162 IPI:IPI00518927 IPI:IPI00900502 PIR:D84749 RefSeq:NP_850218.1 UniGene:At.14693 ProteinModelPortal:Q8VY10 SMR:Q8VY10 STRING:Q8VY10 PRIDE:Q8VY10 EnsemblPlants:AT2G33770.1 GeneID:817943 KEGG:ath:AT2G33770 TAIR:At2g33770 eggNOG:NOG315712 InParanoid:Q8VY10 OMA:RVYEERM PhylomeDB:Q8VY10 ProtClustDB:CLSN2918162 Genevestigator:Q8VY10 Uniprot:Q8VY10
Root	Isotig00819	11	57	-2.502	8.21E-10	TAIR|locus:2057589 - symbol:PHO2 "AT2G33770" species:3702 "Arabidopsis thaliana" [GO:0004842 "ubiquitin-protein ligase activity" evidence=ISS] [GO:0005575 "cellular_component" evidence=ND] [GO:0016036 "cellular response to phosphate starvation" evidence=IMP] [GO:0055062 "phosphate ion homeostasis" evidence=IMP] [GO:0006817 "phosphate ion transport" evidence=IMP] InterPro:IPR000608 Pfam:PF00179 PROSITE:PS50127 GO:GO:0005524 EMBL:CP002685 GenomeReviews:CT485783_GR GO:GO:0016036 GO:GO:0004842 InterPro:IPR016135 Gene3D:G3DSA:3.10.110.10 SUPFAM:SSF54495 GO:GO:0055062 EMBL:U78721 PROSITE:PS00183 GO:GO:0006817 HOGENOM:HBG319025 EMBL:DQ027037 EMBL:AY074292 EMBL:AY091326 EMBL:AK229934 EMBL:AK230162 IPI:IPI00518927 IPI:IPI00900502 PIR:D84749

Tissue	Identification	# of reads (stress)	# of reads (control)	Fold Change	p-value	Description
						RefSeq:NP_850218.1 UniGene:At.14693 ProteinModelPortal:Q8VY10 SMR:Q8VY10 STRING:Q8VY10 PRIDE:Q8VY10 EnsemblPlants:AT2G33770.1 GeneID:817943 KEGG:ath:AT2G33770 TAIR:At2g33770 eggNOG:NOG315712 InParanoid:Q8VY10 OMA:RVYEERM PhylomeDB:Q8VY10 ProtClustDB:CLSN2918162 Genevestigator:Q8VY10 Uniprot:Q8VY10
Root	Isotig00820	11	57	-2.502	8.21E-10	TAIR|locus:2057589 - symbol:PHO2 "AT2G33770" species:3702 "Arabidopsis thaliana" [GO:0004842 "ubiquitin-protein ligase activity" evidence=ISS] [GO:0005575 "cellular_component" evidence=ND] [GO:0016036 "cellular response to phosphate starvation" evidence=IMP] [GO:0055062 "phosphate ion homeostasis" evidence=IMP] [GO:0006817 "phosphate ion transport" evidence=IMP] InterPro:IPR000608 Pfam:PF00179 PROSITE:PS50127 GO:GO:0005524 EMBL:CP002685 GenomeReviews:CT485783_GR GO:GO:0016036 GO:GO:0004842 InterPro:IPR016135 Gene3D:G3DSA:3.10.110.10 SUPFAM:SSF54495 GO:GO:0055062 EMBL:U78721 PROSITE:PS00183 GO:GO:0006817 HOGENOM:HBG319025 EMBL:DQ027037 EMBL:AY074292 EMBL:AY091326 EMBL:AK229934 EMBL:AK230162 IPI:IPI00518927 IPI:IPI00900502 PIR:D84749 RefSeq:NP_850218.1 UniGene:At.14693 ProteinModelPortal:Q8VY10 SMR:Q8VY10 STRING:Q8VY10 PRIDE:Q8VY10 EnsemblPlants:AT2G33770.1 GeneID:817943 KEGG:ath:AT2G33770 TAIR:At2g33770 eggNOG:NOG315712 InParanoid:Q8VY10 OMA:RVYEERM PhylomeDB:Q8VY10 ProtClustDB:CLSN2918162 Genevestigator:Q8VY10 Uniprot:Q8VY10
Root	Isotig00821	12	57	-2.377	2.51E-09	TAIR|locus:2057589 - symbol:PHO2 "AT2G33770" species:3702 "Arabidopsis thaliana" [GO:0004842 "ubiquitin-protein ligase activity" evidence=ISS] [GO:0005575 "cellular_component" evidence=ND] [GO:0016036 "cellular response to phosphate starvation" evidence=IMP] [GO:0055062 "phosphate ion homeostasis" evidence=IMP] [GO:0006817 "phosphate ion transport" evidence=IMP] InterPro:IPR000608 Pfam:PF00179 PROSITE:PS50127 GO:GO:0005524 EMBL:CP002685 GenomeReviews:CT485783_GR GO:GO:0016036 GO:GO:0004842 InterPro:IPR016135 Gene3D:G3DSA:3.10.110.10 SUPFAM:SSF54495 GO:GO:0055062 EMBL:U78721 PROSITE:PS00183 GO:GO:0006817 HOGENOM:HBG319025 EMBL:DQ027037 EMBL:AY074292 EMBL:AY091326 EMBL:AK229934 EMBL:AK230162 IPI:IPI00518927 IPI:IPI00900502 PIR:D84749 RefSeq:NP_850218.1 UniGene:At.14693 ProteinModelPortal:Q8VY10 SMR:Q8VY10 STRING:Q8VY10 PRIDE:Q8VY10 EnsemblPlants:AT2G33770.1 GeneID:817943 KEGG:ath:AT2G33770 TAIR:At2g33770 eggNOG:NOG315712 InParanoid:Q8VY10 OMA:RVYEERM PhylomeDB:Q8VY10 ProtClustDB:CLSN2918162 Genevestigator:Q8VY10 Uniprot:Q8VY10
Root	Isotig00822	12	57	-2.377	2.51E-09	TAIR|locus:2057589 - symbol:PHO2 "AT2G33770" species:3702 "Arabidopsis thaliana" [GO:0004842 "ubiquitin-protein ligase activity" evidence=ISS] [GO:0005575 "cellular_component" evidence=ND] [GO:0016036 "cellular response to phosphate starvation" evidence=IMP] [GO:0055062 "phosphate ion homeostasis" evidence=IMP] [GO:0006817 "phosphate ion transport" evidence=IMP] InterPro:IPR000608 Pfam:PF00179 PROSITE:PS50127 GO:GO:0005524 EMBL:CP002685 GenomeReviews:CT485783_GR GO:GO:0016036 GO:GO:0004842 InterPro:IPR016135 Gene3D:G3DSA:3.10.110.10 SUPFAM:SSF54495 GO:GO:0055062 EMBL:U78721 PROSITE:PS00183 GO:GO:0006817 HOGENOM:HBG319025 EMBL:DQ027037 EMBL:AY074292 EMBL:AY091326 EMBL:AK229934 EMBL:AK230162 IPI:IPI00518927 IPI:IPI00900502 PIR:D84749 RefSeq:NP_850218.1 UniGene:At.14693 ProteinModelPortal:Q8VY10 SMR:Q8VY10 STRING:Q8VY10 PRIDE:Q8VY10 EnsemblPlants:AT2G33770.1 GeneID:817943 KEGG:ath:AT2G33770 TAIR:At2g33770 eggNOG:NOG315712 InParanoid:Q8VY10 OMA:RVYEERM PhylomeDB:Q8VY10 ProtClustDB:CLSN2918162 Genevestigator:Q8VY10 Uniprot:Q8VY10
Root	Isotig00823	18	0	5.041	1.86E-05	UNIPROTKB|Q93RX7 - symbol:SCO6295 "Putative ABC transporter ATP-binding protein" species:1902 "Streptomyces coelicolor" [GO:0016021 "integral to membrane" evidence=IBA] [GO:0042626 "ATPase activity, coupled to transmembrane movement of substances" evidence=IBA] [GO:0055085 "transmembrane transport" evidence=IBA] InterPro:IPR001140 InterPro:IPR003439 InterPro:IPR003593 InterPro:IPR011527 InterPro:IPR017871 Pfam:PF00005 Pfam:PF00664 PROSITE:PS00211 PROSITE:PS50893 SMART:SM00382 GO:GO:0016021 GO:GO:0005524 HOGENOM:HBG758042 GO:GO:0042626 InterPro:IPR017940 SUPFAM:SSF90123 PROSITE:PS50929 HSSP:P08716 GenomeReviews:AL645882_GR KO:K06147 EMBL:AL939127 RefSeq:NP_630393.1 ProteinModelPortal:Q93RX7 GeneID:1101736 KEGG:sco:SCO6295 PATRIC:23742526 OMA:AHVTEFA PhylomeDB:Q93RX7 ProtClustDB:CLSK2519402 Uniprot:Q93RX7
Root	Isotig00824	18	0	5.041	1.86E-05	No hit
Root	Isotig00825	16	0	4.871	5.89E-05	UNIPROTKB|Q93RX7 - symbol:SCO6295 "Putative ABC transporter ATP-binding protein" species:1902 "Streptomyces coelicolor" [GO:0016021 "integral to membrane" evidence=IBA] [GO:0042626 "ATPase activity,

Tissue	Identification	# of reads (stress)	# of reads (control)	Fold Change	p-value	Description
						coupled to transmembrane movement of substances" evidence=IBA] [GO:0055085 "transmembrane transport" evidence=IBA] InterPro:IPR001140 InterPro:IPR003439 InterPro:IPR003593 InterPro:IPR011527 InterPro:IPR017871 Pfam:PF00005 Pfam:PF00664 PROSITE:PS00211 PROSITE:PS50893 SMART:SM00382 GO:GO:0016021 GO:GO:0005524 HOGENOM:HBG758042 GO:GO:0042626 InterPro:IPR017940 SUPFAM:SSF90123 PROSITE:PS50929 HSSP:P08716 GenomeReviews:AL645882_GR KO:K06147 EMBL:AL939127 RefSeq:NP_630393.1 ProteinModelPortal:Q93RX7 GeneID:1101736 KEGG:sco:SCO6295 PATRIC:23742526 OMA:AHVTEFA PhylomeDB:Q93RX7 ProtClustDB:CLSK2519402 Uniprot:Q93RX7
Root	Isotig00826	16	0	4.871	5.89E-05	No hit
Root	Isotig00854	42	121	-1.656	7.13E-12	TAIR|locus:2103085 - symbol:GAPC1 "AT3G04120" species:3702 "Arabidopsis thaliana" [GO:0006096 "glycolysis" evidence=ISS;IDA;TAS] [GO:0005739 "mitochondrion" evidence=IDA] [GO:0006979 "response to oxidative stress" evidence=IEP;IDA] [GO:0005740 "mitochondrial envelope" evidence=IDA] [GO:0005634 "nucleus" evidence=IDA] [GO:0005829 "cytosol" evidence=IDA;TAS] [GO:0006094 "gluconeogenesis" evidence=TAS] [GO:0042542 "response to hydrogen peroxide" evidence=IDA] [GO:0046686 "response to cadmium ion" evidence=IEP] [GO:0005774 "vacuolar membrane" evidence=IDA] [GO:0005886 "plasma membrane" evidence=IDA] [GO:0016020 "membrane" evidence=IDA] [GO:0009651 "response to salt stress" evidence=IEP] [GO:0009507 "chloroplast" evidence=IDA] [GO:0048046 "apoplast" evidence=IDA] [GO:0008886 "glyceraldehyde-3-phosphate dehydrogenase (NADP+) (non-phosphorylating) activity" evidence=IDA] [GO:0010154 "fruit development" evidence=IMP] [GO:0048316 "seed development" evidence=IMP] [GO:0005507 "copper ion binding" evidence=IDA] [GO:0005794 "Golgi apparatus" evidence=RCA] [GO:0004365 "glyceraldehyde-3-phosphate dehydrogenase (NAD+) (phosphorylating) activity" evidence=TAS] [GO:0006950 "response to stress" evidence=IEP] [GO:0009408 "response to heat" evidence=IEP] [GO:0009744 "response to sucrose stimulus" evidence=IEP] InterPro:IPR006424 InterPro:IPR016040 InterPro:IPR020828 InterPro:IPR020829 InterPro:IPR020830 InterPro:IPR020831 Pfam:PF00044 Pfam:PF02800 PIRSF:PIRSF000149 PRINTS:PR00078 PROSITE:PS00071 SMART:SM00846 GO:GO:0005829 GO:GO:0005886 GO:GO:0048046 GO:GO:0005634 GO:GO:0046686 GO:GO:0005774 GO:GO:0009507 EMBL:CP002686 GenomeReviews:BA000014_GR Gene3D:G3DSA:3.40.50.720 GO:GO:0051287 GO:GO:0050661 GO:GO:0005507 GO:GO:0006094 GO:GO:0009651 GO:GO:0009408 GO:GO:0005740 GO:GO:0009744 GO:GO:0048316 GO:GO:0042542 GO:GO:0006096 EMBL:AC016829 eggNOG:COG0057 HOGENOM:HBG571736 PANTHER:PTHR10836 GO:GO:0004365 KO:K00134 TIGRFAMs:TIGR01534 OMA:CESTGVF EMBL:M64116 EMBL:M64119 EMBL:AY052267 EMBL:AY060521 EMBL:AY140084 EMBL:F20074 IPI:IPI00527494 PIR:JQ1287 RefSeq:NP_187062.1 UniGene:At.22963 UniGene:At.24406 UniGene:At.71328 ProteinModelPortal:P25858 SMR:P25858 STRING:P25858 PRIDE:P25858 EnsemblPlants:AT3G04120.1 GeneID:819567 KEGG:ath:AT3G04120 TAIR:At3g04120 InParanoid:P25858 PhylomeDB:P25858 ProtClustDB:PLN02358 ArrayExpress:P25858 Genevestigator:P25858 GermOnline:AT3G04120 GO:GO:0008886 Uniprot:P25858
Root	Isotig00855	38	102	-1.553	1.84E-09	TAIR|locus:2103085 - symbol:GAPC1 "AT3G04120" species:3702 "Arabidopsis thaliana" [GO:0006096 "glycolysis" evidence=ISS;IDA;TAS] [GO:0005739 "mitochondrion" evidence=IDA] [GO:0006979 "response to oxidative stress" evidence=IEP;IDA] [GO:0005740 "mitochondrial envelope" evidence=IDA] [GO:0005634 "nucleus" evidence=IDA] [GO:0005829 "cytosol" evidence=IDA;TAS] [GO:0006094 "gluconeogenesis" evidence=TAS] [GO:0042542 "response to hydrogen peroxide" evidence=IDA] [GO:0046686 "response to cadmium ion" evidence=IEP] [GO:0005774 "vacuolar membrane" evidence=IDA] [GO:0005886 "plasma membrane" evidence=IDA] [GO:0016020 "membrane" evidence=IDA] [GO:0009651 "response to salt stress" evidence=IEP] [GO:0009507 "chloroplast" evidence=IDA] [GO:0048046 "apoplast" evidence=IDA] [GO:0008886 "glyceraldehyde-3-phosphate dehydrogenase (NADP+) (non-phosphorylating) activity" evidence=IDA] [GO:0010154 "fruit development" evidence=IMP] [GO:0048316 "seed development" evidence=IMP] [GO:0005507 "copper ion binding" evidence=IDA] [GO:0005794 "Golgi apparatus" evidence=RCA] [GO:0004365 "glyceraldehyde-3-phosphate dehydrogenase (NAD+) (phosphorylating) activity" evidence=TAS] [GO:0006950 "response to stress" evidence=IEP] [GO:0009408 "response to heat" evidence=IEP] [GO:0009744 "response to sucrose stimulus" evidence=IEP] InterPro:IPR006424 InterPro:IPR016040 InterPro:IPR020828 InterPro:IPR020829 InterPro:IPR020830 InterPro:IPR020831 Pfam:PF00044 Pfam:PF02800 PIRSF:PIRSF000149 PRINTS:PR00078 PROSITE:PS00071 SMART:SM00846 GO:GO:0005829 GO:GO:0005886 GO:GO:0048046 GO:GO:0005634 GO:GO:0046686 GO:GO:0005774 GO:GO:0009507 EMBL:CP002686 GenomeReviews:BA000014_GR Gene3D:G3DSA:3.40.50.720 GO:GO:0051287 GO:GO:0050661 GO:GO:0005507 GO:GO:0006094 GO:GO:0009651 GO:GO:0009408 GO:GO:0005740

Tissue	Identification	# of reads (stress)	# of reads (control)	Fold Change	p-value	Description
						GO:GO:0009744 GO:GO:0048316 GO:GO:0042542 GO:GO:0006096 EMBL:AC016829 eggNOG:COG0057 HOGENOM:HBG571736 PANTHER:PTHR10836 GO:GO:0004365 KO:K00134 TIGRFAMs:TIGR01534 OMA:CESTGVF EMBL:M64116 EMBL:M64119 EMBL:AY052267 EMBL:AY060521 EMBL:AY140084 EMBL:F20074 IPI:IPI00527494 PIR:JQ1287 RefSeq:NP_187062.1 UniGene:At.22963 UniGene:At.24406 UniGene:At.71328 ProteinModelPortal:P25858 SMR:P25858 STRING:P25858 PRIDE:P25858 EnsemblPlants:AT3G04120.1 GeneID:819567 KEGG:ath:AT3G04120 TAIR:At3g04120 InParanoid:P25858 PhylomeDB:P25858 ProtClustDB:PLN02358 ArrayExpress:P25858 Genevestigator:P25858 GermOnline:AT3G04120 GO:GO:0008886 Uniprot:P25858
Root	Isotig00856	42	124	-1.691	1.86E-12	TAIR|locus:2103085 - symbol:GAPC1 "AT3G04120" species:3702 "Arabidopsis thaliana" [GO:0006096 "glycolysis" evidence=ISS;IDA;TAS] [GO:0005739 "mitochondrion" evidence=IDA] [GO:0006979 "response to oxidative stress" evidence=IEP;IDA] [GO:0005740 "mitochondrial envelope" evidence=IDA] [GO:0005634 "nucleus" evidence=IDA] [GO:0005829 "cytosol" evidence=IDA;TAS] [GO:0006094 "gluconeogenesis" evidence=TAS] [GO:0042542 "response to hydrogen peroxide" evidence=IDA] [GO:0046686 "response to cadmium ion" evidence=IEP] [GO:0005774 "vacuolar membrane" evidence=IDA] [GO:0005886 "plasma membrane" evidence=IDA] [GO:0016020 "membrane" evidence=IDA] [GO:0009651 "response to salt stress" evidence=IEP] [GO:0009507 "chloroplast" evidence=IDA] [GO:0048046 "apoplast" evidence=IDA] [GO:0008886 "glyceraldehyde-3-phosphate dehydrogenase (NADP+) (non-phosphorylating) activity" evidence=IDA] [GO:0010154 "fruit development" evidence=IMP] [GO:0048316 "seed development" evidence=IMP] [GO:0005507 "copper ion binding" evidence=IDA] [GO:0005794 "Golgi apparatus" evidence=RCA] [GO:0004365 "glyceraldehyde-3-phosphate dehydrogenase (NAD+) (phosphorylating) activity" evidence=TAS] [GO:0006950 "response to stress" evidence=IEP] [GO:0009408 "response to heat" evidence=IEP] [GO:0009744 "response to sucrose stimulus" evidence=IEP] InterPro:IPR006424 InterPro:IPR016040 InterPro:IPR020828 InterPro:IPR020829 InterPro:IPR020830 InterPro:IPR020831 Pfam:PF00044 Pfam:PF02800 PIRSF:PIRSF000149 PRINTS:PR00078 PROSITE:PS00071 SMART:SM00846 GO:GO:0005829 GO:GO:0005886 GO:GO:0048046 GO:GO:0005634 GO:GO:0046686 GO:GO:0005774 GO:GO:0009507 EMBL:CP002686 GenomeReviews:BA000014_GR Gene3D:G3DSA:3.40.50.720 GO:GO:0051287 GO:GO:0050661 GO:GO:0005507 GO:GO:0006094 GO:GO:0009651 GO:GO:0009408 GO:GO:0005740 GO:GO:0009744 GO:GO:0048316 GO:GO:0042542 GO:GO:0006096 EMBL:AC016829 eggNOG:COG0057 HOGENOM:HBG571736 PANTHER:PTHR10836 GO:GO:0004365 KO:K00134 TIGRFAMs:TIGR01534 OMA:CESTGVF EMBL:M64116 EMBL:M64119 EMBL:AY052267 EMBL:AY060521 EMBL:AY140084 EMBL:F20074 IPI:IPI00527494 PIR:JQ1287 RefSeq:NP_187062.1 UniGene:At.22963 UniGene:At.24406 UniGene:At.71328 ProteinModelPortal:P25858 SMR:P25858 STRING:P25858 PRIDE:P25858 EnsemblPlants:AT3G04120.1 GeneID:819567 KEGG:ath:AT3G04120 TAIR:At3g04120 InParanoid:P25858 PhylomeDB:P25858 ProtClustDB:PLN02358 ArrayExpress:P25858 Genevestigator:P25858 GermOnline:AT3G04120 GO:GO:0008886 Uniprot:P25858
Root	Isotig00858	42	119	-1.631	1.73E-11	TAIR|locus:2103085 - symbol:GAPC1 "AT3G04120" species:3702 "Arabidopsis thaliana" [GO:0006096 "glycolysis" evidence=ISS;IDA;TAS] [GO:0005739 "mitochondrion" evidence=IDA] [GO:0006979 "response to oxidative stress" evidence=IEP;IDA] [GO:0005740 "mitochondrial envelope" evidence=IDA] [GO:0005634 "nucleus" evidence=IDA] [GO:0005829 "cytosol" evidence=IDA;TAS] [GO:0006094 "gluconeogenesis" evidence=TAS] [GO:0042542 "response to hydrogen peroxide" evidence=IDA] [GO:0046686 "response to cadmium ion" evidence=IEP] [GO:0005774 "vacuolar membrane" evidence=IDA] [GO:0005886 "plasma membrane" evidence=IDA] [GO:0016020 "membrane" evidence=IDA] [GO:0009651 "response to salt stress" evidence=IEP] [GO:0009507 "chloroplast" evidence=IDA] [GO:0048046 "apoplast" evidence=IDA] [GO:0008886 "glyceraldehyde-3-phosphate dehydrogenase (NADP+) (non-phosphorylating) activity" evidence=IDA] [GO:0010154 "fruit development" evidence=IMP] [GO:0048316 "seed development" evidence=IMP] [GO:0005507 "copper ion binding" evidence=IDA] [GO:0005794 "Golgi apparatus" evidence=RCA] [GO:0004365 "glyceraldehyde-3-phosphate dehydrogenase (NAD+) (phosphorylating) activity" evidence=TAS] [GO:0006950 "response to stress" evidence=IEP] [GO:0009408 "response to heat" evidence=IEP] [GO:0009744 "response to sucrose stimulus" evidence=IEP] InterPro:IPR006424 InterPro:IPR016040 InterPro:IPR020828 InterPro:IPR020829 InterPro:IPR020830 InterPro:IPR020831 Pfam:PF00044 Pfam:PF02800 PIRSF:PIRSF000149 PRINTS:PR00078 PROSITE:PS00071 SMART:SM00846 GO:GO:0005829 GO:GO:0005886 GO:GO:0048046 GO:GO:0005634 GO:GO:0046686 GO:GO:0005774 GO:GO:0009507 EMBL:CP002686 GenomeReviews:BA000014_GR Gene3D:G3DSA:3.40.50.720 GO:GO:0051287 GO:GO:0050661 GO:GO:0005507 GO:GO:0006094 GO:GO:0009651 GO:GO:0009408 GO:GO:0005740 GO:GO:0009744 GO:GO:0048316 GO:GO:0042542 GO:GO:0006096 EMBL:AC016829 eggNOG:COG0057

Tissue	Identification	# of reads (stress)	# of reads (control)	Fold Change	p-value	Description
						HOGENOM:HBG571736 PANTHER:PTHR10836 GO:GO:0004365 KO:K00134 TIGRFAMs:TIGR01534 OMA:CESTGVF EMBL:M64116 EMBL:M64119 EMBL:AY052267 EMBL:AY060521 EMBL:AY140084 EMBL:F20074 IPI:IPI00527494 PIR:JQ1287 RefSeq:NP_187062.1 UniGene:At.22963 UniGene:At.24406 UniGene:At.71328 ProteinModelPortal:P25858 SMR:P25858 STRING:P25858 PRIDE:P25858 EnsemblPlants:AT3G04120.1 GeneID:819567 KEGG:ath:AT3G04120 TAIR:At3g04120 InParanoid:P25858 PhylomeDB:P25858 ProtClustDB:PLN02358 ArrayExpress:P25858 Genevestigator:P25858 GermOnline:AT3G04120 GO:GO:0008886 Uniprot:P25858
Root	Isotig00859	38	100	-1.525	4.32E-09	TAIR|locus:2103085 - symbol:GAPC1 "AT3G04120" species:3702 "Arabidopsis thaliana" [GO:0006096 "glycolysis" evidence=ISS;IDA;TAS] [GO:0005739 "mitochondrion" evidence=IDA] [GO:0006979 "response to oxidative stress" evidence=IEP;IDA] [GO:0005740 "mitochondrial envelope" evidence=IDA] [GO:0005634 "nucleus" evidence=IDA] [GO:0005829 "cytosol" evidence=IDA;TAS] [GO:0006094 "gluconeogenesis" evidence=TAS] [GO:0042542 "response to hydrogen peroxide" evidence=IDA] [GO:0046686 "response to cadmium ion" evidence=IEP] [GO:0005774 "vacuolar membrane" evidence=IDA] [GO:0005886 "plasma membrane" evidence=IDA] [GO:0016020 "membrane" evidence=IDA] [GO:0009651 "response to salt stress" evidence=IEP] [GO:0009507 "chloroplast" evidence=IDA] [GO:0048046 "apoplast" evidence=IDA] [GO:0008886 "glyceraldehyde-3-phosphate dehydrogenase (NADP+) (non-phosphorylating) activity" evidence=IDA] [GO:0010154 "fruit development" evidence=IMP] [GO:0048316 "seed development" evidence=IMP] [GO:0005507 "copper ion binding" evidence=IDA] [GO:0005794 "Golgi apparatus" evidence=RCA] [GO:0004365 "glyceraldehyde-3-phosphate dehydrogenase (NAD+) (phosphorylating) activity" evidence=TAS] [GO:0006950 "response to stress" evidence=IEP] [GO:0009408 "response to heat" evidence=IEP] [GO:0009744 "response to sucrose stimulus" evidence=IEP] InterPro:IPR006424 InterPro:IPR016040 InterPro:IPR020828 InterPro:IPR020829 InterPro:IPR020830 InterPro:IPR020831 Pfam:PF00044 Pfam:PF02800 PIRSF:PIRSF000149 PRINTS:PR00078 PROSITE:PS00071 SMART:SM00846 GO:GO:0005829 GO:GO:0005886 GO:GO:0048046 GO:GO:0005634 GO:GO:0046686 GO:GO:0005774 GO:GO:0009507 EMBL:CP002686 GenomeReviews:BA000014_GR Gene3D:G3DSA:3.40.50.720 GO:GO:0051287 GO:GO:0050661 GO:GO:0005507 GO:GO:0006094 GO:GO:0009651 GO:GO:0009408 GO:GO:0005740 GO:GO:0009744 GO:GO:0048316 GO:GO:0042542 GO:GO:0006096 EMBL:AC016829 eggNOG:COG0057 HOGENOM:HBG571736 PANTHER:PTHR10836 GO:GO:0004365 KO:K00134 TIGRFAMs:TIGR01534 OMA:CESTGVF EMBL:M64116 EMBL:M64119 EMBL:AY052267 EMBL:AY060521 EMBL:AY140084 EMBL:F20074 IPI:IPI00527494 PIR:JQ1287 RefSeq:NP_187062.1 UniGene:At.22963 UniGene:At.24406 UniGene:At.71328 ProteinModelPortal:P25858 SMR:P25858 STRING:P25858 PRIDE:P25858 EnsemblPlants:AT3G04120.1 GeneID:819567 KEGG:ath:AT3G04120 TAIR:At3g04120 InParanoid:P25858 PhylomeDB:P25858 ProtClustDB:PLN02358 ArrayExpress:P25858 Genevestigator:P25858 GermOnline:AT3G04120 GO:GO:0008886 Uniprot:P25858
Root	Isotig00860	42	122	-1.667	4.57E-12	TAIR|locus:2103085 - symbol:GAPC1 "AT3G04120" species:3702 "Arabidopsis thaliana" [GO:0006096 "glycolysis" evidence=ISS;IDA;TAS] [GO:0005739 "mitochondrion" evidence=IDA] [GO:0006979 "response to oxidative stress" evidence=IEP;IDA] [GO:0005740 "mitochondrial envelope" evidence=IDA] [GO:0005634 "nucleus" evidence=IDA] [GO:0005829 "cytosol" evidence=IDA;TAS] [GO:0006094 "gluconeogenesis" evidence=TAS] [GO:0042542 "response to hydrogen peroxide" evidence=IDA] [GO:0046686 "response to cadmium ion" evidence=IEP] [GO:0005774 "vacuolar membrane" evidence=IDA] [GO:0005886 "plasma membrane" evidence=IDA] [GO:0016020 "membrane" evidence=IDA] [GO:0009651 "response to salt stress" evidence=IEP] [GO:0009507 "chloroplast" evidence=IDA] [GO:0048046 "apoplast" evidence=IDA] [GO:0008886 "glyceraldehyde-3-phosphate dehydrogenase (NADP+) (non-phosphorylating) activity" evidence=IDA] [GO:0010154 "fruit development" evidence=IMP] [GO:0048316 "seed development" evidence=IMP] [GO:0005507 "copper ion binding" evidence=IDA] [GO:0005794 "Golgi apparatus" evidence=RCA] [GO:0004365 "glyceraldehyde-3-phosphate dehydrogenase (NAD+) (phosphorylating) activity" evidence=TAS] [GO:0006950 "response to stress" evidence=IEP] [GO:0009408 "response to heat" evidence=IEP] [GO:0009744 "response to sucrose stimulus" evidence=IEP] InterPro:IPR006424 InterPro:IPR016040 InterPro:IPR020828 InterPro:IPR020829 InterPro:IPR020830 InterPro:IPR020831 Pfam:PF00044 Pfam:PF02800 PIRSF:PIRSF000149 PRINTS:PR00078 PROSITE:PS00071 SMART:SM00846 GO:GO:0005829 GO:GO:0005886 GO:GO:0048046 GO:GO:0005634 GO:GO:0046686 GO:GO:0005774 GO:GO:0009507 EMBL:CP002686 GenomeReviews:BA000014_GR Gene3D:G3DSA:3.40.50.720 GO:GO:0051287 GO:GO:0050661 GO:GO:0005507 GO:GO:0006094 GO:GO:0009651 GO:GO:0009408 GO:GO:0005740 GO:GO:0009744 GO:GO:0048316 GO:GO:0042542 GO:GO:0006096 EMBL:AC016829 eggNOG:COG0057 HOGENOM:HBG571736 PANTHER:PTHR10836 GO:GO:0004365 KO:K00134 TIGRFAMs:TIGR01534

Tissue	Identification	# of reads (stress)	# of reads (control)	Fold Change	p-value	Description
						OMA:CESTGVF EMBL:M64116 EMBL:M64119 EMBL:AY052267 EMBL:AY060521 EMBL:AY140084 EMBL:F20074 IPI:IPI00527494 PIR:JQ1287 RefSeq:NP_187062.1 UniGene:At.22963 UniGene:At.24406 UniGene:At.71328 ProteinModelPortal:P25858 SMR:P25858 STRING:P25858 PRIDE:P25858 EnsemblPlants:AT3G04120.1 GeneID:819567 KEGG:ath:AT3G04120 TAIR:At3g04120 InParanoid:P25858 PhylomeDB:P25858 ProtClustDB:PLN02358 ArrayExpress:P25858 Genevestigator:P25858 GermOnline:AT3G04120 GO:GO:0008886 Uniprot:P25858
Root	Isotig00879	9	67	-3.025	1.96E-13	TAIR|locus:2205677 - symbol:FRUCT5 "AT1G55120" species:3702 "Arabidopsis thaliana" [GO:0004553 "hydrolase activity, hydrolyzing O-glycosyl compounds" evidence=ISS] [GO:0005975 "carbohydrate metabolic process" evidence=IEA] [GO:0031219 "levanase activity" evidence=IDA] [GO:0005773 "vacuole" evidence=IDA] InterPro:IPR001362 InterPro:IPR018053 PROSITE:PS00609 SMART:SM00640 EMBL:CP002684 GenomeReviews:CT485782_GR GO:GO:0048046 GO:GO:0005773 GO:GO:0005618 InterPro:IPR008985 SUPFAM:SSF49899 InterPro:IPR023296 Gene3D:G3DSA:2.115.10.20 SUPFAM:SSF75005 EMBL:AC073944 CAZy:GH32 eggNOG:COG1621 HOGENOM:HBG317462 InterPro:IPR013189 InterPro:IPR013148 Pfam:PF08244 Pfam:PF00251 EMBL:AB029310 EMBL:AK176675 IPI:IPI00548648 PIR:G96592 RefSeq:NP_564676.1 UniGene:At.458 ProteinModelPortal:Q67XZ3 SMR:Q67XZ3 PRIDE:Q67XZ3 EnsemblPlants:AT1G55120.1 GeneID:841955 KEGG:ath:AT1G55120 TAIR:At1g55120 InParanoid:Q67XZ3 OMA:HSIVESY PhylomeDB:Q67XZ3 ProtClustDB:CLSN2688819 ArrayExpress:Q67XZ3 Genevestigator:Q67XZ3 GO:GO:0051669 Uniprot:Q67XZ3
Root	Isotig00880	13	64	-2.429	1.59E-10	TAIR|locus:2205677 - symbol:FRUCT5 "AT1G55120" species:3702 "Arabidopsis thaliana" [GO:0004553 "hydrolase activity, hydrolyzing O-glycosyl compounds" evidence=ISS] [GO:0005975 "carbohydrate metabolic process" evidence=IEA] [GO:0031219 "levanase activity" evidence=IDA] [GO:0005773 "vacuole" evidence=IDA] InterPro:IPR001362 InterPro:IPR018053 PROSITE:PS00609 SMART:SM00640 EMBL:CP002684 GenomeReviews:CT485782_GR GO:GO:0048046 GO:GO:0005773 GO:GO:0005618 InterPro:IPR008985 SUPFAM:SSF49899 InterPro:IPR023296 Gene3D:G3DSA:2.115.10.20 SUPFAM:SSF75005 EMBL:AC073944 CAZy:GH32 eggNOG:COG1621 HOGENOM:HBG317462 InterPro:IPR013189 InterPro:IPR013148 Pfam:PF08244 Pfam:PF00251 EMBL:AB029310 EMBL:AK176675 IPI:IPI00548648 PIR:G96592 RefSeq:NP_564676.1 UniGene:At.458 ProteinModelPortal:Q67XZ3 SMR:Q67XZ3 PRIDE:Q67XZ3 EnsemblPlants:AT1G55120.1 GeneID:841955 KEGG:ath:AT1G55120 TAIR:At1g55120 InParanoid:Q67XZ3 OMA:HSIVESY PhylomeDB:Q67XZ3 ProtClustDB:CLSN2688819 ArrayExpress:Q67XZ3 Genevestigator:Q67XZ3 GO:GO:0051669 Uniprot:Q67XZ3
Root	Isotig00881	8	67	-3.195	4.74E-14	TAIR|locus:2205677 - symbol:FRUCT5 "AT1G55120" species:3702 "Arabidopsis thaliana" [GO:0004553 "hydrolase activity, hydrolyzing O-glycosyl compounds" evidence=ISS] [GO:0005975 "carbohydrate metabolic process" evidence=IEA] [GO:0031219 "levanase activity" evidence=IDA] [GO:0005773 "vacuole" evidence=IDA] InterPro:IPR001362 InterPro:IPR018053 PROSITE:PS00609 SMART:SM00640 EMBL:CP002684 GenomeReviews:CT485782_GR GO:GO:0048046 GO:GO:0005773 GO:GO:0005618 InterPro:IPR008985 SUPFAM:SSF49899 InterPro:IPR023296 Gene3D:G3DSA:2.115.10.20 SUPFAM:SSF75005 EMBL:AC073944 CAZy:GH32 eggNOG:COG1621 HOGENOM:HBG317462 InterPro:IPR013189 InterPro:IPR013148 Pfam:PF08244 Pfam:PF00251 EMBL:AB029310 EMBL:AK176675 IPI:IPI00548648 PIR:G96592 RefSeq:NP_564676.1 UniGene:At.458 ProteinModelPortal:Q67XZ3 SMR:Q67XZ3 PRIDE:Q67XZ3 EnsemblPlants:AT1G55120.1 GeneID:841955 KEGG:ath:AT1G55120 TAIR:At1g55120 InParanoid:Q67XZ3 OMA:HSIVESY PhylomeDB:Q67XZ3 ProtClustDB:CLSN2688819 ArrayExpress:Q67XZ3 Genevestigator:Q67XZ3 GO:GO:0051669 Uniprot:Q67XZ3
Root	Isotig00882	12	64	-2.544	5.12E-11	TAIR|locus:2205677 - symbol:FRUCT5 "AT1G55120" species:3702 "Arabidopsis thaliana" [GO:0004553 "hydrolase activity, hydrolyzing O-glycosyl compounds" evidence=ISS] [GO:0005975 "carbohydrate metabolic process" evidence=IEA] [GO:0031219 "levanase activity" evidence=IDA] [GO:0005773 "vacuole" evidence=IDA] InterPro:IPR001362 InterPro:IPR018053 PROSITE:PS00609 SMART:SM00640 EMBL:CP002684 GenomeReviews:CT485782_GR GO:GO:0048046 GO:GO:0005773 GO:GO:0005618 InterPro:IPR008985 SUPFAM:SSF49899 InterPro:IPR023296 Gene3D:G3DSA:2.115.10.20 SUPFAM:SSF75005 EMBL:AC073944 CAZy:GH32 eggNOG:COG1621 HOGENOM:HBG317462 InterPro:IPR013189 InterPro:IPR013148 Pfam:PF08244 Pfam:PF00251 EMBL:AB029310 EMBL:AK176675 IPI:IPI00548648 PIR:G96592 RefSeq:NP_564676.1 UniGene:At.458 ProteinModelPortal:Q67XZ3 SMR:Q67XZ3 PRIDE:Q67XZ3 EnsemblPlants:AT1G55120.1 GeneID:841955 KEGG:ath:AT1G55120 TAIR:At1g55120 InParanoid:Q67XZ3 OMA:HSIVESY PhylomeDB:Q67XZ3 ProtClustDB:CLSN2688819 ArrayExpress:Q67XZ3 Genevestigator:Q67XZ3 GO:GO:0051669 Uniprot:Q67XZ3
Root	Isotig00903	4	50	-3.773	3.46E-12	TAIR|locus:2005527 - symbol:ELI3-1 "elicitor-activated gene 3-1" species:3702 "Arabidopsis thaliana" [GO:0008270 "zinc ion binding" evidence=IEA] [GO:0016491 "oxidoreductase activity" evidence=IEA] [GO:0009617 "response to

Tissue	Identification	# of reads (stress)	# of reads (control)	Fold Change	p-value	Description
						bacterium" evidence=IEP] [GO:0009626 "plant-type hypersensitive response" evidence=IGI] [GO:0045551 "cinnamyl-alcohol dehydrogenase activity" evidence=IDA] [GO:0009809 "lignin biosynthetic process" evidence=IEP] InterPro:IPR002085 InterPro:IPR002328 InterPro:IPR013149 InterPro:IPR013154 InterPro:IPR016040 Pfam:PF00107 Pfam:PF08240 PROSITE:PS00059 EMBL:CP002687 GenomeReviews:CT486007_GR GO:GO:0009617 EMBL:AL161592 GO:GO:0000166 Gene3D:G3DSA:3.40.50.720 GO:GO:0008270 eggNOG:COG1064 HOGENOM:HBG753318 InterPro:IPR011032 PANTHER:PTHR11695 SUPFAM:SSF50129 EMBL:AL035538 GO:GO:0009809 GO:GO:0045551 GO:GO:0052747 EMBL:Y16848 EMBL:X67816 EMBL:AY302079 EMBL:AF360225 EMBL:AY040066 EMBL:AY050407 EMBL:AY050931 EMBL:AY056385 EMBL:BT002729 EMBL:AK317050 IPI:IPI00545521 IPI:IPI00657154 PIR:T05625 RefSeq:NP_001031805.1 RefSeq:NP_195511.1 UniGene:At.24464 ProteinModelPortal:Q02971 SMR:Q02971 PRIDE:Q02971 EnsemblPlants:AT4G37980.1 GeneID:829954 KEGG:ath:AT4G37980 TAIR:At4g37980 InParanoid:Q02971 OMA:WSSLAST PhylomeDB:Q02971 ProtClustDB:CLSN2685373 ArrayExpress:Q02971 Genevestigator:Q02971 Uniprot:Q02971
Root	Isotig00904	4	50	-3.773	3.46E-12	TAIR|locus:2005527 - symbol:ELI3-1 "elicitor-activated gene 3-1" species:3702 "Arabidopsis thaliana" [GO:0008270 "zinc ion binding" evidence=IEA] [GO:0016491 "oxidoreductase activity" evidence=IEA] [GO:0009617 "response to bacterium" evidence=IEP] [GO:0009626 "plant-type hypersensitive response" evidence=IGI] [GO:0045551 "cinnamyl-alcohol dehydrogenase activity" evidence=IDA] [GO:0009809 "lignin biosynthetic process" evidence=IEP] InterPro:IPR002085 InterPro:IPR002328 InterPro:IPR013149 InterPro:IPR013154 InterPro:IPR016040 Pfam:PF00107 Pfam:PF08240 PROSITE:PS00059 EMBL:CP002687 GenomeReviews:CT486007_GR GO:GO:0009617 EMBL:AL161592 GO:GO:0000166 Gene3D:G3DSA:3.40.50.720 GO:GO:0008270 eggNOG:COG1064 HOGENOM:HBG753318 InterPro:IPR011032 PANTHER:PTHR11695 SUPFAM:SSF50129 EMBL:AL035538 GO:GO:0009809 GO:GO:0045551 GO:GO:0052747 EMBL:Y16848 EMBL:X67816 EMBL:AY302079 EMBL:AF360225 EMBL:AY040066 EMBL:AY050407 EMBL:AY050931 EMBL:AY056385 EMBL:BT002729 EMBL:AK317050 IPI:IPI00545521 IPI:IPI00657154 PIR:T05625 RefSeq:NP_001031805.1 RefSeq:NP_195511.1 UniGene:At.24464 ProteinModelPortal:Q02971 SMR:Q02971 PRIDE:Q02971 EnsemblPlants:AT4G37980.1 GeneID:829954 KEGG:ath:AT4G37980 TAIR:At4g37980 InParanoid:Q02971 OMA:WSSLAST PhylomeDB:Q02971 ProtClustDB:CLSN2685373 ArrayExpress:Q02971 Genevestigator:Q02971 Uniprot:Q02971
Root	Isotig00905	2	34	-4.216	2.89E-09	TAIR|locus:2005527 - symbol:ELI3-1 "elicitor-activated gene 3-1" species:3702 "Arabidopsis thaliana" [GO:0008270 "zinc ion binding" evidence=IEA] [GO:0016491 "oxidoreductase activity" evidence=IEA] [GO:0009617 "response to bacterium" evidence=IEP] [GO:0009626 "plant-type hypersensitive response" evidence=IGI] [GO:0045551 "cinnamyl-alcohol dehydrogenase activity" evidence=IDA] [GO:0009809 "lignin biosynthetic process" evidence=IEP] InterPro:IPR002085 InterPro:IPR002328 InterPro:IPR013149 InterPro:IPR013154 InterPro:IPR016040 Pfam:PF00107 Pfam:PF08240 PROSITE:PS00059 EMBL:CP002687 GenomeReviews:CT486007_GR GO:GO:0009617 EMBL:AL161592 GO:GO:0000166 Gene3D:G3DSA:3.40.50.720 GO:GO:0008270 eggNOG:COG1064 HOGENOM:HBG753318 InterPro:IPR011032 PANTHER:PTHR11695 SUPFAM:SSF50129 EMBL:AL035538 GO:GO:0009809 GO:GO:0045551 GO:GO:0052747 EMBL:Y16848 EMBL:X67816 EMBL:AY302079 EMBL:AF360225 EMBL:AY040066 EMBL:AY050407 EMBL:AY050931 EMBL:AY056385 EMBL:BT002729 EMBL:AK317050 IPI:IPI00545521 IPI:IPI00657154 PIR:T05625 RefSeq:NP_001031805.1 RefSeq:NP_195511.1 UniGene:At.24464 ProteinModelPortal:Q02971 SMR:Q02971 PRIDE:Q02971 EnsemblPlants:AT4G37980.1 GeneID:829954 KEGG:ath:AT4G37980 TAIR:At4g37980 InParanoid:Q02971 OMA:WSSLAST PhylomeDB:Q02971 ProtClustDB:CLSN2685373 ArrayExpress:Q02971 Genevestigator:Q02971 Uniprot:Q02971
Root	Isotig00907	2	34	-4.216	2.89E-09	TAIR|locus:2005527 - symbol:ELI3-1 "elicitor-activated gene 3-1" species:3702 "Arabidopsis thaliana" [GO:0008270 "zinc ion binding" evidence=IEA] [GO:0016491 "oxidoreductase activity" evidence=IEA] [GO:0009617 "response to bacterium" evidence=IEP] [GO:0009626 "plant-type hypersensitive response" evidence=IGI] [GO:0045551 "cinnamyl-alcohol dehydrogenase activity" evidence=IDA] [GO:0009809 "lignin biosynthetic process" evidence=IEP] InterPro:IPR002085 InterPro:IPR002328 InterPro:IPR013149 InterPro:IPR013154 InterPro:IPR016040 Pfam:PF00107 Pfam:PF08240 PROSITE:PS00059 EMBL:CP002687 GenomeReviews:CT486007_GR GO:GO:0009617 EMBL:AL161592 GO:GO:0000166 Gene3D:G3DSA:3.40.50.720 GO:GO:0008270 eggNOG:COG1064 HOGENOM:HBG753318 InterPro:IPR011032 PANTHER:PTHR11695 SUPFAM:SSF50129 EMBL:AL035538 GO:GO:0009809 GO:GO:0045551 GO:GO:0052747 EMBL:Y16848 EMBL:X67816

Tissue	Identification	# of reads (stress)	# of reads (control)	Fold Change	p-value	Description
						EMBL:AY302079 EMBL:AF360225 EMBL:AY040066 EMBL:AY050407 EMBL:AY050931 EMBL:AY056385 EMBL:BT002729 EMBL:AK317050 IPI:IPI00545521 IPI:IPI00657154 PIR:T05625 RefSeq:NP_001031805.1 RefSeq:NP_195511.1 UniGene:At.24464 ProteinModelPortal:Q02971 SMR:Q02971 PRIDE:Q02971 EnsemblPlants:AT4G37980.1 GeneID:829954 KEGG:ath:AT4G37980 TAIR:At4g37980 InParanoid:Q02971 OMA:WSSLAST PhylomeDB:Q02971 ProtClustDB:CLSN2685373 ArrayExpress:Q02971 Genevestigator:Q02971 Uniprot:Q02971
Root	Isotig00909	46	8	2.395	2.70E-07	TAIR|locus:2138121 - symbol:UGE2 "AT4G23920" species:3702 "Arabidopsis thaliana" [GO:0003978 "UDP-glucose 4-epimerase activity" evidence=IGI;IDA] [GO:0005829 "cytosol" evidence=IDA] [GO:0046983 "protein dimerization activity" evidence=IPI] [GO:0042546 "cell wall biogenesis" evidence=IMP] InterPro:IPR001509 InterPro:IPR005886 InterPro:IPR016040 Pfam:PF01370 GO:GO:0005829 EMBL:CP002687 GenomeReviews:CT486007_GR GO:GO:0000166 Gene3D:G3DSA:3.40.50.720 HOGENOM:HBG755066 GO:GO:0046983 GO:GO:0050662 eggNOG:COG1087 GO:GO:0003978 GO:GO:0006012 InterPro:IPR025308 PANTHER:PTHR10366:SF39 Pfam:PF13950 TIGRFAMs:TIGR01179 EMBL:AL078468 EMBL:AL161560 KO:K01784 GO:GO:0042546 ProtClustDB:PLN02240 EMBL:BT008539 IPI:IPI00516246 PIR:T08911 RefSeq:NP_194123.1 UniGene:At.3390 ProteinModelPortal:Q9T0A7 SMR:Q9T0A7 IntAct:Q9T0A7 STRING:Q9T0A7 PRIDE:Q9T0A7 EnsemblPlants:AT4G23920.1 GeneID:828492 KEGG:ath:AT4G23920 TAIR:At4g23920 InParanoid:Q9T0A7 OMA:SAINPYG PhylomeDB:Q9T0A7 ArrayExpress:Q9T0A7 Genevestigator:Q9T0A7 GermOnline:AT4G23920 Uniprot:Q9T0A7
Root	Isotig00911	31	3	3.240	8.04E-07	TAIR|locus:2138121 - symbol:UGE2 "AT4G23920" species:3702 "Arabidopsis thaliana" [GO:0003978 "UDP-glucose 4-epimerase activity" evidence=IGI;IDA] [GO:0005829 "cytosol" evidence=IDA] [GO:0046983 "protein dimerization activity" evidence=IPI] [GO:0042546 "cell wall biogenesis" evidence=IMP] InterPro:IPR001509 InterPro:IPR005886 InterPro:IPR016040 Pfam:PF01370 GO:GO:0005829 EMBL:CP002687 GenomeReviews:CT486007_GR GO:GO:0000166 Gene3D:G3DSA:3.40.50.720 HOGENOM:HBG755066 GO:GO:0046983 GO:GO:0050662 eggNOG:COG1087 GO:GO:0003978 GO:GO:0006012 InterPro:IPR025308 PANTHER:PTHR10366:SF39 Pfam:PF13950 TIGRFAMs:TIGR01179 EMBL:AL078468 EMBL:AL161560 KO:K01784 GO:GO:0042546 ProtClustDB:PLN02240 EMBL:BT008539 IPI:IPI00516246 PIR:T08911 RefSeq:NP_194123.1 UniGene:At.3390 ProteinModelPortal:Q9T0A7 SMR:Q9T0A7 IntAct:Q9T0A7 STRING:Q9T0A7 PRIDE:Q9T0A7 EnsemblPlants:AT4G23920.1 GeneID:828492 KEGG:ath:AT4G23920 TAIR:At4g23920 InParanoid:Q9T0A7 OMA:SAINPYG PhylomeDB:Q9T0A7 ArrayExpress:Q9T0A7 Genevestigator:Q9T0A7 GermOnline:AT4G23920 Uniprot:Q9T0A7
Root	Isotig00915	58	95	-0.841	0.000381099	TAIR|locus:2129256 - symbol:HOG1 "AT4G13940" species:3702 "Arabidopsis thaliana" [GO:0004013 "adenosylhomocysteinase activity" evidence=ISS;IMP] [GO:0006730 "one-carbon metabolic process" evidence=ISS] [GO:0009793 "embryo development ending in seed dormancy" evidence=IMP;NAS] [GO:0005773 "vacuole" evidence=IDA] [GO:0006346 "methylation-dependent chromatin silencing" evidence=IMP] [GO:0005507 "copper ion binding" evidence=IDA] [GO:0005774 "vacuolar membrane" evidence=IDA] [GO:0016020 "membrane" evidence=IDA] [GO:0005886 "plasma membrane" evidence=IDA] [GO:0005829 "cytosol" evidence=IDA] [GO:0009506 "plasmodesma" evidence=IDA] [GO:0005794 "Golgi apparatus" evidence=RCA] [GO:0016441 "posttranscriptional gene silencing" evidence=IMP] InterPro:IPR000043 InterPro:IPR020082 Pfam:PF05221 PIRSF:PIRSF001109 PROSITE:PS00738 PROSITE:PS00739 SMART:SM00996 InterPro:IPR015878 Pfam:PF00670 GO:GO:0005829 GO:GO:0005886 GO:GO:0009506 EMBL:CP002687 GenomeReviews:CT486007_GR GO:GO:0005774 EMBL:Z97335 GO:GO:0005507 GO:GO:0009793 GO:GO:0006730 EMBL:AL161537 GO:GO:0006346 GO:GO:0016441 EMBL:AF059581 EMBL:AF325037 EMBL:AY042866 EMBL:AY049279 EMBL:AY081468 EMBL:AY090284 EMBL:BT002404 EMBL:AY085669 EMBL:Z97059 IPI:IPI00527713 PIR:C71400 RefSeq:NP_193130.1 UniGene:At.24056 UniGene:At.24845 UniGene:At.71956 UniGene:At.74987 UniGene:At.74988 ProteinModelPortal:O23255 SMR:O23255 IntAct:O23255 STRING:O23255 PRIDE:O23255 ProMEX:O23255 EnsemblPlants:AT4G13940.1 GeneID:827028 KEGG:ath:AT4G13940 TAIR:At4g13940 eggNOG:COG0499 HOGENOM:HBG352029 InParanoid:O23255 KO:K01251 OMA:ELFTKTE PhylomeDB:O23255 ProtClustDB:PLN02494 ArrayExpress:O23255 Genevestigator:O23255 GO:GO:0004013 PANTHER:PTHR23420 SMART:SM00997 TIGRFAMs:TIGR00936 Uniprot:O23255
Root	Isotig00916	68	111	-0.836	0.000132787	TAIR|locus:2129256 - symbol:HOG1 "AT4G13940" species:3702 "Arabidopsis thaliana" [GO:0004013 "adenosylhomocysteinase activity" evidence=ISS;IMP] [GO:0006730 "one-carbon metabolic process" evidence=ISS]

Tissue	Identification	# of reads (stress)	# of reads (control)	Fold Change	p-value	Description
						[GO:0009793 "embryo development ending in seed dormancy" evidence=IMP;NAS] [GO:0005773 "vacuole" evidence=IDA] [GO:0006346 "methylation-dependent chromatin silencing" evidence=IMP] [GO:0005507 "copper ion binding" evidence=IDA] [GO:0005774 "vacuolar membrane" evidence=IDA] [GO:0016020 "membrane" evidence=IDA] [GO:0005886 "plasma membrane" evidence=IDA] [GO:0005829 "cytosol" evidence=IDA] [GO:0009506 "plasmodesma" evidence=IDA] [GO:0005794 "Golgi apparatus" evidence=RCA] [GO:0016441 "posttranscriptional gene silencing" evidence=IMP] InterPro:IPR000043 InterPro:IPR020082 Pfam:PF05221 PIRSF:PIRSF001109 PROSITE:PS00738 PROSITE:PS00739 SMART:SM00996 InterPro:IPR015878 Pfam:PF00670 GO:GO:0005829 GO:GO:0005886 GO:GO:0009506 EMBL:CP002687 GenomeReviews:CT486007_GR GO:GO:0005774 EMBL:Z97335 GO:GO:0005507 GO:GO:0009793 GO:GO:0006730 EMBL:AL161537 GO:GO:0006346 GO:GO:0016441 EMBL:AF059581 EMBL:AF325037 EMBL:AY042866 EMBL:AY049279 EMBL:AY081468 EMBL:AY090284 EMBL:BT002404 EMBL:AY085669 EMBL:Z97059 IPI:IPI00527713 PIR:C71400 RefSeq:NP_193130.1 UniGene:At.24056 UniGene:At.24845 UniGene:At.71956 UniGene:At.74987 UniGene:At.74988 ProteinModelPortal:O23255 SMR:O23255 IntAct:O23255 STRING:O23255 PRIDE:O23255 ProMEX:O23255 EnsemblPlants:AT4G13940.1 GeneID:827028 KEGG:ath:AT4G13940 TAIR:At4g13940 eggNOG:COG0499 HOGENOM:HBG352029 InParanoid:O23255 KO:K01251 OMA:ELFTKTE PhylomeDB:O23255 ProtClustDB:PLN02494 ArrayExpress:O23255 Genevestigator:O23255 GO:GO:0004013 PANTHER:PTHR23420 SMART:SM00997 TIGRFAMs:TIGR00936 Uniprot:O23255
Root	Isotig00917	61	110	-0.980	1.37E-05	TAIR|locus:2129256 - symbol:HOG1 "AT4G13940" species:3702 "Arabidopsis thaliana" [GO:0004013 "adenosylhomocysteinase activity" evidence=ISS;IMP] [GO:0006730 "one-carbon metabolic process" evidence=ISS] [GO:0009793 "embryo development ending in seed dormancy" evidence=IMP;NAS] [GO:0005773 "vacuole" evidence=IDA] [GO:0006346 "methylation-dependent chromatin silencing" evidence=IMP] [GO:0005507 "copper ion binding" evidence=IDA] [GO:0005774 "vacuolar membrane" evidence=IDA] [GO:0016020 "membrane" evidence=IDA] [GO:0005886 "plasma membrane" evidence=IDA] [GO:0005829 "cytosol" evidence=IDA] [GO:0009506 "plasmodesma" evidence=IDA] [GO:0005794 "Golgi apparatus" evidence=RCA] [GO:0016441 "posttranscriptional gene silencing" evidence=IMP] InterPro:IPR000043 InterPro:IPR020082 Pfam:PF05221 PIRSF:PIRSF001109 PROSITE:PS00738 PROSITE:PS00739 SMART:SM00996 InterPro:IPR015878 Pfam:PF00670 GO:GO:0005829 GO:GO:0005886 GO:GO:0009506 EMBL:CP002687 GenomeReviews:CT486007_GR GO:GO:0005774 EMBL:Z97335 GO:GO:0005507 GO:GO:0009793 GO:GO:0006730 EMBL:AL161537 GO:GO:0006346 GO:GO:0016441 EMBL:AF059581 EMBL:AF325037 EMBL:AY042866 EMBL:AY049279 EMBL:AY081468 EMBL:AY090284 EMBL:BT002404 EMBL:AY085669 EMBL:Z97059 IPI:IPI00527713 PIR:C71400 RefSeq:NP_193130.1 UniGene:At.24056 UniGene:At.24845 UniGene:At.71956 UniGene:At.74987 UniGene:At.74988 ProteinModelPortal:O23255 SMR:O23255 IntAct:O23255 STRING:O23255 PRIDE:O23255 ProMEX:O23255 EnsemblPlants:AT4G13940.1 GeneID:827028 KEGG:ath:AT4G13940 TAIR:At4g13940 eggNOG:COG0499 HOGENOM:HBG352029 InParanoid:O23255 KO:K01251 OMA:ELFTKTE PhylomeDB:O23255 ProtClustDB:PLN02494 ArrayExpress:O23255 Genevestigator:O23255 GO:GO:0004013 PANTHER:PTHR23420 SMART:SM00997 TIGRFAMs:TIGR00936 Uniprot:O23255
Root	Isotig00919	53	87	-0.844	0.000647735	TAIR|locus:2129256 - symbol:HOG1 "AT4G13940" species:3702 "Arabidopsis thaliana" [GO:0004013 "adenosylhomocysteinase activity" evidence=ISS;IMP] [GO:0006730 "one-carbon metabolic process" evidence=ISS] [GO:0009793 "embryo development ending in seed dormancy" evidence=IMP;NAS] [GO:0005773 "vacuole" evidence=IDA] [GO:0006346 "methylation-dependent chromatin silencing" evidence=IMP] [GO:0005507 "copper ion binding" evidence=IDA] [GO:0005774 "vacuolar membrane" evidence=IDA] [GO:0016020 "membrane" evidence=IDA] [GO:0005886 "plasma membrane" evidence=IDA] [GO:0005829 "cytosol" evidence=IDA] [GO:0009506 "plasmodesma" evidence=IDA] [GO:0005794 "Golgi apparatus" evidence=RCA] [GO:0016441 "posttranscriptional gene silencing" evidence=IMP] InterPro:IPR000043 InterPro:IPR020082 Pfam:PF05221 PIRSF:PIRSF001109 PROSITE:PS00738 PROSITE:PS00739 SMART:SM00996 InterPro:IPR015878 Pfam:PF00670 GO:GO:0005829 GO:GO:0005886 GO:GO:0009506 EMBL:CP002687 GenomeReviews:CT486007_GR GO:GO:0005774 EMBL:Z97335 GO:GO:0005507 GO:GO:0009793 GO:GO:0006730 EMBL:AL161537 GO:GO:0006346 GO:GO:0016441 EMBL:AF059581 EMBL:AF325037 EMBL:AY042866 EMBL:AY049279 EMBL:AY081468 EMBL:AY090284 EMBL:BT002404 EMBL:AY085669 EMBL:Z97059 IPI:IPI00527713 PIR:C71400 RefSeq:NP_193130.1 UniGene:At.24056 UniGene:At.24845 UniGene:At.71956 UniGene:At.74987 UniGene:At.74988 ProteinModelPortal:O23255 SMR:O23255 IntAct:O23255

Tissue	Identification	# of reads (stress)	# of reads (control)	Fold Change	p-value	Description
						STRING:O23255 PRIDE:O23255 ProMEX:O23255 EnsemblPlants:AT4G13940.1 GeneID:827028 KEGG:ath:AT4G13940 TAIR:At4g13940 eggNOG:COG0499 HOGENOM:HBG352029 InParanoid:O23255 KO:K01251 OMA:ELFTKTE PhylomeDB:O23255 ProtClustDB:PLN02494 ArrayExpress:O23255 Genevestigator:O23255 GO:GO:0004013 PANTHER:PTHR23420 SMART:SM00997 TIGRFAMs:TIGR00936 Uniprot:O23255
Root	Isotig00927	26	62	-1.383	1.73E-05	TAIR|locus:2054027 - symbol:TPS11 "trehalose phosphatase/synthase 11" species:3702 "Arabidopsis thaliana" [GO:0005992 "trehalose biosynthetic process" evidence=IEA] [GO:0008152 "metabolic process" evidence=IEA] [GO:0016757 "transferase activity, transferring glycosyl groups" evidence=ISS] [GO:0005739 "mitochondrion" evidence=IDA] [GO:0003825 "alpha,alpha-trehalose-phosphate synthase (UDP-forming) activity" evidence=IMP] [GO:0004805 "trehalose-phosphatase activity" evidence=IMP] [GO:0005829 "cytosol" evidence=IDA] InterPro:IPR001830 InterPro:IPR003337 InterPro:IPR006379 Pfam:PF00982 Pfam:PF02358 GO:GO:0005829 GO:GO:0005739 EMBL:CP002685 GenomeReviews:CT485783_GR InterPro:IPR023214 Gene3D:G3DSA:3.40.50.1000 SUPFAM:SSF56784 EMBL:AC005724 GO:GO:0005992 TIGRFAMs:TIGR01484 CAZy:GT20 eggNOG:COG0380 HOGENOM:HBG559076 GO:GO:0003825 HSSP:P31677 TIGRFAMs:TIGR00685 EMBL:AV822913 EMBL:AY042865 EMBL:AY081537 IPI:IPI00538425 IPI:IPI00889354 PIR:E84567 RefSeq:NP_179460.1 UniGene:At.25317 ProteinModelPortal:Q9ZV48 STRING:Q9ZV48 PRIDE:Q9ZV48 EnsemblPlants:AT2G18700.1 GeneID:816385 KEGG:ath:AT2G18700 TAIR:At2g18700 InParanoid:Q9ZV48 OMA:LQRACKD PhylomeDB:Q9ZV48 ProtClustDB:CLSN2912887 Genevestigator:Q9ZV48 Uniprot:Q9ZV48
Root	Isotig00930	25	60	-1.392	2.15E-05	TAIR|locus:2054027 - symbol:TPS11 "trehalose phosphatase/synthase 11" species:3702 "Arabidopsis thaliana" [GO:0005992 "trehalose biosynthetic process" evidence=IEA] [GO:0008152 "metabolic process" evidence=IEA] [GO:0016757 "transferase activity, transferring glycosyl groups" evidence=ISS] [GO:0005739 "mitochondrion" evidence=IDA] [GO:0003825 "alpha,alpha-trehalose-phosphate synthase (UDP-forming) activity" evidence=IMP] [GO:0004805 "trehalose-phosphatase activity" evidence=IMP] [GO:0005829 "cytosol" evidence=IDA] InterPro:IPR001830 InterPro:IPR003337 InterPro:IPR006379 Pfam:PF00982 Pfam:PF02358 GO:GO:0005829 GO:GO:0005739 EMBL:CP002685 GenomeReviews:CT485783_GR InterPro:IPR023214 Gene3D:G3DSA:3.40.50.1000 SUPFAM:SSF56784 EMBL:AC005724 GO:GO:0005992 TIGRFAMs:TIGR01484 CAZy:GT20 eggNOG:COG0380 HOGENOM:HBG559076 GO:GO:0003825 HSSP:P31677 TIGRFAMs:TIGR00685 EMBL:AV822913 EMBL:AY042865 EMBL:AY081537 IPI:IPI00538425 IPI:IPI00889354 PIR:E84567 RefSeq:NP_179460.1 UniGene:At.25317 ProteinModelPortal:Q9ZV48 STRING:Q9ZV48 PRIDE:Q9ZV48 EnsemblPlants:AT2G18700.1 GeneID:816385 KEGG:ath:AT2G18700 TAIR:At2g18700 InParanoid:Q9ZV48 OMA:LQRACKD PhylomeDB:Q9ZV48 ProtClustDB:CLSN2912887 Genevestigator:Q9ZV48 Uniprot:Q9ZV48
Root	Isotig00931	26	62	-1.383	1.73E-05	TAIR|locus:2054027 - symbol:TPS11 "trehalose phosphatase/synthase 11" species:3702 "Arabidopsis thaliana" [GO:0005992 "trehalose biosynthetic process" evidence=IEA] [GO:0008152 "metabolic process" evidence=IEA] [GO:0016757 "transferase activity, transferring glycosyl groups" evidence=ISS] [GO:0005739 "mitochondrion" evidence=IDA] [GO:0003825 "alpha,alpha-trehalose-phosphate synthase (UDP-forming) activity" evidence=IMP] [GO:0004805 "trehalose-phosphatase activity" evidence=IMP] [GO:0005829 "cytosol" evidence=IDA] InterPro:IPR001830 InterPro:IPR003337 InterPro:IPR006379 Pfam:PF00982 Pfam:PF02358 GO:GO:0005829 GO:GO:0005739 EMBL:CP002685 GenomeReviews:CT485783_GR InterPro:IPR023214 Gene3D:G3DSA:3.40.50.1000 SUPFAM:SSF56784 EMBL:AC005724 GO:GO:0005992 TIGRFAMs:TIGR01484 CAZy:GT20 eggNOG:COG0380 HOGENOM:HBG559076 GO:GO:0003825 HSSP:P31677 TIGRFAMs:TIGR00685 EMBL:AV822913 EMBL:AY042865 EMBL:AY081537 IPI:IPI00538425 IPI:IPI00889354 PIR:E84567 RefSeq:NP_179460.1 UniGene:At.25317 ProteinModelPortal:Q9ZV48 STRING:Q9ZV48 PRIDE:Q9ZV48 EnsemblPlants:AT2G18700.1 GeneID:816385 KEGG:ath:AT2G18700 TAIR:At2g18700 InParanoid:Q9ZV48 OMA:LQRACKD PhylomeDB:Q9ZV48 ProtClustDB:CLSN2912887 Genevestigator:Q9ZV48 Uniprot:Q9ZV48
Root	Isotig00932	17	52	-1.742	3.19E-06	TAIR|locus:2054027 - symbol:TPS11 "trehalose phosphatase/synthase 11" species:3702 "Arabidopsis thaliana" [GO:0005992 "trehalose biosynthetic process" evidence=IEA] [GO:0008152 "metabolic process" evidence=IEA] [GO:0016757 "transferase activity, transferring glycosyl groups" evidence=ISS] [GO:0005739 "mitochondrion" evidence=IDA] [GO:0003825 "alpha,alpha-trehalose-phosphate synthase (UDP-forming) activity" evidence=IMP] [GO:0004805 "trehalose-phosphatase activity" evidence=IMP] [GO:0005829 "cytosol" evidence=IDA] InterPro:IPR001830 InterPro:IPR003337 InterPro:IPR006379 Pfam:PF00982 Pfam:PF02358 GO:GO:0005829 GO:GO:0005739 EMBL:CP002685 GenomeReviews:CT485783_GR InterPro:IPR023214 Gene3D:G3DSA:3.40.50.1000 SUPFAM:SSF56784 EMBL:AC005724 GO:GO:0005992 TIGRFAMs:TIGR01484

Tissue	Identification	# of reads (stress)	# of reads (control)	Fold Change	p-value	Description
						CAZy:GT20 eggNOG:COG0380 HOGENOM:HBG559076 GO:GO:0003825 HSSP:P31677 TIGRFAMs:TIGR00685 EMBL:AV822913 EMBL:AY042865 EMBL:AY081537 IPI:IPI00538425 IPI:IPI00889354 PIR:E84567 RefSeq:NP_179460.1 UniGene:At.25317 ProteinModelPortal:Q9ZV48 STRING:Q9ZV48 PRIDE:Q9ZV48 EnsemblPlants:AT2G18700.1 GeneID:816385 KEGG:ath:AT2G18700 TAIR:At2g18700 InParanoid:Q9ZV48 OMA:LQRACKD PhylomeDB:Q9ZV48 ProtClustDB:CLSN2912887 Genevestigator:Q9ZV48 Uniprot:Q9ZV48
Root	Isotig00933	17	42	-1.434	0.000278343	TAIR|locus:2054027 - symbol:TPS11 "trehalose phosphatase/synthase 11" species:3702 "Arabidopsis thaliana" [GO:0005992 "trehalose biosynthetic process" evidence=IEA] [GO:0008152 "metabolic process" evidence=IEA] [GO:0016757 "transferase activity, transferring glycosyl groups" evidence=ISS] [GO:0005739 "mitochondrion" evidence=IDA] [GO:0003825 "alpha,alpha-trehalose-phosphate synthase (UDP-forming) activity" evidence=IMP] [GO:0004805 "trehalose-phosphatase activity" evidence=IMP] [GO:0005829 "cytosol" evidence=IDA] InterPro:IPR001830 InterPro:IPR003337 InterPro:IPR006379 Pfam:PF00982 Pfam:PF02358 GO:GO:0005829 GO:GO:0005739 EMBL:CP002685 GenomeReviews:CT485783_GR InterPro:IPR023214 Gene3D:G3DSA:3.40.50.1000 SUPFAM:SSF56784 EMBL:AC005724 GO:GO:0005992 TIGRFAMs:TIGR01484 CAZy:GT20 eggNOG:COG0380 HOGENOM:HBG559076 GO:GO:0003825 HSSP:P31677 TIGRFAMs:TIGR00685 EMBL:AV822913 EMBL:AY042865 EMBL:AY081537 IPI:IPI00538425 IPI:IPI00889354 PIR:E84567 RefSeq:NP_179460.1 UniGene:At.25317 ProteinModelPortal:Q9ZV48 STRING:Q9ZV48 PRIDE:Q9ZV48 EnsemblPlants:AT2G18700.1 GeneID:816385 KEGG:ath:AT2G18700 TAIR:At2g18700 InParanoid:Q9ZV48 OMA:LQRACKD PhylomeDB:Q9ZV48 ProtClustDB:CLSN2912887 Genevestigator:Q9ZV48 Uniprot:Q9ZV48
Root	Isotig00934	25	60	-1.392	2.15E-05	TAIR|locus:2054027 - symbol:TPS11 "trehalose phosphatase/synthase 11" species:3702 "Arabidopsis thaliana" [GO:0005992 "trehalose biosynthetic process" evidence=IEA] [GO:0008152 "metabolic process" evidence=IEA] [GO:0016757 "transferase activity, transferring glycosyl groups" evidence=ISS] [GO:0005739 "mitochondrion" evidence=IDA] [GO:0003825 "alpha,alpha-trehalose-phosphate synthase (UDP-forming) activity" evidence=IMP] [GO:0004805 "trehalose-phosphatase activity" evidence=IMP] [GO:0005829 "cytosol" evidence=IDA] InterPro:IPR001830 InterPro:IPR003337 InterPro:IPR006379 Pfam:PF00982 Pfam:PF02358 GO:GO:0005829 GO:GO:0005739 EMBL:CP002685 GenomeReviews:CT485783_GR InterPro:IPR023214 Gene3D:G3DSA:3.40.50.1000 SUPFAM:SSF56784 EMBL:AC005724 GO:GO:0005992 TIGRFAMs:TIGR01484 CAZy:GT20 eggNOG:COG0380 HOGENOM:HBG559076 GO:GO:0003825 HSSP:P31677 TIGRFAMs:TIGR00685 EMBL:AV822913 EMBL:AY042865 EMBL:AY081537 IPI:IPI00538425 IPI:IPI00889354 PIR:E84567 RefSeq:NP_179460.1 UniGene:At.25317 ProteinModelPortal:Q9ZV48 STRING:Q9ZV48 PRIDE:Q9ZV48 EnsemblPlants:AT2G18700.1 GeneID:816385 KEGG:ath:AT2G18700 TAIR:At2g18700 InParanoid:Q9ZV48 OMA:LQRACKD PhylomeDB:Q9ZV48 ProtClustDB:CLSN2912887 Genevestigator:Q9ZV48 Uniprot:Q9ZV48
Root	Isotig00935	16	66	-2.173	1.23E-09	TAIR|locus:2054027 - symbol:TPS11 "trehalose phosphatase/synthase 11" species:3702 "Arabidopsis thaliana" [GO:0005992 "trehalose biosynthetic process" evidence=IEA] [GO:0008152 "metabolic process" evidence=IEA] [GO:0016757 "transferase activity, transferring glycosyl groups" evidence=ISS] [GO:0005739 "mitochondrion" evidence=IDA] [GO:0003825 "alpha,alpha-trehalose-phosphate synthase (UDP-forming) activity" evidence=IMP] [GO:0004805 "trehalose-phosphatase activity" evidence=IMP] [GO:0005829 "cytosol" evidence=IDA] InterPro:IPR001830 InterPro:IPR003337 InterPro:IPR006379 Pfam:PF00982 Pfam:PF02358 GO:GO:0005829 GO:GO:0005739 EMBL:CP002685 GenomeReviews:CT485783_GR InterPro:IPR023214 Gene3D:G3DSA:3.40.50.1000 SUPFAM:SSF56784 EMBL:AC005724 GO:GO:0005992 TIGRFAMs:TIGR01484 CAZy:GT20 eggNOG:COG0380 HOGENOM:HBG559076 GO:GO:0003825 HSSP:P31677 TIGRFAMs:TIGR00685 EMBL:AV822913 EMBL:AY042865 EMBL:AY081537 IPI:IPI00538425 IPI:IPI00889354 PIR:E84567 RefSeq:NP_179460.1 UniGene:At.25317 ProteinModelPortal:Q9ZV48 STRING:Q9ZV48 PRIDE:Q9ZV48 EnsemblPlants:AT2G18700.1 GeneID:816385 KEGG:ath:AT2G18700 TAIR:At2g18700 InParanoid:Q9ZV48 OMA:LQRACKD PhylomeDB:Q9ZV48 ProtClustDB:CLSN2912887 Genevestigator:Q9ZV48 Uniprot:Q9ZV48
Root	Isotig00936	15	64	-2.222	1.31E-09	TAIR|locus:2054027 - symbol:TPS11 "trehalose phosphatase/synthase 11" species:3702 "Arabidopsis thaliana" [GO:0005992 "trehalose biosynthetic process" evidence=IEA] [GO:0008152 "metabolic process" evidence=IEA] [GO:0016757 "transferase activity, transferring glycosyl groups" evidence=ISS] [GO:0005739 "mitochondrion" evidence=IDA] [GO:0003825 "alpha,alpha-trehalose-phosphate synthase (UDP-forming) activity" evidence=IMP] [GO:0004805 "trehalose-phosphatase activity" evidence=IMP] [GO:0005829 "cytosol" evidence=IDA] InterPro:IPR001830 InterPro:IPR003337 InterPro:IPR006379 Pfam:PF00982 Pfam:PF02358 GO:GO:0005829 GO:GO:0005739 EMBL:CP002685 GenomeReviews:CT485783_GR InterPro:IPR023214 Gene3D:G3DSA:3.40.50.1000 SUPFAM:SSF56784 EMBL:AC005724 GO:GO:0005992 TIGRFAMs:TIGR01484

Tissue	Identification	# of reads (stress)	# of reads (control)	Fold Change	p-value	Description
						CAZy:GT20 eggNOG:COG0380 HOGENOM:HBG559076 GO:GO:0003825 HSSP:P31677 TIGRFAMs:TIGR00685 EMBL:AV822913 EMBL:AY042865 EMBL:AY081537 IPI:IPI00538425 IPI:IPI00889354 PIR:E84567 RefSeq:NP_179460.1 UniGene:At.25317 ProteinModelPortal:Q9ZV48 STRING:Q9ZV48 PRIDE:Q9ZV48 EnsemblPlants:AT2G18700.1 GeneID:816385 KEGG:ath:AT2G18700 TAIR:At2g18700 InParanoid:Q9ZV48 OMA:LQRACKD PhylomeDB:Q9ZV48 ProtClustDB:CLSN2912887 Genevestigator:Q9ZV48 Uniprot:Q9ZV48
Root	Isotig00947	7	39	-2.607	2.03E-07	TAIR|locus:2164391 - symbol:NAC103 "AT5G64060" species:3702 "Arabidopsis thaliana" [GO:0005575 "cellular_component" evidence=ND] [GO:0006355 "regulation of transcription, DNA-dependent" evidence=IEA] [GO:0007275 "multicellular organismal development" evidence=ISS] [GO:0003700 "sequence-specific DNA binding transcription factor activity" evidence=ISS] InterPro:IPR003441 Pfam:PF02365 PROSITE:PS51005 EMBL:CP002688 GenomeReviews:BA000015_GR GO:GO:0006355 GO:GO:0003677 HSSP:Q9C932 SUPFAM:SSF101941 EMBL:AB008266 IPI:IPI00520409 RefSeq:NP_201211.1 UniGene:At.50553 ProteinModelPortal:Q9FMJ1 SMR:Q9FMJ1 DNASU:836527 EnsemblPlants:AT5G64060.1 GeneID:836527 KEGG:ath:AT5G64060 TAIR:At5g64060 eggNOG:NOG122520 HOGENOM:HBG317258 InParanoid:Q9FMJ1 OMA:ANRSTEC PhylomeDB:Q9FMJ1 ProtClustDB:CLSN2686235 Genevestigator:Q9FMJ1 Uniprot:Q9FMJ1
Root	Isotig00948	8	38	-2.377	1.13E-06	TAIR|locus:2164391 - symbol:NAC103 "AT5G64060" species:3702 "Arabidopsis thaliana" [GO:0005575 "cellular_component" evidence=ND] [GO:0006355 "regulation of transcription, DNA-dependent" evidence=IEA] [GO:0007275 "multicellular organismal development" evidence=ISS] [GO:0003700 "sequence-specific DNA binding transcription factor activity" evidence=ISS] InterPro:IPR003441 Pfam:PF02365 PROSITE:PS51005 EMBL:CP002688 GenomeReviews:BA000015_GR GO:GO:0006355 GO:GO:0003677 HSSP:Q9C932 SUPFAM:SSF101941 EMBL:AB008266 IPI:IPI00520409 RefSeq:NP_201211.1 UniGene:At.50553 ProteinModelPortal:Q9FMJ1 SMR:Q9FMJ1 DNASU:836527 EnsemblPlants:AT5G64060.1 GeneID:836527 KEGG:ath:AT5G64060 TAIR:At5g64060 eggNOG:NOG122520 HOGENOM:HBG317258 InParanoid:Q9FMJ1 OMA:ANRSTEC PhylomeDB:Q9FMJ1 ProtClustDB:CLSN2686235 Genevestigator:Q9FMJ1 Uniprot:Q9FMJ1
Root	Isotig00949	7	39	-2.607	2.03E-07	TAIR|locus:2164391 - symbol:NAC103 "AT5G64060" species:3702 "Arabidopsis thaliana" [GO:0005575 "cellular_component" evidence=ND] [GO:0006355 "regulation of transcription, DNA-dependent" evidence=IEA] [GO:0007275 "multicellular organismal development" evidence=ISS] [GO:0003700 "sequence-specific DNA binding transcription factor activity" evidence=ISS] InterPro:IPR003441 Pfam:PF02365 PROSITE:PS51005 EMBL:CP002688 GenomeReviews:BA000015_GR GO:GO:0006355 GO:GO:0003677 HSSP:Q9C932 SUPFAM:SSF101941 EMBL:AB008266 IPI:IPI00520409 RefSeq:NP_201211.1 UniGene:At.50553 ProteinModelPortal:Q9FMJ1 SMR:Q9FMJ1 DNASU:836527 EnsemblPlants:AT5G64060.1 GeneID:836527 KEGG:ath:AT5G64060 TAIR:At5g64060 eggNOG:NOG122520 HOGENOM:HBG317258 InParanoid:Q9FMJ1 OMA:ANRSTEC PhylomeDB:Q9FMJ1 ProtClustDB:CLSN2686235 Genevestigator:Q9FMJ1 Uniprot:Q9FMJ1
Root	Isotig00950	8	36	-2.299	3.42E-06	TAIR|locus:2184742 - symbol:NAC082 "AT5G09330" species:3702 "Arabidopsis thaliana" [GO:0005575 "cellular_component" evidence=ND] [GO:0006355 "regulation of transcription, DNA-dependent" evidence=IEA] [GO:0007275 "multicellular organismal development" evidence=ISS] [GO:0003700 "sequence-specific DNA binding transcription factor activity" evidence=ISS] InterPro:IPR003441 Pfam:PF02365 PROSITE:PS51005 EMBL:CP002688 GO:GO:0006355 GO:GO:0003677 EMBL:AL391712 HSSP:Q9C932 SUPFAM:SSF101941 ProtClustDB:CLSN2686235 EMBL:BT020263 EMBL:AK317217 IPI:IPI00527439 RefSeq:NP_001078551.1 RefSeq:NP_001078552.1 RefSeq:NP_196495.1 UniGene:At.48014 UniGene:At.67761 ProteinModelPortal:Q9FY82 SMR:Q9FY82 IntAct:Q9FY82 STRING:Q9FY82 PRIDE:Q9FY82 EnsemblPlants:AT5G09330.1 EnsemblPlants:AT5G09330.2 EnsemblPlants:AT5G09330.3 EnsemblPlants:AT5G09330.4 GeneID:830792 KEGG:ath:AT5G09330 TAIR:At5g09330 InParanoid:Q9FY82 OMA:QDLTAPL PhylomeDB:Q9FY82 Genevestigator:Q9FY82 Uniprot:Q9FY82
Root	Isotig00951	8	38	-2.377	1.13E-06	TAIR|locus:2164391 - symbol:NAC103 "AT5G64060" species:3702 "Arabidopsis thaliana" [GO:0005575 "cellular_component" evidence=ND] [GO:0006355 "regulation of transcription, DNA-dependent" evidence=IEA] [GO:0007275 "multicellular organismal development" evidence=ISS] [GO:0003700 "sequence-specific DNA binding transcription factor activity" evidence=ISS] InterPro:IPR003441 Pfam:PF02365 PROSITE:PS51005 EMBL:CP002688 GenomeReviews:BA000015_GR GO:GO:0006355 GO:GO:0003677 HSSP:Q9C932 SUPFAM:SSF101941 EMBL:AB008266 IPI:IPI00520409 RefSeq:NP_201211.1 UniGene:At.50553 ProteinModelPortal:Q9FMJ1 SMR:Q9FMJ1 DNASU:836527 EnsemblPlants:AT5G64060.1 GeneID:836527 KEGG:ath:AT5G64060 TAIR:At5g64060 eggNOG:NOG122520 HOGENOM:HBG317258 InParanoid:Q9FMJ1 OMA:ANRSTEC PhylomeDB:Q9FMJ1 ProtClustDB:CLSN2686235 Genevestigator:Q9FMJ1 Uniprot:Q9FMJ1

Tissue	Identification	# of reads (stress)	# of reads (control)	Fold Change	p-value	Description
Root	Isotig00952	9	35	-2.088	1.60E-05	TAIR|locus:2184742 - symbol:NAC082 "AT5G09330" species:3702 "Arabidopsis thaliana" [GO:0005575 "cellular_component" evidence=ND] [GO:0006355 "regulation of transcription, DNA-dependent" evidence=IEA] [GO:0007275 "multicellular organismal development" evidence=ISS] [GO:0003700 "sequence-specific DNA binding transcription factor activity" evidence=ISS] InterPro:IPR003441 Pfam:PF02365 PROSITE:PS51005 EMBL:CP002688 GO:GO:0006355 GO:GO:0003677 EMBL:AL391712 HSSP:Q9C932 SUPFAM:SSF101941 ProtClustDB:CLSN2686235 EMBL:BT020263 EMBL:AK317217 IPI:IPI00527439 RefSeq:NP_001078551.1 RefSeq:NP_001078552.1 RefSeq:NP_196495.1 UniGene:At.48014 UniGene:At.67761 ProteinModelPortal:Q9FY82 SMR:Q9FY82 IntAct:Q9FY82 STRING:Q9FY82 PRIDE:Q9FY82 EnsemblPlants:AT5G09330.1 EnsemblPlants:AT5G09330.2 EnsemblPlants:AT5G09330.3 EnsemblPlants:AT5G09330.4 GeneID:830792 KEGG:ath:AT5G09330 TAIR:At5g09330 InParanoid:Q9FY82 OMA:QDLTAPL PhylomeDB:Q9FY82 Genevestigator:Q9FY82 Uniprot:Q9FY82
Root	Isotig00953	8	36	-2.299	3.42E-06	TAIR|locus:2184742 - symbol:NAC082 "AT5G09330" species:3702 "Arabidopsis thaliana" [GO:0005575 "cellular_component" evidence=ND] [GO:0006355 "regulation of transcription, DNA-dependent" evidence=IEA] [GO:0007275 "multicellular organismal development" evidence=ISS] [GO:0003700 "sequence-specific DNA binding transcription factor activity" evidence=ISS] InterPro:IPR003441 Pfam:PF02365 PROSITE:PS51005 EMBL:CP002688 GO:GO:0006355 GO:GO:0003677 EMBL:AL391712 HSSP:Q9C932 SUPFAM:SSF101941 ProtClustDB:CLSN2686235 EMBL:BT020263 EMBL:AK317217 IPI:IPI00527439 RefSeq:NP_001078551.1 RefSeq:NP_001078552.1 RefSeq:NP_196495.1 UniGene:At.48014 UniGene:At.67761 ProteinModelPortal:Q9FY82 SMR:Q9FY82 IntAct:Q9FY82 STRING:Q9FY82 PRIDE:Q9FY82 EnsemblPlants:AT5G09330.1 EnsemblPlants:AT5G09330.2 EnsemblPlants:AT5G09330.3 EnsemblPlants:AT5G09330.4 GeneID:830792 KEGG:ath:AT5G09330 TAIR:At5g09330 InParanoid:Q9FY82 OMA:QDLTAPL PhylomeDB:Q9FY82 Genevestigator:Q9FY82 Uniprot:Q9FY82
Root	Isotig00954	9	35	-2.088	1.60E-05	TAIR|locus:2184742 - symbol:NAC082 "AT5G09330" species:3702 "Arabidopsis thaliana" [GO:0005575 "cellular_component" evidence=ND] [GO:0006355 "regulation of transcription, DNA-dependent" evidence=IEA] [GO:0007275 "multicellular organismal development" evidence=ISS] [GO:0003700 "sequence-specific DNA binding transcription factor activity" evidence=ISS] InterPro:IPR003441 Pfam:PF02365 PROSITE:PS51005 EMBL:CP002688 GO:GO:0006355 GO:GO:0003677 EMBL:AL391712 HSSP:Q9C932 SUPFAM:SSF101941 ProtClustDB:CLSN2686235 EMBL:BT020263 EMBL:AK317217 IPI:IPI00527439 RefSeq:NP_001078551.1 RefSeq:NP_001078552.1 RefSeq:NP_196495.1 UniGene:At.48014 UniGene:At.67761 ProteinModelPortal:Q9FY82 SMR:Q9FY82 IntAct:Q9FY82 STRING:Q9FY82 PRIDE:Q9FY82 EnsemblPlants:AT5G09330.1 EnsemblPlants:AT5G09330.2 EnsemblPlants:AT5G09330.3 EnsemblPlants:AT5G09330.4 GeneID:830792 KEGG:ath:AT5G09330 TAIR:At5g09330 InParanoid:Q9FY82 OMA:QDLTAPL PhylomeDB:Q9FY82 Genevestigator:Q9FY82 Uniprot:Q9FY82
Root	Isotig00955	5	23	-2.331	0.000182103	TAIR|locus:2164391 - symbol:NAC103 "AT5G64060" species:3702 "Arabidopsis thaliana" [GO:0005575 "cellular_component" evidence=ND] [GO:0006355 "regulation of transcription, DNA-dependent" evidence=IEA] [GO:0007275 "multicellular organismal development" evidence=ISS] [GO:0003700 "sequence-specific DNA binding transcription factor activity" evidence=ISS] InterPro:IPR003441 Pfam:PF02365 PROSITE:PS51005 EMBL:CP002688 GenomeReviews:BA000015_GR GO:GO:0006355 GO:GO:0003677 HSSP:Q9C932 SUPFAM:SSF101941 EMBL:AB008266 IPI:IPI00520409 RefSeq:NP_201211.1 UniGene:At.50553 ProteinModelPortal:Q9FMJ1 SMR:Q9FMJ1 DNASU:836527 EnsemblPlants:AT5G64060.1 GeneID:836527 KEGG:ath:AT5G64060 TAIR:At5g64060 eggNOG:NOG122520 HOGENOM:HBG317258 InParanoid:Q9FMJ1 OMA:ANRSTEC PhylomeDB:Q9FMJ1 ProtClustDB:CLSN2686235 Genevestigator:Q9FMJ1 Uniprot:Q9FMJ1
Root	Isotig00956	5	23	-2.331	0.000182103	TAIR|locus:2164391 - symbol:NAC103 "AT5G64060" species:3702 "Arabidopsis thaliana" [GO:0005575 "cellular_component" evidence=ND] [GO:0006355 "regulation of transcription, DNA-dependent" evidence=IEA] [GO:0007275 "multicellular organismal development" evidence=ISS] [GO:0003700 "sequence-specific DNA binding transcription factor activity" evidence=ISS] InterPro:IPR003441 Pfam:PF02365 PROSITE:PS51005 EMBL:CP002688 GenomeReviews:BA000015_GR GO:GO:0006355 GO:GO:0003677 HSSP:Q9C932 SUPFAM:SSF101941 EMBL:AB008266 IPI:IPI00520409 RefSeq:NP_201211.1 UniGene:At.50553 ProteinModelPortal:Q9FMJ1 SMR:Q9FMJ1 DNASU:836527 EnsemblPlants:AT5G64060.1 GeneID:836527 KEGG:ath:AT5G64060 TAIR:At5g64060 eggNOG:NOG122520 HOGENOM:HBG317258 InParanoid:Q9FMJ1 OMA:ANRSTEC PhylomeDB:Q9FMJ1 ProtClustDB:CLSN2686235 Genevestigator:Q9FMJ1 Uniprot:Q9FMJ1

Tissue	Identification	# of reads (stress)	# of reads (control)	Fold Change	p-value	Description
Root	Isotig00967	40	2	4.193	7.78E-10	SGD|S000028422 - symbol:TAR1 "Mitochondrial protein potentially involved in respiratory metabolism" species:4932 "Saccharomyces cerevisiae" [GO:0003674 "molecular_function" evidence=ND] [GO:0043457 "regulation of cellular respiration" evidence=IGI] [GO:0005739 "mitochondrion" evidence=IEA;IMP] SGD:S000028422 GO:GO:0005739 EMBL:BK006945 EMBL:U53879 GO:GO:0043457 EMBL:Z73326 EMBL:AF479964 RefSeq:NP_690845.1 ProteinModelPortal:Q8TGM6 STRING:Q8TGM6 EnsemblFungi:YLR154W-C GeneID:1466405 KEGG:sce:YLR154W-C CYGD:YLR154w-c eggNOG:NOG79861 OMA:SCSKEHR OrthoDB:EOG466ZX8 NextBio:967146 Genevestigator:Q8TGM6 GermOnline:YLR154W-C Uniprot:Q8TGM6
Root	Isotig00968	40	2	4.193	7.78E-10	SGD|S000028422 - symbol:TAR1 "Mitochondrial protein potentially involved in respiratory metabolism" species:4932 "Saccharomyces cerevisiae" [GO:0003674 "molecular_function" evidence=ND] [GO:0043457 "regulation of cellular respiration" evidence=IGI] [GO:0005739 "mitochondrion" evidence=IEA;IMP] SGD:S000028422 GO:GO:0005739 EMBL:BK006945 EMBL:U53879 GO:GO:0043457 EMBL:Z73326 EMBL:AF479964 RefSeq:NP_690845.1 ProteinModelPortal:Q8TGM6 STRING:Q8TGM6 EnsemblFungi:YLR154W-C GeneID:1466405 KEGG:sce:YLR154W-C CYGD:YLR154w-c eggNOG:NOG79861 OMA:SCSKEHR OrthoDB:EOG466ZX8 NextBio:967146 Genevestigator:Q8TGM6 GermOnline:YLR154W-C Uniprot:Q8TGM6
Root	Isotig00969	40	2	4.193	7.78E-10	SGD|S000028422 - symbol:TAR1 "Mitochondrial protein potentially involved in respiratory metabolism" species:4932 "Saccharomyces cerevisiae" [GO:0003674 "molecular_function" evidence=ND] [GO:0043457 "regulation of cellular respiration" evidence=IGI] [GO:0005739 "mitochondrion" evidence=IEA;IMP] SGD:S000028422 GO:GO:0005739 EMBL:BK006945 EMBL:U53879 GO:GO:0043457 EMBL:Z73326 EMBL:AF479964 RefSeq:NP_690845.1 ProteinModelPortal:Q8TGM6 STRING:Q8TGM6 EnsemblFungi:YLR154W-C GeneID:1466405 KEGG:sce:YLR154W-C CYGD:YLR154w-c eggNOG:NOG79861 OMA:SCSKEHR OrthoDB:EOG466ZX8 NextBio:967146 Genevestigator:Q8TGM6 GermOnline:YLR154W-C Uniprot:Q8TGM6
Root	Isotig00970	22	0	5.330	1.93E-06	SGD|S000028422 - symbol:TAR1 "Mitochondrial protein potentially involved in respiratory metabolism" species:4932 "Saccharomyces cerevisiae" [GO:0003674 "molecular_function" evidence=ND] [GO:0043457 "regulation of cellular respiration" evidence=IGI] [GO:0005739 "mitochondrion" evidence=IEA;IMP] SGD:S000028422 GO:GO:0005739 EMBL:BK006945 EMBL:U53879 GO:GO:0043457 EMBL:Z73326 EMBL:AF479964 RefSeq:NP_690845.1 ProteinModelPortal:Q8TGM6 STRING:Q8TGM6 EnsemblFungi:YLR154W-C GeneID:1466405 KEGG:sce:YLR154W-C CYGD:YLR154w-c eggNOG:NOG79861 OMA:SCSKEHR OrthoDB:EOG466ZX8 NextBio:967146 Genevestigator:Q8TGM6 GermOnline:YLR154W-C Uniprot:Q8TGM6
Root	Isotig00971	26	1	4.571	4.15E-07	RGD|727924 - symbol:LOC257642 "rRNA promoter binding protein" species:10116 "Rattus norvegicus" [GO:0003690 "double-stranded DNA binding" evidence=IDA] [GO:0003700 "sequence-specific DNA binding transcription factor activity" evidence=IDA] [GO:0008134 "transcription factor binding" evidence=IDA] [GO:0008283 "cell proliferation" evidence=IDA] [GO:0009303 "rRNA transcription" evidence=IDA] RGD:727924 GO:GO:0008134 GO:GO:0008283 GO:GO:0003700 GO:GO:0003690 GO:GO:0009303 EMBL:U77931 IPI:IPI00195015 RefSeq:NP_671477.1 GeneID:257642 KEGG:rno:257642 NextBio:624170 Genevestigator:Q99JC0 Uniprot:Q99JC0
Root	Isotig00972	26	1	4.571	4.15E-07	RGD|727924 - symbol:LOC257642 "rRNA promoter binding protein" species:10116 "Rattus norvegicus" [GO:0003690 "double-stranded DNA binding" evidence=IDA] [GO:0003700 "sequence-specific DNA binding transcription factor activity" evidence=IDA] [GO:0008134 "transcription factor binding" evidence=IDA] [GO:0008283 "cell proliferation" evidence=IDA] [GO:0009303 "rRNA transcription" evidence=IDA] RGD:727924 GO:GO:0008134 GO:GO:0008283 GO:GO:0003700 GO:GO:0003690 GO:GO:0009303 EMBL:U77931 IPI:IPI00195015 RefSeq:NP_671477.1 GeneID:257642 KEGG:rno:257642 NextBio:624170 Genevestigator:Q99JC0 Uniprot:Q99JC0
Root	Isotig00973	26	1	4.571	4.15E-07	RGD|727924 - symbol:LOC257642 "rRNA promoter binding protein" species:10116 "Rattus norvegicus" [GO:0003690 "double-stranded DNA binding" evidence=IDA] [GO:0003700 "sequence-specific DNA binding transcription factor activity" evidence=IDA] [GO:0008134 "transcription factor binding" evidence=IDA] [GO:0008283 "cell proliferation" evidence=IDA] [GO:0009303 "rRNA transcription" evidence=IDA] RGD:727924 GO:GO:0008134 GO:GO:0008283 GO:GO:0003700 GO:GO:0003690 GO:GO:0009303 EMBL:U77931 IPI:IPI00195015 RefSeq:NP_671477.1 GeneID:257642 KEGG:rno:257642 NextBio:624170 Genevestigator:Q99JC0 Uniprot:Q99JC0
Root	Isotig00974	26	1	4.571	4.15E-07	RGD|727924 - symbol:LOC257642 "rRNA promoter binding protein" species:10116 "Rattus norvegicus" [GO:0003690 "double-stranded DNA binding" evidence=IDA] [GO:0003700 "sequence-specific DNA binding transcription factor activity" evidence=IDA] [GO:0008134 "transcription factor binding" evidence=IDA] [GO:0008283 "cell proliferation" evidence=IDA] [GO:0009303 "rRNA transcription" evidence=IDA] RGD:727924 GO:GO:0008134 GO:GO:0008283 GO:GO:0003700 GO:GO:0003690 GO:GO:0009303 EMBL:U77931 IPI:IPI00195015 RefSeq:NP_671477.1 GeneID:257642 KEGG:rno:257642 NextBio:624170 Genevestigator:Q99JC0 Uniprot:Q99JC0

Tissue	Identification	# of reads (stress)	# of reads (control)	Fold Change	p-value	Description
Root	Isotig00975	26	1	4.571	4.15E-07	RGD|727924 - symbol:LOC257642 "rRNA promoter binding protein" species:10116 "Rattus norvegicus" [GO:0003690 "double-stranded DNA binding" evidence=IDA] [GO:0003700 "sequence-specific DNA binding transcription factor activity" evidence=IDA] [GO:0008134 "transcription factor binding" evidence=IDA] [GO:0008283 "cell proliferation" evidence=IDA] [GO:0009303 "rRNA transcription" evidence=IDA] RGD:727924 GO:GO:0008134 GO:GO:0008283 GO:GO:0003700 GO:GO:0003690 GO:GO:0009303 EMBL:U77931 IPI:IPI00195015 RefSeq:NP_671477.1 GeneID:257642 KEGG:rno:257642 NextBio:624170 Genevestigator:Q99JC0 Uniprot:Q99JC0
Root	Isotig00976	26	1	4.571	4.15E-07	RGD|727924 - symbol:LOC257642 "rRNA promoter binding protein" species:10116 "Rattus norvegicus" [GO:0003690 "double-stranded DNA binding" evidence=IDA] [GO:0003700 "sequence-specific DNA binding transcription factor activity" evidence=IDA] [GO:0008134 "transcription factor binding" evidence=IDA] [GO:0008283 "cell proliferation" evidence=IDA] [GO:0009303 "rRNA transcription" evidence=IDA] RGD:727924 GO:GO:0008134 GO:GO:0008283 GO:GO:0003700 GO:GO:0003690 GO:GO:0009303 EMBL:U77931 IPI:IPI00195015 RefSeq:NP_671477.1 GeneID:257642 KEGG:rno:257642 NextBio:624170 Genevestigator:Q99JC0 Uniprot:Q99JC0
Root	Isotig00977	27	59	-1.257	9.88E-05	TAIR|locus:2041434 - symbol:ABCB4 "AT2G47000" species:3702 "Arabidopsis thaliana" [GO:0042626 "ATPase activity, coupled to transmembrane movement of substances" evidence=ISS] [GO:0005886 "plasma membrane" evidence=IDA] [GO:0008559 "xenobiotic-transporting ATPase activity" evidence=IDA] [GO:0009733 "response to auxin stimulus" evidence=IEP] [GO:0009735 "response to cytokinin stimulus" evidence=IEP] [GO:0009926 "auxin polar transport" evidence=TAS] [GO:0016020 "membrane" evidence=IDA] [GO:0009630 "gravitropism" evidence=IMP] [GO:0010540 "basipetal auxin transport" evidence=IMP] [GO:0010315 "auxin efflux" evidence=IMP] [GO:0048767 "root hair elongation" evidence=IMP] [GO:0009506 "plasmodesma" evidence=IDA] InterPro:IPR001140 InterPro:IPR003439 InterPro:IPR003593 InterPro:IPR011527 InterPro:IPR017871 Pfam:PF00005 Pfam:PF00664 PROSITE:PS00211 PROSITE:PS50893 SMART:SM00382 GO:GO:0016021 GO:GO:0005886 GO:GO:0009506 GO:GO:0005524 EMBL:CP002685 GenomeReviews:CT485783_GR GO:GO:0009735 eggNOG:COG1132 HOGENOM:HBG758042 KO:K05658 InterPro:IPR017940 SUPFAM:SSF90123 PROSITE:PS50929 GO:GO:0008559 GO:GO:0010540 ProtClustDB:CLSN2683573 EMBL:AC004411 IPI:IPI00538726 PIR:T02187 RefSeq:NP_182223.1 UniGene:At.43921 UniGene:At.67170 ProteinModelPortal:O80725 SMR:O80725 STRING:O80725 TCDB:3.A.1.201.7 PRIDE:O80725 ProMEX:O80725 EnsemblPlants:AT2G47000.1 GeneID:819314 KEGG:ath:AT2G47000 TAIR:At2g47000 InParanoid:O80725 OMA:IMDRESK PhylomeDB:O80725 ArrayExpress:O80725 Genevestigator:O80725 GermOnline:AT2G47000 GO:GO:0010315 GO:GO:0009630 GO:GO:0048767 Uniprot:O80725
Root	Isotig00978	27	60	-1.281	6.74E-05	TAIR|locus:2097978 - symbol:ABCB21 "ATP-binding cassette B21" species:3702 "Arabidopsis thaliana" [GO:0006810 "transport" evidence=IEA] [GO:0042626 "ATPase activity, coupled to transmembrane movement of substances" evidence=ISS] [GO:0055085 "transmembrane transport" evidence=IEA] [GO:0005886 "plasma membrane" evidence=IDA] [GO:0005774 "vacuolar membrane" evidence=IDA] InterPro:IPR001140 InterPro:IPR003439 InterPro:IPR003593 InterPro:IPR011527 InterPro:IPR017871 Pfam:PF00005 Pfam:PF00664 PROSITE:PS00211 PROSITE:PS50893 SMART:SM00382 GO:GO:0016021 GO:GO:0005886 GO:GO:0005524 GO:GO:0005774 EMBL:CP002686 GenomeReviews:BA000014_GR eggNOG:COG1132 HOGENOM:HBG758042 GO:GO:0042626 InterPro:IPR017940 SUPFAM:SSF90123 PROSITE:PS50929 EMBL:AL138651 IPI:IPI00546798 PIR:T48007 RefSeq:NP_191774.2 UniGene:At.34174 ProteinModelPortal:Q9M1Q9 STRING:Q9M1Q9 PRIDE:Q9M1Q9 GeneID:825388 KEGG:ath:AT3G62150 TAIR:At3g62150 InParanoid:Q9M1Q9 OMA:PFHKLFA PhylomeDB:Q9M1Q9 ProtClustDB:CLSN2683573 Genevestigator:Q9M1Q9 GermOnline:AT3G62150 Uniprot:Q9M1Q9
Root	Isotig00979	27	58	-1.232	0.000144193	TAIR|locus:2041434 - symbol:ABCB4 "AT2G47000" species:3702 "Arabidopsis thaliana" [GO:0042626 "ATPase activity, coupled to transmembrane movement of substances" evidence=ISS] [GO:0005886 "plasma membrane" evidence=IDA] [GO:0008559 "xenobiotic-transporting ATPase activity" evidence=IDA] [GO:0009733 "response to auxin stimulus" evidence=IEP] [GO:0009735 "response to cytokinin stimulus" evidence=IEP] [GO:0009926 "auxin polar transport" evidence=TAS] [GO:0016020 "membrane" evidence=IDA] [GO:0009630 "gravitropism" evidence=IMP] [GO:0010540 "basipetal auxin transport" evidence=IMP] [GO:0010315 "auxin efflux" evidence=IMP] [GO:0048767 "root hair elongation" evidence=IMP] [GO:0009506 "plasmodesma" evidence=IDA] InterPro:IPR001140 InterPro:IPR003439 InterPro:IPR003593 InterPro:IPR011527 InterPro:IPR017871 Pfam:PF00005 Pfam:PF00664 PROSITE:PS00211 PROSITE:PS50893 SMART:SM00382 GO:GO:0016021 GO:GO:0005886 GO:GO:0009506 GO:GO:0005524 EMBL:CP002685 GenomeReviews:CT485783_GR GO:GO:0009735 eggNOG:COG1132 HOGENOM:HBG758042 KO:K05658 InterPro:IPR017940

Tissue	Identification	# of reads (stress)	# of reads (control)	Fold Change	p-value	Description
						SUPFAM:SSF90123 PROSITE:PS50929 GO:GO:0008559 GO:GO:0010540 ProtClustDB:CLSN2683573 EMBL:AC004411 IPI:IPI00538726 PIR:T02187 RefSeq:NP_182223.1 UniGene:At.43921 UniGene:At.67170 ProteinModelPortal:O80725 SMR:O80725 STRING:O80725 TCDB:3.A.1.201.7 PRIDE:O80725 ProMEX:O80725 EnsemblPlants:AT2G47000.1 GeneID:819314 KEGG:ath:AT2G47000 TAIR:At2g47000 InParanoid:O80725 OMA:IMDRESK PhylomeDB:O80725 ArrayExpress:O80725 Genevestigator:O80725 GermOnline:AT2G47000 GO:GO:0010315 GO:GO:0009630 GO:GO:0048767 Uniprot:O80725
Root	Isotig00980	27	59	-1.257	9.88E-05	TAIR|locus:2097978 - symbol:ABCB21 "ATP-binding cassette B21" species:3702 "Arabidopsis thaliana" [GO:0006810 "transport" evidence=IEA] [GO:0042626 "ATPase activity, coupled to transmembrane movement of substances" evidence=ISS] [GO:0055085 "transmembrane transport" evidence=IEA] [GO:0005886 "plasma membrane" evidence=IDA] [GO:0005774 "vacuolar membrane" evidence=IDA] InterPro:IPR001140 InterPro:IPR003439 InterPro:IPR003593 InterPro:IPR011527 InterPro:IPR017871 Pfam:PF00005 Pfam:PF00664 PROSITE:PS00211 PROSITE:PS50893 SMART:SM00382 GO:GO:0016021 GO:GO:0005886 GO:GO:0005524 GO:GO:0005774 EMBL:CP002686 GenomeReviews:BA000014_GR eggNOG:COG1132 HOGENOM:HBG758042 GO:GO:0042626 InterPro:IPR017940 SUPFAM:SSF90123 PROSITE:PS50929 EMBL:AL138651 IPI:IPI00546798 PIR:T48007 RefSeq:NP_191774.2 UniGene:At.34174 ProteinModelPortal:Q9M1Q9 STRING:Q9M1Q9 PRIDE:Q9M1Q9 GeneID:825388 KEGG:ath:AT3G62150 TAIR:At3g62150 InParanoid:Q9M1Q9 OMA:PFHKLFA PhylomeDB:Q9M1Q9 ProtClustDB:CLSN2683573 Genevestigator:Q9M1Q9 GermOnline:AT3G62150 Uniprot:Q9M1Q9
Root	Isotig00981	27	59	-1.257	9.88E-05	TAIR|locus:2097978 - symbol:ABCB21 "ATP-binding cassette B21" species:3702 "Arabidopsis thaliana" [GO:0006810 "transport" evidence=IEA] [GO:0042626 "ATPase activity, coupled to transmembrane movement of substances" evidence=ISS] [GO:0055085 "transmembrane transport" evidence=IEA] [GO:0005886 "plasma membrane" evidence=IDA] [GO:0005774 "vacuolar membrane" evidence=IDA] InterPro:IPR001140 InterPro:IPR003439 InterPro:IPR003593 InterPro:IPR011527 InterPro:IPR017871 Pfam:PF00005 Pfam:PF00664 PROSITE:PS00211 PROSITE:PS50893 SMART:SM00382 GO:GO:0016021 GO:GO:0005886 GO:GO:0005524 GO:GO:0005774 EMBL:CP002686 GenomeReviews:BA000014_GR eggNOG:COG1132 HOGENOM:HBG758042 GO:GO:0042626 InterPro:IPR017940 SUPFAM:SSF90123 PROSITE:PS50929 EMBL:AL138651 IPI:IPI00546798 PIR:T48007 RefSeq:NP_191774.2 UniGene:At.34174 ProteinModelPortal:Q9M1Q9 STRING:Q9M1Q9 PRIDE:Q9M1Q9 GeneID:825388 KEGG:ath:AT3G62150 TAIR:At3g62150 InParanoid:Q9M1Q9 OMA:PFHKLFA PhylomeDB:Q9M1Q9 ProtClustDB:CLSN2683573 Genevestigator:Q9M1Q9 GermOnline:AT3G62150 Uniprot:Q9M1Q9
Root	Isotig00984	27	58	-1.232	0.000144193	TAIR|locus:2097978 - symbol:ABCB21 "ATP-binding cassette B21" species:3702 "Arabidopsis thaliana" [GO:0006810 "transport" evidence=IEA] [GO:0042626 "ATPase activity, coupled to transmembrane movement of substances" evidence=ISS] [GO:0055085 "transmembrane transport" evidence=IEA] [GO:0005886 "plasma membrane" evidence=IDA] [GO:0005774 "vacuolar membrane" evidence=IDA] InterPro:IPR001140 InterPro:IPR003439 InterPro:IPR003593 InterPro:IPR011527 InterPro:IPR017871 Pfam:PF00005 Pfam:PF00664 PROSITE:PS00211 PROSITE:PS50893 SMART:SM00382 GO:GO:0016021 GO:GO:0005886 GO:GO:0005524 GO:GO:0005774 EMBL:CP002686 GenomeReviews:BA000014_GR eggNOG:COG1132 HOGENOM:HBG758042 GO:GO:0042626 InterPro:IPR017940 SUPFAM:SSF90123 PROSITE:PS50929 EMBL:AL138651 IPI:IPI00546798 PIR:T48007 RefSeq:NP_191774.2 UniGene:At.34174 ProteinModelPortal:Q9M1Q9 STRING:Q9M1Q9 PRIDE:Q9M1Q9 GeneID:825388 KEGG:ath:AT3G62150 TAIR:At3g62150 InParanoid:Q9M1Q9 OMA:PFHKLFA PhylomeDB:Q9M1Q9 ProtClustDB:CLSN2683573 Genevestigator:Q9M1Q9 GermOnline:AT3G62150 Uniprot:Q9M1Q9
Root	Isotig00996	7	24	-1.907	0.000762598	TAIR|locus:2034310 - symbol:ACC1 "AT1G36160" species:3702 "Arabidopsis thaliana" [GO:0003989 "acetyl-CoA carboxylase activity" evidence=ISS] [GO:0010072 "primary shoot apical meristem specification" evidence=IMP] [GO:0030497 "fatty acid elongation" evidence=IMP] [GO:0005886 "plasma membrane" evidence=IDA] [GO:0009793 "embryo development ending in seed dormancy" evidence=IMP] [GO:0009933 "meristem structural organization" evidence=IMP] [GO:0009735 "response to cytokinin stimulus" evidence=IMP] [GO:0048364 "root development" evidence=IMP] [GO:0048367 "shoot development" evidence=IMP] InterPro:IPR000022 InterPro:IPR005479 InterPro:IPR005481 InterPro:IPR005482 InterPro:IPR011761 InterPro:IPR011762 InterPro:IPR011763 InterPro:IPR011764 InterPro:IPR013537 InterPro:IPR013815 InterPro:IPR013816 Pfam:PF00289 Pfam:PF01039 Pfam:PF02785 Pfam:PF02786 Pfam:PF08326 PROSITE:PS00866 PROSITE:PS00867 PROSITE:PS50975 PROSITE:PS50979 PROSITE:PS50980 PROSITE:PS50989 SMART:SM00878 InterPro:IPR013817

Tissue	Identification	# of reads (stress)	# of reads (control)	Fold Change	p-value	Description
						InterPro:IPR016185 InterPro:IPR001882 InterPro:IPR000089 Pfam:PF00364 EMBL:CP002684 GO:GO:0005829 GO:GO:0005886 GO:GO:0005524 GO:GO:0009735 GO:GO:0046872 GO:GO:0030497 GO:GO:0048364 eggNOG:COG0511 KO:K11262 GO:GO:0003989 GO:GO:0004075 InterPro:IPR011054 InterPro:IPR011053 Gene3D:G3DSA:3.30.1490.20 Gene3D:G3DSA:3.30.470.20 Gene3D:G3DSA:3.40.50.20 SUPFAM:SSF51230 SUPFAM:SSF52440 SUPFAM:SSF51246 PROSITE:PS00188 PROSITE:PS50968 OMA:DGLREYK HSSP:Q00955 EMBL:L27074 EMBL:D34630 EMBL:AF062308 EMBL:AC006228 EMBL:AC025781 EMBL:AK229488 IPI:IPI00543409 PIR:D86483 RefSeq:NP_001185143.1 RefSeq:NP_174849.2 UniGene:At.39402 ProteinModelPortal:Q38970 SMR:Q38970 IntAct:Q38970 STRING:Q38970 PRIDE:Q38971 EnsemblPlants:AT1G36160.1 EnsemblPlants:AT1G36160.2 GeneID:840521 KEGG:ath:AT1G36160 TAIR:At1g36160 InParanoid:Q38970 PhylomeDB:Q38970 ProtClustDB:CLSN2681517 Genevestigator:Q38970 GO:GO:0010072 Uniprot:Q38970
Root	Isotig01021	204	22	3.084	3.38E-35	SGD|S000028422 - symbol:TAR1 "Mitochondrial protein potentially involved in respiratory metabolism" species:4932 "Saccharomyces cerevisiae" [GO:0003674 "molecular_function" evidence=ND] [GO:0043457 "regulation of cellular respiration" evidence=IGI] [GO:0005739 "mitochondrion" evidence=IEA;IMP] SGD:S000028422 GO:GO:0005739 EMBL:BK006945 EMBL:U53879 GO:GO:0043457 EMBL:Z73326 EMBL:AF479964 RefSeq:NP_690845.1 ProteinModelPortal:Q8TGM6 STRING:Q8TGM6 EnsemblFungi:YLR154W-C GeneID:1466405 KEGG:sce:YLR154W-C CYGD:YLR154w-c eggNOG:NOG79861 OMA:SCSKEHR OrthoDB:EOG466ZX8 NextBio:967146 Genevestigator:Q8TGM6 GermOnline:YLR154W-C Uniprot:Q8TGM6
Root	Isotig01022	181	15	3.464	1.44E-34	SGD|S000028422 - symbol:TAR1 "Mitochondrial protein potentially involved in respiratory metabolism" species:4932 "Saccharomyces cerevisiae" [GO:0003674 "molecular_function" evidence=ND] [GO:0043457 "regulation of cellular respiration" evidence=IGI] [GO:0005739 "mitochondrion" evidence=IEA;IMP] SGD:S000028422 GO:GO:0005739 EMBL:BK006945 EMBL:U53879 GO:GO:0043457 EMBL:Z73326 EMBL:AF479964 RefSeq:NP_690845.1 ProteinModelPortal:Q8TGM6 STRING:Q8TGM6 EnsemblFungi:YLR154W-C GeneID:1466405 KEGG:sce:YLR154W-C CYGD:YLR154w-c eggNOG:NOG79861 OMA:SCSKEHR OrthoDB:EOG466ZX8 NextBio:967146 Genevestigator:Q8TGM6 GermOnline:YLR154W-C Uniprot:Q8TGM6
Root	Isotig01023	198	21	3.108	1.90E-34	SGD|S000028422 - symbol:TAR1 "Mitochondrial protein potentially involved in respiratory metabolism" species:4932 "Saccharomyces cerevisiae" [GO:0003674 "molecular_function" evidence=ND] [GO:0043457 "regulation of cellular respiration" evidence=IGI] [GO:0005739 "mitochondrion" evidence=IEA;IMP] SGD:S000028422 GO:GO:0005739 EMBL:BK006945 EMBL:U53879 GO:GO:0043457 EMBL:Z73326 EMBL:AF479964 RefSeq:NP_690845.1 ProteinModelPortal:Q8TGM6 STRING:Q8TGM6 EnsemblFungi:YLR154W-C GeneID:1466405 KEGG:sce:YLR154W-C CYGD:YLR154w-c eggNOG:NOG79861 OMA:SCSKEHR OrthoDB:EOG466ZX8 NextBio:967146 Genevestigator:Q8TGM6 GermOnline:YLR154W-C Uniprot:Q8TGM6
Root	Isotig01024	88	7	3.523	7.49E-18	SGD|S000028422 - symbol:TAR1 "Mitochondrial protein potentially involved in respiratory metabolism" species:4932 "Saccharomyces cerevisiae" [GO:0003674 "molecular_function" evidence=ND] [GO:0043457 "regulation of cellular respiration" evidence=IGI] [GO:0005739 "mitochondrion" evidence=IEA;IMP] SGD:S000028422 GO:GO:0005739 EMBL:BK006945 EMBL:U53879 GO:GO:0043457 EMBL:Z73326 EMBL:AF479964 RefSeq:NP_690845.1 ProteinModelPortal:Q8TGM6 STRING:Q8TGM6 EnsemblFungi:YLR154W-C GeneID:1466405 KEGG:sce:YLR154W-C CYGD:YLR154w-c eggNOG:NOG79861 OMA:SCSKEHR OrthoDB:EOG466ZX8 NextBio:967146 Genevestigator:Q8TGM6 GermOnline:YLR154W-C Uniprot:Q8TGM6
Root	Isotig01025	200	22	3.055	2.96E-34	SGD|S000028422 - symbol:TAR1 "Mitochondrial protein potentially involved in respiratory metabolism" species:4932 "Saccharomyces cerevisiae" [GO:0003674 "molecular_function" evidence=ND] [GO:0043457 "regulation of cellular respiration" evidence=IGI] [GO:0005739 "mitochondrion" evidence=IEA;IMP] SGD:S000028422 GO:GO:0005739 EMBL:BK006945 EMBL:U53879 GO:GO:0043457 EMBL:Z73326 EMBL:AF479964 RefSeq:NP_690845.1 ProteinModelPortal:Q8TGM6 STRING:Q8TGM6 EnsemblFungi:YLR154W-C GeneID:1466405 KEGG:sce:YLR154W-C CYGD:YLR154w-c eggNOG:NOG79861 OMA:SCSKEHR OrthoDB:EOG466ZX8 NextBio:967146 Genevestigator:Q8TGM6 GermOnline:YLR154W-C Uniprot:Q8TGM6
Root	Isotig01026	177	15	3.432	1.34E-33	SGD|S000028422 - symbol:TAR1 "Mitochondrial protein potentially involved in respiratory metabolism" species:4932 "Saccharomyces cerevisiae" [GO:0003674 "molecular_function" evidence=ND] [GO:0043457 "regulation of cellular respiration" evidence=IGI] [GO:0005739 "mitochondrion" evidence=IEA;IMP] SGD:S000028422 GO:GO:0005739 EMBL:BK006945 EMBL:U53879 GO:GO:0043457 EMBL:Z73326 EMBL:AF479964 RefSeq:NP_690845.1 ProteinModelPortal:Q8TGM6 STRING:Q8TGM6 EnsemblFungi:YLR154W-

Tissue	Identification	# of reads (stress)	# of reads (control)	Fold Change	p-value	Description
						C GeneID:1466405 KEGG:sce:YLR154W-C CYGD:YLR154w-c eggNOG:NOG79861 OMA:SCSKEHR OrthoDB:EOG466ZX8 NextBio:967146 Genevestigator:Q8TGM6 GermOnline:YLR154W-C Uniprot:Q8TGM6
Root	Isotig01027	194	21	3.079	1.67E-33	SGD|S000028422 - symbol:TAR1 "Mitochondrial protein potentially involved in respiratory metabolism" species:4932 "Saccharomyces cerevisiae" [GO:0003674 "molecular_function" evidence=ND] [GO:0043457 "regulation of cellular respiration" evidence=IGI] [GO:0005739 "mitochondrion" evidence=IEA;IMP] SGD:S000028422 GO:GO:0005739 EMBL:BK006945 EMBL:U53879 GO:GO:0043457 EMBL:Z73326 EMBL:AF479964 RefSeq:NP_690845.1 ProteinModelPortal:Q8TGM6 STRING:Q8TGM6 EnsemblFungi:YLR154W-C GeneID:1466405 KEGG:sce:YLR154W-C CYGD:YLR154w-c eggNOG:NOG79861 OMA:SCSKEHR OrthoDB:EOG466ZX8 NextBio:967146 Genevestigator:Q8TGM6 GermOnline:YLR154W-C Uniprot:Q8TGM6
Root	Isotig01028	84	7	3.456	7.08E-17	SGD|S000028422 - symbol:TAR1 "Mitochondrial protein potentially involved in respiratory metabolism" species:4932 "Saccharomyces cerevisiae" [GO:0003674 "molecular_function" evidence=ND] [GO:0043457 "regulation of cellular respiration" evidence=IGI] [GO:0005739 "mitochondrion" evidence=IEA;IMP] SGD:S000028422 GO:GO:0005739 EMBL:BK006945 EMBL:U53879 GO:GO:0043457 EMBL:Z73326 EMBL:AF479964 RefSeq:NP_690845.1 ProteinModelPortal:Q8TGM6 STRING:Q8TGM6 EnsemblFungi:YLR154W-C GeneID:1466405 KEGG:sce:YLR154W-C CYGD:YLR154w-c eggNOG:NOG79861 OMA:SCSKEHR OrthoDB:EOG466ZX8 NextBio:967146 Genevestigator:Q8TGM6 GermOnline:YLR154W-C Uniprot:Q8TGM6
Root	Isotig01041	19	50	-1.525	3.30E-05	TAIR|locus:2205235 - symbol:LOS1 "AT1G56070" species:3702 "Arabidopsis thaliana" [GO:0003746 "translation elongation factor activity" evidence=ISS] [GO:0008135 "translation factor activity, nucleic acid binding" evidence=ISS] [GO:0009409 "response to cold" evidence=IMP] [GO:0005730 "nucleolus" evidence=IDA] [GO:0005507 "copper ion binding" evidence=IDA] [GO:0005774 "vacuolar membrane" evidence=IDA] [GO:0016020 "membrane" evidence=IDA] [GO:0005886 "plasma membrane" evidence=IDA] [GO:0009507 "chloroplast" evidence=IDA] [GO:0005829 "cytosol" evidence=IDA] [GO:0009506 "plasmodesma" evidence=IDA] [GO:0005794 "Golgi apparatus" evidence=RCA] InterPro:IPR000640 InterPro:IPR000795 InterPro:IPR004161 InterPro:IPR005225 InterPro:IPR005517 Pfam:PF00009 Pfam:PF00679 Pfam:PF03144 Pfam:PF03764 PRINTS:PR00315 PROSITE:PS00301 SMART:SM00838 SMART:SM00889 GO:GO:0005525 TIGRFAMs:TIGR00231 EMBL:CP002684 GO:GO:0005829 GO:GO:0005886 GO:GO:0009506 GO:GO:0005774 GO:GO:0009507 GO:GO:0005730 GO:GO:0003924 GO:GO:0005507 GO:GO:0009409 GO:GO:0003746 InterPro:IPR020568 InterPro:IPR014721 Gene3D:G3DSA:3.30.230.10 SUPFAM:SSF54211 InterPro:IPR009000 SUPFAM:SSF50447 InterPro:IPR009022 Gene3D:G3DSA:3.30.70.240 SUPFAM:SSF54980 KO:K03234 OMA:GVMTQTE HSSP:P32324 EMBL:AF367331 EMBL:AY054461 EMBL:BT000661 EMBL:BT000722 EMBL:BT002714 IPI:IPI00517335 RefSeq:NP_849818.1 UniGene:At.47544 ProteinModelPortal:Q9ASR1 SMR:Q9ASR1 STRING:Q9ASR1 PRIDE:Q9ASR1 ProMEX:Q9ASR1 EnsemblPlants:AT1G56070.1 GeneID:842058 KEGG:ath:AT1G56070 TAIR:At1g56070 InParanoid:Q9ASR1 PhylomeDB:Q9ASR1 ProtClustDB:PLN00116 Genevestigator:Q9ASR1 Uniprot:Q9ASR1
Root	Isotig01100	9	53	-2.687	7.37E-10	TAIR|locus:2025327 - symbol:TLP1 "AT1G76900" species:3702 "Arabidopsis thaliana" [GO:0003700 "sequence-specific DNA binding transcription factor activity" evidence=ISS] [GO:0008081 "phosphoric diester hydrolase activity" evidence=ISS] [GO:0006355 "regulation of transcription, DNA-dependent" evidence=TAS] InterPro:IPR001810 Pfam:PF00646 PROSITE:PS50181 INTERPRO:IPR000007 EMBL:CP002684 GenomeReviews:CT485782_GR GO:GO:0006355 EMBL:AC002291 EMBL:AC079283 SUPFAM:SSF54518 HSSP:O00294 eggNOG:NOG286778 HOGENOM:HBG607533 InterPro:IPR018066 Gene3D:G3DSA:3.20.90.10 Pfam:PF01167 PRINTS:PR01573 PROSITE:PS01200 PROSITE:PS01201 EMBL:AF487267 EMBL:AY139758 EMBL:BT003039 IPI:IPI00531897 PIR:H96797 RefSeq:NP_177816.1 RefSeq:NP_849894.1 UniGene:At.17482 ProteinModelPortal:Q9ZP59 SMR:Q9ZP59 IntAct:Q9ZP59 PRIDE:Q9ZP59 EnsemblPlants:AT1G76900.1 EnsemblPlants:AT1G76900.2 GeneID:844025 KEGG:ath:AT1G76900 TAIR:At1g76900 InParanoid:Q9ZP59 OMA:QDSHEEQ PhylomeDB:Q9ZP59 ProtClustDB:CLSN2682949 Genevestigator:Q9ZP59 Uniprot:Q9ZP59
Root	Isotig01101	9	52	-2.659	1.31E-09	TAIR|locus:2025327 - symbol:TLP1 "AT1G76900" species:3702 "Arabidopsis thaliana" [GO:0003700 "sequence-specific DNA binding transcription factor activity" evidence=ISS] [GO:0008081 "phosphoric diester hydrolase activity" evidence=ISS] [GO:0006355 "regulation of transcription, DNA-dependent" evidence=TAS] InterPro:IPR001810 Pfam:PF00646 PROSITE:PS50181 INTERPRO:IPR000007 EMBL:CP002684 GenomeReviews:CT485782_GR GO:GO:0006355 EMBL:AC002291 EMBL:AC079283 SUPFAM:SSF54518 HSSP:O00294 eggNOG:NOG286778 HOGENOM:HBG607533 InterPro:IPR018066 Gene3D:G3DSA:3.20.90.10 Pfam:PF01167 PRINTS:PR01573 PROSITE:PS01200 PROSITE:PS01201 EMBL:AF487267 EMBL:AY139758 EMBL:BT003039 IPI:IPI00531897

Tissue	Identification	# of reads (stress)	# of reads (control)	Fold Change	p-value	Description
						PIR:H96797 RefSeq:NP_177816.1 RefSeq:NP_849894.1 UniGene:At.17482 ProteinModelPortal:Q9ZP59 SMR:Q9ZP59 IntAct:Q9ZP59 PRIDE:Q9ZP59 EnsemblPlants:AT1G76900.1 EnsemblPlants:AT1G76900.2 GeneID:844025 KEGG:ath:AT1G76900 TAIR:At1g76900 InParanoid:Q9ZP59 OMA:QDSHEEQ PhylomeDB:Q9ZP59 ProtClustDB:CLSN2682949 Genevestigator:Q9ZP59 Uniprot:Q9ZP59
Root	Isotig01102	9	53	-2.687	7.37E-10	TAIR|locus:2025327 - symbol:TLP1 "AT1G76900" species:3702 "Arabidopsis thaliana" [GO:0003700 "sequence-specific DNA binding transcription factor activity" evidence=ISS] [GO:0008081 "phosphoric diester hydrolase activity" evidence=ISS] [GO:0006355 "regulation of transcription, DNA-dependent" evidence=TAS] InterPro:IPR001810 Pfam:PF00646 PROSITE:PS50181 INTERPRO:IPR000007 EMBL:CP002684 GenomeReviews:CT485782_GR GO:GO:0006355 EMBL:AC002291 EMBL:AC079283 SUPFAM:SSF54518 HSSP:O00294 eggNOG:NOG286778 HOGENOM:HBG607533 InterPro:IPR018066 Gene3D:G3DSA:3.20.90.10 Pfam:PF01167 PRINTS:PR01573 PROSITE:PS01200 PROSITE:PS01201 EMBL:AF487267 EMBL:AY139758 EMBL:BT003039 IPI:IPI00531897 PIR:H96797 RefSeq:NP_177816.1 RefSeq:NP_849894.1 UniGene:At.17482 ProteinModelPortal:Q9ZP59 SMR:Q9ZP59 IntAct:Q9ZP59 PRIDE:Q9ZP59 EnsemblPlants:AT1G76900.1 EnsemblPlants:AT1G76900.2 GeneID:844025 KEGG:ath:AT1G76900 TAIR:At1g76900 InParanoid:Q9ZP59 OMA:QDSHEEQ PhylomeDB:Q9ZP59 ProtClustDB:CLSN2682949 Genevestigator:Q9ZP59 Uniprot:Q9ZP59
Root	Isotig01103	9	52	-2.659	1.31E-09	TAIR|locus:2025327 - symbol:TLP1 "AT1G76900" species:3702 "Arabidopsis thaliana" [GO:0003700 "sequence-specific DNA binding transcription factor activity" evidence=ISS] [GO:0008081 "phosphoric diester hydrolase activity" evidence=ISS] [GO:0006355 "regulation of transcription, DNA-dependent" evidence=TAS] InterPro:IPR001810 Pfam:PF00646 PROSITE:PS50181 INTERPRO:IPR000007 EMBL:CP002684 GenomeReviews:CT485782_GR GO:GO:0006355 EMBL:AC002291 EMBL:AC079283 SUPFAM:SSF54518 HSSP:O00294 eggNOG:NOG286778 HOGENOM:HBG607533 InterPro:IPR018066 Gene3D:G3DSA:3.20.90.10 Pfam:PF01167 PRINTS:PR01573 PROSITE:PS01200 PROSITE:PS01201 EMBL:AF487267 EMBL:AY139758 EMBL:BT003039 IPI:IPI00531897 PIR:H96797 RefSeq:NP_177816.1 RefSeq:NP_849894.1 UniGene:At.17482 ProteinModelPortal:Q9ZP59 SMR:Q9ZP59 IntAct:Q9ZP59 PRIDE:Q9ZP59 EnsemblPlants:AT1G76900.1 EnsemblPlants:AT1G76900.2 GeneID:844025 KEGG:ath:AT1G76900 TAIR:At1g76900 InParanoid:Q9ZP59 OMA:QDSHEEQ PhylomeDB:Q9ZP59 ProtClustDB:CLSN2682949 Genevestigator:Q9ZP59 Uniprot:Q9ZP59
Root	Isotig01104	9	52	-2.659	1.31E-09	TAIR|locus:2025327 - symbol:TLP1 "AT1G76900" species:3702 "Arabidopsis thaliana" [GO:0003700 "sequence-specific DNA binding transcription factor activity" evidence=ISS] [GO:0008081 "phosphoric diester hydrolase activity" evidence=ISS] [GO:0006355 "regulation of transcription, DNA-dependent" evidence=TAS] InterPro:IPR001810 Pfam:PF00646 PROSITE:PS50181 INTERPRO:IPR000007 EMBL:CP002684 GenomeReviews:CT485782_GR GO:GO:0006355 EMBL:AC002291 EMBL:AC079283 SUPFAM:SSF54518 HSSP:O00294 eggNOG:NOG286778 HOGENOM:HBG607533 InterPro:IPR018066 Gene3D:G3DSA:3.20.90.10 Pfam:PF01167 PRINTS:PR01573 PROSITE:PS01200 PROSITE:PS01201 EMBL:AF487267 EMBL:AY139758 EMBL:BT003039 IPI:IPI00531897 PIR:H96797 RefSeq:NP_177816.1 RefSeq:NP_849894.1 UniGene:At.17482 ProteinModelPortal:Q9ZP59 SMR:Q9ZP59 IntAct:Q9ZP59 PRIDE:Q9ZP59 EnsemblPlants:AT1G76900.1 EnsemblPlants:AT1G76900.2 GeneID:844025 KEGG:ath:AT1G76900 TAIR:At1g76900 InParanoid:Q9ZP59 OMA:QDSHEEQ PhylomeDB:Q9ZP59 ProtClustDB:CLSN2682949 Genevestigator:Q9ZP59 Uniprot:Q9ZP59
Root	Isotig01105	9	52	-2.659	1.31E-09	TAIR|locus:2025327 - symbol:TLP1 "AT1G76900" species:3702 "Arabidopsis thaliana" [GO:0003700 "sequence-specific DNA binding transcription factor activity" evidence=ISS] [GO:0008081 "phosphoric diester hydrolase activity" evidence=ISS] [GO:0006355 "regulation of transcription, DNA-dependent" evidence=TAS] InterPro:IPR001810 Pfam:PF00646 PROSITE:PS50181 INTERPRO:IPR000007 EMBL:CP002684 GenomeReviews:CT485782_GR GO:GO:0006355 EMBL:AC002291 EMBL:AC079283 SUPFAM:SSF54518 HSSP:O00294 eggNOG:NOG286778 HOGENOM:HBG607533 InterPro:IPR018066 Gene3D:G3DSA:3.20.90.10 Pfam:PF01167 PRINTS:PR01573 PROSITE:PS01200 PROSITE:PS01201 EMBL:AF487267 EMBL:AY139758 EMBL:BT003039 IPI:IPI00531897 PIR:H96797 RefSeq:NP_177816.1 RefSeq:NP_849894.1 UniGene:At.17482 ProteinModelPortal:Q9ZP59 SMR:Q9ZP59 IntAct:Q9ZP59 PRIDE:Q9ZP59 EnsemblPlants:AT1G76900.1 EnsemblPlants:AT1G76900.2 GeneID:844025 KEGG:ath:AT1G76900 TAIR:At1g76900 InParanoid:Q9ZP59 OMA:QDSHEEQ PhylomeDB:Q9ZP59 ProtClustDB:CLSN2682949 Genevestigator:Q9ZP59 Uniprot:Q9ZP59
Root	Isotig01106	9	52	-2.659	1.31E-09	TAIR|locus:2032950 - symbol:TLP10 "AT1G25280" species:3702 "Arabidopsis thaliana" [GO:0003700 "sequence-specific DNA binding transcription factor activity" evidence=ISS] [GO:0008081 "phosphoric diester hydrolase activity" evidence=ISS] [GO:0006355 "regulation of transcription, DNA-dependent" evidence=TAS] [GO:0005886 "plasma membrane" evidence=IDA] InterPro:IPR001810 Pfam:PF00646 PROSITE:PS50181 INTERPRO:IPR000007

Tissue	Identification	# of reads (stress)	# of reads (control)	Fold Change	p-value	Description
						EMBL:CP002684 GenomeReviews:CT485782_GR GO:GO:0005886 GO:GO:0005634 GO:GO:0006355 EMBL:AC079374 SUPFAM:SSF54518 EMBL:AF487271 EMBL:AY140459 EMBL:AY140460 EMBL:AY140461 EMBL:AY140462 EMBL:AY140463 EMBL:AY140464 EMBL:AY140465 EMBL:AY140466 EMBL:AY140467 EMBL:AY140468 EMBL:AY140469 EMBL:AK317199 EMBL:BT001997 EMBL:BT006290 IPI:IPI00539910 PIR:E86382 RefSeq:NP_001117353.1 RefSeq:NP_173899.1 RefSeq:NP_973909.1 UniGene:At.23717 HSSP:O00294 ProteinModelPortal:Q9FRH7 SMR:Q9FRH7 IntAct:Q9FRH7 STRING:Q9FRH7 PRIDE:Q9FRH7 EnsemblPlants:AT1G25280.1 GeneID:839112 KEGG:ath:AT1G25280 TAIR:At1g25280 eggNOG:NOG286778 HOGENOM:HBG607533 InParanoid:Q9FRH7 OMA:HSSACEL PhylomeDB:Q9FRH7 ProtClustDB:CLSN2681282 ArrayExpress:Q8H0W5 Genevestigator:Q9FRH7 InterPro:IPR018066 Gene3D:G3DSA:3.20.90.10 Pfam:PF01167 PRINTS:PR01573 PROSITE:PS01200 PROSITE:PS01201 Uniprot:Q9FRH7
Root	Isotig01107	9	51	-2.631	2.33E-09	TAIR|locus:2032950 - symbol:TLP10 "AT1G25280" species:3702 "Arabidopsis thaliana" [GO:0003700 "sequence-specific DNA binding transcription factor activity" evidence=ISS] [GO:0008081 "phosphoric diester hydrolase activity" evidence=ISS] [GO:0006355 "regulation of transcription, DNA-dependent" evidence=TAS] [GO:0005886 "plasma membrane" evidence=IDA] InterPro:IPR001810 Pfam:PF00646 PROSITE:PS50181 INTERPRO:IPR000007 EMBL:CP002684 GenomeReviews:CT485782_GR GO:GO:0005886 GO:GO:0005634 GO:GO:0006355 EMBL:AC079374 SUPFAM:SSF54518 EMBL:AF487271 EMBL:AY140459 EMBL:AY140460 EMBL:AY140461 EMBL:AY140462 EMBL:AY140463 EMBL:AY140464 EMBL:AY140465 EMBL:AY140466 EMBL:AY140467 EMBL:AY140468 EMBL:AY140469 EMBL:AK317199 EMBL:BT001997 EMBL:BT006290 IPI:IPI00539910 PIR:E86382 RefSeq:NP_001117353.1 RefSeq:NP_173899.1 RefSeq:NP_973909.1 UniGene:At.23717 HSSP:O00294 ProteinModelPortal:Q9FRH7 SMR:Q9FRH7 IntAct:Q9FRH7 STRING:Q9FRH7 PRIDE:Q9FRH7 EnsemblPlants:AT1G25280.1 GeneID:839112 KEGG:ath:AT1G25280 TAIR:At1g25280 eggNOG:NOG286778 HOGENOM:HBG607533 InParanoid:Q9FRH7 OMA:HSSACEL PhylomeDB:Q9FRH7 ProtClustDB:CLSN2681282 ArrayExpress:Q8H0W5 Genevestigator:Q9FRH7 InterPro:IPR018066 Gene3D:G3DSA:3.20.90.10 Pfam:PF01167 PRINTS:PR01573 PROSITE:PS01200 PROSITE:PS01201 Uniprot:Q9FRH7
Root	Isotig01108	9	52	-2.659	1.31E-09	TAIR|locus:2032950 - symbol:TLP10 "AT1G25280" species:3702 "Arabidopsis thaliana" [GO:0003700 "sequence-specific DNA binding transcription factor activity" evidence=ISS] [GO:0008081 "phosphoric diester hydrolase activity" evidence=ISS] [GO:0006355 "regulation of transcription, DNA-dependent" evidence=TAS] [GO:0005886 "plasma membrane" evidence=IDA] InterPro:IPR001810 Pfam:PF00646 PROSITE:PS50181 INTERPRO:IPR000007 EMBL:CP002684 GenomeReviews:CT485782_GR GO:GO:0005886 GO:GO:0005634 GO:GO:0006355 EMBL:AC079374 SUPFAM:SSF54518 EMBL:AF487271 EMBL:AY140459 EMBL:AY140460 EMBL:AY140461 EMBL:AY140462 EMBL:AY140463 EMBL:AY140464 EMBL:AY140465 EMBL:AY140466 EMBL:AY140467 EMBL:AY140468 EMBL:AY140469 EMBL:AK317199 EMBL:BT001997 EMBL:BT006290 IPI:IPI00539910 PIR:E86382 RefSeq:NP_001117353.1 RefSeq:NP_173899.1 RefSeq:NP_973909.1 UniGene:At.23717 HSSP:O00294 ProteinModelPortal:Q9FRH7 SMR:Q9FRH7 IntAct:Q9FRH7 STRING:Q9FRH7 PRIDE:Q9FRH7 EnsemblPlants:AT1G25280.1 GeneID:839112 KEGG:ath:AT1G25280 TAIR:At1g25280 eggNOG:NOG286778 HOGENOM:HBG607533 InParanoid:Q9FRH7 OMA:HSSACEL PhylomeDB:Q9FRH7 ProtClustDB:CLSN2681282 ArrayExpress:Q8H0W5 Genevestigator:Q9FRH7 InterPro:IPR018066 Gene3D:G3DSA:3.20.90.10 Pfam:PF01167 PRINTS:PR01573 PROSITE:PS01200 PROSITE:PS01201 Uniprot:Q9FRH7
Root	Isotig01109	9	51	-2.631	2.33E-09	TAIR|locus:2032950 - symbol:TLP10 "AT1G25280" species:3702 "Arabidopsis thaliana" [GO:0003700 "sequence-specific DNA binding transcription factor activity" evidence=ISS] [GO:0008081 "phosphoric diester hydrolase activity" evidence=ISS] [GO:0006355 "regulation of transcription, DNA-dependent" evidence=TAS] [GO:0005886 "plasma membrane" evidence=IDA] InterPro:IPR001810 Pfam:PF00646 PROSITE:PS50181 INTERPRO:IPR000007 EMBL:CP002684 GenomeReviews:CT485782_GR GO:GO:0005886 GO:GO:0005634 GO:GO:0006355 EMBL:AC079374 SUPFAM:SSF54518 EMBL:AF487271 EMBL:AY140459 EMBL:AY140460 EMBL:AY140461 EMBL:AY140462 EMBL:AY140463 EMBL:AY140464 EMBL:AY140465 EMBL:AY140466 EMBL:AY140467 EMBL:AY140468 EMBL:AY140469 EMBL:AK317199 EMBL:BT001997 EMBL:BT006290 IPI:IPI00539910 PIR:E86382 RefSeq:NP_001117353.1 RefSeq:NP_173899.1 RefSeq:NP_973909.1 UniGene:At.23717 HSSP:O00294 ProteinModelPortal:Q9FRH7 SMR:Q9FRH7 IntAct:Q9FRH7 STRING:Q9FRH7 PRIDE:Q9FRH7 EnsemblPlants:AT1G25280.1 GeneID:839112 KEGG:ath:AT1G25280 TAIR:At1g25280 eggNOG:NOG286778 HOGENOM:HBG607533 InParanoid:Q9FRH7 OMA:HSSACEL PhylomeDB:Q9FRH7 ProtClustDB:CLSN2681282 ArrayExpress:Q8H0W5 Genevestigator:Q9FRH7 InterPro:IPR018066 Gene3D:G3DSA:3.20.90.10 Pfam:PF01167 PRINTS:PR01573 PROSITE:PS01200 PROSITE:PS01201 Uniprot:Q9FRH7

Tissue	Identification	# of reads (stress)	# of reads (control)	Fold Change	p-value	Description
Root	Isotig01110	9	51	-2.631	2.33E-09	TAIR|locus:2032950 - symbol:TLP10 "AT1G25280" species:3702 "Arabidopsis thaliana" [GO:0003700 "sequence-specific DNA binding transcription factor activity" evidence=ISS] [GO:0008081 "phosphoric diester hydrolase activity" evidence=ISS] [GO:0006355 "regulation of transcription, DNA-dependent" evidence=TAS] [GO:0005886 "plasma membrane" evidence=IDA] InterPro:IPR001810 Pfam:PF00646 PROSITE:PS50181 INTERPRO:IPR000007 EMBL:CP002684 GenomeReviews:CT485782_GR GO:GO:0005886 GO:GO:0005634 GO:GO:0006355 EMBL:AC079374 SUPFAM:SSF54518 EMBL:AF487271 EMBL:AY140459 EMBL:AY140460 EMBL:AY140461 EMBL:AY140462 EMBL:AY140463 EMBL:AY140464 EMBL:AY140465 EMBL:AY140466 EMBL:AY140467 EMBL:AY140468 EMBL:AY140469 EMBL:AK317199 EMBL:BT001997 EMBL:BT006290 IPI:IPI00539910 PIR:E86382 RefSeq:NP_001117353.1 RefSeq:NP_173899.1 RefSeq:NP_973909.1 UniGene:At.23717 HSSP:O00294 ProteinModelPortal:Q9FRH7 SMR:Q9FRH7 IntAct:Q9FRH7 STRING:Q9FRH7 PRIDE:Q9FRH7 EnsemblPlants:AT1G25280.1 GeneID:839112 KEGG:ath:AT1G25280 TAIR:At1g25280 eggNOG:NOG286778 HOGENOM:HBG607533 InParanoid:Q9FRH7 OMA:HSSACEL PhylomeDB:Q9FRH7 ProtClustDB:CLSN2681282 ArrayExpress:Q8H0W5 Genevestigator:Q9FRH7 InterPro:IPR018066 Gene3D:G3DSA:3.20.90.10 Pfam:PF01167 PRINTS:PR01573 PROSITE:PS01200 PROSITE:PS01201 Uniprot:Q9FRH7
Root	Isotig01111	9	51	-2.631	2.33E-09	TAIR|locus:2032950 - symbol:TLP10 "AT1G25280" species:3702 "Arabidopsis thaliana" [GO:0003700 "sequence-specific DNA binding transcription factor activity" evidence=ISS] [GO:0008081 "phosphoric diester hydrolase activity" evidence=ISS] [GO:0006355 "regulation of transcription, DNA-dependent" evidence=TAS] [GO:0005886 "plasma membrane" evidence=IDA] InterPro:IPR001810 Pfam:PF00646 PROSITE:PS50181 INTERPRO:IPR000007 EMBL:CP002684 GenomeReviews:CT485782_GR GO:GO:0005886 GO:GO:0005634 GO:GO:0006355 EMBL:AC079374 SUPFAM:SSF54518 EMBL:AF487271 EMBL:AY140459 EMBL:AY140460 EMBL:AY140461 EMBL:AY140462 EMBL:AY140463 EMBL:AY140464 EMBL:AY140465 EMBL:AY140466 EMBL:AY140467 EMBL:AY140468 EMBL:AY140469 EMBL:AK317199 EMBL:BT001997 EMBL:BT006290 IPI:IPI00539910 PIR:E86382 RefSeq:NP_001117353.1 RefSeq:NP_173899.1 RefSeq:NP_973909.1 UniGene:At.23717 HSSP:O00294 ProteinModelPortal:Q9FRH7 SMR:Q9FRH7 IntAct:Q9FRH7 STRING:Q9FRH7 PRIDE:Q9FRH7 EnsemblPlants:AT1G25280.1 GeneID:839112 KEGG:ath:AT1G25280 TAIR:At1g25280 eggNOG:NOG286778 HOGENOM:HBG607533 InParanoid:Q9FRH7 OMA:HSSACEL PhylomeDB:Q9FRH7 ProtClustDB:CLSN2681282 ArrayExpress:Q8H0W5 Genevestigator:Q9FRH7 InterPro:IPR018066 Gene3D:G3DSA:3.20.90.10 Pfam:PF01167 PRINTS:PR01573 PROSITE:PS01200 PROSITE:PS01201 Uniprot:Q9FRH7
Root	Isotig01112	6	25	-2.188	0.000173532	No hit
Root	Isotig01114	5	21	-2.199	0.000555521	No hit
Root	Isotig01116	5	21	-2.199	0.000555521	No hit
Root	Isotig01118	5	22	-2.266	0.000318925	No hit
Root	Isotig01140	77	10	2.816	3.49E-13	TAIR|locus:2202175 - symbol:DA1 "AT1G19270" species:3702 "Arabidopsis thaliana" [GO:0043130 "ubiquitin binding" evidence=IDA;IPI] [GO:0046621 "negative regulation of organ growth" evidence=IMP] [GO:0048317 "seed morphogenesis" evidence=IMP] [GO:0048482 "ovule morphogenesis" evidence=IMP] Pfam:PF00412 InterPro:IPR001781 PROSITE:PS00142 PROSITE:PS00478 PROSITE:PS50023 SMART:SM00132 EMBL:CP002684 GenomeReviews:CT485782_GR GO:GO:0008270 Gene3D:G3DSA:2.10.110.10 InterPro:IPR003903 SMART:SM00726 PROSITE:PS50330 EMBL:AC069143 GO:GO:0043130 GO:GO:0046621 EMBL:BX816571 IPI:IPI00540867 PIR:D86326 RefSeq:NP_173361.1 UniGene:At.21826 ProteinModelPortal:P0C7Q8 SMR:P0C7Q8 STRING:P0C7Q8 PRIDE:P0C7Q8 EnsemblPlants:AT1G19270.1 GeneID:838510 KEGG:ath:AT1G19270 TAIR:At1g19270 eggNOG:NOG312886 HOGENOM:HBG318792 OMA:YHPKCDV PhylomeDB:P0C7Q8 ProtClustDB:CLSN2914207 Genevestigator:P0C7Q8 GO:GO:0048482 GO:GO:0048317 InterPro:IPR022087 Pfam:PF12315 Uniprot:P0C7Q8
Root	Isotig01141	71	7	3.213	1.00E-13	No hit
Root	Isotig01142	70	7	3.193	1.75E-13	No hit
Root	Isotig01144	48	4	3.456	2.82E-10	TAIR|locus:2056799 - symbol:PI4K GAMMA 7 "AT2G03890" species:3702 "Arabidopsis thaliana" [GO:0005575 "cellular_component" evidence=ND] [GO:0004430 "1-phosphatidylinositol 4-kinase activity" evidence=IDA] [GO:0046854 "phosphatidylinositol phosphorylation" evidence=IDA] InterPro:IPR000403 Pfam:PF00454 EMBL:CP002685 GenomeReviews:CT485783_GR GO:GO:0004430 HOGENOM:HBG603679 EMBL:AC007196 ProtClustDB:CLSN2687794 EMBL:AY056101 EMBL:BT002696 IPI:IPI00523971 PIR:D84453 RefSeq:NP_565307.1 UniGene:At.24594 ProteinModelPortal:Q9SI52 STRING:Q9SI52 PRIDE:Q9SI52 EnsemblPlants:AT2G03890.1

Tissue	Identification	# of reads (stress)	# of reads (control)	Fold Change	p-value	Description
						GeneID:814915 KEGG:ath:AT2G03890 TAIR:At2g03890 InParanoid:Q9SI52 OMA:MAVAVFK PhylomeDB:Q9SI52 ArrayExpress:Q9SI52 Genevestigator:Q9SI52 Uniprot:Q9SI52
Root	Isotig01145	48	4	3.456	2.82E-10	TAIR|locus:2056799 - symbol:PI4K GAMMA 7 "AT2G03890" species:3702 "Arabidopsis thaliana" [GO:0005575 "cellular_component" evidence=ND] [GO:0004430 "1-phosphatidylinositol 4-kinase activity" evidence=IDA] [GO:0046854 "phosphatidylinositol phosphorylation" evidence=IDA] InterPro:IPR000403 Pfam:PF00454 EMBL:CP002685 GenomeReviews:CT485783_GR GO:GO:0004430 HOGENOM:HBG603679 EMBL:AC007196 ProtClustDB:CLSN2687794 EMBL:AY056101 EMBL:BT002696 IPI:IPI00523971 PIR:D84453 RefSeq:NP_565307.1 UniGene:At.24594 ProteinModelPortal:Q9SI52 STRING:Q9SI52 PRIDE:Q9SI52 EnsemblPlants:AT2G03890.1 GeneID:814915 KEGG:ath:AT2G03890 TAIR:At2g03890 InParanoid:Q9SI52 OMA:MAVAVFK PhylomeDB:Q9SI52 ArrayExpress:Q9SI52 Genevestigator:Q9SI52 Uniprot:Q9SI52
Root	Isotig01146	59	13	2.053	1.17E-07	TAIR|locus:2056799 - symbol:PI4K GAMMA 7 "AT2G03890" species:3702 "Arabidopsis thaliana" [GO:0005575 "cellular_component" evidence=ND] [GO:0004430 "1-phosphatidylinositol 4-kinase activity" evidence=IDA] [GO:0046854 "phosphatidylinositol phosphorylation" evidence=IDA] InterPro:IPR000403 Pfam:PF00454 EMBL:CP002685 GenomeReviews:CT485783_GR GO:GO:0004430 HOGENOM:HBG603679 EMBL:AC007196 ProtClustDB:CLSN2687794 EMBL:AY056101 EMBL:BT002696 IPI:IPI00523971 PIR:D84453 RefSeq:NP_565307.1 UniGene:At.24594 ProteinModelPortal:Q9SI52 STRING:Q9SI52 PRIDE:Q9SI52 EnsemblPlants:AT2G03890.1 GeneID:814915 KEGG:ath:AT2G03890 TAIR:At2g03890 InParanoid:Q9SI52 OMA:MAVAVFK PhylomeDB:Q9SI52 ArrayExpress:Q9SI52 Genevestigator:Q9SI52 Uniprot:Q9SI52
Root	Isotig01147	54	13	1.925	1.17E-06	TAIR|locus:2056799 - symbol:PI4K GAMMA 7 "AT2G03890" species:3702 "Arabidopsis thaliana" [GO:0005575 "cellular_component" evidence=ND] [GO:0004430 "1-phosphatidylinositol 4-kinase activity" evidence=IDA] [GO:0046854 "phosphatidylinositol phosphorylation" evidence=IDA] InterPro:IPR000403 Pfam:PF00454 EMBL:CP002685 GenomeReviews:CT485783_GR GO:GO:0004430 HOGENOM:HBG603679 EMBL:AC007196 ProtClustDB:CLSN2687794 EMBL:AY056101 EMBL:BT002696 IPI:IPI00523971 PIR:D84453 RefSeq:NP_565307.1 UniGene:At.24594 ProteinModelPortal:Q9SI52 STRING:Q9SI52 PRIDE:Q9SI52 EnsemblPlants:AT2G03890.1 GeneID:814915 KEGG:ath:AT2G03890 TAIR:At2g03890 InParanoid:Q9SI52 OMA:MAVAVFK PhylomeDB:Q9SI52 ArrayExpress:Q9SI52 Genevestigator:Q9SI52 Uniprot:Q9SI52
Root	Isotig01148	59	13	2.053	1.17E-07	TAIR|locus:2056799 - symbol:PI4K GAMMA 7 "AT2G03890" species:3702 "Arabidopsis thaliana" [GO:0005575 "cellular_component" evidence=ND] [GO:0004430 "1-phosphatidylinositol 4-kinase activity" evidence=IDA] [GO:0046854 "phosphatidylinositol phosphorylation" evidence=IDA] InterPro:IPR000403 Pfam:PF00454 EMBL:CP002685 GenomeReviews:CT485783_GR GO:GO:0004430 HOGENOM:HBG603679 EMBL:AC007196 ProtClustDB:CLSN2687794 EMBL:AY056101 EMBL:BT002696 IPI:IPI00523971 PIR:D84453 RefSeq:NP_565307.1 UniGene:At.24594 ProteinModelPortal:Q9SI52 STRING:Q9SI52 PRIDE:Q9SI52 EnsemblPlants:AT2G03890.1 GeneID:814915 KEGG:ath:AT2G03890 TAIR:At2g03890 InParanoid:Q9SI52 OMA:MAVAVFK PhylomeDB:Q9SI52 ArrayExpress:Q9SI52 Genevestigator:Q9SI52 Uniprot:Q9SI52
Root	Isotig01149	54	13	1.925	1.17E-06	TAIR|locus:2056799 - symbol:PI4K GAMMA 7 "AT2G03890" species:3702 "Arabidopsis thaliana" [GO:0005575 "cellular_component" evidence=ND] [GO:0004430 "1-phosphatidylinositol 4-kinase activity" evidence=IDA] [GO:0046854 "phosphatidylinositol phosphorylation" evidence=IDA] InterPro:IPR000403 Pfam:PF00454 EMBL:CP002685 GenomeReviews:CT485783_GR GO:GO:0004430 HOGENOM:HBG603679 EMBL:AC007196 ProtClustDB:CLSN2687794 EMBL:AY056101 EMBL:BT002696 IPI:IPI00523971 PIR:D84453 RefSeq:NP_565307.1 UniGene:At.24594 ProteinModelPortal:Q9SI52 STRING:Q9SI52 PRIDE:Q9SI52 EnsemblPlants:AT2G03890.1 GeneID:814915 KEGG:ath:AT2G03890 TAIR:At2g03890 InParanoid:Q9SI52 OMA:MAVAVFK PhylomeDB:Q9SI52 ArrayExpress:Q9SI52 Genevestigator:Q9SI52 Uniprot:Q9SI52
Root	Isotig01150	59	13	2.053	1.17E-07	TAIR|locus:2056799 - symbol:PI4K GAMMA 7 "AT2G03890" species:3702 "Arabidopsis thaliana" [GO:0005575 "cellular_component" evidence=ND] [GO:0004430 "1-phosphatidylinositol 4-kinase activity" evidence=IDA] [GO:0046854 "phosphatidylinositol phosphorylation" evidence=IDA] InterPro:IPR000403 Pfam:PF00454 EMBL:CP002685 GenomeReviews:CT485783_GR GO:GO:0004430 HOGENOM:HBG603679 EMBL:AC007196 ProtClustDB:CLSN2687794 EMBL:AY056101 EMBL:BT002696 IPI:IPI00523971 PIR:D84453 RefSeq:NP_565307.1 UniGene:At.24594 ProteinModelPortal:Q9SI52 STRING:Q9SI52 PRIDE:Q9SI52 EnsemblPlants:AT2G03890.1 GeneID:814915 KEGG:ath:AT2G03890 TAIR:At2g03890 InParanoid:Q9SI52 OMA:MAVAVFK PhylomeDB:Q9SI52 ArrayExpress:Q9SI52 Genevestigator:Q9SI52 Uniprot:Q9SI52
Root	Isotig01151	54	13	1.925	1.17E-06	TAIR|locus:2056799 - symbol:PI4K GAMMA 7 "AT2G03890" species:3702 "Arabidopsis thaliana" [GO:0005575 "cellular_component" evidence=ND] [GO:0004430 "1-phosphatidylinositol 4-kinase activity" evidence=IDA]

Tissue	Identification	# of reads (stress)	# of reads (control)	Fold Change	p-value	Description
						[GO:0046854 "phosphatidylinositol phosphorylation" evidence=IDA] InterPro:IPR000403 Pfam:PF00454 EMBL:CP002685 GenomeReviews:CT485783_GR GO:GO:0004430 HOGENOM:HBG603679 EMBL:AC007196 ProtClustDB:CLSN2687794 EMBL:AY056101 EMBL:BT002696 IPI:IPI00523971 PIR:D84453 RefSeq:NP_565307.1 UniGene:At.24594 ProteinModelPortal:Q9SI52 STRING:Q9SI52 PRIDE:Q9SI52 EnsemblPlants:AT2G03890.1 GeneID:814915 KEGG:ath:AT2G03890 TAIR:At2g03890 InParanoid:Q9SI52 OMA:MAVAVFK PhylomeDB:Q9SI52 ArrayExpress:Q9SI52 Genevestigator:Q9SI52 Uniprot:Q9SI52
Root	Isotig01152	59	13	2.053	1.17E-07	TAIR|locus:2056799 - symbol:PI4K GAMMA 7 "AT2G03890" species:3702 "Arabidopsis thaliana" [GO:0005575 "cellular_component" evidence=ND] [GO:0004430 "1-phosphatidylinositol 4-kinase activity" evidence=IDA] [GO:0046854 "phosphatidylinositol phosphorylation" evidence=IDA] InterPro:IPR000403 Pfam:PF00454 EMBL:CP002685 GenomeReviews:CT485783_GR GO:GO:0004430 HOGENOM:HBG603679 EMBL:AC007196 ProtClustDB:CLSN2687794 EMBL:AY056101 EMBL:BT002696 IPI:IPI00523971 PIR:D84453 RefSeq:NP_565307.1 UniGene:At.24594 ProteinModelPortal:Q9SI52 STRING:Q9SI52 PRIDE:Q9SI52 EnsemblPlants:AT2G03890.1 GeneID:814915 KEGG:ath:AT2G03890 TAIR:At2g03890 InParanoid:Q9SI52 OMA:MAVAVFK PhylomeDB:Q9SI52 ArrayExpress:Q9SI52 Genevestigator:Q9SI52 Uniprot:Q9SI52
Root	Isotig01153	54	13	1.925	1.17E-06	TAIR|locus:2056799 - symbol:PI4K GAMMA 7 "AT2G03890" species:3702 "Arabidopsis thaliana" [GO:0005575 "cellular_component" evidence=ND] [GO:0004430 "1-phosphatidylinositol 4-kinase activity" evidence=IDA] [GO:0046854 "phosphatidylinositol phosphorylation" evidence=IDA] InterPro:IPR000403 Pfam:PF00454 EMBL:CP002685 GenomeReviews:CT485783_GR GO:GO:0004430 HOGENOM:HBG603679 EMBL:AC007196 ProtClustDB:CLSN2687794 EMBL:AY056101 EMBL:BT002696 IPI:IPI00523971 PIR:D84453 RefSeq:NP_565307.1 UniGene:At.24594 ProteinModelPortal:Q9SI52 STRING:Q9SI52 PRIDE:Q9SI52 EnsemblPlants:AT2G03890.1 GeneID:814915 KEGG:ath:AT2G03890 TAIR:At2g03890 InParanoid:Q9SI52 OMA:MAVAVFK PhylomeDB:Q9SI52 ArrayExpress:Q9SI52 Genevestigator:Q9SI52 Uniprot:Q9SI52
Root	Isotig01154	44	15	1.424	0.000402568	TAIR|locus:2043560 - symbol:PAPS2 "AT2G25850" species:3702 "Arabidopsis thaliana" [GO:0016779 "nucleotidyltransferase activity" evidence=ISS] [GO:0005515 "protein binding" evidence=IPI] [GO:0005634 "nucleus" evidence=IDA] [GO:0043631 "RNA polyadenylation" evidence=IDA] InterPro:IPR002934 InterPro:IPR007010 InterPro:IPR007012 InterPro:IPR011068 InterPro:IPR014492 Pfam:PF01909 Pfam:PF04926 Pfam:PF04928 PIRSF:PIRSF018425 GO:GO:0005634 EMBL:CP002685 GenomeReviews:CT485783_GR GO:GO:0005515 GO:GO:0006351 GO:GO:0003723 EMBL:AC005395 GO:GO:0004652 GO:GO:0043631 eggNOG:COG5186 KO:K14376 Gene3D:G3DSA:3.30.70.590 SUPFAM:SSF55003 HOGENOM:HBG594063 HSSP:P25500 EMBL:AF255297 EMBL:AY039974 EMBL:AY074533 IPI:IPI00518420 PIR:E84653 RefSeq:NP_565611.1 UniGene:At.14867 ProteinModelPortal:O82312 SMR:O82312 IntAct:O82312 STRING:O82312 PRIDE:O82312 EnsemblPlants:AT2G25850.2 GeneID:817127 KEGG:ath:AT2G25850 TAIR:At2g25850 InParanoid:O82312 OMA:MGSESIN PhylomeDB:O82312 ProtClustDB:CLSN2688428 BRENDA:2.7.7.19 ArrayExpress:O82312 Genevestigator:O82312 Uniprot:O82312
Root	Isotig01155	44	15	1.424	0.000402568	TAIR|locus:2043560 - symbol:PAPS2 "AT2G25850" species:3702 "Arabidopsis thaliana" [GO:0016779 "nucleotidyltransferase activity" evidence=ISS] [GO:0005515 "protein binding" evidence=IPI] [GO:0005634 "nucleus" evidence=IDA] [GO:0043631 "RNA polyadenylation" evidence=IDA] InterPro:IPR002934 InterPro:IPR007010 InterPro:IPR007012 InterPro:IPR011068 InterPro:IPR014492 Pfam:PF01909 Pfam:PF04926 Pfam:PF04928 PIRSF:PIRSF018425 GO:GO:0005634 EMBL:CP002685 GenomeReviews:CT485783_GR GO:GO:0005515 GO:GO:0006351 GO:GO:0003723 EMBL:AC005395 GO:GO:0004652 GO:GO:0043631 eggNOG:COG5186 KO:K14376 Gene3D:G3DSA:3.30.70.590 SUPFAM:SSF55003 HOGENOM:HBG594063 HSSP:P25500 EMBL:AF255297 EMBL:AY039974 EMBL:AY074533 IPI:IPI00518420 PIR:E84653 RefSeq:NP_565611.1 UniGene:At.14867 ProteinModelPortal:O82312 SMR:O82312 IntAct:O82312 STRING:O82312 PRIDE:O82312 EnsemblPlants:AT2G25850.2 GeneID:817127 KEGG:ath:AT2G25850 TAIR:At2g25850 InParanoid:O82312 OMA:MGSESIN PhylomeDB:O82312 ProtClustDB:CLSN2688428 BRENDA:2.7.7.19 ArrayExpress:O82312 Genevestigator:O82312 Uniprot:O82312
Root	Isotig01156	44	15	1.424	0.000402568	TAIR|locus:2043560 - symbol:PAPS2 "AT2G25850" species:3702 "Arabidopsis thaliana" [GO:0016779 "nucleotidyltransferase activity" evidence=ISS] [GO:0005515 "protein binding" evidence=IPI] [GO:0005634 "nucleus" evidence=IDA] [GO:0043631 "RNA polyadenylation" evidence=IDA] InterPro:IPR002934 InterPro:IPR007010 InterPro:IPR007012 InterPro:IPR011068 InterPro:IPR014492 Pfam:PF01909 Pfam:PF04926 Pfam:PF04928 PIRSF:PIRSF018425 GO:GO:0005634 EMBL:CP002685 GenomeReviews:CT485783_GR GO:GO:0005515 GO:GO:0006351 GO:GO:0003723 EMBL:AC005395 GO:GO:0004652 GO:GO:0043631 eggNOG:COG5186

Tissue	Identification	# of reads (stress)	# of reads (control)	Fold Change	p-value	Description
						KO:K14376 Gene3D:G3DSA:3.30.70.590 SUPFAM:SSF55003 HOGENOM:HBG594063 HSSP:P25500 EMBL:AF255297 EMBL:AY039974 EMBL:AY074533 IPI:IPI00518420 PIR:E84653 RefSeq:NP_565611.1 UniGene:At.14867 ProteinModelPortal:O82312 SMR:O82312 IntAct:O82312 STRING:O82312 PRIDE:O82312 EnsemblPlants:AT2G25850.2 GeneID:817127 KEGG:ath:AT2G25850 TAIR:At2g25850 InParanoid:O82312 OMA:MGSESIN PhylomeDB:O82312 ProtClustDB:CLSN2688428 BRENDA:2.7.7.19 ArrayExpress:O82312 Genevestigator:O82312 Uniprot:O82312
Root	Isotig01157	44	15	1.424	0.000402568	TAIR|locus:2043560 - symbol:PAPS2 "AT2G25850" species:3702 "Arabidopsis thaliana" [GO:0016779 "nucleotidyltransferase activity" evidence=ISS] [GO:0005515 "protein binding" evidence=IPI] [GO:0005634 "nucleus" evidence=IDA] [GO:0043631 "RNA polyadenylation" evidence=IDA] InterPro:IPR002934 InterPro:IPR007010 InterPro:IPR007012 InterPro:IPR011068 InterPro:IPR014492 Pfam:PF01909 Pfam:PF04926 Pfam:PF04928 PIRSF:PIRSF018425 GO:GO:0005634 EMBL:CP002685 GenomeReviews:CT485783_GR GO:GO:0005515 GO:GO:0006351 GO:GO:0003723 EMBL:AC005395 GO:GO:0004652 GO:GO:0043631 eggNOG:COG5186 KO:K14376 Gene3D:G3DSA:3.30.70.590 SUPFAM:SSF55003 HOGENOM:HBG594063 HSSP:P25500 EMBL:AF255297 EMBL:AY039974 EMBL:AY074533 IPI:IPI00518420 PIR:E84653 RefSeq:NP_565611.1 UniGene:At.14867 ProteinModelPortal:O82312 SMR:O82312 IntAct:O82312 STRING:O82312 PRIDE:O82312 EnsemblPlants:AT2G25850.2 GeneID:817127 KEGG:ath:AT2G25850 TAIR:At2g25850 InParanoid:O82312 OMA:MGSESIN PhylomeDB:O82312 ProtClustDB:CLSN2688428 BRENDA:2.7.7.19 ArrayExpress:O82312 Genevestigator:O82312 Uniprot:O82312
Root	Isotig01160	18	2	3.041	0.000259481	TAIR|locus:2030943 - symbol:PAPS1 "AT1G17980" species:3702 "Arabidopsis thaliana" [GO:0016779 "nucleotidyltransferase activity" evidence=ISS] [GO:0005515 "protein binding" evidence=IPI] [GO:0005634 "nucleus" evidence=IDA] [GO:0043631 "RNA polyadenylation" evidence=IDA] InterPro:IPR002934 InterPro:IPR007010 InterPro:IPR007012 InterPro:IPR011068 InterPro:IPR014492 Pfam:PF01909 Pfam:PF04926 Pfam:PF04928 PIRSF:PIRSF018425 EMBL:CP002684 GenomeReviews:CT485782_GR GO:GO:0005634 GO:GO:0005515 GO:GO:0006351 EMBL:AC034106 GO:GO:0003723 GO:GO:0004652 GO:GO:0043631 UniGene:At.26787 UniGene:At.73166 eggNOG:COG5186 KO:K14376 Gene3D:G3DSA:3.30.70.590 SUPFAM:SSF55003 HSSP:P25500 BRENDA:2.7.7.19 EMBL:BT005990 EMBL:AY323906 EMBL:AK227446 IPI:IPI00540119 PIR:B86315 RefSeq:NP_173240.2 ProteinModelPortal:Q9LMT2 SMR:Q9LMT2 IntAct:Q9LMT2 STRING:Q9LMT2 PRIDE:Q9LMT2 EnsemblPlants:AT1G17980.1 GeneID:838378 KEGG:ath:AT1G17980 TAIR:At1g17980 InParanoid:Q9LMT2 OMA:WDTLFEP PhylomeDB:Q9LMT2 ProtClustDB:CLSN2680711 Genevestigator:Q9LMT2 Uniprot:Q9LMT2
Root	Isotig01161	18	2	3.041	0.000259481	TAIR|locus:2030943 - symbol:PAPS1 "AT1G17980" species:3702 "Arabidopsis thaliana" [GO:0016779 "nucleotidyltransferase activity" evidence=ISS] [GO:0005515 "protein binding" evidence=IPI] [GO:0005634 "nucleus" evidence=IDA] [GO:0043631 "RNA polyadenylation" evidence=IDA] InterPro:IPR002934 InterPro:IPR007010 InterPro:IPR007012 InterPro:IPR011068 InterPro:IPR014492 Pfam:PF01909 Pfam:PF04926 Pfam:PF04928 PIRSF:PIRSF018425 EMBL:CP002684 GenomeReviews:CT485782_GR GO:GO:0005634 GO:GO:0005515 GO:GO:0006351 EMBL:AC034106 GO:GO:0003723 GO:GO:0004652 GO:GO:0043631 UniGene:At.26787 UniGene:At.73166 eggNOG:COG5186 KO:K14376 Gene3D:G3DSA:3.30.70.590 SUPFAM:SSF55003 HSSP:P25500 BRENDA:2.7.7.19 EMBL:BT005990 EMBL:AY323906 EMBL:AK227446 IPI:IPI00540119 PIR:B86315 RefSeq:NP_173240.2 ProteinModelPortal:Q9LMT2 SMR:Q9LMT2 IntAct:Q9LMT2 STRING:Q9LMT2 PRIDE:Q9LMT2 EnsemblPlants:AT1G17980.1 GeneID:838378 KEGG:ath:AT1G17980 TAIR:At1g17980 InParanoid:Q9LMT2 OMA:WDTLFEP PhylomeDB:Q9LMT2 ProtClustDB:CLSN2680711 Genevestigator:Q9LMT2 Uniprot:Q9LMT2
Root	Isotig01172	149	74	0.881	9.59E-06	TAIR|locus:2064965 - symbol:EULS3 "AT2G39050" species:3702 "Arabidopsis thaliana" [GO:0008150 "biological_process" evidence=ND] [GO:0005634 "nucleus" evidence=IDA] [GO:0005737 "cytoplasm" evidence=IDA] [GO:0030246 "carbohydrate binding" evidence=IDA] GO:GO:0005634 GO:GO:0005737 EMBL:CP002685 GenomeReviews:CT485783_GR InterPro:IPR000772 SUPFAM:SSF50370 PROSITE:PS50231 GO:GO:0030246 EMBL:AC005770 Pfam:PF14200 EMBL:AF411801 EMBL:AY093795 EMBL:AY087909 IPI:IPI00536641 PIR:E84812 RefSeq:NP_565899.1 UniGene:At.22599 ProteinModelPortal:Q945P1 SMR:Q945P1 PRIDE:Q945P1 EnsemblPlants:AT2G39050.1 GeneID:818491 KEGG:ath:AT2G39050 TAIR:At2g39050 eggNOG:NOG254161 HOGENOM:HBG750017 InParanoid:Q945P1 OMA:GYRTIRM PhylomeDB:Q945P1 ProtClustDB:CLSN2681741 ArrayExpress:Q945P1 Genevestigator:Q945P1 Uniprot:Q945P1

Tissue	Identification	# of reads (stress)	# of reads (control)	Fold Change	p-value	Description
Root	Isotig01173	150	74	0.890	7.38E-06	TAIR|locus:2064965 - symbol:EULS3 "AT2G39050" species:3702 "Arabidopsis thaliana" [GO:0008150 "biological_process" evidence=ND] [GO:0005634 "nucleus" evidence=IDA] [GO:0005737 "cytoplasm" evidence=IDA] [GO:0030246 "carbohydrate binding" evidence=IDA] GO:GO:0005634 GO:GO:0005737 EMBL:CP002685 GenomeReviews:CT485783_GR InterPro:IPR000772 SUPFAM:SSF50370 PROSITE:PS50231 GO:GO:0030246 EMBL:AC005770 Pfam:PF14200 EMBL:AF411801 EMBL:AY093795 EMBL:AY087909 IPI:IPI00536641 PIR:E84812 RefSeq:NP_565899.1 UniGene:At.22599 ProteinModelPortal:Q945P1 SMR:Q945P1 PRIDE:Q945P1 EnsemblPlants:AT2G39050.1 GeneID:818491 KEGG:ath:AT2G39050 TAIR:At2g39050 eggNOG:NOG254161 HOGENOM:HBG750017 InParanoid:Q945P1 OMA:GYRTIRM PhylomeDB:Q945P1 ProtClustDB:CLSN2681741 ArrayExpress:Q945P1 Genevestigator:Q945P1 Uniprot:Q945P1
Root	Isotig01174	145	70	0.922	5.71E-06	TAIR|locus:2064965 - symbol:EULS3 "AT2G39050" species:3702 "Arabidopsis thaliana" [GO:0008150 "biological_process" evidence=ND] [GO:0005634 "nucleus" evidence=IDA] [GO:0005737 "cytoplasm" evidence=IDA] [GO:0030246 "carbohydrate binding" evidence=IDA] GO:GO:0005634 GO:GO:0005737 EMBL:CP002685 GenomeReviews:CT485783_GR InterPro:IPR000772 SUPFAM:SSF50370 PROSITE:PS50231 GO:GO:0030246 EMBL:AC005770 Pfam:PF14200 EMBL:AF411801 EMBL:AY093795 EMBL:AY087909 IPI:IPI00536641 PIR:E84812 RefSeq:NP_565899.1 UniGene:At.22599 ProteinModelPortal:Q945P1 SMR:Q945P1 PRIDE:Q945P1 EnsemblPlants:AT2G39050.1 GeneID:818491 KEGG:ath:AT2G39050 TAIR:At2g39050 eggNOG:NOG254161 HOGENOM:HBG750017 InParanoid:Q945P1 OMA:GYRTIRM PhylomeDB:Q945P1 ProtClustDB:CLSN2681741 ArrayExpress:Q945P1 Genevestigator:Q945P1 Uniprot:Q945P1
Root	Isotig01175	149	74	0.881	9.59E-06	TAIR|locus:2064965 - symbol:EULS3 "AT2G39050" species:3702 "Arabidopsis thaliana" [GO:0008150 "biological_process" evidence=ND] [GO:0005634 "nucleus" evidence=IDA] [GO:0005737 "cytoplasm" evidence=IDA] [GO:0030246 "carbohydrate binding" evidence=IDA] GO:GO:0005634 GO:GO:0005737 EMBL:CP002685 GenomeReviews:CT485783_GR InterPro:IPR000772 SUPFAM:SSF50370 PROSITE:PS50231 GO:GO:0030246 EMBL:AC005770 Pfam:PF14200 EMBL:AF411801 EMBL:AY093795 EMBL:AY087909 IPI:IPI00536641 PIR:E84812 RefSeq:NP_565899.1 UniGene:At.22599 ProteinModelPortal:Q945P1 SMR:Q945P1 PRIDE:Q945P1 EnsemblPlants:AT2G39050.1 GeneID:818491 KEGG:ath:AT2G39050 TAIR:At2g39050 eggNOG:NOG254161 HOGENOM:HBG750017 InParanoid:Q945P1 OMA:GYRTIRM PhylomeDB:Q945P1 ProtClustDB:CLSN2681741 ArrayExpress:Q945P1 Genevestigator:Q945P1 Uniprot:Q945P1
Root	Isotig01176	146	70	0.932	4.35E-06	TAIR|locus:2064965 - symbol:EULS3 "AT2G39050" species:3702 "Arabidopsis thaliana" [GO:0008150 "biological_process" evidence=ND] [GO:0005634 "nucleus" evidence=IDA] [GO:0005737 "cytoplasm" evidence=IDA] [GO:0030246 "carbohydrate binding" evidence=IDA] GO:GO:0005634 GO:GO:0005737 EMBL:CP002685 GenomeReviews:CT485783_GR InterPro:IPR000772 SUPFAM:SSF50370 PROSITE:PS50231 GO:GO:0030246 EMBL:AC005770 Pfam:PF14200 EMBL:AF411801 EMBL:AY093795 EMBL:AY087909 IPI:IPI00536641 PIR:E84812 RefSeq:NP_565899.1 UniGene:At.22599 ProteinModelPortal:Q945P1 SMR:Q945P1 PRIDE:Q945P1 EnsemblPlants:AT2G39050.1 GeneID:818491 KEGG:ath:AT2G39050 TAIR:At2g39050 eggNOG:NOG254161 HOGENOM:HBG750017 InParanoid:Q945P1 OMA:GYRTIRM PhylomeDB:Q945P1 ProtClustDB:CLSN2681741 ArrayExpress:Q945P1 Genevestigator:Q945P1 Uniprot:Q945P1
Root	Isotig01177	145	70	0.922	5.71E-06	TAIR|locus:2064965 - symbol:EULS3 "AT2G39050" species:3702 "Arabidopsis thaliana" [GO:0008150 "biological_process" evidence=ND] [GO:0005634 "nucleus" evidence=IDA] [GO:0005737 "cytoplasm" evidence=IDA] [GO:0030246 "carbohydrate binding" evidence=IDA] GO:GO:0005634 GO:GO:0005737 EMBL:CP002685 GenomeReviews:CT485783_GR InterPro:IPR000772 SUPFAM:SSF50370 PROSITE:PS50231 GO:GO:0030246 EMBL:AC005770 Pfam:PF14200 EMBL:AF411801 EMBL:AY093795 EMBL:AY087909 IPI:IPI00536641 PIR:E84812 RefSeq:NP_565899.1 UniGene:At.22599 ProteinModelPortal:Q945P1 SMR:Q945P1 PRIDE:Q945P1 EnsemblPlants:AT2G39050.1 GeneID:818491 KEGG:ath:AT2G39050 TAIR:At2g39050 eggNOG:NOG254161 HOGENOM:HBG750017 InParanoid:Q945P1 OMA:GYRTIRM PhylomeDB:Q945P1 ProtClustDB:CLSN2681741 ArrayExpress:Q945P1 Genevestigator:Q945P1 Uniprot:Q945P1
Root	Isotig01178	137	67	0.903	1.46E-05	TAIR|locus:2064965 - symbol:EULS3 "AT2G39050" species:3702 "Arabidopsis thaliana" [GO:0008150 "biological_process" evidence=ND] [GO:0005634 "nucleus" evidence=IDA] [GO:0005737 "cytoplasm" evidence=IDA] [GO:0030246 "carbohydrate binding" evidence=IDA] GO:GO:0005634 GO:GO:0005737 EMBL:CP002685 GenomeReviews:CT485783_GR InterPro:IPR000772 SUPFAM:SSF50370 PROSITE:PS50231 GO:GO:0030246 EMBL:AC005770 Pfam:PF14200 EMBL:AF411801 EMBL:AY093795 EMBL:AY087909 IPI:IPI00536641 PIR:E84812 RefSeq:NP_565899.1 UniGene:At.22599 ProteinModelPortal:Q945P1 SMR:Q945P1 PRIDE:Q945P1 EnsemblPlants:AT2G39050.1 GeneID:818491 KEGG:ath:AT2G39050 TAIR:At2g39050

Tissue	Identification	# of reads (stress)	# of reads (control)	Fold Change	p-value	Description
						eggNOG:NOG254161 HOGENOM:HBG750017 InParanoid:Q945P1 OMA:GYRTIRM PhylomeDB:Q945P1 ProtClustDB:CLSN2681741 ArrayExpress:Q945P1 Genevestigator:Q945P1 Uniprot:Q945P1
Root	Isotig01179	138	67	0.913	1.12E-05	TAIR|locus:2064965 - symbol:EULS3 "AT2G39050" species:3702 "Arabidopsis thaliana" [GO:0008150 "biological_process" evidence=ND] [GO:0005634 "nucleus" evidence=IDA] [GO:0005737 "cytoplasm" evidence=IDA] [GO:0030246 "carbohydrate binding" evidence=IDA] GO:GO:0005634 GO:GO:0005737 EMBL:CP002685 GenomeReviews:CT485783_GR InterPro:IPR000772 SUPFAM:SSF50370 PROSITE:PS50231 GO:GO:0030246 EMBL:AC005770 Pfam:PF14200 EMBL:AF411801 EMBL:AY093795 EMBL:AY087909 IPI:IPI00536641 PIR:E84812 RefSeq:NP_565899.1 UniGene:At.22599 ProteinModelPortal:Q945P1 SMR:Q945P1 PRIDE:Q945P1 EnsemblPlants:AT2G39050.1 GeneID:818491 KEGG:ath:AT2G39050 TAIR:At2g39050 eggNOG:NOG254161 HOGENOM:HBG750017 InParanoid:Q945P1 OMA:GYRTIRM PhylomeDB:Q945P1 ProtClustDB:CLSN2681741 ArrayExpress:Q945P1 Genevestigator:Q945P1 Uniprot:Q945P1
Root	Isotig01180	137	67	0.903	1.46E-05	TAIR|locus:2064965 - symbol:EULS3 "AT2G39050" species:3702 "Arabidopsis thaliana" [GO:0008150 "biological_process" evidence=ND] [GO:0005634 "nucleus" evidence=IDA] [GO:0005737 "cytoplasm" evidence=IDA] [GO:0030246 "carbohydrate binding" evidence=IDA] GO:GO:0005634 GO:GO:0005737 EMBL:CP002685 GenomeReviews:CT485783_GR InterPro:IPR000772 SUPFAM:SSF50370 PROSITE:PS50231 GO:GO:0030246 EMBL:AC005770 Pfam:PF14200 EMBL:AF411801 EMBL:AY093795 EMBL:AY087909 IPI:IPI00536641 PIR:E84812 RefSeq:NP_565899.1 UniGene:At.22599 ProteinModelPortal:Q945P1 SMR:Q945P1 PRIDE:Q945P1 EnsemblPlants:AT2G39050.1 GeneID:818491 KEGG:ath:AT2G39050 TAIR:At2g39050 eggNOG:NOG254161 HOGENOM:HBG750017 InParanoid:Q945P1 OMA:GYRTIRM PhylomeDB:Q945P1 ProtClustDB:CLSN2681741 ArrayExpress:Q945P1 Genevestigator:Q945P1 Uniprot:Q945P1
Root	Isotig01189	40	7	2.386	1.71E-06	TAIR|locus:2102787 - symbol:AT3G46920 "AT3G46920" species:3702 "Arabidopsis thaliana" [GO:0004672 "protein kinase activity" evidence=ISS] [GO:0006468 "protein phosphorylation" evidence=IEA;ISS] [GO:0004712 "protein serine/threonine/tyrosine kinase activity" evidence=ISS] InterPro:IPR000270 InterPro:IPR000719 InterPro:IPR001245 InterPro:IPR008271 InterPro:IPR011009 InterPro:IPR017441 Pfam:PF00564 Pfam:PF07714 PRINTS:PR00109 PROSITE:PS00107 PROSITE:PS00108 PROSITE:PS50011 SMART:SM00666 GO:GO:0005524 EMBL:CP002686 GenomeReviews:BA000014_GR eggNOG:COG0515 SUPFAM:SSF56112 GO:GO:0004674 HSSP:P36897 EMBL:AL096859 IPI:IPI00523356 PIR:T12956 RefSeq:NP_190276.1 UniGene:At.43530 ProteinModelPortal:Q9STG4 SMR:Q9STG4 PRIDE:Q9STG4 EnsemblPlants:AT3G46920.1 GeneID:823845 KEGG:ath:AT3G46920 TAIR:At3g46920 HOGENOM:HBG317897 InParanoid:Q9STG4 OMA:PRPSDGM PhylomeDB:Q9STG4 ProtClustDB:CLSN2915679 Genevestigator:Q9STG4 Uniprot:Q9STG4
Root	Isotig01190	40	7	2.386	1.71E-06	TAIR|locus:2102787 - symbol:AT3G46920 "AT3G46920" species:3702 "Arabidopsis thaliana" [GO:0004672 "protein kinase activity" evidence=ISS] [GO:0006468 "protein phosphorylation" evidence=IEA;ISS] [GO:0004712 "protein serine/threonine/tyrosine kinase activity" evidence=ISS] InterPro:IPR000270 InterPro:IPR000719 InterPro:IPR001245 InterPro:IPR008271 InterPro:IPR011009 InterPro:IPR017441 Pfam:PF00564 Pfam:PF07714 PRINTS:PR00109 PROSITE:PS00107 PROSITE:PS00108 PROSITE:PS50011 SMART:SM00666 GO:GO:0005524 EMBL:CP002686 GenomeReviews:BA000014_GR eggNOG:COG0515 SUPFAM:SSF56112 GO:GO:0004674 HSSP:P36897 EMBL:AL096859 IPI:IPI00523356 PIR:T12956 RefSeq:NP_190276.1 UniGene:At.43530 ProteinModelPortal:Q9STG4 SMR:Q9STG4 PRIDE:Q9STG4 EnsemblPlants:AT3G46920.1 GeneID:823845 KEGG:ath:AT3G46920 TAIR:At3g46920 HOGENOM:HBG317897 InParanoid:Q9STG4 OMA:PRPSDGM PhylomeDB:Q9STG4 ProtClustDB:CLSN2915679 Genevestigator:Q9STG4 Uniprot:Q9STG4
Root	Isotig01191	33	7	2.108	5.57E-05	TAIR|locus:2102787 - symbol:AT3G46920 "AT3G46920" species:3702 "Arabidopsis thaliana" [GO:0004672 "protein kinase activity" evidence=ISS] [GO:0006468 "protein phosphorylation" evidence=IEA;ISS] [GO:0004712 "protein serine/threonine/tyrosine kinase activity" evidence=ISS] InterPro:IPR000270 InterPro:IPR000719 InterPro:IPR001245 InterPro:IPR008271 InterPro:IPR011009 InterPro:IPR017441 Pfam:PF00564 Pfam:PF07714 PRINTS:PR00109 PROSITE:PS00107 PROSITE:PS00108 PROSITE:PS50011 SMART:SM00666 GO:GO:0005524 EMBL:CP002686 GenomeReviews:BA000014_GR eggNOG:COG0515 SUPFAM:SSF56112 GO:GO:0004674 HSSP:P36897 EMBL:AL096859 IPI:IPI00523356 PIR:T12956 RefSeq:NP_190276.1 UniGene:At.43530 ProteinModelPortal:Q9STG4 SMR:Q9STG4 PRIDE:Q9STG4 EnsemblPlants:AT3G46920.1 GeneID:823845 KEGG:ath:AT3G46920 TAIR:At3g46920 HOGENOM:HBG317897 InParanoid:Q9STG4 OMA:PRPSDGM PhylomeDB:Q9STG4 ProtClustDB:CLSN2915679 Genevestigator:Q9STG4 Uniprot:Q9STG4
Root	Isotig01192	33	7	2.108	5.57E-05	TAIR|locus:2102787 - symbol:AT3G46920 "AT3G46920" species:3702 "Arabidopsis thaliana" [GO:0004672 "protein kinase activity" evidence=ISS] [GO:0006468 "protein phosphorylation" evidence=IEA;ISS] [GO:0004712 "protein

Tissue	Identification	# of reads (stress)	# of reads (control)	Fold Change	p-value	Description
						serine/threonine/tyrosine kinase activity" evidence=ISS] InterPro:IPR000270 InterPro:IPR000719 InterPro:IPR001245 InterPro:IPR008271 InterPro:IPR011009 InterPro:IPR017441 Pfam:PF00564 Pfam:PF07714 PRINTS:PR00109 PROSITE:PS00107 PROSITE:PS00108 PROSITE:PS50011 SMART:SM00666 GO:GO:0005524 EMBL:CP002686 GenomeReviews:BA000014_GR eggNOG:COG0515 SUPFAM:SSF56112 GO:GO:0004674 HSSP:P36897 EMBL:AL096859 IPI:IPI00523356 PIR:T12956 RefSeq:NP_190276.1 UniGene:At.43530 ProteinModelPortal:Q9STG4 SMR:Q9STG4 PRIDE:Q9STG4 EnsemblPlants:AT3G46920.1 GeneID:823845 KEGG:ath:AT3G46920 TAIR:At3g46920 HOGENOM:HBG317897 InParanoid:Q9STG4 OMA:PRPSDGM PhylomeDB:Q9STG4 ProtClustDB:CLSN2915679 Genevestigator:Q9STG4 Uniprot:Q9STG4
Root	Isotig01193	30	6	2.193	8.18E-05	TAIR|locus:2102787 - symbol:AT3G46920 "AT3G46920" species:3702 "Arabidopsis thaliana" [GO:0004672 "protein kinase activity" evidence=ISS] [GO:0006468 "protein phosphorylation" evidence=IEA;ISS] [GO:0004712 "protein serine/threonine/tyrosine kinase activity" evidence=ISS] InterPro:IPR000270 InterPro:IPR000719 InterPro:IPR001245 InterPro:IPR008271 InterPro:IPR011009 InterPro:IPR017441 Pfam:PF00564 Pfam:PF07714 PRINTS:PR00109 PROSITE:PS00107 PROSITE:PS00108 PROSITE:PS50011 SMART:SM00666 GO:GO:0005524 EMBL:CP002686 GenomeReviews:BA000014_GR eggNOG:COG0515 SUPFAM:SSF56112 GO:GO:0004674 HSSP:P36897 EMBL:AL096859 IPI:IPI00523356 PIR:T12956 RefSeq:NP_190276.1 UniGene:At.43530 ProteinModelPortal:Q9STG4 SMR:Q9STG4 PRIDE:Q9STG4 EnsemblPlants:AT3G46920.1 GeneID:823845 KEGG:ath:AT3G46920 TAIR:At3g46920 HOGENOM:HBG317897 InParanoid:Q9STG4 OMA:PRPSDGM PhylomeDB:Q9STG4 ProtClustDB:CLSN2915679 Genevestigator:Q9STG4 Uniprot:Q9STG4
Root	Isotig01205	3	52	-4.244	1.90E-13	TAIR|locus:2149284 - symbol:DIN10 "AT5G20250" species:3702 "Arabidopsis thaliana" [GO:0004553 "hydrolase activity, hydrolyzing O-glycosyl compounds" evidence=ISS] [GO:0009507 "chloroplast" evidence=IDA] [GO:0006979 "response to oxidative stress" evidence=IEP] [GO:0009409 "response to cold" evidence=IEP] [GO:0080167 "response to karrikin" evidence=IEP] [GO:0009506 "plasmodesma" evidence=IDA] InterPro:IPR013785 GO:GO:0009506 EMBL:CP002688 GenomeReviews:BA000015_GR GO:GO:0009507 GO:GO:0006979 Gene3D:G3DSA:3.20.20.70 InterPro:IPR017853 SUPFAM:SSF51445 GO:GO:0005975 GO:GO:0009409 GO:GO:0080167 CAZy:GH36 eggNOG:NOG06986 InterPro:IPR008811 Pfam:PF05691 EMBL:AF296825 KO:K06617 GO:GO:0047274 EMBL:AY090237 EMBL:BT000632 EMBL:AK227214 EMBL:AK221808 EMBL:AK316714 EMBL:AK317307 EMBL:AK318923 IPI:IPI00523317 RefSeq:NP_001031910.1 RefSeq:NP_001190347.1 RefSeq:NP_197525.1 RefSeq:NP_851044.2 UniGene:At.25459 UniGene:At.74986 ProteinModelPortal:Q8RX87 IntAct:Q8RX87 STRING:Q8RX87 PRIDE:Q8RX87 EnsemblPlants:AT5G20250.1 EnsemblPlants:AT5G20250.2 EnsemblPlants:AT5G20250.3 GeneID:832147 KEGG:ath:AT5G20250 TAIR:At5g20250 InParanoid:Q8RX87 OMA:SVAYNSV PhylomeDB:Q8RX87 ProtClustDB:PLN02684 Uniprot:Q8RX87
Root	Isotig01206	3	52	-4.244	1.90E-13	TAIR|locus:2149284 - symbol:DIN10 "AT5G20250" species:3702 "Arabidopsis thaliana" [GO:0004553 "hydrolase activity, hydrolyzing O-glycosyl compounds" evidence=ISS] [GO:0009507 "chloroplast" evidence=IDA] [GO:0006979 "response to oxidative stress" evidence=IEP] [GO:0009409 "response to cold" evidence=IEP] [GO:0080167 "response to karrikin" evidence=IEP] [GO:0009506 "plasmodesma" evidence=IDA] InterPro:IPR013785 GO:GO:0009506 EMBL:CP002688 GenomeReviews:BA000015_GR GO:GO:0009507 GO:GO:0006979 Gene3D:G3DSA:3.20.20.70 InterPro:IPR017853 SUPFAM:SSF51445 GO:GO:0005975 GO:GO:0009409 GO:GO:0080167 CAZy:GH36 eggNOG:NOG06986 InterPro:IPR008811 Pfam:PF05691 EMBL:AF296825 KO:K06617 GO:GO:0047274 EMBL:AY090237 EMBL:BT000632 EMBL:AK227214 EMBL:AK221808 EMBL:AK316714 EMBL:AK317307 EMBL:AK318923 IPI:IPI00523317 RefSeq:NP_001031910.1 RefSeq:NP_001190347.1 RefSeq:NP_197525.1 RefSeq:NP_851044.2 UniGene:At.25459 UniGene:At.74986 ProteinModelPortal:Q8RX87 IntAct:Q8RX87 STRING:Q8RX87 PRIDE:Q8RX87 EnsemblPlants:AT5G20250.1 EnsemblPlants:AT5G20250.2 EnsemblPlants:AT5G20250.3 GeneID:832147 KEGG:ath:AT5G20250 TAIR:At5g20250 InParanoid:Q8RX87 OMA:SVAYNSV PhylomeDB:Q8RX87 ProtClustDB:PLN02684 Uniprot:Q8RX87
Root	Isotig01207	3	47	-4.099	4.33E-12	TAIR|locus:2149284 - symbol:DIN10 "AT5G20250" species:3702 "Arabidopsis thaliana" [GO:0004553 "hydrolase activity, hydrolyzing O-glycosyl compounds" evidence=ISS] [GO:0009507 "chloroplast" evidence=IDA] [GO:0006979 "response to oxidative stress" evidence=IEP] [GO:0009409 "response to cold" evidence=IEP] [GO:0080167 "response to karrikin" evidence=IEP] [GO:0009506 "plasmodesma" evidence=IDA] InterPro:IPR013785 GO:GO:0009506 EMBL:CP002688 GenomeReviews:BA000015_GR GO:GO:0009507 GO:GO:0006979 Gene3D:G3DSA:3.20.20.70 InterPro:IPR017853 SUPFAM:SSF51445 GO:GO:0005975 GO:GO:0009409

Tissue	Identification	# of reads (stress)	# of reads (control)	Fold Change	p-value	Description
						GO:GO:0080167 CAZy:GH36 eggNOG:NOG06986 InterPro:IPR008811 Pfam:PF05691 EMBL:AF296825 KO:K06617 GO:GO:0047274 EMBL:AY090237 EMBL:BT000632 EMBL:AK227214 EMBL:AK221808 EMBL:AK316714 EMBL:AK317307 EMBL:AK318923 IPI:IPI00523317 RefSeq:NP_001031910.1 RefSeq:NP_001190347.1 RefSeq:NP_197525.1 RefSeq:NP_851044.2 UniGene:At.25459 UniGene:At.74986 ProteinModelPortal:Q8RX87 IntAct:Q8RX87 STRING:Q8RX87 PRIDE:Q8RX87 EnsemblPlants:AT5G20250.1 EnsemblPlants:AT5G20250.2 EnsemblPlants:AT5G20250.3 GeneID:832147 KEGG:ath:AT5G20250 TAIR:At5g20250 InParanoid:Q8RX87 OMA:SVAYNSV PhylomeDB:Q8RX87 ProtClustDB:PLN02684 Uniprot:Q8RX87
Root	Isotig01208	3	47	-4.099	4.33E-12	TAIR|locus:2149284 - symbol:DIN10 "AT5G20250" species:3702 "Arabidopsis thaliana" [GO:0004553 "hydrolase activity, hydrolyzing O-glycosyl compounds" evidence=ISS] [GO:0009507 "chloroplast" evidence=IDA] [GO:0006979 "response to oxidative stress" evidence=IEP] [GO:0009409 "response to cold" evidence=IEP] [GO:0080167 "response to karrikin" evidence=IEP] [GO:0009506 "plasmodesma" evidence=IDA] InterPro:IPR013785 GO:GO:0009506 EMBL:CP002688 GenomeReviews:BA000015_GR GO:GO:0009507 GO:GO:0006979 Gene3D:G3DSA:3.20.20.70 InterPro:IPR017853 SUPFAM:SSF51445 GO:GO:0005975 GO:GO:0009409 GO:GO:0080167 CAZy:GH36 eggNOG:NOG06986 InterPro:IPR008811 Pfam:PF05691 EMBL:AF296825 KO:K06617 GO:GO:0047274 EMBL:AY090237 EMBL:BT000632 EMBL:AK227214 EMBL:AK221808 EMBL:AK316714 EMBL:AK317307 EMBL:AK318923 IPI:IPI00523317 RefSeq:NP_001031910.1 RefSeq:NP_001190347.1 RefSeq:NP_197525.1 RefSeq:NP_851044.2 UniGene:At.25459 UniGene:At.74986 ProteinModelPortal:Q8RX87 IntAct:Q8RX87 STRING:Q8RX87 PRIDE:Q8RX87 EnsemblPlants:AT5G20250.1 EnsemblPlants:AT5G20250.2 EnsemblPlants:AT5G20250.3 GeneID:832147 KEGG:ath:AT5G20250 TAIR:At5g20250 InParanoid:Q8RX87 OMA:SVAYNSV PhylomeDB:Q8RX87 ProtClustDB:PLN02684 Uniprot:Q8RX87
Root	Isotig01209	3	50	-4.188	6.62E-13	TAIR|locus:2149284 - symbol:DIN10 "AT5G20250" species:3702 "Arabidopsis thaliana" [GO:0004553 "hydrolase activity, hydrolyzing O-glycosyl compounds" evidence=ISS] [GO:0009507 "chloroplast" evidence=IDA] [GO:0006979 "response to oxidative stress" evidence=IEP] [GO:0009409 "response to cold" evidence=IEP] [GO:0080167 "response to karrikin" evidence=IEP] [GO:0009506 "plasmodesma" evidence=IDA] InterPro:IPR013785 GO:GO:0009506 EMBL:CP002688 GenomeReviews:BA000015_GR GO:GO:0009507 GO:GO:0006979 Gene3D:G3DSA:3.20.20.70 InterPro:IPR017853 SUPFAM:SSF51445 GO:GO:0005975 GO:GO:0009409 GO:GO:0080167 CAZy:GH36 eggNOG:NOG06986 InterPro:IPR008811 Pfam:PF05691 EMBL:AF296825 KO:K06617 GO:GO:0047274 EMBL:AY090237 EMBL:BT000632 EMBL:AK227214 EMBL:AK221808 EMBL:AK316714 EMBL:AK317307 EMBL:AK318923 IPI:IPI00523317 RefSeq:NP_001031910.1 RefSeq:NP_001190347.1 RefSeq:NP_197525.1 RefSeq:NP_851044.2 UniGene:At.25459 UniGene:At.74986 ProteinModelPortal:Q8RX87 IntAct:Q8RX87 STRING:Q8RX87 PRIDE:Q8RX87 EnsemblPlants:AT5G20250.1 EnsemblPlants:AT5G20250.2 EnsemblPlants:AT5G20250.3 GeneID:832147 KEGG:ath:AT5G20250 TAIR:At5g20250 InParanoid:Q8RX87 OMA:SVAYNSV PhylomeDB:Q8RX87 ProtClustDB:PLN02684 Uniprot:Q8RX87
Root	Isotig01210	3	50	-4.188	6.62E-13	TAIR|locus:2149284 - symbol:DIN10 "AT5G20250" species:3702 "Arabidopsis thaliana" [GO:0004553 "hydrolase activity, hydrolyzing O-glycosyl compounds" evidence=ISS] [GO:0009507 "chloroplast" evidence=IDA] [GO:0006979 "response to oxidative stress" evidence=IEP] [GO:0009409 "response to cold" evidence=IEP] [GO:0080167 "response to karrikin" evidence=IEP] [GO:0009506 "plasmodesma" evidence=IDA] InterPro:IPR013785 GO:GO:0009506 EMBL:CP002688 GenomeReviews:BA000015_GR GO:GO:0009507 GO:GO:0006979 Gene3D:G3DSA:3.20.20.70 InterPro:IPR017853 SUPFAM:SSF51445 GO:GO:0005975 GO:GO:0009409 GO:GO:0080167 CAZy:GH36 eggNOG:NOG06986 InterPro:IPR008811 Pfam:PF05691 EMBL:AF296825 KO:K06617 GO:GO:0047274 EMBL:AY090237 EMBL:BT000632 EMBL:AK227214 EMBL:AK221808 EMBL:AK316714 EMBL:AK317307 EMBL:AK318923 IPI:IPI00523317 RefSeq:NP_001031910.1 RefSeq:NP_001190347.1 RefSeq:NP_197525.1 RefSeq:NP_851044.2 UniGene:At.25459 UniGene:At.74986 ProteinModelPortal:Q8RX87 IntAct:Q8RX87 STRING:Q8RX87 PRIDE:Q8RX87 EnsemblPlants:AT5G20250.1 EnsemblPlants:AT5G20250.2 EnsemblPlants:AT5G20250.3 GeneID:832147 KEGG:ath:AT5G20250 TAIR:At5g20250 InParanoid:Q8RX87 OMA:SVAYNSV PhylomeDB:Q8RX87 ProtClustDB:PLN02684 Uniprot:Q8RX87
Root	Isotig01211	0	19	-5.377	3.67E-06	TAIR|locus:2149284 - symbol:DIN10 "AT5G20250" species:3702 "Arabidopsis thaliana" [GO:0004553 "hydrolase activity, hydrolyzing O-glycosyl compounds" evidence=ISS] [GO:0009507 "chloroplast" evidence=IDA] [GO:0006979

Tissue	Identification	# of reads (stress)	# of reads (control)	Fold Change	p-value	Description
						"response to oxidative stress" evidence=IEP] [GO:0009409 "response to cold" evidence=IEP] [GO:0080167 "response to karrikin" evidence=IEP] [GO:0009506 "plasmodesma" evidence=IDA] InterPro:IPR013785 GO:GO:0009506 EMBL:CP002688 GenomeReviews:BA000015_GR GO:GO:0009507 GO:GO:0006979 Gene3D:G3DSA:3.20.20.70 InterPro:IPR017853 SUPFAM:SSF51445 GO:GO:0005975 GO:GO:0009409 GO:GO:0080167 CAZy:GH36 eggNOG:NOG06986 InterPro:IPR008811 Pfam:PF05691 EMBL:AF296825 KO:K06617 GO:GO:0047274 EMBL:AY090237 EMBL:BT000632 EMBL:AK227214 EMBL:AK221808 EMBL:AK316714 EMBL:AK317307 EMBL:AK318923 IPI:IPI00523317 RefSeq:NP_001031910.1 RefSeq:NP_001190347.1 RefSeq:NP_197525.1 RefSeq:NP_851044.2 UniGene:At.25459 UniGene:At.74986 ProteinModelPortal:Q8RX87 IntAct:Q8RX87 STRING:Q8RX87 PRIDE:Q8RX87 EnsemblPlants:AT5G20250.1 EnsemblPlants:AT5G20250.2 EnsemblPlants:AT5G20250.3 GeneID:832147 KEGG:ath:AT5G20250 TAIR:At5g20250 InParanoid:Q8RX87 OMA:SVAYNSV PhylomeDB:Q8RX87 ProtClustDB:PLN02684 Uniprot:Q8RX87
Root	Isotig01212	0	17	-5.216	1.26E-05	TAIR|locus:2149284 - symbol:DIN10 "AT5G20250" species:3702 "Arabidopsis thaliana" [GO:0004553 "hydrolase activity, hydrolyzing O-glycosyl compounds" evidence=ISS] [GO:0009507 "chloroplast" evidence=IDA] [GO:0006979 "response to oxidative stress" evidence=IEP] [GO:0009409 "response to cold" evidence=IEP] [GO:0080167 "response to karrikin" evidence=IEP] [GO:0009506 "plasmodesma" evidence=IDA] InterPro:IPR013785 GO:GO:0009506 EMBL:CP002688 GenomeReviews:BA000015_GR GO:GO:0009507 GO:GO:0006979 Gene3D:G3DSA:3.20.20.70 InterPro:IPR017853 SUPFAM:SSF51445 GO:GO:0005975 GO:GO:0009409 GO:GO:0080167 CAZy:GH36 eggNOG:NOG06986 InterPro:IPR008811 Pfam:PF05691 EMBL:AF296825 KO:K06617 GO:GO:0047274 EMBL:AY090237 EMBL:BT000632 EMBL:AK227214 EMBL:AK221808 EMBL:AK316714 EMBL:AK317307 EMBL:AK318923 IPI:IPI00523317 RefSeq:NP_001031910.1 RefSeq:NP_001190347.1 RefSeq:NP_197525.1 RefSeq:NP_851044.2 UniGene:At.25459 UniGene:At.74986 ProteinModelPortal:Q8RX87 IntAct:Q8RX87 STRING:Q8RX87 PRIDE:Q8RX87 EnsemblPlants:AT5G20250.1 EnsemblPlants:AT5G20250.2 EnsemblPlants:AT5G20250.3 GeneID:832147 KEGG:ath:AT5G20250 TAIR:At5g20250 InParanoid:Q8RX87 OMA:SVAYNSV PhylomeDB:Q8RX87 ProtClustDB:PLN02684 Uniprot:Q8RX87
Root	Isotig01227	15	42	-1.614	7.30E-05	TAIR|locus:2182432 - symbol:AT5G35200 species:3702 "Arabidopsis thaliana" [GO:0005543 "phospholipid binding" evidence=IEA] [GO:0005545 "1-phosphatidylinositol binding" evidence=IEA] [GO:0030276 "clathrin binding" evidence=IEA] [GO:0048268 "clathrin coat assembly" evidence=IEA] [GO:0005886 "plasma membrane" evidence=IDA] InterPro:IPR011417 InterPro:IPR014712 Pfam:PF07651 GO:GO:0005886 EMBL:CP002688 GenomeReviews:BA000015_GR GO:GO:0005794 GO:GO:0005545 GO:GO:0005905 GO:GO:0006897 GO:GO:0030136 GO:GO:0030276 HOGENOM:HBG744562 GO:GO:0030118 GO:GO:0048268 InterPro:IPR008942 InterPro:IPR013809 Gene3D:G3DSA:1.25.40.90 Gene3D:G3DSA:1.20.58.150 SMART:SM00273 SUPFAM:SSF48464 PROSITE:PS50942 eggNOG:NOG267212 EMBL:AP002031 EMBL:AF080121 EMBL:AY081284 EMBL:BT000375 IPI:IPI00518637 RefSeq:NP_198370.1 UniGene:At.30612 ProteinModelPortal:Q9LHS0 STRING:Q9LHS0 PRIDE:Q9LHS0 EnsemblPlants:AT5G35200.1 GeneID:833474 KEGG:ath:AT5G35200 TAIR:At5g35200 InParanoid:Q9LHS0 OMA:EMQRNDA PhylomeDB:Q9LHS0 ProtClustDB:CLSN2686467 ArrayExpress:Q9LHS0 Genevestigator:Q9LHS0 GermOnline:AT5G35200 Uniprot:Q9LHS0
Root	Isotig01228	15	43	-1.648	4.63E-05	TAIR|locus:2182432 - symbol:AT5G35200 species:3702 "Arabidopsis thaliana" [GO:0005543 "phospholipid binding" evidence=IEA] [GO:0005545 "1-phosphatidylinositol binding" evidence=IEA] [GO:0030276 "clathrin binding" evidence=IEA] [GO:0048268 "clathrin coat assembly" evidence=IEA] [GO:0005886 "plasma membrane" evidence=IDA] InterPro:IPR011417 InterPro:IPR014712 Pfam:PF07651 GO:GO:0005886 EMBL:CP002688 GenomeReviews:BA000015_GR GO:GO:0005794 GO:GO:0005545 GO:GO:0005905 GO:GO:0006897 GO:GO:0030136 GO:GO:0030276 HOGENOM:HBG744562 GO:GO:0030118 GO:GO:0048268 InterPro:IPR008942 InterPro:IPR013809 Gene3D:G3DSA:1.25.40.90 Gene3D:G3DSA:1.20.58.150 SMART:SM00273 SUPFAM:SSF48464 PROSITE:PS50942 eggNOG:NOG267212 EMBL:AP002031 EMBL:AF080121 EMBL:AY081284 EMBL:BT000375 IPI:IPI00518637 RefSeq:NP_198370.1 UniGene:At.30612 ProteinModelPortal:Q9LHS0 STRING:Q9LHS0 PRIDE:Q9LHS0 EnsemblPlants:AT5G35200.1 GeneID:833474 KEGG:ath:AT5G35200 TAIR:At5g35200 InParanoid:Q9LHS0 OMA:EMQRNDA PhylomeDB:Q9LHS0 ProtClustDB:CLSN2686467 ArrayExpress:Q9LHS0 Genevestigator:Q9LHS0 GermOnline:AT5G35200 Uniprot:Q9LHS0

Tissue	Identification	# of reads (stress)	# of reads (control)	Fold Change	p-value	Description
Root	Isotig01229	10	41	-2.165	1.78E-06	TAIR|locus:2182432 - symbol:AT5G35200 species:3702 "Arabidopsis thaliana" [GO:0005543 "phospholipid binding" evidence=IEA] [GO:0005545 "1-phosphatidylinositol binding" evidence=IEA] [GO:0030276 "clathrin binding" evidence=IEA] [GO:0048268 "clathrin coat assembly" evidence=IEA] [GO:0005886 "plasma membrane" evidence=IDA] InterPro:IPR011417 InterPro:IPR014712 Pfam:PF07651 GO:GO:0005886 EMBL:CP002688 GenomeReviews:BA000015_GR GO:GO:0005794 GO:GO:0005545 GO:GO:0005905 GO:GO:0006897 GO:GO:0030136 GO:GO:0030276 HOGENOM:HBG744562 GO:GO:0030118 GO:GO:0048268 InterPro:IPR008942 InterPro:IPR013809 Gene3D:G3DSA:1.25.40.90 Gene3D:G3DSA:1.20.58.150 SMART:SM00273 SUPFAM:SSF48464 PROSITE:PS50942 eggNOG:NOG267212 EMBL:AP002031 EMBL:AF080121 EMBL:AY081284 EMBL:BT000375 IPI:IPI00518637 RefSeq:NP_198370.1 UniGene:At.30612 ProteinModelPortal:Q9LHS0 STRING:Q9LHS0 PRIDE:Q9LHS0 EnsemblPlants:AT5G35200.1 GeneID:833474 KEGG:ath:AT5G35200 TAIR:At5g35200 InParanoid:Q9LHS0 OMA:EMQRNDA PhylomeDB:Q9LHS0 ProtClustDB:CLSN2686467 ArrayExpress:Q9LHS0 Genevestigator:Q9LHS0 GermOnline:AT5G35200 Uniprot:Q9LHS0
Root	Isotig01230	15	43	-1.648	4.63E-05	TAIR|locus:2182432 - symbol:AT5G35200 species:3702 "Arabidopsis thaliana" [GO:0005543 "phospholipid binding" evidence=IEA] [GO:0005545 "1-phosphatidylinositol binding" evidence=IEA] [GO:0030276 "clathrin binding" evidence=IEA] [GO:0048268 "clathrin coat assembly" evidence=IEA] [GO:0005886 "plasma membrane" evidence=IDA] InterPro:IPR011417 InterPro:IPR014712 Pfam:PF07651 GO:GO:0005886 EMBL:CP002688 GenomeReviews:BA000015_GR GO:GO:0005794 GO:GO:0005545 GO:GO:0005905 GO:GO:0006897 GO:GO:0030136 GO:GO:0030276 HOGENOM:HBG744562 GO:GO:0030118 GO:GO:0048268 InterPro:IPR008942 InterPro:IPR013809 Gene3D:G3DSA:1.25.40.90 Gene3D:G3DSA:1.20.58.150 SMART:SM00273 SUPFAM:SSF48464 PROSITE:PS50942 eggNOG:NOG267212 EMBL:AP002031 EMBL:AF080121 EMBL:AY081284 EMBL:BT000375 IPI:IPI00518637 RefSeq:NP_198370.1 UniGene:At.30612 ProteinModelPortal:Q9LHS0 STRING:Q9LHS0 PRIDE:Q9LHS0 EnsemblPlants:AT5G35200.1 GeneID:833474 KEGG:ath:AT5G35200 TAIR:At5g35200 InParanoid:Q9LHS0 OMA:EMQRNDA PhylomeDB:Q9LHS0 ProtClustDB:CLSN2686467 ArrayExpress:Q9LHS0 Genevestigator:Q9LHS0 GermOnline:AT5G35200 Uniprot:Q9LHS0
Root	Isotig01231	15	44	-1.682	2.92E-05	TAIR|locus:2182432 - symbol:AT5G35200 species:3702 "Arabidopsis thaliana" [GO:0005543 "phospholipid binding" evidence=IEA] [GO:0005545 "1-phosphatidylinositol binding" evidence=IEA] [GO:0030276 "clathrin binding" evidence=IEA] [GO:0048268 "clathrin coat assembly" evidence=IEA] [GO:0005886 "plasma membrane" evidence=IDA] InterPro:IPR011417 InterPro:IPR014712 Pfam:PF07651 GO:GO:0005886 EMBL:CP002688 GenomeReviews:BA000015_GR GO:GO:0005794 GO:GO:0005545 GO:GO:0005905 GO:GO:0006897 GO:GO:0030136 GO:GO:0030276 HOGENOM:HBG744562 GO:GO:0030118 GO:GO:0048268 InterPro:IPR008942 InterPro:IPR013809 Gene3D:G3DSA:1.25.40.90 Gene3D:G3DSA:1.20.58.150 SMART:SM00273 SUPFAM:SSF48464 PROSITE:PS50942 eggNOG:NOG267212 EMBL:AP002031 EMBL:AF080121 EMBL:AY081284 EMBL:BT000375 IPI:IPI00518637 RefSeq:NP_198370.1 UniGene:At.30612 ProteinModelPortal:Q9LHS0 STRING:Q9LHS0 PRIDE:Q9LHS0 EnsemblPlants:AT5G35200.1 GeneID:833474 KEGG:ath:AT5G35200 TAIR:At5g35200 InParanoid:Q9LHS0 OMA:EMQRNDA PhylomeDB:Q9LHS0 ProtClustDB:CLSN2686467 ArrayExpress:Q9LHS0 Genevestigator:Q9LHS0 GermOnline:AT5G35200 Uniprot:Q9LHS0
Root	Isotig01232	10	42	-2.199	1.05E-06	TAIR|locus:2182432 - symbol:AT5G35200 species:3702 "Arabidopsis thaliana" [GO:0005543 "phospholipid binding" evidence=IEA] [GO:0005545 "1-phosphatidylinositol binding" evidence=IEA] [GO:0030276 "clathrin binding" evidence=IEA] [GO:0048268 "clathrin coat assembly" evidence=IEA] [GO:0005886 "plasma membrane" evidence=IDA] InterPro:IPR011417 InterPro:IPR014712 Pfam:PF07651 GO:GO:0005886 EMBL:CP002688 GenomeReviews:BA000015_GR GO:GO:0005794 GO:GO:0005545 GO:GO:0005905 GO:GO:0006897 GO:GO:0030136 GO:GO:0030276 HOGENOM:HBG744562 GO:GO:0030118 GO:GO:0048268 InterPro:IPR008942 InterPro:IPR013809 Gene3D:G3DSA:1.25.40.90 Gene3D:G3DSA:1.20.58.150 SMART:SM00273 SUPFAM:SSF48464 PROSITE:PS50942 eggNOG:NOG267212 EMBL:AP002031 EMBL:AF080121 EMBL:AY081284 EMBL:BT000375 IPI:IPI00518637 RefSeq:NP_198370.1 UniGene:At.30612 ProteinModelPortal:Q9LHS0 STRING:Q9LHS0 PRIDE:Q9LHS0 EnsemblPlants:AT5G35200.1 GeneID:833474 KEGG:ath:AT5G35200 TAIR:At5g35200 InParanoid:Q9LHS0 OMA:EMQRNDA PhylomeDB:Q9LHS0 ProtClustDB:CLSN2686467 ArrayExpress:Q9LHS0 Genevestigator:Q9LHS0 GermOnline:AT5G35200 Uniprot:Q9LHS0

Tissue	Identification	# of reads (stress)	# of reads (control)	Fold Change	p-value	Description
Root	Isotig01241	714	55	3.569	4.00E-134	TIGR_CMR|GSU_1062 - symbol:GSU_1062 "cytochrome c family protein, putative" species:243231 "Geobacter sulfurreducens PCA" [GO:0009055 "electron carrier activity" evidence=ISS] EMBL:AE017180 GenomeReviews:AE017180_GR RefSeq:NP_952115.1 GeneID:2688672 KEGG:gsu:GSU1062 PATRIC:22024890 TIGR:GSU1062 HOGENOM:HBG090252 OMA:RIPELND ProtClustDB:CLSK743107 Uniprot:Q74EA1
Root	Isotig01242	714	55	3.569	4.00E-134	No hit
Root	Isotig01243	723	56	3.562	1.45E-135	No hit
Root	Isotig01244	723	56	3.562	1.45E-135	No hit
Root	Isotig01245	630	50	3.526	1.73E-117	No hit
Root	Isotig01246	38	6	2.534	1.37E-06	No hit
Root	Isotig01247	38	6	2.534	1.37E-06	No hit
Root	Isotig01256	78	31	1.202	3.56E-05	UNIPROTKB|Q02817 - symbol:MUC2 "Mucin-2" species:9606 "Homo sapiens" [GO:0005515 "protein binding" evidence=IPI] [GO:0005796 "Golgi lumen" evidence=TAS] [GO:0016266 "O-glycan processing" evidence=TAS] [GO:0043687 "post-translational protein modification" evidence=TAS] [GO:0044267 "cellular protein metabolic process" evidence=TAS] [GO:0070702 "inner mucus layer" evidence=ISS] [GO:0070703 "outer mucus layer" evidence=ISS] Reactome:REACT_17015 InterPro:IPR001007 PROSITE:PS01208 PROSITE:PS50184 SMART:SM00214 GO:GO:0005515 InterPro:IPR002919 Pfam:PF01826 SUPFAM:SSF57567 GO:GO:0043687 eggNOG:NOG12793 GO:GO:0005796 InterPro:IPR001846 Pfam:PF00094 SMART:SM00216 PROSITE:PS51233 GO:GO:0016266 InterPro:IPR014853 Pfam:PF08742 SMART:SM00832 InterPro:IPR006207 PROSITE:PS01185 PROSITE:PS01225 SMART:SM00041 DrugBank:DB01411 EMBL:L21998 EMBL:M74027 EMBL:M94131 EMBL:M94132 IPI:IPI00027201 PIR:A49963 RefSeq:NP_002448.2 UniGene:Hs.315 ProteinModelPortal:Q02817 SMR:Q02817 DIP:DIP-48824N IntAct:Q02817 STRING:Q02817 GlycoSuiteDB:Q02817 PhosphoSite:Q02817 DMDM:2506877 PRIDE:Q02817 Ensembl:ENST00000359061 GeneID:4583 KEGG:hsa:4583 CTD:4583 GeneCards:GC11P001064 HGNC:HGNC:7512 HPA:CAB005317 HPA:CAB016275 HPA:HPA006197 MIM:158370 neXtProt:NX_Q02817 PharmGKB:PA31316 HOVERGEN:HBG004380 InParanoid:Q02817 KO:K10955 NextBio:17613 ArrayExpress:Q02817 Bgee:Q02817 CleanEx:HS_MUC2 Genevestigator:Q02817 GermOnline:ENSG00000198788 GO:GO:0070702 GO:GO:0070703 InterPro:IPR006552 SMART:SM00215 Uniprot:Q02817
Root	Isotig01258	28	3	3.093	4.38E-06	MGI|MGI:2144837 - symbol:Ptpn23 "protein tyrosine phosphatase, non-receptor type 23" species:10090 "Mus musculus" [GO:0005634 "nucleus" evidence=ISO] [GO:0005737 "cytoplasm" evidence=ISO] [GO:0005768 "endosome" evidence=ISO] [GO:0016311 "dephosphorylation" evidence=IEA] [GO:0016791 "phosphatase activity" evidence=IEA] [GO:0004725 "protein tyrosine phosphatase activity" evidence=IEA] [GO:0005932 "microtubule basal body" evidence=ISO] [GO:0005856 "cytoskeleton" evidence=IEA] [GO:0016787 "hydrolase activity" evidence=IEA] [GO:0030030 "cell projection organization" evidence=IEA] [GO:0004721 "phosphoprotein phosphatase activity" evidence=IEA] [GO:0005929 "cilium" evidence=IEA] [GO:0031410 "cytoplasmic vesicle" evidence=IEA] [GO:0042995 "cell projection" evidence=IEA] [GO:0006470 "protein dephosphorylation" evidence=IEA] [GO:0060271 "cilium morphogenesis" evidence=ISO] InterPro:IPR000242 InterPro:IPR000387 InterPro:IPR016130 InterPro:IPR025304 Pfam:PF00102 Pfam:PF13949 PRINTS:PR00700 PROSITE:PS00383 PROSITE:PS50005 PROSITE:PS50055 PROSITE:PS50056 PROSITE:PS50293 SMART:SM00194 MGI:MGI:2144837 GO:GO:0005634 GO:GO:0005768 GO:GO:0016023 GO:GO:0005929 GO:GO:0005932 InterPro:IPR004328 Gene3D:G3DSA:1.25.40.280 Pfam:PF03097 SMART:SM01041 PROSITE:PS51180 GeneTree:ENSGT00650000093161 GO:GO:0060271 GO:GO:0004725 eggNOG:COG5599 KO:K01104 PDB:2W10 PDBsum:2W10 CTD:25930 HOVERGEN:HBG082231 OMA:GPGPHYL EMBL:BY750106 EMBL:CF734421 EMBL:CB248963 EMBL:BC006582 EMBL:BC022721 EMBL:BC059902 EMBL:AK173178 IPI:IPI00464166 IPI:IPI00606716 RefSeq:NP_001074512.1 UniGene:Mm.335477 ProteinModelPortal:Q6PB44 SMR:Q6PB44 DIP:DIP-48351N IntAct:Q6PB44 STRING:Q6PB44 PhosphoSite:Q6PB44 PRIDE:Q6PB44 Ensembl:ENSMUST00000040021 GeneID:104831 KEGG:mmu:104831 UCSC:uc009rty.1 UCSC:uc012hbi.1 HOGENOM:HBG444522 InParanoid:Q6PB44 OrthoDB:EOG4NCMBX PhylomeDB:Q6PB44 NextBio:357310 ArrayExpress:Q6PB44 Bgee:Q6PB44 Genevestigator:Q6PB44 GermOnline:ENSMUSG00000036057 Uniprot:Q6PB44
Root	Isotig01266	1	13	-3.829	0.000362856	TAIR|locus:2025886 - symbol:AT1G59740 "AT1G59740" species:3702 "Arabidopsis thaliana" [GO:0005215 "transporter activity" evidence=ISS] [GO:0006857 "oligopeptide transport" evidence=ISS] [GO:0016020 "membrane" evidence=ISS] [GO:0009624 "response to nematode" evidence=IEP] [GO:0005886 "plasma membrane"

Tissue	Identification	# of reads (stress)	# of reads (control)	Fold Change	p-value	Description
						evidence=IDA] InterPro:IPR000109 InterPro:IPR018456 Pfam:PF00854 PROSITE:PS01022 PROSITE:PS01023 GO:GO:0016021 EMBL:CP002684 GenomeReviews:CT485782_GR GO:GO:0005886 GO:GO:0009624 EMBL:AC007258 GO:GO:0005215 InterPro:IPR016196 SUPFAM:SSF103473 eggNOG:COG3104 OMA:AYMISAI GO:GO:0006857 HOGENOM:HBG605267 ProtClustDB:CLSN2679698 EMBL:AY035037 EMBL:AY051041 IPI:IPI00521525 PIR:C96621 RefSeq:NP_176183.1 UniGene:At.11372 ProteinModelPortal:Q93VV5 PRIDE:Q93VV5 EnsemblPlants:AT1G59740.1 GeneID:842267 KEGG:ath:AT1G59740 TAIR:At1g59740 InParanoid:Q93VV5 PhylomeDB:Q93VV5 ArrayExpress:Q93VV5 Genevestigator:Q93VV5 Uniprot:Q93VV5
Root	Isotig01268	1	13	-3.829	0.000362856	TAIR|locus:2025886 - symbol:AT1G59740 "AT1G59740" species:3702 "Arabidopsis thaliana" [GO:0005215 "transporter activity" evidence=ISS] [GO:0006857 "oligopeptide transport" evidence=ISS] [GO:0016020 "membrane" evidence=ISS] [GO:0009624 "response to nematode" evidence=IEP] [GO:0005886 "plasma membrane" evidence=IDA] InterPro:IPR000109 InterPro:IPR018456 Pfam:PF00854 PROSITE:PS01022 PROSITE:PS01023 GO:GO:0016021 EMBL:CP002684 GenomeReviews:CT485782_GR GO:GO:0005886 GO:GO:0009624 EMBL:AC007258 GO:GO:0005215 InterPro:IPR016196 SUPFAM:SSF103473 eggNOG:COG3104 OMA:AYMISAI GO:GO:0006857 HOGENOM:HBG605267 ProtClustDB:CLSN2679698 EMBL:AY035037 EMBL:AY051041 IPI:IPI00521525 PIR:C96621 RefSeq:NP_176183.1 UniGene:At.11372 ProteinModelPortal:Q93VV5 PRIDE:Q93VV5 EnsemblPlants:AT1G59740.1 GeneID:842267 KEGG:ath:AT1G59740 TAIR:At1g59740 InParanoid:Q93VV5 PhylomeDB:Q93VV5 ArrayExpress:Q93VV5 Genevestigator:Q93VV5 Uniprot:Q93VV5
Root	Isotig01278	21	2	3.263	4.62E-05	No hit
Root	Isotig01279	20	2	3.193	8.23E-05	No hit
Root	Isotig01280	21	2	3.263	4.62E-05	No hit
Root	Isotig01281	21	2	3.263	4.62E-05	No hit
Root	Isotig01282	20	2	3.193	8.23E-05	No hit
Root	Isotig01283	20	2	3.193	8.23E-05	No hit
Root	Isotig01296	85	27	1.526	2.24E-07	TAIR|locus:2075356 - symbol:AT3G46220 "AT3G46220" species:3702 "Arabidopsis thaliana" [GO:0008150 "biological_process" evidence=ND] EMBL:CP002686 GenomeReviews:BA000014_GR GO:GO:0016874 EMBL:AL355775 UniGene:At.1484 UniGene:At.70182 IPI:IPI00517015 PIR:T49263 RefSeq:NP_566883.4 PRIDE:Q9LX73 EnsemblPlants:AT3G46220.1 GeneID:823767 KEGG:ath:AT3G46220 TAIR:At3g46220 eggNOG:NOG277822 HOGENOM:HBG597219 InParanoid:Q9LX73 PhylomeDB:Q9LX73 ProtClustDB:PLN03083 Genevestigator:Q9LX73 InterPro:IPR018611 Pfam:PF09743 Uniprot:Q9LX73
Root	Isotig01297	85	27	1.526	2.24E-07	TAIR|locus:2075356 - symbol:AT3G46220 "AT3G46220" species:3702 "Arabidopsis thaliana" [GO:0008150 "biological_process" evidence=ND] EMBL:CP002686 GenomeReviews:BA000014_GR GO:GO:0016874 EMBL:AL355775 UniGene:At.1484 UniGene:At.70182 IPI:IPI00517015 PIR:T49263 RefSeq:NP_566883.4 PRIDE:Q9LX73 EnsemblPlants:AT3G46220.1 GeneID:823767 KEGG:ath:AT3G46220 TAIR:At3g46220 eggNOG:NOG277822 HOGENOM:HBG597219 InParanoid:Q9LX73 PhylomeDB:Q9LX73 ProtClustDB:PLN03083 Genevestigator:Q9LX73 InterPro:IPR018611 Pfam:PF09743 Uniprot:Q9LX73
Root	Isotig01298	85	27	1.526	2.24E-07	TAIR|locus:2075356 - symbol:AT3G46220 "AT3G46220" species:3702 "Arabidopsis thaliana" [GO:0008150 "biological_process" evidence=ND] EMBL:CP002686 GenomeReviews:BA000014_GR GO:GO:0016874 EMBL:AL355775 UniGene:At.1484 UniGene:At.70182 IPI:IPI00517015 PIR:T49263 RefSeq:NP_566883.4 PRIDE:Q9LX73 EnsemblPlants:AT3G46220.1 GeneID:823767 KEGG:ath:AT3G46220 TAIR:At3g46220 eggNOG:NOG277822 HOGENOM:HBG597219 InParanoid:Q9LX73 PhylomeDB:Q9LX73 ProtClustDB:PLN03083 Genevestigator:Q9LX73 InterPro:IPR018611 Pfam:PF09743 Uniprot:Q9LX73
Root	Isotig01304	17	40	-1.363	0.000639398	TAIR|locus:2029491 - symbol:RAP2.4 "AT1G78080" species:3702 "Arabidopsis thaliana" [GO:0006355 "regulation of transcription, DNA-dependent" evidence=ISS] [GO:0003700 "sequence-specific DNA binding transcription factor activity" evidence=ISS;IDA;TAS] [GO:0005515 "protein binding" evidence=IPI] [GO:0005634 "nucleus" evidence=IC;IDA] [GO:0006970 "response to osmotic stress" evidence=IEP] [GO:0071472 "cellular response to salt stress" evidence=IEP] [GO:0003677 "DNA binding" evidence=IDA;TAS] [GO:0009414 "response to water deprivation" evidence=IEP;IMP] [GO:0009416 "response to light stimulus" evidence=IEP] [GO:0009651 "response to salt stress" evidence=IEP] [GO:0009873 "ethylene mediated signaling pathway" evidence=IMP] [GO:0010017 "red or far-red light signaling pathway" evidence=IGI] [GO:0009611 "response to wounding" evidence=IEP] [GO:0009736 "cytokinin mediated signaling pathway" evidence=IMP] [GO:0045595 "regulation of cell differentiation" evidence=IMP] [GO:0009409 "response to cold" evidence=IEP] [GO:0043565 "sequence-specific DNA binding" evidence=IDA] InterPro:IPR001471 InterPro:IPR016177 Pfam:PF00847 PRINTS:PR00367 PROSITE:PS51032

Tissue	Identification	# of reads (stress)	# of reads (control)	Fold Change	p-value	Description
						SMART:SM00380 EMBL:CP002684 GenomeReviews:CT485782_GR GO:GO:0005634 GO:GO:0005515 GO:GO:0009611 GO:GO:0009873 GO:GO:0045595 GO:GO:0043565 GO:GO:0003700 GO:GO:0006351 GO:GO:0009409 GO:GO:0009414 KO:K09286 Gene3D:G3DSA:3.30.730.10 SUPFAM:SSF54171 HSSP:O80337 GO:GO:0009736 GO:GO:0071472 GO:GO:0010017 EMBL:AC009243 HOGENOM:HBG599504 EMBL:AC012680 UniGene:At.25524 EMBL:AF370152 EMBL:AY150468 EMBL:AF003097 IPI:IPI00519366 RefSeq:NP_177931.1 UniGene:At.71798 ProteinModelPortal:Q8H1E4 SMR:Q8H1E4 MINT:MINT-7262714 STRING:Q8H1E4 PRIDE:Q8H1E4 EnsemblPlants:AT1G78080.1 GeneID:844143 KEGG:ath:AT1G78080 TAIR:At1g78080 eggNOG:NOG306355 InParanoid:Q8H1E4 OMA:KLEAICK PhylomeDB:Q8H1E4 ProtClustDB:CLSN2679481 Genevestigator:Q8H1E4 Uniprot:Q8H1E4
Root	Isotig01311	6	25	-2.188	0.000173532	TAIR|locus:505006369 - symbol:ALDH2C4 "AT3G24503" species:3702 "Arabidopsis thaliana" [GO:0004028 "3-chloroallyl aldehyde dehydrogenase activity" evidence=ISS] [GO:0004029 "aldehyde dehydrogenase (NAD) activity" evidence=ISS] [GO:0009699 "phenylpropanoid biosynthetic process" evidence=IDA] [GO:0050269 "coniferyl-aldehyde dehydrogenase activity" evidence=IDA] InterPro:IPR015590 InterPro:IPR016160 InterPro:IPR016161 InterPro:IPR016162 InterPro:IPR016163 Pfam:PF00171 PROSITE:PS00070 PROSITE:PS00687 GO:GO:0005829 EMBL:CP002686 GenomeReviews:BA000014_GR GO:GO:0009699 eggNOG:COG1012 Gene3D:G3DSA:3.40.309.10 Gene3D:G3DSA:3.40.605.10 SUPFAM:SSF53720 HOGENOM:HBG752218 GO:GO:0004029 HSSP:P05091 EMBL:AF349448 EMBL:AB020746 EMBL:AY056398 EMBL:AK221230 IPI:IPI00543086 RefSeq:NP_566749.1 UniGene:At.22894 ProteinModelPortal:Q56YU0 SMR:Q56YU0 IntAct:Q56YU0 STRING:Q56YU0 PRIDE:Q56YU0 ProMEX:Q56YU0 EnsemblPlants:AT3G24503.1 GeneID:822042 KEGG:ath:AT3G24503 TAIR:At3g24503 InParanoid:Q56YU0 KO:K12355 OMA:EMAMEAG PhylomeDB:Q56YU0 ProtClustDB:PLN02766 Genevestigator:Q56YU0 GO:GO:0050269 Uniprot:Q56YU0
Root	Isotig01313	5	21	-2.199	0.000555521	TAIR|locus:505006369 - symbol:ALDH2C4 "AT3G24503" species:3702 "Arabidopsis thaliana" [GO:0004028 "3-chloroallyl aldehyde dehydrogenase activity" evidence=ISS] [GO:0004029 "aldehyde dehydrogenase (NAD) activity" evidence=ISS] [GO:0009699 "phenylpropanoid biosynthetic process" evidence=IDA] [GO:0050269 "coniferyl-aldehyde dehydrogenase activity" evidence=IDA] InterPro:IPR015590 InterPro:IPR016160 InterPro:IPR016161 InterPro:IPR016162 InterPro:IPR016163 Pfam:PF00171 PROSITE:PS00070 PROSITE:PS00687 GO:GO:0005829 EMBL:CP002686 GenomeReviews:BA000014_GR GO:GO:0009699 eggNOG:COG1012 Gene3D:G3DSA:3.40.309.10 Gene3D:G3DSA:3.40.605.10 SUPFAM:SSF53720 HOGENOM:HBG752218 GO:GO:0004029 HSSP:P05091 EMBL:AF349448 EMBL:AB020746 EMBL:AY056398 EMBL:AK221230 IPI:IPI00543086 RefSeq:NP_566749.1 UniGene:At.22894 ProteinModelPortal:Q56YU0 SMR:Q56YU0 IntAct:Q56YU0 STRING:Q56YU0 PRIDE:Q56YU0 ProMEX:Q56YU0 EnsemblPlants:AT3G24503.1 GeneID:822042 KEGG:ath:AT3G24503 TAIR:At3g24503 InParanoid:Q56YU0 KO:K12355 OMA:EMAMEAG PhylomeDB:Q56YU0 ProtClustDB:PLN02766 Genevestigator:Q56YU0 GO:GO:0050269 Uniprot:Q56YU0
Root	Isotig01336	65	18	1.723	7.69E-07	TAIR|locus:2193874 - symbol:AT1G55530 species:3702 "Arabidopsis thaliana" [GO:0008270 "zinc ion binding" evidence=IEA;ISS] InterPro:IPR001841 PROSITE:PS50089 SMART:SM00184 EMBL:CP002684 GenomeReviews:CT485782_GR GO:GO:0008270 InterPro:IPR013083 Gene3D:G3DSA:3.30.40.10 HSSP:Q9LRB7 EMBL:AC005223 eggNOG:NOG235630 HOGENOM:HBG749479 EMBL:AY039608 EMBL:AF424578 EMBL:BT000502 IPI:IPI00534528 PIR:F96597 RefSeq:NP_564693.1 UniGene:At.19898 ProteinModelPortal:Q9ZVU8 SMR:Q9ZVU8 STRING:Q9ZVU8 EnsemblPlants:AT1G55530.1 GeneID:842000 KEGG:ath:AT1G55530 TAIR:At1g55530 InParanoid:Q9ZVU8 OMA:FVEEMED PhylomeDB:Q9ZVU8 ProtClustDB:CLSN2917296 Genevestigator:Q9ZVU8 Uniprot:Q9ZVU8
Root	Isotig01337	65	18	1.723	7.69E-07	TAIR|locus:2193874 - symbol:AT1G55530 species:3702 "Arabidopsis thaliana" [GO:0008270 "zinc ion binding" evidence=IEA;ISS] InterPro:IPR001841 PROSITE:PS50089 SMART:SM00184 EMBL:CP002684 GenomeReviews:CT485782_GR GO:GO:0008270 InterPro:IPR013083 Gene3D:G3DSA:3.30.40.10 HSSP:Q9LRB7 EMBL:AC005223 eggNOG:NOG235630 HOGENOM:HBG749479 EMBL:AY039608 EMBL:AF424578 EMBL:BT000502 IPI:IPI00534528 PIR:F96597 RefSeq:NP_564693.1 UniGene:At.19898 ProteinModelPortal:Q9ZVU8 SMR:Q9ZVU8 STRING:Q9ZVU8 EnsemblPlants:AT1G55530.1 GeneID:842000 KEGG:ath:AT1G55530 TAIR:At1g55530 InParanoid:Q9ZVU8 OMA:FVEEMED PhylomeDB:Q9ZVU8 ProtClustDB:CLSN2917296 Genevestigator:Q9ZVU8 Uniprot:Q9ZVU8
Root	Isotig01340	63	16	1.848	3.27E-07	TAIR|locus:2193874 - symbol:AT1G55530 species:3702 "Arabidopsis thaliana" [GO:0008270 "zinc ion binding" evidence=IEA;ISS] InterPro:IPR001841 PROSITE:PS50089 SMART:SM00184 EMBL:CP002684 GenomeReviews:CT485782_GR GO:GO:0008270 InterPro:IPR013083 Gene3D:G3DSA:3.30.40.10 HSSP:Q9LRB7

Tissue	Identification	# of reads (stress)	# of reads (control)	Fold Change	p-value	Description
						EMBL:AC005223 eggNOG:NOG235630 HOGENOM:HBG749479 EMBL:AY039608 EMBL:AF424578 EMBL:BT000502 IPI:IPI00534528 PIR:F96597 RefSeq:NP_564693.1 UniGene:At.19898 ProteinModelPortal:Q9ZVU8 SMR:Q9ZVU8 STRING:Q9ZVU8 EnsemblPlants:AT1G55530.1 GeneID:842000 KEGG:ath:AT1G55530 TAIR:At1g55530 InParanoid:Q9ZVU8 OMA:FVEEMED PhylomeDB:Q9ZVU8 ProtClustDB:CLSN2917296 Genevestigator:Q9ZVU8 Uniprot:Q9ZVU8
Root	Isotig01341	63	16	1.848	3.27E-07	TAIR|locus:2193874 - symbol:AT1G55530 species:3702 "Arabidopsis thaliana" [GO:0008270 "zinc ion binding" evidence=IEA;ISS] InterPro:IPR001841 PROSITE:PS50089 SMART:SM00184 EMBL:CP002684 GenomeReviews:CT485782_GR GO:GO:0008270 InterPro:IPR013083 Gene3D:G3DSA:3.30.40.10 HSSP:Q9LRB7 EMBL:AC005223 eggNOG:NOG235630 HOGENOM:HBG749479 EMBL:AY039608 EMBL:AF424578 EMBL:BT000502 IPI:IPI00534528 PIR:F96597 RefSeq:NP_564693.1 UniGene:At.19898 ProteinModelPortal:Q9ZVU8 SMR:Q9ZVU8 STRING:Q9ZVU8 EnsemblPlants:AT1G55530.1 GeneID:842000 KEGG:ath:AT1G55530 TAIR:At1g55530 InParanoid:Q9ZVU8 OMA:FVEEMED PhylomeDB:Q9ZVU8 ProtClustDB:CLSN2917296 Genevestigator:Q9ZVU8 Uniprot:Q9ZVU8
Root	Isotig01345	26	69	-1.537	9.31E-07	TAIR|locus:2178843 - symbol:AT5G58620 "AT5G58620" species:3702 "Arabidopsis thaliana" [GO:0003700 "sequence-specific DNA binding transcription factor activity" evidence=ISS] [GO:0006355 "regulation of transcription, DNA-dependent" evidence=TAS] InterPro:IPR002110 InterPro:IPR000571 Pfam:PF00642 PROSITE:PS50088 PROSITE:PS50103 SMART:SM00248 SMART:SM00356 EMBL:CP002688 GenomeReviews:BA000015_GR GO:GO:0006355 GO:GO:0003677 GO:GO:0008270 InterPro:IPR020683 Gene3D:G3DSA:1.25.40.20 Pfam:PF12796 SUPFAM:SSF48403 PROSITE:PS50297 EMBL:AB020755 eggNOG:NOG69137 HOGENOM:HBG594924 EMBL:AY054176 EMBL:AY062491 EMBL:AY103314 EMBL:BT002083 IPI:IPI00544490 RefSeq:NP_200670.1 UniGene:At.49189 UniGene:At.68520 ProteinModelPortal:Q9LUZ4 SMR:Q9LUZ4 IntAct:Q9LUZ4 PRIDE:Q9LUZ4 EnsemblPlants:AT5G58620.1 GeneID:835976 KEGG:ath:AT5G58620 TAIR:At5g58620 InParanoid:Q9LUZ4 OMA:RSCGSDG PhylomeDB:Q9LUZ4 ProtClustDB:CLSN2914838 ArrayExpress:Q9LUZ4 Genevestigator:Q9LUZ4 Uniprot:Q9LUZ4
Root	Isotig01346	27	66	-1.418	6.20E-06	TAIR|locus:2178843 - symbol:AT5G58620 "AT5G58620" species:3702 "Arabidopsis thaliana" [GO:0003700 "sequence-specific DNA binding transcription factor activity" evidence=ISS] [GO:0006355 "regulation of transcription, DNA-dependent" evidence=TAS] InterPro:IPR002110 InterPro:IPR000571 Pfam:PF00642 PROSITE:PS50088 PROSITE:PS50103 SMART:SM00248 SMART:SM00356 EMBL:CP002688 GenomeReviews:BA000015_GR GO:GO:0006355 GO:GO:0003677 GO:GO:0008270 InterPro:IPR020683 Gene3D:G3DSA:1.25.40.20 Pfam:PF12796 SUPFAM:SSF48403 PROSITE:PS50297 EMBL:AB020755 eggNOG:NOG69137 HOGENOM:HBG594924 EMBL:AY054176 EMBL:AY062491 EMBL:AY103314 EMBL:BT002083 IPI:IPI00544490 RefSeq:NP_200670.1 UniGene:At.49189 UniGene:At.68520 ProteinModelPortal:Q9LUZ4 SMR:Q9LUZ4 IntAct:Q9LUZ4 PRIDE:Q9LUZ4 EnsemblPlants:AT5G58620.1 GeneID:835976 KEGG:ath:AT5G58620 TAIR:At5g58620 InParanoid:Q9LUZ4 OMA:RSCGSDG PhylomeDB:Q9LUZ4 ProtClustDB:CLSN2914838 ArrayExpress:Q9LUZ4 Genevestigator:Q9LUZ4 Uniprot:Q9LUZ4
Root	Isotig01347	26	65	-1.451	5.07E-06	TAIR|locus:2178843 - symbol:AT5G58620 "AT5G58620" species:3702 "Arabidopsis thaliana" [GO:0003700 "sequence-specific DNA binding transcription factor activity" evidence=ISS] [GO:0006355 "regulation of transcription, DNA-dependent" evidence=TAS] InterPro:IPR002110 InterPro:IPR000571 Pfam:PF00642 PROSITE:PS50088 PROSITE:PS50103 SMART:SM00248 SMART:SM00356 EMBL:CP002688 GenomeReviews:BA000015_GR GO:GO:0006355 GO:GO:0003677 GO:GO:0008270 InterPro:IPR020683 Gene3D:G3DSA:1.25.40.20 Pfam:PF12796 SUPFAM:SSF48403 PROSITE:PS50297 EMBL:AB020755 eggNOG:NOG69137 HOGENOM:HBG594924 EMBL:AY054176 EMBL:AY062491 EMBL:AY103314 EMBL:BT002083 IPI:IPI00544490 RefSeq:NP_200670.1 UniGene:At.49189 UniGene:At.68520 ProteinModelPortal:Q9LUZ4 SMR:Q9LUZ4 IntAct:Q9LUZ4 PRIDE:Q9LUZ4 EnsemblPlants:AT5G58620.1 GeneID:835976 KEGG:ath:AT5G58620 TAIR:At5g58620 InParanoid:Q9LUZ4 OMA:RSCGSDG PhylomeDB:Q9LUZ4 ProtClustDB:CLSN2914838 ArrayExpress:Q9LUZ4 Genevestigator:Q9LUZ4 Uniprot:Q9LUZ4
Root	Isotig01348	23	71	-1.755	4.46E-08	TAIR|locus:2178843 - symbol:AT5G58620 "AT5G58620" species:3702 "Arabidopsis thaliana" [GO:0003700 "sequence-specific DNA binding transcription factor activity" evidence=ISS] [GO:0006355 "regulation of transcription, DNA-dependent" evidence=TAS] InterPro:IPR002110 InterPro:IPR000571 Pfam:PF00642 PROSITE:PS50088 PROSITE:PS50103 SMART:SM00248 SMART:SM00356 EMBL:CP002688 GenomeReviews:BA000015_GR GO:GO:0006355 GO:GO:0003677 GO:GO:0008270 InterPro:IPR020683 Gene3D:G3DSA:1.25.40.20 Pfam:PF12796 SUPFAM:SSF48403 PROSITE:PS50297 EMBL:AB020755 eggNOG:NOG69137

Tissue	Identification	# of reads (stress)	# of reads (control)	Fold Change	p-value	Description
						HOGENOM:HBG594924 EMBL:AY054176 EMBL:AY062491 EMBL:AY103314 EMBL:BT002083 IPI:IPI00544490 RefSeq:NP_200670.1 UniGene:At.49189 UniGene:At.68520 ProteinModelPortal:Q9LUZ4 SMR:Q9LUZ4 IntAct:Q9LUZ4 PRIDE:Q9LUZ4 EnsemblPlants:AT5G58620.1 GeneID:835976 KEGG:ath:AT5G58620 TAIR:At5g58620 InParanoid:Q9LUZ4 OMA:RSCGSDG PhylomeDB:Q9LUZ4 ProtClustDB:CLSN2914838 ArrayExpress:Q9LUZ4 Genevestigator:Q9LUZ4 Uniprot:Q9LUZ4
Root	Isotig01349	24	68	-1.631	3.67E-07	TAIR|locus:2178843 - symbol:AT5G58620 "AT5G58620" species:3702 "Arabidopsis thaliana" [GO:0003700 "sequence-specific DNA binding transcription factor activity" evidence=ISS] [GO:0006355 "regulation of transcription, DNA-dependent" evidence=TAS] InterPro:IPR002110 InterPro:IPR000571 Pfam:PF00642 PROSITE:PS50088 PROSITE:PS50103 SMART:SM00248 SMART:SM00356 EMBL:CP002688 GenomeReviews:BA000015_GR GO:GO:0006355 GO:GO:0003677 GO:GO:0008270 InterPro:IPR020683 Gene3D:G3DSA:1.25.40.20 Pfam:PF12796 SUPFAM:SSF48403 PROSITE:PS50297 EMBL:AB020755 eggNOG:NOG69137 HOGENOM:HBG594924 EMBL:AY054176 EMBL:AY062491 EMBL:AY103314 EMBL:BT002083 IPI:IPI00544490 RefSeq:NP_200670.1 UniGene:At.49189 UniGene:At.68520 ProteinModelPortal:Q9LUZ4 SMR:Q9LUZ4 IntAct:Q9LUZ4 PRIDE:Q9LUZ4 EnsemblPlants:AT5G58620.1 GeneID:835976 KEGG:ath:AT5G58620 TAIR:At5g58620 InParanoid:Q9LUZ4 OMA:RSCGSDG PhylomeDB:Q9LUZ4 ProtClustDB:CLSN2914838 ArrayExpress:Q9LUZ4 Genevestigator:Q9LUZ4 Uniprot:Q9LUZ4
Root	Isotig01350	30	67	-1.288	2.34E-05	TAIR|locus:2178843 - symbol:AT5G58620 "AT5G58620" species:3702 "Arabidopsis thaliana" [GO:0003700 "sequence-specific DNA binding transcription factor activity" evidence=ISS] [GO:0006355 "regulation of transcription, DNA-dependent" evidence=TAS] InterPro:IPR002110 InterPro:IPR000571 Pfam:PF00642 PROSITE:PS50088 PROSITE:PS50103 SMART:SM00248 SMART:SM00356 EMBL:CP002688 GenomeReviews:BA000015_GR GO:GO:0006355 GO:GO:0003677 GO:GO:0008270 InterPro:IPR020683 Gene3D:G3DSA:1.25.40.20 Pfam:PF12796 SUPFAM:SSF48403 PROSITE:PS50297 EMBL:AB020755 eggNOG:NOG69137 HOGENOM:HBG594924 EMBL:AY054176 EMBL:AY062491 EMBL:AY103314 EMBL:BT002083 IPI:IPI00544490 RefSeq:NP_200670.1 UniGene:At.49189 UniGene:At.68520 ProteinModelPortal:Q9LUZ4 SMR:Q9LUZ4 IntAct:Q9LUZ4 PRIDE:Q9LUZ4 EnsemblPlants:AT5G58620.1 GeneID:835976 KEGG:ath:AT5G58620 TAIR:At5g58620 InParanoid:Q9LUZ4 OMA:RSCGSDG PhylomeDB:Q9LUZ4 ProtClustDB:CLSN2914838 ArrayExpress:Q9LUZ4 Genevestigator:Q9LUZ4 Uniprot:Q9LUZ4
Root	Isotig01351	31	64	-1.175	0.000120563	TAIR|locus:2178843 - symbol:AT5G58620 "AT5G58620" species:3702 "Arabidopsis thaliana" [GO:0003700 "sequence-specific DNA binding transcription factor activity" evidence=ISS] [GO:0006355 "regulation of transcription, DNA-dependent" evidence=TAS] InterPro:IPR002110 InterPro:IPR000571 Pfam:PF00642 PROSITE:PS50088 PROSITE:PS50103 SMART:SM00248 SMART:SM00356 EMBL:CP002688 GenomeReviews:BA000015_GR GO:GO:0006355 GO:GO:0003677 GO:GO:0008270 InterPro:IPR020683 Gene3D:G3DSA:1.25.40.20 Pfam:PF12796 SUPFAM:SSF48403 PROSITE:PS50297 EMBL:AB020755 eggNOG:NOG69137 HOGENOM:HBG594924 EMBL:AY054176 EMBL:AY062491 EMBL:AY103314 EMBL:BT002083 IPI:IPI00544490 RefSeq:NP_200670.1 UniGene:At.49189 UniGene:At.68520 ProteinModelPortal:Q9LUZ4 SMR:Q9LUZ4 IntAct:Q9LUZ4 PRIDE:Q9LUZ4 EnsemblPlants:AT5G58620.1 GeneID:835976 KEGG:ath:AT5G58620 TAIR:At5g58620 InParanoid:Q9LUZ4 OMA:RSCGSDG PhylomeDB:Q9LUZ4 ProtClustDB:CLSN2914838 ArrayExpress:Q9LUZ4 Genevestigator:Q9LUZ4 Uniprot:Q9LUZ4
Root	Isotig01352	23	67	-1.672	2.81E-07	TAIR|locus:2178843 - symbol:AT5G58620 "AT5G58620" species:3702 "Arabidopsis thaliana" [GO:0003700 "sequence-specific DNA binding transcription factor activity" evidence=ISS] [GO:0006355 "regulation of transcription, DNA-dependent" evidence=TAS] InterPro:IPR002110 InterPro:IPR000571 Pfam:PF00642 PROSITE:PS50088 PROSITE:PS50103 SMART:SM00248 SMART:SM00356 EMBL:CP002688 GenomeReviews:BA000015_GR GO:GO:0006355 GO:GO:0003677 GO:GO:0008270 InterPro:IPR020683 Gene3D:G3DSA:1.25.40.20 Pfam:PF12796 SUPFAM:SSF48403 PROSITE:PS50297 EMBL:AB020755 eggNOG:NOG69137 HOGENOM:HBG594924 EMBL:AY054176 EMBL:AY062491 EMBL:AY103314 EMBL:BT002083 IPI:IPI00544490 RefSeq:NP_200670.1 UniGene:At.49189 UniGene:At.68520 ProteinModelPortal:Q9LUZ4 SMR:Q9LUZ4 IntAct:Q9LUZ4 PRIDE:Q9LUZ4 EnsemblPlants:AT5G58620.1 GeneID:835976 KEGG:ath:AT5G58620 TAIR:At5g58620 InParanoid:Q9LUZ4 OMA:RSCGSDG PhylomeDB:Q9LUZ4 ProtClustDB:CLSN2914838 ArrayExpress:Q9LUZ4 Genevestigator:Q9LUZ4 Uniprot:Q9LUZ4
Root	Isotig01353	30	63	-1.199	0.000105278	TAIR|locus:2178843 - symbol:AT5G58620 "AT5G58620" species:3702 "Arabidopsis thaliana" [GO:0003700 "sequence-specific DNA binding transcription factor activity" evidence=ISS] [GO:0006355 "regulation of transcription, DNA-dependent" evidence=TAS] InterPro:IPR002110 InterPro:IPR000571 Pfam:PF00642 PROSITE:PS50088

Tissue	Identification	# of reads (stress)	# of reads (control)	Fold Change	p-value	Description
						PROSITE:PS50103 SMART:SM00248 SMART:SM00356 EMBL:CP002688 GenomeReviews:BA000015_GR GO:GO:0006355 GO:GO:0003677 GO:GO:0008270 InterPro:IPR020683 Gene3D:G3DSA:1.25.40.20 Pfam:PF12796 SUPFAM:SSF48403 PROSITE:PS50297 EMBL:AB020755 eggNOG:NOG69137 HOGENOM:HBG594924 EMBL:AY054176 EMBL:AY062491 EMBL:AY103314 EMBL:BT002083 IPI:IPI00544490 RefSeq:NP_200670.1 UniGene:At.49189 UniGene:At.68520 ProteinModelPortal:Q9LUZ4 SMR:Q9LUZ4 IntAct:Q9LUZ4 PRIDE:Q9LUZ4 EnsemblPlants:AT5G58620.1 GeneID:835976 KEGG:ath:AT5G58620 TAIR:At5g58620 InParanoid:Q9LUZ4 OMA:RSCGSDG PhylomeDB:Q9LUZ4 ProtClustDB:CLSN2914838 ArrayExpress:Q9LUZ4 Genevestigator:Q9LUZ4 Uniprot:Q9LUZ4
Root	Isotig01354	53	20	1.277	0.000352505	TAIR|locus:2052005 - symbol:OZF1 "AT2G19810" species:3702 "Arabidopsis thaliana" [GO:0003676 "nucleic acid binding" evidence=ISS] [GO:0003700 "sequence-specific DNA binding transcription factor activity" evidence=ISS] [GO:0006355 "regulation of transcription, DNA-dependent" evidence=TAS] [GO:0005886 "plasma membrane" evidence=IDA] [GO:0006979 "response to oxidative stress" evidence=IMP] InterPro:IPR000571 PROSITE:PS50103 SMART:SM00356 GO:GO:0005886 EMBL:CP002685 GenomeReviews:CT485783_GR GO:GO:0006979 GO:GO:0006355 GO:GO:0003677 GO:GO:0008270 EMBL:AC005169 EMBL:AY136395 EMBL:BT002114 EMBL:AY084253 IPI:IPI00535519 PIR:D84581 RefSeq:NP_179571.1 UniGene:At.12895 ProteinModelPortal:O82199 SMR:O82199 PRIDE:O82199 EnsemblPlants:AT2G19810.1 GeneID:816500 KEGG:ath:AT2G19810 TAIR:At2g19810 eggNOG:NOG288631 HOGENOM:HBG744620 InParanoid:O82199 OMA:WENGLEE PhylomeDB:O82199 ProtClustDB:CLSN2691160 ArrayExpress:O82199 Genevestigator:O82199 Uniprot:O82199
Root	Isotig01355	51	20	1.222	0.000710992	TAIR|locus:2052005 - symbol:OZF1 "AT2G19810" species:3702 "Arabidopsis thaliana" [GO:0003676 "nucleic acid binding" evidence=ISS] [GO:0003700 "sequence-specific DNA binding transcription factor activity" evidence=ISS] [GO:0006355 "regulation of transcription, DNA-dependent" evidence=TAS] [GO:0005886 "plasma membrane" evidence=IDA] [GO:0006979 "response to oxidative stress" evidence=IMP] InterPro:IPR000571 PROSITE:PS50103 SMART:SM00356 GO:GO:0005886 EMBL:CP002685 GenomeReviews:CT485783_GR GO:GO:0006979 GO:GO:0006355 GO:GO:0003677 GO:GO:0008270 EMBL:AC005169 EMBL:AY136395 EMBL:BT002114 EMBL:AY084253 IPI:IPI00535519 PIR:D84581 RefSeq:NP_179571.1 UniGene:At.12895 ProteinModelPortal:O82199 SMR:O82199 PRIDE:O82199 EnsemblPlants:AT2G19810.1 GeneID:816500 KEGG:ath:AT2G19810 TAIR:At2g19810 eggNOG:NOG288631 HOGENOM:HBG744620 InParanoid:O82199 OMA:WENGLEE PhylomeDB:O82199 ProtClustDB:CLSN2691160 ArrayExpress:O82199 Genevestigator:O82199 Uniprot:O82199
Root	Isotig01356	46	17	1.307	0.000699665	TAIR|locus:2052005 - symbol:OZF1 "AT2G19810" species:3702 "Arabidopsis thaliana" [GO:0003676 "nucleic acid binding" evidence=ISS] [GO:0003700 "sequence-specific DNA binding transcription factor activity" evidence=ISS] [GO:0006355 "regulation of transcription, DNA-dependent" evidence=TAS] [GO:0005886 "plasma membrane" evidence=IDA] [GO:0006979 "response to oxidative stress" evidence=IMP] InterPro:IPR000571 PROSITE:PS50103 SMART:SM00356 GO:GO:0005886 EMBL:CP002685 GenomeReviews:CT485783_GR GO:GO:0006979 GO:GO:0006355 GO:GO:0003677 GO:GO:0008270 EMBL:AC005169 EMBL:AY136395 EMBL:BT002114 EMBL:AY084253 IPI:IPI00535519 PIR:D84581 RefSeq:NP_179571.1 UniGene:At.12895 ProteinModelPortal:O82199 SMR:O82199 PRIDE:O82199 EnsemblPlants:AT2G19810.1 GeneID:816500 KEGG:ath:AT2G19810 TAIR:At2g19810 eggNOG:NOG288631 HOGENOM:HBG744620 InParanoid:O82199 OMA:WENGLEE PhylomeDB:O82199 ProtClustDB:CLSN2691160 ArrayExpress:O82199 Genevestigator:O82199 Uniprot:O82199
Root	Isotig01358	51	20	1.222	0.000710992	TAIR|locus:2052005 - symbol:OZF1 "AT2G19810" species:3702 "Arabidopsis thaliana" [GO:0003676 "nucleic acid binding" evidence=ISS] [GO:0003700 "sequence-specific DNA binding transcription factor activity" evidence=ISS] [GO:0006355 "regulation of transcription, DNA-dependent" evidence=TAS] [GO:0005886 "plasma membrane" evidence=IDA] [GO:0006979 "response to oxidative stress" evidence=IMP] InterPro:IPR000571 PROSITE:PS50103 SMART:SM00356 GO:GO:0005886 EMBL:CP002685 GenomeReviews:CT485783_GR GO:GO:0006979 GO:GO:0006355 GO:GO:0003677 GO:GO:0008270 EMBL:AC005169 EMBL:AY136395 EMBL:BT002114 EMBL:AY084253 IPI:IPI00535519 PIR:D84581 RefSeq:NP_179571.1 UniGene:At.12895 ProteinModelPortal:O82199 SMR:O82199 PRIDE:O82199 EnsemblPlants:AT2G19810.1 GeneID:816500 KEGG:ath:AT2G19810 TAIR:At2g19810 eggNOG:NOG288631 HOGENOM:HBG744620 InParanoid:O82199 OMA:WENGLEE PhylomeDB:O82199 ProtClustDB:CLSN2691160 ArrayExpress:O82199 Genevestigator:O82199 Uniprot:O82199

Tissue	Identification	# of reads (stress)	# of reads (control)	Fold Change	p-value	Description
Root	Isotig01360	48	18	1.286	0.000627857	TAIR|locus:2052005 - symbol:OZF1 "AT2G19810" species:3702 "Arabidopsis thaliana" [GO:0003676 "nucleic acid binding" evidence=ISS] [GO:0003700 "sequence-specific DNA binding transcription factor activity" evidence=ISS] [GO:0006355 "regulation of transcription, DNA-dependent" evidence=TAS] [GO:0005886 "plasma membrane" evidence=IDA] [GO:0006979 "response to oxidative stress" evidence=IMP] InterPro:IPR000571 PROSITE:PS50103 SMART:SM00356 GO:GO:0005886 EMBL:CP002685 GenomeReviews:CT485783_GR GO:GO:0006979 GO:GO:0006355 GO:GO:0003677 GO:GO:0008270 EMBL:AC005169 EMBL:AY136395 EMBL:BT002114 EMBL:AY084253 IPI:IPI00535519 PIR:D84581 RefSeq:NP_179571.1 UniGene:At.12895 ProteinModelPortal:O82199 SMR:O82199 PRIDE:O82199 EnsemblPlants:AT2G19810.1 GeneID:816500 KEGG:ath:AT2G19810 TAIR:At2g19810 eggNOG:NOG288631 HOGENOM:HBG744620 InParanoid:O82199 OMA:WENGLEE PhylomeDB:O82199 ProtClustDB:CLSN2691160 ArrayExpress:O82199 Genevestigator:O82199 Uniprot:O82199
Root	Isotig01368	16	55	-1.910	3.35E-07	TAIR|locus:2170867 - symbol:APS4 "AT5G43780" species:3702 "Arabidopsis thaliana" [GO:0004781 "sulfate adenylyltransferase (ATP) activity" evidence=ISS;IMP] [GO:0009507 "chloroplast" evidence=IDA] [GO:0000103 "sulfate assimilation" evidence=TAS] [GO:0005739 "mitochondrion" evidence=IDA] [GO:0009570 "chloroplast stroma" evidence=IDA] InterPro:IPR002650 GO:GO:0005739 GO:GO:0005524 GO:GO:0009570 EMBL:CP002688 EMBL:AB026651 GO:GO:0000103 InterPro:IPR014729 Gene3D:G3DSA:3.40.50.620 KO:K13811 ProtClustDB:CLSN2684174 GO:GO:0004781 InterPro:IPR015947 SUPFAM:SSF88697 TIGRFAMs:TIGR00339 EMBL:AF110407 EMBL:AJ012586 EMBL:AY072193 EMBL:AY117323 EMBL:AK226204 IPI:IPI00531868 PIR:T52659 RefSeq:NP_199191.1 UniGene:At.23710 UniGene:At.66691 ProteinModelPortal:Q9S7D8 SMR:Q9S7D8 STRING:Q9S7D8 PRIDE:Q9S7D8 GeneID:834400 KEGG:ath:AT5G43780 TAIR:At5g43780 InParanoid:Q9S7D8 OMA:GITPMFI PhylomeDB:Q9S7D8 ArrayExpress:Q9S7D8 Genevestigator:Q9S7D8 Uniprot:Q9S7D8
Root	Isotig01370	16	55	-1.910	3.35E-07	TAIR|locus:2170867 - symbol:APS4 "AT5G43780" species:3702 "Arabidopsis thaliana" [GO:0004781 "sulfate adenylyltransferase (ATP) activity" evidence=ISS;IMP] [GO:0009507 "chloroplast" evidence=IDA] [GO:0000103 "sulfate assimilation" evidence=TAS] [GO:0005739 "mitochondrion" evidence=IDA] [GO:0009570 "chloroplast stroma" evidence=IDA] InterPro:IPR002650 GO:GO:0005739 GO:GO:0005524 GO:GO:0009570 EMBL:CP002688 EMBL:AB026651 GO:GO:0000103 InterPro:IPR014729 Gene3D:G3DSA:3.40.50.620 KO:K13811 ProtClustDB:CLSN2684174 GO:GO:0004781 InterPro:IPR015947 SUPFAM:SSF88697 TIGRFAMs:TIGR00339 EMBL:AF110407 EMBL:AJ012586 EMBL:AY072193 EMBL:AY117323 EMBL:AK226204 IPI:IPI00531868 PIR:T52659 RefSeq:NP_199191.1 UniGene:At.23710 UniGene:At.66691 ProteinModelPortal:Q9S7D8 SMR:Q9S7D8 STRING:Q9S7D8 PRIDE:Q9S7D8 GeneID:834400 KEGG:ath:AT5G43780 TAIR:At5g43780 InParanoid:Q9S7D8 OMA:GITPMFI PhylomeDB:Q9S7D8 ArrayExpress:Q9S7D8 Genevestigator:Q9S7D8 Uniprot:Q9S7D8
Root	Isotig01371	16	55	-1.910	3.35E-07	TAIR|locus:2170867 - symbol:APS4 "AT5G43780" species:3702 "Arabidopsis thaliana" [GO:0004781 "sulfate adenylyltransferase (ATP) activity" evidence=ISS;IMP] [GO:0009507 "chloroplast" evidence=IDA] [GO:0000103 "sulfate assimilation" evidence=TAS] [GO:0005739 "mitochondrion" evidence=IDA] [GO:0009570 "chloroplast stroma" evidence=IDA] InterPro:IPR002650 GO:GO:0005739 GO:GO:0005524 GO:GO:0009570 EMBL:CP002688 EMBL:AB026651 GO:GO:0000103 InterPro:IPR014729 Gene3D:G3DSA:3.40.50.620 KO:K13811 ProtClustDB:CLSN2684174 GO:GO:0004781 InterPro:IPR015947 SUPFAM:SSF88697 TIGRFAMs:TIGR00339 EMBL:AF110407 EMBL:AJ012586 EMBL:AY072193 EMBL:AY117323 EMBL:AK226204 IPI:IPI00531868 PIR:T52659 RefSeq:NP_199191.1 UniGene:At.23710 UniGene:At.66691 ProteinModelPortal:Q9S7D8 SMR:Q9S7D8 STRING:Q9S7D8 PRIDE:Q9S7D8 GeneID:834400 KEGG:ath:AT5G43780 TAIR:At5g43780 InParanoid:Q9S7D8 OMA:GITPMFI PhylomeDB:Q9S7D8 ArrayExpress:Q9S7D8 Genevestigator:Q9S7D8 Uniprot:Q9S7D8
Root	Isotig01372	4	53	-3.857	5.31E-13	TAIR|locus:2179887 - symbol:G6PD2 "AT5G13110" species:3702 "Arabidopsis thaliana" [GO:0004345 "glucose-6-phosphate dehydrogenase activity" evidence=ISS;IDA] [GO:0006006 "glucose metabolic process" evidence=ISS;IDA] [GO:0009051 "pentose-phosphate shunt, oxidative branch" evidence=IDA] [GO:0009507 "chloroplast" evidence=ISS] [GO:0009570 "chloroplast stroma" evidence=IDA] InterPro:IPR001282 InterPro:IPR016040 InterPro:IPR019796 InterPro:IPR022674 InterPro:IPR022675 Pfam:PF00479 Pfam:PF02781 PRINTS:PR00079 PROSITE:PS00069 GO:GO:0009570 EMBL:CP002688 GenomeReviews:BA000015_GR Gene3D:G3DSA:3.40.50.720 GO:GO:0050661 GO:GO:0009051 EMBL:AL391711 eggNOG:COG0364 HOGENOM:HBG322449 KO:K00036 GO:GO:0004345 PANTHER:PTHR23429 TIGRFAMs:TIGR00871

Tissue	Identification	# of reads (stress)	# of reads (control)	Fold Change	p-value	Description
						EMBL:AY065042 EMBL:X84229 IPI:IPI00531306 PIR:S71245 RefSeq:NP_196815.2 UniGene:At.61 ProteinModelPortal:Q9FY99 SMR:Q9FY99 IntAct:Q9FY99 STRING:Q9FY99 PRIDE:Q9FY99 EnsemblPlants:AT5G13110.1 GeneID:831150 KEGG:ath:AT5G13110 TAIR:At5g13110 InParanoid:Q9FY99 OMA:FANQMFE PhylomeDB:Q9FY99 ProtClustDB:PLN02333 ArrayExpress:Q9FY99 Genevestigator:Q9FY99 GermOnline:AT5G13110 Uniprot:Q9FY99
Root	Isotig01373	5	55	-3.588	7.73E-13	TAIR|locus:2179887 - symbol:G6PD2 "AT5G13110" species:3702 "Arabidopsis thaliana" [GO:0004345 "glucose-6-phosphate dehydrogenase activity" evidence=ISS;IDA] [GO:0006006 "glucose metabolic process" evidence=ISS;IDA] [GO:0009051 "pentose-phosphate shunt, oxidative branch" evidence=IDA] [GO:0009507 "chloroplast" evidence=ISS] [GO:0009570 "chloroplast stroma" evidence=IDA] InterPro:IPR001282 InterPro:IPR016040 InterPro:IPR019796 InterPro:IPR022674 InterPro:IPR022675 Pfam:PF00479 Pfam:PF02781 PRINTS:PR00079 PROSITE:PS00069 GO:GO:0009570 EMBL:CP002688 GenomeReviews:BA000015_GR Gene3D:G3DSA:3.40.50.720 GO:GO:0050661 GO:GO:0009051 EMBL:AL391711 eggNOG:COG0364 HOGENOM:HBG322449 KO:K00036 GO:GO:0004345 PANTHER:PTHR23429 TIGRFAMs:TIGR00871 EMBL:AY065042 EMBL:X84229 IPI:IPI00531306 PIR:S71245 RefSeq:NP_196815.2 UniGene:At.61 ProteinModelPortal:Q9FY99 SMR:Q9FY99 IntAct:Q9FY99 STRING:Q9FY99 PRIDE:Q9FY99 EnsemblPlants:AT5G13110.1 GeneID:831150 KEGG:ath:AT5G13110 TAIR:At5g13110 InParanoid:Q9FY99 OMA:FANQMFE PhylomeDB:Q9FY99 ProtClustDB:PLN02333 ArrayExpress:Q9FY99 Genevestigator:Q9FY99 GermOnline:AT5G13110 Uniprot:Q9FY99
Root	Isotig01374	4	54	-3.884	2.85E-13	TAIR|locus:2179887 - symbol:G6PD2 "AT5G13110" species:3702 "Arabidopsis thaliana" [GO:0004345 "glucose-6-phosphate dehydrogenase activity" evidence=ISS;IDA] [GO:0006006 "glucose metabolic process" evidence=ISS;IDA] [GO:0009051 "pentose-phosphate shunt, oxidative branch" evidence=IDA] [GO:0009507 "chloroplast" evidence=ISS] [GO:0009570 "chloroplast stroma" evidence=IDA] InterPro:IPR001282 InterPro:IPR016040 InterPro:IPR019796 InterPro:IPR022674 InterPro:IPR022675 Pfam:PF00479 Pfam:PF02781 PRINTS:PR00079 PROSITE:PS00069 GO:GO:0009570 EMBL:CP002688 GenomeReviews:BA000015_GR Gene3D:G3DSA:3.40.50.720 GO:GO:0050661 GO:GO:0009051 EMBL:AL391711 eggNOG:COG0364 HOGENOM:HBG322449 KO:K00036 GO:GO:0004345 PANTHER:PTHR23429 TIGRFAMs:TIGR00871 EMBL:AY065042 EMBL:X84229 IPI:IPI00531306 PIR:S71245 RefSeq:NP_196815.2 UniGene:At.61 ProteinModelPortal:Q9FY99 SMR:Q9FY99 IntAct:Q9FY99 STRING:Q9FY99 PRIDE:Q9FY99 EnsemblPlants:AT5G13110.1 GeneID:831150 KEGG:ath:AT5G13110 TAIR:At5g13110 InParanoid:Q9FY99 OMA:FANQMFE PhylomeDB:Q9FY99 ProtClustDB:PLN02333 ArrayExpress:Q9FY99 Genevestigator:Q9FY99 GermOnline:AT5G13110 Uniprot:Q9FY99
Root	Isotig01375	5	56	-3.614	4.16E-13	TAIR|locus:2179887 - symbol:G6PD2 "AT5G13110" species:3702 "Arabidopsis thaliana" [GO:0004345 "glucose-6-phosphate dehydrogenase activity" evidence=ISS;IDA] [GO:0006006 "glucose metabolic process" evidence=ISS;IDA] [GO:0009051 "pentose-phosphate shunt, oxidative branch" evidence=IDA] [GO:0009507 "chloroplast" evidence=ISS] [GO:0009570 "chloroplast stroma" evidence=IDA] InterPro:IPR001282 InterPro:IPR016040 InterPro:IPR019796 InterPro:IPR022674 InterPro:IPR022675 Pfam:PF00479 Pfam:PF02781 PRINTS:PR00079 PROSITE:PS00069 GO:GO:0009570 EMBL:CP002688 GenomeReviews:BA000015_GR Gene3D:G3DSA:3.40.50.720 GO:GO:0050661 GO:GO:0009051 EMBL:AL391711 eggNOG:COG0364 HOGENOM:HBG322449 KO:K00036 GO:GO:0004345 PANTHER:PTHR23429 TIGRFAMs:TIGR00871 EMBL:AY065042 EMBL:X84229 IPI:IPI00531306 PIR:S71245 RefSeq:NP_196815.2 UniGene:At.61 ProteinModelPortal:Q9FY99 SMR:Q9FY99 IntAct:Q9FY99 STRING:Q9FY99 PRIDE:Q9FY99 EnsemblPlants:AT5G13110.1 GeneID:831150 KEGG:ath:AT5G13110 TAIR:At5g13110 InParanoid:Q9FY99 OMA:FANQMFE PhylomeDB:Q9FY99 ProtClustDB:PLN02333 ArrayExpress:Q9FY99 Genevestigator:Q9FY99 GermOnline:AT5G13110 Uniprot:Q9FY99
Root	Isotig01376	4	53	-3.857	5.31E-13	TAIR|locus:2179887 - symbol:G6PD2 "AT5G13110" species:3702 "Arabidopsis thaliana" [GO:0004345 "glucose-6-phosphate dehydrogenase activity" evidence=ISS;IDA] [GO:0006006 "glucose metabolic process" evidence=ISS;IDA] [GO:0009051 "pentose-phosphate shunt, oxidative branch" evidence=IDA] [GO:0009507 "chloroplast" evidence=ISS] [GO:0009570 "chloroplast stroma" evidence=IDA] InterPro:IPR001282 InterPro:IPR016040 InterPro:IPR019796 InterPro:IPR022674 InterPro:IPR022675 Pfam:PF00479 Pfam:PF02781 PRINTS:PR00079 PROSITE:PS00069 GO:GO:0009570 EMBL:CP002688 GenomeReviews:BA000015_GR Gene3D:G3DSA:3.40.50.720 GO:GO:0050661 GO:GO:0009051 EMBL:AL391711 eggNOG:COG0364 HOGENOM:HBG322449 KO:K00036 GO:GO:0004345 PANTHER:PTHR23429 TIGRFAMs:TIGR00871

Tissue	Identification	# of reads (stress)	# of reads (control)	Fold Change	p-value	Description
						EMBL:AY065042 EMBL:X84229 IPI:IPI00531306 PIR:S71245 RefSeq:NP_196815.2 UniGene:At.61 ProteinModelPortal:Q9FY99 SMR:Q9FY99 IntAct:Q9FY99 STRING:Q9FY99 PRIDE:Q9FY99 EnsemblPlants:AT5G13110.1 GeneID:831150 KEGG:ath:AT5G13110 TAIR:At5g13110 InParanoid:Q9FY99 OMA:FANQMFE PhylomeDB:Q9FY99 ProtClustDB:PLN02333 ArrayExpress:Q9FY99 Genevestigator:Q9FY99 GermOnline:AT5G13110 Uniprot:Q9FY99
Root	Isotig01377	5	55	-3.588	7.73E-13	TAIR|locus:2179887 - symbol:G6PD2 "AT5G13110" species:3702 "Arabidopsis thaliana" [GO:0004345 "glucose-6-phosphate dehydrogenase activity" evidence=ISS;IDA] [GO:0006006 "glucose metabolic process" evidence=ISS;IDA] [GO:0009051 "pentose-phosphate shunt, oxidative branch" evidence=IDA] [GO:0009507 "chloroplast" evidence=ISS] [GO:0009570 "chloroplast stroma" evidence=IDA] InterPro:IPR001282 InterPro:IPR016040 InterPro:IPR019796 InterPro:IPR022674 InterPro:IPR022675 Pfam:PF00479 Pfam:PF02781 PRINTS:PR00079 PROSITE:PS00069 GO:GO:0009570 EMBL:CP002688 GenomeReviews:BA000015_GR Gene3D:G3DSA:3.40.50.720 GO:GO:0050661 GO:GO:0009051 EMBL:AL391711 eggNOG:COG0364 HOGENOM:HBG322449 KO:K00036 GO:GO:0004345 PANTHER:PTHR23429 TIGRFAMs:TIGR00871 EMBL:AY065042 EMBL:X84229 IPI:IPI00531306 PIR:S71245 RefSeq:NP_196815.2 UniGene:At.61 ProteinModelPortal:Q9FY99 SMR:Q9FY99 IntAct:Q9FY99 STRING:Q9FY99 PRIDE:Q9FY99 EnsemblPlants:AT5G13110.1 GeneID:831150 KEGG:ath:AT5G13110 TAIR:At5g13110 InParanoid:Q9FY99 OMA:FANQMFE PhylomeDB:Q9FY99 ProtClustDB:PLN02333 ArrayExpress:Q9FY99 Genevestigator:Q9FY99 GermOnline:AT5G13110 Uniprot:Q9FY99
Root	Isotig01378	1	26	-4.829	8.91E-08	TAIR|locus:2179887 - symbol:G6PD2 "AT5G13110" species:3702 "Arabidopsis thaliana" [GO:0004345 "glucose-6-phosphate dehydrogenase activity" evidence=ISS;IDA] [GO:0006006 "glucose metabolic process" evidence=ISS;IDA] [GO:0009051 "pentose-phosphate shunt, oxidative branch" evidence=IDA] [GO:0009507 "chloroplast" evidence=ISS] [GO:0009570 "chloroplast stroma" evidence=IDA] InterPro:IPR001282 InterPro:IPR016040 InterPro:IPR019796 InterPro:IPR022674 InterPro:IPR022675 Pfam:PF00479 Pfam:PF02781 PRINTS:PR00079 PROSITE:PS00069 GO:GO:0009570 EMBL:CP002688 GenomeReviews:BA000015_GR Gene3D:G3DSA:3.40.50.720 GO:GO:0050661 GO:GO:0009051 EMBL:AL391711 eggNOG:COG0364 HOGENOM:HBG322449 KO:K00036 GO:GO:0004345 PANTHER:PTHR23429 TIGRFAMs:TIGR00871 EMBL:AY065042 EMBL:X84229 IPI:IPI00531306 PIR:S71245 RefSeq:NP_196815.2 UniGene:At.61 ProteinModelPortal:Q9FY99 SMR:Q9FY99 IntAct:Q9FY99 STRING:Q9FY99 PRIDE:Q9FY99 EnsemblPlants:AT5G13110.1 GeneID:831150 KEGG:ath:AT5G13110 TAIR:At5g13110 InParanoid:Q9FY99 OMA:FANQMFE PhylomeDB:Q9FY99 ProtClustDB:PLN02333 ArrayExpress:Q9FY99 Genevestigator:Q9FY99 GermOnline:AT5G13110 Uniprot:Q9FY99
Root	Isotig01379	2	28	-3.936	1.29E-07	TAIR|locus:2179887 - symbol:G6PD2 "AT5G13110" species:3702 "Arabidopsis thaliana" [GO:0004345 "glucose-6-phosphate dehydrogenase activity" evidence=ISS;IDA] [GO:0006006 "glucose metabolic process" evidence=ISS;IDA] [GO:0009051 "pentose-phosphate shunt, oxidative branch" evidence=IDA] [GO:0009507 "chloroplast" evidence=ISS] [GO:0009570 "chloroplast stroma" evidence=IDA] InterPro:IPR001282 InterPro:IPR016040 InterPro:IPR019796 InterPro:IPR022674 InterPro:IPR022675 Pfam:PF00479 Pfam:PF02781 PRINTS:PR00079 PROSITE:PS00069 GO:GO:0009570 EMBL:CP002688 GenomeReviews:BA000015_GR Gene3D:G3DSA:3.40.50.720 GO:GO:0050661 GO:GO:0009051 EMBL:AL391711 eggNOG:COG0364 HOGENOM:HBG322449 KO:K00036 GO:GO:0004345 PANTHER:PTHR23429 TIGRFAMs:TIGR00871 EMBL:AY065042 EMBL:X84229 IPI:IPI00531306 PIR:S71245 RefSeq:NP_196815.2 UniGene:At.61 ProteinModelPortal:Q9FY99 SMR:Q9FY99 IntAct:Q9FY99 STRING:Q9FY99 PRIDE:Q9FY99 EnsemblPlants:AT5G13110.1 GeneID:831150 KEGG:ath:AT5G13110 TAIR:At5g13110 InParanoid:Q9FY99 OMA:FANQMFE PhylomeDB:Q9FY99 ProtClustDB:PLN02333 ArrayExpress:Q9FY99 Genevestigator:Q9FY99 GermOnline:AT5G13110 Uniprot:Q9FY99
Root	Isotig01384	44	11	1.871	1.68E-05	TAIR|locus:2029416 - symbol:AT1G77840 species:3702 "Arabidopsis thaliana" [GO:0003743 "translation initiation factor activity" evidence=IEA;ISS] [GO:0006413 "translational initiation" evidence=IEA] [GO:0006446 "regulation of translational initiation" evidence=ISS] [GO:0005634 "nucleus" evidence=IDA] [GO:0005737 "cytoplasm" evidence=IDA] InterPro:IPR002735 InterPro:IPR003307 InterPro:IPR016021 InterPro:IPR016024 InterPro:IPR016189 InterPro:IPR016190 Pfam:PF01873 Pfam:PF02020 PROSITE:PS51363 SMART:SM00515 SMART:SM00653 GO:GO:0005525 EMBL:CP002684 GenomeReviews:CT485782_GR GO:GO:0005634 GO:GO:0005737 SUPFAM:SSF48371 GO:GO:0003743 GO:GO:0016070 EMBL:AC009243 EMBL:AC012193 Gene3D:G3DSA:1.25.40.180 eggNOG:COG1601 Gene3D:G3DSA:3.30.30.50 SUPFAM:SSF100966

Tissue	Identification	# of reads (stress)	# of reads (control)	Fold Change	p-value	Description
						SUPFAM:SSF75689 HOGENOM:HBG559646 KO:K03262 ProtClustDB:CLSN2715102 EMBL:AY062694 EMBL:AY065097 EMBL:AY114664 EMBL:BT002163 IPI:IPI00536490 RefSeq:NP_177907.1 UniGene:At.27261 ProteinModelPortal:Q9S825 SMR:Q9S825 STRING:Q9S825 PRIDE:Q9S825 DNASU:844119 EnsemblPlants:AT1G77840.1 GeneID:844119 KEGG:ath:AT1G77840 TAIR:At1g77840 InParanoid:Q9S825 OMA:WKNVKPF PhylomeDB:Q9S825 ArrayExpress:Q9S825 Genevestigator:Q9S825 GermOnline:AT1G77840 Uniprot:Q9S825
Root	Isotig01385	43	11	1.838	2.63E-05	TAIR|locus:2029416 - symbol:AT1G77840 species:3702 "Arabidopsis thaliana" [GO:0003743 "translation initiation factor activity" evidence=IEA;ISS] [GO:0006413 "translational initiation" evidence=IEA] [GO:0006446 "regulation of translational initiation" evidence=ISS] [GO:0005634 "nucleus" evidence=IDA] [GO:0005737 "cytoplasm" evidence=IDA] InterPro:IPR002735 InterPro:IPR003307 InterPro:IPR016021 InterPro:IPR016024 InterPro:IPR016189 InterPro:IPR016190 Pfam:PF01873 Pfam:PF02020 PROSITE:PS51363 SMART:SM00515 SMART:SM00653 GO:GO:0005525 EMBL:CP002684 GenomeReviews:CT485782_GR GO:GO:0005634 GO:GO:0005737 SUPFAM:SSF48371 GO:GO:0003743 GO:GO:0016070 EMBL:AC009243 EMBL:AC012193 Gene3D:G3DSA:1.25.40.180 eggNOG:COG1601 Gene3D:G3DSA:3.30.30.50 SUPFAM:SSF100966 SUPFAM:SSF75689 HOGENOM:HBG559646 KO:K03262 ProtClustDB:CLSN2715102 EMBL:AY062694 EMBL:AY065097 EMBL:AY114664 EMBL:BT002163 IPI:IPI00536490 RefSeq:NP_177907.1 UniGene:At.27261 ProteinModelPortal:Q9S825 SMR:Q9S825 STRING:Q9S825 PRIDE:Q9S825 DNASU:844119 EnsemblPlants:AT1G77840.1 GeneID:844119 KEGG:ath:AT1G77840 TAIR:At1g77840 InParanoid:Q9S825 OMA:WKNVKPF PhylomeDB:Q9S825 ArrayExpress:Q9S825 Genevestigator:Q9S825 GermOnline:AT1G77840 Uniprot:Q9S825
Root	Isotig01386	33	10	1.593	0.000866735	TAIR|locus:2196376 - symbol:AT1G36730 species:3702 "Arabidopsis thaliana" [GO:0003743 "translation initiation factor activity" evidence=IEA;ISS] [GO:0006413 "translational initiation" evidence=IEA] [GO:0006446 "regulation of translational initiation" evidence=ISS] [GO:0005829 "cytosol" evidence=IDA] InterPro:IPR002735 InterPro:IPR003307 InterPro:IPR016021 InterPro:IPR016024 InterPro:IPR016189 InterPro:IPR016190 Pfam:PF01873 Pfam:PF02020 PROSITE:PS51363 SMART:SM00515 SMART:SM00653 GO:GO:0005525 EMBL:CP002684 GenomeReviews:CT485782_GR GO:GO:0005829 SUPFAM:SSF48371 GO:GO:0003743 GO:GO:0016070 Gene3D:G3DSA:1.25.40.180 UniGene:At.3809 eggNOG:COG1601 Gene3D:G3DSA:3.30.30.50 SUPFAM:SSF100966 SUPFAM:SSF75689 EMBL:AC025782 EMBL:AY056208 EMBL:AY064061 EMBL:AY096371 IPI:IPI00524003 PIR:C86487 RefSeq:NP_174877.1 UniGene:At.22980 UniGene:At.67142 ProteinModelPortal:Q9C8F1 SMR:Q9C8F1 PRIDE:Q9C8F1 EnsemblPlants:AT1G36730.1 GeneID:840582 KEGG:ath:AT1G36730 TAIR:At1g36730 HOGENOM:HBG559646 InParanoid:Q9C8F1 KO:K03262 OMA:HENGNSK PhylomeDB:Q9C8F1 ProtClustDB:CLSN2715102 ArrayExpress:Q9C8F1 Genevestigator:Q9C8F1 GermOnline:AT1G36730 Uniprot:Q9C8F1
Root	Isotig01404	0	12	-4.714	0.000297064	TAIR|locus:2066286 - symbol:PLA2A "AT2G26560" species:3702 "Arabidopsis thaliana" [GO:0045735 "nutrient reservoir activity" evidence=ISS] [GO:0005737 "cytoplasm" evidence=IDA] [GO:0006629 "lipid metabolic process" evidence=IDA] [GO:0009626 "plant-type hypersensitive response" evidence=IEP;IMP] [GO:0016020 "membrane" evidence=IDA] [GO:0016298 "lipase activity" evidence=IDA] [GO:0046686 "response to cadmium ion" evidence=IEP] [GO:0008219 "cell death" evidence=IGI] [GO:0031408 "oxylipin biosynthetic process" evidence=IMP] [GO:0051607 "defense response to virus" evidence=IMP] [GO:0071456 "cellular response to hypoxia" evidence=IEP] InterPro:IPR002641 InterPro:IPR016035 Pfam:PF01734 GO:GO:0046686 GO:GO:0005737 EMBL:CP002685 GenomeReviews:CT485783_GR GO:GO:0016020 GO:GO:0071456 GO:GO:0051607 GO:GO:0016298 GO:GO:0009626 GO:GO:0031408 EMBL:AC002505 SUPFAM:SSF52151 eggNOG:COG3621 HSSP:Q8LPW4 HOGENOM:HBG581721 EMBL:AY062644 EMBL:AY093305 IPI:IPI00536283 PIR:T00989 RefSeq:NP_180224.1 UniGene:At.12388 ProteinModelPortal:O48723 SMR:O48723 IntAct:O48723 STRING:O48723 PRIDE:O48723 DNASU:817197 EnsemblPlants:AT2G26560.1 GeneID:817197 KEGG:ath:AT2G26560 TAIR:At2g26560 InParanoid:O48723 OMA:NENAYET PhylomeDB:O48723 ProtClustDB:CLSN2913024 ArrayExpress:O48723 Genevestigator:O48723 Uniprot:O48723
Root	Isotig01405	0	12	-4.714	0.000297064	TAIR|locus:2114995 - symbol:PLP1 "AT4G37070" species:3702 "Arabidopsis thaliana" [GO:0005575 "cellular_component" evidence=ND] [GO:0045735 "nutrient reservoir activity" evidence=ISS] [GO:0004620 "phospholipase activity" evidence=IDA] [GO:0010311 "lateral root formation" evidence=IMP] InterPro:IPR002641 InterPro:IPR016035 Pfam:PF01734 EMBL:CP002687 GO:GO:0006629 GO:GO:0010311 EMBL:Z99707 EMBL:AL161590 SUPFAM:SSF52151 GO:GO:0004620 EMBL:BT029750 IPI:IPI00528835 PIR:H85437

Tissue	Identification	# of reads (stress)	# of reads (control)	Fold Change	p-value	Description
						RefSeq:NP_849511.1 UniGene:At.23952 HSSP:Q8LPW4 ProteinModelPortal:O23179 SMR:O23179 PRIDE:O23179 EnsemblPlants:AT4G37070.2 GeneID:829861 KEGG:ath:AT4G37070 TAIR:At4g37070 InParanoid:O23179 OMA:ITMESSR PhylomeDB:O23179 ProtClustDB:CLSN2685599 ArrayExpress:O23179 Genevestigator:O23179 Uniprot:O23179
Root	Isotig01406	0	11	-4.588	0.000569522	TAIR|locus:2066286 - symbol:PLA2A "AT2G26560" species:3702 "Arabidopsis thaliana" [GO:0045735 "nutrient reservoir activity" evidence=ISS] [GO:0005737 "cytoplasm" evidence=IDA] [GO:0006629 "lipid metabolic process" evidence=IDA] [GO:0009626 "plant-type hypersensitive response" evidence=IEP;IMP] [GO:0016020 "membrane" evidence=IDA] [GO:0016298 "lipase activity" evidence=IDA] [GO:0046686 "response to cadmium ion" evidence=IEP] [GO:0008219 "cell death" evidence=IGI] [GO:0031408 "oxylipin biosynthetic process" evidence=IMP] [GO:0051607 "defense response to virus" evidence=IMP] [GO:0071456 "cellular response to hypoxia" evidence=IEP] InterPro:IPR002641 InterPro:IPR016035 Pfam:PF01734 GO:GO:0046686 GO:GO:0005737 EMBL:CP002685 GenomeReviews:CT485783_GR GO:GO:0016020 GO:GO:0071456 GO:GO:0051607 GO:GO:0016298 GO:GO:0009626 GO:GO:0031408 EMBL:AC002505 SUPFAM:SSF52151 eggNOG:COG3621 HSSP:Q8LPW4 HOGENOM:HBG581721 EMBL:AY062644 EMBL:AY093305 IPI:IPI00536283 PIR:T00989 RefSeq:NP_180224.1 UniGene:At.12388 ProteinModelPortal:O48723 SMR:O48723 IntAct:O48723 STRING:O48723 PRIDE:O48723 DNASU:817197 EnsemblPlants:AT2G26560.1 GeneID:817197 KEGG:ath:AT2G26560 TAIR:At2g26560 InParanoid:O48723 OMA:NENAYET PhylomeDB:O48723 ProtClustDB:CLSN2913024 ArrayExpress:O48723 Genevestigator:O48723 Uniprot:O48723
Root	Isotig01407	0	12	-4.714	0.000297064	TAIR|locus:2114995 - symbol:PLP1 "AT4G37070" species:3702 "Arabidopsis thaliana" [GO:0005575 "cellular_component" evidence=ND] [GO:0045735 "nutrient reservoir activity" evidence=ISS] [GO:0004620 "phospholipase activity" evidence=IDA] [GO:0010311 "lateral root formation" evidence=IMP] InterPro:IPR002641 InterPro:IPR016035 Pfam:PF01734 EMBL:CP002687 GO:GO:0006629 GO:GO:0010311 EMBL:Z99707 EMBL:AL161590 SUPFAM:SSF52151 GO:GO:0004620 EMBL:BT029750 IPI:IPI00528835 PIR:H85437 RefSeq:NP_849511.1 UniGene:At.23952 HSSP:Q8LPW4 ProteinModelPortal:O23179 SMR:O23179 PRIDE:O23179 EnsemblPlants:AT4G37070.2 GeneID:829861 KEGG:ath:AT4G37070 TAIR:At4g37070 InParanoid:O23179 OMA:ITMESSR PhylomeDB:O23179 ProtClustDB:CLSN2685599 ArrayExpress:O23179 Genevestigator:O23179 Uniprot:O23179
Root	Isotig01408	0	11	-4.588	0.000569522	TAIR|locus:2066286 - symbol:PLA2A "AT2G26560" species:3702 "Arabidopsis thaliana" [GO:0045735 "nutrient reservoir activity" evidence=ISS] [GO:0005737 "cytoplasm" evidence=IDA] [GO:0006629 "lipid metabolic process" evidence=IDA] [GO:0009626 "plant-type hypersensitive response" evidence=IEP;IMP] [GO:0016020 "membrane" evidence=IDA] [GO:0016298 "lipase activity" evidence=IDA] [GO:0046686 "response to cadmium ion" evidence=IEP] [GO:0008219 "cell death" evidence=IGI] [GO:0031408 "oxylipin biosynthetic process" evidence=IMP] [GO:0051607 "defense response to virus" evidence=IMP] [GO:0071456 "cellular response to hypoxia" evidence=IEP] InterPro:IPR002641 InterPro:IPR016035 Pfam:PF01734 GO:GO:0046686 GO:GO:0005737 EMBL:CP002685 GenomeReviews:CT485783_GR GO:GO:0016020 GO:GO:0071456 GO:GO:0051607 GO:GO:0016298 GO:GO:0009626 GO:GO:0031408 EMBL:AC002505 SUPFAM:SSF52151 eggNOG:COG3621 HSSP:Q8LPW4 HOGENOM:HBG581721 EMBL:AY062644 EMBL:AY093305 IPI:IPI00536283 PIR:T00989 RefSeq:NP_180224.1 UniGene:At.12388 ProteinModelPortal:O48723 SMR:O48723 IntAct:O48723 STRING:O48723 PRIDE:O48723 DNASU:817197 EnsemblPlants:AT2G26560.1 GeneID:817197 KEGG:ath:AT2G26560 TAIR:At2g26560 InParanoid:O48723 OMA:NENAYET PhylomeDB:O48723 ProtClustDB:CLSN2913024 ArrayExpress:O48723 Genevestigator:O48723 Uniprot:O48723
Root	Isotig01409	0	12	-4.714	0.000297064	TAIR|locus:2114995 - symbol:PLP1 "AT4G37070" species:3702 "Arabidopsis thaliana" [GO:0005575 "cellular_component" evidence=ND] [GO:0045735 "nutrient reservoir activity" evidence=ISS] [GO:0004620 "phospholipase activity" evidence=IDA] [GO:0010311 "lateral root formation" evidence=IMP] InterPro:IPR002641 InterPro:IPR016035 Pfam:PF01734 EMBL:CP002687 GO:GO:0006629 GO:GO:0010311 EMBL:Z99707 EMBL:AL161590 SUPFAM:SSF52151 GO:GO:0004620 EMBL:BT029750 IPI:IPI00528835 PIR:H85437 RefSeq:NP_849511.1 UniGene:At.23952 HSSP:Q8LPW4 ProteinModelPortal:O23179 SMR:O23179 PRIDE:O23179 EnsemblPlants:AT4G37070.2 GeneID:829861 KEGG:ath:AT4G37070 TAIR:At4g37070 InParanoid:O23179 OMA:ITMESSR PhylomeDB:O23179 ProtClustDB:CLSN2685599 ArrayExpress:O23179 Genevestigator:O23179 Uniprot:O23179
Root	Isotig01410	0	11	-4.588	0.000569522	TAIR|locus:2066286 - symbol:PLA2A "AT2G26560" species:3702 "Arabidopsis thaliana" [GO:0045735 "nutrient reservoir activity" evidence=ISS] [GO:0005737 "cytoplasm" evidence=IDA] [GO:0006629 "lipid metabolic process"

Tissue	Identification	# of reads (stress)	# of reads (control)	Fold Change	p-value	Description
						evidence=IDA] [GO:0009626 "plant-type hypersensitive response" evidence=IEP;IMP] [GO:0016020 "membrane" evidence=IDA] [GO:0016298 "lipase activity" evidence=IDA] [GO:0046686 "response to cadmium ion" evidence=IEP] [GO:0008219 "cell death" evidence=IGI] [GO:0031408 "oxylipin biosynthetic process" evidence=IMP] [GO:0051607 "defense response to virus" evidence=IMP] [GO:0071456 "cellular response to hypoxia" evidence=IEP] InterPro:IPR002641 InterPro:IPR016035 Pfam:PF01734 GO:GO:0046686 GO:GO:0005737 EMBL:CP002685 GenomeReviews:CT485783_GR GO:GO:0016020 GO:GO:0071456 GO:GO:0051607 GO:GO:0016298 GO:GO:0009626 GO:GO:0031408 EMBL:AC002505 SUPFAM:SSF52151 eggNOG:COG3621 HSSP:Q8LPW4 HOGENOM:HBG581721 EMBL:AY062644 EMBL:AY093305 IPI:IPI00536283 PIR:T00989 RefSeq:NP_180224.1 UniGene:At.12388 ProteinModelPortal:O48723 SMR:O48723 IntAct:O48723 STRING:O48723 PRIDE:O48723 DNASU:817197 EnsemblPlants:AT2G26560.1 GeneID:817197 KEGG:ath:AT2G26560 TAIR:At2g26560 InParanoid:O48723 OMA:NENAYET PhylomeDB:O48723 ProtClustDB:CLSN2913024 ArrayExpress:O48723 Genevestigator:O48723 Uniprot:O48723
Root	Isotig01411	0	11	-4.588	0.000569522	TAIR|locus:2066286 - symbol:PLA2A "AT2G26560" species:3702 "Arabidopsis thaliana" [GO:0045735 "nutrient reservoir activity" evidence=ISS] [GO:0005737 "cytoplasm" evidence=IDA] [GO:0006629 "lipid metabolic process" evidence=IDA] [GO:0009626 "plant-type hypersensitive response" evidence=IEP;IMP] [GO:0016020 "membrane" evidence=IDA] [GO:0016298 "lipase activity" evidence=IDA] [GO:0046686 "response to cadmium ion" evidence=IEP] [GO:0008219 "cell death" evidence=IGI] [GO:0031408 "oxylipin biosynthetic process" evidence=IMP] [GO:0051607 "defense response to virus" evidence=IMP] [GO:0071456 "cellular response to hypoxia" evidence=IEP] InterPro:IPR002641 InterPro:IPR016035 Pfam:PF01734 GO:GO:0046686 GO:GO:0005737 EMBL:CP002685 GenomeReviews:CT485783_GR GO:GO:0016020 GO:GO:0071456 GO:GO:0051607 GO:GO:0016298 GO:GO:0009626 GO:GO:0031408 EMBL:AC002505 SUPFAM:SSF52151 eggNOG:COG3621 HSSP:Q8LPW4 HOGENOM:HBG581721 EMBL:AY062644 EMBL:AY093305 IPI:IPI00536283 PIR:T00989 RefSeq:NP_180224.1 UniGene:At.12388 ProteinModelPortal:O48723 SMR:O48723 IntAct:O48723 STRING:O48723 PRIDE:O48723 DNASU:817197 EnsemblPlants:AT2G26560.1 GeneID:817197 KEGG:ath:AT2G26560 TAIR:At2g26560 InParanoid:O48723 OMA:NENAYET PhylomeDB:O48723 ProtClustDB:CLSN2913024 ArrayExpress:O48723 Genevestigator:O48723 Uniprot:O48723
Root	Isotig01427	56	19	1.430	6.16E-05	TAIR|locus:2160917 - symbol:RBOHD "AT5G47910" species:3702 "Arabidopsis thaliana" [GO:0006952 "defense response" evidence=IMP] [GO:0072593 "reactive oxygen species metabolic process" evidence=IMP;TAS] [GO:0016174 "NAD(P)H oxidase activity" evidence=IMP;TAS] [GO:0009408 "response to heat" evidence=IMP] [GO:0043069 "negative regulation of programmed cell death" evidence=IGI] [GO:0005886 "plasma membrane" evidence=IDA] [GO:0050832 "defense response to fungus" evidence=IMP] [GO:0009611 "response to wounding" evidence=IEP] [GO:0005794 "Golgi apparatus" evidence=IDA] InterPro:IPR000778 InterPro:IPR011992 InterPro:IPR013112 InterPro:IPR013121 InterPro:IPR013130 InterPro:IPR013623 InterPro:IPR017927 InterPro:IPR017938 Pfam:PF01794 Pfam:PF08022 Pfam:PF08030 Pfam:PF08414 PRINTS:PR00466 PROSITE:PS51384 Prosite:PS00018 GO:GO:0016021 GO:GO:0005886 EMBL:CP002688 GenomeReviews:BA000015_GR GO:GO:0005509 GO:GO:0009611 GO:GO:0050832 GO:GO:0005506 GO:GO:0009055 GO:GO:0050660 InterPro:IPR018247 InterPro:IPR018249 Gene3D:G3DSA:1.10.238.10 PROSITE:PS50222 GO:GO:0009408 GO:GO:0043069 EMBL:AB016886 GO:GO:0072593 GO:GO:0004601 SUPFAM:SSF63380 HOGENOM:HBG591219 GO:GO:0016174 eggNOG:COG4097 KO:K13447 EMBL:AF055357 EMBL:AF424625 EMBL:BT002651 IPI:IPI00522047 PIR:T51804 RefSeq:NP_199602.1 UniGene:At.23270 ProteinModelPortal:Q9FIJ0 SMR:Q9FIJ0 STRING:Q9FIJ0 PeroxiBase:3286 PRIDE:Q9FIJ0 EnsemblPlants:AT5G47910.1 GeneID:834842 KEGG:ath:AT5G47910 TAIR:At5g47910 InParanoid:Q9FIJ0 OMA:FTYKFIQ PhylomeDB:Q9FIJ0 ProtClustDB:CLSN2916327 Genevestigator:Q9FIJ0 Uniprot:Q9FIJ0
Root	Isotig01428	65	16	1.893	1.34E-07	TAIR|locus:2160917 - symbol:RBOHD "AT5G47910" species:3702 "Arabidopsis thaliana" [GO:0006952 "defense response" evidence=IMP] [GO:0072593 "reactive oxygen species metabolic process" evidence=IMP;TAS] [GO:0016174 "NAD(P)H oxidase activity" evidence=IMP;TAS] [GO:0009408 "response to heat" evidence=IMP] [GO:0043069 "negative regulation of programmed cell death" evidence=IGI] [GO:0005886 "plasma membrane" evidence=IDA] [GO:0050832 "defense response to fungus" evidence=IMP] [GO:0009611 "response to wounding" evidence=IEP] [GO:0005794 "Golgi apparatus" evidence=IDA] InterPro:IPR000778 InterPro:IPR011992 InterPro:IPR013112 InterPro:IPR013121 InterPro:IPR013130 InterPro:IPR013623 InterPro:IPR017927 InterPro:IPR017938 Pfam:PF01794 Pfam:PF08022 Pfam:PF08030 Pfam:PF08414 PRINTS:PR00466 PROSITE:PS51384 Prosite:PS00018 GO:GO:0016021 GO:GO:0005886 EMBL:CP002688

Tissue	Identification	# of reads (stress)	# of reads (control)	Fold Change	p-value	Description
						GenomeReviews:BA000015_GR GO:GO:0005509 GO:GO:0009611 GO:GO:0050832 GO:GO:0005506 GO:GO:0009055 GO:GO:0050660 InterPro:IPR018247 InterPro:IPR018249 Gene3D:G3DSA:1.10.238.10 PROSITE:PS50222 GO:GO:0009408 GO:GO:0043069 EMBL:AB016886 GO:GO:0072593 GO:GO:0004601 SUPFAM:SSF63380 HOGENOM:HBG591219 GO:GO:0016174 eggNOG:COG4097 KO:K13447 EMBL:AF055357 EMBL:AF424625 EMBL:BT002651 IPI:IPI00522047 PIR:T51804 RefSeq:NP_199602.1 UniGene:At.23270 ProteinModelPortal:Q9FIJ0 SMR:Q9FIJ0 STRING:Q9FIJ0 PeroxiBase:3286 PRIDE:Q9FIJ0 EnsemblPlants:AT5G47910.1 GeneID:834842 KEGG:ath:AT5G47910 TAIR:At5g47910 InParanoid:Q9FIJ0 OMA:FTYKFIQ PhylomeDB:Q9FIJ0 ProtClustDB:CLSN2916327 Genevestigator:Q9FIJ0 Uniprot:Q9FIJ0
Root	Isotig01431	55	18	1.482	4.54E-05	TAIR|locus:2036104 - symbol:RBOHB "respiratory burst oxidase homolog B" species:3702 "Arabidopsis thaliana" [GO:0016020 "membrane" evidence=IEA] [GO:0016021 "integral to membrane" evidence=IEA] [GO:0009408 "response to heat" evidence=IMP] [GO:0009845 "seed germination" evidence=IMP] [GO:0016174 "NAD(P)H oxidase activity" evidence=IMP] [GO:0006952 "defense response" evidence=TAS] InterPro:IPR000778 InterPro:IPR002048 InterPro:IPR011992 InterPro:IPR013112 InterPro:IPR013121 InterPro:IPR013130 InterPro:IPR013623 InterPro:IPR017927 InterPro:IPR017938 Pfam:PF01794 Pfam:PF08022 Pfam:PF08030 Pfam:PF08414 PRINTS:PR00466 PROSITE:PS51384 SMART:SM00054 Prosite:PS00018 GO:GO:0016021 EMBL:CP002684 GenomeReviews:CT485782_GR GO:GO:0005509 GO:GO:0005506 GO:GO:0009055 GO:GO:0050660 InterPro:IPR018249 Gene3D:G3DSA:1.10.238.10 PROSITE:PS50222 GO:GO:0009408 GO:GO:0009845 GO:GO:0004601 SUPFAM:SSF63380 EMBL:AC000106 eggNOG:NOG287712 HOGENOM:HBG591219 GO:GO:0016174 UniGene:At.10379 UniGene:At.42241 EMBL:AF055354 EMBL:BT005716 IPI:IPI00517116 IPI:IPI00520396 PIR:A86223 PIR:B86223 RefSeq:NP_172383.3 RefSeq:NP_973799.1 ProteinModelPortal:Q9SBI0 SMR:Q9SBI0 STRING:Q9SBI0 PeroxiBase:3283 PRIDE:Q9SBI0 EnsemblPlants:AT1G09090.2 GeneID:837430 KEGG:ath:AT1G09090 TAIR:At1g09090 InParanoid:Q9SBI0 OMA:LWISICI PhylomeDB:Q9SBI0 ArrayExpress:Q84TJ9 Genevestigator:Q9SBI0 Uniprot:Q9SBI0
Root	Isotig01432	53	16	1.599	2.32E-05	TAIR|locus:2036104 - symbol:RBOHB "respiratory burst oxidase homolog B" species:3702 "Arabidopsis thaliana" [GO:0016020 "membrane" evidence=IEA] [GO:0016021 "integral to membrane" evidence=IEA] [GO:0009408 "response to heat" evidence=IMP] [GO:0009845 "seed germination" evidence=IMP] [GO:0016174 "NAD(P)H oxidase activity" evidence=IMP] [GO:0006952 "defense response" evidence=TAS] InterPro:IPR000778 InterPro:IPR002048 InterPro:IPR011992 InterPro:IPR013112 InterPro:IPR013121 InterPro:IPR013130 InterPro:IPR013623 InterPro:IPR017927 InterPro:IPR017938 Pfam:PF01794 Pfam:PF08022 Pfam:PF08030 Pfam:PF08414 PRINTS:PR00466 PROSITE:PS51384 SMART:SM00054 Prosite:PS00018 GO:GO:0016021 EMBL:CP002684 GenomeReviews:CT485782_GR GO:GO:0005509 GO:GO:0005506 GO:GO:0009055 GO:GO:0050660 InterPro:IPR018249 Gene3D:G3DSA:1.10.238.10 PROSITE:PS50222 GO:GO:0009408 GO:GO:0009845 GO:GO:0004601 SUPFAM:SSF63380 EMBL:AC000106 eggNOG:NOG287712 HOGENOM:HBG591219 GO:GO:0016174 UniGene:At.10379 UniGene:At.42241 EMBL:AF055354 EMBL:BT005716 IPI:IPI00517116 IPI:IPI00520396 PIR:A86223 PIR:B86223 RefSeq:NP_172383.3 RefSeq:NP_973799.1 ProteinModelPortal:Q9SBI0 SMR:Q9SBI0 STRING:Q9SBI0 PeroxiBase:3283 PRIDE:Q9SBI0 EnsemblPlants:AT1G09090.2 GeneID:837430 KEGG:ath:AT1G09090 TAIR:At1g09090 InParanoid:Q9SBI0 OMA:LWISICI PhylomeDB:Q9SBI0 ArrayExpress:Q84TJ9 Genevestigator:Q9SBI0 Uniprot:Q9SBI0
Root	Isotig01449	9	28	-1.766	0.000557996	TAIR|locus:2038836 - symbol:PDE331 "AT2G01190" species:3702 "Arabidopsis thaliana" [GO:0005634 "nucleus" evidence=IDA] [GO:0005829 "cytosol" evidence=IDA] InterPro:IPR000270 Pfam:PF00564 SMART:SM00666 GO:GO:0005829 GO:GO:0005634 EMBL:CP002685 EMBL:AC006200 ProtClustDB:CLSN2687650 EMBL:AY070060 EMBL:AY117302 IPI:IPI00548472 PIR:F84421 RefSeq:NP_565256.1 UniGene:At.19855 UniGene:At.72006 UniGene:At.74187 UniGene:At.74214 ProteinModelPortal:Q9ZU48 IntAct:Q9ZU48 PRIDE:Q9ZU48 EnsemblPlants:AT2G01190.1 GeneID:814647 KEGG:ath:AT2G01190 TAIR:At2g01190 InParanoid:Q9ZU48 OMA:YDRTISA PhylomeDB:Q9ZU48 ArrayExpress:Q9ZU48 Genevestigator:Q9ZU48 Uniprot:Q9ZU48
Root	Isotig01455	18	67	-2.025	4.84E-09	TAIR|locus:2195623 - symbol:AT1G60420 species:3702 "Arabidopsis thaliana" [GO:0016209 "antioxidant activity" evidence=IEA] [GO:0016491 "oxidoreductase activity" evidence=IEA] [GO:0046686 "response to cadmium ion" evidence=IEP] [GO:0009860 "pollen tube growth" evidence=IMP] [GO:0010183 "pollen tube guidance" evidence=IMP] [GO:0080092 "regulation of pollen tube growth" evidence=IMP] [GO:0005829 "cytosol" evidence=IDA] InterPro:IPR011424 InterPro:IPR017937 Pfam:PF07649 PROSITE:PS00194 EMBL:CP002684 GenomeReviews:CT485782_GR GO:GO:0005829 GO:GO:0046686 GO:GO:0009860 InterPro:IPR012336

Tissue	Identification	# of reads (stress)	# of reads (control)	Fold Change	p-value	Description
						Gene3D:G3DSA:3.40.30.10 SUPFAM:SSF52833 GO:GO:0045454 PROSITE:PS51352 GO:GO:0080092 GO:GO:0010183 eggNOG:COG0526 GO:GO:0047134 EMBL:AC004473 EMBL:AY065433 EMBL:AY117231 EMBL:AY087396 IPI:IPI00537432 PIR:T02292 RefSeq:NP_564756.1 UniGene:At.10685 UniGene:At.64112 PDB:1V5N PDBsum:1V5N ProteinModelPortal:O80763 SMR:O80763 IntAct:O80763 PRIDE:O80763 EnsemblPlants:AT1G60420.1 GeneID:842337 KEGG:ath:AT1G60420 TAIR:At1g60420 HOGENOM:HBG594354 InParanoid:O80763 OMA:QMPWLAL PhylomeDB:O80763 ProtClustDB:CLSN2917334 ArrayExpress:O80763 Genevestigator:O80763 Uniprot:O80763
Root	Isotig01456	22	75	-1.898	2.97E-09	TAIR|locus:2195623 - symbol:AT1G60420 species:3702 "Arabidopsis thaliana" [GO:0016209 "antioxidant activity" evidence=IEA] [GO:0016491 "oxidoreductase activity" evidence=IEA] [GO:0046686 "response to cadmium ion" evidence=IEP] [GO:0009860 "pollen tube growth" evidence=IMP] [GO:0010183 "pollen tube guidance" evidence=IMP] [GO:0080092 "regulation of pollen tube growth" evidence=IMP] [GO:0005829 "cytosol" evidence=IDA] InterPro:IPR011424 InterPro:IPR017937 Pfam:PF07649 PROSITE:PS00194 EMBL:CP002684 GenomeReviews:CT485782_GR GO:GO:0005829 GO:GO:0046686 GO:GO:0009860 InterPro:IPR012336 Gene3D:G3DSA:3.40.30.10 SUPFAM:SSF52833 GO:GO:0045454 PROSITE:PS51352 GO:GO:0080092 GO:GO:0010183 eggNOG:COG0526 GO:GO:0047134 EMBL:AC004473 EMBL:AY065433 EMBL:AY117231 EMBL:AY087396 IPI:IPI00537432 PIR:T02292 RefSeq:NP_564756.1 UniGene:At.10685 UniGene:At.64112 PDB:1V5N PDBsum:1V5N ProteinModelPortal:O80763 SMR:O80763 IntAct:O80763 PRIDE:O80763 EnsemblPlants:AT1G60420.1 GeneID:842337 KEGG:ath:AT1G60420 TAIR:At1g60420 HOGENOM:HBG594354 InParanoid:O80763 OMA:QMPWLAL PhylomeDB:O80763 ProtClustDB:CLSN2917334 ArrayExpress:O80763 Genevestigator:O80763 Uniprot:O80763
Root	Isotig01457	19	71	-2.031	1.58E-09	TAIR|locus:2195623 - symbol:AT1G60420 species:3702 "Arabidopsis thaliana" [GO:0016209 "antioxidant activity" evidence=IEA] [GO:0016491 "oxidoreductase activity" evidence=IEA] [GO:0046686 "response to cadmium ion" evidence=IEP] [GO:0009860 "pollen tube growth" evidence=IMP] [GO:0010183 "pollen tube guidance" evidence=IMP] [GO:0080092 "regulation of pollen tube growth" evidence=IMP] [GO:0005829 "cytosol" evidence=IDA] InterPro:IPR011424 InterPro:IPR017937 Pfam:PF07649 PROSITE:PS00194 EMBL:CP002684 GenomeReviews:CT485782_GR GO:GO:0005829 GO:GO:0046686 GO:GO:0009860 InterPro:IPR012336 Gene3D:G3DSA:3.40.30.10 SUPFAM:SSF52833 GO:GO:0045454 PROSITE:PS51352 GO:GO:0080092 GO:GO:0010183 eggNOG:COG0526 GO:GO:0047134 EMBL:AC004473 EMBL:AY065433 EMBL:AY117231 EMBL:AY087396 IPI:IPI00537432 PIR:T02292 RefSeq:NP_564756.1 UniGene:At.10685 UniGene:At.64112 PDB:1V5N PDBsum:1V5N ProteinModelPortal:O80763 SMR:O80763 IntAct:O80763 PRIDE:O80763 EnsemblPlants:AT1G60420.1 GeneID:842337 KEGG:ath:AT1G60420 TAIR:At1g60420 HOGENOM:HBG594354 InParanoid:O80763 OMA:QMPWLAL PhylomeDB:O80763 ProtClustDB:CLSN2917334 ArrayExpress:O80763 Genevestigator:O80763 Uniprot:O80763
Root	Isotig01458	15	66	-2.266	4.53E-10	TAIR|locus:2195623 - symbol:AT1G60420 species:3702 "Arabidopsis thaliana" [GO:0016209 "antioxidant activity" evidence=IEA] [GO:0016491 "oxidoreductase activity" evidence=IEA] [GO:0046686 "response to cadmium ion" evidence=IEP] [GO:0009860 "pollen tube growth" evidence=IMP] [GO:0010183 "pollen tube guidance" evidence=IMP] [GO:0080092 "regulation of pollen tube growth" evidence=IMP] [GO:0005829 "cytosol" evidence=IDA] InterPro:IPR011424 InterPro:IPR017937 Pfam:PF07649 PROSITE:PS00194 EMBL:CP002684 GenomeReviews:CT485782_GR GO:GO:0005829 GO:GO:0046686 GO:GO:0009860 InterPro:IPR012336 Gene3D:G3DSA:3.40.30.10 SUPFAM:SSF52833 GO:GO:0045454 PROSITE:PS51352 GO:GO:0080092 GO:GO:0010183 eggNOG:COG0526 GO:GO:0047134 EMBL:AC004473 EMBL:AY065433 EMBL:AY117231 EMBL:AY087396 IPI:IPI00537432 PIR:T02292 RefSeq:NP_564756.1 UniGene:At.10685 UniGene:At.64112 PDB:1V5N PDBsum:1V5N ProteinModelPortal:O80763 SMR:O80763 IntAct:O80763 PRIDE:O80763 EnsemblPlants:AT1G60420.1 GeneID:842337 KEGG:ath:AT1G60420 TAIR:At1g60420 HOGENOM:HBG594354 InParanoid:O80763 OMA:QMPWLAL PhylomeDB:O80763 ProtClustDB:CLSN2917334 ArrayExpress:O80763 Genevestigator:O80763 Uniprot:O80763
Root	Isotig01459	19	74	-2.090	3.44E-10	TAIR|locus:2195623 - symbol:AT1G60420 species:3702 "Arabidopsis thaliana" [GO:0016209 "antioxidant activity" evidence=IEA] [GO:0016491 "oxidoreductase activity" evidence=IEA] [GO:0046686 "response to cadmium ion" evidence=IEP] [GO:0009860 "pollen tube growth" evidence=IMP] [GO:0010183 "pollen tube guidance" evidence=IMP] [GO:0080092 "regulation of pollen tube growth" evidence=IMP] [GO:0005829 "cytosol" evidence=IDA] InterPro:IPR011424 InterPro:IPR017937 Pfam:PF07649 PROSITE:PS00194 EMBL:CP002684 GenomeReviews:CT485782_GR GO:GO:0005829 GO:GO:0046686 GO:GO:0009860 InterPro:IPR012336

Tissue	Identification	# of reads (stress)	# of reads (control)	Fold Change	p-value	Description
						Gene3D:G3DSA:3.40.30.10 SUPFAM:SSF52833 GO:GO:0045454 PROSITE:PS51352 GO:GO:0080092 GO:GO:0010183 eggNOG:COG0526 GO:GO:0047134 EMBL:AC004473 EMBL:AY065433 EMBL:AY117231 EMBL:AY087396 IPI:IPI00537432 PIR:T02292 RefSeq:NP_564756.1 UniGene:At.10685 UniGene:At.64112 PDB:1V5N PDBsum:1V5N ProteinModelPortal:O80763 SMR:O80763 IntAct:O80763 PRIDE:O80763 EnsemblPlants:AT1G60420.1 GeneID:842337 KEGG:ath:AT1G60420 TAIR:At1g60420 HOGENOM:HBG594354 InParanoid:O80763 OMA:QMPWLAL PhylomeDB:O80763 ProtClustDB:CLSN2917334 ArrayExpress:O80763 Genevestigator:O80763 Uniprot:O80763
Root	Isotig01460	16	70	-2.258	1.49E-10	TAIR|locus:2195623 - symbol:AT1G60420 species:3702 "Arabidopsis thaliana" [GO:0016209 "antioxidant activity" evidence=IEA] [GO:0016491 "oxidoreductase activity" evidence=IEA] [GO:0046686 "response to cadmium ion" evidence=IEP] [GO:0009860 "pollen tube growth" evidence=IMP] [GO:0010183 "pollen tube guidance" evidence=IMP] [GO:0080092 "regulation of pollen tube growth" evidence=IMP] [GO:0005829 "cytosol" evidence=IDA] InterPro:IPR011424 InterPro:IPR017937 Pfam:PF07649 PROSITE:PS00194 EMBL:CP002684 GenomeReviews:CT485782_GR GO:GO:0005829 GO:GO:0046686 GO:GO:0009860 InterPro:IPR012336 Gene3D:G3DSA:3.40.30.10 SUPFAM:SSF52833 GO:GO:0045454 PROSITE:PS51352 GO:GO:0080092 GO:GO:0010183 eggNOG:COG0526 GO:GO:0047134 EMBL:AC004473 EMBL:AY065433 EMBL:AY117231 EMBL:AY087396 IPI:IPI00537432 PIR:T02292 RefSeq:NP_564756.1 UniGene:At.10685 UniGene:At.64112 PDB:1V5N PDBsum:1V5N ProteinModelPortal:O80763 SMR:O80763 IntAct:O80763 PRIDE:O80763 EnsemblPlants:AT1G60420.1 GeneID:842337 KEGG:ath:AT1G60420 TAIR:At1g60420 HOGENOM:HBG594354 InParanoid:O80763 OMA:QMPWLAL PhylomeDB:O80763 ProtClustDB:CLSN2917334 ArrayExpress:O80763 Genevestigator:O80763 Uniprot:O80763
Root	Isotig01461	0	17	-5.216	1.26E-05	TAIR|locus:2083604 - symbol:LAC7 "laccase 7" species:3702 "Arabidopsis thaliana" [GO:0046274 "lignin catabolic process" evidence=IEA] [GO:0048046 "apoplast" evidence=IEA] [GO:0055114 "oxidation-reduction process" evidence=IEA] InterPro:IPR001117 InterPro:IPR002355 InterPro:IPR011706 InterPro:IPR011707 InterPro:IPR017761 Pfam:PF00394 Pfam:PF07731 Pfam:PF07732 PROSITE:PS00079 PROSITE:PS00080 GO:GO:0048046 EMBL:CP002686 GenomeReviews:BA000014_GR GO:GO:0005507 InterPro:IPR008972 Gene3D:G3DSA:2.60.40.420 SUPFAM:SSF49503 EMBL:AC011436 eggNOG:COG2132 HOGENOM:HBG749556 GO:GO:0052716 GO:GO:0046274 PANTHER:PTHR11709:SF9 TIGRFAMs:TIGR03389 HSSP:P37064 EMBL:AK117639 EMBL:BT004971 IPI:IPI00528510 RefSeq:NP_187533.1 UniGene:At.40144 ProteinModelPortal:Q9SR40 SMR:Q9SR40 PRIDE:Q9SR40 EnsemblPlants:AT3G09220.1 GeneID:820078 KEGG:ath:AT3G09220 TAIR:At3g09220 InParanoid:Q9SR40 OMA:WHAHASF PhylomeDB:Q9SR40 ProtClustDB:CLSN2685122 ArrayExpress:Q9SR40 Genevestigator:Q9SR40 Uniprot:Q9SR40
Root	Isotig01469	6	53	-3.272	1.24E-11	TAIR|locus:2204858 - symbol:AT1G02260 "AT1G02260" species:3702 "Arabidopsis thaliana" [GO:0005215 "transporter activity" evidence=ISS] [GO:0015137 "citrate transmembrane transporter activity" evidence=IEA] [GO:0015746 "citrate transport" evidence=IEA] [GO:0016021 "integral to membrane" evidence=IEA;ISS] [GO:0055085 "transmembrane transport" evidence=IEA] InterPro:IPR004680 Pfam:PF03600 GO:GO:0016021 EMBL:CP002684 GenomeReviews:CT485782_GR eggNOG:COG1055 OMA:SANVICA GO:GO:0015137 EMBL:U89959 HOGENOM:HBG727257 EMBL:BT023463 IPI:IPI00519219 RefSeq:NP_171728.2 UniGene:At.48101 STRING:O81915 PRIDE:O81915 EnsemblPlants:AT1G02260.1 GeneID:839344 KEGG:ath:AT1G02260 TAIR:At1g02260 InParanoid:O81915 PhylomeDB:O81915 ProtClustDB:CLSN2918462 Genevestigator:O81915 Uniprot:O81915
Root	Isotig01470	6	59	-3.427	3.13E-13	TAIR|locus:2204858 - symbol:AT1G02260 "AT1G02260" species:3702 "Arabidopsis thaliana" [GO:0005215 "transporter activity" evidence=ISS] [GO:0015137 "citrate transmembrane transporter activity" evidence=IEA] [GO:0015746 "citrate transport" evidence=IEA] [GO:0016021 "integral to membrane" evidence=IEA;ISS] [GO:0055085 "transmembrane transport" evidence=IEA] InterPro:IPR004680 Pfam:PF03600 GO:GO:0016021 EMBL:CP002684 GenomeReviews:CT485782_GR eggNOG:COG1055 OMA:SANVICA GO:GO:0015137 EMBL:U89959 HOGENOM:HBG727257 EMBL:BT023463 IPI:IPI00519219 RefSeq:NP_171728.2 UniGene:At.48101 STRING:O81915 PRIDE:O81915 EnsemblPlants:AT1G02260.1 GeneID:839344 KEGG:ath:AT1G02260 TAIR:At1g02260 InParanoid:O81915 PhylomeDB:O81915 ProtClustDB:CLSN2918462 Genevestigator:O81915 Uniprot:O81915
Root	Isotig01471	6	57	-3.377	1.07E-12	TAIR|locus:2204858 - symbol:AT1G02260 "AT1G02260" species:3702 "Arabidopsis thaliana" [GO:0005215 "transporter activity" evidence=ISS] [GO:0015137 "citrate transmembrane transporter activity" evidence=IEA] [GO:0015746 "citrate transport" evidence=IEA] [GO:0016021 "integral to membrane" evidence=IEA;ISS]

Tissue	Identification	# of reads (stress)	# of reads (control)	Fold Change	p-value	Description
						[GO:0055085 "transmembrane transport" evidence=IEA] InterPro:IPR004680 Pfam:PF03600 GO:GO:0016021 EMBL:CP002684 GenomeReviews:CT485782_GR eggNOG:COG1055 OMA:SANVICA GO:GO:0015137 EMBL:U89959 HOGENOM:HBG727257 EMBL:BT023463 IPI:IPI00519219 RefSeq:NP_171728.2 UniGene:At.48101 STRING:O81915 PRIDE:O81915 EnsemblPlants:AT1G02260.1 GeneID:839344 KEGG:ath:AT1G02260 TAIR:At1g02260 InParanoid:O81915 PhylomeDB:O81915 ProtClustDB:CLSN2918462 Genevestigator:O81915 Uniprot:O81915
Root	Isotig01472	6	33	-2.588	1.92E-06	TAIR|locus:2204858 - symbol:AT1G02260 "AT1G02260" species:3702 "Arabidopsis thaliana" [GO:0005215 "transporter activity" evidence=ISS] [GO:0015137 "citrate transmembrane transporter activity" evidence=IEA] [GO:0015746 "citrate transport" evidence=IEA] [GO:0016021 "integral to membrane" evidence=IEA;ISS] [GO:0055085 "transmembrane transport" evidence=IEA] InterPro:IPR004680 Pfam:PF03600 GO:GO:0016021 EMBL:CP002684 GenomeReviews:CT485782_GR eggNOG:COG1055 OMA:SANVICA GO:GO:0015137 EMBL:U89959 HOGENOM:HBG727257 EMBL:BT023463 IPI:IPI00519219 RefSeq:NP_171728.2 UniGene:At.48101 STRING:O81915 PRIDE:O81915 EnsemblPlants:AT1G02260.1 GeneID:839344 KEGG:ath:AT1G02260 TAIR:At1g02260 InParanoid:O81915 PhylomeDB:O81915 ProtClustDB:CLSN2918462 Genevestigator:O81915 Uniprot:O81915
Root	Isotig01473	0	22	-5.588	6.01E-07	No hit
Root	Isotig01474	0	28	-5.936	1.79E-08	No hit
Root	Isotig01475	0	26	-5.829	5.70E-08	No hit
Root	Isotig01490	25	70	-1.614	3.05E-07	TAIR|locus:2136308 - symbol:CESA2 "AT4G39350" species:3702 "Arabidopsis thaliana" [GO:0016757 "transferase activity, transferring glycosyl groups" evidence=ISS] [GO:0016759 "cellulose synthase activity" evidence=ISS;IMP] [GO:0005886 "plasma membrane" evidence=IDA;TAS] [GO:0009833 "primary cell wall biogenesis" evidence=TAS] [GO:0030244 "cellulose biosynthetic process" evidence=IMP] InterPro:IPR001841 InterPro:IPR005150 Pfam:PF03552 PROSITE:PS50089 SMART:SM00184 Prosite:PS00518 GO:GO:0016021 GO:GO:0005886 EMBL:CP002687 GenomeReviews:CT486007_GR GO:GO:0008270 InterPro:IPR013083 Gene3D:G3DSA:3.30.40.10 GO:GO:0007047 EMBL:AL161595 CAZy:GT2 eggNOG:COG1215 GO:GO:0016760 GO:GO:0030244 EMBL:AL050351 HOGENOM:HBG744549 KO:K10999 EMBL:AF027173 EMBL:AY059858 EMBL:AY093308 IPI:IPI00521119 PIR:T08583 RefSeq:NP_195645.1 UniGene:At.3315 ProteinModelPortal:O48947 SMR:O48947 STRING:O48947 PRIDE:O48947 EnsemblPlants:AT4G39350.1 GeneID:830090 KEGG:ath:AT4G39350 GeneFarm:5085 TAIR:At4g39350 InParanoid:O48947 OMA:IHALENV PhylomeDB:O48947 ProtClustDB:PLN02436 Genevestigator:O48947 GermOnline:AT4G39350 Uniprot:O48947
Root	Isotig01491	26	70	-1.558	6.04E-07	TAIR|locus:2136308 - symbol:CESA2 "AT4G39350" species:3702 "Arabidopsis thaliana" [GO:0016757 "transferase activity, transferring glycosyl groups" evidence=ISS] [GO:0016759 "cellulose synthase activity" evidence=ISS;IMP] [GO:0005886 "plasma membrane" evidence=IDA;TAS] [GO:0009833 "primary cell wall biogenesis" evidence=TAS] [GO:0030244 "cellulose biosynthetic process" evidence=IMP] InterPro:IPR001841 InterPro:IPR005150 Pfam:PF03552 PROSITE:PS50089 SMART:SM00184 Prosite:PS00518 GO:GO:0016021 GO:GO:0005886 EMBL:CP002687 GenomeReviews:CT486007_GR GO:GO:0008270 InterPro:IPR013083 Gene3D:G3DSA:3.30.40.10 GO:GO:0007047 EMBL:AL161595 CAZy:GT2 eggNOG:COG1215 GO:GO:0016760 GO:GO:0030244 EMBL:AL050351 HOGENOM:HBG744549 KO:K10999 EMBL:AF027173 EMBL:AY059858 EMBL:AY093308 IPI:IPI00521119 PIR:T08583 RefSeq:NP_195645.1 UniGene:At.3315 ProteinModelPortal:O48947 SMR:O48947 STRING:O48947 PRIDE:O48947 EnsemblPlants:AT4G39350.1 GeneID:830090 KEGG:ath:AT4G39350 GeneFarm:5085 TAIR:At4g39350 InParanoid:O48947 OMA:IHALENV PhylomeDB:O48947 ProtClustDB:PLN02436 Genevestigator:O48947 GermOnline:AT4G39350 Uniprot:O48947
Root	Isotig01492	23	66	-1.650	4.42E-07	TAIR|locus:2136308 - symbol:CESA2 "AT4G39350" species:3702 "Arabidopsis thaliana" [GO:0016757 "transferase activity, transferring glycosyl groups" evidence=ISS] [GO:0016759 "cellulose synthase activity" evidence=ISS;IMP] [GO:0005886 "plasma membrane" evidence=IDA;TAS] [GO:0009833 "primary cell wall biogenesis" evidence=TAS] [GO:0030244 "cellulose biosynthetic process" evidence=IMP] InterPro:IPR001841 InterPro:IPR005150 Pfam:PF03552 PROSITE:PS50089 SMART:SM00184 Prosite:PS00518 GO:GO:0016021 GO:GO:0005886 EMBL:CP002687 GenomeReviews:CT486007_GR GO:GO:0008270 InterPro:IPR013083 Gene3D:G3DSA:3.30.40.10 GO:GO:0007047 EMBL:AL161595 CAZy:GT2 eggNOG:COG1215 GO:GO:0016760 GO:GO:0030244 EMBL:AL050351 HOGENOM:HBG744549 KO:K10999 EMBL:AF027173 EMBL:AY059858 EMBL:AY093308 IPI:IPI00521119 PIR:T08583 RefSeq:NP_195645.1 UniGene:At.3315 ProteinModelPortal:O48947 SMR:O48947 STRING:O48947 PRIDE:O48947 EnsemblPlants:AT4G39350.1 GeneID:830090

Tissue	Identification	# of reads (stress)	# of reads (control)	Fold Change	p-value	Description
						KEGG:ath:AT4G39350 GeneFarm:5085 TAIR:At4g39350 InParanoid:O48947 OMA:IHALENV PhylomeDB:O48947 ProtClustDB:PLN02436 Genevestigator:O48947 GermOnline:AT4G39350 Uniprot:O48947
Root	Isotig01493	25	69	-1.594	4.74E-07	TAIR|locus:2136308 - symbol:CESA2 "AT4G39350" species:3702 "Arabidopsis thaliana" [GO:0016757 "transferase activity, transferring glycosyl groups" evidence=ISS] [GO:0016759 "cellulose synthase activity" evidence=ISS;IMP] [GO:0005886 "plasma membrane" evidence=IDA;TAS] [GO:0009833 "primary cell wall biogenesis" evidence=TAS] [GO:0030244 "cellulose biosynthetic process" evidence=IMP] InterPro:IPR001841 InterPro:IPR005150 Pfam:PF03552 PROSITE:PS50089 SMART:SM00184 Prosite:PS00518 GO:GO:0016021 GO:GO:0005886 EMBL:CP002687 GenomeReviews:CT486007_GR GO:GO:0008270 InterPro:IPR013083 Gene3D:G3DSA:3.30.40.10 GO:GO:0007047 EMBL:AL161595 CAZy:GT2 eggNOG:COG1215 GO:GO:0016760 GO:GO:0030244 EMBL:AL050351 HOGENOM:HBG744549 KO:K10999 EMBL:AF027173 EMBL:AY059858 EMBL:AY093308 IPI:IPI00521119 PIR:T08583 RefSeq:NP_195645.1 UniGene:At.3315 ProteinModelPortal:O48947 SMR:O48947 STRING:O48947 PRIDE:O48947 EnsemblPlants:AT4G39350.1 GeneID:830090 KEGG:ath:AT4G39350 GeneFarm:5085 TAIR:At4g39350 InParanoid:O48947 OMA:IHALENV PhylomeDB:O48947 ProtClustDB:PLN02436 Genevestigator:O48947 GermOnline:AT4G39350 Uniprot:O48947
Root	Isotig01494	26	69	-1.537	9.31E-07	TAIR|locus:2136308 - symbol:CESA2 "AT4G39350" species:3702 "Arabidopsis thaliana" [GO:0016757 "transferase activity, transferring glycosyl groups" evidence=ISS] [GO:0016759 "cellulose synthase activity" evidence=ISS;IMP] [GO:0005886 "plasma membrane" evidence=IDA;TAS] [GO:0009833 "primary cell wall biogenesis" evidence=TAS] [GO:0030244 "cellulose biosynthetic process" evidence=IMP] InterPro:IPR001841 InterPro:IPR005150 Pfam:PF03552 PROSITE:PS50089 SMART:SM00184 Prosite:PS00518 GO:GO:0016021 GO:GO:0005886 EMBL:CP002687 GenomeReviews:CT486007_GR GO:GO:0008270 InterPro:IPR013083 Gene3D:G3DSA:3.30.40.10 GO:GO:0007047 EMBL:AL161595 CAZy:GT2 eggNOG:COG1215 GO:GO:0016760 GO:GO:0030244 EMBL:AL050351 HOGENOM:HBG744549 KO:K10999 EMBL:AF027173 EMBL:AY059858 EMBL:AY093308 IPI:IPI00521119 PIR:T08583 RefSeq:NP_195645.1 UniGene:At.3315 ProteinModelPortal:O48947 SMR:O48947 STRING:O48947 PRIDE:O48947 EnsemblPlants:AT4G39350.1 GeneID:830090 KEGG:ath:AT4G39350 GeneFarm:5085 TAIR:At4g39350 InParanoid:O48947 OMA:IHALENV PhylomeDB:O48947 ProtClustDB:PLN02436 Genevestigator:O48947 GermOnline:AT4G39350 Uniprot:O48947
Root	Isotig01495	23	65	-1.628	6.92E-07	TAIR|locus:2136308 - symbol:CESA2 "AT4G39350" species:3702 "Arabidopsis thaliana" [GO:0016757 "transferase activity, transferring glycosyl groups" evidence=ISS] [GO:0016759 "cellulose synthase activity" evidence=ISS;IMP] [GO:0005886 "plasma membrane" evidence=IDA;TAS] [GO:0009833 "primary cell wall biogenesis" evidence=TAS] [GO:0030244 "cellulose biosynthetic process" evidence=IMP] InterPro:IPR001841 InterPro:IPR005150 Pfam:PF03552 PROSITE:PS50089 SMART:SM00184 Prosite:PS00518 GO:GO:0016021 GO:GO:0005886 EMBL:CP002687 GenomeReviews:CT486007_GR GO:GO:0008270 InterPro:IPR013083 Gene3D:G3DSA:3.30.40.10 GO:GO:0007047 EMBL:AL161595 CAZy:GT2 eggNOG:COG1215 GO:GO:0016760 GO:GO:0030244 EMBL:AL050351 HOGENOM:HBG744549 KO:K10999 EMBL:AF027173 EMBL:AY059858 EMBL:AY093308 IPI:IPI00521119 PIR:T08583 RefSeq:NP_195645.1 UniGene:At.3315 ProteinModelPortal:O48947 SMR:O48947 STRING:O48947 PRIDE:O48947 EnsemblPlants:AT4G39350.1 GeneID:830090 KEGG:ath:AT4G39350 GeneFarm:5085 TAIR:At4g39350 InParanoid:O48947 OMA:IHALENV PhylomeDB:O48947 ProtClustDB:PLN02436 Genevestigator:O48947 GermOnline:AT4G39350 Uniprot:O48947
Root	Isotig01496	13	40	-1.750	4.15E-05	TAIR|locus:2196245 - symbol:CAT2 "cationic amino acid transporter 2" species:3702 "Arabidopsis thaliana" [GO:0006810 "transport" evidence=IEA;ISS] [GO:0006865 "amino acid transport" evidence=IEA] [GO:0015171 "amino acid transmembrane transporter activity" evidence=IEA] [GO:0016020 "membrane" evidence=ISS] [GO:0055085 "transmembrane transport" evidence=IEA] [GO:0009705 "plant-type vacuole membrane" evidence=IDA] [GO:0005773 "vacuole" evidence=IDA] [GO:0005774 "vacuolar membrane" evidence=IDA] InterPro:IPR002293 PIRSF:PIRSF006060 GO:GO:0016021 EMBL:CP002684 eggNOG:COG0531 PANTHER:PTHR11785 GO:GO:0015171 GO:GO:0009705 InterPro:IPR015606 PANTHER:PTHR11785:SF53 EMBL:AF367304 EMBL:BT000457 EMBL:BT046174 IPI:IPI00542604 RefSeq:NP_849822.1 UniGene:At.43759 ProteinModelPortal:Q9ASS7 PRIDE:Q8H182 EnsemblPlants:AT1G58030.1 GeneID:842170 KEGG:ath:AT1G58030 TAIR:At1g58030 InParanoid:Q9ASS7 OMA:RRIVAGW PhylomeDB:Q9ASS7 ProtClustDB:CLSN2680360 Genevestigator:Q9ASS7 Uniprot:Q9ASS7
Root	Isotig01497	14	40	-1.644	8.83E-05	TAIR|locus:2196245 - symbol:CAT2 "cationic amino acid transporter 2" species:3702 "Arabidopsis thaliana" [GO:0006810 "transport" evidence=IEA;ISS] [GO:0006865 "amino acid transport" evidence=IEA] [GO:0015171 "amino acid transmembrane transporter activity" evidence=IEA] [GO:0016020 "membrane" evidence=ISS]

Tissue	Identification	# of reads (stress)	# of reads (control)	Fold Change	p-value	Description
						[GO:0055085 "transmembrane transport" evidence=IEA] [GO:0009705 "plant-type vacuole membrane" evidence=IDA] [GO:0005773 "vacuole" evidence=IDA] [GO:0005774 "vacuolar membrane" evidence=IDA] InterPro:IPR002293 PIRSF:PIRSF006060 GO:GO:0016021 EMBL:CP002684 eggNOG:COG0531 PANTHER:PTHR11785 GO:GO:0015171 GO:GO:0009705 InterPro:IPR015606 PANTHER:PTHR11785:SF53 EMBL:AF367304 EMBL:BT000457 EMBL:BT046174 IPI:IPI00542604 RefSeq:NP_849822.1 UniGene:At.43759 ProteinModelPortal:Q9ASS7 PRIDE:Q8H182 EnsemblPlants:AT1G58030.1 GeneID:842170 KEGG:ath:AT1G58030 TAIR:At1g58030 InParanoid:Q9ASS7 OMA:RRIVAGW PhylomeDB:Q9ASS7 ProtClustDB:CLSN2680360 Genevestigator:Q9ASS7 Uniprot:Q9ASS7
Root	Isotig01498	14	42	-1.714	3.49E-05	TAIR|locus:2196245 - symbol:CAT2 "cationic amino acid transporter 2" species:3702 "Arabidopsis thaliana" [GO:0006810 "transport" evidence=IEA;ISS] [GO:0006865 "amino acid transport" evidence=IEA] [GO:0015171 "amino acid transmembrane transporter activity" evidence=IEA] [GO:0016020 "membrane" evidence=ISS] [GO:0055085 "transmembrane transport" evidence=IEA] [GO:0009705 "plant-type vacuole membrane" evidence=IDA] [GO:0005773 "vacuole" evidence=IDA] [GO:0005774 "vacuolar membrane" evidence=IDA] InterPro:IPR002293 PIRSF:PIRSF006060 GO:GO:0016021 EMBL:CP002684 eggNOG:COG0531 PANTHER:PTHR11785 GO:GO:0015171 GO:GO:0009705 InterPro:IPR015606 PANTHER:PTHR11785:SF53 EMBL:AF367304 EMBL:BT000457 EMBL:BT046174 IPI:IPI00542604 RefSeq:NP_849822.1 UniGene:At.43759 ProteinModelPortal:Q9ASS7 PRIDE:Q8H182 EnsemblPlants:AT1G58030.1 GeneID:842170 KEGG:ath:AT1G58030 TAIR:At1g58030 InParanoid:Q9ASS7 OMA:RRIVAGW PhylomeDB:Q9ASS7 ProtClustDB:CLSN2680360 Genevestigator:Q9ASS7 Uniprot:Q9ASS7
Root	Isotig01499	14	40	-1.644	8.83E-05	TAIR|locus:2196245 - symbol:CAT2 "cationic amino acid transporter 2" species:3702 "Arabidopsis thaliana" [GO:0006810 "transport" evidence=IEA;ISS] [GO:0006865 "amino acid transport" evidence=IEA] [GO:0015171 "amino acid transmembrane transporter activity" evidence=IEA] [GO:0016020 "membrane" evidence=ISS] [GO:0055085 "transmembrane transport" evidence=IEA] [GO:0009705 "plant-type vacuole membrane" evidence=IDA] [GO:0005773 "vacuole" evidence=IDA] [GO:0005774 "vacuolar membrane" evidence=IDA] InterPro:IPR002293 PIRSF:PIRSF006060 GO:GO:0016021 EMBL:CP002684 eggNOG:COG0531 PANTHER:PTHR11785 GO:GO:0015171 GO:GO:0009705 InterPro:IPR015606 PANTHER:PTHR11785:SF53 EMBL:AF367304 EMBL:BT000457 EMBL:BT046174 IPI:IPI00542604 RefSeq:NP_849822.1 UniGene:At.43759 ProteinModelPortal:Q9ASS7 PRIDE:Q8H182 EnsemblPlants:AT1G58030.1 GeneID:842170 KEGG:ath:AT1G58030 TAIR:At1g58030 InParanoid:Q9ASS7 OMA:RRIVAGW PhylomeDB:Q9ASS7 ProtClustDB:CLSN2680360 Genevestigator:Q9ASS7 Uniprot:Q9ASS7
Root	Isotig01500	15	42	-1.614	7.30E-05	TAIR|locus:2196245 - symbol:CAT2 "cationic amino acid transporter 2" species:3702 "Arabidopsis thaliana" [GO:0006810 "transport" evidence=IEA;ISS] [GO:0006865 "amino acid transport" evidence=IEA] [GO:0015171 "amino acid transmembrane transporter activity" evidence=IEA] [GO:0016020 "membrane" evidence=ISS] [GO:0055085 "transmembrane transport" evidence=IEA] [GO:0009705 "plant-type vacuole membrane" evidence=IDA] [GO:0005773 "vacuole" evidence=IDA] [GO:0005774 "vacuolar membrane" evidence=IDA] InterPro:IPR002293 PIRSF:PIRSF006060 GO:GO:0016021 EMBL:CP002684 eggNOG:COG0531 PANTHER:PTHR11785 GO:GO:0015171 GO:GO:0009705 InterPro:IPR015606 PANTHER:PTHR11785:SF53 EMBL:AF367304 EMBL:BT000457 EMBL:BT046174 IPI:IPI00542604 RefSeq:NP_849822.1 UniGene:At.43759 ProteinModelPortal:Q9ASS7 PRIDE:Q8H182 EnsemblPlants:AT1G58030.1 GeneID:842170 KEGG:ath:AT1G58030 TAIR:At1g58030 InParanoid:Q9ASS7 OMA:RRIVAGW PhylomeDB:Q9ASS7 ProtClustDB:CLSN2680360 Genevestigator:Q9ASS7 Uniprot:Q9ASS7
Root	Isotig01501	15	40	-1.544	0.00017868	TAIR|locus:2196245 - symbol:CAT2 "cationic amino acid transporter 2" species:3702 "Arabidopsis thaliana" [GO:0006810 "transport" evidence=IEA;ISS] [GO:0006865 "amino acid transport" evidence=IEA] [GO:0015171 "amino acid transmembrane transporter activity" evidence=IEA] [GO:0016020 "membrane" evidence=ISS] [GO:0055085 "transmembrane transport" evidence=IEA] [GO:0009705 "plant-type vacuole membrane" evidence=IDA] [GO:0005773 "vacuole" evidence=IDA] [GO:0005774 "vacuolar membrane" evidence=IDA] InterPro:IPR002293 PIRSF:PIRSF006060 GO:GO:0016021 EMBL:CP002684 eggNOG:COG0531 PANTHER:PTHR11785 GO:GO:0015171 GO:GO:0009705 InterPro:IPR015606 PANTHER:PTHR11785:SF53 EMBL:AF367304 EMBL:BT000457 EMBL:BT046174 IPI:IPI00542604 RefSeq:NP_849822.1 UniGene:At.43759 ProteinModelPortal:Q9ASS7 PRIDE:Q8H182 EnsemblPlants:AT1G58030.1 GeneID:842170 KEGG:ath:AT1G58030 TAIR:At1g58030 InParanoid:Q9ASS7 OMA:RRIVAGW PhylomeDB:Q9ASS7 ProtClustDB:CLSN2680360 Genevestigator:Q9ASS7 Uniprot:Q9ASS7

Tissue	Identification	# of reads (stress)	# of reads (control)	Fold Change	p-value	Description
Root	Isotig01508	38	9	1.949	3.95E-05	TAIR|locus:2016407 - symbol:AT1G19450 "AT1G19450" species:3702 "Arabidopsis thaliana" [GO:0005351 "sugar:hydrogen symporter activity" evidence=ISS] [GO:0006810 "transport" evidence=IEA] [GO:0015144 "carbohydrate transmembrane transporter activity" evidence=ISS] [GO:0016020 "membrane" evidence=ISS] [GO:0055085 "transmembrane transport" evidence=IEA] [GO:0005773 "vacuole" evidence=IDA] [GO:0005774 "vacuolar membrane" evidence=IDA] InterPro:IPR003663 InterPro:IPR005828 InterPro:IPR005829 Pfam:PF00083 PRINTS:PR00171 PROSITE:PS00216 PROSITE:PS00217 GO:GO:0016021 EMBL:CP002684 GenomeReviews:CT485782_GR GO:GO:0005774 eggNOG:COG0477 InterPro:IPR016196 SUPFAM:SSF103473 GO:GO:0008643 InterPro:IPR020846 PROSITE:PS50850 HOGENOM:HBG744444 GO:GO:0022891 TIGRFAMs:TIGR00879 EMBL:AC025808 EMBL:AY059848 EMBL:AY093274 IPI:IPI00534617 RefSeq:NP_173377.1 UniGene:At.22941 ProteinModelPortal:Q93YP9 IntAct:Q93YP9 PRIDE:Q93YP9 EnsemblPlants:AT1G19450.1 GeneID:838529 KEGG:ath:AT1G19450 TAIR:At1g19450 InParanoid:Q93YP9 OMA:SIGMTIS PhylomeDB:Q93YP9 ProtClustDB:CLSN2682491 ArrayExpress:Q93YP9 Genevestigator:Q93YP9 GermOnline:AT1G19450 Uniprot:Q93YP9
Root	Isotig01509	38	9	1.949	3.95E-05	TAIR|locus:2016407 - symbol:AT1G19450 "AT1G19450" species:3702 "Arabidopsis thaliana" [GO:0005351 "sugar:hydrogen symporter activity" evidence=ISS] [GO:0006810 "transport" evidence=IEA] [GO:0015144 "carbohydrate transmembrane transporter activity" evidence=ISS] [GO:0016020 "membrane" evidence=ISS] [GO:0055085 "transmembrane transport" evidence=IEA] [GO:0005773 "vacuole" evidence=IDA] [GO:0005774 "vacuolar membrane" evidence=IDA] InterPro:IPR003663 InterPro:IPR005828 InterPro:IPR005829 Pfam:PF00083 PRINTS:PR00171 PROSITE:PS00216 PROSITE:PS00217 GO:GO:0016021 EMBL:CP002684 GenomeReviews:CT485782_GR GO:GO:0005774 eggNOG:COG0477 InterPro:IPR016196 SUPFAM:SSF103473 GO:GO:0008643 InterPro:IPR020846 PROSITE:PS50850 HOGENOM:HBG744444 GO:GO:0022891 TIGRFAMs:TIGR00879 EMBL:AC025808 EMBL:AY059848 EMBL:AY093274 IPI:IPI00534617 RefSeq:NP_173377.1 UniGene:At.22941 ProteinModelPortal:Q93YP9 IntAct:Q93YP9 PRIDE:Q93YP9 EnsemblPlants:AT1G19450.1 GeneID:838529 KEGG:ath:AT1G19450 TAIR:At1g19450 InParanoid:Q93YP9 OMA:SIGMTIS PhylomeDB:Q93YP9 ProtClustDB:CLSN2682491 ArrayExpress:Q93YP9 Genevestigator:Q93YP9 GermOnline:AT1G19450 Uniprot:Q93YP9
Root	Isotig01510	37	11	1.621	0.00035625	TAIR|locus:2016407 - symbol:AT1G19450 "AT1G19450" species:3702 "Arabidopsis thaliana" [GO:0005351 "sugar:hydrogen symporter activity" evidence=ISS] [GO:0006810 "transport" evidence=IEA] [GO:0015144 "carbohydrate transmembrane transporter activity" evidence=ISS] [GO:0016020 "membrane" evidence=ISS] [GO:0055085 "transmembrane transport" evidence=IEA] [GO:0005773 "vacuole" evidence=IDA] [GO:0005774 "vacuolar membrane" evidence=IDA] InterPro:IPR003663 InterPro:IPR005828 InterPro:IPR005829 Pfam:PF00083 PRINTS:PR00171 PROSITE:PS00216 PROSITE:PS00217 GO:GO:0016021 EMBL:CP002684 GenomeReviews:CT485782_GR GO:GO:0005774 eggNOG:COG0477 InterPro:IPR016196 SUPFAM:SSF103473 GO:GO:0008643 InterPro:IPR020846 PROSITE:PS50850 HOGENOM:HBG744444 GO:GO:0022891 TIGRFAMs:TIGR00879 EMBL:AC025808 EMBL:AY059848 EMBL:AY093274 IPI:IPI00534617 RefSeq:NP_173377.1 UniGene:At.22941 ProteinModelPortal:Q93YP9 IntAct:Q93YP9 PRIDE:Q93YP9 EnsemblPlants:AT1G19450.1 GeneID:838529 KEGG:ath:AT1G19450 TAIR:At1g19450 InParanoid:Q93YP9 OMA:SIGMTIS PhylomeDB:Q93YP9 ProtClustDB:CLSN2682491 ArrayExpress:Q93YP9 Genevestigator:Q93YP9 GermOnline:AT1G19450 Uniprot:Q93YP9
Root	Isotig01511	37	11	1.621	0.00035625	TAIR|locus:2016407 - symbol:AT1G19450 "AT1G19450" species:3702 "Arabidopsis thaliana" [GO:0005351 "sugar:hydrogen symporter activity" evidence=ISS] [GO:0006810 "transport" evidence=IEA] [GO:0015144 "carbohydrate transmembrane transporter activity" evidence=ISS] [GO:0016020 "membrane" evidence=ISS] [GO:0055085 "transmembrane transport" evidence=IEA] [GO:0005773 "vacuole" evidence=IDA] [GO:0005774 "vacuolar membrane" evidence=IDA] InterPro:IPR003663 InterPro:IPR005828 InterPro:IPR005829 Pfam:PF00083 PRINTS:PR00171 PROSITE:PS00216 PROSITE:PS00217 GO:GO:0016021 EMBL:CP002684 GenomeReviews:CT485782_GR GO:GO:0005774 eggNOG:COG0477 InterPro:IPR016196 SUPFAM:SSF103473 GO:GO:0008643 InterPro:IPR020846 PROSITE:PS50850 HOGENOM:HBG744444 GO:GO:0022891 TIGRFAMs:TIGR00879 EMBL:AC025808 EMBL:AY059848 EMBL:AY093274 IPI:IPI00534617 RefSeq:NP_173377.1 UniGene:At.22941 ProteinModelPortal:Q93YP9 IntAct:Q93YP9 PRIDE:Q93YP9 EnsemblPlants:AT1G19450.1 GeneID:838529 KEGG:ath:AT1G19450 TAIR:At1g19450 InParanoid:Q93YP9 OMA:SIGMTIS PhylomeDB:Q93YP9 ProtClustDB:CLSN2682491 ArrayExpress:Q93YP9 Genevestigator:Q93YP9 GermOnline:AT1G19450 Uniprot:Q93YP9

Tissue	Identification	# of reads (stress)	# of reads (control)	Fold Change	p-value	Description
Root	Isotig01512	36	10	1.719	0.000240715	TAIR|locus:2025132 - symbol:ERDL6 "AT1G75220" species:3702 "Arabidopsis thaliana" [GO:0005351 "sugar:hydrogen symporter activity" evidence=ISS] [GO:0006810 "transport" evidence=IEA] [GO:0015144 "carbohydrate transmembrane transporter activity" evidence=ISS] [GO:0016020 "membrane" evidence=ISS] [GO:0055085 "transmembrane transport" evidence=IEA] [GO:0005774 "vacuolar membrane" evidence=IDA] [GO:0005794 "Golgi apparatus" evidence=IDA] InterPro:IPR003663 InterPro:IPR005828 InterPro:IPR005829 Pfam:PF00083 PRINTS:PR00171 PROSITE:PS00216 PROSITE:PS00217 GO:GO:0016021 EMBL:CP002684 GenomeReviews:CT485782_GR GO:GO:0005774 eggNOG:COG0477 InterPro:IPR016196 SUPFAM:SSF103473 GO:GO:0008643 InterPro:IPR020846 PROSITE:PS50850 HOGENOM:HBG744444 GO:GO:0022891 TIGRFAMs:TIGR00879 EMBL:AC025814 ProtClustDB:CLSN2682491 EMBL:AF412060 EMBL:AY124845 IPI:IPI00528914 PIR:E96782 RefSeq:NP_177658.1 UniGene:At.22004 ProteinModelPortal:Q9FRL3 IntAct:Q9FRL3 PRIDE:Q9FRL3 ProMEX:Q9FRL3 EnsemblPlants:AT1G75220.1 GeneID:843859 KEGG:ath:AT1G75220 TAIR:At1g75220 InParanoid:Q9FRL3 OMA:CISFAKD PhylomeDB:Q9FRL3 Genevestigator:Q9FRL3 GermOnline:AT1G75220 Uniprot:Q9FRL3
Root	Isotig01513	36	10	1.719	0.000240715	TAIR|locus:2025132 - symbol:ERDL6 "AT1G75220" species:3702 "Arabidopsis thaliana" [GO:0005351 "sugar:hydrogen symporter activity" evidence=ISS] [GO:0006810 "transport" evidence=IEA] [GO:0015144 "carbohydrate transmembrane transporter activity" evidence=ISS] [GO:0016020 "membrane" evidence=ISS] [GO:0055085 "transmembrane transport" evidence=IEA] [GO:0005774 "vacuolar membrane" evidence=IDA] [GO:0005794 "Golgi apparatus" evidence=IDA] InterPro:IPR003663 InterPro:IPR005828 InterPro:IPR005829 Pfam:PF00083 PRINTS:PR00171 PROSITE:PS00216 PROSITE:PS00217 GO:GO:0016021 EMBL:CP002684 GenomeReviews:CT485782_GR GO:GO:0005774 eggNOG:COG0477 InterPro:IPR016196 SUPFAM:SSF103473 GO:GO:0008643 InterPro:IPR020846 PROSITE:PS50850 HOGENOM:HBG744444 GO:GO:0022891 TIGRFAMs:TIGR00879 EMBL:AC025814 ProtClustDB:CLSN2682491 EMBL:AF412060 EMBL:AY124845 IPI:IPI00528914 PIR:E96782 RefSeq:NP_177658.1 UniGene:At.22004 ProteinModelPortal:Q9FRL3 IntAct:Q9FRL3 PRIDE:Q9FRL3 ProMEX:Q9FRL3 EnsemblPlants:AT1G75220.1 GeneID:843859 KEGG:ath:AT1G75220 TAIR:At1g75220 InParanoid:Q9FRL3 OMA:CISFAKD PhylomeDB:Q9FRL3 Genevestigator:Q9FRL3 GermOnline:AT1G75220 Uniprot:Q9FRL3
Root	Isotig01514	9	27	-1.714	0.000905257	TAIR|locus:2040721 - symbol:SLT1 "AT2G37570" species:3702 "Arabidopsis thaliana" [GO:0003674 "molecular_function" evidence=ND] [GO:0042538 "hyperosmotic salinity response" evidence=IMP] EMBL:AC004684 EMBL:CP002685 GenomeReviews:CT485783_GR GO:GO:0042538 UniGene:At.12789 EMBL:AF213400 EMBL:AY054667 EMBL:AY081508 IPI:IPI00542332 PIR:T02523 RefSeq:NP_565864.1 UniGene:At.74051 ProteinModelPortal:O80926 SMR:O80926 EnsemblPlants:AT2G37570.1 GeneID:818333 KEGG:ath:AT2G37570 TAIR:At2g37570 eggNOG:NOG282356 HOGENOM:HBG591490 InParanoid:O80926 OMA:SEHCPPG PhylomeDB:O80926 ProtClustDB:CLSN2688307 ArrayExpress:O80926 Genevestigator:O80926 Uniprot:O80926
Root	Isotig01515	9	27	-1.714	0.000905257	TAIR|locus:2040721 - symbol:SLT1 "AT2G37570" species:3702 "Arabidopsis thaliana" [GO:0003674 "molecular_function" evidence=ND] [GO:0042538 "hyperosmotic salinity response" evidence=IMP] EMBL:AC004684 EMBL:CP002685 GenomeReviews:CT485783_GR GO:GO:0042538 UniGene:At.12789 EMBL:AF213400 EMBL:AY054667 EMBL:AY081508 IPI:IPI00542332 PIR:T02523 RefSeq:NP_565864.1 UniGene:At.74051 ProteinModelPortal:O80926 SMR:O80926 EnsemblPlants:AT2G37570.1 GeneID:818333 KEGG:ath:AT2G37570 TAIR:At2g37570 eggNOG:NOG282356 HOGENOM:HBG591490 InParanoid:O80926 OMA:SEHCPPG PhylomeDB:O80926 ProtClustDB:CLSN2688307 ArrayExpress:O80926 Genevestigator:O80926 Uniprot:O80926
Root	Isotig01516	9	27	-1.714	0.000905257	TAIR|locus:2040721 - symbol:SLT1 "AT2G37570" species:3702 "Arabidopsis thaliana" [GO:0003674 "molecular_function" evidence=ND] [GO:0042538 "hyperosmotic salinity response" evidence=IMP] EMBL:AC004684 EMBL:CP002685 GenomeReviews:CT485783_GR GO:GO:0042538 UniGene:At.12789 EMBL:AF213400 EMBL:AY054667 EMBL:AY081508 IPI:IPI00542332 PIR:T02523 RefSeq:NP_565864.1 UniGene:At.74051 ProteinModelPortal:O80926 SMR:O80926 EnsemblPlants:AT2G37570.1 GeneID:818333 KEGG:ath:AT2G37570 TAIR:At2g37570 eggNOG:NOG282356 HOGENOM:HBG591490 InParanoid:O80926 OMA:SEHCPPG PhylomeDB:O80926 ProtClustDB:CLSN2688307 ArrayExpress:O80926 Genevestigator:O80926 Uniprot:O80926
Root	Isotig01517	9	27	-1.714	0.000905257	TAIR|locus:2040721 - symbol:SLT1 "AT2G37570" species:3702 "Arabidopsis thaliana" [GO:0003674 "molecular_function" evidence=ND] [GO:0042538 "hyperosmotic salinity response" evidence=IMP] EMBL:AC004684 EMBL:CP002685 GenomeReviews:CT485783_GR GO:GO:0042538 UniGene:At.12789 EMBL:AF213400 EMBL:AY054667 EMBL:AY081508 IPI:IPI00542332 PIR:T02523 RefSeq:NP_565864.1 UniGene:At.74051 ProteinModelPortal:O80926 SMR:O80926 EnsemblPlants:AT2G37570.1 GeneID:818333 KEGG:ath:AT2G37570

Tissue	Identification	# of reads (stress)	# of reads (control)	Fold Change	p-value	Description
						TAIR:At2g37570 eggNOG:NOG282356 HOGENOM:HBG591490 InParanoid:O80926 OMA:SEHCPPG PhylomeDB:O80926 ProtClustDB:CLSN2688307 ArrayExpress:O80926 Genevestigator:O80926 Uniprot:O80926
Root	Isotig01518	7	25	-1.965	0.000455586	TAIR|locus:2040721 - symbol:SLT1 "AT2G37570" species:3702 "Arabidopsis thaliana" [GO:0003674 "molecular_function" evidence=ND] [GO:0042538 "hyperosmotic salinity response" evidence=IMP] EMBL:AC004684 EMBL:CP002685 GenomeReviews:CT485783_GR GO:GO:0042538 UniGene:At.12789 EMBL:AF213400 EMBL:AY054667 EMBL:AY081508 IPI:IPI00542332 PIR:T02523 RefSeq:NP_565864.1 UniGene:At.74051 ProteinModelPortal:O80926 SMR:O80926 EnsemblPlants:AT2G37570.1 GeneID:818333 KEGG:ath:AT2G37570 TAIR:At2g37570 eggNOG:NOG282356 HOGENOM:HBG591490 InParanoid:O80926 OMA:SEHCPPG PhylomeDB:O80926 ProtClustDB:CLSN2688307 ArrayExpress:O80926 Genevestigator:O80926 Uniprot:O80926
Root	Isotig01519	7	25	-1.965	0.000455586	TAIR|locus:2040721 - symbol:SLT1 "AT2G37570" species:3702 "Arabidopsis thaliana" [GO:0003674 "molecular_function" evidence=ND] [GO:0042538 "hyperosmotic salinity response" evidence=IMP] EMBL:AC004684 EMBL:CP002685 GenomeReviews:CT485783_GR GO:GO:0042538 UniGene:At.12789 EMBL:AF213400 EMBL:AY054667 EMBL:AY081508 IPI:IPI00542332 PIR:T02523 RefSeq:NP_565864.1 UniGene:At.74051 ProteinModelPortal:O80926 SMR:O80926 EnsemblPlants:AT2G37570.1 GeneID:818333 KEGG:ath:AT2G37570 TAIR:At2g37570 eggNOG:NOG282356 HOGENOM:HBG591490 InParanoid:O80926 OMA:SEHCPPG PhylomeDB:O80926 ProtClustDB:CLSN2688307 ArrayExpress:O80926 Genevestigator:O80926 Uniprot:O80926
Root	Isotig01520	15	37	-1.432	0.000655976	TAIR|locus:2030170 - symbol:CKL2 "casein kinase 1-like protein 2" species:3702 "Arabidopsis thaliana" [GO:0004672 "protein kinase activity" evidence=IEA] [GO:0004674 "protein serine/threonine kinase activity" evidence=IEA] [GO:0005524 "ATP binding" evidence=IEA] [GO:0016301 "kinase activity" evidence=ISS] [GO:0005634 "nucleus" evidence=IDA] [GO:0005737 "cytoplasm" evidence=IDA] [GO:0005886 "plasma membrane" evidence=IDA] InterPro:IPR000719 InterPro:IPR008271 InterPro:IPR011009 InterPro:IPR017441 Pfam:PF00069 PROSITE:PS00107 PROSITE:PS00108 PROSITE:PS50011 EMBL:CP002684 GenomeReviews:CT485782_GR GO:GO:0005886 GO:GO:0005524 GO:GO:0005634 GO:GO:0005737 eggNOG:COG0515 SUPFAM:SSF56112 GO:GO:0004674 HOGENOM:HBG755340 EMBL:AC010926 KO:K02218 OMA:MDLRVGN HSSP:Q06486 EMBL:AY059850 EMBL:BT010328 EMBL:AY943843 IPI:IPI00517772 PIR:H96751 RefSeq:NP_177415.1 UniGene:At.19326 ProteinModelPortal:Q9CAI5 SMR:Q9CAI5 IntAct:Q9CAI5 STRING:Q9CAI5 PRIDE:Q9CAI5 DNASU:843603 EnsemblPlants:AT1G72710.1 GeneID:843603 KEGG:ath:AT1G72710 TAIR:At1g72710 InParanoid:Q9CAI5 PhylomeDB:Q9CAI5 ProtClustDB:CLSN2912728 Genevestigator:Q9CAI5 Uniprot:Q9CAI5
Root	Isotig01523	14	37	-1.531	0.000341051	TAIR|locus:2030170 - symbol:CKL2 "casein kinase 1-like protein 2" species:3702 "Arabidopsis thaliana" [GO:0004672 "protein kinase activity" evidence=IEA] [GO:0004674 "protein serine/threonine kinase activity" evidence=IEA] [GO:0005524 "ATP binding" evidence=IEA] [GO:0016301 "kinase activity" evidence=ISS] [GO:0005634 "nucleus" evidence=IDA] [GO:0005737 "cytoplasm" evidence=IDA] [GO:0005886 "plasma membrane" evidence=IDA] InterPro:IPR000719 InterPro:IPR008271 InterPro:IPR011009 InterPro:IPR017441 Pfam:PF00069 PROSITE:PS00107 PROSITE:PS00108 PROSITE:PS50011 EMBL:CP002684 GenomeReviews:CT485782_GR GO:GO:0005886 GO:GO:0005524 GO:GO:0005634 GO:GO:0005737 eggNOG:COG0515 SUPFAM:SSF56112 GO:GO:0004674 HOGENOM:HBG755340 EMBL:AC010926 KO:K02218 OMA:MDLRVGN HSSP:Q06486 EMBL:AY059850 EMBL:BT010328 EMBL:AY943843 IPI:IPI00517772 PIR:H96751 RefSeq:NP_177415.1 UniGene:At.19326 ProteinModelPortal:Q9CAI5 SMR:Q9CAI5 IntAct:Q9CAI5 STRING:Q9CAI5 PRIDE:Q9CAI5 DNASU:843603 EnsemblPlants:AT1G72710.1 GeneID:843603 KEGG:ath:AT1G72710 TAIR:At1g72710 InParanoid:Q9CAI5 PhylomeDB:Q9CAI5 ProtClustDB:CLSN2912728 Genevestigator:Q9CAI5 Uniprot:Q9CAI5
Root	Isotig01524	16	38	-1.377	0.000799708	TAIR|locus:2030170 - symbol:CKL2 "casein kinase 1-like protein 2" species:3702 "Arabidopsis thaliana" [GO:0004672 "protein kinase activity" evidence=IEA] [GO:0004674 "protein serine/threonine kinase activity" evidence=IEA] [GO:0005524 "ATP binding" evidence=IEA] [GO:0016301 "kinase activity" evidence=ISS] [GO:0005634 "nucleus" evidence=IDA] [GO:0005737 "cytoplasm" evidence=IDA] [GO:0005886 "plasma membrane" evidence=IDA] InterPro:IPR000719 InterPro:IPR008271 InterPro:IPR011009 InterPro:IPR017441 Pfam:PF00069 PROSITE:PS00107 PROSITE:PS00108 PROSITE:PS50011 EMBL:CP002684 GenomeReviews:CT485782_GR GO:GO:0005886 GO:GO:0005524 GO:GO:0005634 GO:GO:0005737 eggNOG:COG0515 SUPFAM:SSF56112 GO:GO:0004674 HOGENOM:HBG755340 EMBL:AC010926 KO:K02218 OMA:MDLRVGN HSSP:Q06486 EMBL:AY059850 EMBL:BT010328 EMBL:AY943843 IPI:IPI00517772 PIR:H96751 RefSeq:NP_177415.1 UniGene:At.19326 ProteinModelPortal:Q9CAI5 SMR:Q9CAI5 IntAct:Q9CAI5 STRING:Q9CAI5 PRIDE:Q9CAI5 DNASU:843603 EnsemblPlants:AT1G72710.1 GeneID:843603 KEGG:ath:AT1G72710 TAIR:At1g72710 InParanoid:Q9CAI5 PhylomeDB:Q9CAI5 ProtClustDB:CLSN2912728 Genevestigator:Q9CAI5 Uniprot:Q9CAI5

Tissue	Identification	# of reads (stress)	# of reads (control)	Fold Change	p-value	Description
Root	Isotig01525	16	38	-1.377	0.000799708	TAIR|locus:2030170 - symbol:CKL2 "casein kinase 1-like protein 2" species:3702 "Arabidopsis thaliana" [GO:0004672 "protein kinase activity" evidence=IEA] [GO:0004674 "protein serine/threonine kinase activity" evidence=IEA] [GO:0005524 "ATP binding" evidence=IEA] [GO:0016301 "kinase activity" evidence=ISS] [GO:0005634 "nucleus" evidence=IDA] [GO:0005737 "cytoplasm" evidence=IDA] [GO:0005886 "plasma membrane" evidence=IDA] InterPro:IPR000719 InterPro:IPR008271 InterPro:IPR011009 InterPro:IPR017441 Pfam:PF00069 PROSITE:PS00107 PROSITE:PS00108 PROSITE:PS50011 EMBL:CP002684 GenomeReviews:CT485782_GR GO:GO:0005886 GO:GO:0005524 GO:GO:0005634 GO:GO:0005737 eggNOG:COG0515 SUPFAM:SSF56112 GO:GO:0004674 HOGENOM:HBG755340 EMBL:AC010926 KO:K02218 OMA:MDLRVGN HSSP:Q06486 EMBL:AY059850 EMBL:BT010328 EMBL:AY943843 IPI:IPI00517772 PIR:H96751 RefSeq:NP_177415.1 UniGene:At.19326 ProteinModelPortal:Q9CAI5 SMR:Q9CAI5 IntAct:Q9CAI5 STRING:Q9CAI5 PRIDE:Q9CAI5 DNASU:843603 EnsemblPlants:AT1G72710.1 GeneID:843603 KEGG:ath:AT1G72710 TAIR:At1g72710 InParanoid:Q9CAI5 PhylomeDB:Q9CAI5 ProtClustDB:CLSN2912728 Genevestigator:Q9CAI5 Uniprot:Q9CAI5
Root	Isotig01532	7	55	-3.103	1.58E-11	TAIR|locus:2198035 - symbol:PMEAMT "AT1G48600" species:3702 "Arabidopsis thaliana" [GO:0000234 "phosphoethanolamine N-methyltransferase activity" evidence=ISS] [GO:0006656 "phosphatidylcholine biosynthetic process" evidence=IGI;IDA] [GO:0052667 "phosphomethylethanolamine N-methyltransferase activity" evidence=IDA] EMBL:CP002684 GenomeReviews:CT485782_GR GO:GO:0005737 EMBL:AC020889 eggNOG:COG0500 GO:GO:0006656 HOGENOM:HBG320513 ProtClustDB:PLN02336 GO:GO:0000234 EMBL:AF428454 EMBL:AY063866 EMBL:AY133811 IPI:IPI00539739 PIR:F96525 RefSeq:NP_175293.1 UniGene:At.22426 ProteinModelPortal:Q944H0 SMR:Q944H0 PRIDE:Q944H0 EnsemblPlants:AT1G48600.1 GeneID:841281 KEGG:ath:AT1G48600 TAIR:At1g48600 InParanoid:Q944H0 PhylomeDB:Q944H0 ArrayExpress:Q944H0 Genevestigator:Q944H0 GO:GO:0052667 Uniprot:Q944H0
Root	Isotig01533	6	53	-3.272	1.24E-11	TAIR|locus:2198035 - symbol:PMEAMT "AT1G48600" species:3702 "Arabidopsis thaliana" [GO:0000234 "phosphoethanolamine N-methyltransferase activity" evidence=ISS] [GO:0006656 "phosphatidylcholine biosynthetic process" evidence=IGI;IDA] [GO:0052667 "phosphomethylethanolamine N-methyltransferase activity" evidence=IDA] EMBL:CP002684 GenomeReviews:CT485782_GR GO:GO:0005737 EMBL:AC020889 eggNOG:COG0500 GO:GO:0006656 HOGENOM:HBG320513 ProtClustDB:PLN02336 GO:GO:0000234 EMBL:AF428454 EMBL:AY063866 EMBL:AY133811 IPI:IPI00539739 PIR:F96525 RefSeq:NP_175293.1 UniGene:At.22426 ProteinModelPortal:Q944H0 SMR:Q944H0 PRIDE:Q944H0 EnsemblPlants:AT1G48600.1 GeneID:841281 KEGG:ath:AT1G48600 TAIR:At1g48600 InParanoid:Q944H0 PhylomeDB:Q944H0 ArrayExpress:Q944H0 Genevestigator:Q944H0 GO:GO:0052667 Uniprot:Q944H0
Root	Isotig01534	7	55	-3.103	1.58E-11	TAIR|locus:2088535 - symbol:XPL1 "AT3G18000" species:3702 "Arabidopsis thaliana" [GO:0000234 "phosphoethanolamine N-methyltransferase activity" evidence=IGI;ISS] [GO:0005737 "cytoplasm" evidence=ISS] [GO:0006656 "phosphatidylcholine biosynthetic process" evidence=IGI] [GO:0008168 "methyltransferase activity" evidence=ISS] [GO:0009555 "pollen development" evidence=IMP] [GO:0009826 "unidimensional cell growth" evidence=IMP] [GO:0042425 "choline biosynthetic process" evidence=IMP] [GO:0048528 "post-embryonic root development" evidence=IMP] [GO:0009860 "pollen tube growth" evidence=IMP] [GO:0010183 "pollen tube guidance" evidence=IMP] [GO:0005829 "cytosol" evidence=IDA] InterPro:IPR013216 Pfam:PF08241 GO:GO:0005829 EMBL:CP002686 GenomeReviews:BA000014_GR GO:GO:0009555 GO:GO:0009860 eggNOG:COG0500 GO:GO:0006656 GO:GO:0010183 EMBL:AB019230 GO:GO:0048528 EMBL:AF197940 EMBL:AF367299 EMBL:AY058175 EMBL:AY091683 EMBL:AY093093 IPI:IPI00542357 RefSeq:NP_188427.2 UniGene:At.21159 ProteinModelPortal:Q9FR44 SMR:Q9FR44 STRING:Q9FR44 PRIDE:Q9FR44 EnsemblPlants:AT3G18000.1 GeneID:821324 KEGG:ath:AT3G18000 TAIR:At3g18000 HOGENOM:HBG320513 InParanoid:Q9FR44 KO:K05929 OMA:DQRWGLF PhylomeDB:Q9FR44 ProtClustDB:PLN02336 Genevestigator:Q9FR44 GermOnline:AT3G18000 GO:GO:0000234 GO:GO:0042425 Uniprot:Q9FR44
Root	Isotig01535	6	53	-3.272	1.24E-11	TAIR|locus:2088535 - symbol:XPL1 "AT3G18000" species:3702 "Arabidopsis thaliana" [GO:0000234 "phosphoethanolamine N-methyltransferase activity" evidence=IGI;ISS] [GO:0005737 "cytoplasm" evidence=ISS] [GO:0006656 "phosphatidylcholine biosynthetic process" evidence=IGI] [GO:0008168 "methyltransferase activity" evidence=ISS] [GO:0009555 "pollen development" evidence=IMP] [GO:0009826 "unidimensional cell growth" evidence=IMP] [GO:0042425 "choline biosynthetic process" evidence=IMP] [GO:0048528 "post-embryonic root development" evidence=IMP] [GO:0009860 "pollen tube growth" evidence=IMP] [GO:0010183 "pollen tube guidance" evidence=IMP] [GO:0005829 "cytosol" evidence=IDA] InterPro:IPR013216 Pfam:PF08241 GO:GO:0005829 EMBL:CP002686 GenomeReviews:BA000014_GR GO:GO:0009555 GO:GO:0009860

Tissue	Identification	# of reads (stress)	# of reads (control)	Fold Change	p-value	Description
						eggNOG:COG0500 GO:GO:0006656 GO:GO:0010183 EMBL:AB019230 GO:GO:0048528 EMBL:AF197940 EMBL:AF367299 EMBL:AY058175 EMBL:AY091683 EMBL:AY093093 IPI:IPI00542357 RefSeq:NP_188427.2 UniGene:At.21159 ProteinModelPortal:Q9FR44 SMR:Q9FR44 STRING:Q9FR44 PRIDE:Q9FR44 EnsemblPlants:AT3G18000.1 GeneID:821324 KEGG:ath:AT3G18000 TAIR:At3g18000 HOGENOM:HBG320513 InParanoid:Q9FR44 KO:K05929 OMA:DQRWGLF PhylomeDB:Q9FR44 ProtClustDB:PLN02336 Genevestigator:Q9FR44 GermOnline:AT3G18000 GO:GO:0000234 GO:GO:0042425 Uniprot:Q9FR44
Root	Isotig01536	6	52	-3.244	2.28E-11	TAIR|locus:2198035 - symbol:PMEAMT "AT1G48600" species:3702 "Arabidopsis thaliana" [GO:0000234 "phosphoethanolamine N-methyltransferase activity" evidence=ISS] [GO:0006656 "phosphatidylcholine biosynthetic process" evidence=IGI;IDA] [GO:0052667 "phosphomethylethanolamine N-methyltransferase activity" evidence=IDA] EMBL:CP002684 GenomeReviews:CT485782_GR GO:GO:0005737 EMBL:AC020889 eggNOG:COG0500 GO:GO:0006656 HOGENOM:HBG320513 ProtClustDB:PLN02336 GO:GO:0000234 EMBL:AF428454 EMBL:AY063866 EMBL:AY133811 IPI:IPI00539739 PIR:F96525 RefSeq:NP_175293.1 UniGene:At.22426 ProteinModelPortal:Q944H0 SMR:Q944H0 PRIDE:Q944H0 EnsemblPlants:AT1G48600.1 GeneID:841281 KEGG:ath:AT1G48600 TAIR:At1g48600 InParanoid:Q944H0 PhylomeDB:Q944H0 ArrayExpress:Q944H0 Genevestigator:Q944H0 GO:GO:0052667 Uniprot:Q944H0
Root	Isotig01537	6	52	-3.244	2.28E-11	TAIR|locus:2088535 - symbol:XPL1 "AT3G18000" species:3702 "Arabidopsis thaliana" [GO:0000234 "phosphoethanolamine N-methyltransferase activity" evidence=IGI;ISS] [GO:0005737 "cytoplasm" evidence=ISS] [GO:0006656 "phosphatidylcholine biosynthetic process" evidence=IGI] [GO:0008168 "methyltransferase activity" evidence=ISS] [GO:0009555 "pollen development" evidence=IMP] [GO:0009826 "unidimensional cell growth" evidence=IMP] [GO:0042425 "choline biosynthetic process" evidence=IMP] [GO:0048528 "post-embryonic root development" evidence=IMP] [GO:0009860 "pollen tube growth" evidence=IMP] [GO:0010183 "pollen tube guidance" evidence=IMP] [GO:0005829 "cytosol" evidence=IDA] InterPro:IPR013216 Pfam:PF08241 GO:GO:0005829 EMBL:CP002686 GenomeReviews:BA000014_GR GO:GO:0009555 GO:GO:0009860 eggNOG:COG0500 GO:GO:0006656 GO:GO:0010183 EMBL:AB019230 GO:GO:0048528 EMBL:AF197940 EMBL:AF367299 EMBL:AY058175 EMBL:AY091683 EMBL:AY093093 IPI:IPI00542357 RefSeq:NP_188427.2 UniGene:At.21159 ProteinModelPortal:Q9FR44 SMR:Q9FR44 STRING:Q9FR44 PRIDE:Q9FR44 EnsemblPlants:AT3G18000.1 GeneID:821324 KEGG:ath:AT3G18000 TAIR:At3g18000 HOGENOM:HBG320513 InParanoid:Q9FR44 KO:K05929 OMA:DQRWGLF PhylomeDB:Q9FR44 ProtClustDB:PLN02336 Genevestigator:Q9FR44 GermOnline:AT3G18000 GO:GO:0000234 GO:GO:0042425 Uniprot:Q9FR44
Root	Isotig01538	42	2	4.263	2.46E-10	No hit
Root	Isotig01539	51	3	3.958	8.66E-12	No hit
Root	Isotig01540	31	3	3.240	8.04E-07	No hit
Root	Isotig01541	40	4	3.193	2.57E-08	No hit
Root	Isotig01543	0	11	-4.588	0.000569522	No hit
Root	Isotig01549	0	13	-4.829	0.000155955	TAIR|locus:2089784 - symbol:AT3G17770 species:3702 "Arabidopsis thaliana" [GO:0004371 "glycerone kinase activity" evidence=IEA;ISS] [GO:0005524 "ATP binding" evidence=IEA] [GO:0006071 "glycerol metabolic process" evidence=IEA;ISS] InterPro:IPR004006 InterPro:IPR004007 InterPro:IPR012734 Pfam:PF02733 Pfam:PF02734 PROSITE:PS51480 PROSITE:PS51481 GO:GO:0005524 EMBL:CP002686 GenomeReviews:BA000014_GR GO:GO:0006071 eggNOG:COG2376 HOGENOM:HBG473357 GO:GO:0004371 SUPFAM:SSF101473 TIGRFAMs:TIGR02361 OMA:GFVGPGL EMBL:BT023733 IPI:IPI00529007 RefSeq:NP_188404.1 UniGene:At.38659 UniGene:At.67784 ProteinModelPortal:Q494P3 SMR:Q494P3 PRIDE:Q494P3 EnsemblPlants:AT3G17770.1 GeneID:821045 KEGG:ath:AT3G17770 TAIR:At3g17770 InParanoid:Q494P3 PhylomeDB:Q494P3 ProtClustDB:CLSN2684338 Genevestigator:Q494P3 Uniprot:Q494P3
Root	Isotig01574	0	28	-5.936	1.79E-08	TAIR|locus:2010718 - symbol:NAS4 "AT1G56430" species:3702 "Arabidopsis thaliana" [GO:0005575 "cellular_component" evidence=ND] [GO:0030410 "nicotianamine synthase activity" evidence=IGI] [GO:0030418 "nicotianamine biosynthetic process" evidence=IGI] InterPro:IPR004298 Pfam:PF03059 PROSITE:PS51142 EMBL:CP002684 GenomeReviews:CT485782_GR EMBL:AC058785 HOGENOM:HBG594037 KO:K05953 ProtClustDB:PLN03075 GO:GO:0030410 GO:GO:0030418 EMBL:AY099758 EMBL:AY128890 IPI:IPI00540066 PIR:B96606 RefSeq:NP_176038.1 UniGene:At.66920 ProteinModelPortal:Q9C7X5 SMR:Q9C7X5 IntAct:Q9C7X5 STRING:Q9C7X5 PRIDE:Q9C7X5 DNASU:842096 EnsemblPlants:AT1G56430.1 GeneID:842096 KEGG:ath:AT1G56430 TAIR:At1g56430 eggNOG:NOG305103 InParanoid:Q9C7X5 OMA:SENIQEM PhylomeDB:Q9C7X5 ArrayExpress:Q9C7X5 Genevestigator:Q9C7X5 GermOnline:AT1G56430 Uniprot:Q9C7X5

Tissue	Identification	# of reads (stress)	# of reads (control)	Fold Change	p-value	Description
Root	Isotig01575	0	23	-5.653	3.32E-07	TAIR|locus:2010718 - symbol:NAS4 "AT1G56430" species:3702 "Arabidopsis thaliana" [GO:0005575 "cellular_component" evidence=ND] [GO:0030410 "nicotianamine synthase activity" evidence=IGI] [GO:0030418 "nicotianamine biosynthetic process" evidence=IGI] InterPro:IPR004298 Pfam:PF03059 PROSITE:PS51142 EMBL:CP002684 GenomeReviews:CT485782_GR EMBL:AC058785 HOGENOM:HBG594037 KO:K05953 ProtClustDB:PLN03075 GO:GO:0030410 GO:GO:0030418 EMBL:AY099758 EMBL:AY128890 IPI:IPI00540066 PIR:B96606 RefSeq:NP_176038.1 UniGene:At.66920 ProteinModelPortal:Q9C7X5 SMR:Q9C7X5 IntAct:Q9C7X5 STRING:Q9C7X5 PRIDE:Q9C7X5 DNASU:842096 EnsemblPlants:AT1G56430.1 GeneID:842096 KEGG:ath:AT1G56430 TAIR:At1g56430 eggNOG:NOG305103 InParanoid:Q9C7X5 OMA:SENIQEM PhylomeDB:Q9C7X5 ArrayExpress:Q9C7X5 Genevestigator:Q9C7X5 GermOnline:AT1G56430 Uniprot:Q9C7X5
Root	Isotig01576	0	29	-5.987	1.01E-08	TAIR|locus:2010718 - symbol:NAS4 "AT1G56430" species:3702 "Arabidopsis thaliana" [GO:0005575 "cellular_component" evidence=ND] [GO:0030410 "nicotianamine synthase activity" evidence=IGI] [GO:0030418 "nicotianamine biosynthetic process" evidence=IGI] InterPro:IPR004298 Pfam:PF03059 PROSITE:PS51142 EMBL:CP002684 GenomeReviews:CT485782_GR EMBL:AC058785 HOGENOM:HBG594037 KO:K05953 ProtClustDB:PLN03075 GO:GO:0030410 GO:GO:0030418 EMBL:AY099758 EMBL:AY128890 IPI:IPI00540066 PIR:B96606 RefSeq:NP_176038.1 UniGene:At.66920 ProteinModelPortal:Q9C7X5 SMR:Q9C7X5 IntAct:Q9C7X5 STRING:Q9C7X5 PRIDE:Q9C7X5 DNASU:842096 EnsemblPlants:AT1G56430.1 GeneID:842096 KEGG:ath:AT1G56430 TAIR:At1g56430 eggNOG:NOG305103 InParanoid:Q9C7X5 OMA:SENIQEM PhylomeDB:Q9C7X5 ArrayExpress:Q9C7X5 Genevestigator:Q9C7X5 GermOnline:AT1G56430 Uniprot:Q9C7X5
Root	Isotig01577	0	23	-5.653	3.32E-07	TAIR|locus:2010718 - symbol:NAS4 "AT1G56430" species:3702 "Arabidopsis thaliana" [GO:0005575 "cellular_component" evidence=ND] [GO:0030410 "nicotianamine synthase activity" evidence=IGI] [GO:0030418 "nicotianamine biosynthetic process" evidence=IGI] InterPro:IPR004298 Pfam:PF03059 PROSITE:PS51142 EMBL:CP002684 GenomeReviews:CT485782_GR EMBL:AC058785 HOGENOM:HBG594037 KO:K05953 ProtClustDB:PLN03075 GO:GO:0030410 GO:GO:0030418 EMBL:AY099758 EMBL:AY128890 IPI:IPI00540066 PIR:B96606 RefSeq:NP_176038.1 UniGene:At.66920 ProteinModelPortal:Q9C7X5 SMR:Q9C7X5 IntAct:Q9C7X5 STRING:Q9C7X5 PRIDE:Q9C7X5 DNASU:842096 EnsemblPlants:AT1G56430.1 GeneID:842096 KEGG:ath:AT1G56430 TAIR:At1g56430 eggNOG:NOG305103 InParanoid:Q9C7X5 OMA:SENIQEM PhylomeDB:Q9C7X5 ArrayExpress:Q9C7X5 Genevestigator:Q9C7X5 GermOnline:AT1G56430 Uniprot:Q9C7X5
Root	Isotig01578	0	18	-5.299	6.77E-06	TAIR|locus:2010718 - symbol:NAS4 "AT1G56430" species:3702 "Arabidopsis thaliana" [GO:0005575 "cellular_component" evidence=ND] [GO:0030410 "nicotianamine synthase activity" evidence=IGI] [GO:0030418 "nicotianamine biosynthetic process" evidence=IGI] InterPro:IPR004298 Pfam:PF03059 PROSITE:PS51142 EMBL:CP002684 GenomeReviews:CT485782_GR EMBL:AC058785 HOGENOM:HBG594037 KO:K05953 ProtClustDB:PLN03075 GO:GO:0030410 GO:GO:0030418 EMBL:AY099758 EMBL:AY128890 IPI:IPI00540066 PIR:B96606 RefSeq:NP_176038.1 UniGene:At.66920 ProteinModelPortal:Q9C7X5 SMR:Q9C7X5 IntAct:Q9C7X5 STRING:Q9C7X5 PRIDE:Q9C7X5 DNASU:842096 EnsemblPlants:AT1G56430.1 GeneID:842096 KEGG:ath:AT1G56430 TAIR:At1g56430 eggNOG:NOG305103 InParanoid:Q9C7X5 OMA:SENIQEM PhylomeDB:Q9C7X5 ArrayExpress:Q9C7X5 Genevestigator:Q9C7X5 GermOnline:AT1G56430 Uniprot:Q9C7X5
Root	Isotig01579	0	19	-5.377	3.67E-06	TAIR|locus:2010718 - symbol:NAS4 "AT1G56430" species:3702 "Arabidopsis thaliana" [GO:0005575 "cellular_component" evidence=ND] [GO:0030410 "nicotianamine synthase activity" evidence=IGI] [GO:0030418 "nicotianamine biosynthetic process" evidence=IGI] InterPro:IPR004298 Pfam:PF03059 PROSITE:PS51142 EMBL:CP002684 GenomeReviews:CT485782_GR EMBL:AC058785 HOGENOM:HBG594037 KO:K05953 ProtClustDB:PLN03075 GO:GO:0030410 GO:GO:0030418 EMBL:AY099758 EMBL:AY128890 IPI:IPI00540066 PIR:B96606 RefSeq:NP_176038.1 UniGene:At.66920 ProteinModelPortal:Q9C7X5 SMR:Q9C7X5 IntAct:Q9C7X5 STRING:Q9C7X5 PRIDE:Q9C7X5 DNASU:842096 EnsemblPlants:AT1G56430.1 GeneID:842096 KEGG:ath:AT1G56430 TAIR:At1g56430 eggNOG:NOG305103 InParanoid:Q9C7X5 OMA:SENIQEM PhylomeDB:Q9C7X5 ArrayExpress:Q9C7X5 Genevestigator:Q9C7X5 GermOnline:AT1G56430 Uniprot:Q9C7X5
Root	Isotig01580	68	7	3.151	5.28E-13	No hit
Root	Isotig01585	26	3	2.987	1.35E-05	No hit
Root	Isotig01598	37	103	-1.606	6.08E-10	TAIR|locus:2170318 - symbol:ATMS1 "AT5G17920" species:3702 "Arabidopsis thaliana" [GO:0003871 "5-methyltetrahydropteroyltriglutamate-homocysteine S-methyltransferase activity" evidence=ISS] [GO:0005829 "cytosol" evidence=ISS;IDA] [GO:0009086 "methionine biosynthetic process" evidence=ISS] [GO:0008705 "methionine synthase activity" evidence=IDA] [GO:0046686 "response to cadmium ion" evidence=IEP] [GO:0005507 "copper ion binding" evidence=IDA] [GO:0005774 "vacuolar membrane" evidence=IDA] [GO:0005886 "plasma

Tissue	Identification	# of reads (stress)	# of reads (control)	Fold Change	p-value	Description
						membrane" evidence=IDA] [GO:0016020 "membrane" evidence=IDA] [GO:0009651 "response to salt stress" evidence=IEP] [GO:0005777 "peroxisome" evidence=IDA] [GO:0009507 "chloroplast" evidence=IDA] [GO:0048046 "apoplast" evidence=IDA] [GO:0009570 "chloroplast stroma" evidence=IDA] [GO:0010043 "response to zinc ion" evidence=IEP] [GO:0009506 "plasmodesma" evidence=IDA] [GO:0005794 "Golgi apparatus" evidence=RCA] InterPro:IPR002629 InterPro:IPR006276 InterPro:IPR013215 Pfam:PF01717 Pfam:PF08267 PIRSF:PIRSF000382 GO:GO:0005829 GO:GO:0005886 GO:GO:0009506 GO:GO:0048046 GO:GO:0009570 GO:GO:0046686 GO:GO:0005774 EMBL:CP002688 GenomeReviews:BA000015_GR GO:GO:0005777 GO:GO:0010043 GO:GO:0008270 GO:GO:0005507 GO:GO:0009651 EMBL:AB011480 UniGene:At.24550 EMBL:U97200 EMBL:AF370522 EMBL:AY048201 EMBL:AY056098 EMBL:AY057499 EMBL:AY069876 EMBL:AY070771 EMBL:AY091692 IPI:IPI00522440 RefSeq:NP_001078599.1 RefSeq:NP_197294.1 UniGene:At.22340 UniGene:At.71028 PDB:1U1H PDB:1U1J PDB:1U1U PDB:1U22 PDBsum:1U1H PDBsum:1U1J PDBsum:1U1U PDBsum:1U22 ProteinModelPortal:O50008 SMR:O50008 IntAct:O50008 STRING:O50008 SWISS-2DPAGE:O50008 PRIDE:O50008 ProMEX:O50008 EnsemblPlants:AT5G17920.1 EnsemblPlants:AT5G17920.2 GeneID:831660 KEGG:ath:AT5G17920 TAIR:At5g17920 eggNOG:COG0620 HOGENOM:HBG287495 InParanoid:O50008 KO:K00549 OMA:NIWANDF PhylomeDB:O50008 ProtClustDB:PLN02475 BRENDA:2.1.1.14 Genevestigator:O50008 GermOnline:AT5G17920 GO:GO:0003871 GO:GO:0008705 TIGRFAMs:TIGR01371 Uniprot:O50008
Root	Isotig01599	48	112	-1.351	1.39E-08	TAIR|locus:2170318 - symbol:ATMS1 "AT5G17920" species:3702 "Arabidopsis thaliana" [GO:0003871 "5-methyltetrahydropteroyltriglutamate-homocysteine S-methyltransferase activity" evidence=ISS] [GO:0005829 "cytosol" evidence=ISS;IDA] [GO:0009086 "methionine biosynthetic process" evidence=ISS] [GO:0008705 "methionine synthase activity" evidence=IDA] [GO:0046686 "response to cadmium ion" evidence=IEP] [GO:0005507 "copper ion binding" evidence=IDA] [GO:0005774 "vacuolar membrane" evidence=IDA] [GO:0005886 "plasma membrane" evidence=IDA] [GO:0016020 "membrane" evidence=IDA] [GO:0009651 "response to salt stress" evidence=IEP] [GO:0005777 "peroxisome" evidence=IDA] [GO:0009507 "chloroplast" evidence=IDA] [GO:0048046 "apoplast" evidence=IDA] [GO:0009570 "chloroplast stroma" evidence=IDA] [GO:0010043 "response to zinc ion" evidence=IEP] [GO:0009506 "plasmodesma" evidence=IDA] [GO:0005794 "Golgi apparatus" evidence=RCA] InterPro:IPR002629 InterPro:IPR006276 InterPro:IPR013215 Pfam:PF01717 Pfam:PF08267 PIRSF:PIRSF000382 GO:GO:0005829 GO:GO:0005886 GO:GO:0009506 GO:GO:0048046 GO:GO:0009570 GO:GO:0046686 GO:GO:0005774 EMBL:CP002688 GenomeReviews:BA000015_GR GO:GO:0005777 GO:GO:0010043 GO:GO:0008270 GO:GO:0005507 GO:GO:0009651 EMBL:AB011480 UniGene:At.24550 EMBL:U97200 EMBL:AF370522 EMBL:AY048201 EMBL:AY056098 EMBL:AY057499 EMBL:AY069876 EMBL:AY070771 EMBL:AY091692 IPI:IPI00522440 RefSeq:NP_001078599.1 RefSeq:NP_197294.1 UniGene:At.22340 UniGene:At.71028 PDB:1U1H PDB:1U1J PDB:1U1U PDB:1U22 PDBsum:1U1H PDBsum:1U1J PDBsum:1U1U PDBsum:1U22 ProteinModelPortal:O50008 SMR:O50008 IntAct:O50008 STRING:O50008 SWISS-2DPAGE:O50008 PRIDE:O50008 ProMEX:O50008 EnsemblPlants:AT5G17920.1 EnsemblPlants:AT5G17920.2 GeneID:831660 KEGG:ath:AT5G17920 TAIR:At5g17920 eggNOG:COG0620 HOGENOM:HBG287495 InParanoid:O50008 KO:K00549 OMA:NIWANDF PhylomeDB:O50008 ProtClustDB:PLN02475 BRENDA:2.1.1.14 Genevestigator:O50008 GermOnline:AT5G17920 GO:GO:0003871 GO:GO:0008705 TIGRFAMs:TIGR01371 Uniprot:O50008
Root	Isotig01600	43	96	-1.288	4.15E-07	TAIR|locus:2170318 - symbol:ATMS1 "AT5G17920" species:3702 "Arabidopsis thaliana" [GO:0003871 "5-methyltetrahydropteroyltriglutamate-homocysteine S-methyltransferase activity" evidence=ISS] [GO:0005829 "cytosol" evidence=ISS;IDA] [GO:0009086 "methionine biosynthetic process" evidence=ISS] [GO:0008705 "methionine synthase activity" evidence=IDA] [GO:0046686 "response to cadmium ion" evidence=IEP] [GO:0005507 "copper ion binding" evidence=IDA] [GO:0005774 "vacuolar membrane" evidence=IDA] [GO:0005886 "plasma membrane" evidence=IDA] [GO:0016020 "membrane" evidence=IDA] [GO:0009651 "response to salt stress" evidence=IEP] [GO:0005777 "peroxisome" evidence=IDA] [GO:0009507 "chloroplast" evidence=IDA] [GO:0048046 "apoplast" evidence=IDA] [GO:0009570 "chloroplast stroma" evidence=IDA] [GO:0010043 "response to zinc ion" evidence=IEP] [GO:0009506 "plasmodesma" evidence=IDA] [GO:0005794 "Golgi apparatus" evidence=RCA] InterPro:IPR002629 InterPro:IPR006276 InterPro:IPR013215 Pfam:PF01717 Pfam:PF08267 PIRSF:PIRSF000382 GO:GO:0005829 GO:GO:0005886 GO:GO:0009506 GO:GO:0048046 GO:GO:0009570 GO:GO:0046686 GO:GO:0005774 EMBL:CP002688 GenomeReviews:BA000015_GR GO:GO:0005777 GO:GO:0010043 GO:GO:0008270 GO:GO:0005507 GO:GO:0009651 EMBL:AB011480 UniGene:At.24550 EMBL:U97200 EMBL:AF370522 EMBL:AY048201 EMBL:AY056098 EMBL:AY057499 EMBL:AY069876 EMBL:AY070771

Tissue	Identification	# of reads (stress)	# of reads (control)	Fold Change	p-value	Description
						EMBL:AY091692 IPI:IPI00522440 RefSeq:NP_001078599.1 RefSeq:NP_197294.1 UniGene:At.22340 UniGene:At.71028 PDB:1U1H PDB:1U1J PDB:1U1U PDB:1U22 PDBsum:1U1H PDBsum:1U1J PDBsum:1U1U PDBsum:1U22 ProteinModelPortal:O50008 SMR:O50008 IntAct:O50008 STRING:O50008 SWISS-2DPAGE:O50008 PRIDE:O50008 ProMEX:O50008 EnsemblPlants:AT5G17920.1 EnsemblPlants:AT5G17920.2 GeneID:831660 KEGG:ath:AT5G17920 TAIR:At5g17920 eggNOG:COG0620 HOGENOM:HBG287495 InParanoid:O50008 KO:K00549 OMA:NIWANDF PhylomeDB:O50008 ProtClustDB:PLN02475 BRENDA:2.1.1.14 Genevestigator:O50008 GermOnline:AT5G17920 GO:GO:0003871 GO:GO:0008705 TIGRFAMs:TIGR01371 Uniprot:O50008
Root	Isotig01610	74	31	1.126	0.000132088	TAIR|locus:2036451 - symbol:AT1G50920 "AT1G50920" species:3702 "Arabidopsis thaliana" [GO:0000166 "nucleotide binding" evidence=ISS] [GO:0005525 "GTP binding" evidence=IEA] [GO:0016020 "membrane" evidence=IDA] InterPro:IPR006073 InterPro:IPR010674 InterPro:IPR024926 Pfam:PF06858 PIRSF:PIRSF038919 PRINTS:PR00326 GO:GO:0005525 EMBL:CP002684 GenomeReviews:CT485782_GR GO:GO:0005730 GO:GO:0016020 GO:GO:0042254 EMBL:AC079284 EMBL:AY096652 IPI:IPI00535306 PIR:C96546 RefSeq:NP_175505.1 UniGene:At.25047 UniGene:At.27911 ProteinModelPortal:Q9C6I8 SMR:Q9C6I8 STRING:Q9C6I8 PRIDE:Q9C6I8 EnsemblPlants:AT1G50920.1 GeneID:841514 KEGG:ath:AT1G50920 TAIR:At1g50920 eggNOG:COG1084 HOGENOM:HBG628350 InParanoid:Q9C6I8 KO:K06943 OMA:FHSIRPL PhylomeDB:Q9C6I8 ProtClustDB:CLSN2718068 ArrayExpress:Q9C6I8 Genevestigator:Q9C6I8 GermOnline:AT1G50920 InterPro:IPR012973 Pfam:PF08155 Uniprot:Q9C6I8
Root	Isotig01612	63	28	1.041	0.000938592	TAIR|locus:2036451 - symbol:AT1G50920 "AT1G50920" species:3702 "Arabidopsis thaliana" [GO:0000166 "nucleotide binding" evidence=ISS] [GO:0005525 "GTP binding" evidence=IEA] [GO:0016020 "membrane" evidence=IDA] InterPro:IPR006073 InterPro:IPR010674 InterPro:IPR024926 Pfam:PF06858 PIRSF:PIRSF038919 PRINTS:PR00326 GO:GO:0005525 EMBL:CP002684 GenomeReviews:CT485782_GR GO:GO:0005730 GO:GO:0016020 GO:GO:0042254 EMBL:AC079284 EMBL:AY096652 IPI:IPI00535306 PIR:C96546 RefSeq:NP_175505.1 UniGene:At.25047 UniGene:At.27911 ProteinModelPortal:Q9C6I8 SMR:Q9C6I8 STRING:Q9C6I8 PRIDE:Q9C6I8 EnsemblPlants:AT1G50920.1 GeneID:841514 KEGG:ath:AT1G50920 TAIR:At1g50920 eggNOG:COG1084 HOGENOM:HBG628350 InParanoid:Q9C6I8 KO:K06943 OMA:FHSIRPL PhylomeDB:Q9C6I8 ProtClustDB:CLSN2718068 ArrayExpress:Q9C6I8 Genevestigator:Q9C6I8 GermOnline:AT1G50920 InterPro:IPR012973 Pfam:PF08155 Uniprot:Q9C6I8
Root	Isotig01617	19	47	-1.436	0.000118801	TAIR|locus:2118701 - symbol:AT4G30600 species:3702 "Arabidopsis thaliana" [GO:0000166 "nucleotide binding" evidence=IEA] [GO:0003924 "GTPase activity" evidence=IEA] [GO:0005047 "signal recognition particle binding" evidence=IEA] [GO:0005525 "GTP binding" evidence=IEA;ISS] [GO:0005786 "signal recognition particle, endoplasmic reticulum targeting" evidence=ISS] [GO:0006605 "protein targeting" evidence=ISS] [GO:0006614 "SRP-dependent cotranslational protein targeting to membrane" evidence=IEA] [GO:0006886 "intracellular protein transport" evidence=IEA] [GO:0017111 "nucleoside-triphosphatase activity" evidence=IEA] [GO:0005783 "endoplasmic reticulum" evidence=IDA] InterPro:IPR000897 InterPro:IPR003593 InterPro:IPR007222 InterPro:IPR011012 InterPro:IPR013822 Pfam:PF00448 Pfam:PF02881 Pfam:PF04086 PROSITE:PS00300 SMART:SM00382 SMART:SM00962 GO:GO:0005525 EMBL:CP002687 GenomeReviews:CT486007_GR GO:GO:0004872 GO:GO:0003924 EMBL:AL161577 SUPFAM:SSF64356 eggNOG:COG0552 HOGENOM:HBG562580 GO:GO:0006614 Gene3D:G3DSA:1.20.120.140 SUPFAM:SSF47364 GO:GO:0005785 GO:GO:0005047 KO:K13431 OMA:QGESMMD EMBL:AY065066 EMBL:AY133612 IPI:IPI00534083 PIR:A85358 RefSeq:NP_194789.1 UniGene:At.4581 HSSP:P83749 ProteinModelPortal:Q9M0A0 SMR:Q9M0A0 STRING:Q9M0A0 PRIDE:Q9M0A0 EnsemblPlants:AT4G30600.1 GeneID:829183 KEGG:ath:AT4G30600 TAIR:At4g30600 InParanoid:Q9M0A0 PhylomeDB:Q9M0A0 ProtClustDB:CLSN2685629 ArrayExpress:Q9M0A0 Genevestigator:Q9M0A0 Uniprot:Q9M0A0
Root	Isotig01618	19	43	-1.307	0.000609156	TAIR|locus:2118701 - symbol:AT4G30600 species:3702 "Arabidopsis thaliana" [GO:0000166 "nucleotide binding" evidence=IEA] [GO:0003924 "GTPase activity" evidence=IEA] [GO:0005047 "signal recognition particle binding" evidence=IEA] [GO:0005525 "GTP binding" evidence=IEA;ISS] [GO:0005786 "signal recognition particle, endoplasmic reticulum targeting" evidence=ISS] [GO:0006605 "protein targeting" evidence=ISS] [GO:0006614 "SRP-dependent cotranslational protein targeting to membrane" evidence=IEA] [GO:0006886 "intracellular protein transport" evidence=IEA] [GO:0017111 "nucleoside-triphosphatase activity" evidence=IEA] [GO:0005783 "endoplasmic reticulum" evidence=IDA] InterPro:IPR000897 InterPro:IPR003593 InterPro:IPR007222 InterPro:IPR011012 InterPro:IPR013822 Pfam:PF00448 Pfam:PF02881 Pfam:PF04086 PROSITE:PS00300

Tissue	Identification	# of reads (stress)	# of reads (control)	Fold Change	p-value	Description
						SMART:SM00382 SMART:SM00962 GO:GO:0005525 EMBL:CP002687 GenomeReviews:CT486007_GR GO:GO:0004872 GO:GO:0003924 EMBL:AL161577 SUPFAM:SSF64356 eggNOG:COG0552 HOGENOM:HBG562580 GO:GO:0006614 Gene3D:G3DSA:1.20.120.140 SUPFAM:SSF47364 GO:GO:0005785 GO:GO:0005047 KO:K13431 OMA:QGESMMD EMBL:AY065066 EMBL:AY133612 IPI:IPI00534083 PIR:A85358 RefSeq:NP_194789.1 UniGene:At.4581 HSSP:P83749 ProteinModelPortal:Q9M0A0 SMR:Q9M0A0 STRING:Q9M0A0 PRIDE:Q9M0A0 EnsemblPlants:AT4G30600.1 GeneID:829183 KEGG:ath:AT4G30600 TAIR:At4g30600 InParanoid:Q9M0A0 PhylomeDB:Q9M0A0 ProtClustDB:CLSN2685629 ArrayExpress:Q9M0A0 Genevestigator:Q9M0A0 Uniprot:Q9M0A0
Root	Isotig01628	14	1	3.678	0.000484579	No hit
Root	Isotig01629	25	0	5.515	3.68E-07	No hit
Root	Isotig01630	30	0	5.778	2.46E-08	No hit
Root	Isotig01632	30	0	5.778	2.46E-08	No hit
Root	Isotig01633	19	0	5.119	1.05E-05	No hit
Root	Isotig01639	105	14	2.778	3.30E-17	No hit
Root	Isotig01640	105	14	2.778	3.30E-17	No hit
Root	Isotig01641	105	14	2.778	3.30E-17	No hit
Root	Isotig01659	53	0	6.599	2.10E-13	TAIR|locus:2179117 - symbol:AT5G01300 "AT5G01300" species:3702 "Arabidopsis thaliana" [GO:0005575 "cellular_component" evidence=ND] [GO:0008150 "biological_process" evidence=ND] [GO:0008429 "phosphatidylethanolamine binding" evidence=ISS] Pfam:PF01161 EMBL:CP002688 GenomeReviews:BA000015_GR InterPro:IPR008914 Gene3D:G3DSA:3.90.280.10 SUPFAM:SSF49777 EMBL:AL161746 KO:K06910 HOGENOM:HBG649023 InterPro:IPR005247 TIGRFAMs:TIGR00481 UniGene:At.33912 UniGene:At.48967 EMBL:AY065369 EMBL:AY096477 IPI:IPI00535873 PIR:T48152 RefSeq:NP_195750.1 HSSP:P77368 ProteinModelPortal:Q9M042 SMR:Q9M042 PRIDE:Q9M042 ProMEX:Q9M042 EnsemblPlants:AT5G01300.1 GeneID:830983 KEGG:ath:AT5G01300 TAIR:At5g01300 InParanoid:Q9M042 OMA:LEWYNVP PhylomeDB:Q9M042 ProtClustDB:CLSN2687113 ArrayExpress:Q9M042 Genevestigator:Q9M042 Uniprot:Q9M042
Root	Isotig01660	59	0	6.754	1.18E-14	TAIR|locus:2179117 - symbol:AT5G01300 "AT5G01300" species:3702 "Arabidopsis thaliana" [GO:0005575 "cellular_component" evidence=ND] [GO:0008150 "biological_process" evidence=ND] [GO:0008429 "phosphatidylethanolamine binding" evidence=ISS] Pfam:PF01161 EMBL:CP002688 GenomeReviews:BA000015_GR InterPro:IPR008914 Gene3D:G3DSA:3.90.280.10 SUPFAM:SSF49777 EMBL:AL161746 KO:K06910 HOGENOM:HBG649023 InterPro:IPR005247 TIGRFAMs:TIGR00481 UniGene:At.33912 UniGene:At.48967 EMBL:AY065369 EMBL:AY096477 IPI:IPI00535873 PIR:T48152 RefSeq:NP_195750.1 HSSP:P77368 ProteinModelPortal:Q9M042 SMR:Q9M042 PRIDE:Q9M042 ProMEX:Q9M042 EnsemblPlants:AT5G01300.1 GeneID:830983 KEGG:ath:AT5G01300 TAIR:At5g01300 InParanoid:Q9M042 OMA:LEWYNVP PhylomeDB:Q9M042 ProtClustDB:CLSN2687113 ArrayExpress:Q9M042 Genevestigator:Q9M042 Uniprot:Q9M042
Root	Isotig01661	57	8	2.704	8.93E-10	TAIR|locus:2086102 - symbol:AT3G17020 "AT3G17020" species:3702 "Arabidopsis thaliana" [GO:0003674 "molecular_function" evidence=ND] [GO:0006950 "response to stress" evidence=ISS] [GO:0009409 "response to cold" evidence=IEP] [GO:0005886 "plasma membrane" evidence=IDA] InterPro:IPR006016 Pfam:PF00582 GO:GO:0005886 EMBL:CP002686 GenomeReviews:BA000014_GR GO:GO:0009409 EMBL:AB026636 InterPro:IPR014729 Gene3D:G3DSA:3.40.50.620 HOGENOM:HBG733992 InterPro:IPR006015 PRINTS:PR01438 EMBL:AF378888 EMBL:AY050480 IPI:IPI00534820 RefSeq:NP_566564.1 UniGene:At.24032 ProteinModelPortal:Q9LSP5 SMR:Q9LSP5 IntAct:Q9LSP5 STRING:Q9LSP5 PRIDE:Q9LSP5 EnsemblPlants:AT3G17020.1 GeneID:820958 KEGG:ath:AT3G17020 TAIR:At3g17020 eggNOG:NOG302561 InParanoid:Q9LSP5 OMA:GEMQLWE PhylomeDB:Q9LSP5 ProtClustDB:CLSN2688467 Genevestigator:Q9LSP5 Uniprot:Q9LSP5
Root	Isotig01662	64	8	2.871	2.12E-11	TAIR|locus:2086102 - symbol:AT3G17020 "AT3G17020" species:3702 "Arabidopsis thaliana" [GO:0003674 "molecular_function" evidence=ND] [GO:0006950 "response to stress" evidence=ISS] [GO:0009409 "response to cold" evidence=IEP] [GO:0005886 "plasma membrane" evidence=IDA] InterPro:IPR006016 Pfam:PF00582 GO:GO:0005886 EMBL:CP002686 GenomeReviews:BA000014_GR GO:GO:0009409 EMBL:AB026636 InterPro:IPR014729 Gene3D:G3DSA:3.40.50.620 HOGENOM:HBG733992 InterPro:IPR006015 PRINTS:PR01438 EMBL:AF378888 EMBL:AY050480 IPI:IPI00534820 RefSeq:NP_566564.1 UniGene:At.24032

Tissue	Identification	# of reads (stress)	# of reads (control)	Fold Change	p-value	Description
						ProteinModelPortal:Q9LSP5 SMR:Q9LSP5 IntAct:Q9LSP5 STRING:Q9LSP5 PRIDE:Q9LSP5 EnsemblPlants:AT3G17020.1 GeneID:820958 KEGG:ath:AT3G17020 TAIR:At3g17020 eggNOG:NOG302561 InParanoid:Q9LSP5 OMA:GEMQLWE PhylomeDB:Q9LSP5 ProtClustDB:CLSN2688467 Genevestigator:Q9LSP5 Uniprot:Q9LSP5
Root	Isotig01663	57	8	2.704	8.93E-10	TAIR|locus:2086102 - symbol:AT3G17020 "AT3G17020" species:3702 "Arabidopsis thaliana" [GO:0003674 "molecular_function" evidence=ND] [GO:0006950 "response to stress" evidence=ISS] [GO:0009409 "response to cold" evidence=IEP] [GO:0005886 "plasma membrane" evidence=IDA] InterPro:IPR006016 Pfam:PF00582 GO:GO:0005886 EMBL:CP002686 GenomeReviews:BA000014_GR GO:GO:0009409 EMBL:AB026636 InterPro:IPR014729 Gene3D:G3DSA:3.40.50.620 HOGENOM:HBG733992 InterPro:IPR006015 PRINTS:PR01438 EMBL:AF378888 EMBL:AY050480 IPI:IPI00534820 RefSeq:NP_566564.1 UniGene:At.24032 ProteinModelPortal:Q9LSP5 SMR:Q9LSP5 IntAct:Q9LSP5 STRING:Q9LSP5 PRIDE:Q9LSP5 EnsemblPlants:AT3G17020.1 GeneID:820958 KEGG:ath:AT3G17020 TAIR:At3g17020 eggNOG:NOG302561 InParanoid:Q9LSP5 OMA:GEMQLWE PhylomeDB:Q9LSP5 ProtClustDB:CLSN2688467 Genevestigator:Q9LSP5 Uniprot:Q9LSP5
Root	Isotig01669	12	61	-2.475	2.75E-10	UNIPROTKB|Q43848 - symbol:Q43848 "Transketolase, chloroplastic" species:4113 "Solanum tuberosum" [GO:0004802 "transketolase activity" evidence=ISS] [GO:0009535 "chloroplast thylakoid membrane" evidence=ISS] InterPro:IPR005476 InterPro:IPR005478 InterPro:IPR009014 InterPro:IPR015941 Pfam:PF02780 InterPro:IPR005475 Pfam:PF00456 Pfam:PF02779 InterPro:IPR005474 GO:GO:0046872 GO:GO:0009535 SMART:SM00861 InterPro:IPR020826 Gene3D:G3DSA:3.40.50.920 SUPFAM:SSF52922 PROSITE:PS00801 PROSITE:PS00802 GO:GO:0004802 TIGRFAMs:TIGR00232 EMBL:Z50099 PIR:S58083 ProteinModelPortal:Q43848 SMR:Q43848 PRIDE:Q43848 Uniprot:Q43848
Root	Isotig01670	13	62	-2.383	4.79E-10	TAIR|locus:2101871 - symbol:AT3G60750 species:3702 "Arabidopsis thaliana" [GO:0003824 "catalytic activity" evidence=IEA] [GO:0004802 "transketolase activity" evidence=IEA;ISS] [GO:0009941 "chloroplast envelope" evidence=IDA] [GO:0009507 "chloroplast" evidence=IDA] [GO:0009570 "chloroplast stroma" evidence=IDA] [GO:0046686 "response to cadmium ion" evidence=IEP] [GO:0009651 "response to salt stress" evidence=IEP] [GO:0005829 "cytosol" evidence=RCA] InterPro:IPR005476 InterPro:IPR005478 InterPro:IPR009014 InterPro:IPR015941 Pfam:PF02780 InterPro:IPR005475 Pfam:PF00456 Pfam:PF02779 InterPro:IPR005474 GO:GO:0009570 GO:GO:0046686 EMBL:CP002686 GO:GO:0009941 GO:GO:0009651 SMART:SM00861 InterPro:IPR020826 Gene3D:G3DSA:3.40.50.920 SUPFAM:SSF52922 PROSITE:PS00801 PROSITE:PS00802 GO:GO:0004802 KO:K00615 TIGRFAMs:TIGR00232 OMA:LAFNENV EMBL:AY091094 EMBL:AY133860 IPI:IPI00537782 RefSeq:NP_567103.1 UniGene:At.24156 UniGene:At.68080 HSSP:P23254 ProteinModelPortal:Q8RWV0 SMR:Q8RWV0 IntAct:Q8RWV0 STRING:Q8RWV0 PRIDE:Q8RWV0 ProMEX:Q8RWV0 EnsemblPlants:AT3G60750.1 GeneID:825246 KEGG:ath:AT3G60750 TAIR:At3g60750 InParanoid:Q8RWV0 PhylomeDB:Q8RWV0 ProtClustDB:PLN02790 ArrayExpress:Q8RWV0 Genevestigator:Q8RWV0 Uniprot:Q8RWV0
Root	Isotig01671	14	63	-2.299	8.06E-10	UNIPROTKB|Q43848 - symbol:Q43848 "Transketolase, chloroplastic" species:4113 "Solanum tuberosum" [GO:0004802 "transketolase activity" evidence=ISS] [GO:0009535 "chloroplast thylakoid membrane" evidence=ISS] InterPro:IPR005476 InterPro:IPR005478 InterPro:IPR009014 InterPro:IPR015941 Pfam:PF02780 InterPro:IPR005475 Pfam:PF00456 Pfam:PF02779 InterPro:IPR005474 GO:GO:0046872 GO:GO:0009535 SMART:SM00861 InterPro:IPR020826 Gene3D:G3DSA:3.40.50.920 SUPFAM:SSF52922 PROSITE:PS00801 PROSITE:PS00802 GO:GO:0004802 TIGRFAMs:TIGR00232 EMBL:Z50099 PIR:S58083 ProteinModelPortal:Q43848 SMR:Q43848 PRIDE:Q43848 Uniprot:Q43848
Root	Isotig01672	6	31	-2.498	6.06E-06	UNIPROTKB|Q43848 - symbol:Q43848 "Transketolase, chloroplastic" species:4113 "Solanum tuberosum" [GO:0004802 "transketolase activity" evidence=ISS] [GO:0009535 "chloroplast thylakoid membrane" evidence=ISS] InterPro:IPR005476 InterPro:IPR005478 InterPro:IPR009014 InterPro:IPR015941 Pfam:PF02780 InterPro:IPR005475 Pfam:PF00456 Pfam:PF02779 InterPro:IPR005474 GO:GO:0046872 GO:GO:0009535 SMART:SM00861 InterPro:IPR020826 Gene3D:G3DSA:3.40.50.920 SUPFAM:SSF52922 PROSITE:PS00801 PROSITE:PS00802 GO:GO:0004802 TIGRFAMs:TIGR00232 EMBL:Z50099 PIR:S58083 ProteinModelPortal:Q43848 SMR:Q43848 PRIDE:Q43848 Uniprot:Q43848
Root	Isotig01701	0	35	-6.258	3.45E-10	TAIR|locus:2158187 - symbol:ARA12 "AT5G67360" species:3702 "Arabidopsis thaliana" [GO:0006508 "proteolysis" evidence=ISS] [GO:0004252 "serine-type endopeptidase activity" evidence=IDA] [GO:0005576 "extracellular region" evidence=IDA] [GO:0005618 "cell wall" evidence=IDA] [GO:0009505 "plant-type cell wall" evidence=IDA]

Tissue	Identification	# of reads (stress)	# of reads (control)	Fold Change	p-value	Description
						[GO:0010214 "seed coat development" evidence=IMP] [GO:0048359 "mucilage metabolic process involved seed coat development" evidence=IMP] [GO:0080001 "mucilage extrusion from seed coat" evidence=IMP] [GO:0048046 "apoplast" evidence=IDA] InterPro:IPR000209 InterPro:IPR010259 Pfam:PF00082 Pfam:PF05922 InterPro:IPR003137 Prosite:PS00137 Prosite:PS00138 PANTHER:PTHR10795 Pfam:PF02225 GO:GO:0048046 EMBL:CP002688 GenomeReviews:BA000015_GR GO:GO:0042802 GO:GO:0006508 GO:GO:0004252 GO:GO:0009505 GO:GO:0043086 InterPro:IPR023828 InterPro:IPR015500 Gene3D:G3DSA:3.40.50.200 PRINTS:PR00723 SUPFAM:SSF52743 PROSITE:PS00136 eggNOG:COG1404 EMBL:AB007645 GO:GO:0080001 HOGENOM:HBG591621 EMBL:AF065639 EMBL:AF360285 EMBL:AY091773 EMBL:AY142612 EMBL:BT001082 EMBL:X85974 IPI:IPI00548134 PIR:JC7519 PIR:S52770 RefSeq:NP_569048.1 UniGene:At.23238 UniGene:At.67722 UniGene:At.71531 ProteinModelPortal:O65351 SMR:O65351 STRING:O65351 MEROPS:S08.112 PRIDE:O65351 EnsemblPlants:AT5G67360.1 GeneID:836871 KEGG:ath:AT5G67360 GeneFarm:1964 TAIR:At5g67360 InParanoid:O65351 OMA:TYIVHMA PhylomeDB:O65351 ProtClustDB:CLSN2690100 ArrayExpress:O65351 Genevestigator:O65351 GermOnline:AT5G67360 GO:GO:0048359 Uniprot:O65351
Root	Isotig01702	0	31	-6.083	3.24E-09	TAIR|locus:2158187 - symbol:ARA12 "AT5G67360" species:3702 "Arabidopsis thaliana" [GO:0006508 "proteolysis" evidence=ISS] [GO:0004252 "serine-type endopeptidase activity" evidence=IDA] [GO:0005576 "extracellular region" evidence=IDA] [GO:0005618 "cell wall" evidence=IDA] [GO:0009505 "plant-type cell wall" evidence=IDA] [GO:0010214 "seed coat development" evidence=IMP] [GO:0048359 "mucilage metabolic process involved seed coat development" evidence=IMP] [GO:0080001 "mucilage extrusion from seed coat" evidence=IMP] [GO:0048046 "apoplast" evidence=IDA] InterPro:IPR000209 InterPro:IPR010259 Pfam:PF00082 Pfam:PF05922 InterPro:IPR003137 Prosite:PS00137 Prosite:PS00138 PANTHER:PTHR10795 Pfam:PF02225 GO:GO:0048046 EMBL:CP002688 GenomeReviews:BA000015_GR GO:GO:0042802 GO:GO:0006508 GO:GO:0004252 GO:GO:0009505 GO:GO:0043086 InterPro:IPR023828 InterPro:IPR015500 Gene3D:G3DSA:3.40.50.200 PRINTS:PR00723 SUPFAM:SSF52743 PROSITE:PS00136 eggNOG:COG1404 EMBL:AB007645 GO:GO:0080001 HOGENOM:HBG591621 EMBL:AF065639 EMBL:AF360285 EMBL:AY091773 EMBL:AY142612 EMBL:BT001082 EMBL:X85974 IPI:IPI00548134 PIR:JC7519 PIR:S52770 RefSeq:NP_569048.1 UniGene:At.23238 UniGene:At.67722 UniGene:At.71531 ProteinModelPortal:O65351 SMR:O65351 STRING:O65351 MEROPS:S08.112 PRIDE:O65351 EnsemblPlants:AT5G67360.1 GeneID:836871 KEGG:ath:AT5G67360 GeneFarm:1964 TAIR:At5g67360 InParanoid:O65351 OMA:TYIVHMA PhylomeDB:O65351 ProtClustDB:CLSN2690100 ArrayExpress:O65351 Genevestigator:O65351 GermOnline:AT5G67360 GO:GO:0048359 Uniprot:O65351
Root	Isotig01703	0	37	-6.338	1.15E-10	TAIR|locus:2158187 - symbol:ARA12 "AT5G67360" species:3702 "Arabidopsis thaliana" [GO:0006508 "proteolysis" evidence=ISS] [GO:0004252 "serine-type endopeptidase activity" evidence=IDA] [GO:0005576 "extracellular region" evidence=IDA] [GO:0005618 "cell wall" evidence=IDA] [GO:0009505 "plant-type cell wall" evidence=IDA] [GO:0010214 "seed coat development" evidence=IMP] [GO:0048359 "mucilage metabolic process involved seed coat development" evidence=IMP] [GO:0080001 "mucilage extrusion from seed coat" evidence=IMP] [GO:0048046 "apoplast" evidence=IDA] InterPro:IPR000209 InterPro:IPR010259 Pfam:PF00082 Pfam:PF05922 InterPro:IPR003137 Prosite:PS00137 Prosite:PS00138 PANTHER:PTHR10795 Pfam:PF02225 GO:GO:0048046 EMBL:CP002688 GenomeReviews:BA000015_GR GO:GO:0042802 GO:GO:0006508 GO:GO:0004252 GO:GO:0009505 GO:GO:0043086 InterPro:IPR023828 InterPro:IPR015500 Gene3D:G3DSA:3.40.50.200 PRINTS:PR00723 SUPFAM:SSF52743 PROSITE:PS00136 eggNOG:COG1404 EMBL:AB007645 GO:GO:0080001 HOGENOM:HBG591621 EMBL:AF065639 EMBL:AF360285 EMBL:AY091773 EMBL:AY142612 EMBL:BT001082 EMBL:X85974 IPI:IPI00548134 PIR:JC7519 PIR:S52770 RefSeq:NP_569048.1 UniGene:At.23238 UniGene:At.67722 UniGene:At.71531 ProteinModelPortal:O65351 SMR:O65351 STRING:O65351 MEROPS:S08.112 PRIDE:O65351 EnsemblPlants:AT5G67360.1 GeneID:836871 KEGG:ath:AT5G67360 GeneFarm:1964 TAIR:At5g67360 InParanoid:O65351 OMA:TYIVHMA PhylomeDB:O65351 ProtClustDB:CLSN2690100 ArrayExpress:O65351 Genevestigator:O65351 GermOnline:AT5G67360 GO:GO:0048359 Uniprot:O65351
Root	Isotig01704	0	33	-6.173	1.05E-09	TAIR|locus:2158187 - symbol:ARA12 "AT5G67360" species:3702 "Arabidopsis thaliana" [GO:0006508 "proteolysis" evidence=ISS] [GO:0004252 "serine-type endopeptidase activity" evidence=IDA] [GO:0005576 "extracellular region" evidence=IDA] [GO:0005618 "cell wall" evidence=IDA] [GO:0009505 "plant-type cell wall" evidence=IDA] [GO:0010214 "seed coat development" evidence=IMP] [GO:0048359 "mucilage metabolic process involved seed

Tissue	Identification	# of reads (stress)	# of reads (control)	Fold Change	p-value	Description
						coat development" evidence=IMP] [GO:0080001 "mucilage extrusion from seed coat" evidence=IMP] [GO:0048046 "apoplast" evidence=IDA] InterPro:IPR000209 InterPro:IPR010259 Pfam:PF00082 Pfam:PF05922 InterPro:IPR003137 Prosite:PS00137 Prosite:PS00138 PANTHER:PTHR10795 Pfam:PF02225 GO:GO:0048046 EMBL:CP002688 GenomeReviews:BA000015_GR GO:GO:0042802 GO:GO:0006508 GO:GO:0004252 GO:GO:0009505 GO:GO:0043086 InterPro:IPR023828 InterPro:IPR015500 Gene3D:G3DSA:3.40.50.200 PRINTS:PR00723 SUPFAM:SSF52743 PROSITE:PS00136 eggNOG:COG1404 EMBL:AB007645 GO:GO:0080001 HOGENOM:HBG591621 EMBL:AF065639 EMBL:AF360285 EMBL:AY091773 EMBL:AY142612 EMBL:BT001082 EMBL:X85974 IPI:IPI00548134 PIR:JC7519 PIR:S52770 RefSeq:NP_569048.1 UniGene:At.23238 UniGene:At.67722 UniGene:At.71531 ProteinModelPortal:O65351 SMR:O65351 STRING:O65351 MEROPS:S08.112 PRIDE:O65351 EnsemblPlants:AT5G67360.1 GeneID:836871 KEGG:ath:AT5G67360 GeneFarm:1964 TAIR:At5g67360 InParanoid:O65351 OMA:TYIVHMA PhylomeDB:O65351 ProtClustDB:CLSN2690100 ArrayExpress:O65351 Genevestigator:O65351 GermOnline:AT5G67360 GO:GO:0048359 Uniprot:O65351
Root	Isotig01705	0	31	-6.083	3.24E-09	TAIR|locus:2158187 - symbol:ARA12 "AT5G67360" species:3702 "Arabidopsis thaliana" [GO:0006508 "proteolysis" evidence=ISS] [GO:0004252 "serine-type endopeptidase activity" evidence=IDA] [GO:0005576 "extracellular region" evidence=IDA] [GO:0005618 "cell wall" evidence=IDA] [GO:0009505 "plant-type cell wall" evidence=IDA] [GO:0010214 "seed coat development" evidence=IMP] [GO:0048359 "mucilage metabolic process involved seed coat development" evidence=IMP] [GO:0080001 "mucilage extrusion from seed coat" evidence=IMP] [GO:0048046 "apoplast" evidence=IDA] InterPro:IPR000209 InterPro:IPR010259 Pfam:PF00082 Pfam:PF05922 InterPro:IPR003137 Prosite:PS00137 Prosite:PS00138 PANTHER:PTHR10795 Pfam:PF02225 GO:GO:0048046 EMBL:CP002688 GenomeReviews:BA000015_GR GO:GO:0042802 GO:GO:0006508 GO:GO:0004252 GO:GO:0009505 GO:GO:0043086 InterPro:IPR023828 InterPro:IPR015500 Gene3D:G3DSA:3.40.50.200 PRINTS:PR00723 SUPFAM:SSF52743 PROSITE:PS00136 eggNOG:COG1404 EMBL:AB007645 GO:GO:0080001 HOGENOM:HBG591621 EMBL:AF065639 EMBL:AF360285 EMBL:AY091773 EMBL:AY142612 EMBL:BT001082 EMBL:X85974 IPI:IPI00548134 PIR:JC7519 PIR:S52770 RefSeq:NP_569048.1 UniGene:At.23238 UniGene:At.67722 UniGene:At.71531 ProteinModelPortal:O65351 SMR:O65351 STRING:O65351 MEROPS:S08.112 PRIDE:O65351 EnsemblPlants:AT5G67360.1 GeneID:836871 KEGG:ath:AT5G67360 GeneFarm:1964 TAIR:At5g67360 InParanoid:O65351 OMA:TYIVHMA PhylomeDB:O65351 ProtClustDB:CLSN2690100 ArrayExpress:O65351 Genevestigator:O65351 GermOnline:AT5G67360 GO:GO:0048359 Uniprot:O65351
Root	Isotig01706	0	33	-6.173	1.05E-09	TAIR|locus:2158187 - symbol:ARA12 "AT5G67360" species:3702 "Arabidopsis thaliana" [GO:0006508 "proteolysis" evidence=ISS] [GO:0004252 "serine-type endopeptidase activity" evidence=IDA] [GO:0005576 "extracellular region" evidence=IDA] [GO:0005618 "cell wall" evidence=IDA] [GO:0009505 "plant-type cell wall" evidence=IDA] [GO:0010214 "seed coat development" evidence=IMP] [GO:0048359 "mucilage metabolic process involved seed coat development" evidence=IMP] [GO:0080001 "mucilage extrusion from seed coat" evidence=IMP] [GO:0048046 "apoplast" evidence=IDA] InterPro:IPR000209 InterPro:IPR010259 Pfam:PF00082 Pfam:PF05922 InterPro:IPR003137 Prosite:PS00137 Prosite:PS00138 PANTHER:PTHR10795 Pfam:PF02225 GO:GO:0048046 EMBL:CP002688 GenomeReviews:BA000015_GR GO:GO:0042802 GO:GO:0006508 GO:GO:0004252 GO:GO:0009505 GO:GO:0043086 InterPro:IPR023828 InterPro:IPR015500 Gene3D:G3DSA:3.40.50.200 PRINTS:PR00723 SUPFAM:SSF52743 PROSITE:PS00136 eggNOG:COG1404 EMBL:AB007645 GO:GO:0080001 HOGENOM:HBG591621 EMBL:AF065639 EMBL:AF360285 EMBL:AY091773 EMBL:AY142612 EMBL:BT001082 EMBL:X85974 IPI:IPI00548134 PIR:JC7519 PIR:S52770 RefSeq:NP_569048.1 UniGene:At.23238 UniGene:At.67722 UniGene:At.71531 ProteinModelPortal:O65351 SMR:O65351 STRING:O65351 MEROPS:S08.112 PRIDE:O65351 EnsemblPlants:AT5G67360.1 GeneID:836871 KEGG:ath:AT5G67360 GeneFarm:1964 TAIR:At5g67360 InParanoid:O65351 OMA:TYIVHMA PhylomeDB:O65351 ProtClustDB:CLSN2690100 ArrayExpress:O65351 Genevestigator:O65351 GermOnline:AT5G67360 GO:GO:0048359 Uniprot:O65351
Root	Isotig01707	0	15	-5.036	4.38E-05	TAIR|locus:2131566 - symbol:SLP2 "AT4G34980" species:3702 "Arabidopsis thaliana" [GO:0004252 "serine-type endopeptidase activity" evidence=IBA] [GO:0005618 "cell wall" evidence=IBA] [GO:0006508 "proteolysis" evidence=ISS] [GO:0008152 "metabolic process" evidence=IBA] [GO:0008236 "serine-type peptidase activity" evidence=ISS] [GO:0009827 "plant-type cell wall modification" evidence=TAS] [GO:0048196 "plant extracellular matrix" evidence=ISS] [GO:0016020 "membrane" evidence=IDA] InterPro:IPR000209 InterPro:IPR010259

Tissue	Identification	# of reads (stress)	# of reads (control)	Fold Change	p-value	Description
						Pfam:PF00082 Pfam:PF05922 InterPro:IPR003137 Prosite:PS00138 PANTHER:PTHR10795 Pfam:PF02225 EMBL:CP002687 GenomeReviews:CT486007_GR GO:GO:0005576 GO:GO:0042802 GO:GO:0016020 GO:GO:0006508 GO:GO:0004252 EMBL:AL022023 EMBL:AL161586 GO:GO:0043086 InterPro:IPR023828 InterPro:IPR015500 InterPro:IPR009020 Gene3D:G3DSA:3.40.50.200 PRINTS:PR00723 SUPFAM:SSF52743 SUPFAM:SSF54897 HSSP:Q99405 eggNOG:COG1404 GO:GO:0009827 HOGENOM:HBG591621 EMBL:AY074375 EMBL:AY096357 IPI:IPI00522734 PIR:T05768 RefSeq:NP_567972.1 UniGene:At.27496 UniGene:At.28167 UniGene:At.69469 ProteinModelPortal:O49607 SMR:O49607 STRING:O49607 MEROPS:S08.A39 PRIDE:O49607 EnsemblPlants:AT4G34980.1 GeneID:829650 KEGG:ath:AT4G34980 TAIR:At4g34980 InParanoid:O49607 OMA:IFPTHYH PhylomeDB:O49607 ProtClustDB:CLSN2689763 Genevestigator:O49607 Uniprot:O49607
Root	Isotig01708	0	17	-5.216	1.26E-05	TAIR|locus:2131566 - symbol:SLP2 "AT4G34980" species:3702 "Arabidopsis thaliana" [GO:0004252 "serine-type endopeptidase activity" evidence=IBA] [GO:0005618 "cell wall" evidence=IBA] [GO:0006508 "proteolysis" evidence=ISS] [GO:0008152 "metabolic process" evidence=IBA] [GO:0008236 "serine-type peptidase activity" evidence=ISS] [GO:0009827 "plant-type cell wall modification" evidence=TAS] [GO:0048196 "plant extracellular matrix" evidence=ISS] [GO:0016020 "membrane" evidence=IDA] InterPro:IPR000209 InterPro:IPR010259 Pfam:PF00082 Pfam:PF05922 InterPro:IPR003137 Prosite:PS00138 PANTHER:PTHR10795 Pfam:PF02225 EMBL:CP002687 GenomeReviews:CT486007_GR GO:GO:0005576 GO:GO:0042802 GO:GO:0016020 GO:GO:0006508 GO:GO:0004252 EMBL:AL022023 EMBL:AL161586 GO:GO:0043086 InterPro:IPR023828 InterPro:IPR015500 InterPro:IPR009020 Gene3D:G3DSA:3.40.50.200 PRINTS:PR00723 SUPFAM:SSF52743 SUPFAM:SSF54897 HSSP:Q99405 eggNOG:COG1404 GO:GO:0009827 HOGENOM:HBG591621 EMBL:AY074375 EMBL:AY096357 IPI:IPI00522734 PIR:T05768 RefSeq:NP_567972.1 UniGene:At.27496 UniGene:At.28167 UniGene:At.69469 ProteinModelPortal:O49607 SMR:O49607 STRING:O49607 MEROPS:S08.A39 PRIDE:O49607 EnsemblPlants:AT4G34980.1 GeneID:829650 KEGG:ath:AT4G34980 TAIR:At4g34980 InParanoid:O49607 OMA:IFPTHYH PhylomeDB:O49607 ProtClustDB:CLSN2689763 Genevestigator:O49607 Uniprot:O49607
Root	Isotig01770	0	13	-4.829	0.000155955	TAIR|locus:2180315 - symbol:EMB2775 "EMBRYO DEFECTIVE 2775" species:3702 "Arabidopsis thaliana" [GO:0000166 "nucleotide binding" evidence=IEA] [GO:0003677 "DNA binding" evidence=IEA] [GO:0005524 "ATP binding" evidence=IEA] [GO:0005634 "nucleus" evidence=IEA] [GO:0009793 "embryo development ending in seed dormancy" evidence=IMP] [GO:0017111 "nucleoside-triphosphatase activity" evidence=IEA] InterPro:IPR003593 InterPro:IPR008921 SMART:SM00382 InterPro:IPR013748 GO:GO:0005524 EMBL:CP002688 GenomeReviews:BA000015_GR GO:GO:0006260 eggNOG:COG0470 GO:GO:0003689 SUPFAM:SSF48019 GO:GO:0005663 Pfam:PF08542 KO:K10756 OMA:TRIMCLL HOGENOM:HBG330815 HSSP:Q8U4J3 EMBL:AY074381 EMBL:AY091236 IPI:IPI00528406 RefSeq:NP_198126.1 UniGene:At.19785 ProteinModelPortal:Q8VXX4 SMR:Q8VXX4 STRING:Q8VXX4 PRIDE:Q8VXX4 EnsemblPlants:AT5G27740.1 GeneID:832836 KEGG:ath:AT5G27740 TAIR:At5g27740 InParanoid:Q8VXX4 PhylomeDB:Q8VXX4 ProtClustDB:CLSN2687133 ArrayExpress:Q8VXX4 Genevestigator:Q8VXX4 Uniprot:Q8VXX4
Root	Isotig01771	3	44	-4.003	2.84E-11	UNIPROTKB|Q6VMW0 - symbol:OMT2 "8-hydroxyquercetin 8-O-methyltransferase" species:34256 "Mentha x piperita" [GO:0009812 "flavonoid metabolic process" evidence=IDA] [GO:0030761 "8-hydroxyquercitin 8-O-methyltransferase activity" evidence=IDA] [GO:0032259 "methylation" evidence=IDA] InterPro:IPR001077 InterPro:IPR012967 InterPro:IPR016461 Pfam:PF00891 Pfam:PF08100 PIRSF:PIRSF005739 InterPro:IPR011991 Gene3D:G3DSA:1.10.10.10 GO:GO:0046983 GO:GO:0008171 GO:GO:0009812 EMBL:AY337459 ProteinModelPortal:Q6VMW0 BRENDA:2.1.1.88 GO:GO:0030761 Uniprot:Q6VMW0
Root	Isotig01772	1	34	-5.216	6.54E-10	UNIPROTKB|Q6VMW0 - symbol:OMT2 "8-hydroxyquercetin 8-O-methyltransferase" species:34256 "Mentha x piperita" [GO:0009812 "flavonoid metabolic process" evidence=IDA] [GO:0030761 "8-hydroxyquercitin 8-O-methyltransferase activity" evidence=IDA] [GO:0032259 "methylation" evidence=IDA] InterPro:IPR001077 InterPro:IPR012967 InterPro:IPR016461 Pfam:PF00891 Pfam:PF08100 PIRSF:PIRSF005739 InterPro:IPR011991 Gene3D:G3DSA:1.10.10.10 GO:GO:0046983 GO:GO:0008171 GO:GO:0009812 EMBL:AY337459 ProteinModelPortal:Q6VMW0 BRENDA:2.1.1.88 GO:GO:0030761 Uniprot:Q6VMW0
Root	Isotig01773	3	41	-3.902	1.87E-10	UNIPROTKB|Q6VMW0 - symbol:OMT2 "8-hydroxyquercetin 8-O-methyltransferase" species:34256 "Mentha x piperita" [GO:0009812 "flavonoid metabolic process" evidence=IDA] [GO:0030761 "8-hydroxyquercitin 8-O-methyltransferase activity" evidence=IDA] [GO:0032259 "methylation" evidence=IDA] InterPro:IPR001077 InterPro:IPR012967 InterPro:IPR016461 Pfam:PF00891 Pfam:PF08100 PIRSF:PIRSF005739 InterPro:IPR011991

Tissue	Identification	# of reads (stress)	# of reads (control)	Fold Change	p-value	Description
						Gene3D:G3DSA:1.10.10.10 GO:GO:0046983 GO:GO:0008171 GO:GO:0009812 EMBL:AY337459 ProteinModelPortal:Q6VMW0 BRENDA:2.1.1.88 GO:GO:0030761 Uniprot:Q6VMW0
Root	Isotig01774	3	44	-4.003	2.84E-11	UNIPROTKB|Q6VMW0 - symbol:OMT2 "8-hydroxyquercetin 8-O-methyltransferase" species:34256 "Mentha x piperita" [GO:0009812 "flavonoid metabolic process" evidence=IDA] [GO:0030761 "8-hydroxyquercitin 8-O-methyltransferase activity" evidence=IDA] [GO:0032259 "methylation" evidence=IDA] InterPro:IPR001077 InterPro:IPR012967 InterPro:IPR016461 Pfam:PF00891 Pfam:PF08100 PIRSF:PIRSF005739 InterPro:IPR011991 Gene3D:G3DSA:1.10.10.10 GO:GO:0046983 GO:GO:0008171 GO:GO:0009812 EMBL:AY337459 ProteinModelPortal:Q6VMW0 BRENDA:2.1.1.88 GO:GO:0030761 Uniprot:Q6VMW0
Root	Isotig01775	2	28	-3.936	1.29E-07	UNIPROTKB|Q6VMW0 - symbol:OMT2 "8-hydroxyquercetin 8-O-methyltransferase" species:34256 "Mentha x piperita" [GO:0009812 "flavonoid metabolic process" evidence=IDA] [GO:0030761 "8-hydroxyquercitin 8-O-methyltransferase activity" evidence=IDA] [GO:0032259 "methylation" evidence=IDA] InterPro:IPR001077 InterPro:IPR012967 InterPro:IPR016461 Pfam:PF00891 Pfam:PF08100 PIRSF:PIRSF005739 InterPro:IPR011991 Gene3D:G3DSA:1.10.10.10 GO:GO:0046983 GO:GO:0008171 GO:GO:0009812 EMBL:AY337459 ProteinModelPortal:Q6VMW0 BRENDA:2.1.1.88 GO:GO:0030761 Uniprot:Q6VMW0
Root	Isotig01776	1	31	-5.083	4.06E-09	UNIPROTKB|Q6VMW0 - symbol:OMT2 "8-hydroxyquercetin 8-O-methyltransferase" species:34256 "Mentha x piperita" [GO:0009812 "flavonoid metabolic process" evidence=IDA] [GO:0030761 "8-hydroxyquercitin 8-O-methyltransferase activity" evidence=IDA] [GO:0032259 "methylation" evidence=IDA] InterPro:IPR001077 InterPro:IPR012967 InterPro:IPR016461 Pfam:PF00891 Pfam:PF08100 PIRSF:PIRSF005739 InterPro:IPR011991 Gene3D:G3DSA:1.10.10.10 GO:GO:0046983 GO:GO:0008171 GO:GO:0009812 EMBL:AY337459 ProteinModelPortal:Q6VMW0 BRENDA:2.1.1.88 GO:GO:0030761 Uniprot:Q6VMW0
Root	Isotig01777	1	34	-5.216	6.54E-10	UNIPROTKB|Q6VMW0 - symbol:OMT2 "8-hydroxyquercetin 8-O-methyltransferase" species:34256 "Mentha x piperita" [GO:0009812 "flavonoid metabolic process" evidence=IDA] [GO:0030761 "8-hydroxyquercitin 8-O-methyltransferase activity" evidence=IDA] [GO:0032259 "methylation" evidence=IDA] InterPro:IPR001077 InterPro:IPR012967 InterPro:IPR016461 Pfam:PF00891 Pfam:PF08100 PIRSF:PIRSF005739 InterPro:IPR011991 Gene3D:G3DSA:1.10.10.10 GO:GO:0046983 GO:GO:0008171 GO:GO:0009812 EMBL:AY337459 ProteinModelPortal:Q6VMW0 BRENDA:2.1.1.88 GO:GO:0030761 Uniprot:Q6VMW0
Root	Isotig01778	18	44	-1.418	0.000224092	TAIR|locus:2185168 - symbol:SIS "AT5G02020" species:3702 "Arabidopsis thaliana" [GO:0003674 "molecular_function" evidence=ND] [GO:0009651 "response to salt stress" evidence=IMP] EMBL:CP002688 GO:GO:0009651 EMBL:AL162508 EMBL:AY058150 EMBL:AY094053 IPI:IPI00528966 PIR:T48223 RefSeq:NP_195822.1 UniGene:At.23336 UniGene:At.70618 EnsemblPlants:AT5G02020.1 GeneID:831782 KEGG:ath:AT5G02020 TAIR:At5g02020 eggNOG:NOG285266 InParanoid:Q9LZM9 OMA:GSKESSY PhylomeDB:Q9LZM9 ProtClustDB:CLSN2687303 Genevestigator:Q9LZM9 Uniprot:Q9LZM9
Root	Isotig01779	5	44	-3.266	6.87E-10	TAIR|locus:2185168 - symbol:SIS "AT5G02020" species:3702 "Arabidopsis thaliana" [GO:0003674 "molecular_function" evidence=ND] [GO:0009651 "response to salt stress" evidence=IMP] EMBL:CP002688 GO:GO:0009651 EMBL:AL162508 EMBL:AY058150 EMBL:AY094053 IPI:IPI00528966 PIR:T48223 RefSeq:NP_195822.1 UniGene:At.23336 UniGene:At.70618 EnsemblPlants:AT5G02020.1 GeneID:831782 KEGG:ath:AT5G02020 TAIR:At5g02020 eggNOG:NOG285266 InParanoid:Q9LZM9 OMA:GSKESSY PhylomeDB:Q9LZM9 ProtClustDB:CLSN2687303 Genevestigator:Q9LZM9 Uniprot:Q9LZM9
Root	Isotig01780	18	44	-1.418	0.000224092	TAIR|locus:2185168 - symbol:SIS "AT5G02020" species:3702 "Arabidopsis thaliana" [GO:0003674 "molecular_function" evidence=ND] [GO:0009651 "response to salt stress" evidence=IMP] EMBL:CP002688 GO:GO:0009651 EMBL:AL162508 EMBL:AY058150 EMBL:AY094053 IPI:IPI00528966 PIR:T48223 RefSeq:NP_195822.1 UniGene:At.23336 UniGene:At.70618 EnsemblPlants:AT5G02020.1 GeneID:831782 KEGG:ath:AT5G02020 TAIR:At5g02020 eggNOG:NOG285266 InParanoid:Q9LZM9 OMA:GSKESSY PhylomeDB:Q9LZM9 ProtClustDB:CLSN2687303 Genevestigator:Q9LZM9 Uniprot:Q9LZM9
Root	Isotig01781	5	44	-3.266	6.87E-10	TAIR|locus:2185168 - symbol:SIS "AT5G02020" species:3702 "Arabidopsis thaliana" [GO:0003674 "molecular_function" evidence=ND] [GO:0009651 "response to salt stress" evidence=IMP] EMBL:CP002688 GO:GO:0009651 EMBL:AL162508 EMBL:AY058150 EMBL:AY094053 IPI:IPI00528966 PIR:T48223 RefSeq:NP_195822.1 UniGene:At.23336 UniGene:At.70618 EnsemblPlants:AT5G02020.1 GeneID:831782 KEGG:ath:AT5G02020 TAIR:At5g02020 eggNOG:NOG285266 InParanoid:Q9LZM9 OMA:GSKESSY PhylomeDB:Q9LZM9 ProtClustDB:CLSN2687303 Genevestigator:Q9LZM9 Uniprot:Q9LZM9
Root	Isotig01783	5	35	-2.936	1.63E-07	TAIR|locus:504954988 - symbol:AT5G05965 "AT5G05965" species:3702 "Arabidopsis thaliana" [GO:0003674 "molecular_function" evidence=ND] [GO:0008150 "biological_process" evidence=ND] EMBL:CP002688

Tissue	Identification	# of reads (stress)	# of reads (control)	Fold Change	p-value	Description
						GenomeReviews:BA000015_GR EMBL:AY735673 IPI:IPI00516315 RefSeq:NP_680147.1 UniGene:At.43072 UniGene:At.66777 EnsemblPlants:AT5G05965.1 GeneID:830482 KEGG:ath:AT5G05965 TAIR:At5g05965 eggNOG:NOG286329 HOGENOM:HBG317139 OMA:DERSSHE PhylomeDB:Q5XV49 ProtClustDB:CLSN2917816 Genevestigator:Q5XV49 Uniprot:Q5XV49
Root	Isotig01800	39	69	-0.952	0.000771235	TAIR|locus:2135887 - symbol:SHD "SHEPHERD" species:3702 "Arabidopsis thaliana" [GO:0005524 "ATP binding" evidence=IEA;ISS] [GO:0006457 "protein folding" evidence=ISS] [GO:0051082 "unfolded protein binding" evidence=IEA] [GO:0005783 "endoplasmic reticulum" evidence=ISS;IDA] [GO:0009934 "regulation of meristem structural organization" evidence=IMP] [GO:0010075 "regulation of meristem growth" evidence=IMP] [GO:0005634 "nucleus" evidence=IDA] [GO:0009409 "response to cold" evidence=IEP] [GO:0005739 "mitochondrion" evidence=IDA] [GO:0009507 "chloroplast" evidence=IDA] [GO:0005773 "vacuole" evidence=IDA] [GO:0046686 "response to cadmium ion" evidence=IEP] [GO:0009306 "protein secretion" evidence=IDA] [GO:0005774 "vacuolar membrane" evidence=IDA] [GO:0016020 "membrane" evidence=IDA] [GO:0005886 "plasma membrane" evidence=IDA] [GO:0009414 "response to water deprivation" evidence=IMP] [GO:0009651 "response to salt stress" evidence=IMP] [GO:0005829 "cytosol" evidence=RCA] [GO:0009506 "plasmodesma" evidence=IDA] [GO:0005794 "Golgi apparatus" evidence=RCA] InterPro:IPR001404 InterPro:IPR003594 InterPro:IPR019805 Pfam:PF00183 PIRSF:PIRSF002583 PROSITE:PS00298 SMART:SM00387 GO:GO:0005739 GO:GO:0005886 GO:GO:0009506 GO:GO:0005524 EMBL:CP002687 GenomeReviews:CT486007_GR GO:GO:0005634 GO:GO:0046686 GO:GO:0005774 GO:GO:0009507 GO:GO:0006457 GO:GO:0051082 GO:GO:0009306 GO:GO:0009409 GO:GO:0009651 GO:GO:0009414 GO:GO:0005788 PROSITE:PS00014 EMBL:AL078637 EMBL:AL161561 Gene3D:G3DSA:3.30.565.10 SUPFAM:SSF55874 InterPro:IPR020568 SUPFAM:SSF54211 EMBL:AC002343 GO:GO:0010075 GO:GO:0009934 eggNOG:COG0326 HOGENOM:HBG631012 PANTHER:PTHR11528 EMBL:AB064528 EMBL:AY039895 EMBL:AY072394 EMBL:BT004527 EMBL:AK221485 IPI:IPI00524027 PIR:T09882 RefSeq:NP_194150.1 UniGene:At.24111 ProteinModelPortal:Q9STX5 SMR:Q9STX5 STRING:Q9STX5 PRIDE:Q9STX5 EnsemblPlants:AT4G24190.1 GeneID:828520 KEGG:ath:AT4G24190 TAIR:At4g24190 InParanoid:Q9STX5 PhylomeDB:Q9STX5 ProtClustDB:CLSN2716298 Genevestigator:Q9STX5 InterPro:IPR020575 PRINTS:PR00775 Uniprot:Q9STX5
Root	Isotig01801	40	70	-0.936	0.000837113	TAIR|locus:2135887 - symbol:SHD "SHEPHERD" species:3702 "Arabidopsis thaliana" [GO:0005524 "ATP binding" evidence=IEA;ISS] [GO:0006457 "protein folding" evidence=ISS] [GO:0051082 "unfolded protein binding" evidence=IEA] [GO:0005783 "endoplasmic reticulum" evidence=ISS;IDA] [GO:0009934 "regulation of meristem structural organization" evidence=IMP] [GO:0010075 "regulation of meristem growth" evidence=IMP] [GO:0005634 "nucleus" evidence=IDA] [GO:0009409 "response to cold" evidence=IEP] [GO:0005739 "mitochondrion" evidence=IDA] [GO:0009507 "chloroplast" evidence=IDA] [GO:0005773 "vacuole" evidence=IDA] [GO:0046686 "response to cadmium ion" evidence=IEP] [GO:0009306 "protein secretion" evidence=IDA] [GO:0005774 "vacuolar membrane" evidence=IDA] [GO:0016020 "membrane" evidence=IDA] [GO:0005886 "plasma membrane" evidence=IDA] [GO:0009414 "response to water deprivation" evidence=IMP] [GO:0009651 "response to salt stress" evidence=IMP] [GO:0005829 "cytosol" evidence=RCA] [GO:0009506 "plasmodesma" evidence=IDA] [GO:0005794 "Golgi apparatus" evidence=RCA] InterPro:IPR001404 InterPro:IPR003594 InterPro:IPR019805 Pfam:PF00183 PIRSF:PIRSF002583 PROSITE:PS00298 SMART:SM00387 GO:GO:0005739 GO:GO:0005886 GO:GO:0009506 GO:GO:0005524 EMBL:CP002687 GenomeReviews:CT486007_GR GO:GO:0005634 GO:GO:0046686 GO:GO:0005774 GO:GO:0009507 GO:GO:0006457 GO:GO:0051082 GO:GO:0009306 GO:GO:0009409 GO:GO:0009651 GO:GO:0009414 GO:GO:0005788 PROSITE:PS00014 EMBL:AL078637 EMBL:AL161561 Gene3D:G3DSA:3.30.565.10 SUPFAM:SSF55874 InterPro:IPR020568 SUPFAM:SSF54211 EMBL:AC002343 GO:GO:0010075 GO:GO:0009934 eggNOG:COG0326 HOGENOM:HBG631012 PANTHER:PTHR11528 EMBL:AB064528 EMBL:AY039895 EMBL:AY072394 EMBL:BT004527 EMBL:AK221485 IPI:IPI00524027 PIR:T09882 RefSeq:NP_194150.1 UniGene:At.24111 ProteinModelPortal:Q9STX5 SMR:Q9STX5 STRING:Q9STX5 PRIDE:Q9STX5 EnsemblPlants:AT4G24190.1 GeneID:828520 KEGG:ath:AT4G24190 TAIR:At4g24190 InParanoid:Q9STX5 PhylomeDB:Q9STX5 ProtClustDB:CLSN2716298 Genevestigator:Q9STX5 InterPro:IPR020575 PRINTS:PR00775 Uniprot:Q9STX5
Root	Isotig01822	4	26	-2.829	9.39E-06	TAIR|locus:2180617 - symbol:AT5G25930 species:3702 "Arabidopsis thaliana" [GO:0006468 "protein phosphorylation" evidence=IEA] [GO:0016301 "kinase activity" evidence=ISS] [GO:0005886 "plasma membrane" evidence=IDA] [GO:0016020 "membrane" evidence=IDA] Pfam:PF00560 InterPro:IPR001611 InterPro:IPR000719 InterPro:IPR008271 InterPro:IPR011009 InterPro:IPR017441 Pfam:PF00069 PROSITE:PS00107

Tissue	Identification	# of reads (stress)	# of reads (control)	Fold Change	p-value	Description
						PROSITE:PS00108 PROSITE:PS50011 GO:GO:0016021 GO:GO:0005886 GO:GO:0005524 EMBL:CP002688 GO:GO:0004872 SUPFAM:SSF56112 GO:GO:0004674 HOGENOM:HBG755340 InterPro:IPR013320 Gene3D:G3DSA:2.60.120.200 KO:K00924 EMBL:AF149413 InterPro:IPR013210 Pfam:PF08263 EMBL:BT004058 EMBL:FJ708783 IPI:IPI00529952 RefSeq:NP_197965.1 UniGene:At.30881 UniGene:At.66768 ProteinModelPortal:Q9XGZ2 SMR:Q9XGZ2 STRING:Q9XGZ2 PRIDE:Q9XGZ2 EnsemblPlants:AT5G25930.1 GeneID:832662 KEGG:ath:AT5G25930 TAIR:At5g25930 InParanoid:Q9XGZ2 OMA:NIREENV PhylomeDB:Q9XGZ2 ProtClustDB:CLSN2916431 ArrayExpress:Q9XGZ2 Genevestigator:Q9XGZ2 Uniprot:Q9XGZ2
Root	Isotig01823	5	26	-2.507	3.30E-05	TAIR|locus:2180617 - symbol:AT5G25930 species:3702 "Arabidopsis thaliana" [GO:0006468 "protein phosphorylation" evidence=IEA] [GO:0016301 "kinase activity" evidence=ISS] [GO:0005886 "plasma membrane" evidence=IDA] [GO:0016020 "membrane" evidence=IDA] Pfam:PF00560 InterPro:IPR001611 InterPro:IPR000719 InterPro:IPR008271 InterPro:IPR011009 InterPro:IPR017441 Pfam:PF00069 PROSITE:PS00107 PROSITE:PS00108 PROSITE:PS50011 GO:GO:0016021 GO:GO:0005886 GO:GO:0005524 EMBL:CP002688 GO:GO:0004872 SUPFAM:SSF56112 GO:GO:0004674 HOGENOM:HBG755340 InterPro:IPR013320 Gene3D:G3DSA:2.60.120.200 KO:K00924 EMBL:AF149413 InterPro:IPR013210 Pfam:PF08263 EMBL:BT004058 EMBL:FJ708783 IPI:IPI00529952 RefSeq:NP_197965.1 UniGene:At.30881 UniGene:At.66768 ProteinModelPortal:Q9XGZ2 SMR:Q9XGZ2 STRING:Q9XGZ2 PRIDE:Q9XGZ2 EnsemblPlants:AT5G25930.1 GeneID:832662 KEGG:ath:AT5G25930 TAIR:At5g25930 InParanoid:Q9XGZ2 OMA:NIREENV PhylomeDB:Q9XGZ2 ProtClustDB:CLSN2916431 ArrayExpress:Q9XGZ2 Genevestigator:Q9XGZ2 Uniprot:Q9XGZ2
Root	Isotig01824	3	25	-3.188	4.22E-06	TAIR|locus:2180617 - symbol:AT5G25930 species:3702 "Arabidopsis thaliana" [GO:0006468 "protein phosphorylation" evidence=IEA] [GO:0016301 "kinase activity" evidence=ISS] [GO:0005886 "plasma membrane" evidence=IDA] [GO:0016020 "membrane" evidence=IDA] Pfam:PF00560 InterPro:IPR001611 InterPro:IPR000719 InterPro:IPR008271 InterPro:IPR011009 InterPro:IPR017441 Pfam:PF00069 PROSITE:PS00107 PROSITE:PS00108 PROSITE:PS50011 GO:GO:0016021 GO:GO:0005886 GO:GO:0005524 EMBL:CP002688 GO:GO:0004872 SUPFAM:SSF56112 GO:GO:0004674 HOGENOM:HBG755340 InterPro:IPR013320 Gene3D:G3DSA:2.60.120.200 KO:K00924 EMBL:AF149413 InterPro:IPR013210 Pfam:PF08263 EMBL:BT004058 EMBL:FJ708783 IPI:IPI00529952 RefSeq:NP_197965.1 UniGene:At.30881 UniGene:At.66768 ProteinModelPortal:Q9XGZ2 SMR:Q9XGZ2 STRING:Q9XGZ2 PRIDE:Q9XGZ2 EnsemblPlants:AT5G25930.1 GeneID:832662 KEGG:ath:AT5G25930 TAIR:At5g25930 InParanoid:Q9XGZ2 OMA:NIREENV PhylomeDB:Q9XGZ2 ProtClustDB:CLSN2916431 ArrayExpress:Q9XGZ2 Genevestigator:Q9XGZ2 Uniprot:Q9XGZ2
Root	Isotig01825	4	25	-2.773	1.71E-05	TAIR|locus:2180617 - symbol:AT5G25930 species:3702 "Arabidopsis thaliana" [GO:0006468 "protein phosphorylation" evidence=IEA] [GO:0016301 "kinase activity" evidence=ISS] [GO:0005886 "plasma membrane" evidence=IDA] [GO:0016020 "membrane" evidence=IDA] Pfam:PF00560 InterPro:IPR001611 InterPro:IPR000719 InterPro:IPR008271 InterPro:IPR011009 InterPro:IPR017441 Pfam:PF00069 PROSITE:PS00107 PROSITE:PS00108 PROSITE:PS50011 GO:GO:0016021 GO:GO:0005886 GO:GO:0005524 EMBL:CP002688 GO:GO:0004872 SUPFAM:SSF56112 GO:GO:0004674 HOGENOM:HBG755340 InterPro:IPR013320 Gene3D:G3DSA:2.60.120.200 KO:K00924 EMBL:AF149413 InterPro:IPR013210 Pfam:PF08263 EMBL:BT004058 EMBL:FJ708783 IPI:IPI00529952 RefSeq:NP_197965.1 UniGene:At.30881 UniGene:At.66768 ProteinModelPortal:Q9XGZ2 SMR:Q9XGZ2 STRING:Q9XGZ2 PRIDE:Q9XGZ2 EnsemblPlants:AT5G25930.1 GeneID:832662 KEGG:ath:AT5G25930 TAIR:At5g25930 InParanoid:Q9XGZ2 OMA:NIREENV PhylomeDB:Q9XGZ2 ProtClustDB:CLSN2916431 ArrayExpress:Q9XGZ2 Genevestigator:Q9XGZ2 Uniprot:Q9XGZ2
Root	Isotig01826	4	25	-2.773	1.71E-05	TAIR|locus:2180617 - symbol:AT5G25930 species:3702 "Arabidopsis thaliana" [GO:0006468 "protein phosphorylation" evidence=IEA] [GO:0016301 "kinase activity" evidence=ISS] [GO:0005886 "plasma membrane" evidence=IDA] [GO:0016020 "membrane" evidence=IDA] Pfam:PF00560 InterPro:IPR001611 InterPro:IPR000719 InterPro:IPR008271 InterPro:IPR011009 InterPro:IPR017441 Pfam:PF00069 PROSITE:PS00107 PROSITE:PS00108 PROSITE:PS50011 GO:GO:0016021 GO:GO:0005886 GO:GO:0005524 EMBL:CP002688 GO:GO:0004872 SUPFAM:SSF56112 GO:GO:0004674 HOGENOM:HBG755340 InterPro:IPR013320 Gene3D:G3DSA:2.60.120.200 KO:K00924 EMBL:AF149413 InterPro:IPR013210 Pfam:PF08263 EMBL:BT004058 EMBL:FJ708783 IPI:IPI00529952 RefSeq:NP_197965.1 UniGene:At.30881 UniGene:At.66768

Tissue	Identification	# of reads (stress)	# of reads (control)	Fold Change	p-value	Description
						ProteinModelPortal:Q9XGZ2 SMR:Q9XGZ2 STRING:Q9XGZ2 PRIDE:Q9XGZ2 EnsemblPlants:AT5G25930.1 GeneID:832662 KEGG:ath:AT5G25930 TAIR:At5g25930 InParanoid:Q9XGZ2 OMA:NIREENV PhylomeDB:Q9XGZ2 ProtClustDB:CLSN2916431 ArrayExpress:Q9XGZ2 Genevestigator:Q9XGZ2 Uniprot:Q9XGZ2
Root	Isotig01827	5	25	-2.451	5.85E-05	TAIR|locus:2180617 - symbol:AT5G25930 species:3702 "Arabidopsis thaliana" [GO:0006468 "protein phosphorylation" evidence=IEA] [GO:0016301 "kinase activity" evidence=ISS] [GO:0005886 "plasma membrane" evidence=IDA] [GO:0016020 "membrane" evidence=IDA] Pfam:PF00560 InterPro:IPR001611 InterPro:IPR000719 InterPro:IPR008271 InterPro:IPR011009 InterPro:IPR017441 Pfam:PF00069 PROSITE:PS00107 PROSITE:PS00108 PROSITE:PS50011 GO:GO:0016021 GO:GO:0005886 GO:GO:0005524 EMBL:CP002688 GO:GO:0004872 SUPFAM:SSF56112 GO:GO:0004674 HOGENOM:HBG755340 InterPro:IPR013320 Gene3D:G3DSA:2.60.120.200 KO:K00924 EMBL:AF149413 InterPro:IPR013210 Pfam:PF08263 EMBL:BT004058 EMBL:FJ708783 IPI:IPI00529952 RefSeq:NP_197965.1 UniGene:At.30881 UniGene:At.66768 ProteinModelPortal:Q9XGZ2 SMR:Q9XGZ2 STRING:Q9XGZ2 PRIDE:Q9XGZ2 EnsemblPlants:AT5G25930.1 GeneID:832662 KEGG:ath:AT5G25930 TAIR:At5g25930 InParanoid:Q9XGZ2 OMA:NIREENV PhylomeDB:Q9XGZ2 ProtClustDB:CLSN2916431 ArrayExpress:Q9XGZ2 Genevestigator:Q9XGZ2 Uniprot:Q9XGZ2
Root	Isotig01846	34	5	2.637	3.03E-06	TAIR|locus:2169985 - symbol:NFS1 "AT5G65720" species:3702 "Arabidopsis thaliana" [GO:0008483 "transaminase activity" evidence=ISS] [GO:0005739 "mitochondrion" evidence=IDA] [GO:0031071 "cysteine desulfurase activity" evidence=IDA] [GO:0005524 "ATP binding" evidence=IDA] [GO:0016226 "iron-sulfur cluster assembly" evidence=IDA] [GO:0008270 "zinc ion binding" evidence=IDA] InterPro:IPR000192 InterPro:IPR010240 InterPro:IPR015421 InterPro:IPR015422 Pfam:PF00266 GO:GO:0005739 GO:GO:0005524 EMBL:CP002688 GenomeReviews:BA000015_GR GO:GO:0030170 InterPro:IPR015424 Gene3D:G3DSA:3.40.640.10 Gene3D:G3DSA:3.90.1150.10 SUPFAM:SSF53383 EMBL:AB010075 EMBL:AL021684 GO:GO:0008270 GO:GO:0016226 HOGENOM:HBG635316 GO:GO:0031071 GO:GO:0006534 eggNOG:COG1104 KO:K04487 OMA:CIHHVKR InterPro:IPR016454 PIRSF:PIRSF005572 TIGRFAMs:TIGR02006 EMBL:AJ243393 EMBL:AF229854 EMBL:AY050874 EMBL:AY096358 IPI:IPI00545655 PIR:T05896 RefSeq:NP_201373.1 UniGene:At.696 ProteinModelPortal:O49543 SMR:O49543 STRING:O49543 PRIDE:O49543 EnsemblPlants:AT5G65720.1 GeneID:836701 KEGG:ath:AT5G65720 TAIR:At5g65720 InParanoid:O49543 PhylomeDB:O49543 ProtClustDB:PLN02651 BRENDA:2.8.1.6 ArrayExpress:O49543 Genevestigator:O49543 GermOnline:AT5G65720 Uniprot:O49543
Root	Isotig01847	36	5	2.719	1.04E-06	TAIR|locus:2169985 - symbol:NFS1 "AT5G65720" species:3702 "Arabidopsis thaliana" [GO:0008483 "transaminase activity" evidence=ISS] [GO:0005739 "mitochondrion" evidence=IDA] [GO:0031071 "cysteine desulfurase activity" evidence=IDA] [GO:0005524 "ATP binding" evidence=IDA] [GO:0016226 "iron-sulfur cluster assembly" evidence=IDA] [GO:0008270 "zinc ion binding" evidence=IDA] InterPro:IPR000192 InterPro:IPR010240 InterPro:IPR015421 InterPro:IPR015422 Pfam:PF00266 GO:GO:0005739 GO:GO:0005524 EMBL:CP002688 GenomeReviews:BA000015_GR GO:GO:0030170 InterPro:IPR015424 Gene3D:G3DSA:3.40.640.10 Gene3D:G3DSA:3.90.1150.10 SUPFAM:SSF53383 EMBL:AB010075 EMBL:AL021684 GO:GO:0008270 GO:GO:0016226 HOGENOM:HBG635316 GO:GO:0031071 GO:GO:0006534 eggNOG:COG1104 KO:K04487 OMA:CIHHVKR InterPro:IPR016454 PIRSF:PIRSF005572 TIGRFAMs:TIGR02006 EMBL:AJ243393 EMBL:AF229854 EMBL:AY050874 EMBL:AY096358 IPI:IPI00545655 PIR:T05896 RefSeq:NP_201373.1 UniGene:At.696 ProteinModelPortal:O49543 SMR:O49543 STRING:O49543 PRIDE:O49543 EnsemblPlants:AT5G65720.1 GeneID:836701 KEGG:ath:AT5G65720 TAIR:At5g65720 InParanoid:O49543 PhylomeDB:O49543 ProtClustDB:PLN02651 BRENDA:2.8.1.6 ArrayExpress:O49543 Genevestigator:O49543 GermOnline:AT5G65720 Uniprot:O49543
Root	Isotig01848	32	5	2.549	8.76E-06	TAIR|locus:2169985 - symbol:NFS1 "AT5G65720" species:3702 "Arabidopsis thaliana" [GO:0008483 "transaminase activity" evidence=ISS] [GO:0005739 "mitochondrion" evidence=IDA] [GO:0031071 "cysteine desulfurase activity" evidence=IDA] [GO:0005524 "ATP binding" evidence=IDA] [GO:0016226 "iron-sulfur cluster assembly" evidence=IDA] [GO:0008270 "zinc ion binding" evidence=IDA] InterPro:IPR000192 InterPro:IPR010240 InterPro:IPR015421 InterPro:IPR015422 Pfam:PF00266 GO:GO:0005739 GO:GO:0005524 EMBL:CP002688 GenomeReviews:BA000015_GR GO:GO:0030170 InterPro:IPR015424 Gene3D:G3DSA:3.40.640.10 Gene3D:G3DSA:3.90.1150.10 SUPFAM:SSF53383 EMBL:AB010075 EMBL:AL021684 GO:GO:0008270 GO:GO:0016226 HOGENOM:HBG635316 GO:GO:0031071 GO:GO:0006534 eggNOG:COG1104 KO:K04487

Tissue	Identification	# of reads (stress)	# of reads (control)	Fold Change	p-value	Description
						OMA:CIHHVKR InterPro:IPR016454 PIRSF:PIRSF005572 TIGRFAMs:TIGR02006 EMBL:AJ243393 EMBL:AF229854 EMBL:AY050874 EMBL:AY096358 IPI:IPI00545655 PIR:T05896 RefSeq:NP_201373.1 UniGene:At.696 ProteinModelPortal:O49543 SMR:O49543 STRING:O49543 PRIDE:O49543 EnsemblPlants:AT5G65720.1 GeneID:836701 KEGG:ath:AT5G65720 TAIR:At5g65720 InParanoid:O49543 PhylomeDB:O49543 ProtClustDB:PLN02651 BRENDA:2.8.1.6 ArrayExpress:O49543 Genevestigator:O49543 GermOnline:AT5G65720 Uniprot:O49543
Root	Isotig01849	34	5	2.637	3.03E-06	TAIR|locus:2169985 - symbol:NFS1 "AT5G65720" species:3702 "Arabidopsis thaliana" [GO:0008483 "transaminase activity" evidence=ISS] [GO:0005739 "mitochondrion" evidence=IDA] [GO:0031071 "cysteine desulfurase activity" evidence=IDA] [GO:0005524 "ATP binding" evidence=IDA] [GO:0016226 "iron-sulfur cluster assembly" evidence=IDA] [GO:0008270 "zinc ion binding" evidence=IDA] InterPro:IPR000192 InterPro:IPR010240 InterPro:IPR015421 InterPro:IPR015422 Pfam:PF00266 GO:GO:0005739 GO:GO:0005524 EMBL:CP002688 GenomeReviews:BA000015_GR GO:GO:0030170 InterPro:IPR015424 Gene3D:G3DSA:3.40.640.10 Gene3D:G3DSA:3.90.1150.10 SUPFAM:SSF53383 EMBL:AB010075 EMBL:AL021684 GO:GO:0008270 GO:GO:0016226 HOGENOM:HBG635316 GO:GO:0031071 GO:GO:0006534 eggNOG:COG1104 KO:K04487 OMA:CIHHVKR InterPro:IPR016454 PIRSF:PIRSF005572 TIGRFAMs:TIGR02006 EMBL:AJ243393 EMBL:AF229854 EMBL:AY050874 EMBL:AY096358 IPI:IPI00545655 PIR:T05896 RefSeq:NP_201373.1 UniGene:At.696 ProteinModelPortal:O49543 SMR:O49543 STRING:O49543 PRIDE:O49543 EnsemblPlants:AT5G65720.1 GeneID:836701 KEGG:ath:AT5G65720 TAIR:At5g65720 InParanoid:O49543 PhylomeDB:O49543 ProtClustDB:PLN02651 BRENDA:2.8.1.6 ArrayExpress:O49543 Genevestigator:O49543 GermOnline:AT5G65720 Uniprot:O49543
Root	Isotig01850	36	5	2.719	1.04E-06	TAIR|locus:2169985 - symbol:NFS1 "AT5G65720" species:3702 "Arabidopsis thaliana" [GO:0008483 "transaminase activity" evidence=ISS] [GO:0005739 "mitochondrion" evidence=IDA] [GO:0031071 "cysteine desulfurase activity" evidence=IDA] [GO:0005524 "ATP binding" evidence=IDA] [GO:0016226 "iron-sulfur cluster assembly" evidence=IDA] [GO:0008270 "zinc ion binding" evidence=IDA] InterPro:IPR000192 InterPro:IPR010240 InterPro:IPR015421 InterPro:IPR015422 Pfam:PF00266 GO:GO:0005739 GO:GO:0005524 EMBL:CP002688 GenomeReviews:BA000015_GR GO:GO:0030170 InterPro:IPR015424 Gene3D:G3DSA:3.40.640.10 Gene3D:G3DSA:3.90.1150.10 SUPFAM:SSF53383 EMBL:AB010075 EMBL:AL021684 GO:GO:0008270 GO:GO:0016226 HOGENOM:HBG635316 GO:GO:0031071 GO:GO:0006534 eggNOG:COG1104 KO:K04487 OMA:CIHHVKR InterPro:IPR016454 PIRSF:PIRSF005572 TIGRFAMs:TIGR02006 EMBL:AJ243393 EMBL:AF229854 EMBL:AY050874 EMBL:AY096358 IPI:IPI00545655 PIR:T05896 RefSeq:NP_201373.1 UniGene:At.696 ProteinModelPortal:O49543 SMR:O49543 STRING:O49543 PRIDE:O49543 EnsemblPlants:AT5G65720.1 GeneID:836701 KEGG:ath:AT5G65720 TAIR:At5g65720 InParanoid:O49543 PhylomeDB:O49543 ProtClustDB:PLN02651 BRENDA:2.8.1.6 ArrayExpress:O49543 Genevestigator:O49543 GermOnline:AT5G65720 Uniprot:O49543
Root	Isotig01851	32	5	2.549	8.76E-06	TAIR|locus:2169985 - symbol:NFS1 "AT5G65720" species:3702 "Arabidopsis thaliana" [GO:0008483 "transaminase activity" evidence=ISS] [GO:0005739 "mitochondrion" evidence=IDA] [GO:0031071 "cysteine desulfurase activity" evidence=IDA] [GO:0005524 "ATP binding" evidence=IDA] [GO:0016226 "iron-sulfur cluster assembly" evidence=IDA] [GO:0008270 "zinc ion binding" evidence=IDA] InterPro:IPR000192 InterPro:IPR010240 InterPro:IPR015421 InterPro:IPR015422 Pfam:PF00266 GO:GO:0005739 GO:GO:0005524 EMBL:CP002688 GenomeReviews:BA000015_GR GO:GO:0030170 InterPro:IPR015424 Gene3D:G3DSA:3.40.640.10 Gene3D:G3DSA:3.90.1150.10 SUPFAM:SSF53383 EMBL:AB010075 EMBL:AL021684 GO:GO:0008270 GO:GO:0016226 HOGENOM:HBG635316 GO:GO:0031071 GO:GO:0006534 eggNOG:COG1104 KO:K04487 OMA:CIHHVKR InterPro:IPR016454 PIRSF:PIRSF005572 TIGRFAMs:TIGR02006 EMBL:AJ243393 EMBL:AF229854 EMBL:AY050874 EMBL:AY096358 IPI:IPI00545655 PIR:T05896 RefSeq:NP_201373.1 UniGene:At.696 ProteinModelPortal:O49543 SMR:O49543 STRING:O49543 PRIDE:O49543 EnsemblPlants:AT5G65720.1 GeneID:836701 KEGG:ath:AT5G65720 TAIR:At5g65720 InParanoid:O49543 PhylomeDB:O49543 ProtClustDB:PLN02651 BRENDA:2.8.1.6 ArrayExpress:O49543 Genevestigator:O49543 GermOnline:AT5G65720 Uniprot:O49543
Root	Isotig01858	0	14	-4.936	8.24E-05	TAIR|locus:2205190 - symbol:NRT2:1 "AT1G08090" species:3702 "Arabidopsis thaliana" [GO:0015112 "nitrate transmembrane transporter activity" evidence=ISS;TAS] [GO:0015706 "nitrate transport" evidence=IMP] [GO:0016020 "membrane" evidence=ISS] [GO:0048527 "lateral root development" evidence=IMP] [GO:0010167 "response to nitrate" evidence=IEP] InterPro:IPR011701 Pfam:PF07690 GO:GO:0016021 EMBL:CP002684

Tissue	Identification	# of reads (stress)	# of reads (control)	Fold Change	p-value	Description
						GenomeReviews:CT485782_GR GO:GO:0005886 GO:GO:0048527 InterPro:IPR016196 SUPFAM:SSF103473 PROSITE:PS50850 GO:GO:0015112 GO:GO:0042128 GO:GO:0010167 EMBL:AC026875 eggNOG:COG2223 EMBL:Z97058 EMBL:AF019748 EMBL:AF093754 IPI:IPI00522021 PIR:T51836 RefSeq:NP_172288.1 UniGene:At.5407 ProteinModelPortal:O82811 SMR:O82811 IntAct:O82811 STRING:O82811 TCDB:2.A.1.8.12 PRIDE:O82811 ProMEX:O82811 EnsemblPlants:AT1G08090.1 GeneID:837327 KEGG:ath:AT1G08090 TAIR:At1g08090 HOGENOM:HBG617617 InParanoid:O82811 OMA:ANFAIVP PhylomeDB:O82811 ProtClustDB:PLN00028 Genevestigator:O82811 Uniprot:O82811
Root	Isotig01859	0	14	-4.936	8.24E-05	TAIR|locus:2205190 - symbol:NRT2:1 "AT1G08090" species:3702 "Arabidopsis thaliana" [GO:0015112 "nitrate transmembrane transporter activity" evidence=ISS;TAS] [GO:0015706 "nitrate transport" evidence=IMP] [GO:0016020 "membrane" evidence=ISS] [GO:0048527 "lateral root development" evidence=IMP] [GO:0010167 "response to nitrate" evidence=IEP] InterPro:IPR011701 Pfam:PF07690 GO:GO:0016021 EMBL:CP002684 GenomeReviews:CT485782_GR GO:GO:0005886 GO:GO:0048527 InterPro:IPR016196 SUPFAM:SSF103473 PROSITE:PS50850 GO:GO:0015112 GO:GO:0042128 GO:GO:0010167 EMBL:AC026875 eggNOG:COG2223 EMBL:Z97058 EMBL:AF019748 EMBL:AF093754 IPI:IPI00522021 PIR:T51836 RefSeq:NP_172288.1 UniGene:At.5407 ProteinModelPortal:O82811 SMR:O82811 IntAct:O82811 STRING:O82811 TCDB:2.A.1.8.12 PRIDE:O82811 ProMEX:O82811 EnsemblPlants:AT1G08090.1 GeneID:837327 KEGG:ath:AT1G08090 TAIR:At1g08090 HOGENOM:HBG617617 InParanoid:O82811 OMA:ANFAIVP PhylomeDB:O82811 ProtClustDB:PLN00028 Genevestigator:O82811 Uniprot:O82811
Root	Isotig01860	0	13	-4.829	0.000155955	TAIR|locus:2205190 - symbol:NRT2:1 "AT1G08090" species:3702 "Arabidopsis thaliana" [GO:0015112 "nitrate transmembrane transporter activity" evidence=ISS;TAS] [GO:0015706 "nitrate transport" evidence=IMP] [GO:0016020 "membrane" evidence=ISS] [GO:0048527 "lateral root development" evidence=IMP] [GO:0010167 "response to nitrate" evidence=IEP] InterPro:IPR011701 Pfam:PF07690 GO:GO:0016021 EMBL:CP002684 GenomeReviews:CT485782_GR GO:GO:0005886 GO:GO:0048527 InterPro:IPR016196 SUPFAM:SSF103473 PROSITE:PS50850 GO:GO:0015112 GO:GO:0042128 GO:GO:0010167 EMBL:AC026875 eggNOG:COG2223 EMBL:Z97058 EMBL:AF019748 EMBL:AF093754 IPI:IPI00522021 PIR:T51836 RefSeq:NP_172288.1 UniGene:At.5407 ProteinModelPortal:O82811 SMR:O82811 IntAct:O82811 STRING:O82811 TCDB:2.A.1.8.12 PRIDE:O82811 ProMEX:O82811 EnsemblPlants:AT1G08090.1 GeneID:837327 KEGG:ath:AT1G08090 TAIR:At1g08090 HOGENOM:HBG617617 InParanoid:O82811 OMA:ANFAIVP PhylomeDB:O82811 ProtClustDB:PLN00028 Genevestigator:O82811 Uniprot:O82811
Root	Isotig01861	0	13	-4.829	0.000155955	TAIR|locus:2205190 - symbol:NRT2:1 "AT1G08090" species:3702 "Arabidopsis thaliana" [GO:0015112 "nitrate transmembrane transporter activity" evidence=ISS;TAS] [GO:0015706 "nitrate transport" evidence=IMP] [GO:0016020 "membrane" evidence=ISS] [GO:0048527 "lateral root development" evidence=IMP] [GO:0010167 "response to nitrate" evidence=IEP] InterPro:IPR011701 Pfam:PF07690 GO:GO:0016021 EMBL:CP002684 GenomeReviews:CT485782_GR GO:GO:0005886 GO:GO:0048527 InterPro:IPR016196 SUPFAM:SSF103473 PROSITE:PS50850 GO:GO:0015112 GO:GO:0042128 GO:GO:0010167 EMBL:AC026875 eggNOG:COG2223 EMBL:Z97058 EMBL:AF019748 EMBL:AF093754 IPI:IPI00522021 PIR:T51836 RefSeq:NP_172288.1 UniGene:At.5407 ProteinModelPortal:O82811 SMR:O82811 IntAct:O82811 STRING:O82811 TCDB:2.A.1.8.12 PRIDE:O82811 ProMEX:O82811 EnsemblPlants:AT1G08090.1 GeneID:837327 KEGG:ath:AT1G08090 TAIR:At1g08090 HOGENOM:HBG617617 InParanoid:O82811 OMA:ANFAIVP PhylomeDB:O82811 ProtClustDB:PLN00028 Genevestigator:O82811 Uniprot:O82811
Root	Isotig01870	0	11	-4.588	0.000569522	TAIR|locus:2153924 - symbol:DMR6 "AT5G24530" species:3702 "Arabidopsis thaliana" [GO:0005575 "cellular_component" evidence=ND] [GO:0009813 "flavonoid biosynthetic process" evidence=ISS] [GO:0016491 "oxidoreductase activity" evidence=IEA] [GO:0016706 "oxidoreductase activity, acting on paired donors, with incorporation or reduction of molecular oxygen, 2-oxoglutarate as one donor, and incorporation of one atom each of oxygen into both donors" evidence=ISS] [GO:0009617 "response to bacterium" evidence=IGI] [GO:0009620 "response to fungus" evidence=IEP;IGI] InterPro:IPR005123 Pfam:PF03171 PROSITE:PS51471 EMBL:CP002688 GO:GO:0009617 GO:GO:0016706 HSSP:Q96323 GO:GO:0009620 EMBL:AB010068 EMBL:AF386975 EMBL:AY081455 IPI:IPI00531388 RefSeq:NP_197841.1 UniGene:At.23761 ProteinModelPortal:Q9FLV0 SMR:Q9FLV0 IntAct:Q9FLV0 STRING:Q9FLV0 PRIDE:Q9FLV0 EnsemblPlants:AT5G24530.1 GeneID:832524 KEGG:ath:AT5G24530 TAIR:At5g24530 InParanoid:Q9FLV0 OMA:NPHPDAF PhylomeDB:Q9FLV0 ProtClustDB:PLN02639 ArrayExpress:Q9FLV0 Genevestigator:Q9FLV0 Uniprot:Q9FLV0

Tissue	Identification	# of reads (stress)	# of reads (control)	Fold Change	p-value	Description
Root	Isotig01871	0	11	-4.588	0.000569522	TAIR|locus:2153924 - symbol:DMR6 "AT5G24530" species:3702 "Arabidopsis thaliana" [GO:0005575 "cellular_component" evidence=ND] [GO:0009813 "flavonoid biosynthetic process" evidence=ISS] [GO:0016491 "oxidoreductase activity" evidence=IEA] [GO:0016706 "oxidoreductase activity, acting on paired donors, with incorporation or reduction of molecular oxygen, 2-oxoglutarate as one donor, and incorporation of one atom each of oxygen into both donors" evidence=ISS] [GO:0009617 "response to bacterium" evidence=IGI] [GO:0009620 "response to fungus" evidence=IEP;IGI] InterPro:IPR005123 Pfam:PF03171 PROSITE:PS51471 EMBL:CP002688 GO:GO:0009617 GO:GO:0016706 HSSP:Q96323 GO:GO:0009620 EMBL:AB010068 EMBL:AF386975 EMBL:AY081455 IPI:IPI00531388 RefSeq:NP_197841.1 UniGene:At.23761 ProteinModelPortal:Q9FLV0 SMR:Q9FLV0 IntAct:Q9FLV0 STRING:Q9FLV0 PRIDE:Q9FLV0 EnsemblPlants:AT5G24530.1 GeneID:832524 KEGG:ath:AT5G24530 TAIR:At5g24530 InParanoid:Q9FLV0 OMA:NPHPDAF PhylomeDB:Q9FLV0 ProtClustDB:PLN02639 ArrayExpress:Q9FLV0 Genevestigator:Q9FLV0 Uniprot:Q9FLV0
Root	Isotig01872	0	11	-4.588	0.000569522	TAIR|locus:2153924 - symbol:DMR6 "AT5G24530" species:3702 "Arabidopsis thaliana" [GO:0005575 "cellular_component" evidence=ND] [GO:0009813 "flavonoid biosynthetic process" evidence=ISS] [GO:0016491 "oxidoreductase activity" evidence=IEA] [GO:0016706 "oxidoreductase activity, acting on paired donors, with incorporation or reduction of molecular oxygen, 2-oxoglutarate as one donor, and incorporation of one atom each of oxygen into both donors" evidence=ISS] [GO:0009617 "response to bacterium" evidence=IGI] [GO:0009620 "response to fungus" evidence=IEP;IGI] InterPro:IPR005123 Pfam:PF03171 PROSITE:PS51471 EMBL:CP002688 GO:GO:0009617 GO:GO:0016706 HSSP:Q96323 GO:GO:0009620 EMBL:AB010068 EMBL:AF386975 EMBL:AY081455 IPI:IPI00531388 RefSeq:NP_197841.1 UniGene:At.23761 ProteinModelPortal:Q9FLV0 SMR:Q9FLV0 IntAct:Q9FLV0 STRING:Q9FLV0 PRIDE:Q9FLV0 EnsemblPlants:AT5G24530.1 GeneID:832524 KEGG:ath:AT5G24530 TAIR:At5g24530 InParanoid:Q9FLV0 OMA:NPHPDAF PhylomeDB:Q9FLV0 ProtClustDB:PLN02639 ArrayExpress:Q9FLV0 Genevestigator:Q9FLV0 Uniprot:Q9FLV0
Root	Isotig01873	0	11	-4.588	0.000569522	TAIR|locus:2153924 - symbol:DMR6 "AT5G24530" species:3702 "Arabidopsis thaliana" [GO:0005575 "cellular_component" evidence=ND] [GO:0009813 "flavonoid biosynthetic process" evidence=ISS] [GO:0016491 "oxidoreductase activity" evidence=IEA] [GO:0016706 "oxidoreductase activity, acting on paired donors, with incorporation or reduction of molecular oxygen, 2-oxoglutarate as one donor, and incorporation of one atom each of oxygen into both donors" evidence=ISS] [GO:0009617 "response to bacterium" evidence=IGI] [GO:0009620 "response to fungus" evidence=IEP;IGI] InterPro:IPR005123 Pfam:PF03171 PROSITE:PS51471 EMBL:CP002688 GO:GO:0009617 GO:GO:0016706 HSSP:Q96323 GO:GO:0009620 EMBL:AB010068 EMBL:AF386975 EMBL:AY081455 IPI:IPI00531388 RefSeq:NP_197841.1 UniGene:At.23761 ProteinModelPortal:Q9FLV0 SMR:Q9FLV0 IntAct:Q9FLV0 STRING:Q9FLV0 PRIDE:Q9FLV0 EnsemblPlants:AT5G24530.1 GeneID:832524 KEGG:ath:AT5G24530 TAIR:At5g24530 InParanoid:Q9FLV0 OMA:NPHPDAF PhylomeDB:Q9FLV0 ProtClustDB:PLN02639 ArrayExpress:Q9FLV0 Genevestigator:Q9FLV0 Uniprot:Q9FLV0
Root	Isotig01882	7	40	-2.644	1.14E-07	TAIR|locus:2180547 - symbol:NADP-ME3 "AT5G25880" species:3702 "Arabidopsis thaliana" [GO:0004470 "malic enzyme activity" evidence=ISS] [GO:0006108 "malate metabolic process" evidence=ISS;IDA] [GO:0016652 "oxidoreductase activity, acting on NADH or NADPH, NAD or NADP as acceptor" evidence=ISS] [GO:0004473 "malate dehydrogenase (oxaloacetate-decarboxylating) (NADP+) activity" evidence=IDA] [GO:0051260 "protein homooligomerization" evidence=IDA] [GO:0005829 "cytosol" evidence=IDA] InterPro:IPR001891 InterPro:IPR012301 InterPro:IPR012302 InterPro:IPR015884 InterPro:IPR016040 Pfam:PF00390 Pfam:PF03949 PIRSF:PIRSF000106 PRINTS:PR00072 PROSITE:PS00331 SMART:SM00919 GO:GO:0005829 EMBL:CP002688 GenomeReviews:BA000015_GR Gene3D:G3DSA:3.40.50.720 GO:GO:0051287 GO:GO:0046872 GO:GO:0051260 EMBL:AF149413 GO:GO:0006108 HOGENOM:HBG289821 GO:GO:0016619 Gene3D:G3DSA:3.40.50.10380 KO:K00029 GO:GO:0004473 HSSP:P40927 ProtClustDB:PLN03129 IPI:IPI00535942 RefSeq:NP_197960.1 UniGene:At.43327 ProteinModelPortal:Q9XGZ0 SMR:Q9XGZ0 STRING:Q9XGZ0 PRIDE:Q9XGZ0 EnsemblPlants:AT5G25880.1 GeneID:832657 KEGG:ath:AT5G25880 TAIR:At5g25880 InParanoid:Q9XGZ0 OMA:RERETHY PhylomeDB:Q9XGZ0 Genevestigator:Q9XGZ0 Uniprot:Q9XGZ0
Root	Isotig01883	7	40	-2.644	1.14E-07	TAIR|locus:2180547 - symbol:NADP-ME3 "AT5G25880" species:3702 "Arabidopsis thaliana" [GO:0004470 "malic enzyme activity" evidence=ISS] [GO:0006108 "malate metabolic process" evidence=ISS;IDA] [GO:0016652 "oxidoreductase activity, acting on NADH or NADPH, NAD or NADP as acceptor" evidence=ISS] [GO:0004473 "malate dehydrogenase (oxaloacetate-decarboxylating) (NADP+) activity" evidence=IDA] [GO:0051260 "protein homooligomerization" evidence=IDA] [GO:0005829 "cytosol" evidence=IDA] InterPro:IPR001891 InterPro:IPR012301 InterPro:IPR012302 InterPro:IPR015884 InterPro:IPR016040 Pfam:PF00390 Pfam:PF03949

Tissue	Identification	# of reads (stress)	# of reads (control)	Fold Change	p-value	Description
						PIRSF:PIRSF000106 PRINTS:PR00072 PROSITE:PS00331 SMART:SM00919 GO:GO:0005829 EMBL:CP002688 GenomeReviews:BA000015_GR Gene3D:G3DSA:3.40.50.720 GO:GO:0051287 GO:GO:0046872 GO:GO:0051260 EMBL:AF149413 GO:GO:0006108 HOGENOM:HBG289821 GO:GO:0016619 Gene3D:G3DSA:3.40.50.10380 KO:K00029 GO:GO:0004473 HSSP:P40927 ProtClustDB:PLN03129 IPI:IPI00535942 RefSeq:NP_197960.1 UniGene:At.43327 ProteinModelPortal:Q9XGZ0 SMR:Q9XGZ0 STRING:Q9XGZ0 PRIDE:Q9XGZ0 EnsemblPlants:AT5G25880.1 GeneID:832657 KEGG:ath:AT5G25880 TAIR:At5g25880 InParanoid:Q9XGZ0 OMA:RERETHY PhylomeDB:Q9XGZ0 Genevestigator:Q9XGZ0 Uniprot:Q9XGZ0
Root	Isotig01884	7	40	-2.644	1.14E-07	TAIR|locus:2180547 - symbol:NADP-ME3 "AT5G25880" species:3702 "Arabidopsis thaliana" [GO:0004470 "malic enzyme activity" evidence=ISS] [GO:0006108 "malate metabolic process" evidence=ISS;IDA] [GO:0016652 "oxidoreductase activity, acting on NADH or NADPH, NAD or NADP as acceptor" evidence=ISS] [GO:0004473 "malate dehydrogenase (oxaloacetate-decarboxylating) (NADP+) activity" evidence=IDA] [GO:0051260 "protein homooligomerization" evidence=IDA] [GO:0005829 "cytosol" evidence=IDA] InterPro:IPR001891 InterPro:IPR012301 InterPro:IPR012302 InterPro:IPR015884 InterPro:IPR016040 Pfam:PF00390 Pfam:PF03949 PIRSF:PIRSF000106 PRINTS:PR00072 PROSITE:PS00331 SMART:SM00919 GO:GO:0005829 EMBL:CP002688 GenomeReviews:BA000015_GR Gene3D:G3DSA:3.40.50.720 GO:GO:0051287 GO:GO:0046872 GO:GO:0051260 EMBL:AF149413 GO:GO:0006108 HOGENOM:HBG289821 GO:GO:0016619 Gene3D:G3DSA:3.40.50.10380 KO:K00029 GO:GO:0004473 HSSP:P40927 ProtClustDB:PLN03129 IPI:IPI00535942 RefSeq:NP_197960.1 UniGene:At.43327 ProteinModelPortal:Q9XGZ0 SMR:Q9XGZ0 STRING:Q9XGZ0 PRIDE:Q9XGZ0 EnsemblPlants:AT5G25880.1 GeneID:832657 KEGG:ath:AT5G25880 TAIR:At5g25880 InParanoid:Q9XGZ0 OMA:RERETHY PhylomeDB:Q9XGZ0 Genevestigator:Q9XGZ0 Uniprot:Q9XGZ0
Root	Isotig01894	1	26	-4.829	8.91E-08	TAIR|locus:2024046 - symbol:AT1G24620 species:3702 "Arabidopsis thaliana" [GO:0005509 "calcium ion binding" evidence=IEA;ISS] [GO:0005634 "nucleus" evidence=IDA] [GO:0009409 "response to cold" evidence=IEP] [GO:0005737 "cytoplasm" evidence=IDA] [GO:0048767 "root hair elongation" evidence=IMP] InterPro:IPR002048 InterPro:IPR011992 SMART:SM00054 Prosite:PS00018 EMBL:CP002684 GenomeReviews:CT485782_GR GO:GO:0005634 GO:GO:0005737 GO:GO:0005509 InterPro:IPR018247 InterPro:IPR018249 Gene3D:G3DSA:1.10.238.10 PROSITE:PS50222 GO:GO:0009409 GO:GO:0048767 EMBL:AC000103 eggNOG:COG5126 HOGENOM:HBG746798 HSSP:P62157 KO:K13448 EMBL:DQ446288 IPI:IPI00543078 PIR:F86379 RefSeq:NP_173866.1 UniGene:At.51279 ProteinModelPortal:Q9FYK2 SMR:Q9FYK2 PRIDE:Q9FYK2 EnsemblPlants:AT1G24620.1 GeneID:839076 KEGG:ath:AT1G24620 TAIR:At1g24620 InParanoid:Q9FYK2 OMA:GDECSIA PhylomeDB:Q9FYK2 ProtClustDB:CLSN2682405 Genevestigator:Q9FYK2 Uniprot:Q9FYK2
Root	Isotig01895	1	26	-4.829	8.91E-08	TAIR|locus:2024046 - symbol:AT1G24620 species:3702 "Arabidopsis thaliana" [GO:0005509 "calcium ion binding" evidence=IEA;ISS] [GO:0005634 "nucleus" evidence=IDA] [GO:0009409 "response to cold" evidence=IEP] [GO:0005737 "cytoplasm" evidence=IDA] [GO:0048767 "root hair elongation" evidence=IMP] InterPro:IPR002048 InterPro:IPR011992 SMART:SM00054 Prosite:PS00018 EMBL:CP002684 GenomeReviews:CT485782_GR GO:GO:0005634 GO:GO:0005737 GO:GO:0005509 InterPro:IPR018247 InterPro:IPR018249 Gene3D:G3DSA:1.10.238.10 PROSITE:PS50222 GO:GO:0009409 GO:GO:0048767 EMBL:AC000103 eggNOG:COG5126 HOGENOM:HBG746798 HSSP:P62157 KO:K13448 EMBL:DQ446288 IPI:IPI00543078 PIR:F86379 RefSeq:NP_173866.1 UniGene:At.51279 ProteinModelPortal:Q9FYK2 SMR:Q9FYK2 PRIDE:Q9FYK2 EnsemblPlants:AT1G24620.1 GeneID:839076 KEGG:ath:AT1G24620 TAIR:At1g24620 InParanoid:Q9FYK2 OMA:GDECSIA PhylomeDB:Q9FYK2 ProtClustDB:CLSN2682405 Genevestigator:Q9FYK2 Uniprot:Q9FYK2
Root	Isotig01896	1	26	-4.829	8.91E-08	TAIR|locus:2024046 - symbol:AT1G24620 species:3702 "Arabidopsis thaliana" [GO:0005509 "calcium ion binding" evidence=IEA;ISS] [GO:0005634 "nucleus" evidence=IDA] [GO:0009409 "response to cold" evidence=IEP] [GO:0005737 "cytoplasm" evidence=IDA] [GO:0048767 "root hair elongation" evidence=IMP] InterPro:IPR002048 InterPro:IPR011992 SMART:SM00054 Prosite:PS00018 EMBL:CP002684 GenomeReviews:CT485782_GR GO:GO:0005634 GO:GO:0005737 GO:GO:0005509 InterPro:IPR018247 InterPro:IPR018249 Gene3D:G3DSA:1.10.238.10 PROSITE:PS50222 GO:GO:0009409 GO:GO:0048767 EMBL:AC000103 eggNOG:COG5126 HOGENOM:HBG746798 HSSP:P62157 KO:K13448 EMBL:DQ446288 IPI:IPI00543078 PIR:F86379 RefSeq:NP_173866.1 UniGene:At.51279 ProteinModelPortal:Q9FYK2 SMR:Q9FYK2 PRIDE:Q9FYK2 EnsemblPlants:AT1G24620.1 GeneID:839076 KEGG:ath:AT1G24620 TAIR:At1g24620 InParanoid:Q9FYK2 OMA:GDECSIA PhylomeDB:Q9FYK2 ProtClustDB:CLSN2682405 Genevestigator:Q9FYK2 Uniprot:Q9FYK2
Root	Isotig01897	1	26	-4.829	8.91E-08	TAIR|locus:2024046 - symbol:AT1G24620 species:3702 "Arabidopsis thaliana" [GO:0005509 "calcium ion binding" evidence=IEA;ISS] [GO:0005634 "nucleus" evidence=IDA] [GO:0009409 "response to cold" evidence=IEP]

Tissue	Identification	# of reads (stress)	# of reads (control)	Fold Change	p-value	Description
						[GO:0005737 "cytoplasm" evidence=IDA] [GO:0048767 "root hair elongation" evidence=IMP] InterPro:IPR002048 InterPro:IPR011992 SMART:SM00054 Prosite:PS00018 EMBL:CP002684 GenomeReviews:CT485782_GR GO:GO:0005634 GO:GO:0005737 GO:GO:0005509 InterPro:IPR018247 InterPro:IPR018249 Gene3D:G3DSA:1.10.238.10 PROSITE:PS50222 GO:GO:0009409 GO:GO:0048767 EMBL:AC000103 eggNOG:COG5126 HOGENOM:HBG746798 HSSP:P62157 KO:K13448 EMBL:DQ446288 IPI:IPI00543078 PIR:F86379 RefSeq:NP_173866.1 UniGene:At.51279 ProteinModelPortal:Q9FYK2 SMR:Q9FYK2 PRIDE:Q9FYK2 EnsemblPlants:AT1G24620.1 GeneID:839076 KEGG:ath:AT1G24620 TAIR:At1g24620 InParanoid:Q9FYK2 OMA:GDECSIA PhylomeDB:Q9FYK2 ProtClustDB:CLSN2682405 Genevestigator:Q9FYK2 Uniprot:Q9FYK2
Root	Isotig01898	1	26	-4.829	8.91E-08	TAIR|locus:2024046 - symbol:AT1G24620 species:3702 "Arabidopsis thaliana" [GO:0005509 "calcium ion binding" evidence=IEA;ISS] [GO:0005634 "nucleus" evidence=IDA] [GO:0009409 "response to cold" evidence=IEP] [GO:0005737 "cytoplasm" evidence=IDA] [GO:0048767 "root hair elongation" evidence=IMP] InterPro:IPR002048 InterPro:IPR011992 SMART:SM00054 Prosite:PS00018 EMBL:CP002684 GenomeReviews:CT485782_GR GO:GO:0005634 GO:GO:0005737 GO:GO:0005509 InterPro:IPR018247 InterPro:IPR018249 Gene3D:G3DSA:1.10.238.10 PROSITE:PS50222 GO:GO:0009409 GO:GO:0048767 EMBL:AC000103 eggNOG:COG5126 HOGENOM:HBG746798 HSSP:P62157 KO:K13448 EMBL:DQ446288 IPI:IPI00543078 PIR:F86379 RefSeq:NP_173866.1 UniGene:At.51279 ProteinModelPortal:Q9FYK2 SMR:Q9FYK2 PRIDE:Q9FYK2 EnsemblPlants:AT1G24620.1 GeneID:839076 KEGG:ath:AT1G24620 TAIR:At1g24620 InParanoid:Q9FYK2 OMA:GDECSIA PhylomeDB:Q9FYK2 ProtClustDB:CLSN2682405 Genevestigator:Q9FYK2 Uniprot:Q9FYK2
Root	Isotig01899	1	26	-4.829	8.91E-08	TAIR|locus:2024046 - symbol:AT1G24620 species:3702 "Arabidopsis thaliana" [GO:0005509 "calcium ion binding" evidence=IEA;ISS] [GO:0005634 "nucleus" evidence=IDA] [GO:0009409 "response to cold" evidence=IEP] [GO:0005737 "cytoplasm" evidence=IDA] [GO:0048767 "root hair elongation" evidence=IMP] InterPro:IPR002048 InterPro:IPR011992 SMART:SM00054 Prosite:PS00018 EMBL:CP002684 GenomeReviews:CT485782_GR GO:GO:0005634 GO:GO:0005737 GO:GO:0005509 InterPro:IPR018247 InterPro:IPR018249 Gene3D:G3DSA:1.10.238.10 PROSITE:PS50222 GO:GO:0009409 GO:GO:0048767 EMBL:AC000103 eggNOG:COG5126 HOGENOM:HBG746798 HSSP:P62157 KO:K13448 EMBL:DQ446288 IPI:IPI00543078 PIR:F86379 RefSeq:NP_173866.1 UniGene:At.51279 ProteinModelPortal:Q9FYK2 SMR:Q9FYK2 PRIDE:Q9FYK2 EnsemblPlants:AT1G24620.1 GeneID:839076 KEGG:ath:AT1G24620 TAIR:At1g24620 InParanoid:Q9FYK2 OMA:GDECSIA PhylomeDB:Q9FYK2 ProtClustDB:CLSN2682405 Genevestigator:Q9FYK2 Uniprot:Q9FYK2
Root	Isotig01933	2	21	-3.521	1.10E-05	TAIR|locus:2181072 - symbol:AT5G01740 "AT5G01740" species:3702 "Arabidopsis thaliana" [GO:0003674 "molecular_function" evidence=ND] [GO:0005575 "cellular_component" evidence=ND] [GO:0008150 "biological_process" evidence=ND] EMBL:CP002688 GenomeReviews:BA000015_GR EMBL:AL162351 HOGENOM:HBG606004 InterPro:IPR009798 Pfam:PF07107 EMBL:AY075604 EMBL:BT014870 IPI:IPI00541380 PIR:T48195 RefSeq:NP_195794.1 UniGene:At.33512 ProteinModelPortal:Q9LZX2 DNASU:831686 EnsemblPlants:AT5G01740.1 GeneID:831686 KEGG:ath:AT5G01740 TAIR:At5g01740 eggNOG:NOG315363 InParanoid:Q9LZX2 OMA:CVIAEGW PhylomeDB:Q9LZX2 ProtClustDB:CLSN2687137 Genevestigator:Q9LZX2 Uniprot:Q9LZX2
Root	Isotig01955	130	72	0.723	0.000483064	TAIR|locus:2159300 - symbol:ABCF1 "AT5G60790" species:3702 "Arabidopsis thaliana" [GO:0005215 "transporter activity" evidence=ISS] [GO:0005886 "plasma membrane" evidence=IDA] InterPro:IPR003439 InterPro:IPR003593 InterPro:IPR017871 Pfam:PF00005 PROSITE:PS00211 PROSITE:PS50893 SMART:SM00382 GO:GO:0005886 GO:GO:0005524 EMBL:CP002688 GenomeReviews:BA000015_GR GO:GO:0006810 GO:GO:0016887 HOGENOM:HBG758042 EMBL:AB015472 EMBL:AY140066 EMBL:BT000946 EMBL:BT008363 EMBL:AK317570 EMBL:AK317690 IPI:IPI00540333 RefSeq:NP_200887.1 UniGene:At.9321 ProteinModelPortal:Q9FJH6 SMR:Q9FJH6 IntAct:Q9FJH6 STRING:Q9FJH6 PRIDE:Q9FJH6 EnsemblPlants:AT5G60790.1 GeneID:836200 KEGG:ath:AT5G60790 TAIR:At5g60790 eggNOG:COG0488 InParanoid:Q9FJH6 KO:K06185 OMA:YHQHLHE PhylomeDB:Q9FJH6 ProtClustDB:CLSN2686703 ArrayExpress:Q9FJH6 Genevestigator:Q9FJH6 Uniprot:Q9FJH6
Root	Isotig01957	130	72	0.723	0.000483064	TAIR|locus:2159300 - symbol:ABCF1 "AT5G60790" species:3702 "Arabidopsis thaliana" [GO:0005215 "transporter activity" evidence=ISS] [GO:0005886 "plasma membrane" evidence=IDA] InterPro:IPR003439 InterPro:IPR003593 InterPro:IPR017871 Pfam:PF00005 PROSITE:PS00211 PROSITE:PS50893 SMART:SM00382 GO:GO:0005886 GO:GO:0005524 EMBL:CP002688 GenomeReviews:BA000015_GR GO:GO:0006810 GO:GO:0016887 HOGENOM:HBG758042 EMBL:AB015472 EMBL:AY140066 EMBL:BT000946 EMBL:BT008363 EMBL:AK317570 EMBL:AK317690 IPI:IPI00540333 RefSeq:NP_200887.1 UniGene:At.9321 ProteinModelPortal:Q9FJH6 SMR:Q9FJH6 IntAct:Q9FJH6 STRING:Q9FJH6 PRIDE:Q9FJH6 EnsemblPlants:AT5G60790.1 GeneID:836200

Tissue	Identification	# of reads (stress)	# of reads (control)	Fold Change	p-value	Description
						KEGG:ath:AT5G60790 TAIR:At5g60790 eggNOG:COG0488 InParanoid:Q9FJH6 KO:K06185 OMA:YHQHLHE PhylomeDB:Q9FJH6 ProtClustDB:CLSN2686703 ArrayExpress:Q9FJH6 Genevestigator:Q9FJH6 Uniprot:Q9FJH6
Root	Isotig01959	7	32	-2.322	1.06E-05	TAIR|locus:2157022 - symbol:GH9A1 "AT5G49720" species:3702 "Arabidopsis thaliana" [GO:0004553 "hydrolase activity, hydrolyzing O-glycosyl compounds" evidence=ISS] [GO:0009504 "cell plate" evidence=IDA] [GO:0009826 "unidimensional cell growth" evidence=IMP] [GO:0009735 "response to cytokinin stimulus" evidence=IMP] [GO:0048367 "shoot development" evidence=IMP] [GO:0030244 "cellulose biosynthetic process" evidence=IMP;TAS] [GO:0005886 "plasma membrane" evidence=IDA] [GO:0005769 "early endosome" evidence=IDA] [GO:0005794 "Golgi apparatus" evidence=IDA] [GO:0042538 "hyperosmotic salinity response" evidence=IMP] [GO:0043622 "cortical microtubule organization" evidence=IMP] [GO:0008810 "cellulase activity" evidence=ISS] InterPro:IPR001701 InterPro:IPR008928 InterPro:IPR012341 InterPro:IPR018221 Pfam:PF00759 PROSITE:PS00592 PROSITE:PS00698 GO:GO:0016021 GO:GO:0005886 EMBL:CP002688 GenomeReviews:BA000015_GR GO:GO:0009735 GO:GO:0005794 GO:GO:0030245 GO:GO:0009826 GO:GO:0007047 Gene3D:G3DSA:1.50.10.10 SUPFAM:SSF48208 GO:GO:0048367 GO:GO:0005769 GO:GO:0042538 GO:GO:0043622 UniGene:At.23416 GO:GO:0009504 GO:GO:0030244 GO:GO:0008810 CAZy:GH9 EMBL:AB025613 HSSP:P26221 HOGENOM:HBG746347 ProtClustDB:CLSN2679649 EMBL:U37702 EMBL:AF073875 EMBL:AF074092 EMBL:AF074375 EMBL:AY037218 EMBL:BT002221 EMBL:AY086165 EMBL:AK221941 EMBL:AK222193 IPI:IPI00523242 PIR:S71215 RefSeq:NP_199783.1 UniGene:At.21098 ProteinModelPortal:Q38890 SMR:Q38890 STRING:Q38890 PRIDE:Q38890 EnsemblPlants:AT5G49720.1 GeneID:835035 KEGG:ath:AT5G49720 TAIR:At5g49720 eggNOG:NOG257354 InParanoid:Q38890 OMA:DEFIWGG PhylomeDB:Q38890 Genevestigator:Q38890 GermOnline:AT5G49720 Uniprot:Q38890
Root	Isotig01960	7	31	-2.276	1.84E-05	TAIR|locus:2157022 - symbol:GH9A1 "AT5G49720" species:3702 "Arabidopsis thaliana" [GO:0004553 "hydrolase activity, hydrolyzing O-glycosyl compounds" evidence=ISS] [GO:0009504 "cell plate" evidence=IDA] [GO:0009826 "unidimensional cell growth" evidence=IMP] [GO:0009735 "response to cytokinin stimulus" evidence=IMP] [GO:0048367 "shoot development" evidence=IMP] [GO:0030244 "cellulose biosynthetic process" evidence=IMP;TAS] [GO:0005886 "plasma membrane" evidence=IDA] [GO:0005769 "early endosome" evidence=IDA] [GO:0005794 "Golgi apparatus" evidence=IDA] [GO:0042538 "hyperosmotic salinity response" evidence=IMP] [GO:0043622 "cortical microtubule organization" evidence=IMP] [GO:0008810 "cellulase activity" evidence=ISS] InterPro:IPR001701 InterPro:IPR008928 InterPro:IPR012341 InterPro:IPR018221 Pfam:PF00759 PROSITE:PS00592 PROSITE:PS00698 GO:GO:0016021 GO:GO:0005886 EMBL:CP002688 GenomeReviews:BA000015_GR GO:GO:0009735 GO:GO:0005794 GO:GO:0030245 GO:GO:0009826 GO:GO:0007047 Gene3D:G3DSA:1.50.10.10 SUPFAM:SSF48208 GO:GO:0048367 GO:GO:0005769 GO:GO:0042538 GO:GO:0043622 UniGene:At.23416 GO:GO:0009504 GO:GO:0030244 GO:GO:0008810 CAZy:GH9 EMBL:AB025613 HSSP:P26221 HOGENOM:HBG746347 ProtClustDB:CLSN2679649 EMBL:U37702 EMBL:AF073875 EMBL:AF074092 EMBL:AF074375 EMBL:AY037218 EMBL:BT002221 EMBL:AY086165 EMBL:AK221941 EMBL:AK222193 IPI:IPI00523242 PIR:S71215 RefSeq:NP_199783.1 UniGene:At.21098 ProteinModelPortal:Q38890 SMR:Q38890 STRING:Q38890 PRIDE:Q38890 EnsemblPlants:AT5G49720.1 GeneID:835035 KEGG:ath:AT5G49720 TAIR:At5g49720 eggNOG:NOG257354 InParanoid:Q38890 OMA:DEFIWGG PhylomeDB:Q38890 Genevestigator:Q38890 GermOnline:AT5G49720 Uniprot:Q38890
Root	Isotig01961	7	31	-2.276	1.84E-05	TAIR|locus:2157022 - symbol:GH9A1 "AT5G49720" species:3702 "Arabidopsis thaliana" [GO:0004553 "hydrolase activity, hydrolyzing O-glycosyl compounds" evidence=ISS] [GO:0009504 "cell plate" evidence=IDA] [GO:0009826 "unidimensional cell growth" evidence=IMP] [GO:0009735 "response to cytokinin stimulus" evidence=IMP] [GO:0048367 "shoot development" evidence=IMP] [GO:0030244 "cellulose biosynthetic process" evidence=IMP;TAS] [GO:0005886 "plasma membrane" evidence=IDA] [GO:0005769 "early endosome" evidence=IDA] [GO:0005794 "Golgi apparatus" evidence=IDA] [GO:0042538 "hyperosmotic salinity response" evidence=IMP] [GO:0043622 "cortical microtubule organization" evidence=IMP] [GO:0008810 "cellulase activity" evidence=ISS] InterPro:IPR001701 InterPro:IPR008928 InterPro:IPR012341 InterPro:IPR018221 Pfam:PF00759 PROSITE:PS00592 PROSITE:PS00698 GO:GO:0016021 GO:GO:0005886 EMBL:CP002688 GenomeReviews:BA000015_GR GO:GO:0009735 GO:GO:0005794 GO:GO:0030245 GO:GO:0009826 GO:GO:0007047 Gene3D:G3DSA:1.50.10.10 SUPFAM:SSF48208 GO:GO:0048367 GO:GO:0005769 GO:GO:0042538 GO:GO:0043622 UniGene:At.23416 GO:GO:0009504 GO:GO:0030244 GO:GO:0008810 CAZy:GH9 EMBL:AB025613 HSSP:P26221 HOGENOM:HBG746347 ProtClustDB:CLSN2679649 EMBL:U37702 EMBL:AF073875 EMBL:AF074092 EMBL:AF074375 EMBL:AY037218 EMBL:BT002221 EMBL:AY086165

Tissue	Identification	# of reads (stress)	# of reads (control)	Fold Change	p-value	Description
						EMBL:AK221941 EMBL:AK222193 IPI:IPI00523242 PIR:S71215 RefSeq:NP_199783.1 UniGene:At.21098 ProteinModelPortal:Q38890 SMR:Q38890 STRING:Q38890 PRIDE:Q38890 EnsemblPlants:AT5G49720.1 GeneID:835035 KEGG:ath:AT5G49720 TAIR:At5g49720 eggNOG:NOG257354 InParanoid:Q38890 OMA:DEFIWGG PhylomeDB:Q38890 Genevestigator:Q38890 GermOnline:AT5G49720 Uniprot:Q38890
Root	Isotig01962	7	30	-2.229	3.18E-05	TAIR|locus:2157022 - symbol:GH9A1 "AT5G49720" species:3702 "Arabidopsis thaliana" [GO:0004553 "hydrolase activity, hydrolyzing O-glycosyl compounds" evidence=ISS] [GO:0009504 "cell plate" evidence=IDA] [GO:0009826 "unidimensional cell growth" evidence=IMP] [GO:0009735 "response to cytokinin stimulus" evidence=IMP] [GO:0048367 "shoot development" evidence=IMP] [GO:0030244 "cellulose biosynthetic process" evidence=IMP;TAS] [GO:0005886 "plasma membrane" evidence=IDA] [GO:0005769 "early endosome" evidence=IDA] [GO:0005794 "Golgi apparatus" evidence=IDA] [GO:0042538 "hyperosmotic salinity response" evidence=IMP] [GO:0043622 "cortical microtubule organization" evidence=IMP] [GO:0008810 "cellulase activity" evidence=ISS] InterPro:IPR001701 InterPro:IPR008928 InterPro:IPR012341 InterPro:IPR018221 Pfam:PF00759 PROSITE:PS00592 PROSITE:PS00698 GO:GO:0016021 GO:GO:0005886 EMBL:CP002688 GenomeReviews:BA000015_GR GO:GO:0009735 GO:GO:0005794 GO:GO:0030245 GO:GO:0009826 GO:GO:0007047 Gene3D:G3DSA:1.50.10.10 SUPFAM:SSF48208 GO:GO:0048367 GO:GO:0005769 GO:GO:0042538 GO:GO:0043622 UniGene:At.23416 GO:GO:0009504 GO:GO:0030244 GO:GO:0008810 CAZy:GH9 EMBL:AB025613 HSSP:P26221 HOGENOM:HBG746347 ProtClustDB:CLSN2679649 EMBL:U37702 EMBL:AF073875 EMBL:AF074092 EMBL:AF074375 EMBL:AY037218 EMBL:BT002221 EMBL:AY086165 EMBL:AK221941 EMBL:AK222193 IPI:IPI00523242 PIR:S71215 RefSeq:NP_199783.1 UniGene:At.21098 ProteinModelPortal:Q38890 SMR:Q38890 STRING:Q38890 PRIDE:Q38890 EnsemblPlants:AT5G49720.1 GeneID:835035 KEGG:ath:AT5G49720 TAIR:At5g49720 eggNOG:NOG257354 InParanoid:Q38890 OMA:DEFIWGG PhylomeDB:Q38890 Genevestigator:Q38890 GermOnline:AT5G49720 Uniprot:Q38890
Root	Isotig01964	66	24	1.330	3.85E-05	TAIR|locus:2136849 - symbol:PFK3 "AT4G26270" species:3702 "Arabidopsis thaliana" [GO:0003872 "6-phosphofructokinase activity" evidence=ISS;IDA] [GO:0005945 "6-phosphofructokinase complex" evidence=ISS] [GO:0006096 "glycolysis" evidence=ISS;IDA] [GO:0005829 "cytosol" evidence=IDA] InterPro:IPR000023 InterPro:IPR012004 Pfam:PF00365 PIRSF:PIRSF000534 GO:GO:0005524 EMBL:CP002687 GenomeReviews:CT486007_GR EMBL:AL049171 EMBL:AL161564 GO:GO:0006096 HSSP:P00512 eggNOG:COG0205 HOGENOM:HBG316947 KO:K00850 ProtClustDB:PLN02564 GO:GO:0005945 GO:GO:0003872 InterPro:IPR022953 PRINTS:PR00476 SUPFAM:SSF53784 EMBL:AY049245 EMBL:AY090267 IPI:IPI00545493 PIR:T06011 RefSeq:NP_567742.1 UniGene:At.2887 ProteinModelPortal:Q94AA4 SMR:Q94AA4 IntAct:Q94AA4 STRING:Q94AA4 PRIDE:Q94AA4 DNASU:828733 EnsemblPlants:AT4G26270.1 GeneID:828733 KEGG:ath:AT4G26270 TAIR:At4g26270 InParanoid:Q94AA4 OMA:HVEAESI PhylomeDB:Q94AA4 ArrayExpress:Q9STQ7 Genevestigator:Q94AA4 Uniprot:Q94AA4
Root	Isotig01994	25	4	2.515	9.62E-05	TAIR|locus:2028879 - symbol:SRO2 "similar to RCD one 2" species:3702 "Arabidopsis thaliana" [GO:0003950 "NAD+ ADP-ribosyltransferase activity" evidence=IEA] [GO:0008150 "biological_process" evidence=ND] InterPro:IPR012317 PROSITE:PS51059 GO:GO:0007275 EMBL:CP002684 GO:GO:0005634 GO:GO:0006950 EMBL:AC005990 EMBL:AC007945 GO:GO:0003950 InterPro:IPR022003 Pfam:PF12174 EMBL:BT022003 IPI:IPI00547820 PIR:C86369 RefSeq:NP_173769.1 UniGene:At.41541 UniGene:At.49909 ProteinModelPortal:Q9ZUD9 PRIDE:Q9ZUD9 GeneID:838965 KEGG:ath:AT1G23550 TAIR:At1g23550 eggNOG:NOG262161 HOGENOM:HBG320473 InParanoid:Q9ZUD9 OMA:THMNTHI PhylomeDB:Q9ZUD9 ProtClustDB:CLSN2682095 Genevestigator:Q9ZUD9 Uniprot:Q9ZUD9
Root	Isotig01995	22	3	2.745	0.000124336	TAIR|locus:2028879 - symbol:SRO2 "similar to RCD one 2" species:3702 "Arabidopsis thaliana" [GO:0003950 "NAD+ ADP-ribosyltransferase activity" evidence=IEA] [GO:0008150 "biological_process" evidence=ND] InterPro:IPR012317 PROSITE:PS51059 GO:GO:0007275 EMBL:CP002684 GO:GO:0005634 GO:GO:0006950 EMBL:AC005990 EMBL:AC007945 GO:GO:0003950 InterPro:IPR022003 Pfam:PF12174 EMBL:BT022003 IPI:IPI00547820 PIR:C86369 RefSeq:NP_173769.1 UniGene:At.41541 UniGene:At.49909 ProteinModelPortal:Q9ZUD9 PRIDE:Q9ZUD9 GeneID:838965 KEGG:ath:AT1G23550 TAIR:At1g23550 eggNOG:NOG262161 HOGENOM:HBG320473 InParanoid:Q9ZUD9 OMA:THMNTHI PhylomeDB:Q9ZUD9 ProtClustDB:CLSN2682095 Genevestigator:Q9ZUD9 Uniprot:Q9ZUD9
Root	Isotig01996	25	4	2.515	9.62E-05	TAIR|locus:2028879 - symbol:SRO2 "similar to RCD one 2" species:3702 "Arabidopsis thaliana" [GO:0003950 "NAD+ ADP-ribosyltransferase activity" evidence=IEA] [GO:0008150 "biological_process" evidence=ND] InterPro:IPR012317 PROSITE:PS51059 GO:GO:0007275 EMBL:CP002684 GO:GO:0005634 GO:GO:0006950

Tissue	Identification	# of reads (stress)	# of reads (control)	Fold Change	p-value	Description
						EMBL:AC005990 EMBL:AC007945 GO:GO:0003950 InterPro:IPR022003 Pfam:PF12174 EMBL:BT022003 IPI:IPI00547820 PIR:C86369 RefSeq:NP_173769.1 UniGene:At.41541 UniGene:At.49909 ProteinModelPortal:Q9ZUD9 PRIDE:Q9ZUD9 GeneID:838965 KEGG:ath:AT1G23550 TAIR:At1g23550 eggNOG:NOG262161 HOGENOM:HBG320473 InParanoid:Q9ZUD9 OMA:THMNTHI PhylomeDB:Q9ZUD9 ProtClustDB:CLSN2682095 Genevestigator:Q9ZUD9 Uniprot:Q9ZUD9
Root	Isotig01997	22	3	2.745	0.000124336	TAIR|locus:2028879 - symbol:SRO2 "similar to RCD one 2" species:3702 "Arabidopsis thaliana" [GO:0003950 "NAD+ ADP-ribosyltransferase activity" evidence=IEA] [GO:0008150 "biological_process" evidence=ND] InterPro:IPR012317 PROSITE:PS51059 GO:GO:0007275 EMBL:CP002684 GO:GO:0005634 GO:GO:0006950 EMBL:AC005990 EMBL:AC007945 GO:GO:0003950 InterPro:IPR022003 Pfam:PF12174 EMBL:BT022003 IPI:IPI00547820 PIR:C86369 RefSeq:NP_173769.1 UniGene:At.41541 UniGene:At.49909 ProteinModelPortal:Q9ZUD9 PRIDE:Q9ZUD9 GeneID:838965 KEGG:ath:AT1G23550 TAIR:At1g23550 eggNOG:NOG262161 HOGENOM:HBG320473 InParanoid:Q9ZUD9 OMA:THMNTHI PhylomeDB:Q9ZUD9 ProtClustDB:CLSN2682095 Genevestigator:Q9ZUD9 Uniprot:Q9ZUD9
Root	Isotig02003	0	13	-4.829	0.000155955	TAIR|locus:2014912 - symbol:WAK2 "wall-associated kinase 2" species:3702 "Arabidopsis thaliana" [GO:0004672 "protein kinase activity" evidence=IEA] [GO:0004674 "protein serine/threonine kinase activity" evidence=IEA;ISS] [GO:0005509 "calcium ion binding" evidence=IEA] [GO:0005524 "ATP binding" evidence=IEA] [GO:0006468 "protein phosphorylation" evidence=ISS] [GO:0005886 "plasma membrane" evidence=IDA] [GO:0009751 "response to salicylic acid stimulus" evidence=IDA] [GO:0009311 "oligosaccharide metabolic process" evidence=IMP] [GO:0009826 "unidimensional cell growth" evidence=IMP] [GO:0009992 "cellular water homeostasis" evidence=IMP;IDA] InterPro:IPR000719 InterPro:IPR000742 InterPro:IPR001881 InterPro:IPR006210 InterPro:IPR008271 InterPro:IPR011009 InterPro:IPR018097 InterPro:IPR025287 Pfam:PF00069 Pfam:PF07645 Pfam:PF13947 PROSITE:PS00107 PROSITE:PS00108 PROSITE:PS01187 PROSITE:PS50011 PROSITE:PS50026 SMART:SM00179 SMART:SM00181 InterPro:IPR000152 GO:GO:0016021 EMBL:CP002684 GenomeReviews:CT485782_GR GO:GO:0005524 GO:GO:0005509 eggNOG:COG0515 SUPFAM:SSF56112 GO:GO:0004674 HOGENOM:HBG755340 GO:GO:0009751 InterPro:IPR013032 PROSITE:PS00022 InterPro:IPR013320 Gene3D:G3DSA:2.60.120.200 GO:GO:0009826 PROSITE:PS01186 GO:GO:0009311 PROSITE:PS00010 GO:GO:0009992 HSSP:P35555 EMBL:AC036104 UniGene:At.23880 ProtClustDB:CLSN2679473 EMBL:AJ012423 EMBL:AF083722 EMBL:AY062531 EMBL:BT010335 IPI:IPI00532952 PIR:T52588 RefSeq:NP_173549.1 UniGene:At.21118 UniGene:At.69963 ProteinModelPortal:Q9LMP1 SMR:Q9LMP1 PRIDE:Q9LMP1 EnsemblPlants:AT1G21270.1 GeneID:838723 KEGG:ath:AT1G21270 GeneFarm:188 TAIR:At1g21270 InParanoid:Q9LMP1 OMA:SCFASAT PhylomeDB:Q9LMP1 Genevestigator:Q9LMP1 GermOnline:AT1G21270 Uniprot:Q9LMP1
Root	Isotig02004	0	21	-5.521	1.09E-06	TAIR|locus:2014952 - symbol:WAK4 "wall associated kinase 4" species:3702 "Arabidopsis thaliana" [GO:0004672 "protein kinase activity" evidence=IEA] [GO:0004674 "protein serine/threonine kinase activity" evidence=IEA;ISS] [GO:0005509 "calcium ion binding" evidence=IEA] [GO:0005524 "ATP binding" evidence=IEA] [GO:0006468 "protein phosphorylation" evidence=ISS] [GO:0005886 "plasma membrane" evidence=IDA] [GO:0009826 "unidimensional cell growth" evidence=IMP] [GO:0048527 "lateral root development" evidence=IMP] InterPro:IPR000719 InterPro:IPR000742 InterPro:IPR001245 InterPro:IPR001881 InterPro:IPR006210 InterPro:IPR008271 InterPro:IPR011009 InterPro:IPR018097 InterPro:IPR025287 Pfam:PF07645 Pfam:PF07714 Pfam:PF13947 PROSITE:PS00107 PROSITE:PS00108 PROSITE:PS01187 PROSITE:PS50011 PROSITE:PS50026 SMART:SM00179 SMART:SM00181 InterPro:IPR000152 GO:GO:0016021 EMBL:CP002684 GenomeReviews:CT485782_GR GO:GO:0005524 GO:GO:0005509 eggNOG:COG0515 SUPFAM:SSF56112 GO:GO:0004674 HOGENOM:HBG755340 GO:GO:0048527 PROSITE:PS00022 InterPro:IPR013320 Gene3D:G3DSA:2.60.120.200 GO:GO:0009826 PROSITE:PS01186 PROSITE:PS00010 HSSP:P01130 EMBL:AC036104 ProtClustDB:CLSN2679473 EMBL:AJ009695 IPI:IPI00540619 PIR:D86345 RefSeq:NP_173544.1 UniGene:At.51702 ProteinModelPortal:Q9LMN6 SMR:Q9LMN6 PRIDE:Q9LMN6 EnsemblPlants:AT1G21210.1 GeneID:838716 KEGG:ath:AT1G21210 GeneFarm:177 TAIR:At1g21210 InParanoid:Q9LMN6 OMA:HEANGEC PhylomeDB:Q9LMN6 Genevestigator:Q9LMN6 GermOnline:AT1G21210 Uniprot:Q9LMN6
Root	Isotig02005	1	15	-4.036	9.83E-05	TAIR|locus:2014952 - symbol:WAK4 "wall associated kinase 4" species:3702 "Arabidopsis thaliana" [GO:0004672 "protein kinase activity" evidence=IEA] [GO:0004674 "protein serine/threonine kinase activity" evidence=IEA;ISS] [GO:0005509 "calcium ion binding" evidence=IEA] [GO:0005524 "ATP binding" evidence=IEA] [GO:0006468 "protein phosphorylation" evidence=ISS] [GO:0005886 "plasma membrane" evidence=IDA] [GO:0009826

Tissue	Identification	# of reads (stress)	# of reads (control)	Fold Change	p-value	Description
						"unidimensional cell growth" evidence=IMP] [GO:0048527 "lateral root development" evidence=IMP] InterPro:IPR000719 InterPro:IPR000742 InterPro:IPR001245 InterPro:IPR001881 InterPro:IPR006210 InterPro:IPR008271 InterPro:IPR011009 InterPro:IPR018097 InterPro:IPR025287 Pfam:PF07645 Pfam:PF07714 Pfam:PF13947 PROSITE:PS00107 PROSITE:PS00108 PROSITE:PS01187 PROSITE:PS50011 PROSITE:PS50026 SMART:SM00179 SMART:SM00181 InterPro:IPR000152 GO:GO:0016021 EMBL:CP002684 GenomeReviews:CT485782_GR GO:GO:0005524 GO:GO:0005509 eggNOG:COG0515 SUPFAM:SSF56112 GO:GO:0004674 HOGENOM:HBG755340 GO:GO:0048527 PROSITE:PS00022 InterPro:IPR013320 Gene3D:G3DSA:2.60.120.200 GO:GO:0009826 PROSITE:PS01186 PROSITE:PS00010 HSSP:P01130 EMBL:AC036104 ProtClustDB:CLSN2679473 EMBL:AJ009695 IPI:IPI00540619 PIR:D86345 RefSeq:NP_173544.1 UniGene:At.51702 ProteinModelPortal:Q9LMN6 SMR:Q9LMN6 PRIDE:Q9LMN6 EnsemblPlants:AT1G21210.1 GeneID:838716 KEGG:ath:AT1G21210 GeneFarm:177 TAIR:At1g21210 InParanoid:Q9LMN6 OMA:HEANGEC PhylomeDB:Q9LMN6 Genevestigator:Q9LMN6 GermOnline:AT1G21210 Uniprot:Q9LMN6
Root	Isotig02006	0	11	-4.588	0.000569522	TAIR|locus:2014952 - symbol:WAK4 "wall associated kinase 4" species:3702 "Arabidopsis thaliana" [GO:0004672 "protein kinase activity" evidence=IEA] [GO:0004674 "protein serine/threonine kinase activity" evidence=IEA;ISS] [GO:0005509 "calcium ion binding" evidence=IEA] [GO:0005524 "ATP binding" evidence=IEA] [GO:0006468 "protein phosphorylation" evidence=ISS] [GO:0005886 "plasma membrane" evidence=IDA] [GO:0009826 "unidimensional cell growth" evidence=IMP] [GO:0048527 "lateral root development" evidence=IMP] InterPro:IPR000719 InterPro:IPR000742 InterPro:IPR001245 InterPro:IPR001881 InterPro:IPR006210 InterPro:IPR008271 InterPro:IPR011009 InterPro:IPR018097 InterPro:IPR025287 Pfam:PF07645 Pfam:PF07714 Pfam:PF13947 PROSITE:PS00107 PROSITE:PS00108 PROSITE:PS01187 PROSITE:PS50011 PROSITE:PS50026 SMART:SM00179 SMART:SM00181 InterPro:IPR000152 GO:GO:0016021 EMBL:CP002684 GenomeReviews:CT485782_GR GO:GO:0005524 GO:GO:0005509 eggNOG:COG0515 SUPFAM:SSF56112 GO:GO:0004674 HOGENOM:HBG755340 GO:GO:0048527 PROSITE:PS00022 InterPro:IPR013320 Gene3D:G3DSA:2.60.120.200 GO:GO:0009826 PROSITE:PS01186 PROSITE:PS00010 HSSP:P01130 EMBL:AC036104 ProtClustDB:CLSN2679473 EMBL:AJ009695 IPI:IPI00540619 PIR:D86345 RefSeq:NP_173544.1 UniGene:At.51702 ProteinModelPortal:Q9LMN6 SMR:Q9LMN6 PRIDE:Q9LMN6 EnsemblPlants:AT1G21210.1 GeneID:838716 KEGG:ath:AT1G21210 GeneFarm:177 TAIR:At1g21210 InParanoid:Q9LMN6 OMA:HEANGEC PhylomeDB:Q9LMN6 Genevestigator:Q9LMN6 GermOnline:AT1G21210 Uniprot:Q9LMN6
Root	Isotig02007	0	11	-4.588	0.000569522	TAIR|locus:2014952 - symbol:WAK4 "wall associated kinase 4" species:3702 "Arabidopsis thaliana" [GO:0004672 "protein kinase activity" evidence=IEA] [GO:0004674 "protein serine/threonine kinase activity" evidence=IEA;ISS] [GO:0005509 "calcium ion binding" evidence=IEA] [GO:0005524 "ATP binding" evidence=IEA] [GO:0006468 "protein phosphorylation" evidence=ISS] [GO:0005886 "plasma membrane" evidence=IDA] [GO:0009826 "unidimensional cell growth" evidence=IMP] [GO:0048527 "lateral root development" evidence=IMP] InterPro:IPR000719 InterPro:IPR000742 InterPro:IPR001245 InterPro:IPR001881 InterPro:IPR006210 InterPro:IPR008271 InterPro:IPR011009 InterPro:IPR018097 InterPro:IPR025287 Pfam:PF07645 Pfam:PF07714 Pfam:PF13947 PROSITE:PS00107 PROSITE:PS00108 PROSITE:PS01187 PROSITE:PS50011 PROSITE:PS50026 SMART:SM00179 SMART:SM00181 InterPro:IPR000152 GO:GO:0016021 EMBL:CP002684 GenomeReviews:CT485782_GR GO:GO:0005524 GO:GO:0005509 eggNOG:COG0515 SUPFAM:SSF56112 GO:GO:0004674 HOGENOM:HBG755340 GO:GO:0048527 PROSITE:PS00022 InterPro:IPR013320 Gene3D:G3DSA:2.60.120.200 GO:GO:0009826 PROSITE:PS01186 PROSITE:PS00010 HSSP:P01130 EMBL:AC036104 ProtClustDB:CLSN2679473 EMBL:AJ009695 IPI:IPI00540619 PIR:D86345 RefSeq:NP_173544.1 UniGene:At.51702 ProteinModelPortal:Q9LMN6 SMR:Q9LMN6 PRIDE:Q9LMN6 EnsemblPlants:AT1G21210.1 GeneID:838716 KEGG:ath:AT1G21210 GeneFarm:177 TAIR:At1g21210 InParanoid:Q9LMN6 OMA:HEANGEC PhylomeDB:Q9LMN6 Genevestigator:Q9LMN6 GermOnline:AT1G21210 Uniprot:Q9LMN6
Root	Isotig02043	8	49	-2.744	2.14E-09	TAIR|locus:2037533 - symbol:AT1G78880 "AT1G78880" species:3702 "Arabidopsis thaliana" [GO:0003674 "molecular_function" evidence=ND] [GO:0008150 "biological_process" evidence=ND] [GO:0005886 "plasma membrane" evidence=IDA] EMBL:CP002684 GenomeReviews:CT485782_GR GO:GO:0005886 GO:GO:0006508 EMBL:AC005679 GO:GO:0008233 HOGENOM:HBG318540 ProtClustDB:CLSN2679796 EMBL:AY072176 EMBL:AY096686 EMBL:AY120734 EMBL:BT000364 IPI:IPI00537430 PIR:C96818 RefSeq:NP_178009.1 UniGene:At.21193 PRIDE:Q9ZVA7 EnsemblPlants:AT1G78880.1 GeneID:844225 KEGG:ath:AT1G78880 TAIR:At1g78880 eggNOG:NOG292696 InParanoid:Q9ZVA7 OMA:WNISCER PhylomeDB:Q9ZVA7 ArrayExpress:Q9ZVA7 Genevestigator:Q9ZVA7 Uniprot:Q9ZVA7

Tissue	Identification	# of reads (stress)	# of reads (control)	Fold Change	p-value	Description
Root	Isotig02045	6	46	-3.068	8.71E-10	No hit
Root	Isotig02078	19	0	5.119	1.05E-05	TAIR|locus:2088404 - symbol:AT3G26840 "AT3G26840" species:3702 "Arabidopsis thaliana" [GO:0003824 "catalytic activity" evidence=ISS] [GO:0008152 "metabolic process" evidence=IEA] [GO:0016746 "transferase activity, transferring acyl groups" evidence=IEA] [GO:0016747 "transferase activity, transferring acyl groups other than amino-acyl groups" evidence=IEA] [GO:0010287 "plastoglobule" evidence=IDA] InterPro:IPR002123 InterPro:IPR007130 Pfam:PF03982 SMART:SM00563 EMBL:CP002686 GenomeReviews:BA000014_GR GO:GO:0016747 EMBL:AB016889 GO:GO:0010287 eggNOG:COG0204 HOGENOM:HBG562431 EMBL:AY056370 EMBL:AF360145 IPI:IPI00541496 RefSeq:NP_566801.1 UniGene:At.6034 UniGene:At.66477 ProteinModelPortal:Q9LW26 SMR:Q9LW26 PRIDE:Q9LW26 EnsemblPlants:AT3G26840.1 GeneID:822299 KEGG:ath:AT3G26840 TAIR:At3g26840 InParanoid:Q9LW26 OMA:DDICEIV PhylomeDB:Q9LW26 ProtClustDB:CLSN2684876 ArrayExpress:Q9LW26 Genevestigator:Q9LW26 Uniprot:Q9LW26
Root	Isotig02079	19	0	5.119	1.05E-05	TAIR|locus:2088404 - symbol:AT3G26840 "AT3G26840" species:3702 "Arabidopsis thaliana" [GO:0003824 "catalytic activity" evidence=ISS] [GO:0008152 "metabolic process" evidence=IEA] [GO:0016746 "transferase activity, transferring acyl groups" evidence=IEA] [GO:0016747 "transferase activity, transferring acyl groups other than amino-acyl groups" evidence=IEA] [GO:0010287 "plastoglobule" evidence=IDA] InterPro:IPR002123 InterPro:IPR007130 Pfam:PF03982 SMART:SM00563 EMBL:CP002686 GenomeReviews:BA000014_GR GO:GO:0016747 EMBL:AB016889 GO:GO:0010287 eggNOG:COG0204 HOGENOM:HBG562431 EMBL:AY056370 EMBL:AF360145 IPI:IPI00541496 RefSeq:NP_566801.1 UniGene:At.6034 UniGene:At.66477 ProteinModelPortal:Q9LW26 SMR:Q9LW26 PRIDE:Q9LW26 EnsemblPlants:AT3G26840.1 GeneID:822299 KEGG:ath:AT3G26840 TAIR:At3g26840 InParanoid:Q9LW26 OMA:DDICEIV PhylomeDB:Q9LW26 ProtClustDB:CLSN2684876 ArrayExpress:Q9LW26 Genevestigator:Q9LW26 Uniprot:Q9LW26
Root	Isotig02082	23	60	-1.512	6.18E-06	TAIR|locus:2102926 - symbol:AT3G30390 "AT3G30390" species:3702 "Arabidopsis thaliana" [GO:0006865 "amino acid transport" evidence=ISS] [GO:0015171 "amino acid transmembrane transporter activity" evidence=ISS] [GO:0016020 "membrane" evidence=ISS] [GO:0005773 "vacuole" evidence=IDA] [GO:0005774 "vacuolar membrane" evidence=IDA] GO:GO:0016021 GO:GO:0005774 EMBL:CP002686 InterPro:IPR013057 Pfam:PF01490 HOGENOM:HBG601878 EMBL:AY052201 EMBL:AF428287 EMBL:AP001314 IPI:IPI00520585 RefSeq:NP_001030795.1 RefSeq:NP_566854.1 UniGene:At.21575 ProteinModelPortal:Q9LI61 STRING:Q9LI61 PRIDE:Q9LI61 EnsemblPlants:AT3G30390.1 EnsemblPlants:AT3G30390.2 GeneID:822740 KEGG:ath:AT3G30390 TAIR:At3g30390 InParanoid:Q9LI61 OMA:AFICHYN PhylomeDB:Q9LI61 ProtClustDB:CLSN2687270 Genevestigator:Q9LI61 Uniprot:Q9LI61
Root	Isotig02083	23	59	-1.488	9.46E-06	TAIR|locus:2102926 - symbol:AT3G30390 "AT3G30390" species:3702 "Arabidopsis thaliana" [GO:0006865 "amino acid transport" evidence=ISS] [GO:0015171 "amino acid transmembrane transporter activity" evidence=ISS] [GO:0016020 "membrane" evidence=ISS] [GO:0005773 "vacuole" evidence=IDA] [GO:0005774 "vacuolar membrane" evidence=IDA] GO:GO:0016021 GO:GO:0005774 EMBL:CP002686 InterPro:IPR013057 Pfam:PF01490 HOGENOM:HBG601878 EMBL:AY052201 EMBL:AF428287 EMBL:AP001314 IPI:IPI00520585 RefSeq:NP_001030795.1 RefSeq:NP_566854.1 UniGene:At.21575 ProteinModelPortal:Q9LI61 STRING:Q9LI61 PRIDE:Q9LI61 EnsemblPlants:AT3G30390.1 EnsemblPlants:AT3G30390.2 GeneID:822740 KEGG:ath:AT3G30390 TAIR:At3g30390 InParanoid:Q9LI61 OMA:AFICHYN PhylomeDB:Q9LI61 ProtClustDB:CLSN2687270 Genevestigator:Q9LI61 Uniprot:Q9LI61
Root	Isotig02094	4	29	-2.987	1.53E-06	TAIR|locus:2198928 - symbol:NHO1 "AT1G80460" species:3702 "Arabidopsis thaliana" [GO:0005975 "carbohydrate metabolic process" evidence=ISS] [GO:0019200 "carbohydrate kinase activity" evidence=ISS] [GO:0004370 "glycerol kinase activity" evidence=ISS;IDA] [GO:0009617 "response to bacterium" evidence=IEP] [GO:0019563 "glycerol catabolic process" evidence=IMP] [GO:0002237 "response to molecule of bacterial origin" evidence=IEP] [GO:0010188 "response to microbial phytotoxin" evidence=IEP] [GO:0080167 "response to karrikin" evidence=IEP] [GO:0005829 "cytosol" evidence=IDA] InterPro:IPR000577 InterPro:IPR005999 InterPro:IPR018483 InterPro:IPR018484 InterPro:IPR018485 Pfam:PF00370 Pfam:PF02782 PROSITE:PS00933 EMBL:CP002684 GO:GO:0005829 GO:GO:0080167 GO:GO:0002237 PANTHER:PTHR10196 EMBL:AC018849 GO:GO:0010188 GO:GO:0019563 GO:GO:0006072 KO:K00864 GO:GO:0004370 PANTHER:PTHR10196:SF9 TIGRFAMs:TIGR01311 HSSP:P08859 OMA:DSGVRLE EMBL:AY234854 EMBL:AK316784 IPI:IPI00529626 PIR:D96836 RefSeq:NP_178161.1 UniGene:At.24313 UniGene:At.67688 ProteinModelPortal:Q9M8L4 SMR:Q9M8L4 STRING:Q9M8L4 PRIDE:Q9M8L4 EnsemblPlants:AT1G80460.1 GeneID:844385

Tissue	Identification	# of reads (stress)	# of reads (control)	Fold Change	p-value	Description
						KEGG:ath:AT1G80460 TAIR:At1g80460 InParanoid:Q9M8L4 PhylomeDB:Q9M8L4 ProtClustDB:PLN02295 Genevestigator:Q9M8L4 Uniprot:Q9M8L4
Root	Isotig02095	4	25	-2.773	1.71E-05	TAIR|locus:2198928 - symbol:NHO1 "AT1G80460" species:3702 "Arabidopsis thaliana" [GO:0005975 "carbohydrate metabolic process" evidence=ISS] [GO:0019200 "carbohydrate kinase activity" evidence=ISS] [GO:0004370 "glycerol kinase activity" evidence=ISS;IDA] [GO:0009617 "response to bacterium" evidence=IEP] [GO:0019563 "glycerol catabolic process" evidence=IMP] [GO:0002237 "response to molecule of bacterial origin" evidence=IEP] [GO:0010188 "response to microbial phytotoxin" evidence=IEP] [GO:0080167 "response to karrikin" evidence=IEP] [GO:0005829 "cytosol" evidence=IDA] InterPro:IPR000577 InterPro:IPR005999 InterPro:IPR018483 InterPro:IPR018484 InterPro:IPR018485 Pfam:PF00370 Pfam:PF02782 PROSITE:PS00933 EMBL:CP002684 GO:GO:0005829 GO:GO:0080167 GO:GO:0002237 PANTHER:PTHR10196 EMBL:AC018849 GO:GO:0010188 GO:GO:0019563 GO:GO:0006072 KO:K00864 GO:GO:0004370 PANTHER:PTHR10196:SF9 TIGRFAMs:TIGR01311 HSSP:P08859 OMA:DSGVRLE EMBL:AY234854 EMBL:AK316784 IPI:IPI00529626 PIR:D96836 RefSeq:NP_178161.1 UniGene:At.24313 UniGene:At.67688 ProteinModelPortal:Q9M8L4 SMR:Q9M8L4 STRING:Q9M8L4 PRIDE:Q9M8L4 EnsemblPlants:AT1G80460.1 GeneID:844385 KEGG:ath:AT1G80460 TAIR:At1g80460 InParanoid:Q9M8L4 PhylomeDB:Q9M8L4 ProtClustDB:PLN02295 Genevestigator:Q9M8L4 Uniprot:Q9M8L4
Root	Isotig02096	3	28	-3.351	6.51E-07	TAIR|locus:2198928 - symbol:NHO1 "AT1G80460" species:3702 "Arabidopsis thaliana" [GO:0005975 "carbohydrate metabolic process" evidence=ISS] [GO:0019200 "carbohydrate kinase activity" evidence=ISS] [GO:0004370 "glycerol kinase activity" evidence=ISS;IDA] [GO:0009617 "response to bacterium" evidence=IEP] [GO:0019563 "glycerol catabolic process" evidence=IMP] [GO:0002237 "response to molecule of bacterial origin" evidence=IEP] [GO:0010188 "response to microbial phytotoxin" evidence=IEP] [GO:0080167 "response to karrikin" evidence=IEP] [GO:0005829 "cytosol" evidence=IDA] InterPro:IPR000577 InterPro:IPR005999 InterPro:IPR018483 InterPro:IPR018484 InterPro:IPR018485 Pfam:PF00370 Pfam:PF02782 PROSITE:PS00933 EMBL:CP002684 GO:GO:0005829 GO:GO:0080167 GO:GO:0002237 PANTHER:PTHR10196 EMBL:AC018849 GO:GO:0010188 GO:GO:0019563 GO:GO:0006072 KO:K00864 GO:GO:0004370 PANTHER:PTHR10196:SF9 TIGRFAMs:TIGR01311 HSSP:P08859 OMA:DSGVRLE EMBL:AY234854 EMBL:AK316784 IPI:IPI00529626 PIR:D96836 RefSeq:NP_178161.1 UniGene:At.24313 UniGene:At.67688 ProteinModelPortal:Q9M8L4 SMR:Q9M8L4 STRING:Q9M8L4 PRIDE:Q9M8L4 EnsemblPlants:AT1G80460.1 GeneID:844385 KEGG:ath:AT1G80460 TAIR:At1g80460 InParanoid:Q9M8L4 PhylomeDB:Q9M8L4 ProtClustDB:PLN02295 Genevestigator:Q9M8L4 Uniprot:Q9M8L4
Root	Isotig02097	3	22	-3.003	2.69E-05	TAIR|locus:2198928 - symbol:NHO1 "AT1G80460" species:3702 "Arabidopsis thaliana" [GO:0005975 "carbohydrate metabolic process" evidence=ISS] [GO:0019200 "carbohydrate kinase activity" evidence=ISS] [GO:0004370 "glycerol kinase activity" evidence=ISS;IDA] [GO:0009617 "response to bacterium" evidence=IEP] [GO:0019563 "glycerol catabolic process" evidence=IMP] [GO:0002237 "response to molecule of bacterial origin" evidence=IEP] [GO:0010188 "response to microbial phytotoxin" evidence=IEP] [GO:0080167 "response to karrikin" evidence=IEP] [GO:0005829 "cytosol" evidence=IDA] InterPro:IPR000577 InterPro:IPR005999 InterPro:IPR018483 InterPro:IPR018484 InterPro:IPR018485 Pfam:PF00370 Pfam:PF02782 PROSITE:PS00933 EMBL:CP002684 GO:GO:0005829 GO:GO:0080167 GO:GO:0002237 PANTHER:PTHR10196 EMBL:AC018849 GO:GO:0010188 GO:GO:0019563 GO:GO:0006072 KO:K00864 GO:GO:0004370 PANTHER:PTHR10196:SF9 TIGRFAMs:TIGR01311 HSSP:P08859 OMA:DSGVRLE EMBL:AY234854 EMBL:AK316784 IPI:IPI00529626 PIR:D96836 RefSeq:NP_178161.1 UniGene:At.24313 UniGene:At.67688 ProteinModelPortal:Q9M8L4 SMR:Q9M8L4 STRING:Q9M8L4 PRIDE:Q9M8L4 EnsemblPlants:AT1G80460.1 GeneID:844385 KEGG:ath:AT1G80460 TAIR:At1g80460 InParanoid:Q9M8L4 PhylomeDB:Q9M8L4 ProtClustDB:PLN02295 Genevestigator:Q9M8L4 Uniprot:Q9M8L4
Root	Isotig02102	1	17	-4.216	2.69E-05	TAIR|locus:2077572 - symbol:RPM1 "AT3G07040" species:3702 "Arabidopsis thaliana" [GO:0006952 "defense response" evidence=ISS;TAS] [GO:0005515 "protein binding" evidence=IPI] [GO:0005886 "plasma membrane" evidence=IDA] [GO:0000166 "nucleotide binding" evidence=ISS] [GO:0009626 "plant-type hypersensitive response" evidence=IDA] Pfam:PF00560 InterPro:IPR001611 InterPro:IPR000767 InterPro:IPR002182 Pfam:PF00931 PRINTS:PR00364 GO:GO:0005886 GO:GO:0005524 EMBL:CP002686 GenomeReviews:BA000014_GR GO:GO:0005515 eggNOG:COG4886 GO:GO:0012505 GO:GO:0043531 GO:GO:0009626 EMBL:AC016827 HOGENOM:HBG749068 EMBL:X87851 IPI:IPI00526839 PIR:A57072 RefSeq:NP_187360.1 UniGene:At.40415 ProteinModelPortal:Q39214 SMR:Q39214 STRING:Q39214 PRIDE:Q39214 EnsemblPlants:AT3G07040.1

Tissue	Identification	# of reads (stress)	# of reads (control)	Fold Change	p-value	Description
						GeneID:819889 KEGG:ath:AT3G07040 TAIR:At3g07040 InParanoid:Q39214 KO:K13457 OMA:WREISIA PhylomeDB:Q39214 ProtClustDB:CLSN2915520 ArrayExpress:Q39214 Genevestigator:Q39214 Uniprot:Q39214
Root	Isotig02103	0	17	-5.216	1.26E-05	TAIR|locus:2077572 - symbol:RPM1 "AT3G07040" species:3702 "Arabidopsis thaliana" [GO:0006952 "defense response" evidence=ISS;TAS] [GO:0005515 "protein binding" evidence=IPI] [GO:0005886 "plasma membrane" evidence=IDA] [GO:0000166 "nucleotide binding" evidence=ISS] [GO:0009626 "plant-type hypersensitive response" evidence=IDA] Pfam:PF00560 InterPro:IPR001611 InterPro:IPR000767 InterPro:IPR002182 Pfam:PF00931 PRINTS:PR00364 GO:GO:0005886 GO:GO:0005524 EMBL:CP002686 GenomeReviews:BA000014_GR GO:GO:0005515 eggNOG:COG4886 GO:GO:0012505 GO:GO:0043531 GO:GO:0009626 EMBL:AC016827 HOGENOM:HBG749068 EMBL:X87851 IPI:IPI00526839 PIR:A57072 RefSeq:NP_187360.1 UniGene:At.40415 ProteinModelPortal:Q39214 SMR:Q39214 STRING:Q39214 PRIDE:Q39214 EnsemblPlants:AT3G07040.1 GeneID:819889 KEGG:ath:AT3G07040 TAIR:At3g07040 InParanoid:Q39214 KO:K13457 OMA:WREISIA PhylomeDB:Q39214 ProtClustDB:CLSN2915520 ArrayExpress:Q39214 Genevestigator:Q39214 Uniprot:Q39214
Root	Isotig02145	47	0	6.426	3.99E-12	TAIR|locus:2201041 - symbol:AT1G05510 "AT1G05510" species:3702 "Arabidopsis thaliana" [GO:0003674 "molecular_function" evidence=ND] [GO:0005575 "cellular_component" evidence=ND] [GO:0009737 "response to abscisic acid stimulus" evidence=IEP] EMBL:CP002684 GenomeReviews:CT485782_GR GO:GO:0009737 EMBL:AC005106 UniGene:At.20860 InterPro:IPR010686 Pfam:PF06884 eggNOG:NOG05920 HOGENOM:HBG473015 ProtClustDB:CLSN2682017 EMBL:AY070405 EMBL:AY096580 EMBL:AK226452 IPI:IPI00527396 RefSeq:NP_172042.1 UniGene:At.28368 IntAct:Q9ZVY7 PRIDE:Q9ZVY7 ProMEX:Q9ZVY7 DNASU:837053 EnsemblPlants:AT1G05510.1 GeneID:837053 KEGG:ath:AT1G05510 TAIR:At1g05510 InParanoid:Q9ZVY7 OMA:CGHINED PhylomeDB:Q9ZVY7 ArrayExpress:Q9ZVY7 Genevestigator:Q9ZVY7 Uniprot:Q9ZVY7
Root	Isotig02146	47	0	6.426	3.99E-12	TAIR|locus:2201041 - symbol:AT1G05510 "AT1G05510" species:3702 "Arabidopsis thaliana" [GO:0003674 "molecular_function" evidence=ND] [GO:0005575 "cellular_component" evidence=ND] [GO:0009737 "response to abscisic acid stimulus" evidence=IEP] EMBL:CP002684 GenomeReviews:CT485782_GR GO:GO:0009737 EMBL:AC005106 UniGene:At.20860 InterPro:IPR010686 Pfam:PF06884 eggNOG:NOG05920 HOGENOM:HBG473015 ProtClustDB:CLSN2682017 EMBL:AY070405 EMBL:AY096580 EMBL:AK226452 IPI:IPI00527396 RefSeq:NP_172042.1 UniGene:At.28368 IntAct:Q9ZVY7 PRIDE:Q9ZVY7 ProMEX:Q9ZVY7 DNASU:837053 EnsemblPlants:AT1G05510.1 GeneID:837053 KEGG:ath:AT1G05510 TAIR:At1g05510 InParanoid:Q9ZVY7 OMA:CGHINED PhylomeDB:Q9ZVY7 ArrayExpress:Q9ZVY7 Genevestigator:Q9ZVY7 Uniprot:Q9ZVY7
Root	Isotig02147	47	0	6.426	3.99E-12	TAIR|locus:2201041 - symbol:AT1G05510 "AT1G05510" species:3702 "Arabidopsis thaliana" [GO:0003674 "molecular_function" evidence=ND] [GO:0005575 "cellular_component" evidence=ND] [GO:0009737 "response to abscisic acid stimulus" evidence=IEP] EMBL:CP002684 GenomeReviews:CT485782_GR GO:GO:0009737 EMBL:AC005106 UniGene:At.20860 InterPro:IPR010686 Pfam:PF06884 eggNOG:NOG05920 HOGENOM:HBG473015 ProtClustDB:CLSN2682017 EMBL:AY070405 EMBL:AY096580 EMBL:AK226452 IPI:IPI00527396 RefSeq:NP_172042.1 UniGene:At.28368 IntAct:Q9ZVY7 PRIDE:Q9ZVY7 ProMEX:Q9ZVY7 DNASU:837053 EnsemblPlants:AT1G05510.1 GeneID:837053 KEGG:ath:AT1G05510 TAIR:At1g05510 InParanoid:Q9ZVY7 OMA:CGHINED PhylomeDB:Q9ZVY7 ArrayExpress:Q9ZVY7 Genevestigator:Q9ZVY7 Uniprot:Q9ZVY7
Root	Isotig02148	47	0	6.426	3.99E-12	TAIR|locus:2201041 - symbol:AT1G05510 "AT1G05510" species:3702 "Arabidopsis thaliana" [GO:0003674 "molecular_function" evidence=ND] [GO:0005575 "cellular_component" evidence=ND] [GO:0009737 "response to abscisic acid stimulus" evidence=IEP] EMBL:CP002684 GenomeReviews:CT485782_GR GO:GO:0009737 EMBL:AC005106 UniGene:At.20860 InterPro:IPR010686 Pfam:PF06884 eggNOG:NOG05920 HOGENOM:HBG473015 ProtClustDB:CLSN2682017 EMBL:AY070405 EMBL:AY096580 EMBL:AK226452 IPI:IPI00527396 RefSeq:NP_172042.1 UniGene:At.28368 IntAct:Q9ZVY7 PRIDE:Q9ZVY7 ProMEX:Q9ZVY7 DNASU:837053 EnsemblPlants:AT1G05510.1 GeneID:837053 KEGG:ath:AT1G05510 TAIR:At1g05510 InParanoid:Q9ZVY7 OMA:CGHINED PhylomeDB:Q9ZVY7 ArrayExpress:Q9ZVY7 Genevestigator:Q9ZVY7 Uniprot:Q9ZVY7
Root	Isotig02149	47	0	6.426	3.99E-12	TAIR|locus:2201041 - symbol:AT1G05510 "AT1G05510" species:3702 "Arabidopsis thaliana" [GO:0003674 "molecular_function" evidence=ND] [GO:0005575 "cellular_component" evidence=ND] [GO:0009737 "response to abscisic acid stimulus" evidence=IEP] EMBL:CP002684 GenomeReviews:CT485782_GR GO:GO:0009737 EMBL:AC005106 UniGene:At.20860 InterPro:IPR010686 Pfam:PF06884 eggNOG:NOG05920

Tissue	Identification	# of reads (stress)	# of reads (control)	Fold Change	p-value	Description
						HOGENOM:HBG473015 ProtClustDB:CLSN2682017 EMBL:AY070405 EMBL:AY096580 EMBL:AK226452 IPI:IPI00527396 RefSeq:NP_172042.1 UniGene:At.28368 IntAct:Q9ZVY7 PRIDE:Q9ZVY7 ProMEX:Q9ZVY7 DNASU:837053 EnsemblPlants:AT1G05510.1 GeneID:837053 KEGG:ath:AT1G05510 TAIR:At1g05510 InParanoid:Q9ZVY7 OMA:CGHINED PhylomeDB:Q9ZVY7 ArrayExpress:Q9ZVY7 Genevestigator:Q9ZVY7 Uniprot:Q9ZVY7
Root	Isotig02150	47	0	6.426	3.99E-12	TAIR|locus:2201041 - symbol:AT1G05510 "AT1G05510" species:3702 "Arabidopsis thaliana" [GO:0003674 "molecular_function" evidence=ND] [GO:0005575 "cellular_component" evidence=ND] [GO:0009737 "response to abscisic acid stimulus" evidence=IEP] EMBL:CP002684 GenomeReviews:CT485782_GR GO:GO:0009737 EMBL:AC005106 UniGene:At.20860 InterPro:IPR010686 Pfam:PF06884 eggNOG:NOG05920 HOGENOM:HBG473015 ProtClustDB:CLSN2682017 EMBL:AY070405 EMBL:AY096580 EMBL:AK226452 IPI:IPI00527396 RefSeq:NP_172042.1 UniGene:At.28368 IntAct:Q9ZVY7 PRIDE:Q9ZVY7 ProMEX:Q9ZVY7 DNASU:837053 EnsemblPlants:AT1G05510.1 GeneID:837053 KEGG:ath:AT1G05510 TAIR:At1g05510 InParanoid:Q9ZVY7 OMA:CGHINED PhylomeDB:Q9ZVY7 ArrayExpress:Q9ZVY7 Genevestigator:Q9ZVY7 Uniprot:Q9ZVY7
Root	Isotig02151	47	0	6.426	3.99E-12	TAIR|locus:2201041 - symbol:AT1G05510 "AT1G05510" species:3702 "Arabidopsis thaliana" [GO:0003674 "molecular_function" evidence=ND] [GO:0005575 "cellular_component" evidence=ND] [GO:0009737 "response to abscisic acid stimulus" evidence=IEP] EMBL:CP002684 GenomeReviews:CT485782_GR GO:GO:0009737 EMBL:AC005106 UniGene:At.20860 InterPro:IPR010686 Pfam:PF06884 eggNOG:NOG05920 HOGENOM:HBG473015 ProtClustDB:CLSN2682017 EMBL:AY070405 EMBL:AY096580 EMBL:AK226452 IPI:IPI00527396 RefSeq:NP_172042.1 UniGene:At.28368 IntAct:Q9ZVY7 PRIDE:Q9ZVY7 ProMEX:Q9ZVY7 DNASU:837053 EnsemblPlants:AT1G05510.1 GeneID:837053 KEGG:ath:AT1G05510 TAIR:At1g05510 InParanoid:Q9ZVY7 OMA:CGHINED PhylomeDB:Q9ZVY7 ArrayExpress:Q9ZVY7 Genevestigator:Q9ZVY7 Uniprot:Q9ZVY7
Root	Isotig02152	47	0	6.426	3.99E-12	TAIR|locus:2201041 - symbol:AT1G05510 "AT1G05510" species:3702 "Arabidopsis thaliana" [GO:0003674 "molecular_function" evidence=ND] [GO:0005575 "cellular_component" evidence=ND] [GO:0009737 "response to abscisic acid stimulus" evidence=IEP] EMBL:CP002684 GenomeReviews:CT485782_GR GO:GO:0009737 EMBL:AC005106 UniGene:At.20860 InterPro:IPR010686 Pfam:PF06884 eggNOG:NOG05920 HOGENOM:HBG473015 ProtClustDB:CLSN2682017 EMBL:AY070405 EMBL:AY096580 EMBL:AK226452 IPI:IPI00527396 RefSeq:NP_172042.1 UniGene:At.28368 IntAct:Q9ZVY7 PRIDE:Q9ZVY7 ProMEX:Q9ZVY7 DNASU:837053 EnsemblPlants:AT1G05510.1 GeneID:837053 KEGG:ath:AT1G05510 TAIR:At1g05510 InParanoid:Q9ZVY7 OMA:CGHINED PhylomeDB:Q9ZVY7 ArrayExpress:Q9ZVY7 Genevestigator:Q9ZVY7 Uniprot:Q9ZVY7
Root	Isotig02153	37	104	-1.620	3.92E-10	TAIR|locus:2009600 - symbol:PPC1 "AT1G53310" species:3702 "Arabidopsis thaliana" [GO:0006099 "tricarboxylic acid cycle" evidence=ISS] [GO:0008964 "phosphoenolpyruvate carboxylase activity" evidence=ISS] [GO:0005829 "cytosol" evidence=IDA] [GO:0048046 "apoplast" evidence=IDA] [GO:0016036 "cellular response to phosphate starvation" evidence=IDA] [GO:0051262 "protein tetramerization" evidence=IDA] [GO:0005515 "protein binding" evidence=IPI] InterPro:IPR015813 InterPro:IPR018129 InterPro:IPR021135 InterPro:IPR022805 Pfam:PF00311 PRINTS:PR00150 PROSITE:PS00393 PROSITE:PS00781 EMBL:CP002684 GenomeReviews:CT485782_GR GO:GO:0005829 GO:GO:0048046 GO:GO:0005515 GO:GO:0051262 EMBL:AC008007 GO:GO:0006099 SUPFAM:SSF51621 GO:GO:0016036 GO:GO:0015979 EMBL:AJ532901 EMBL:AY057507 EMBL:BT000647 IPI:IPI00541694 PIR:D96573 RefSeq:NP_001031178.1 RefSeq:NP_001031179.1 RefSeq:NP_175738.1 UniGene:At.23221 ProteinModelPortal:Q9MAH0 SMR:Q9MAH0 IntAct:Q9MAH0 STRING:Q9MAH0 PRIDE:Q9MAH0 ProMEX:Q9MAH0 EnsemblPlants:AT1G53310.1 EnsemblPlants:AT1G53310.2 EnsemblPlants:AT1G53310.3 GeneID:841765 KEGG:ath:AT1G53310 GeneFarm:5060 TAIR:At1g53310 eggNOG:COG2352 HOGENOM:HBG351035 InParanoid:Q9MAH0 KO:K01595 OMA:HISKEIA PhylomeDB:Q9MAH0 ProtClustDB:CLSN2679806 BRENDA:4.1.1.31 ArrayExpress:Q9MAH0 Genevestigator:Q9MAH0 GermOnline:AT1G53310 GO:GO:0008964 GO:GO:0015977 Uniprot:Q9MAH0
Root	Isotig02154	34	102	-1.714	1.12E-10	TAIR|locus:2009600 - symbol:PPC1 "AT1G53310" species:3702 "Arabidopsis thaliana" [GO:0006099 "tricarboxylic acid cycle" evidence=ISS] [GO:0008964 "phosphoenolpyruvate carboxylase activity" evidence=ISS] [GO:0005829 "cytosol" evidence=IDA] [GO:0048046 "apoplast" evidence=IDA] [GO:0016036 "cellular response to phosphate starvation" evidence=IDA] [GO:0051262 "protein tetramerization" evidence=IDA] [GO:0005515 "protein binding" evidence=IPI] InterPro:IPR015813 InterPro:IPR018129 InterPro:IPR021135 InterPro:IPR022805 Pfam:PF00311

Tissue	Identification	# of reads (stress)	# of reads (control)	Fold Change	p-value	Description
						PRINTS:PR00150 PROSITE:PS00393 PROSITE:PS00781 EMBL:CP002684 GenomeReviews:CT485782_GR GO:GO:0005829 GO:GO:0048046 GO:GO:0005515 GO:GO:0051262 EMBL:AC008007 GO:GO:0006099 SUPFAM:SSF51621 GO:GO:0016036 GO:GO:0015979 EMBL:AJ532901 EMBL:AY057507 EMBL:BT000647 IPI:IPI00541694 PIR:D96573 RefSeq:NP_001031178.1 RefSeq:NP_001031179.1 RefSeq:NP_175738.1 UniGene:At.23221 ProteinModelPortal:Q9MAH0 SMR:Q9MAH0 IntAct:Q9MAH0 STRING:Q9MAH0 PRIDE:Q9MAH0 ProMEX:Q9MAH0 EnsemblPlants:AT1G53310.1 EnsemblPlants:AT1G53310.2 EnsemblPlants:AT1G53310.3 GeneID:841765 KEGG:ath:AT1G53310 GeneFarm:5060 TAIR:At1g53310 eggNOG:COG2352 HOGENOM:HBG351035 InParanoid:Q9MAH0 KO:K01595 OMA:HISKEIA PhylomeDB:Q9MAH0 ProtClustDB:CLSN2679806 BRENDA:4.1.1.31 ArrayExpress:Q9MAH0 Genevestigator:Q9MAH0 GermOnline:AT1G53310 GO:GO:0008964 GO:GO:0015977 Uniprot:Q9MAH0
Root	Isotig02155	34	102	-1.714	1.12E-10	TAIR|locus:2009600 - symbol:PPC1 "AT1G53310" species:3702 "Arabidopsis thaliana" [GO:0006099 "tricarboxylic acid cycle" evidence=ISS] [GO:0008964 "phosphoenolpyruvate carboxylase activity" evidence=ISS] [GO:0005829 "cytosol" evidence=IDA] [GO:0048046 "apoplast" evidence=IDA] [GO:0016036 "cellular response to phosphate starvation" evidence=IDA] [GO:0051262 "protein tetramerization" evidence=IDA] [GO:0005515 "protein binding" evidence=IPI] InterPro:IPR015813 InterPro:IPR018129 InterPro:IPR021135 InterPro:IPR022805 Pfam:PF00311 PRINTS:PR00150 PROSITE:PS00393 PROSITE:PS00781 EMBL:CP002684 GenomeReviews:CT485782_GR GO:GO:0005829 GO:GO:0048046 GO:GO:0005515 GO:GO:0051262 EMBL:AC008007 GO:GO:0006099 SUPFAM:SSF51621 GO:GO:0016036 GO:GO:0015979 EMBL:AJ532901 EMBL:AY057507 EMBL:BT000647 IPI:IPI00541694 PIR:D96573 RefSeq:NP_001031178.1 RefSeq:NP_001031179.1 RefSeq:NP_175738.1 UniGene:At.23221 ProteinModelPortal:Q9MAH0 SMR:Q9MAH0 IntAct:Q9MAH0 STRING:Q9MAH0 PRIDE:Q9MAH0 ProMEX:Q9MAH0 EnsemblPlants:AT1G53310.1 EnsemblPlants:AT1G53310.2 EnsemblPlants:AT1G53310.3 GeneID:841765 KEGG:ath:AT1G53310 GeneFarm:5060 TAIR:At1g53310 eggNOG:COG2352 HOGENOM:HBG351035 InParanoid:Q9MAH0 KO:K01595 OMA:HISKEIA PhylomeDB:Q9MAH0 ProtClustDB:CLSN2679806 BRENDA:4.1.1.31 ArrayExpress:Q9MAH0 Genevestigator:Q9MAH0 GermOnline:AT1G53310 GO:GO:0008964 GO:GO:0015977 Uniprot:Q9MAH0
Root	Isotig02156	37	103	-1.606	6.08E-10	TAIR|locus:2009600 - symbol:PPC1 "AT1G53310" species:3702 "Arabidopsis thaliana" [GO:0006099 "tricarboxylic acid cycle" evidence=ISS] [GO:0008964 "phosphoenolpyruvate carboxylase activity" evidence=ISS] [GO:0005829 "cytosol" evidence=IDA] [GO:0048046 "apoplast" evidence=IDA] [GO:0016036 "cellular response to phosphate starvation" evidence=IDA] [GO:0051262 "protein tetramerization" evidence=IDA] [GO:0005515 "protein binding" evidence=IPI] InterPro:IPR015813 InterPro:IPR018129 InterPro:IPR021135 InterPro:IPR022805 Pfam:PF00311 PRINTS:PR00150 PROSITE:PS00393 PROSITE:PS00781 EMBL:CP002684 GenomeReviews:CT485782_GR GO:GO:0005829 GO:GO:0048046 GO:GO:0005515 GO:GO:0051262 EMBL:AC008007 GO:GO:0006099 SUPFAM:SSF51621 GO:GO:0016036 GO:GO:0015979 EMBL:AJ532901 EMBL:AY057507 EMBL:BT000647 IPI:IPI00541694 PIR:D96573 RefSeq:NP_001031178.1 RefSeq:NP_001031179.1 RefSeq:NP_175738.1 UniGene:At.23221 ProteinModelPortal:Q9MAH0 SMR:Q9MAH0 IntAct:Q9MAH0 STRING:Q9MAH0 PRIDE:Q9MAH0 ProMEX:Q9MAH0 EnsemblPlants:AT1G53310.1 EnsemblPlants:AT1G53310.2 EnsemblPlants:AT1G53310.3 GeneID:841765 KEGG:ath:AT1G53310 GeneFarm:5060 TAIR:At1g53310 eggNOG:COG2352 HOGENOM:HBG351035 InParanoid:Q9MAH0 KO:K01595 OMA:HISKEIA PhylomeDB:Q9MAH0 ProtClustDB:CLSN2679806 BRENDA:4.1.1.31 ArrayExpress:Q9MAH0 Genevestigator:Q9MAH0 GermOnline:AT1G53310 GO:GO:0008964 GO:GO:0015977 Uniprot:Q9MAH0
Root	Isotig02157	34	101	-1.700	1.76E-10	TAIR|locus:2009600 - symbol:PPC1 "AT1G53310" species:3702 "Arabidopsis thaliana" [GO:0006099 "tricarboxylic acid cycle" evidence=ISS] [GO:0008964 "phosphoenolpyruvate carboxylase activity" evidence=ISS] [GO:0005829 "cytosol" evidence=IDA] [GO:0048046 "apoplast" evidence=IDA] [GO:0016036 "cellular response to phosphate starvation" evidence=IDA] [GO:0051262 "protein tetramerization" evidence=IDA] [GO:0005515 "protein binding" evidence=IPI] InterPro:IPR015813 InterPro:IPR018129 InterPro:IPR021135 InterPro:IPR022805 Pfam:PF00311 PRINTS:PR00150 PROSITE:PS00393 PROSITE:PS00781 EMBL:CP002684 GenomeReviews:CT485782_GR GO:GO:0005829 GO:GO:0048046 GO:GO:0005515 GO:GO:0051262 EMBL:AC008007 GO:GO:0006099 SUPFAM:SSF51621 GO:GO:0016036 GO:GO:0015979 EMBL:AJ532901 EMBL:AY057507 EMBL:BT000647 IPI:IPI00541694 PIR:D96573 RefSeq:NP_001031178.1 RefSeq:NP_001031179.1 RefSeq:NP_175738.1 UniGene:At.23221 ProteinModelPortal:Q9MAH0 SMR:Q9MAH0 IntAct:Q9MAH0 STRING:Q9MAH0 PRIDE:Q9MAH0 ProMEX:Q9MAH0 EnsemblPlants:AT1G53310.1 EnsemblPlants:AT1G53310.2 EnsemblPlants:AT1G53310.3 GeneID:841765 KEGG:ath:AT1G53310 GeneFarm:5060 TAIR:At1g53310

Tissue	Identification	# of reads (stress)	# of reads (control)	Fold Change	p-value	Description
						eggNOG:COG2352 HOGENOM:HBG351035 InParanoid:Q9MAH0 KO:K01595 OMA:HISKEIA PhylomeDB:Q9MAH0 ProtClustDB:CLSN2679806 BRENDA:4.1.1.31 ArrayExpress:Q9MAH0 Genevestigator:Q9MAH0 GermOnline:AT1G53310 GO:GO:0008964 GO:GO:0015977 Uniprot:Q9MAH0
Root	Isotig02158	34	101	-1.700	1.76E-10	TAIR|locus:2009600 - symbol:PPC1 "AT1G53310" species:3702 "Arabidopsis thaliana" [GO:0006099 "tricarboxylic acid cycle" evidence=ISS] [GO:0008964 "phosphoenolpyruvate carboxylase activity" evidence=ISS] [GO:0005829 "cytosol" evidence=IDA] [GO:0048046 "apoplast" evidence=IDA] [GO:0016036 "cellular response to phosphate starvation" evidence=IDA] [GO:0051262 "protein tetramerization" evidence=IDA] [GO:0005515 "protein binding" evidence=IPI] InterPro:IPR015813 InterPro:IPR018129 InterPro:IPR021135 InterPro:IPR022805 Pfam:PF00311 PRINTS:PR00150 PROSITE:PS00393 PROSITE:PS00781 EMBL:CP002684 GenomeReviews:CT485782_GR GO:GO:0005829 GO:GO:0048046 GO:GO:0005515 GO:GO:0051262 EMBL:AC008007 GO:GO:0006099 SUPFAM:SSF51621 GO:GO:0016036 GO:GO:0015979 EMBL:AJ532901 EMBL:AY057507 EMBL:BT000647 IPI:IPI00541694 PIR:D96573 RefSeq:NP_001031178.1 RefSeq:NP_001031179.1 RefSeq:NP_175738.1 UniGene:At.23221 ProteinModelPortal:Q9MAH0 SMR:Q9MAH0 IntAct:Q9MAH0 STRING:Q9MAH0 PRIDE:Q9MAH0 ProMEX:Q9MAH0 EnsemblPlants:AT1G53310.1 EnsemblPlants:AT1G53310.2 EnsemblPlants:AT1G53310.3 GeneID:841765 KEGG:ath:AT1G53310 GeneFarm:5060 TAIR:At1g53310 eggNOG:COG2352 HOGENOM:HBG351035 InParanoid:Q9MAH0 KO:K01595 OMA:HISKEIA PhylomeDB:Q9MAH0 ProtClustDB:CLSN2679806 BRENDA:4.1.1.31 ArrayExpress:Q9MAH0 Genevestigator:Q9MAH0 GermOnline:AT1G53310 GO:GO:0008964 GO:GO:0015977 Uniprot:Q9MAH0
Root	Isotig02171	2	62	-5.083	8.94E-17	TAIR|locus:2172244 - symbol:HA11 "H(+)-ATPase 11" species:3702 "Arabidopsis thaliana" [GO:0006754 "ATP biosynthetic process" evidence=IEA] [GO:0006812 "cation transport" evidence=IEA] [GO:0008152 "metabolic process" evidence=IEA] [GO:0016887 "ATPase activity" evidence=ISS] [GO:0005886 "plasma membrane" evidence=IDA] [GO:0016020 "membrane" evidence=IDA] InterPro:IPR000695 InterPro:IPR001757 InterPro:IPR005834 InterPro:IPR006534 InterPro:IPR008250 Pfam:PF00122 Pfam:PF00702 PRINTS:PR00119 PRINTS:PR00120 InterPro:IPR018303 InterPro:IPR004014 Pfam:PF00690 Prosite:PS00154 GO:GO:0016021 GO:GO:0005886 GO:GO:0005524 EMBL:CP002688 GenomeReviews:BA000015_GR GO:GO:0046872 InterPro:IPR023214 SUPFAM:SSF56784 GO:GO:0006754 eggNOG:COG0474 InterPro:IPR023300 InterPro:IPR023299 InterPro:IPR023298 Gene3D:G3DSA:2.70.150.10 Gene3D:G3DSA:3.40.1110.10 Gene3D:G3DSA:1.20.1110.10 SMART:SM00831 TIGRFAMs:TIGR01494 GO:GO:0008553 EMBL:AB020751 HOGENOM:HBG706356 KO:K01535 PANTHER:PTHR24093:SF61 TIGRFAMs:TIGR01647 EMBL:AY125493 IPI:IPI00531044 RefSeq:NP_201073.1 UniGene:At.28239 UniGene:At.68527 ProteinModelPortal:Q9LV11 SMR:Q9LV11 STRING:Q9LV11 PRIDE:Q9LV11 EnsemblPlants:AT5G62670.1 GeneID:836388 KEGG:ath:AT5G62670 TAIR:At5g62670 InParanoid:Q9LV11 OMA:SHFQRAV PhylomeDB:Q9LV11 ProtClustDB:CLSN2687353 ArrayExpress:Q9LV11 Genevestigator:Q9LV11 GermOnline:AT5G62670 Uniprot:Q9LV11
Root	Isotig02172	2	57	-4.962	1.85E-15	TAIR|locus:2172244 - symbol:HA11 "H(+)-ATPase 11" species:3702 "Arabidopsis thaliana" [GO:0006754 "ATP biosynthetic process" evidence=IEA] [GO:0006812 "cation transport" evidence=IEA] [GO:0008152 "metabolic process" evidence=IEA] [GO:0016887 "ATPase activity" evidence=ISS] [GO:0005886 "plasma membrane" evidence=IDA] [GO:0016020 "membrane" evidence=IDA] InterPro:IPR000695 InterPro:IPR001757 InterPro:IPR005834 InterPro:IPR006534 InterPro:IPR008250 Pfam:PF00122 Pfam:PF00702 PRINTS:PR00119 PRINTS:PR00120 InterPro:IPR018303 InterPro:IPR004014 Pfam:PF00690 Prosite:PS00154 GO:GO:0016021 GO:GO:0005886 GO:GO:0005524 EMBL:CP002688 GenomeReviews:BA000015_GR GO:GO:0046872 InterPro:IPR023214 SUPFAM:SSF56784 GO:GO:0006754 eggNOG:COG0474 InterPro:IPR023300 InterPro:IPR023299 InterPro:IPR023298 Gene3D:G3DSA:2.70.150.10 Gene3D:G3DSA:3.40.1110.10 Gene3D:G3DSA:1.20.1110.10 SMART:SM00831 TIGRFAMs:TIGR01494 GO:GO:0008553 EMBL:AB020751 HOGENOM:HBG706356 KO:K01535 PANTHER:PTHR24093:SF61 TIGRFAMs:TIGR01647 EMBL:AY125493 IPI:IPI00531044 RefSeq:NP_201073.1 UniGene:At.28239 UniGene:At.68527 ProteinModelPortal:Q9LV11 SMR:Q9LV11 STRING:Q9LV11 PRIDE:Q9LV11 EnsemblPlants:AT5G62670.1 GeneID:836388 KEGG:ath:AT5G62670 TAIR:At5g62670 InParanoid:Q9LV11 OMA:SHFQRAV PhylomeDB:Q9LV11 ProtClustDB:CLSN2687353 ArrayExpress:Q9LV11 Genevestigator:Q9LV11 GermOnline:AT5G62670 Uniprot:Q9LV11
Root	Isotig02173	2	57	-4.962	1.85E-15	TAIR|locus:2172244 - symbol:HA11 "H(+)-ATPase 11" species:3702 "Arabidopsis thaliana" [GO:0006754 "ATP biosynthetic process" evidence=IEA] [GO:0006812 "cation transport" evidence=IEA] [GO:0008152 "metabolic

Tissue	Identification	# of reads (stress)	# of reads (control)	Fold Change	p-value	Description
						process" evidence=IEA] [GO:0016887 "ATPase activity" evidence=ISS] [GO:0005886 "plasma membrane" evidence=IDA] [GO:0016020 "membrane" evidence=IDA] InterPro:IPR000695 InterPro:IPR001757 InterPro:IPR005834 InterPro:IPR006534 InterPro:IPR008250 Pfam:PF00122 Pfam:PF00702 PRINTS:PR00119 PRINTS:PR00120 InterPro:IPR018303 InterPro:IPR004014 Pfam:PF00690 Prosite:PS00154 GO:GO:0016021 GO:GO:0005886 GO:GO:0005524 EMBL:CP002688 GenomeReviews:BA000015_GR GO:GO:0046872 InterPro:IPR023214 SUPFAM:SSF56784 GO:GO:0006754 eggNOG:COG0474 InterPro:IPR023300 InterPro:IPR023299 InterPro:IPR023298 Gene3D:G3DSA:2.70.150.10 Gene3D:G3DSA:3.40.1110.10 Gene3D:G3DSA:1.20.1110.10 SMART:SM00831 TIGRFAMs:TIGR01494 GO:GO:0008553 EMBL:AB020751 HOGENOM:HBG706356 KO:K01535 PANTHER:PTHR24093:SF61 TIGRFAMs:TIGR01647 EMBL:AY125493 IPI:IPI00531044 RefSeq:NP_201073.1 UniGene:At.28239 UniGene:At.68527 ProteinModelPortal:Q9LV11 SMR:Q9LV11 STRING:Q9LV11 PRIDE:Q9LV11 EnsemblPlants:AT5G62670.1 GeneID:836388 KEGG:ath:AT5G62670 TAIR:At5g62670 InParanoid:Q9LV11 OMA:SHFQRAV PhylomeDB:Q9LV11 ProtClustDB:CLSN2687353 ArrayExpress:Q9LV11 Genevestigator:Q9LV11 GermOnline:AT5G62670 Uniprot:Q9LV11
Root	Isotig02174	2	52	-4.829	3.94E-14	TAIR|locus:2172244 - symbol:HA11 "H(+)-ATPase 11" species:3702 "Arabidopsis thaliana" [GO:0006754 "ATP biosynthetic process" evidence=IEA] [GO:0006812 "cation transport" evidence=IEA] [GO:0008152 "metabolic process" evidence=IEA] [GO:0016887 "ATPase activity" evidence=ISS] [GO:0005886 "plasma membrane" evidence=IDA] [GO:0016020 "membrane" evidence=IDA] InterPro:IPR000695 InterPro:IPR001757 InterPro:IPR005834 InterPro:IPR006534 InterPro:IPR008250 Pfam:PF00122 Pfam:PF00702 PRINTS:PR00119 PRINTS:PR00120 InterPro:IPR018303 InterPro:IPR004014 Pfam:PF00690 Prosite:PS00154 GO:GO:0016021 GO:GO:0005886 GO:GO:0005524 EMBL:CP002688 GenomeReviews:BA000015_GR GO:GO:0046872 InterPro:IPR023214 SUPFAM:SSF56784 GO:GO:0006754 eggNOG:COG0474 InterPro:IPR023300 InterPro:IPR023299 InterPro:IPR023298 Gene3D:G3DSA:2.70.150.10 Gene3D:G3DSA:3.40.1110.10 Gene3D:G3DSA:1.20.1110.10 SMART:SM00831 TIGRFAMs:TIGR01494 GO:GO:0008553 EMBL:AB020751 HOGENOM:HBG706356 KO:K01535 PANTHER:PTHR24093:SF61 TIGRFAMs:TIGR01647 EMBL:AY125493 IPI:IPI00531044 RefSeq:NP_201073.1 UniGene:At.28239 UniGene:At.68527 ProteinModelPortal:Q9LV11 SMR:Q9LV11 STRING:Q9LV11 PRIDE:Q9LV11 EnsemblPlants:AT5G62670.1 GeneID:836388 KEGG:ath:AT5G62670 TAIR:At5g62670 InParanoid:Q9LV11 OMA:SHFQRAV PhylomeDB:Q9LV11 ProtClustDB:CLSN2687353 ArrayExpress:Q9LV11 Genevestigator:Q9LV11 GermOnline:AT5G62670 Uniprot:Q9LV11
Root	Isotig02175	3	58	-4.402	4.50E-15	TAIR|locus:2172244 - symbol:HA11 "H(+)-ATPase 11" species:3702 "Arabidopsis thaliana" [GO:0006754 "ATP biosynthetic process" evidence=IEA] [GO:0006812 "cation transport" evidence=IEA] [GO:0008152 "metabolic process" evidence=IEA] [GO:0016887 "ATPase activity" evidence=ISS] [GO:0005886 "plasma membrane" evidence=IDA] [GO:0016020 "membrane" evidence=IDA] InterPro:IPR000695 InterPro:IPR001757 InterPro:IPR005834 InterPro:IPR006534 InterPro:IPR008250 Pfam:PF00122 Pfam:PF00702 PRINTS:PR00119 PRINTS:PR00120 InterPro:IPR018303 InterPro:IPR004014 Pfam:PF00690 Prosite:PS00154 GO:GO:0016021 GO:GO:0005886 GO:GO:0005524 EMBL:CP002688 GenomeReviews:BA000015_GR GO:GO:0046872 InterPro:IPR023214 SUPFAM:SSF56784 GO:GO:0006754 eggNOG:COG0474 InterPro:IPR023300 InterPro:IPR023299 InterPro:IPR023298 Gene3D:G3DSA:2.70.150.10 Gene3D:G3DSA:3.40.1110.10 Gene3D:G3DSA:1.20.1110.10 SMART:SM00831 TIGRFAMs:TIGR01494 GO:GO:0008553 EMBL:AB020751 HOGENOM:HBG706356 KO:K01535 PANTHER:PTHR24093:SF61 TIGRFAMs:TIGR01647 EMBL:AY125493 IPI:IPI00531044 RefSeq:NP_201073.1 UniGene:At.28239 UniGene:At.68527 ProteinModelPortal:Q9LV11 SMR:Q9LV11 STRING:Q9LV11 PRIDE:Q9LV11 EnsemblPlants:AT5G62670.1 GeneID:836388 KEGG:ath:AT5G62670 TAIR:At5g62670 InParanoid:Q9LV11 OMA:SHFQRAV PhylomeDB:Q9LV11 ProtClustDB:CLSN2687353 ArrayExpress:Q9LV11 Genevestigator:Q9LV11 GermOnline:AT5G62670 Uniprot:Q9LV11
Root	Isotig02176	3	53	-4.272	1.02E-13	TAIR|locus:2172244 - symbol:HA11 "H(+)-ATPase 11" species:3702 "Arabidopsis thaliana" [GO:0006754 "ATP biosynthetic process" evidence=IEA] [GO:0006812 "cation transport" evidence=IEA] [GO:0008152 "metabolic process" evidence=IEA] [GO:0016887 "ATPase activity" evidence=ISS] [GO:0005886 "plasma membrane" evidence=IDA] [GO:0016020 "membrane" evidence=IDA] InterPro:IPR000695 InterPro:IPR001757 InterPro:IPR005834 InterPro:IPR006534 InterPro:IPR008250 Pfam:PF00122 Pfam:PF00702 PRINTS:PR00119 PRINTS:PR00120 InterPro:IPR018303 InterPro:IPR004014 Pfam:PF00690 Prosite:PS00154 GO:GO:0016021

Tissue	Identification	# of reads (stress)	# of reads (control)	Fold Change	p-value	Description
						GO:GO:0005886 GO:GO:0005524 EMBL:CP002688 GenomeReviews:BA000015_GR GO:GO:0046872 InterPro:IPR023214 SUPFAM:SSF56784 GO:GO:0006754 eggNOG:COG0474 InterPro:IPR023300 InterPro:IPR023299 InterPro:IPR023298 Gene3D:G3DSA:2.70.150.10 Gene3D:G3DSA:3.40.1110.10 Gene3D:G3DSA:1.20.1110.10 SMART:SM00831 TIGRFAMs:TIGR01494 GO:GO:0008553 EMBL:AB020751 HOGENOM:HBG706356 KO:K01535 PANTHER:PTHR24093:SF61 TIGRFAMs:TIGR01647 EMBL:AY125493 IPI:IPI00531044 RefSeq:NP_201073.1 UniGene:At.28239 UniGene:At.68527 ProteinModelPortal:Q9LV11 SMR:Q9LV11 STRING:Q9LV11 PRIDE:Q9LV11 EnsemblPlants:AT5G62670.1 GeneID:836388 KEGG:ath:AT5G62670 TAIR:At5g62670 InParanoid:Q9LV11 OMA:SHFQRAV PhylomeDB:Q9LV11 ProtClustDB:CLSN2687353 ArrayExpress:Q9LV11 Genevestigator:Q9LV11 GermOnline:AT5G62670 Uniprot:Q9LV11
Root	Isotig02201	96	0	7.456	6.34E-22	TAIR|locus:2024522 - symbol:DAA1 "DUO1-activated ATPase 1" species:3702 "Arabidopsis thaliana" [GO:0000166 "nucleotide binding" evidence=IEA] [GO:0005524 "ATP binding" evidence=IEA] [GO:0016887 "ATPase activity" evidence=ISS] [GO:0017111 "nucleoside-triphosphatase activity" evidence=IEA] [GO:0048235 "pollen sperm cell differentiation" evidence=IEP] InterPro:IPR003593 InterPro:IPR003959 InterPro:IPR003960 Pfam:PF00004 PROSITE:PS00674 SMART:SM00382 EMBL:CP002684 GO:GO:0005524 eggNOG:COG0464 GO:GO:0017111 GO:GO:0048235 EMBL:AC007764 IPI:IPI00891776 RefSeq:NP_001117544.1 UniGene:At.16450 ProteinModelPortal:B3H7I9 SMR:B3H7I9 PRIDE:B3H7I9 EnsemblPlants:AT1G64110.3 GeneID:842716 KEGG:ath:AT1G64110 TAIR:At1g64110 PhylomeDB:B3H7I9 ProtClustDB:CLSN2689017 Genevestigator:B3H7I9 Uniprot:B3H7I9
Root	Isotig02202	99	0	7.500	1.74E-22	TAIR|locus:2024522 - symbol:DAA1 "DUO1-activated ATPase 1" species:3702 "Arabidopsis thaliana" [GO:0000166 "nucleotide binding" evidence=IEA] [GO:0005524 "ATP binding" evidence=IEA] [GO:0016887 "ATPase activity" evidence=ISS] [GO:0017111 "nucleoside-triphosphatase activity" evidence=IEA] [GO:0048235 "pollen sperm cell differentiation" evidence=IEP] InterPro:IPR003593 InterPro:IPR003959 InterPro:IPR003960 Pfam:PF00004 PROSITE:PS00674 SMART:SM00382 EMBL:CP002684 GO:GO:0005524 eggNOG:COG0464 GO:GO:0017111 GO:GO:0048235 EMBL:AC007764 IPI:IPI00891776 RefSeq:NP_001117544.1 UniGene:At.16450 ProteinModelPortal:B3H7I9 SMR:B3H7I9 PRIDE:B3H7I9 EnsemblPlants:AT1G64110.3 GeneID:842716 KEGG:ath:AT1G64110 TAIR:At1g64110 PhylomeDB:B3H7I9 ProtClustDB:CLSN2689017 Genevestigator:B3H7I9 Uniprot:B3H7I9
Root	Isotig02203	92	0	7.395	3.60E-21	TAIR|locus:2024522 - symbol:DAA1 "DUO1-activated ATPase 1" species:3702 "Arabidopsis thaliana" [GO:0000166 "nucleotide binding" evidence=IEA] [GO:0005524 "ATP binding" evidence=IEA] [GO:0016887 "ATPase activity" evidence=ISS] [GO:0017111 "nucleoside-triphosphatase activity" evidence=IEA] [GO:0048235 "pollen sperm cell differentiation" evidence=IEP] InterPro:IPR003593 InterPro:IPR003959 InterPro:IPR003960 Pfam:PF00004 PROSITE:PS00674 SMART:SM00382 EMBL:CP002684 GO:GO:0005524 eggNOG:COG0464 GO:GO:0017111 GO:GO:0048235 EMBL:AC007764 IPI:IPI00891776 RefSeq:NP_001117544.1 UniGene:At.16450 ProteinModelPortal:B3H7I9 SMR:B3H7I9 PRIDE:B3H7I9 EnsemblPlants:AT1G64110.3 GeneID:842716 KEGG:ath:AT1G64110 TAIR:At1g64110 PhylomeDB:B3H7I9 ProtClustDB:CLSN2689017 Genevestigator:B3H7I9 Uniprot:B3H7I9
Root	Isotig02204	95	0	7.441	9.78E-22	TAIR|locus:2024522 - symbol:DAA1 "DUO1-activated ATPase 1" species:3702 "Arabidopsis thaliana" [GO:0000166 "nucleotide binding" evidence=IEA] [GO:0005524 "ATP binding" evidence=IEA] [GO:0016887 "ATPase activity" evidence=ISS] [GO:0017111 "nucleoside-triphosphatase activity" evidence=IEA] [GO:0048235 "pollen sperm cell differentiation" evidence=IEP] InterPro:IPR003593 InterPro:IPR003959 InterPro:IPR003960 Pfam:PF00004 PROSITE:PS00674 SMART:SM00382 EMBL:CP002684 GO:GO:0005524 eggNOG:COG0464 GO:GO:0017111 GO:GO:0048235 EMBL:AC007764 IPI:IPI00891776 RefSeq:NP_001117544.1 UniGene:At.16450 ProteinModelPortal:B3H7I9 SMR:B3H7I9 PRIDE:B3H7I9 EnsemblPlants:AT1G64110.3 GeneID:842716 KEGG:ath:AT1G64110 TAIR:At1g64110 PhylomeDB:B3H7I9 ProtClustDB:CLSN2689017 Genevestigator:B3H7I9 Uniprot:B3H7I9
Root	Isotig02205	49	0	6.486	1.48E-12	TAIR|locus:2024522 - symbol:DAA1 "DUO1-activated ATPase 1" species:3702 "Arabidopsis thaliana" [GO:0000166 "nucleotide binding" evidence=IEA] [GO:0005524 "ATP binding" evidence=IEA] [GO:0016887 "ATPase activity" evidence=ISS] [GO:0017111 "nucleoside-triphosphatase activity" evidence=IEA] [GO:0048235 "pollen sperm cell differentiation" evidence=IEP] InterPro:IPR003593 InterPro:IPR003959 InterPro:IPR003960 Pfam:PF00004 PROSITE:PS00674 SMART:SM00382 EMBL:CP002684 GO:GO:0005524 eggNOG:COG0464 GO:GO:0017111 GO:GO:0048235 EMBL:AC007764 IPI:IPI00891776 RefSeq:NP_001117544.1 UniGene:At.16450

Tissue	Identification	# of reads (stress)	# of reads (control)	Fold Change	p-value	Description
						ProteinModelPortal:B3H7I9 SMR:B3H7I9 PRIDE:B3H7I9 EnsemblPlants:AT1G64110.3 GeneID:842716 KEGG:ath:AT1G64110 TAIR:At1g64110 PhylomeDB:B3H7I9 ProtClustDB:CLSN2689017 Genevestigator:B3H7I9 Uniprot:B3H7I9
Root	Isotig02206	52	0	6.571	3.42E-13	TAIR|locus:2024522 - symbol:DAA1 "DUO1-activated ATPase 1" species:3702 "Arabidopsis thaliana" [GO:0000166 "nucleotide binding" evidence=IEA] [GO:0005524 "ATP binding" evidence=IEA] [GO:0016887 "ATPase activity" evidence=ISS] [GO:0017111 "nucleoside-triphosphatase activity" evidence=IEA] [GO:0048235 "pollen sperm cell differentiation" evidence=IEP] InterPro:IPR003593 InterPro:IPR003959 InterPro:IPR003960 Pfam:PF00004 PROSITE:PS00674 SMART:SM00382 EMBL:CP002684 GO:GO:0005524 eggNOG:COG0464 GO:GO:0017111 GO:GO:0048235 EMBL:AC007764 IPI:IPI00891776 RefSeq:NP_001117544.1 UniGene:At.16450 ProteinModelPortal:B3H7I9 SMR:B3H7I9 PRIDE:B3H7I9 EnsemblPlants:AT1G64110.3 GeneID:842716 KEGG:ath:AT1G64110 TAIR:At1g64110 PhylomeDB:B3H7I9 ProtClustDB:CLSN2689017 Genevestigator:B3H7I9 Uniprot:B3H7I9
Root	Isotig02211	1	17	-4.216	2.69E-05	TAIR|locus:2143443 - symbol:FLA11 "AT5G03170" species:3702 "Arabidopsis thaliana" [GO:0031225 "anchored to membrane" evidence=TAS] [GO:0009834 "secondary cell wall biogenesis" evidence=IMP] [GO:0005886 "plasma membrane" evidence=IDA] Pfam:PF02469 InterPro:IPR000782 GO:GO:0005886 EMBL:CP002688 GenomeReviews:BA000015_GR GO:GO:0031225 EMBL:AB005240 Gene3D:G3DSA:2.30.180.10 SMART:SM00554 SUPFAM:SSF82153 PROSITE:PS50213 GO:GO:0009834 EMBL:AL163002 EMBL:AF360183 EMBL:AY039999 EMBL:AY085388 IPI:IPI00524222 PIR:T48338 RefSeq:NP_195937.1 UniGene:At.27796 UniGene:At.69706 ProteinModelPortal:Q8LEJ6 SMR:Q8LEJ6 STRING:Q8LEJ6 PRIDE:Q8LEJ6 EnsemblPlants:AT5G03170.1 GeneID:831914 KEGG:ath:AT5G03170 GeneFarm:3783 TAIR:At5g03170 eggNOG:NOG311981 HOGENOM:HBG750250 InParanoid:Q8LEJ6 OMA:AVYQVDQ PhylomeDB:Q8LEJ6 ProtClustDB:CLSN2916901 ArrayExpress:Q9LYW8 Genevestigator:Q8LEJ6 GermOnline:AT5G03170 Uniprot:Q8LEJ6
Root	Isotig02212	1	18	-4.299	1.41E-05	TAIR|locus:2143443 - symbol:FLA11 "AT5G03170" species:3702 "Arabidopsis thaliana" [GO:0031225 "anchored to membrane" evidence=TAS] [GO:0009834 "secondary cell wall biogenesis" evidence=IMP] [GO:0005886 "plasma membrane" evidence=IDA] Pfam:PF02469 InterPro:IPR000782 GO:GO:0005886 EMBL:CP002688 GenomeReviews:BA000015_GR GO:GO:0031225 EMBL:AB005240 Gene3D:G3DSA:2.30.180.10 SMART:SM00554 SUPFAM:SSF82153 PROSITE:PS50213 GO:GO:0009834 EMBL:AL163002 EMBL:AF360183 EMBL:AY039999 EMBL:AY085388 IPI:IPI00524222 PIR:T48338 RefSeq:NP_195937.1 UniGene:At.27796 UniGene:At.69706 ProteinModelPortal:Q8LEJ6 SMR:Q8LEJ6 STRING:Q8LEJ6 PRIDE:Q8LEJ6 EnsemblPlants:AT5G03170.1 GeneID:831914 KEGG:ath:AT5G03170 GeneFarm:3783 TAIR:At5g03170 eggNOG:NOG311981 HOGENOM:HBG750250 InParanoid:Q8LEJ6 OMA:AVYQVDQ PhylomeDB:Q8LEJ6 ProtClustDB:CLSN2916901 ArrayExpress:Q9LYW8 Genevestigator:Q8LEJ6 GermOnline:AT5G03170 Uniprot:Q8LEJ6
Root	Isotig02213	2	34	-4.216	2.89E-09	TAIR|locus:2076244 - symbol:PAL4 "phenylalanine ammonia-lyase 4" species:3702 "Arabidopsis thaliana" [GO:0003824 "catalytic activity" evidence=IEA] [GO:0005737 "cytoplasm" evidence=IEA] [GO:0006559 "L-phenylalanine catabolic process" evidence=IEA] [GO:0009058 "biosynthetic process" evidence=IEA] [GO:0016841 "ammonia-lyase activity" evidence=IEA] InterPro:IPR001106 InterPro:IPR005922 InterPro:IPR008948 InterPro:IPR022313 Pfam:PF00221 PROSITE:PS00488 GO:GO:0005737 EMBL:CP002686 GenomeReviews:BA000014_GR GO:GO:0009698 GO:GO:0009058 SUPFAM:SSF48557 GO:GO:0006559 InterPro:IPR024083 Gene3D:G3DSA:1.10.275.10 EMBL:AC009400 eggNOG:COG2986 HOGENOM:HBG318028 KO:K10775 ProtClustDB:PLN02457 BRENDA:4.3.1.24 InterPro:IPR023144 Gene3D:G3DSA:1.10.274.20 TIGRFAMs:TIGR01226 GO:GO:0016841 EMBL:AY303130 EMBL:AY099657 EMBL:BT000252 IPI:IPI00533524 RefSeq:NP_187645.1 UniGene:At.18429 UniGene:At.67148 ProteinModelPortal:Q9SS45 SMR:Q9SS45 IntAct:Q9SS45 STRING:Q9SS45 PRIDE:Q9SS45 EnsemblPlants:AT3G10340.1 GeneID:820196 KEGG:ath:AT3G10340 TAIR:At3g10340 InParanoid:Q9SS45 OMA:GERQATQ PhylomeDB:Q9SS45 ArrayExpress:Q9SS45 Genevestigator:Q9SS45 GermOnline:AT3G10340 Uniprot:Q9SS45
Root	Isotig02214	2	31	-4.083	1.93E-08	TAIR|locus:2101958 - symbol:PAL2 "AT3G53260" species:3702 "Arabidopsis thaliana" [GO:0006979 "response to oxidative stress" evidence=IEP] [GO:0080167 "response to karrikin" evidence=IEP] [GO:0005829 "cytosol" evidence=IDA] [GO:0006952 "defense response" evidence=TAS] [GO:0009611 "response to wounding" evidence=TAS] [GO:0009699 "phenylpropanoid biosynthetic process" evidence=TAS] [GO:0045548 "phenylalanine ammonia-lyase activity" evidence=ISS;TAS] InterPro:IPR001106 InterPro:IPR005922 InterPro:IPR008948

Tissue	Identification	# of reads (stress)	# of reads (control)	Fold Change	p-value	Description
						InterPro:IPR022313 Pfam:PF00221 PROSITE:PS00488 GO:GO:0005829 EMBL:CP002686 GenomeReviews:BA000014_GR GO:GO:0006979 GO:GO:0009699 GO:GO:0080167 SUPFAM:SSF48557 GO:GO:0006559 InterPro:IPR024083 Gene3D:G3DSA:1.10.275.10 EMBL:AL132958 eggNOG:COG2986 UniGene:At.21614 HOGENOM:HBG318028 KO:K10775 ProtClustDB:PLN02457 BRENDA:4.3.1.24 GO:GO:0045548 InterPro:IPR023144 Gene3D:G3DSA:1.10.274.20 TIGRFAMs:TIGR01226 EMBL:L33678 EMBL:AY303129 EMBL:AF367308 EMBL:AY092957 EMBL:AY133595 EMBL:BT000035 IPI:IPI00525638 PIR:T46172 RefSeq:NP_190894.1 ProteinModelPortal:P45724 SMR:P45724 IntAct:P45724 STRING:P45724 PRIDE:P45724 EnsemblPlants:AT3G53260.1 GeneID:824493 KEGG:ath:AT3G53260 TAIR:At3g53260 InParanoid:P45724 OMA:PLNWGLA PhylomeDB:P45724 Genevestigator:P45724 GermOnline:AT3G53260 Uniprot:P45724
Root	Isotig02215	2	34	-4.216	2.89E-09	TAIR|locus:2076244 - symbol:PAL4 "phenylalanine ammonia-lyase 4" species:3702 "Arabidopsis thaliana" [GO:0003824 "catalytic activity" evidence=IEA] [GO:0005737 "cytoplasm" evidence=IEA] [GO:0006559 "L-phenylalanine catabolic process" evidence=IEA] [GO:0009058 "biosynthetic process" evidence=IEA] [GO:0016841 "ammonia-lyase activity" evidence=IEA] InterPro:IPR001106 InterPro:IPR005922 InterPro:IPR008948 InterPro:IPR022313 Pfam:PF00221 PROSITE:PS00488 GO:GO:0005737 EMBL:CP002686 GenomeReviews:BA000014_GR GO:GO:0009698 GO:GO:0009058 SUPFAM:SSF48557 GO:GO:0006559 InterPro:IPR024083 Gene3D:G3DSA:1.10.275.10 EMBL:AC009400 eggNOG:COG2986 HOGENOM:HBG318028 KO:K10775 ProtClustDB:PLN02457 BRENDA:4.3.1.24 InterPro:IPR023144 Gene3D:G3DSA:1.10.274.20 TIGRFAMs:TIGR01226 GO:GO:0016841 EMBL:AY303130 EMBL:AY099657 EMBL:BT000252 IPI:IPI00533524 RefSeq:NP_187645.1 UniGene:At.18429 UniGene:At.67148 ProteinModelPortal:Q9SS45 SMR:Q9SS45 IntAct:Q9SS45 STRING:Q9SS45 PRIDE:Q9SS45 EnsemblPlants:AT3G10340.1 GeneID:820196 KEGG:ath:AT3G10340 TAIR:At3g10340 InParanoid:Q9SS45 OMA:GERQATQ PhylomeDB:Q9SS45 ArrayExpress:Q9SS45 Genevestigator:Q9SS45 GermOnline:AT3G10340 Uniprot:Q9SS45
Root	Isotig02216	2	34	-4.216	2.89E-09	TAIR|locus:2076244 - symbol:PAL4 "phenylalanine ammonia-lyase 4" species:3702 "Arabidopsis thaliana" [GO:0003824 "catalytic activity" evidence=IEA] [GO:0005737 "cytoplasm" evidence=IEA] [GO:0006559 "L-phenylalanine catabolic process" evidence=IEA] [GO:0009058 "biosynthetic process" evidence=IEA] [GO:0016841 "ammonia-lyase activity" evidence=IEA] InterPro:IPR001106 InterPro:IPR005922 InterPro:IPR008948 InterPro:IPR022313 Pfam:PF00221 PROSITE:PS00488 GO:GO:0005737 EMBL:CP002686 GenomeReviews:BA000014_GR GO:GO:0009698 GO:GO:0009058 SUPFAM:SSF48557 GO:GO:0006559 InterPro:IPR024083 Gene3D:G3DSA:1.10.275.10 EMBL:AC009400 eggNOG:COG2986 HOGENOM:HBG318028 KO:K10775 ProtClustDB:PLN02457 BRENDA:4.3.1.24 InterPro:IPR023144 Gene3D:G3DSA:1.10.274.20 TIGRFAMs:TIGR01226 GO:GO:0016841 EMBL:AY303130 EMBL:AY099657 EMBL:BT000252 IPI:IPI00533524 RefSeq:NP_187645.1 UniGene:At.18429 UniGene:At.67148 ProteinModelPortal:Q9SS45 SMR:Q9SS45 IntAct:Q9SS45 STRING:Q9SS45 PRIDE:Q9SS45 EnsemblPlants:AT3G10340.1 GeneID:820196 KEGG:ath:AT3G10340 TAIR:At3g10340 InParanoid:Q9SS45 OMA:GERQATQ PhylomeDB:Q9SS45 ArrayExpress:Q9SS45 Genevestigator:Q9SS45 GermOnline:AT3G10340 Uniprot:Q9SS45
Root	Isotig02217	2	31	-4.083	1.93E-08	TAIR|locus:2101958 - symbol:PAL2 "AT3G53260" species:3702 "Arabidopsis thaliana" [GO:0006979 "response to oxidative stress" evidence=IEP] [GO:0080167 "response to karrikin" evidence=IEP] [GO:0005829 "cytosol" evidence=IDA] [GO:0006952 "defense response" evidence=TAS] [GO:0009611 "response to wounding" evidence=TAS] [GO:0009699 "phenylpropanoid biosynthetic process" evidence=TAS] [GO:0045548 "phenylalanine ammonia-lyase activity" evidence=ISS;TAS] InterPro:IPR001106 InterPro:IPR005922 InterPro:IPR008948 InterPro:IPR022313 Pfam:PF00221 PROSITE:PS00488 GO:GO:0005829 EMBL:CP002686 GenomeReviews:BA000014_GR GO:GO:0006979 GO:GO:0009699 GO:GO:0080167 SUPFAM:SSF48557 GO:GO:0006559 InterPro:IPR024083 Gene3D:G3DSA:1.10.275.10 EMBL:AL132958 eggNOG:COG2986 UniGene:At.21614 HOGENOM:HBG318028 KO:K10775 ProtClustDB:PLN02457 BRENDA:4.3.1.24 GO:GO:0045548 InterPro:IPR023144 Gene3D:G3DSA:1.10.274.20 TIGRFAMs:TIGR01226 EMBL:L33678 EMBL:AY303129 EMBL:AF367308 EMBL:AY092957 EMBL:AY133595 EMBL:BT000035 IPI:IPI00525638 PIR:T46172 RefSeq:NP_190894.1 ProteinModelPortal:P45724 SMR:P45724 IntAct:P45724 STRING:P45724 PRIDE:P45724 EnsemblPlants:AT3G53260.1 GeneID:824493 KEGG:ath:AT3G53260 TAIR:At3g53260 InParanoid:P45724 OMA:PLNWGLA PhylomeDB:P45724 Genevestigator:P45724 GermOnline:AT3G53260 Uniprot:P45724

Tissue	Identification	# of reads (stress)	# of reads (control)	Fold Change	p-value	Description
Root	Isotig02218	2	31	-4.083	1.93E-08	TAIR|locus:2076244 - symbol:PAL4 "phenylalanine ammonia-lyase 4" species:3702 "Arabidopsis thaliana" [GO:0003824 "catalytic activity" evidence=IEA] [GO:0005737 "cytoplasm" evidence=IEA] [GO:0006559 "L-phenylalanine catabolic process" evidence=IEA] [GO:0009058 "biosynthetic process" evidence=IEA] [GO:0016841 "ammonia-lyase activity" evidence=IEA] InterPro:IPR001106 InterPro:IPR005922 InterPro:IPR008948 InterPro:IPR022313 Pfam:PF00221 PROSITE:PS00488 GO:GO:0005737 EMBL:CP002686 GenomeReviews:BA000014_GR GO:GO:0009698 GO:GO:0009058 SUPFAM:SSF48557 GO:GO:0006559 InterPro:IPR024083 Gene3D:G3DSA:1.10.275.10 EMBL:AC009400 eggNOG:COG2986 HOGENOM:HBG318028 KO:K10775 ProtClustDB:PLN02457 BRENDA:4.3.1.24 InterPro:IPR023144 Gene3D:G3DSA:1.10.274.20 TIGRFAMs:TIGR01226 GO:GO:0016841 EMBL:AY303130 EMBL:AY099657 EMBL:BT000252 IPI:IPI00533524 RefSeq:NP_187645.1 UniGene:At.18429 UniGene:At.67148 ProteinModelPortal:Q9SS45 SMR:Q9SS45 IntAct:Q9SS45 STRING:Q9SS45 PRIDE:Q9SS45 EnsemblPlants:AT3G10340.1 GeneID:820196 KEGG:ath:AT3G10340 TAIR:At3g10340 InParanoid:Q9SS45 OMA:GERQATQ PhylomeDB:Q9SS45 ArrayExpress:Q9SS45 Genevestigator:Q9SS45 GermOnline:AT3G10340 Uniprot:Q9SS45
Root	Isotig02237	52	16	1.571	3.49E-05	TAIR|locus:2046079 - symbol:AT2G12400 "AT2G12400" species:3702 "Arabidopsis thaliana" [GO:0003674 "molecular_function" evidence=ND] [GO:0008150 "biological_process" evidence=ND] [GO:0009506 "plasmodesma" evidence=IDA] GO:GO:0009506 EMBL:CP002685 GenomeReviews:CT485783_GR eggNOG:NOG277729 HOGENOM:HBG319596 EMBL:BT002043 IPI:IPI00530884 RefSeq:NP_178935.2 UniGene:At.14383 PRIDE:Q8H0U2 EnsemblPlants:AT2G12400.1 GeneID:815712 KEGG:ath:AT2G12400 TAIR:At2g12400 InParanoid:Q8H0U2 OMA:MPLLCNP PhylomeDB:Q8H0U2 ProtClustDB:CLSN2690686 Genevestigator:Q8H0U2 Uniprot:Q8H0U2
Root	Isotig02238	52	15	1.665	1.60E-05	TAIR|locus:2046079 - symbol:AT2G12400 "AT2G12400" species:3702 "Arabidopsis thaliana" [GO:0003674 "molecular_function" evidence=ND] [GO:0008150 "biological_process" evidence=ND] [GO:0009506 "plasmodesma" evidence=IDA] GO:GO:0009506 EMBL:CP002685 GenomeReviews:CT485783_GR eggNOG:NOG277729 HOGENOM:HBG319596 EMBL:BT002043 IPI:IPI00530884 RefSeq:NP_178935.2 UniGene:At.14383 PRIDE:Q8H0U2 EnsemblPlants:AT2G12400.1 GeneID:815712 KEGG:ath:AT2G12400 TAIR:At2g12400 InParanoid:Q8H0U2 OMA:MPLLCNP PhylomeDB:Q8H0U2 ProtClustDB:CLSN2690686 Genevestigator:Q8H0U2 Uniprot:Q8H0U2
Root	Isotig02239	51	16	1.543	5.22E-05	TAIR|locus:2046079 - symbol:AT2G12400 "AT2G12400" species:3702 "Arabidopsis thaliana" [GO:0003674 "molecular_function" evidence=ND] [GO:0008150 "biological_process" evidence=ND] [GO:0009506 "plasmodesma" evidence=IDA] GO:GO:0009506 EMBL:CP002685 GenomeReviews:CT485783_GR eggNOG:NOG277729 HOGENOM:HBG319596 EMBL:BT002043 IPI:IPI00530884 RefSeq:NP_178935.2 UniGene:At.14383 PRIDE:Q8H0U2 EnsemblPlants:AT2G12400.1 GeneID:815712 KEGG:ath:AT2G12400 TAIR:At2g12400 InParanoid:Q8H0U2 OMA:MPLLCNP PhylomeDB:Q8H0U2 ProtClustDB:CLSN2690686 Genevestigator:Q8H0U2 Uniprot:Q8H0U2
Root	Isotig02240	52	15	1.665	1.60E-05	TAIR|locus:2046079 - symbol:AT2G12400 "AT2G12400" species:3702 "Arabidopsis thaliana" [GO:0003674 "molecular_function" evidence=ND] [GO:0008150 "biological_process" evidence=ND] [GO:0009506 "plasmodesma" evidence=IDA] GO:GO:0009506 EMBL:CP002685 GenomeReviews:CT485783_GR eggNOG:NOG277729 HOGENOM:HBG319596 EMBL:BT002043 IPI:IPI00530884 RefSeq:NP_178935.2 UniGene:At.14383 PRIDE:Q8H0U2 EnsemblPlants:AT2G12400.1 GeneID:815712 KEGG:ath:AT2G12400 TAIR:At2g12400 InParanoid:Q8H0U2 OMA:MPLLCNP PhylomeDB:Q8H0U2 ProtClustDB:CLSN2690686 Genevestigator:Q8H0U2 Uniprot:Q8H0U2
Root	Isotig02241	51	15	1.637	2.43E-05	TAIR|locus:2046079 - symbol:AT2G12400 "AT2G12400" species:3702 "Arabidopsis thaliana" [GO:0003674 "molecular_function" evidence=ND] [GO:0008150 "biological_process" evidence=ND] [GO:0009506 "plasmodesma" evidence=IDA] GO:GO:0009506 EMBL:CP002685 GenomeReviews:CT485783_GR eggNOG:NOG277729 HOGENOM:HBG319596 EMBL:BT002043 IPI:IPI00530884 RefSeq:NP_178935.2 UniGene:At.14383 PRIDE:Q8H0U2 EnsemblPlants:AT2G12400.1 GeneID:815712 KEGG:ath:AT2G12400 TAIR:At2g12400 InParanoid:Q8H0U2 OMA:MPLLCNP PhylomeDB:Q8H0U2 ProtClustDB:CLSN2690686 Genevestigator:Q8H0U2 Uniprot:Q8H0U2
Root	Isotig02242	51	15	1.637	2.43E-05	TAIR|locus:2046079 - symbol:AT2G12400 "AT2G12400" species:3702 "Arabidopsis thaliana" [GO:0003674 "molecular_function" evidence=ND] [GO:0008150 "biological_process" evidence=ND] [GO:0009506 "plasmodesma" evidence=IDA] GO:GO:0009506 EMBL:CP002685 GenomeReviews:CT485783_GR eggNOG:NOG277729 HOGENOM:HBG319596 EMBL:BT002043 IPI:IPI00530884 RefSeq:NP_178935.2 UniGene:At.14383

Tissue	Identification	# of reads (stress)	# of reads (control)	Fold Change	p-value	Description
						PRIDE:Q8H0U2 EnsemblPlants:AT2G12400.1 GeneID:815712 KEGG:ath:AT2G12400 TAIR:At2g12400 InParanoid:Q8H0U2 OMA:MPLLCNP PhylomeDB:Q8H0U2 ProtClustDB:CLSN2690686 Genevestigator:Q8H0U2 Uniprot:Q8H0U2
Root	Isotig02255	7	39	-2.607	2.03E-07	TAIR|locus:2172249 - symbol:AT5G62680 "AT5G62680" species:3702 "Arabidopsis thaliana" [GO:0005215 "transporter activity" evidence=ISS] [GO:0006857 "oligopeptide transport" evidence=ISS] [GO:0016020 "membrane" evidence=ISS] [GO:0005886 "plasma membrane" evidence=IDA] [GO:0080167 "response to karrikin" evidence=IEP] [GO:0009506 "plasmodesma" evidence=IDA] InterPro:IPR000109 Pfam:PF00854 PROSITE:PS01022 PROSITE:PS01023 GO:GO:0016021 GO:GO:0005886 GO:GO:0009506 EMBL:CP002688 GenomeReviews:BA000015_GR GO:GO:0080167 GO:GO:0005215 InterPro:IPR016196 SUPFAM:SSF103473 EMBL:AB020751 eggNOG:COG3104 GO:GO:0006857 UniGene:At.28239 UniGene:At.68527 ProtClustDB:CLSN2687358 EMBL:AK316918 IPI:IPI00527254 RefSeq:NP_201074.1 ProteinModelPortal:Q9LV10 STRING:Q9LV10 PRIDE:Q9LV10 EnsemblPlants:AT5G62680.1 GeneID:836389 KEGG:ath:AT5G62680 TAIR:At5g62680 InParanoid:Q9LV10 OMA:IDSFFNW PhylomeDB:Q9LV10 Genevestigator:Q9LV10 Uniprot:Q9LV10
Root	Isotig02256	7	38	-2.570	3.60E-07	TAIR|locus:2172249 - symbol:AT5G62680 "AT5G62680" species:3702 "Arabidopsis thaliana" [GO:0005215 "transporter activity" evidence=ISS] [GO:0006857 "oligopeptide transport" evidence=ISS] [GO:0016020 "membrane" evidence=ISS] [GO:0005886 "plasma membrane" evidence=IDA] [GO:0080167 "response to karrikin" evidence=IEP] [GO:0009506 "plasmodesma" evidence=IDA] InterPro:IPR000109 Pfam:PF00854 PROSITE:PS01022 PROSITE:PS01023 GO:GO:0016021 GO:GO:0005886 GO:GO:0009506 EMBL:CP002688 GenomeReviews:BA000015_GR GO:GO:0080167 GO:GO:0005215 InterPro:IPR016196 SUPFAM:SSF103473 EMBL:AB020751 eggNOG:COG3104 GO:GO:0006857 UniGene:At.28239 UniGene:At.68527 ProtClustDB:CLSN2687358 EMBL:AK316918 IPI:IPI00527254 RefSeq:NP_201074.1 ProteinModelPortal:Q9LV10 STRING:Q9LV10 PRIDE:Q9LV10 EnsemblPlants:AT5G62680.1 GeneID:836389 KEGG:ath:AT5G62680 TAIR:At5g62680 InParanoid:Q9LV10 OMA:IDSFFNW PhylomeDB:Q9LV10 Genevestigator:Q9LV10 Uniprot:Q9LV10
Root	Isotig02257	7	39	-2.607	2.03E-07	TAIR|locus:2172249 - symbol:AT5G62680 "AT5G62680" species:3702 "Arabidopsis thaliana" [GO:0005215 "transporter activity" evidence=ISS] [GO:0006857 "oligopeptide transport" evidence=ISS] [GO:0016020 "membrane" evidence=ISS] [GO:0005886 "plasma membrane" evidence=IDA] [GO:0080167 "response to karrikin" evidence=IEP] [GO:0009506 "plasmodesma" evidence=IDA] InterPro:IPR000109 Pfam:PF00854 PROSITE:PS01022 PROSITE:PS01023 GO:GO:0016021 GO:GO:0005886 GO:GO:0009506 EMBL:CP002688 GenomeReviews:BA000015_GR GO:GO:0080167 GO:GO:0005215 InterPro:IPR016196 SUPFAM:SSF103473 EMBL:AB020751 eggNOG:COG3104 GO:GO:0006857 UniGene:At.28239 UniGene:At.68527 ProtClustDB:CLSN2687358 EMBL:AK316918 IPI:IPI00527254 RefSeq:NP_201074.1 ProteinModelPortal:Q9LV10 STRING:Q9LV10 PRIDE:Q9LV10 EnsemblPlants:AT5G62680.1 GeneID:836389 KEGG:ath:AT5G62680 TAIR:At5g62680 InParanoid:Q9LV10 OMA:IDSFFNW PhylomeDB:Q9LV10 Genevestigator:Q9LV10 Uniprot:Q9LV10
Root	Isotig02258	7	29	-2.180	5.47E-05	TAIR|locus:2172249 - symbol:AT5G62680 "AT5G62680" species:3702 "Arabidopsis thaliana" [GO:0005215 "transporter activity" evidence=ISS] [GO:0006857 "oligopeptide transport" evidence=ISS] [GO:0016020 "membrane" evidence=ISS] [GO:0005886 "plasma membrane" evidence=IDA] [GO:0080167 "response to karrikin" evidence=IEP] [GO:0009506 "plasmodesma" evidence=IDA] InterPro:IPR000109 Pfam:PF00854 PROSITE:PS01022 PROSITE:PS01023 GO:GO:0016021 GO:GO:0005886 GO:GO:0009506 EMBL:CP002688 GenomeReviews:BA000015_GR GO:GO:0080167 GO:GO:0005215 InterPro:IPR016196 SUPFAM:SSF103473 EMBL:AB020751 eggNOG:COG3104 GO:GO:0006857 UniGene:At.28239 UniGene:At.68527 ProtClustDB:CLSN2687358 EMBL:AK316918 IPI:IPI00527254 RefSeq:NP_201074.1 ProteinModelPortal:Q9LV10 STRING:Q9LV10 PRIDE:Q9LV10 EnsemblPlants:AT5G62680.1 GeneID:836389 KEGG:ath:AT5G62680 TAIR:At5g62680 InParanoid:Q9LV10 OMA:IDSFFNW PhylomeDB:Q9LV10 Genevestigator:Q9LV10 Uniprot:Q9LV10
Root	Isotig02259	7	28	-2.129	9.37E-05	TAIR|locus:2172249 - symbol:AT5G62680 "AT5G62680" species:3702 "Arabidopsis thaliana" [GO:0005215 "transporter activity" evidence=ISS] [GO:0006857 "oligopeptide transport" evidence=ISS] [GO:0016020 "membrane" evidence=ISS] [GO:0005886 "plasma membrane" evidence=IDA] [GO:0080167 "response to karrikin" evidence=IEP] [GO:0009506 "plasmodesma" evidence=IDA] InterPro:IPR000109 Pfam:PF00854 PROSITE:PS01022 PROSITE:PS01023 GO:GO:0016021 GO:GO:0005886 GO:GO:0009506 EMBL:CP002688

Tissue	Identification	# of reads (stress)	# of reads (control)	Fold Change	p-value	Description
						GenomeReviews:BA000015_GR GO:GO:0080167 GO:GO:0005215 InterPro:IPR016196 SUPFAM:SSF103473 EMBL:AB020751 eggNOG:COG3104 GO:GO:0006857 UniGene:At.28239 UniGene:At.68527 ProtClustDB:CLSN2687358 EMBL:AK316918 IPI:IPI00527254 RefSeq:NP_201074.1 ProteinModelPortal:Q9LV10 STRING:Q9LV10 PRIDE:Q9LV10 EnsemblPlants:AT5G62680.1 GeneID:836389 KEGG:ath:AT5G62680 TAIR:At5g62680 InParanoid:Q9LV10 OMA:IDSFFNW PhylomeDB:Q9LV10 Genevestigator:Q9LV10 Uniprot:Q9LV10
Root	Isotig02260	7	29	-2.180	5.47E-05	TAIR|locus:2172249 - symbol:AT5G62680 "AT5G62680" species:3702 "Arabidopsis thaliana" [GO:0005215 "transporter activity" evidence=ISS] [GO:0006857 "oligopeptide transport" evidence=ISS] [GO:0016020 "membrane" evidence=ISS] [GO:0005886 "plasma membrane" evidence=IDA] [GO:0080167 "response to karrikin" evidence=IEP] [GO:0009506 "plasmodesma" evidence=IDA] InterPro:IPR000109 Pfam:PF00854 PROSITE:PS01022 PROSITE:PS01023 GO:GO:0016021 GO:GO:0005886 GO:GO:0009506 EMBL:CP002688 GenomeReviews:BA000015_GR GO:GO:0080167 GO:GO:0005215 InterPro:IPR016196 SUPFAM:SSF103473 EMBL:AB020751 eggNOG:COG3104 GO:GO:0006857 UniGene:At.28239 UniGene:At.68527 ProtClustDB:CLSN2687358 EMBL:AK316918 IPI:IPI00527254 RefSeq:NP_201074.1 ProteinModelPortal:Q9LV10 STRING:Q9LV10 PRIDE:Q9LV10 EnsemblPlants:AT5G62680.1 GeneID:836389 KEGG:ath:AT5G62680 TAIR:At5g62680 InParanoid:Q9LV10 OMA:IDSFFNW PhylomeDB:Q9LV10 Genevestigator:Q9LV10 Uniprot:Q9LV10
Root	Isotig02261	39	11	1.697	0.000152266	TAIR|locus:2167240 - symbol:GAD "AT5G17330" species:3702 "Arabidopsis thaliana" [GO:0005516 "calmodulin binding" evidence=IDA;TAS;IPI] [GO:0046686 "response to cadmium ion" evidence=IEP] [GO:0005829 "cytosol" evidence=IDA] [GO:0004351 "glutamate decarboxylase activity" evidence=IDA] InterPro:IPR002129 InterPro:IPR010107 InterPro:IPR015421 Pfam:PF00282 PROSITE:PS00392 GO:GO:0005829 GO:GO:0046686 EMBL:CP002688 GenomeReviews:BA000015_GR GO:GO:0030170 InterPro:IPR015424 Gene3D:G3DSA:3.40.640.10 SUPFAM:SSF53383 GO:GO:0006536 GO:GO:0005516 EMBL:AB005238 eggNOG:COG0076 PANTHER:PTHR11999 EMBL:U10034 EMBL:AY094464 EMBL:BT001047 IPI:IPI00530557 RefSeq:NP_197235.1 UniGene:At.25228 PDB:3HBX PDBsum:3HBX ProteinModelPortal:Q42521 SMR:Q42521 STRING:Q42521 PRIDE:Q42521 EnsemblPlants:AT5G17330.1 GeneID:831599 KEGG:ath:AT5G17330 TAIR:At5g17330 HOGENOM:HBG365574 InParanoid:Q42521 KO:K01580 OMA:RRWQNKM PhylomeDB:Q42521 ProtClustDB:CLSN2683665 ArrayExpress:Q42521 Genevestigator:Q42521 GermOnline:AT5G17330 GO:GO:0004351 PANTHER:PTHR11999:SF1 TIGRFAMs:TIGR01788 Uniprot:Q42521
Root	Isotig02263	39	11	1.697	0.000152266	TAIR|locus:2167240 - symbol:GAD "AT5G17330" species:3702 "Arabidopsis thaliana" [GO:0005516 "calmodulin binding" evidence=IDA;TAS;IPI] [GO:0046686 "response to cadmium ion" evidence=IEP] [GO:0005829 "cytosol" evidence=IDA] [GO:0004351 "glutamate decarboxylase activity" evidence=IDA] InterPro:IPR002129 InterPro:IPR010107 InterPro:IPR015421 Pfam:PF00282 PROSITE:PS00392 GO:GO:0005829 GO:GO:0046686 EMBL:CP002688 GenomeReviews:BA000015_GR GO:GO:0030170 InterPro:IPR015424 Gene3D:G3DSA:3.40.640.10 SUPFAM:SSF53383 GO:GO:0006536 GO:GO:0005516 EMBL:AB005238 eggNOG:COG0076 PANTHER:PTHR11999 EMBL:U10034 EMBL:AY094464 EMBL:BT001047 IPI:IPI00530557 RefSeq:NP_197235.1 UniGene:At.25228 PDB:3HBX PDBsum:3HBX ProteinModelPortal:Q42521 SMR:Q42521 STRING:Q42521 PRIDE:Q42521 EnsemblPlants:AT5G17330.1 GeneID:831599 KEGG:ath:AT5G17330 TAIR:At5g17330 HOGENOM:HBG365574 InParanoid:Q42521 KO:K01580 OMA:RRWQNKM PhylomeDB:Q42521 ProtClustDB:CLSN2683665 ArrayExpress:Q42521 Genevestigator:Q42521 GermOnline:AT5G17330 GO:GO:0004351 PANTHER:PTHR11999:SF1 TIGRFAMs:TIGR01788 Uniprot:Q42521
Root	Isotig02267	0	91	-7.637	2.33E-22	TAIR|locus:2046432 - symbol:PHT1;5 "AT2G32830" species:3702 "Arabidopsis thaliana" [GO:0005315 "inorganic phosphate transmembrane transporter activity" evidence=ISS] [GO:0015114 "phosphate ion transmembrane transporter activity" evidence=ISS] [GO:0016021 "integral to membrane" evidence=IEA] [GO:0006817 "phosphate ion transport" evidence=IMP] InterPro:IPR004738 InterPro:IPR005828 Pfam:PF00083 PROSITE:PS00216 PROSITE:PS00217 GO:GO:0016021 EMBL:CP002685 GenomeReviews:CT485783_GR GO:GO:0015293 GO:GO:0005315 eggNOG:COG0477 InterPro:IPR016196 SUPFAM:SSF103473 EMBL:AC003033 InterPro:IPR020846 PROSITE:PS50850 EMBL:AC003974 HOGENOM:HBG744844 KO:K08176 TIGRFAMs:TIGR00887 EMBL:AB000093 EMBL:AY075694 EMBL:AK117670 IPI:IPI00543210 PIR:T01124 RefSeq:NP_180842.1 UniGene:At.38016 ProteinModelPortal:Q8GYF4 EnsemblPlants:AT2G32830.1 GeneID:817844 KEGG:ath:AT2G32830 TAIR:At2g32830 InParanoid:Q8GYF4 OMA:NISWRIM

Tissue	Identification	# of reads (stress)	# of reads (control)	Fold Change	p-value	Description
						PhylomeDB:Q8GYF4 ProtClustDB:CLSN2913065 Genevestigator:Q8GYF4 GermOnline:AT2G32830 Uniprot:Q8GYF4
Root	Isotig02268	0	91	-7.637	2.33E-22	TAIR|locus:2046432 - symbol:PHT1;5 "AT2G32830" species:3702 "Arabidopsis thaliana" [GO:0005315 "inorganic phosphate transmembrane transporter activity" evidence=ISS] [GO:0015114 "phosphate ion transmembrane transporter activity" evidence=ISS] [GO:0016021 "integral to membrane" evidence=IEA] [GO:0006817 "phosphate ion transport" evidence=IMP] InterPro:IPR004738 InterPro:IPR005828 Pfam:PF00083 PROSITE:PS00216 PROSITE:PS00217 GO:GO:0016021 EMBL:CP002685 GenomeReviews:CT485783_GR GO:GO:0015293 GO:GO:0005315 eggNOG:COG0477 InterPro:IPR016196 SUPFAM:SSF103473 EMBL:AC003033 InterPro:IPR020846 PROSITE:PS50850 EMBL:AC003974 HOGENOM:HBG744844 KO:K08176 TIGRFAMs:TIGR00887 EMBL:AB000093 EMBL:AY075694 EMBL:AK117670 IPI:IPI00543210 PIR:T01124 RefSeq:NP_180842.1 UniGene:At.38016 ProteinModelPortal:Q8GYF4 EnsemblPlants:AT2G32830.1 GeneID:817844 KEGG:ath:AT2G32830 TAIR:At2g32830 InParanoid:Q8GYF4 OMA:NISWRIM PhylomeDB:Q8GYF4 ProtClustDB:CLSN2913065 Genevestigator:Q8GYF4 GermOnline:AT2G32830 Uniprot:Q8GYF4
Root	Isotig02269	0	80	-7.451	3.98E-20	TAIR|locus:2046432 - symbol:PHT1;5 "AT2G32830" species:3702 "Arabidopsis thaliana" [GO:0005315 "inorganic phosphate transmembrane transporter activity" evidence=ISS] [GO:0015114 "phosphate ion transmembrane transporter activity" evidence=ISS] [GO:0016021 "integral to membrane" evidence=IEA] [GO:0006817 "phosphate ion transport" evidence=IMP] InterPro:IPR004738 InterPro:IPR005828 Pfam:PF00083 PROSITE:PS00216 PROSITE:PS00217 GO:GO:0016021 EMBL:CP002685 GenomeReviews:CT485783_GR GO:GO:0015293 GO:GO:0005315 eggNOG:COG0477 InterPro:IPR016196 SUPFAM:SSF103473 EMBL:AC003033 InterPro:IPR020846 PROSITE:PS50850 EMBL:AC003974 HOGENOM:HBG744844 KO:K08176 TIGRFAMs:TIGR00887 EMBL:AB000093 EMBL:AY075694 EMBL:AK117670 IPI:IPI00543210 PIR:T01124 RefSeq:NP_180842.1 UniGene:At.38016 ProteinModelPortal:Q8GYF4 EnsemblPlants:AT2G32830.1 GeneID:817844 KEGG:ath:AT2G32830 TAIR:At2g32830 InParanoid:Q8GYF4 OMA:NISWRIM PhylomeDB:Q8GYF4 ProtClustDB:CLSN2913065 Genevestigator:Q8GYF4 GermOnline:AT2G32830 Uniprot:Q8GYF4
Root	Isotig02270	0	80	-7.451	3.98E-20	TAIR|locus:2046432 - symbol:PHT1;5 "AT2G32830" species:3702 "Arabidopsis thaliana" [GO:0005315 "inorganic phosphate transmembrane transporter activity" evidence=ISS] [GO:0015114 "phosphate ion transmembrane transporter activity" evidence=ISS] [GO:0016021 "integral to membrane" evidence=IEA] [GO:0006817 "phosphate ion transport" evidence=IMP] InterPro:IPR004738 InterPro:IPR005828 Pfam:PF00083 PROSITE:PS00216 PROSITE:PS00217 GO:GO:0016021 EMBL:CP002685 GenomeReviews:CT485783_GR GO:GO:0015293 GO:GO:0005315 eggNOG:COG0477 InterPro:IPR016196 SUPFAM:SSF103473 EMBL:AC003033 InterPro:IPR020846 PROSITE:PS50850 EMBL:AC003974 HOGENOM:HBG744844 KO:K08176 TIGRFAMs:TIGR00887 EMBL:AB000093 EMBL:AY075694 EMBL:AK117670 IPI:IPI00543210 PIR:T01124 RefSeq:NP_180842.1 UniGene:At.38016 ProteinModelPortal:Q8GYF4 EnsemblPlants:AT2G32830.1 GeneID:817844 KEGG:ath:AT2G32830 TAIR:At2g32830 InParanoid:Q8GYF4 OMA:NISWRIM PhylomeDB:Q8GYF4 ProtClustDB:CLSN2913065 Genevestigator:Q8GYF4 GermOnline:AT2G32830 Uniprot:Q8GYF4
Root	Isotig02271	0	63	-7.106	1.51E-16	TAIR|locus:2046432 - symbol:PHT1;5 "AT2G32830" species:3702 "Arabidopsis thaliana" [GO:0005315 "inorganic phosphate transmembrane transporter activity" evidence=ISS] [GO:0015114 "phosphate ion transmembrane transporter activity" evidence=ISS] [GO:0016021 "integral to membrane" evidence=IEA] [GO:0006817 "phosphate ion transport" evidence=IMP] InterPro:IPR004738 InterPro:IPR005828 Pfam:PF00083 PROSITE:PS00216 PROSITE:PS00217 GO:GO:0016021 EMBL:CP002685 GenomeReviews:CT485783_GR GO:GO:0015293 GO:GO:0005315 eggNOG:COG0477 InterPro:IPR016196 SUPFAM:SSF103473 EMBL:AC003033 InterPro:IPR020846 PROSITE:PS50850 EMBL:AC003974 HOGENOM:HBG744844 KO:K08176 TIGRFAMs:TIGR00887 EMBL:AB000093 EMBL:AY075694 EMBL:AK117670 IPI:IPI00543210 PIR:T01124 RefSeq:NP_180842.1 UniGene:At.38016 ProteinModelPortal:Q8GYF4 EnsemblPlants:AT2G32830.1 GeneID:817844 KEGG:ath:AT2G32830 TAIR:At2g32830 InParanoid:Q8GYF4 OMA:NISWRIM PhylomeDB:Q8GYF4 ProtClustDB:CLSN2913065 Genevestigator:Q8GYF4 GermOnline:AT2G32830 Uniprot:Q8GYF4
Root	Isotig02272	0	52	-6.829	3.96E-14	TAIR|locus:2046432 - symbol:PHT1;5 "AT2G32830" species:3702 "Arabidopsis thaliana" [GO:0005315 "inorganic phosphate transmembrane transporter activity" evidence=ISS] [GO:0015114 "phosphate ion transmembrane

Tissue	Identification	# of reads (stress)	# of reads (control)	Fold Change	p-value	Description
						transporter activity" evidence=ISS] [GO:0016021 "integral to membrane" evidence=IEA] [GO:0006817 "phosphate ion transport" evidence=IMP] InterPro:IPR004738 InterPro:IPR005828 Pfam:PF00083 PROSITE:PS00216 PROSITE:PS00217 GO:GO:0016021 EMBL:CP002685 GenomeReviews:CT485783_GR GO:GO:0015293 GO:GO:0005315 eggNOG:COG0477 InterPro:IPR016196 SUPFAM:SSF103473 EMBL:AC003033 InterPro:IPR020846 PROSITE:PS50850 EMBL:AC003974 HOGENOM:HBG744844 KO:K08176 TIGRFAMs:TIGR00887 EMBL:AB000093 EMBL:AY075694 EMBL:AK117670 IPI:IPI00543210 PIR:T01124 RefSeq:NP_180842.1 UniGene:At.38016 ProteinModelPortal:Q8GYF4 EnsemblPlants:AT2G32830.1 GeneID:817844 KEGG:ath:AT2G32830 TAIR:At2g32830 InParanoid:Q8GYF4 OMA:NISWRIM PhylomeDB:Q8GYF4 ProtClustDB:CLSN2913065 Genevestigator:Q8GYF4 GermOnline:AT2G32830 Uniprot:Q8GYF4
Root	Isotig02279	18	1	4.041	4.47E-05	No hit
Root	Isotig02280	18	1	4.041	4.47E-05	No hit
Root	Isotig02281	15	0	4.778	0.000105609	No hit
Root	Isotig02282	15	0	4.778	0.000105609	No hit
Root	Isotig02283	13	0	4.571	0.000344357	No hit
Root	Isotig02284	13	0	4.571	0.000344357	No hit
Root	Isotig02285	17	45	-1.533	7.67E-05	TAIR|locus:2082787 - symbol:AT3G61490 species:3702 "Arabidopsis thaliana" [GO:0004650 "polygalacturonase activity" evidence=IEA;ISS] [GO:0005975 "carbohydrate metabolic process" evidence=IEA;ISS] InterPro:IPR000743 Pfam:PF00295 EMBL:CP002686 GenomeReviews:BA000014_GR GO:GO:0005576 EMBL:AL132962 GO:GO:0005975 CAZy:GH28 eggNOG:COG5434 GO:GO:0004650 InterPro:IPR006626 InterPro:IPR012334 InterPro:IPR011050 Gene3D:G3DSA:2.160.20.10 SMART:SM00710 SUPFAM:SSF51126 GO:GO:0007047 HOGENOM:HBG604585 IPI:IPI00527942 PIR:T47941 RefSeq:NP_001190154.1 RefSeq:NP_191708.1 RefSeq:NP_974473.1 UniGene:At.43625 ProteinModelPortal:Q9M318 SMR:Q9M318 PRIDE:Q9M318 EnsemblPlants:AT3G61490.1 EnsemblPlants:AT3G61490.2 EnsemblPlants:AT3G61490.3 GeneID:825322 KEGG:ath:AT3G61490 TAIR:At3g61490 InParanoid:Q9M318 OMA:CISPYSA PhylomeDB:Q9M318 ProtClustDB:CLSN2915754 Genevestigator:Q9M318 Uniprot:Q9M318
Root	Isotig02286	18	46	-1.483	9.61E-05	TAIR|locus:2082787 - symbol:AT3G61490 species:3702 "Arabidopsis thaliana" [GO:0004650 "polygalacturonase activity" evidence=IEA;ISS] [GO:0005975 "carbohydrate metabolic process" evidence=IEA;ISS] InterPro:IPR000743 Pfam:PF00295 EMBL:CP002686 GenomeReviews:BA000014_GR GO:GO:0005576 EMBL:AL132962 GO:GO:0005975 CAZy:GH28 eggNOG:COG5434 GO:GO:0004650 InterPro:IPR006626 InterPro:IPR012334 InterPro:IPR011050 Gene3D:G3DSA:2.160.20.10 SMART:SM00710 SUPFAM:SSF51126 GO:GO:0007047 HOGENOM:HBG604585 IPI:IPI00527942 PIR:T47941 RefSeq:NP_001190154.1 RefSeq:NP_191708.1 RefSeq:NP_974473.1 UniGene:At.43625 ProteinModelPortal:Q9M318 SMR:Q9M318 PRIDE:Q9M318 EnsemblPlants:AT3G61490.1 EnsemblPlants:AT3G61490.2 EnsemblPlants:AT3G61490.3 GeneID:825322 KEGG:ath:AT3G61490 TAIR:At3g61490 InParanoid:Q9M318 OMA:CISPYSA PhylomeDB:Q9M318 ProtClustDB:CLSN2915754 Genevestigator:Q9M318 Uniprot:Q9M318
Root	Isotig02287	17	45	-1.533	7.67E-05	TAIR|locus:2082787 - symbol:AT3G61490 species:3702 "Arabidopsis thaliana" [GO:0004650 "polygalacturonase activity" evidence=IEA;ISS] [GO:0005975 "carbohydrate metabolic process" evidence=IEA;ISS] InterPro:IPR000743 Pfam:PF00295 EMBL:CP002686 GenomeReviews:BA000014_GR GO:GO:0005576 EMBL:AL132962 GO:GO:0005975 CAZy:GH28 eggNOG:COG5434 GO:GO:0004650 InterPro:IPR006626 InterPro:IPR012334 InterPro:IPR011050 Gene3D:G3DSA:2.160.20.10 SMART:SM00710 SUPFAM:SSF51126 GO:GO:0007047 HOGENOM:HBG604585 IPI:IPI00527942 PIR:T47941 RefSeq:NP_001190154.1 RefSeq:NP_191708.1 RefSeq:NP_974473.1 UniGene:At.43625 ProteinModelPortal:Q9M318 SMR:Q9M318 PRIDE:Q9M318 EnsemblPlants:AT3G61490.1 EnsemblPlants:AT3G61490.2 EnsemblPlants:AT3G61490.3 GeneID:825322 KEGG:ath:AT3G61490 TAIR:At3g61490 InParanoid:Q9M318 OMA:CISPYSA PhylomeDB:Q9M318 ProtClustDB:CLSN2915754 Genevestigator:Q9M318 Uniprot:Q9M318
Root	Isotig02288	17	45	-1.533	7.67E-05	TAIR|locus:2082787 - symbol:AT3G61490 species:3702 "Arabidopsis thaliana" [GO:0004650 "polygalacturonase activity" evidence=IEA;ISS] [GO:0005975 "carbohydrate metabolic process" evidence=IEA;ISS] InterPro:IPR000743 Pfam:PF00295 EMBL:CP002686 GenomeReviews:BA000014_GR GO:GO:0005576 EMBL:AL132962 GO:GO:0005975 CAZy:GH28 eggNOG:COG5434 GO:GO:0004650 InterPro:IPR006626 InterPro:IPR012334 InterPro:IPR011050 Gene3D:G3DSA:2.160.20.10 SMART:SM00710 SUPFAM:SSF51126 GO:GO:0007047 HOGENOM:HBG604585 IPI:IPI00527942 PIR:T47941 RefSeq:NP_001190154.1 RefSeq:NP_191708.1

Tissue	Identification	# of reads (stress)	# of reads (control)	Fold Change	p-value	Description
						RefSeq:NP_974473.1 UniGene:At.43625 ProteinModelPortal:Q9M318 SMR:Q9M318 PRIDE:Q9M318 EnsemblPlants:AT3G61490.1 EnsemblPlants:AT3G61490.2 EnsemblPlants:AT3G61490.3 GeneID:825322 KEGG:ath:AT3G61490 TAIR:At3g61490 InParanoid:Q9M318 OMA:CISPYSA PhylomeDB:Q9M318 ProtClustDB:CLSN2915754 Genevestigator:Q9M318 Uniprot:Q9M318
Root	Isotig02289	18	46	-1.483	9.61E-05	TAIR|locus:2082787 - symbol:AT3G61490 species:3702 "Arabidopsis thaliana" [GO:0004650 "polygalacturonase activity" evidence=IEA;ISS] [GO:0005975 "carbohydrate metabolic process" evidence=IEA;ISS] InterPro:IPR000743 Pfam:PF00295 EMBL:CP002686 GenomeReviews:BA000014_GR GO:GO:0005576 EMBL:AL132962 GO:GO:0005975 CAZy:GH28 eggNOG:COG5434 GO:GO:0004650 InterPro:IPR006626 InterPro:IPR012334 InterPro:IPR011050 Gene3D:G3DSA:2.160.20.10 SMART:SM00710 SUPFAM:SSF51126 GO:GO:0007047 HOGENOM:HBG604585 IPI:IPI00527942 PIR:T47941 RefSeq:NP_001190154.1 RefSeq:NP_191708.1 RefSeq:NP_974473.1 UniGene:At.43625 ProteinModelPortal:Q9M318 SMR:Q9M318 PRIDE:Q9M318 EnsemblPlants:AT3G61490.1 EnsemblPlants:AT3G61490.2 EnsemblPlants:AT3G61490.3 GeneID:825322 KEGG:ath:AT3G61490 TAIR:At3g61490 InParanoid:Q9M318 OMA:CISPYSA PhylomeDB:Q9M318 ProtClustDB:CLSN2915754 Genevestigator:Q9M318 Uniprot:Q9M318
Root	Isotig02290	17	45	-1.533	7.67E-05	TAIR|locus:2082787 - symbol:AT3G61490 species:3702 "Arabidopsis thaliana" [GO:0004650 "polygalacturonase activity" evidence=IEA;ISS] [GO:0005975 "carbohydrate metabolic process" evidence=IEA;ISS] InterPro:IPR000743 Pfam:PF00295 EMBL:CP002686 GenomeReviews:BA000014_GR GO:GO:0005576 EMBL:AL132962 GO:GO:0005975 CAZy:GH28 eggNOG:COG5434 GO:GO:0004650 InterPro:IPR006626 InterPro:IPR012334 InterPro:IPR011050 Gene3D:G3DSA:2.160.20.10 SMART:SM00710 SUPFAM:SSF51126 GO:GO:0007047 HOGENOM:HBG604585 IPI:IPI00527942 PIR:T47941 RefSeq:NP_001190154.1 RefSeq:NP_191708.1 RefSeq:NP_974473.1 UniGene:At.43625 ProteinModelPortal:Q9M318 SMR:Q9M318 PRIDE:Q9M318 EnsemblPlants:AT3G61490.1 EnsemblPlants:AT3G61490.2 EnsemblPlants:AT3G61490.3 GeneID:825322 KEGG:ath:AT3G61490 TAIR:At3g61490 InParanoid:Q9M318 OMA:CISPYSA PhylomeDB:Q9M318 ProtClustDB:CLSN2915754 Genevestigator:Q9M318 Uniprot:Q9M318
Root	Isotig02291	17	41	-1.399	0.000423111	TAIR|locus:2089668 - symbol:CYP77A5P ""cytochrome P450, family 77, subfamily A, polypeptide 5 pseudogene"" species:3702 "Arabidopsis thaliana" [GO:0003824 "catalytic activity" evidence=IEA] [GO:0008152 "metabolic process" evidence=IEA] [GO:0009507 "chloroplast" evidence=IDA] InterPro:IPR001354 Pfam:PF02746 GO:GO:0009507 GO:GO:0003824 eggNOG:COG4948 InterPro:IPR013342 InterPro:IPR013341 PANTHER:PTHR13794 SMART:SM00922 EMBL:AF370323 EMBL:AY063111 IPI:IPI00548779 RefSeq:NP_566605.1 UniGene:At.22113 UniGene:At.33094 HSSP:O34508 ProteinModelPortal:Q94K39 SMR:Q94K39 STRING:Q94K39 PRIDE:Q94K39 EnsemblPlants:AT3G18270.1 GeneID:821355 KEGG:ath:AT3G18270 TAIR:At3g18270 InParanoid:Q94K39 OMA:LYADANE PhylomeDB:Q94K39 ProtClustDB:CLSN2688525 ArrayExpress:Q94K39 Genevestigator:Q94K39 Uniprot:Q94K39
Root	Isotig02292	17	41	-1.399	0.000423111	TAIR|locus:2089668 - symbol:CYP77A5P ""cytochrome P450, family 77, subfamily A, polypeptide 5 pseudogene"" species:3702 "Arabidopsis thaliana" [GO:0003824 "catalytic activity" evidence=IEA] [GO:0008152 "metabolic process" evidence=IEA] [GO:0009507 "chloroplast" evidence=IDA] InterPro:IPR001354 Pfam:PF02746 GO:GO:0009507 GO:GO:0003824 eggNOG:COG4948 InterPro:IPR013342 InterPro:IPR013341 PANTHER:PTHR13794 SMART:SM00922 EMBL:AF370323 EMBL:AY063111 IPI:IPI00548779 RefSeq:NP_566605.1 UniGene:At.22113 UniGene:At.33094 HSSP:O34508 ProteinModelPortal:Q94K39 SMR:Q94K39 STRING:Q94K39 PRIDE:Q94K39 EnsemblPlants:AT3G18270.1 GeneID:821355 KEGG:ath:AT3G18270 TAIR:At3g18270 InParanoid:Q94K39 OMA:LYADANE PhylomeDB:Q94K39 ProtClustDB:CLSN2688525 ArrayExpress:Q94K39 Genevestigator:Q94K39 Uniprot:Q94K39
Root	Isotig02293	17	41	-1.399	0.000423111	TAIR|locus:2089668 - symbol:CYP77A5P ""cytochrome P450, family 77, subfamily A, polypeptide 5 pseudogene"" species:3702 "Arabidopsis thaliana" [GO:0003824 "catalytic activity" evidence=IEA] [GO:0008152 "metabolic process" evidence=IEA] [GO:0009507 "chloroplast" evidence=IDA] InterPro:IPR001354 Pfam:PF02746 GO:GO:0009507 GO:GO:0003824 eggNOG:COG4948 InterPro:IPR013342 InterPro:IPR013341 PANTHER:PTHR13794 SMART:SM00922 EMBL:AF370323 EMBL:AY063111 IPI:IPI00548779 RefSeq:NP_566605.1 UniGene:At.22113 UniGene:At.33094 HSSP:O34508 ProteinModelPortal:Q94K39 SMR:Q94K39 STRING:Q94K39 PRIDE:Q94K39 EnsemblPlants:AT3G18270.1 GeneID:821355 KEGG:ath:AT3G18270 TAIR:At3g18270 InParanoid:Q94K39 OMA:LYADANE PhylomeDB:Q94K39 ProtClustDB:CLSN2688525 ArrayExpress:Q94K39 Genevestigator:Q94K39 Uniprot:Q94K39

Tissue	Identification	# of reads (stress)	# of reads (control)	Fold Change	p-value	Description
Root	Isotig02294	16	41	-1.487	0.000224899	TAIR|locus:2089668 - symbol:CYP77A5P ""cytochrome P450, family 77, subfamily A, polypeptide 5 pseudogene"" species:3702 "Arabidopsis thaliana" [GO:0003824 "catalytic activity" evidence=IEA] [GO:0008152 "metabolic process" evidence=IEA] [GO:0009507 "chloroplast" evidence=IDA] InterPro:IPR001354 Pfam:PF02746 GO:GO:0009507 GO:GO:0003824 eggNOG:COG4948 InterPro:IPR013342 InterPro:IPR013341 PANTHER:PTHR13794 SMART:SM00922 EMBL:AF370323 EMBL:AY063111 IPI:IPI00548779 RefSeq:NP_566605.1 UniGene:At.22113 UniGene:At.33094 HSSP:O34508 ProteinModelPortal:Q94K39 SMR:Q94K39 STRING:Q94K39 PRIDE:Q94K39 EnsemblPlants:AT3G18270.1 GeneID:821355 KEGG:ath:AT3G18270 TAIR:At3g18270 InParanoid:Q94K39 OMA:LYADANE PhylomeDB:Q94K39 ProtClustDB:CLSN2688525 ArrayExpress:Q94K39 Genevestigator:Q94K39 Uniprot:Q94K39
Root	Isotig02295	17	41	-1.399	0.000423111	TAIR|locus:2089668 - symbol:CYP77A5P ""cytochrome P450, family 77, subfamily A, polypeptide 5 pseudogene"" species:3702 "Arabidopsis thaliana" [GO:0003824 "catalytic activity" evidence=IEA] [GO:0008152 "metabolic process" evidence=IEA] [GO:0009507 "chloroplast" evidence=IDA] InterPro:IPR001354 Pfam:PF02746 GO:GO:0009507 GO:GO:0003824 eggNOG:COG4948 InterPro:IPR013342 InterPro:IPR013341 PANTHER:PTHR13794 SMART:SM00922 EMBL:AF370323 EMBL:AY063111 IPI:IPI00548779 RefSeq:NP_566605.1 UniGene:At.22113 UniGene:At.33094 HSSP:O34508 ProteinModelPortal:Q94K39 SMR:Q94K39 STRING:Q94K39 PRIDE:Q94K39 EnsemblPlants:AT3G18270.1 GeneID:821355 KEGG:ath:AT3G18270 TAIR:At3g18270 InParanoid:Q94K39 OMA:LYADANE PhylomeDB:Q94K39 ProtClustDB:CLSN2688525 ArrayExpress:Q94K39 Genevestigator:Q94K39 Uniprot:Q94K39
Root	Isotig02296	16	41	-1.487	0.000224899	TAIR|locus:2089668 - symbol:CYP77A5P ""cytochrome P450, family 77, subfamily A, polypeptide 5 pseudogene"" species:3702 "Arabidopsis thaliana" [GO:0003824 "catalytic activity" evidence=IEA] [GO:0008152 "metabolic process" evidence=IEA] [GO:0009507 "chloroplast" evidence=IDA] InterPro:IPR001354 Pfam:PF02746 GO:GO:0009507 GO:GO:0003824 eggNOG:COG4948 InterPro:IPR013342 InterPro:IPR013341 PANTHER:PTHR13794 SMART:SM00922 EMBL:AF370323 EMBL:AY063111 IPI:IPI00548779 RefSeq:NP_566605.1 UniGene:At.22113 UniGene:At.33094 HSSP:O34508 ProteinModelPortal:Q94K39 SMR:Q94K39 STRING:Q94K39 PRIDE:Q94K39 EnsemblPlants:AT3G18270.1 GeneID:821355 KEGG:ath:AT3G18270 TAIR:At3g18270 InParanoid:Q94K39 OMA:LYADANE PhylomeDB:Q94K39 ProtClustDB:CLSN2688525 ArrayExpress:Q94K39 Genevestigator:Q94K39 Uniprot:Q94K39
Root	Isotig02297	10	39	-2.092	5.12E-06	TAIR|locus:2178128 - symbol:ACT7 "AT5G09810" species:3702 "Arabidopsis thaliana" [GO:0007010 "cytoskeleton organization" evidence=TAS] [GO:0009733 "response to auxin stimulus" evidence=IEP] [GO:0009845 "seed germination" evidence=IMP] [GO:0048364 "root development" evidence=IMP] [GO:0005739 "mitochondrion" evidence=IDA] [GO:0005730 "nucleolus" evidence=IDA] [GO:0005618 "cell wall" evidence=IDA] [GO:0005886 "plasma membrane" evidence=IDA] [GO:0010053 "root epidermal cell differentiation" evidence=IMP] [GO:0048767 "root hair elongation" evidence=IMP] [GO:0051301 "cell division" evidence=IMP] [GO:0005515 "protein binding" evidence=IPI] [GO:0009570 "chloroplast stroma" evidence=IDA] [GO:0009941 "chloroplast envelope" evidence=IDA] [GO:0005829 "cytosol" evidence=IDA] [GO:0009506 "plasmodesma" evidence=IDA] [GO:0005200 "structural constituent of cytoskeleton" evidence=ISS] [GO:0005856 "cytoskeleton" evidence=ISS] [GO:0009416 "response to light stimulus" evidence=IEP] [GO:0009611 "response to wounding" evidence=IEP] InterPro:IPR004001 PROSITE:PS00406 PROSITE:PS00432 InterPro:IPR004000 GO:GO:0005829 GO:GO:0005739 GO:GO:0005886 GO:GO:0009506 GO:GO:0005524 GO:GO:0009570 EMBL:CP002688 GenomeReviews:BA000015_GR GO:GO:0005618 GO:GO:0005515 GO:GO:0009733 GO:GO:0009611 GO:GO:0005730 GO:GO:0005856 GO:GO:0051301 GO:GO:0009941 EMBL:AB016893 GO:GO:0048767 GO:GO:0009845 GO:GO:0009416 HOGENOM:HBG559892 PANTHER:PTHR11937 Pfam:PF00022 PRINTS:PR00190 SMART:SM00268 KO:K10355 InterPro:IPR020902 PROSITE:PS01132 ProtClustDB:CLSN2682469 UniGene:At.23346 EMBL:U37281 EMBL:U27811 EMBL:AY062702 EMBL:AY063980 EMBL:AY096397 EMBL:AY114679 EMBL:AY120779 IPI:IPI00524611 PIR:S68107 RefSeq:NP_196543.1 UniGene:At.23605 UniGene:At.24396 ProteinModelPortal:P53492 SMR:P53492 IntAct:P53492 STRING:P53492 PRIDE:P53492 ProMEX:P53492 EnsemblPlants:AT5G09810.1 GeneID:830841 KEGG:ath:AT5G09810 TAIR:At5g09810 InParanoid:P53492 OMA:NSICVIL PhylomeDB:P53492 ArrayExpress:P53492 Genevestigator:P53492 GermOnline:AT5G09810 Uniprot:P53492
Root	Isotig02298	13	40	-1.750	4.15E-05	TAIR|locus:2178128 - symbol:ACT7 "AT5G09810" species:3702 "Arabidopsis thaliana" [GO:0007010 "cytoskeleton organization" evidence=TAS] [GO:0009733 "response to auxin stimulus" evidence=IEP] [GO:0009845 "seed germination" evidence=IMP] [GO:0048364 "root development" evidence=IMP] [GO:0005739 "mitochondrion"

Tissue	Identification	# of reads (stress)	# of reads (control)	Fold Change	p-value	Description
						evidence=IDA] [GO:0005730 "nucleolus" evidence=IDA] [GO:0005618 "cell wall" evidence=IDA] [GO:0005886 "plasma membrane" evidence=IDA] [GO:0010053 "root epidermal cell differentiation" evidence=IMP] [GO:0048767 "root hair elongation" evidence=IMP] [GO:0051301 "cell division" evidence=IMP] [GO:0005515 "protein binding" evidence=IPI] [GO:0009570 "chloroplast stroma" evidence=IDA] [GO:0009941 "chloroplast envelope" evidence=IDA] [GO:0005829 "cytosol" evidence=IDA] [GO:0009506 "plasmodesma" evidence=IDA] [GO:0005200 "structural constituent of cytoskeleton" evidence=ISS] [GO:0005856 "cytoskeleton" evidence=ISS] [GO:0009416 "response to light stimulus" evidence=IEP] [GO:0009611 "response to wounding" evidence=IEP] InterPro:IPR004001 PROSITE:PS00406 PROSITE:PS00432 InterPro:IPR004000 GO:GO:0005829 GO:GO:0005739 GO:GO:0005886 GO:GO:0009506 GO:GO:0005524 GO:GO:0009570 EMBL:CP002688 GenomeReviews:BA000015_GR GO:GO:0005618 GO:GO:0005515 GO:GO:0009733 GO:GO:0009611 GO:GO:0005730 GO:GO:0005856 GO:GO:0051301 GO:GO:0009941 EMBL:AB016893 GO:GO:0048767 GO:GO:0009845 GO:GO:0009416 HOGENOM:HBG559892 PANTHER:PTHR11937 Pfam:PF00022 PRINTS:PR00190 SMART:SM00268 KO:K10355 InterPro:IPR020902 PROSITE:PS01132 ProtClustDB:CLSN2682469 UniGene:At.23346 EMBL:U37281 EMBL:U27811 EMBL:AY062702 EMBL:AY063980 EMBL:AY096397 EMBL:AY114679 EMBL:AY120779 IPI:IPI00524611 PIR:S68107 RefSeq:NP_196543.1 UniGene:At.23605 UniGene:At.24396 ProteinModelPortal:P53492 SMR:P53492 IntAct:P53492 STRING:P53492 PRIDE:P53492 ProMEX:P53492 EnsemblPlants:AT5G09810.1 GeneID:830841 KEGG:ath:AT5G09810 TAIR:At5g09810 InParanoid:P53492 OMA:NSICVIL PhylomeDB:P53492 ArrayExpress:P53492 Genevestigator:P53492 GermOnline:AT5G09810 Uniprot:P53492
Root	Isotig02299	10	39	-2.092	5.12E-06	TAIR|locus:2178128 - symbol:ACT7 "AT5G09810" species:3702 "Arabidopsis thaliana" [GO:0007010 "cytoskeleton organization" evidence=TAS] [GO:0009733 "response to auxin stimulus" evidence=IEP] [GO:0009845 "seed germination" evidence=IMP] [GO:0048364 "root development" evidence=IMP] [GO:0005739 "mitochondrion" evidence=IDA] [GO:0005730 "nucleolus" evidence=IDA] [GO:0005618 "cell wall" evidence=IDA] [GO:0005886 "plasma membrane" evidence=IDA] [GO:0010053 "root epidermal cell differentiation" evidence=IMP] [GO:0048767 "root hair elongation" evidence=IMP] [GO:0051301 "cell division" evidence=IMP] [GO:0005515 "protein binding" evidence=IPI] [GO:0009570 "chloroplast stroma" evidence=IDA] [GO:0009941 "chloroplast envelope" evidence=IDA] [GO:0005829 "cytosol" evidence=IDA] [GO:0009506 "plasmodesma" evidence=IDA] [GO:0005200 "structural constituent of cytoskeleton" evidence=ISS] [GO:0005856 "cytoskeleton" evidence=ISS] [GO:0009416 "response to light stimulus" evidence=IEP] [GO:0009611 "response to wounding" evidence=IEP] InterPro:IPR004001 PROSITE:PS00406 PROSITE:PS00432 InterPro:IPR004000 GO:GO:0005829 GO:GO:0005739 GO:GO:0005886 GO:GO:0009506 GO:GO:0005524 GO:GO:0009570 EMBL:CP002688 GenomeReviews:BA000015_GR GO:GO:0005618 GO:GO:0005515 GO:GO:0009733 GO:GO:0009611 GO:GO:0005730 GO:GO:0005856 GO:GO:0051301 GO:GO:0009941 EMBL:AB016893 GO:GO:0048767 GO:GO:0009845 GO:GO:0009416 HOGENOM:HBG559892 PANTHER:PTHR11937 Pfam:PF00022 PRINTS:PR00190 SMART:SM00268 KO:K10355 InterPro:IPR020902 PROSITE:PS01132 ProtClustDB:CLSN2682469 UniGene:At.23346 EMBL:U37281 EMBL:U27811 EMBL:AY062702 EMBL:AY063980 EMBL:AY096397 EMBL:AY114679 EMBL:AY120779 IPI:IPI00524611 PIR:S68107 RefSeq:NP_196543.1 UniGene:At.23605 UniGene:At.24396 ProteinModelPortal:P53492 SMR:P53492 IntAct:P53492 STRING:P53492 PRIDE:P53492 ProMEX:P53492 EnsemblPlants:AT5G09810.1 GeneID:830841 KEGG:ath:AT5G09810 TAIR:At5g09810 InParanoid:P53492 OMA:NSICVIL PhylomeDB:P53492 ArrayExpress:P53492 Genevestigator:P53492 GermOnline:AT5G09810 Uniprot:P53492
Root	Isotig02300	13	40	-1.750	4.15E-05	TAIR|locus:2178128 - symbol:ACT7 "AT5G09810" species:3702 "Arabidopsis thaliana" [GO:0007010 "cytoskeleton organization" evidence=TAS] [GO:0009733 "response to auxin stimulus" evidence=IEP] [GO:0009845 "seed germination" evidence=IMP] [GO:0048364 "root development" evidence=IMP] [GO:0005739 "mitochondrion" evidence=IDA] [GO:0005730 "nucleolus" evidence=IDA] [GO:0005618 "cell wall" evidence=IDA] [GO:0005886 "plasma membrane" evidence=IDA] [GO:0010053 "root epidermal cell differentiation" evidence=IMP] [GO:0048767 "root hair elongation" evidence=IMP] [GO:0051301 "cell division" evidence=IMP] [GO:0005515 "protein binding" evidence=IPI] [GO:0009570 "chloroplast stroma" evidence=IDA] [GO:0009941 "chloroplast envelope" evidence=IDA] [GO:0005829 "cytosol" evidence=IDA] [GO:0009506 "plasmodesma" evidence=IDA] [GO:0005200 "structural constituent of cytoskeleton" evidence=ISS] [GO:0005856 "cytoskeleton" evidence=ISS] [GO:0009416 "response to light stimulus" evidence=IEP] [GO:0009611 "response to wounding" evidence=IEP] InterPro:IPR004001 PROSITE:PS00406 PROSITE:PS00432 InterPro:IPR004000 GO:GO:0005829 GO:GO:0005739 GO:GO:0005886 GO:GO:0009506 GO:GO:0005524 GO:GO:0009570 EMBL:CP002688 GenomeReviews:BA000015_GR

Tissue	Identification	# of reads (stress)	# of reads (control)	Fold Change	p-value	Description
						GO:GO:0005618 GO:GO:0005515 GO:GO:0009733 GO:GO:0009611 GO:GO:0005730 GO:GO:0005856 GO:GO:0051301 GO:GO:0009941 EMBL:AB016893 GO:GO:0048767 GO:GO:0009845 GO:GO:0009416 HOGENOM:HBG559892 PANTHER:PTHR11937 Pfam:PF00022 PRINTS:PR00190 SMART:SM00268 KO:K10355 InterPro:IPR020902 PROSITE:PS01132 ProtClustDB:CLSN2682469 UniGene:At.23346 EMBL:U37281 EMBL:U27811 EMBL:AY062702 EMBL:AY063980 EMBL:AY096397 EMBL:AY114679 EMBL:AY120779 IPI:IPI00524611 PIR:S68107 RefSeq:NP_196543.1 UniGene:At.23605 UniGene:At.24396 ProteinModelPortal:P53492 SMR:P53492 IntAct:P53492 STRING:P53492 PRIDE:P53492 ProMEX:P53492 EnsemblPlants:AT5G09810.1 GeneID:830841 KEGG:ath:AT5G09810 TAIR:At5g09810 InParanoid:P53492 OMA:NSICVIL PhylomeDB:P53492 ArrayExpress:P53492 Genevestigator:P53492 GermOnline:AT5G09810 Uniprot:P53492
Root	Isotig02301	9	40	-2.281	1.10E-06	TAIR|locus:2178128 - symbol:ACT7 "AT5G09810" species:3702 "Arabidopsis thaliana" [GO:0007010 "cytoskeleton organization" evidence=TAS] [GO:0009733 "response to auxin stimulus" evidence=IEP] [GO:0009845 "seed germination" evidence=IMP] [GO:0048364 "root development" evidence=IMP] [GO:0005739 "mitochondrion" evidence=IDA] [GO:0005730 "nucleolus" evidence=IDA] [GO:0005618 "cell wall" evidence=IDA] [GO:0005886 "plasma membrane" evidence=IDA] [GO:0010053 "root epidermal cell differentiation" evidence=IMP] [GO:0048767 "root hair elongation" evidence=IMP] [GO:0051301 "cell division" evidence=IMP] [GO:0005515 "protein binding" evidence=IPI] [GO:0009570 "chloroplast stroma" evidence=IDA] [GO:0009941 "chloroplast envelope" evidence=IDA] [GO:0005829 "cytosol" evidence=IDA] [GO:0009506 "plasmodesma" evidence=IDA] [GO:0005200 "structural constituent of cytoskeleton" evidence=ISS] [GO:0005856 "cytoskeleton" evidence=ISS] [GO:0009416 "response to light stimulus" evidence=IEP] [GO:0009611 "response to wounding" evidence=IEP] InterPro:IPR004001 PROSITE:PS00406 PROSITE:PS00432 InterPro:IPR004000 GO:GO:0005829 GO:GO:0005739 GO:GO:0005886 GO:GO:0009506 GO:GO:0005524 GO:GO:0009570 EMBL:CP002688 GenomeReviews:BA000015_GR GO:GO:0005618 GO:GO:0005515 GO:GO:0009733 GO:GO:0009611 GO:GO:0005730 GO:GO:0005856 GO:GO:0051301 GO:GO:0009941 EMBL:AB016893 GO:GO:0048767 GO:GO:0009845 GO:GO:0009416 HOGENOM:HBG559892 PANTHER:PTHR11937 Pfam:PF00022 PRINTS:PR00190 SMART:SM00268 KO:K10355 InterPro:IPR020902 PROSITE:PS01132 ProtClustDB:CLSN2682469 UniGene:At.23346 EMBL:U37281 EMBL:U27811 EMBL:AY062702 EMBL:AY063980 EMBL:AY096397 EMBL:AY114679 EMBL:AY120779 IPI:IPI00524611 PIR:S68107 RefSeq:NP_196543.1 UniGene:At.23605 UniGene:At.24396 ProteinModelPortal:P53492 SMR:P53492 IntAct:P53492 STRING:P53492 PRIDE:P53492 ProMEX:P53492 EnsemblPlants:AT5G09810.1 GeneID:830841 KEGG:ath:AT5G09810 TAIR:At5g09810 InParanoid:P53492 OMA:NSICVIL PhylomeDB:P53492 ArrayExpress:P53492 Genevestigator:P53492 GermOnline:AT5G09810 Uniprot:P53492
Root	Isotig02302	9	40	-2.281	1.10E-06	TAIR|locus:2178128 - symbol:ACT7 "AT5G09810" species:3702 "Arabidopsis thaliana" [GO:0007010 "cytoskeleton organization" evidence=TAS] [GO:0009733 "response to auxin stimulus" evidence=IEP] [GO:0009845 "seed germination" evidence=IMP] [GO:0048364 "root development" evidence=IMP] [GO:0005739 "mitochondrion" evidence=IDA] [GO:0005730 "nucleolus" evidence=IDA] [GO:0005618 "cell wall" evidence=IDA] [GO:0005886 "plasma membrane" evidence=IDA] [GO:0010053 "root epidermal cell differentiation" evidence=IMP] [GO:0048767 "root hair elongation" evidence=IMP] [GO:0051301 "cell division" evidence=IMP] [GO:0005515 "protein binding" evidence=IPI] [GO:0009570 "chloroplast stroma" evidence=IDA] [GO:0009941 "chloroplast envelope" evidence=IDA] [GO:0005829 "cytosol" evidence=IDA] [GO:0009506 "plasmodesma" evidence=IDA] [GO:0005200 "structural constituent of cytoskeleton" evidence=ISS] [GO:0005856 "cytoskeleton" evidence=ISS] [GO:0009416 "response to light stimulus" evidence=IEP] [GO:0009611 "response to wounding" evidence=IEP] InterPro:IPR004001 PROSITE:PS00406 PROSITE:PS00432 InterPro:IPR004000 GO:GO:0005829 GO:GO:0005739 GO:GO:0005886 GO:GO:0009506 GO:GO:0005524 GO:GO:0009570 EMBL:CP002688 GenomeReviews:BA000015_GR GO:GO:0005618 GO:GO:0005515 GO:GO:0009733 GO:GO:0009611 GO:GO:0005730 GO:GO:0005856 GO:GO:0051301 GO:GO:0009941 EMBL:AB016893 GO:GO:0048767 GO:GO:0009845 GO:GO:0009416 HOGENOM:HBG559892 PANTHER:PTHR11937 Pfam:PF00022 PRINTS:PR00190 SMART:SM00268 KO:K10355 InterPro:IPR020902 PROSITE:PS01132 ProtClustDB:CLSN2682469 UniGene:At.23346 EMBL:U37281 EMBL:U27811 EMBL:AY062702 EMBL:AY063980 EMBL:AY096397 EMBL:AY114679 EMBL:AY120779 IPI:IPI00524611 PIR:S68107 RefSeq:NP_196543.1 UniGene:At.23605 UniGene:At.24396 ProteinModelPortal:P53492 SMR:P53492 IntAct:P53492 STRING:P53492 PRIDE:P53492 ProMEX:P53492 EnsemblPlants:AT5G09810.1 GeneID:830841 KEGG:ath:AT5G09810 TAIR:At5g09810 InParanoid:P53492

Tissue	Identification	# of reads (stress)	# of reads (control)	Fold Change	p-value	Description
						OMA:NSICVIL PhylomeDB:P53492 ArrayExpress:P53492 Genevestigator:P53492 GermOnline:AT5G09810 Uniprot:P53492
Root	Isotig02357	14	36	-1.492	0.000528966	TAIR|locus:2012572 - symbol:MIOX1 "myo-inositol oxygenase 1" species:3702 "Arabidopsis thaliana" [GO:0005737 "cytoplasm" evidence=IEA] [GO:0016491 "oxidoreductase activity" evidence=ISS] [GO:0050113 "inositol oxygenase activity" evidence=IMP] [GO:0006949 "syncytium formation" evidence=IGI] InterPro:IPR007828 Pfam:PF05153 EMBL:CP002684 GenomeReviews:CT485782_GR GO:GO:0005737 GO:GO:0005506 EMBL:AC010657 EMBL:AC012188 GO:GO:0006949 GO:GO:0019853 GO:GO:0019310 EMBL:AY136388 EMBL:BT000187 EMBL:AK118307 EMBL:AK175115 EMBL:AK175818 EMBL:AK175833 EMBL:AK176690 EMBL:AK221931 IPI:IPI00531494 RefSeq:NP_172904.2 UniGene:At.23143 ProteinModelPortal:Q8L799 SMR:Q8L799 PRIDE:Q8L799 EnsemblPlants:AT1G14520.1 GeneID:838014 KEGG:ath:AT1G14520 TAIR:At1g14520 eggNOG:NOG135479 HOGENOM:HBG332059 InParanoid:Q8L799 KO:K00469 OMA:CELLNEF PhylomeDB:Q8L799 ProtClustDB:CLSN2687348 BioCyc:MetaCyc:AT1G14520-MONOMER Genevestigator:Q8L799 GO:GO:0050113 PANTHER:PTHR12588 Uniprot:Q8L799
Root	Isotig02358	14	36	-1.492	0.000528966	TAIR|locus:2012572 - symbol:MIOX1 "myo-inositol oxygenase 1" species:3702 "Arabidopsis thaliana" [GO:0005737 "cytoplasm" evidence=IEA] [GO:0016491 "oxidoreductase activity" evidence=ISS] [GO:0050113 "inositol oxygenase activity" evidence=IMP] [GO:0006949 "syncytium formation" evidence=IGI] InterPro:IPR007828 Pfam:PF05153 EMBL:CP002684 GenomeReviews:CT485782_GR GO:GO:0005737 GO:GO:0005506 EMBL:AC010657 EMBL:AC012188 GO:GO:0006949 GO:GO:0019853 GO:GO:0019310 EMBL:AY136388 EMBL:BT000187 EMBL:AK118307 EMBL:AK175115 EMBL:AK175818 EMBL:AK175833 EMBL:AK176690 EMBL:AK221931 IPI:IPI00531494 RefSeq:NP_172904.2 UniGene:At.23143 ProteinModelPortal:Q8L799 SMR:Q8L799 PRIDE:Q8L799 EnsemblPlants:AT1G14520.1 GeneID:838014 KEGG:ath:AT1G14520 TAIR:At1g14520 eggNOG:NOG135479 HOGENOM:HBG332059 InParanoid:Q8L799 KO:K00469 OMA:CELLNEF PhylomeDB:Q8L799 ProtClustDB:CLSN2687348 BioCyc:MetaCyc:AT1G14520-MONOMER Genevestigator:Q8L799 GO:GO:0050113 PANTHER:PTHR12588 Uniprot:Q8L799
Root	Isotig02359	14	37	-1.531	0.000341051	TAIR|locus:2012572 - symbol:MIOX1 "myo-inositol oxygenase 1" species:3702 "Arabidopsis thaliana" [GO:0005737 "cytoplasm" evidence=IEA] [GO:0016491 "oxidoreductase activity" evidence=ISS] [GO:0050113 "inositol oxygenase activity" evidence=IMP] [GO:0006949 "syncytium formation" evidence=IGI] InterPro:IPR007828 Pfam:PF05153 EMBL:CP002684 GenomeReviews:CT485782_GR GO:GO:0005737 GO:GO:0005506 EMBL:AC010657 EMBL:AC012188 GO:GO:0006949 GO:GO:0019853 GO:GO:0019310 EMBL:AY136388 EMBL:BT000187 EMBL:AK118307 EMBL:AK175115 EMBL:AK175818 EMBL:AK175833 EMBL:AK176690 EMBL:AK221931 IPI:IPI00531494 RefSeq:NP_172904.2 UniGene:At.23143 ProteinModelPortal:Q8L799 SMR:Q8L799 PRIDE:Q8L799 EnsemblPlants:AT1G14520.1 GeneID:838014 KEGG:ath:AT1G14520 TAIR:At1g14520 eggNOG:NOG135479 HOGENOM:HBG332059 InParanoid:Q8L799 KO:K00469 OMA:CELLNEF PhylomeDB:Q8L799 ProtClustDB:CLSN2687348 BioCyc:MetaCyc:AT1G14520-MONOMER Genevestigator:Q8L799 GO:GO:0050113 PANTHER:PTHR12588 Uniprot:Q8L799
Root	Isotig02360	14	37	-1.531	0.000341051	TAIR|locus:2012572 - symbol:MIOX1 "myo-inositol oxygenase 1" species:3702 "Arabidopsis thaliana" [GO:0005737 "cytoplasm" evidence=IEA] [GO:0016491 "oxidoreductase activity" evidence=ISS] [GO:0050113 "inositol oxygenase activity" evidence=IMP] [GO:0006949 "syncytium formation" evidence=IGI] InterPro:IPR007828 Pfam:PF05153 EMBL:CP002684 GenomeReviews:CT485782_GR GO:GO:0005737 GO:GO:0005506 EMBL:AC010657 EMBL:AC012188 GO:GO:0006949 GO:GO:0019853 GO:GO:0019310 EMBL:AY136388 EMBL:BT000187 EMBL:AK118307 EMBL:AK175115 EMBL:AK175818 EMBL:AK175833 EMBL:AK176690 EMBL:AK221931 IPI:IPI00531494 RefSeq:NP_172904.2 UniGene:At.23143 ProteinModelPortal:Q8L799 SMR:Q8L799 PRIDE:Q8L799 EnsemblPlants:AT1G14520.1 GeneID:838014 KEGG:ath:AT1G14520 TAIR:At1g14520 eggNOG:NOG135479 HOGENOM:HBG332059 InParanoid:Q8L799 KO:K00469 OMA:CELLNEF PhylomeDB:Q8L799 ProtClustDB:CLSN2687348 BioCyc:MetaCyc:AT1G14520-MONOMER Genevestigator:Q8L799 GO:GO:0050113 PANTHER:PTHR12588 Uniprot:Q8L799
Root	Isotig02361	14	36	-1.492	0.000528966	TAIR|locus:2012572 - symbol:MIOX1 "myo-inositol oxygenase 1" species:3702 "Arabidopsis thaliana" [GO:0005737 "cytoplasm" evidence=IEA] [GO:0016491 "oxidoreductase activity" evidence=ISS] [GO:0050113 "inositol oxygenase activity" evidence=IMP] [GO:0006949 "syncytium formation" evidence=IGI] InterPro:IPR007828 Pfam:PF05153 EMBL:CP002684 GenomeReviews:CT485782_GR GO:GO:0005737 GO:GO:0005506 EMBL:AC010657 EMBL:AC012188 GO:GO:0006949 GO:GO:0019853 GO:GO:0019310 EMBL:AY136388 EMBL:BT000187 EMBL:AK118307 EMBL:AK175115 EMBL:AK175818 EMBL:AK175833 EMBL:AK176690 EMBL:AK221931

Tissue	Identification	# of reads (stress)	# of reads (control)	Fold Change	p-value	Description
						IPI:IPI00531494 RefSeq:NP_172904.2 UniGene:At.23143 ProteinModelPortal:Q8L799 SMR:Q8L799 PRIDE:Q8L799 EnsemblPlants:AT1G14520.1 GeneID:838014 KEGG:ath:AT1G14520 TAIR:At1g14520 eggNOG:NOG135479 HOGENOM:HBG332059 InParanoid:Q8L799 KO:K00469 OMA:CELLNEF PhylomeDB:Q8L799 ProtClustDB:CLSN2687348 BioCyc:MetaCyc:AT1G14520-MONOMER Genevestigator:Q8L799 GO:GO:0050113 PANTHER:PTHR12588 Uniprot:Q8L799
Root	Isotig02362	14	36	-1.492	0.000528966	TAIR|locus:2012572 - symbol:MIOX1 "myo-inositol oxygenase 1" species:3702 "Arabidopsis thaliana" [GO:0005737 "cytoplasm" evidence=IEA] [GO:0016491 "oxidoreductase activity" evidence=ISS] [GO:0050113 "inositol oxygenase activity" evidence=IMP] [GO:0006949 "syncytium formation" evidence=IGI] InterPro:IPR007828 Pfam:PF05153 EMBL:CP002684 GenomeReviews:CT485782_GR GO:GO:0005737 GO:GO:0005506 EMBL:AC010657 EMBL:AC012188 GO:GO:0006949 GO:GO:0019853 GO:GO:0019310 EMBL:AY136388 EMBL:BT000187 EMBL:AK118307 EMBL:AK175115 EMBL:AK175818 EMBL:AK175833 EMBL:AK176690 EMBL:AK221931 IPI:IPI00531494 RefSeq:NP_172904.2 UniGene:At.23143 ProteinModelPortal:Q8L799 SMR:Q8L799 PRIDE:Q8L799 EnsemblPlants:AT1G14520.1 GeneID:838014 KEGG:ath:AT1G14520 TAIR:At1g14520 eggNOG:NOG135479 HOGENOM:HBG332059 InParanoid:Q8L799 KO:K00469 OMA:CELLNEF PhylomeDB:Q8L799 ProtClustDB:CLSN2687348 BioCyc:MetaCyc:AT1G14520-MONOMER Genevestigator:Q8L799 GO:GO:0050113 PANTHER:PTHR12588 Uniprot:Q8L799
Root	Isotig02387	2	20	-3.451	2.08E-05	TAIR|locus:2195341 - symbol:AT1G09200 species:3702 "Arabidopsis thaliana" [GO:0003677 "DNA binding" evidence=IEA;ISS] [GO:0006334 "nucleosome assembly" evidence=IEA] [GO:0005515 "protein binding" evidence=IPI] InterPro:IPR000164 InterPro:IPR007125 InterPro:IPR009072 Pfam:PF00125 PRINTS:PR00622 PROSITE:PS00322 PROSITE:PS00959 SMART:SM00428 EMBL:CP002684 GenomeReviews:CT485782_GR GO:GO:0005634 EMBL:CP002688 GenomeReviews:BA000015_GR EMBL:AL353995 EMBL:CP002686 GenomeReviews:BA000014_GR EMBL:AB011479 GO:GO:0005515 GO:GO:0003677 Gene3D:G3DSA:1.10.20.10 SUPFAM:SSF47113 GO:GO:0006334 UniGene:At.1824 GO:GO:0000786 EMBL:AC003114 eggNOG:COG2036 PANTHER:PTHR11426 HOGENOM:HBG715487 EMBL:AB024028 UniGene:At.17610 UniGene:At.22540 UniGene:At.32384 UniGene:At.71559 KO:K11253 OMA:DLRFQSQ EMBL:M35387 EMBL:M17131 EMBL:M17130 EMBL:AK117157 EMBL:AF370577 EMBL:AY037250 EMBL:AY039904 EMBL:AY077654 EMBL:AY081741 EMBL:BT003051 EMBL:BT003162 EMBL:BT005805 EMBL:BT006068 EMBL:AY084316 IPI:IPI00517184 PIR:S06250 RefSeq:NP_189372.1 RefSeq:NP_201339.1 RefSeq:NP_563838.1 RefSeq:NP_568227.1 RefSeq:NP_568228.1 UniGene:At.20804 UniGene:At.21310 UniGene:At.21486 UniGene:At.63772 UniGene:At.73076 ProteinModelPortal:P59226 SMR:P59226 DIP:DIP-48531N STRING:P59226 PRIDE:P59226 EnsemblPlants:AT1G09200.1 EnsemblPlants:AT3G27360.1 EnsemblPlants:AT5G10390.1 EnsemblPlants:AT5G10400.1 EnsemblPlants:AT5G65360.1 GeneID:822357 GeneID:830903 GeneID:830904 GeneID:836661 GeneID:837440 KEGG:ath:AT1G09200 KEGG:ath:AT3G27360 KEGG:ath:AT5G10390 KEGG:ath:AT5G10400 KEGG:ath:AT5G65360 TAIR:At1g09200 TAIR:At3g27360 TAIR:At5g10390 TAIR:At5g10400 TAIR:At5g65360 InParanoid:P59226 PhylomeDB:P59226 ProtClustDB:PLN00121 ArrayExpress:P59226 Genevestigator:P59226 GermOnline:AT1G09200 Uniprot:P59226
Root	Isotig02388	2	20	-3.451	2.08E-05	TAIR|locus:2195341 - symbol:AT1G09200 species:3702 "Arabidopsis thaliana" [GO:0003677 "DNA binding" evidence=IEA;ISS] [GO:0006334 "nucleosome assembly" evidence=IEA] [GO:0005515 "protein binding" evidence=IPI] InterPro:IPR000164 InterPro:IPR007125 InterPro:IPR009072 Pfam:PF00125 PRINTS:PR00622 PROSITE:PS00322 PROSITE:PS00959 SMART:SM00428 EMBL:CP002684 GenomeReviews:CT485782_GR GO:GO:0005634 EMBL:CP002688 GenomeReviews:BA000015_GR EMBL:AL353995 EMBL:CP002686 GenomeReviews:BA000014_GR EMBL:AB011479 GO:GO:0005515 GO:GO:0003677 Gene3D:G3DSA:1.10.20.10 SUPFAM:SSF47113 GO:GO:0006334 UniGene:At.1824 GO:GO:0000786 EMBL:AC003114 eggNOG:COG2036 PANTHER:PTHR11426 HOGENOM:HBG715487 EMBL:AB024028 UniGene:At.17610 UniGene:At.22540 UniGene:At.32384 UniGene:At.71559 KO:K11253 OMA:DLRFQSQ EMBL:M35387 EMBL:M17131 EMBL:M17130 EMBL:AK117157 EMBL:AF370577 EMBL:AY037250 EMBL:AY039904 EMBL:AY077654 EMBL:AY081741 EMBL:BT003051 EMBL:BT003162 EMBL:BT005805 EMBL:BT006068 EMBL:AY084316 IPI:IPI00517184 PIR:S06250 RefSeq:NP_189372.1 RefSeq:NP_201339.1 RefSeq:NP_563838.1 RefSeq:NP_568227.1 RefSeq:NP_568228.1 UniGene:At.20804 UniGene:At.21310 UniGene:At.21486 UniGene:At.63772 UniGene:At.73076 ProteinModelPortal:P59226 SMR:P59226 DIP:DIP-48531N STRING:P59226 PRIDE:P59226 EnsemblPlants:AT1G09200.1 EnsemblPlants:AT3G27360.1 EnsemblPlants:AT5G10390.1 EnsemblPlants:AT5G10400.1 EnsemblPlants:AT5G65360.1 GeneID:822357 GeneID:830903 GeneID:830904

Tissue	Identification	# of reads (stress)	# of reads (control)	Fold Change	p-value	Description
						GeneID:836661 GeneID:837440 KEGG:ath:AT1G09200 KEGG:ath:AT3G27360 KEGG:ath:AT5G10390 KEGG:ath:AT5G10400 KEGG:ath:AT5G65360 TAIR:At1g09200 TAIR:At3g27360 TAIR:At5g10390 TAIR:At5g10400 TAIR:At5g65360 InParanoid:P59226 PhylomeDB:P59226 ProtClustDB:PLN00121 ArrayExpress:P59226 Genevestigator:P59226 GermOnline:AT1G09200 Uniprot:P59226
Root	Isotig02389	1	20	-4.451	3.92E-06	TAIR|locus:2195341 - symbol:AT1G09200 species:3702 "Arabidopsis thaliana" [GO:0003677 "DNA binding" evidence=IEA;ISS] [GO:0006334 "nucleosome assembly" evidence=IEA] [GO:0005515 "protein binding" evidence=IPI] InterPro:IPR000164 InterPro:IPR007125 InterPro:IPR009072 Pfam:PF00125 PRINTS:PR00622 PROSITE:PS00322 PROSITE:PS00959 SMART:SM00428 EMBL:CP002684 GenomeReviews:CT485782_GR GO:GO:0005634 EMBL:CP002688 GenomeReviews:BA000015_GR EMBL:AL353995 EMBL:CP002686 GenomeReviews:BA000014_GR EMBL:AB011479 GO:GO:0005515 GO:GO:0003677 Gene3D:G3DSA:1.10.20.10 SUPFAM:SSF47113 GO:GO:0006334 UniGene:At.1824 GO:GO:0000786 EMBL:AC003114 eggNOG:COG2036 PANTHER:PTHR11426 HOGENOM:HBG715487 EMBL:AB024028 UniGene:At.17610 UniGene:At.22540 UniGene:At.32384 UniGene:At.71559 KO:K11253 OMA:DLRFQSQ EMBL:M35387 EMBL:M17131 EMBL:M17130 EMBL:AK117157 EMBL:AF370577 EMBL:AY037250 EMBL:AY039904 EMBL:AY077654 EMBL:AY081741 EMBL:BT003051 EMBL:BT003162 EMBL:BT005805 EMBL:BT006068 EMBL:AY084316 IPI:IPI00517184 PIR:S06250 RefSeq:NP_189372.1 RefSeq:NP_201339.1 RefSeq:NP_563838.1 RefSeq:NP_568227.1 RefSeq:NP_568228.1 UniGene:At.20804 UniGene:At.21310 UniGene:At.21486 UniGene:At.63772 UniGene:At.73076 ProteinModelPortal:P59226 SMR:P59226 DIP:DIP-48531N STRING:P59226 PRIDE:P59226 EnsemblPlants:AT1G09200.1 EnsemblPlants:AT3G27360.1 EnsemblPlants:AT5G10390.1 EnsemblPlants:AT5G10400.1 EnsemblPlants:AT5G65360.1 GeneID:822357 GeneID:830903 GeneID:830904 GeneID:836661 GeneID:837440 KEGG:ath:AT1G09200 KEGG:ath:AT3G27360 KEGG:ath:AT5G10390 KEGG:ath:AT5G10400 KEGG:ath:AT5G65360 TAIR:At1g09200 TAIR:At3g27360 TAIR:At5g10390 TAIR:At5g10400 TAIR:At5g65360 InParanoid:P59226 PhylomeDB:P59226 ProtClustDB:PLN00121 ArrayExpress:P59226 Genevestigator:P59226 GermOnline:AT1G09200 Uniprot:P59226
Root	Isotig02390	1	20	-4.451	3.92E-06	TAIR|locus:2195341 - symbol:AT1G09200 species:3702 "Arabidopsis thaliana" [GO:0003677 "DNA binding" evidence=IEA;ISS] [GO:0006334 "nucleosome assembly" evidence=IEA] [GO:0005515 "protein binding" evidence=IPI] InterPro:IPR000164 InterPro:IPR007125 InterPro:IPR009072 Pfam:PF00125 PRINTS:PR00622 PROSITE:PS00322 PROSITE:PS00959 SMART:SM00428 EMBL:CP002684 GenomeReviews:CT485782_GR GO:GO:0005634 EMBL:CP002688 GenomeReviews:BA000015_GR EMBL:AL353995 EMBL:CP002686 GenomeReviews:BA000014_GR EMBL:AB011479 GO:GO:0005515 GO:GO:0003677 Gene3D:G3DSA:1.10.20.10 SUPFAM:SSF47113 GO:GO:0006334 UniGene:At.1824 GO:GO:0000786 EMBL:AC003114 eggNOG:COG2036 PANTHER:PTHR11426 HOGENOM:HBG715487 EMBL:AB024028 UniGene:At.17610 UniGene:At.22540 UniGene:At.32384 UniGene:At.71559 KO:K11253 OMA:DLRFQSQ EMBL:M35387 EMBL:M17131 EMBL:M17130 EMBL:AK117157 EMBL:AF370577 EMBL:AY037250 EMBL:AY039904 EMBL:AY077654 EMBL:AY081741 EMBL:BT003051 EMBL:BT003162 EMBL:BT005805 EMBL:BT006068 EMBL:AY084316 IPI:IPI00517184 PIR:S06250 RefSeq:NP_189372.1 RefSeq:NP_201339.1 RefSeq:NP_563838.1 RefSeq:NP_568227.1 RefSeq:NP_568228.1 UniGene:At.20804 UniGene:At.21310 UniGene:At.21486 UniGene:At.63772 UniGene:At.73076 ProteinModelPortal:P59226 SMR:P59226 DIP:DIP-48531N STRING:P59226 PRIDE:P59226 EnsemblPlants:AT1G09200.1 EnsemblPlants:AT3G27360.1 EnsemblPlants:AT5G10390.1 EnsemblPlants:AT5G10400.1 EnsemblPlants:AT5G65360.1 GeneID:822357 GeneID:830903 GeneID:830904 GeneID:836661 GeneID:837440 KEGG:ath:AT1G09200 KEGG:ath:AT3G27360 KEGG:ath:AT5G10390 KEGG:ath:AT5G10400 KEGG:ath:AT5G65360 TAIR:At1g09200 TAIR:At3g27360 TAIR:At5g10390 TAIR:At5g10400 TAIR:At5g65360 InParanoid:P59226 PhylomeDB:P59226 ProtClustDB:PLN00121 ArrayExpress:P59226 Genevestigator:P59226 GermOnline:AT1G09200 Uniprot:P59226
Root	Isotig02391	1	20	-4.451	3.92E-06	TAIR|locus:2195341 - symbol:AT1G09200 species:3702 "Arabidopsis thaliana" [GO:0003677 "DNA binding" evidence=IEA;ISS] [GO:0006334 "nucleosome assembly" evidence=IEA] [GO:0005515 "protein binding" evidence=IPI] InterPro:IPR000164 InterPro:IPR007125 InterPro:IPR009072 Pfam:PF00125 PRINTS:PR00622 PROSITE:PS00322 PROSITE:PS00959 SMART:SM00428 EMBL:CP002684 GenomeReviews:CT485782_GR GO:GO:0005634 EMBL:CP002688 GenomeReviews:BA000015_GR EMBL:AL353995 EMBL:CP002686 GenomeReviews:BA000014_GR EMBL:AB011479 GO:GO:0005515 GO:GO:0003677 Gene3D:G3DSA:1.10.20.10 SUPFAM:SSF47113 GO:GO:0006334 UniGene:At.1824 GO:GO:0000786 EMBL:AC003114 eggNOG:COG2036 PANTHER:PTHR11426 HOGENOM:HBG715487 EMBL:AB024028 UniGene:At.17610 UniGene:At.22540

Tissue	Identification	# of reads (stress)	# of reads (control)	Fold Change	p-value	Description
						UniGene:At.32384 UniGene:At.71559 KO:K11253 OMA:DLRFQSQ EMBL:M35387 EMBL:M17131 EMBL:M17130 EMBL:AK117157 EMBL:AF370577 EMBL:AY037250 EMBL:AY039904 EMBL:AY077654 EMBL:AY081741 EMBL:BT003051 EMBL:BT003162 EMBL:BT005805 EMBL:BT006068 EMBL:AY084316 IPI:IPI00517184 PIR:S06250 RefSeq:NP_189372.1 RefSeq:NP_201339.1 RefSeq:NP_563838.1 RefSeq:NP_568227.1 RefSeq:NP_568228.1 UniGene:At.20804 UniGene:At.21310 UniGene:At.21486 UniGene:At.63772 UniGene:At.73076 ProteinModelPortal:P59226 SMR:P59226 DIP:DIP-48531N STRING:P59226 PRIDE:P59226 EnsemblPlants:AT1G09200.1 EnsemblPlants:AT3G27360.1 EnsemblPlants:AT5G10390.1 EnsemblPlants:AT5G10400.1 EnsemblPlants:AT5G65360.1 GeneID:822357 GeneID:830903 GeneID:830904 GeneID:836661 GeneID:837440 KEGG:ath:AT1G09200 KEGG:ath:AT3G27360 KEGG:ath:AT5G10390 KEGG:ath:AT5G10400 KEGG:ath:AT5G65360 TAIR:At1g09200 TAIR:At3g27360 TAIR:At5g10390 TAIR:At5g10400 TAIR:At5g65360 InParanoid:P59226 PhylomeDB:P59226 ProtClustDB:PLN00121 ArrayExpress:P59226 Genevestigator:P59226 GermOnline:AT1G09200 Uniprot:P59226
Root	Isotig02392	1	20	-4.451	3.92E-06	TAIR|locus:2195341 - symbol:AT1G09200 species:3702 "Arabidopsis thaliana" [GO:0003677 "DNA binding" evidence=IEA;ISS] [GO:0006334 "nucleosome assembly" evidence=IEA] [GO:0005515 "protein binding" evidence=IPI] InterPro:IPR000164 InterPro:IPR007125 InterPro:IPR009072 Pfam:PF00125 PRINTS:PR00622 PROSITE:PS00322 PROSITE:PS00959 SMART:SM00428 EMBL:CP002684 GenomeReviews:CT485782_GR GO:GO:0005634 EMBL:CP002688 GenomeReviews:BA000015_GR EMBL:AL353995 EMBL:CP002686 GenomeReviews:BA000014_GR EMBL:AB011479 GO:GO:0005515 GO:GO:0003677 Gene3D:G3DSA:1.10.20.10 SUPFAM:SSF47113 GO:GO:0006334 UniGene:At.1824 GO:GO:0000786 EMBL:AC003114 eggNOG:COG2036 PANTHER:PTHR11426 HOGENOM:HBG715487 EMBL:AB024028 UniGene:At.17610 UniGene:At.22540 UniGene:At.32384 UniGene:At.71559 KO:K11253 OMA:DLRFQSQ EMBL:M35387 EMBL:M17131 EMBL:M17130 EMBL:AK117157 EMBL:AF370577 EMBL:AY037250 EMBL:AY039904 EMBL:AY077654 EMBL:AY081741 EMBL:BT003051 EMBL:BT003162 EMBL:BT005805 EMBL:BT006068 EMBL:AY084316 IPI:IPI00517184 PIR:S06250 RefSeq:NP_189372.1 RefSeq:NP_201339.1 RefSeq:NP_563838.1 RefSeq:NP_568227.1 RefSeq:NP_568228.1 UniGene:At.20804 UniGene:At.21310 UniGene:At.21486 UniGene:At.63772 UniGene:At.73076 ProteinModelPortal:P59226 SMR:P59226 DIP:DIP-48531N STRING:P59226 PRIDE:P59226 EnsemblPlants:AT1G09200.1 EnsemblPlants:AT3G27360.1 EnsemblPlants:AT5G10390.1 EnsemblPlants:AT5G10400.1 EnsemblPlants:AT5G65360.1 GeneID:822357 GeneID:830903 GeneID:830904 GeneID:836661 GeneID:837440 KEGG:ath:AT1G09200 KEGG:ath:AT3G27360 KEGG:ath:AT5G10390 KEGG:ath:AT5G10400 KEGG:ath:AT5G65360 TAIR:At1g09200 TAIR:At3g27360 TAIR:At5g10390 TAIR:At5g10400 TAIR:At5g65360 InParanoid:P59226 PhylomeDB:P59226 ProtClustDB:PLN00121 ArrayExpress:P59226 Genevestigator:P59226 GermOnline:AT1G09200 Uniprot:P59226
Root	Isotig02405	1	20	-4.451	3.92E-06	TAIR|locus:2157737 - symbol:WR3 "AT5G50200" species:3702 "Arabidopsis thaliana" [GO:0010167 "response to nitrate" evidence=IEP] [GO:0015112 "nitrate transmembrane transporter activity" evidence=IMP] [GO:0015706 "nitrate transport" evidence=IMP] [GO:0005886 "plasma membrane" evidence=IDA] [GO:0009611 "response to wounding" evidence=IEP] GO:GO:0016021 GO:GO:0005886 EMBL:CP002688 GenomeReviews:BA000015_GR GO:GO:0009611 GO:GO:0015112 GO:GO:0042128 EMBL:AB024031 GO:GO:0010167 EMBL:AJ310933 EMBL:AJ311926 EMBL:AY085586 IPI:IPI00528407 RefSeq:NP_199831.1 RefSeq:NP_851159.1 RefSeq:NP_851160.1 UniGene:At.21028 IntAct:Q9FGS5 STRING:Q9FGS5 TCDB:8.A.20.1.1 PRIDE:Q9FGS5 EnsemblPlants:AT5G50200.1 EnsemblPlants:AT5G50200.2 EnsemblPlants:AT5G50200.3 GeneID:835085 KEGG:ath:AT5G50200 TAIR:At5g50200 eggNOG:NOG312409 InParanoid:Q9FGS5 OMA:VKVKLCY PhylomeDB:Q9FGS5 ProtClustDB:CLSN2687019 Genevestigator:Q9FGS5 InterPro:IPR016605 PIRSF:PIRSF012939 Uniprot:Q9FGS5
Root	Isotig02406	1	20	-4.451	3.92E-06	TAIR|locus:2157737 - symbol:WR3 "AT5G50200" species:3702 "Arabidopsis thaliana" [GO:0010167 "response to nitrate" evidence=IEP] [GO:0015112 "nitrate transmembrane transporter activity" evidence=IMP] [GO:0015706 "nitrate transport" evidence=IMP] [GO:0005886 "plasma membrane" evidence=IDA] [GO:0009611 "response to wounding" evidence=IEP] GO:GO:0016021 GO:GO:0005886 EMBL:CP002688 GenomeReviews:BA000015_GR GO:GO:0009611 GO:GO:0015112 GO:GO:0042128 EMBL:AB024031 GO:GO:0010167 EMBL:AJ310933 EMBL:AJ311926 EMBL:AY085586 IPI:IPI00528407 RefSeq:NP_199831.1 RefSeq:NP_851159.1 RefSeq:NP_851160.1 UniGene:At.21028 IntAct:Q9FGS5 STRING:Q9FGS5 TCDB:8.A.20.1.1 PRIDE:Q9FGS5 EnsemblPlants:AT5G50200.1 EnsemblPlants:AT5G50200.2 EnsemblPlants:AT5G50200.3 GeneID:835085 KEGG:ath:AT5G50200 TAIR:At5g50200 eggNOG:NOG312409 InParanoid:Q9FGS5 OMA:VKVKLCY

Tissue	Identification	# of reads (stress)	# of reads (control)	Fold Change	p-value	Description
						PhylomeDB:Q9FGS5 ProtClustDB:CLSN2687019 Genevestigator:Q9FGS5 InterPro:IPR016605 PIRSF:PIRSF012939 Uniprot:Q9FGS5
Root	Isotig02407	1	20	-4.451	3.92E-06	TAIR|locus:2157737 - symbol:WR3 "AT5G50200" species:3702 "Arabidopsis thaliana" [GO:0010167 "response to nitrate" evidence=IEP] [GO:0015112 "nitrate transmembrane transporter activity" evidence=IMP] [GO:0015706 "nitrate transport" evidence=IMP] [GO:0005886 "plasma membrane" evidence=IDA] [GO:0009611 "response to wounding" evidence=IEP] GO:GO:0016021 GO:GO:0005886 EMBL:CP002688 GenomeReviews:BA000015_GR GO:GO:0009611 GO:GO:0015112 GO:GO:0042128 EMBL:AB024031 GO:GO:0010167 EMBL:AJ310933 EMBL:AJ311926 EMBL:AY085586 IPI:IPI00528407 RefSeq:NP_199831.1 RefSeq:NP_851159.1 RefSeq:NP_851160.1 UniGene:At.21028 IntAct:Q9FGS5 STRING:Q9FGS5 TCDB:8.A.20.1.1 PRIDE:Q9FGS5 EnsemblPlants:AT5G50200.1 EnsemblPlants:AT5G50200.2 EnsemblPlants:AT5G50200.3 GeneID:835085 KEGG:ath:AT5G50200 TAIR:At5g50200 eggNOG:NOG312409 InParanoid:Q9FGS5 OMA:VKVKLCY PhylomeDB:Q9FGS5 ProtClustDB:CLSN2687019 Genevestigator:Q9FGS5 InterPro:IPR016605 PIRSF:PIRSF012939 Uniprot:Q9FGS5
Root	Isotig02408	1	20	-4.451	3.92E-06	TAIR|locus:2157737 - symbol:WR3 "AT5G50200" species:3702 "Arabidopsis thaliana" [GO:0010167 "response to nitrate" evidence=IEP] [GO:0015112 "nitrate transmembrane transporter activity" evidence=IMP] [GO:0015706 "nitrate transport" evidence=IMP] [GO:0005886 "plasma membrane" evidence=IDA] [GO:0009611 "response to wounding" evidence=IEP] GO:GO:0016021 GO:GO:0005886 EMBL:CP002688 GenomeReviews:BA000015_GR GO:GO:0009611 GO:GO:0015112 GO:GO:0042128 EMBL:AB024031 GO:GO:0010167 EMBL:AJ310933 EMBL:AJ311926 EMBL:AY085586 IPI:IPI00528407 RefSeq:NP_199831.1 RefSeq:NP_851159.1 RefSeq:NP_851160.1 UniGene:At.21028 IntAct:Q9FGS5 STRING:Q9FGS5 TCDB:8.A.20.1.1 PRIDE:Q9FGS5 EnsemblPlants:AT5G50200.1 EnsemblPlants:AT5G50200.2 EnsemblPlants:AT5G50200.3 GeneID:835085 KEGG:ath:AT5G50200 TAIR:At5g50200 eggNOG:NOG312409 InParanoid:Q9FGS5 OMA:VKVKLCY PhylomeDB:Q9FGS5 ProtClustDB:CLSN2687019 Genevestigator:Q9FGS5 InterPro:IPR016605 PIRSF:PIRSF012939 Uniprot:Q9FGS5
Root	Isotig02409	1	20	-4.451	3.92E-06	TAIR|locus:2157737 - symbol:WR3 "AT5G50200" species:3702 "Arabidopsis thaliana" [GO:0010167 "response to nitrate" evidence=IEP] [GO:0015112 "nitrate transmembrane transporter activity" evidence=IMP] [GO:0015706 "nitrate transport" evidence=IMP] [GO:0005886 "plasma membrane" evidence=IDA] [GO:0009611 "response to wounding" evidence=IEP] GO:GO:0016021 GO:GO:0005886 EMBL:CP002688 GenomeReviews:BA000015_GR GO:GO:0009611 GO:GO:0015112 GO:GO:0042128 EMBL:AB024031 GO:GO:0010167 EMBL:AJ310933 EMBL:AJ311926 EMBL:AY085586 IPI:IPI00528407 RefSeq:NP_199831.1 RefSeq:NP_851159.1 RefSeq:NP_851160.1 UniGene:At.21028 IntAct:Q9FGS5 STRING:Q9FGS5 TCDB:8.A.20.1.1 PRIDE:Q9FGS5 EnsemblPlants:AT5G50200.1 EnsemblPlants:AT5G50200.2 EnsemblPlants:AT5G50200.3 GeneID:835085 KEGG:ath:AT5G50200 TAIR:At5g50200 eggNOG:NOG312409 InParanoid:Q9FGS5 OMA:VKVKLCY PhylomeDB:Q9FGS5 ProtClustDB:CLSN2687019 Genevestigator:Q9FGS5 InterPro:IPR016605 PIRSF:PIRSF012939 Uniprot:Q9FGS5
Root	Isotig02410	1	20	-4.451	3.92E-06	TAIR|locus:2157737 - symbol:WR3 "AT5G50200" species:3702 "Arabidopsis thaliana" [GO:0010167 "response to nitrate" evidence=IEP] [GO:0015112 "nitrate transmembrane transporter activity" evidence=IMP] [GO:0015706 "nitrate transport" evidence=IMP] [GO:0005886 "plasma membrane" evidence=IDA] [GO:0009611 "response to wounding" evidence=IEP] GO:GO:0016021 GO:GO:0005886 EMBL:CP002688 GenomeReviews:BA000015_GR GO:GO:0009611 GO:GO:0015112 GO:GO:0042128 EMBL:AB024031 GO:GO:0010167 EMBL:AJ310933 EMBL:AJ311926 EMBL:AY085586 IPI:IPI00528407 RefSeq:NP_199831.1 RefSeq:NP_851159.1 RefSeq:NP_851160.1 UniGene:At.21028 IntAct:Q9FGS5 STRING:Q9FGS5 TCDB:8.A.20.1.1 PRIDE:Q9FGS5 EnsemblPlants:AT5G50200.1 EnsemblPlants:AT5G50200.2 EnsemblPlants:AT5G50200.3 GeneID:835085 KEGG:ath:AT5G50200 TAIR:At5g50200 eggNOG:NOG312409 InParanoid:Q9FGS5 OMA:VKVKLCY PhylomeDB:Q9FGS5 ProtClustDB:CLSN2687019 Genevestigator:Q9FGS5 InterPro:IPR016605 PIRSF:PIRSF012939 Uniprot:Q9FGS5
Root	Isotig02417	0	26	-5.829	5.70E-08	UNIPROTKB|B6TYV8 - symbol:CNR2 "Cell number regulator 2" species:4577 "Zea mays" [GO:0008285 "negative regulation of cell proliferation" evidence=IMP] GO:GO:0016021 GO:GO:0008285 InterPro:IPR006461 Pfam:PF04749 TIGRFAMs:TIGR01571 EMBL:HM008654 EMBL:EU970173 EMBL:BT068908 RefSeq:NP_001144684.1 UniGene:Zm.7882 EnsemblPlants:GRMZM2G151230_T01 GeneID:100277715 KEGG:zma:100277715 PhylomeDB:B6TYV8 Uniprot:B6TYV8

Tissue	Identification	# of reads (stress)	# of reads (control)	Fold Change	p-value	Description
Root	Isotig02418	0	26	-5.829	5.70E-08	UNIPROTKB|B6TYV8 - symbol:CNR2 "Cell number regulator 2" species:4577 "Zea mays" [GO:0008285 "negative regulation of cell proliferation" evidence=IMP] GO:GO:0016021 GO:GO:0008285 InterPro:IPR006461 Pfam:PF04749 TIGRFAMs:TIGR01571 EMBL:HM008654 EMBL:EU970173 EMBL:BT068908 RefSeq:NP_001144684.1 UniGene:Zm.7882 EnsemblPlants:GRMZM2G151230_T01 GeneID:100277715 KEGG:zma:100277715 PhylomeDB:B6TYV8 Uniprot:B6TYV8
Root	Isotig02419	0	26	-5.829	5.70E-08	UNIPROTKB|B6TYV8 - symbol:CNR2 "Cell number regulator 2" species:4577 "Zea mays" [GO:0008285 "negative regulation of cell proliferation" evidence=IMP] GO:GO:0016021 GO:GO:0008285 InterPro:IPR006461 Pfam:PF04749 TIGRFAMs:TIGR01571 EMBL:HM008654 EMBL:EU970173 EMBL:BT068908 RefSeq:NP_001144684.1 UniGene:Zm.7882 EnsemblPlants:GRMZM2G151230_T01 GeneID:100277715 KEGG:zma:100277715 PhylomeDB:B6TYV8 Uniprot:B6TYV8
Root	Isotig02420	0	26	-5.829	5.70E-08	UNIPROTKB|B6TYV8 - symbol:CNR2 "Cell number regulator 2" species:4577 "Zea mays" [GO:0008285 "negative regulation of cell proliferation" evidence=IMP] GO:GO:0016021 GO:GO:0008285 InterPro:IPR006461 Pfam:PF04749 TIGRFAMs:TIGR01571 EMBL:HM008654 EMBL:EU970173 EMBL:BT068908 RefSeq:NP_001144684.1 UniGene:Zm.7882 EnsemblPlants:GRMZM2G151230_T01 GeneID:100277715 KEGG:zma:100277715 PhylomeDB:B6TYV8 Uniprot:B6TYV8
Root	Isotig02421	0	26	-5.829	5.70E-08	UNIPROTKB|B6TYV8 - symbol:CNR2 "Cell number regulator 2" species:4577 "Zea mays" [GO:0008285 "negative regulation of cell proliferation" evidence=IMP] GO:GO:0016021 GO:GO:0008285 InterPro:IPR006461 Pfam:PF04749 TIGRFAMs:TIGR01571 EMBL:HM008654 EMBL:EU970173 EMBL:BT068908 RefSeq:NP_001144684.1 UniGene:Zm.7882 EnsemblPlants:GRMZM2G151230_T01 GeneID:100277715 KEGG:zma:100277715 PhylomeDB:B6TYV8 Uniprot:B6TYV8
Root	Isotig02422	0	26	-5.829	5.70E-08	UNIPROTKB|B6TYV8 - symbol:CNR2 "Cell number regulator 2" species:4577 "Zea mays" [GO:0008285 "negative regulation of cell proliferation" evidence=IMP] GO:GO:0016021 GO:GO:0008285 InterPro:IPR006461 Pfam:PF04749 TIGRFAMs:TIGR01571 EMBL:HM008654 EMBL:EU970173 EMBL:BT068908 RefSeq:NP_001144684.1 UniGene:Zm.7882 EnsemblPlants:GRMZM2G151230_T01 GeneID:100277715 KEGG:zma:100277715 PhylomeDB:B6TYV8 Uniprot:B6TYV8
Root	Isotig02423	37	8	2.080	2.32E-05	TAIR|locus:1005716170 - symbol:CAM6 "AT5G21274" species:3702 "Arabidopsis thaliana" [GO:0005509 "calcium ion binding" evidence=ISS] [GO:0005515 "protein binding" evidence=IPI] [GO:0005829 "cytosol" evidence=RCA] [GO:0019722 "calcium-mediated signaling" evidence=TAS] InterPro:IPR002048 InterPro:IPR011992 SMART:SM00054 Prosite:PS00018 EMBL:CP002688 GenomeReviews:BA000015_GR GO:GO:0005509 GO:GO:0005515 GO:GO:0019722 InterPro:IPR018247 InterPro:IPR018249 Gene3D:G3DSA:1.10.238.10 PROSITE:PS50222 EMBL:AC140977 eggNOG:COG5126 HOGENOM:HBG746798 ProtClustDB:CLSN2681765 EMBL:Z12024 EMBL:AY050350 EMBL:AY094037 IPI:IPI00520449 PIR:S35187 RefSeq:NP_850860.1 UniGene:At.21106 ProteinModelPortal:Q03509 SMR:Q03509 IntAct:Q03509 STRING:Q03509 PRIDE:Q03509 EnsemblPlants:AT5G21274.1 GeneID:832245 KEGG:ath:AT5G21274 TAIR:At5g21274 InParanoid:Q03509 OMA:MANKRAK PhylomeDB:Q03509 Genevestigator:Q03509 Uniprot:Q03509
Root	Isotig02424	36	8	2.041	3.74E-05	TAIR|locus:1005716170 - symbol:CAM6 "AT5G21274" species:3702 "Arabidopsis thaliana" [GO:0005509 "calcium ion binding" evidence=ISS] [GO:0005515 "protein binding" evidence=IPI] [GO:0005829 "cytosol" evidence=RCA] [GO:0019722 "calcium-mediated signaling" evidence=TAS] InterPro:IPR002048 InterPro:IPR011992 SMART:SM00054 Prosite:PS00018 EMBL:CP002688 GenomeReviews:BA000015_GR GO:GO:0005509 GO:GO:0005515 GO:GO:0019722 InterPro:IPR018247 InterPro:IPR018249 Gene3D:G3DSA:1.10.238.10 PROSITE:PS50222 EMBL:AC140977 eggNOG:COG5126 HOGENOM:HBG746798 ProtClustDB:CLSN2681765 EMBL:Z12024 EMBL:AY050350 EMBL:AY094037 IPI:IPI00520449 PIR:S35187 RefSeq:NP_850860.1 UniGene:At.21106 ProteinModelPortal:Q03509 SMR:Q03509 IntAct:Q03509 STRING:Q03509 PRIDE:Q03509 EnsemblPlants:AT5G21274.1 GeneID:832245 KEGG:ath:AT5G21274 TAIR:At5g21274 InParanoid:Q03509 OMA:MANKRAK PhylomeDB:Q03509 Genevestigator:Q03509 Uniprot:Q03509
Root	Isotig02425	38	8	2.119	1.43E-05	TAIR|locus:1005716170 - symbol:CAM6 "AT5G21274" species:3702 "Arabidopsis thaliana" [GO:0005509 "calcium ion binding" evidence=ISS] [GO:0005515 "protein binding" evidence=IPI] [GO:0005829 "cytosol" evidence=RCA] [GO:0019722 "calcium-mediated signaling" evidence=TAS] InterPro:IPR002048 InterPro:IPR011992 SMART:SM00054 Prosite:PS00018 EMBL:CP002688 GenomeReviews:BA000015_GR GO:GO:0005509 GO:GO:0005515 GO:GO:0019722 InterPro:IPR018247 InterPro:IPR018249 Gene3D:G3DSA:1.10.238.10 PROSITE:PS50222 EMBL:AC140977 eggNOG:COG5126 HOGENOM:HBG746798 ProtClustDB:CLSN2681765 EMBL:Z12024 EMBL:AY050350 EMBL:AY094037 IPI:IPI00520449 PIR:S35187 RefSeq:NP_850860.1

Tissue	Identification	# of reads (stress)	# of reads (control)	Fold Change	p-value	Description
						UniGene:At.21106 ProteinModelPortal:Q03509 SMR:Q03509 IntAct:Q03509 STRING:Q03509 PRIDE:Q03509 EnsemblPlants:AT5G21274.1 GeneID:832245 KEGG:ath:AT5G21274 TAIR:At5g21274 InParanoid:Q03509 OMA:MANKRAK PhylomeDB:Q03509 Genevestigator:Q03509 Uniprot:Q03509
Root	Isotig02426	37	8	2.080	2.32E-05	TAIR|locus:1005716170 - symbol:CAM6 "AT5G21274" species:3702 "Arabidopsis thaliana" [GO:0005509 "calcium ion binding" evidence=ISS] [GO:0005515 "protein binding" evidence=IPI] [GO:0005829 "cytosol" evidence=RCA] [GO:0019722 "calcium-mediated signaling" evidence=TAS] InterPro:IPR002048 InterPro:IPR011992 SMART:SM00054 Prosite:PS00018 EMBL:CP002688 GenomeReviews:BA000015_GR GO:GO:0005509 GO:GO:0005515 GO:GO:0019722 InterPro:IPR018247 InterPro:IPR018249 Gene3D:G3DSA:1.10.238.10 PROSITE:PS50222 EMBL:AC140977 eggNOG:COG5126 HOGENOM:HBG746798 ProtClustDB:CLSN2681765 EMBL:Z12024 EMBL:AY050350 EMBL:AY094037 IPI:IPI00520449 PIR:S35187 RefSeq:NP_850860.1 UniGene:At.21106 ProteinModelPortal:Q03509 SMR:Q03509 IntAct:Q03509 STRING:Q03509 PRIDE:Q03509 EnsemblPlants:AT5G21274.1 GeneID:832245 KEGG:ath:AT5G21274 TAIR:At5g21274 InParanoid:Q03509 OMA:MANKRAK PhylomeDB:Q03509 Genevestigator:Q03509 Uniprot:Q03509
Root	Isotig02427	37	8	2.080	2.32E-05	TAIR|locus:1005716170 - symbol:CAM6 "AT5G21274" species:3702 "Arabidopsis thaliana" [GO:0005509 "calcium ion binding" evidence=ISS] [GO:0005515 "protein binding" evidence=IPI] [GO:0005829 "cytosol" evidence=RCA] [GO:0019722 "calcium-mediated signaling" evidence=TAS] InterPro:IPR002048 InterPro:IPR011992 SMART:SM00054 Prosite:PS00018 EMBL:CP002688 GenomeReviews:BA000015_GR GO:GO:0005509 GO:GO:0005515 GO:GO:0019722 InterPro:IPR018247 InterPro:IPR018249 Gene3D:G3DSA:1.10.238.10 PROSITE:PS50222 EMBL:AC140977 eggNOG:COG5126 HOGENOM:HBG746798 ProtClustDB:CLSN2681765 EMBL:Z12024 EMBL:AY050350 EMBL:AY094037 IPI:IPI00520449 PIR:S35187 RefSeq:NP_850860.1 UniGene:At.21106 ProteinModelPortal:Q03509 SMR:Q03509 IntAct:Q03509 STRING:Q03509 PRIDE:Q03509 EnsemblPlants:AT5G21274.1 GeneID:832245 KEGG:ath:AT5G21274 TAIR:At5g21274 InParanoid:Q03509 OMA:MANKRAK PhylomeDB:Q03509 Genevestigator:Q03509 Uniprot:Q03509
Root	Isotig02428	36	8	2.041	3.74E-05	TAIR|locus:1005716170 - symbol:CAM6 "AT5G21274" species:3702 "Arabidopsis thaliana" [GO:0005509 "calcium ion binding" evidence=ISS] [GO:0005515 "protein binding" evidence=IPI] [GO:0005829 "cytosol" evidence=RCA] [GO:0019722 "calcium-mediated signaling" evidence=TAS] InterPro:IPR002048 InterPro:IPR011992 SMART:SM00054 Prosite:PS00018 EMBL:CP002688 GenomeReviews:BA000015_GR GO:GO:0005509 GO:GO:0005515 GO:GO:0019722 InterPro:IPR018247 InterPro:IPR018249 Gene3D:G3DSA:1.10.238.10 PROSITE:PS50222 EMBL:AC140977 eggNOG:COG5126 HOGENOM:HBG746798 ProtClustDB:CLSN2681765 EMBL:Z12024 EMBL:AY050350 EMBL:AY094037 IPI:IPI00520449 PIR:S35187 RefSeq:NP_850860.1 UniGene:At.21106 ProteinModelPortal:Q03509 SMR:Q03509 IntAct:Q03509 STRING:Q03509 PRIDE:Q03509 EnsemblPlants:AT5G21274.1 GeneID:832245 KEGG:ath:AT5G21274 TAIR:At5g21274 InParanoid:Q03509 OMA:MANKRAK PhylomeDB:Q03509 Genevestigator:Q03509 Uniprot:Q03509
Root	Isotig02435	0	19	-5.377	3.67E-06	TAIR|locus:2035074 - symbol:AT1G52720 "AT1G52720" species:3702 "Arabidopsis thaliana" [GO:0003674 "molecular_function" evidence=ND] [GO:0008150 "biological_process" evidence=ND] EMBL:CP002684 GenomeReviews:CT485782_GR EMBL:AC008016 HOGENOM:HBG597979 ProtClustDB:CLSN2688413 EMBL:AY085970 EMBL:BT004752 EMBL:AK228288 IPI:IPI00543215 RefSeq:NP_564614.1 UniGene:At.11637 PRIDE:Q9SSS6 EnsemblPlants:AT1G52720.1 GeneID:841705 KEGG:ath:AT1G52720 TAIR:At1g52720 eggNOG:NOG251124 InParanoid:Q9SSS6 OMA:TWWAPLF PhylomeDB:Q9SSS6 Genevestigator:Q9SSS6 Uniprot:Q9SSS6
Root	Isotig02436	0	18	-5.299	6.77E-06	TAIR|locus:2093247 - symbol:AT3G15630 "AT3G15630" species:3702 "Arabidopsis thaliana" [GO:0003674 "molecular_function" evidence=ND] [GO:0008150 "biological_process" evidence=ND] EMBL:CP002686 GenomeReviews:BA000014_GR EMBL:AB017071 EMBL:AY052241 EMBL:AY060508 IPI:IPI00533188 RefSeq:NP_566521.1 UniGene:At.20536 STRING:Q9LW16 EnsemblPlants:AT3G15630.1 GeneID:820805 KEGG:ath:AT3G15630 TAIR:At3g15630 eggNOG:NOG293987 HOGENOM:HBG597979 InParanoid:Q9LW16 OMA:TIISCSA PhylomeDB:Q9LW16 ProtClustDB:CLSN2688413 Genevestigator:Q9LW16 Uniprot:Q9LW16
Root	Isotig02437	0	18	-5.299	6.77E-06	TAIR|locus:2093247 - symbol:AT3G15630 "AT3G15630" species:3702 "Arabidopsis thaliana" [GO:0003674 "molecular_function" evidence=ND] [GO:0008150 "biological_process" evidence=ND] EMBL:CP002686 GenomeReviews:BA000014_GR EMBL:AB017071 EMBL:AY052241 EMBL:AY060508 IPI:IPI00533188 RefSeq:NP_566521.1 UniGene:At.20536 STRING:Q9LW16 EnsemblPlants:AT3G15630.1 GeneID:820805 KEGG:ath:AT3G15630 TAIR:At3g15630 eggNOG:NOG293987 HOGENOM:HBG597979 InParanoid:Q9LW16 OMA:TIISCSA PhylomeDB:Q9LW16 ProtClustDB:CLSN2688413 Genevestigator:Q9LW16 Uniprot:Q9LW16

Tissue	Identification	# of reads (stress)	# of reads (control)	Fold Change	p-value	Description
Root	Isotig02438	0	19	-5.377	3.67E-06	TAIR|locus:2035074 - symbol:AT1G52720 "AT1G52720" species:3702 "Arabidopsis thaliana" [GO:0003674 "molecular_function" evidence=ND] [GO:0008150 "biological_process" evidence=ND] EMBL:CP002684 GenomeReviews:CT485782_GR EMBL:AC008016 HOGENOM:HBG597979 ProtClustDB:CLSN2688413 EMBL:AY085970 EMBL:BT004752 EMBL:AK228288 IPI:IPI00543215 RefSeq:NP_564614.1 UniGene:At.11637 PRIDE:Q9SSS6 EnsemblPlants:AT1G52720.1 GeneID:841705 KEGG:ath:AT1G52720 TAIR:At1g52720 eggNOG:NOG251124 InParanoid:Q9SSS6 OMA:TWWAPLF PhylomeDB:Q9SSS6 Genevestigator:Q9SSS6 Uniprot:Q9SSS6
Root	Isotig02439	0	18	-5.299	6.77E-06	TAIR|locus:2093247 - symbol:AT3G15630 "AT3G15630" species:3702 "Arabidopsis thaliana" [GO:0003674 "molecular_function" evidence=ND] [GO:0008150 "biological_process" evidence=ND] EMBL:CP002686 GenomeReviews:BA000014_GR EMBL:AB017071 EMBL:AY052241 EMBL:AY060508 IPI:IPI00533188 RefSeq:NP_566521.1 UniGene:At.20536 STRING:Q9LW16 EnsemblPlants:AT3G15630.1 GeneID:820805 KEGG:ath:AT3G15630 TAIR:At3g15630 eggNOG:NOG293987 HOGENOM:HBG597979 InParanoid:Q9LW16 OMA:TIISCSA PhylomeDB:Q9LW16 ProtClustDB:CLSN2688413 Genevestigator:Q9LW16 Uniprot:Q9LW16
Root	Isotig02440	0	18	-5.299	6.77E-06	TAIR|locus:2093247 - symbol:AT3G15630 "AT3G15630" species:3702 "Arabidopsis thaliana" [GO:0003674 "molecular_function" evidence=ND] [GO:0008150 "biological_process" evidence=ND] EMBL:CP002686 GenomeReviews:BA000014_GR EMBL:AB017071 EMBL:AY052241 EMBL:AY060508 IPI:IPI00533188 RefSeq:NP_566521.1 UniGene:At.20536 STRING:Q9LW16 EnsemblPlants:AT3G15630.1 GeneID:820805 KEGG:ath:AT3G15630 TAIR:At3g15630 eggNOG:NOG293987 HOGENOM:HBG597979 InParanoid:Q9LW16 OMA:TIISCSA PhylomeDB:Q9LW16 ProtClustDB:CLSN2688413 Genevestigator:Q9LW16 Uniprot:Q9LW16
Root	Isotig02450	1	25	-4.773	1.66E-07	TAIR|locus:2036873 - symbol:BGLU46 "beta glucosidase 46" species:3702 "Arabidopsis thaliana" [GO:0003824 "catalytic activity" evidence=IEA] [GO:0004553 "hydrolase activity, hydrolyzing O-glycosyl compounds" evidence=IEA;ISS] [GO:0043169 "cation binding" evidence=IEA] [GO:0008422 "beta-glucosidase activity" evidence=IDA] [GO:0009809 "lignin biosynthetic process" evidence=IMP] [GO:0047782 "coniferin beta-glucosidase activity" evidence=IDA] InterPro:IPR001360 InterPro:IPR013781 InterPro:IPR018120 Pfam:PF00232 PRINTS:PR00131 PROSITE:PS00572 PROSITE:PS00653 EMBL:CP002684 GenomeReviews:CT485782_GR GO:GO:0043169 InterPro:IPR017853 Gene3D:G3DSA:3.20.20.80 SUPFAM:SSF51445 GO:GO:0005975 CAZy:GH1 eggNOG:COG2723 HOGENOM:HBG316462 PANTHER:PTHR10353 EMBL:AC004392 KO:K05350 HSSP:P11546 ProtClustDB:CLSN2682045 GO:GO:0047782 GO:GO:0009809 EMBL:BX816529 EMBL:BT015331 EMBL:BT015708 IPI:IPI00518755 PIR:T02128 RefSeq:NP_850968.1 UniGene:At.27913 ProteinModelPortal:O80690 SMR:O80690 STRING:O80690 PRIDE:O80690 EnsemblPlants:AT1G61820.1 GeneID:842479 KEGG:ath:AT1G61820 TAIR:At1g61820 InParanoid:O80690 OMA:RYMEDIQ PhylomeDB:O80690 Uniprot:O80690
Root	Isotig02461	11	36	-1.839	5.72E-05	TAIR|locus:2174403 - symbol:UBP23 "ubiquitin-specific protease 23" species:3702 "Arabidopsis thaliana" [GO:0004221 "ubiquitin thiolesterase activity" evidence=IEA] [GO:0004843 "ubiquitin-specific protease activity" evidence=ISS] [GO:0006511 "ubiquitin-dependent protein catabolic process" evidence=IEA] InterPro:IPR001394 InterPro:IPR018200 Pfam:PF00443 PROSITE:PS00972 PROSITE:PS00973 PROSITE:PS50235 EMBL:CP002688 GenomeReviews:BA000015_GR GO:GO:0006511 EMBL:AB013396 GO:GO:0008234 GO:GO:0004221 eggNOG:COG5077 HSSP:P54578 KO:K11855 EMBL:AF302671 IPI:IPI00532960 RefSeq:NP_568873.1 UniGene:At.22531 ProteinModelPortal:Q9FPS4 SMR:Q9FPS4 STRING:Q9FPS4 MEROPS:C19.A12 PRIDE:Q9FPS4 EnsemblPlants:AT5G57990.1 GeneID:835910 KEGG:ath:AT5G57990 GeneFarm:1994 TAIR:At5g57990 HOGENOM:HBG317149 InParanoid:Q9FPS4 OMA:MEVATSS PhylomeDB:Q9FPS4 ProtClustDB:CLSN2716507 Genevestigator:Q9FPS4 Uniprot:Q9FPS4
Root	Isotig02462	11	36	-1.839	5.72E-05	TAIR|locus:2174403 - symbol:UBP23 "ubiquitin-specific protease 23" species:3702 "Arabidopsis thaliana" [GO:0004221 "ubiquitin thiolesterase activity" evidence=IEA] [GO:0004843 "ubiquitin-specific protease activity" evidence=ISS] [GO:0006511 "ubiquitin-dependent protein catabolic process" evidence=IEA] InterPro:IPR001394 InterPro:IPR018200 Pfam:PF00443 PROSITE:PS00972 PROSITE:PS00973 PROSITE:PS50235 EMBL:CP002688 GenomeReviews:BA000015_GR GO:GO:0006511 EMBL:AB013396 GO:GO:0008234 GO:GO:0004221 eggNOG:COG5077 HSSP:P54578 KO:K11855 EMBL:AF302671 IPI:IPI00532960 RefSeq:NP_568873.1 UniGene:At.22531 ProteinModelPortal:Q9FPS4 SMR:Q9FPS4 STRING:Q9FPS4 MEROPS:C19.A12 PRIDE:Q9FPS4 EnsemblPlants:AT5G57990.1 GeneID:835910 KEGG:ath:AT5G57990 GeneFarm:1994 TAIR:At5g57990 HOGENOM:HBG317149 InParanoid:Q9FPS4 OMA:MEVATSS PhylomeDB:Q9FPS4 ProtClustDB:CLSN2716507 Genevestigator:Q9FPS4 Uniprot:Q9FPS4

Tissue	Identification	# of reads (stress)	# of reads (control)	Fold Change	p-value	Description
Root	Isotig02464	3	17	-2.631	0.00056383	TAIR|locus:2199297 - symbol:ENDO 2 "AT1G68290" species:3702 "Arabidopsis thaliana" [GO:0003676 "nucleic acid binding" evidence=ISS] [GO:0004519 "endonuclease activity" evidence=ISS] [GO:0006308 "DNA catabolic process" evidence=IEA;ISS] [GO:0000014 "single-stranded DNA specific endodeoxyribonuclease activity" evidence=IDA] [GO:0043765 "T/G mismatch-specific endonuclease activity" evidence=IDA] InterPro:IPR003154 InterPro:IPR008947 Pfam:PF02265 EMBL:CP002684 GO:GO:0003676 EMBL:AC016447 GO:GO:0004519 GO:GO:0006308 Gene3D:G3DSA:1.10.575.10 SUPFAM:SSF48537 HSSP:P24289 ProtClustDB:CLSN2681945 IPI:IPI00543025 PIR:E96706 RefSeq:NP_176996.1 UniGene:At.35544 UniGene:At.43330 ProteinModelPortal:Q9C9G4 SMR:Q9C9G4 PRIDE:Q9C9G4 EnsemblPlants:AT1G68290.1 GeneID:843158 KEGG:ath:AT1G68290 TAIR:At1g68290 InParanoid:Q9C9G4 OMA:AACDWAY PhylomeDB:Q9C9G4 Genevestigator:Q9C9G4 Uniprot:Q9C9G4
Root	Isotig02465	3	17	-2.631	0.00056383	TAIR|locus:2199297 - symbol:ENDO 2 "AT1G68290" species:3702 "Arabidopsis thaliana" [GO:0003676 "nucleic acid binding" evidence=ISS] [GO:0004519 "endonuclease activity" evidence=ISS] [GO:0006308 "DNA catabolic process" evidence=IEA;ISS] [GO:0000014 "single-stranded DNA specific endodeoxyribonuclease activity" evidence=IDA] [GO:0043765 "T/G mismatch-specific endonuclease activity" evidence=IDA] InterPro:IPR003154 InterPro:IPR008947 Pfam:PF02265 EMBL:CP002684 GO:GO:0003676 EMBL:AC016447 GO:GO:0004519 GO:GO:0006308 Gene3D:G3DSA:1.10.575.10 SUPFAM:SSF48537 HSSP:P24289 ProtClustDB:CLSN2681945 IPI:IPI00543025 PIR:E96706 RefSeq:NP_176996.1 UniGene:At.35544 UniGene:At.43330 ProteinModelPortal:Q9C9G4 SMR:Q9C9G4 PRIDE:Q9C9G4 EnsemblPlants:AT1G68290.1 GeneID:843158 KEGG:ath:AT1G68290 TAIR:At1g68290 InParanoid:Q9C9G4 OMA:AACDWAY PhylomeDB:Q9C9G4 Genevestigator:Q9C9G4 Uniprot:Q9C9G4
Root	Isotig02471	67	20	1.615	1.65E-06	TAIR|locus:2167903 - symbol:PRH75 "AT5G62190" species:3702 "Arabidopsis thaliana" [GO:0008026 "ATP-dependent helicase activity" evidence=ISS] [GO:0005730 "nucleolus" evidence=IDA] [GO:0005634 "nucleus" evidence=IDA] [GO:0016070 "RNA metabolic process" evidence=TAS] [GO:0017151 "DEAD/H-box RNA helicase binding" evidence=ISS] InterPro:IPR000629 InterPro:IPR001650 InterPro:IPR011545 InterPro:IPR012562 Pfam:PF00270 Pfam:PF00271 Pfam:PF08152 PROSITE:PS00039 PROSITE:PS51194 SMART:SM00490 InterPro:IPR014001 GO:GO:0005524 EMBL:CP002688 GenomeReviews:BA000015_GR GO:GO:0005730 GO:GO:0003723 GO:GO:0016070 SMART:SM00487 PROSITE:PS51192 EMBL:AB019235 GO:GO:0008026 eggNOG:COG0513 HOGENOM:HBG737336 InterPro:IPR014014 PROSITE:PS51195 EMBL:X99938 EMBL:AY039576 EMBL:AY056137 EMBL:BT008581 IPI:IPI00520493 RefSeq:NP_201025.1 UniGene:At.22598 ProteinModelPortal:Q39189 SMR:Q39189 IntAct:Q39189 STRING:Q39189 PRIDE:Q39189 EnsemblPlants:AT5G62190.1 GeneID:836340 KEGG:ath:AT5G62190 GeneFarm:918 TAIR:At5g62190 InParanoid:Q39189 OMA:PIQATTF PhylomeDB:Q39189 ProtClustDB:CLSN2687322 ArrayExpress:Q9LVB7 Genevestigator:Q39189 GermOnline:AT5G62190 Uniprot:Q39189
Root	Isotig02472	63	19	1.600	3.91E-06	TAIR|locus:2167903 - symbol:PRH75 "AT5G62190" species:3702 "Arabidopsis thaliana" [GO:0008026 "ATP-dependent helicase activity" evidence=ISS] [GO:0005730 "nucleolus" evidence=IDA] [GO:0005634 "nucleus" evidence=IDA] [GO:0016070 "RNA metabolic process" evidence=TAS] [GO:0017151 "DEAD/H-box RNA helicase binding" evidence=ISS] InterPro:IPR000629 InterPro:IPR001650 InterPro:IPR011545 InterPro:IPR012562 Pfam:PF00270 Pfam:PF00271 Pfam:PF08152 PROSITE:PS00039 PROSITE:PS51194 SMART:SM00490 InterPro:IPR014001 GO:GO:0005524 EMBL:CP002688 GenomeReviews:BA000015_GR GO:GO:0005730 GO:GO:0003723 GO:GO:0016070 SMART:SM00487 PROSITE:PS51192 EMBL:AB019235 GO:GO:0008026 eggNOG:COG0513 HOGENOM:HBG737336 InterPro:IPR014014 PROSITE:PS51195 EMBL:X99938 EMBL:AY039576 EMBL:AY056137 EMBL:BT008581 IPI:IPI00520493 RefSeq:NP_201025.1 UniGene:At.22598 ProteinModelPortal:Q39189 SMR:Q39189 IntAct:Q39189 STRING:Q39189 PRIDE:Q39189 EnsemblPlants:AT5G62190.1 GeneID:836340 KEGG:ath:AT5G62190 GeneFarm:918 TAIR:At5g62190 InParanoid:Q39189 OMA:PIQATTF PhylomeDB:Q39189 ProtClustDB:CLSN2687322 ArrayExpress:Q9LVB7 Genevestigator:Q39189 GermOnline:AT5G62190 Uniprot:Q39189
Root	Isotig02473	58	14	1.922	4.89E-07	TAIR|locus:2167903 - symbol:PRH75 "AT5G62190" species:3702 "Arabidopsis thaliana" [GO:0008026 "ATP-dependent helicase activity" evidence=ISS] [GO:0005730 "nucleolus" evidence=IDA] [GO:0005634 "nucleus" evidence=IDA] [GO:0016070 "RNA metabolic process" evidence=TAS] [GO:0017151 "DEAD/H-box RNA helicase binding" evidence=ISS] InterPro:IPR000629 InterPro:IPR001650 InterPro:IPR011545 InterPro:IPR012562 Pfam:PF00270 Pfam:PF00271 Pfam:PF08152 PROSITE:PS00039 PROSITE:PS51194 SMART:SM00490 InterPro:IPR014001 GO:GO:0005524 EMBL:CP002688 GenomeReviews:BA000015_GR GO:GO:0005730

Tissue	Identification	# of reads (stress)	# of reads (control)	Fold Change	p-value	Description
						GO:GO:0003723 GO:GO:0016070 SMART:SM00487 PROSITE:PS51192 EMBL:AB019235 GO:GO:0008026 eggNOG:COG0513 HOGENOM:HBG737336 InterPro:IPR014014 PROSITE:PS51195 EMBL:X99938 EMBL:AY039576 EMBL:AY056137 EMBL:BT008581 IPI:IPI00520493 RefSeq:NP_201025.1 UniGene:At.22598 ProteinModelPortal:Q39189 SMR:Q39189 IntAct:Q39189 STRING:Q39189 PRIDE:Q39189 EnsemblPlants:AT5G62190.1 GeneID:836340 KEGG:ath:AT5G62190 GeneFarm:918 TAIR:At5g62190 InParanoid:Q39189 OMA:PIQATTF PhylomeDB:Q39189 ProtClustDB:CLSN2687322 ArrayExpress:Q9LVB7 Genevestigator:Q39189 GermOnline:AT5G62190 Uniprot:Q39189
Root	Isotig02474	53	6	3.014	4.34E-10	TAIR|locus:2167903 - symbol:PRH75 "AT5G62190" species:3702 "Arabidopsis thaliana" [GO:0008026 "ATP-dependent helicase activity" evidence=ISS] [GO:0005730 "nucleolus" evidence=IDA] [GO:0005634 "nucleus" evidence=IDA] [GO:0016070 "RNA metabolic process" evidence=TAS] [GO:0017151 "DEAD/H-box RNA helicase binding" evidence=ISS] InterPro:IPR000629 InterPro:IPR001650 InterPro:IPR011545 InterPro:IPR012562 Pfam:PF00270 Pfam:PF00271 Pfam:PF08152 PROSITE:PS00039 PROSITE:PS51194 SMART:SM00490 InterPro:IPR014001 GO:GO:0005524 EMBL:CP002688 GenomeReviews:BA000015_GR GO:GO:0005730 GO:GO:0003723 GO:GO:0016070 SMART:SM00487 PROSITE:PS51192 EMBL:AB019235 GO:GO:0008026 eggNOG:COG0513 HOGENOM:HBG737336 InterPro:IPR014014 PROSITE:PS51195 EMBL:X99938 EMBL:AY039576 EMBL:AY056137 EMBL:BT008581 IPI:IPI00520493 RefSeq:NP_201025.1 UniGene:At.22598 ProteinModelPortal:Q39189 SMR:Q39189 IntAct:Q39189 STRING:Q39189 PRIDE:Q39189 EnsemblPlants:AT5G62190.1 GeneID:836340 KEGG:ath:AT5G62190 GeneFarm:918 TAIR:At5g62190 InParanoid:Q39189 OMA:PIQATTF PhylomeDB:Q39189 ProtClustDB:CLSN2687322 ArrayExpress:Q9LVB7 Genevestigator:Q39189 GermOnline:AT5G62190 Uniprot:Q39189
Root	Isotig02475	54	13	1.925	1.17E-06	TAIR|locus:2167903 - symbol:PRH75 "AT5G62190" species:3702 "Arabidopsis thaliana" [GO:0008026 "ATP-dependent helicase activity" evidence=ISS] [GO:0005730 "nucleolus" evidence=IDA] [GO:0005634 "nucleus" evidence=IDA] [GO:0016070 "RNA metabolic process" evidence=TAS] [GO:0017151 "DEAD/H-box RNA helicase binding" evidence=ISS] InterPro:IPR000629 InterPro:IPR001650 InterPro:IPR011545 InterPro:IPR012562 Pfam:PF00270 Pfam:PF00271 Pfam:PF08152 PROSITE:PS00039 PROSITE:PS51194 SMART:SM00490 InterPro:IPR014001 GO:GO:0005524 EMBL:CP002688 GenomeReviews:BA000015_GR GO:GO:0005730 GO:GO:0003723 GO:GO:0016070 SMART:SM00487 PROSITE:PS51192 EMBL:AB019235 GO:GO:0008026 eggNOG:COG0513 HOGENOM:HBG737336 InterPro:IPR014014 PROSITE:PS51195 EMBL:X99938 EMBL:AY039576 EMBL:AY056137 EMBL:BT008581 IPI:IPI00520493 RefSeq:NP_201025.1 UniGene:At.22598 ProteinModelPortal:Q39189 SMR:Q39189 IntAct:Q39189 STRING:Q39189 PRIDE:Q39189 EnsemblPlants:AT5G62190.1 GeneID:836340 KEGG:ath:AT5G62190 GeneFarm:918 TAIR:At5g62190 InParanoid:Q39189 OMA:PIQATTF PhylomeDB:Q39189 ProtClustDB:CLSN2687322 ArrayExpress:Q9LVB7 Genevestigator:Q39189 GermOnline:AT5G62190 Uniprot:Q39189
Root	Isotig02486	107	162	-0.727	4.25E-05	UNIPROTKB|B9RXH6 - symbol:RCOM_0903730 "Aspartic proteinase, putative" species:3988 "Ricinus communis" [GO:0005783 "endoplasmic reticulum" evidence=IDA] InterPro:IPR001461 InterPro:IPR001969 InterPro:IPR007856 InterPro:IPR009007 Pfam:PF00026 Pfam:PF05184 PRINTS:PR00792 PROSITE:PS00141 GO:GO:0005783 GO:GO:0006508 GO:GO:0006629 InterPro:IPR008138 InterPro:IPR011001 InterPro:IPR008139 Gene3D:G3DSA:1.10.225.10 Pfam:PF03489 SMART:SM00741 SUPFAM:SSF47862 PROSITE:PS50015 GO:GO:0004190 InterPro:IPR021109 SUPFAM:SSF50630 Gene3D:G3DSA:2.40.70.10 PANTHER:PTHR13683 EMBL:EQ973828 RefSeq:XP_002518445.1 GeneID:8277993 KEGG:rcu:RCOM_0903730 KO:K08245 PhylomeDB:B9RXH6 ProtClustDB:CLSN2926707 Uniprot:B9RXH6
Root	Isotig02487	104	164	-0.786	1.05E-05	UNIPROTKB|B9RXH6 - symbol:RCOM_0903730 "Aspartic proteinase, putative" species:3988 "Ricinus communis" [GO:0005783 "endoplasmic reticulum" evidence=IDA] InterPro:IPR001461 InterPro:IPR001969 InterPro:IPR007856 InterPro:IPR009007 Pfam:PF00026 Pfam:PF05184 PRINTS:PR00792 PROSITE:PS00141 GO:GO:0005783 GO:GO:0006508 GO:GO:0006629 InterPro:IPR008138 InterPro:IPR011001 InterPro:IPR008139 Gene3D:G3DSA:1.10.225.10 Pfam:PF03489 SMART:SM00741 SUPFAM:SSF47862 PROSITE:PS50015 GO:GO:0004190 InterPro:IPR021109 SUPFAM:SSF50630 Gene3D:G3DSA:2.40.70.10 PANTHER:PTHR13683 EMBL:EQ973828 RefSeq:XP_002518445.1 GeneID:8277993 KEGG:rcu:RCOM_0903730 KO:K08245 PhylomeDB:B9RXH6 ProtClustDB:CLSN2926707 Uniprot:B9RXH6
Root	Isotig02488	99	149	-0.719	0.000102289	UNIPROTKB|B9RXH6 - symbol:RCOM_0903730 "Aspartic proteinase, putative" species:3988 "Ricinus communis" [GO:0005783 "endoplasmic reticulum" evidence=IDA] InterPro:IPR001461 InterPro:IPR001969 InterPro:IPR007856 InterPro:IPR009007 Pfam:PF00026 Pfam:PF05184 PRINTS:PR00792 PROSITE:PS00141 GO:GO:0005783

Tissue	Identification	# of reads (stress)	# of reads (control)	Fold Change	p-value	Description
						GO:GO:0006508 GO:GO:0006629 InterPro:IPR008138 InterPro:IPR011001 InterPro:IPR008139 Gene3D:G3DSA:1.10.225.10 Pfam:PF03489 SMART:SM00741 SUPFAM:SSF47862 PROSITE:PS50015 GO:GO:0004190 InterPro:IPR021109 SUPFAM:SSF50630 Gene3D:G3DSA:2.40.70.10 PANTHER:PTHR13683 EMBL:EQ973828 RefSeq:XP_002518445.1 GeneID:8277993 KEGG:rcu:RCOM_0903730 KO:K08245 PhylomeDB:B9RXH6 ProtClustDB:CLSN2926707 Uniprot:B9RXH6
Root	Isotig02489	96	151	-0.782	2.55E-05	UNIPROTKB|B9RXH6 - symbol:RCOM_0903730 "Aspartic proteinase, putative" species:3988 "Ricinus communis" [GO:0005783 "endoplasmic reticulum" evidence=IDA] InterPro:IPR001461 InterPro:IPR001969 InterPro:IPR007856 InterPro:IPR009007 Pfam:PF00026 Pfam:PF05184 PRINTS:PR00792 PROSITE:PS00141 GO:GO:0005783 GO:GO:0006508 GO:GO:0006629 InterPro:IPR008138 InterPro:IPR011001 InterPro:IPR008139 Gene3D:G3DSA:1.10.225.10 Pfam:PF03489 SMART:SM00741 SUPFAM:SSF47862 PROSITE:PS50015 GO:GO:0004190 InterPro:IPR021109 SUPFAM:SSF50630 Gene3D:G3DSA:2.40.70.10 PANTHER:PTHR13683 EMBL:EQ973828 RefSeq:XP_002518445.1 GeneID:8277993 KEGG:rcu:RCOM_0903730 KO:K08245 PhylomeDB:B9RXH6 ProtClustDB:CLSN2926707 Uniprot:B9RXH6
Root	Isotig02493	3	26	-3.244	2.27E-06	TAIR|locus:2083053 - symbol:AT3G49930 "AT3G49930" species:3702 "Arabidopsis thaliana" [GO:0003676 "nucleic acid binding" evidence=ISS] [GO:0005622 "intracellular" evidence=IEA] [GO:0008270 "zinc ion binding" evidence=ISS] [GO:0003700 "sequence-specific DNA binding transcription factor activity" evidence=ISS] [GO:0006355 "regulation of transcription, DNA-dependent" evidence=TAS] InterPro:IPR007087 InterPro:IPR013087 InterPro:IPR015880 PROSITE:PS00028 PROSITE:PS50157 SMART:SM00355 EMBL:CP002686 GenomeReviews:BA000014_GR GO:GO:0006355 GO:GO:0008270 GO:GO:0003676 Gene3D:G3DSA:3.30.160.60 GO:GO:0005622 EMBL:AL132978 HSSP:Q38895 HOGENOM:HBG600018 EMBL:BT029377 IPI:IPI00542862 PIR:T45846 RefSeq:NP_190562.1 UniGene:At.35551 ProteinModelPortal:Q9SN24 SMR:Q9SN24 EnsemblPlants:AT3G49930.1 GeneID:824155 KEGG:ath:AT3G49930 TAIR:At3g49930 eggNOG:NOG248161 InParanoid:Q9SN24 OMA:THNCSIC PhylomeDB:Q9SN24 ProtClustDB:CLSN2684382 Genevestigator:Q9SN24 Uniprot:Q9SN24
Root	Isotig02495	3	26	-3.244	2.27E-06	TAIR|locus:2199246 - symbol:STZ "AT1G27730" species:3702 "Arabidopsis thaliana" [GO:0003676 "nucleic acid binding" evidence=ISS] [GO:0008270 "zinc ion binding" evidence=ISS] [GO:0003700 "sequence-specific DNA binding transcription factor activity" evidence=ISS] [GO:0005634 "nucleus" evidence=IDA] [GO:0009409 "response to cold" evidence=IEP] [GO:0009414 "response to water deprivation" evidence=IEP;IMP] [GO:0009737 "response to abscisic acid stimulus" evidence=IEP] [GO:0035264 "multicellular organism growth" evidence=IMP] [GO:0043565 "sequence-specific DNA binding" evidence=IDA] [GO:0045892 "negative regulation of transcription, DNA-dependent" evidence=IDA] [GO:0010200 "response to chitin" evidence=IEP] [GO:0009611 "response to wounding" evidence=IEP] [GO:0006979 "response to oxidative stress" evidence=IMP] [GO:0009644 "response to high light intensity" evidence=IEP] [GO:0010117 "photoprotection" evidence=IMP] [GO:0015979 "photosynthesis" evidence=IMP] [GO:0009651 "response to salt stress" evidence=IGI;IEP] InterPro:IPR007087 InterPro:IPR013087 InterPro:IPR015880 PROSITE:PS00028 PROSITE:PS50157 SMART:SM00355 EMBL:CP002684 GO:GO:0005634 GO:GO:0006355 GO:GO:0008270 GO:GO:0006351 GO:GO:0003676 EMBL:AC012375 Gene3D:G3DSA:3.30.160.60 EMBL:X95573 EMBL:X98670 EMBL:X98671 EMBL:AF250336 EMBL:AY034998 EMBL:AY063006 IPI:IPI00536591 RefSeq:NP_174094.1 UniGene:At.24624 ProteinModelPortal:Q96289 SMR:Q96289 STRING:Q96289 GeneID:839666 KEGG:ath:AT1G27730 TAIR:At1g27730 eggNOG:NOG276050 InParanoid:Q9SFY6 OMA:HVSSSHR ProtClustDB:CLSN2679322 ArrayExpress:Q96289 Genevestigator:Q96289 Uniprot:Q96289
Root	Isotig02506	13	75	-2.657	3.29E-13	TAIR|locus:2177003 - symbol:TUB6 "AT5G12250" species:3702 "Arabidopsis thaliana" [GO:0009409 "response to cold" evidence=IEP] [GO:0005200 "structural constituent of cytoskeleton" evidence=ISS] [GO:0007017 "microtubule-based process" evidence=ISS] [GO:0015630 "microtubule cytoskeleton" evidence=ISS] [GO:0005886 "plasma membrane" evidence=IDA] [GO:0009651 "response to salt stress" evidence=IEP] InterPro:IPR000217 InterPro:IPR002453 InterPro:IPR003008 InterPro:IPR008280 InterPro:IPR017975 InterPro:IPR018316 Pfam:PF00091 Pfam:PF03953 PRINTS:PR01161 PRINTS:PR01163 PROSITE:PS00227 SMART:SM00864 SMART:SM00865 GO:GO:0005525 GO:GO:0005886 GO:GO:0005737 EMBL:CP002688 GenomeReviews:BA000015_GR GO:GO:0005198 GO:GO:0003924 GO:GO:0009409 GO:GO:0009651 GO:GO:0005874 GO:GO:0051258 Gene3D:G3DSA:3.40.50.1440 SUPFAM:SSF52490 GO:GO:0007018 EMBL:AB007727 Gene3D:G3DSA:3.30.1330.20 SUPFAM:SSF55307 HOGENOM:HBG750007 InterPro:IPR023123 Gene3D:G3DSA:1.10.287.600 PANTHER:PTHR11588 InterPro:IPR013838 KO:K07375 ProtClustDB:PLN00220

Tissue	Identification	# of reads (stress)	# of reads (control)	Fold Change	p-value	Description
						PROSITE:PS00228 OMA:EVINDEH EMBL:M84703 EMBL:AF360260 EMBL:AY040074 IPI:IPI00545934 PIR:JQ1590 RefSeq:NP_196786.1 UniGene:At.23425 UniGene:At.72672 UniGene:At.74848 ProteinModelPortal:P29514 SMR:P29514 STRING:P29514 PRIDE:P29514 EnsemblPlants:AT5G12250.1 GeneID:831100 KEGG:ath:AT5G12250 TAIR:At5g12250 InParanoid:P29514 PhylomeDB:P29514 ArrayExpress:P29514 Genevestigator:P29514 GermOnline:AT5G12250 Uniprot:P29514
Root	Isotig02507	8	60	-3.036	3.21E-12	TAIR|locus:2177003 - symbol:TUB6 "AT5G12250" species:3702 "Arabidopsis thaliana" [GO:0009409 "response to cold" evidence=IEP] [GO:0005200 "structural constituent of cytoskeleton" evidence=ISS] [GO:0007017 "microtubule-based process" evidence=ISS] [GO:0015630 "microtubule cytoskeleton" evidence=ISS] [GO:0005886 "plasma membrane" evidence=IDA] [GO:0009651 "response to salt stress" evidence=IEP] InterPro:IPR000217 InterPro:IPR002453 InterPro:IPR003008 InterPro:IPR008280 InterPro:IPR017975 InterPro:IPR018316 Pfam:PF00091 Pfam:PF03953 PRINTS:PR01161 PRINTS:PR01163 PROSITE:PS00227 SMART:SM00864 SMART:SM00865 GO:GO:0005525 GO:GO:0005886 GO:GO:0005737 EMBL:CP002688 GenomeReviews:BA000015_GR GO:GO:0005198 GO:GO:0003924 GO:GO:0009409 GO:GO:0009651 GO:GO:0005874 GO:GO:0051258 Gene3D:G3DSA:3.40.50.1440 SUPFAM:SSF52490 GO:GO:0007018 EMBL:AB007727 Gene3D:G3DSA:3.30.1330.20 SUPFAM:SSF55307 HOGENOM:HBG750007 InterPro:IPR023123 Gene3D:G3DSA:1.10.287.600 PANTHER:PTHR11588 InterPro:IPR013838 KO:K07375 ProtClustDB:PLN00220 PROSITE:PS00228 OMA:EVINDEH EMBL:M84703 EMBL:AF360260 EMBL:AY040074 IPI:IPI00545934 PIR:JQ1590 RefSeq:NP_196786.1 UniGene:At.23425 UniGene:At.72672 UniGene:At.74848 ProteinModelPortal:P29514 SMR:P29514 STRING:P29514 PRIDE:P29514 EnsemblPlants:AT5G12250.1 GeneID:831100 KEGG:ath:AT5G12250 TAIR:At5g12250 InParanoid:P29514 PhylomeDB:P29514 ArrayExpress:P29514 Genevestigator:P29514 GermOnline:AT5G12250 Uniprot:P29514
Root	Isotig02508	15	39	-1.507	0.000277271	TAIR|locus:2177003 - symbol:TUB6 "AT5G12250" species:3702 "Arabidopsis thaliana" [GO:0009409 "response to cold" evidence=IEP] [GO:0005200 "structural constituent of cytoskeleton" evidence=ISS] [GO:0007017 "microtubule-based process" evidence=ISS] [GO:0015630 "microtubule cytoskeleton" evidence=ISS] [GO:0005886 "plasma membrane" evidence=IDA] [GO:0009651 "response to salt stress" evidence=IEP] InterPro:IPR000217 InterPro:IPR002453 InterPro:IPR003008 InterPro:IPR008280 InterPro:IPR017975 InterPro:IPR018316 Pfam:PF00091 Pfam:PF03953 PRINTS:PR01161 PRINTS:PR01163 PROSITE:PS00227 SMART:SM00864 SMART:SM00865 GO:GO:0005525 GO:GO:0005886 GO:GO:0005737 EMBL:CP002688 GenomeReviews:BA000015_GR GO:GO:0005198 GO:GO:0003924 GO:GO:0009409 GO:GO:0009651 GO:GO:0005874 GO:GO:0051258 Gene3D:G3DSA:3.40.50.1440 SUPFAM:SSF52490 GO:GO:0007018 EMBL:AB007727 Gene3D:G3DSA:3.30.1330.20 SUPFAM:SSF55307 HOGENOM:HBG750007 InterPro:IPR023123 Gene3D:G3DSA:1.10.287.600 PANTHER:PTHR11588 InterPro:IPR013838 KO:K07375 ProtClustDB:PLN00220 PROSITE:PS00228 OMA:EVINDEH EMBL:M84703 EMBL:AF360260 EMBL:AY040074 IPI:IPI00545934 PIR:JQ1590 RefSeq:NP_196786.1 UniGene:At.23425 UniGene:At.72672 UniGene:At.74848 ProteinModelPortal:P29514 SMR:P29514 STRING:P29514 PRIDE:P29514 EnsemblPlants:AT5G12250.1 GeneID:831100 KEGG:ath:AT5G12250 TAIR:At5g12250 InParanoid:P29514 PhylomeDB:P29514 ArrayExpress:P29514 Genevestigator:P29514 GermOnline:AT5G12250 Uniprot:P29514
Root	Isotig02509	13	76	-2.676	1.86E-13	TAIR|locus:2177003 - symbol:TUB6 "AT5G12250" species:3702 "Arabidopsis thaliana" [GO:0009409 "response to cold" evidence=IEP] [GO:0005200 "structural constituent of cytoskeleton" evidence=ISS] [GO:0007017 "microtubule-based process" evidence=ISS] [GO:0015630 "microtubule cytoskeleton" evidence=ISS] [GO:0005886 "plasma membrane" evidence=IDA] [GO:0009651 "response to salt stress" evidence=IEP] InterPro:IPR000217 InterPro:IPR002453 InterPro:IPR003008 InterPro:IPR008280 InterPro:IPR017975 InterPro:IPR018316 Pfam:PF00091 Pfam:PF03953 PRINTS:PR01161 PRINTS:PR01163 PROSITE:PS00227 SMART:SM00864 SMART:SM00865 GO:GO:0005525 GO:GO:0005886 GO:GO:0005737 EMBL:CP002688 GenomeReviews:BA000015_GR GO:GO:0005198 GO:GO:0003924 GO:GO:0009409 GO:GO:0009651 GO:GO:0005874 GO:GO:0051258 Gene3D:G3DSA:3.40.50.1440 SUPFAM:SSF52490 GO:GO:0007018 EMBL:AB007727 Gene3D:G3DSA:3.30.1330.20 SUPFAM:SSF55307 HOGENOM:HBG750007 InterPro:IPR023123 Gene3D:G3DSA:1.10.287.600 PANTHER:PTHR11588 InterPro:IPR013838 KO:K07375 ProtClustDB:PLN00220 PROSITE:PS00228 OMA:EVINDEH EMBL:M84703 EMBL:AF360260 EMBL:AY040074 IPI:IPI00545934 PIR:JQ1590 RefSeq:NP_196786.1 UniGene:At.23425 UniGene:At.72672 UniGene:At.74848 ProteinModelPortal:P29514 SMR:P29514 STRING:P29514 PRIDE:P29514 EnsemblPlants:AT5G12250.1

Tissue	Identification	# of reads (stress)	# of reads (control)	Fold Change	p-value	Description
						GeneID:831100 KEGG:ath:AT5G12250 TAIR:At5g12250 InParanoid:P29514 PhylomeDB:P29514 ArrayExpress:P29514 Genevestigator:P29514 GermOnline:AT5G12250 Uniprot:P29514
Root	Isotig02510	8	61	-3.060	1.76E-12	TAIR|locus:2177003 - symbol:TUB6 "AT5G12250" species:3702 "Arabidopsis thaliana" [GO:0009409 "response to cold" evidence=IEP] [GO:0005200 "structural constituent of cytoskeleton" evidence=ISS] [GO:0007017 "microtubule-based process" evidence=ISS] [GO:0015630 "microtubule cytoskeleton" evidence=ISS] [GO:0005886 "plasma membrane" evidence=IDA] [GO:0009651 "response to salt stress" evidence=IEP] InterPro:IPR000217 InterPro:IPR002453 InterPro:IPR003008 InterPro:IPR008280 InterPro:IPR017975 InterPro:IPR018316 Pfam:PF00091 Pfam:PF03953 PRINTS:PR01161 PRINTS:PR01163 PROSITE:PS00227 SMART:SM00864 SMART:SM00865 GO:GO:0005525 GO:GO:0005886 GO:GO:0005737 EMBL:CP002688 GenomeReviews:BA000015_GR GO:GO:0005198 GO:GO:0003924 GO:GO:0009409 GO:GO:0009651 GO:GO:0005874 GO:GO:0051258 Gene3D:G3DSA:3.40.50.1440 SUPFAM:SSF52490 GO:GO:0007018 EMBL:AB007727 Gene3D:G3DSA:3.30.1330.20 SUPFAM:SSF55307 HOGENOM:HBG750007 InterPro:IPR023123 Gene3D:G3DSA:1.10.287.600 PANTHER:PTHR11588 InterPro:IPR013838 KO:K07375 ProtClustDB:PLN00220 PROSITE:PS00228 OMA:EVINDEH EMBL:M84703 EMBL:AF360260 EMBL:AY040074 IPI:IPI00545934 PIR:JQ1590 RefSeq:NP_196786.1 UniGene:At.23425 UniGene:At.72672 UniGene:At.74848 ProteinModelPortal:P29514 SMR:P29514 STRING:P29514 PRIDE:P29514 EnsemblPlants:AT5G12250.1 GeneID:831100 KEGG:ath:AT5G12250 TAIR:At5g12250 InParanoid:P29514 PhylomeDB:P29514 ArrayExpress:P29514 Genevestigator:P29514 GermOnline:AT5G12250 Uniprot:P29514
Root	Isotig02516	10	34	-1.895	6.62E-05	TAIR|locus:2095913 - symbol:AT3G03990 "AT3G03990" species:3702 "Arabidopsis thaliana" [GO:0003824 "catalytic activity" evidence=ISS] EMBL:CP002686 GO:GO:0016787 EMBL:AC011698 ProtClustDB:CLSN2684507 EMBL:AY064145 EMBL:AY097402 IPI:IPI00546981 RefSeq:NP_566220.1 UniGene:At.27069 ProteinModelPortal:Q9SQR3 SMR:Q9SQR3 MEROPS:S33.A18 PRIDE:Q9SQR3 EnsemblPlants:AT3G03990.1 GeneID:819554 KEGG:ath:AT3G03990 TAIR:At3g03990 InParanoid:Q9SQR3 OMA:CAYVGHS PhylomeDB:Q9SQR3 Genevestigator:Q9SQR3 Uniprot:Q9SQR3
Root	Isotig02517	14	38	-1.570	0.000218579	TAIR|locus:2095913 - symbol:AT3G03990 "AT3G03990" species:3702 "Arabidopsis thaliana" [GO:0003824 "catalytic activity" evidence=ISS] EMBL:CP002686 GO:GO:0016787 EMBL:AC011698 ProtClustDB:CLSN2684507 EMBL:AY064145 EMBL:AY097402 IPI:IPI00546981 RefSeq:NP_566220.1 UniGene:At.27069 ProteinModelPortal:Q9SQR3 SMR:Q9SQR3 MEROPS:S33.A18 PRIDE:Q9SQR3 EnsemblPlants:AT3G03990.1 GeneID:819554 KEGG:ath:AT3G03990 TAIR:At3g03990 InParanoid:Q9SQR3 OMA:CAYVGHS PhylomeDB:Q9SQR3 Genevestigator:Q9SQR3 Uniprot:Q9SQR3
Root	Isotig02519	14	38	-1.570	0.000218579	TAIR|locus:2095913 - symbol:AT3G03990 "AT3G03990" species:3702 "Arabidopsis thaliana" [GO:0003824 "catalytic activity" evidence=ISS] EMBL:CP002686 GO:GO:0016787 EMBL:AC011698 ProtClustDB:CLSN2684507 EMBL:AY064145 EMBL:AY097402 IPI:IPI00546981 RefSeq:NP_566220.1 UniGene:At.27069 ProteinModelPortal:Q9SQR3 SMR:Q9SQR3 MEROPS:S33.A18 PRIDE:Q9SQR3 EnsemblPlants:AT3G03990.1 GeneID:819554 KEGG:ath:AT3G03990 TAIR:At3g03990 InParanoid:Q9SQR3 OMA:CAYVGHS PhylomeDB:Q9SQR3 Genevestigator:Q9SQR3 Uniprot:Q9SQR3
Root	Isotig02536	3	23	-3.068	1.45E-05	TAIR|locus:2119246 - symbol:AT4G33500 species:3702 "Arabidopsis thaliana" [GO:0003824 "catalytic activity" evidence=IEA] [GO:0004721 "phosphoprotein phosphatase activity" evidence=IEA] [GO:0004722 "protein serine/threonine phosphatase activity" evidence=ISS] [GO:0009507 "chloroplast" evidence=IDA] InterPro:IPR001932 Pfam:PF07228 PROSITE:PS01032 SMART:SM00331 SMART:SM00332 EMBL:CP002687 GenomeReviews:CT486007_GR GO:GO:0009507 GO:GO:0046872 EMBL:AL161583 EMBL:AL035678 GO:GO:0004721 eggNOG:COG0631 Gene3D:G3DSA:3.60.40.10 SUPFAM:SSF81606 EMBL:AY035047 EMBL:AY051052 IPI:IPI00523794 PIR:T06001 RefSeq:NP_567923.1 UniGene:At.382 ProteinModelPortal:Q93V88 STRING:Q93V88 PRIDE:Q93V88 EnsemblPlants:AT4G33500.1 GeneID:829488 KEGG:ath:AT4G33500 TAIR:At4g33500 InParanoid:Q93V88 OMA:IAQKPVI PhylomeDB:Q93V88 ProtClustDB:CLSN2722376 Genevestigator:Q93V88 Uniprot:Q93V88
Root	Isotig02537	3	23	-3.068	1.45E-05	TAIR|locus:2119246 - symbol:AT4G33500 species:3702 "Arabidopsis thaliana" [GO:0003824 "catalytic activity" evidence=IEA] [GO:0004721 "phosphoprotein phosphatase activity" evidence=IEA] [GO:0004722 "protein serine/threonine phosphatase activity" evidence=ISS] [GO:0009507 "chloroplast" evidence=IDA] InterPro:IPR001932 Pfam:PF07228 PROSITE:PS01032 SMART:SM00331 SMART:SM00332 EMBL:CP002687 GenomeReviews:CT486007_GR GO:GO:0009507 GO:GO:0046872 EMBL:AL161583 EMBL:AL035678 GO:GO:0004721 eggNOG:COG0631 Gene3D:G3DSA:3.60.40.10 SUPFAM:SSF81606 EMBL:AY035047

Tissue	Identification	# of reads (stress)	# of reads (control)	Fold Change	p-value	Description
						EMBL:AY051052 IPI:IPI00523794 PIR:T06001 RefSeq:NP_567923.1 UniGene:At.382 ProteinModelPortal:Q93V88 STRING:Q93V88 PRIDE:Q93V88 EnsemblPlants:AT4G33500.1 GeneID:829488 KEGG:ath:AT4G33500 TAIR:At4g33500 InParanoid:Q93V88 OMA:IAQKPVI PhylomeDB:Q93V88 ProtClustDB:CLSN2722376 Genevestigator:Q93V88 Uniprot:Q93V88
Root	Isotig02538	3	17	-2.631	0.00056383	TAIR|locus:2119246 - symbol:AT4G33500 species:3702 "Arabidopsis thaliana" [GO:0003824 "catalytic activity" evidence=IEA] [GO:0004721 "phosphoprotein phosphatase activity" evidence=IEA] [GO:0004722 "protein serine/threonine phosphatase activity" evidence=ISS] [GO:0009507 "chloroplast" evidence=IDA] InterPro:IPR001932 Pfam:PF07228 PROSITE:PS01032 SMART:SM00331 SMART:SM00332 EMBL:CP002687 GenomeReviews:CT486007_GR GO:GO:0009507 GO:GO:0046872 EMBL:AL161583 EMBL:AL035678 GO:GO:0004721 eggNOG:COG0631 Gene3D:G3DSA:3.60.40.10 SUPFAM:SSF81606 EMBL:AY035047 EMBL:AY051052 IPI:IPI00523794 PIR:T06001 RefSeq:NP_567923.1 UniGene:At.382 ProteinModelPortal:Q93V88 STRING:Q93V88 PRIDE:Q93V88 EnsemblPlants:AT4G33500.1 GeneID:829488 KEGG:ath:AT4G33500 TAIR:At4g33500 InParanoid:Q93V88 OMA:IAQKPVI PhylomeDB:Q93V88 ProtClustDB:CLSN2722376 Genevestigator:Q93V88 Uniprot:Q93V88
Root	Isotig02561	16	1	3.871	0.000146741	TAIR|locus:2119886 - symbol:AT4G29090 species:3702 "Arabidopsis thaliana" [GO:0003676 "nucleic acid binding" evidence=IEA] [GO:0003723 "RNA binding" evidence=ISS] [GO:0003964 "RNA-directed DNA polymerase activity" evidence=ISS] [GO:0006278 "RNA-dependent DNA replication" evidence=ISS] InterPro:IPR012337 EMBL:CP002687 GenomeReviews:CT486007_GR GO:GO:0003676 SUPFAM:SSF53098 GO:GO:0003964 EMBL:AL078470 EMBL:AL161574 ProtClustDB:CLSN2683532 EMBL:DQ056660 IPI:IPI00548066 PIR:T08964 RefSeq:NP_194638.1 UniGene:At.54541 ProteinModelPortal:Q9SZD8 PRIDE:Q9SZD8 EnsemblPlants:AT4G29090.1 GeneID:829030 KEGG:ath:AT4G29090 TAIR:At4g29090 eggNOG:NOG253635 InParanoid:Q9SZD8 OMA:KESACIR PhylomeDB:Q9SZD8 ArrayExpress:Q9SZD8 Genevestigator:Q9SZD8 Uniprot:Q9SZD8
Root	Isotig02562	16	1	3.871	0.000146741	TAIR|locus:2119886 - symbol:AT4G29090 species:3702 "Arabidopsis thaliana" [GO:0003676 "nucleic acid binding" evidence=IEA] [GO:0003723 "RNA binding" evidence=ISS] [GO:0003964 "RNA-directed DNA polymerase activity" evidence=ISS] [GO:0006278 "RNA-dependent DNA replication" evidence=ISS] InterPro:IPR012337 EMBL:CP002687 GenomeReviews:CT486007_GR GO:GO:0003676 SUPFAM:SSF53098 GO:GO:0003964 EMBL:AL078470 EMBL:AL161574 ProtClustDB:CLSN2683532 EMBL:DQ056660 IPI:IPI00548066 PIR:T08964 RefSeq:NP_194638.1 UniGene:At.54541 ProteinModelPortal:Q9SZD8 PRIDE:Q9SZD8 EnsemblPlants:AT4G29090.1 GeneID:829030 KEGG:ath:AT4G29090 TAIR:At4g29090 eggNOG:NOG253635 InParanoid:Q9SZD8 OMA:KESACIR PhylomeDB:Q9SZD8 ArrayExpress:Q9SZD8 Genevestigator:Q9SZD8 Uniprot:Q9SZD8
Root	Isotig02586	0	19	-5.377	3.67E-06	No hit
Root	Isotig02587	0	19	-5.377	3.67E-06	No hit
Root	Isotig02620	13	1	3.571	0.000882483	TAIR|locus:2142908 - symbol:AT5G35930 species:3702 "Arabidopsis thaliana" [GO:0000036 "acyl carrier activity" evidence=IEA] [GO:0003824 "catalytic activity" evidence=IEA] [GO:0005575 "cellular_component" evidence=ND] [GO:0008152 "metabolic process" evidence=IEA] [GO:0016208 "AMP binding" evidence=ISS] [GO:0031177 "phosphopantetheine binding" evidence=IEA] [GO:0048037 "cofactor binding" evidence=IEA] InterPro:IPR000873 InterPro:IPR006162 Pfam:PF00501 PROSITE:PS00012 InterPro:IPR006163 InterPro:IPR009081 Pfam:PF00550 Prosite:PS00455 EMBL:CP002688 InterPro:IPR020845 GO:GO:0016874 GO:GO:0003824 GO:GO:0000036 GO:GO:0048037 GO:GO:0031177 Gene3D:G3DSA:1.10.1200.10 SUPFAM:SSF47336 PROSITE:PS50075 eggNOG:COG1520 KO:K00142 InterPro:IPR018391 InterPro:IPR011047 Gene3D:G3DSA:2.140.10.10 SMART:SM00564 SUPFAM:SSF50998 EMBL:AB025602 EMBL:AY099772 IPI:IPI00520530 RefSeq:NP_198442.2 UniGene:At.30571 SMR:F4K1G2 PRIDE:Q9LTK8 EnsemblPlants:AT5G35930.1 GeneID:833582 KEGG:ath:AT5G35930 TAIR:At5g35930 Uniprot:F4K1G2
Root	Isotig02621	13	1	3.571	0.000882483	TAIR|locus:2142908 - symbol:AT5G35930 species:3702 "Arabidopsis thaliana" [GO:0000036 "acyl carrier activity" evidence=IEA] [GO:0003824 "catalytic activity" evidence=IEA] [GO:0005575 "cellular_component" evidence=ND] [GO:0008152 "metabolic process" evidence=IEA] [GO:0016208 "AMP binding" evidence=ISS] [GO:0031177 "phosphopantetheine binding" evidence=IEA] [GO:0048037 "cofactor binding" evidence=IEA] InterPro:IPR000873 InterPro:IPR006162 Pfam:PF00501 PROSITE:PS00012 InterPro:IPR006163 InterPro:IPR009081 Pfam:PF00550 Prosite:PS00455 EMBL:CP002688 InterPro:IPR020845 GO:GO:0016874 GO:GO:0003824 GO:GO:0000036 GO:GO:0048037 GO:GO:0031177 Gene3D:G3DSA:1.10.1200.10 SUPFAM:SSF47336 PROSITE:PS50075

Tissue	Identification	# of reads (stress)	# of reads (control)	Fold Change	p-value	Description
						eggNOG:COG1520 KO:K00142 InterPro:IPR018391 InterPro:IPR011047 Gene3D:G3DSA:2.140.10.10 SMART:SM00564 SUPFAM:SSF50998 EMBL:AB025602 EMBL:AY099772 IPI:IPI00520530 RefSeq:NP_198442.2 UniGene:At.30571 SMR:F4K1G2 PRIDE:Q9LTK8 EnsemblPlants:AT5G35930.1 GeneID:833582 KEGG:ath:AT5G35930 TAIR:At5g35930 Uniprot:F4K1G2
Root	Isotig02622	13	1	3.571	0.000882483	TAIR|locus:2142908 - symbol:AT5G35930 species:3702 "Arabidopsis thaliana" [GO:0000036 "acyl carrier activity" evidence=IEA] [GO:0003824 "catalytic activity" evidence=IEA] [GO:0005575 "cellular_component" evidence=ND] [GO:0008152 "metabolic process" evidence=IEA] [GO:0016208 "AMP binding" evidence=ISS] [GO:0031177 "phosphopantetheine binding" evidence=IEA] [GO:0048037 "cofactor binding" evidence=IEA] InterPro:IPR000873 InterPro:IPR006162 Pfam:PF00501 PROSITE:PS00012 InterPro:IPR006163 InterPro:IPR009081 Pfam:PF00550 Prosite:PS00455 EMBL:CP002688 InterPro:IPR020845 GO:GO:0016874 GO:GO:0003824 GO:GO:0000036 GO:GO:0048037 GO:GO:0031177 Gene3D:G3DSA:1.10.1200.10 SUPFAM:SSF47336 PROSITE:PS50075 eggNOG:COG1520 KO:K00142 InterPro:IPR018391 InterPro:IPR011047 Gene3D:G3DSA:2.140.10.10 SMART:SM00564 SUPFAM:SSF50998 EMBL:AB025602 EMBL:AY099772 IPI:IPI00520530 RefSeq:NP_198442.2 UniGene:At.30571 SMR:F4K1G2 PRIDE:Q9LTK8 EnsemblPlants:AT5G35930.1 GeneID:833582 KEGG:ath:AT5G35930 TAIR:At5g35930 Uniprot:F4K1G2
Root	Isotig02623	13	1	3.571	0.000882483	TAIR|locus:2142908 - symbol:AT5G35930 species:3702 "Arabidopsis thaliana" [GO:0000036 "acyl carrier activity" evidence=IEA] [GO:0003824 "catalytic activity" evidence=IEA] [GO:0005575 "cellular_component" evidence=ND] [GO:0008152 "metabolic process" evidence=IEA] [GO:0016208 "AMP binding" evidence=ISS] [GO:0031177 "phosphopantetheine binding" evidence=IEA] [GO:0048037 "cofactor binding" evidence=IEA] InterPro:IPR000873 InterPro:IPR006162 Pfam:PF00501 PROSITE:PS00012 InterPro:IPR006163 InterPro:IPR009081 Pfam:PF00550 Prosite:PS00455 EMBL:CP002688 InterPro:IPR020845 GO:GO:0016874 GO:GO:0003824 GO:GO:0000036 GO:GO:0048037 GO:GO:0031177 Gene3D:G3DSA:1.10.1200.10 SUPFAM:SSF47336 PROSITE:PS50075 eggNOG:COG1520 KO:K00142 InterPro:IPR018391 InterPro:IPR011047 Gene3D:G3DSA:2.140.10.10 SMART:SM00564 SUPFAM:SSF50998 EMBL:AB025602 EMBL:AY099772 IPI:IPI00520530 RefSeq:NP_198442.2 UniGene:At.30571 SMR:F4K1G2 PRIDE:Q9LTK8 EnsemblPlants:AT5G35930.1 GeneID:833582 KEGG:ath:AT5G35930 TAIR:At5g35930 Uniprot:F4K1G2
Root	Isotig02640	19	50	-1.525	3.30E-05	TAIR|locus:2135237 - symbol:AGT2 "AT4G39660" species:3702 "Arabidopsis thaliana" [GO:0008453 "alanine-glyoxylate transaminase activity" evidence=ISS;IDA] [GO:0005739 "mitochondrion" evidence=IDA] [GO:0019544 "arginine catabolic process to glutamate" evidence=IDA] [GO:0046686 "response to cadmium ion" evidence=IEP] [GO:0005774 "vacuolar membrane" evidence=IDA] [GO:0016020 "membrane" evidence=IDA] [GO:0008270 "zinc ion binding" evidence=IDA] InterPro:IPR005814 InterPro:IPR015421 InterPro:IPR015422 Pfam:PF00202 PROSITE:PS00600 GO:GO:0005739 EMBL:CP002687 GenomeReviews:CT486007_GR GO:GO:0046686 GO:GO:0005774 GO:GO:0030170 InterPro:IPR015424 Gene3D:G3DSA:3.40.640.10 Gene3D:G3DSA:3.90.1150.10 SUPFAM:SSF53383 GO:GO:0008270 EMBL:AF166351 EMBL:AL022605 EMBL:AL161595 EMBL:AY054264 EMBL:BT002306 IPI:IPI00519673 PIR:T05003 RefSeq:NP_568064.1 UniGene:At.22488 ProteinModelPortal:Q940M2 SMR:Q940M2 STRING:Q940M2 PRIDE:Q940M2 EnsemblPlants:AT4G39660.1 GeneID:830120 KEGG:ath:AT4G39660 GeneFarm:5077 TAIR:At4g39660 eggNOG:COG0160 HOGENOM:HBG725944 InParanoid:Q940M2 KO:K00827 OMA:GIVTVSC PhylomeDB:Q940M2 ProtClustDB:CLSN2683116 ArrayExpress:Q9SU41 Genevestigator:Q940M2 GermOnline:AT4G39660 GO:GO:0008453 GO:GO:0019544 GO:GO:0009853 PANTHER:PTHR11986 Uniprot:Q940M2
Root	Isotig02641	19	45	-1.373	0.000271992	TAIR|locus:2135237 - symbol:AGT2 "AT4G39660" species:3702 "Arabidopsis thaliana" [GO:0008453 "alanine-glyoxylate transaminase activity" evidence=ISS;IDA] [GO:0005739 "mitochondrion" evidence=IDA] [GO:0019544 "arginine catabolic process to glutamate" evidence=IDA] [GO:0046686 "response to cadmium ion" evidence=IEP] [GO:0005774 "vacuolar membrane" evidence=IDA] [GO:0016020 "membrane" evidence=IDA] [GO:0008270 "zinc ion binding" evidence=IDA] InterPro:IPR005814 InterPro:IPR015421 InterPro:IPR015422 Pfam:PF00202 PROSITE:PS00600 GO:GO:0005739 EMBL:CP002687 GenomeReviews:CT486007_GR GO:GO:0046686 GO:GO:0005774 GO:GO:0030170 InterPro:IPR015424 Gene3D:G3DSA:3.40.640.10 Gene3D:G3DSA:3.90.1150.10 SUPFAM:SSF53383 GO:GO:0008270 EMBL:AF166351 EMBL:AL022605 EMBL:AL161595 EMBL:AY054264 EMBL:BT002306 IPI:IPI00519673 PIR:T05003 RefSeq:NP_568064.1 UniGene:At.22488 ProteinModelPortal:Q940M2 SMR:Q940M2 STRING:Q940M2 PRIDE:Q940M2 EnsemblPlants:AT4G39660.1 GeneID:830120 KEGG:ath:AT4G39660 GeneFarm:5077 TAIR:At4g39660 eggNOG:COG0160 HOGENOM:HBG725944 InParanoid:Q940M2 KO:K00827 OMA:GIVTVSC PhylomeDB:Q940M2

Tissue	Identification	# of reads (stress)	# of reads (control)	Fold Change	p-value	Description
						ProtClustDB:CLSN2683116 ArrayExpress:Q9SU41 Genevestigator:Q940M2 GermOnline:AT4G39660 GO:GO:0008453 GO:GO:0019544 GO:GO:0009853 PANTHER:PTHR11986 Uniprot:Q940M2
Root	Isotig02644	1	20	-4.451	3.92E-06	TAIR|locus:2008855 - symbol:NRT1.1 "AT1G12110" species:3702 "Arabidopsis thaliana" [GO:0005215 "transporter activity" evidence=ISS] [GO:0009414 "response to water deprivation" evidence=IMP] [GO:0010167 "response to nitrate" evidence=IMP] [GO:0005886 "plasma membrane" evidence=IDA] [GO:0005515 "protein binding" evidence=IPI] [GO:0015112 "nitrate transmembrane transporter activity" evidence=IMP] [GO:0015706 "nitrate transport" evidence=IMP] InterPro:IPR000109 InterPro:IPR018456 Pfam:PF00854 PROSITE:PS01022 PROSITE:PS01023 GO:GO:0016021 EMBL:CP002684 GenomeReviews:CT485782_GR GO:GO:0005886 GO:GO:0005515 GO:GO:0009734 GO:GO:0009635 GO:GO:0015293 GO:GO:0009414 InterPro:IPR016196 SUPFAM:SSF103473 EMBL:AC002131 GO:GO:0015112 GO:GO:0042128 GO:GO:0010167 eggNOG:COG3104 GO:GO:0006857 HOGENOM:HBG605267 EMBL:L10357 EMBL:BT002016 EMBL:BT008783 EMBL:AK317498 IPI:IPI00534102 PIR:A45772 RefSeq:NP_563899.1 UniGene:At.19624 ProteinModelPortal:Q05085 IntAct:Q05085 STRING:Q05085 TCDB:2.A.17.3.1 PRIDE:Q05085 EnsemblPlants:AT1G12110.1 GeneID:837763 KEGG:ath:AT1G12110 TAIR:At1g12110 InParanoid:Q05085 OMA:ICAFAIV PhylomeDB:Q05085 ProtClustDB:CLSN2916999 ArrayExpress:Q05085 Genevestigator:Q05085 GermOnline:AT1G12110 Uniprot:Q05085
Root	Isotig02645	1	20	-4.451	3.92E-06	TAIR|locus:2008855 - symbol:NRT1.1 "AT1G12110" species:3702 "Arabidopsis thaliana" [GO:0005215 "transporter activity" evidence=ISS] [GO:0009414 "response to water deprivation" evidence=IMP] [GO:0010167 "response to nitrate" evidence=IMP] [GO:0005886 "plasma membrane" evidence=IDA] [GO:0005515 "protein binding" evidence=IPI] [GO:0015112 "nitrate transmembrane transporter activity" evidence=IMP] [GO:0015706 "nitrate transport" evidence=IMP] InterPro:IPR000109 InterPro:IPR018456 Pfam:PF00854 PROSITE:PS01022 PROSITE:PS01023 GO:GO:0016021 EMBL:CP002684 GenomeReviews:CT485782_GR GO:GO:0005886 GO:GO:0005515 GO:GO:0009734 GO:GO:0009635 GO:GO:0015293 GO:GO:0009414 InterPro:IPR016196 SUPFAM:SSF103473 EMBL:AC002131 GO:GO:0015112 GO:GO:0042128 GO:GO:0010167 eggNOG:COG3104 GO:GO:0006857 HOGENOM:HBG605267 EMBL:L10357 EMBL:BT002016 EMBL:BT008783 EMBL:AK317498 IPI:IPI00534102 PIR:A45772 RefSeq:NP_563899.1 UniGene:At.19624 ProteinModelPortal:Q05085 IntAct:Q05085 STRING:Q05085 TCDB:2.A.17.3.1 PRIDE:Q05085 EnsemblPlants:AT1G12110.1 GeneID:837763 KEGG:ath:AT1G12110 TAIR:At1g12110 InParanoid:Q05085 OMA:ICAFAIV PhylomeDB:Q05085 ProtClustDB:CLSN2916999 ArrayExpress:Q05085 Genevestigator:Q05085 GermOnline:AT1G12110 Uniprot:Q05085
Root	Isotig02646	1	20	-4.451	3.92E-06	TAIR|locus:2008855 - symbol:NRT1.1 "AT1G12110" species:3702 "Arabidopsis thaliana" [GO:0005215 "transporter activity" evidence=ISS] [GO:0009414 "response to water deprivation" evidence=IMP] [GO:0010167 "response to nitrate" evidence=IMP] [GO:0005886 "plasma membrane" evidence=IDA] [GO:0005515 "protein binding" evidence=IPI] [GO:0015112 "nitrate transmembrane transporter activity" evidence=IMP] [GO:0015706 "nitrate transport" evidence=IMP] InterPro:IPR000109 InterPro:IPR018456 Pfam:PF00854 PROSITE:PS01022 PROSITE:PS01023 GO:GO:0016021 EMBL:CP002684 GenomeReviews:CT485782_GR GO:GO:0005886 GO:GO:0005515 GO:GO:0009734 GO:GO:0009635 GO:GO:0015293 GO:GO:0009414 InterPro:IPR016196 SUPFAM:SSF103473 EMBL:AC002131 GO:GO:0015112 GO:GO:0042128 GO:GO:0010167 eggNOG:COG3104 GO:GO:0006857 HOGENOM:HBG605267 EMBL:L10357 EMBL:BT002016 EMBL:BT008783 EMBL:AK317498 IPI:IPI00534102 PIR:A45772 RefSeq:NP_563899.1 UniGene:At.19624 ProteinModelPortal:Q05085 IntAct:Q05085 STRING:Q05085 TCDB:2.A.17.3.1 PRIDE:Q05085 EnsemblPlants:AT1G12110.1 GeneID:837763 KEGG:ath:AT1G12110 TAIR:At1g12110 InParanoid:Q05085 OMA:ICAFAIV PhylomeDB:Q05085 ProtClustDB:CLSN2916999 ArrayExpress:Q05085 Genevestigator:Q05085 GermOnline:AT1G12110 Uniprot:Q05085
Root	Isotig02656	10	50	-2.451	1.32E-08	TAIR|locus:2127776 - symbol:CESA1 "AT4G32410" species:3702 "Arabidopsis thaliana" [GO:0016757 "transferase activity, transferring glycosyl groups" evidence=ISS] [GO:0016759 "cellulose synthase activity" evidence=ISS] [GO:0009832 "plant-type cell wall biogenesis" evidence=TAS] [GO:0030244 "cellulose biosynthetic process" evidence=IMP;TAS] [GO:0009833 "primary cell wall biogenesis" evidence=IMP] [GO:0005886 "plasma membrane" evidence=IDA] [GO:0005794 "Golgi apparatus" evidence=IDA] [GO:0042538 "hyperosmotic salinity response" evidence=IMP] InterPro:IPR001841 InterPro:IPR005150 Pfam:PF03552 PROSITE:PS50089 SMART:SM00184 Prosite:PS00518 GO:GO:0016021 GO:GO:0005886 EMBL:CP002687 GenomeReviews:CT486007_GR GO:GO:0005794 GO:GO:0008270 InterPro:IPR013083 Gene3D:G3DSA:3.30.40.10 GO:GO:0007047

Tissue	Identification	# of reads (stress)	# of reads (control)	Fold Change	p-value	Description
						EMBL:AL161581 EMBL:AL034567 CAZy:GT2 GO:GO:0042538 eggNOG:COG1215 GO:GO:0016760 GO:GO:0030244 EMBL:AF027172 EMBL:BT008654 EMBL:AK222115 EMBL:AK226243 IPI:IPI00536785 PIR:T05351 RefSeq:NP_194967.1 UniGene:At.21246 ProteinModelPortal:O48946 SMR:O48946 DIP:DIP-59354N STRING:O48946 PRIDE:O48946 EnsemblPlants:AT4G32410.1 GeneID:829376 KEGG:ath:AT4G32410 GeneFarm:5084 TAIR:At4g32410 HOGENOM:HBG744549 InParanoid:O48946 KO:K10999 OMA:DIENEFN PhylomeDB:O48946 ProtClustDB:PLN02400 Genevestigator:O48946 GermOnline:AT4G32410 GO:GO:0009833 Uniprot:O48946
Root	Isotig02657	10	49	-2.422	2.30E-08	TAIR|locus:2127776 - symbol:CESA1 "AT4G32410" species:3702 "Arabidopsis thaliana" [GO:0016757 "transferase activity, transferring glycosyl groups" evidence=ISS] [GO:0016759 "cellulose synthase activity" evidence=ISS] [GO:0009832 "plant-type cell wall biogenesis" evidence=TAS] [GO:0030244 "cellulose biosynthetic process" evidence=IMP;TAS] [GO:0009833 "primary cell wall biogenesis" evidence=IMP] [GO:0005886 "plasma membrane" evidence=IDA] [GO:0005794 "Golgi apparatus" evidence=IDA] [GO:0042538 "hyperosmotic salinity response" evidence=IMP] InterPro:IPR001841 InterPro:IPR005150 Pfam:PF03552 PROSITE:PS50089 SMART:SM00184 Prosite:PS00518 GO:GO:0016021 GO:GO:0005886 EMBL:CP002687 GenomeReviews:CT486007_GR GO:GO:0005794 GO:GO:0008270 InterPro:IPR013083 Gene3D:G3DSA:3.30.40.10 GO:GO:0007047 EMBL:AL161581 EMBL:AL034567 CAZy:GT2 GO:GO:0042538 eggNOG:COG1215 GO:GO:0016760 GO:GO:0030244 EMBL:AF027172 EMBL:BT008654 EMBL:AK222115 EMBL:AK226243 IPI:IPI00536785 PIR:T05351 RefSeq:NP_194967.1 UniGene:At.21246 ProteinModelPortal:O48946 SMR:O48946 DIP:DIP-59354N STRING:O48946 PRIDE:O48946 EnsemblPlants:AT4G32410.1 GeneID:829376 KEGG:ath:AT4G32410 GeneFarm:5084 TAIR:At4g32410 HOGENOM:HBG744549 InParanoid:O48946 KO:K10999 OMA:DIENEFN PhylomeDB:O48946 ProtClustDB:PLN02400 Genevestigator:O48946 GermOnline:AT4G32410 GO:GO:0009833 Uniprot:O48946
Root	Isotig02658	11	50	-2.313	3.93E-08	TAIR|locus:2127776 - symbol:CESA1 "AT4G32410" species:3702 "Arabidopsis thaliana" [GO:0016757 "transferase activity, transferring glycosyl groups" evidence=ISS] [GO:0016759 "cellulose synthase activity" evidence=ISS] [GO:0009832 "plant-type cell wall biogenesis" evidence=TAS] [GO:0030244 "cellulose biosynthetic process" evidence=IMP;TAS] [GO:0009833 "primary cell wall biogenesis" evidence=IMP] [GO:0005886 "plasma membrane" evidence=IDA] [GO:0005794 "Golgi apparatus" evidence=IDA] [GO:0042538 "hyperosmotic salinity response" evidence=IMP] InterPro:IPR001841 InterPro:IPR005150 Pfam:PF03552 PROSITE:PS50089 SMART:SM00184 Prosite:PS00518 GO:GO:0016021 GO:GO:0005886 EMBL:CP002687 GenomeReviews:CT486007_GR GO:GO:0005794 GO:GO:0008270 InterPro:IPR013083 Gene3D:G3DSA:3.30.40.10 GO:GO:0007047 EMBL:AL161581 EMBL:AL034567 CAZy:GT2 GO:GO:0042538 eggNOG:COG1215 GO:GO:0016760 GO:GO:0030244 EMBL:AF027172 EMBL:BT008654 EMBL:AK222115 EMBL:AK226243 IPI:IPI00536785 PIR:T05351 RefSeq:NP_194967.1 UniGene:At.21246 ProteinModelPortal:O48946 SMR:O48946 DIP:DIP-59354N STRING:O48946 PRIDE:O48946 EnsemblPlants:AT4G32410.1 GeneID:829376 KEGG:ath:AT4G32410 GeneFarm:5084 TAIR:At4g32410 HOGENOM:HBG744549 InParanoid:O48946 KO:K10999 OMA:DIENEFN PhylomeDB:O48946 ProtClustDB:PLN02400 Genevestigator:O48946 GermOnline:AT4G32410 GO:GO:0009833 Uniprot:O48946
Root	Isotig02659	10	37	-2.016	1.44E-05	TAIR|locus:2127776 - symbol:CESA1 "AT4G32410" species:3702 "Arabidopsis thaliana" [GO:0016757 "transferase activity, transferring glycosyl groups" evidence=ISS] [GO:0016759 "cellulose synthase activity" evidence=ISS] [GO:0009832 "plant-type cell wall biogenesis" evidence=TAS] [GO:0030244 "cellulose biosynthetic process" evidence=IMP;TAS] [GO:0009833 "primary cell wall biogenesis" evidence=IMP] [GO:0005886 "plasma membrane" evidence=IDA] [GO:0005794 "Golgi apparatus" evidence=IDA] [GO:0042538 "hyperosmotic salinity response" evidence=IMP] InterPro:IPR001841 InterPro:IPR005150 Pfam:PF03552 PROSITE:PS50089 SMART:SM00184 Prosite:PS00518 GO:GO:0016021 GO:GO:0005886 EMBL:CP002687 GenomeReviews:CT486007_GR GO:GO:0005794 GO:GO:0008270 InterPro:IPR013083 Gene3D:G3DSA:3.30.40.10 GO:GO:0007047 EMBL:AL161581 EMBL:AL034567 CAZy:GT2 GO:GO:0042538 eggNOG:COG1215 GO:GO:0016760 GO:GO:0030244 EMBL:AF027172 EMBL:BT008654 EMBL:AK222115 EMBL:AK226243 IPI:IPI00536785 PIR:T05351 RefSeq:NP_194967.1 UniGene:At.21246 ProteinModelPortal:O48946 SMR:O48946 DIP:DIP-59354N STRING:O48946 PRIDE:O48946 EnsemblPlants:AT4G32410.1 GeneID:829376 KEGG:ath:AT4G32410 GeneFarm:5084 TAIR:At4g32410 HOGENOM:HBG744549 InParanoid:O48946 KO:K10999 OMA:DIENEFN PhylomeDB:O48946 ProtClustDB:PLN02400 Genevestigator:O48946 GermOnline:AT4G32410 GO:GO:0009833 Uniprot:O48946

Tissue	Identification	# of reads (stress)	# of reads (control)	Fold Change	p-value	Description
Root	Isotig02672	20	0	5.193	5.94E-06	TAIR|locus:2036029 - symbol:AMI1 "AT1G08980" species:3702 "Arabidopsis thaliana" [GO:0004040 "amidase activity" evidence=ISS;IDA] [GO:0009684 "indoleacetic acid biosynthetic process" evidence=IDA] [GO:0016810 "hydrolase activity, acting on carbon-nitrogen (but not peptide) bonds" evidence=IDA] [GO:0043864 "indoleacetamide hydrolase activity" evidence=IDA] [GO:0005737 "cytoplasm" evidence=IDA] [GO:0005829 "cytosol" evidence=IDA] InterPro:IPR000120 Pfam:PF01425 EMBL:CP002684 GenomeReviews:CT485782_GR GO:GO:0005829 GO:GO:0004040 GO:GO:0016884 eggNOG:COG0154 InterPro:IPR020556 InterPro:IPR023631 Gene3D:G3DSA:3.90.1300.10 PANTHER:PTHR11895 SUPFAM:SSF75304 PROSITE:PS00571 EMBL:AF202077 EMBL:AC000106 EMBL:AY037198 EMBL:BT004554 IPI:IPI00537072 IPI:IPI00548661 PIR:F86221 RefSeq:NP_563831.1 UniGene:At.21299 HSSP:Q8RJN5 ProteinModelPortal:Q9FR37 SMR:Q9FR37 STRING:Q9FR37 PRIDE:Q9FR37 EnsemblPlants:AT1G08980.1 GeneID:837418 KEGG:ath:AT1G08980 TAIR:At1g08980 HOGENOM:HBG481888 InParanoid:Q9FR37 OMA:IQGAEAW PhylomeDB:Q9FR37 ProtClustDB:PLN02722 Genevestigator:Q9FR37 GO:GO:0043864 GO:GO:0009684 Uniprot:Q9FR37
Root	Isotig02673	21	0	5.263	3.38E-06	TAIR|locus:2036029 - symbol:AMI1 "AT1G08980" species:3702 "Arabidopsis thaliana" [GO:0004040 "amidase activity" evidence=ISS;IDA] [GO:0009684 "indoleacetic acid biosynthetic process" evidence=IDA] [GO:0016810 "hydrolase activity, acting on carbon-nitrogen (but not peptide) bonds" evidence=IDA] [GO:0043864 "indoleacetamide hydrolase activity" evidence=IDA] [GO:0005737 "cytoplasm" evidence=IDA] [GO:0005829 "cytosol" evidence=IDA] InterPro:IPR000120 Pfam:PF01425 EMBL:CP002684 GenomeReviews:CT485782_GR GO:GO:0005829 GO:GO:0004040 GO:GO:0016884 eggNOG:COG0154 InterPro:IPR020556 InterPro:IPR023631 Gene3D:G3DSA:3.90.1300.10 PANTHER:PTHR11895 SUPFAM:SSF75304 PROSITE:PS00571 EMBL:AF202077 EMBL:AC000106 EMBL:AY037198 EMBL:BT004554 IPI:IPI00537072 IPI:IPI00548661 PIR:F86221 RefSeq:NP_563831.1 UniGene:At.21299 HSSP:Q8RJN5 ProteinModelPortal:Q9FR37 SMR:Q9FR37 STRING:Q9FR37 PRIDE:Q9FR37 EnsemblPlants:AT1G08980.1 GeneID:837418 KEGG:ath:AT1G08980 TAIR:At1g08980 HOGENOM:HBG481888 InParanoid:Q9FR37 OMA:IQGAEAW PhylomeDB:Q9FR37 ProtClustDB:PLN02722 Genevestigator:Q9FR37 GO:GO:0043864 GO:GO:0009684 Uniprot:Q9FR37
Root	Isotig02674	20	0	5.193	5.94E-06	TAIR|locus:2036029 - symbol:AMI1 "AT1G08980" species:3702 "Arabidopsis thaliana" [GO:0004040 "amidase activity" evidence=ISS;IDA] [GO:0009684 "indoleacetic acid biosynthetic process" evidence=IDA] [GO:0016810 "hydrolase activity, acting on carbon-nitrogen (but not peptide) bonds" evidence=IDA] [GO:0043864 "indoleacetamide hydrolase activity" evidence=IDA] [GO:0005737 "cytoplasm" evidence=IDA] [GO:0005829 "cytosol" evidence=IDA] InterPro:IPR000120 Pfam:PF01425 EMBL:CP002684 GenomeReviews:CT485782_GR GO:GO:0005829 GO:GO:0004040 GO:GO:0016884 eggNOG:COG0154 InterPro:IPR020556 InterPro:IPR023631 Gene3D:G3DSA:3.90.1300.10 PANTHER:PTHR11895 SUPFAM:SSF75304 PROSITE:PS00571 EMBL:AF202077 EMBL:AC000106 EMBL:AY037198 EMBL:BT004554 IPI:IPI00537072 IPI:IPI00548661 PIR:F86221 RefSeq:NP_563831.1 UniGene:At.21299 HSSP:Q8RJN5 ProteinModelPortal:Q9FR37 SMR:Q9FR37 STRING:Q9FR37 PRIDE:Q9FR37 EnsemblPlants:AT1G08980.1 GeneID:837418 KEGG:ath:AT1G08980 TAIR:At1g08980 HOGENOM:HBG481888 InParanoid:Q9FR37 OMA:IQGAEAW PhylomeDB:Q9FR37 ProtClustDB:PLN02722 Genevestigator:Q9FR37 GO:GO:0043864 GO:GO:0009684 Uniprot:Q9FR37
Root	Isotig02675	21	0	5.263	3.38E-06	TAIR|locus:2036029 - symbol:AMI1 "AT1G08980" species:3702 "Arabidopsis thaliana" [GO:0004040 "amidase activity" evidence=ISS;IDA] [GO:0009684 "indoleacetic acid biosynthetic process" evidence=IDA] [GO:0016810 "hydrolase activity, acting on carbon-nitrogen (but not peptide) bonds" evidence=IDA] [GO:0043864 "indoleacetamide hydrolase activity" evidence=IDA] [GO:0005737 "cytoplasm" evidence=IDA] [GO:0005829 "cytosol" evidence=IDA] InterPro:IPR000120 Pfam:PF01425 EMBL:CP002684 GenomeReviews:CT485782_GR GO:GO:0005829 GO:GO:0004040 GO:GO:0016884 eggNOG:COG0154 InterPro:IPR020556 InterPro:IPR023631 Gene3D:G3DSA:3.90.1300.10 PANTHER:PTHR11895 SUPFAM:SSF75304 PROSITE:PS00571 EMBL:AF202077 EMBL:AC000106 EMBL:AY037198 EMBL:BT004554 IPI:IPI00537072 IPI:IPI00548661 PIR:F86221 RefSeq:NP_563831.1 UniGene:At.21299 HSSP:Q8RJN5 ProteinModelPortal:Q9FR37 SMR:Q9FR37 STRING:Q9FR37 PRIDE:Q9FR37 EnsemblPlants:AT1G08980.1 GeneID:837418 KEGG:ath:AT1G08980 TAIR:At1g08980 HOGENOM:HBG481888 InParanoid:Q9FR37 OMA:IQGAEAW PhylomeDB:Q9FR37 ProtClustDB:PLN02722 Genevestigator:Q9FR37 GO:GO:0043864 GO:GO:0009684 Uniprot:Q9FR37
Root	Isotig02680	97	4	4.471	2.23E-22	TAIR|locus:2017899 - symbol:SGB1 "AT1G79820" species:3702 "Arabidopsis thaliana" [GO:0005351 "sugar:hydrogen symporter activity" evidence=ISS] [GO:0006810 "transport" evidence=IEA] [GO:0015144 "carbohydrate transmembrane transporter activity" evidence=ISS] [GO:0016020 "membrane" evidence=ISS] [GO:0055085 "transmembrane transport" evidence=IEA] [GO:0005794 "Golgi apparatus" evidence=IDA]

Tissue	Identification	# of reads (stress)	# of reads (control)	Fold Change	p-value	Description
						InterPro:IPR003663 InterPro:IPR005828 InterPro:IPR005829 Pfam:PF00083 PRINTS:PR00171 PROSITE:PS00216 PROSITE:PS00217 GO:GO:0016021 EMBL:CP002684 GenomeReviews:CT485782_GR GO:GO:0031969 eggNOG:COG0477 InterPro:IPR016196 SUPFAM:SSF103473 GO:GO:0008643 InterPro:IPR020846 PROSITE:PS50850 EMBL:AC010793 EMBL:AC011717 HOGENOM:HBG744444 GO:GO:0022891 TIGRFAMs:TIGR00879 UniGene:At.14743 UniGene:At.48399 EMBL:AY080624 EMBL:BT008550 EMBL:BT008693 IPI:IPI00536398 IPI:IPI00657574 IPI:IPI00810560 IPI:IPI00810565 PIR:B96829 RefSeq:NP_001031303.1 RefSeq:NP_178100.3 RefSeq:NP_850983.1 ProteinModelPortal:Q2V4B9 PRIDE:Q2V4B9 EnsemblPlants:AT1G79820.1 EnsemblPlants:AT1G79820.2 GeneID:844321 KEGG:ath:AT1G79820 TAIR:At1g79820 InParanoid:Q2V4B9 OMA:MELCVES PhylomeDB:Q2V4B9 ProtClustDB:CLSN2680669 Genevestigator:Q2V4B9 GermOnline:AT1G79820 Uniprot:Q2V4B9
Root	Isotig02681	93	4	4.410	2.14E-21	TAIR|locus:2019554 - symbol:AT1G67300 "AT1G67300" species:3702 "Arabidopsis thaliana" [GO:0005351 "sugar:hydrogen symporter activity" evidence=ISS] [GO:0006810 "transport" evidence=IEA] [GO:0015144 "carbohydrate transmembrane transporter activity" evidence=ISS] [GO:0016020 "membrane" evidence=IEA;ISS] [GO:0016021 "integral to membrane" evidence=IEA] [GO:0055085 "transmembrane transport" evidence=IEA] InterPro:IPR003663 InterPro:IPR005828 InterPro:IPR005829 Pfam:PF00083 PRINTS:PR00171 PROSITE:PS00216 PROSITE:PS00217 GO:GO:0016021 EMBL:CP002684 GenomeReviews:CT485782_GR GO:GO:0031969 eggNOG:COG0477 InterPro:IPR016196 SUPFAM:SSF103473 GO:GO:0008643 InterPro:IPR020846 PROSITE:PS50850 HOGENOM:HBG744444 GO:GO:0022891 TIGRFAMs:TIGR00879 EMBL:AC002130 EMBL:AK226899 IPI:IPI00539133 PIR:F96696 RefSeq:NP_176898.1 UniGene:At.21607 UniGene:At.67017 ProteinModelPortal:Q9FYG3 EnsemblPlants:AT1G67300.1 GeneID:843050 KEGG:ath:AT1G67300 TAIR:At1g67300 InParanoid:Q9FYG3 PhylomeDB:Q9FYG3 ProtClustDB:CLSN2681973 Genevestigator:Q9FYG3 Uniprot:Q9FYG3
Root	Isotig02682	84	6	3.678	1.27E-17	TAIR|locus:2019554 - symbol:AT1G67300 "AT1G67300" species:3702 "Arabidopsis thaliana" [GO:0005351 "sugar:hydrogen symporter activity" evidence=ISS] [GO:0006810 "transport" evidence=IEA] [GO:0015144 "carbohydrate transmembrane transporter activity" evidence=ISS] [GO:0016020 "membrane" evidence=IEA;ISS] [GO:0016021 "integral to membrane" evidence=IEA] [GO:0055085 "transmembrane transport" evidence=IEA] InterPro:IPR003663 InterPro:IPR005828 InterPro:IPR005829 Pfam:PF00083 PRINTS:PR00171 PROSITE:PS00216 PROSITE:PS00217 GO:GO:0016021 EMBL:CP002684 GenomeReviews:CT485782_GR GO:GO:0031969 eggNOG:COG0477 InterPro:IPR016196 SUPFAM:SSF103473 GO:GO:0008643 InterPro:IPR020846 PROSITE:PS50850 HOGENOM:HBG744444 GO:GO:0022891 TIGRFAMs:TIGR00879 EMBL:AC002130 EMBL:AK226899 IPI:IPI00539133 PIR:F96696 RefSeq:NP_176898.1 UniGene:At.21607 UniGene:At.67017 ProteinModelPortal:Q9FYG3 EnsemblPlants:AT1G67300.1 GeneID:843050 KEGG:ath:AT1G67300 TAIR:At1g67300 InParanoid:Q9FYG3 PhylomeDB:Q9FYG3 ProtClustDB:CLSN2681973 Genevestigator:Q9FYG3 Uniprot:Q9FYG3
Root	Isotig02683	97	4	4.471	2.23E-22	TAIR|locus:2019554 - symbol:AT1G67300 "AT1G67300" species:3702 "Arabidopsis thaliana" [GO:0005351 "sugar:hydrogen symporter activity" evidence=ISS] [GO:0006810 "transport" evidence=IEA] [GO:0015144 "carbohydrate transmembrane transporter activity" evidence=ISS] [GO:0016020 "membrane" evidence=IEA;ISS] [GO:0016021 "integral to membrane" evidence=IEA] [GO:0055085 "transmembrane transport" evidence=IEA] InterPro:IPR003663 InterPro:IPR005828 InterPro:IPR005829 Pfam:PF00083 PRINTS:PR00171 PROSITE:PS00216 PROSITE:PS00217 GO:GO:0016021 EMBL:CP002684 GenomeReviews:CT485782_GR GO:GO:0031969 eggNOG:COG0477 InterPro:IPR016196 SUPFAM:SSF103473 GO:GO:0008643 InterPro:IPR020846 PROSITE:PS50850 HOGENOM:HBG744444 GO:GO:0022891 TIGRFAMs:TIGR00879 EMBL:AC002130 EMBL:AK226899 IPI:IPI00539133 PIR:F96696 RefSeq:NP_176898.1 UniGene:At.21607 UniGene:At.67017 ProteinModelPortal:Q9FYG3 EnsemblPlants:AT1G67300.1 GeneID:843050 KEGG:ath:AT1G67300 TAIR:At1g67300 InParanoid:Q9FYG3 PhylomeDB:Q9FYG3 ProtClustDB:CLSN2681973 Genevestigator:Q9FYG3 Uniprot:Q9FYG3
Root	Isotig02688	109	30	1.732	1.32E-10	SGD|S000028567 - symbol:RRT15 "Putative protein of unknown function" species:4932 "Saccharomyces cerevisiae" [GO:0006351 "transcription, DNA-dependent" evidence=IEA] [GO:0005575 "cellular_component" evidence=ND] [GO:0008150 "biological_process" evidence=ND] [GO:0006355 "regulation of transcription, DNA-dependent" evidence=IEA] [GO:0003674 "molecular_function" evidence=ND] SGD:S000028567 GO:GO:0006355 GO:GO:0006351 EMBL:BK006945 EMBL:U51921 RefSeq:NP_878128.1 ProteinModelPortal:Q3E811 STRING:Q3E811 EnsemblFungi:YLR162W-A GeneID:1466416 KEGG:sce:YLR162W-A CYGD:YLR162w-a

Tissue	Identification	# of reads (stress)	# of reads (control)	Fold Change	p-value	Description
						GeneTree:ENSGT00530000064745 OMA:FYINCIM OrthoDB:EOG45TGXP NextBio:983475 ArrayExpress:Q3E811 Genevestigator:Q3E811 Uniprot:Q3E811
Root	Isotig02689	109	30	1.732	1.32E-10	SGD|S000028567 - symbol:RRT15 "Putative protein of unknown function" species:4932 "Saccharomyces cerevisiae" [GO:0006351 "transcription, DNA-dependent" evidence=IEA] [GO:0005575 "cellular_component" evidence=ND] [GO:0008150 "biological_process" evidence=ND] [GO:0006355 "regulation of transcription, DNA-dependent" evidence=IEA] [GO:0003674 "molecular_function" evidence=ND] SGD:S000028567 GO:GO:0006355 GO:GO:0006351 EMBL:BK006945 EMBL:U51921 RefSeq:NP_878128.1 ProteinModelPortal:Q3E811 STRING:Q3E811 EnsemblFungi:YLR162W-A GeneID:1466416 KEGG:sce:YLR162W-A CYGD:YLR162w-a GeneTree:ENSGT00530000064745 OMA:FYINCIM OrthoDB:EOG45TGXP NextBio:983475 ArrayExpress:Q3E811 Genevestigator:Q3E811 Uniprot:Q3E811
Root	Isotig02690	109	30	1.732	1.32E-10	SGD|S000028567 - symbol:RRT15 "Putative protein of unknown function" species:4932 "Saccharomyces cerevisiae" [GO:0006351 "transcription, DNA-dependent" evidence=IEA] [GO:0005575 "cellular_component" evidence=ND] [GO:0008150 "biological_process" evidence=ND] [GO:0006355 "regulation of transcription, DNA-dependent" evidence=IEA] [GO:0003674 "molecular_function" evidence=ND] SGD:S000028567 GO:GO:0006355 GO:GO:0006351 EMBL:BK006945 EMBL:U51921 RefSeq:NP_878128.1 ProteinModelPortal:Q3E811 STRING:Q3E811 EnsemblFungi:YLR162W-A GeneID:1466416 KEGG:sce:YLR162W-A CYGD:YLR162w-a GeneTree:ENSGT00530000064745 OMA:FYINCIM OrthoDB:EOG45TGXP NextBio:983475 ArrayExpress:Q3E811 Genevestigator:Q3E811 Uniprot:Q3E811
Root	Isotig02705	6	29	-2.402	1.89E-05	TAIR|locus:2006742 - symbol:PAP3 "AT1G14700" species:3702 "Arabidopsis thaliana" [GO:0004722 "protein serine/threonine phosphatase activity" evidence=ISS] [GO:0005773 "vacuole" evidence=IDA] [GO:0003993 "acid phosphatase activity" evidence=ISS] InterPro:IPR004843 InterPro:IPR024927 Pfam:PF00149 PIRSF:PIRSF000898 EMBL:CP002684 GenomeReviews:CT485782_GR GO:GO:0005773 GO:GO:0005576 GO:GO:0046872 GO:GO:0003993 eggNOG:COG1409 EMBL:AC006917 KO:K01078 ProtClustDB:CLSN2688499 EMBL:AY686599 EMBL:AY070051 EMBL:BT000877 IPI:IPI00522072 IPI:IPI00846554 PIR:D86281 RefSeq:NP_001077538.1 RefSeq:NP_172923.3 UniGene:At.28419 ProteinModelPortal:Q8H129 SMR:Q8H129 STRING:Q8H129 EnsemblPlants:AT1G14700.1 GeneID:838035 KEGG:ath:AT1G14700 TAIR:At1g14700 HOGENOM:HBG560703 InParanoid:Q8H129 OMA:MRSFIVN PhylomeDB:Q8H129 Genevestigator:Q8H129 Uniprot:Q8H129
Root	Isotig02707	6	130	-4.566	2.60E-32	TAIR|locus:2126026 - symbol:PIP1;4 "AT4G00430" species:3702 "Arabidopsis thaliana" [GO:0006810 "transport" evidence=ISS] [GO:0015250 "water channel activity" evidence=ISS] [GO:0016020 "membrane" evidence=ISS;IDA] [GO:0005886 "plasma membrane" evidence=IDA] [GO:0009414 "response to water deprivation" evidence=IEP] InterPro:IPR000425 InterPro:IPR012269 InterPro:IPR022357 Pfam:PF00230 PRINTS:PR00783 PROSITE:PS00221 GO:GO:0016021 GO:GO:0005886 EMBL:CP002687 GenomeReviews:CT486007_GR GO:GO:0055085 GO:GO:0009414 GO:GO:0005215 GO:GO:0006833 EMBL:AF013293 EMBL:AF195115 EMBL:AL161471 InterPro:IPR023271 Gene3D:G3DSA:1.20.1080.10 SUPFAM:SSF81338 eggNOG:COG0580 HOGENOM:HBG705794 PANTHER:PTHR19139 TIGRFAMs:TIGR00861 OMA:ITHNFKD KO:K09872 ProtClustDB:CLSN2682869 UniGene:At.20518 UniGene:At.20892 EMBL:D26609 EMBL:D85192 EMBL:AY099825 EMBL:AY120785 EMBL:BT000330 EMBL:BT006313 IPI:IPI00521990 PIR:T01528 RefSeq:NP_567178.1 ProteinModelPortal:Q39196 SMR:Q39196 IntAct:Q39196 STRING:Q39196 PRIDE:Q39196 EnsemblPlants:AT4G00430.1 GeneID:827956 KEGG:ath:AT4G00430 GeneFarm:2265 TAIR:At4g00430 InParanoid:Q39196 PhylomeDB:Q39196 ArrayExpress:Q39196 Genevestigator:Q39196 Uniprot:Q39196
Root	Isotig02708	2	122	-6.060	7.53E-32	TAIR|locus:2126026 - symbol:PIP1;4 "AT4G00430" species:3702 "Arabidopsis thaliana" [GO:0006810 "transport" evidence=ISS] [GO:0015250 "water channel activity" evidence=ISS] [GO:0016020 "membrane" evidence=ISS;IDA] [GO:0005886 "plasma membrane" evidence=IDA] [GO:0009414 "response to water deprivation" evidence=IEP] InterPro:IPR000425 InterPro:IPR012269 InterPro:IPR022357 Pfam:PF00230 PRINTS:PR00783 PROSITE:PS00221 GO:GO:0016021 GO:GO:0005886 EMBL:CP002687 GenomeReviews:CT486007_GR GO:GO:0055085 GO:GO:0009414 GO:GO:0005215 GO:GO:0006833 EMBL:AF013293 EMBL:AF195115 EMBL:AL161471 InterPro:IPR023271 Gene3D:G3DSA:1.20.1080.10 SUPFAM:SSF81338 eggNOG:COG0580 HOGENOM:HBG705794 PANTHER:PTHR19139 TIGRFAMs:TIGR00861 OMA:ITHNFKD KO:K09872 ProtClustDB:CLSN2682869 UniGene:At.20518 UniGene:At.20892 EMBL:D26609 EMBL:D85192 EMBL:AY099825 EMBL:AY120785 EMBL:BT000330 EMBL:BT006313 IPI:IPI00521990 PIR:T01528 RefSeq:NP_567178.1 ProteinModelPortal:Q39196 SMR:Q39196 IntAct:Q39196 STRING:Q39196 PRIDE:Q39196

Tissue	Identification	# of reads (stress)	# of reads (control)	Fold Change	p-value	Description
						EnsemblPlants:AT4G00430.1 GeneID:827956 KEGG:ath:AT4G00430 GeneFarm:2265 TAIR:At4g00430 InParanoid:Q39196 PhylomeDB:Q39196 ArrayExpress:Q39196 Genevestigator:Q39196 Uniprot:Q39196
Root	Isotig02709	2	122	-6.060	7.53E-32	TAIR|locus:2126026 - symbol:PIP1;4 "AT4G00430" species:3702 "Arabidopsis thaliana" [GO:0006810 "transport" evidence=ISS] [GO:0015250 "water channel activity" evidence=ISS] [GO:0016020 "membrane" evidence=ISS;IDA] [GO:0005886 "plasma membrane" evidence=IDA] [GO:0009414 "response to water deprivation" evidence=IEP] InterPro:IPR000425 InterPro:IPR012269 InterPro:IPR022357 Pfam:PF00230 PRINTS:PR00783 PROSITE:PS00221 GO:GO:0016021 GO:GO:0005886 EMBL:CP002687 GenomeReviews:CT486007_GR GO:GO:0055085 GO:GO:0009414 GO:GO:0005215 GO:GO:0006833 EMBL:AF013293 EMBL:AF195115 EMBL:AL161471 InterPro:IPR023271 Gene3D:G3DSA:1.20.1080.10 SUPFAM:SSF81338 eggNOG:COG0580 HOGENOM:HBG705794 PANTHER:PTHR19139 TIGRFAMs:TIGR00861 OMA:ITHNFKD KO:K09872 ProtClustDB:CLSN2682869 UniGene:At.20518 UniGene:At.20892 EMBL:D26609 EMBL:D85192 EMBL:AY099825 EMBL:AY120785 EMBL:BT000330 EMBL:BT006313 IPI:IPI00521990 PIR:T01528 RefSeq:NP_567178.1 ProteinModelPortal:Q39196 SMR:Q39196 IntAct:Q39196 STRING:Q39196 PRIDE:Q39196 EnsemblPlants:AT4G00430.1 GeneID:827956 KEGG:ath:AT4G00430 GeneFarm:2265 TAIR:At4g00430 InParanoid:Q39196 PhylomeDB:Q39196 ArrayExpress:Q39196 Genevestigator:Q39196 Uniprot:Q39196
Root	Isotig02743	0	28	-5.936	1.79E-08	TAIR|locus:2030407 - symbol:AAE1 "acyl activating enzyme 1" species:3702 "Arabidopsis thaliana" [GO:0008152 "metabolic process" evidence=IEA] [GO:0016208 "AMP binding" evidence=ISS] [GO:0005777 "peroxisome" evidence=IDA] InterPro:IPR000873 Pfam:PF00501 Prosite:PS00455 EMBL:CP002684 GO:GO:0005777 InterPro:IPR020845 InterPro:IPR025110 Pfam:PF13193 EMBL:AC027665 GO:GO:0003824 EMBL:AC069251 EMBL:AF372942 EMBL:AY078039 EMBL:AF503760 IPI:IPI01019567 PIR:G86338 RefSeq:NP_564116.1 UniGene:At.20481 SMR:F4HUK6 PRIDE:Q9LMW3 EnsemblPlants:AT1G20560.1 GeneID:838644 KEGG:ath:AT1G20560 TAIR:At1g20560 Uniprot:F4HUK6
Root	Isotig02744	0	22	-5.588	6.01E-07	TAIR|locus:2057249 - symbol:AT2G17650 species:3702 "Arabidopsis thaliana" [GO:0003824 "catalytic activity" evidence=IEA] [GO:0008152 "metabolic process" evidence=IEA] InterPro:IPR000873 Pfam:PF00501 Prosite:PS00455 EMBL:CP002685 GenomeReviews:CT485783_GR eggNOG:COG0318 HOGENOM:HBG547964 InterPro:IPR020845 InterPro:IPR025110 Pfam:PF13193 HSSP:P08659 GO:GO:0003824 EMBL:AF503761 EMBL:AC007509 EMBL:AK118689 EMBL:BT005926 IPI:IPI00521948 PIR:G84554 PIR:T08866 RefSeq:NP_179356.1 UniGene:At.40116 ProteinModelPortal:Q9SEY5 SMR:Q9SEY5 PRIDE:Q9SEY5 EnsemblPlants:AT2G17650.1 GeneID:816272 KEGG:ath:AT2G17650 TAIR:At2g17650 InParanoid:Q9SEY5 OMA:LHPVHVY PhylomeDB:Q9SEY5 ProtClustDB:CLSN2913309 ArrayExpress:Q9SEY5 Genevestigator:Q9SEY5 Uniprot:Q9SEY5
Root	Isotig02745	0	22	-5.588	6.01E-07	TAIR|locus:2030407 - symbol:AAE1 "acyl activating enzyme 1" species:3702 "Arabidopsis thaliana" [GO:0008152 "metabolic process" evidence=IEA] [GO:0016208 "AMP binding" evidence=ISS] [GO:0005777 "peroxisome" evidence=IDA] InterPro:IPR000873 Pfam:PF00501 Prosite:PS00455 EMBL:CP002684 GO:GO:0005777 InterPro:IPR020845 InterPro:IPR025110 Pfam:PF13193 EMBL:AC027665 GO:GO:0003824 EMBL:AC069251 EMBL:AF372942 EMBL:AY078039 EMBL:AF503760 IPI:IPI01019567 PIR:G86338 RefSeq:NP_564116.1 UniGene:At.20481 SMR:F4HUK6 PRIDE:Q9LMW3 EnsemblPlants:AT1G20560.1 GeneID:838644 KEGG:ath:AT1G20560 TAIR:At1g20560 Uniprot:F4HUK6
Root	Isotig02759	2	73	-5.319	1.23E-19	TAIR|locus:2084031 - symbol:PIP2A "AT3G53420" species:3702 "Arabidopsis thaliana" [GO:0006810 "transport" evidence=ISS] [GO:0015250 "water channel activity" evidence=ISS;IDA] [GO:0016020 "membrane" evidence=ISS;IDA] [GO:0009507 "chloroplast" evidence=IDA] [GO:0005886 "plasma membrane" evidence=IDA;TAS] [GO:0005773 "vacuole" evidence=IDA] [GO:0009414 "response to water deprivation" evidence=IEP] [GO:0080170 "hydrogen peroxide transmembrane transport" evidence=IDA] [GO:0031625 "ubiquitin protein ligase binding" evidence=IPI] [GO:0009737 "response to abscisic acid stimulus" evidence=IDA] [GO:0009506 "plasmodesma" evidence=IDA] [GO:0006833 "water transport" evidence=IDA] InterPro:IPR000425 InterPro:IPR012269 InterPro:IPR022357 Pfam:PF00230 PRINTS:PR00783 PROSITE:PS00221 GO:GO:0016021 GO:GO:0005886 GO:GO:0009506 GO:GO:0009737 GO:GO:0005773 GO:GO:0009507 EMBL:CP002686 GenomeReviews:BA000014_GR EMBL:AL132966 GO:GO:0009414 GO:GO:0031625 InterPro:IPR023271 Gene3D:G3DSA:1.20.1080.10 SUPFAM:SSF81338 eggNOG:COG0580 HOGENOM:HBG705794 PANTHER:PTHR19139 TIGRFAMs:TIGR00861 GO:GO:0015250 GO:GO:0080170 KO:K09872 EMBL:X75883 EMBL:AY039579 EMBL:AY044327 EMBL:AY056085 EMBL:AF428426 EMBL:AY072374 EMBL:AY087854 IPI:IPI00532482 PIR:S44084 RefSeq:NP_001030851.1 RefSeq:NP_190910.1 UniGene:At.47609

Tissue	Identification	# of reads (stress)	# of reads (control)	Fold Change	p-value	Description
						ProteinModelPortal:P43286 SMR:P43286 MINT:MINT-6951259 STRING:P43286 TCDB:1.A.8.11.4 PRIDE:P43286 ProMEX:P43286 EnsemblPlants:AT3G53420.1 EnsemblPlants:AT3G53420.2 GeneID:824510 KEGG:ath:AT3G53420 GeneFarm:4866 TAIR:At3g53420 InParanoid:P43286 OMA:VFMAITI PhylomeDB:P43286 ProtClustDB:CLSN2683965 ArrayExpress:P43286 Genevestigator:P43286 GermOnline:AT3G53420 Uniprot:P43286
Root	Isotig02760	3	83	-4.919	9.50E-22	TAIR|locus:2084031 - symbol:PIP2A "AT3G53420" species:3702 "Arabidopsis thaliana" [GO:0006810 "transport" evidence=ISS] [GO:0015250 "water channel activity" evidence=ISS;IDA] [GO:0016020 "membrane" evidence=ISS;IDA] [GO:0009507 "chloroplast" evidence=IDA] [GO:0005886 "plasma membrane" evidence=IDA;TAS] [GO:0005773 "vacuole" evidence=IDA] [GO:0009414 "response to water deprivation" evidence=IEP] [GO:0080170 "hydrogen peroxide transmembrane transport" evidence=IDA] [GO:0031625 "ubiquitin protein ligase binding" evidence=IPI] [GO:0009737 "response to abscisic acid stimulus" evidence=IDA] [GO:0009506 "plasmodesma" evidence=IDA] [GO:0006833 "water transport" evidence=IDA] InterPro:IPR000425 InterPro:IPR012269 InterPro:IPR022357 Pfam:PF00230 PRINTS:PR00783 PROSITE:PS00221 GO:GO:0016021 GO:GO:0005886 GO:GO:0009506 GO:GO:0009737 GO:GO:0005773 GO:GO:0009507 EMBL:CP002686 GenomeReviews:BA000014_GR EMBL:AL132966 GO:GO:0009414 GO:GO:0031625 InterPro:IPR023271 Gene3D:G3DSA:1.20.1080.10 SUPFAM:SSF81338 eggNOG:COG0580 HOGENOM:HBG705794 PANTHER:PTHR19139 TIGRFAMs:TIGR00861 GO:GO:0015250 GO:GO:0080170 KO:K09872 EMBL:X75883 EMBL:AY039579 EMBL:AY044327 EMBL:AY056085 EMBL:AF428426 EMBL:AY072374 EMBL:AY087854 IPI:IPI00532482 PIR:S44084 RefSeq:NP_001030851.1 RefSeq:NP_190910.1 UniGene:At.47609 ProteinModelPortal:P43286 SMR:P43286 MINT:MINT-6951259 STRING:P43286 TCDB:1.A.8.11.4 PRIDE:P43286 ProMEX:P43286 EnsemblPlants:AT3G53420.1 EnsemblPlants:AT3G53420.2 GeneID:824510 KEGG:ath:AT3G53420 GeneFarm:4866 TAIR:At3g53420 InParanoid:P43286 OMA:VFMAITI PhylomeDB:P43286 ProtClustDB:CLSN2683965 ArrayExpress:P43286 Genevestigator:P43286 GermOnline:AT3G53420 Uniprot:P43286
Root	Isotig02761	2	46	-4.653	1.60E-12	TAIR|locus:2084031 - symbol:PIP2A "AT3G53420" species:3702 "Arabidopsis thaliana" [GO:0006810 "transport" evidence=ISS] [GO:0015250 "water channel activity" evidence=ISS;IDA] [GO:0016020 "membrane" evidence=ISS;IDA] [GO:0009507 "chloroplast" evidence=IDA] [GO:0005886 "plasma membrane" evidence=IDA;TAS] [GO:0005773 "vacuole" evidence=IDA] [GO:0009414 "response to water deprivation" evidence=IEP] [GO:0080170 "hydrogen peroxide transmembrane transport" evidence=IDA] [GO:0031625 "ubiquitin protein ligase binding" evidence=IPI] [GO:0009737 "response to abscisic acid stimulus" evidence=IDA] [GO:0009506 "plasmodesma" evidence=IDA] [GO:0006833 "water transport" evidence=IDA] InterPro:IPR000425 InterPro:IPR012269 InterPro:IPR022357 Pfam:PF00230 PRINTS:PR00783 PROSITE:PS00221 GO:GO:0016021 GO:GO:0005886 GO:GO:0009506 GO:GO:0009737 GO:GO:0005773 GO:GO:0009507 EMBL:CP002686 GenomeReviews:BA000014_GR EMBL:AL132966 GO:GO:0009414 GO:GO:0031625 InterPro:IPR023271 Gene3D:G3DSA:1.20.1080.10 SUPFAM:SSF81338 eggNOG:COG0580 HOGENOM:HBG705794 PANTHER:PTHR19139 TIGRFAMs:TIGR00861 GO:GO:0015250 GO:GO:0080170 KO:K09872 EMBL:X75883 EMBL:AY039579 EMBL:AY044327 EMBL:AY056085 EMBL:AF428426 EMBL:AY072374 EMBL:AY087854 IPI:IPI00532482 PIR:S44084 RefSeq:NP_001030851.1 RefSeq:NP_190910.1 UniGene:At.47609 ProteinModelPortal:P43286 SMR:P43286 MINT:MINT-6951259 STRING:P43286 TCDB:1.A.8.11.4 PRIDE:P43286 ProMEX:P43286 EnsemblPlants:AT3G53420.1 EnsemblPlants:AT3G53420.2 GeneID:824510 KEGG:ath:AT3G53420 GeneFarm:4866 TAIR:At3g53420 InParanoid:P43286 OMA:VFMAITI PhylomeDB:P43286 ProtClustDB:CLSN2683965 ArrayExpress:P43286 Genevestigator:P43286 GermOnline:AT3G53420 Uniprot:P43286
Root	Isotig02762	2	46	-4.653	1.60E-12	TAIR|locus:2084031 - symbol:PIP2A "AT3G53420" species:3702 "Arabidopsis thaliana" [GO:0006810 "transport" evidence=ISS] [GO:0015250 "water channel activity" evidence=ISS;IDA] [GO:0016020 "membrane" evidence=ISS;IDA] [GO:0009507 "chloroplast" evidence=IDA] [GO:0005886 "plasma membrane" evidence=IDA;TAS] [GO:0005773 "vacuole" evidence=IDA] [GO:0009414 "response to water deprivation" evidence=IEP] [GO:0080170 "hydrogen peroxide transmembrane transport" evidence=IDA] [GO:0031625 "ubiquitin protein ligase binding" evidence=IPI] [GO:0009737 "response to abscisic acid stimulus" evidence=IDA] [GO:0009506 "plasmodesma" evidence=IDA] [GO:0006833 "water transport" evidence=IDA] InterPro:IPR000425 InterPro:IPR012269 InterPro:IPR022357 Pfam:PF00230 PRINTS:PR00783 PROSITE:PS00221 GO:GO:0016021 GO:GO:0005886 GO:GO:0009506 GO:GO:0009737 GO:GO:0005773 GO:GO:0009507 EMBL:CP002686

Tissue	Identification	# of reads (stress)	# of reads (control)	Fold Change	p-value	Description
						GenomeReviews:BA000014_GR EMBL:AL132966 GO:GO:0009414 GO:GO:0031625 InterPro:IPR023271 Gene3D:G3DSA:1.20.1080.10 SUPFAM:SSF81338 eggNOG:COG0580 HOGENOM:HBG705794 PANTHER:PTHR19139 TIGRFAMs:TIGR00861 GO:GO:0015250 GO:GO:0080170 KO:K09872 EMBL:X75883 EMBL:AY039579 EMBL:AY044327 EMBL:AY056085 EMBL:AF428426 EMBL:AY072374 EMBL:AY087854 IPI:IPI00532482 PIR:S44084 RefSeq:NP_001030851.1 RefSeq:NP_190910.1 UniGene:At.47609 ProteinModelPortal:P43286 SMR:P43286 MINT:MINT-6951259 STRING:P43286 TCDB:1.A.8.11.4 PRIDE:P43286 ProMEX:P43286 EnsemblPlants:AT3G53420.1 EnsemblPlants:AT3G53420.2 GeneID:824510 KEGG:ath:AT3G53420 GeneFarm:4866 TAIR:At3g53420 InParanoid:P43286 OMA:VFMAITI PhylomeDB:P43286 ProtClustDB:CLSN2683965 ArrayExpress:P43286 Genevestigator:P43286 GermOnline:AT3G53420 Uniprot:P43286
Root	Isotig02765	18	1	4.041	4.47E-05	No hit
Root	Isotig02766	12	0	4.456	0.000626491	No hit
Root	Isotig02779	191	61	1.518	1.05E-14	TAIR|locus:2020088 - symbol:AT1G54290 species:3702 "Arabidopsis thaliana" [GO:0003743 "translation initiation factor activity" evidence=IEA;ISS] [GO:0006412 "translation" evidence=IEA] [GO:0006413 "translational initiation" evidence=IEA;ISS] InterPro:IPR001950 InterPro:IPR005874 Pfam:PF01253 PIRSF:PIRSF004499 PROSITE:PS50296 EMBL:CP002684 GenomeReviews:CT485782_GR GO:GO:0006417 EMBL:AC005287 GO:GO:0003743 eggNOG:COG0023 SUPFAM:SSF55159 HOGENOM:HBG522548 KO:K03113 OMA:QNLKTFD TIGRFAMs:TIGR01160 ProtClustDB:CLSN2682406 EMBL:AF372910 EMBL:BT000649 IPI:IPI00530446 PIR:D96584 RefSeq:NP_175831.1 UniGene:At.20055 ProteinModelPortal:Q94JV4 SMR:Q94JV4 IntAct:Q94JV4 STRING:Q94JV4 PRIDE:Q94JV4 EnsemblPlants:AT1G54290.1 GeneID:841870 KEGG:ath:AT1G54290 TAIR:At1g54290 InParanoid:Q94JV4 PhylomeDB:Q94JV4 ArrayExpress:Q94JV4 Genevestigator:Q94JV4 GermOnline:AT1G54290 Uniprot:Q94JV4
Root	Isotig02780	191	60	1.542	5.18E-15	TAIR|locus:2020088 - symbol:AT1G54290 species:3702 "Arabidopsis thaliana" [GO:0003743 "translation initiation factor activity" evidence=IEA;ISS] [GO:0006412 "translation" evidence=IEA] [GO:0006413 "translational initiation" evidence=IEA;ISS] InterPro:IPR001950 InterPro:IPR005874 Pfam:PF01253 PIRSF:PIRSF004499 PROSITE:PS50296 EMBL:CP002684 GenomeReviews:CT485782_GR GO:GO:0006417 EMBL:AC005287 GO:GO:0003743 eggNOG:COG0023 SUPFAM:SSF55159 HOGENOM:HBG522548 KO:K03113 OMA:QNLKTFD TIGRFAMs:TIGR01160 ProtClustDB:CLSN2682406 EMBL:AF372910 EMBL:BT000649 IPI:IPI00530446 PIR:D96584 RefSeq:NP_175831.1 UniGene:At.20055 ProteinModelPortal:Q94JV4 SMR:Q94JV4 IntAct:Q94JV4 STRING:Q94JV4 PRIDE:Q94JV4 EnsemblPlants:AT1G54290.1 GeneID:841870 KEGG:ath:AT1G54290 TAIR:At1g54290 InParanoid:Q94JV4 PhylomeDB:Q94JV4 ArrayExpress:Q94JV4 Genevestigator:Q94JV4 GermOnline:AT1G54290 Uniprot:Q94JV4
Root	Isotig02781	193	60	1.557	2.39E-15	TAIR|locus:2020088 - symbol:AT1G54290 species:3702 "Arabidopsis thaliana" [GO:0003743 "translation initiation factor activity" evidence=IEA;ISS] [GO:0006412 "translation" evidence=IEA] [GO:0006413 "translational initiation" evidence=IEA;ISS] InterPro:IPR001950 InterPro:IPR005874 Pfam:PF01253 PIRSF:PIRSF004499 PROSITE:PS50296 EMBL:CP002684 GenomeReviews:CT485782_GR GO:GO:0006417 EMBL:AC005287 GO:GO:0003743 eggNOG:COG0023 SUPFAM:SSF55159 HOGENOM:HBG522548 KO:K03113 OMA:QNLKTFD TIGRFAMs:TIGR01160 ProtClustDB:CLSN2682406 EMBL:AF372910 EMBL:BT000649 IPI:IPI00530446 PIR:D96584 RefSeq:NP_175831.1 UniGene:At.20055 ProteinModelPortal:Q94JV4 SMR:Q94JV4 IntAct:Q94JV4 STRING:Q94JV4 PRIDE:Q94JV4 EnsemblPlants:AT1G54290.1 GeneID:841870 KEGG:ath:AT1G54290 TAIR:At1g54290 InParanoid:Q94JV4 PhylomeDB:Q94JV4 ArrayExpress:Q94JV4 Genevestigator:Q94JV4 GermOnline:AT1G54290 Uniprot:Q94JV4
Root	Isotig02782	193	59	1.581	1.16E-15	TAIR|locus:2020088 - symbol:AT1G54290 species:3702 "Arabidopsis thaliana" [GO:0003743 "translation initiation factor activity" evidence=IEA;ISS] [GO:0006412 "translation" evidence=IEA] [GO:0006413 "translational initiation" evidence=IEA;ISS] InterPro:IPR001950 InterPro:IPR005874 Pfam:PF01253 PIRSF:PIRSF004499 PROSITE:PS50296 EMBL:CP002684 GenomeReviews:CT485782_GR GO:GO:0006417 EMBL:AC005287 GO:GO:0003743 eggNOG:COG0023 SUPFAM:SSF55159 HOGENOM:HBG522548 KO:K03113 OMA:QNLKTFD TIGRFAMs:TIGR01160 ProtClustDB:CLSN2682406 EMBL:AF372910 EMBL:BT000649 IPI:IPI00530446 PIR:D96584 RefSeq:NP_175831.1 UniGene:At.20055 ProteinModelPortal:Q94JV4 SMR:Q94JV4 IntAct:Q94JV4 STRING:Q94JV4 PRIDE:Q94JV4 EnsemblPlants:AT1G54290.1 GeneID:841870 KEGG:ath:AT1G54290 TAIR:At1g54290 InParanoid:Q94JV4 PhylomeDB:Q94JV4 ArrayExpress:Q94JV4 Genevestigator:Q94JV4 GermOnline:AT1G54290 Uniprot:Q94JV4

Tissue	Identification	# of reads (stress)	# of reads (control)	Fold Change	p-value	Description
Root	Isotig02803	5	21	-2.199	0.000555521	TAIR|locus:2046673 - symbol:AT2G24550 "AT2G24550" species:3702 "Arabidopsis thaliana" [GO:0003674 "molecular_function" evidence=ND] [GO:0005575 "cellular_component" evidence=ND] [GO:0008150 "biological_process" evidence=ND] EMBL:CP002685 GenomeReviews:CT485783_GR EMBL:AF411794 EMBL:AY099874 EMBL:BT000317 IPI:IPI00944291 RefSeq:NP_565573.4 UniGene:At.22389 IntAct:Q945P6 PRIDE:Q945P6 EnsemblPlants:AT2G24550.1 GeneID:816991 KEGG:ath:AT2G24550 TAIR:At2g24550 eggNOG:NOG249819 PhylomeDB:Q945P6 ProtClustDB:CLSN2685860 Genevestigator:Q945P6 Uniprot:Q945P6
Root	Isotig02804	5	21	-2.199	0.000555521	TAIR|locus:2046673 - symbol:AT2G24550 "AT2G24550" species:3702 "Arabidopsis thaliana" [GO:0003674 "molecular_function" evidence=ND] [GO:0005575 "cellular_component" evidence=ND] [GO:0008150 "biological_process" evidence=ND] EMBL:CP002685 GenomeReviews:CT485783_GR EMBL:AF411794 EMBL:AY099874 EMBL:BT000317 IPI:IPI00944291 RefSeq:NP_565573.4 UniGene:At.22389 IntAct:Q945P6 PRIDE:Q945P6 EnsemblPlants:AT2G24550.1 GeneID:816991 KEGG:ath:AT2G24550 TAIR:At2g24550 eggNOG:NOG249819 PhylomeDB:Q945P6 ProtClustDB:CLSN2685860 Genevestigator:Q945P6 Uniprot:Q945P6
Root	Isotig02805	2	14	-2.936	0.000925454	TAIR|locus:2046673 - symbol:AT2G24550 "AT2G24550" species:3702 "Arabidopsis thaliana" [GO:0003674 "molecular_function" evidence=ND] [GO:0005575 "cellular_component" evidence=ND] [GO:0008150 "biological_process" evidence=ND] EMBL:CP002685 GenomeReviews:CT485783_GR EMBL:AF411794 EMBL:AY099874 EMBL:BT000317 IPI:IPI00944291 RefSeq:NP_565573.4 UniGene:At.22389 IntAct:Q945P6 PRIDE:Q945P6 EnsemblPlants:AT2G24550.1 GeneID:816991 KEGG:ath:AT2G24550 TAIR:At2g24550 eggNOG:NOG249819 PhylomeDB:Q945P6 ProtClustDB:CLSN2685860 Genevestigator:Q945P6 Uniprot:Q945P6
Root	Isotig02806	4	22	-2.588	0.000101225	TAIR|locus:2046673 - symbol:AT2G24550 "AT2G24550" species:3702 "Arabidopsis thaliana" [GO:0003674 "molecular_function" evidence=ND] [GO:0005575 "cellular_component" evidence=ND] [GO:0008150 "biological_process" evidence=ND] EMBL:CP002685 GenomeReviews:CT485783_GR EMBL:AF411794 EMBL:AY099874 EMBL:BT000317 IPI:IPI00944291 RefSeq:NP_565573.4 UniGene:At.22389 IntAct:Q945P6 PRIDE:Q945P6 EnsemblPlants:AT2G24550.1 GeneID:816991 KEGG:ath:AT2G24550 TAIR:At2g24550 eggNOG:NOG249819 PhylomeDB:Q945P6 ProtClustDB:CLSN2685860 Genevestigator:Q945P6 Uniprot:Q945P6
Root	Isotig02871	50	19	1.267	0.000561936	TAIR|locus:2017854 - symbol:NADP-ME4 "AT1G79750" species:3702 "Arabidopsis thaliana" [GO:0004470 "malic enzyme activity" evidence=ISS] [GO:0006108 "malate metabolic process" evidence=ISS;IDA;TAS] [GO:0016652 "oxidoreductase activity, acting on NADH or NADPH, NAD or NADP as acceptor" evidence=ISS] [GO:0004473 "malate dehydrogenase (oxaloacetate-decarboxylating) (NADP+) activity" evidence=IDA] [GO:0006633 "fatty acid biosynthetic process" evidence=TAS] [GO:0009507 "chloroplast" evidence=IDA] [GO:0042803 "protein homodimerization activity" evidence=IDA] [GO:0051289 "protein homotetramerization" evidence=IDA] [GO:0008270 "zinc ion binding" evidence=IDA] [GO:0050897 "cobalt ion binding" evidence=IDA] InterPro:IPR001891 InterPro:IPR012301 InterPro:IPR012302 InterPro:IPR015884 InterPro:IPR016040 Pfam:PF00390 Pfam:PF03949 PIRSF:PIRSF000106 PRINTS:PR00072 PROSITE:PS00331 SMART:SM00919 EMBL:CP002684 GO:GO:0009507 GO:GO:0042803 Gene3D:G3DSA:3.40.50.720 GO:GO:0051287 GO:GO:0008270 GO:GO:0006633 GO:GO:0051289 GO:GO:0050897 EMBL:AC011717 GO:GO:0006108 GO:GO:0016619 Gene3D:G3DSA:3.40.50.10380 KO:K00029 GO:GO:0004473 OMA:IQTKVDQ UniGene:At.21634 HSSP:P40927 ProtClustDB:PLN03129 EMBL:AY060584 EMBL:AY142064 IPI:IPI00535518 PIR:E96828 RefSeq:NP_178093.1 ProteinModelPortal:Q9CA83 SMR:Q9CA83 IntAct:Q9CA83 STRING:Q9CA83 PRIDE:Q9CA83 EnsemblPlants:AT1G79750.1 GeneID:844314 KEGG:ath:AT1G79750 TAIR:At1g79750 InParanoid:Q9CA83 PhylomeDB:Q9CA83 Genevestigator:Q9CA83 Uniprot:Q9CA83
Root	Isotig02872	32	60	-1.036	0.000772006	UNIPROTKB|B9RYP6 - symbol:RCOM_1312280 "Heat shock protein, putative" species:3988 "Ricinus communis" [GO:0005783 "endoplasmic reticulum" evidence=IDA] InterPro:IPR001023 PRINTS:PR00301 Pfam:PF00012 Prosite:PS00297 Prosite:PS00329 Prosite:PS01036 GO:GO:0005783 GO:GO:0005524 GO:GO:0006950 KO:K09490 InterPro:IPR018181 InterPro:IPR013126 PANTHER:PTHR19375 ProtClustDB:CLSN2713985 GO:GO:0032440 EMBL:EQ973832 RefSeq:XP_002518865.1 ProteinModelPortal:B9RYP6 GeneID:8286262 KEGG:rcu:RCOM_1312280 PhylomeDB:B9RYP6 Uniprot:B9RYP6
Root	Isotig02893	40	10	1.871	4.07E-05	TAIR|locus:2164855 - symbol:AT5G39570 "AT5G39570" species:3702 "Arabidopsis thaliana" [GO:0003674 "molecular_function" evidence=ND] [GO:0008150 "biological_process" evidence=ND] [GO:0005634 "nucleus" evidence=IDA] [GO:0005829 "cytosol" evidence=IDA] GO:GO:0005829 GO:GO:0005634 EMBL:CP002688 GenomeReviews:BA000015_GR EMBL:AB012243 EMBL:AF083682 EMBL:BT000496 EMBL:AY058080 IPI:IPI00534812 RefSeq:NP_568565.2 UniGene:At.20492 EnsemblPlants:AT5G39570.1 GeneID:833953 KEGG:ath:AT5G39570 TAIR:At5g39570 PhylomeDB:Q9FKA5 Genevestigator:Q9FKA5 Uniprot:Q9FKA5

Tissue	Identification	# of reads (stress)	# of reads (control)	Fold Change	p-value	Description
Root	Isotig02895	36	7	2.234	1.28E-05	TAIR|locus:2164855 - symbol:AT5G39570 "AT5G39570" species:3702 "Arabidopsis thaliana" [GO:0003674 "molecular_function" evidence=ND] [GO:0008150 "biological_process" evidence=ND] [GO:0005634 "nucleus" evidence=IDA] [GO:0005829 "cytosol" evidence=IDA] GO:GO:0005829 GO:GO:0005634 EMBL:CP002688 GenomeReviews:BA000015_GR EMBL:AB012243 EMBL:AF083682 EMBL:BT000496 EMBL:AY058080 IPI:IPI00534812 RefSeq:NP_568565.2 UniGene:At.20492 EnsemblPlants:AT5G39570.1 GeneID:833953 KEGG:ath:AT5G39570 TAIR:At5g39570 PhylomeDB:Q9FKA5 Genevestigator:Q9FKA5 Uniprot:Q9FKA5
Root	Isotig02914	23	55	-1.387	5.00E-05	TAIR|locus:2141385 - symbol:SAM-2 "AT4G01850" species:3702 "Arabidopsis thaliana" [GO:0004478 "methionine adenosyltransferase activity" evidence=ISS] [GO:0006556 "S-adenosylmethionine biosynthetic process" evidence=ISS] [GO:0005730 "nucleolus" evidence=IDA] [GO:0005618 "cell wall" evidence=IDA] [GO:0005507 "copper ion binding" evidence=IDA] [GO:0071281 "cellular response to iron ion" evidence=IEP] [GO:0005829 "cytosol" evidence=IDA;RCA] [GO:0070062 "extracellular vesicular exosome" evidence=IDA] [GO:0009506 "plasmodesma" evidence=IDA] InterPro:IPR002133 InterPro:IPR022628 InterPro:IPR022629 InterPro:IPR022630 InterPro:IPR022631 InterPro:IPR022636 Pfam:PF00438 Pfam:PF02772 Pfam:PF02773 PIRSF:PIRSF000497 PROSITE:PS00376 PROSITE:PS00377 GO:GO:0005829 GO:GO:0009506 GO:GO:0005524 EMBL:CP002687 GenomeReviews:CT486007_GR GO:GO:0005618 GO:GO:0071281 GO:GO:0005730 GO:GO:0005507 EMBL:AC007138 EMBL:AL161493 GO:GO:0070062 GO:GO:0006730 KO:K00789 GO:GO:0006556 UniGene:At.24731 eggNOG:COG0192 HOGENOM:HBG443662 ProtClustDB:PLN02243 GO:GO:0004478 PANTHER:PTHR11964 SUPFAM:SSF55973 TIGRFAMs:TIGR01034 EMBL:M33217 EMBL:AY072327 EMBL:AY094454 EMBL:BT000575 EMBL:Z33778 EMBL:Z29136 IPI:IPI00529348 PIR:JQ0410 RefSeq:NP_001078345.1 RefSeq:NP_192094.1 UniGene:At.24433 UniGene:At.71920 ProteinModelPortal:P17562 SMR:P17562 STRING:P17562 PRIDE:P17562 ProMEX:P17562 EnsemblPlants:AT4G01850.1 EnsemblPlants:AT4G01850.2 GeneID:826987 KEGG:ath:AT4G01850 TAIR:At4g01850 InParanoid:P17562 OMA:ATIDYEK PhylomeDB:P17562 ArrayExpress:P17562 Genevestigator:P17562 GermOnline:AT4G01850 Uniprot:P17562
Root	Isotig02915	23	53	-1.333	0.000111751	TAIR|locus:2141385 - symbol:SAM-2 "AT4G01850" species:3702 "Arabidopsis thaliana" [GO:0004478 "methionine adenosyltransferase activity" evidence=ISS] [GO:0006556 "S-adenosylmethionine biosynthetic process" evidence=ISS] [GO:0005730 "nucleolus" evidence=IDA] [GO:0005618 "cell wall" evidence=IDA] [GO:0005507 "copper ion binding" evidence=IDA] [GO:0071281 "cellular response to iron ion" evidence=IEP] [GO:0005829 "cytosol" evidence=IDA;RCA] [GO:0070062 "extracellular vesicular exosome" evidence=IDA] [GO:0009506 "plasmodesma" evidence=IDA] InterPro:IPR002133 InterPro:IPR022628 InterPro:IPR022629 InterPro:IPR022630 InterPro:IPR022631 InterPro:IPR022636 Pfam:PF00438 Pfam:PF02772 Pfam:PF02773 PIRSF:PIRSF000497 PROSITE:PS00376 PROSITE:PS00377 GO:GO:0005829 GO:GO:0009506 GO:GO:0005524 EMBL:CP002687 GenomeReviews:CT486007_GR GO:GO:0005618 GO:GO:0071281 GO:GO:0005730 GO:GO:0005507 EMBL:AC007138 EMBL:AL161493 GO:GO:0070062 GO:GO:0006730 KO:K00789 GO:GO:0006556 UniGene:At.24731 eggNOG:COG0192 HOGENOM:HBG443662 ProtClustDB:PLN02243 GO:GO:0004478 PANTHER:PTHR11964 SUPFAM:SSF55973 TIGRFAMs:TIGR01034 EMBL:M33217 EMBL:AY072327 EMBL:AY094454 EMBL:BT000575 EMBL:Z33778 EMBL:Z29136 IPI:IPI00529348 PIR:JQ0410 RefSeq:NP_001078345.1 RefSeq:NP_192094.1 UniGene:At.24433 UniGene:At.71920 ProteinModelPortal:P17562 SMR:P17562 STRING:P17562 PRIDE:P17562 ProMEX:P17562 EnsemblPlants:AT4G01850.1 EnsemblPlants:AT4G01850.2 GeneID:826987 KEGG:ath:AT4G01850 TAIR:At4g01850 InParanoid:P17562 OMA:ATIDYEK PhylomeDB:P17562 ArrayExpress:P17562 Genevestigator:P17562 GermOnline:AT4G01850 Uniprot:P17562
Root	Isotig02916	23	54	-1.360	7.49E-05	TAIR|locus:2141385 - symbol:SAM-2 "AT4G01850" species:3702 "Arabidopsis thaliana" [GO:0004478 "methionine adenosyltransferase activity" evidence=ISS] [GO:0006556 "S-adenosylmethionine biosynthetic process" evidence=ISS] [GO:0005730 "nucleolus" evidence=IDA] [GO:0005618 "cell wall" evidence=IDA] [GO:0005507 "copper ion binding" evidence=IDA] [GO:0071281 "cellular response to iron ion" evidence=IEP] [GO:0005829 "cytosol" evidence=IDA;RCA] [GO:0070062 "extracellular vesicular exosome" evidence=IDA] [GO:0009506 "plasmodesma" evidence=IDA] InterPro:IPR002133 InterPro:IPR022628 InterPro:IPR022629 InterPro:IPR022630 InterPro:IPR022631 InterPro:IPR022636 Pfam:PF00438 Pfam:PF02772 Pfam:PF02773 PIRSF:PIRSF000497 PROSITE:PS00376 PROSITE:PS00377 GO:GO:0005829 GO:GO:0009506 GO:GO:0005524 EMBL:CP002687 GenomeReviews:CT486007_GR GO:GO:0005618 GO:GO:0071281 GO:GO:0005730 GO:GO:0005507 EMBL:AC007138 EMBL:AL161493 GO:GO:0070062 GO:GO:0006730 KO:K00789 GO:GO:0006556

Tissue	Identification	# of reads (stress)	# of reads (control)	Fold Change	p-value	Description
						UniGene:At.24731 eggNOG:COG0192 HOGENOM:HBG443662 ProtClustDB:PLN02243 GO:GO:0004478 PANTHER:PTHR11964 SUPFAM:SSF55973 TIGRFAMs:TIGR01034 EMBL:M33217 EMBL:AY072327 EMBL:AY094454 EMBL:BT000575 EMBL:Z33778 EMBL:Z29136 IPI:IPI00529348 PIR:JQ0410 RefSeq:NP_001078345.1 RefSeq:NP_192094.1 UniGene:At.24433 UniGene:At.71920 ProteinModelPortal:P17562 SMR:P17562 STRING:P17562 PRIDE:P17562 ProMEX:P17562 EnsemblPlants:AT4G01850.1 EnsemblPlants:AT4G01850.2 GeneID:826987 KEGG:ath:AT4G01850 TAIR:At4g01850 InParanoid:P17562 OMA:ATIDYEK PhylomeDB:P17562 ArrayExpress:P17562 Genevestigator:P17562 GermOnline:AT4G01850 Uniprot:P17562
Root	Isotig02917	23	55	-1.387	5.00E-05	TAIR|locus:2141385 - symbol:SAM-2 "AT4G01850" species:3702 "Arabidopsis thaliana" [GO:0004478 "methionine adenosyltransferase activity" evidence=ISS] [GO:0006556 "S-adenosylmethionine biosynthetic process" evidence=ISS] [GO:0005730 "nucleolus" evidence=IDA] [GO:0005618 "cell wall" evidence=IDA] [GO:0005507 "copper ion binding" evidence=IDA] [GO:0071281 "cellular response to iron ion" evidence=IEP] [GO:0005829 "cytosol" evidence=IDA;RCA] [GO:0070062 "extracellular vesicular exosome" evidence=IDA] [GO:0009506 "plasmodesma" evidence=IDA] InterPro:IPR002133 InterPro:IPR022628 InterPro:IPR022629 InterPro:IPR022630 InterPro:IPR022631 InterPro:IPR022636 Pfam:PF00438 Pfam:PF02772 Pfam:PF02773 PIRSF:PIRSF000497 PROSITE:PS00376 PROSITE:PS00377 GO:GO:0005829 GO:GO:0009506 GO:GO:0005524 EMBL:CP002687 GenomeReviews:CT486007_GR GO:GO:0005618 GO:GO:0071281 GO:GO:0005730 GO:GO:0005507 EMBL:AC007138 EMBL:AL161493 GO:GO:0070062 GO:GO:0006730 KO:K00789 GO:GO:0006556 UniGene:At.24731 eggNOG:COG0192 HOGENOM:HBG443662 ProtClustDB:PLN02243 GO:GO:0004478 PANTHER:PTHR11964 SUPFAM:SSF55973 TIGRFAMs:TIGR01034 EMBL:M33217 EMBL:AY072327 EMBL:AY094454 EMBL:BT000575 EMBL:Z33778 EMBL:Z29136 IPI:IPI00529348 PIR:JQ0410 RefSeq:NP_001078345.1 RefSeq:NP_192094.1 UniGene:At.24433 UniGene:At.71920 ProteinModelPortal:P17562 SMR:P17562 STRING:P17562 PRIDE:P17562 ProMEX:P17562 EnsemblPlants:AT4G01850.1 EnsemblPlants:AT4G01850.2 GeneID:826987 KEGG:ath:AT4G01850 TAIR:At4g01850 InParanoid:P17562 OMA:ATIDYEK PhylomeDB:P17562 ArrayExpress:P17562 Genevestigator:P17562 GermOnline:AT4G01850 Uniprot:P17562
Root	Isotig02918	23	52	-1.306	0.000165922	TAIR|locus:2141385 - symbol:SAM-2 "AT4G01850" species:3702 "Arabidopsis thaliana" [GO:0004478 "methionine adenosyltransferase activity" evidence=ISS] [GO:0006556 "S-adenosylmethionine biosynthetic process" evidence=ISS] [GO:0005730 "nucleolus" evidence=IDA] [GO:0005618 "cell wall" evidence=IDA] [GO:0005507 "copper ion binding" evidence=IDA] [GO:0071281 "cellular response to iron ion" evidence=IEP] [GO:0005829 "cytosol" evidence=IDA;RCA] [GO:0070062 "extracellular vesicular exosome" evidence=IDA] [GO:0009506 "plasmodesma" evidence=IDA] InterPro:IPR002133 InterPro:IPR022628 InterPro:IPR022629 InterPro:IPR022630 InterPro:IPR022631 InterPro:IPR022636 Pfam:PF00438 Pfam:PF02772 Pfam:PF02773 PIRSF:PIRSF000497 PROSITE:PS00376 PROSITE:PS00377 GO:GO:0005829 GO:GO:0009506 GO:GO:0005524 EMBL:CP002687 GenomeReviews:CT486007_GR GO:GO:0005618 GO:GO:0071281 GO:GO:0005730 GO:GO:0005507 EMBL:AC007138 EMBL:AL161493 GO:GO:0070062 GO:GO:0006730 KO:K00789 GO:GO:0006556 UniGene:At.24731 eggNOG:COG0192 HOGENOM:HBG443662 ProtClustDB:PLN02243 GO:GO:0004478 PANTHER:PTHR11964 SUPFAM:SSF55973 TIGRFAMs:TIGR01034 EMBL:M33217 EMBL:AY072327 EMBL:AY094454 EMBL:BT000575 EMBL:Z33778 EMBL:Z29136 IPI:IPI00529348 PIR:JQ0410 RefSeq:NP_001078345.1 RefSeq:NP_192094.1 UniGene:At.24433 UniGene:At.71920 ProteinModelPortal:P17562 SMR:P17562 STRING:P17562 PRIDE:P17562 ProMEX:P17562 EnsemblPlants:AT4G01850.1 EnsemblPlants:AT4G01850.2 GeneID:826987 KEGG:ath:AT4G01850 TAIR:At4g01850 InParanoid:P17562 OMA:ATIDYEK PhylomeDB:P17562 ArrayExpress:P17562 Genevestigator:P17562 GermOnline:AT4G01850 Uniprot:P17562
Root	Isotig02919	23	53	-1.333	0.000111751	TAIR|locus:2141385 - symbol:SAM-2 "AT4G01850" species:3702 "Arabidopsis thaliana" [GO:0004478 "methionine adenosyltransferase activity" evidence=ISS] [GO:0006556 "S-adenosylmethionine biosynthetic process" evidence=ISS] [GO:0005730 "nucleolus" evidence=IDA] [GO:0005618 "cell wall" evidence=IDA] [GO:0005507 "copper ion binding" evidence=IDA] [GO:0071281 "cellular response to iron ion" evidence=IEP] [GO:0005829 "cytosol" evidence=IDA;RCA] [GO:0070062 "extracellular vesicular exosome" evidence=IDA] [GO:0009506 "plasmodesma" evidence=IDA] InterPro:IPR002133 InterPro:IPR022628 InterPro:IPR022629 InterPro:IPR022630 InterPro:IPR022631 InterPro:IPR022636 Pfam:PF00438 Pfam:PF02772 Pfam:PF02773 PIRSF:PIRSF000497 PROSITE:PS00376 PROSITE:PS00377 GO:GO:0005829 GO:GO:0009506 GO:GO:0005524 EMBL:CP002687

Tissue	Identification	# of reads (stress)	# of reads (control)	Fold Change	p-value	Description
						GenomeReviews:CT486007_GR GO:GO:0005618 GO:GO:0071281 GO:GO:0005730 GO:GO:0005507 EMBL:AC007138 EMBL:AL161493 GO:GO:0070062 GO:GO:0006730 KO:K00789 GO:GO:0006556 UniGene:At.24731 eggNOG:COG0192 HOGENOM:HBG443662 ProtClustDB:PLN02243 GO:GO:0004478 PANTHER:PTHR11964 SUPFAM:SSF55973 TIGRFAMs:TIGR01034 EMBL:M33217 EMBL:AY072327 EMBL:AY094454 EMBL:BT000575 EMBL:Z33778 EMBL:Z29136 IPI:IPI00529348 PIR:JQ0410 RefSeq:NP_001078345.1 RefSeq:NP_192094.1 UniGene:At.24433 UniGene:At.71920 ProteinModelPortal:P17562 SMR:P17562 STRING:P17562 PRIDE:P17562 ProMEX:P17562 EnsemblPlants:AT4G01850.1 EnsemblPlants:AT4G01850.2 GeneID:826987 KEGG:ath:AT4G01850 TAIR:At4g01850 InParanoid:P17562 OMA:ATIDYEK PhylomeDB:P17562 ArrayExpress:P17562 Genevestigator:P17562 GermOnline:AT4G01850 Uniprot:P17562
Root	Isotig02920	3	57	-4.377	8.39E-15	TAIR|locus:2064895 - symbol:PHT1;4 "AT2G38940" species:3702 "Arabidopsis thaliana" [GO:0005351 "sugar:hydrogen symporter activity" evidence=ISS] [GO:0015114 "phosphate ion transmembrane transporter activity" evidence=ISS] [GO:0015144 "carbohydrate transmembrane transporter activity" evidence=ISS] [GO:0016020 "membrane" evidence=ISS;IDA] [GO:0005886 "plasma membrane" evidence=IDA] [GO:0005773 "vacuole" evidence=IDA] [GO:0005634 "nucleus" evidence=IDA] [GO:0009737 "response to abscisic acid stimulus" evidence=IDA] [GO:0009506 "plasmodesma" evidence=IDA] [GO:0005794 "Golgi apparatus" evidence=IDA] [GO:0005315 "inorganic phosphate transmembrane transporter activity" evidence=ISS] InterPro:IPR004738 InterPro:IPR005828 Pfam:PF00083 PROSITE:PS00216 PROSITE:PS00217 GO:GO:0016021 GO:GO:0005886 GO:GO:0009506 GO:GO:0009737 GO:GO:0005634 GO:GO:0005773 EMBL:CP002685 GenomeReviews:CT485783_GR GO:GO:0015293 GO:GO:0005315 eggNOG:COG0477 InterPro:IPR016196 SUPFAM:SSF103473 InterPro:IPR020846 PROSITE:PS50850 EMBL:AC005770 HOGENOM:HBG744844 KO:K08176 TIGRFAMs:TIGR00887 EMBL:U62331 EMBL:AF022872 EMBL:AB016166 IPI:IPI00537042 PIR:C84811 RefSeq:NP_181428.1 UniGene:At.255 UniGene:At.67089 ProteinModelPortal:Q96303 STRING:Q96303 PRIDE:Q96303 EnsemblPlants:AT2G38940.1 GeneID:818479 KEGG:ath:AT2G38940 GeneFarm:5189 TAIR:At2g38940 InParanoid:Q96303 OMA:CYLLIAG PhylomeDB:Q96303 ProtClustDB:CLSN2683235 ArrayExpress:Q96303 Genevestigator:Q96303 GermOnline:AT2G38940 Uniprot:Q96303
Root	Isotig02921	3	57	-4.377	8.39E-15	TAIR|locus:2064895 - symbol:PHT1;4 "AT2G38940" species:3702 "Arabidopsis thaliana" [GO:0005351 "sugar:hydrogen symporter activity" evidence=ISS] [GO:0015114 "phosphate ion transmembrane transporter activity" evidence=ISS] [GO:0015144 "carbohydrate transmembrane transporter activity" evidence=ISS] [GO:0016020 "membrane" evidence=ISS;IDA] [GO:0005886 "plasma membrane" evidence=IDA] [GO:0005773 "vacuole" evidence=IDA] [GO:0005634 "nucleus" evidence=IDA] [GO:0009737 "response to abscisic acid stimulus" evidence=IDA] [GO:0009506 "plasmodesma" evidence=IDA] [GO:0005794 "Golgi apparatus" evidence=IDA] [GO:0005315 "inorganic phosphate transmembrane transporter activity" evidence=ISS] InterPro:IPR004738 InterPro:IPR005828 Pfam:PF00083 PROSITE:PS00216 PROSITE:PS00217 GO:GO:0016021 GO:GO:0005886 GO:GO:0009506 GO:GO:0009737 GO:GO:0005634 GO:GO:0005773 EMBL:CP002685 GenomeReviews:CT485783_GR GO:GO:0015293 GO:GO:0005315 eggNOG:COG0477 InterPro:IPR016196 SUPFAM:SSF103473 InterPro:IPR020846 PROSITE:PS50850 EMBL:AC005770 HOGENOM:HBG744844 KO:K08176 TIGRFAMs:TIGR00887 EMBL:U62331 EMBL:AF022872 EMBL:AB016166 IPI:IPI00537042 PIR:C84811 RefSeq:NP_181428.1 UniGene:At.255 UniGene:At.67089 ProteinModelPortal:Q96303 STRING:Q96303 PRIDE:Q96303 EnsemblPlants:AT2G38940.1 GeneID:818479 KEGG:ath:AT2G38940 GeneFarm:5189 TAIR:At2g38940 InParanoid:Q96303 OMA:CYLLIAG PhylomeDB:Q96303 ProtClustDB:CLSN2683235 ArrayExpress:Q96303 Genevestigator:Q96303 GermOnline:AT2G38940 Uniprot:Q96303
Root	Isotig02922	3	57	-4.377	8.39E-15	TAIR|locus:2064895 - symbol:PHT1;4 "AT2G38940" species:3702 "Arabidopsis thaliana" [GO:0005351 "sugar:hydrogen symporter activity" evidence=ISS] [GO:0015114 "phosphate ion transmembrane transporter activity" evidence=ISS] [GO:0015144 "carbohydrate transmembrane transporter activity" evidence=ISS] [GO:0016020 "membrane" evidence=ISS;IDA] [GO:0005886 "plasma membrane" evidence=IDA] [GO:0005773 "vacuole" evidence=IDA] [GO:0005634 "nucleus" evidence=IDA] [GO:0009737 "response to abscisic acid stimulus" evidence=IDA] [GO:0009506 "plasmodesma" evidence=IDA] [GO:0005794 "Golgi apparatus" evidence=IDA] [GO:0005315 "inorganic phosphate transmembrane transporter activity" evidence=ISS] InterPro:IPR004738 InterPro:IPR005828 Pfam:PF00083 PROSITE:PS00216 PROSITE:PS00217 GO:GO:0016021 GO:GO:0005886

Tissue	Identification	# of reads (stress)	# of reads (control)	Fold Change	p-value	Description
						GO:GO:0009506 GO:GO:0009737 GO:GO:0005634 GO:GO:0005773 EMBL:CP002685 GenomeReviews:CT485783_GR GO:GO:0015293 GO:GO:0005315 eggNOG:COG0477 InterPro:IPR016196 SUPFAM:SSF103473 InterPro:IPR020846 PROSITE:PS50850 EMBL:AC005770 HOGENOM:HBG744844 KO:K08176 TIGRFAMs:TIGR00887 EMBL:U62331 EMBL:AF022872 EMBL:AB016166 IPI:IPI00537042 PIR:C84811 RefSeq:NP_181428.1 UniGene:At.255 UniGene:At.67089 ProteinModelPortal:Q96303 STRING:Q96303 PRIDE:Q96303 EnsemblPlants:AT2G38940.1 GeneID:818479 KEGG:ath:AT2G38940 GeneFarm:5189 TAIR:At2g38940 InParanoid:Q96303 OMA:CYLLIAG PhylomeDB:Q96303 ProtClustDB:CLSN2683235 ArrayExpress:Q96303 Genevestigator:Q96303 GermOnline:AT2G38940 Uniprot:Q96303
Root	Isotig02923	3	48	-4.129	2.32E-12	TAIR|locus:2064895 - symbol:PHT1;4 "AT2G38940" species:3702 "Arabidopsis thaliana" [GO:0005351 "sugar:hydrogen symporter activity" evidence=ISS] [GO:0015114 "phosphate ion transmembrane transporter activity" evidence=ISS] [GO:0015144 "carbohydrate transmembrane transporter activity" evidence=ISS] [GO:0016020 "membrane" evidence=ISS;IDA] [GO:0005886 "plasma membrane" evidence=IDA] [GO:0005773 "vacuole" evidence=IDA] [GO:0005634 "nucleus" evidence=IDA] [GO:0009737 "response to abscisic acid stimulus" evidence=IDA] [GO:0009506 "plasmodesma" evidence=IDA] [GO:0005794 "Golgi apparatus" evidence=IDA] [GO:0005315 "inorganic phosphate transmembrane transporter activity" evidence=ISS] InterPro:IPR004738 InterPro:IPR005828 Pfam:PF00083 PROSITE:PS00216 PROSITE:PS00217 GO:GO:0016021 GO:GO:0005886 GO:GO:0009506 GO:GO:0009737 GO:GO:0005634 GO:GO:0005773 EMBL:CP002685 GenomeReviews:CT485783_GR GO:GO:0015293 GO:GO:0005315 eggNOG:COG0477 InterPro:IPR016196 SUPFAM:SSF103473 InterPro:IPR020846 PROSITE:PS50850 EMBL:AC005770 HOGENOM:HBG744844 KO:K08176 TIGRFAMs:TIGR00887 EMBL:U62331 EMBL:AF022872 EMBL:AB016166 IPI:IPI00537042 PIR:C84811 RefSeq:NP_181428.1 UniGene:At.255 UniGene:At.67089 ProteinModelPortal:Q96303 STRING:Q96303 PRIDE:Q96303 EnsemblPlants:AT2G38940.1 GeneID:818479 KEGG:ath:AT2G38940 GeneFarm:5189 TAIR:At2g38940 InParanoid:Q96303 OMA:CYLLIAG PhylomeDB:Q96303 ProtClustDB:CLSN2683235 ArrayExpress:Q96303 Genevestigator:Q96303 GermOnline:AT2G38940 Uniprot:Q96303
Root	Isotig02924	3	48	-4.129	2.32E-12	TAIR|locus:2064895 - symbol:PHT1;4 "AT2G38940" species:3702 "Arabidopsis thaliana" [GO:0005351 "sugar:hydrogen symporter activity" evidence=ISS] [GO:0015114 "phosphate ion transmembrane transporter activity" evidence=ISS] [GO:0015144 "carbohydrate transmembrane transporter activity" evidence=ISS] [GO:0016020 "membrane" evidence=ISS;IDA] [GO:0005886 "plasma membrane" evidence=IDA] [GO:0005773 "vacuole" evidence=IDA] [GO:0005634 "nucleus" evidence=IDA] [GO:0009737 "response to abscisic acid stimulus" evidence=IDA] [GO:0009506 "plasmodesma" evidence=IDA] [GO:0005794 "Golgi apparatus" evidence=IDA] [GO:0005315 "inorganic phosphate transmembrane transporter activity" evidence=ISS] InterPro:IPR004738 InterPro:IPR005828 Pfam:PF00083 PROSITE:PS00216 PROSITE:PS00217 GO:GO:0016021 GO:GO:0005886 GO:GO:0009506 GO:GO:0009737 GO:GO:0005634 GO:GO:0005773 EMBL:CP002685 GenomeReviews:CT485783_GR GO:GO:0015293 GO:GO:0005315 eggNOG:COG0477 InterPro:IPR016196 SUPFAM:SSF103473 InterPro:IPR020846 PROSITE:PS50850 EMBL:AC005770 HOGENOM:HBG744844 KO:K08176 TIGRFAMs:TIGR00887 EMBL:U62331 EMBL:AF022872 EMBL:AB016166 IPI:IPI00537042 PIR:C84811 RefSeq:NP_181428.1 UniGene:At.255 UniGene:At.67089 ProteinModelPortal:Q96303 STRING:Q96303 PRIDE:Q96303 EnsemblPlants:AT2G38940.1 GeneID:818479 KEGG:ath:AT2G38940 GeneFarm:5189 TAIR:At2g38940 InParanoid:Q96303 OMA:CYLLIAG PhylomeDB:Q96303 ProtClustDB:CLSN2683235 ArrayExpress:Q96303 Genevestigator:Q96303 GermOnline:AT2G38940 Uniprot:Q96303
Root	Isotig02925	3	48	-4.129	2.32E-12	TAIR|locus:2064895 - symbol:PHT1;4 "AT2G38940" species:3702 "Arabidopsis thaliana" [GO:0005351 "sugar:hydrogen symporter activity" evidence=ISS] [GO:0015114 "phosphate ion transmembrane transporter activity" evidence=ISS] [GO:0015144 "carbohydrate transmembrane transporter activity" evidence=ISS] [GO:0016020 "membrane" evidence=ISS;IDA] [GO:0005886 "plasma membrane" evidence=IDA] [GO:0005773 "vacuole" evidence=IDA] [GO:0005634 "nucleus" evidence=IDA] [GO:0009737 "response to abscisic acid stimulus" evidence=IDA] [GO:0009506 "plasmodesma" evidence=IDA] [GO:0005794 "Golgi apparatus" evidence=IDA] [GO:0005315 "inorganic phosphate transmembrane transporter activity" evidence=ISS] InterPro:IPR004738 InterPro:IPR005828 Pfam:PF00083 PROSITE:PS00216 PROSITE:PS00217 GO:GO:0016021 GO:GO:0005886 GO:GO:0009506 GO:GO:0009737 GO:GO:0005634 GO:GO:0005773 EMBL:CP002685

Tissue	Identification	# of reads (stress)	# of reads (control)	Fold Change	p-value	Description
						GenomeReviews:CT485783_GR GO:GO:0015293 GO:GO:0005315 eggNOG:COG0477 InterPro:IPR016196 SUPFAM:SSF103473 InterPro:IPR020846 PROSITE:PS50850 EMBL:AC005770 HOGENOM:HBG744844 KO:K08176 TIGRFAMs:TIGR00887 EMBL:U62331 EMBL:AF022872 EMBL:AB016166 IPI:IPI00537042 PIR:C84811 RefSeq:NP_181428.1 UniGene:At.255 UniGene:At.67089 ProteinModelPortal:Q96303 STRING:Q96303 PRIDE:Q96303 EnsemblPlants:AT2G38940.1 GeneID:818479 KEGG:ath:AT2G38940 GeneFarm:5189 TAIR:At2g38940 InParanoid:Q96303 OMA:CYLLIAG PhylomeDB:Q96303 ProtClustDB:CLSN2683235 ArrayExpress:Q96303 Genevestigator:Q96303 GermOnline:AT2G38940 Uniprot:Q96303
Root	Isotig02932	33	8	1.915	0.000152977	TAIR|locus:2009041 - symbol:AT1G33970 species:3702 "Arabidopsis thaliana" [GO:0005525 "GTP binding" evidence=IEA] [GO:0005575 "cellular_component" evidence=ND] [GO:0009617 "response to bacterium" evidence=ISS] InterPro:IPR006703 Pfam:PF04548 GO:GO:0005525 EMBL:AC015446 EMBL:AC079286 EMBL:AY087627 IPI:IPI00519038 PIR:D86463 ProteinModelPortal:Q9FE56 SMR:Q9FE56 STRING:Q9FE56 PRIDE:Q9FE56 TAIR:At1g33970 InParanoid:Q9FE56 PhylomeDB:Q9FE56 Genevestigator:Q9FE56 Uniprot:Q9FE56
Root	Isotig02933	33	8	1.915	0.000152977	TAIR|locus:2009041 - symbol:AT1G33970 species:3702 "Arabidopsis thaliana" [GO:0005525 "GTP binding" evidence=IEA] [GO:0005575 "cellular_component" evidence=ND] [GO:0009617 "response to bacterium" evidence=ISS] InterPro:IPR006703 Pfam:PF04548 GO:GO:0005525 EMBL:AC015446 EMBL:AC079286 EMBL:AY087627 IPI:IPI00519038 PIR:D86463 ProteinModelPortal:Q9FE56 SMR:Q9FE56 STRING:Q9FE56 PRIDE:Q9FE56 TAIR:At1g33970 InParanoid:Q9FE56 PhylomeDB:Q9FE56 Genevestigator:Q9FE56 Uniprot:Q9FE56
Root	Isotig02934	33	8	1.915	0.000152977	TAIR|locus:2009041 - symbol:AT1G33970 species:3702 "Arabidopsis thaliana" [GO:0005525 "GTP binding" evidence=IEA] [GO:0005575 "cellular_component" evidence=ND] [GO:0009617 "response to bacterium" evidence=ISS] InterPro:IPR006703 Pfam:PF04548 GO:GO:0005525 EMBL:AC015446 EMBL:AC079286 EMBL:AY087627 IPI:IPI00519038 PIR:D86463 ProteinModelPortal:Q9FE56 SMR:Q9FE56 STRING:Q9FE56 PRIDE:Q9FE56 TAIR:At1g33970 InParanoid:Q9FE56 PhylomeDB:Q9FE56 Genevestigator:Q9FE56 Uniprot:Q9FE56
Root	Isotig02935	33	8	1.915	0.000152977	TAIR|locus:2009041 - symbol:AT1G33970 species:3702 "Arabidopsis thaliana" [GO:0005525 "GTP binding" evidence=IEA] [GO:0005575 "cellular_component" evidence=ND] [GO:0009617 "response to bacterium" evidence=ISS] InterPro:IPR006703 Pfam:PF04548 GO:GO:0005525 EMBL:AC015446 EMBL:AC079286 EMBL:AY087627 IPI:IPI00519038 PIR:D86463 ProteinModelPortal:Q9FE56 SMR:Q9FE56 STRING:Q9FE56 PRIDE:Q9FE56 TAIR:At1g33970 InParanoid:Q9FE56 PhylomeDB:Q9FE56 Genevestigator:Q9FE56 Uniprot:Q9FE56
Root	Isotig02936	33	8	1.915	0.000152977	TAIR|locus:2009041 - symbol:AT1G33970 species:3702 "Arabidopsis thaliana" [GO:0005525 "GTP binding" evidence=IEA] [GO:0005575 "cellular_component" evidence=ND] [GO:0009617 "response to bacterium" evidence=ISS] InterPro:IPR006703 Pfam:PF04548 GO:GO:0005525 EMBL:AC015446 EMBL:AC079286 EMBL:AY087627 IPI:IPI00519038 PIR:D86463 ProteinModelPortal:Q9FE56 SMR:Q9FE56 STRING:Q9FE56 PRIDE:Q9FE56 TAIR:At1g33970 InParanoid:Q9FE56 PhylomeDB:Q9FE56 Genevestigator:Q9FE56 Uniprot:Q9FE56
Root	Isotig02937	33	8	1.915	0.000152977	TAIR|locus:2009041 - symbol:AT1G33970 species:3702 "Arabidopsis thaliana" [GO:0005525 "GTP binding" evidence=IEA] [GO:0005575 "cellular_component" evidence=ND] [GO:0009617 "response to bacterium" evidence=ISS] InterPro:IPR006703 Pfam:PF04548 GO:GO:0005525 EMBL:AC015446 EMBL:AC079286 EMBL:AY087627 IPI:IPI00519038 PIR:D86463 ProteinModelPortal:Q9FE56 SMR:Q9FE56 STRING:Q9FE56 PRIDE:Q9FE56 TAIR:At1g33970 InParanoid:Q9FE56 PhylomeDB:Q9FE56 Genevestigator:Q9FE56 Uniprot:Q9FE56
Root	Isotig02950	13	47	-1.983	1.33E-06	TAIR|locus:2180872 - symbol:UGD3 "AT5G15490" species:3702 "Arabidopsis thaliana" [GO:0003979 "UDP-glucose 6-dehydrogenase activity" evidence=IGI;ISS] [GO:0005618 "cell wall" evidence=IDA] [GO:0005634 "nucleus" evidence=IDA] [GO:0005829 "cytosol" evidence=IDA] [GO:0005975 "carbohydrate metabolic process" evidence=IGI] [GO:0052546 "cell wall pectin metabolic process" evidence=IGI] InterPro:IPR001732 InterPro:IPR008927 InterPro:IPR014026 InterPro:IPR014027 InterPro:IPR016040 InterPro:IPR017476 Pfam:PF00984 Pfam:PF03720 Pfam:PF03721 PIRSF:PIRSF000124 SMART:SM00984 GO:GO:0005829 GO:GO:0005634 EMBL:CP002688 GenomeReviews:BA000015_GR GO:GO:0005618 Gene3D:G3DSA:3.40.50.720 GO:GO:0051287 SUPFAM:SSF48179 EMBL:AL391143 InterPro:IPR021157 Gene3D:G3DSA:1.20.5.100

Tissue	Identification	# of reads (stress)	# of reads (control)	Fold Change	p-value	Description
						GO:GO:0052546 GO:GO:0003979 HOGENOM:HBG400967 KO:K00012 SUPFAM:SSF52413 TIGRFAMs:TIGR03026 ProtClustDB:PLN02353 EMBL:AY056200 EMBL:BT015794 IPI:IPI00527538 PIR:T51527 RefSeq:NP_197053.1 UniGene:At.20910 HSSP:P11759 ProteinModelPortal:Q9LF33 SMR:Q9LF33 STRING:Q9LF33 PRIDE:Q9LF33 EnsemblPlants:AT5G15490.1 GeneID:831402 KEGG:ath:AT5G15490 TAIR:At5g15490 InParanoid:Q9LF33 OMA:FQRIFEN PhylomeDB:Q9LF33 ArrayExpress:Q9LF33 Genevestigator:Q9LF33 Uniprot:Q9LF33
Root	Isotig02951	13	46	-1.952	2.20E-06	TAIR|locus:2180872 - symbol:UGD3 "AT5G15490" species:3702 "Arabidopsis thaliana" [GO:0003979 "UDP-glucose 6-dehydrogenase activity" evidence=IGI;ISS] [GO:0005618 "cell wall" evidence=IDA] [GO:0005634 "nucleus" evidence=IDA] [GO:0005829 "cytosol" evidence=IDA] [GO:0005975 "carbohydrate metabolic process" evidence=IGI] [GO:0052546 "cell wall pectin metabolic process" evidence=IGI] InterPro:IPR001732 InterPro:IPR008927 InterPro:IPR014026 InterPro:IPR014027 InterPro:IPR016040 InterPro:IPR017476 Pfam:PF00984 Pfam:PF03720 Pfam:PF03721 PIRSF:PIRSF000124 SMART:SM00984 GO:GO:0005829 GO:GO:0005634 EMBL:CP002688 GenomeReviews:BA000015_GR GO:GO:0005618 Gene3D:G3DSA:3.40.50.720 GO:GO:0051287 SUPFAM:SSF48179 EMBL:AL391143 InterPro:IPR021157 Gene3D:G3DSA:1.20.5.100 GO:GO:0052546 GO:GO:0003979 HOGENOM:HBG400967 KO:K00012 SUPFAM:SSF52413 TIGRFAMs:TIGR03026 ProtClustDB:PLN02353 EMBL:AY056200 EMBL:BT015794 IPI:IPI00527538 PIR:T51527 RefSeq:NP_197053.1 UniGene:At.20910 HSSP:P11759 ProteinModelPortal:Q9LF33 SMR:Q9LF33 STRING:Q9LF33 PRIDE:Q9LF33 EnsemblPlants:AT5G15490.1 GeneID:831402 KEGG:ath:AT5G15490 TAIR:At5g15490 InParanoid:Q9LF33 OMA:FQRIFEN PhylomeDB:Q9LF33 ArrayExpress:Q9LF33 Genevestigator:Q9LF33 Uniprot:Q9LF33
Root	Isotig02953	9	32	-1.959	7.57E-05	TAIR|locus:2180872 - symbol:UGD3 "AT5G15490" species:3702 "Arabidopsis thaliana" [GO:0003979 "UDP-glucose 6-dehydrogenase activity" evidence=IGI;ISS] [GO:0005618 "cell wall" evidence=IDA] [GO:0005634 "nucleus" evidence=IDA] [GO:0005829 "cytosol" evidence=IDA] [GO:0005975 "carbohydrate metabolic process" evidence=IGI] [GO:0052546 "cell wall pectin metabolic process" evidence=IGI] InterPro:IPR001732 InterPro:IPR008927 InterPro:IPR014026 InterPro:IPR014027 InterPro:IPR016040 InterPro:IPR017476 Pfam:PF00984 Pfam:PF03720 Pfam:PF03721 PIRSF:PIRSF000124 SMART:SM00984 GO:GO:0005829 GO:GO:0005634 EMBL:CP002688 GenomeReviews:BA000015_GR GO:GO:0005618 Gene3D:G3DSA:3.40.50.720 GO:GO:0051287 SUPFAM:SSF48179 EMBL:AL391143 InterPro:IPR021157 Gene3D:G3DSA:1.20.5.100 GO:GO:0052546 GO:GO:0003979 HOGENOM:HBG400967 KO:K00012 SUPFAM:SSF52413 TIGRFAMs:TIGR03026 ProtClustDB:PLN02353 EMBL:AY056200 EMBL:BT015794 IPI:IPI00527538 PIR:T51527 RefSeq:NP_197053.1 UniGene:At.20910 HSSP:P11759 ProteinModelPortal:Q9LF33 SMR:Q9LF33 STRING:Q9LF33 PRIDE:Q9LF33 EnsemblPlants:AT5G15490.1 GeneID:831402 KEGG:ath:AT5G15490 TAIR:At5g15490 InParanoid:Q9LF33 OMA:FQRIFEN PhylomeDB:Q9LF33 ArrayExpress:Q9LF33 Genevestigator:Q9LF33 Uniprot:Q9LF33
Root	Isotig02954	9	31	-1.913	0.000125821	TAIR|locus:2180872 - symbol:UGD3 "AT5G15490" species:3702 "Arabidopsis thaliana" [GO:0003979 "UDP-glucose 6-dehydrogenase activity" evidence=IGI;ISS] [GO:0005618 "cell wall" evidence=IDA] [GO:0005634 "nucleus" evidence=IDA] [GO:0005829 "cytosol" evidence=IDA] [GO:0005975 "carbohydrate metabolic process" evidence=IGI] [GO:0052546 "cell wall pectin metabolic process" evidence=IGI] InterPro:IPR001732 InterPro:IPR008927 InterPro:IPR014026 InterPro:IPR014027 InterPro:IPR016040 InterPro:IPR017476 Pfam:PF00984 Pfam:PF03720 Pfam:PF03721 PIRSF:PIRSF000124 SMART:SM00984 GO:GO:0005829 GO:GO:0005634 EMBL:CP002688 GenomeReviews:BA000015_GR GO:GO:0005618 Gene3D:G3DSA:3.40.50.720 GO:GO:0051287 SUPFAM:SSF48179 EMBL:AL391143 InterPro:IPR021157 Gene3D:G3DSA:1.20.5.100 GO:GO:0052546 GO:GO:0003979 HOGENOM:HBG400967 KO:K00012 SUPFAM:SSF52413 TIGRFAMs:TIGR03026 ProtClustDB:PLN02353 EMBL:AY056200 EMBL:BT015794 IPI:IPI00527538 PIR:T51527 RefSeq:NP_197053.1 UniGene:At.20910 HSSP:P11759 ProteinModelPortal:Q9LF33 SMR:Q9LF33 STRING:Q9LF33 PRIDE:Q9LF33 EnsemblPlants:AT5G15490.1 GeneID:831402 KEGG:ath:AT5G15490 TAIR:At5g15490 InParanoid:Q9LF33 OMA:FQRIFEN PhylomeDB:Q9LF33 ArrayExpress:Q9LF33 Genevestigator:Q9LF33 Uniprot:Q9LF33
Root	Isotig02960	30	2	3.778	2.52E-07	TAIR|locus:2183901 - symbol:AT5G13200 "AT5G13200" species:3702 "Arabidopsis thaliana" [GO:0003674 "molecular_function" evidence=ND] [GO:0008150 "biological_process" evidence=ND] Pfam:PF02893 EMBL:CP002688 GenomeReviews:BA000015_GR EMBL:AL391711 EMBL:AL163491 InterPro:IPR004182 SMART:SM00568 HOGENOM:HBG745499 EMBL:BT010714 EMBL:BT012428 EMBL:AK229276 IPI:IPI00528201

Tissue	Identification	# of reads (stress)	# of reads (control)	Fold Change	p-value	Description
						PIR:T48567 RefSeq:NP_196824.1 UniGene:At.25214 STRING:Q9LYV6 PRIDE:Q9LYV6 DNASU:831159 EnsemblPlants:AT5G13200.1 GeneID:831159 KEGG:ath:AT5G13200 TAIR:At5g13200 eggNOG:NOG289836 InParanoid:Q9LYV6 OMA:WHNLKTG PhylomeDB:Q9LYV6 ProtClustDB:CLSN2686867 Genevestigator:Q9LYV6 Uniprot:Q9LYV6
Root	Isotig02961	30	2	3.778	2.52E-07	TAIR|locus:2183901 - symbol:AT5G13200 "AT5G13200" species:3702 "Arabidopsis thaliana" [GO:0003674 "molecular_function" evidence=ND] [GO:0008150 "biological_process" evidence=ND] Pfam:PF02893 EMBL:CP002688 GenomeReviews:BA000015_GR EMBL:AL391711 EMBL:AL163491 InterPro:IPR004182 SMART:SM00568 HOGENOM:HBG745499 EMBL:BT010714 EMBL:BT012428 EMBL:AK229276 IPI:IPI00528201 PIR:T48567 RefSeq:NP_196824.1 UniGene:At.25214 STRING:Q9LYV6 PRIDE:Q9LYV6 DNASU:831159 EnsemblPlants:AT5G13200.1 GeneID:831159 KEGG:ath:AT5G13200 TAIR:At5g13200 eggNOG:NOG289836 InParanoid:Q9LYV6 OMA:WHNLKTG PhylomeDB:Q9LYV6 ProtClustDB:CLSN2686867 Genevestigator:Q9LYV6 Uniprot:Q9LYV6
Root	Isotig02962	24	1	4.456	1.32E-06	TAIR|locus:2183901 - symbol:AT5G13200 "AT5G13200" species:3702 "Arabidopsis thaliana" [GO:0003674 "molecular_function" evidence=ND] [GO:0008150 "biological_process" evidence=ND] Pfam:PF02893 EMBL:CP002688 GenomeReviews:BA000015_GR EMBL:AL391711 EMBL:AL163491 InterPro:IPR004182 SMART:SM00568 HOGENOM:HBG745499 EMBL:BT010714 EMBL:BT012428 EMBL:AK229276 IPI:IPI00528201 PIR:T48567 RefSeq:NP_196824.1 UniGene:At.25214 STRING:Q9LYV6 PRIDE:Q9LYV6 DNASU:831159 EnsemblPlants:AT5G13200.1 GeneID:831159 KEGG:ath:AT5G13200 TAIR:At5g13200 eggNOG:NOG289836 InParanoid:Q9LYV6 OMA:WHNLKTG PhylomeDB:Q9LYV6 ProtClustDB:CLSN2686867 Genevestigator:Q9LYV6 Uniprot:Q9LYV6
Root	Isotig02963	24	1	4.456	1.32E-06	TAIR|locus:2183901 - symbol:AT5G13200 "AT5G13200" species:3702 "Arabidopsis thaliana" [GO:0003674 "molecular_function" evidence=ND] [GO:0008150 "biological_process" evidence=ND] Pfam:PF02893 EMBL:CP002688 GenomeReviews:BA000015_GR EMBL:AL391711 EMBL:AL163491 InterPro:IPR004182 SMART:SM00568 HOGENOM:HBG745499 EMBL:BT010714 EMBL:BT012428 EMBL:AK229276 IPI:IPI00528201 PIR:T48567 RefSeq:NP_196824.1 UniGene:At.25214 STRING:Q9LYV6 PRIDE:Q9LYV6 DNASU:831159 EnsemblPlants:AT5G13200.1 GeneID:831159 KEGG:ath:AT5G13200 TAIR:At5g13200 eggNOG:NOG289836 InParanoid:Q9LYV6 OMA:WHNLKTG PhylomeDB:Q9LYV6 ProtClustDB:CLSN2686867 Genevestigator:Q9LYV6 Uniprot:Q9LYV6
Root	Isotig02964	26	2	3.571	2.56E-06	TAIR|locus:2183901 - symbol:AT5G13200 "AT5G13200" species:3702 "Arabidopsis thaliana" [GO:0003674 "molecular_function" evidence=ND] [GO:0008150 "biological_process" evidence=ND] Pfam:PF02893 EMBL:CP002688 GenomeReviews:BA000015_GR EMBL:AL391711 EMBL:AL163491 InterPro:IPR004182 SMART:SM00568 HOGENOM:HBG745499 EMBL:BT010714 EMBL:BT012428 EMBL:AK229276 IPI:IPI00528201 PIR:T48567 RefSeq:NP_196824.1 UniGene:At.25214 STRING:Q9LYV6 PRIDE:Q9LYV6 DNASU:831159 EnsemblPlants:AT5G13200.1 GeneID:831159 KEGG:ath:AT5G13200 TAIR:At5g13200 eggNOG:NOG289836 InParanoid:Q9LYV6 OMA:WHNLKTG PhylomeDB:Q9LYV6 ProtClustDB:CLSN2686867 Genevestigator:Q9LYV6 Uniprot:Q9LYV6
Root	Isotig02965	72	0	7.041	2.73E-17	TAIR|locus:2115235 - symbol:AT4G36600 "AT4G36600" species:3702 "Arabidopsis thaliana" [GO:0003674 "molecular_function" evidence=ND] [GO:0009793 "embryo development ending in seed dormancy" evidence=ISS] Pfam:PF02987 EMBL:BT012603 IPI:IPI00516442 PRIDE:Q6NKU0 TAIR:At4g36600 InParanoid:Q6NKU0 PhylomeDB:Q6NKU0 Genevestigator:Q6NKU0 InterPro:IPR004238 Uniprot:Q6NKU0
Root	Isotig02966	64	0	6.871	1.11E-15	TAIR|locus:2035109 - symbol:LEA7 "AT1G52690" species:3702 "Arabidopsis thaliana" [GO:0003674 "molecular_function" evidence=ND] [GO:0005575 "cellular_component" evidence=ND] [GO:0009793 "embryo development ending in seed dormancy" evidence=ISS] EMBL:CP002684 EMBL:AC008016 EMBL:AF385732 EMBL:AY093968 EMBL:AY087466 EMBL:BT000737 EMBL:BT000739 EMBL:X91919 IPI:IPI00526817 PIR:H96567 RefSeq:NP_175678.1 RefSeq:NP_974009.1 UniGene:At.21275 STRING:Q96270 PRIDE:Q96270 ProMEX:Q96270 DNASU:841701 EnsemblPlants:AT1G52690.1 EnsemblPlants:AT1G52690.2 GeneID:841701 KEGG:ath:AT1G52690 TAIR:At1g52690 InParanoid:Q96270 OMA:MASHQEQ PhylomeDB:Q96270 ProtClustDB:CLSN2679745 ArrayExpress:Q96270 Genevestigator:Q96270 Uniprot:Q96270
Root	Isotig02967	46	0	6.395	6.55E-12	No hit
Root	Isotig02968	62	0	6.825	2.85E-15	TAIR|locus:2035109 - symbol:LEA7 "AT1G52690" species:3702 "Arabidopsis thaliana" [GO:0003674 "molecular_function" evidence=ND] [GO:0005575 "cellular_component" evidence=ND] [GO:0009793 "embryo development ending in seed dormancy" evidence=ISS] EMBL:CP002684 EMBL:AC008016 EMBL:AF385732

Tissue	Identification	# of reads (stress)	# of reads (control)	Fold Change	p-value	Description
						EMBL:AY093968 EMBL:AY087466 EMBL:BT000737 EMBL:BT000739 EMBL:X91919 IPI:IPI00526817 PIR:H96567 RefSeq:NP_175678.1 RefSeq:NP_974009.1 UniGene:At.21275 STRING:Q96270 PRIDE:Q96270 ProMEX:Q96270 DNASU:841701 EnsemblPlants:AT1G52690.1 EnsemblPlants:AT1G52690.2 GeneID:841701 KEGG:ath:AT1G52690 TAIR:At1g52690 InParanoid:Q96270 OMA:MASHQEQ PhylomeDB:Q96270 ProtClustDB:CLSN2679745 ArrayExpress:Q96270 Genevestigator:Q96270 Uniprot:Q96270
Root	Isotig02969	44	0	6.330	1.78E-11	No hit
Root	Isotig02980	0	24	-5.714	1.84E-07	TAIR|locus:2153529 - symbol:PRX52 "AT5G05340" species:3702 "Arabidopsis thaliana" [GO:0004601 "peroxidase activity" evidence=ISS] [GO:0006979 "response to oxidative stress" evidence=IEA] [GO:0055114 "oxidation-reduction process" evidence=IEA] [GO:0005618 "cell wall" evidence=IDA] [GO:0048046 "apoplast" evidence=IDA] [GO:0005515 "protein binding" evidence=IPI] [GO:0005829 "cytosol" evidence=IDA] [GO:0005794 "Golgi apparatus" evidence=IDA] InterPro:IPR000823 InterPro:IPR002016 InterPro:IPR010255 InterPro:IPR019793 InterPro:IPR019794 Pfam:PF00141 PRINTS:PR00458 PRINTS:PR00461 PROSITE:PS00435 PROSITE:PS00436 PROSITE:PS50873 GO:GO:0005829 GO:GO:0048046 EMBL:CP002688 GenomeReviews:BA000015_GR GO:GO:0005618 GO:GO:0005515 GO:GO:0020037 GO:GO:0004601 GO:GO:0042744 HOGENOM:HBG597790 SUPFAM:SSF48113 EMBL:AB010692 KO:K00430 EMBL:AY065270 IPI:IPI00527468 RefSeq:NP_196153.1 UniGene:At.28537 ProteinModelPortal:Q9FLC0 SMR:Q9FLC0 IntAct:Q9FLC0 STRING:Q9FLC0 PeroxiBase:218 PRIDE:Q9FLC0 EnsemblPlants:AT5G05340.1 GeneID:830416 KEGG:ath:AT5G05340 GeneFarm:1905 TAIR:At5g05340 eggNOG:NOG298084 InParanoid:Q9FLC0 OMA:TNFRARI PhylomeDB:Q9FLC0 ProtClustDB:CLSN2916362 ArrayExpress:Q9FLC0 Genevestigator:Q9FLC0 GermOnline:AT5G05340 Uniprot:Q9FLC0
Root	Isotig02981	0	28	-5.936	1.79E-08	TAIR|locus:2153529 - symbol:PRX52 "AT5G05340" species:3702 "Arabidopsis thaliana" [GO:0004601 "peroxidase activity" evidence=ISS] [GO:0006979 "response to oxidative stress" evidence=IEA] [GO:0055114 "oxidation-reduction process" evidence=IEA] [GO:0005618 "cell wall" evidence=IDA] [GO:0048046 "apoplast" evidence=IDA] [GO:0005515 "protein binding" evidence=IPI] [GO:0005829 "cytosol" evidence=IDA] [GO:0005794 "Golgi apparatus" evidence=IDA] InterPro:IPR000823 InterPro:IPR002016 InterPro:IPR010255 InterPro:IPR019793 InterPro:IPR019794 Pfam:PF00141 PRINTS:PR00458 PRINTS:PR00461 PROSITE:PS00435 PROSITE:PS00436 PROSITE:PS50873 GO:GO:0005829 GO:GO:0048046 EMBL:CP002688 GenomeReviews:BA000015_GR GO:GO:0005618 GO:GO:0005515 GO:GO:0020037 GO:GO:0004601 GO:GO:0042744 HOGENOM:HBG597790 SUPFAM:SSF48113 EMBL:AB010692 KO:K00430 EMBL:AY065270 IPI:IPI00527468 RefSeq:NP_196153.1 UniGene:At.28537 ProteinModelPortal:Q9FLC0 SMR:Q9FLC0 IntAct:Q9FLC0 STRING:Q9FLC0 PeroxiBase:218 PRIDE:Q9FLC0 EnsemblPlants:AT5G05340.1 GeneID:830416 KEGG:ath:AT5G05340 GeneFarm:1905 TAIR:At5g05340 eggNOG:NOG298084 InParanoid:Q9FLC0 OMA:TNFRARI PhylomeDB:Q9FLC0 ProtClustDB:CLSN2916362 ArrayExpress:Q9FLC0 Genevestigator:Q9FLC0 GermOnline:AT5G05340 Uniprot:Q9FLC0
Root	Isotig02982	0	24	-5.714	1.84E-07	TAIR|locus:2153529 - symbol:PRX52 "AT5G05340" species:3702 "Arabidopsis thaliana" [GO:0004601 "peroxidase activity" evidence=ISS] [GO:0006979 "response to oxidative stress" evidence=IEA] [GO:0055114 "oxidation-reduction process" evidence=IEA] [GO:0005618 "cell wall" evidence=IDA] [GO:0048046 "apoplast" evidence=IDA] [GO:0005515 "protein binding" evidence=IPI] [GO:0005829 "cytosol" evidence=IDA] [GO:0005794 "Golgi apparatus" evidence=IDA] InterPro:IPR000823 InterPro:IPR002016 InterPro:IPR010255 InterPro:IPR019793 InterPro:IPR019794 Pfam:PF00141 PRINTS:PR00458 PRINTS:PR00461 PROSITE:PS00435 PROSITE:PS00436 PROSITE:PS50873 GO:GO:0005829 GO:GO:0048046 EMBL:CP002688 GenomeReviews:BA000015_GR GO:GO:0005618 GO:GO:0005515 GO:GO:0020037 GO:GO:0004601 GO:GO:0042744 HOGENOM:HBG597790 SUPFAM:SSF48113 EMBL:AB010692 KO:K00430 EMBL:AY065270 IPI:IPI00527468 RefSeq:NP_196153.1 UniGene:At.28537 ProteinModelPortal:Q9FLC0 SMR:Q9FLC0 IntAct:Q9FLC0 STRING:Q9FLC0 PeroxiBase:218 PRIDE:Q9FLC0 EnsemblPlants:AT5G05340.1 GeneID:830416 KEGG:ath:AT5G05340 GeneFarm:1905 TAIR:At5g05340 eggNOG:NOG298084 InParanoid:Q9FLC0 OMA:TNFRARI PhylomeDB:Q9FLC0 ProtClustDB:CLSN2916362 ArrayExpress:Q9FLC0 Genevestigator:Q9FLC0 GermOnline:AT5G05340 Uniprot:Q9FLC0
Root	Isotig02983	0	28	-5.936	1.79E-08	TAIR|locus:2153529 - symbol:PRX52 "AT5G05340" species:3702 "Arabidopsis thaliana" [GO:0004601 "peroxidase activity" evidence=ISS] [GO:0006979 "response to oxidative stress" evidence=IEA] [GO:0055114 "oxidation-reduction process" evidence=IEA] [GO:0005618 "cell wall" evidence=IDA] [GO:0048046 "apoplast" evidence=IDA] [GO:0005515 "protein binding" evidence=IPI] [GO:0005829 "cytosol" evidence=IDA] [GO:0005794 "Golgi apparatus"

Tissue	Identification	# of reads (stress)	# of reads (control)	Fold Change	p-value	Description
						evidence=IDA] InterPro:IPR000823 InterPro:IPR002016 InterPro:IPR010255 InterPro:IPR019793 InterPro:IPR019794 Pfam:PF00141 PRINTS:PR00458 PRINTS:PR00461 PROSITE:PS00435 PROSITE:PS00436 PROSITE:PS50873 GO:GO:0005829 GO:GO:0048046 EMBL:CP002688 GenomeReviews:BA000015_GR GO:GO:0005618 GO:GO:0005515 GO:GO:0020037 GO:GO:0004601 GO:GO:0042744 HOGENOM:HBG597790 SUPFAM:SSF48113 EMBL:AB010692 KO:K00430 EMBL:AY065270 IPI:IPI00527468 RefSeq:NP_196153.1 UniGene:At.28537 ProteinModelPortal:Q9FLC0 SMR:Q9FLC0 IntAct:Q9FLC0 STRING:Q9FLC0 PeroxiBase:218 PRIDE:Q9FLC0 EnsemblPlants:AT5G05340.1 GeneID:830416 KEGG:ath:AT5G05340 GeneFarm:1905 TAIR:At5g05340 eggNOG:NOG298084 InParanoid:Q9FLC0 OMA:TNFRARI PhylomeDB:Q9FLC0 ProtClustDB:CLSN2916362 ArrayExpress:Q9FLC0 Genevestigator:Q9FLC0 GermOnline:AT5G05340 Uniprot:Q9FLC0
Root	Isotig03000	32	61	-1.060	0.000551724	DICTYBASE|DDB_G0277823 - symbol:alaS "alanyl-tRNA synthetase,alanine-tRNA ligase" species:44689 "Dictyostelium discoideum" [GO:0004813 "alanine-tRNA ligase activity" evidence=ISS] [GO:0005524 "ATP binding" evidence=ISS] [GO:0005737 "cytoplasm" evidence=ISS] [GO:0006419 "alanyl-tRNA aminoacylation" evidence=ISS] InterPro:IPR002318 InterPro:IPR003156 InterPro:IPR012947 InterPro:IPR018162 InterPro:IPR018163 InterPro:IPR018164 InterPro:IPR018165 InterPro:IPR023033 Pfam:PF01411 Pfam:PF02272 Pfam:PF07973 PRINTS:PR00980 PROSITE:PS50860 SMART:SM00863 dictyBase:DDB_G0277823 GO:GO:0005524 GO:GO:0005737 GenomeReviews:CM000152_GR GO:GO:0046872 EMBL:AAFI02000023 GO:GO:0004813 GO:GO:0006419 SUPFAM:SSF55186 GO:GO:0000049 PANTHER:PTHR11777:SF6 eggNOG:COG0013 HOGENOM:HBG354397 KO:K01872 SUPFAM:SSF101353 TIGRFAMs:TIGR00344 EMBL:AF188717 RefSeq:XP_642382.1 HSSP:O67323 ProteinModelPortal:Q54Y20 STRING:Q54Y20 PRIDE:Q54Y20 EnsemblProtists:DDB0214894 GeneID:8621587 KEGG:ddi:DDB_G0277823 OMA:EGSFGTF PhylomeDB:Q54Y20 ProtClustDB:CLSZ2429158 Uniprot:Q54Y20
Root	Isotig03001	32	61	-1.060	0.000551724	TAIR|locus:2011962 - symbol:ALATS "Alanyl-tRNA synthetase" species:3702 "Arabidopsis thaliana" [GO:0000166 "nucleotide binding" evidence=IEA] [GO:0003676 "nucleic acid binding" evidence=IEA] [GO:0004813 "alanine-tRNA ligase activity" evidence=IEA] [GO:0005524 "ATP binding" evidence=IEA] [GO:0006419 "alanyl-tRNA aminoacylation" evidence=ISS] [GO:0016876 "ligase activity, forming aminoacyl-tRNA and related compounds" evidence=IEA] [GO:0005739 "mitochondrion" evidence=IDA] [GO:0009507 "chloroplast" evidence=IDA] [GO:0046686 "response to cadmium ion" evidence=IEP] [GO:0005829 "cytosol" evidence=IDA] InterPro:IPR002318 InterPro:IPR003156 InterPro:IPR012947 InterPro:IPR018162 InterPro:IPR018163 InterPro:IPR018164 InterPro:IPR018165 InterPro:IPR023033 Pfam:PF01411 Pfam:PF02272 Pfam:PF07973 PRINTS:PR00980 PROSITE:PS50860 SMART:SM00863 EMBL:CP002684 GenomeReviews:CT485782_GR GO:GO:0005829 GO:GO:0005739 GO:GO:0005524 GO:GO:0046686 GO:GO:0009507 GO:GO:0046872 GO:GO:0004813 GO:GO:0006419 SUPFAM:SSF55186 GO:GO:0000049 EMBL:AC007980 eggNOG:COG0013 HOGENOM:HBG354397 KO:K01872 SUPFAM:SSF101353 TIGRFAMs:TIGR00344 OMA:EGSFGTF EMBL:Z22673 EMBL:BT002526 EMBL:BT008807 EMBL:Y12555 IPI:IPI00537243 IPI:IPI00760312 PIR:D96538 RefSeq:NP_175439.2 UniGene:At.1836 ProteinModelPortal:P36428 SMR:P36428 STRING:P36428 PRIDE:P36428 ProMEX:P36428 EnsemblPlants:AT1G50200.1 GeneID:841442 KEGG:ath:AT1G50200 GeneFarm:2386 TAIR:At1g50200 InParanoid:P36428 PhylomeDB:P36428 ProtClustDB:PLN02900 ArrayExpress:P36428 Genevestigator:P36428 GermOnline:AT1G50200 Uniprot:P36428
Root	Isotig03002	32	61	-1.060	0.000551724	DICTYBASE|DDB_G0277823 - symbol:alaS "alanyl-tRNA synthetase,alanine-tRNA ligase" species:44689 "Dictyostelium discoideum" [GO:0004813 "alanine-tRNA ligase activity" evidence=ISS] [GO:0005524 "ATP binding" evidence=ISS] [GO:0005737 "cytoplasm" evidence=ISS] [GO:0006419 "alanyl-tRNA aminoacylation" evidence=ISS] InterPro:IPR002318 InterPro:IPR003156 InterPro:IPR012947 InterPro:IPR018162 InterPro:IPR018163 InterPro:IPR018164 InterPro:IPR018165 InterPro:IPR023033 Pfam:PF01411 Pfam:PF02272 Pfam:PF07973 PRINTS:PR00980 PROSITE:PS50860 SMART:SM00863 dictyBase:DDB_G0277823 GO:GO:0005524 GO:GO:0005737 GenomeReviews:CM000152_GR GO:GO:0046872 EMBL:AAFI02000023 GO:GO:0004813 GO:GO:0006419 SUPFAM:SSF55186 GO:GO:0000049 PANTHER:PTHR11777:SF6 eggNOG:COG0013 HOGENOM:HBG354397 KO:K01872 SUPFAM:SSF101353 TIGRFAMs:TIGR00344 EMBL:AF188717 RefSeq:XP_642382.1 HSSP:O67323 ProteinModelPortal:Q54Y20 STRING:Q54Y20 PRIDE:Q54Y20 EnsemblProtists:DDB0214894 GeneID:8621587 KEGG:ddi:DDB_G0277823 OMA:EGSFGTF PhylomeDB:Q54Y20 ProtClustDB:CLSZ2429158 Uniprot:Q54Y20

Tissue	Identification	# of reads (stress)	# of reads (control)	Fold Change	p-value	Description
Root	Isotig03003	32	61	-1.060	0.000551724	TAIR|locus:2011962 - symbol:ALATS "Alanyl-tRNA synthetase" species:3702 "Arabidopsis thaliana" [GO:0000166 "nucleotide binding" evidence=IEA] [GO:0003676 "nucleic acid binding" evidence=IEA] [GO:0004813 "alanine-tRNA ligase activity" evidence=IEA] [GO:0005524 "ATP binding" evidence=IEA] [GO:0006419 "alanyl-tRNA aminoacylation" evidence=ISS] [GO:0016876 "ligase activity, forming aminoacyl-tRNA and related compounds" evidence=IEA] [GO:0005739 "mitochondrion" evidence=IDA] [GO:0009507 "chloroplast" evidence=IDA] [GO:0046686 "response to cadmium ion" evidence=IEP] [GO:0005829 "cytosol" evidence=IDA] InterPro:IPR002318 InterPro:IPR003156 InterPro:IPR012947 InterPro:IPR018162 InterPro:IPR018163 InterPro:IPR018164 InterPro:IPR018165 InterPro:IPR023033 Pfam:PF01411 Pfam:PF02272 Pfam:PF07973 PRINTS:PR00980 PROSITE:PS50860 SMART:SM00863 EMBL:CP002684 GenomeReviews:CT485782_GR GO:GO:0005829 GO:GO:0005739 GO:GO:0005524 GO:GO:0046686 GO:GO:0009507 GO:GO:0046872 GO:GO:0004813 GO:GO:0006419 SUPFAM:SSF55186 GO:GO:0000049 EMBL:AC007980 eggNOG:COG0013 HOGENOM:HBG354397 KO:K01872 SUPFAM:SSF101353 TIGRFAMs:TIGR00344 OMA:EGSFGTF EMBL:Z22673 EMBL:BT002526 EMBL:BT008807 EMBL:Y12555 IPI:IPI00537243 IPI:IPI00760312 PIR:D96538 RefSeq:NP_175439.2 UniGene:At.1836 ProteinModelPortal:P36428 SMR:P36428 STRING:P36428 PRIDE:P36428 ProMEX:P36428 EnsemblPlants:AT1G50200.1 GeneID:841442 KEGG:ath:AT1G50200 GeneFarm:2386 TAIR:At1g50200 InParanoid:P36428 PhylomeDB:P36428 ProtClustDB:PLN02900 ArrayExpress:P36428 Genevestigator:P36428 GermOnline:AT1G50200 Uniprot:P36428
Root	Isotig03012	91	294	-1.821	3.25E-30	No hit
Root	Isotig03013	92	293	-1.800	1.12E-29	No hit
Root	Isotig03014	98	275	-1.618	2.83E-24	No hit
Root	Isotig03015	99	274	-1.598	8.59E-24	No hit
Root	Isotig03020	11	67	-2.736	2.75E-12	TAIR|locus:2121013 - symbol:AT4G37900 "AT4G37900" species:3702 "Arabidopsis thaliana" [GO:0005575 "cellular_component" evidence=ND] [GO:0008150 "biological_process" evidence=ND] EMBL:CP002687 GenomeReviews:CT486007_GR EMBL:AL161592 EMBL:AL035538 InterPro:IPR009836 Pfam:PF07173 eggNOG:NOG117547 HSSP:P02876 IPI:IPI00547179 PIR:T05617 RefSeq:NP_195503.1 UniGene:At.43494 PRIDE:Q9SZJ2 EnsemblPlants:AT4G37900.1 GeneID:829946 KEGG:ath:AT4G37900 TAIR:At4g37900 HOGENOM:HBG318807 InParanoid:Q9SZJ2 OMA:FMTAVEF PhylomeDB:Q9SZJ2 ProtClustDB:CLSN2681427 ArrayExpress:Q9SZJ2 Genevestigator:Q9SZJ2 Uniprot:Q9SZJ2
Root	Isotig03021	11	69	-2.778	8.64E-13	TAIR|locus:2121013 - symbol:AT4G37900 "AT4G37900" species:3702 "Arabidopsis thaliana" [GO:0005575 "cellular_component" evidence=ND] [GO:0008150 "biological_process" evidence=ND] EMBL:CP002687 GenomeReviews:CT486007_GR EMBL:AL161592 EMBL:AL035538 InterPro:IPR009836 Pfam:PF07173 eggNOG:NOG117547 HSSP:P02876 IPI:IPI00547179 PIR:T05617 RefSeq:NP_195503.1 UniGene:At.43494 PRIDE:Q9SZJ2 EnsemblPlants:AT4G37900.1 GeneID:829946 KEGG:ath:AT4G37900 TAIR:At4g37900 HOGENOM:HBG318807 InParanoid:Q9SZJ2 OMA:FMTAVEF PhylomeDB:Q9SZJ2 ProtClustDB:CLSN2681427 ArrayExpress:Q9SZJ2 Genevestigator:Q9SZJ2 Uniprot:Q9SZJ2
Root	Isotig03022	8	71	-3.279	4.18E-15	TAIR|locus:2121013 - symbol:AT4G37900 "AT4G37900" species:3702 "Arabidopsis thaliana" [GO:0005575 "cellular_component" evidence=ND] [GO:0008150 "biological_process" evidence=ND] EMBL:CP002687 GenomeReviews:CT486007_GR EMBL:AL161592 EMBL:AL035538 InterPro:IPR009836 Pfam:PF07173 eggNOG:NOG117547 HSSP:P02876 IPI:IPI00547179 PIR:T05617 RefSeq:NP_195503.1 UniGene:At.43494 PRIDE:Q9SZJ2 EnsemblPlants:AT4G37900.1 GeneID:829946 KEGG:ath:AT4G37900 TAIR:At4g37900 HOGENOM:HBG318807 InParanoid:Q9SZJ2 OMA:FMTAVEF PhylomeDB:Q9SZJ2 ProtClustDB:CLSN2681427 ArrayExpress:Q9SZJ2 Genevestigator:Q9SZJ2 Uniprot:Q9SZJ2
Root	Isotig03023	8	73	-3.319	1.24E-15	TAIR|locus:2121013 - symbol:AT4G37900 "AT4G37900" species:3702 "Arabidopsis thaliana" [GO:0005575 "cellular_component" evidence=ND] [GO:0008150 "biological_process" evidence=ND] EMBL:CP002687 GenomeReviews:CT486007_GR EMBL:AL161592 EMBL:AL035538 InterPro:IPR009836 Pfam:PF07173 eggNOG:NOG117547 HSSP:P02876 IPI:IPI00547179 PIR:T05617 RefSeq:NP_195503.1 UniGene:At.43494 PRIDE:Q9SZJ2 EnsemblPlants:AT4G37900.1 GeneID:829946 KEGG:ath:AT4G37900 TAIR:At4g37900 HOGENOM:HBG318807 InParanoid:Q9SZJ2 OMA:FMTAVEF PhylomeDB:Q9SZJ2 ProtClustDB:CLSN2681427 ArrayExpress:Q9SZJ2 Genevestigator:Q9SZJ2 Uniprot:Q9SZJ2
Root	Isotig03024	726	13	5.674	1.21E-165	TAIR|locus:2103488 - symbol:SIP2 "AT3G57520" species:3702 "Arabidopsis thaliana" [GO:0004553 "hydrolase activity, hydrolyzing O-glycosyl compounds" evidence=ISS] [GO:0006979 "response to oxidative stress" evidence=IEP] [GO:0080167 "response to karrikin" evidence=IEP] [GO:0034484 "raffinose catabolic process"

Tissue	Identification	# of reads (stress)	# of reads (control)	Fold Change	p-value	Description
						evidence=IDA] [GO:0047274 "galactinol-sucrose galactosyltransferase activity" evidence=IDA] [GO:0009506 "plasmodesma" evidence=IDA] InterPro:IPR013785 GO:GO:0009506 EMBL:CP002686 GenomeReviews:BA000014_GR GO:GO:0006979 Gene3D:G3DSA:3.20.20.70 InterPro:IPR017853 SUPFAM:SSF51445 GO:GO:0080167 EMBL:AL133248 CAZy:GH36 eggNOG:NOG06986 InterPro:IPR008811 Pfam:PF05691 GO:GO:0034484 HOGENOM:HBG611539 KO:K06617 GO:GO:0047274 EMBL:AY050772 EMBL:AK226370 IPI:IPI00526258 IPI:IPI00541537 IPI:IPI00544535 PIR:T46188 RefSeq:NP_191311.1 RefSeq:NP_850715.1 UniGene:At.22207 UniGene:At.30900 ProteinModelPortal:Q94A08 STRING:Q94A08 PRIDE:Q94A08 EnsemblPlants:AT3G57520.1 GeneID:824919 KEGG:ath:AT3G57520 TAIR:At3g57520 InParanoid:Q9SCM1 OMA:QFMLLES PhylomeDB:Q94A08 ProtClustDB:PLN02219 BioCyc:MetaCyc:AT3G57520-MONOMER ArrayExpress:Q94A08 Uniprot:Q94A08
Root	Isotig03025	726	13	5.674	1.21E-165	TAIR|locus:2103488 - symbol:SIP2 "AT3G57520" species:3702 "Arabidopsis thaliana" [GO:0004553 "hydrolase activity, hydrolyzing O-glycosyl compounds" evidence=ISS] [GO:0006979 "response to oxidative stress" evidence=IEP] [GO:0080167 "response to karrikin" evidence=IEP] [GO:0034484 "raffinose catabolic process" evidence=IDA] [GO:0047274 "galactinol-sucrose galactosyltransferase activity" evidence=IDA] [GO:0009506 "plasmodesma" evidence=IDA] InterPro:IPR013785 GO:GO:0009506 EMBL:CP002686 GenomeReviews:BA000014_GR GO:GO:0006979 Gene3D:G3DSA:3.20.20.70 InterPro:IPR017853 SUPFAM:SSF51445 GO:GO:0080167 EMBL:AL133248 CAZy:GH36 eggNOG:NOG06986 InterPro:IPR008811 Pfam:PF05691 GO:GO:0034484 HOGENOM:HBG611539 KO:K06617 GO:GO:0047274 EMBL:AY050772 EMBL:AK226370 IPI:IPI00526258 IPI:IPI00541537 IPI:IPI00544535 PIR:T46188 RefSeq:NP_191311.1 RefSeq:NP_850715.1 UniGene:At.22207 UniGene:At.30900 ProteinModelPortal:Q94A08 STRING:Q94A08 PRIDE:Q94A08 EnsemblPlants:AT3G57520.1 GeneID:824919 KEGG:ath:AT3G57520 TAIR:At3g57520 InParanoid:Q9SCM1 OMA:QFMLLES PhylomeDB:Q94A08 ProtClustDB:PLN02219 BioCyc:MetaCyc:AT3G57520-MONOMER ArrayExpress:Q94A08 Uniprot:Q94A08
Root	Isotig03026	406	5	6.214	5.27E-93	TAIR|locus:2103488 - symbol:SIP2 "AT3G57520" species:3702 "Arabidopsis thaliana" [GO:0004553 "hydrolase activity, hydrolyzing O-glycosyl compounds" evidence=ISS] [GO:0006979 "response to oxidative stress" evidence=IEP] [GO:0080167 "response to karrikin" evidence=IEP] [GO:0034484 "raffinose catabolic process" evidence=IDA] [GO:0047274 "galactinol-sucrose galactosyltransferase activity" evidence=IDA] [GO:0009506 "plasmodesma" evidence=IDA] InterPro:IPR013785 GO:GO:0009506 EMBL:CP002686 GenomeReviews:BA000014_GR GO:GO:0006979 Gene3D:G3DSA:3.20.20.70 InterPro:IPR017853 SUPFAM:SSF51445 GO:GO:0080167 EMBL:AL133248 CAZy:GH36 eggNOG:NOG06986 InterPro:IPR008811 Pfam:PF05691 GO:GO:0034484 HOGENOM:HBG611539 KO:K06617 GO:GO:0047274 EMBL:AY050772 EMBL:AK226370 IPI:IPI00526258 IPI:IPI00541537 IPI:IPI00544535 PIR:T46188 RefSeq:NP_191311.1 RefSeq:NP_850715.1 UniGene:At.22207 UniGene:At.30900 ProteinModelPortal:Q94A08 STRING:Q94A08 PRIDE:Q94A08 EnsemblPlants:AT3G57520.1 GeneID:824919 KEGG:ath:AT3G57520 TAIR:At3g57520 InParanoid:Q9SCM1 OMA:QFMLLES PhylomeDB:Q94A08 ProtClustDB:PLN02219 BioCyc:MetaCyc:AT3G57520-MONOMER ArrayExpress:Q94A08 Uniprot:Q94A08
Root	Isotig03027	406	5	6.214	5.27E-93	TAIR|locus:2103488 - symbol:SIP2 "AT3G57520" species:3702 "Arabidopsis thaliana" [GO:0004553 "hydrolase activity, hydrolyzing O-glycosyl compounds" evidence=ISS] [GO:0006979 "response to oxidative stress" evidence=IEP] [GO:0080167 "response to karrikin" evidence=IEP] [GO:0034484 "raffinose catabolic process" evidence=IDA] [GO:0047274 "galactinol-sucrose galactosyltransferase activity" evidence=IDA] [GO:0009506 "plasmodesma" evidence=IDA] InterPro:IPR013785 GO:GO:0009506 EMBL:CP002686 GenomeReviews:BA000014_GR GO:GO:0006979 Gene3D:G3DSA:3.20.20.70 InterPro:IPR017853 SUPFAM:SSF51445 GO:GO:0080167 EMBL:AL133248 CAZy:GH36 eggNOG:NOG06986 InterPro:IPR008811 Pfam:PF05691 GO:GO:0034484 HOGENOM:HBG611539 KO:K06617 GO:GO:0047274 EMBL:AY050772 EMBL:AK226370 IPI:IPI00526258 IPI:IPI00541537 IPI:IPI00544535 PIR:T46188 RefSeq:NP_191311.1 RefSeq:NP_850715.1 UniGene:At.22207 UniGene:At.30900 ProteinModelPortal:Q94A08 STRING:Q94A08 PRIDE:Q94A08 EnsemblPlants:AT3G57520.1 GeneID:824919 KEGG:ath:AT3G57520 TAIR:At3g57520 InParanoid:Q9SCM1 OMA:QFMLLES PhylomeDB:Q94A08 ProtClustDB:PLN02219 BioCyc:MetaCyc:AT3G57520-MONOMER ArrayExpress:Q94A08 Uniprot:Q94A08
Root	Isotig03028	55	20	1.330	0.000171637	TAIR|locus:2036134 - symbol:AVP1 "AT1G15690" species:3702 "Arabidopsis thaliana" [GO:0009414 "response to water deprivation" evidence=IMP] [GO:0009651 "response to salt stress" evidence=IMP] [GO:0009941 "chloroplast envelope" evidence=IDA] [GO:0009678 "hydrogen-translocating pyrophosphatase activity" evidence=IDA]

Tissue	Identification	# of reads (stress)	# of reads (control)	Fold Change	p-value	Description
						[GO:0009705 "plant-type vacuole membrane" evidence=IDA] [GO:0010248 "establishment or maintenance of transmembrane electrochemical gradient" evidence=TAS] [GO:0005739 "mitochondrion" evidence=IDA] [GO:0005773 "vacuole" evidence=IDA] [GO:0009507 "chloroplast" evidence=IDA] [GO:0005886 "plasma membrane" evidence=IDA] [GO:0009926 "auxin polar transport" evidence=IGI] [GO:0010008 "endosome membrane" evidence=IDA] [GO:0000325 "plant-type vacuole" evidence=IDA] [GO:0005774 "vacuolar membrane" evidence=IDA] [GO:0016020 "membrane" evidence=IDA] [GO:0048366 "leaf development" evidence=IMP] [GO:0005829 "cytosol" evidence=RCA] [GO:0005794 "Golgi apparatus" evidence=IDA] InterPro:IPR004131 Pfam:PF03030 PIRSF:PIRSF001265 GO:GO:0016021 EMBL:CP002684 GenomeReviews:CT485782_GR GO:GO:0005739 GO:GO:0005886 GO:GO:0009926 GO:GO:0009941 GO:GO:0009651 GO:GO:0009414 GO:GO:0010008 GO:GO:0009705 GO:GO:0048366 GO:GO:0015992 EMBL:AC034256 UniGene:At.21942 UniGene:At.66944 EMBL:M81892 EMBL:AB015138 EMBL:AY065016 EMBL:AY078953 EMBL:BT002481 EMBL:Z17694 EMBL:Z17695 EMBL:AK221989 IPI:IPI00521876 PIR:A38230 RefSeq:NP_173021.1 UniGene:At.67102 UniGene:At.74973 STRING:P31414 TCDB:3.A.10.1.1 PRIDE:P31414 EnsemblPlants:AT1G15690.1 GeneID:838138 KEGG:ath:AT1G15690 GeneFarm:5159 TAIR:At1g15690 eggNOG:COG3808 InParanoid:P31414 KO:K01507 OMA:MAITSIV PhylomeDB:P31414 ProtClustDB:PLN02255 BRENDA:3.6.1.1 ArrayExpress:P31414 Genevestigator:P31414 GermOnline:AT1G15690 GO:GO:0009678 GO:GO:0004427 GO:GO:0010248 TIGRFAMs:TIGR01104 Uniprot:P31414
Root	Isotig03029	55	20	1.330	0.000171637	TAIR|locus:2036134 - symbol:AVP1 "AT1G15690" species:3702 "Arabidopsis thaliana" [GO:0009414 "response to water deprivation" evidence=IMP] [GO:0009651 "response to salt stress" evidence=IMP] [GO:0009941 "chloroplast envelope" evidence=IDA] [GO:0009678 "hydrogen-translocating pyrophosphatase activity" evidence=IDA] [GO:0009705 "plant-type vacuole membrane" evidence=IDA] [GO:0010248 "establishment or maintenance of transmembrane electrochemical gradient" evidence=TAS] [GO:0005739 "mitochondrion" evidence=IDA] [GO:0005773 "vacuole" evidence=IDA] [GO:0009507 "chloroplast" evidence=IDA] [GO:0005886 "plasma membrane" evidence=IDA] [GO:0009926 "auxin polar transport" evidence=IGI] [GO:0010008 "endosome membrane" evidence=IDA] [GO:0000325 "plant-type vacuole" evidence=IDA] [GO:0005774 "vacuolar membrane" evidence=IDA] [GO:0016020 "membrane" evidence=IDA] [GO:0048366 "leaf development" evidence=IMP] [GO:0005829 "cytosol" evidence=RCA] [GO:0005794 "Golgi apparatus" evidence=IDA] InterPro:IPR004131 Pfam:PF03030 PIRSF:PIRSF001265 GO:GO:0016021 EMBL:CP002684 GenomeReviews:CT485782_GR GO:GO:0005739 GO:GO:0005886 GO:GO:0009926 GO:GO:0009941 GO:GO:0009651 GO:GO:0009414 GO:GO:0010008 GO:GO:0009705 GO:GO:0048366 GO:GO:0015992 EMBL:AC034256 UniGene:At.21942 UniGene:At.66944 EMBL:M81892 EMBL:AB015138 EMBL:AY065016 EMBL:AY078953 EMBL:BT002481 EMBL:Z17694 EMBL:Z17695 EMBL:AK221989 IPI:IPI00521876 PIR:A38230 RefSeq:NP_173021.1 UniGene:At.67102 UniGene:At.74973 STRING:P31414 TCDB:3.A.10.1.1 PRIDE:P31414 EnsemblPlants:AT1G15690.1 GeneID:838138 KEGG:ath:AT1G15690 GeneFarm:5159 TAIR:At1g15690 eggNOG:COG3808 InParanoid:P31414 KO:K01507 OMA:MAITSIV PhylomeDB:P31414 ProtClustDB:PLN02255 BRENDA:3.6.1.1 ArrayExpress:P31414 Genevestigator:P31414 GermOnline:AT1G15690 GO:GO:0009678 GO:GO:0004427 GO:GO:0010248 TIGRFAMs:TIGR01104 Uniprot:P31414
Root	Isotig03044	6	29	-2.402	1.89E-05	TAIR|locus:2143320 - symbol:AT5G27920 "AT5G27920" species:3702 "Arabidopsis thaliana" [GO:0004842 "ubiquitin-protein ligase activity" evidence=ISS] [GO:0008150 "biological_process" evidence=ND] EMBL:CP002688 GenomeReviews:BA000015_GR InterPro:IPR006553 SMART:SM00367 HOGENOM:HBG316674 EMBL:BT005940 EMBL:AK117239 IPI:IPI00538711 RefSeq:NP_568502.1 UniGene:At.30778 ProteinModelPortal:Q8GZ31 SMR:Q8GZ31 PRIDE:Q8GZ31 EnsemblPlants:AT5G27920.1 GeneID:832858 KEGG:ath:AT5G27920 TAIR:At5g27920 InParanoid:Q8GZ31 OMA:GFWALAY PhylomeDB:Q8GZ31 ProtClustDB:CLSN2689880 Genevestigator:Q8GZ31 Uniprot:Q8GZ31
Root	Isotig03045	6	29	-2.402	1.89E-05	TAIR|locus:2143320 - symbol:AT5G27920 "AT5G27920" species:3702 "Arabidopsis thaliana" [GO:0004842 "ubiquitin-protein ligase activity" evidence=ISS] [GO:0008150 "biological_process" evidence=ND] EMBL:CP002688 GenomeReviews:BA000015_GR InterPro:IPR006553 SMART:SM00367 HOGENOM:HBG316674 EMBL:BT005940 EMBL:AK117239 IPI:IPI00538711 RefSeq:NP_568502.1 UniGene:At.30778 ProteinModelPortal:Q8GZ31 SMR:Q8GZ31 PRIDE:Q8GZ31 EnsemblPlants:AT5G27920.1 GeneID:832858 KEGG:ath:AT5G27920 TAIR:At5g27920 InParanoid:Q8GZ31 OMA:GFWALAY PhylomeDB:Q8GZ31 ProtClustDB:CLSN2689880 Genevestigator:Q8GZ31 Uniprot:Q8GZ31

Tissue	Identification	# of reads (stress)	# of reads (control)	Fold Change	p-value	Description
Root	Isotig03046	6	29	-2.402	1.89E-05	TAIR|locus:2143320 - symbol:AT5G27920 "AT5G27920" species:3702 "Arabidopsis thaliana" [GO:0004842 "ubiquitin-protein ligase activity" evidence=ISS] [GO:0008150 "biological_process" evidence=ND] EMBL:CP002688 GenomeReviews:BA000015_GR InterPro:IPR006553 SMART:SM00367 HOGENOM:HBG316674 EMBL:BT005940 EMBL:AK117239 IPI:IPI00538711 RefSeq:NP_568502.1 UniGene:At.30778 ProteinModelPortal:Q8GZ31 SMR:Q8GZ31 PRIDE:Q8GZ31 EnsemblPlants:AT5G27920.1 GeneID:832858 KEGG:ath:AT5G27920 TAIR:At5g27920 InParanoid:Q8GZ31 OMA:GFWALAY PhylomeDB:Q8GZ31 ProtClustDB:CLSN2689880 Genevestigator:Q8GZ31 Uniprot:Q8GZ31
Root	Isotig03047	6	29	-2.402	1.89E-05	TAIR|locus:2143320 - symbol:AT5G27920 "AT5G27920" species:3702 "Arabidopsis thaliana" [GO:0004842 "ubiquitin-protein ligase activity" evidence=ISS] [GO:0008150 "biological_process" evidence=ND] EMBL:CP002688 GenomeReviews:BA000015_GR InterPro:IPR006553 SMART:SM00367 HOGENOM:HBG316674 EMBL:BT005940 EMBL:AK117239 IPI:IPI00538711 RefSeq:NP_568502.1 UniGene:At.30778 ProteinModelPortal:Q8GZ31 SMR:Q8GZ31 PRIDE:Q8GZ31 EnsemblPlants:AT5G27920.1 GeneID:832858 KEGG:ath:AT5G27920 TAIR:At5g27920 InParanoid:Q8GZ31 OMA:GFWALAY PhylomeDB:Q8GZ31 ProtClustDB:CLSN2689880 Genevestigator:Q8GZ31 Uniprot:Q8GZ31
Root	Isotig03084	2	16	-3.129	0.000262867	TAIR|locus:2125642 - symbol:AT4G32760 "AT4G32760" species:3702 "Arabidopsis thaliana" [GO:0005795 "Golgi stack" evidence=ISS] [GO:0006886 "intracellular protein transport" evidence=IEA;ISS] [GO:0006891 "intra-Golgi vesicle-mediated transport" evidence=ISS] [GO:0008565 "protein transporter activity" evidence=ISS] [GO:0005886 "plasma membrane" evidence=IDA] [GO:0005829 "cytosol" evidence=RCA] InterPro:IPR002014 InterPro:IPR004152 Pfam:PF00790 Pfam:PF03127 PROSITE:PS50179 PROSITE:PS50909 GO:GO:0005886 EMBL:CP002687 GenomeReviews:CT486007_GR GO:GO:0006886 GO:GO:0005622 InterPro:IPR008942 Gene3D:G3DSA:1.25.40.90 SUPFAM:SSF48464 InterPro:IPR018205 SMART:SM00288 HSSP:O60784 HOGENOM:HBG597182 EMBL:AY120724 EMBL:BT000033 IPI:IPI00535455 RefSeq:NP_195002.2 UniGene:At.46217 UniGene:At.74836 ProteinModelPortal:Q8L860 SMR:Q8L860 STRING:Q8L860 PRIDE:Q8L860 EnsemblPlants:AT4G32760.1 GeneID:829412 KEGG:ath:AT4G32760 TAIR:At4g32760 InParanoid:Q8L860 OMA:VNNNSPY PhylomeDB:Q8L860 ProtClustDB:CLSN2681621 Genevestigator:Q8L860 Uniprot:Q8L860
Root	Isotig03085	2	16	-3.129	0.000262867	TAIR|locus:2125642 - symbol:AT4G32760 "AT4G32760" species:3702 "Arabidopsis thaliana" [GO:0005795 "Golgi stack" evidence=ISS] [GO:0006886 "intracellular protein transport" evidence=IEA;ISS] [GO:0006891 "intra-Golgi vesicle-mediated transport" evidence=ISS] [GO:0008565 "protein transporter activity" evidence=ISS] [GO:0005886 "plasma membrane" evidence=IDA] [GO:0005829 "cytosol" evidence=RCA] InterPro:IPR002014 InterPro:IPR004152 Pfam:PF00790 Pfam:PF03127 PROSITE:PS50179 PROSITE:PS50909 GO:GO:0005886 EMBL:CP002687 GenomeReviews:CT486007_GR GO:GO:0006886 GO:GO:0005622 InterPro:IPR008942 Gene3D:G3DSA:1.25.40.90 SUPFAM:SSF48464 InterPro:IPR018205 SMART:SM00288 HSSP:O60784 HOGENOM:HBG597182 EMBL:AY120724 EMBL:BT000033 IPI:IPI00535455 RefSeq:NP_195002.2 UniGene:At.46217 UniGene:At.74836 ProteinModelPortal:Q8L860 SMR:Q8L860 STRING:Q8L860 PRIDE:Q8L860 EnsemblPlants:AT4G32760.1 GeneID:829412 KEGG:ath:AT4G32760 TAIR:At4g32760 InParanoid:Q8L860 OMA:VNNNSPY PhylomeDB:Q8L860 ProtClustDB:CLSN2681621 Genevestigator:Q8L860 Uniprot:Q8L860
Root	Isotig03086	2	16	-3.129	0.000262867	TAIR|locus:2125642 - symbol:AT4G32760 "AT4G32760" species:3702 "Arabidopsis thaliana" [GO:0005795 "Golgi stack" evidence=ISS] [GO:0006886 "intracellular protein transport" evidence=IEA;ISS] [GO:0006891 "intra-Golgi vesicle-mediated transport" evidence=ISS] [GO:0008565 "protein transporter activity" evidence=ISS] [GO:0005886 "plasma membrane" evidence=IDA] [GO:0005829 "cytosol" evidence=RCA] InterPro:IPR002014 InterPro:IPR004152 Pfam:PF00790 Pfam:PF03127 PROSITE:PS50179 PROSITE:PS50909 GO:GO:0005886 EMBL:CP002687 GenomeReviews:CT486007_GR GO:GO:0006886 GO:GO:0005622 InterPro:IPR008942 Gene3D:G3DSA:1.25.40.90 SUPFAM:SSF48464 InterPro:IPR018205 SMART:SM00288 HSSP:O60784 HOGENOM:HBG597182 EMBL:AY120724 EMBL:BT000033 IPI:IPI00535455 RefSeq:NP_195002.2 UniGene:At.46217 UniGene:At.74836 ProteinModelPortal:Q8L860 SMR:Q8L860 STRING:Q8L860 PRIDE:Q8L860 EnsemblPlants:AT4G32760.1 GeneID:829412 KEGG:ath:AT4G32760 TAIR:At4g32760 InParanoid:Q8L860 OMA:VNNNSPY PhylomeDB:Q8L860 ProtClustDB:CLSN2681621 Genevestigator:Q8L860 Uniprot:Q8L860
Root	Isotig03087	2	16	-3.129	0.000262867	TAIR|locus:2125642 - symbol:AT4G32760 "AT4G32760" species:3702 "Arabidopsis thaliana" [GO:0005795 "Golgi stack" evidence=ISS] [GO:0006886 "intracellular protein transport" evidence=IEA;ISS] [GO:0006891 "intra-Golgi vesicle-mediated transport" evidence=ISS] [GO:0008565 "protein transporter activity" evidence=ISS] [GO:0005886 "plasma membrane" evidence=IDA] [GO:0005829 "cytosol" evidence=RCA] InterPro:IPR002014 InterPro:IPR004152 Pfam:PF00790 Pfam:PF03127 PROSITE:PS50179 PROSITE:PS50909 GO:GO:0005886

Tissue	Identification	# of reads (stress)	# of reads (control)	Fold Change	p-value	Description
						EMBL:CP002687 GenomeReviews:CT486007_GR GO:GO:0006886 GO:GO:0005622 InterPro:IPR008942 Gene3D:G3DSA:1.25.40.90 SUPFAM:SSF48464 InterPro:IPR018205 SMART:SM00288 HSSP:O60784 HOGENOM:HBG597182 EMBL:AY120724 EMBL:BT000033 IPI:IPI00535455 RefSeq:NP_195002.2 UniGene:At.46217 UniGene:At.74836 ProteinModelPortal:Q8L860 SMR:Q8L860 STRING:Q8L860 PRIDE:Q8L860 EnsemblPlants:AT4G32760.1 GeneID:829412 KEGG:ath:AT4G32760 TAIR:At4g32760 InParanoid:Q8L860 OMA:VNNNSPY PhylomeDB:Q8L860 ProtClustDB:CLSN2681621 Genevestigator:Q8L860 Uniprot:Q8L860
Root	Isotig03088	2	19	-3.377	3.93E-05	TAIR|locus:2204858 - symbol:AT1G02260 "AT1G02260" species:3702 "Arabidopsis thaliana" [GO:0005215 "transporter activity" evidence=ISS] [GO:0015137 "citrate transmembrane transporter activity" evidence=IEA] [GO:0015746 "citrate transport" evidence=IEA] [GO:0016021 "integral to membrane" evidence=IEA;ISS] [GO:0055085 "transmembrane transport" evidence=IEA] InterPro:IPR004680 Pfam:PF03600 GO:GO:0016021 EMBL:CP002684 GenomeReviews:CT485782_GR eggNOG:COG1055 OMA:SANVICA GO:GO:0015137 EMBL:U89959 HOGENOM:HBG727257 EMBL:BT023463 IPI:IPI00519219 RefSeq:NP_171728.2 UniGene:At.48101 STRING:O81915 PRIDE:O81915 EnsemblPlants:AT1G02260.1 GeneID:839344 KEGG:ath:AT1G02260 TAIR:At1g02260 InParanoid:O81915 PhylomeDB:O81915 ProtClustDB:CLSN2918462 Genevestigator:O81915 Uniprot:O81915
Root	Isotig03089	2	19	-3.377	3.93E-05	TAIR|locus:2204858 - symbol:AT1G02260 "AT1G02260" species:3702 "Arabidopsis thaliana" [GO:0005215 "transporter activity" evidence=ISS] [GO:0015137 "citrate transmembrane transporter activity" evidence=IEA] [GO:0015746 "citrate transport" evidence=IEA] [GO:0016021 "integral to membrane" evidence=IEA;ISS] [GO:0055085 "transmembrane transport" evidence=IEA] InterPro:IPR004680 Pfam:PF03600 GO:GO:0016021 EMBL:CP002684 GenomeReviews:CT485782_GR eggNOG:COG1055 OMA:SANVICA GO:GO:0015137 EMBL:U89959 HOGENOM:HBG727257 EMBL:BT023463 IPI:IPI00519219 RefSeq:NP_171728.2 UniGene:At.48101 STRING:O81915 PRIDE:O81915 EnsemblPlants:AT1G02260.1 GeneID:839344 KEGG:ath:AT1G02260 TAIR:At1g02260 InParanoid:O81915 PhylomeDB:O81915 ProtClustDB:CLSN2918462 Genevestigator:O81915 Uniprot:O81915
Root	Isotig03090	2	19	-3.377	3.93E-05	TAIR|locus:2204858 - symbol:AT1G02260 "AT1G02260" species:3702 "Arabidopsis thaliana" [GO:0005215 "transporter activity" evidence=ISS] [GO:0015137 "citrate transmembrane transporter activity" evidence=IEA] [GO:0015746 "citrate transport" evidence=IEA] [GO:0016021 "integral to membrane" evidence=IEA;ISS] [GO:0055085 "transmembrane transport" evidence=IEA] InterPro:IPR004680 Pfam:PF03600 GO:GO:0016021 EMBL:CP002684 GenomeReviews:CT485782_GR eggNOG:COG1055 OMA:SANVICA GO:GO:0015137 EMBL:U89959 HOGENOM:HBG727257 EMBL:BT023463 IPI:IPI00519219 RefSeq:NP_171728.2 UniGene:At.48101 STRING:O81915 PRIDE:O81915 EnsemblPlants:AT1G02260.1 GeneID:839344 KEGG:ath:AT1G02260 TAIR:At1g02260 InParanoid:O81915 PhylomeDB:O81915 ProtClustDB:CLSN2918462 Genevestigator:O81915 Uniprot:O81915
Root	Isotig03091	2	19	-3.377	3.93E-05	TAIR|locus:2204858 - symbol:AT1G02260 "AT1G02260" species:3702 "Arabidopsis thaliana" [GO:0005215 "transporter activity" evidence=ISS] [GO:0015137 "citrate transmembrane transporter activity" evidence=IEA] [GO:0015746 "citrate transport" evidence=IEA] [GO:0016021 "integral to membrane" evidence=IEA;ISS] [GO:0055085 "transmembrane transport" evidence=IEA] InterPro:IPR004680 Pfam:PF03600 GO:GO:0016021 EMBL:CP002684 GenomeReviews:CT485782_GR eggNOG:COG1055 OMA:SANVICA GO:GO:0015137 EMBL:U89959 HOGENOM:HBG727257 EMBL:BT023463 IPI:IPI00519219 RefSeq:NP_171728.2 UniGene:At.48101 STRING:O81915 PRIDE:O81915 EnsemblPlants:AT1G02260.1 GeneID:839344 KEGG:ath:AT1G02260 TAIR:At1g02260 InParanoid:O81915 PhylomeDB:O81915 ProtClustDB:CLSN2918462 Genevestigator:O81915 Uniprot:O81915
Root	Isotig03100	2	18	-3.299	7.42E-05	TAIR|locus:2117617 - symbol:PIRL4 "AT4G35470" species:3702 "Arabidopsis thaliana" [GO:0008150 "biological_process" evidence=ND] [GO:0005886 "plasma membrane" evidence=IDA] Pfam:PF00560 InterPro:IPR001611 PROSITE:PS51450 GO:GO:0005886 EMBL:CP002687 GenomeReviews:CT486007_GR eggNOG:COG4886 InterPro:IPR003591 SMART:SM00369 EMBL:AL117188 EMBL:AL161587 HSSP:P07359 ProtClustDB:CLSN2683891 EMBL:AY072324 EMBL:AY128730 EMBL:AY849574 IPI:IPI00541009 PIR:T41744 RefSeq:NP_195272.1 UniGene:At.22486 ProteinModelPortal:Q9SVW8 SMR:Q9SVW8 IntAct:Q9SVW8 STRING:Q9SVW8 PRIDE:Q9SVW8 EnsemblPlants:AT4G35470.1 GeneID:829699 KEGG:ath:AT4G35470 TAIR:At4g35470 HOGENOM:HBG601308 InParanoid:Q9SVW8 OMA:EWLPDSI PhylomeDB:Q9SVW8 Genevestigator:Q9SVW8 Uniprot:Q9SVW8

Tissue	Identification	# of reads (stress)	# of reads (control)	Fold Change	p-value	Description
Root	Isotig03101	2	18	-3.299	7.42E-05	TAIR|locus:2117617 - symbol:PIRL4 "AT4G35470" species:3702 "Arabidopsis thaliana" [GO:0008150 "biological_process" evidence=ND] [GO:0005886 "plasma membrane" evidence=IDA] Pfam:PF00560 InterPro:IPR001611 PROSITE:PS51450 GO:GO:0005886 EMBL:CP002687 GenomeReviews:CT486007_GR eggNOG:COG4886 InterPro:IPR003591 SMART:SM00369 EMBL:AL117188 EMBL:AL161587 HSSP:P07359 ProtClustDB:CLSN2683891 EMBL:AY072324 EMBL:AY128730 EMBL:AY849574 IPI:IPI00541009 PIR:T41744 RefSeq:NP_195272.1 UniGene:At.22486 ProteinModelPortal:Q9SVW8 SMR:Q9SVW8 IntAct:Q9SVW8 STRING:Q9SVW8 PRIDE:Q9SVW8 EnsemblPlants:AT4G35470.1 GeneID:829699 KEGG:ath:AT4G35470 TAIR:At4g35470 HOGENOM:HBG601308 InParanoid:Q9SVW8 OMA:EWLPDSI PhylomeDB:Q9SVW8 Genevestigator:Q9SVW8 Uniprot:Q9SVW8
Root	Isotig03102	2	18	-3.299	7.42E-05	TAIR|locus:2117617 - symbol:PIRL4 "AT4G35470" species:3702 "Arabidopsis thaliana" [GO:0008150 "biological_process" evidence=ND] [GO:0005886 "plasma membrane" evidence=IDA] Pfam:PF00560 InterPro:IPR001611 PROSITE:PS51450 GO:GO:0005886 EMBL:CP002687 GenomeReviews:CT486007_GR eggNOG:COG4886 InterPro:IPR003591 SMART:SM00369 EMBL:AL117188 EMBL:AL161587 HSSP:P07359 ProtClustDB:CLSN2683891 EMBL:AY072324 EMBL:AY128730 EMBL:AY849574 IPI:IPI00541009 PIR:T41744 RefSeq:NP_195272.1 UniGene:At.22486 ProteinModelPortal:Q9SVW8 SMR:Q9SVW8 IntAct:Q9SVW8 STRING:Q9SVW8 PRIDE:Q9SVW8 EnsemblPlants:AT4G35470.1 GeneID:829699 KEGG:ath:AT4G35470 TAIR:At4g35470 HOGENOM:HBG601308 InParanoid:Q9SVW8 OMA:EWLPDSI PhylomeDB:Q9SVW8 Genevestigator:Q9SVW8 Uniprot:Q9SVW8
Root	Isotig03103	2	18	-3.299	7.42E-05	TAIR|locus:2117617 - symbol:PIRL4 "AT4G35470" species:3702 "Arabidopsis thaliana" [GO:0008150 "biological_process" evidence=ND] [GO:0005886 "plasma membrane" evidence=IDA] Pfam:PF00560 InterPro:IPR001611 PROSITE:PS51450 GO:GO:0005886 EMBL:CP002687 GenomeReviews:CT486007_GR eggNOG:COG4886 InterPro:IPR003591 SMART:SM00369 EMBL:AL117188 EMBL:AL161587 HSSP:P07359 ProtClustDB:CLSN2683891 EMBL:AY072324 EMBL:AY128730 EMBL:AY849574 IPI:IPI00541009 PIR:T41744 RefSeq:NP_195272.1 UniGene:At.22486 ProteinModelPortal:Q9SVW8 SMR:Q9SVW8 IntAct:Q9SVW8 STRING:Q9SVW8 PRIDE:Q9SVW8 EnsemblPlants:AT4G35470.1 GeneID:829699 KEGG:ath:AT4G35470 TAIR:At4g35470 HOGENOM:HBG601308 InParanoid:Q9SVW8 OMA:EWLPDSI PhylomeDB:Q9SVW8 Genevestigator:Q9SVW8 Uniprot:Q9SVW8
Root	Isotig03145	5	36	-2.977	8.93E-08	TAIR|locus:2145944 - symbol:CUE1 "CAB UNDEREXPRESSED 1" species:3702 "Arabidopsis thaliana" [GO:0006810 "transport" evidence=IEA] [GO:0015297 "antiporter activity" evidence=ISS] [GO:0009528 "plastid inner membrane" evidence=TAS] [GO:0009941 "chloroplast envelope" evidence=IDA] [GO:0015120 "phosphoglycerate transmembrane transporter activity" evidence=IDA] [GO:0015121 "phosphoenolpyruvate:phosphate antiporter activity" evidence=IDA] [GO:0015713 "phosphoglycerate transport" evidence=IDA] [GO:0015714 "phosphoenolpyruvate transport" evidence=IDA] [GO:0009507 "chloroplast" evidence=IDA] [GO:0005739 "mitochondrion" evidence=IDA] [GO:0009536 "plastid" evidence=IDA] InterPro:IPR000620 InterPro:IPR004696 Pfam:PF00892 Pfam:PF03151 GO:GO:0016021 GO:GO:0005739 EMBL:CP002688 GenomeReviews:BA000015_GR GO:GO:0031969 GO:GO:0008643 EMBL:AC051625 InterPro:IPR004853 eggNOG:NOG312636 KO:K15283 GO:GO:0015120 TIGRFAMs:TIGR00817 EMBL:U66321 EMBL:AF209210 EMBL:AY080788 EMBL:AY133809 EMBL:AY086100 IPI:IPI00534885 RefSeq:NP_198317.1 UniGene:At.14730 STRING:Q8RXN3 PRIDE:Q8RXN3 EnsemblPlants:AT5G33320.1 GeneID:833308 KEGG:ath:AT5G33320 TAIR:At5g33320 InParanoid:Q8RXN3 OMA:HILALCA PhylomeDB:Q8RXN3 ProtClustDB:CLSN2720506 Genevestigator:Q8RXN3 GO:GO:0009528 GO:GO:0015121 Uniprot:Q8RXN3
Root	Isotig03156	7	36	-2.492	1.13E-06	TAIR|locus:2064965 - symbol:EULS3 "AT2G39050" species:3702 "Arabidopsis thaliana" [GO:0008150 "biological_process" evidence=ND] [GO:0005634 "nucleus" evidence=IDA] [GO:0005737 "cytoplasm" evidence=IDA] [GO:0030246 "carbohydrate binding" evidence=IDA] GO:GO:0005634 GO:GO:0005737 EMBL:CP002685 GenomeReviews:CT485783_GR InterPro:IPR000772 SUPFAM:SSF50370 PROSITE:PS50231 GO:GO:0030246 EMBL:AC005770 Pfam:PF14200 EMBL:AF411801 EMBL:AY093795 EMBL:AY087909 IPI:IPI00536641 PIR:E84812 RefSeq:NP_565899.1 UniGene:At.22599 ProteinModelPortal:Q945P1 SMR:Q945P1 PRIDE:Q945P1 EnsemblPlants:AT2G39050.1 GeneID:818491 KEGG:ath:AT2G39050 TAIR:At2g39050 eggNOG:NOG254161 HOGENOM:HBG750017 InParanoid:Q945P1 OMA:GYRTIRM PhylomeDB:Q945P1 ProtClustDB:CLSN2681741 ArrayExpress:Q945P1 Genevestigator:Q945P1 Uniprot:Q945P1
Root	Isotig03157	7	36	-2.492	1.13E-06	TAIR|locus:2064965 - symbol:EULS3 "AT2G39050" species:3702 "Arabidopsis thaliana" [GO:0008150 "biological_process" evidence=ND] [GO:0005634 "nucleus" evidence=IDA] [GO:0005737 "cytoplasm"

Tissue	Identification	# of reads (stress)	# of reads (control)	Fold Change	p-value	Description
						evidence=IDA] [GO:0030246 "carbohydrate binding" evidence=IDA] GO:GO:0005634 GO:GO:0005737 EMBL:CP002685 GenomeReviews:CT485783_GR InterPro:IPR000772 SUPFAM:SSF50370 PROSITE:PS50231 GO:GO:0030246 EMBL:AC005770 Pfam:PF14200 EMBL:AF411801 EMBL:AY093795 EMBL:AY087909 IPI:IPI00536641 PIR:E84812 RefSeq:NP_565899.1 UniGene:At.22599 ProteinModelPortal:Q945P1 SMR:Q945P1 PRIDE:Q945P1 EnsemblPlants:AT2G39050.1 GeneID:818491 KEGG:ath:AT2G39050 TAIR:At2g39050 eggNOG:NOG254161 HOGENOM:HBG750017 InParanoid:Q945P1 OMA:GYRTIRM PhylomeDB:Q945P1 ProtClustDB:CLSN2681741 ArrayExpress:Q945P1 Genevestigator:Q945P1 Uniprot:Q945P1
Root	Isotig03158	7	36	-2.492	1.13E-06	TAIR|locus:2064965 - symbol:EULS3 "AT2G39050" species:3702 "Arabidopsis thaliana" [GO:0008150 "biological_process" evidence=ND] [GO:0005634 "nucleus" evidence=IDA] [GO:0005737 "cytoplasm" evidence=IDA] [GO:0030246 "carbohydrate binding" evidence=IDA] GO:GO:0005634 GO:GO:0005737 EMBL:CP002685 GenomeReviews:CT485783_GR InterPro:IPR000772 SUPFAM:SSF50370 PROSITE:PS50231 GO:GO:0030246 EMBL:AC005770 Pfam:PF14200 EMBL:AF411801 EMBL:AY093795 EMBL:AY087909 IPI:IPI00536641 PIR:E84812 RefSeq:NP_565899.1 UniGene:At.22599 ProteinModelPortal:Q945P1 SMR:Q945P1 PRIDE:Q945P1 EnsemblPlants:AT2G39050.1 GeneID:818491 KEGG:ath:AT2G39050 TAIR:At2g39050 eggNOG:NOG254161 HOGENOM:HBG750017 InParanoid:Q945P1 OMA:GYRTIRM PhylomeDB:Q945P1 ProtClustDB:CLSN2681741 ArrayExpress:Q945P1 Genevestigator:Q945P1 Uniprot:Q945P1
Root	Isotig03159	7	36	-2.492	1.13E-06	TAIR|locus:2064965 - symbol:EULS3 "AT2G39050" species:3702 "Arabidopsis thaliana" [GO:0008150 "biological_process" evidence=ND] [GO:0005634 "nucleus" evidence=IDA] [GO:0005737 "cytoplasm" evidence=IDA] [GO:0030246 "carbohydrate binding" evidence=IDA] GO:GO:0005634 GO:GO:0005737 EMBL:CP002685 GenomeReviews:CT485783_GR InterPro:IPR000772 SUPFAM:SSF50370 PROSITE:PS50231 GO:GO:0030246 EMBL:AC005770 Pfam:PF14200 EMBL:AF411801 EMBL:AY093795 EMBL:AY087909 IPI:IPI00536641 PIR:E84812 RefSeq:NP_565899.1 UniGene:At.22599 ProteinModelPortal:Q945P1 SMR:Q945P1 PRIDE:Q945P1 EnsemblPlants:AT2G39050.1 GeneID:818491 KEGG:ath:AT2G39050 TAIR:At2g39050 eggNOG:NOG254161 HOGENOM:HBG750017 InParanoid:Q945P1 OMA:GYRTIRM PhylomeDB:Q945P1 ProtClustDB:CLSN2681741 ArrayExpress:Q945P1 Genevestigator:Q945P1 Uniprot:Q945P1
Root	Isotig03162	14	0	4.678	0.000190242	TAIR|locus:2117607 - symbol:AKR2 "AT4G35450" species:3702 "Arabidopsis thaliana" [GO:0009816 "defense response to bacterium, incompatible interaction" evidence=IMP] [GO:0046686 "response to cadmium ion" evidence=IEP] [GO:0006457 "protein folding" evidence=IDA] [GO:0031359 "integral to chloroplast outer membrane" evidence=IDA] [GO:0045036 "protein targeting to chloroplast" evidence=IMP;IDA] [GO:0005634 "nucleus" evidence=IDA] [GO:0005737 "cytoplasm" evidence=IDA] [GO:0009791 "post-embryonic development" evidence=IMP] [GO:0010228 "vegetative to reproductive phase transition of meristem" evidence=IMP] InterPro:IPR002110 PROSITE:PS50088 SMART:SM00248 EMBL:CP002687 GenomeReviews:CT486007_GR GO:GO:0005634 GO:GO:0046686 GO:GO:0006457 eggNOG:COG0666 InterPro:IPR020683 Gene3D:G3DSA:1.25.40.20 Pfam:PF12796 SUPFAM:SSF48403 PROSITE:PS50297 GO:GO:0010228 GO:GO:0009816 EMBL:AF034387 EMBL:U70425 EMBL:AL117188 EMBL:AL161587 EMBL:AF386982 EMBL:AY081477 EMBL:AY087377 IPI:IPI00546461 PIR:T41742 RefSeq:NP_195270.1 RefSeq:NP_849497.1 RefSeq:NP_849498.1 UniGene:At.24072 ProteinModelPortal:Q9SAR5 SMR:Q9SAR5 STRING:Q9SAR5 PRIDE:Q9SAR5 EnsemblPlants:AT4G35450.1 EnsemblPlants:AT4G35450.2 EnsemblPlants:AT4G35450.3 GeneID:829697 KEGG:ath:AT4G35450 GeneFarm:4730 TAIR:At4g35450 HOGENOM:HBG610978 InParanoid:Q9SAR5 OMA:PSIREMA PhylomeDB:Q9SAR5 ProtClustDB:CLSN2683889 ArrayExpress:Q9SAR5 Genevestigator:Q9SAR5 GO:GO:0031359 GO:GO:0045036 Uniprot:Q9SAR5
Root	Isotig03163	14	0	4.678	0.000190242	TAIR|locus:2117607 - symbol:AKR2 "AT4G35450" species:3702 "Arabidopsis thaliana" [GO:0009816 "defense response to bacterium, incompatible interaction" evidence=IMP] [GO:0046686 "response to cadmium ion" evidence=IEP] [GO:0006457 "protein folding" evidence=IDA] [GO:0031359 "integral to chloroplast outer membrane" evidence=IDA] [GO:0045036 "protein targeting to chloroplast" evidence=IMP;IDA] [GO:0005634 "nucleus" evidence=IDA] [GO:0005737 "cytoplasm" evidence=IDA] [GO:0009791 "post-embryonic development" evidence=IMP] [GO:0010228 "vegetative to reproductive phase transition of meristem" evidence=IMP] InterPro:IPR002110 PROSITE:PS50088 SMART:SM00248 EMBL:CP002687 GenomeReviews:CT486007_GR GO:GO:0005634 GO:GO:0046686 GO:GO:0006457 eggNOG:COG0666 InterPro:IPR020683 Gene3D:G3DSA:1.25.40.20 Pfam:PF12796 SUPFAM:SSF48403 PROSITE:PS50297 GO:GO:0010228 GO:GO:0009816 EMBL:AF034387 EMBL:U70425 EMBL:AL117188 EMBL:AL161587 EMBL:AF386982 EMBL:AY081477 EMBL:AY087377 IPI:IPI00546461 PIR:T41742 RefSeq:NP_195270.1 RefSeq:NP_849497.1

Tissue	Identification	# of reads (stress)	# of reads (control)	Fold Change	p-value	Description
						RefSeq:NP_849498.1 UniGene:At.24072 ProteinModelPortal:Q9SAR5 SMR:Q9SAR5 STRING:Q9SAR5 PRIDE:Q9SAR5 EnsemblPlants:AT4G35450.1 EnsemblPlants:AT4G35450.2 EnsemblPlants:AT4G35450.3 GeneID:829697 KEGG:ath:AT4G35450 GeneFarm:4730 TAIR:At4g35450 HOGENOM:HBG610978 InParanoid:Q9SAR5 OMA:PSIREMA PhylomeDB:Q9SAR5 ProtClustDB:CLSN2683889 ArrayExpress:Q9SAR5 Genevestigator:Q9SAR5 GO:GO:0031359 GO:GO:0045036 Uniprot:Q9SAR5
Root	Isotig03188	5	20	-2.129	0.000961902	UNIPROTKB|G4MVS3 - symbol:MGG_01790 "Acyl-CoA desaturase" species:242507 "Magnaporthe oryzae 70-15" [GO:0043581 "mycelium development" evidence=IEP] InterPro:IPR001199 InterPro:IPR001522 InterPro:IPR005804 InterPro:IPR009160 InterPro:IPR015876 InterPro:IPR018506 Pfam:PF00173 Pfam:PF00487 PIRSF:PIRSF000345 PRINTS:PR00075 PROSITE:PS00191 PROSITE:PS00476 PROSITE:PS50255 GO:GO:0005783 GO:GO:0016021 GO:GO:0006633 GO:GO:0020037 KO:K00507 GO:GO:0004768 Gene3D:G3DSA:3.10.120.10 SUPFAM:SSF55856 EMBL:CM001232 RefSeq:XP_363864.1 UniGene:Mgr.16443 GeneID:2679094 Uniprot:G4MVS3
Root	Isotig03189	5	20	-2.129	0.000961902	UNIPROTKB|G4MVS3 - symbol:MGG_01790 "Acyl-CoA desaturase" species:242507 "Magnaporthe oryzae 70-15" [GO:0043581 "mycelium development" evidence=IEP] InterPro:IPR001199 InterPro:IPR001522 InterPro:IPR005804 InterPro:IPR009160 InterPro:IPR015876 InterPro:IPR018506 Pfam:PF00173 Pfam:PF00487 PIRSF:PIRSF000345 PRINTS:PR00075 PROSITE:PS00191 PROSITE:PS00476 PROSITE:PS50255 GO:GO:0005783 GO:GO:0016021 GO:GO:0006633 GO:GO:0020037 KO:K00507 GO:GO:0004768 Gene3D:G3DSA:3.10.120.10 SUPFAM:SSF55856 EMBL:CM001232 RefSeq:XP_363864.1 UniGene:Mgr.16443 GeneID:2679094 Uniprot:G4MVS3
Root	Isotig03200	5	21	-2.199	0.000555521	TAIR|locus:2173108 - symbol:BAG1 "AT5G52060" species:3702 "Arabidopsis thaliana" [GO:0005739 "mitochondrion" evidence=IDA] InterPro:IPR003103 Pfam:PF02179 PROSITE:PS51035 Prosite:PS00299 GO:GO:0005739 EMBL:CP002688 GenomeReviews:BA000015_GR GO:GO:0051087 InterPro:IPR019955 PROSITE:PS50053 EMBL:AB015478 EMBL:AK227095 EMBL:BT030041 IPI:IPI00540126 RefSeq:NP_200019.2 UniGene:At.29642 ProteinModelPortal:Q0WUQ1 SMR:Q0WUQ1 PRIDE:Q9FJ86 EnsemblPlants:AT5G52060.1 GeneID:835281 KEGG:ath:AT5G52060 TAIR:At5g52060 eggNOG:NOG291823 HOGENOM:HBG602117 InParanoid:Q0WUQ1 PhylomeDB:Q0WUQ1 ProtClustDB:CLSN2920552 Genevestigator:Q0WUQ1 Uniprot:Q0WUQ1
Root	Isotig03201	5	21	-2.199	0.000555521	TAIR|locus:2173108 - symbol:BAG1 "AT5G52060" species:3702 "Arabidopsis thaliana" [GO:0005739 "mitochondrion" evidence=IDA] InterPro:IPR003103 Pfam:PF02179 PROSITE:PS51035 Prosite:PS00299 GO:GO:0005739 EMBL:CP002688 GenomeReviews:BA000015_GR GO:GO:0051087 InterPro:IPR019955 PROSITE:PS50053 EMBL:AB015478 EMBL:AK227095 EMBL:BT030041 IPI:IPI00540126 RefSeq:NP_200019.2 UniGene:At.29642 ProteinModelPortal:Q0WUQ1 SMR:Q0WUQ1 PRIDE:Q9FJ86 EnsemblPlants:AT5G52060.1 GeneID:835281 KEGG:ath:AT5G52060 TAIR:At5g52060 eggNOG:NOG291823 HOGENOM:HBG602117 InParanoid:Q0WUQ1 PhylomeDB:Q0WUQ1 ProtClustDB:CLSN2920552 Genevestigator:Q0WUQ1 Uniprot:Q0WUQ1
Root	Isotig03202	4	22	-2.588	0.000101225	TAIR|locus:2173108 - symbol:BAG1 "AT5G52060" species:3702 "Arabidopsis thaliana" [GO:0005739 "mitochondrion" evidence=IDA] InterPro:IPR003103 Pfam:PF02179 PROSITE:PS51035 Prosite:PS00299 GO:GO:0005739 EMBL:CP002688 GenomeReviews:BA000015_GR GO:GO:0051087 InterPro:IPR019955 PROSITE:PS50053 EMBL:AB015478 EMBL:AK227095 EMBL:BT030041 IPI:IPI00540126 RefSeq:NP_200019.2 UniGene:At.29642 ProteinModelPortal:Q0WUQ1 SMR:Q0WUQ1 PRIDE:Q9FJ86 EnsemblPlants:AT5G52060.1 GeneID:835281 KEGG:ath:AT5G52060 TAIR:At5g52060 eggNOG:NOG291823 HOGENOM:HBG602117 InParanoid:Q0WUQ1 PhylomeDB:Q0WUQ1 ProtClustDB:CLSN2920552 Genevestigator:Q0WUQ1 Uniprot:Q0WUQ1
Root	Isotig03203	4	22	-2.588	0.000101225	TAIR|locus:2173108 - symbol:BAG1 "AT5G52060" species:3702 "Arabidopsis thaliana" [GO:0005739 "mitochondrion" evidence=IDA] InterPro:IPR003103 Pfam:PF02179 PROSITE:PS51035 Prosite:PS00299 GO:GO:0005739 EMBL:CP002688 GenomeReviews:BA000015_GR GO:GO:0051087 InterPro:IPR019955 PROSITE:PS50053 EMBL:AB015478 EMBL:AK227095 EMBL:BT030041 IPI:IPI00540126 RefSeq:NP_200019.2 UniGene:At.29642 ProteinModelPortal:Q0WUQ1 SMR:Q0WUQ1 PRIDE:Q9FJ86 EnsemblPlants:AT5G52060.1 GeneID:835281 KEGG:ath:AT5G52060 TAIR:At5g52060 eggNOG:NOG291823 HOGENOM:HBG602117 InParanoid:Q0WUQ1 PhylomeDB:Q0WUQ1 ProtClustDB:CLSN2920552 Genevestigator:Q0WUQ1 Uniprot:Q0WUQ1
Root	Isotig03224	16	38	-1.377	0.000799708	TAIR|locus:2170698 - symbol:HIR1 "HYPERSENSITIVE-INDUCED RESPONSE PROTEIN 1" species:3702 "Arabidopsis thaliana" [GO:0005886 "plasma membrane" evidence=IDA] [GO:0005773 "vacuole" evidence=IDA] [GO:0005774 "vacuolar membrane" evidence=IDA] [GO:0016020 "membrane" evidence=IDA] [GO:0005515 "protein

Tissue	Identification	# of reads (stress)	# of reads (control)	Fold Change	p-value	Description
						binding" evidence=IPI] [GO:0043424 "protein histidine kinase binding" evidence=IPI] [GO:0005829 "cytosol" evidence=RCA] [GO:0009506 "plasmodesma" evidence=IDA] [GO:0005794 "Golgi apparatus" evidence=IDA] InterPro:IPR001972 InterPro:IPR001107 Pfam:PF01145 GO:GO:0005886 GO:GO:0009506 GO:GO:0005774 EMBL:CP002688 GenomeReviews:BA000015_GR GO:GO:0043424 EMBL:AB009053 eggNOG:COG0330 PANTHER:PTHR10264 SMART:SM00244 EMBL:AY062850 EMBL:AY114572 EMBL:AY086631 IPI:IPI00546529 RefSeq:NP_201080.1 UniGene:At.71021 ProteinModelPortal:Q9FM19 SMR:Q9FM19 IntAct:Q9FM19 PRIDE:Q9FM19 EnsemblPlants:AT5G62740.1 GeneID:836395 KEGG:ath:AT5G62740 TAIR:At5g62740 HOGENOM:HBG519201 InParanoid:Q9FM19 OMA:KANDAYY PhylomeDB:Q9FM19 ProtClustDB:CLSN2682581 ArrayExpress:Q9FM19 Genevestigator:Q9FM19 Uniprot:Q9FM19
Root	Isotig03226	16	38	-1.377	0.000799708	TAIR|locus:2170698 - symbol:HIR1 "HYPERSENSITIVE-INDUCED RESPONSE PROTEIN 1" species:3702 "Arabidopsis thaliana" [GO:0005886 "plasma membrane" evidence=IDA] [GO:0005773 "vacuole" evidence=IDA] [GO:0005774 "vacuolar membrane" evidence=IDA] [GO:0016020 "membrane" evidence=IDA] [GO:0005515 "protein binding" evidence=IPI] [GO:0043424 "protein histidine kinase binding" evidence=IPI] [GO:0005829 "cytosol" evidence=RCA] [GO:0009506 "plasmodesma" evidence=IDA] [GO:0005794 "Golgi apparatus" evidence=IDA] InterPro:IPR001972 InterPro:IPR001107 Pfam:PF01145 GO:GO:0005886 GO:GO:0009506 GO:GO:0005774 EMBL:CP002688 GenomeReviews:BA000015_GR GO:GO:0043424 EMBL:AB009053 eggNOG:COG0330 PANTHER:PTHR10264 SMART:SM00244 EMBL:AY062850 EMBL:AY114572 EMBL:AY086631 IPI:IPI00546529 RefSeq:NP_201080.1 UniGene:At.71021 ProteinModelPortal:Q9FM19 SMR:Q9FM19 IntAct:Q9FM19 PRIDE:Q9FM19 EnsemblPlants:AT5G62740.1 GeneID:836395 KEGG:ath:AT5G62740 TAIR:At5g62740 HOGENOM:HBG519201 InParanoid:Q9FM19 OMA:KANDAYY PhylomeDB:Q9FM19 ProtClustDB:CLSN2682581 ArrayExpress:Q9FM19 Genevestigator:Q9FM19 Uniprot:Q9FM19
Root	Isotig03231	56	23	1.155	0.000692158	TAIR|locus:2064965 - symbol:EULS3 "AT2G39050" species:3702 "Arabidopsis thaliana" [GO:0008150 "biological_process" evidence=ND] [GO:0005634 "nucleus" evidence=IDA] [GO:0005737 "cytoplasm" evidence=IDA] [GO:0030246 "carbohydrate binding" evidence=IDA] GO:GO:0005634 GO:GO:0005737 EMBL:CP002685 GenomeReviews:CT485783_GR InterPro:IPR000772 SUPFAM:SSF50370 PROSITE:PS50231 GO:GO:0030246 EMBL:AC005770 Pfam:PF14200 EMBL:AF411801 EMBL:AY093795 EMBL:AY087909 IPI:IPI00536641 PIR:E84812 RefSeq:NP_565899.1 UniGene:At.22599 ProteinModelPortal:Q945P1 SMR:Q945P1 PRIDE:Q945P1 EnsemblPlants:AT2G39050.1 GeneID:818491 KEGG:ath:AT2G39050 TAIR:At2g39050 eggNOG:NOG254161 HOGENOM:HBG750017 InParanoid:Q945P1 OMA:GYRTIRM PhylomeDB:Q945P1 ProtClustDB:CLSN2681741 ArrayExpress:Q945P1 Genevestigator:Q945P1 Uniprot:Q945P1
Root	Isotig03232	50	16	1.515	7.79E-05	TAIR|locus:2064965 - symbol:EULS3 "AT2G39050" species:3702 "Arabidopsis thaliana" [GO:0008150 "biological_process" evidence=ND] [GO:0005634 "nucleus" evidence=IDA] [GO:0005737 "cytoplasm" evidence=IDA] [GO:0030246 "carbohydrate binding" evidence=IDA] GO:GO:0005634 GO:GO:0005737 EMBL:CP002685 GenomeReviews:CT485783_GR InterPro:IPR000772 SUPFAM:SSF50370 PROSITE:PS50231 GO:GO:0030246 EMBL:AC005770 Pfam:PF14200 EMBL:AF411801 EMBL:AY093795 EMBL:AY087909 IPI:IPI00536641 PIR:E84812 RefSeq:NP_565899.1 UniGene:At.22599 ProteinModelPortal:Q945P1 SMR:Q945P1 PRIDE:Q945P1 EnsemblPlants:AT2G39050.1 GeneID:818491 KEGG:ath:AT2G39050 TAIR:At2g39050 eggNOG:NOG254161 HOGENOM:HBG750017 InParanoid:Q945P1 OMA:GYRTIRM PhylomeDB:Q945P1 ProtClustDB:CLSN2681741 ArrayExpress:Q945P1 Genevestigator:Q945P1 Uniprot:Q945P1
Root	Isotig03233	56	23	1.155	0.000692158	TAIR|locus:2064965 - symbol:EULS3 "AT2G39050" species:3702 "Arabidopsis thaliana" [GO:0008150 "biological_process" evidence=ND] [GO:0005634 "nucleus" evidence=IDA] [GO:0005737 "cytoplasm" evidence=IDA] [GO:0030246 "carbohydrate binding" evidence=IDA] GO:GO:0005634 GO:GO:0005737 EMBL:CP002685 GenomeReviews:CT485783_GR InterPro:IPR000772 SUPFAM:SSF50370 PROSITE:PS50231 GO:GO:0030246 EMBL:AC005770 Pfam:PF14200 EMBL:AF411801 EMBL:AY093795 EMBL:AY087909 IPI:IPI00536641 PIR:E84812 RefSeq:NP_565899.1 UniGene:At.22599 ProteinModelPortal:Q945P1 SMR:Q945P1 PRIDE:Q945P1 EnsemblPlants:AT2G39050.1 GeneID:818491 KEGG:ath:AT2G39050 TAIR:At2g39050 eggNOG:NOG254161 HOGENOM:HBG750017 InParanoid:Q945P1 OMA:GYRTIRM PhylomeDB:Q945P1 ProtClustDB:CLSN2681741 ArrayExpress:Q945P1 Genevestigator:Q945P1 Uniprot:Q945P1
Root	Isotig03234	50	16	1.515	7.79E-05	TAIR|locus:2064965 - symbol:EULS3 "AT2G39050" species:3702 "Arabidopsis thaliana" [GO:0008150 "biological_process" evidence=ND] [GO:0005634 "nucleus" evidence=IDA] [GO:0005737 "cytoplasm" evidence=IDA] [GO:0030246 "carbohydrate binding" evidence=IDA] GO:GO:0005634 GO:GO:0005737 EMBL:CP002685 GenomeReviews:CT485783_GR InterPro:IPR000772 SUPFAM:SSF50370 PROSITE:PS50231

Tissue	Identification	# of reads (stress)	# of reads (control)	Fold Change	p-value	Description
						GO:GO:0030246 EMBL:AC005770 Pfam:PF14200 EMBL:AF411801 EMBL:AY093795 EMBL:AY087909 IPI:IPI00536641 PIR:E84812 RefSeq:NP_565899.1 UniGene:At.22599 ProteinModelPortal:Q945P1 SMR:Q945P1 PRIDE:Q945P1 EnsemblPlants:AT2G39050.1 GeneID:818491 KEGG:ath:AT2G39050 TAIR:At2g39050 eggNOG:NOG254161 HOGENOM:HBG750017 InParanoid:Q945P1 OMA:GYRTIRM PhylomeDB:Q945P1 ProtClustDB:CLSN2681741 ArrayExpress:Q945P1 Genevestigator:Q945P1 Uniprot:Q945P1
Root	Isotig03235	23	0	5.395	1.11E-06	TAIR|locus:2087183 - symbol:MYB305 "AT3G24310" species:3702 "Arabidopsis thaliana" [GO:0003677 "DNA binding" evidence=ISS] [GO:0003700 "sequence-specific DNA binding transcription factor activity" evidence=ISS] [GO:0006355 "regulation of transcription, DNA-dependent" evidence=ISS] InterPro:IPR001005 InterPro:IPR009057 Pfam:PF00249 SMART:SM00717 GO:GO:0005634 GO:GO:0006355 GO:GO:0003677 Gene3D:G3DSA:1.10.10.60 SUPFAM:SSF46689 InterPro:IPR017930 PROSITE:PS51294 InterPro:IPR015495 PANTHER:PTHR10641 HSSP:Q03237 EMBL:AY550298 IPI:IPI00525453 ProteinModelPortal:Q6QAD8 PRIDE:Q6QAD8 TAIR:At3g24310 PhylomeDB:Q6QAD8 ArrayExpress:Q6QAD8 Genevestigator:Q6QAD8 Uniprot:Q6QAD8
Root	Isotig03236	23	0	5.395	1.11E-06	TAIR|locus:2087183 - symbol:MYB305 "AT3G24310" species:3702 "Arabidopsis thaliana" [GO:0003677 "DNA binding" evidence=ISS] [GO:0003700 "sequence-specific DNA binding transcription factor activity" evidence=ISS] [GO:0006355 "regulation of transcription, DNA-dependent" evidence=ISS] InterPro:IPR001005 InterPro:IPR009057 Pfam:PF00249 SMART:SM00717 GO:GO:0005634 GO:GO:0006355 GO:GO:0003677 Gene3D:G3DSA:1.10.10.60 SUPFAM:SSF46689 InterPro:IPR017930 PROSITE:PS51294 InterPro:IPR015495 PANTHER:PTHR10641 HSSP:Q03237 EMBL:AY550298 IPI:IPI00525453 ProteinModelPortal:Q6QAD8 PRIDE:Q6QAD8 TAIR:At3g24310 PhylomeDB:Q6QAD8 ArrayExpress:Q6QAD8 Genevestigator:Q6QAD8 Uniprot:Q6QAD8
Root	Isotig03237	23	0	5.395	1.11E-06	TAIR|locus:2087183 - symbol:MYB305 "AT3G24310" species:3702 "Arabidopsis thaliana" [GO:0003677 "DNA binding" evidence=ISS] [GO:0003700 "sequence-specific DNA binding transcription factor activity" evidence=ISS] [GO:0006355 "regulation of transcription, DNA-dependent" evidence=ISS] InterPro:IPR001005 InterPro:IPR009057 Pfam:PF00249 SMART:SM00717 GO:GO:0005634 GO:GO:0006355 GO:GO:0003677 Gene3D:G3DSA:1.10.10.60 SUPFAM:SSF46689 InterPro:IPR017930 PROSITE:PS51294 InterPro:IPR015495 PANTHER:PTHR10641 HSSP:Q03237 EMBL:AY550298 IPI:IPI00525453 ProteinModelPortal:Q6QAD8 PRIDE:Q6QAD8 TAIR:At3g24310 PhylomeDB:Q6QAD8 ArrayExpress:Q6QAD8 Genevestigator:Q6QAD8 Uniprot:Q6QAD8
Root	Isotig03238	23	0	5.395	1.11E-06	TAIR|locus:2087183 - symbol:MYB305 "AT3G24310" species:3702 "Arabidopsis thaliana" [GO:0003677 "DNA binding" evidence=ISS] [GO:0003700 "sequence-specific DNA binding transcription factor activity" evidence=ISS] [GO:0006355 "regulation of transcription, DNA-dependent" evidence=ISS] InterPro:IPR001005 InterPro:IPR009057 Pfam:PF00249 SMART:SM00717 GO:GO:0005634 GO:GO:0006355 GO:GO:0003677 Gene3D:G3DSA:1.10.10.60 SUPFAM:SSF46689 InterPro:IPR017930 PROSITE:PS51294 InterPro:IPR015495 PANTHER:PTHR10641 HSSP:Q03237 EMBL:AY550298 IPI:IPI00525453 ProteinModelPortal:Q6QAD8 PRIDE:Q6QAD8 TAIR:At3g24310 PhylomeDB:Q6QAD8 ArrayExpress:Q6QAD8 Genevestigator:Q6QAD8 Uniprot:Q6QAD8
Root	Isotig03267	81	7	3.404	3.81E-16	No hit
Root	Isotig03268	80	7	3.386	6.66E-16	No hit
Root	Isotig03295	4	41	-3.487	9.45E-10	TAIR|locus:2076904 - symbol:GRF7 "AT3G02520" species:3702 "Arabidopsis thaliana" [GO:0005635 "nuclear envelope" evidence=IDA] [GO:0005737 "cytoplasm" evidence=IDA] [GO:0005886 "plasma membrane" evidence=IDA] [GO:0045309 "protein phosphorylated amino acid binding" evidence=TAS] [GO:0009507 "chloroplast" evidence=IDA] [GO:0005829 "cytosol" evidence=RCA] [GO:0009506 "plasmodesma" evidence=IDA] [GO:0005794 "Golgi apparatus" evidence=IDA] [GO:0009570 "chloroplast stroma" evidence=IDA] InterPro:IPR000308 PIRSF:PIRSF000868 PRINTS:PR00305 HOGENOM:HBG611720 GO:GO:0005886 GO:GO:0009506 GO:GO:0019904 InterPro:IPR023409 InterPro:IPR023410 Gene3D:G3DSA:1.20.190.20 PANTHER:PTHR18860 Pfam:PF00244 SMART:SM00101 SUPFAM:SSF48445 PROSITE:PS00796 PROSITE:PS00797 GO:GO:0009507 ProtClustDB:CLSN2688003 EMBL:U60445 EMBL:AF145299 EMBL:AC021640 EMBL:CP002686 EMBL:AY065274 EMBL:AY096526 EMBL:AY087723 IPI:IPI00536399 RefSeq:NP_566174.1 UniGene:At.21863 ProteinModelPortal:Q96300 SMR:Q96300 IntAct:Q96300 STRING:Q96300 PRIDE:Q96300 EnsemblPlants:AT3G02520.1 GeneID:821060 GenomeReviews:BA000014_GR KEGG:ath:AT3G02520 TAIR:At3g02520 InParanoid:Q96300 OMA:KVCQDVL PhylomeDB:Q96300 ArrayExpress:Q96300 Genevestigator:Q96300 GermOnline:AT3G02520 Uniprot:Q96300
Root	Isotig03296	3	43	-3.970	5.33E-11	TAIR|locus:2076904 - symbol:GRF7 "AT3G02520" species:3702 "Arabidopsis thaliana" [GO:0005635 "nuclear envelope" evidence=IDA] [GO:0005737 "cytoplasm" evidence=IDA] [GO:0005886 "plasma membrane" evidence=IDA] [GO:0045309 "protein phosphorylated amino acid binding" evidence=TAS] [GO:0009507

Tissue	Identification	# of reads (stress)	# of reads (control)	Fold Change	p-value	Description
						"chloroplast" evidence=IDA] [GO:0005829 "cytosol" evidence=RCA] [GO:0009506 "plasmodesma" evidence=IDA] [GO:0005794 "Golgi apparatus" evidence=IDA] [GO:0009570 "chloroplast stroma" evidence=IDA] InterPro:IPR000308 PIRSF:PIRSF000868 PRINTS:PR00305 HOGENOM:HBG611720 GO:GO:0005886 GO:GO:0009506 GO:GO:0019904 InterPro:IPR023409 InterPro:IPR023410 Gene3D:G3DSA:1.20.190.20 PANTHER:PTHR18860 Pfam:PF00244 SMART:SM00101 SUPFAM:SSF48445 PROSITE:PS00796 PROSITE:PS00797 GO:GO:0009507 ProtClustDB:CLSN2688003 EMBL:U60445 EMBL:AF145299 EMBL:AC021640 EMBL:CP002686 EMBL:AY065274 EMBL:AY096526 EMBL:AY087723 IPI:IPI00536399 RefSeq:NP_566174.1 UniGene:At.21863 ProteinModelPortal:Q96300 SMR:Q96300 IntAct:Q96300 STRING:Q96300 PRIDE:Q96300 EnsemblPlants:AT3G02520.1 GeneID:821060 GenomeReviews:BA000014_GR KEGG:ath:AT3G02520 TAIR:At3g02520 InParanoid:Q96300 OMA:KVCQDVL PhylomeDB:Q96300 ArrayExpress:Q96300 Genevestigator:Q96300 GermOnline:AT3G02520 Uniprot:Q96300
Root	Isotig03297	4	41	-3.487	9.45E-10	TAIR|locus:2076904 - symbol:GRF7 "AT3G02520" species:3702 "Arabidopsis thaliana" [GO:0005635 "nuclear envelope" evidence=IDA] [GO:0005737 "cytoplasm" evidence=IDA] [GO:0005886 "plasma membrane" evidence=IDA] [GO:0045309 "protein phosphorylated amino acid binding" evidence=TAS] [GO:0009507 "chloroplast" evidence=IDA] [GO:0005829 "cytosol" evidence=RCA] [GO:0009506 "plasmodesma" evidence=IDA] [GO:0005794 "Golgi apparatus" evidence=IDA] [GO:0009570 "chloroplast stroma" evidence=IDA] InterPro:IPR000308 PIRSF:PIRSF000868 PRINTS:PR00305 HOGENOM:HBG611720 GO:GO:0005886 GO:GO:0009506 GO:GO:0019904 InterPro:IPR023409 InterPro:IPR023410 Gene3D:G3DSA:1.20.190.20 PANTHER:PTHR18860 Pfam:PF00244 SMART:SM00101 SUPFAM:SSF48445 PROSITE:PS00796 PROSITE:PS00797 GO:GO:0009507 ProtClustDB:CLSN2688003 EMBL:U60445 EMBL:AF145299 EMBL:AC021640 EMBL:CP002686 EMBL:AY065274 EMBL:AY096526 EMBL:AY087723 IPI:IPI00536399 RefSeq:NP_566174.1 UniGene:At.21863 ProteinModelPortal:Q96300 SMR:Q96300 IntAct:Q96300 STRING:Q96300 PRIDE:Q96300 EnsemblPlants:AT3G02520.1 GeneID:821060 GenomeReviews:BA000014_GR KEGG:ath:AT3G02520 TAIR:At3g02520 InParanoid:Q96300 OMA:KVCQDVL PhylomeDB:Q96300 ArrayExpress:Q96300 Genevestigator:Q96300 GermOnline:AT3G02520 Uniprot:Q96300
Root	Isotig03298	3	43	-3.970	5.33E-11	TAIR|locus:2076904 - symbol:GRF7 "AT3G02520" species:3702 "Arabidopsis thaliana" [GO:0005635 "nuclear envelope" evidence=IDA] [GO:0005737 "cytoplasm" evidence=IDA] [GO:0005886 "plasma membrane" evidence=IDA] [GO:0045309 "protein phosphorylated amino acid binding" evidence=TAS] [GO:0009507 "chloroplast" evidence=IDA] [GO:0005829 "cytosol" evidence=RCA] [GO:0009506 "plasmodesma" evidence=IDA] [GO:0005794 "Golgi apparatus" evidence=IDA] [GO:0009570 "chloroplast stroma" evidence=IDA] InterPro:IPR000308 PIRSF:PIRSF000868 PRINTS:PR00305 HOGENOM:HBG611720 GO:GO:0005886 GO:GO:0009506 GO:GO:0019904 InterPro:IPR023409 InterPro:IPR023410 Gene3D:G3DSA:1.20.190.20 PANTHER:PTHR18860 Pfam:PF00244 SMART:SM00101 SUPFAM:SSF48445 PROSITE:PS00796 PROSITE:PS00797 GO:GO:0009507 ProtClustDB:CLSN2688003 EMBL:U60445 EMBL:AF145299 EMBL:AC021640 EMBL:CP002686 EMBL:AY065274 EMBL:AY096526 EMBL:AY087723 IPI:IPI00536399 RefSeq:NP_566174.1 UniGene:At.21863 ProteinModelPortal:Q96300 SMR:Q96300 IntAct:Q96300 STRING:Q96300 PRIDE:Q96300 EnsemblPlants:AT3G02520.1 GeneID:821060 GenomeReviews:BA000014_GR KEGG:ath:AT3G02520 TAIR:At3g02520 InParanoid:Q96300 OMA:KVCQDVL PhylomeDB:Q96300 ArrayExpress:Q96300 Genevestigator:Q96300 GermOnline:AT3G02520 Uniprot:Q96300
Root	Isotig03311	1	14	-3.936	0.000188601	TAIR|locus:2117353 - symbol:PAP24 "AT4G24890" species:3702 "Arabidopsis thaliana" [GO:0004722 "protein serine/threonine phosphatase activity" evidence=ISS] [GO:0008150 "biological_process" evidence=ND] [GO:0005618 "cell wall" evidence=IDA] [GO:0003993 "acid phosphatase activity" evidence=ISS] InterPro:IPR004843 InterPro:IPR008963 InterPro:IPR015914 Pfam:PF00149 EMBL:CP002687 GenomeReviews:CT486007_GR GO:GO:0005618 GO:GO:0005576 GO:GO:0046872 GO:GO:0003993 eggNOG:COG1409 EMBL:AL161562 EMBL:AL035523 Gene3D:G3DSA:2.60.40.380 SUPFAM:SSF49363 EMBL:AY842025 EMBL:AY142520 IPI:IPI00548773 PIR:T05510 RefSeq:NP_194219.2 UniGene:At.32313 ProteinModelPortal:Q8H1R2 SMR:Q8H1R2 PRIDE:Q8H1R2 EnsemblPlants:AT4G24890.1 GeneID:828591 KEGG:ath:AT4G24890 TAIR:At4g24890 InParanoid:Q8H1R2 OMA:AICEPEN PhylomeDB:Q8H1R2 ProtClustDB:CLSN2918186 Genevestigator:Q8H1R2 Uniprot:Q8H1R2
Root	Isotig03312	1	14	-3.936	0.000188601	TAIR|locus:2117353 - symbol:PAP24 "AT4G24890" species:3702 "Arabidopsis thaliana" [GO:0004722 "protein serine/threonine phosphatase activity" evidence=ISS] [GO:0008150 "biological_process" evidence=ND] [GO:0005618 "cell wall" evidence=IDA] [GO:0003993 "acid phosphatase activity" evidence=ISS] InterPro:IPR004843

Tissue	Identification	# of reads (stress)	# of reads (control)	Fold Change	p-value	Description
						InterPro:IPR008963 InterPro:IPR015914 Pfam:PF00149 EMBL:CP002687 GenomeReviews:CT486007_GR GO:GO:0005618 GO:GO:0005576 GO:GO:0046872 GO:GO:0003993 eggNOG:COG1409 EMBL:AL161562 EMBL:AL035523 Gene3D:G3DSA:2.60.40.380 SUPFAM:SSF49363 EMBL:AY842025 EMBL:AY142520 IPI:IPI00548773 PIR:T05510 RefSeq:NP_194219.2 UniGene:At.32313 ProteinModelPortal:Q8H1R2 SMR:Q8H1R2 PRIDE:Q8H1R2 EnsemblPlants:AT4G24890.1 GeneID:828591 KEGG:ath:AT4G24890 TAIR:At4g24890 InParanoid:Q8H1R2 OMA:AICEPEN PhylomeDB:Q8H1R2 ProtClustDB:CLSN2918186 Genevestigator:Q8H1R2 Uniprot:Q8H1R2
Root	Isotig03313	1	14	-3.936	0.000188601	TAIR|locus:2117353 - symbol:PAP24 "AT4G24890" species:3702 "Arabidopsis thaliana" [GO:0004722 "protein serine/threonine phosphatase activity" evidence=ISS] [GO:0008150 "biological_process" evidence=ND] [GO:0005618 "cell wall" evidence=IDA] [GO:0003993 "acid phosphatase activity" evidence=ISS] InterPro:IPR004843 InterPro:IPR008963 InterPro:IPR015914 Pfam:PF00149 EMBL:CP002687 GenomeReviews:CT486007_GR GO:GO:0005618 GO:GO:0005576 GO:GO:0046872 GO:GO:0003993 eggNOG:COG1409 EMBL:AL161562 EMBL:AL035523 Gene3D:G3DSA:2.60.40.380 SUPFAM:SSF49363 EMBL:AY842025 EMBL:AY142520 IPI:IPI00548773 PIR:T05510 RefSeq:NP_194219.2 UniGene:At.32313 ProteinModelPortal:Q8H1R2 SMR:Q8H1R2 PRIDE:Q8H1R2 EnsemblPlants:AT4G24890.1 GeneID:828591 KEGG:ath:AT4G24890 TAIR:At4g24890 InParanoid:Q8H1R2 OMA:AICEPEN PhylomeDB:Q8H1R2 ProtClustDB:CLSN2918186 Genevestigator:Q8H1R2 Uniprot:Q8H1R2
Root	Isotig03314	1	14	-3.936	0.000188601	TAIR|locus:2117353 - symbol:PAP24 "AT4G24890" species:3702 "Arabidopsis thaliana" [GO:0004722 "protein serine/threonine phosphatase activity" evidence=ISS] [GO:0008150 "biological_process" evidence=ND] [GO:0005618 "cell wall" evidence=IDA] [GO:0003993 "acid phosphatase activity" evidence=ISS] InterPro:IPR004843 InterPro:IPR008963 InterPro:IPR015914 Pfam:PF00149 EMBL:CP002687 GenomeReviews:CT486007_GR GO:GO:0005618 GO:GO:0005576 GO:GO:0046872 GO:GO:0003993 eggNOG:COG1409 EMBL:AL161562 EMBL:AL035523 Gene3D:G3DSA:2.60.40.380 SUPFAM:SSF49363 EMBL:AY842025 EMBL:AY142520 IPI:IPI00548773 PIR:T05510 RefSeq:NP_194219.2 UniGene:At.32313 ProteinModelPortal:Q8H1R2 SMR:Q8H1R2 PRIDE:Q8H1R2 EnsemblPlants:AT4G24890.1 GeneID:828591 KEGG:ath:AT4G24890 TAIR:At4g24890 InParanoid:Q8H1R2 OMA:AICEPEN PhylomeDB:Q8H1R2 ProtClustDB:CLSN2918186 Genevestigator:Q8H1R2 Uniprot:Q8H1R2
Root	Isotig03381	11	31	-1.624	0.000622299	TAIR|locus:2079924 - symbol:UBQ1 "AT3G52590" species:3702 "Arabidopsis thaliana" [GO:0009793 "embryo development ending in seed dormancy" evidence=NAS] [GO:0003735 "structural constituent of ribosome" evidence=ISS] [GO:0016567 "protein ubiquitination" evidence=TAS] InterPro:IPR000626 Pfam:PF00240 SMART:SM00213 Prosite:PS00299 EMBL:CP002687 GenomeReviews:CT486007_GR GO:GO:0005634 GO:GO:0005737 EMBL:AL161503 InterPro:IPR019955 PROSITE:PS50053 UniGene:At.48785 InterPro:IPR019954 eggNOG:COG5272 EMBL:AC012392 InterPro:IPR019956 PRINTS:PR00348 UniGene:At.23108 KO:K08770 EMBL:L05361 EMBL:AY139999 EMBL:BT000701 IPI:IPI00520650 IPI:IPI00895605 RefSeq:NP_001118922.1 RefSeq:NP_849292.1 RefSeq:NP_849299.4 RefSeq:NP_849301.4 RefSeq:NP_974516.4 UniGene:At.24970 UniGene:At.25274 UniGene:At.47590 UniGene:At.74401 ProteinModelPortal:Q8H159 SMR:Q8H159 EnsemblPlants:AT4G05320.2 EnsemblPlants:AT4G05320.4 GeneID:825880 GeneID:828148 KEGG:ath:AT4G02890 KEGG:ath:AT4G05320 TAIR:At4g05320 PhylomeDB:Q8H159 ProtClustDB:CLSN2692469 Uniprot:Q8H159
Root	Isotig03382	9	34	-2.047	2.70E-05	UNIPROTKB|Q5A109 - symbol:UBI3 "Ubiquitin-ribosomal protein fusion S27a" species:237561 "Candida albicans SC5314" [GO:0003735 "structural constituent of ribosome" evidence=IGI] [GO:0005576 "extracellular region" evidence=IDA] [GO:0016567 "protein ubiquitination" evidence=IGI] [GO:0031386 "protein tag" evidence=IGI] [GO:0042254 "ribosome biogenesis" evidence=IGI] InterPro:IPR000626 InterPro:IPR002906 Pfam:PF00240 Pfam:PF01599 SMART:SM00213 CGD:CAL0001506 Prosite:PS00299 GO:GO:0005576 GO:GO:0006412 GO:GO:0016567 GO:GO:0003735 InterPro:IPR019955 PROSITE:PS50053 GO:GO:0005840 GO:GO:0042254 GO:GO:0031386 InterPro:IPR019954 EMBL:AACQ01000086 EMBL:AACQ01000085 InterPro:IPR019956 PRINTS:PR00348 KO:K02977 RefSeq:XP_715397.1 RefSeq:XP_715467.1 ProteinModelPortal:Q5A109 SMR:Q5A109 STRING:Q5A109 GeneID:3642848 GeneID:3642934 KEGG:cal:CaO19.10599 KEGG:cal:CaO19.3087 PhylomeDB:Q5A109 Uniprot:Q5A109
Root	Isotig03455	24	53	-1.272	0.000195269	TAIR|locus:2077793 - symbol:iPGAM2 "AT3G08590" species:3702 "Arabidopsis thaliana" [GO:0046537 "2,3-bisphosphoglycerate-independent phosphoglycerate mutase activity" evidence=IGI;ISS] [GO:0046686 "response to cadmium ion" evidence=IEP] [GO:0005829 "cytosol" evidence=IDA] [GO:0048046 "apoplast" evidence=IDA]

Tissue	Identification	# of reads (stress)	# of reads (control)	Fold Change	p-value	Description
						[GO:0009506 "plasmodesma" evidence=IDA] [GO:0009555 "pollen development" evidence=IGI] [GO:0010118 "stomatal movement" evidence=IGI] InterPro:IPR005995 InterPro:IPR006124 InterPro:IPR011258 InterPro:IPR017849 InterPro:IPR017850 Pfam:PF01676 Pfam:PF06415 PIRSF:PIRSF001492 GO:GO:0005829 GO:GO:0009506 GO:GO:0048046 GO:GO:0046686 EMBL:CP002686 GenomeReviews:BA000014_GR GO:GO:0009555 GO:GO:0010118 GO:GO:0030145 GO:GO:0006096 EMBL:AC012562 Gene3D:G3DSA:3.40.720.10 SUPFAM:SSF53649 eggNOG:COG0696 HOGENOM:HBG613255 KO:K15633 Gene3D:G3DSA:3.40.1450.10 SUPFAM:SSF64158 TIGRFAMs:TIGR01307 ProtClustDB:PLN02538 GO:GO:0046537 EMBL:AY039969 EMBL:AF424615 EMBL:AY113910 IPI:IPI00520882 RefSeq:NP_187471.1 RefSeq:NP_850542.1 UniGene:At.68440 ProteinModelPortal:Q9M9K1 SMR:Q9M9K1 STRING:Q9M9K1 PRIDE:Q9M9K1 EnsemblPlants:AT3G08590.1 EnsemblPlants:AT3G08590.2 GeneID:820006 KEGG:ath:AT3G08590 TAIR:At3g08590 InParanoid:Q9M9K1 OMA:AGRIYAQ PhylomeDB:Q9M9K1 ArrayExpress:Q9M9K1 Genevestigator:Q9M9K1 GermOnline:AT3G08590 Uniprot:Q9M9K1
Root	Isotig03472	5	29	-2.665	5.76E-06	TAIR|locus:2086177 - symbol:LPD1 "AT3G16950" species:3702 "Arabidopsis thaliana" [GO:0004148 "dihydrolipoyl dehydrogenase activity" evidence=ISS] [GO:0006086 "acetyl-CoA biosynthetic process from pyruvate" evidence=TAS] [GO:0009570 "chloroplast stroma" evidence=IDA] [GO:0009941 "chloroplast envelope" evidence=IDA] [GO:0009507 "chloroplast" evidence=IDA] [GO:0005730 "nucleolus" evidence=IDA] [GO:0005829 "cytosol" evidence=RCA] InterPro:IPR001327 InterPro:IPR004099 InterPro:IPR006258 InterPro:IPR012999 InterPro:IPR013027 InterPro:IPR016156 InterPro:IPR023753 Pfam:PF00070 Pfam:PF02852 Pfam:PF07992 PRINTS:PR00368 PROSITE:PS00076 GO:GO:0009570 EMBL:CP002686 GO:GO:0005730 GO:GO:0050660 GO:GO:0009941 EMBL:AB026636 GO:GO:0045454 Gene3D:G3DSA:3.30.390.30 SUPFAM:SSF55424 eggNOG:COG1249 KO:K00382 GO:GO:0004148 TIGRFAMs:TIGR01350 GO:GO:0006086 IPI:IPI00846791 RefSeq:NP_001078165.1 UniGene:At.16905 ProteinModelPortal:A8MS68 SMR:A8MS68 STRING:A8MS68 PRIDE:A8MS68 EnsemblPlants:AT3G16950.2 GeneID:820951 KEGG:ath:AT3G16950 TAIR:At3g16950 PhylomeDB:A8MS68 ProtClustDB:CLSN2688461 Genevestigator:A8MS68 Uniprot:A8MS68
Root	Isotig03473	4	24	-2.714	3.10E-05	TAIR|locus:2086177 - symbol:LPD1 "AT3G16950" species:3702 "Arabidopsis thaliana" [GO:0004148 "dihydrolipoyl dehydrogenase activity" evidence=ISS] [GO:0006086 "acetyl-CoA biosynthetic process from pyruvate" evidence=TAS] [GO:0009570 "chloroplast stroma" evidence=IDA] [GO:0009941 "chloroplast envelope" evidence=IDA] [GO:0009507 "chloroplast" evidence=IDA] [GO:0005730 "nucleolus" evidence=IDA] [GO:0005829 "cytosol" evidence=RCA] InterPro:IPR001327 InterPro:IPR004099 InterPro:IPR006258 InterPro:IPR012999 InterPro:IPR013027 InterPro:IPR016156 InterPro:IPR023753 Pfam:PF00070 Pfam:PF02852 Pfam:PF07992 PRINTS:PR00368 PROSITE:PS00076 GO:GO:0009570 EMBL:CP002686 GO:GO:0005730 GO:GO:0050660 GO:GO:0009941 EMBL:AB026636 GO:GO:0045454 Gene3D:G3DSA:3.30.390.30 SUPFAM:SSF55424 eggNOG:COG1249 KO:K00382 GO:GO:0004148 TIGRFAMs:TIGR01350 GO:GO:0006086 IPI:IPI00846791 RefSeq:NP_001078165.1 UniGene:At.16905 ProteinModelPortal:A8MS68 SMR:A8MS68 STRING:A8MS68 PRIDE:A8MS68 EnsemblPlants:AT3G16950.2 GeneID:820951 KEGG:ath:AT3G16950 TAIR:At3g16950 PhylomeDB:A8MS68 ProtClustDB:CLSN2688461 Genevestigator:A8MS68 Uniprot:A8MS68
Root	Isotig03478	17	0	4.958	3.30E-05	No hit
Root	Isotig03479	17	0	4.958	3.30E-05	No hit
Root	Isotig03487	0	12	-4.714	0.000297064	TAIR|locus:2122749 - symbol:HKT1 "AT4G10310" species:3702 "Arabidopsis thaliana" [GO:0015081 "sodium ion transmembrane transporter activity" evidence=ISS;IDA] [GO:0006814 "sodium ion transport" evidence=IMP;IDA] [GO:0009651 "response to salt stress" evidence=IGI;IMP] [GO:0005886 "plasma membrane" evidence=IDA] [GO:0006813 "potassium ion transport" evidence=IMP] [GO:0006970 "response to osmotic stress" evidence=IEP] InterPro:IPR003445 InterPro:IPR004773 Pfam:PF02386 GO:GO:0016021 GO:GO:0005886 EMBL:CP002687 GenomeReviews:CT486007_GR GO:GO:0009651 GO:GO:0006814 EMBL:AF096373 EMBL:AL049488 EMBL:AL161517 GO:GO:0015079 EMBL:AF237672 EMBL:AY685182 EMBL:BT005751 IPI:IPI00518872 PIR:T01969 PIR:T04046 RefSeq:NP_567354.1 UniGene:At.2986 ProteinModelPortal:Q84TI7 STRING:Q84TI7 TCDB:2.A.38.3.2 PRIDE:Q84TI7 EnsemblPlants:AT4G10310.1 GeneID:826623 KEGG:ath:AT4G10310 GeneFarm:5166 TAIR:At4g10310 eggNOG:COG0168 HOGENOM:HBG599187 InParanoid:Q84TI7 OMA:VNSRHTG PhylomeDB:Q84TI7 ProtClustDB:CLSN2917526 BioCyc:MetaCyc:MONOMER-14577 Genevestigator:Q84TI7 GermOnline:AT4G10310 TIGRFAMs:TIGR00934 Uniprot:Q84TI7
Root	Isotig03489	0	12	-4.714	0.000297064	TAIR|locus:2122749 - symbol:HKT1 "AT4G10310" species:3702 "Arabidopsis thaliana" [GO:0015081 "sodium ion transmembrane transporter activity" evidence=ISS;IDA] [GO:0006814 "sodium ion transport" evidence=IMP;IDA]

Tissue	Identification	# of reads (stress)	# of reads (control)	Fold Change	p-value	Description
						[GO:0009651 "response to salt stress" evidence=IGI;IMP] [GO:0005886 "plasma membrane" evidence=IDA] [GO:0006813 "potassium ion transport" evidence=IMP] [GO:0006970 "response to osmotic stress" evidence=IEP] InterPro:IPR003445 InterPro:IPR004773 Pfam:PF02386 GO:GO:0016021 GO:GO:0005886 EMBL:CP002687 GenomeReviews:CT486007_GR GO:GO:0009651 GO:GO:0006814 EMBL:AF096373 EMBL:AL049488 EMBL:AL161517 GO:GO:0015079 EMBL:AF237672 EMBL:AY685182 EMBL:BT005751 IPI:IPI00518872 PIR:T01969 PIR:T04046 RefSeq:NP_567354.1 UniGene:At.2986 ProteinModelPortal:Q84TI7 STRING:Q84TI7 TCDB:2.A.38.3.2 PRIDE:Q84TI7 EnsemblPlants:AT4G10310.1 GeneID:826623 KEGG:ath:AT4G10310 GeneFarm:5166 TAIR:At4g10310 eggNOG:COG0168 HOGENOM:HBG599187 InParanoid:Q84TI7 OMA:VNSRHTG PhylomeDB:Q84TI7 ProtClustDB:CLSN2917526 BioCyc:MetaCyc:MONOMER-14577 Genevestigator:Q84TI7 GermOnline:AT4G10310 TIGRFAMs:TIGR00934 Uniprot:Q84TI7
Root	Isotig03509	19	3	2.534	0.000639389	No hit
Root	Isotig03510	19	3	2.534	0.000639389	No hit
Root	Isotig03512	44	6	2.745	5.73E-08	TAIR|locus:2141060 - symbol:AGD8 "AT4G17890" species:3702 "Arabidopsis thaliana" [GO:0003677 "DNA binding" evidence=ISS] [GO:0005634 "nucleus" evidence=ISS] [GO:0008060 "ARF GTPase activator activity" evidence=IEA] [GO:0008270 "zinc ion binding" evidence=IEA] [GO:0032312 "regulation of ARF GTPase activity" evidence=IEA] [GO:0005829 "cytosol" evidence=IDA] InterPro:IPR001164 Pfam:PF01412 PRINTS:PR00405 PROSITE:PS50115 SMART:SM00105 GO:GO:0005829 EMBL:CP002687 GenomeReviews:CT486007_GR GO:GO:0008270 EMBL:AL161547 eggNOG:COG5347 GO:GO:0008060 GO:GO:0032312 SUPFAM:SSF57863 HSSP:Q9NP61 HOGENOM:HBG736886 EMBL:AL021889 EMBL:AF360177 EMBL:BT000968 EMBL:AK175252 EMBL:AK176287 EMBL:AK176618 EMBL:AK221239 EMBL:AK221371 EMBL:AK229721 EMBL:AK317033 IPI:IPI00525155 IPI:IPI00657128 PIR:T05075 RefSeq:NP_001031658.1 RefSeq:NP_567543.1 UniGene:At.26724 UniGene:At.66582 ProteinModelPortal:Q8H100 SMR:Q8H100 PRIDE:Q8H100 EnsemblPlants:AT4G17890.1 GeneID:827512 KEGG:ath:AT4G17890 TAIR:At4g17890 InParanoid:Q8H100 OMA:NIGRSAD PhylomeDB:Q8H100 ProtClustDB:CLSN2686195 Genevestigator:Q8H100 Uniprot:Q8H100
Root	Isotig03513	35	5	2.678	1.78E-06	TAIR|locus:2141060 - symbol:AGD8 "AT4G17890" species:3702 "Arabidopsis thaliana" [GO:0003677 "DNA binding" evidence=ISS] [GO:0005634 "nucleus" evidence=ISS] [GO:0008060 "ARF GTPase activator activity" evidence=IEA] [GO:0008270 "zinc ion binding" evidence=IEA] [GO:0032312 "regulation of ARF GTPase activity" evidence=IEA] [GO:0005829 "cytosol" evidence=IDA] InterPro:IPR001164 Pfam:PF01412 PRINTS:PR00405 PROSITE:PS50115 SMART:SM00105 GO:GO:0005829 EMBL:CP002687 GenomeReviews:CT486007_GR GO:GO:0008270 EMBL:AL161547 eggNOG:COG5347 GO:GO:0008060 GO:GO:0032312 SUPFAM:SSF57863 HSSP:Q9NP61 HOGENOM:HBG736886 EMBL:AL021889 EMBL:AF360177 EMBL:BT000968 EMBL:AK175252 EMBL:AK176287 EMBL:AK176618 EMBL:AK221239 EMBL:AK221371 EMBL:AK229721 EMBL:AK317033 IPI:IPI00525155 IPI:IPI00657128 PIR:T05075 RefSeq:NP_001031658.1 RefSeq:NP_567543.1 UniGene:At.26724 UniGene:At.66582 ProteinModelPortal:Q8H100 SMR:Q8H100 PRIDE:Q8H100 EnsemblPlants:AT4G17890.1 GeneID:827512 KEGG:ath:AT4G17890 TAIR:At4g17890 InParanoid:Q8H100 OMA:NIGRSAD PhylomeDB:Q8H100 ProtClustDB:CLSN2686195 Genevestigator:Q8H100 Uniprot:Q8H100
Root	Isotig03514	41	5	2.907	6.91E-08	TAIR|locus:2141060 - symbol:AGD8 "AT4G17890" species:3702 "Arabidopsis thaliana" [GO:0003677 "DNA binding" evidence=ISS] [GO:0005634 "nucleus" evidence=ISS] [GO:0008060 "ARF GTPase activator activity" evidence=IEA] [GO:0008270 "zinc ion binding" evidence=IEA] [GO:0032312 "regulation of ARF GTPase activity" evidence=IEA] [GO:0005829 "cytosol" evidence=IDA] InterPro:IPR001164 Pfam:PF01412 PRINTS:PR00405 PROSITE:PS50115 SMART:SM00105 GO:GO:0005829 EMBL:CP002687 GenomeReviews:CT486007_GR GO:GO:0008270 EMBL:AL161547 eggNOG:COG5347 GO:GO:0008060 GO:GO:0032312 SUPFAM:SSF57863 HSSP:Q9NP61 HOGENOM:HBG736886 EMBL:AL021889 EMBL:AF360177 EMBL:BT000968 EMBL:AK175252 EMBL:AK176287 EMBL:AK176618 EMBL:AK221239 EMBL:AK221371 EMBL:AK229721 EMBL:AK317033 IPI:IPI00525155 IPI:IPI00657128 PIR:T05075 RefSeq:NP_001031658.1 RefSeq:NP_567543.1 UniGene:At.26724 UniGene:At.66582 ProteinModelPortal:Q8H100 SMR:Q8H100 PRIDE:Q8H100 EnsemblPlants:AT4G17890.1 GeneID:827512 KEGG:ath:AT4G17890 TAIR:At4g17890 InParanoid:Q8H100 OMA:NIGRSAD PhylomeDB:Q8H100 ProtClustDB:CLSN2686195 Genevestigator:Q8H100 Uniprot:Q8H100
Root	Isotig03521	0	39	-6.414	3.85E-11	UNIPROTKB|A8QW53 - symbol:OMT3 "5-pentadecatrienyl resorcinol O-methyltransferase" species:4558 "Sorghum bicolor" [GO:0008171 "O-methyltransferase activity" evidence=IDA] [GO:0008757 "S-adenosylmethionine-dependent methyltransferase activity" evidence=IDA] [GO:0032259 "methylation" evidence=IDA] InterPro:IPR001077 InterPro:IPR012967 InterPro:IPR016461 Pfam:PF00891 Pfam:PF08100 PIRSF:PIRSF005739

Tissue	Identification	# of reads (stress)	# of reads (control)	Fold Change	p-value	Description
						InterPro:IPR011991 Gene3D:G3DSA:1.10.10.10 GO:GO:0046983 GO:GO:0008171 GO:GO:0008757 EMBL:EF189708 EMBL:CM000765 RefSeq:XP_002447425.1 UniGene:Sbi.19698 ProteinModelPortal:A8QW53 EnsemblPlants:Sb06g000820.1 GeneID:8080259 KEGG:sbi:SORBI_06g000820 eggNOG:NOG272168 PhylomeDB:A8QW53 ProtClustDB:CLSN2725062 Uniprot:A8QW53
Root	Isotig03522	1	47	-5.684	2.93E-13	UNIPROTKB|A8QW53 - symbol:OMT3 "5-pentadecatrienyl resorcinol O-methyltransferase" species:4558 "Sorghum bicolor" [GO:0008171 "O-methyltransferase activity" evidence=IDA] [GO:0008757 "S-adenosylmethionine-dependent methyltransferase activity" evidence=IDA] [GO:0032259 "methylation" evidence=IDA] InterPro:IPR001077 InterPro:IPR012967 InterPro:IPR016461 Pfam:PF00891 Pfam:PF08100 PIRSF:PIRSF005739 InterPro:IPR011991 Gene3D:G3DSA:1.10.10.10 GO:GO:0046983 GO:GO:0008171 GO:GO:0008757 EMBL:EF189708 EMBL:CM000765 RefSeq:XP_002447425.1 UniGene:Sbi.19698 ProteinModelPortal:A8QW53 EnsemblPlants:Sb06g000820.1 GeneID:8080259 KEGG:sbi:SORBI_06g000820 eggNOG:NOG272168 PhylomeDB:A8QW53 ProtClustDB:CLSN2725062 Uniprot:A8QW53
Root	Isotig03523	1	46	-5.653	5.25E-13	UNIPROTKB|A8QW53 - symbol:OMT3 "5-pentadecatrienyl resorcinol O-methyltransferase" species:4558 "Sorghum bicolor" [GO:0008171 "O-methyltransferase activity" evidence=IDA] [GO:0008757 "S-adenosylmethionine-dependent methyltransferase activity" evidence=IDA] [GO:0032259 "methylation" evidence=IDA] InterPro:IPR001077 InterPro:IPR012967 InterPro:IPR016461 Pfam:PF00891 Pfam:PF08100 PIRSF:PIRSF005739 InterPro:IPR011991 Gene3D:G3DSA:1.10.10.10 GO:GO:0046983 GO:GO:0008171 GO:GO:0008757 EMBL:EF189708 EMBL:CM000765 RefSeq:XP_002447425.1 UniGene:Sbi.19698 ProteinModelPortal:A8QW53 EnsemblPlants:Sb06g000820.1 GeneID:8080259 KEGG:sbi:SORBI_06g000820 eggNOG:NOG272168 PhylomeDB:A8QW53 ProtClustDB:CLSN2725062 Uniprot:A8QW53
Root	Isotig03524	52	106	-1.156	1.02E-06	TAIR|locus:2120222 - symbol:RD19 "AT4G39090" species:3702 "Arabidopsis thaliana" [GO:0006508 "proteolysis" evidence=ISS] [GO:0008234 "cysteine-type peptidase activity" evidence=ISS] [GO:0009269 "response to desiccation" evidence=IEP] [GO:0006970 "response to osmotic stress" evidence=IGI] [GO:0005634 "nucleus" evidence=IDA] [GO:0005773 "vacuole" evidence=IDA] [GO:0042742 "defense response to bacterium" evidence=IMP] [GO:0004197 "cysteine-type endopeptidase activity" evidence=ISS] [GO:0009414 "response to water deprivation" evidence=TAS] [GO:0009651 "response to salt stress" evidence=IEP] InterPro:IPR000668 InterPro:IPR013128 Pfam:PF00112 PRINTS:PR00705 SMART:SM00645 InterPro:IPR000169 EMBL:CP002687 GenomeReviews:CT486007_GR GO:GO:0005634 GO:GO:0005773 GO:GO:0042742 eggNOG:COG4870 HOGENOM:HBG746690 ProtClustDB:CLSN2688311 GO:GO:0004197 GO:GO:0006508 InterPro:IPR013201 PANTHER:PTHR12411 Pfam:PF08246 SMART:SM00848 PROSITE:PS00640 PROSITE:PS00139 PROSITE:PS00639 GO:GO:0009651 EMBL:AL035679 EMBL:AL161594 MEROPS:C01.022 EMBL:D13042 EMBL:AY080598 EMBL:AY133844 IPI:IPI00544363 PIR:JN0718 RefSeq:NP_568052.1 UniGene:At.2850 UniGene:At.74924 ProteinModelPortal:P43296 SMR:P43296 STRING:P43296 PRIDE:P43296 EnsemblPlants:AT4G39090.1 GeneID:830064 KEGG:ath:AT4G39090 TAIR:At4g39090 InParanoid:P43296 OMA:EDFDWRD PhylomeDB:P43296 ArrayExpress:P43296 Genevestigator:P43296 GermOnline:AT4G39090 Uniprot:P43296
Root	Isotig03525	45	105	-1.351	3.91E-08	TAIR|locus:2120222 - symbol:RD19 "AT4G39090" species:3702 "Arabidopsis thaliana" [GO:0006508 "proteolysis" evidence=ISS] [GO:0008234 "cysteine-type peptidase activity" evidence=ISS] [GO:0009269 "response to desiccation" evidence=IEP] [GO:0006970 "response to osmotic stress" evidence=IGI] [GO:0005634 "nucleus" evidence=IDA] [GO:0005773 "vacuole" evidence=IDA] [GO:0042742 "defense response to bacterium" evidence=IMP] [GO:0004197 "cysteine-type endopeptidase activity" evidence=ISS] [GO:0009414 "response to water deprivation" evidence=TAS] [GO:0009651 "response to salt stress" evidence=IEP] InterPro:IPR000668 InterPro:IPR013128 Pfam:PF00112 PRINTS:PR00705 SMART:SM00645 InterPro:IPR000169 EMBL:CP002687 GenomeReviews:CT486007_GR GO:GO:0005634 GO:GO:0005773 GO:GO:0042742 eggNOG:COG4870 HOGENOM:HBG746690 ProtClustDB:CLSN2688311 GO:GO:0004197 GO:GO:0006508 InterPro:IPR013201 PANTHER:PTHR12411 Pfam:PF08246 SMART:SM00848 PROSITE:PS00640 PROSITE:PS00139 PROSITE:PS00639 GO:GO:0009651 EMBL:AL035679 EMBL:AL161594 MEROPS:C01.022 EMBL:D13042 EMBL:AY080598 EMBL:AY133844 IPI:IPI00544363 PIR:JN0718 RefSeq:NP_568052.1 UniGene:At.2850 UniGene:At.74924 ProteinModelPortal:P43296 SMR:P43296 STRING:P43296 PRIDE:P43296 EnsemblPlants:AT4G39090.1 GeneID:830064 KEGG:ath:AT4G39090 TAIR:At4g39090 InParanoid:P43296 OMA:EDFDWRD PhylomeDB:P43296 ArrayExpress:P43296 Genevestigator:P43296 GermOnline:AT4G39090 Uniprot:P43296

Tissue	Identification	# of reads (stress)	# of reads (control)	Fold Change	p-value	Description
Root	Isotig03536	35	10	1.678	0.000370903	TAIR|locus:2195077 - symbol:AT1G12760 "AT1G12760" species:3702 "Arabidopsis thaliana" [GO:0008270 "zinc ion binding" evidence=ISS] [GO:0004842 "ubiquitin-protein ligase activity" evidence=IDA] InterPro:IPR001841 PROSITE:PS50089 SMART:SM00184 Prosite:PS00518 GO:GO:0016021 EMBL:CP002684 GenomeReviews:CT485782_GR GO:GO:0008270 InterPro:IPR013083 Gene3D:G3DSA:3.30.40.10 HSSP:Q9LRB7 GO:GO:0004842 EMBL:AC025417 eggNOG:NOG277561 HOGENOM:HBG750480 ProtClustDB:CLSN2682050 EMBL:DQ059089 EMBL:AY136370 EMBL:BT000279 IPI:IPI00516637 RefSeq:NP_172736.2 UniGene:At.43884 ProteinModelPortal:Q9LN71 SMR:Q9LN71 PRIDE:Q9LN71 EnsemblPlants:AT1G12760.1 GeneID:837832 KEGG:ath:AT1G12760 TAIR:At1g12760 InParanoid:Q9LN71 OMA:IVWNLAF PhylomeDB:Q9LN71 Genevestigator:Q9LN71 Uniprot:Q9LN71
Root	Isotig03537	38	10	1.797	9.99E-05	TAIR|locus:2195077 - symbol:AT1G12760 "AT1G12760" species:3702 "Arabidopsis thaliana" [GO:0008270 "zinc ion binding" evidence=ISS] [GO:0004842 "ubiquitin-protein ligase activity" evidence=IDA] InterPro:IPR001841 PROSITE:PS50089 SMART:SM00184 Prosite:PS00518 GO:GO:0016021 EMBL:CP002684 GenomeReviews:CT485782_GR GO:GO:0008270 InterPro:IPR013083 Gene3D:G3DSA:3.30.40.10 HSSP:Q9LRB7 GO:GO:0004842 EMBL:AC025417 eggNOG:NOG277561 HOGENOM:HBG750480 ProtClustDB:CLSN2682050 EMBL:DQ059089 EMBL:AY136370 EMBL:BT000279 IPI:IPI00516637 RefSeq:NP_172736.2 UniGene:At.43884 ProteinModelPortal:Q9LN71 SMR:Q9LN71 PRIDE:Q9LN71 EnsemblPlants:AT1G12760.1 GeneID:837832 KEGG:ath:AT1G12760 TAIR:At1g12760 InParanoid:Q9LN71 OMA:IVWNLAF PhylomeDB:Q9LN71 Genevestigator:Q9LN71 Uniprot:Q9LN71
Root	Isotig03538	39	10	1.835	6.39E-05	TAIR|locus:2195077 - symbol:AT1G12760 "AT1G12760" species:3702 "Arabidopsis thaliana" [GO:0008270 "zinc ion binding" evidence=ISS] [GO:0004842 "ubiquitin-protein ligase activity" evidence=IDA] InterPro:IPR001841 PROSITE:PS50089 SMART:SM00184 Prosite:PS00518 GO:GO:0016021 EMBL:CP002684 GenomeReviews:CT485782_GR GO:GO:0008270 InterPro:IPR013083 Gene3D:G3DSA:3.30.40.10 HSSP:Q9LRB7 GO:GO:0004842 EMBL:AC025417 eggNOG:NOG277561 HOGENOM:HBG750480 ProtClustDB:CLSN2682050 EMBL:DQ059089 EMBL:AY136370 EMBL:BT000279 IPI:IPI00516637 RefSeq:NP_172736.2 UniGene:At.43884 ProteinModelPortal:Q9LN71 SMR:Q9LN71 PRIDE:Q9LN71 EnsemblPlants:AT1G12760.1 GeneID:837832 KEGG:ath:AT1G12760 TAIR:At1g12760 InParanoid:Q9LN71 OMA:IVWNLAF PhylomeDB:Q9LN71 Genevestigator:Q9LN71 Uniprot:Q9LN71
Root	Isotig03542	14	0	4.678	0.000190242	TAIR|locus:2177105 - symbol:ZIFL1 "zinc induced facilitator-like 1" species:3702 "Arabidopsis thaliana" [GO:0015520 "tetracycline:hydrogen antiporter activity" evidence=IEA] [GO:0080167 "response to karrikin" evidence=IEP] InterPro:IPR001958 InterPro:IPR011701 Pfam:PF07690 PRINTS:PR01035 GO:GO:0016021 GO:GO:0005886 EMBL:CP002688 GenomeReviews:BA000015_GR GO:GO:0055085 GO:GO:0080167 GO:GO:0005215 InterPro:IPR016196 SUPFAM:SSF103473 EMBL:AB011484 InterPro:IPR020846 PROSITE:PS50850 eggNOG:NOG309217 EMBL:AY039542 EMBL:AY062867 EMBL:AY102150 EMBL:AK318697 EMBL:AY086758 EMBL:AK221880 IPI:IPI00525646 IPI:IPI00540772 IPI:IPI00657118 IPI:IPI00971349 RefSeq:NP_001031877.1 RefSeq:NP_568290.3 RefSeq:NP_851036.1 UniGene:At.19822 ProteinModelPortal:Q94BZ1 STRING:Q94BZ1 PRIDE:Q94BZ1 EnsemblPlants:AT5G13750.1 GeneID:831220 KEGG:ath:AT5G13750 TAIR:At5g13750 InParanoid:Q94BZ1 OMA:VVFNTLF PhylomeDB:Q94BZ1 ProtClustDB:CLSN2679920 Genevestigator:Q94BZ1 Uniprot:Q94BZ1
Root	Isotig03543	14	0	4.678	0.000190242	TAIR|locus:2177105 - symbol:ZIFL1 "zinc induced facilitator-like 1" species:3702 "Arabidopsis thaliana" [GO:0015520 "tetracycline:hydrogen antiporter activity" evidence=IEA] [GO:0080167 "response to karrikin" evidence=IEP] InterPro:IPR001958 InterPro:IPR011701 Pfam:PF07690 PRINTS:PR01035 GO:GO:0016021 GO:GO:0005886 EMBL:CP002688 GenomeReviews:BA000015_GR GO:GO:0055085 GO:GO:0080167 GO:GO:0005215 InterPro:IPR016196 SUPFAM:SSF103473 EMBL:AB011484 InterPro:IPR020846 PROSITE:PS50850 eggNOG:NOG309217 EMBL:AY039542 EMBL:AY062867 EMBL:AY102150 EMBL:AK318697 EMBL:AY086758 EMBL:AK221880 IPI:IPI00525646 IPI:IPI00540772 IPI:IPI00657118 IPI:IPI00971349 RefSeq:NP_001031877.1 RefSeq:NP_568290.3 RefSeq:NP_851036.1 UniGene:At.19822 ProteinModelPortal:Q94BZ1 STRING:Q94BZ1 PRIDE:Q94BZ1 EnsemblPlants:AT5G13750.1 GeneID:831220 KEGG:ath:AT5G13750 TAIR:At5g13750 InParanoid:Q94BZ1 OMA:VVFNTLF PhylomeDB:Q94BZ1 ProtClustDB:CLSN2679920 Genevestigator:Q94BZ1 Uniprot:Q94BZ1
Root	Isotig03551	1	15	-4.036	9.83E-05	TAIR|locus:2159634 - symbol:AT5G10650 species:3702 "Arabidopsis thaliana" [GO:0008270 "zinc ion binding" evidence=IEA;ISS] InterPro:IPR001841 PROSITE:PS50089 SMART:SM00184 EMBL:CP002688 GO:GO:0008270 InterPro:IPR013083 Gene3D:G3DSA:3.30.40.10 EMBL:BT015918 EMBL:BT020582 IPI:IPI00534438

Tissue	Identification	# of reads (stress)	# of reads (control)	Fold Change	p-value	Description
						RefSeq:NP_001031869.1 RefSeq:NP_196626.2 UniGene:At.32350 ProteinModelPortal:Q5XEP8 SMR:Q5XEP8 PRIDE:Q5XEP8 EnsemblPlants:AT5G10650.1 EnsemblPlants:AT5G10650.2 GeneID:830929 KEGG:ath:AT5G10650 TAIR:At5g10650 HOGENOM:HBG316343 InParanoid:Q5XEP8 OMA:ISSHGNG PhylomeDB:Q5XEP8 ProtClustDB:CLSN2690147 Genevestigator:Q5XEP8 Uniprot:Q5XEP8
Root	Isotig03552	1	15	-4.036	9.83E-05	TAIR|locus:2159634 - symbol:AT5G10650 species:3702 "Arabidopsis thaliana" [GO:0008270 "zinc ion binding" evidence=IEA;ISS] InterPro:IPR001841 PROSITE:PS50089 SMART:SM00184 EMBL:CP002688 GO:GO:0008270 InterPro:IPR013083 Gene3D:G3DSA:3.30.40.10 EMBL:BT015918 EMBL:BT020582 IPI:IPI00534438 RefSeq:NP_001031869.1 RefSeq:NP_196626.2 UniGene:At.32350 ProteinModelPortal:Q5XEP8 SMR:Q5XEP8 PRIDE:Q5XEP8 EnsemblPlants:AT5G10650.1 EnsemblPlants:AT5G10650.2 GeneID:830929 KEGG:ath:AT5G10650 TAIR:At5g10650 HOGENOM:HBG316343 InParanoid:Q5XEP8 OMA:ISSHGNG PhylomeDB:Q5XEP8 ProtClustDB:CLSN2690147 Genevestigator:Q5XEP8 Uniprot:Q5XEP8
Root	Isotig03553	1	14	-3.936	0.000188601	TAIR|locus:2159634 - symbol:AT5G10650 species:3702 "Arabidopsis thaliana" [GO:0008270 "zinc ion binding" evidence=IEA;ISS] InterPro:IPR001841 PROSITE:PS50089 SMART:SM00184 EMBL:CP002688 GO:GO:0008270 InterPro:IPR013083 Gene3D:G3DSA:3.30.40.10 EMBL:BT015918 EMBL:BT020582 IPI:IPI00534438 RefSeq:NP_001031869.1 RefSeq:NP_196626.2 UniGene:At.32350 ProteinModelPortal:Q5XEP8 SMR:Q5XEP8 PRIDE:Q5XEP8 EnsemblPlants:AT5G10650.1 EnsemblPlants:AT5G10650.2 GeneID:830929 KEGG:ath:AT5G10650 TAIR:At5g10650 HOGENOM:HBG316343 InParanoid:Q5XEP8 OMA:ISSHGNG PhylomeDB:Q5XEP8 ProtClustDB:CLSN2690147 Genevestigator:Q5XEP8 Uniprot:Q5XEP8
Root	Isotig03566	15	44	-1.682	2.92E-05	TAIR|locus:2082642 - symbol:PIP2;5 "plasma membrane intrinsic protein 2;5" species:3702 "Arabidopsis thaliana" [GO:0006810 "transport" evidence=IEA;ISS] [GO:0015250 "water channel activity" evidence=ISS] [GO:0016020 "membrane" evidence=IEA;ISS] [GO:0016021 "integral to membrane" evidence=IEA] [GO:0055085 "transmembrane transport" evidence=IEA] [GO:0005515 "protein binding" evidence=IPI] InterPro:IPR000425 InterPro:IPR012269 InterPro:IPR022357 Pfam:PF00230 PRINTS:PR00783 PROSITE:PS00221 GO:GO:0016021 GO:GO:0005886 EMBL:CP002686 GenomeReviews:BA000014_GR GO:GO:0005515 EMBL:AL049655 GO:GO:0055085 GO:GO:0005215 GO:GO:0006833 InterPro:IPR023271 Gene3D:G3DSA:1.20.1080.10 SUPFAM:SSF81338 eggNOG:COG0580 HOGENOM:HBG705794 PANTHER:PTHR19139 TIGRFAMs:TIGR00861 KO:K09872 EMBL:AF370351 EMBL:AY062981 EMBL:BT000670 EMBL:AY084843 IPI:IPI00520414 PIR:T06738 RefSeq:NP_191042.1 UniGene:At.3117 ProteinModelPortal:Q9SV31 SMR:Q9SV31 IntAct:Q9SV31 PRIDE:Q9SV31 ProMEX:Q9SV31 EnsemblPlants:AT3G54820.1 GeneID:824647 KEGG:ath:AT3G54820 GeneFarm:4870 TAIR:At3g54820 InParanoid:Q9SV31 OMA:FIVHLAT PhylomeDB:Q9SV31 ProtClustDB:CLSN2915746 ArrayExpress:Q9SV31 Genevestigator:Q9SV31 GermOnline:AT3G54820 Uniprot:Q9SV31
Root	Isotig03567	12	34	-1.631	0.000323514	TAIR|locus:2082642 - symbol:PIP2;5 "plasma membrane intrinsic protein 2;5" species:3702 "Arabidopsis thaliana" [GO:0006810 "transport" evidence=IEA;ISS] [GO:0015250 "water channel activity" evidence=ISS] [GO:0016020 "membrane" evidence=IEA;ISS] [GO:0016021 "integral to membrane" evidence=IEA] [GO:0055085 "transmembrane transport" evidence=IEA] [GO:0005515 "protein binding" evidence=IPI] InterPro:IPR000425 InterPro:IPR012269 InterPro:IPR022357 Pfam:PF00230 PRINTS:PR00783 PROSITE:PS00221 GO:GO:0016021 GO:GO:0005886 EMBL:CP002686 GenomeReviews:BA000014_GR GO:GO:0005515 EMBL:AL049655 GO:GO:0055085 GO:GO:0005215 GO:GO:0006833 InterPro:IPR023271 Gene3D:G3DSA:1.20.1080.10 SUPFAM:SSF81338 eggNOG:COG0580 HOGENOM:HBG705794 PANTHER:PTHR19139 TIGRFAMs:TIGR00861 KO:K09872 EMBL:AF370351 EMBL:AY062981 EMBL:BT000670 EMBL:AY084843 IPI:IPI00520414 PIR:T06738 RefSeq:NP_191042.1 UniGene:At.3117 ProteinModelPortal:Q9SV31 SMR:Q9SV31 IntAct:Q9SV31 PRIDE:Q9SV31 ProMEX:Q9SV31 EnsemblPlants:AT3G54820.1 GeneID:824647 KEGG:ath:AT3G54820 GeneFarm:4870 TAIR:At3g54820 InParanoid:Q9SV31 OMA:FIVHLAT PhylomeDB:Q9SV31 ProtClustDB:CLSN2915746 ArrayExpress:Q9SV31 Genevestigator:Q9SV31 GermOnline:AT3G54820 Uniprot:Q9SV31
Root	Isotig03568	9	33	-2.003	4.53E-05	TAIR|locus:2082642 - symbol:PIP2;5 "plasma membrane intrinsic protein 2;5" species:3702 "Arabidopsis thaliana" [GO:0006810 "transport" evidence=IEA;ISS] [GO:0015250 "water channel activity" evidence=ISS] [GO:0016020 "membrane" evidence=IEA;ISS] [GO:0016021 "integral to membrane" evidence=IEA] [GO:0055085 "transmembrane transport" evidence=IEA] [GO:0005515 "protein binding" evidence=IPI] InterPro:IPR000425 InterPro:IPR012269 InterPro:IPR022357 Pfam:PF00230 PRINTS:PR00783 PROSITE:PS00221 GO:GO:0016021 GO:GO:0005886 EMBL:CP002686 GenomeReviews:BA000014_GR GO:GO:0005515 EMBL:AL049655 GO:GO:0055085 GO:GO:0005215 GO:GO:0006833 InterPro:IPR023271 Gene3D:G3DSA:1.20.1080.10 SUPFAM:SSF81338 eggNOG:COG0580 HOGENOM:HBG705794 PANTHER:PTHR19139 TIGRFAMs:TIGR00861 KO:K09872

Tissue	Identification	# of reads (stress)	# of reads (control)	Fold Change	p-value	Description
						EMBL:AF370351 EMBL:AY062981 EMBL:BT000670 EMBL:AY084843 IPI:IPI00520414 PIR:T06738 RefSeq:NP_191042.1 UniGene:At.3117 ProteinModelPortal:Q9SV31 SMR:Q9SV31 IntAct:Q9SV31 PRIDE:Q9SV31 ProMEX:Q9SV31 EnsemblPlants:AT3G54820.1 GeneID:824647 KEGG:ath:AT3G54820 GeneFarm:4870 TAIR:At3g54820 InParanoid:Q9SV31 OMA:FIVHLAT PhylomeDB:Q9SV31 ProtClustDB:CLSN2915746 ArrayExpress:Q9SV31 Genevestigator:Q9SV31 GermOnline:AT3G54820 Uniprot:Q9SV31
Root	Isotig03581	0	18	-5.299	6.77E-06	TAIR|locus:2183730 - symbol:AT5G10770 "AT5G10770" species:3702 "Arabidopsis thaliana" [GO:0003677 "DNA binding" evidence=ISS] [GO:0004190 "aspartic-type endopeptidase activity" evidence=IEA] [GO:0006508 "proteolysis" evidence=IEA] InterPro:IPR001461 InterPro:IPR009007 Pfam:PF00026 PRINTS:PR00792 EMBL:CP002688 GO:GO:0006508 GO:GO:0004190 InterPro:IPR021109 SUPFAM:SSF50630 Gene3D:G3DSA:2.40.70.10 PANTHER:PTHR13683 HSSP:P00799 EMBL:AY075656 EMBL:BT001004 IPI:IPI00543783 RefSeq:NP_196638.2 UniGene:At.32336 ProteinModelPortal:Q8S9J6 MEROPS:A01.A15 PRIDE:Q8S9J6 EnsemblPlants:AT5G10770.1 GeneID:830944 KEGG:ath:AT5G10770 TAIR:At5g10770 InParanoid:Q8S9J6 OMA:LEVVYDG PhylomeDB:Q8S9J6 ProtClustDB:CLSN2686424 Genevestigator:Q8S9J6 Uniprot:Q8S9J6
Root	Isotig03582	0	18	-5.299	6.77E-06	TAIR|locus:2183730 - symbol:AT5G10770 "AT5G10770" species:3702 "Arabidopsis thaliana" [GO:0003677 "DNA binding" evidence=ISS] [GO:0004190 "aspartic-type endopeptidase activity" evidence=IEA] [GO:0006508 "proteolysis" evidence=IEA] InterPro:IPR001461 InterPro:IPR009007 Pfam:PF00026 PRINTS:PR00792 EMBL:CP002688 GO:GO:0006508 GO:GO:0004190 InterPro:IPR021109 SUPFAM:SSF50630 Gene3D:G3DSA:2.40.70.10 PANTHER:PTHR13683 HSSP:P00799 EMBL:AY075656 EMBL:BT001004 IPI:IPI00543783 RefSeq:NP_196638.2 UniGene:At.32336 ProteinModelPortal:Q8S9J6 MEROPS:A01.A15 PRIDE:Q8S9J6 EnsemblPlants:AT5G10770.1 GeneID:830944 KEGG:ath:AT5G10770 TAIR:At5g10770 InParanoid:Q8S9J6 OMA:LEVVYDG PhylomeDB:Q8S9J6 ProtClustDB:CLSN2686424 Genevestigator:Q8S9J6 Uniprot:Q8S9J6
Root	Isotig03583	0	15	-5.036	4.38E-05	TAIR|locus:2183730 - symbol:AT5G10770 "AT5G10770" species:3702 "Arabidopsis thaliana" [GO:0003677 "DNA binding" evidence=ISS] [GO:0004190 "aspartic-type endopeptidase activity" evidence=IEA] [GO:0006508 "proteolysis" evidence=IEA] InterPro:IPR001461 InterPro:IPR009007 Pfam:PF00026 PRINTS:PR00792 EMBL:CP002688 GO:GO:0006508 GO:GO:0004190 InterPro:IPR021109 SUPFAM:SSF50630 Gene3D:G3DSA:2.40.70.10 PANTHER:PTHR13683 HSSP:P00799 EMBL:AY075656 EMBL:BT001004 IPI:IPI00543783 RefSeq:NP_196638.2 UniGene:At.32336 ProteinModelPortal:Q8S9J6 MEROPS:A01.A15 PRIDE:Q8S9J6 EnsemblPlants:AT5G10770.1 GeneID:830944 KEGG:ath:AT5G10770 TAIR:At5g10770 InParanoid:Q8S9J6 OMA:LEVVYDG PhylomeDB:Q8S9J6 ProtClustDB:CLSN2686424 Genevestigator:Q8S9J6 Uniprot:Q8S9J6
Root	Isotig03608	1	21	-4.521	2.07E-06	TAIR|locus:2060984 - symbol:HIS1-3 "histone H1-3" species:3702 "Arabidopsis thaliana" [GO:0000786 "nucleosome" evidence=IEA;ISS] [GO:0003677 "DNA binding" evidence=ISS] [GO:0005634 "nucleus" evidence=IEA] [GO:0006334 "nucleosome assembly" evidence=ISS] [GO:0009414 "response to water deprivation" evidence=IEP] [GO:0031492 "nucleosomal DNA binding" evidence=ISS] InterPro:IPR005818 InterPro:IPR005819 Pfam:PF00538 PRINTS:PR00624 PROSITE:PS51504 SMART:SM00526 GO:GO:0005634 EMBL:CP002685 GO:GO:0003677 InterPro:IPR011991 Gene3D:G3DSA:1.10.10.10 GO:GO:0009414 GO:GO:0006334 GO:GO:0000786 EMBL:AC006201 KO:K11275 EMBL:U72241 EMBL:U73781 EMBL:AY045797 EMBL:AY079414 EMBL:AY084603 IPI:IPI00539258 PIR:F84559 RefSeq:NP_179396.1 UniGene:At.24439 ProteinModelPortal:P94109 SMR:P94109 STRING:P94109 PRIDE:P94109 EnsemblPlants:AT2G18050.1 GeneID:816317 KEGG:ath:AT2G18050 TAIR:At2g18050 eggNOG:NOG258576 InParanoid:P94109 OMA:TKRTRSS PhylomeDB:P94109 ProtClustDB:CLSN2683930 ArrayExpress:P94109 Genevestigator:P94109 Uniprot:P94109
Root	Isotig03609	1	21	-4.521	2.07E-06	TAIR|locus:2060984 - symbol:HIS1-3 "histone H1-3" species:3702 "Arabidopsis thaliana" [GO:0000786 "nucleosome" evidence=IEA;ISS] [GO:0003677 "DNA binding" evidence=ISS] [GO:0005634 "nucleus" evidence=IEA] [GO:0006334 "nucleosome assembly" evidence=ISS] [GO:0009414 "response to water deprivation" evidence=IEP] [GO:0031492 "nucleosomal DNA binding" evidence=ISS] InterPro:IPR005818 InterPro:IPR005819 Pfam:PF00538 PRINTS:PR00624 PROSITE:PS51504 SMART:SM00526 GO:GO:0005634 EMBL:CP002685 GO:GO:0003677 InterPro:IPR011991 Gene3D:G3DSA:1.10.10.10 GO:GO:0009414 GO:GO:0006334 GO:GO:0000786 EMBL:AC006201 KO:K11275 EMBL:U72241 EMBL:U73781 EMBL:AY045797 EMBL:AY079414 EMBL:AY084603 IPI:IPI00539258 PIR:F84559 RefSeq:NP_179396.1 UniGene:At.24439

Tissue	Identification	# of reads (stress)	# of reads (control)	Fold Change	p-value	Description
						ProteinModelPortal:P94109 SMR:P94109 STRING:P94109 PRIDE:P94109 EnsemblPlants:AT2G18050.1 GeneID:816317 KEGG:ath:AT2G18050 TAIR:At2g18050 eggNOG:NOG258576 InParanoid:P94109 OMA:TKRTRSS PhylomeDB:P94109 ProtClustDB:CLSN2683930 ArrayExpress:P94109 Genevestigator:P94109 Uniprot:P94109
Root	Isotig03610	0	18	-5.299	6.77E-06	TAIR|locus:2064332 - symbol:AT2G30620 "AT2G30620" species:3702 "Arabidopsis thaliana" [GO:0000786 "nucleosome" evidence=ISS] [GO:0003677 "DNA binding" evidence=IEA;ISS] [GO:0005634 "nucleus" evidence=ISS] [GO:0006334 "nucleosome assembly" evidence=IEA;ISS] [GO:0005730 "nucleolus" evidence=IDA] InterPro:IPR005818 InterPro:IPR005819 Pfam:PF00538 PRINTS:PR00624 PROSITE:PS51504 SMART:SM00526 EMBL:CP002685 GenomeReviews:CT485783_GR GO:GO:0005730 GO:GO:0003677 InterPro:IPR011991 Gene3D:G3DSA:1.10.10.10 GO:GO:0006334 GO:GO:0000786 EMBL:U93215 KO:K11275 eggNOG:NOG126832 EMBL:X62459 EMBL:AF360211 EMBL:AY040059 EMBL:AY085789 IPI:IPI00532307 PIR:S19699 RefSeq:NP_180620.1 UniGene:At.20406 ProteinModelPortal:P26569 STRING:P26569 PRIDE:P26569 EnsemblPlants:AT2G30620.1 GeneID:817612 KEGG:ath:AT2G30620 TAIR:At2g30620 HOGENOM:HBG184949 InParanoid:P26569 OMA:HRTPSSH PhylomeDB:P26569 ProtClustDB:CLSN2912930 ArrayExpress:P26569 Genevestigator:P26569 GermOnline:AT2G30620 Uniprot:P26569
Root	Isotig03665	54	0	6.626	1.30E-13	TAIR|locus:2202965 - symbol:MSL6 "AT1G78610" species:3702 "Arabidopsis thaliana" [GO:0016020 "membrane" evidence=ISS] [GO:0055085 "transmembrane transport" evidence=IEA] [GO:0005886 "plasma membrane" evidence=IDA] [GO:0005774 "vacuolar membrane" evidence=IDA] [GO:0009506 "plasmodesma" evidence=IDA] InterPro:IPR006685 Pfam:PF00924 GO:GO:0016021 EMBL:CP002684 GenomeReviews:CT485782_GR GO:GO:0005886 GO:GO:0009506 GO:GO:0005774 GO:GO:0005216 InterPro:IPR010920 SUPFAM:SSF50182 eggNOG:COG0668 HOGENOM:HBG316372 InterPro:IPR016688 PIRSF:PIRSF017209 ProtClustDB:CLSN2679546 EMBL:AC007260 EMBL:BX813849 IPI:IPI00538543 PIR:G96814 RefSeq:NP_177982.1 UniGene:At.34269 ProteinModelPortal:Q9SYM1 PRIDE:Q9SYM1 EnsemblPlants:AT1G78610.1 GeneID:844197 KEGG:ath:AT1G78610 TAIR:At1g78610 InParanoid:Q9SYM1 OMA:KQRITSY PhylomeDB:Q9SYM1 Genevestigator:Q9SYM1 Uniprot:Q9SYM1
Root	Isotig03666	53	0	6.599	2.10E-13	TAIR|locus:2202965 - symbol:MSL6 "AT1G78610" species:3702 "Arabidopsis thaliana" [GO:0016020 "membrane" evidence=ISS] [GO:0055085 "transmembrane transport" evidence=IEA] [GO:0005886 "plasma membrane" evidence=IDA] [GO:0005774 "vacuolar membrane" evidence=IDA] [GO:0009506 "plasmodesma" evidence=IDA] InterPro:IPR006685 Pfam:PF00924 GO:GO:0016021 EMBL:CP002684 GenomeReviews:CT485782_GR GO:GO:0005886 GO:GO:0009506 GO:GO:0005774 GO:GO:0005216 InterPro:IPR010920 SUPFAM:SSF50182 eggNOG:COG0668 HOGENOM:HBG316372 InterPro:IPR016688 PIRSF:PIRSF017209 ProtClustDB:CLSN2679546 EMBL:AC007260 EMBL:BX813849 IPI:IPI00538543 PIR:G96814 RefSeq:NP_177982.1 UniGene:At.34269 ProteinModelPortal:Q9SYM1 PRIDE:Q9SYM1 EnsemblPlants:AT1G78610.1 GeneID:844197 KEGG:ath:AT1G78610 TAIR:At1g78610 InParanoid:Q9SYM1 OMA:KQRITSY PhylomeDB:Q9SYM1 Genevestigator:Q9SYM1 Uniprot:Q9SYM1
Root	Isotig03667	46	0	6.395	6.55E-12	TAIR|locus:2202965 - symbol:MSL6 "AT1G78610" species:3702 "Arabidopsis thaliana" [GO:0016020 "membrane" evidence=ISS] [GO:0055085 "transmembrane transport" evidence=IEA] [GO:0005886 "plasma membrane" evidence=IDA] [GO:0005774 "vacuolar membrane" evidence=IDA] [GO:0009506 "plasmodesma" evidence=IDA] InterPro:IPR006685 Pfam:PF00924 GO:GO:0016021 EMBL:CP002684 GenomeReviews:CT485782_GR GO:GO:0005886 GO:GO:0009506 GO:GO:0005774 GO:GO:0005216 InterPro:IPR010920 SUPFAM:SSF50182 eggNOG:COG0668 HOGENOM:HBG316372 InterPro:IPR016688 PIRSF:PIRSF017209 ProtClustDB:CLSN2679546 EMBL:AC007260 EMBL:BX813849 IPI:IPI00538543 PIR:G96814 RefSeq:NP_177982.1 UniGene:At.34269 ProteinModelPortal:Q9SYM1 PRIDE:Q9SYM1 EnsemblPlants:AT1G78610.1 GeneID:844197 KEGG:ath:AT1G78610 TAIR:At1g78610 InParanoid:Q9SYM1 OMA:KQRITSY PhylomeDB:Q9SYM1 Genevestigator:Q9SYM1 Uniprot:Q9SYM1
Root	Isotig03668	45	0	6.363	1.08E-11	TAIR|locus:2202965 - symbol:MSL6 "AT1G78610" species:3702 "Arabidopsis thaliana" [GO:0016020 "membrane" evidence=ISS] [GO:0055085 "transmembrane transport" evidence=IEA] [GO:0005886 "plasma membrane" evidence=IDA] [GO:0005774 "vacuolar membrane" evidence=IDA] [GO:0009506 "plasmodesma" evidence=IDA] InterPro:IPR006685 Pfam:PF00924 GO:GO:0016021 EMBL:CP002684 GenomeReviews:CT485782_GR GO:GO:0005886 GO:GO:0009506 GO:GO:0005774 GO:GO:0005216 InterPro:IPR010920 SUPFAM:SSF50182 eggNOG:COG0668 HOGENOM:HBG316372 InterPro:IPR016688 PIRSF:PIRSF017209 ProtClustDB:CLSN2679546 EMBL:AC007260 EMBL:BX813849 IPI:IPI00538543 PIR:G96814 RefSeq:NP_177982.1 UniGene:At.34269

Tissue	Identification	# of reads (stress)	# of reads (control)	Fold Change	p-value	Description
						ProteinModelPortal:Q9SYM1 PRIDE:Q9SYM1 EnsemblPlants:AT1G78610.1 GeneID:844197 KEGG:ath:AT1G78610 TAIR:At1g78610 InParanoid:Q9SYM1 OMA:KQRITSY PhylomeDB:Q9SYM1 Genevestigator:Q9SYM1 Uniprot:Q9SYM1
Root	Isotig03673	36	105	-1.673	1.24E-10	TAIR|locus:2063354 - symbol:ACO3 "AT2G05710" species:3702 "Arabidopsis thaliana" [GO:0003994 "aconitate hydratase activity" evidence=ISS;IMP] [GO:0005739 "mitochondrion" evidence=IDA] [GO:0006979 "response to oxidative stress" evidence=IMP;IDA] [GO:0009507 "chloroplast" evidence=IDA] [GO:0005618 "cell wall" evidence=IDA] [GO:0046686 "response to cadmium ion" evidence=IEP] [GO:0005829 "cytosol" evidence=RCA;NAS] [GO:0006101 "citrate metabolic process" evidence=IMP] [GO:0006102 "isocitrate metabolic process" evidence=IMP] [GO:0005524 "ATP binding" evidence=IDA] [GO:0005774 "vacuolar membrane" evidence=IDA] [GO:0009651 "response to salt stress" evidence=IEP] [GO:0009737 "response to abscisic acid stimulus" evidence=IEP] [GO:0005507 "copper ion binding" evidence=IDA] [GO:0009570 "chloroplast stroma" evidence=IDA] [GO:0005794 "Golgi apparatus" evidence=RCA] InterPro:IPR000573 InterPro:IPR001030 InterPro:IPR006249 InterPro:IPR015928 InterPro:IPR015931 InterPro:IPR015932 InterPro:IPR015934 InterPro:IPR015937 Pfam:PF00330 Pfam:PF00694 PRINTS:PR00415 Prosite:PS00450 GO:GO:0005829 GO:GO:0005739 GO:GO:0005524 GO:GO:0009737 GO:GO:0009570 GO:GO:0046686 GO:GO:0005774 GO:GO:0005618 EMBL:CP002685 GenomeReviews:CT485783_GR GO:GO:0006979 GO:GO:0005507 GO:GO:0009651 GO:GO:0051539 GO:GO:0006097 GO:GO:0006099 GO:GO:0006101 eggNOG:COG1048 HOGENOM:HBG289738 KO:K01681 ProtClustDB:PLN00070 GO:GO:0003994 GO:GO:0052632 GO:GO:0052633 GO:GO:0006102 InterPro:IPR018136 Gene3D:G3DSA:3.30.499.10 Gene3D:G3DSA:3.20.19.10 Gene3D:G3DSA:3.40.1060.10 PANTHER:PTHR11670 PANTHER:PTHR11670:SF1 SUPFAM:SSF52016 SUPFAM:SSF53732 TIGRFAMs:TIGR01341 PROSITE:PS01244 EMBL:AC007170 EMBL:AY136414 EMBL:BT008809 IPI:IPI00543590 PIR:B84471 RefSeq:NP_178634.2 UniGene:At.26759 UniGene:At.67769 HSSP:P21399 ProteinModelPortal:Q9SIB9 SMR:Q9SIB9 IntAct:Q9SIB9 STRING:Q9SIB9 ANU-2DPAGE:Q9SIB9 PRIDE:Q9SIB9 EnsemblPlants:AT2G05710.1 GeneID:815120 KEGG:ath:AT2G05710 GeneFarm:4276 TAIR:At2g05710 InParanoid:Q9SIB9 OMA:IKYLVQT PhylomeDB:Q9SIB9 Genevestigator:Q9SIB9 Uniprot:Q9SIB9
Root	Isotig03674	34	102	-1.714	1.12E-10	TAIR|locus:2063354 - symbol:ACO3 "AT2G05710" species:3702 "Arabidopsis thaliana" [GO:0003994 "aconitate hydratase activity" evidence=ISS;IMP] [GO:0005739 "mitochondrion" evidence=IDA] [GO:0006979 "response to oxidative stress" evidence=IMP;IDA] [GO:0009507 "chloroplast" evidence=IDA] [GO:0005618 "cell wall" evidence=IDA] [GO:0046686 "response to cadmium ion" evidence=IEP] [GO:0005829 "cytosol" evidence=RCA;NAS] [GO:0006101 "citrate metabolic process" evidence=IMP] [GO:0006102 "isocitrate metabolic process" evidence=IMP] [GO:0005524 "ATP binding" evidence=IDA] [GO:0005774 "vacuolar membrane" evidence=IDA] [GO:0009651 "response to salt stress" evidence=IEP] [GO:0009737 "response to abscisic acid stimulus" evidence=IEP] [GO:0005507 "copper ion binding" evidence=IDA] [GO:0009570 "chloroplast stroma" evidence=IDA] [GO:0005794 "Golgi apparatus" evidence=RCA] InterPro:IPR000573 InterPro:IPR001030 InterPro:IPR006249 InterPro:IPR015928 InterPro:IPR015931 InterPro:IPR015932 InterPro:IPR015934 InterPro:IPR015937 Pfam:PF00330 Pfam:PF00694 PRINTS:PR00415 Prosite:PS00450 GO:GO:0005829 GO:GO:0005739 GO:GO:0005524 GO:GO:0009737 GO:GO:0009570 GO:GO:0046686 GO:GO:0005774 GO:GO:0005618 EMBL:CP002685 GenomeReviews:CT485783_GR GO:GO:0006979 GO:GO:0005507 GO:GO:0009651 GO:GO:0051539 GO:GO:0006097 GO:GO:0006099 GO:GO:0006101 eggNOG:COG1048 HOGENOM:HBG289738 KO:K01681 ProtClustDB:PLN00070 GO:GO:0003994 GO:GO:0052632 GO:GO:0052633 GO:GO:0006102 InterPro:IPR018136 Gene3D:G3DSA:3.30.499.10 Gene3D:G3DSA:3.20.19.10 Gene3D:G3DSA:3.40.1060.10 PANTHER:PTHR11670 PANTHER:PTHR11670:SF1 SUPFAM:SSF52016 SUPFAM:SSF53732 TIGRFAMs:TIGR01341 PROSITE:PS01244 EMBL:AC007170 EMBL:AY136414 EMBL:BT008809 IPI:IPI00543590 PIR:B84471 RefSeq:NP_178634.2 UniGene:At.26759 UniGene:At.67769 HSSP:P21399 ProteinModelPortal:Q9SIB9 SMR:Q9SIB9 IntAct:Q9SIB9 STRING:Q9SIB9 ANU-2DPAGE:Q9SIB9 PRIDE:Q9SIB9 EnsemblPlants:AT2G05710.1 GeneID:815120 KEGG:ath:AT2G05710 GeneFarm:4276 TAIR:At2g05710 InParanoid:Q9SIB9 OMA:IKYLVQT PhylomeDB:Q9SIB9 Genevestigator:Q9SIB9 Uniprot:Q9SIB9
Root	Isotig03675	35	101	-1.658	3.63E-10	TAIR|locus:2063354 - symbol:ACO3 "AT2G05710" species:3702 "Arabidopsis thaliana" [GO:0003994 "aconitate hydratase activity" evidence=ISS;IMP] [GO:0005739 "mitochondrion" evidence=IDA] [GO:0006979 "response to oxidative stress" evidence=IMP;IDA] [GO:0009507 "chloroplast" evidence=IDA] [GO:0005618 "cell wall" evidence=IDA] [GO:0046686 "response to cadmium ion" evidence=IEP] [GO:0005829 "cytosol" evidence=RCA;NAS] [GO:0006101 "citrate metabolic process" evidence=IMP] [GO:0006102 "isocitrate metabolic

Tissue	Identification	# of reads (stress)	# of reads (control)	Fold Change	p-value	Description
						process" evidence=IMP] [GO:0005524 "ATP binding" evidence=IDA] [GO:0005774 "vacuolar membrane" evidence=IDA] [GO:0009651 "response to salt stress" evidence=IEP] [GO:0009737 "response to abscisic acid stimulus" evidence=IEP] [GO:0005507 "copper ion binding" evidence=IDA] [GO:0009570 "chloroplast stroma" evidence=IDA] [GO:0005794 "Golgi apparatus" evidence=RCA] InterPro:IPR000573 InterPro:IPR001030 InterPro:IPR006249 InterPro:IPR015928 InterPro:IPR015931 InterPro:IPR015932 InterPro:IPR015934 InterPro:IPR015937 Pfam:PF00330 Pfam:PF00694 PRINTS:PR00415 Prosite:PS00450 GO:GO:0005829 GO:GO:0005739 GO:GO:0005524 GO:GO:0009737 GO:GO:0009570 GO:GO:0046686 GO:GO:0005774 GO:GO:0005618 EMBL:CP002685 GenomeReviews:CT485783_GR GO:GO:0006979 GO:GO:0005507 GO:GO:0009651 GO:GO:0051539 GO:GO:0006097 GO:GO:0006099 GO:GO:0006101 eggNOG:COG1048 HOGENOM:HBG289738 KO:K01681 ProtClustDB:PLN00070 GO:GO:0003994 GO:GO:0052632 GO:GO:0052633 GO:GO:0006102 InterPro:IPR018136 Gene3D:G3DSA:3.30.499.10 Gene3D:G3DSA:3.20.19.10 Gene3D:G3DSA:3.40.1060.10 PANTHER:PTHR11670 PANTHER:PTHR11670:SF1 SUPFAM:SSF52016 SUPFAM:SSF53732 TIGRFAMs:TIGR01341 PROSITE:PS01244 EMBL:AC007170 EMBL:AY136414 EMBL:BT008809 IPI:IPI00543590 PIR:B84471 RefSeq:NP_178634.2 UniGene:At.26759 UniGene:At.67769 HSSP:P21399 ProteinModelPortal:Q9SIB9 SMR:Q9SIB9 IntAct:Q9SIB9 STRING:Q9SIB9 ANU-2DPAGE:Q9SIB9 PRIDE:Q9SIB9 EnsemblPlants:AT2G05710.1 GeneID:815120 KEGG:ath:AT2G05710 GeneFarm:4276 TAIR:At2g05710 InParanoid:Q9SIB9 OMA:IKYLVQT PhylomeDB:Q9SIB9 Genevestigator:Q9SIB9 Uniprot:Q9SIB9
Root	Isotig03676	33	98	-1.699	3.29E-10	TAIR|locus:2063354 - symbol:ACO3 "AT2G05710" species:3702 "Arabidopsis thaliana" [GO:0003994 "aconitate hydratase activity" evidence=ISS;IMP] [GO:0005739 "mitochondrion" evidence=IDA] [GO:0006979 "response to oxidative stress" evidence=IMP;IDA] [GO:0009507 "chloroplast" evidence=IDA] [GO:0005618 "cell wall" evidence=IDA] [GO:0046686 "response to cadmium ion" evidence=IEP] [GO:0005829 "cytosol" evidence=RCA;NAS] [GO:0006101 "citrate metabolic process" evidence=IMP] [GO:0006102 "isocitrate metabolic process" evidence=IMP] [GO:0005524 "ATP binding" evidence=IDA] [GO:0005774 "vacuolar membrane" evidence=IDA] [GO:0009651 "response to salt stress" evidence=IEP] [GO:0009737 "response to abscisic acid stimulus" evidence=IEP] [GO:0005507 "copper ion binding" evidence=IDA] [GO:0009570 "chloroplast stroma" evidence=IDA] [GO:0005794 "Golgi apparatus" evidence=RCA] InterPro:IPR000573 InterPro:IPR001030 InterPro:IPR006249 InterPro:IPR015928 InterPro:IPR015931 InterPro:IPR015932 InterPro:IPR015934 InterPro:IPR015937 Pfam:PF00330 Pfam:PF00694 PRINTS:PR00415 Prosite:PS00450 GO:GO:0005829 GO:GO:0005739 GO:GO:0005524 GO:GO:0009737 GO:GO:0009570 GO:GO:0046686 GO:GO:0005774 GO:GO:0005618 EMBL:CP002685 GenomeReviews:CT485783_GR GO:GO:0006979 GO:GO:0005507 GO:GO:0009651 GO:GO:0051539 GO:GO:0006097 GO:GO:0006099 GO:GO:0006101 eggNOG:COG1048 HOGENOM:HBG289738 KO:K01681 ProtClustDB:PLN00070 GO:GO:0003994 GO:GO:0052632 GO:GO:0052633 GO:GO:0006102 InterPro:IPR018136 Gene3D:G3DSA:3.30.499.10 Gene3D:G3DSA:3.20.19.10 Gene3D:G3DSA:3.40.1060.10 PANTHER:PTHR11670 PANTHER:PTHR11670:SF1 SUPFAM:SSF52016 SUPFAM:SSF53732 TIGRFAMs:TIGR01341 PROSITE:PS01244 EMBL:AC007170 EMBL:AY136414 EMBL:BT008809 IPI:IPI00543590 PIR:B84471 RefSeq:NP_178634.2 UniGene:At.26759 UniGene:At.67769 HSSP:P21399 ProteinModelPortal:Q9SIB9 SMR:Q9SIB9 IntAct:Q9SIB9 STRING:Q9SIB9 ANU-2DPAGE:Q9SIB9 PRIDE:Q9SIB9 EnsemblPlants:AT2G05710.1 GeneID:815120 KEGG:ath:AT2G05710 GeneFarm:4276 TAIR:At2g05710 InParanoid:Q9SIB9 OMA:IKYLVQT PhylomeDB:Q9SIB9 Genevestigator:Q9SIB9 Uniprot:Q9SIB9
Root	Isotig03685	86	35	1.168	2.21E-05	TAIR|locus:2205235 - symbol:LOS1 "AT1G56070" species:3702 "Arabidopsis thaliana" [GO:0003746 "translation elongation factor activity" evidence=ISS] [GO:0008135 "translation factor activity, nucleic acid binding" evidence=ISS] [GO:0009409 "response to cold" evidence=IMP] [GO:0005730 "nucleolus" evidence=IDA] [GO:0005507 "copper ion binding" evidence=IDA] [GO:0005774 "vacuolar membrane" evidence=IDA] [GO:0016020 "membrane" evidence=IDA] [GO:0005886 "plasma membrane" evidence=IDA] [GO:0009507 "chloroplast" evidence=IDA] [GO:0005829 "cytosol" evidence=IDA] [GO:0009506 "plasmodesma" evidence=IDA] [GO:0005794 "Golgi apparatus" evidence=RCA] InterPro:IPR000640 InterPro:IPR000795 InterPro:IPR004161 InterPro:IPR005225 InterPro:IPR005517 Pfam:PF00009 Pfam:PF00679 Pfam:PF03144 Pfam:PF03764 PRINTS:PR00315 PROSITE:PS00301 SMART:SM00838 SMART:SM00889 GO:GO:0005525 TIGRFAMs:TIGR00231 EMBL:CP002684 GO:GO:0005829 GO:GO:0005886 GO:GO:0009506 GO:GO:0005774 GO:GO:0009507 GO:GO:0005730 GO:GO:0003924 GO:GO:0005507 GO:GO:0009409 GO:GO:0003746 InterPro:IPR020568 InterPro:IPR014721 Gene3D:G3DSA:3.30.230.10 SUPFAM:SSF54211 InterPro:IPR009000 SUPFAM:SSF50447 InterPro:IPR009022 Gene3D:G3DSA:3.30.70.240 SUPFAM:SSF54980 KO:K03234 OMA:GVMTQTE HSSP:P32324 EMBL:AF367331 EMBL:AY054461 EMBL:BT000661 EMBL:BT000722 EMBL:BT002714 IPI:IPI00517335

Tissue	Identification	# of reads (stress)	# of reads (control)	Fold Change	p-value	Description
						RefSeq:NP_849818.1 UniGene:At.47544 ProteinModelPortal:Q9ASR1 SMR:Q9ASR1 STRING:Q9ASR1 PRIDE:Q9ASR1 ProMEX:Q9ASR1 EnsemblPlants:AT1G56070.1 GeneID:842058 KEGG:ath:AT1G56070 TAIR:At1g56070 InParanoid:Q9ASR1 PhylomeDB:Q9ASR1 ProtClustDB:PLN00116 Genevestigator:Q9ASR1 Uniprot:Q9ASR1
Root	Isotig03686	94	37	1.216	4.65E-06	TAIR|locus:2205235 - symbol:LOS1 "AT1G56070" species:3702 "Arabidopsis thaliana" [GO:0003746 "translation elongation factor activity" evidence=ISS] [GO:0008135 "translation factor activity, nucleic acid binding" evidence=ISS] [GO:0009409 "response to cold" evidence=IMP] [GO:0005730 "nucleolus" evidence=IDA] [GO:0005507 "copper ion binding" evidence=IDA] [GO:0005774 "vacuolar membrane" evidence=IDA] [GO:0016020 "membrane" evidence=IDA] [GO:0005886 "plasma membrane" evidence=IDA] [GO:0009507 "chloroplast" evidence=IDA] [GO:0005829 "cytosol" evidence=IDA] [GO:0009506 "plasmodesma" evidence=IDA] [GO:0005794 "Golgi apparatus" evidence=RCA] InterPro:IPR000640 InterPro:IPR000795 InterPro:IPR004161 InterPro:IPR005225 InterPro:IPR005517 Pfam:PF00009 Pfam:PF00679 Pfam:PF03144 Pfam:PF03764 PRINTS:PR00315 PROSITE:PS00301 SMART:SM00838 SMART:SM00889 GO:GO:0005525 TIGRFAMs:TIGR00231 EMBL:CP002684 GO:GO:0005829 GO:GO:0005886 GO:GO:0009506 GO:GO:0005774 GO:GO:0009507 GO:GO:0005730 GO:GO:0003924 GO:GO:0005507 GO:GO:0009409 GO:GO:0003746 InterPro:IPR020568 InterPro:IPR014721 Gene3D:G3DSA:3.30.230.10 SUPFAM:SSF54211 InterPro:IPR009000 SUPFAM:SSF50447 InterPro:IPR009022 Gene3D:G3DSA:3.30.70.240 SUPFAM:SSF54980 KO:K03234 OMA:GVMTQTE HSSP:P32324 EMBL:AF367331 EMBL:AY054461 EMBL:BT000661 EMBL:BT000722 EMBL:BT002714 IPI:IPI00517335 RefSeq:NP_849818.1 UniGene:At.47544 ProteinModelPortal:Q9ASR1 SMR:Q9ASR1 STRING:Q9ASR1 PRIDE:Q9ASR1 ProMEX:Q9ASR1 EnsemblPlants:AT1G56070.1 GeneID:842058 KEGG:ath:AT1G56070 TAIR:At1g56070 InParanoid:Q9ASR1 PhylomeDB:Q9ASR1 ProtClustDB:PLN00116 Genevestigator:Q9ASR1 Uniprot:Q9ASR1
Root	Isotig03693	13	36	-1.598	0.000266476	TAIR|locus:2061838 - symbol:KT2 "AT2G40540" species:3702 "Arabidopsis thaliana" [GO:0015079 "potassium ion transmembrane transporter activity" evidence=ISS;TAS] [GO:0006813 "potassium ion transport" evidence=IC] [GO:0016020 "membrane" evidence=NAS] [GO:0005886 "plasma membrane" evidence=IDA] InterPro:IPR003855 Pfam:PF02705 GO:GO:0016021 GO:GO:0005886 EMBL:CP002685 GenomeReviews:CT485783_GR GO:GO:0015079 EMBL:AC002336 eggNOG:COG3158 KO:K03549 TIGRFAMs:TIGR00794 HOGENOM:HBG606427 EMBL:AF012657 EMBL:BT004049 EMBL:BT005048 IPI:IPI00518306 PIR:G84830 RefSeq:NP_001078032.1 RefSeq:NP_565936.1 UniGene:At.10816 ProteinModelPortal:O22881 PRIDE:O22881 EnsemblPlants:AT2G40540.1 EnsemblPlants:AT2G40540.2 GeneID:818649 KEGG:ath:AT2G40540 GeneFarm:2394 TAIR:At2g40540 InParanoid:O22881 OMA:FKFDIMA PhylomeDB:O22881 ProtClustDB:CLSN2688842 ArrayExpress:O22881 Genevestigator:O22881 GermOnline:AT2G40540 Uniprot:O22881
Root	Isotig03694	13	36	-1.598	0.000266476	TAIR|locus:2061838 - symbol:KT2 "AT2G40540" species:3702 "Arabidopsis thaliana" [GO:0015079 "potassium ion transmembrane transporter activity" evidence=ISS;TAS] [GO:0006813 "potassium ion transport" evidence=IC] [GO:0016020 "membrane" evidence=NAS] [GO:0005886 "plasma membrane" evidence=IDA] InterPro:IPR003855 Pfam:PF02705 GO:GO:0016021 GO:GO:0005886 EMBL:CP002685 GenomeReviews:CT485783_GR GO:GO:0015079 EMBL:AC002336 eggNOG:COG3158 KO:K03549 TIGRFAMs:TIGR00794 HOGENOM:HBG606427 EMBL:AF012657 EMBL:BT004049 EMBL:BT005048 IPI:IPI00518306 PIR:G84830 RefSeq:NP_001078032.1 RefSeq:NP_565936.1 UniGene:At.10816 ProteinModelPortal:O22881 PRIDE:O22881 EnsemblPlants:AT2G40540.1 EnsemblPlants:AT2G40540.2 GeneID:818649 KEGG:ath:AT2G40540 GeneFarm:2394 TAIR:At2g40540 InParanoid:O22881 OMA:FKFDIMA PhylomeDB:O22881 ProtClustDB:CLSN2688842 ArrayExpress:O22881 Genevestigator:O22881 GermOnline:AT2G40540 Uniprot:O22881
Root	Isotig03705	42	0	6.263	4.87E-11	TAIR|locus:2125314 - symbol:PLDDELTA "AT4G35790" species:3702 "Arabidopsis thaliana" [GO:0004630 "phospholipase D activity" evidence=ISS;IMP;IDA] [GO:0046473 "phosphatidic acid metabolic process" evidence=IMP] [GO:0012501 "programmed cell death" evidence=IMP] [GO:0009409 "response to cold" evidence=IEP;IMP] [GO:0005773 "vacuole" evidence=IDA] [GO:0005886 "plasma membrane" evidence=IDA] [GO:0016020 "membrane" evidence=IDA] [GO:0009506 "plasmodesma" evidence=IDA] InterPro:IPR000008 InterPro:IPR001736 InterPro:IPR008973 InterPro:IPR011402 Pfam:PF00168 Pfam:PF00614 PIRSF:PIRSF036470 PROSITE:PS50035 SMART:SM00155 SMART:SM00239 GO:GO:0005886 GO:GO:0009506 EMBL:CP002687 GenomeReviews:CT486007_GR GO:GO:0005773 GO:GO:0005509 GO:GO:0046470 GO:GO:0016042

Tissue	Identification	# of reads (stress)	# of reads (control)	Fold Change	p-value	Description
						GO:GO:0009409 InterPro:IPR018029 SUPFAM:SSF49562 PROSITE:PS50004 EMBL:AL031986 EMBL:AL161588 GO:GO:0012501 eggNOG:COG1502 InterPro:IPR015679 PANTHER:PTHR18896 GO:GO:0070290 GO:GO:0004630 GO:GO:0046473 KO:K01115 HOGENOM:HBG748198 InterPro:IPR024632 Pfam:PF12357 EMBL:AF322228 EMBL:AF274239 EMBL:AF306345 EMBL:AB031047 EMBL:AF424632 EMBL:AK227213 IPI:IPI00520107 IPI:IPI00541650 RefSeq:NP_567989.1 RefSeq:NP_849501.1 UniGene:At.22959 ProteinModelPortal:Q9C5Y0 SMR:Q9C5Y0 STRING:Q9C5Y0 PRIDE:Q9C5Y0 EnsemblPlants:AT4G35790.1 GeneID:829733 KEGG:ath:AT4G35790 TAIR:At4g35790 InParanoid:Q9C5Y0 OMA:GRISWIL PhylomeDB:Q9C5Y0 ProtClustDB:PLN03008 ArrayExpress:Q9C5Y0 Genevestigator:Q9C5Y0 Uniprot:Q9C5Y0
Root	Isotig03706	42	0	6.263	4.87E-11	TAIR|locus:2125314 - symbol:PLDDELTA "AT4G35790" species:3702 "Arabidopsis thaliana" [GO:0004630 "phospholipase D activity" evidence=ISS;IMP;IDA] [GO:0046473 "phosphatidic acid metabolic process" evidence=IMP] [GO:0012501 "programmed cell death" evidence=IMP] [GO:0009409 "response to cold" evidence=IEP;IMP] [GO:0005773 "vacuole" evidence=IDA] [GO:0005886 "plasma membrane" evidence=IDA] [GO:0016020 "membrane" evidence=IDA] [GO:0009506 "plasmodesma" evidence=IDA] InterPro:IPR000008 InterPro:IPR001736 InterPro:IPR008973 InterPro:IPR011402 Pfam:PF00168 Pfam:PF00614 PIRSF:PIRSF036470 PROSITE:PS50035 SMART:SM00155 SMART:SM00239 GO:GO:0005886 GO:GO:0009506 EMBL:CP002687 GenomeReviews:CT486007_GR GO:GO:0005773 GO:GO:0005509 GO:GO:0046470 GO:GO:0016042 GO:GO:0009409 InterPro:IPR018029 SUPFAM:SSF49562 PROSITE:PS50004 EMBL:AL031986 EMBL:AL161588 GO:GO:0012501 eggNOG:COG1502 InterPro:IPR015679 PANTHER:PTHR18896 GO:GO:0070290 GO:GO:0004630 GO:GO:0046473 KO:K01115 HOGENOM:HBG748198 InterPro:IPR024632 Pfam:PF12357 EMBL:AF322228 EMBL:AF274239 EMBL:AF306345 EMBL:AB031047 EMBL:AF424632 EMBL:AK227213 IPI:IPI00520107 IPI:IPI00541650 RefSeq:NP_567989.1 RefSeq:NP_849501.1 UniGene:At.22959 ProteinModelPortal:Q9C5Y0 SMR:Q9C5Y0 STRING:Q9C5Y0 PRIDE:Q9C5Y0 EnsemblPlants:AT4G35790.1 GeneID:829733 KEGG:ath:AT4G35790 TAIR:At4g35790 InParanoid:Q9C5Y0 OMA:GRISWIL PhylomeDB:Q9C5Y0 ProtClustDB:PLN03008 ArrayExpress:Q9C5Y0 Genevestigator:Q9C5Y0 Uniprot:Q9C5Y0
Root	Isotig03707	42	0	6.263	4.87E-11	TAIR|locus:2125314 - symbol:PLDDELTA "AT4G35790" species:3702 "Arabidopsis thaliana" [GO:0004630 "phospholipase D activity" evidence=ISS;IMP;IDA] [GO:0046473 "phosphatidic acid metabolic process" evidence=IMP] [GO:0012501 "programmed cell death" evidence=IMP] [GO:0009409 "response to cold" evidence=IEP;IMP] [GO:0005773 "vacuole" evidence=IDA] [GO:0005886 "plasma membrane" evidence=IDA] [GO:0016020 "membrane" evidence=IDA] [GO:0009506 "plasmodesma" evidence=IDA] InterPro:IPR000008 InterPro:IPR001736 InterPro:IPR008973 InterPro:IPR011402 Pfam:PF00168 Pfam:PF00614 PIRSF:PIRSF036470 PROSITE:PS50035 SMART:SM00155 SMART:SM00239 GO:GO:0005886 GO:GO:0009506 EMBL:CP002687 GenomeReviews:CT486007_GR GO:GO:0005773 GO:GO:0005509 GO:GO:0046470 GO:GO:0016042 GO:GO:0009409 InterPro:IPR018029 SUPFAM:SSF49562 PROSITE:PS50004 EMBL:AL031986 EMBL:AL161588 GO:GO:0012501 eggNOG:COG1502 InterPro:IPR015679 PANTHER:PTHR18896 GO:GO:0070290 GO:GO:0004630 GO:GO:0046473 KO:K01115 HOGENOM:HBG748198 InterPro:IPR024632 Pfam:PF12357 EMBL:AF322228 EMBL:AF274239 EMBL:AF306345 EMBL:AB031047 EMBL:AF424632 EMBL:AK227213 IPI:IPI00520107 IPI:IPI00541650 RefSeq:NP_567989.1 RefSeq:NP_849501.1 UniGene:At.22959 ProteinModelPortal:Q9C5Y0 SMR:Q9C5Y0 STRING:Q9C5Y0 PRIDE:Q9C5Y0 EnsemblPlants:AT4G35790.1 GeneID:829733 KEGG:ath:AT4G35790 TAIR:At4g35790 InParanoid:Q9C5Y0 OMA:GRISWIL PhylomeDB:Q9C5Y0 ProtClustDB:PLN03008 ArrayExpress:Q9C5Y0 Genevestigator:Q9C5Y0 Uniprot:Q9C5Y0
Root	Isotig03708	42	0	6.263	4.87E-11	TAIR|locus:2125314 - symbol:PLDDELTA "AT4G35790" species:3702 "Arabidopsis thaliana" [GO:0004630 "phospholipase D activity" evidence=ISS;IMP;IDA] [GO:0046473 "phosphatidic acid metabolic process" evidence=IMP] [GO:0012501 "programmed cell death" evidence=IMP] [GO:0009409 "response to cold" evidence=IEP;IMP] [GO:0005773 "vacuole" evidence=IDA] [GO:0005886 "plasma membrane" evidence=IDA] [GO:0016020 "membrane" evidence=IDA] [GO:0009506 "plasmodesma" evidence=IDA] InterPro:IPR000008 InterPro:IPR001736 InterPro:IPR008973 InterPro:IPR011402 Pfam:PF00168 Pfam:PF00614 PIRSF:PIRSF036470 PROSITE:PS50035 SMART:SM00155 SMART:SM00239 GO:GO:0005886 GO:GO:0009506 EMBL:CP002687 GenomeReviews:CT486007_GR GO:GO:0005773 GO:GO:0005509 GO:GO:0046470 GO:GO:0016042 GO:GO:0009409 InterPro:IPR018029 SUPFAM:SSF49562 PROSITE:PS50004 EMBL:AL031986 EMBL:AL161588 GO:GO:0012501 eggNOG:COG1502 InterPro:IPR015679 PANTHER:PTHR18896 GO:GO:0070290 GO:GO:0004630 GO:GO:0046473 KO:K01115 HOGENOM:HBG748198 InterPro:IPR024632 Pfam:PF12357 EMBL:AF322228 EMBL:AF274239 EMBL:AF306345 EMBL:AB031047 EMBL:AF424632 EMBL:AK227213

Tissue	Identification	# of reads (stress)	# of reads (control)	Fold Change	p-value	Description
						IPI:IPI00520107 IPI:IPI00541650 RefSeq:NP_567989.1 RefSeq:NP_849501.1 UniGene:At.22959 ProteinModelPortal:Q9C5Y0 SMR:Q9C5Y0 STRING:Q9C5Y0 PRIDE:Q9C5Y0 EnsemblPlants:AT4G35790.1 GeneID:829733 KEGG:ath:AT4G35790 TAIR:At4g35790 InParanoid:Q9C5Y0 OMA:GRISWIL PhylomeDB:Q9C5Y0 ProtClustDB:PLN03008 ArrayExpress:Q9C5Y0 Genevestigator:Q9C5Y0 Uniprot:Q9C5Y0
Root	Isotig03713	0	63	-7.106	1.51E-16	TAIR|locus:2087837 - symbol:LOX5 species:3702 "Arabidopsis thaliana" [GO:0005506 "iron ion binding" evidence=IEA] [GO:0016165 "lipoxygenase activity" evidence=IEA;ISS;IDA] [GO:0016702 "oxidoreductase activity, acting on single donors with incorporation of molecular oxygen, incorporation of two atoms of oxygen" evidence=IEA] [GO:0046872 "metal ion binding" evidence=IEA] [GO:0009507 "chloroplast" evidence=IDA] [GO:0010386 "lateral root primordium development" evidence=IMP] [GO:0048364 "root development" evidence=IMP] InterPro:IPR000907 InterPro:IPR001024 InterPro:IPR001246 InterPro:IPR013819 InterPro:IPR020833 InterPro:IPR020834 Pfam:PF00305 Pfam:PF01477 PRINTS:PR00087 PRINTS:PR00468 PROSITE:PS00081 PROSITE:PS00711 PROSITE:PS50095 PROSITE:PS51393 SMART:SM00308 GO:GO:0009507 EMBL:CP002686 GenomeReviews:BA000014_GR GO:GO:0005506 GO:GO:0031408 EMBL:AB022215 GO:GO:0010386 InterPro:IPR008976 Gene3D:G3DSA:2.60.60.20 SUPFAM:SSF49723 GO:GO:0016165 PANTHER:PTHR11771 SUPFAM:SSF48484 eggNOG:NOG69653 KO:K15718 ProtClustDB:PLN02337 HSSP:P08170 EMBL:AJ302043 IPI:IPI00543707 RefSeq:NP_188879.2 UniGene:At.37889 ProteinModelPortal:Q9LUW0 SMR:Q9LUW0 STRING:Q9LUW0 PRIDE:Q9LUW0 EnsemblPlants:AT3G22400.1 GeneID:821808 KEGG:ath:AT3G22400 TAIR:At3g22400 InParanoid:Q9LUW0 OMA:FDWDESM PhylomeDB:Q9LUW0 Genevestigator:Q9LUW0 Uniprot:Q9LUW0
Root	Isotig03714	0	59	-7.012	1.12E-15	TAIR|locus:2087837 - symbol:LOX5 species:3702 "Arabidopsis thaliana" [GO:0005506 "iron ion binding" evidence=IEA] [GO:0016165 "lipoxygenase activity" evidence=IEA;ISS;IDA] [GO:0016702 "oxidoreductase activity, acting on single donors with incorporation of molecular oxygen, incorporation of two atoms of oxygen" evidence=IEA] [GO:0046872 "metal ion binding" evidence=IEA] [GO:0009507 "chloroplast" evidence=IDA] [GO:0010386 "lateral root primordium development" evidence=IMP] [GO:0048364 "root development" evidence=IMP] InterPro:IPR000907 InterPro:IPR001024 InterPro:IPR001246 InterPro:IPR013819 InterPro:IPR020833 InterPro:IPR020834 Pfam:PF00305 Pfam:PF01477 PRINTS:PR00087 PRINTS:PR00468 PROSITE:PS00081 PROSITE:PS00711 PROSITE:PS50095 PROSITE:PS51393 SMART:SM00308 GO:GO:0009507 EMBL:CP002686 GenomeReviews:BA000014_GR GO:GO:0005506 GO:GO:0031408 EMBL:AB022215 GO:GO:0010386 InterPro:IPR008976 Gene3D:G3DSA:2.60.60.20 SUPFAM:SSF49723 GO:GO:0016165 PANTHER:PTHR11771 SUPFAM:SSF48484 eggNOG:NOG69653 KO:K15718 ProtClustDB:PLN02337 HSSP:P08170 EMBL:AJ302043 IPI:IPI00543707 RefSeq:NP_188879.2 UniGene:At.37889 ProteinModelPortal:Q9LUW0 SMR:Q9LUW0 STRING:Q9LUW0 PRIDE:Q9LUW0 EnsemblPlants:AT3G22400.1 GeneID:821808 KEGG:ath:AT3G22400 TAIR:At3g22400 InParanoid:Q9LUW0 OMA:FDWDESM PhylomeDB:Q9LUW0 Genevestigator:Q9LUW0 Uniprot:Q9LUW0
Root	Isotig03715	0	50	-6.773	1.11E-13	TAIR|locus:2087837 - symbol:LOX5 species:3702 "Arabidopsis thaliana" [GO:0005506 "iron ion binding" evidence=IEA] [GO:0016165 "lipoxygenase activity" evidence=IEA;ISS;IDA] [GO:0016702 "oxidoreductase activity, acting on single donors with incorporation of molecular oxygen, incorporation of two atoms of oxygen" evidence=IEA] [GO:0046872 "metal ion binding" evidence=IEA] [GO:0009507 "chloroplast" evidence=IDA] [GO:0010386 "lateral root primordium development" evidence=IMP] [GO:0048364 "root development" evidence=IMP] InterPro:IPR000907 InterPro:IPR001024 InterPro:IPR001246 InterPro:IPR013819 InterPro:IPR020833 InterPro:IPR020834 Pfam:PF00305 Pfam:PF01477 PRINTS:PR00087 PRINTS:PR00468 PROSITE:PS00081 PROSITE:PS00711 PROSITE:PS50095 PROSITE:PS51393 SMART:SM00308 GO:GO:0009507 EMBL:CP002686 GenomeReviews:BA000014_GR GO:GO:0005506 GO:GO:0031408 EMBL:AB022215 GO:GO:0010386 InterPro:IPR008976 Gene3D:G3DSA:2.60.60.20 SUPFAM:SSF49723 GO:GO:0016165 PANTHER:PTHR11771 SUPFAM:SSF48484 eggNOG:NOG69653 KO:K15718 ProtClustDB:PLN02337 HSSP:P08170 EMBL:AJ302043 IPI:IPI00543707 RefSeq:NP_188879.2 UniGene:At.37889 ProteinModelPortal:Q9LUW0 SMR:Q9LUW0 STRING:Q9LUW0 PRIDE:Q9LUW0 EnsemblPlants:AT3G22400.1 GeneID:821808 KEGG:ath:AT3G22400 TAIR:At3g22400 InParanoid:Q9LUW0 OMA:FDWDESM PhylomeDB:Q9LUW0 Genevestigator:Q9LUW0 Uniprot:Q9LUW0
Root	Isotig03716	0	46	-6.653	9.06E-13	TAIR|locus:2087837 - symbol:LOX5 species:3702 "Arabidopsis thaliana" [GO:0005506 "iron ion binding" evidence=IEA] [GO:0016165 "lipoxygenase activity" evidence=IEA;ISS;IDA] [GO:0016702 "oxidoreductase activity, acting on single donors with incorporation of molecular oxygen, incorporation of two atoms of oxygen"

Tissue	Identification	# of reads (stress)	# of reads (control)	Fold Change	p-value	Description
						evidence=IEA] [GO:0046872 "metal ion binding" evidence=IEA] [GO:0009507 "chloroplast" evidence=IDA] [GO:0010386 "lateral root primordium development" evidence=IMP] [GO:0048364 "root development" evidence=IMP] InterPro:IPR000907 InterPro:IPR001024 InterPro:IPR001246 InterPro:IPR013819 InterPro:IPR020833 InterPro:IPR020834 Pfam:PF00305 Pfam:PF01477 PRINTS:PR00087 PRINTS:PR00468 PROSITE:PS00081 PROSITE:PS00711 PROSITE:PS50095 PROSITE:PS51393 SMART:SM00308 GO:GO:0009507 EMBL:CP002686 GenomeReviews:BA000014_GR GO:GO:0005506 GO:GO:0031408 EMBL:AB022215 GO:GO:0010386 InterPro:IPR008976 Gene3D:G3DSA:2.60.60.20 SUPFAM:SSF49723 GO:GO:0016165 PANTHER:PTHR11771 SUPFAM:SSF48484 eggNOG:NOG69653 KO:K15718 ProtClustDB:PLN02337 HSSP:P08170 EMBL:AJ302043 IPI:IPI00543707 RefSeq:NP_188879.2 UniGene:At.37889 ProteinModelPortal:Q9LUW0 SMR:Q9LUW0 STRING:Q9LUW0 PRIDE:Q9LUW0 EnsemblPlants:AT3G22400.1 GeneID:821808 KEGG:ath:AT3G22400 TAIR:At3g22400 InParanoid:Q9LUW0 OMA:FDWDESM PhylomeDB:Q9LUW0 Genevestigator:Q9LUW0 Uniprot:Q9LUW0
Root	Isotig03730	35	72	-1.170	4.82E-05	TAIR|locus:2175118 - symbol:AT5G60390 "AT5G60390" species:3702 "Arabidopsis thaliana" [GO:0003746 "translation elongation factor activity" evidence=ISS] [GO:0006414 "translational elongation" evidence=IEA] [GO:0005516 "calmodulin binding" evidence=ISS] [GO:0005886 "plasma membrane" evidence=IDA] [GO:0005739 "mitochondrion" evidence=IDA] [GO:0005773 "vacuole" evidence=IDA] [GO:0005634 "nucleus" evidence=IDA] [GO:0005737 "cytoplasm" evidence=IDA] [GO:0005829 "cytosol" evidence=RCA] [GO:0009506 "plasmodesma" evidence=IDA] [GO:0005794 "Golgi apparatus" evidence=RCA] PROSITE:PS00301 EMBL:CP002684 EMBL:U63815 EMBL:AC026875 EMBL:X16430 EMBL:AY039583 EMBL:BT000595 IPI:IPI00548618 PIR:S06724 UniGene:At.69737 UniGene:At.74830 EnsemblPlants:AT1G07940.1 EnsemblPlants:AT1G07940.2 TAIR:At1g07940 OMA:FLKAGDA GermOnline:AT1G07940 Uniprot:P0DH99
Root	Isotig03741	5	36	-2.977	8.93E-08	TAIR|locus:2032950 - symbol:TLP10 "AT1G25280" species:3702 "Arabidopsis thaliana" [GO:0003700 "sequence-specific DNA binding transcription factor activity" evidence=ISS] [GO:0008081 "phosphoric diester hydrolase activity" evidence=ISS] [GO:0006355 "regulation of transcription, DNA-dependent" evidence=TAS] [GO:0005886 "plasma membrane" evidence=IDA] InterPro:IPR001810 Pfam:PF00646 PROSITE:PS50181 INTERPRO:IPR000007 EMBL:CP002684 GenomeReviews:CT485782_GR GO:GO:0005886 GO:GO:0005634 GO:GO:0006355 EMBL:AC079374 SUPFAM:SSF54518 EMBL:AF487271 EMBL:AY140459 EMBL:AY140460 EMBL:AY140461 EMBL:AY140462 EMBL:AY140463 EMBL:AY140464 EMBL:AY140465 EMBL:AY140466 EMBL:AY140467 EMBL:AY140468 EMBL:AY140469 EMBL:AK317199 EMBL:BT001997 EMBL:BT006290 IPI:IPI00539910 PIR:E86382 RefSeq:NP_001117353.1 RefSeq:NP_173899.1 RefSeq:NP_973909.1 UniGene:At.23717 HSSP:O00294 ProteinModelPortal:Q9FRH7 SMR:Q9FRH7 IntAct:Q9FRH7 STRING:Q9FRH7 PRIDE:Q9FRH7 EnsemblPlants:AT1G25280.1 GeneID:839112 KEGG:ath:AT1G25280 TAIR:At1g25280 eggNOG:NOG286778 HOGENOM:HBG607533 InParanoid:Q9FRH7 OMA:HSSACEL PhylomeDB:Q9FRH7 ProtClustDB:CLSN2681282 ArrayExpress:Q8H0W5 Genevestigator:Q9FRH7 InterPro:IPR018066 Gene3D:G3DSA:3.20.90.10 Pfam:PF01167 PRINTS:PR01573 PROSITE:PS01200 PROSITE:PS01201 Uniprot:Q9FRH7
Root	Isotig03742	4	37	-3.338	1.13E-08	TAIR|locus:2032950 - symbol:TLP10 "AT1G25280" species:3702 "Arabidopsis thaliana" [GO:0003700 "sequence-specific DNA binding transcription factor activity" evidence=ISS] [GO:0008081 "phosphoric diester hydrolase activity" evidence=ISS] [GO:0006355 "regulation of transcription, DNA-dependent" evidence=TAS] [GO:0005886 "plasma membrane" evidence=IDA] InterPro:IPR001810 Pfam:PF00646 PROSITE:PS50181 INTERPRO:IPR000007 EMBL:CP002684 GenomeReviews:CT485782_GR GO:GO:0005886 GO:GO:0005634 GO:GO:0006355 EMBL:AC079374 SUPFAM:SSF54518 EMBL:AF487271 EMBL:AY140459 EMBL:AY140460 EMBL:AY140461 EMBL:AY140462 EMBL:AY140463 EMBL:AY140464 EMBL:AY140465 EMBL:AY140466 EMBL:AY140467 EMBL:AY140468 EMBL:AY140469 EMBL:AK317199 EMBL:BT001997 EMBL:BT006290 IPI:IPI00539910 PIR:E86382 RefSeq:NP_001117353.1 RefSeq:NP_173899.1 RefSeq:NP_973909.1 UniGene:At.23717 HSSP:O00294 ProteinModelPortal:Q9FRH7 SMR:Q9FRH7 IntAct:Q9FRH7 STRING:Q9FRH7 PRIDE:Q9FRH7 EnsemblPlants:AT1G25280.1 GeneID:839112 KEGG:ath:AT1G25280 TAIR:At1g25280 eggNOG:NOG286778 HOGENOM:HBG607533 InParanoid:Q9FRH7 OMA:HSSACEL PhylomeDB:Q9FRH7 ProtClustDB:CLSN2681282 ArrayExpress:Q8H0W5 Genevestigator:Q9FRH7 InterPro:IPR018066 Gene3D:G3DSA:3.20.90.10 Pfam:PF01167 PRINTS:PR01573 PROSITE:PS01200 PROSITE:PS01201 Uniprot:Q9FRH7
Root	Isotig03743	5	36	-2.977	8.93E-08	TAIR|locus:2032950 - symbol:TLP10 "AT1G25280" species:3702 "Arabidopsis thaliana" [GO:0003700 "sequence-specific DNA binding transcription factor activity" evidence=ISS] [GO:0008081 "phosphoric diester hydrolase activity" evidence=ISS] [GO:0006355 "regulation of transcription, DNA-dependent" evidence=TAS] [GO:0005886 "plasma

Tissue	Identification	# of reads (stress)	# of reads (control)	Fold Change	p-value	Description
						membrane" evidence=IDA] InterPro:IPR001810 Pfam:PF00646 PROSITE:PS50181 INTERPRO:IPR000007 EMBL:CP002684 GenomeReviews:CT485782_GR GO:GO:0005886 GO:GO:0005634 GO:GO:0006355 EMBL:AC079374 SUPFAM:SSF54518 EMBL:AF487271 EMBL:AY140459 EMBL:AY140460 EMBL:AY140461 EMBL:AY140462 EMBL:AY140463 EMBL:AY140464 EMBL:AY140465 EMBL:AY140466 EMBL:AY140467 EMBL:AY140468 EMBL:AY140469 EMBL:AK317199 EMBL:BT001997 EMBL:BT006290 IPI:IPI00539910 PIR:E86382 RefSeq:NP_001117353.1 RefSeq:NP_173899.1 RefSeq:NP_973909.1 UniGene:At.23717 HSSP:O00294 ProteinModelPortal:Q9FRH7 SMR:Q9FRH7 IntAct:Q9FRH7 STRING:Q9FRH7 PRIDE:Q9FRH7 EnsemblPlants:AT1G25280.1 GeneID:839112 KEGG:ath:AT1G25280 TAIR:At1g25280 eggNOG:NOG286778 HOGENOM:HBG607533 InParanoid:Q9FRH7 OMA:HSSACEL PhylomeDB:Q9FRH7 ProtClustDB:CLSN2681282 ArrayExpress:Q8H0W5 Genevestigator:Q9FRH7 InterPro:IPR018066 Gene3D:G3DSA:3.20.90.10 Pfam:PF01167 PRINTS:PR01573 PROSITE:PS01200 PROSITE:PS01201 Uniprot:Q9FRH7
Root	Isotig03744	4	37	-3.338	1.13E-08	TAIR|locus:2032950 - symbol:TLP10 "AT1G25280" species:3702 "Arabidopsis thaliana" [GO:0003700 "sequence-specific DNA binding transcription factor activity" evidence=ISS] [GO:0008081 "phosphoric diester hydrolase activity" evidence=ISS] [GO:0006355 "regulation of transcription, DNA-dependent" evidence=TAS] [GO:0005886 "plasma membrane" evidence=IDA] InterPro:IPR001810 Pfam:PF00646 PROSITE:PS50181 INTERPRO:IPR000007 EMBL:CP002684 GenomeReviews:CT485782_GR GO:GO:0005886 GO:GO:0005634 GO:GO:0006355 EMBL:AC079374 SUPFAM:SSF54518 EMBL:AF487271 EMBL:AY140459 EMBL:AY140460 EMBL:AY140461 EMBL:AY140462 EMBL:AY140463 EMBL:AY140464 EMBL:AY140465 EMBL:AY140466 EMBL:AY140467 EMBL:AY140468 EMBL:AY140469 EMBL:AK317199 EMBL:BT001997 EMBL:BT006290 IPI:IPI00539910 PIR:E86382 RefSeq:NP_001117353.1 RefSeq:NP_173899.1 RefSeq:NP_973909.1 UniGene:At.23717 HSSP:O00294 ProteinModelPortal:Q9FRH7 SMR:Q9FRH7 IntAct:Q9FRH7 STRING:Q9FRH7 PRIDE:Q9FRH7 EnsemblPlants:AT1G25280.1 GeneID:839112 KEGG:ath:AT1G25280 TAIR:At1g25280 eggNOG:NOG286778 HOGENOM:HBG607533 InParanoid:Q9FRH7 OMA:HSSACEL PhylomeDB:Q9FRH7 ProtClustDB:CLSN2681282 ArrayExpress:Q8H0W5 Genevestigator:Q9FRH7 InterPro:IPR018066 Gene3D:G3DSA:3.20.90.10 Pfam:PF01167 PRINTS:PR01573 PROSITE:PS01200 PROSITE:PS01201 Uniprot:Q9FRH7
Root	Isotig03751	25	6	1.930	0.000926157	TAIR|locus:2044807 - symbol:AT2G35040 species:3702 "Arabidopsis thaliana" [GO:0003824 "catalytic activity" evidence=IEA] [GO:0003937 "IMP cyclohydrolase activity" evidence=IEA;ISS] [GO:0004643 "phosphoribosylaminoimidazolecarboxamide formyltransferase activity" evidence=IEA;ISS] [GO:0006164 "purine nucleotide biosynthetic process" evidence=ISS] [GO:0009570 "chloroplast stroma" evidence=IDA] [GO:0009409 "response to cold" evidence=IEP] [GO:0010319 "stromule" evidence=IDA] [GO:0009507 "chloroplast" evidence=IDA] HAMAP:MF_00139 InterPro:IPR002695 InterPro:IPR016193 Pfam:PF01808 PIRSF:PIRSF000414 SMART:SM00798 GO:GO:0009570 EMBL:CP002685 GenomeReviews:CT485783_GR GO:GO:0006164 GO:GO:0010319 GO:GO:0009409 SUPFAM:SSF53927 InterPro:IPR011607 Gene3D:G3DSA:3.40.50.1380 Pfam:PF02142 SMART:SM00851 SUPFAM:SSF52335 KO:K00602 GO:GO:0003937 GO:GO:0004643 InterPro:IPR024051 Gene3D:G3DSA:3.40.140.20 PANTHER:PTHR11692 TIGRFAMs:TIGR00355 OMA:FTGTRHF HSSP:P31335 UniGene:At.66385 EMBL:AY091122 EMBL:AY133727 IPI:IPI00542590 RefSeq:NP_850240.1 UniGene:At.43666 ProteinModelPortal:Q8RWT5 SMR:Q8RWT5 STRING:Q8RWT5 PRIDE:Q8RWT5 ProMEX:Q8RWT5 EnsemblPlants:AT2G35040.1 GeneID:818069 KEGG:ath:AT2G35040 TAIR:At2g35040 InParanoid:Q8RWT5 PhylomeDB:Q8RWT5 ProtClustDB:PLN02891 Genevestigator:Q8RWT5 Uniprot:Q8RWT5
Root	Isotig03752	25	6	1.930	0.000926157	TAIR|locus:2044807 - symbol:AT2G35040 species:3702 "Arabidopsis thaliana" [GO:0003824 "catalytic activity" evidence=IEA] [GO:0003937 "IMP cyclohydrolase activity" evidence=IEA;ISS] [GO:0004643 "phosphoribosylaminoimidazolecarboxamide formyltransferase activity" evidence=IEA;ISS] [GO:0006164 "purine nucleotide biosynthetic process" evidence=ISS] [GO:0009570 "chloroplast stroma" evidence=IDA] [GO:0009409 "response to cold" evidence=IEP] [GO:0010319 "stromule" evidence=IDA] [GO:0009507 "chloroplast" evidence=IDA] HAMAP:MF_00139 InterPro:IPR002695 InterPro:IPR016193 Pfam:PF01808 PIRSF:PIRSF000414 SMART:SM00798 GO:GO:0009570 EMBL:CP002685 GenomeReviews:CT485783_GR GO:GO:0006164 GO:GO:0010319 GO:GO:0009409 SUPFAM:SSF53927 InterPro:IPR011607 Gene3D:G3DSA:3.40.50.1380 Pfam:PF02142 SMART:SM00851 SUPFAM:SSF52335 KO:K00602 GO:GO:0003937 GO:GO:0004643 InterPro:IPR024051 Gene3D:G3DSA:3.40.140.20 PANTHER:PTHR11692 TIGRFAMs:TIGR00355 OMA:FTGTRHF HSSP:P31335 UniGene:At.66385 EMBL:AY091122 EMBL:AY133727 IPI:IPI00542590 RefSeq:NP_850240.1 UniGene:At.43666 ProteinModelPortal:Q8RWT5 SMR:Q8RWT5 STRING:Q8RWT5 PRIDE:Q8RWT5

Tissue	Identification	# of reads (stress)	# of reads (control)	Fold Change	p-value	Description
						ProMEX:Q8RWT5 EnsemblPlants:AT2G35040.1 GeneID:818069 KEGG:ath:AT2G35040 TAIR:At2g35040 InParanoid:Q8RWT5 PhylomeDB:Q8RWT5 ProtClustDB:PLN02891 Genevestigator:Q8RWT5 Uniprot:Q8RWT5
Root	Isotig03786	4	23	-2.653	5.61E-05	TAIR|locus:2202290 - symbol:TPS7 "AT1G06410" species:3702 "Arabidopsis thaliana" [GO:0004805 "trehalose-phosphatase activity" evidence=IGI;ISS;IMP] [GO:0005992 "trehalose biosynthetic process" evidence=IEA;ISS] [GO:0008152 "metabolic process" evidence=IEA] [GO:0016757 "transferase activity, transferring glycosyl groups" evidence=ISS] [GO:0003825 "alpha,alpha-trehalose-phosphate synthase (UDP-forming) activity" evidence=IGI;IMP] [GO:0005829 "cytosol" evidence=IDA] InterPro:IPR001830 InterPro:IPR003337 InterPro:IPR006379 Pfam:PF00982 Pfam:PF02358 EMBL:CP002684 GenomeReviews:CT485782_GR GO:GO:0005829 InterPro:IPR023214 Gene3D:G3DSA:3.40.50.1000 SUPFAM:SSF56784 EMBL:AC068143 GO:GO:0005992 TIGRFAMs:TIGR01484 CAZy:GT20 eggNOG:COG0380 HOGENOM:HBG559076 KO:K00697 GO:GO:0003825 HSSP:P31677 KO:K01087 TIGRFAMs:TIGR00685 EMBL:AK228947 IPI:IPI00530498 PIR:A86200 RefSeq:NP_172129.1 UniGene:At.43841 ProteinModelPortal:Q9LMI0 SMR:Q9LMI0 STRING:Q9LMI0 PRIDE:Q9LMI0 EnsemblPlants:AT1G06410.1 GeneID:837152 KEGG:ath:AT1G06410 TAIR:At1g06410 InParanoid:Q9LMI0 OMA:CKDHFRR PhylomeDB:Q9LMI0 ProtClustDB:CLSN2682317 Genevestigator:Q9LMI0 Uniprot:Q9LMI0
Root	Isotig03788	2	16	-3.129	0.000262867	TAIR|locus:2202290 - symbol:TPS7 "AT1G06410" species:3702 "Arabidopsis thaliana" [GO:0004805 "trehalose-phosphatase activity" evidence=IGI;ISS;IMP] [GO:0005992 "trehalose biosynthetic process" evidence=IEA;ISS] [GO:0008152 "metabolic process" evidence=IEA] [GO:0016757 "transferase activity, transferring glycosyl groups" evidence=ISS] [GO:0003825 "alpha,alpha-trehalose-phosphate synthase (UDP-forming) activity" evidence=IGI;IMP] [GO:0005829 "cytosol" evidence=IDA] InterPro:IPR001830 InterPro:IPR003337 InterPro:IPR006379 Pfam:PF00982 Pfam:PF02358 EMBL:CP002684 GenomeReviews:CT485782_GR GO:GO:0005829 InterPro:IPR023214 Gene3D:G3DSA:3.40.50.1000 SUPFAM:SSF56784 EMBL:AC068143 GO:GO:0005992 TIGRFAMs:TIGR01484 CAZy:GT20 eggNOG:COG0380 HOGENOM:HBG559076 KO:K00697 GO:GO:0003825 HSSP:P31677 KO:K01087 TIGRFAMs:TIGR00685 EMBL:AK228947 IPI:IPI00530498 PIR:A86200 RefSeq:NP_172129.1 UniGene:At.43841 ProteinModelPortal:Q9LMI0 SMR:Q9LMI0 STRING:Q9LMI0 PRIDE:Q9LMI0 EnsemblPlants:AT1G06410.1 GeneID:837152 KEGG:ath:AT1G06410 TAIR:At1g06410 InParanoid:Q9LMI0 OMA:CKDHFRR PhylomeDB:Q9LMI0 ProtClustDB:CLSN2682317 Genevestigator:Q9LMI0 Uniprot:Q9LMI0
Root	Isotig03821	31	5	2.503	1.48E-05	TAIR|locus:2206330 - symbol:GTB1 "global transcription factor group B1" species:3702 "Arabidopsis thaliana" [GO:0003723 "RNA binding" evidence=IEA] [GO:0006333 "chromatin assembly or disassembly" evidence=ISS] [GO:0006352 "transcription initiation, DNA-dependent" evidence=ISS] [GO:0016788 "hydrolase activity, acting on ester bonds" evidence=IEA] [GO:0005829 "cytosol" evidence=IDA] [GO:0009506 "plasmodesma" evidence=IDA] InterPro:IPR000980 InterPro:IPR003029 InterPro:IPR006641 InterPro:IPR017072 PIRSF:PIRSF036947 PROSITE:PS50126 SMART:SM00252 SMART:SM00732 EMBL:CP002684 GO:GO:0005829 GO:GO:0009506 Gene3D:G3DSA:3.30.505.10 GO:GO:0006357 GO:GO:0003746 GO:GO:0016788 InterPro:IPR012340 Gene3D:G3DSA:2.40.50.140 InterPro:IPR016027 SUPFAM:SSF50249 GO:GO:0006139 InterPro:IPR022967 SMART:SM00316 EMBL:AC010795 GO:GO:0032784 eggNOG:COG2183 KO:K11292 InterPro:IPR023323 InterPro:IPR023097 Gene3D:G3DSA:1.10.150.310 Gene3D:G3DSA:1.10.3500.10 PANTHER:PTHR10145 IPI:IPI00846769 RefSeq:NP_176723.3 UniGene:At.20510 ProteinModelPortal:A8MS85 SMR:A8MS85 PRIDE:A8MS85 EnsemblPlants:AT1G65440.1 GeneID:842855 KEGG:ath:AT1G65440 TAIR:At1g65440 InParanoid:A8MS85 OMA:WGSESGG PhylomeDB:A8MS85 ProtClustDB:CLSN2682387 Genevestigator:A8MS85 Uniprot:A8MS85
Root	Isotig03822	33	5	2.593	5.16E-06	TAIR|locus:2206330 - symbol:GTB1 "global transcription factor group B1" species:3702 "Arabidopsis thaliana" [GO:0003723 "RNA binding" evidence=IEA] [GO:0006333 "chromatin assembly or disassembly" evidence=ISS] [GO:0006352 "transcription initiation, DNA-dependent" evidence=ISS] [GO:0016788 "hydrolase activity, acting on ester bonds" evidence=IEA] [GO:0005829 "cytosol" evidence=IDA] [GO:0009506 "plasmodesma" evidence=IDA] InterPro:IPR000980 InterPro:IPR003029 InterPro:IPR006641 InterPro:IPR017072 PIRSF:PIRSF036947 PROSITE:PS50126 SMART:SM00252 SMART:SM00732 EMBL:CP002684 GO:GO:0005829 GO:GO:0009506 Gene3D:G3DSA:3.30.505.10 GO:GO:0006357 GO:GO:0003746 GO:GO:0016788 InterPro:IPR012340 Gene3D:G3DSA:2.40.50.140 InterPro:IPR016027 SUPFAM:SSF50249 GO:GO:0006139 InterPro:IPR022967 SMART:SM00316 EMBL:AC010795 GO:GO:0032784 eggNOG:COG2183 KO:K11292 InterPro:IPR023323 InterPro:IPR023097 Gene3D:G3DSA:1.10.150.310 Gene3D:G3DSA:1.10.3500.10 PANTHER:PTHR10145 IPI:IPI00846769 RefSeq:NP_176723.3 UniGene:At.20510 ProteinModelPortal:A8MS85 SMR:A8MS85 PRIDE:A8MS85 EnsemblPlants:AT1G65440.1 GeneID:842855 KEGG:ath:AT1G65440 TAIR:At1g65440

Tissue	Identification	# of reads (stress)	# of reads (control)	Fold Change	p-value	Description
						InParanoid:A8MS85 OMA:WGSESGG PhylomeDB:A8MS85 ProtClustDB:CLSN2682387 Genevestigator:A8MS85 Uniprot:A8MS85
Root	Isotig03823	29	5	2.407	4.20E-05	TAIR|locus:2206330 - symbol:GTB1 "global transcription factor group B1" species:3702 "Arabidopsis thaliana" [GO:0003723 "RNA binding" evidence=IEA] [GO:0006333 "chromatin assembly or disassembly" evidence=ISS] [GO:0006352 "transcription initiation, DNA-dependent" evidence=ISS] [GO:0016788 "hydrolase activity, acting on ester bonds" evidence=IEA] [GO:0005829 "cytosol" evidence=IDA] [GO:0009506 "plasmodesma" evidence=IDA] InterPro:IPR000980 InterPro:IPR003029 InterPro:IPR006641 InterPro:IPR017072 PIRSF:PIRSF036947 PROSITE:PS50126 SMART:SM00252 SMART:SM00732 EMBL:CP002684 GO:GO:0005829 GO:GO:0009506 Gene3D:G3DSA:3.30.505.10 GO:GO:0006357 GO:GO:0003746 GO:GO:0016788 InterPro:IPR012340 Gene3D:G3DSA:2.40.50.140 InterPro:IPR016027 SUPFAM:SSF50249 GO:GO:0006139 InterPro:IPR022967 SMART:SM00316 EMBL:AC010795 GO:GO:0032784 eggNOG:COG2183 KO:K11292 InterPro:IPR023323 InterPro:IPR023097 Gene3D:G3DSA:1.10.150.310 Gene3D:G3DSA:1.10.3500.10 PANTHER:PTHR10145 IPI:IPI00846769 RefSeq:NP_176723.3 UniGene:At.20510 ProteinModelPortal:A8MS85 SMR:A8MS85 PRIDE:A8MS85 EnsemblPlants:AT1G65440.1 GeneID:842855 KEGG:ath:AT1G65440 TAIR:At1g65440 InParanoid:A8MS85 OMA:WGSESGG PhylomeDB:A8MS85 ProtClustDB:CLSN2682387 Genevestigator:A8MS85 Uniprot:A8MS85
Root	Isotig03824	31	5	2.503	1.48E-05	TAIR|locus:2206330 - symbol:GTB1 "global transcription factor group B1" species:3702 "Arabidopsis thaliana" [GO:0003723 "RNA binding" evidence=IEA] [GO:0006333 "chromatin assembly or disassembly" evidence=ISS] [GO:0006352 "transcription initiation, DNA-dependent" evidence=ISS] [GO:0016788 "hydrolase activity, acting on ester bonds" evidence=IEA] [GO:0005829 "cytosol" evidence=IDA] [GO:0009506 "plasmodesma" evidence=IDA] InterPro:IPR000980 InterPro:IPR003029 InterPro:IPR006641 InterPro:IPR017072 PIRSF:PIRSF036947 PROSITE:PS50126 SMART:SM00252 SMART:SM00732 EMBL:CP002684 GO:GO:0005829 GO:GO:0009506 Gene3D:G3DSA:3.30.505.10 GO:GO:0006357 GO:GO:0003746 GO:GO:0016788 InterPro:IPR012340 Gene3D:G3DSA:2.40.50.140 InterPro:IPR016027 SUPFAM:SSF50249 GO:GO:0006139 InterPro:IPR022967 SMART:SM00316 EMBL:AC010795 GO:GO:0032784 eggNOG:COG2183 KO:K11292 InterPro:IPR023323 InterPro:IPR023097 Gene3D:G3DSA:1.10.150.310 Gene3D:G3DSA:1.10.3500.10 PANTHER:PTHR10145 IPI:IPI00846769 RefSeq:NP_176723.3 UniGene:At.20510 ProteinModelPortal:A8MS85 SMR:A8MS85 PRIDE:A8MS85 EnsemblPlants:AT1G65440.1 GeneID:842855 KEGG:ath:AT1G65440 TAIR:At1g65440 InParanoid:A8MS85 OMA:WGSESGG PhylomeDB:A8MS85 ProtClustDB:CLSN2682387 Genevestigator:A8MS85 Uniprot:A8MS85
Root	Isotig03829	20	2	3.193	8.23E-05	TAIR|locus:2154905 - symbol:MAN7 "endo-beta-mannase 7" species:3702 "Arabidopsis thaliana" [GO:0003824 "catalytic activity" evidence=IEA] [GO:0004553 "hydrolase activity, hydrolyzing O-glycosyl compounds" evidence=IEA;ISS] [GO:0005975 "carbohydrate metabolic process" evidence=IEA] [GO:0043169 "cation binding" evidence=IEA] InterPro:IPR001547 InterPro:IPR013781 Pfam:PF00150 PROSITE:PS00659 EMBL:CP002688 GenomeReviews:BA000015_GR GO:GO:0005576 GO:GO:0043169 InterPro:IPR017853 Gene3D:G3DSA:3.20.20.80 SUPFAM:SSF51445 GO:GO:0005975 CAZy:GH5 EMBL:AB013389 eggNOG:COG3934 HOGENOM:HBG316339 GO:GO:0016985 EMBL:BT000452 EMBL:BT008749 IPI:IPI00536325 RefSeq:NP_201447.1 UniGene:At.28865 UniGene:At.67287 ProteinModelPortal:Q9FJZ3 IntAct:Q9FJZ3 PRIDE:Q9FJZ3 EnsemblPlants:AT5G66460.1 GeneID:836778 KEGG:ath:AT5G66460 TAIR:At5g66460 InParanoid:Q9FJZ3 OMA:NRIPGID PhylomeDB:Q9FJZ3 ProtClustDB:CLSN2916497 ArrayExpress:Q9FJZ3 Genevestigator:Q9FJZ3 Uniprot:Q9FJZ3
Root	Isotig03830	20	2	3.193	8.23E-05	TAIR|locus:2154905 - symbol:MAN7 "endo-beta-mannase 7" species:3702 "Arabidopsis thaliana" [GO:0003824 "catalytic activity" evidence=IEA] [GO:0004553 "hydrolase activity, hydrolyzing O-glycosyl compounds" evidence=IEA;ISS] [GO:0005975 "carbohydrate metabolic process" evidence=IEA] [GO:0043169 "cation binding" evidence=IEA] InterPro:IPR001547 InterPro:IPR013781 Pfam:PF00150 PROSITE:PS00659 EMBL:CP002688 GenomeReviews:BA000015_GR GO:GO:0005576 GO:GO:0043169 InterPro:IPR017853 Gene3D:G3DSA:3.20.20.80 SUPFAM:SSF51445 GO:GO:0005975 CAZy:GH5 EMBL:AB013389 eggNOG:COG3934 HOGENOM:HBG316339 GO:GO:0016985 EMBL:BT000452 EMBL:BT008749 IPI:IPI00536325 RefSeq:NP_201447.1 UniGene:At.28865 UniGene:At.67287 ProteinModelPortal:Q9FJZ3 IntAct:Q9FJZ3 PRIDE:Q9FJZ3 EnsemblPlants:AT5G66460.1 GeneID:836778 KEGG:ath:AT5G66460 TAIR:At5g66460 InParanoid:Q9FJZ3 OMA:NRIPGID PhylomeDB:Q9FJZ3 ProtClustDB:CLSN2916497 ArrayExpress:Q9FJZ3 Genevestigator:Q9FJZ3 Uniprot:Q9FJZ3

Tissue	Identification	# of reads (stress)	# of reads (control)	Fold Change	p-value	Description
Root	Isotig03831	20	2	3.193	8.23E-05	TAIR|locus:2154905 - symbol:MAN7 "endo-beta-mannase 7" species:3702 "Arabidopsis thaliana" [GO:0003824 "catalytic activity" evidence=IEA] [GO:0004553 "hydrolase activity, hydrolyzing O-glycosyl compounds" evidence=IEA;ISS] [GO:0005975 "carbohydrate metabolic process" evidence=IEA] [GO:0043169 "cation binding" evidence=IEA] InterPro:IPR001547 InterPro:IPR013781 Pfam:PF00150 PROSITE:PS00659 EMBL:CP002688 GenomeReviews:BA000015_GR GO:GO:0005576 GO:GO:0043169 InterPro:IPR017853 Gene3D:G3DSA:3.20.20.80 SUPFAM:SSF51445 GO:GO:0005975 CAZy:GH5 EMBL:AB013389 eggNOG:COG3934 HOGENOM:HBG316339 GO:GO:0016985 EMBL:BT000452 EMBL:BT008749 IPI:IPI00536325 RefSeq:NP_201447.1 UniGene:At.28865 UniGene:At.67287 ProteinModelPortal:Q9FJZ3 IntAct:Q9FJZ3 PRIDE:Q9FJZ3 EnsemblPlants:AT5G66460.1 GeneID:836778 KEGG:ath:AT5G66460 TAIR:At5g66460 InParanoid:Q9FJZ3 OMA:NRIPGID PhylomeDB:Q9FJZ3 ProtClustDB:CLSN2916497 ArrayExpress:Q9FJZ3 Genevestigator:Q9FJZ3 Uniprot:Q9FJZ3
Root	Isotig03832	20	2	3.193	8.23E-05	TAIR|locus:2154905 - symbol:MAN7 "endo-beta-mannase 7" species:3702 "Arabidopsis thaliana" [GO:0003824 "catalytic activity" evidence=IEA] [GO:0004553 "hydrolase activity, hydrolyzing O-glycosyl compounds" evidence=IEA;ISS] [GO:0005975 "carbohydrate metabolic process" evidence=IEA] [GO:0043169 "cation binding" evidence=IEA] InterPro:IPR001547 InterPro:IPR013781 Pfam:PF00150 PROSITE:PS00659 EMBL:CP002688 GenomeReviews:BA000015_GR GO:GO:0005576 GO:GO:0043169 InterPro:IPR017853 Gene3D:G3DSA:3.20.20.80 SUPFAM:SSF51445 GO:GO:0005975 CAZy:GH5 EMBL:AB013389 eggNOG:COG3934 HOGENOM:HBG316339 GO:GO:0016985 EMBL:BT000452 EMBL:BT008749 IPI:IPI00536325 RefSeq:NP_201447.1 UniGene:At.28865 UniGene:At.67287 ProteinModelPortal:Q9FJZ3 IntAct:Q9FJZ3 PRIDE:Q9FJZ3 EnsemblPlants:AT5G66460.1 GeneID:836778 KEGG:ath:AT5G66460 TAIR:At5g66460 InParanoid:Q9FJZ3 OMA:NRIPGID PhylomeDB:Q9FJZ3 ProtClustDB:CLSN2916497 ArrayExpress:Q9FJZ3 Genevestigator:Q9FJZ3 Uniprot:Q9FJZ3
Root	Isotig03857	7	79	-3.625	6.61E-18	TAIR|locus:2194864 - symbol:SIAR1 "AT1G44800" species:3702 "Arabidopsis thaliana" [GO:0016020 "membrane" evidence=ISS] [GO:0005515 "protein binding" evidence=IPI] [GO:0005886 "plasma membrane" evidence=IDA] [GO:0006865 "amino acid transport" evidence=IDA] [GO:0015171 "amino acid transmembrane transporter activity" evidence=IDA] [GO:0032973 "amino acid export" evidence=IDA] [GO:0034639 "L-amino acid efflux transmembrane transporter activity" evidence=IDA] [GO:0043090 "amino acid import" evidence=IDA] [GO:0080144 "amino acid homeostasis" evidence=IMP] InterPro:IPR000620 Pfam:PF00892 EMBL:CP002684 GO:GO:0005515 GO:GO:0016020 EMBL:AC020576 ProtClustDB:CLSN2679863 EMBL:AY049306 EMBL:AY149964 IPI:IPI00530851 PIR:A96507 RefSeq:NP_175101.1 UniGene:At.14882 IntAct:Q9LPF1 STRING:Q9LPF1 PRIDE:Q9LPF1 EnsemblPlants:AT1G44800.1 GeneID:841044 KEGG:ath:AT1G44800 TAIR:At1g44800 InParanoid:Q9LPF1 OMA:LIFRLET PhylomeDB:Q9LPF1 Genevestigator:Q9LPF1 Uniprot:Q9LPF1
Root	Isotig03858	7	79	-3.625	6.61E-18	TAIR|locus:2194864 - symbol:SIAR1 "AT1G44800" species:3702 "Arabidopsis thaliana" [GO:0016020 "membrane" evidence=ISS] [GO:0005515 "protein binding" evidence=IPI] [GO:0005886 "plasma membrane" evidence=IDA] [GO:0006865 "amino acid transport" evidence=IDA] [GO:0015171 "amino acid transmembrane transporter activity" evidence=IDA] [GO:0032973 "amino acid export" evidence=IDA] [GO:0034639 "L-amino acid efflux transmembrane transporter activity" evidence=IDA] [GO:0043090 "amino acid import" evidence=IDA] [GO:0080144 "amino acid homeostasis" evidence=IMP] InterPro:IPR000620 Pfam:PF00892 EMBL:CP002684 GO:GO:0005515 GO:GO:0016020 EMBL:AC020576 ProtClustDB:CLSN2679863 EMBL:AY049306 EMBL:AY149964 IPI:IPI00530851 PIR:A96507 RefSeq:NP_175101.1 UniGene:At.14882 IntAct:Q9LPF1 STRING:Q9LPF1 PRIDE:Q9LPF1 EnsemblPlants:AT1G44800.1 GeneID:841044 KEGG:ath:AT1G44800 TAIR:At1g44800 InParanoid:Q9LPF1 OMA:LIFRLET PhylomeDB:Q9LPF1 Genevestigator:Q9LPF1 Uniprot:Q9LPF1
Root	Isotig03859	8	67	-3.195	4.74E-14	TAIR|locus:2194864 - symbol:SIAR1 "AT1G44800" species:3702 "Arabidopsis thaliana" [GO:0016020 "membrane" evidence=ISS] [GO:0005515 "protein binding" evidence=IPI] [GO:0005886 "plasma membrane" evidence=IDA] [GO:0006865 "amino acid transport" evidence=IDA] [GO:0015171 "amino acid transmembrane transporter activity" evidence=IDA] [GO:0032973 "amino acid export" evidence=IDA] [GO:0034639 "L-amino acid efflux transmembrane transporter activity" evidence=IDA] [GO:0043090 "amino acid import" evidence=IDA] [GO:0080144 "amino acid homeostasis" evidence=IMP] InterPro:IPR000620 Pfam:PF00892 EMBL:CP002684 GO:GO:0005515 GO:GO:0016020 EMBL:AC020576 ProtClustDB:CLSN2679863 EMBL:AY049306 EMBL:AY149964 IPI:IPI00530851 PIR:A96507 RefSeq:NP_175101.1 UniGene:At.14882 IntAct:Q9LPF1 STRING:Q9LPF1 PRIDE:Q9LPF1 EnsemblPlants:AT1G44800.1 GeneID:841044 KEGG:ath:AT1G44800 TAIR:At1g44800 InParanoid:Q9LPF1 OMA:LIFRLET PhylomeDB:Q9LPF1 Genevestigator:Q9LPF1 Uniprot:Q9LPF1

Tissue	Identification	# of reads (stress)	# of reads (control)	Fold Change	p-value	Description
Root	Isotig03860	8	67	-3.195	4.74E-14	TAIR|locus:2194864 - symbol:SIAR1 "AT1G44800" species:3702 "Arabidopsis thaliana" [GO:0016020 "membrane" evidence=ISS] [GO:0005515 "protein binding" evidence=IPI] [GO:0005886 "plasma membrane" evidence=IDA] [GO:0006865 "amino acid transport" evidence=IDA] [GO:0015171 "amino acid transmembrane transporter activity" evidence=IDA] [GO:0032973 "amino acid export" evidence=IDA] [GO:0034639 "L-amino acid efflux transmembrane transporter activity" evidence=IDA] [GO:0043090 "amino acid import" evidence=IDA] [GO:0080144 "amino acid homeostasis" evidence=IMP] InterPro:IPR000620 Pfam:PF00892 EMBL:CP002684 GO:GO:0005515 GO:GO:0016020 EMBL:AC020576 ProtClustDB:CLSN2679863 EMBL:AY049306 EMBL:AY149964 IPI:IPI00530851 PIR:A96507 RefSeq:NP_175101.1 UniGene:At.14882 IntAct:Q9LPF1 STRING:Q9LPF1 PRIDE:Q9LPF1 EnsemblPlants:AT1G44800.1 GeneID:841044 KEGG:ath:AT1G44800 TAIR:At1g44800 InParanoid:Q9LPF1 OMA:LIFRLET PhylomeDB:Q9LPF1 Genevestigator:Q9LPF1 Uniprot:Q9LPF1
Root	Isotig03897	0	35	-6.258	3.45E-10	TAIR|locus:2165041 - symbol:AT5G56790 species:3702 "Arabidopsis thaliana" [GO:0004672 "protein kinase activity" evidence=IEA] [GO:0004713 "protein tyrosine kinase activity" evidence=IEA] [GO:0005524 "ATP binding" evidence=IEA] [GO:0006468 "protein phosphorylation" evidence=IEA] [GO:0016301 "kinase activity" evidence=ISS] InterPro:IPR000719 InterPro:IPR001245 InterPro:IPR008266 InterPro:IPR011009 Pfam:PF07714 PROSITE:PS00109 PROSITE:PS50011 GO:GO:0005524 EMBL:CP002688 eggNOG:COG0515 SUPFAM:SSF56112 GO:GO:0004713 HSSP:P06213 InterPro:IPR014729 Gene3D:G3DSA:3.40.50.620 EMBL:BT000971 IPI:IPI00529362 RefSeq:NP_568843.1 UniGene:At.26344 ProteinModelPortal:Q8H0Z8 SMR:Q8H0Z8 PRIDE:Q8H0Z8 EnsemblPlants:AT5G56790.1 GeneID:835781 KEGG:ath:AT5G56790 TAIR:At5g56790 InParanoid:Q8H0Z8 OMA:DDIHESC PhylomeDB:Q8H0Z8 ProtClustDB:CLSN2684799 Genevestigator:Q8H0Z8 Uniprot:Q8H0Z8
Root	Isotig03898	0	33	-6.173	1.05E-09	TAIR|locus:2165041 - symbol:AT5G56790 species:3702 "Arabidopsis thaliana" [GO:0004672 "protein kinase activity" evidence=IEA] [GO:0004713 "protein tyrosine kinase activity" evidence=IEA] [GO:0005524 "ATP binding" evidence=IEA] [GO:0006468 "protein phosphorylation" evidence=IEA] [GO:0016301 "kinase activity" evidence=ISS] InterPro:IPR000719 InterPro:IPR001245 InterPro:IPR008266 InterPro:IPR011009 Pfam:PF07714 PROSITE:PS00109 PROSITE:PS50011 GO:GO:0005524 EMBL:CP002688 eggNOG:COG0515 SUPFAM:SSF56112 GO:GO:0004713 HSSP:P06213 InterPro:IPR014729 Gene3D:G3DSA:3.40.50.620 EMBL:BT000971 IPI:IPI00529362 RefSeq:NP_568843.1 UniGene:At.26344 ProteinModelPortal:Q8H0Z8 SMR:Q8H0Z8 PRIDE:Q8H0Z8 EnsemblPlants:AT5G56790.1 GeneID:835781 KEGG:ath:AT5G56790 TAIR:At5g56790 InParanoid:Q8H0Z8 OMA:DDIHESC PhylomeDB:Q8H0Z8 ProtClustDB:CLSN2684799 Genevestigator:Q8H0Z8 Uniprot:Q8H0Z8
Root	Isotig03899	0	34	-6.216	6.02E-10	TAIR|locus:2165041 - symbol:AT5G56790 species:3702 "Arabidopsis thaliana" [GO:0004672 "protein kinase activity" evidence=IEA] [GO:0004713 "protein tyrosine kinase activity" evidence=IEA] [GO:0005524 "ATP binding" evidence=IEA] [GO:0006468 "protein phosphorylation" evidence=IEA] [GO:0016301 "kinase activity" evidence=ISS] InterPro:IPR000719 InterPro:IPR001245 InterPro:IPR008266 InterPro:IPR011009 Pfam:PF07714 PROSITE:PS00109 PROSITE:PS50011 GO:GO:0005524 EMBL:CP002688 eggNOG:COG0515 SUPFAM:SSF56112 GO:GO:0004713 HSSP:P06213 InterPro:IPR014729 Gene3D:G3DSA:3.40.50.620 EMBL:BT000971 IPI:IPI00529362 RefSeq:NP_568843.1 UniGene:At.26344 ProteinModelPortal:Q8H0Z8 SMR:Q8H0Z8 PRIDE:Q8H0Z8 EnsemblPlants:AT5G56790.1 GeneID:835781 KEGG:ath:AT5G56790 TAIR:At5g56790 InParanoid:Q8H0Z8 OMA:DDIHESC PhylomeDB:Q8H0Z8 ProtClustDB:CLSN2684799 Genevestigator:Q8H0Z8 Uniprot:Q8H0Z8
Root	Isotig03900	0	32	-6.129	1.84E-09	TAIR|locus:2165041 - symbol:AT5G56790 species:3702 "Arabidopsis thaliana" [GO:0004672 "protein kinase activity" evidence=IEA] [GO:0004713 "protein tyrosine kinase activity" evidence=IEA] [GO:0005524 "ATP binding" evidence=IEA] [GO:0006468 "protein phosphorylation" evidence=IEA] [GO:0016301 "kinase activity" evidence=ISS] InterPro:IPR000719 InterPro:IPR001245 InterPro:IPR008266 InterPro:IPR011009 Pfam:PF07714 PROSITE:PS00109 PROSITE:PS50011 GO:GO:0005524 EMBL:CP002688 eggNOG:COG0515 SUPFAM:SSF56112 GO:GO:0004713 HSSP:P06213 InterPro:IPR014729 Gene3D:G3DSA:3.40.50.620 EMBL:BT000971 IPI:IPI00529362 RefSeq:NP_568843.1 UniGene:At.26344 ProteinModelPortal:Q8H0Z8 SMR:Q8H0Z8 PRIDE:Q8H0Z8 EnsemblPlants:AT5G56790.1 GeneID:835781 KEGG:ath:AT5G56790 TAIR:At5g56790 InParanoid:Q8H0Z8 OMA:DDIHESC PhylomeDB:Q8H0Z8 ProtClustDB:CLSN2684799 Genevestigator:Q8H0Z8 Uniprot:Q8H0Z8
Root	Isotig03901	5	38	-3.055	2.67E-08	UNIPROTKB|P93508 - symbol:P93508 "Calreticulin" species:3988 "Ricinus communis" [GO:0005788 "endoplasmic reticulum lumen" evidence=IDA] InterPro:IPR001580 InterPro:IPR009033 InterPro:IPR009169 InterPro:IPR018124

Tissue	Identification	# of reads (stress)	# of reads (control)	Fold Change	p-value	Description
						Pfam:PF00262 PIRSF:PIRSF002356 PRINTS:PR00626 PROSITE:PS00803 PROSITE:PS00804 PROSITE:PS00805 GO:GO:0005509 GO:GO:0006457 GO:GO:0005529 GO:GO:0051082 InterPro:IPR008985 InterPro:IPR013320 Gene3D:G3DSA:2.60.120.200 SUPFAM:SSF49899 GO:GO:0005788 PROSITE:PS00014 PANTHER:PTHR11073 SUPFAM:SSF63887 KO:K08057 EMBL:U74631 EMBL:U74630 PIR:T10172 RefSeq:XP_002512501.1 ProteinModelPortal:P93508 SMR:P93508 GeneID:8269812 KEGG:rcu:RCOM_1434770 PhylomeDB:P93508 ProtClustDB:CLSN2719690 Uniprot:P93508
Root	Isotig03902	5	38	-3.055	2.67E-08	UNIPROTKB|P93508 - symbol:P93508 "Calreticulin" species:3988 "Ricinus communis" [GO:0005788 "endoplasmic reticulum lumen" evidence=IDA] InterPro:IPR001580 InterPro:IPR009033 InterPro:IPR009169 InterPro:IPR018124 Pfam:PF00262 PIRSF:PIRSF002356 PRINTS:PR00626 PROSITE:PS00803 PROSITE:PS00804 PROSITE:PS00805 GO:GO:0005509 GO:GO:0006457 GO:GO:0005529 GO:GO:0051082 InterPro:IPR008985 InterPro:IPR013320 Gene3D:G3DSA:2.60.120.200 SUPFAM:SSF49899 GO:GO:0005788 PROSITE:PS00014 PANTHER:PTHR11073 SUPFAM:SSF63887 KO:K08057 EMBL:U74631 EMBL:U74630 PIR:T10172 RefSeq:XP_002512501.1 ProteinModelPortal:P93508 SMR:P93508 GeneID:8269812 KEGG:rcu:RCOM_1434770 PhylomeDB:P93508 ProtClustDB:CLSN2719690 Uniprot:P93508
Root	Isotig03903	5	38	-3.055	2.67E-08	UNIPROTKB|P93508 - symbol:P93508 "Calreticulin" species:3988 "Ricinus communis" [GO:0005788 "endoplasmic reticulum lumen" evidence=IDA] InterPro:IPR001580 InterPro:IPR009033 InterPro:IPR009169 InterPro:IPR018124 Pfam:PF00262 PIRSF:PIRSF002356 PRINTS:PR00626 PROSITE:PS00803 PROSITE:PS00804 PROSITE:PS00805 GO:GO:0005509 GO:GO:0006457 GO:GO:0005529 GO:GO:0051082 InterPro:IPR008985 InterPro:IPR013320 Gene3D:G3DSA:2.60.120.200 SUPFAM:SSF49899 GO:GO:0005788 PROSITE:PS00014 PANTHER:PTHR11073 SUPFAM:SSF63887 KO:K08057 EMBL:U74631 EMBL:U74630 PIR:T10172 RefSeq:XP_002512501.1 ProteinModelPortal:P93508 SMR:P93508 GeneID:8269812 KEGG:rcu:RCOM_1434770 PhylomeDB:P93508 ProtClustDB:CLSN2719690 Uniprot:P93508
Root	Isotig03904	5	38	-3.055	2.67E-08	UNIPROTKB|P93508 - symbol:P93508 "Calreticulin" species:3988 "Ricinus communis" [GO:0005788 "endoplasmic reticulum lumen" evidence=IDA] InterPro:IPR001580 InterPro:IPR009033 InterPro:IPR009169 InterPro:IPR018124 Pfam:PF00262 PIRSF:PIRSF002356 PRINTS:PR00626 PROSITE:PS00803 PROSITE:PS00804 PROSITE:PS00805 GO:GO:0005509 GO:GO:0006457 GO:GO:0005529 GO:GO:0051082 InterPro:IPR008985 InterPro:IPR013320 Gene3D:G3DSA:2.60.120.200 SUPFAM:SSF49899 GO:GO:0005788 PROSITE:PS00014 PANTHER:PTHR11073 SUPFAM:SSF63887 KO:K08057 EMBL:U74631 EMBL:U74630 PIR:T10172 RefSeq:XP_002512501.1 ProteinModelPortal:P93508 SMR:P93508 GeneID:8269812 KEGG:rcu:RCOM_1434770 PhylomeDB:P93508 ProtClustDB:CLSN2719690 Uniprot:P93508
Root	Isotig03915	19	3	2.534	0.000639389	TAIR|locus:2206440 - symbol:AT1G79520 species:3702 "Arabidopsis thaliana" [GO:0006812 "cation transport" evidence=IEA;ISS] [GO:0008324 "cation transmembrane transporter activity" evidence=IEA;ISS] [GO:0016020 "membrane" evidence=ISS] [GO:0055085 "transmembrane transport" evidence=IEA] [GO:0005886 "plasma membrane" evidence=IDA] InterPro:IPR002524 Pfam:PF01545 GO:GO:0016021 EMBL:CP002684 GenomeReviews:CT485782_GR GO:GO:0005886 GO:GO:0005774 EMBL:AC007202 GO:GO:0008324 PANTHER:PTHR11562 TIGRFAMs:TIGR01297 eggNOG:COG0053 HOGENOM:HBG387227 IPI:IPI00525346 PIR:E96826 RefSeq:NP_178070.2 UniGene:At.43219 ProteinModelPortal:Q9SAJ7 PRIDE:Q9SAJ7 EnsemblPlants:AT1G79520.1 GeneID:844290 KEGG:ath:AT1G79520 TAIR:At1g79520 InParanoid:Q9SAJ7 OMA:YRSIQSL PhylomeDB:Q9SAJ7 Genevestigator:Q9SAJ7 Uniprot:Q9SAJ7
Root	Isotig03989	4	30	-3.036	8.34E-07	TAIR|locus:2058641 - symbol:DEAR3 "AT2G23340" species:3702 "Arabidopsis thaliana" [GO:0003677 "DNA binding" evidence=ISS] [GO:0003700 "sequence-specific DNA binding transcription factor activity" evidence=ISS] [GO:0005634 "nucleus" evidence=ISS] [GO:0006355 "regulation of transcription, DNA-dependent" evidence=IEA;ISS] InterPro:IPR001471 InterPro:IPR016177 Pfam:PF00847 PRINTS:PR00367 PROSITE:PS51032 SMART:SM00380 GO:GO:0005634 EMBL:CP002685 GenomeReviews:CT485783_GR GO:GO:0009873 GO:GO:0003677 GO:GO:0003700 GO:GO:0006351 Gene3D:G3DSA:3.30.730.10 SUPFAM:SSF54171 HSSP:O80337 EMBL:AC002391 HOGENOM:HBG749764 EMBL:AK118495 EMBL:BT003741 IPI:IPI00528876 PIR:T00498 RefSeq:NP_179915.1 UniGene:At.43320 ProteinModelPortal:O22174 SMR:O22174 EnsemblPlants:AT2G23340.1 GeneID:816866 KEGG:ath:AT2G23340 TAIR:At2g23340 eggNOG:NOG273209 InParanoid:O22174 OMA:LPDPENS PhylomeDB:O22174 ProtClustDB:CLSN2683886 ArrayExpress:O22174 Genevestigator:O22174 Uniprot:O22174
Root	Isotig03990	4	30	-3.036	8.34E-07	TAIR|locus:2058641 - symbol:DEAR3 "AT2G23340" species:3702 "Arabidopsis thaliana" [GO:0003677 "DNA binding" evidence=ISS] [GO:0003700 "sequence-specific DNA binding transcription factor activity" evidence=ISS]

Tissue	Identification	# of reads (stress)	# of reads (control)	Fold Change	p-value	Description
						[GO:0005634 "nucleus" evidence=ISS] [GO:0006355 "regulation of transcription, DNA-dependent" evidence=IEA;ISS] InterPro:IPR001471 InterPro:IPR016177 Pfam:PF00847 PRINTS:PR00367 PROSITE:PS51032 SMART:SM00380 GO:GO:0005634 EMBL:CP002685 GenomeReviews:CT485783_GR GO:GO:0009873 GO:GO:0003677 GO:GO:0003700 GO:GO:0006351 Gene3D:G3DSA:3.30.730.10 SUPFAM:SSF54171 HSSP:O80337 EMBL:AC002391 HOGENOM:HBG749764 EMBL:AK118495 EMBL:BT003741 IPI:IPI00528876 PIR:T00498 RefSeq:NP_179915.1 UniGene:At.43320 ProteinModelPortal:O22174 SMR:O22174 EnsemblPlants:AT2G23340.1 GeneID:816866 KEGG:ath:AT2G23340 TAIR:At2g23340 eggNOG:NOG273209 InParanoid:O22174 OMA:LPDPENS PhylomeDB:O22174 ProtClustDB:CLSN2683886 ArrayExpress:O22174 Genevestigator:O22174 Uniprot:O22174
Root	Isotig03991	3	27	-3.299	1.22E-06	TAIR|locus:1005716457 - symbol:RAP2.9 "related to AP2 9" species:3702 "Arabidopsis thaliana" [GO:0003677 "DNA binding" evidence=IEA] [GO:0003700 "sequence-specific DNA binding transcription factor activity" evidence=IEA;ISS] [GO:0005634 "nucleus" evidence=ISS] [GO:0006355 "regulation of transcription, DNA-dependent" evidence=IEA;ISS] InterPro:IPR001471 InterPro:IPR016177 Pfam:PF00847 PRINTS:PR00367 PROSITE:PS51032 SMART:SM00380 EMBL:CP002687 GenomeReviews:CT486007_GR GO:GO:0005634 GO:GO:0009873 GO:GO:0003677 GO:GO:0003700 GO:GO:0006351 KO:K09286 Gene3D:G3DSA:3.30.730.10 SUPFAM:SSF54171 HSSP:O80337 HOGENOM:HBG749764 EMBL:AB073166 EMBL:AY560869 EMBL:BT024689 EMBL:AF003102 EMBL:Z37270 IPI:IPI00535753 RefSeq:NP_849340.1 UniGene:At.47204 ProteinModelPortal:Q8W3M3 SMR:Q8W3M3 PRIDE:Q8W3M3 EnsemblPlants:AT4G06746.1 GeneID:826148 KEGG:ath:AT4G06746 TAIR:At4g06746 eggNOG:NOG320996 InParanoid:Q8W3M3 OMA:NKLPEAY PhylomeDB:Q8W3M3 ProtClustDB:CLSN2918026 ArrayExpress:O23111 Genevestigator:Q8W3M3 Uniprot:Q8W3M3
Root	Isotig03992	3	27	-3.299	1.22E-06	TAIR|locus:1005716457 - symbol:RAP2.9 "related to AP2 9" species:3702 "Arabidopsis thaliana" [GO:0003677 "DNA binding" evidence=IEA] [GO:0003700 "sequence-specific DNA binding transcription factor activity" evidence=IEA;ISS] [GO:0005634 "nucleus" evidence=ISS] [GO:0006355 "regulation of transcription, DNA-dependent" evidence=IEA;ISS] InterPro:IPR001471 InterPro:IPR016177 Pfam:PF00847 PRINTS:PR00367 PROSITE:PS51032 SMART:SM00380 EMBL:CP002687 GenomeReviews:CT486007_GR GO:GO:0005634 GO:GO:0009873 GO:GO:0003677 GO:GO:0003700 GO:GO:0006351 KO:K09286 Gene3D:G3DSA:3.30.730.10 SUPFAM:SSF54171 HSSP:O80337 HOGENOM:HBG749764 EMBL:AB073166 EMBL:AY560869 EMBL:BT024689 EMBL:AF003102 EMBL:Z37270 IPI:IPI00535753 RefSeq:NP_849340.1 UniGene:At.47204 ProteinModelPortal:Q8W3M3 SMR:Q8W3M3 PRIDE:Q8W3M3 EnsemblPlants:AT4G06746.1 GeneID:826148 KEGG:ath:AT4G06746 TAIR:At4g06746 eggNOG:NOG320996 InParanoid:Q8W3M3 OMA:NKLPEAY PhylomeDB:Q8W3M3 ProtClustDB:CLSN2918026 ArrayExpress:O23111 Genevestigator:Q8W3M3 Uniprot:Q8W3M3
Root	Isotig04001	26	4	2.571	5.65E-05	TAIR|locus:2149214 - symbol:AT5G20400 "AT5G20400" species:3702 "Arabidopsis thaliana" [GO:0008150 "biological_process" evidence=ND] [GO:0005829 "cytosol" evidence=IDA] [GO:0016706 "oxidoreductase activity, acting on paired donors, with incorporation or reduction of molecular oxygen, 2-oxoglutarate as one donor, and incorporation of one atom each of oxygen into both donors" evidence=ISS] InterPro:IPR005123 Pfam:PF03171 PROSITE:PS51471 GO:GO:0005829 EMBL:CP002688 GenomeReviews:BA000015_GR eggNOG:COG3491 HOGENOM:HBG749762 GO:GO:0016706 HSSP:Q96323 GO:GO:0016702 EMBL:BT002900 EMBL:BT005178 IPI:IPI00523248 RefSeq:NP_197540.1 UniGene:At.31186 ProteinModelPortal:Q84K82 SMR:Q84K82 STRING:Q84K82 PRIDE:Q84K82 EnsemblPlants:AT5G20400.1 GeneID:832162 KEGG:ath:AT5G20400 TAIR:At5g20400 InParanoid:Q84K82 OMA:MYGESAT PhylomeDB:Q84K82 ProtClustDB:PLN00417 ArrayExpress:Q84K82 Genevestigator:Q84K82 Uniprot:Q84K82
Root	Isotig04002	26	4	2.571	5.65E-05	TAIR|locus:2149214 - symbol:AT5G20400 "AT5G20400" species:3702 "Arabidopsis thaliana" [GO:0008150 "biological_process" evidence=ND] [GO:0005829 "cytosol" evidence=IDA] [GO:0016706 "oxidoreductase activity, acting on paired donors, with incorporation or reduction of molecular oxygen, 2-oxoglutarate as one donor, and incorporation of one atom each of oxygen into both donors" evidence=ISS] InterPro:IPR005123 Pfam:PF03171 PROSITE:PS51471 GO:GO:0005829 EMBL:CP002688 GenomeReviews:BA000015_GR eggNOG:COG3491 HOGENOM:HBG749762 GO:GO:0016706 HSSP:Q96323 GO:GO:0016702 EMBL:BT002900 EMBL:BT005178 IPI:IPI00523248 RefSeq:NP_197540.1 UniGene:At.31186 ProteinModelPortal:Q84K82 SMR:Q84K82 STRING:Q84K82 PRIDE:Q84K82 EnsemblPlants:AT5G20400.1 GeneID:832162 KEGG:ath:AT5G20400

Tissue	Identification	# of reads (stress)	# of reads (control)	Fold Change	p-value	Description
						TAIR:At5g20400 InParanoid:Q84K82 OMA:MYGESAT PhylomeDB:Q84K82 ProtClustDB:PLN00417 ArrayExpress:Q84K82 Genevestigator:Q84K82 Uniprot:Q84K82
Root	Isotig04003	26	4	2.571	5.65E-05	TAIR|locus:2149214 - symbol:AT5G20400 "AT5G20400" species:3702 "Arabidopsis thaliana" [GO:0008150 "biological_process" evidence=ND] [GO:0005829 "cytosol" evidence=IDA] [GO:0016706 "oxidoreductase activity, acting on paired donors, with incorporation or reduction of molecular oxygen, 2-oxoglutarate as one donor, and incorporation of one atom each of oxygen into both donors" evidence=ISS] InterPro:IPR005123 Pfam:PF03171 PROSITE:PS51471 GO:GO:0005829 EMBL:CP002688 GenomeReviews:BA000015_GR eggNOG:COG3491 HOGENOM:HBG749762 GO:GO:0016706 HSSP:Q96323 GO:GO:0016702 EMBL:BT002900 EMBL:BT005178 IPI:IPI00523248 RefSeq:NP_197540.1 UniGene:At.31186 ProteinModelPortal:Q84K82 SMR:Q84K82 STRING:Q84K82 PRIDE:Q84K82 EnsemblPlants:AT5G20400.1 GeneID:832162 KEGG:ath:AT5G20400 TAIR:At5g20400 InParanoid:Q84K82 OMA:MYGESAT PhylomeDB:Q84K82 ProtClustDB:PLN00417 ArrayExpress:Q84K82 Genevestigator:Q84K82 Uniprot:Q84K82
Root	Isotig04004	26	4	2.571	5.65E-05	TAIR|locus:2149214 - symbol:AT5G20400 "AT5G20400" species:3702 "Arabidopsis thaliana" [GO:0008150 "biological_process" evidence=ND] [GO:0005829 "cytosol" evidence=IDA] [GO:0016706 "oxidoreductase activity, acting on paired donors, with incorporation or reduction of molecular oxygen, 2-oxoglutarate as one donor, and incorporation of one atom each of oxygen into both donors" evidence=ISS] InterPro:IPR005123 Pfam:PF03171 PROSITE:PS51471 GO:GO:0005829 EMBL:CP002688 GenomeReviews:BA000015_GR eggNOG:COG3491 HOGENOM:HBG749762 GO:GO:0016706 HSSP:Q96323 GO:GO:0016702 EMBL:BT002900 EMBL:BT005178 IPI:IPI00523248 RefSeq:NP_197540.1 UniGene:At.31186 ProteinModelPortal:Q84K82 SMR:Q84K82 STRING:Q84K82 PRIDE:Q84K82 EnsemblPlants:AT5G20400.1 GeneID:832162 KEGG:ath:AT5G20400 TAIR:At5g20400 InParanoid:Q84K82 OMA:MYGESAT PhylomeDB:Q84K82 ProtClustDB:PLN00417 ArrayExpress:Q84K82 Genevestigator:Q84K82 Uniprot:Q84K82
Root	Isotig04025	0	13	-4.829	0.000155955	TAIR|locus:2170101 - symbol:ERF110 "AT5G50080" species:3702 "Arabidopsis thaliana" [GO:0003677 "DNA binding" evidence=ISS] [GO:0003700 "sequence-specific DNA binding transcription factor activity" evidence=ISS] [GO:0005634 "nucleus" evidence=ISS] [GO:0006355 "regulation of transcription, DNA-dependent" evidence=IEA;ISS] InterPro:IPR001471 InterPro:IPR016177 Pfam:PF00847 PRINTS:PR00367 PROSITE:PS51032 SMART:SM00380 GO:GO:0005634 EMBL:CP002688 GenomeReviews:BA000015_GR GO:GO:0009873 GO:GO:0003677 GO:GO:0003700 GO:GO:0006351 Gene3D:G3DSA:3.30.730.10 SUPFAM:SSF54171 HSSP:O80337 EMBL:AB026650 EMBL:AJ580378 IPI:IPI00543457 RefSeq:NP_199819.3 UniGene:At.29751 ProteinModelPortal:Q70II3 SMR:Q70II3 STRING:Q70II3 EnsemblPlants:AT5G50080.1 GeneID:835072 KEGG:ath:AT5G50080 TAIR:At5g50080 eggNOG:NOG295694 InParanoid:Q70II3 PhylomeDB:Q70II3 ProtClustDB:CLSN2920550 Genevestigator:Q70II3 Uniprot:Q70II3
Root	Isotig04026	0	13	-4.829	0.000155955	TAIR|locus:2197076 - symbol:RAP2.12 "AT1G53910" species:3702 "Arabidopsis thaliana" [GO:0006355 "regulation of transcription, DNA-dependent" evidence=ISS] [GO:0003700 "sequence-specific DNA binding transcription factor activity" evidence=ISS;TAS] [GO:0001666 "response to hypoxia" evidence=IMP] [GO:0005634 "nucleus" evidence=IC;IDA] [GO:0005886 "plasma membrane" evidence=IDA] [GO:0070483 "detection of hypoxia" evidence=IEP] [GO:0003677 "DNA binding" evidence=TAS] InterPro:IPR001471 InterPro:IPR016177 Pfam:PF00847 PRINTS:PR00367 PROSITE:PS51032 SMART:SM00380 EMBL:CP002684 GenomeReviews:CT485782_GR GO:GO:0005886 GO:GO:0005634 GO:GO:0009873 GO:GO:0003677 GO:GO:0003700 GO:GO:0006351 KO:K09286 Gene3D:G3DSA:3.30.730.10 SUPFAM:SSF54171 HSSP:O80337 EMBL:AC009324 GO:GO:0070483 HOGENOM:HBG595502 EMBL:AY037260 EMBL:AY057545 EMBL:AY113051 EMBL:AY088204 EMBL:AF003105 IPI:IPI00535772 PIR:D96579 RefSeq:NP_001031185.1 RefSeq:NP_175794.1 UniGene:At.20952 UniGene:At.21295 UniGene:At.68722 ProteinModelPortal:Q9SSA8 SMR:Q9SSA8 IntAct:Q9SSA8 STRING:Q9SSA8 EnsemblPlants:AT1G53910.1 EnsemblPlants:AT1G53910.2 GeneID:841829 KEGG:ath:AT1G53910 TAIR:At1g53910 eggNOG:NOG262386 InParanoid:Q9SSA8 OMA:NEDAVTT PhylomeDB:Q9SSA8 ProtClustDB:CLSN2682146 Genevestigator:Q9SSA8 Uniprot:Q9SSA8
Root	Isotig04027	0	11	-4.588	0.000569522	TAIR|locus:2170101 - symbol:ERF110 "AT5G50080" species:3702 "Arabidopsis thaliana" [GO:0003677 "DNA binding" evidence=ISS] [GO:0003700 "sequence-specific DNA binding transcription factor activity" evidence=ISS] [GO:0005634 "nucleus" evidence=ISS] [GO:0006355 "regulation of transcription, DNA-dependent" evidence=IEA;ISS] InterPro:IPR001471 InterPro:IPR016177 Pfam:PF00847 PRINTS:PR00367 PROSITE:PS51032 SMART:SM00380 GO:GO:0005634 EMBL:CP002688 GenomeReviews:BA000015_GR GO:GO:0009873 GO:GO:0003677 GO:GO:0003700 GO:GO:0006351 Gene3D:G3DSA:3.30.730.10 SUPFAM:SSF54171

Tissue	Identification	# of reads (stress)	# of reads (control)	Fold Change	p-value	Description
						HSSP:O80337 EMBL:AB026650 EMBL:AJ580378 IPI:IPI00543457 RefSeq:NP_199819.3 UniGene:At.29751 ProteinModelPortal:Q70II3 SMR:Q70II3 STRING:Q70II3 EnsemblPlants:AT5G50080.1 GeneID:835072 KEGG:ath:AT5G50080 TAIR:At5g50080 eggNOG:NOG295694 InParanoid:Q70II3 PhylomeDB:Q70II3 ProtClustDB:CLSN2920550 Genevestigator:Q70II3 Uniprot:Q70II3
Root	Isotig04028	0	11	-4.588	0.000569522	TAIR|locus:2197076 - symbol:RAP2.12 "AT1G53910" species:3702 "Arabidopsis thaliana" [GO:0006355 "regulation of transcription, DNA-dependent" evidence=ISS] [GO:0003700 "sequence-specific DNA binding transcription factor activity" evidence=ISS;TAS] [GO:0001666 "response to hypoxia" evidence=IMP] [GO:0005634 "nucleus" evidence=IC;IDA] [GO:0005886 "plasma membrane" evidence=IDA] [GO:0070483 "detection of hypoxia" evidence=IEP] [GO:0003677 "DNA binding" evidence=TAS] InterPro:IPR001471 InterPro:IPR016177 Pfam:PF00847 PRINTS:PR00367 PROSITE:PS51032 SMART:SM00380 EMBL:CP002684 GenomeReviews:CT485782_GR GO:GO:0005886 GO:GO:0005634 GO:GO:0009873 GO:GO:0003677 GO:GO:0003700 GO:GO:0006351 KO:K09286 Gene3D:G3DSA:3.30.730.10 SUPFAM:SSF54171 HSSP:O80337 EMBL:AC009324 GO:GO:0070483 HOGENOM:HBG595502 EMBL:AY037260 EMBL:AY057545 EMBL:AY113051 EMBL:AY088204 EMBL:AF003105 IPI:IPI00535772 PIR:D96579 RefSeq:NP_001031185.1 RefSeq:NP_175794.1 UniGene:At.20952 UniGene:At.21295 UniGene:At.68722 ProteinModelPortal:Q9SSA8 SMR:Q9SSA8 IntAct:Q9SSA8 STRING:Q9SSA8 EnsemblPlants:AT1G53910.1 EnsemblPlants:AT1G53910.2 GeneID:841829 KEGG:ath:AT1G53910 TAIR:At1g53910 eggNOG:NOG262386 InParanoid:Q9SSA8 OMA:NEDAVTT PhylomeDB:Q9SSA8 ProtClustDB:CLSN2682146 Genevestigator:Q9SSA8 Uniprot:Q9SSA8
Root	Isotig04033	208	17	3.484	1.21E-39	TAIR|locus:2128096 - symbol:AT4G31290 "AT4G31290" species:3702 "Arabidopsis thaliana" [GO:0003674 "molecular_function" evidence=ND] [GO:0008150 "biological_process" evidence=ND] EMBL:CP002687 GenomeReviews:CT486007_GR InterPro:IPR013024 Gene3D:G3DSA:3.10.490.10 eggNOG:COG3703 HOGENOM:HBG522249 InterPro:IPR006840 PANTHER:PTHR12192 Pfam:PF04752 UniGene:At.47478 EMBL:BT006411 EMBL:AK227743 IPI:IPI00527527 RefSeq:NP_567871.1 ProteinModelPortal:Q84MC1 STRING:Q84MC1 PRIDE:Q84MC1 EnsemblPlants:AT4G31290.1 GeneID:829256 KEGG:ath:AT4G31290 TAIR:At4g31290 InParanoid:Q84MC1 OMA:WLENDAR PhylomeDB:Q84MC1 ProtClustDB:CLSN2686752 Genevestigator:Q84MC1 Uniprot:Q84MC1
Root	Isotig04034	210	17	3.498	3.98E-40	TAIR|locus:2128096 - symbol:AT4G31290 "AT4G31290" species:3702 "Arabidopsis thaliana" [GO:0003674 "molecular_function" evidence=ND] [GO:0008150 "biological_process" evidence=ND] EMBL:CP002687 GenomeReviews:CT486007_GR InterPro:IPR013024 Gene3D:G3DSA:3.10.490.10 eggNOG:COG3703 HOGENOM:HBG522249 InterPro:IPR006840 PANTHER:PTHR12192 Pfam:PF04752 UniGene:At.47478 EMBL:BT006411 EMBL:AK227743 IPI:IPI00527527 RefSeq:NP_567871.1 ProteinModelPortal:Q84MC1 STRING:Q84MC1 PRIDE:Q84MC1 EnsemblPlants:AT4G31290.1 GeneID:829256 KEGG:ath:AT4G31290 TAIR:At4g31290 InParanoid:Q84MC1 OMA:WLENDAR PhylomeDB:Q84MC1 ProtClustDB:CLSN2686752 Genevestigator:Q84MC1 Uniprot:Q84MC1
Root	Isotig04035	165	11	3.778	1.15E-33	TAIR|locus:2128096 - symbol:AT4G31290 "AT4G31290" species:3702 "Arabidopsis thaliana" [GO:0003674 "molecular_function" evidence=ND] [GO:0008150 "biological_process" evidence=ND] EMBL:CP002687 GenomeReviews:CT486007_GR InterPro:IPR013024 Gene3D:G3DSA:3.10.490.10 eggNOG:COG3703 HOGENOM:HBG522249 InterPro:IPR006840 PANTHER:PTHR12192 Pfam:PF04752 UniGene:At.47478 EMBL:BT006411 EMBL:AK227743 IPI:IPI00527527 RefSeq:NP_567871.1 ProteinModelPortal:Q84MC1 STRING:Q84MC1 PRIDE:Q84MC1 EnsemblPlants:AT4G31290.1 GeneID:829256 KEGG:ath:AT4G31290 TAIR:At4g31290 InParanoid:Q84MC1 OMA:WLENDAR PhylomeDB:Q84MC1 ProtClustDB:CLSN2686752 Genevestigator:Q84MC1 Uniprot:Q84MC1
Root	Isotig04036	167	11	3.795	3.74E-34	TAIR|locus:2128096 - symbol:AT4G31290 "AT4G31290" species:3702 "Arabidopsis thaliana" [GO:0003674 "molecular_function" evidence=ND] [GO:0008150 "biological_process" evidence=ND] EMBL:CP002687 GenomeReviews:CT486007_GR InterPro:IPR013024 Gene3D:G3DSA:3.10.490.10 eggNOG:COG3703 HOGENOM:HBG522249 InterPro:IPR006840 PANTHER:PTHR12192 Pfam:PF04752 UniGene:At.47478 EMBL:BT006411 EMBL:AK227743 IPI:IPI00527527 RefSeq:NP_567871.1 ProteinModelPortal:Q84MC1 STRING:Q84MC1 PRIDE:Q84MC1 EnsemblPlants:AT4G31290.1 GeneID:829256 KEGG:ath:AT4G31290 TAIR:At4g31290 InParanoid:Q84MC1 OMA:WLENDAR PhylomeDB:Q84MC1 ProtClustDB:CLSN2686752 Genevestigator:Q84MC1 Uniprot:Q84MC1
Root	Isotig04049	19	0	5.119	1.05E-05	No hit

Tissue	Identification	# of reads (stress)	# of reads (control)	Fold Change	p-value	Description
Root	Isotig04050	17	0	4.958	3.30E-05	TAIR|locus:2091353 - symbol:AT3G28040 "AT3G28040" species:3702 "Arabidopsis thaliana" [GO:0004674 "protein serine/threonine kinase activity" evidence=ISS] [GO:0005524 "ATP binding" evidence=ISS] [GO:0006468 "protein phosphorylation" evidence=IEA;ISS] [GO:0007169 "transmembrane receptor protein tyrosine kinase signaling pathway" evidence=ISS] [GO:0016301 "kinase activity" evidence=ISS] Pfam:PF00560 InterPro:IPR001611 InterPro:IPR000719 InterPro:IPR011009 InterPro:IPR017441 Pfam:PF00069 PROSITE:PS00107 PROSITE:PS00108 PROSITE:PS50011 PROSITE:PS51450 GO:GO:0016021 GO:GO:0005524 EMBL:CP002686 GenomeReviews:BA000014_GR eggNOG:COG0515 GO:GO:0004872 SUPFAM:SSF56112 HOGENOM:HBG755340 GO:GO:0004672 InterPro:IPR013210 Pfam:PF08263 EMBL:AB028616 EMBL:FJ708729 IPI:IPI00540962 RefSeq:NP_189443.2 UniGene:At.28161 ProteinModelPortal:Q9LRT1 SMR:Q9LRT1 PRIDE:Q9LRT1 EnsemblPlants:AT3G28040.1 GeneID:822428 KEGG:ath:AT3G28040 GeneFarm:2365 TAIR:At3g28040 InParanoid:Q9LRT1 OMA:CTSQIPS PhylomeDB:Q9LRT1 ProtClustDB:CLSN2680201 Uniprot:Q9LRT1
Root	Isotig04065	1	32	-5.129	2.20E-09	TAIR|locus:2013001 - symbol:AT1G71695 species:3702 "Arabidopsis thaliana" [GO:0004601 "peroxidase activity" evidence=IEA;ISS] [GO:0006979 "response to oxidative stress" evidence=IEA] [GO:0020037 "heme binding" evidence=IEA] [GO:0055114 "oxidation-reduction process" evidence=IEA] [GO:0005773 "vacuole" evidence=IDA] [GO:0005618 "cell wall" evidence=IDA] [GO:0009505 "plant-type cell wall" evidence=IDA] [GO:0016020 "membrane" evidence=IDA] [GO:0009506 "plasmodesma" evidence=IDA] InterPro:IPR000823 InterPro:IPR002016 InterPro:IPR010255 InterPro:IPR019793 InterPro:IPR019794 Pfam:PF00141 PRINTS:PR00458 PRINTS:PR00461 PROSITE:PS00435 PROSITE:PS00436 PROSITE:PS50873 EMBL:CP002684 GenomeReviews:CT485782_GR GO:GO:0009506 GO:GO:0005773 GO:GO:0005576 GO:GO:0016020 GO:GO:0020037 GO:GO:0009505 EMBL:AC012654 EMBL:AC016163 GO:GO:0004601 GO:GO:0042744 HOGENOM:HBG597790 SUPFAM:SSF48113 KO:K00430 EMBL:X98318 EMBL:X98773 EMBL:AF334732 EMBL:BT000715 EMBL:AY087964 IPI:IPI00538176 PIR:A96739 RefSeq:NP_177313.1 UniGene:At.67041 UniGene:At.94 ProteinModelPortal:Q96520 SMR:Q96520 STRING:Q96520 PeroxiBase:93 PRIDE:Q96520 EnsemblPlants:AT1G71695.1 GeneID:843498 KEGG:ath:AT1G71695 GeneFarm:1474 TAIR:At1g71695 eggNOG:NOG252730 InParanoid:Q96520 OMA:NCSARNT PhylomeDB:Q96520 ProtClustDB:CLSN2679267 ArrayExpress:Q96520 Genevestigator:Q96520 GermOnline:AT1G71695 Uniprot:Q96520
Root	Isotig04066	1	32	-5.129	2.20E-09	TAIR|locus:2013001 - symbol:AT1G71695 species:3702 "Arabidopsis thaliana" [GO:0004601 "peroxidase activity" evidence=IEA;ISS] [GO:0006979 "response to oxidative stress" evidence=IEA] [GO:0020037 "heme binding" evidence=IEA] [GO:0055114 "oxidation-reduction process" evidence=IEA] [GO:0005773 "vacuole" evidence=IDA] [GO:0005618 "cell wall" evidence=IDA] [GO:0009505 "plant-type cell wall" evidence=IDA] [GO:0016020 "membrane" evidence=IDA] [GO:0009506 "plasmodesma" evidence=IDA] InterPro:IPR000823 InterPro:IPR002016 InterPro:IPR010255 InterPro:IPR019793 InterPro:IPR019794 Pfam:PF00141 PRINTS:PR00458 PRINTS:PR00461 PROSITE:PS00435 PROSITE:PS00436 PROSITE:PS50873 EMBL:CP002684 GenomeReviews:CT485782_GR GO:GO:0009506 GO:GO:0005773 GO:GO:0005576 GO:GO:0016020 GO:GO:0020037 GO:GO:0009505 EMBL:AC012654 EMBL:AC016163 GO:GO:0004601 GO:GO:0042744 HOGENOM:HBG597790 SUPFAM:SSF48113 KO:K00430 EMBL:X98318 EMBL:X98773 EMBL:AF334732 EMBL:BT000715 EMBL:AY087964 IPI:IPI00538176 PIR:A96739 RefSeq:NP_177313.1 UniGene:At.67041 UniGene:At.94 ProteinModelPortal:Q96520 SMR:Q96520 STRING:Q96520 PeroxiBase:93 PRIDE:Q96520 EnsemblPlants:AT1G71695.1 GeneID:843498 KEGG:ath:AT1G71695 GeneFarm:1474 TAIR:At1g71695 eggNOG:NOG252730 InParanoid:Q96520 OMA:NCSARNT PhylomeDB:Q96520 ProtClustDB:CLSN2679267 ArrayExpress:Q96520 Genevestigator:Q96520 GermOnline:AT1G71695 Uniprot:Q96520
Root	Isotig04067	1	32	-5.129	2.20E-09	TAIR|locus:2013001 - symbol:AT1G71695 species:3702 "Arabidopsis thaliana" [GO:0004601 "peroxidase activity" evidence=IEA;ISS] [GO:0006979 "response to oxidative stress" evidence=IEA] [GO:0020037 "heme binding" evidence=IEA] [GO:0055114 "oxidation-reduction process" evidence=IEA] [GO:0005773 "vacuole" evidence=IDA] [GO:0005618 "cell wall" evidence=IDA] [GO:0009505 "plant-type cell wall" evidence=IDA] [GO:0016020 "membrane" evidence=IDA] [GO:0009506 "plasmodesma" evidence=IDA] InterPro:IPR000823 InterPro:IPR002016 InterPro:IPR010255 InterPro:IPR019793 InterPro:IPR019794 Pfam:PF00141 PRINTS:PR00458 PRINTS:PR00461 PROSITE:PS00435 PROSITE:PS00436 PROSITE:PS50873 EMBL:CP002684 GenomeReviews:CT485782_GR GO:GO:0009506 GO:GO:0005773 GO:GO:0005576 GO:GO:0016020 GO:GO:0020037 GO:GO:0009505 EMBL:AC012654 EMBL:AC016163 GO:GO:0004601 GO:GO:0042744 HOGENOM:HBG597790 SUPFAM:SSF48113 KO:K00430 EMBL:X98318 EMBL:X98773 EMBL:AF334732 EMBL:BT000715

Tissue	Identification	# of reads (stress)	# of reads (control)	Fold Change	p-value	Description
						EMBL:AY087964 IPI:IPI00538176 PIR:A96739 RefSeq:NP_177313.1 UniGene:At.67041 UniGene:At.94 ProteinModelPortal:Q96520 SMR:Q96520 STRING:Q96520 PeroxiBase:93 PRIDE:Q96520 EnsemblPlants:AT1G71695.1 GeneID:843498 KEGG:ath:AT1G71695 GeneFarm:1474 TAIR:At1g71695 eggNOG:NOG252730 InParanoid:Q96520 OMA:NCSARNT PhylomeDB:Q96520 ProtClustDB:CLSN2679267 ArrayExpress:Q96520 Genevestigator:Q96520 GermOnline:AT1G71695 Uniprot:Q96520
Root	Isotig04068	1	32	-5.129	2.20E-09	TAIR|locus:2013001 - symbol:AT1G71695 species:3702 "Arabidopsis thaliana" [GO:0004601 "peroxidase activity" evidence=IEA;ISS] [GO:0006979 "response to oxidative stress" evidence=IEA] [GO:0020037 "heme binding" evidence=IEA] [GO:0055114 "oxidation-reduction process" evidence=IEA] [GO:0005773 "vacuole" evidence=IDA] [GO:0005618 "cell wall" evidence=IDA] [GO:0009505 "plant-type cell wall" evidence=IDA] [GO:0016020 "membrane" evidence=IDA] [GO:0009506 "plasmodesma" evidence=IDA] InterPro:IPR000823 InterPro:IPR002016 InterPro:IPR010255 InterPro:IPR019793 InterPro:IPR019794 Pfam:PF00141 PRINTS:PR00458 PRINTS:PR00461 PROSITE:PS00435 PROSITE:PS00436 PROSITE:PS50873 EMBL:CP002684 GenomeReviews:CT485782_GR GO:GO:0009506 GO:GO:0005773 GO:GO:0005576 GO:GO:0016020 GO:GO:0020037 GO:GO:0009505 EMBL:AC012654 EMBL:AC016163 GO:GO:0004601 GO:GO:0042744 HOGENOM:HBG597790 SUPFAM:SSF48113 KO:K00430 EMBL:X98318 EMBL:X98773 EMBL:AF334732 EMBL:BT000715 EMBL:AY087964 IPI:IPI00538176 PIR:A96739 RefSeq:NP_177313.1 UniGene:At.67041 UniGene:At.94 ProteinModelPortal:Q96520 SMR:Q96520 STRING:Q96520 PeroxiBase:93 PRIDE:Q96520 EnsemblPlants:AT1G71695.1 GeneID:843498 KEGG:ath:AT1G71695 GeneFarm:1474 TAIR:At1g71695 eggNOG:NOG252730 InParanoid:Q96520 OMA:NCSARNT PhylomeDB:Q96520 ProtClustDB:CLSN2679267 ArrayExpress:Q96520 Genevestigator:Q96520 GermOnline:AT1G71695 Uniprot:Q96520
Root	Isotig04089	26	0	5.571	2.13E-07	TAIR|locus:2060984 - symbol:HIS1-3 "histone H1-3" species:3702 "Arabidopsis thaliana" [GO:0000786 "nucleosome" evidence=IEA;ISS] [GO:0003677 "DNA binding" evidence=ISS] [GO:0005634 "nucleus" evidence=IEA] [GO:0006334 "nucleosome assembly" evidence=ISS] [GO:0009414 "response to water deprivation" evidence=IEP] [GO:0031492 "nucleosomal DNA binding" evidence=ISS] InterPro:IPR005818 InterPro:IPR005819 Pfam:PF00538 PRINTS:PR00624 PROSITE:PS51504 SMART:SM00526 GO:GO:0005634 EMBL:CP002685 GO:GO:0003677 InterPro:IPR011991 Gene3D:G3DSA:1.10.10.10 GO:GO:0009414 GO:GO:0006334 GO:GO:0000786 EMBL:AC006201 KO:K11275 EMBL:U72241 EMBL:U73781 EMBL:AY045797 EMBL:AY079414 EMBL:AY084603 IPI:IPI00539258 PIR:F84559 RefSeq:NP_179396.1 UniGene:At.24439 ProteinModelPortal:P94109 SMR:P94109 STRING:P94109 PRIDE:P94109 EnsemblPlants:AT2G18050.1 GeneID:816317 KEGG:ath:AT2G18050 TAIR:At2g18050 eggNOG:NOG258576 InParanoid:P94109 OMA:TKRTRSS PhylomeDB:P94109 ProtClustDB:CLSN2683930 ArrayExpress:P94109 Genevestigator:P94109 Uniprot:P94109
Root	Isotig04090	26	0	5.571	2.13E-07	TAIR|locus:2060984 - symbol:HIS1-3 "histone H1-3" species:3702 "Arabidopsis thaliana" [GO:0000786 "nucleosome" evidence=IEA;ISS] [GO:0003677 "DNA binding" evidence=ISS] [GO:0005634 "nucleus" evidence=IEA] [GO:0006334 "nucleosome assembly" evidence=ISS] [GO:0009414 "response to water deprivation" evidence=IEP] [GO:0031492 "nucleosomal DNA binding" evidence=ISS] InterPro:IPR005818 InterPro:IPR005819 Pfam:PF00538 PRINTS:PR00624 PROSITE:PS51504 SMART:SM00526 GO:GO:0005634 EMBL:CP002685 GO:GO:0003677 InterPro:IPR011991 Gene3D:G3DSA:1.10.10.10 GO:GO:0009414 GO:GO:0006334 GO:GO:0000786 EMBL:AC006201 KO:K11275 EMBL:U72241 EMBL:U73781 EMBL:AY045797 EMBL:AY079414 EMBL:AY084603 IPI:IPI00539258 PIR:F84559 RefSeq:NP_179396.1 UniGene:At.24439 ProteinModelPortal:P94109 SMR:P94109 STRING:P94109 PRIDE:P94109 EnsemblPlants:AT2G18050.1 GeneID:816317 KEGG:ath:AT2G18050 TAIR:At2g18050 eggNOG:NOG258576 InParanoid:P94109 OMA:TKRTRSS PhylomeDB:P94109 ProtClustDB:CLSN2683930 ArrayExpress:P94109 Genevestigator:P94109 Uniprot:P94109
Root	Isotig04091	26	0	5.571	2.13E-07	TAIR|locus:2060984 - symbol:HIS1-3 "histone H1-3" species:3702 "Arabidopsis thaliana" [GO:0000786 "nucleosome" evidence=IEA;ISS] [GO:0003677 "DNA binding" evidence=ISS] [GO:0005634 "nucleus" evidence=IEA] [GO:0006334 "nucleosome assembly" evidence=ISS] [GO:0009414 "response to water deprivation" evidence=IEP] [GO:0031492 "nucleosomal DNA binding" evidence=ISS] InterPro:IPR005818 InterPro:IPR005819 Pfam:PF00538 PRINTS:PR00624 PROSITE:PS51504 SMART:SM00526 GO:GO:0005634 EMBL:CP002685 GO:GO:0003677 InterPro:IPR011991 Gene3D:G3DSA:1.10.10.10 GO:GO:0009414 GO:GO:0006334 GO:GO:0000786 EMBL:AC006201 KO:K11275 EMBL:U72241 EMBL:U73781 EMBL:AY045797 EMBL:AY079414 EMBL:AY084603 IPI:IPI00539258 PIR:F84559 RefSeq:NP_179396.1 UniGene:At.24439

Tissue	Identification	# of reads (stress)	# of reads (control)	Fold Change	p-value	Description
						ProteinModelPortal:P94109 SMR:P94109 STRING:P94109 PRIDE:P94109 EnsemblPlants:AT2G18050.1 GeneID:816317 KEGG:ath:AT2G18050 TAIR:At2g18050 eggNOG:NOG258576 InParanoid:P94109 OMA:TKRTRSS PhylomeDB:P94109 ProtClustDB:CLSN2683930 ArrayExpress:P94109 Genevestigator:P94109 Uniprot:P94109
Root	Isotig04092	26	0	5.571	2.13E-07	TAIR|locus:2060984 - symbol:HIS1-3 "histone H1-3" species:3702 "Arabidopsis thaliana" [GO:0000786 "nucleosome" evidence=IEA;ISS] [GO:0003677 "DNA binding" evidence=ISS] [GO:0005634 "nucleus" evidence=IEA] [GO:0006334 "nucleosome assembly" evidence=ISS] [GO:0009414 "response to water deprivation" evidence=IEP] [GO:0031492 "nucleosomal DNA binding" evidence=ISS] InterPro:IPR005818 InterPro:IPR005819 Pfam:PF00538 PRINTS:PR00624 PROSITE:PS51504 SMART:SM00526 GO:GO:0005634 EMBL:CP002685 GO:GO:0003677 InterPro:IPR011991 Gene3D:G3DSA:1.10.10.10 GO:GO:0009414 GO:GO:0006334 GO:GO:0000786 EMBL:AC006201 KO:K11275 EMBL:U72241 EMBL:U73781 EMBL:AY045797 EMBL:AY079414 EMBL:AY084603 IPI:IPI00539258 PIR:F84559 RefSeq:NP_179396.1 UniGene:At.24439 ProteinModelPortal:P94109 SMR:P94109 STRING:P94109 PRIDE:P94109 EnsemblPlants:AT2G18050.1 GeneID:816317 KEGG:ath:AT2G18050 TAIR:At2g18050 eggNOG:NOG258576 InParanoid:P94109 OMA:TKRTRSS PhylomeDB:P94109 ProtClustDB:CLSN2683930 ArrayExpress:P94109 Genevestigator:P94109 Uniprot:P94109
Root	Isotig04101	0	15	-5.036	4.38E-05	TAIR|locus:2012607 - symbol:AT1G14550 species:3702 "Arabidopsis thaliana" [GO:0004601 "peroxidase activity" evidence=IEA;ISS] [GO:0020037 "heme binding" evidence=IEA] [GO:0071456 "cellular response to hypoxia" evidence=IEP] InterPro:IPR000823 InterPro:IPR002016 InterPro:IPR010255 InterPro:IPR019793 InterPro:IPR019794 Pfam:PF00141 PRINTS:PR00458 PRINTS:PR00461 PROSITE:PS00435 PROSITE:PS00436 PROSITE:PS50873 EMBL:CP002684 GenomeReviews:CT485782_GR GO:GO:0005576 GO:GO:0006979 GO:GO:0071456 GO:GO:0020037 GO:GO:0004601 EMBL:AC010657 EMBL:AC012188 HOGENOM:HBG597790 SUPFAM:SSF48113 KO:K00430 ProtClustDB:CLSN2682957 IPI:IPI00544334 PIR:C86280 RefSeq:NP_172907.1 UniGene:At.51610 ProteinModelPortal:Q9M9Q9 SMR:Q9M9Q9 STRING:Q9M9Q9 PeroxiBase:81 PRIDE:Q9M9Q9 EnsemblPlants:AT1G14550.1 GeneID:838017 KEGG:ath:AT1G14550 GeneFarm:1516 TAIR:At1g14550 eggNOG:NOG274724 InParanoid:Q9M9Q9 OMA:YENSSDI PhylomeDB:Q9M9Q9 ArrayExpress:Q9M9Q9 Genevestigator:Q9M9Q9 GermOnline:AT1G14550 Uniprot:Q9M9Q9
Root	Isotig04102	0	16	-5.129	2.34E-05	TAIR|locus:2012607 - symbol:AT1G14550 species:3702 "Arabidopsis thaliana" [GO:0004601 "peroxidase activity" evidence=IEA;ISS] [GO:0020037 "heme binding" evidence=IEA] [GO:0071456 "cellular response to hypoxia" evidence=IEP] InterPro:IPR000823 InterPro:IPR002016 InterPro:IPR010255 InterPro:IPR019793 InterPro:IPR019794 Pfam:PF00141 PRINTS:PR00458 PRINTS:PR00461 PROSITE:PS00435 PROSITE:PS00436 PROSITE:PS50873 EMBL:CP002684 GenomeReviews:CT485782_GR GO:GO:0005576 GO:GO:0006979 GO:GO:0071456 GO:GO:0020037 GO:GO:0004601 EMBL:AC010657 EMBL:AC012188 HOGENOM:HBG597790 SUPFAM:SSF48113 KO:K00430 ProtClustDB:CLSN2682957 IPI:IPI00544334 PIR:C86280 RefSeq:NP_172907.1 UniGene:At.51610 ProteinModelPortal:Q9M9Q9 SMR:Q9M9Q9 STRING:Q9M9Q9 PeroxiBase:81 PRIDE:Q9M9Q9 EnsemblPlants:AT1G14550.1 GeneID:838017 KEGG:ath:AT1G14550 GeneFarm:1516 TAIR:At1g14550 eggNOG:NOG274724 InParanoid:Q9M9Q9 OMA:YENSSDI PhylomeDB:Q9M9Q9 ArrayExpress:Q9M9Q9 Genevestigator:Q9M9Q9 GermOnline:AT1G14550 Uniprot:Q9M9Q9
Root	Isotig04103	0	15	-5.036	4.38E-05	TAIR|locus:2012607 - symbol:AT1G14550 species:3702 "Arabidopsis thaliana" [GO:0004601 "peroxidase activity" evidence=IEA;ISS] [GO:0020037 "heme binding" evidence=IEA] [GO:0071456 "cellular response to hypoxia" evidence=IEP] InterPro:IPR000823 InterPro:IPR002016 InterPro:IPR010255 InterPro:IPR019793 InterPro:IPR019794 Pfam:PF00141 PRINTS:PR00458 PRINTS:PR00461 PROSITE:PS00435 PROSITE:PS00436 PROSITE:PS50873 EMBL:CP002684 GenomeReviews:CT485782_GR GO:GO:0005576 GO:GO:0006979 GO:GO:0071456 GO:GO:0020037 GO:GO:0004601 EMBL:AC010657 EMBL:AC012188 HOGENOM:HBG597790 SUPFAM:SSF48113 KO:K00430 ProtClustDB:CLSN2682957 IPI:IPI00544334 PIR:C86280 RefSeq:NP_172907.1 UniGene:At.51610 ProteinModelPortal:Q9M9Q9 SMR:Q9M9Q9 STRING:Q9M9Q9 PeroxiBase:81 PRIDE:Q9M9Q9 EnsemblPlants:AT1G14550.1 GeneID:838017 KEGG:ath:AT1G14550 GeneFarm:1516 TAIR:At1g14550 eggNOG:NOG274724 InParanoid:Q9M9Q9 OMA:YENSSDI PhylomeDB:Q9M9Q9 ArrayExpress:Q9M9Q9 Genevestigator:Q9M9Q9 GermOnline:AT1G14550 Uniprot:Q9M9Q9
Root	Isotig04104	0	16	-5.129	2.34E-05	TAIR|locus:2012607 - symbol:AT1G14550 species:3702 "Arabidopsis thaliana" [GO:0004601 "peroxidase activity" evidence=IEA;ISS] [GO:0020037 "heme binding" evidence=IEA] [GO:0071456 "cellular response to hypoxia" evidence=IEP] InterPro:IPR000823 InterPro:IPR002016 InterPro:IPR010255 InterPro:IPR019793

Tissue	Identification	# of reads (stress)	# of reads (control)	Fold Change	p-value	Description
						InterPro:IPR019794 Pfam:PF00141 PRINTS:PR00458 PRINTS:PR00461 PROSITE:PS00435 PROSITE:PS00436 PROSITE:PS50873 EMBL:CP002684 GenomeReviews:CT485782_GR GO:GO:0005576 GO:GO:0006979 GO:GO:0071456 GO:GO:0020037 GO:GO:0004601 EMBL:AC010657 EMBL:AC012188 HOGENOM:HBG597790 SUPFAM:SSF48113 KO:K00430 ProtClustDB:CLSN2682957 IPI:IPI00544334 PIR:C86280 RefSeq:NP_172907.1 UniGene:At.51610 ProteinModelPortal:Q9M9Q9 SMR:Q9M9Q9 STRING:Q9M9Q9 PeroxiBase:81 PRIDE:Q9M9Q9 EnsemblPlants:AT1G14550.1 GeneID:838017 KEGG:ath:AT1G14550 GeneFarm:1516 TAIR:At1g14550 eggNOG:NOG274724 InParanoid:Q9M9Q9 OMA:YENSSDI PhylomeDB:Q9M9Q9 ArrayExpress:Q9M9Q9 Genevestigator:Q9M9Q9 GermOnline:AT1G14550 Uniprot:Q9M9Q9
Root	Isotig04105	350	0	9.322	4.44E-62	TIGR_CMR|BA_1222 - symbol:BA_1222 "conserved hypothetical protein" species:198094 "Bacillus anthracis str. Ames" [GO:0003674 "molecular_function" evidence=ND] [GO:0005575 "cellular_component" evidence=ND] [GO:0008150 "biological_process" evidence=ND] EMBL:AE016879 EMBL:AE017334 GenomeReviews:AE016879_GR GenomeReviews:AE017334_GR InterPro:IPR008160 Pfam:PF01391 EMBL:AB281483 EMBL:AJ516936 RefSeq:NP_843695.1 RefSeq:YP_017836.1 PDB:2R6Q PDBsum:2R6Q ProteinModelPortal:Q81JD7 SMR:Q81JD7 IntAct:Q81JD7 EnsemblBacteria:EBBACT00000011819 EnsemblBacteria:EBBACT00000016984 GeneID:1084744 GeneID:2814858 KEGG:ban:BA_1222 KEGG:bar:GBAA_1222 PATRIC:18780046 TIGR:BA_1222 TIGR:GBAA1222 HOGENOM:HBG342646 OMA:SDGLNPD ProtClustDB:CLSK824721 Uniprot:Q81JD7
Root	Isotig04106	292	0	9.061	8.14E-54	UNIPROTKB|G4MN25 - symbol:MGG_05575 "Putative uncharacterized protein" species:242507 "Magnaporthe oryzae 70-15" [GO:0003674 "molecular_function" evidence=ND] [GO:0005575 "cellular_component" evidence=ND] [GO:0008150 "biological_process" evidence=ND] EMBL:CM001231 RefSeq:XP_360201.1 GeneID:2676055 InterPro:IPR021054 Pfam:PF12296 Uniprot:G4MN25
Root	Isotig04107	334	0	9.255	7.77E-60	TIGR_CMR|BA_1222 - symbol:BA_1222 "conserved hypothetical protein" species:198094 "Bacillus anthracis str. Ames" [GO:0003674 "molecular_function" evidence=ND] [GO:0005575 "cellular_component" evidence=ND] [GO:0008150 "biological_process" evidence=ND] EMBL:AE016879 EMBL:AE017334 GenomeReviews:AE016879_GR GenomeReviews:AE017334_GR InterPro:IPR008160 Pfam:PF01391 EMBL:AB281483 EMBL:AJ516936 RefSeq:NP_843695.1 RefSeq:YP_017836.1 PDB:2R6Q PDBsum:2R6Q ProteinModelPortal:Q81JD7 SMR:Q81JD7 IntAct:Q81JD7 EnsemblBacteria:EBBACT00000011819 EnsemblBacteria:EBBACT00000016984 GeneID:1084744 GeneID:2814858 KEGG:ban:BA_1222 KEGG:bar:GBAA_1222 PATRIC:18780046 TIGR:BA_1222 TIGR:GBAA1222 HOGENOM:HBG342646 OMA:SDGLNPD ProtClustDB:CLSK824721 Uniprot:Q81JD7
Root	Isotig04108	276	0	8.980	1.82E-51	UNIPROTKB|G4MN25 - symbol:MGG_05575 "Putative uncharacterized protein" species:242507 "Magnaporthe oryzae 70-15" [GO:0003674 "molecular_function" evidence=ND] [GO:0005575 "cellular_component" evidence=ND] [GO:0008150 "biological_process" evidence=ND] EMBL:CM001231 RefSeq:XP_360201.1 GeneID:2676055 InterPro:IPR021054 Pfam:PF12296 Uniprot:G4MN25
Root	Isotig04113	0	11	-4.588	0.000569522	UNIPROTKB|Q8L7F6 - symbol:CnB "Calcineurin B-like protein" species:3888 "Pisum sativum" [GO:0005515 "protein binding" evidence=IPI] InterPro:IPR001125 InterPro:IPR002048 InterPro:IPR011992 PRINTS:PR00450 SMART:SM00054 GO:GO:0005509 GO:GO:0005515 InterPro:IPR018249 Gene3D:G3DSA:1.10.238.10 PROSITE:PS50222 EMBL:AY134619 EMBL:AY883569 ProteinModelPortal:Q8L7F6 SMR:Q8L7F6 IntAct:Q8L7F6 Uniprot:Q8L7F6
Root	Isotig04114	0	11	-4.588	0.000569522	UNIPROTKB|Q8L7F6 - symbol:CnB "Calcineurin B-like protein" species:3888 "Pisum sativum" [GO:0005515 "protein binding" evidence=IPI] InterPro:IPR001125 InterPro:IPR002048 InterPro:IPR011992 PRINTS:PR00450 SMART:SM00054 GO:GO:0005509 GO:GO:0005515 InterPro:IPR018249 Gene3D:G3DSA:1.10.238.10 PROSITE:PS50222 EMBL:AY134619 EMBL:AY883569 ProteinModelPortal:Q8L7F6 SMR:Q8L7F6 IntAct:Q8L7F6 Uniprot:Q8L7F6
Root	Isotig04115	0	11	-4.588	0.000569522	UNIPROTKB|Q8L7F6 - symbol:CnB "Calcineurin B-like protein" species:3888 "Pisum sativum" [GO:0005515 "protein binding" evidence=IPI] InterPro:IPR001125 InterPro:IPR002048 InterPro:IPR011992 PRINTS:PR00450 SMART:SM00054 GO:GO:0005509 GO:GO:0005515 InterPro:IPR018249 Gene3D:G3DSA:1.10.238.10 PROSITE:PS50222 EMBL:AY134619 EMBL:AY883569 ProteinModelPortal:Q8L7F6 SMR:Q8L7F6 IntAct:Q8L7F6 Uniprot:Q8L7F6
Root	Isotig04116	0	11	-4.588	0.000569522	UNIPROTKB|Q8L7F6 - symbol:CnB "Calcineurin B-like protein" species:3888 "Pisum sativum" [GO:0005515 "protein binding" evidence=IPI] InterPro:IPR001125 InterPro:IPR002048 InterPro:IPR011992 PRINTS:PR00450 SMART:SM00054 GO:GO:0005509 GO:GO:0005515 InterPro:IPR018249 Gene3D:G3DSA:1.10.238.10

Tissue	Identification	# of reads (stress)	# of reads (control)	Fold Change	p-value	Description
						PROSITE:PS50222 EMBL:AY134619 EMBL:AY883569 ProteinModelPortal:Q8L7F6 SMR:Q8L7F6 IntAct:Q8L7F6 Uniprot:Q8L7F6
Root	Isotig04117	24	4	2.456	0.000163328	TAIR|locus:2081695 - symbol:ATGSTF13 "AT3G62760" species:3702 "Arabidopsis thaliana" [GO:0004364 "glutathione transferase activity" evidence=ISS] [GO:0005737 "cytoplasm" evidence=NAS] [GO:0009407 "toxin catabolic process" evidence=TAS] InterPro:IPR004045 Pfam:PF02798 PROSITE:PS50404 InterPro:IPR004046 Pfam:PF00043 GO:GO:0005829 EMBL:CP002686 GenomeReviews:BA000014_GR EMBL:AL162651 GO:GO:0009636 InterPro:IPR012336 Gene3D:G3DSA:3.40.30.10 SUPFAM:SSF52833 InterPro:IPR010987 Gene3D:G3DSA:1.20.1050.10 SUPFAM:SSF47616 PROSITE:PS50405 InterPro:IPR017933 GO:GO:0004364 eggNOG:COG0625 HOGENOM:HBG753188 GO:GO:0009407 HSSP:Q9ZP62 IPI:IPI00528803 PIR:T48065 RefSeq:NP_191835.1 UniGene:At.54041 ProteinModelPortal:Q9LZI9 SMR:Q9LZI9 PRIDE:Q9LZI9 EnsemblPlants:AT3G62760.1 GeneID:825451 KEGG:ath:AT3G62760 TAIR:At3g62760 InParanoid:Q9LZI9 OMA:KAWWERL PhylomeDB:Q9LZI9 ProtClustDB:CLSN2684163 Genevestigator:Q9LZI9 Uniprot:Q9LZI9
Root	Isotig04118	24	4	2.456	0.000163328	TAIR|locus:2081695 - symbol:ATGSTF13 "AT3G62760" species:3702 "Arabidopsis thaliana" [GO:0004364 "glutathione transferase activity" evidence=ISS] [GO:0005737 "cytoplasm" evidence=NAS] [GO:0009407 "toxin catabolic process" evidence=TAS] InterPro:IPR004045 Pfam:PF02798 PROSITE:PS50404 InterPro:IPR004046 Pfam:PF00043 GO:GO:0005829 EMBL:CP002686 GenomeReviews:BA000014_GR EMBL:AL162651 GO:GO:0009636 InterPro:IPR012336 Gene3D:G3DSA:3.40.30.10 SUPFAM:SSF52833 InterPro:IPR010987 Gene3D:G3DSA:1.20.1050.10 SUPFAM:SSF47616 PROSITE:PS50405 InterPro:IPR017933 GO:GO:0004364 eggNOG:COG0625 HOGENOM:HBG753188 GO:GO:0009407 HSSP:Q9ZP62 IPI:IPI00528803 PIR:T48065 RefSeq:NP_191835.1 UniGene:At.54041 ProteinModelPortal:Q9LZI9 SMR:Q9LZI9 PRIDE:Q9LZI9 EnsemblPlants:AT3G62760.1 GeneID:825451 KEGG:ath:AT3G62760 TAIR:At3g62760 InParanoid:Q9LZI9 OMA:KAWWERL PhylomeDB:Q9LZI9 ProtClustDB:CLSN2684163 Genevestigator:Q9LZI9 Uniprot:Q9LZI9
Root	Isotig04119	24	4	2.456	0.000163328	TAIR|locus:2081695 - symbol:ATGSTF13 "AT3G62760" species:3702 "Arabidopsis thaliana" [GO:0004364 "glutathione transferase activity" evidence=ISS] [GO:0005737 "cytoplasm" evidence=NAS] [GO:0009407 "toxin catabolic process" evidence=TAS] InterPro:IPR004045 Pfam:PF02798 PROSITE:PS50404 InterPro:IPR004046 Pfam:PF00043 GO:GO:0005829 EMBL:CP002686 GenomeReviews:BA000014_GR EMBL:AL162651 GO:GO:0009636 InterPro:IPR012336 Gene3D:G3DSA:3.40.30.10 SUPFAM:SSF52833 InterPro:IPR010987 Gene3D:G3DSA:1.20.1050.10 SUPFAM:SSF47616 PROSITE:PS50405 InterPro:IPR017933 GO:GO:0004364 eggNOG:COG0625 HOGENOM:HBG753188 GO:GO:0009407 HSSP:Q9ZP62 IPI:IPI00528803 PIR:T48065 RefSeq:NP_191835.1 UniGene:At.54041 ProteinModelPortal:Q9LZI9 SMR:Q9LZI9 PRIDE:Q9LZI9 EnsemblPlants:AT3G62760.1 GeneID:825451 KEGG:ath:AT3G62760 TAIR:At3g62760 InParanoid:Q9LZI9 OMA:KAWWERL PhylomeDB:Q9LZI9 ProtClustDB:CLSN2684163 Genevestigator:Q9LZI9 Uniprot:Q9LZI9
Root	Isotig04120	24	4	2.456	0.000163328	TAIR|locus:2081695 - symbol:ATGSTF13 "AT3G62760" species:3702 "Arabidopsis thaliana" [GO:0004364 "glutathione transferase activity" evidence=ISS] [GO:0005737 "cytoplasm" evidence=NAS] [GO:0009407 "toxin catabolic process" evidence=TAS] InterPro:IPR004045 Pfam:PF02798 PROSITE:PS50404 InterPro:IPR004046 Pfam:PF00043 GO:GO:0005829 EMBL:CP002686 GenomeReviews:BA000014_GR EMBL:AL162651 GO:GO:0009636 InterPro:IPR012336 Gene3D:G3DSA:3.40.30.10 SUPFAM:SSF52833 InterPro:IPR010987 Gene3D:G3DSA:1.20.1050.10 SUPFAM:SSF47616 PROSITE:PS50405 InterPro:IPR017933 GO:GO:0004364 eggNOG:COG0625 HOGENOM:HBG753188 GO:GO:0009407 HSSP:Q9ZP62 IPI:IPI00528803 PIR:T48065 RefSeq:NP_191835.1 UniGene:At.54041 ProteinModelPortal:Q9LZI9 SMR:Q9LZI9 PRIDE:Q9LZI9 EnsemblPlants:AT3G62760.1 GeneID:825451 KEGG:ath:AT3G62760 TAIR:At3g62760 InParanoid:Q9LZI9 OMA:KAWWERL PhylomeDB:Q9LZI9 ProtClustDB:CLSN2684163 Genevestigator:Q9LZI9 Uniprot:Q9LZI9
Root	Isotig04121	14	0	4.678	0.000190242	No hit
Root	Isotig04122	14	0	4.678	0.000190242	No hit
Root	Isotig04159	0	11	-4.588	0.000569522	TAIR|locus:2200281 - symbol:ANN5 "annexin 5" species:3702 "Arabidopsis thaliana" [GO:0005509 "calcium ion binding" evidence=IEA;ISS] [GO:0005544 "calcium-dependent phospholipid binding" evidence=IEA;ISS] [GO:0009408 "response to heat" evidence=IEP] [GO:0009409 "response to cold" evidence=IEP] [GO:0009414 "response to water deprivation" evidence=IEP] [GO:0009639 "response to red or far red light" evidence=IEP] [GO:0009651 "response to salt stress" evidence=IEP] InterPro:IPR001464 InterPro:IPR018252 InterPro:IPR018502 Pfam:PF00191 PRINTS:PR00196 PROSITE:PS00223 SMART:SM00335 EMBL:CP002684 GenomeReviews:CT485782_GR GO:GO:0005509 GO:GO:0009409 GO:GO:0009651 EMBL:AC012563 GO:GO:0009414 GO:GO:0009408 GO:GO:0009639 eggNOG:NOG267770 GO:GO:0005544

Tissue	Identification	# of reads (stress)	# of reads (control)	Fold Change	p-value	Description
						Gene3D:G3DSA:1.10.220.10 PANTHER:PTHR10502 SUPFAM:SSF47874 HOGENOM:HBG745057 InterPro:IPR009118 PANTHER:PTHR10502:SF10 EMBL:AY014797 EMBL:DQ446409 EMBL:DQ652921 IPI:IPI00532119 PIR:B96704 RefSeq:NP_564920.1 UniGene:At.18922 HSSP:P08758 ProteinModelPortal:Q9C9X3 SMR:Q9C9X3 PRIDE:Q9C9X3 EnsemblPlants:AT1G68090.1 GeneID:843137 KEGG:ath:AT1G68090 GeneFarm:1476 TAIR:At1g68090 InParanoid:Q9C9X3 OMA:LYKSMRR PhylomeDB:Q9C9X3 ProtClustDB:CLSN2917400 Genevestigator:Q9C9X3 Uniprot:Q9C9X3
Root	Isotig04160	0	11	-4.588	0.000569522	TAIR|locus:2200281 - symbol:ANN5 "annexin 5" species:3702 "Arabidopsis thaliana" [GO:0005509 "calcium ion binding" evidence=IEA;ISS] [GO:0005544 "calcium-dependent phospholipid binding" evidence=IEA;ISS] [GO:0009408 "response to heat" evidence=IEP] [GO:0009409 "response to cold" evidence=IEP] [GO:0009414 "response to water deprivation" evidence=IEP] [GO:0009639 "response to red or far red light" evidence=IEP] [GO:0009651 "response to salt stress" evidence=IEP] InterPro:IPR001464 InterPro:IPR018252 InterPro:IPR018502 Pfam:PF00191 PRINTS:PR00196 PROSITE:PS00223 SMART:SM00335 EMBL:CP002684 GenomeReviews:CT485782_GR GO:GO:0005509 GO:GO:0009409 GO:GO:0009651 EMBL:AC012563 GO:GO:0009414 GO:GO:0009408 GO:GO:0009639 eggNOG:NOG267770 GO:GO:0005544 Gene3D:G3DSA:1.10.220.10 PANTHER:PTHR10502 SUPFAM:SSF47874 HOGENOM:HBG745057 InterPro:IPR009118 PANTHER:PTHR10502:SF10 EMBL:AY014797 EMBL:DQ446409 EMBL:DQ652921 IPI:IPI00532119 PIR:B96704 RefSeq:NP_564920.1 UniGene:At.18922 HSSP:P08758 ProteinModelPortal:Q9C9X3 SMR:Q9C9X3 PRIDE:Q9C9X3 EnsemblPlants:AT1G68090.1 GeneID:843137 KEGG:ath:AT1G68090 GeneFarm:1476 TAIR:At1g68090 InParanoid:Q9C9X3 OMA:LYKSMRR PhylomeDB:Q9C9X3 ProtClustDB:CLSN2917400 Genevestigator:Q9C9X3 Uniprot:Q9C9X3
Root	Isotig04173	1	20	-4.451	3.92E-06	TAIR|locus:2206835 - symbol:NUDT21 "AT1G73540" species:3702 "Arabidopsis thaliana" [GO:0008150 "biological_process" evidence=ND] [GO:0016787 "hydrolase activity" evidence=IEA;ISS] InterPro:IPR000086 InterPro:IPR015797 InterPro:IPR020084 Pfam:PF00293 PROSITE:PS00893 PROSITE:PS51462 EMBL:CP002684 GenomeReviews:CT485782_GR GO:GO:0009507 GO:GO:0046872 Gene3D:G3DSA:3.90.79.10 SUPFAM:SSF55811 GO:GO:0016787 HOGENOM:HBG713158 EMBL:AC012396 EMBL:AY072369 EMBL:BT002080 IPI:IPI00544022 PIR:B96762 RefSeq:NP_177495.2 UniGene:At.34983 ProteinModelPortal:Q8VY81 SMR:Q8VY81 STRING:Q8VY81 PRIDE:Q8VY81 EnsemblPlants:AT1G73540.1 GeneID:843688 KEGG:ath:AT1G73540 TAIR:At1g73540 eggNOG:NOG240663 InParanoid:Q8VY81 OMA:RDLQRYN PhylomeDB:Q8VY81 ProtClustDB:CLSN2914542 ArrayExpress:Q8VY81 Genevestigator:Q8VY81 GermOnline:AT1G73540 Uniprot:Q8VY81
Root	Isotig04174	1	20	-4.451	3.92E-06	TAIR|locus:2206835 - symbol:NUDT21 "AT1G73540" species:3702 "Arabidopsis thaliana" [GO:0008150 "biological_process" evidence=ND] [GO:0016787 "hydrolase activity" evidence=IEA;ISS] InterPro:IPR000086 InterPro:IPR015797 InterPro:IPR020084 Pfam:PF00293 PROSITE:PS00893 PROSITE:PS51462 EMBL:CP002684 GenomeReviews:CT485782_GR GO:GO:0009507 GO:GO:0046872 Gene3D:G3DSA:3.90.79.10 SUPFAM:SSF55811 GO:GO:0016787 HOGENOM:HBG713158 EMBL:AC012396 EMBL:AY072369 EMBL:BT002080 IPI:IPI00544022 PIR:B96762 RefSeq:NP_177495.2 UniGene:At.34983 ProteinModelPortal:Q8VY81 SMR:Q8VY81 STRING:Q8VY81 PRIDE:Q8VY81 EnsemblPlants:AT1G73540.1 GeneID:843688 KEGG:ath:AT1G73540 TAIR:At1g73540 eggNOG:NOG240663 InParanoid:Q8VY81 OMA:RDLQRYN PhylomeDB:Q8VY81 ProtClustDB:CLSN2914542 ArrayExpress:Q8VY81 Genevestigator:Q8VY81 GermOnline:AT1G73540 Uniprot:Q8VY81
Root	Isotig04175	1	19	-4.377	7.43E-06	TAIR|locus:2206835 - symbol:NUDT21 "AT1G73540" species:3702 "Arabidopsis thaliana" [GO:0008150 "biological_process" evidence=ND] [GO:0016787 "hydrolase activity" evidence=IEA;ISS] InterPro:IPR000086 InterPro:IPR015797 InterPro:IPR020084 Pfam:PF00293 PROSITE:PS00893 PROSITE:PS51462 EMBL:CP002684 GenomeReviews:CT485782_GR GO:GO:0009507 GO:GO:0046872 Gene3D:G3DSA:3.90.79.10 SUPFAM:SSF55811 GO:GO:0016787 HOGENOM:HBG713158 EMBL:AC012396 EMBL:AY072369 EMBL:BT002080 IPI:IPI00544022 PIR:B96762 RefSeq:NP_177495.2 UniGene:At.34983 ProteinModelPortal:Q8VY81 SMR:Q8VY81 STRING:Q8VY81 PRIDE:Q8VY81 EnsemblPlants:AT1G73540.1 GeneID:843688 KEGG:ath:AT1G73540 TAIR:At1g73540 eggNOG:NOG240663 InParanoid:Q8VY81 OMA:RDLQRYN PhylomeDB:Q8VY81 ProtClustDB:CLSN2914542 ArrayExpress:Q8VY81 Genevestigator:Q8VY81 GermOnline:AT1G73540 Uniprot:Q8VY81
Root	Isotig04176	1	19	-4.377	7.43E-06	TAIR|locus:2206835 - symbol:NUDT21 "AT1G73540" species:3702 "Arabidopsis thaliana" [GO:0008150 "biological_process" evidence=ND] [GO:0016787 "hydrolase activity" evidence=IEA;ISS] InterPro:IPR000086

Tissue	Identification	# of reads (stress)	# of reads (control)	Fold Change	p-value	Description
						InterPro:IPR015797 InterPro:IPR020084 Pfam:PF00293 PROSITE:PS00893 PROSITE:PS51462 EMBL:CP002684 GenomeReviews:CT485782_GR GO:GO:0009507 GO:GO:0046872 Gene3D:G3DSA:3.90.79.10 SUPFAM:SSF55811 GO:GO:0016787 HOGENOM:HBG713158 EMBL:AC012396 EMBL:AY072369 EMBL:BT002080 IPI:IPI00544022 PIR:B96762 RefSeq:NP_177495.2 UniGene:At.34983 ProteinModelPortal:Q8VY81 SMR:Q8VY81 STRING:Q8VY81 PRIDE:Q8VY81 EnsemblPlants:AT1G73540.1 GeneID:843688 KEGG:ath:AT1G73540 TAIR:At1g73540 eggNOG:NOG240663 InParanoid:Q8VY81 OMA:RDLQRYN PhylomeDB:Q8VY81 ProtClustDB:CLSN2914542 ArrayExpress:Q8VY81 Genevestigator:Q8VY81 GermOnline:AT1G73540 Uniprot:Q8VY81
Root	Isotig04225	14	1	3.678	0.000484579	TAIR|locus:2008445 - symbol:AT1G61260 "AT1G61260" species:3702 "Arabidopsis thaliana" [GO:0003674 "molecular_function" evidence=ND] [GO:0008150 "biological_process" evidence=ND] InterPro:IPR008480 Pfam:PF05553 EMBL:AK227510 IPI:IPI00518212 TAIR:At1g61260 InParanoid:Q0WTN8 PhylomeDB:Q0WTN8 Genevestigator:Q0WTN8 Uniprot:Q0WTN8
Root	Isotig04226	14	1	3.678	0.000484579	TAIR|locus:2008445 - symbol:AT1G61260 "AT1G61260" species:3702 "Arabidopsis thaliana" [GO:0003674 "molecular_function" evidence=ND] [GO:0008150 "biological_process" evidence=ND] InterPro:IPR008480 Pfam:PF05553 EMBL:AK227510 IPI:IPI00518212 TAIR:At1g61260 InParanoid:Q0WTN8 PhylomeDB:Q0WTN8 Genevestigator:Q0WTN8 Uniprot:Q0WTN8
Root	Isotig04227	14	1	3.678	0.000484579	TAIR|locus:2008445 - symbol:AT1G61260 "AT1G61260" species:3702 "Arabidopsis thaliana" [GO:0003674 "molecular_function" evidence=ND] [GO:0008150 "biological_process" evidence=ND] InterPro:IPR008480 Pfam:PF05553 EMBL:AK227510 IPI:IPI00518212 TAIR:At1g61260 InParanoid:Q0WTN8 PhylomeDB:Q0WTN8 Genevestigator:Q0WTN8 Uniprot:Q0WTN8
Root	Isotig04228	14	1	3.678	0.000484579	TAIR|locus:2008445 - symbol:AT1G61260 "AT1G61260" species:3702 "Arabidopsis thaliana" [GO:0003674 "molecular_function" evidence=ND] [GO:0008150 "biological_process" evidence=ND] InterPro:IPR008480 Pfam:PF05553 EMBL:AK227510 IPI:IPI00518212 TAIR:At1g61260 InParanoid:Q0WTN8 PhylomeDB:Q0WTN8 Genevestigator:Q0WTN8 Uniprot:Q0WTN8
Root	Isotig04233	324	59	2.328	4.19E-41	No hit
Root	Isotig04234	325	59	2.333	2.58E-41	No hit
Root	Isotig04235	324	49	2.596	2.43E-46	No hit
Root	Isotig04236	325	49	2.601	1.46E-46	No hit
Root	Isotig04414	1	118	-7.012	9.07E-30	UNIPROTKB|B2HIN2 - symbol:fadD26 "Long-chain-fatty-acid--AMP ligase FadD26" species:216594 "Mycobacterium marinum M" [GO:0008610 "lipid biosynthetic process" evidence=ISS] InterPro:IPR000873 Pfam:PF00501 eggNOG:COG0318 HOGENOM:HBG547964 GO:GO:0016874 GO:GO:0008610 GO:GO:0006631 KO:K12426 OMA:PARWIQQ ProtClustDB:PRK05850 EMBL:CP000854 RefSeq:YP_001850081.1 ProteinModelPortal:B2HIN2 STRING:B2HIN2 EnsemblBacteria:EBMYCT00000032275 GeneID:6226036 GenomeReviews:CP000854_GR KEGG:mmi:MMAR_1777 PATRIC:18064429 Uniprot:B2HIN2
Root	Isotig04415	1	118	-7.012	9.07E-30	UNIPROTKB|B2HIN2 - symbol:fadD26 "Long-chain-fatty-acid--AMP ligase FadD26" species:216594 "Mycobacterium marinum M" [GO:0008610 "lipid biosynthetic process" evidence=ISS] InterPro:IPR000873 Pfam:PF00501 eggNOG:COG0318 HOGENOM:HBG547964 GO:GO:0016874 GO:GO:0008610 GO:GO:0006631 KO:K12426 OMA:PARWIQQ ProtClustDB:PRK05850 EMBL:CP000854 RefSeq:YP_001850081.1 ProteinModelPortal:B2HIN2 STRING:B2HIN2 EnsemblBacteria:EBMYCT00000032275 GeneID:6226036 GenomeReviews:CP000854_GR KEGG:mmi:MMAR_1777 PATRIC:18064429 Uniprot:B2HIN2
Root	Isotig04416	0	51	-6.801	6.64E-14	UNIPROTKB|B2HIN2 - symbol:fadD26 "Long-chain-fatty-acid--AMP ligase FadD26" species:216594 "Mycobacterium marinum M" [GO:0008610 "lipid biosynthetic process" evidence=ISS] InterPro:IPR000873 Pfam:PF00501 eggNOG:COG0318 HOGENOM:HBG547964 GO:GO:0016874 GO:GO:0008610 GO:GO:0006631 KO:K12426 OMA:PARWIQQ ProtClustDB:PRK05850 EMBL:CP000854 RefSeq:YP_001850081.1 ProteinModelPortal:B2HIN2 STRING:B2HIN2 EnsemblBacteria:EBMYCT00000032275 GeneID:6226036 GenomeReviews:CP000854_GR KEGG:mmi:MMAR_1777 PATRIC:18064429 Uniprot:B2HIN2
Root	Isotig04438	11	98	-3.284	2.74E-20	TAIR|locus:2200216 - symbol:ATTPS6 "AT1G68020" species:3702 "Arabidopsis thaliana" [GO:0004805 "trehalose-phosphatase activity" evidence=IGI;ISS;IMP] [GO:0005992 "trehalose biosynthetic process" evidence=IGI;ISS] [GO:0016757 "transferase activity, transferring glycosyl groups" evidence=ISS] [GO:0003825 "alpha,alpha-trehalose-phosphate synthase (UDP-forming) activity" evidence=IGI;IMP] InterPro:IPR001830 InterPro:IPR003337 InterPro:IPR006379 Pfam:PF00982 Pfam:PF02358 EMBL:CP002684 GenomeReviews:CT485782_GR InterPro:IPR023214 Gene3D:G3DSA:3.40.50.1000 SUPFAM:SSF56784 EMBL:AC012563 GO:GO:0004805

Tissue	Identification	# of reads (stress)	# of reads (control)	Fold Change	p-value	Description
						GO:GO:0005992 TIGRFAMs:TIGR01484 CAZy:GT20 eggNOG:COG0380 HOGENOM:HBG559076 KO:K00697 GO:GO:0003825 HSSP:P31677 TIGRFAMs:TIGR00685 OMA:LMQHPEW EMBL:AY046028 IPI:IPI00516798 IPI:IPI00538850 PIR:C96703 RefSeq:NP_564918.1 RefSeq:NP_974105.1 UniGene:At.19364 ProteinModelPortal:Q94AH8 SMR:Q94AH8 STRING:Q94AH8 PRIDE:Q94AH8 EnsemblPlants:AT1G68020.2 GeneID:843130 KEGG:ath:AT1G68020 TAIR:At1g68020 InParanoid:Q94AH8 PhylomeDB:Q94AH8 ProtClustDB:PLN02205 ArrayExpress:Q94AH8 Genevestigator:Q94AH8 Uniprot:Q94AH8
Root	Isotig04439	9	72	-3.129	9.81E-15	TAIR|locus:2200216 - symbol:ATTPS6 "AT1G68020" species:3702 "Arabidopsis thaliana" [GO:0004805 "trehalose-phosphatase activity" evidence=IGI;ISS;IMP] [GO:0005992 "trehalose biosynthetic process" evidence=IGI;ISS] [GO:0016757 "transferase activity, transferring glycosyl groups" evidence=ISS] [GO:0003825 "alpha,alpha-trehalose-phosphate synthase (UDP-forming) activity" evidence=IGI;IMP] InterPro:IPR001830 InterPro:IPR003337 InterPro:IPR006379 Pfam:PF00982 Pfam:PF02358 EMBL:CP002684 GenomeReviews:CT485782_GR InterPro:IPR023214 Gene3D:G3DSA:3.40.50.1000 SUPFAM:SSF56784 EMBL:AC012563 GO:GO:0004805 GO:GO:0005992 TIGRFAMs:TIGR01484 CAZy:GT20 eggNOG:COG0380 HOGENOM:HBG559076 KO:K00697 GO:GO:0003825 HSSP:P31677 TIGRFAMs:TIGR00685 OMA:LMQHPEW EMBL:AY046028 IPI:IPI00516798 IPI:IPI00538850 PIR:C96703 RefSeq:NP_564918.1 RefSeq:NP_974105.1 UniGene:At.19364 ProteinModelPortal:Q94AH8 SMR:Q94AH8 STRING:Q94AH8 PRIDE:Q94AH8 EnsemblPlants:AT1G68020.2 GeneID:843130 KEGG:ath:AT1G68020 TAIR:At1g68020 InParanoid:Q94AH8 PhylomeDB:Q94AH8 ProtClustDB:PLN02205 ArrayExpress:Q94AH8 Genevestigator:Q94AH8 Uniprot:Q94AH8
Root	Isotig04440	9	92	-3.483	5.13E-20	TAIR|locus:2200216 - symbol:ATTPS6 "AT1G68020" species:3702 "Arabidopsis thaliana" [GO:0004805 "trehalose-phosphatase activity" evidence=IGI;ISS;IMP] [GO:0005992 "trehalose biosynthetic process" evidence=IGI;ISS] [GO:0016757 "transferase activity, transferring glycosyl groups" evidence=ISS] [GO:0003825 "alpha,alpha-trehalose-phosphate synthase (UDP-forming) activity" evidence=IGI;IMP] InterPro:IPR001830 InterPro:IPR003337 InterPro:IPR006379 Pfam:PF00982 Pfam:PF02358 EMBL:CP002684 GenomeReviews:CT485782_GR InterPro:IPR023214 Gene3D:G3DSA:3.40.50.1000 SUPFAM:SSF56784 EMBL:AC012563 GO:GO:0004805 GO:GO:0005992 TIGRFAMs:TIGR01484 CAZy:GT20 eggNOG:COG0380 HOGENOM:HBG559076 KO:K00697 GO:GO:0003825 HSSP:P31677 TIGRFAMs:TIGR00685 OMA:LMQHPEW EMBL:AY046028 IPI:IPI00516798 IPI:IPI00538850 PIR:C96703 RefSeq:NP_564918.1 RefSeq:NP_974105.1 UniGene:At.19364 ProteinModelPortal:Q94AH8 SMR:Q94AH8 STRING:Q94AH8 PRIDE:Q94AH8 EnsemblPlants:AT1G68020.2 GeneID:843130 KEGG:ath:AT1G68020 TAIR:At1g68020 InParanoid:Q94AH8 PhylomeDB:Q94AH8 ProtClustDB:PLN02205 ArrayExpress:Q94AH8 Genevestigator:Q94AH8 Uniprot:Q94AH8
Root	Isotig04456	273	6	5.379	3.36E-63	TAIR|locus:2103488 - symbol:SIP2 "AT3G57520" species:3702 "Arabidopsis thaliana" [GO:0004553 "hydrolase activity, hydrolyzing O-glycosyl compounds" evidence=ISS] [GO:0006979 "response to oxidative stress" evidence=IEP] [GO:0080167 "response to karrikin" evidence=IEP] [GO:0034484 "raffinose catabolic process" evidence=IDA] [GO:0047274 "galactinol-sucrose galactosyltransferase activity" evidence=IDA] [GO:0009506 "plasmodesma" evidence=IDA] InterPro:IPR013785 GO:GO:0009506 EMBL:CP002686 GenomeReviews:BA000014_GR GO:GO:0006979 Gene3D:G3DSA:3.20.20.70 InterPro:IPR017853 SUPFAM:SSF51445 GO:GO:0080167 EMBL:AL133248 CAZy:GH36 eggNOG:NOG06986 InterPro:IPR008811 Pfam:PF05691 GO:GO:0034484 HOGENOM:HBG611539 KO:K06617 GO:GO:0047274 EMBL:AY050772 EMBL:AK226370 IPI:IPI00526258 IPI:IPI00541537 IPI:IPI00544535 PIR:T46188 RefSeq:NP_191311.1 RefSeq:NP_850715.1 UniGene:At.22207 UniGene:At.30900 ProteinModelPortal:Q94A08 STRING:Q94A08 PRIDE:Q94A08 EnsemblPlants:AT3G57520.1 GeneID:824919 KEGG:ath:AT3G57520 TAIR:At3g57520 InParanoid:Q9SCM1 OMA:QFMLLES PhylomeDB:Q94A08 ProtClustDB:PLN02219 BioCyc:MetaCyc:AT3G57520-MONOMER ArrayExpress:Q94A08 Uniprot:Q94A08
Root	Isotig04459	76	25	1.475	1.79E-06	TAIR|locus:2093227 - symbol:PLDALPHA1 "AT3G15730" species:3702 "Arabidopsis thaliana" [GO:0004630 "phospholipase D activity" evidence=ISS;IDA;IMP] [GO:0005634 "nucleus" evidence=IDA] [GO:0005739 "mitochondrion" evidence=IDA] [GO:0005886 "plasma membrane" evidence=IDA] [GO:0016020 "membrane" evidence=IDA] [GO:0030136 "clathrin-coated vesicle" evidence=IDA] [GO:0006631 "fatty acid metabolic process" evidence=IMP;TAS] [GO:0005515 "protein binding" evidence=IPI] [GO:0009507 "chloroplast" evidence=IDA] [GO:0046686 "response to cadmium ion" evidence=IEP] [GO:0009789 "positive regulation of abscisic acid mediated signaling pathway" evidence=IMP] [GO:0010119 "regulation of stomatal movement" evidence=IMP] [GO:0009845 "seed germination" evidence=IMP] [GO:0005829 "cytosol" evidence=IDA] [GO:0009506 "plasmodesma" evidence=IDA] [GO:0005546 "phosphatidylinositol-4,5-bisphosphate binding" evidence=IDA] InterPro:IPR000008

Tissue	Identification	# of reads (stress)	# of reads (control)	Fold Change	p-value	Description
						InterPro:IPR001736 InterPro:IPR008973 InterPro:IPR011402 Pfam:PF00168 Pfam:PF00614 PIRSF:PIRSF036470 PROSITE:PS50035 SMART:SM00155 SMART:SM00239 GO:GO:0005829 GO:GO:0005739 GO:GO:0005886 GO:GO:0009506 GO:GO:0005634 GO:GO:0005773 GO:GO:0046686 GO:GO:0009507 EMBL:CP002686 GenomeReviews:BA000014_GR GO:GO:0005509 GO:GO:0005515 GO:GO:0009738 GO:GO:0009873 GO:GO:0009789 GO:GO:0010119 GO:GO:0046470 GO:GO:0016042 GO:GO:0006631 GO:GO:0030136 GO:GO:0009845 SUPFAM:SSF49562 PROSITE:PS50004 GO:GO:0005546 EMBL:AB017071 eggNOG:COG1502 InterPro:IPR015679 PANTHER:PTHR18896 GO:GO:0070290 GO:GO:0004630 KO:K01115 EMBL:U36381 EMBL:AF428278 IPI:IPI00527854 RefSeq:NP_188194.1 UniGene:At.23882 ProteinModelPortal:Q38882 SMR:Q38882 IntAct:Q38882 STRING:Q38882 PRIDE:Q38882 DNASU:820816 EnsemblPlants:AT3G15730.1 GeneID:820816 KEGG:ath:AT3G15730 TAIR:At3g15730 HOGENOM:HBG748198 InParanoid:Q38882 OMA:KLQYFDV PhylomeDB:Q38882 ProtClustDB:PLN02270 ArrayExpress:Q38882 Genevestigator:Q38882 GermOnline:AT3G15730 InterPro:IPR024632 Pfam:PF12357 Uniprot:Q38882
Root	Isotig04460	55	17	1.565	2.20E-05	TAIR|locus:2093227 - symbol:PLDALPHA1 "AT3G15730" species:3702 "Arabidopsis thaliana" [GO:0004630 "phospholipase D activity" evidence=ISS;IDA;IMP] [GO:0005634 "nucleus" evidence=IDA] [GO:0005739 "mitochondrion" evidence=IDA] [GO:0005886 "plasma membrane" evidence=IDA] [GO:0016020 "membrane" evidence=IDA] [GO:0030136 "clathrin-coated vesicle" evidence=IDA] [GO:0006631 "fatty acid metabolic process" evidence=IMP;TAS] [GO:0005515 "protein binding" evidence=IPI] [GO:0009507 "chloroplast" evidence=IDA] [GO:0046686 "response to cadmium ion" evidence=IEP] [GO:0009789 "positive regulation of abscisic acid mediated signaling pathway" evidence=IMP] [GO:0010119 "regulation of stomatal movement" evidence=IMP] [GO:0009845 "seed germination" evidence=IMP] [GO:0005829 "cytosol" evidence=IDA] [GO:0009506 "plasmodesma" evidence=IDA] [GO:0005546 "phosphatidylinositol-4,5-bisphosphate binding" evidence=IDA] InterPro:IPR000008 InterPro:IPR001736 InterPro:IPR008973 InterPro:IPR011402 Pfam:PF00168 Pfam:PF00614 PIRSF:PIRSF036470 PROSITE:PS50035 SMART:SM00155 SMART:SM00239 GO:GO:0005829 GO:GO:0005739 GO:GO:0005886 GO:GO:0009506 GO:GO:0005634 GO:GO:0005773 GO:GO:0046686 GO:GO:0009507 EMBL:CP002686 GenomeReviews:BA000014_GR GO:GO:0005509 GO:GO:0005515 GO:GO:0009738 GO:GO:0009873 GO:GO:0009789 GO:GO:0010119 GO:GO:0046470 GO:GO:0016042 GO:GO:0006631 GO:GO:0030136 GO:GO:0009845 SUPFAM:SSF49562 PROSITE:PS50004 GO:GO:0005546 EMBL:AB017071 eggNOG:COG1502 InterPro:IPR015679 PANTHER:PTHR18896 GO:GO:0070290 GO:GO:0004630 KO:K01115 EMBL:U36381 EMBL:AF428278 IPI:IPI00527854 RefSeq:NP_188194.1 UniGene:At.23882 ProteinModelPortal:Q38882 SMR:Q38882 IntAct:Q38882 STRING:Q38882 PRIDE:Q38882 DNASU:820816 EnsemblPlants:AT3G15730.1 GeneID:820816 KEGG:ath:AT3G15730 TAIR:At3g15730 HOGENOM:HBG748198 InParanoid:Q38882 OMA:KLQYFDV PhylomeDB:Q38882 ProtClustDB:PLN02270 ArrayExpress:Q38882 Genevestigator:Q38882 GermOnline:AT3G15730 InterPro:IPR024632 Pfam:PF12357 Uniprot:Q38882
Root	Isotig04461	46	15	1.488	0.000184664	TAIR|locus:2093227 - symbol:PLDALPHA1 "AT3G15730" species:3702 "Arabidopsis thaliana" [GO:0004630 "phospholipase D activity" evidence=ISS;IDA;IMP] [GO:0005634 "nucleus" evidence=IDA] [GO:0005739 "mitochondrion" evidence=IDA] [GO:0005886 "plasma membrane" evidence=IDA] [GO:0016020 "membrane" evidence=IDA] [GO:0030136 "clathrin-coated vesicle" evidence=IDA] [GO:0006631 "fatty acid metabolic process" evidence=IMP;TAS] [GO:0005515 "protein binding" evidence=IPI] [GO:0009507 "chloroplast" evidence=IDA] [GO:0046686 "response to cadmium ion" evidence=IEP] [GO:0009789 "positive regulation of abscisic acid mediated signaling pathway" evidence=IMP] [GO:0010119 "regulation of stomatal movement" evidence=IMP] [GO:0009845 "seed germination" evidence=IMP] [GO:0005829 "cytosol" evidence=IDA] [GO:0009506 "plasmodesma" evidence=IDA] [GO:0005546 "phosphatidylinositol-4,5-bisphosphate binding" evidence=IDA] InterPro:IPR000008 InterPro:IPR001736 InterPro:IPR008973 InterPro:IPR011402 Pfam:PF00168 Pfam:PF00614 PIRSF:PIRSF036470 PROSITE:PS50035 SMART:SM00155 SMART:SM00239 GO:GO:0005829 GO:GO:0005739 GO:GO:0005886 GO:GO:0009506 GO:GO:0005634 GO:GO:0005773 GO:GO:0046686 GO:GO:0009507 EMBL:CP002686 GenomeReviews:BA000014_GR GO:GO:0005509 GO:GO:0005515 GO:GO:0009738 GO:GO:0009873 GO:GO:0009789 GO:GO:0010119 GO:GO:0046470 GO:GO:0016042 GO:GO:0006631 GO:GO:0030136 GO:GO:0009845 SUPFAM:SSF49562 PROSITE:PS50004 GO:GO:0005546 EMBL:AB017071 eggNOG:COG1502 InterPro:IPR015679 PANTHER:PTHR18896 GO:GO:0070290 GO:GO:0004630 KO:K01115 EMBL:U36381 EMBL:AF428278 IPI:IPI00527854 RefSeq:NP_188194.1 UniGene:At.23882 ProteinModelPortal:Q38882 SMR:Q38882 IntAct:Q38882 STRING:Q38882 PRIDE:Q38882 DNASU:820816 EnsemblPlants:AT3G15730.1 GeneID:820816 KEGG:ath:AT3G15730 TAIR:At3g15730 HOGENOM:HBG748198 InParanoid:Q38882

Tissue	Identification	# of reads (stress)	# of reads (control)	Fold Change	p-value	Description
						OMA:KLQYFDV PhylomeDB:Q38882 ProtClustDB:PLN02270 ArrayExpress:Q38882 Genevestigator:Q38882 GermOnline:AT3G15730 InterPro:IPR024632 Pfam:PF12357 Uniprot:Q38882
Root	Isotig04483	10	42	-2.199	1.05E-06	TAIR|locus:2152445 - symbol:SAG12 "AT5G45890" species:3702 "Arabidopsis thaliana" [GO:0006508 "proteolysis" evidence=ISS] [GO:0008234 "cysteine-type peptidase activity" evidence=ISS] [GO:0009723 "response to ethylene stimulus" evidence=IEP] [GO:0007568 "aging" evidence=IEP;TAS] [GO:0010150 "leaf senescence" evidence=TAS] [GO:0010282 "senescence-associated vacuole" evidence=IDA] [GO:0009817 "defense response to fungus, incompatible interaction" evidence=IEP] InterPro:IPR000668 InterPro:IPR013128 Pfam:PF00112 PRINTS:PR00705 SMART:SM00645 InterPro:IPR000169 EMBL:CP002688 GO:GO:0004197 GO:GO:0006508 InterPro:IPR013201 PANTHER:PTHR12411 Pfam:PF08246 SMART:SM00848 PROSITE:PS00640 PROSITE:PS00139 PROSITE:PS00639 GO:GO:0010150 GO:GO:0009817 EMBL:AB016870 HSSP:O65039 EMBL:AF370131 EMBL:AY040073 IPI:IPI00544181 RefSeq:NP_568651.1 UniGene:At.75256 UniGene:At.7710 ProteinModelPortal:Q9FJ47 SMR:Q9FJ47 IntAct:Q9FJ47 STRING:Q9FJ47 MEROPS:C01.117 PRIDE:Q9FJ47 EnsemblPlants:AT5G45890.1 GeneID:834629 KEGG:ath:AT5G45890 TAIR:At5g45890 InParanoid:Q9FJ47 OMA:NDEQALM PhylomeDB:Q9FJ47 ProtClustDB:CLSN2917735 ArrayExpress:Q9FJ47 Genevestigator:Q9FJ47 GO:GO:0010282 Uniprot:Q9FJ47
Root	Isotig04485	0	27	-5.884	3.19E-08	TAIR|locus:2152445 - symbol:SAG12 "AT5G45890" species:3702 "Arabidopsis thaliana" [GO:0006508 "proteolysis" evidence=ISS] [GO:0008234 "cysteine-type peptidase activity" evidence=ISS] [GO:0009723 "response to ethylene stimulus" evidence=IEP] [GO:0007568 "aging" evidence=IEP;TAS] [GO:0010150 "leaf senescence" evidence=TAS] [GO:0010282 "senescence-associated vacuole" evidence=IDA] [GO:0009817 "defense response to fungus, incompatible interaction" evidence=IEP] InterPro:IPR000668 InterPro:IPR013128 Pfam:PF00112 PRINTS:PR00705 SMART:SM00645 InterPro:IPR000169 EMBL:CP002688 GO:GO:0004197 GO:GO:0006508 InterPro:IPR013201 PANTHER:PTHR12411 Pfam:PF08246 SMART:SM00848 PROSITE:PS00640 PROSITE:PS00139 PROSITE:PS00639 GO:GO:0010150 GO:GO:0009817 EMBL:AB016870 HSSP:O65039 EMBL:AF370131 EMBL:AY040073 IPI:IPI00544181 RefSeq:NP_568651.1 UniGene:At.75256 UniGene:At.7710 ProteinModelPortal:Q9FJ47 SMR:Q9FJ47 IntAct:Q9FJ47 STRING:Q9FJ47 MEROPS:C01.117 PRIDE:Q9FJ47 EnsemblPlants:AT5G45890.1 GeneID:834629 KEGG:ath:AT5G45890 TAIR:At5g45890 InParanoid:Q9FJ47 OMA:NDEQALM PhylomeDB:Q9FJ47 ProtClustDB:CLSN2917735 ArrayExpress:Q9FJ47 Genevestigator:Q9FJ47 GO:GO:0010282 Uniprot:Q9FJ47
Root	Isotig04489	8	35	-2.258	5.90E-06	TAIR|locus:2088050 - symbol:RFR1 "AT3G23590" species:3702 "Arabidopsis thaliana" [GO:0003674 "molecular_function" evidence=ND] [GO:0005575 "cellular_component" evidence=ND] [GO:2000762 "regulation of phenylpropanoid metabolic process" evidence=IMP] EMBL:CP002686 EMBL:AB023036 IPI:IPI00527950 RefSeq:NP_189001.1 UniGene:At.23102 IntAct:Q9LUG9 PRIDE:Q9LUG9 EnsemblPlants:AT3G23590.1 GeneID:821938 KEGG:ath:AT3G23590 TAIR:At3g23590 InParanoid:Q9LUG9 OMA:VNGRINQ PhylomeDB:Q9LUG9 ProtClustDB:CLSN2684326 Genevestigator:Q9LUG9 Uniprot:Q9LUG9
Root	Isotig04498	48	218	-2.312	1.88E-30	TAIR|locus:2057981 - symbol:PAL1 "PHE ammonia lyase 1" species:3702 "Arabidopsis thaliana" [GO:0005737 "cytoplasm" evidence=IEA] [GO:0009699 "phenylpropanoid biosynthetic process" evidence=TAS] [GO:0045548 "phenylalanine ammonia-lyase activity" evidence=ISS;IDA;TAS] [GO:0006979 "response to oxidative stress" evidence=IEP] [GO:0080167 "response to karrikin" evidence=IEP] [GO:0009555 "pollen development" evidence=IMP] [GO:0009819 "drought recovery" evidence=IMP] [GO:0010224 "response to UV-B" evidence=IMP] [GO:0046244 "salicylic acid catabolic process" evidence=IMP] [GO:0046274 "lignin catabolic process" evidence=IMP] [GO:0006952 "defense response" evidence=TAS] [GO:0009611 "response to wounding" evidence=TAS] InterPro:IPR001106 InterPro:IPR005922 InterPro:IPR008948 InterPro:IPR022313 Pfam:PF00221 PROSITE:PS00488 GO:GO:0005737 EMBL:CP002685 GenomeReviews:CT485783_GR GO:GO:0006979 GO:GO:0009058 GO:GO:0080167 EMBL:AC006922 GO:GO:0009555 SUPFAM:SSF48557 GO:GO:0006559 InterPro:IPR024083 Gene3D:G3DSA:1.10.275.10 GO:GO:0010224 eggNOG:COG2986 GO:GO:0046274 EMBL:L33677 EMBL:AY303128 EMBL:AY045919 EMBL:AY079363 EMBL:BT003330 EMBL:X62747 IPI:IPI00519512 PIR:G84787 PIR:S52990 RefSeq:NP_181241.1 UniGene:At.21614 UniGene:At.22705 ProteinModelPortal:P35510 SMR:P35510 IntAct:P35510 STRING:P35510 PRIDE:P35510 EnsemblPlants:AT2G37040.1 GeneID:818280 KEGG:ath:AT2G37040 TAIR:At2g37040 HOGENOM:HBG318028 InParanoid:P35510 KO:K10775 OMA:QRGADFL PhylomeDB:P35510 ProtClustDB:PLN02457 BRENDA:4.3.1.24 Genevestigator:P35510 GermOnline:AT2G37040 GO:GO:0045548 GO:GO:0009819 GO:GO:0046244 InterPro:IPR023144 Gene3D:G3DSA:1.10.274.20 TIGRFAMs:TIGR01226 Uniprot:P35510

Tissue	Identification	# of reads (stress)	# of reads (control)	Fold Change	p-value	Description
Root	Isotig04499	37	173	-2.354	5.33E-25	TAIR|locus:2057981 - symbol:PAL1 "PHE ammonia lyase 1" species:3702 "Arabidopsis thaliana" [GO:0005737 "cytoplasm" evidence=IEA] [GO:0009699 "phenylpropanoid biosynthetic process" evidence=TAS] [GO:0045548 "phenylalanine ammonia-lyase activity" evidence=ISS;IDA;TAS] [GO:0006979 "response to oxidative stress" evidence=IEP] [GO:0080167 "response to karrikin" evidence=IEP] [GO:0009555 "pollen development" evidence=IMP] [GO:0009819 "drought recovery" evidence=IMP] [GO:0010224 "response to UV-B" evidence=IMP] [GO:0046244 "salicylic acid catabolic process" evidence=IMP] [GO:0046274 "lignin catabolic process" evidence=IMP] [GO:0006952 "defense response" evidence=TAS] [GO:0009611 "response to wounding" evidence=TAS] InterPro:IPR001106 InterPro:IPR005922 InterPro:IPR008948 InterPro:IPR022313 Pfam:PF00221 PROSITE:PS00488 GO:GO:0005737 EMBL:CP002685 GenomeReviews:CT485783_GR GO:GO:0006979 GO:GO:0009058 GO:GO:0080167 EMBL:AC006922 GO:GO:0009555 SUPFAM:SSF48557 GO:GO:0006559 InterPro:IPR024083 Gene3D:G3DSA:1.10.275.10 GO:GO:0010224 eggNOG:COG2986 GO:GO:0046274 EMBL:L33677 EMBL:AY303128 EMBL:AY045919 EMBL:AY079363 EMBL:BT003330 EMBL:X62747 IPI:IPI00519512 PIR:G84787 PIR:S52990 RefSeq:NP_181241.1 UniGene:At.21614 UniGene:At.22705 ProteinModelPortal:P35510 SMR:P35510 IntAct:P35510 STRING:P35510 PRIDE:P35510 EnsemblPlants:AT2G37040.1 GeneID:818280 KEGG:ath:AT2G37040 TAIR:At2g37040 HOGENOM:HBG318028 InParanoid:P35510 KO:K10775 OMA:QRGADFL PhylomeDB:P35510 ProtClustDB:PLN02457 BRENDA:4.3.1.24 Genevestigator:P35510 GermOnline:AT2G37040 GO:GO:0045548 GO:GO:0009819 GO:GO:0046244 InterPro:IPR023144 Gene3D:G3DSA:1.10.274.20 TIGRFAMs:TIGR01226 Uniprot:P35510
Root	Isotig04500	59	190	-1.816	4.94E-20	TAIR|locus:2057981 - symbol:PAL1 "PHE ammonia lyase 1" species:3702 "Arabidopsis thaliana" [GO:0005737 "cytoplasm" evidence=IEA] [GO:0009699 "phenylpropanoid biosynthetic process" evidence=TAS] [GO:0045548 "phenylalanine ammonia-lyase activity" evidence=ISS;IDA;TAS] [GO:0006979 "response to oxidative stress" evidence=IEP] [GO:0080167 "response to karrikin" evidence=IEP] [GO:0009555 "pollen development" evidence=IMP] [GO:0009819 "drought recovery" evidence=IMP] [GO:0010224 "response to UV-B" evidence=IMP] [GO:0046244 "salicylic acid catabolic process" evidence=IMP] [GO:0046274 "lignin catabolic process" evidence=IMP] [GO:0006952 "defense response" evidence=TAS] [GO:0009611 "response to wounding" evidence=TAS] InterPro:IPR001106 InterPro:IPR005922 InterPro:IPR008948 InterPro:IPR022313 Pfam:PF00221 PROSITE:PS00488 GO:GO:0005737 EMBL:CP002685 GenomeReviews:CT485783_GR GO:GO:0006979 GO:GO:0009058 GO:GO:0080167 EMBL:AC006922 GO:GO:0009555 SUPFAM:SSF48557 GO:GO:0006559 InterPro:IPR024083 Gene3D:G3DSA:1.10.275.10 GO:GO:0010224 eggNOG:COG2986 GO:GO:0046274 EMBL:L33677 EMBL:AY303128 EMBL:AY045919 EMBL:AY079363 EMBL:BT003330 EMBL:X62747 IPI:IPI00519512 PIR:G84787 PIR:S52990 RefSeq:NP_181241.1 UniGene:At.21614 UniGene:At.22705 ProteinModelPortal:P35510 SMR:P35510 IntAct:P35510 STRING:P35510 PRIDE:P35510 EnsemblPlants:AT2G37040.1 GeneID:818280 KEGG:ath:AT2G37040 TAIR:At2g37040 HOGENOM:HBG318028 InParanoid:P35510 KO:K10775 OMA:QRGADFL PhylomeDB:P35510 ProtClustDB:PLN02457 BRENDA:4.3.1.24 Genevestigator:P35510 GermOnline:AT2G37040 GO:GO:0045548 GO:GO:0009819 GO:GO:0046244 InterPro:IPR023144 Gene3D:G3DSA:1.10.274.20 TIGRFAMs:TIGR01226 Uniprot:P35510
Root	Isotig04515	119	61	0.835	0.000156576	TAIR|locus:2161815 - symbol:HSP81-2 "AT5G56030" species:3702 "Arabidopsis thaliana" [GO:0005524 "ATP binding" evidence=ISS;IDA] [GO:0006457 "protein folding" evidence=ISS] [GO:0009408 "response to heat" evidence=IEP] [GO:0005515 "protein binding" evidence=IPI] [GO:0006952 "defense response" evidence=IMP] [GO:0009908 "flower development" evidence=IMP] [GO:0048366 "leaf development" evidence=IMP] [GO:0050821 "protein stabilization" evidence=IMP] [GO:0005618 "cell wall" evidence=IDA] [GO:0010286 "heat acclimation" evidence=IEP] [GO:0005739 "mitochondrion" evidence=IDA] [GO:0005634 "nucleus" evidence=IDA] [GO:0005829 "cytosol" evidence=IDA] [GO:0009414 "response to water deprivation" evidence=IMP] [GO:0009651 "response to salt stress" evidence=IMP] [GO:0009816 "defense response to bacterium, incompatible interaction" evidence=IMP] [GO:0016887 "ATPase activity" evidence=IDA] [GO:0009506 "plasmodesma" evidence=IDA] [GO:0010187 "negative regulation of seed germination" evidence=IMP] [GO:0090332 "stomatal closure" evidence=IMP] [GO:0005794 "Golgi apparatus" evidence=IDA] InterPro:IPR001404 InterPro:IPR003594 InterPro:IPR019805 Pfam:PF00183 PIRSF:PIRSF002583 PROSITE:PS00298 SMART:SM00387 GO:GO:0005829 GO:GO:0005739 GO:GO:0009506 GO:GO:0005524 GO:GO:0005634 EMBL:CP002688 GenomeReviews:BA000015_GR GO:GO:0005618 GO:GO:0006457 GO:GO:0050821 GO:GO:0051082 GO:GO:0016887 GO:GO:0009651 GO:GO:0009414 GO:GO:0010187 GO:GO:0048366 GO:GO:0010286 GO:GO:0009908 GO:GO:0009816 Gene3D:G3DSA:3.30.565.10 SUPFAM:SSF55874 InterPro:IPR020568 SUPFAM:SSF54211 EMBL:AB011476 eggNOG:COG0326 HOGENOM:HBG631012 PANTHER:PTHR11528 InterPro:IPR020575 PRINTS:PR00775

Tissue	Identification	# of reads (stress)	# of reads (control)	Fold Change	p-value	Description
						KO:K04079 ProtClustDB:CLSN2686109 EMBL:AY062750 EMBL:AY128805 EMBL:BT001944 EMBL:BT002535 IPI:IPI00537112 RefSeq:NP_200414.1 UniGene:At.25243 UniGene:At.27546 UniGene:At.45945 ProteinModelPortal:P55737 SMR:P55737 IntAct:P55737 STRING:P55737 PRIDE:P55737 EnsemblPlants:AT5G56030.1 GeneID:835701 KEGG:ath:AT5G56030 TAIR:At5g56030 InParanoid:P55737 OMA:GFSKNIK PhylomeDB:P55737 Genevestigator:P55737 GermOnline:AT5G56030 GO:GO:0090332 Uniprot:P55737
Root	Isotig04516	14	39	-1.607	0.000139286	TAIR|locus:2036199 - symbol:AT1G15740 "AT1G15740" species:3702 "Arabidopsis thaliana" [GO:0005575 "cellular_component" evidence=ND] EMBL:CP002684 GenomeReviews:CT485782_GR HSSP:P25147 eggNOG:NOG69615 UniGene:At.48194 UniGene:At.69991 EMBL:AY142631 IPI:IPI00545317 RefSeq:NP_563980.2 ProteinModelPortal:Q8H1Q4 SMR:Q8H1Q4 STRING:Q8H1Q4 PRIDE:Q8H1Q4 EnsemblPlants:AT1G15740.1 GeneID:838143 KEGG:ath:AT1G15740 TAIR:At1g15740 HOGENOM:HBG592941 InParanoid:Q8H1Q4 OMA:GSNGLRH PhylomeDB:Q8H1Q4 ProtClustDB:CLSN2679913 Genevestigator:Q8H1Q4 Uniprot:Q8H1Q4
Root	Isotig04517	15	38	-1.470	0.00042777	TAIR|locus:2036199 - symbol:AT1G15740 "AT1G15740" species:3702 "Arabidopsis thaliana" [GO:0005575 "cellular_component" evidence=ND] EMBL:CP002684 GenomeReviews:CT485782_GR HSSP:P25147 eggNOG:NOG69615 UniGene:At.48194 UniGene:At.69991 EMBL:AY142631 IPI:IPI00545317 RefSeq:NP_563980.2 ProteinModelPortal:Q8H1Q4 SMR:Q8H1Q4 STRING:Q8H1Q4 PRIDE:Q8H1Q4 EnsemblPlants:AT1G15740.1 GeneID:838143 KEGG:ath:AT1G15740 TAIR:At1g15740 HOGENOM:HBG592941 InParanoid:Q8H1Q4 OMA:GSNGLRH PhylomeDB:Q8H1Q4 ProtClustDB:CLSN2679913 Genevestigator:Q8H1Q4 Uniprot:Q8H1Q4
Root	Isotig04518	15	38	-1.470	0.00042777	TAIR|locus:2036199 - symbol:AT1G15740 "AT1G15740" species:3702 "Arabidopsis thaliana" [GO:0005575 "cellular_component" evidence=ND] EMBL:CP002684 GenomeReviews:CT485782_GR HSSP:P25147 eggNOG:NOG69615 UniGene:At.48194 UniGene:At.69991 EMBL:AY142631 IPI:IPI00545317 RefSeq:NP_563980.2 ProteinModelPortal:Q8H1Q4 SMR:Q8H1Q4 STRING:Q8H1Q4 PRIDE:Q8H1Q4 EnsemblPlants:AT1G15740.1 GeneID:838143 KEGG:ath:AT1G15740 TAIR:At1g15740 HOGENOM:HBG592941 InParanoid:Q8H1Q4 OMA:GSNGLRH PhylomeDB:Q8H1Q4 ProtClustDB:CLSN2679913 Genevestigator:Q8H1Q4 Uniprot:Q8H1Q4
Root	Isotig04520	34	4	2.958	7.26E-07	TAIR|locus:2159300 - symbol:ABCF1 "AT5G60790" species:3702 "Arabidopsis thaliana" [GO:0005215 "transporter activity" evidence=ISS] [GO:0005886 "plasma membrane" evidence=IDA] InterPro:IPR003439 InterPro:IPR003593 InterPro:IPR017871 Pfam:PF00005 PROSITE:PS00211 PROSITE:PS50893 SMART:SM00382 GO:GO:0005886 GO:GO:0005524 EMBL:CP002688 GenomeReviews:BA000015_GR GO:GO:0006810 GO:GO:0016887 HOGENOM:HBG758042 EMBL:AB015472 EMBL:AY140066 EMBL:BT000946 EMBL:BT008363 EMBL:AK317570 EMBL:AK317690 IPI:IPI00540333 RefSeq:NP_200887.1 UniGene:At.9321 ProteinModelPortal:Q9FJH6 SMR:Q9FJH6 IntAct:Q9FJH6 STRING:Q9FJH6 PRIDE:Q9FJH6 EnsemblPlants:AT5G60790.1 GeneID:836200 KEGG:ath:AT5G60790 TAIR:At5g60790 eggNOG:COG0488 InParanoid:Q9FJH6 KO:K06185 OMA:YHQHLHE PhylomeDB:Q9FJH6 ProtClustDB:CLSN2686703 ArrayExpress:Q9FJH6 Genevestigator:Q9FJH6 Uniprot:Q9FJH6
Root	Isotig04521	35	4	3.000	4.17E-07	TAIR|locus:2159300 - symbol:ABCF1 "AT5G60790" species:3702 "Arabidopsis thaliana" [GO:0005215 "transporter activity" evidence=ISS] [GO:0005886 "plasma membrane" evidence=IDA] InterPro:IPR003439 InterPro:IPR003593 InterPro:IPR017871 Pfam:PF00005 PROSITE:PS00211 PROSITE:PS50893 SMART:SM00382 GO:GO:0005886 GO:GO:0005524 EMBL:CP002688 GenomeReviews:BA000015_GR GO:GO:0006810 GO:GO:0016887 HOGENOM:HBG758042 EMBL:AB015472 EMBL:AY140066 EMBL:BT000946 EMBL:BT008363 EMBL:AK317570 EMBL:AK317690 IPI:IPI00540333 RefSeq:NP_200887.1 UniGene:At.9321 ProteinModelPortal:Q9FJH6 SMR:Q9FJH6 IntAct:Q9FJH6 STRING:Q9FJH6 PRIDE:Q9FJH6 EnsemblPlants:AT5G60790.1 GeneID:836200 KEGG:ath:AT5G60790 TAIR:At5g60790 eggNOG:COG0488 InParanoid:Q9FJH6 KO:K06185 OMA:YHQHLHE PhylomeDB:Q9FJH6 ProtClustDB:CLSN2686703 ArrayExpress:Q9FJH6 Genevestigator:Q9FJH6 Uniprot:Q9FJH6
Root	Isotig04522	64	4	3.871	3.12E-14	TAIR|locus:2181833 - symbol:HSC70-1 "heat shock cognate protein 70-1" species:3702 "Arabidopsis thaliana" [GO:0005524 "ATP binding" evidence=IEA] [GO:0005829 "cytosol" evidence=IDA;RCA;TAS] [GO:0006457 "protein folding" evidence=TAS] [GO:0009408 "response to heat" evidence=IEP;IMP] [GO:0005634 "nucleus" evidence=IDA] [GO:0009409 "response to cold" evidence=IEP] [GO:0009507 "chloroplast" evidence=IDA] [GO:0005730 "nucleolus" evidence=IDA] [GO:0009615 "response to virus" evidence=IEP] [GO:0022626 "cytosolic ribosome" evidence=IDA] [GO:0005618 "cell wall" evidence=IDA] [GO:0046686 "response to cadmium ion" evidence=IEP] [GO:0005774 "vacuolar membrane" evidence=IDA] [GO:0005886 "plasma membrane" evidence=IDA] [GO:0016020 "membrane"

Tissue	Identification	# of reads (stress)	# of reads (control)	Fold Change	p-value	Description
						evidence=IDA] [GO:0005515 "protein binding" evidence=IPI] [GO:0005737 "cytoplasm" evidence=IDA] [GO:0042742 "defense response to bacterium" evidence=IMP] [GO:0050832 "defense response to fungus" evidence=IMP] [GO:0048046 "apoplast" evidence=IDA] [GO:0002020 "protease binding" evidence=IPI] [GO:0009506 "plasmodesma" evidence=IDA] [GO:0010187 "negative regulation of seed germination" evidence=IMP] [GO:0090332 "stomatal closure" evidence=IMP] [GO:0005794 "Golgi apparatus" evidence=IDA] InterPro:IPR001023 PRINTS:PR00301 Pfam:PF00012 Prosite:PS00297 Prosite:PS00329 Prosite:PS01036 GO:GO:0005886 GO:GO:0009506 GO:GO:0005524 GO:GO:0048046 GO:GO:0046686 GO:GO:0005774 EMBL:CP002688 GenomeReviews:BA000015_GR GO:GO:0005618 GO:GO:0009507 GO:GO:0042742 GO:GO:0005730 GO:GO:0050832 GO:GO:0002020 GO:GO:0009409 GO:GO:0009408 GO:GO:0009615 GO:GO:0010187 UniGene:At.23663 GO:GO:0022626 eggNOG:COG0443 HOGENOM:HBG334976 InterPro:IPR018181 InterPro:IPR013126 PANTHER:PTHR19375 EMBL:AL162971 KO:K03283 OMA:MAKSTAI GO:GO:0090332 EMBL:X74604 EMBL:AY035123 EMBL:AY057481 EMBL:AY120747 EMBL:BT002754 EMBL:M23108 EMBL:M23105 EMBL:X77199 IPI:IPI00543293 PIR:S46302 PIR:T48271 RefSeq:NP_195870.1 UniGene:At.75212 ProteinModelPortal:P22953 SMR:P22953 IntAct:P22953 STRING:P22953 TCDB:1.A.33.1.1 SWISS-2DPAGE:P22953 World-2DPAGE:0003:P22953 PRIDE:P22953 ProMEX:P22953 EnsemblPlants:AT5G02500.1 GeneID:831020 KEGG:ath:AT5G02500 TAIR:At5g02500 InParanoid:P22953 PhylomeDB:P22953 ProtClustDB:CLSN2916777 Genevestigator:P22953 GermOnline:AT5G02500 Uniprot:P22953
Root	Isotig04523	64	9	2.701	8.63E-11	TAIR|locus:2181833 - symbol:HSC70-1 "heat shock cognate protein 70-1" species:3702 "Arabidopsis thaliana" [GO:0005524 "ATP binding" evidence=IEA] [GO:0005829 "cytosol" evidence=IDA;RCA;TAS] [GO:0006457 "protein folding" evidence=TAS] [GO:0009408 "response to heat" evidence=IEP;IMP] [GO:0005634 "nucleus" evidence=IDA] [GO:0009409 "response to cold" evidence=IEP] [GO:0009507 "chloroplast" evidence=IDA] [GO:0005730 "nucleolus" evidence=IDA] [GO:0009615 "response to virus" evidence=IEP] [GO:0022626 "cytosolic ribosome" evidence=IDA] [GO:0005618 "cell wall" evidence=IDA] [GO:0046686 "response to cadmium ion" evidence=IEP] [GO:0005774 "vacuolar membrane" evidence=IDA] [GO:0005886 "plasma membrane" evidence=IDA] [GO:0016020 "membrane" evidence=IDA] [GO:0005515 "protein binding" evidence=IPI] [GO:0005737 "cytoplasm" evidence=IDA] [GO:0042742 "defense response to bacterium" evidence=IMP] [GO:0050832 "defense response to fungus" evidence=IMP] [GO:0048046 "apoplast" evidence=IDA] [GO:0002020 "protease binding" evidence=IPI] [GO:0009506 "plasmodesma" evidence=IDA] [GO:0010187 "negative regulation of seed germination" evidence=IMP] [GO:0090332 "stomatal closure" evidence=IMP] [GO:0005794 "Golgi apparatus" evidence=IDA] InterPro:IPR001023 PRINTS:PR00301 Pfam:PF00012 Prosite:PS00297 Prosite:PS00329 Prosite:PS01036 GO:GO:0005886 GO:GO:0009506 GO:GO:0005524 GO:GO:0048046 GO:GO:0046686 GO:GO:0005774 EMBL:CP002688 GenomeReviews:BA000015_GR GO:GO:0005618 GO:GO:0009507 GO:GO:0042742 GO:GO:0005730 GO:GO:0050832 GO:GO:0002020 GO:GO:0009409 GO:GO:0009408 GO:GO:0009615 GO:GO:0010187 UniGene:At.23663 GO:GO:0022626 eggNOG:COG0443 HOGENOM:HBG334976 InterPro:IPR018181 InterPro:IPR013126 PANTHER:PTHR19375 EMBL:AL162971 KO:K03283 OMA:MAKSTAI GO:GO:0090332 EMBL:X74604 EMBL:AY035123 EMBL:AY057481 EMBL:AY120747 EMBL:BT002754 EMBL:M23108 EMBL:M23105 EMBL:X77199 IPI:IPI00543293 PIR:S46302 PIR:T48271 RefSeq:NP_195870.1 UniGene:At.75212 ProteinModelPortal:P22953 SMR:P22953 IntAct:P22953 STRING:P22953 TCDB:1.A.33.1.1 SWISS-2DPAGE:P22953 World-2DPAGE:0003:P22953 PRIDE:P22953 ProMEX:P22953 EnsemblPlants:AT5G02500.1 GeneID:831020 KEGG:ath:AT5G02500 TAIR:At5g02500 InParanoid:P22953 PhylomeDB:P22953 ProtClustDB:CLSN2916777 Genevestigator:P22953 GermOnline:AT5G02500 Uniprot:P22953
Root	Isotig04524	61	6	3.217	5.21E-12	TAIR|locus:2181833 - symbol:HSC70-1 "heat shock cognate protein 70-1" species:3702 "Arabidopsis thaliana" [GO:0005524 "ATP binding" evidence=IEA] [GO:0005829 "cytosol" evidence=IDA;RCA;TAS] [GO:0006457 "protein folding" evidence=TAS] [GO:0009408 "response to heat" evidence=IEP;IMP] [GO:0005634 "nucleus" evidence=IDA] [GO:0009409 "response to cold" evidence=IEP] [GO:0009507 "chloroplast" evidence=IDA] [GO:0005730 "nucleolus" evidence=IDA] [GO:0009615 "response to virus" evidence=IEP] [GO:0022626 "cytosolic ribosome" evidence=IDA] [GO:0005618 "cell wall" evidence=IDA] [GO:0046686 "response to cadmium ion" evidence=IEP] [GO:0005774 "vacuolar membrane" evidence=IDA] [GO:0005886 "plasma membrane" evidence=IDA] [GO:0016020 "membrane" evidence=IDA] [GO:0005515 "protein binding" evidence=IPI] [GO:0005737 "cytoplasm" evidence=IDA] [GO:0042742 "defense response to bacterium" evidence=IMP] [GO:0050832 "defense response to fungus" evidence=IMP] [GO:0048046 "apoplast" evidence=IDA] [GO:0002020 "protease binding" evidence=IPI] [GO:0009506 "plasmodesma" evidence=IDA] [GO:0010187 "negative regulation of seed germination" evidence=IMP] [GO:0090332 "stomatal closure" evidence=IMP] [GO:0005794 "Golgi apparatus" evidence=IDA] InterPro:IPR001023

Tissue	Identification	# of reads (stress)	# of reads (control)	Fold Change	p-value	Description
						PRINTS:PR00301 Pfam:PF00012 Prosite:PS00297 Prosite:PS00329 Prosite:PS01036 GO:GO:0005886 GO:GO:0009506 GO:GO:0005524 GO:GO:0048046 GO:GO:0046686 GO:GO:0005774 EMBL:CP002688 GenomeReviews:BA000015_GR GO:GO:0005618 GO:GO:0009507 GO:GO:0042742 GO:GO:0005730 GO:GO:0050832 GO:GO:0002020 GO:GO:0009409 GO:GO:0009408 GO:GO:0009615 GO:GO:0010187 UniGene:At.23663 GO:GO:0022626 eggNOG:COG0443 HOGENOM:HBG334976 InterPro:IPR018181 InterPro:IPR013126 PANTHER:PTHR19375 EMBL:AL162971 KO:K03283 OMA:MAKSTAI GO:GO:0090332 EMBL:X74604 EMBL:AY035123 EMBL:AY057481 EMBL:AY120747 EMBL:BT002754 EMBL:M23108 EMBL:M23105 EMBL:X77199 IPI:IPI00543293 PIR:S46302 PIR:T48271 RefSeq:NP_195870.1 UniGene:At.75212 ProteinModelPortal:P22953 SMR:P22953 IntAct:P22953 STRING:P22953 TCDB:1.A.33.1.1 SWISS-2DPAGE:P22953 World-2DPAGE:0003:P22953 PRIDE:P22953 ProMEX:P22953 EnsemblPlants:AT5G02500.1 GeneID:831020 KEGG:ath:AT5G02500 TAIR:At5g02500 InParanoid:P22953 PhylomeDB:P22953 ProtClustDB:CLSN2916777 Genevestigator:P22953 GermOnline:AT5G02500 Uniprot:P22953
Root	Isotig04526	17	43	-1.468	0.000182074	TAIR|locus:2179250 - symbol:AT5G25100 "AT5G25100" species:3702 "Arabidopsis thaliana" [GO:0016021 "integral to membrane" evidence=ISS] [GO:0005794 "Golgi apparatus" evidence=IDA] [GO:0005886 "plasma membrane" evidence=IDA] [GO:0009506 "plasmodesma" evidence=IDA] InterPro:IPR004240 Pfam:PF02990 GO:GO:0016021 GO:GO:0005886 GO:GO:0009506 EMBL:CP002688 GenomeReviews:BA000015_GR GO:GO:0005794 eggNOG:NOG315241 HOGENOM:HBG524073 PANTHER:PTHR10766 OMA:VIACSQI EMBL:AF360135 IPI:IPI00521208 RefSeq:NP_568465.1 UniGene:At.19004 UniGene:At.74873 STRING:Q9C5N2 PRIDE:Q9C5N2 EnsemblPlants:AT5G25100.1 GeneID:832581 KEGG:ath:AT5G25100 TAIR:At5g25100 InParanoid:Q9C5N2 PhylomeDB:Q9C5N2 ProtClustDB:CLSN2917711 ArrayExpress:Q9C5N2 Genevestigator:Q9C5N2 Uniprot:Q9C5N2
Root	Isotig04527	16	40	-1.451	0.000345313	TAIR|locus:2179250 - symbol:AT5G25100 "AT5G25100" species:3702 "Arabidopsis thaliana" [GO:0016021 "integral to membrane" evidence=ISS] [GO:0005794 "Golgi apparatus" evidence=IDA] [GO:0005886 "plasma membrane" evidence=IDA] [GO:0009506 "plasmodesma" evidence=IDA] InterPro:IPR004240 Pfam:PF02990 GO:GO:0016021 GO:GO:0005886 GO:GO:0009506 EMBL:CP002688 GenomeReviews:BA000015_GR GO:GO:0005794 eggNOG:NOG315241 HOGENOM:HBG524073 PANTHER:PTHR10766 OMA:VIACSQI EMBL:AF360135 IPI:IPI00521208 RefSeq:NP_568465.1 UniGene:At.19004 UniGene:At.74873 STRING:Q9C5N2 PRIDE:Q9C5N2 EnsemblPlants:AT5G25100.1 GeneID:832581 KEGG:ath:AT5G25100 TAIR:At5g25100 InParanoid:Q9C5N2 PhylomeDB:Q9C5N2 ProtClustDB:CLSN2917711 ArrayExpress:Q9C5N2 Genevestigator:Q9C5N2 Uniprot:Q9C5N2
Root	Isotig04537	11	33	-1.714	0.000243809	TAIR|locus:2026177 - symbol:AT1G62660 species:3702 "Arabidopsis thaliana" [GO:0004553 "hydrolase activity, hydrolyzing O-glycosyl compounds" evidence=IEA;ISS] [GO:0071370 "cellular response to gibberellin stimulus" evidence=IEP] [GO:0000325 "plant-type vacuole" evidence=IDA] [GO:0005773 "vacuole" evidence=IDA] [GO:0009617 "response to bacterium" evidence=IEP] InterPro:IPR001362 InterPro:IPR018053 InterPro:IPR021792 Pfam:PF11837 PROSITE:PS00609 SMART:SM00640 EMBL:CP002684 GO:GO:0005975 InterPro:IPR008985 SUPFAM:SSF49899 InterPro:IPR023296 SUPFAM:SSF75005 CAZy:GH32 KO:K01193 GO:GO:0004575 InterPro:IPR013189 InterPro:IPR013148 Pfam:PF08244 Pfam:PF00251 ProtClustDB:CLSN2687777 EMBL:AY048269 EMBL:AY062481 EMBL:AY063904 EMBL:AY114066 EMBL:X99111 IPI:IPI00517592 PIR:S71268 RefSeq:NP_564798.1 UniGene:At.24113 UniGene:At.67066 ProteinModelPortal:Q43348 SMR:Q43348 STRING:Q43348 PRIDE:Q43348 ProMEX:Q43348 EnsemblPlants:AT1G62660.1 GeneID:842563 KEGG:ath:AT1G62660 TAIR:At1g62660 InParanoid:Q43348 OMA:HSIVEGF PhylomeDB:Q43348 ArrayExpress:Q43348 Genevestigator:Q43348 Uniprot:Q43348
Root	Isotig04538	9	38	-2.207	3.25E-06	TAIR|locus:2026177 - symbol:AT1G62660 species:3702 "Arabidopsis thaliana" [GO:0004553 "hydrolase activity, hydrolyzing O-glycosyl compounds" evidence=IEA;ISS] [GO:0071370 "cellular response to gibberellin stimulus" evidence=IEP] [GO:0000325 "plant-type vacuole" evidence=IDA] [GO:0005773 "vacuole" evidence=IDA] [GO:0009617 "response to bacterium" evidence=IEP] InterPro:IPR001362 InterPro:IPR018053 InterPro:IPR021792 Pfam:PF11837 PROSITE:PS00609 SMART:SM00640 EMBL:CP002684 GO:GO:0005975 InterPro:IPR008985 SUPFAM:SSF49899 InterPro:IPR023296 SUPFAM:SSF75005 CAZy:GH32 KO:K01193 GO:GO:0004575 InterPro:IPR013189 InterPro:IPR013148 Pfam:PF08244 Pfam:PF00251 ProtClustDB:CLSN2687777 EMBL:AY048269 EMBL:AY062481 EMBL:AY063904 EMBL:AY114066 EMBL:X99111 IPI:IPI00517592 PIR:S71268 RefSeq:NP_564798.1 UniGene:At.24113 UniGene:At.67066 ProteinModelPortal:Q43348 SMR:Q43348 STRING:Q43348 PRIDE:Q43348 ProMEX:Q43348 EnsemblPlants:AT1G62660.1 GeneID:842563

Tissue	Identification	# of reads (stress)	# of reads (control)	Fold Change	p-value	Description
						KEGG:ath:AT1G62660 TAIR:At1g62660 InParanoid:Q43348 OMA:HSIVEGF PhylomeDB:Q43348 ArrayExpress:Q43348 Genevestigator:Q43348 Uniprot:Q43348
Root	Isotig04558	72	0	7.041	2.73E-17	No hit
Root	Isotig04559	40	0	6.193	1.34E-10	No hit
Root	Isotig04560	40	0	6.193	1.34E-10	No hit
Root	Isotig04564	3	39	-3.829	6.57E-10	TAIR|locus:2057981 - symbol:PAL1 "PHE ammonia lyase 1" species:3702 "Arabidopsis thaliana" [GO:0005737 "cytoplasm" evidence=IEA] [GO:0009699 "phenylpropanoid biosynthetic process" evidence=TAS] [GO:0045548 "phenylalanine ammonia-lyase activity" evidence=ISS;IDA;TAS] [GO:0006979 "response to oxidative stress" evidence=IEP] [GO:0080167 "response to karrikin" evidence=IEP] [GO:0009555 "pollen development" evidence=IMP] [GO:0009819 "drought recovery" evidence=IMP] [GO:0010224 "response to UV-B" evidence=IMP] [GO:0046244 "salicylic acid catabolic process" evidence=IMP] [GO:0046274 "lignin catabolic process" evidence=IMP] [GO:0006952 "defense response" evidence=TAS] [GO:0009611 "response to wounding" evidence=TAS] InterPro:IPR001106 InterPro:IPR005922 InterPro:IPR008948 InterPro:IPR022313 Pfam:PF00221 PROSITE:PS00488 GO:GO:0005737 EMBL:CP002685 GenomeReviews:CT485783_GR GO:GO:0006979 GO:GO:0009058 GO:GO:0080167 EMBL:AC006922 GO:GO:0009555 SUPFAM:SSF48557 GO:GO:0006559 InterPro:IPR024083 Gene3D:G3DSA:1.10.275.10 GO:GO:0010224 eggNOG:COG2986 GO:GO:0046274 EMBL:L33677 EMBL:AY303128 EMBL:AY045919 EMBL:AY079363 EMBL:BT003330 EMBL:X62747 IPI:IPI00519512 PIR:G84787 PIR:S52990 RefSeq:NP_181241.1 UniGene:At.21614 UniGene:At.22705 ProteinModelPortal:P35510 SMR:P35510 IntAct:P35510 STRING:P35510 PRIDE:P35510 EnsemblPlants:AT2G37040.1 GeneID:818280 KEGG:ath:AT2G37040 TAIR:At2g37040 HOGENOM:HBG318028 InParanoid:P35510 KO:K10775 OMA:QRGADFL PhylomeDB:P35510 ProtClustDB:PLN02457 BRENDA:4.3.1.24 Genevestigator:P35510 GermOnline:AT2G37040 GO:GO:0045548 GO:GO:0009819 GO:GO:0046244 InterPro:IPR023144 Gene3D:G3DSA:1.10.274.20 TIGRFAMs:TIGR01226 Uniprot:P35510
Root	Isotig04565	3	31	-3.498	9.97E-08	TAIR|locus:2057981 - symbol:PAL1 "PHE ammonia lyase 1" species:3702 "Arabidopsis thaliana" [GO:0005737 "cytoplasm" evidence=IEA] [GO:0009699 "phenylpropanoid biosynthetic process" evidence=TAS] [GO:0045548 "phenylalanine ammonia-lyase activity" evidence=ISS;IDA;TAS] [GO:0006979 "response to oxidative stress" evidence=IEP] [GO:0080167 "response to karrikin" evidence=IEP] [GO:0009555 "pollen development" evidence=IMP] [GO:0009819 "drought recovery" evidence=IMP] [GO:0010224 "response to UV-B" evidence=IMP] [GO:0046244 "salicylic acid catabolic process" evidence=IMP] [GO:0046274 "lignin catabolic process" evidence=IMP] [GO:0006952 "defense response" evidence=TAS] [GO:0009611 "response to wounding" evidence=TAS] InterPro:IPR001106 InterPro:IPR005922 InterPro:IPR008948 InterPro:IPR022313 Pfam:PF00221 PROSITE:PS00488 GO:GO:0005737 EMBL:CP002685 GenomeReviews:CT485783_GR GO:GO:0006979 GO:GO:0009058 GO:GO:0080167 EMBL:AC006922 GO:GO:0009555 SUPFAM:SSF48557 GO:GO:0006559 InterPro:IPR024083 Gene3D:G3DSA:1.10.275.10 GO:GO:0010224 eggNOG:COG2986 GO:GO:0046274 EMBL:L33677 EMBL:AY303128 EMBL:AY045919 EMBL:AY079363 EMBL:BT003330 EMBL:X62747 IPI:IPI00519512 PIR:G84787 PIR:S52990 RefSeq:NP_181241.1 UniGene:At.21614 UniGene:At.22705 ProteinModelPortal:P35510 SMR:P35510 IntAct:P35510 STRING:P35510 PRIDE:P35510 EnsemblPlants:AT2G37040.1 GeneID:818280 KEGG:ath:AT2G37040 TAIR:At2g37040 HOGENOM:HBG318028 InParanoid:P35510 KO:K10775 OMA:QRGADFL PhylomeDB:P35510 ProtClustDB:PLN02457 BRENDA:4.3.1.24 Genevestigator:P35510 GermOnline:AT2G37040 GO:GO:0045548 GO:GO:0009819 GO:GO:0046244 InterPro:IPR023144 Gene3D:G3DSA:1.10.274.20 TIGRFAMs:TIGR01226 Uniprot:P35510
Root	Isotig04566	1	18	-4.299	1.41E-05	TAIR|locus:2057981 - symbol:PAL1 "PHE ammonia lyase 1" species:3702 "Arabidopsis thaliana" [GO:0005737 "cytoplasm" evidence=IEA] [GO:0009699 "phenylpropanoid biosynthetic process" evidence=TAS] [GO:0045548 "phenylalanine ammonia-lyase activity" evidence=ISS;IDA;TAS] [GO:0006979 "response to oxidative stress" evidence=IEP] [GO:0080167 "response to karrikin" evidence=IEP] [GO:0009555 "pollen development" evidence=IMP] [GO:0009819 "drought recovery" evidence=IMP] [GO:0010224 "response to UV-B" evidence=IMP] [GO:0046244 "salicylic acid catabolic process" evidence=IMP] [GO:0046274 "lignin catabolic process" evidence=IMP] [GO:0006952 "defense response" evidence=TAS] [GO:0009611 "response to wounding" evidence=TAS] InterPro:IPR001106 InterPro:IPR005922 InterPro:IPR008948 InterPro:IPR022313 Pfam:PF00221 PROSITE:PS00488 GO:GO:0005737 EMBL:CP002685 GenomeReviews:CT485783_GR GO:GO:0006979 GO:GO:0009058 GO:GO:0080167 EMBL:AC006922 GO:GO:0009555 SUPFAM:SSF48557 GO:GO:0006559 InterPro:IPR024083 Gene3D:G3DSA:1.10.275.10 GO:GO:0010224 eggNOG:COG2986 GO:GO:0046274

Tissue	Identification	# of reads (stress)	# of reads (control)	Fold Change	p-value	Description
						EMBL:L33677 EMBL:AY303128 EMBL:AY045919 EMBL:AY079363 EMBL:BT003330 EMBL:X62747 IPI:IPI00519512 PIR:G84787 PIR:S52990 RefSeq:NP_181241.1 UniGene:At.21614 UniGene:At.22705 ProteinModelPortal:P35510 SMR:P35510 IntAct:P35510 STRING:P35510 PRIDE:P35510 EnsemblPlants:AT2G37040.1 GeneID:818280 KEGG:ath:AT2G37040 TAIR:At2g37040 HOGENOM:HBG318028 InParanoid:P35510 KO:K10775 OMA:QRGADFL PhylomeDB:P35510 ProtClustDB:PLN02457 BRENDA:4.3.1.24 Genevestigator:P35510 GermOnline:AT2G37040 GO:GO:0045548 GO:GO:0009819 GO:GO:0046244 InterPro:IPR023144 Gene3D:G3DSA:1.10.274.20 TIGRFAMs:TIGR01226 Uniprot:P35510
Root	Isotig04594	7	28	-2.129	9.37E-05	TAIR|locus:2153919 - symbol:AT5G24610 "AT5G24610" species:3702 "Arabidopsis thaliana" [GO:0003674 "molecular_function" evidence=ND] [GO:0005575 "cellular_component" evidence=ND] [GO:0008150 "biological_process" evidence=ND] EMBL:CP002688 GenomeReviews:BA000015_GR EMBL:AB010068 EMBL:AY084888 EMBL:BT025509 EMBL:AK175922 EMBL:AK176057 EMBL:AK176365 IPI:IPI00528071 RefSeq:NP_197849.1 UniGene:At.30951 STRING:Q9FLU3 PRIDE:Q9FLU3 EnsemblPlants:AT5G24610.1 GeneID:832532 KEGG:ath:AT5G24610 TAIR:At5g24610 eggNOG:NOG287708 HOGENOM:HBG587284 InParanoid:Q9FLU3 OMA:FGNMYRC PhylomeDB:Q9FLU3 ProtClustDB:CLSN2684245 ArrayExpress:Q9FLU3 Genevestigator:Q9FLU3 Uniprot:Q9FLU3
Root	Isotig04595	7	28	-2.129	9.37E-05	TAIR|locus:2153919 - symbol:AT5G24610 "AT5G24610" species:3702 "Arabidopsis thaliana" [GO:0003674 "molecular_function" evidence=ND] [GO:0005575 "cellular_component" evidence=ND] [GO:0008150 "biological_process" evidence=ND] EMBL:CP002688 GenomeReviews:BA000015_GR EMBL:AB010068 EMBL:AY084888 EMBL:BT025509 EMBL:AK175922 EMBL:AK176057 EMBL:AK176365 IPI:IPI00528071 RefSeq:NP_197849.1 UniGene:At.30951 STRING:Q9FLU3 PRIDE:Q9FLU3 EnsemblPlants:AT5G24610.1 GeneID:832532 KEGG:ath:AT5G24610 TAIR:At5g24610 eggNOG:NOG287708 HOGENOM:HBG587284 InParanoid:Q9FLU3 OMA:FGNMYRC PhylomeDB:Q9FLU3 ProtClustDB:CLSN2684245 ArrayExpress:Q9FLU3 Genevestigator:Q9FLU3 Uniprot:Q9FLU3
Root	Isotig04596	7	26	-2.022	0.00027047	TAIR|locus:2114708 - symbol:AT3G49550 "AT3G49550" species:3702 "Arabidopsis thaliana" [GO:0003674 "molecular_function" evidence=ND] [GO:0008150 "biological_process" evidence=ND] EMBL:CP002686 GenomeReviews:BA000014_GR EMBL:AL132964 UniGene:At.71999 eggNOG:NOG287708 HOGENOM:HBG587284 ProtClustDB:CLSN2684245 EMBL:BT010506 EMBL:AK176168 IPI:IPI00533036 PIR:T46232 RefSeq:NP_190524.1 UniGene:At.35592 PRIDE:Q9SCK4 EnsemblPlants:AT3G49550.1 GeneID:824117 KEGG:ath:AT3G49550 TAIR:At3g49550 InParanoid:Q9SCK4 OMA:HSSPFER PhylomeDB:Q9SCK4 Genevestigator:Q9SCK4 Uniprot:Q9SCK4
Root	Isotig04606	67	7	3.130	9.17E-13	SGD|S000028422 - symbol:TAR1 "Mitochondrial protein potentially involved in respiratory metabolism" species:4932 "Saccharomyces cerevisiae" [GO:0003674 "molecular_function" evidence=ND] [GO:0043457 "regulation of cellular respiration" evidence=IGI] [GO:0005739 "mitochondrion" evidence=IEA;IMP] SGD:S000028422 GO:GO:0005739 EMBL:BK006945 EMBL:U53879 GO:GO:0043457 EMBL:Z73326 EMBL:AF479964 RefSeq:NP_690845.1 ProteinModelPortal:Q8TGM6 STRING:Q8TGM6 EnsemblFungi:YLR154W-C GeneID:1466405 KEGG:sce:YLR154W-C CYGD:YLR154w-c eggNOG:NOG79861 OMA:SCSKEHR OrthoDB:EOG466ZX8 NextBio:967146 Genevestigator:Q8TGM6 GermOnline:YLR154W-C Uniprot:Q8TGM6
Root	Isotig04607	67	7	3.130	9.17E-13	SGD|S000028422 - symbol:TAR1 "Mitochondrial protein potentially involved in respiratory metabolism" species:4932 "Saccharomyces cerevisiae" [GO:0003674 "molecular_function" evidence=ND] [GO:0043457 "regulation of cellular respiration" evidence=IGI] [GO:0005739 "mitochondrion" evidence=IEA;IMP] SGD:S000028422 GO:GO:0005739 EMBL:BK006945 EMBL:U53879 GO:GO:0043457 EMBL:Z73326 EMBL:AF479964 RefSeq:NP_690845.1 ProteinModelPortal:Q8TGM6 STRING:Q8TGM6 EnsemblFungi:YLR154W-C GeneID:1466405 KEGG:sce:YLR154W-C CYGD:YLR154w-c eggNOG:NOG79861 OMA:SCSKEHR OrthoDB:EOG466ZX8 NextBio:967146 Genevestigator:Q8TGM6 GermOnline:YLR154W-C Uniprot:Q8TGM6
Root	Isotig04608	66	7	3.108	1.59E-12	SGD|S000028422 - symbol:TAR1 "Mitochondrial protein potentially involved in respiratory metabolism" species:4932 "Saccharomyces cerevisiae" [GO:0003674 "molecular_function" evidence=ND] [GO:0043457 "regulation of cellular respiration" evidence=IGI] [GO:0005739 "mitochondrion" evidence=IEA;IMP] SGD:S000028422 GO:GO:0005739 EMBL:BK006945 EMBL:U53879 GO:GO:0043457 EMBL:Z73326 EMBL:AF479964 RefSeq:NP_690845.1 ProteinModelPortal:Q8TGM6 STRING:Q8TGM6 EnsemblFungi:YLR154W-C GeneID:1466405 KEGG:sce:YLR154W-C CYGD:YLR154w-c eggNOG:NOG79861 OMA:SCSKEHR OrthoDB:EOG466ZX8 NextBio:967146 Genevestigator:Q8TGM6 GermOnline:YLR154W-C Uniprot:Q8TGM6

Tissue	Identification	# of reads (stress)	# of reads (control)	Fold Change	p-value	Description
Root	Isotig04612	9	46	-2.483	3.98E-08	TAIR|locus:2024542 - symbol:AT1G64190 species:3702 "Arabidopsis thaliana" [GO:0003824 "catalytic activity" evidence=IEA] [GO:0004616 "phosphogluconate dehydrogenase (decarboxylating) activity" evidence=IEA;ISS] [GO:0016491 "oxidoreductase activity" evidence=IEA] [GO:0050661 "NADP binding" evidence=IEA] [GO:0050662 "coenzyme binding" evidence=IEA] [GO:0016020 "membrane" evidence=IDA] [GO:0009651 "response to salt stress" evidence=IEP] [GO:0009507 "chloroplast" evidence=IDA] [GO:0009570 "chloroplast stroma" evidence=IDA] [GO:0005829 "cytosol" evidence=IDA] InterPro:IPR006113 InterPro:IPR006114 InterPro:IPR006115 InterPro:IPR008927 InterPro:IPR012284 InterPro:IPR013328 InterPro:IPR016040 Pfam:PF00393 Pfam:PF03446 PIRSF:PIRSF000109 EMBL:CP002684 GenomeReviews:CT485782_GR GO:GO:0005829 GO:GO:0009570 GO:GO:0016020 Gene3D:G3DSA:3.40.50.720 GO:GO:0004616 GO:GO:0006098 Gene3D:G3DSA:1.10.1040.10 SUPFAM:SSF48179 KO:K00033 HOGENOM:HBG286913 GO:GO:0050661 Gene3D:G3DSA:1.20.5.320 TIGRFAMs:TIGR00873 HSSP:P00349 GO:GO:0009651 EMBL:AC007764 UniGene:At.43563 UniGene:At.70356 ProtClustDB:PLN02350 EMBL:BT004568 EMBL:AK227596 IPI:IPI00516481 RefSeq:NP_176601.1 ProteinModelPortal:Q9SH69 SMR:Q9SH69 STRING:Q9SH69 PRIDE:Q9SH69 EnsemblPlants:AT1G64190.1 GeneID:842724 KEGG:ath:AT1G64190 TAIR:At1g64190 InParanoid:Q9SH69 OMA:NTERRIS PhylomeDB:Q9SH69 Genevestigator:Q9SH69 Uniprot:Q9SH69
Root	Isotig04613	8	44	-2.588	3.84E-08	TAIR|locus:2024542 - symbol:AT1G64190 species:3702 "Arabidopsis thaliana" [GO:0003824 "catalytic activity" evidence=IEA] [GO:0004616 "phosphogluconate dehydrogenase (decarboxylating) activity" evidence=IEA;ISS] [GO:0016491 "oxidoreductase activity" evidence=IEA] [GO:0050661 "NADP binding" evidence=IEA] [GO:0050662 "coenzyme binding" evidence=IEA] [GO:0016020 "membrane" evidence=IDA] [GO:0009651 "response to salt stress" evidence=IEP] [GO:0009507 "chloroplast" evidence=IDA] [GO:0009570 "chloroplast stroma" evidence=IDA] [GO:0005829 "cytosol" evidence=IDA] InterPro:IPR006113 InterPro:IPR006114 InterPro:IPR006115 InterPro:IPR008927 InterPro:IPR012284 InterPro:IPR013328 InterPro:IPR016040 Pfam:PF00393 Pfam:PF03446 PIRSF:PIRSF000109 EMBL:CP002684 GenomeReviews:CT485782_GR GO:GO:0005829 GO:GO:0009570 GO:GO:0016020 Gene3D:G3DSA:3.40.50.720 GO:GO:0004616 GO:GO:0006098 Gene3D:G3DSA:1.10.1040.10 SUPFAM:SSF48179 KO:K00033 HOGENOM:HBG286913 GO:GO:0050661 Gene3D:G3DSA:1.20.5.320 TIGRFAMs:TIGR00873 HSSP:P00349 GO:GO:0009651 EMBL:AC007764 UniGene:At.43563 UniGene:At.70356 ProtClustDB:PLN02350 EMBL:BT004568 EMBL:AK227596 IPI:IPI00516481 RefSeq:NP_176601.1 ProteinModelPortal:Q9SH69 SMR:Q9SH69 STRING:Q9SH69 PRIDE:Q9SH69 EnsemblPlants:AT1G64190.1 GeneID:842724 KEGG:ath:AT1G64190 TAIR:At1g64190 InParanoid:Q9SH69 OMA:NTERRIS PhylomeDB:Q9SH69 Genevestigator:Q9SH69 Uniprot:Q9SH69
Root	Isotig04614	3	37	-3.753	2.31E-09	TAIR|locus:2024542 - symbol:AT1G64190 species:3702 "Arabidopsis thaliana" [GO:0003824 "catalytic activity" evidence=IEA] [GO:0004616 "phosphogluconate dehydrogenase (decarboxylating) activity" evidence=IEA;ISS] [GO:0016491 "oxidoreductase activity" evidence=IEA] [GO:0050661 "NADP binding" evidence=IEA] [GO:0050662 "coenzyme binding" evidence=IEA] [GO:0016020 "membrane" evidence=IDA] [GO:0009651 "response to salt stress" evidence=IEP] [GO:0009507 "chloroplast" evidence=IDA] [GO:0009570 "chloroplast stroma" evidence=IDA] [GO:0005829 "cytosol" evidence=IDA] InterPro:IPR006113 InterPro:IPR006114 InterPro:IPR006115 InterPro:IPR008927 InterPro:IPR012284 InterPro:IPR013328 InterPro:IPR016040 Pfam:PF00393 Pfam:PF03446 PIRSF:PIRSF000109 EMBL:CP002684 GenomeReviews:CT485782_GR GO:GO:0005829 GO:GO:0009570 GO:GO:0016020 Gene3D:G3DSA:3.40.50.720 GO:GO:0004616 GO:GO:0006098 Gene3D:G3DSA:1.10.1040.10 SUPFAM:SSF48179 KO:K00033 HOGENOM:HBG286913 GO:GO:0050661 Gene3D:G3DSA:1.20.5.320 TIGRFAMs:TIGR00873 HSSP:P00349 GO:GO:0009651 EMBL:AC007764 UniGene:At.43563 UniGene:At.70356 ProtClustDB:PLN02350 EMBL:BT004568 EMBL:AK227596 IPI:IPI00516481 RefSeq:NP_176601.1 ProteinModelPortal:Q9SH69 SMR:Q9SH69 STRING:Q9SH69 PRIDE:Q9SH69 EnsemblPlants:AT1G64190.1 GeneID:842724 KEGG:ath:AT1G64190 TAIR:At1g64190 InParanoid:Q9SH69 OMA:NTERRIS PhylomeDB:Q9SH69 Genevestigator:Q9SH69 Uniprot:Q9SH69
Root	Isotig04621	102	0	7.543	4.82E-23	TAIR|locus:2010341 - symbol:ERF1-2 "AT1G12920" species:3702 "Arabidopsis thaliana" [GO:0003747 "translation release factor activity" evidence=IGI;ISS] [GO:0006415 "translational termination" evidence=IGI;ISS] [GO:0018444 "translation release factor complex" evidence=TAS] [GO:0005886 "plasma membrane" evidence=IDA] [GO:0005829 "cytosol" evidence=IDA] InterPro:IPR004403 InterPro:IPR005140 InterPro:IPR005141 InterPro:IPR005142 Pfam:PF03465 Pfam:PF03463 Pfam:PF03464 EMBL:CP002684 GenomeReviews:CT485782_GR GO:GO:0005829 GO:GO:0005886 EMBL:AC012187 eggNOG:COG1503 HOGENOM:HBG497188 KO:K03265 ProtClustDB:CLSN2682479 GO:GO:0016149 InterPro:IPR024049 Gene3D:G3DSA:3.30.960.10

Tissue	Identification	# of reads (stress)	# of reads (control)	Fold Change	p-value	Description
						PANTHER:PTHR10113 SUPFAM:SSF55481 TIGRFAMs:TIGR03676 EMBL:AY034963 EMBL:AY086623 EMBL:X69374 EMBL:U40218 EMBL:Z18188 IPI:IPI00537037 PIR:H86262 PIR:S31445 RefSeq:NP_172752.1 UniGene:At.23958 ProteinModelPortal:Q9LPV8 SMR:Q9LPV8 STRING:Q9LPV8 PRIDE:Q9LPV8 EnsemblPlants:AT1G12920.1 GeneID:837850 KEGG:ath:AT1G12920 TAIR:At1g12920 InParanoid:Q9LPV8 OMA:HETDRNI PhylomeDB:Q9LPV8 ArrayExpress:Q9LPV8 Genevestigator:Q9LPV8 GermOnline:AT1G12920 Uniprot:Q9LPV8
Root	Isotig04622	101	0	7.529	7.39E-23	TAIR|locus:2010341 - symbol:ERF1-2 "AT1G12920" species:3702 "Arabidopsis thaliana" [GO:0003747 "translation release factor activity" evidence=IGI;ISS] [GO:0006415 "translational termination" evidence=IGI;ISS] [GO:0018444 "translation release factor complex" evidence=TAS] [GO:0005886 "plasma membrane" evidence=IDA] [GO:0005829 "cytosol" evidence=IDA] InterPro:IPR004403 InterPro:IPR005140 InterPro:IPR005141 InterPro:IPR005142 Pfam:PF03465 Pfam:PF03463 Pfam:PF03464 EMBL:CP002684 GenomeReviews:CT485782_GR GO:GO:0005829 GO:GO:0005886 EMBL:AC012187 eggNOG:COG1503 HOGENOM:HBG497188 KO:K03265 ProtClustDB:CLSN2682479 GO:GO:0016149 InterPro:IPR024049 Gene3D:G3DSA:3.30.960.10 PANTHER:PTHR10113 SUPFAM:SSF55481 TIGRFAMs:TIGR03676 EMBL:AY034963 EMBL:AY086623 EMBL:X69374 EMBL:U40218 EMBL:Z18188 IPI:IPI00537037 PIR:H86262 PIR:S31445 RefSeq:NP_172752.1 UniGene:At.23958 ProteinModelPortal:Q9LPV8 SMR:Q9LPV8 STRING:Q9LPV8 PRIDE:Q9LPV8 EnsemblPlants:AT1G12920.1 GeneID:837850 KEGG:ath:AT1G12920 TAIR:At1g12920 InParanoid:Q9LPV8 OMA:HETDRNI PhylomeDB:Q9LPV8 ArrayExpress:Q9LPV8 Genevestigator:Q9LPV8 GermOnline:AT1G12920 Uniprot:Q9LPV8
Root	Isotig04623	50	0	6.515	9.09E-13	TAIR|locus:2010341 - symbol:ERF1-2 "AT1G12920" species:3702 "Arabidopsis thaliana" [GO:0003747 "translation release factor activity" evidence=IGI;ISS] [GO:0006415 "translational termination" evidence=IGI;ISS] [GO:0018444 "translation release factor complex" evidence=TAS] [GO:0005886 "plasma membrane" evidence=IDA] [GO:0005829 "cytosol" evidence=IDA] InterPro:IPR004403 InterPro:IPR005140 InterPro:IPR005141 InterPro:IPR005142 Pfam:PF03465 Pfam:PF03463 Pfam:PF03464 EMBL:CP002684 GenomeReviews:CT485782_GR GO:GO:0005829 GO:GO:0005886 EMBL:AC012187 eggNOG:COG1503 HOGENOM:HBG497188 KO:K03265 ProtClustDB:CLSN2682479 GO:GO:0016149 InterPro:IPR024049 Gene3D:G3DSA:3.30.960.10 PANTHER:PTHR10113 SUPFAM:SSF55481 TIGRFAMs:TIGR03676 EMBL:AY034963 EMBL:AY086623 EMBL:X69374 EMBL:U40218 EMBL:Z18188 IPI:IPI00537037 PIR:H86262 PIR:S31445 RefSeq:NP_172752.1 UniGene:At.23958 ProteinModelPortal:Q9LPV8 SMR:Q9LPV8 STRING:Q9LPV8 PRIDE:Q9LPV8 EnsemblPlants:AT1G12920.1 GeneID:837850 KEGG:ath:AT1G12920 TAIR:At1g12920 InParanoid:Q9LPV8 OMA:HETDRNI PhylomeDB:Q9LPV8 ArrayExpress:Q9LPV8 Genevestigator:Q9LPV8 GermOnline:AT1G12920 Uniprot:Q9LPV8
Root	Isotig04659	28	93	-1.861	7.09E-11	TAIR|locus:3439219 - symbol:TUA6 "AT4G14960" species:3702 "Arabidopsis thaliana" [GO:0005874 "microtubule" evidence=IDA] [GO:0045298 "tubulin complex" evidence=IDA] [GO:0005773 "vacuole" evidence=IDA] [GO:0005200 "structural constituent of cytoskeleton" evidence=ISS] [GO:0005730 "nucleolus" evidence=IDA] [GO:0000226 "microtubule cytoskeleton organization" evidence=IMP] [GO:0005618 "cell wall" evidence=IDA] [GO:0016020 "membrane" evidence=IDA] [GO:0005886 "plasma membrane" evidence=IDA] [GO:0009651 "response to salt stress" evidence=IEP] [GO:0005829 "cytosol" evidence=IDA] [GO:0005515 "protein binding" evidence=IPI] [GO:0071258 "cellular response to gravity" evidence=IMP] [GO:0009506 "plasmodesma" evidence=IDA] InterPro:IPR000217 InterPro:IPR002452 InterPro:IPR003008 InterPro:IPR008280 InterPro:IPR017975 InterPro:IPR018316 Pfam:PF00091 Pfam:PF03953 PRINTS:PR01161 PRINTS:PR01162 PROSITE:PS00227 SMART:SM00864 SMART:SM00865 GO:GO:0005525 GO:GO:0005829 GO:GO:0005886 GO:GO:0009506 EMBL:CP002687 GenomeReviews:CT486007_GR GO:GO:0005773 GO:GO:0005618 GO:GO:0005515 GO:GO:0005730 GO:GO:0005198 GO:GO:0003924 GO:GO:0009651 EMBL:AL161540 GO:GO:0000226 GO:GO:0005874 GO:GO:0051258 Gene3D:G3DSA:3.40.50.1440 SUPFAM:SSF52490 GO:GO:0007018 EMBL:Z97337 Gene3D:G3DSA:3.30.1330.20 SUPFAM:SSF55307 GO:GO:0045298 eggNOG:COG5023 GO:GO:0071258 HOGENOM:HBG750007 KO:K07374 InterPro:IPR023123 Gene3D:G3DSA:1.10.287.600 PANTHER:PTHR11588 ProtClustDB:PLN00221 UniGene:At.22368 UniGene:At.48153 EMBL:M84699 EMBL:AY058858 EMBL:AY065347 EMBL:AY079036 EMBL:AY117174 IPI:IPI00520226 PIR:JQ1597 RefSeq:NP_193232.1 ProteinModelPortal:P29511 SMR:P29511 IntAct:P29511 STRING:P29511 PRIDE:P29511 EnsemblPlants:AT4G14960.2 GeneID:827154 KEGG:ath:AT4G14960 TAIR:At4g14960 InParanoid:P29511 OMA:MPSDKAI PhylomeDB:P29511 ArrayExpress:P29511 Genevestigator:P29511 Uniprot:P29511

Tissue	Identification	# of reads (stress)	# of reads (control)	Fold Change	p-value	Description
Root	Isotig04666	13	37	-1.638	0.000168748	UNIPROTKB|Q84N28 - symbol:OMT1 "Flavone O-methyltransferase 1" species:4565 "Triticum aestivum" [GO:0009611 "response to wounding" evidence=IDA] [GO:0009723 "response to ethylene stimulus" evidence=IDA] [GO:0009751 "response to salicylic acid stimulus" evidence=IDA] [GO:0042542 "response to hydrogen peroxide" evidence=IDA] InterPro:IPR001077 InterPro:IPR012967 InterPro:IPR016461 Pfam:PF00891 Pfam:PF08100 PIRSF:PIRSF005739 GO:GO:0009611 InterPro:IPR011991 Gene3D:G3DSA:1.10.10.10 GO:GO:0009723 GO:GO:0009751 GO:GO:0046983 GO:GO:0042542 GO:GO:0008171 GO:GO:0009813 EMBL:AY226581 UniGene:Ta.336 HSSP:P28002 ProteinModelPortal:Q84N28 SMR:Q84N28 Gramene:Q84N28 Uniprot:Q84N28
Root	Isotig04678	4	19	-2.377	0.000578515	TAIR|locus:2134603 - symbol:CRK41 "cysteine-rich RLK (RECEPTOR-like protein kinase) 41" species:3702 "Arabidopsis thaliana" [GO:0004672 "protein kinase activity" evidence=IEA;ISS] [GO:0004674 "protein serine/threonine kinase activity" evidence=IEA] [GO:0005524 "ATP binding" evidence=IEA] [GO:0006468 "protein phosphorylation" evidence=IEA] InterPro:IPR000719 InterPro:IPR001245 InterPro:IPR008271 InterPro:IPR011009 InterPro:IPR017441 Pfam:PF07714 PROSITE:PS00107 PROSITE:PS00108 PROSITE:PS50011 GO:GO:0016021 GO:GO:0005524 EMBL:CP002687 GenomeReviews:CT486007_GR eggNOG:COG0515 GO:GO:0004872 SUPFAM:SSF56112 GO:GO:0004674 HOGENOM:HBG755340 EMBL:AF013294 EMBL:AL161491 InterPro:IPR002902 Pfam:PF01657 PROSITE:PS51473 ProtClustDB:CLSN2685554 IPI:IPI00521692 PIR:T01550 RefSeq:NP_567204.3 UniGene:At.34491 ProteinModelPortal:O23081 SMR:O23081 PRIDE:O23081 EnsemblPlants:AT4G00970.1 GeneID:827936 KEGG:ath:AT4G00970 GeneFarm:2566 TAIR:At4g00970 InParanoid:O23081 OMA:VDINSAI PhylomeDB:O23081 ArrayExpress:O23081 Genevestigator:O23081 Uniprot:O23081
Root	Isotig04693	0	43	-6.555	4.45E-12	TAIR|locus:2087368 - symbol:scpl40 "serine carboxypeptidase-like 40" species:3702 "Arabidopsis thaliana" [GO:0004185 "serine-type carboxypeptidase activity" evidence=IEA;ISS] [GO:0006508 "proteolysis" evidence=IEA;ISS] InterPro:IPR001563 InterPro:IPR018202 Pfam:PF00450 PRINTS:PR00724 PROSITE:PS00131 PROSITE:PS00560 EMBL:CP002686 GenomeReviews:BA000014_GR GO:GO:0005576 GO:GO:0006508 EMBL:AL163818 GO:GO:0004185 eggNOG:COG2939 PANTHER:PTHR11802 HOGENOM:HBG588032 EMBL:AY087589 EMBL:AK228170 EMBL:BT003126 IPI:IPI00536894 PIR:T49188 RefSeq:NP_191906.1 UniGene:At.33917 UniGene:At.71216 ProteinModelPortal:Q0WRX3 SMR:Q0WRX3 MEROPS:S10.A41 PRIDE:Q0WRX3 EnsemblPlants:AT3G63470.1 GeneID:825522 KEGG:ath:AT3G63470 TAIR:At3g63470 InParanoid:Q0WRX3 OMA:WNNAANV PhylomeDB:Q0WRX3 ProtClustDB:CLSN2914723 Genevestigator:Q0WRX3 Uniprot:Q0WRX3
Root	Isotig04694	0	40	-6.451	2.24E-11	TAIR|locus:2087368 - symbol:scpl40 "serine carboxypeptidase-like 40" species:3702 "Arabidopsis thaliana" [GO:0004185 "serine-type carboxypeptidase activity" evidence=IEA;ISS] [GO:0006508 "proteolysis" evidence=IEA;ISS] InterPro:IPR001563 InterPro:IPR018202 Pfam:PF00450 PRINTS:PR00724 PROSITE:PS00131 PROSITE:PS00560 EMBL:CP002686 GenomeReviews:BA000014_GR GO:GO:0005576 GO:GO:0006508 EMBL:AL163818 GO:GO:0004185 eggNOG:COG2939 PANTHER:PTHR11802 HOGENOM:HBG588032 EMBL:AY087589 EMBL:AK228170 EMBL:BT003126 IPI:IPI00536894 PIR:T49188 RefSeq:NP_191906.1 UniGene:At.33917 UniGene:At.71216 ProteinModelPortal:Q0WRX3 SMR:Q0WRX3 MEROPS:S10.A41 PRIDE:Q0WRX3 EnsemblPlants:AT3G63470.1 GeneID:825522 KEGG:ath:AT3G63470 TAIR:At3g63470 InParanoid:Q0WRX3 OMA:WNNAANV PhylomeDB:Q0WRX3 ProtClustDB:CLSN2914723 Genevestigator:Q0WRX3 Uniprot:Q0WRX3
Root	Isotig04695	0	41	-6.487	1.30E-11	TAIR|locus:2087368 - symbol:scpl40 "serine carboxypeptidase-like 40" species:3702 "Arabidopsis thaliana" [GO:0004185 "serine-type carboxypeptidase activity" evidence=IEA;ISS] [GO:0006508 "proteolysis" evidence=IEA;ISS] InterPro:IPR001563 InterPro:IPR018202 Pfam:PF00450 PRINTS:PR00724 PROSITE:PS00131 PROSITE:PS00560 EMBL:CP002686 GenomeReviews:BA000014_GR GO:GO:0005576 GO:GO:0006508 EMBL:AL163818 GO:GO:0004185 eggNOG:COG2939 PANTHER:PTHR11802 HOGENOM:HBG588032 EMBL:AY087589 EMBL:AK228170 EMBL:BT003126 IPI:IPI00536894 PIR:T49188 RefSeq:NP_191906.1 UniGene:At.33917 UniGene:At.71216 ProteinModelPortal:Q0WRX3 SMR:Q0WRX3 MEROPS:S10.A41 PRIDE:Q0WRX3 EnsemblPlants:AT3G63470.1 GeneID:825522 KEGG:ath:AT3G63470 TAIR:At3g63470 InParanoid:Q0WRX3 OMA:WNNAANV PhylomeDB:Q0WRX3 ProtClustDB:CLSN2914723 Genevestigator:Q0WRX3 Uniprot:Q0WRX3
Root	Isotig04699	42	14	1.456	0.000438168	TAIR|locus:2142120 - symbol:IIL1 "AT4G13430" species:3702 "Arabidopsis thaliana" [GO:0008152 "metabolic process" evidence=ISS] [GO:0016829 "lyase activity" evidence=ISS] [GO:0046686 "response to cadmium ion" evidence=IEP] [GO:0009507 "chloroplast" evidence=IDA] [GO:0009570 "chloroplast stroma" evidence=IDA]

Tissue	Identification	# of reads (stress)	# of reads (control)	Fold Change	p-value	Description
						[GO:0019761 "glucosinolate biosynthetic process" evidence=IMP] [GO:0050486 "intramolecular transferase activity, transferring hydroxy groups" evidence=IMP] InterPro:IPR001030 InterPro:IPR006251 InterPro:IPR015931 InterPro:IPR015932 InterPro:IPR015937 Pfam:PF00330 PRINTS:PR00415 EMBL:CP002687 GenomeReviews:CT486007_GR GO:GO:0009570 GO:GO:0046686 GO:GO:0046872 GO:GO:0019761 GO:GO:0051539 Gene3D:G3DSA:3.30.499.10 Gene3D:G3DSA:3.40.1060.10 PANTHER:PTHR11670 SUPFAM:SSF53732 EMBL:AL161536 GO:GO:0009098 EMBL:AL049608 GO:GO:0003861 PANTHER:PTHR11670:SF16 TIGRFAMs:TIGR01343 EMBL:AY045842 EMBL:AY117151 IPI:IPI00543884 PIR:T06300 RefSeq:NP_567405.1 UniGene:At.24538 UniGene:At.67680 ProteinModelPortal:Q94AR8 SMR:Q94AR8 STRING:Q94AR8 PRIDE:Q94AR8 EnsemblPlants:AT4G13430.1 GeneID:826975 KEGG:ath:AT4G13430 TAIR:At4g13430 eggNOG:COG0065 HOGENOM:HBG330745 InParanoid:Q94AR8 KO:K01703 OMA:FTMANMA PhylomeDB:Q94AR8 ProtClustDB:CLSN2689413 BioCyc:ARA:AT4G13430-MONOMER BioCyc:MetaCyc:AT4G13430-MONOMER Genevestigator:Q94AR8 GO:GO:0050486 Uniprot:Q94AR8
Root	Isotig04701	41	13	1.528	0.000317378	TAIR|locus:2142120 - symbol:IIL1 "AT4G13430" species:3702 "Arabidopsis thaliana" [GO:0008152 "metabolic process" evidence=ISS] [GO:0016829 "lyase activity" evidence=ISS] [GO:0046686 "response to cadmium ion" evidence=IEP] [GO:0009507 "chloroplast" evidence=IDA] [GO:0009570 "chloroplast stroma" evidence=IDA] [GO:0019761 "glucosinolate biosynthetic process" evidence=IMP] [GO:0050486 "intramolecular transferase activity, transferring hydroxy groups" evidence=IMP] InterPro:IPR001030 InterPro:IPR006251 InterPro:IPR015931 InterPro:IPR015932 InterPro:IPR015937 Pfam:PF00330 PRINTS:PR00415 EMBL:CP002687 GenomeReviews:CT486007_GR GO:GO:0009570 GO:GO:0046686 GO:GO:0046872 GO:GO:0019761 GO:GO:0051539 Gene3D:G3DSA:3.30.499.10 Gene3D:G3DSA:3.40.1060.10 PANTHER:PTHR11670 SUPFAM:SSF53732 EMBL:AL161536 GO:GO:0009098 EMBL:AL049608 GO:GO:0003861 PANTHER:PTHR11670:SF16 TIGRFAMs:TIGR01343 EMBL:AY045842 EMBL:AY117151 IPI:IPI00543884 PIR:T06300 RefSeq:NP_567405.1 UniGene:At.24538 UniGene:At.67680 ProteinModelPortal:Q94AR8 SMR:Q94AR8 STRING:Q94AR8 PRIDE:Q94AR8 EnsemblPlants:AT4G13430.1 GeneID:826975 KEGG:ath:AT4G13430 TAIR:At4g13430 eggNOG:COG0065 HOGENOM:HBG330745 InParanoid:Q94AR8 KO:K01703 OMA:FTMANMA PhylomeDB:Q94AR8 ProtClustDB:CLSN2689413 BioCyc:ARA:AT4G13430-MONOMER BioCyc:MetaCyc:AT4G13430-MONOMER Genevestigator:Q94AR8 GO:GO:0050486 Uniprot:Q94AR8
Root	Isotig04705	8	45	-2.621	2.17E-08	TAIR|locus:2081988 - symbol:AT3G55840 "AT3G55840" species:3702 "Arabidopsis thaliana" [GO:0005575 "cellular_component" evidence=ND] [GO:0008150 "biological_process" evidence=ND] [GO:0005515 "protein binding" evidence=IPI] GO:GO:0005737 EMBL:CP002686 GO:GO:0005515 GO:GO:0006952 EMBL:AL163832 EMBL:DQ132634 EMBL:AY034950 EMBL:AY070026 EMBL:AY085394 IPI:IPI00532415 PIR:T49196 RefSeq:NP_191143.1 UniGene:At.19788 UniGene:At.70251 ProteinModelPortal:Q9LY61 IntAct:Q9LY61 PRIDE:Q9LY61 GeneID:824750 KEGG:ath:AT3G55840 TAIR:At3g55840 eggNOG:NOG286983 InParanoid:Q9LY61 OMA:EANANRT PhylomeDB:Q9LY61 ProtClustDB:CLSN2683387 ArrayExpress:Q9LY61 Genevestigator:Q9LY61 InterPro:IPR009743 InterPro:IPR009869 Pfam:PF07014 Pfam:PF07231 Uniprot:Q9LY61
Root	Isotig04706	6	41	-2.902	1.75E-08	TAIR|locus:2081988 - symbol:AT3G55840 "AT3G55840" species:3702 "Arabidopsis thaliana" [GO:0005575 "cellular_component" evidence=ND] [GO:0008150 "biological_process" evidence=ND] [GO:0005515 "protein binding" evidence=IPI] GO:GO:0005737 EMBL:CP002686 GO:GO:0005515 GO:GO:0006952 EMBL:AL163832 EMBL:DQ132634 EMBL:AY034950 EMBL:AY070026 EMBL:AY085394 IPI:IPI00532415 PIR:T49196 RefSeq:NP_191143.1 UniGene:At.19788 UniGene:At.70251 ProteinModelPortal:Q9LY61 IntAct:Q9LY61 PRIDE:Q9LY61 GeneID:824750 KEGG:ath:AT3G55840 TAIR:At3g55840 eggNOG:NOG286983 InParanoid:Q9LY61 OMA:EANANRT PhylomeDB:Q9LY61 ProtClustDB:CLSN2683387 ArrayExpress:Q9LY61 Genevestigator:Q9LY61 InterPro:IPR009743 InterPro:IPR009869 Pfam:PF07014 Pfam:PF07231 Uniprot:Q9LY61
Root	Isotig04707	6	32	-2.544	3.42E-06	TAIR|locus:2081988 - symbol:AT3G55840 "AT3G55840" species:3702 "Arabidopsis thaliana" [GO:0005575 "cellular_component" evidence=ND] [GO:0008150 "biological_process" evidence=ND] [GO:0005515 "protein binding" evidence=IPI] GO:GO:0005737 EMBL:CP002686 GO:GO:0005515 GO:GO:0006952 EMBL:AL163832 EMBL:DQ132634 EMBL:AY034950 EMBL:AY070026 EMBL:AY085394 IPI:IPI00532415 PIR:T49196 RefSeq:NP_191143.1 UniGene:At.19788 UniGene:At.70251 ProteinModelPortal:Q9LY61 IntAct:Q9LY61 PRIDE:Q9LY61 GeneID:824750 KEGG:ath:AT3G55840 TAIR:At3g55840 eggNOG:NOG286983 InParanoid:Q9LY61 OMA:EANANRT PhylomeDB:Q9LY61 ProtClustDB:CLSN2683387 ArrayExpress:Q9LY61 Genevestigator:Q9LY61 InterPro:IPR009743 InterPro:IPR009869 Pfam:PF07014 Pfam:PF07231 Uniprot:Q9LY61

Tissue	Identification	# of reads (stress)	# of reads (control)	Fold Change	p-value	Description
Root	Isotig04714	85	36	1.110	5.11E-05	ZFIN|ZDB-GENE-070410-9 - symbol:zgc:158463 "zgc:158463" species:7955 "Danio rerio" [GO:0003674 "molecular_function" evidence=ND] [GO:0005575 "cellular_component" evidence=ND] [GO:0008150 "biological_process" evidence=ND] ZFIN:ZDB-GENE-070410-9 EMBL:BX296557 EMBL:BX537263 EMBL:BC129240 IPI:IPI00837201 RefSeq:NP_001091866.1 RefSeq:XP_003200836.1 UniGene:Dr.155369 Ensembl:ENSDART00000102863 Ensembl:ENSDART00000126982 GeneID:100037361 GeneID:100151188 KEGG:dre:100037361 KEGG:dre:100151188 eggNOG:NOG298153 GeneTree:ENSGT00390000016898 InParanoid:A2VD29 OMA:ACHQHAL OrthoDB:EOG4FJ8BF Bgee:A2VD29 Uniprot:A2VD29
Root	Isotig04715	85	36	1.110	5.11E-05	ZFIN|ZDB-GENE-070410-9 - symbol:zgc:158463 "zgc:158463" species:7955 "Danio rerio" [GO:0003674 "molecular_function" evidence=ND] [GO:0005575 "cellular_component" evidence=ND] [GO:0008150 "biological_process" evidence=ND] ZFIN:ZDB-GENE-070410-9 EMBL:BX296557 EMBL:BX537263 EMBL:BC129240 IPI:IPI00837201 RefSeq:NP_001091866.1 RefSeq:XP_003200836.1 UniGene:Dr.155369 Ensembl:ENSDART00000102863 Ensembl:ENSDART00000126982 GeneID:100037361 GeneID:100151188 KEGG:dre:100037361 KEGG:dre:100151188 eggNOG:NOG298153 GeneTree:ENSGT00390000016898 InParanoid:A2VD29 OMA:ACHQHAL OrthoDB:EOG4FJ8BF Bgee:A2VD29 Uniprot:A2VD29
Root	Isotig04716	82	32	1.229	1.62E-05	ZFIN|ZDB-GENE-070410-9 - symbol:zgc:158463 "zgc:158463" species:7955 "Danio rerio" [GO:0003674 "molecular_function" evidence=ND] [GO:0005575 "cellular_component" evidence=ND] [GO:0008150 "biological_process" evidence=ND] ZFIN:ZDB-GENE-070410-9 EMBL:BX296557 EMBL:BX537263 EMBL:BC129240 IPI:IPI00837201 RefSeq:NP_001091866.1 RefSeq:XP_003200836.1 UniGene:Dr.155369 Ensembl:ENSDART00000102863 Ensembl:ENSDART00000126982 GeneID:100037361 GeneID:100151188 KEGG:dre:100037361 KEGG:dre:100151188 eggNOG:NOG298153 GeneTree:ENSGT00390000016898 InParanoid:A2VD29 OMA:ACHQHAL OrthoDB:EOG4FJ8BF Bgee:A2VD29 Uniprot:A2VD29
Root	Isotig04720	0	22	-5.588	6.01E-07	TAIR|locus:2087368 - symbol:scpl40 "serine carboxypeptidase-like 40" species:3702 "Arabidopsis thaliana" [GO:0004185 "serine-type carboxypeptidase activity" evidence=IEA;ISS] [GO:0006508 "proteolysis" evidence=IEA;ISS] InterPro:IPR001563 InterPro:IPR018202 Pfam:PF00450 PRINTS:PR00724 PROSITE:PS00131 PROSITE:PS00560 EMBL:CP002686 GenomeReviews:BA000014_GR GO:GO:0005576 GO:GO:0006508 EMBL:AL163818 GO:GO:0004185 eggNOG:COG2939 PANTHER:PTHR11802 HOGENOM:HBG588032 EMBL:AY087589 EMBL:AK228170 EMBL:BT003126 IPI:IPI00536894 PIR:T49188 RefSeq:NP_191906.1 UniGene:At.33917 UniGene:At.71216 ProteinModelPortal:Q0WRX3 SMR:Q0WRX3 MEROPS:S10.A41 PRIDE:Q0WRX3 EnsemblPlants:AT3G63470.1 GeneID:825522 KEGG:ath:AT3G63470 TAIR:At3g63470 InParanoid:Q0WRX3 OMA:WNNAANV PhylomeDB:Q0WRX3 ProtClustDB:CLSN2914723 Genevestigator:Q0WRX3 Uniprot:Q0WRX3
Root	Isotig04721	0	21	-5.521	1.09E-06	TAIR|locus:2087368 - symbol:scpl40 "serine carboxypeptidase-like 40" species:3702 "Arabidopsis thaliana" [GO:0004185 "serine-type carboxypeptidase activity" evidence=IEA;ISS] [GO:0006508 "proteolysis" evidence=IEA;ISS] InterPro:IPR001563 InterPro:IPR018202 Pfam:PF00450 PRINTS:PR00724 PROSITE:PS00131 PROSITE:PS00560 EMBL:CP002686 GenomeReviews:BA000014_GR GO:GO:0005576 GO:GO:0006508 EMBL:AL163818 GO:GO:0004185 eggNOG:COG2939 PANTHER:PTHR11802 HOGENOM:HBG588032 EMBL:AY087589 EMBL:AK228170 EMBL:BT003126 IPI:IPI00536894 PIR:T49188 RefSeq:NP_191906.1 UniGene:At.33917 UniGene:At.71216 ProteinModelPortal:Q0WRX3 SMR:Q0WRX3 MEROPS:S10.A41 PRIDE:Q0WRX3 EnsemblPlants:AT3G63470.1 GeneID:825522 KEGG:ath:AT3G63470 TAIR:At3g63470 InParanoid:Q0WRX3 OMA:WNNAANV PhylomeDB:Q0WRX3 ProtClustDB:CLSN2914723 Genevestigator:Q0WRX3 Uniprot:Q0WRX3
Root	Isotig04722	0	22	-5.588	6.01E-07	TAIR|locus:2087368 - symbol:scpl40 "serine carboxypeptidase-like 40" species:3702 "Arabidopsis thaliana" [GO:0004185 "serine-type carboxypeptidase activity" evidence=IEA;ISS] [GO:0006508 "proteolysis" evidence=IEA;ISS] InterPro:IPR001563 InterPro:IPR018202 Pfam:PF00450 PRINTS:PR00724 PROSITE:PS00131 PROSITE:PS00560 EMBL:CP002686 GenomeReviews:BA000014_GR GO:GO:0005576 GO:GO:0006508 EMBL:AL163818 GO:GO:0004185 eggNOG:COG2939 PANTHER:PTHR11802 HOGENOM:HBG588032 EMBL:AY087589 EMBL:AK228170 EMBL:BT003126 IPI:IPI00536894 PIR:T49188 RefSeq:NP_191906.1 UniGene:At.33917 UniGene:At.71216 ProteinModelPortal:Q0WRX3 SMR:Q0WRX3 MEROPS:S10.A41 PRIDE:Q0WRX3 EnsemblPlants:AT3G63470.1 GeneID:825522 KEGG:ath:AT3G63470 TAIR:At3g63470 InParanoid:Q0WRX3 OMA:WNNAANV PhylomeDB:Q0WRX3 ProtClustDB:CLSN2914723 Genevestigator:Q0WRX3 Uniprot:Q0WRX3

Tissue	Identification	# of reads (stress)	# of reads (control)	Fold Change	p-value	Description
Root	Isotig04723	10	57	-2.640	2.52E-10	TAIR|locus:2181665 - symbol:AT5G13420 species:3702 "Arabidopsis thaliana" [GO:0003824 "catalytic activity" evidence=IEA] [GO:0004801 "sedoheptulose-7-phosphate:D-glyceraldehyde-3-phosphate glyceronetransferase activity" evidence=IEA;ISS] [GO:0005739 "mitochondrion" evidence=IDA] [GO:0009507 "chloroplast" evidence=IDA] [GO:0009570 "chloroplast stroma" evidence=IDA] [GO:0046686 "response to cadmium ion" evidence=IEP] [GO:0005829 "cytosol" evidence=RCA] HAMAP:MF_00493 InterPro:IPR001585 InterPro:IPR004732 InterPro:IPR013785 InterPro:IPR018225 Pfam:PF00923 PIRSF:PIRSF036915 PROSITE:PS01054 GO:GO:0005739 GO:GO:0009570 GO:GO:0046686 EMBL:CP002688 GenomeReviews:BA000015_GR Gene3D:G3DSA:3.20.20.70 GO:GO:0006098 EMBL:AL163572 eggNOG:COG0176 PANTHER:PTHR10683 KO:K00616 GO:GO:0004801 HOGENOM:HBG321149 TIGRFAMs:TIGR00876 OMA:MPEKTLD EMBL:AY056296 EMBL:AY114075 EMBL:AY087135 IPI:IPI00531555 PIR:T48589 RefSeq:NP_196846.1 UniGene:At.22457 ProteinModelPortal:Q9LYR4 SMR:Q9LYR4 IntAct:Q9LYR4 STRING:Q9LYR4 PRIDE:Q9LYR4 EnsemblPlants:AT5G13420.1 GeneID:831183 KEGG:ath:AT5G13420 TAIR:At5g13420 InParanoid:Q9LYR4 PhylomeDB:Q9LYR4 ProtClustDB:CLSN2687015 ArrayExpress:Q9LYR4 Genevestigator:Q9LYR4 Uniprot:Q9LYR4
Root	Isotig04724	10	57	-2.640	2.52E-10	TAIR|locus:2181665 - symbol:AT5G13420 species:3702 "Arabidopsis thaliana" [GO:0003824 "catalytic activity" evidence=IEA] [GO:0004801 "sedoheptulose-7-phosphate:D-glyceraldehyde-3-phosphate glyceronetransferase activity" evidence=IEA;ISS] [GO:0005739 "mitochondrion" evidence=IDA] [GO:0009507 "chloroplast" evidence=IDA] [GO:0009570 "chloroplast stroma" evidence=IDA] [GO:0046686 "response to cadmium ion" evidence=IEP] [GO:0005829 "cytosol" evidence=RCA] HAMAP:MF_00493 InterPro:IPR001585 InterPro:IPR004732 InterPro:IPR013785 InterPro:IPR018225 Pfam:PF00923 PIRSF:PIRSF036915 PROSITE:PS01054 GO:GO:0005739 GO:GO:0009570 GO:GO:0046686 EMBL:CP002688 GenomeReviews:BA000015_GR Gene3D:G3DSA:3.20.20.70 GO:GO:0006098 EMBL:AL163572 eggNOG:COG0176 PANTHER:PTHR10683 KO:K00616 GO:GO:0004801 HOGENOM:HBG321149 TIGRFAMs:TIGR00876 OMA:MPEKTLD EMBL:AY056296 EMBL:AY114075 EMBL:AY087135 IPI:IPI00531555 PIR:T48589 RefSeq:NP_196846.1 UniGene:At.22457 ProteinModelPortal:Q9LYR4 SMR:Q9LYR4 IntAct:Q9LYR4 STRING:Q9LYR4 PRIDE:Q9LYR4 EnsemblPlants:AT5G13420.1 GeneID:831183 KEGG:ath:AT5G13420 TAIR:At5g13420 InParanoid:Q9LYR4 PhylomeDB:Q9LYR4 ProtClustDB:CLSN2687015 ArrayExpress:Q9LYR4 Genevestigator:Q9LYR4 Uniprot:Q9LYR4
Root	Isotig04725	9	40	-2.281	1.10E-06	TAIR|locus:2181665 - symbol:AT5G13420 species:3702 "Arabidopsis thaliana" [GO:0003824 "catalytic activity" evidence=IEA] [GO:0004801 "sedoheptulose-7-phosphate:D-glyceraldehyde-3-phosphate glyceronetransferase activity" evidence=IEA;ISS] [GO:0005739 "mitochondrion" evidence=IDA] [GO:0009507 "chloroplast" evidence=IDA] [GO:0009570 "chloroplast stroma" evidence=IDA] [GO:0046686 "response to cadmium ion" evidence=IEP] [GO:0005829 "cytosol" evidence=RCA] HAMAP:MF_00493 InterPro:IPR001585 InterPro:IPR004732 InterPro:IPR013785 InterPro:IPR018225 Pfam:PF00923 PIRSF:PIRSF036915 PROSITE:PS01054 GO:GO:0005739 GO:GO:0009570 GO:GO:0046686 EMBL:CP002688 GenomeReviews:BA000015_GR Gene3D:G3DSA:3.20.20.70 GO:GO:0006098 EMBL:AL163572 eggNOG:COG0176 PANTHER:PTHR10683 KO:K00616 GO:GO:0004801 HOGENOM:HBG321149 TIGRFAMs:TIGR00876 OMA:MPEKTLD EMBL:AY056296 EMBL:AY114075 EMBL:AY087135 IPI:IPI00531555 PIR:T48589 RefSeq:NP_196846.1 UniGene:At.22457 ProteinModelPortal:Q9LYR4 SMR:Q9LYR4 IntAct:Q9LYR4 STRING:Q9LYR4 PRIDE:Q9LYR4 EnsemblPlants:AT5G13420.1 GeneID:831183 KEGG:ath:AT5G13420 TAIR:At5g13420 InParanoid:Q9LYR4 PhylomeDB:Q9LYR4 ProtClustDB:CLSN2687015 ArrayExpress:Q9LYR4 Genevestigator:Q9LYR4 Uniprot:Q9LYR4
Root	Isotig04732	6	24	-2.129	0.00029855	TAIR|locus:2183730 - symbol:AT5G10770 "AT5G10770" species:3702 "Arabidopsis thaliana" [GO:0003677 "DNA binding" evidence=ISS] [GO:0004190 "aspartic-type endopeptidase activity" evidence=IEA] [GO:0006508 "proteolysis" evidence=IEA] InterPro:IPR001461 InterPro:IPR009007 Pfam:PF00026 PRINTS:PR00792 EMBL:CP002688 GO:GO:0006508 GO:GO:0004190 InterPro:IPR021109 SUPFAM:SSF50630 Gene3D:G3DSA:2.40.70.10 PANTHER:PTHR13683 HSSP:P00799 EMBL:AY075656 EMBL:BT001004 IPI:IPI00543783 RefSeq:NP_196638.2 UniGene:At.32336 ProteinModelPortal:Q8S9J6 MEROPS:A01.A15 PRIDE:Q8S9J6 EnsemblPlants:AT5G10770.1 GeneID:830944 KEGG:ath:AT5G10770 TAIR:At5g10770 InParanoid:Q8S9J6 OMA:LEVVYDG PhylomeDB:Q8S9J6 ProtClustDB:CLSN2686424 Genevestigator:Q8S9J6 Uniprot:Q8S9J6
Root	Isotig04733	5	22	-2.266	0.000318925	TAIR|locus:2183730 - symbol:AT5G10770 "AT5G10770" species:3702 "Arabidopsis thaliana" [GO:0003677 "DNA binding" evidence=ISS] [GO:0004190 "aspartic-type endopeptidase activity" evidence=IEA] [GO:0006508 "proteolysis" evidence=IEA] InterPro:IPR001461 InterPro:IPR009007 Pfam:PF00026 PRINTS:PR00792 EMBL:CP002688 GO:GO:0006508 GO:GO:0004190 InterPro:IPR021109 SUPFAM:SSF50630

Tissue	Identification	# of reads (stress)	# of reads (control)	Fold Change	p-value	Description
						Gene3D:G3DSA:2.40.70.10 PANTHER:PTHR13683 HSSP:P00799 EMBL:AY075656 EMBL:BT001004 IPI:IPI00543783 RefSeq:NP_196638.2 UniGene:At.32336 ProteinModelPortal:Q8S9J6 MEROPS:A01.A15 PRIDE:Q8S9J6 EnsemblPlants:AT5G10770.1 GeneID:830944 KEGG:ath:AT5G10770 TAIR:At5g10770 InParanoid:Q8S9J6 OMA:LEVVYDG PhylomeDB:Q8S9J6 ProtClustDB:CLSN2686424 Genevestigator:Q8S9J6 Uniprot:Q8S9J6
Root	Isotig04734	5	23	-2.331	0.000182103	TAIR|locus:2183730 - symbol:AT5G10770 "AT5G10770" species:3702 "Arabidopsis thaliana" [GO:0003677 "DNA binding" evidence=ISS] [GO:0004190 "aspartic-type endopeptidase activity" evidence=IEA] [GO:0006508 "proteolysis" evidence=IEA] InterPro:IPR001461 InterPro:IPR009007 Pfam:PF00026 PRINTS:PR00792 EMBL:CP002688 GO:GO:0006508 GO:GO:0004190 InterPro:IPR021109 SUPFAM:SSF50630 Gene3D:G3DSA:2.40.70.10 PANTHER:PTHR13683 HSSP:P00799 EMBL:AY075656 EMBL:BT001004 IPI:IPI00543783 RefSeq:NP_196638.2 UniGene:At.32336 ProteinModelPortal:Q8S9J6 MEROPS:A01.A15 PRIDE:Q8S9J6 EnsemblPlants:AT5G10770.1 GeneID:830944 KEGG:ath:AT5G10770 TAIR:At5g10770 InParanoid:Q8S9J6 OMA:LEVVYDG PhylomeDB:Q8S9J6 ProtClustDB:CLSN2686424 Genevestigator:Q8S9J6 Uniprot:Q8S9J6
Root	Isotig04750	49	17	1.398	0.000229772	TAIR|locus:2127173 - symbol:GSH1 "AT4G23100" species:3702 "Arabidopsis thaliana" [GO:0009908 "flower development" evidence=IMP] [GO:0009507 "chloroplast" evidence=IDA] [GO:0004357 "glutamate-cysteine ligase activity" evidence=IMP;IDA] [GO:0006750 "glutathione biosynthetic process" evidence=IMP;IDA;TAS] [GO:0009408 "response to heat" evidence=IMP] [GO:0009570 "chloroplast stroma" evidence=IDA] [GO:0009753 "response to jasmonic acid stimulus" evidence=IEP] [GO:0010193 "response to ozone" evidence=IEP] [GO:0046686 "response to cadmium ion" evidence=IEP;IMP] [GO:0050832 "defense response to fungus" evidence=IMP] [GO:0002213 "defense response to insect" evidence=IMP] [GO:0019761 "glucosinolate biosynthetic process" evidence=IMP] [GO:0042742 "defense response to bacterium" evidence=IMP] [GO:0052544 "defense response by callose deposition in cell wall" evidence=IMP] [GO:0009700 "indole phytoalexin biosynthetic process" evidence=IMP] [GO:0009816 "defense response to bacterium, incompatible interaction" evidence=IMP] InterPro:IPR006336 InterPro:IPR011556 Pfam:PF04107 GO:GO:0005524 EMBL:CP002687 GenomeReviews:CT486007_GR GO:GO:0009570 GO:GO:0046686 GO:GO:0009753 GO:GO:0050832 GO:GO:0019761 GO:GO:0052544 GO:GO:0010193 GO:GO:0009408 GO:GO:0002213 GO:GO:0009908 EMBL:AL031018 EMBL:AL161558 GO:GO:0009816 GO:GO:0004357 GO:GO:0006750 BRENDA:6.3.2.2 EMBL:Z29490 EMBL:Y09944 EMBL:AF068299 EMBL:DQ993178 EMBL:AF419576 EMBL:AF428393 EMBL:AY056372 EMBL:AY143970 IPI:IPI00536188 PIR:T05142 RefSeq:NP_001190808.1 RefSeq:NP_194041.1 UniGene:At.25074 ProteinModelPortal:P46309 SMR:P46309 STRING:P46309 PRIDE:P46309 EnsemblPlants:AT4G23100.1 EnsemblPlants:AT4G23100.3 GeneID:828409 KEGG:ath:AT4G23100 TAIR:At4g23100 eggNOG:COG3572 HOGENOM:HBG294235 InParanoid:P46309 KO:K01919 OMA:SIEPGGQ PhylomeDB:P46309 ProtClustDB:PLN02611 BioCyc:ARA:AT4G23100-MONOMER BioCyc:MetaCyc:AT4G23100-MONOMER ArrayExpress:P46309 Genevestigator:P46309 GO:GO:0009700 TIGRFAMs:TIGR01436 Uniprot:P46309
Root	Isotig04751	48	14	1.649	3.85E-05	TAIR|locus:2127173 - symbol:GSH1 "AT4G23100" species:3702 "Arabidopsis thaliana" [GO:0009908 "flower development" evidence=IMP] [GO:0009507 "chloroplast" evidence=IDA] [GO:0004357 "glutamate-cysteine ligase activity" evidence=IMP;IDA] [GO:0006750 "glutathione biosynthetic process" evidence=IMP;IDA;TAS] [GO:0009408 "response to heat" evidence=IMP] [GO:0009570 "chloroplast stroma" evidence=IDA] [GO:0009753 "response to jasmonic acid stimulus" evidence=IEP] [GO:0010193 "response to ozone" evidence=IEP] [GO:0046686 "response to cadmium ion" evidence=IEP;IMP] [GO:0050832 "defense response to fungus" evidence=IMP] [GO:0002213 "defense response to insect" evidence=IMP] [GO:0019761 "glucosinolate biosynthetic process" evidence=IMP] [GO:0042742 "defense response to bacterium" evidence=IMP] [GO:0052544 "defense response by callose deposition in cell wall" evidence=IMP] [GO:0009700 "indole phytoalexin biosynthetic process" evidence=IMP] [GO:0009816 "defense response to bacterium, incompatible interaction" evidence=IMP] InterPro:IPR006336 InterPro:IPR011556 Pfam:PF04107 GO:GO:0005524 EMBL:CP002687 GenomeReviews:CT486007_GR GO:GO:0009570 GO:GO:0046686 GO:GO:0009753 GO:GO:0050832 GO:GO:0019761 GO:GO:0052544 GO:GO:0010193 GO:GO:0009408 GO:GO:0002213 GO:GO:0009908 EMBL:AL031018 EMBL:AL161558 GO:GO:0009816 GO:GO:0004357 GO:GO:0006750 BRENDA:6.3.2.2 EMBL:Z29490 EMBL:Y09944 EMBL:AF068299 EMBL:DQ993178 EMBL:AF419576 EMBL:AF428393 EMBL:AY056372 EMBL:AY143970 IPI:IPI00536188 PIR:T05142 RefSeq:NP_001190808.1 RefSeq:NP_194041.1 UniGene:At.25074 ProteinModelPortal:P46309 SMR:P46309 STRING:P46309 PRIDE:P46309 EnsemblPlants:AT4G23100.1

Tissue	Identification	# of reads (stress)	# of reads (control)	Fold Change	p-value	Description
						EnsemblPlants:AT4G23100.3 GeneID:828409 KEGG:ath:AT4G23100 TAIR:At4g23100 eggNOG:COG3572 HOGENOM:HBG294235 InParanoid:P46309 KO:K01919 OMA:SIEPGGQ PhylomeDB:P46309 ProtClustDB:PLN02611 BioCyc:ARA:AT4G23100-MONOMER BioCyc:MetaCyc:AT4G23100-MONOMER ArrayExpress:P46309 Genevestigator:P46309 GO:GO:0009700 TIGRFAMs:TIGR01436 Uniprot:P46309
Root	Isotig04765	24	4	2.456	0.000163328	TAIR|locus:2198646 - symbol:AT1G20110 "AT1G20110" species:3702 "Arabidopsis thaliana" [GO:0007165 "signal transduction" evidence=ISS] [GO:0035091 "phosphatidylinositol binding" evidence=ISS] InterPro:IPR000306 Pfam:PF01363 SMART:SM00064 InterPro:IPR011011 EMBL:CP002684 GO:GO:0046872 InterPro:IPR017455 InterPro:IPR013083 Gene3D:G3DSA:3.30.40.10 SUPFAM:SSF57903 PROSITE:PS50178 HSSP:Q15075 EMBL:AF367315 EMBL:AF428277 EMBL:AY143942 IPI:IPI00537339 RefSeq:NP_564103.1 UniGene:At.15444 ProteinModelPortal:Q9ASS2 SMR:Q9ASS2 IntAct:Q9ASS2 PRIDE:Q9ASS2 EnsemblPlants:AT1G20110.1 GeneID:838600 KEGG:ath:AT1G20110 TAIR:At1g20110 InParanoid:Q9ASS2 OMA:ENITRCE PhylomeDB:Q9ASS2 ProtClustDB:CLSN2917026 Genevestigator:Q9ASS2 Uniprot:Q9ASS2
Root	Isotig04766	24	4	2.456	0.000163328	TAIR|locus:2198646 - symbol:AT1G20110 "AT1G20110" species:3702 "Arabidopsis thaliana" [GO:0007165 "signal transduction" evidence=ISS] [GO:0035091 "phosphatidylinositol binding" evidence=ISS] InterPro:IPR000306 Pfam:PF01363 SMART:SM00064 InterPro:IPR011011 EMBL:CP002684 GO:GO:0046872 InterPro:IPR017455 InterPro:IPR013083 Gene3D:G3DSA:3.30.40.10 SUPFAM:SSF57903 PROSITE:PS50178 HSSP:Q15075 EMBL:AF367315 EMBL:AF428277 EMBL:AY143942 IPI:IPI00537339 RefSeq:NP_564103.1 UniGene:At.15444 ProteinModelPortal:Q9ASS2 SMR:Q9ASS2 IntAct:Q9ASS2 PRIDE:Q9ASS2 EnsemblPlants:AT1G20110.1 GeneID:838600 KEGG:ath:AT1G20110 TAIR:At1g20110 InParanoid:Q9ASS2 OMA:ENITRCE PhylomeDB:Q9ASS2 ProtClustDB:CLSN2917026 Genevestigator:Q9ASS2 Uniprot:Q9ASS2
Root	Isotig04767	24	5	2.134	0.000532871	TAIR|locus:2198646 - symbol:AT1G20110 "AT1G20110" species:3702 "Arabidopsis thaliana" [GO:0007165 "signal transduction" evidence=ISS] [GO:0035091 "phosphatidylinositol binding" evidence=ISS] InterPro:IPR000306 Pfam:PF01363 SMART:SM00064 InterPro:IPR011011 EMBL:CP002684 GO:GO:0046872 InterPro:IPR017455 InterPro:IPR013083 Gene3D:G3DSA:3.30.40.10 SUPFAM:SSF57903 PROSITE:PS50178 HSSP:Q15075 EMBL:AF367315 EMBL:AF428277 EMBL:AY143942 IPI:IPI00537339 RefSeq:NP_564103.1 UniGene:At.15444 ProteinModelPortal:Q9ASS2 SMR:Q9ASS2 IntAct:Q9ASS2 PRIDE:Q9ASS2 EnsemblPlants:AT1G20110.1 GeneID:838600 KEGG:ath:AT1G20110 TAIR:At1g20110 InParanoid:Q9ASS2 OMA:ENITRCE PhylomeDB:Q9ASS2 ProtClustDB:CLSN2917026 Genevestigator:Q9ASS2 Uniprot:Q9ASS2
Root	Isotig04792	24	53	-1.272	0.000195269	TAIR|locus:2091965 - symbol:AT3G27090 "AT3G27090" species:3702 "Arabidopsis thaliana" [GO:0003674 "molecular_function" evidence=ND] [GO:0008150 "biological_process" evidence=ND] GenomeReviews:BA000014_GR InterPro:IPR013989 Pfam:PF10539 SMART:SM00767 PROSITE:PS51222 EMBL:AY087011 IPI:IPI00536337 PRIDE:Q8LBT1 TAIR:At3g27090 eggNOG:NOG302982 PhylomeDB:Q8LBT1 Genevestigator:Q8LBT1 Uniprot:Q8LBT1
Root	Isotig04793	17	69	-2.150	6.81E-10	TAIR|locus:2091965 - symbol:AT3G27090 "AT3G27090" species:3702 "Arabidopsis thaliana" [GO:0003674 "molecular_function" evidence=ND] [GO:0008150 "biological_process" evidence=ND] GenomeReviews:BA000014_GR InterPro:IPR013989 Pfam:PF10539 SMART:SM00767 PROSITE:PS51222 EMBL:AY087011 IPI:IPI00536337 PRIDE:Q8LBT1 TAIR:At3g27090 eggNOG:NOG302982 PhylomeDB:Q8LBT1 Genevestigator:Q8LBT1 Uniprot:Q8LBT1
Root	Isotig04794	14	64	-2.322	4.70E-10	TAIR|locus:2091965 - symbol:AT3G27090 "AT3G27090" species:3702 "Arabidopsis thaliana" [GO:0003674 "molecular_function" evidence=ND] [GO:0008150 "biological_process" evidence=ND] GenomeReviews:BA000014_GR InterPro:IPR013989 Pfam:PF10539 SMART:SM00767 PROSITE:PS51222 EMBL:AY087011 IPI:IPI00536337 PRIDE:Q8LBT1 TAIR:At3g27090 eggNOG:NOG302982 PhylomeDB:Q8LBT1 Genevestigator:Q8LBT1 Uniprot:Q8LBT1
Root	Isotig04795	124	14	3.018	1.26E-21	TAIR|locus:2206405 - symbol:ALDH5F1 "AT1G79440" species:3702 "Arabidopsis thaliana" [GO:0004028 "3-chloroallyl aldehyde dehydrogenase activity" evidence=ISS] [GO:0004777 "succinate-semialdehyde dehydrogenase activity" evidence=ISS;IDA] [GO:0005759 "mitochondrial matrix" evidence=IDA] [GO:0006540 "glutamate decarboxylation to succinate" evidence=IMP;IDA] [GO:0009450 "gamma-aminobutyric acid catabolic process" evidence=IDA] [GO:0051287 "NAD binding" evidence=IDA] [GO:0005739 "mitochondrion" evidence=IDA] [GO:0006950 "response to stress" evidence=IMP] [GO:0009408 "response to heat" evidence=IMP] [GO:0009416 "response to light stimulus" evidence=IMP] [GO:0072593 "reactive oxygen species metabolic process" evidence=IMP] [GO:0009507 "chloroplast" evidence=IDA] [GO:0005507 "copper ion binding" evidence=IDA] [GO:0009570 "chloroplast stroma" evidence=IDA] InterPro:IPR010102 InterPro:IPR015590 InterPro:IPR016160

Tissue	Identification	# of reads (stress)	# of reads (control)	Fold Change	p-value	Description
						InterPro:IPR016161 InterPro:IPR016162 InterPro:IPR016163 Pfam:PF00171 PROSITE:PS00070 PROSITE:PS00687 EMBL:CP002684 GenomeReviews:CT485782_GR GO:GO:0009570 GO:GO:0051287 GO:GO:0005759 eggNOG:COG1012 Gene3D:G3DSA:3.40.309.10 Gene3D:G3DSA:3.40.605.10 SUPFAM:SSF53720 HOGENOM:HBG752218 GO:GO:0005507 GO:GO:0009408 GO:GO:0009416 GO:GO:0072593 HSSP:P05091 EMBL:AC007202 GO:GO:0009450 GO:GO:0006540 KO:K00135 TIGRFAMs:TIGR01780 GO:GO:0004777 OMA:MIQNKDD EMBL:AF117335 EMBL:AF428367 EMBL:AY056147 IPI:IPI00532908 PIR:E96825 RefSeq:NP_178062.1 UniGene:At.11884 ProteinModelPortal:Q9SAK4 SMR:Q9SAK4 STRING:Q9SAK4 PRIDE:Q9SAK4 EnsemblPlants:AT1G79440.1 GeneID:844282 KEGG:ath:AT1G79440 GeneFarm:4340 TAIR:At1g79440 InParanoid:Q9SAK4 PhylomeDB:Q9SAK4 ProtClustDB:PLN02278 ArrayExpress:Q9SEK4 Genevestigator:Q9SAK4 Uniprot:Q9SAK4
Root	Isotig04796	121	13	3.089	1.41E-21	TAIR|locus:2206405 - symbol:ALDH5F1 "AT1G79440" species:3702 "Arabidopsis thaliana" [GO:0004028 "3-chloroallyl aldehyde dehydrogenase activity" evidence=ISS] [GO:0004777 "succinate-semialdehyde dehydrogenase activity" evidence=ISS;IDA] [GO:0005759 "mitochondrial matrix" evidence=IDA] [GO:0006540 "glutamate decarboxylation to succinate" evidence=IMP;IDA] [GO:0009450 "gamma-aminobutyric acid catabolic process" evidence=IDA] [GO:0051287 "NAD binding" evidence=IDA] [GO:0005739 "mitochondrion" evidence=IDA] [GO:0006950 "response to stress" evidence=IMP] [GO:0009408 "response to heat" evidence=IMP] [GO:0009416 "response to light stimulus" evidence=IMP] [GO:0072593 "reactive oxygen species metabolic process" evidence=IMP] [GO:0009507 "chloroplast" evidence=IDA] [GO:0005507 "copper ion binding" evidence=IDA] [GO:0009570 "chloroplast stroma" evidence=IDA] InterPro:IPR010102 InterPro:IPR015590 InterPro:IPR016160 InterPro:IPR016161 InterPro:IPR016162 InterPro:IPR016163 Pfam:PF00171 PROSITE:PS00070 PROSITE:PS00687 EMBL:CP002684 GenomeReviews:CT485782_GR GO:GO:0009570 GO:GO:0051287 GO:GO:0005759 eggNOG:COG1012 Gene3D:G3DSA:3.40.309.10 Gene3D:G3DSA:3.40.605.10 SUPFAM:SSF53720 HOGENOM:HBG752218 GO:GO:0005507 GO:GO:0009408 GO:GO:0009416 GO:GO:0072593 HSSP:P05091 EMBL:AC007202 GO:GO:0009450 GO:GO:0006540 KO:K00135 TIGRFAMs:TIGR01780 GO:GO:0004777 OMA:MIQNKDD EMBL:AF117335 EMBL:AF428367 EMBL:AY056147 IPI:IPI00532908 PIR:E96825 RefSeq:NP_178062.1 UniGene:At.11884 ProteinModelPortal:Q9SAK4 SMR:Q9SAK4 STRING:Q9SAK4 PRIDE:Q9SAK4 EnsemblPlants:AT1G79440.1 GeneID:844282 KEGG:ath:AT1G79440 GeneFarm:4340 TAIR:At1g79440 InParanoid:Q9SAK4 PhylomeDB:Q9SAK4 ProtClustDB:PLN02278 ArrayExpress:Q9SEK4 Genevestigator:Q9SAK4 Uniprot:Q9SAK4
Root	Isotig04801	2	18	-3.299	7.42E-05	TAIR|locus:2051859 - symbol:MLO15 "AT2G44110" species:3702 "Arabidopsis thaliana" [GO:0005516 "calmodulin binding" evidence=ISS] [GO:0006952 "defense response" evidence=ISS] [GO:0008219 "cell death" evidence=IEA;ISS] [GO:0016021 "integral to membrane" evidence=IEA;ISS] InterPro:IPR004326 Pfam:PF03094 GO:GO:0016021 EMBL:CP002685 GenomeReviews:CT485783_GR GO:GO:0006952 GO:GO:0008219 GO:GO:0005516 GO:GO:0009607 EMBL:AC004005 HOGENOM:HBG319688 KO:K08472 eggNOG:NOG307472 EMBL:AF369576 IPI:IPI00536590 PIR:T00691 RefSeq:NP_181939.1 UniGene:At.19505 PRIDE:O80580 EnsemblPlants:AT2G44110.1 GeneID:819017 KEGG:ath:AT2G44110 TAIR:At2g44110 InParanoid:O80580 PhylomeDB:O80580 ProtClustDB:CLSN2683190 ArrayExpress:O80580 Genevestigator:O80580 Uniprot:O80580
Root	Isotig04802	2	18	-3.299	7.42E-05	TAIR|locus:2051859 - symbol:MLO15 "AT2G44110" species:3702 "Arabidopsis thaliana" [GO:0005516 "calmodulin binding" evidence=ISS] [GO:0006952 "defense response" evidence=ISS] [GO:0008219 "cell death" evidence=IEA;ISS] [GO:0016021 "integral to membrane" evidence=IEA;ISS] InterPro:IPR004326 Pfam:PF03094 GO:GO:0016021 EMBL:CP002685 GenomeReviews:CT485783_GR GO:GO:0006952 GO:GO:0008219 GO:GO:0005516 GO:GO:0009607 EMBL:AC004005 HOGENOM:HBG319688 KO:K08472 eggNOG:NOG307472 EMBL:AF369576 IPI:IPI00536590 PIR:T00691 RefSeq:NP_181939.1 UniGene:At.19505 PRIDE:O80580 EnsemblPlants:AT2G44110.1 GeneID:819017 KEGG:ath:AT2G44110 TAIR:At2g44110 InParanoid:O80580 PhylomeDB:O80580 ProtClustDB:CLSN2683190 ArrayExpress:O80580 Genevestigator:O80580 Uniprot:O80580
Root	Isotig04810	44	6	2.745	5.73E-08	TAIR|locus:2178128 - symbol:ACT7 "AT5G09810" species:3702 "Arabidopsis thaliana" [GO:0007010 "cytoskeleton organization" evidence=TAS] [GO:0009733 "response to auxin stimulus" evidence=IEP] [GO:0009845 "seed germination" evidence=IMP] [GO:0048364 "root development" evidence=IMP] [GO:0005739 "mitochondrion" evidence=IDA] [GO:0005730 "nucleolus" evidence=IDA] [GO:0005618 "cell wall" evidence=IDA] [GO:0005886 "plasma membrane" evidence=IDA] [GO:0010053 "root epidermal cell differentiation" evidence=IMP] [GO:0048767 "root hair elongation" evidence=IMP] [GO:0051301 "cell division" evidence=IMP] [GO:0005515 "protein binding" evidence=IPI] [GO:0009570 "chloroplast stroma" evidence=IDA] [GO:0009941 "chloroplast envelope" evidence=IDA]

Tissue	Identification	# of reads (stress)	# of reads (control)	Fold Change	p-value	Description
						[GO:0005829 "cytosol" evidence=IDA] [GO:0009506 "plasmodesma" evidence=IDA] [GO:0005200 "structural constituent of cytoskeleton" evidence=ISS] [GO:0005856 "cytoskeleton" evidence=ISS] [GO:0009416 "response to light stimulus" evidence=IEP] [GO:0009611 "response to wounding" evidence=IEP] InterPro:IPR004001 PROSITE:PS00406 PROSITE:PS00432 InterPro:IPR004000 GO:GO:0005829 GO:GO:0005739 GO:GO:0005886 GO:GO:0009506 GO:GO:0005524 GO:GO:0009570 EMBL:CP002688 GenomeReviews:BA000015_GR GO:GO:0005618 GO:GO:0005515 GO:GO:0009733 GO:GO:0009611 GO:GO:0005730 GO:GO:0005856 GO:GO:0051301 GO:GO:0009941 EMBL:AB016893 GO:GO:0048767 GO:GO:0009845 GO:GO:0009416 HOGENOM:HBG559892 PANTHER:PTHR11937 Pfam:PF00022 PRINTS:PR00190 SMART:SM00268 KO:K10355 InterPro:IPR020902 PROSITE:PS01132 ProtClustDB:CLSN2682469 UniGene:At.23346 EMBL:U37281 EMBL:U27811 EMBL:AY062702 EMBL:AY063980 EMBL:AY096397 EMBL:AY114679 EMBL:AY120779 IPI:IPI00524611 PIR:S68107 RefSeq:NP_196543.1 UniGene:At.23605 UniGene:At.24396 ProteinModelPortal:P53492 SMR:P53492 IntAct:P53492 STRING:P53492 PRIDE:P53492 ProMEX:P53492 EnsemblPlants:AT5G09810.1 GeneID:830841 KEGG:ath:AT5G09810 TAIR:At5g09810 InParanoid:P53492 OMA:NSICVIL PhylomeDB:P53492 ArrayExpress:P53492 Genevestigator:P53492 GermOnline:AT5G09810 Uniprot:P53492
Root	Isotig04811	46	11	1.935	6.77E-06	TAIR|locus:2178128 - symbol:ACT7 "AT5G09810" species:3702 "Arabidopsis thaliana" [GO:0007010 "cytoskeleton organization" evidence=TAS] [GO:0009733 "response to auxin stimulus" evidence=IEP] [GO:0009845 "seed germination" evidence=IMP] [GO:0048364 "root development" evidence=IMP] [GO:0005739 "mitochondrion" evidence=IDA] [GO:0005730 "nucleolus" evidence=IDA] [GO:0005618 "cell wall" evidence=IDA] [GO:0005886 "plasma membrane" evidence=IDA] [GO:0010053 "root epidermal cell differentiation" evidence=IMP] [GO:0048767 "root hair elongation" evidence=IMP] [GO:0051301 "cell division" evidence=IMP] [GO:0005515 "protein binding" evidence=IPI] [GO:0009570 "chloroplast stroma" evidence=IDA] [GO:0009941 "chloroplast envelope" evidence=IDA] [GO:0005829 "cytosol" evidence=IDA] [GO:0009506 "plasmodesma" evidence=IDA] [GO:0005200 "structural constituent of cytoskeleton" evidence=ISS] [GO:0005856 "cytoskeleton" evidence=ISS] [GO:0009416 "response to light stimulus" evidence=IEP] [GO:0009611 "response to wounding" evidence=IEP] InterPro:IPR004001 PROSITE:PS00406 PROSITE:PS00432 InterPro:IPR004000 GO:GO:0005829 GO:GO:0005739 GO:GO:0005886 GO:GO:0009506 GO:GO:0005524 GO:GO:0009570 EMBL:CP002688 GenomeReviews:BA000015_GR GO:GO:0005618 GO:GO:0005515 GO:GO:0009733 GO:GO:0009611 GO:GO:0005730 GO:GO:0005856 GO:GO:0051301 GO:GO:0009941 EMBL:AB016893 GO:GO:0048767 GO:GO:0009845 GO:GO:0009416 HOGENOM:HBG559892 PANTHER:PTHR11937 Pfam:PF00022 PRINTS:PR00190 SMART:SM00268 KO:K10355 InterPro:IPR020902 PROSITE:PS01132 ProtClustDB:CLSN2682469 UniGene:At.23346 EMBL:U37281 EMBL:U27811 EMBL:AY062702 EMBL:AY063980 EMBL:AY096397 EMBL:AY114679 EMBL:AY120779 IPI:IPI00524611 PIR:S68107 RefSeq:NP_196543.1 UniGene:At.23605 UniGene:At.24396 ProteinModelPortal:P53492 SMR:P53492 IntAct:P53492 STRING:P53492 PRIDE:P53492 ProMEX:P53492 EnsemblPlants:AT5G09810.1 GeneID:830841 KEGG:ath:AT5G09810 TAIR:At5g09810 InParanoid:P53492 OMA:NSICVIL PhylomeDB:P53492 ArrayExpress:P53492 Genevestigator:P53492 GermOnline:AT5G09810 Uniprot:P53492
Root	Isotig04812	39	5	2.835	2.06E-07	TAIR|locus:2178128 - symbol:ACT7 "AT5G09810" species:3702 "Arabidopsis thaliana" [GO:0007010 "cytoskeleton organization" evidence=TAS] [GO:0009733 "response to auxin stimulus" evidence=IEP] [GO:0009845 "seed germination" evidence=IMP] [GO:0048364 "root development" evidence=IMP] [GO:0005739 "mitochondrion" evidence=IDA] [GO:0005730 "nucleolus" evidence=IDA] [GO:0005618 "cell wall" evidence=IDA] [GO:0005886 "plasma membrane" evidence=IDA] [GO:0010053 "root epidermal cell differentiation" evidence=IMP] [GO:0048767 "root hair elongation" evidence=IMP] [GO:0051301 "cell division" evidence=IMP] [GO:0005515 "protein binding" evidence=IPI] [GO:0009570 "chloroplast stroma" evidence=IDA] [GO:0009941 "chloroplast envelope" evidence=IDA] [GO:0005829 "cytosol" evidence=IDA] [GO:0009506 "plasmodesma" evidence=IDA] [GO:0005200 "structural constituent of cytoskeleton" evidence=ISS] [GO:0005856 "cytoskeleton" evidence=ISS] [GO:0009416 "response to light stimulus" evidence=IEP] [GO:0009611 "response to wounding" evidence=IEP] InterPro:IPR004001 PROSITE:PS00406 PROSITE:PS00432 InterPro:IPR004000 GO:GO:0005829 GO:GO:0005739 GO:GO:0005886 GO:GO:0009506 GO:GO:0005524 GO:GO:0009570 EMBL:CP002688 GenomeReviews:BA000015_GR GO:GO:0005618 GO:GO:0005515 GO:GO:0009733 GO:GO:0009611 GO:GO:0005730 GO:GO:0005856 GO:GO:0051301 GO:GO:0009941 EMBL:AB016893 GO:GO:0048767 GO:GO:0009845 GO:GO:0009416 HOGENOM:HBG559892 PANTHER:PTHR11937 Pfam:PF00022 PRINTS:PR00190 SMART:SM00268 KO:K10355 InterPro:IPR020902 PROSITE:PS01132 ProtClustDB:CLSN2682469 UniGene:At.23346 EMBL:U37281

Tissue	Identification	# of reads (stress)	# of reads (control)	Fold Change	p-value	Description
						EMBL:U27811 EMBL:AY062702 EMBL:AY063980 EMBL:AY096397 EMBL:AY114679 EMBL:AY120779 IPI:IPI00524611 PIR:S68107 RefSeq:NP_196543.1 UniGene:At.23605 UniGene:At.24396 ProteinModelPortal:P53492 SMR:P53492 IntAct:P53492 STRING:P53492 PRIDE:P53492 ProMEX:P53492 EnsemblPlants:AT5G09810.1 GeneID:830841 KEGG:ath:AT5G09810 TAIR:At5g09810 InParanoid:P53492 OMA:NSICVIL PhylomeDB:P53492 ArrayExpress:P53492 Genevestigator:P53492 GermOnline:AT5G09810 Uniprot:P53492
Root	Isotig04826	2	15	-3.036	0.000493759	TAIR|locus:2125766 - symbol:CRK40 "cysteine-rich RLK (RECEPTOR-like protein kinase) 40" species:3702 "Arabidopsis thaliana" [GO:0006468 "protein phosphorylation" evidence=IEA] [GO:0016301 "kinase activity" evidence=ISS] InterPro:IPR000719 InterPro:IPR008271 InterPro:IPR011009 InterPro:IPR017441 Pfam:PF00069 PROSITE:PS00107 PROSITE:PS00108 PROSITE:PS50011 GO:GO:0016021 GO:GO:0005524 EMBL:CP002687 GenomeReviews:CT486007_GR eggNOG:COG0515 GO:GO:0004872 SUPFAM:SSF56112 GO:GO:0004674 HOGENOM:HBG755340 EMBL:AL161501 EMBL:AF074021 InterPro:IPR002902 Pfam:PF01657 PROSITE:PS51473 ProtClustDB:CLSN2685351 EMBL:AY127017 IPI:IPI00540978 PIR:F85057 RefSeq:NP_192366.1 UniGene:At.33996 ProteinModelPortal:Q9SYS3 SMR:Q9SYS3 IntAct:Q9SYS3 STRING:Q9SYS3 PRIDE:Q9SYS3 EnsemblPlants:AT4G04570.1 GeneID:825789 KEGG:ath:AT4G04570 GeneFarm:748 TAIR:At4g04570 InParanoid:Q9SYS3 OMA:QSESGAM PhylomeDB:Q9SYS3 Genevestigator:Q9SYS3 Uniprot:Q9SYS3
Root	Isotig04832	27	5	2.304	0.000117535	TAIR|locus:2027082 - symbol:SKP2A "AT1G21410" species:3702 "Arabidopsis thaliana" [GO:0005515 "protein binding" evidence=IPI] [GO:0019005 "SCF ubiquitin ligase complex" evidence=IDA;IPI] [GO:0031146 "SCF-dependent proteasomal ubiquitin-dependent protein catabolic process" evidence=IDA;IPI] [GO:0005634 "nucleus" evidence=IDA] [GO:0010386 "lateral root primordium development" evidence=IMP] [GO:0051781 "positive regulation of cell division" evidence=IMP] [GO:0004842 "ubiquitin-protein ligase activity" evidence=IDA] [GO:0016567 "protein ubiquitination" evidence=IDA] [GO:0071365 "cellular response to auxin stimulus" evidence=IDA] InterPro:IPR001810 Pfam:PF00646 PROSITE:PS50181 SMART:SM00256 EMBL:CP002684 GenomeReviews:CT485782_GR GO:GO:0005634 GO:GO:0005515 GO:GO:0009734 GO:GO:0051781 SUPFAM:SSF81383 GO:GO:0019005 GO:GO:0031146 EMBL:AC015447 GO:GO:0004842 InterPro:IPR006553 SMART:SM00367 GO:GO:0010386 EMBL:AY058081 EMBL:AY090308 EMBL:AY085898 IPI:IPI00519620 PIR:B86347 RefSeq:NP_564139.1 UniGene:At.25230 ProteinModelPortal:Q9LPL4 SMR:Q9LPL4 IntAct:Q9LPL4 STRING:Q9LPL4 EnsemblPlants:AT1G21410.1 GeneID:838740 KEGG:ath:AT1G21410 TAIR:At1g21410 eggNOG:NOG257455 InParanoid:Q9LPL4 OMA:NLNISQC PhylomeDB:Q9LPL4 ProtClustDB:CLSN2687892 Genevestigator:Q9LPL4 Uniprot:Q9LPL4
Root	Isotig04833	27	5	2.304	0.000117535	TAIR|locus:2027082 - symbol:SKP2A "AT1G21410" species:3702 "Arabidopsis thaliana" [GO:0005515 "protein binding" evidence=IPI] [GO:0019005 "SCF ubiquitin ligase complex" evidence=IDA;IPI] [GO:0031146 "SCF-dependent proteasomal ubiquitin-dependent protein catabolic process" evidence=IDA;IPI] [GO:0005634 "nucleus" evidence=IDA] [GO:0010386 "lateral root primordium development" evidence=IMP] [GO:0051781 "positive regulation of cell division" evidence=IMP] [GO:0004842 "ubiquitin-protein ligase activity" evidence=IDA] [GO:0016567 "protein ubiquitination" evidence=IDA] [GO:0071365 "cellular response to auxin stimulus" evidence=IDA] InterPro:IPR001810 Pfam:PF00646 PROSITE:PS50181 SMART:SM00256 EMBL:CP002684 GenomeReviews:CT485782_GR GO:GO:0005634 GO:GO:0005515 GO:GO:0009734 GO:GO:0051781 SUPFAM:SSF81383 GO:GO:0019005 GO:GO:0031146 EMBL:AC015447 GO:GO:0004842 InterPro:IPR006553 SMART:SM00367 GO:GO:0010386 EMBL:AY058081 EMBL:AY090308 EMBL:AY085898 IPI:IPI00519620 PIR:B86347 RefSeq:NP_564139.1 UniGene:At.25230 ProteinModelPortal:Q9LPL4 SMR:Q9LPL4 IntAct:Q9LPL4 STRING:Q9LPL4 EnsemblPlants:AT1G21410.1 GeneID:838740 KEGG:ath:AT1G21410 TAIR:At1g21410 eggNOG:NOG257455 InParanoid:Q9LPL4 OMA:NLNISQC PhylomeDB:Q9LPL4 ProtClustDB:CLSN2687892 Genevestigator:Q9LPL4 Uniprot:Q9LPL4
Root	Isotig04837	9	28	-1.766	0.000557996	TAIR|locus:2007253 - symbol:MTK "AT1G49820" species:3702 "Arabidopsis thaliana" [GO:0046522 "S-methyl-5-thioribose kinase activity" evidence=ISS;IDA;IMP] [GO:0019509 "L-methionine salvage from methylthioadenosine" evidence=TAS] [GO:0042802 "identical protein binding" evidence=IPI] [GO:0071281 "cellular response to iron ion" evidence=IEP] [GO:0071369 "cellular response to ethylene stimulus" evidence=IEP] [GO:0071732 "cellular response to nitric oxide" evidence=IEP] [GO:0005829 "cytosol" evidence=IDA] InterPro:IPR009212 InterPro:IPR011009 PIRSF:PIRSF031134 Pfam:PF01636 EMBL:CP002684 GenomeReviews:CT485782_GR GO:GO:0005829 GO:GO:0005524 GO:GO:0042802 GO:GO:0071281 SUPFAM:SSF56112 GO:GO:0019509

Tissue	Identification	# of reads (stress)	# of reads (control)	Fold Change	p-value	Description
						GO:GO:0071732 InterPro:IPR002575 GO:GO:0071369 EMBL:AC079674 EMBL:AY057512 EMBL:AY090315 IPI:IPI00544339 PIR:A96535 RefSeq:NP_564555.1 UniGene:At.20360 PDB:2PYW PDBsum:2PYW ProteinModelPortal:Q9C6D2 SMR:Q9C6D2 STRING:Q9C6D2 PRIDE:Q9C6D2 EnsemblPlants:AT1G49820.1 GeneID:841405 KEGG:ath:AT1G49820 TAIR:At1g49820 eggNOG:COG4857 HOGENOM:HBG305135 InParanoid:Q9C6D2 OMA:REIGDGN PhylomeDB:Q9C6D2 ProtClustDB:PLN02756 Genevestigator:Q9C6D2 GO:GO:0046522 TIGRFAMs:TIGR01767 Uniprot:Q9C6D2
Root	Isotig04838	8	30	-2.036	8.51E-05	TAIR|locus:2007253 - symbol:MTK "AT1G49820" species:3702 "Arabidopsis thaliana" [GO:0046522 "S-methyl-5-thioribose kinase activity" evidence=ISS;IDA;IMP] [GO:0019509 "L-methionine salvage from methylthioadenosine" evidence=TAS] [GO:0042802 "identical protein binding" evidence=IPI] [GO:0071281 "cellular response to iron ion" evidence=IEP] [GO:0071369 "cellular response to ethylene stimulus" evidence=IEP] [GO:0071732 "cellular response to nitric oxide" evidence=IEP] [GO:0005829 "cytosol" evidence=IDA] InterPro:IPR009212 InterPro:IPR011009 PIRSF:PIRSF031134 Pfam:PF01636 EMBL:CP002684 GenomeReviews:CT485782_GR GO:GO:0005829 GO:GO:0005524 GO:GO:0042802 GO:GO:0071281 SUPFAM:SSF56112 GO:GO:0019509 GO:GO:0071732 InterPro:IPR002575 GO:GO:0071369 EMBL:AC079674 EMBL:AY057512 EMBL:AY090315 IPI:IPI00544339 PIR:A96535 RefSeq:NP_564555.1 UniGene:At.20360 PDB:2PYW PDBsum:2PYW ProteinModelPortal:Q9C6D2 SMR:Q9C6D2 STRING:Q9C6D2 PRIDE:Q9C6D2 EnsemblPlants:AT1G49820.1 GeneID:841405 KEGG:ath:AT1G49820 TAIR:At1g49820 eggNOG:COG4857 HOGENOM:HBG305135 InParanoid:Q9C6D2 OMA:REIGDGN PhylomeDB:Q9C6D2 ProtClustDB:PLN02756 Genevestigator:Q9C6D2 GO:GO:0046522 TIGRFAMs:TIGR01767 Uniprot:Q9C6D2
Root	Isotig04855	0	35	-6.258	3.45E-10	UNIPROTKB|P29717 - symbol:XOG1 "Glucan 1,3-beta-glucosidase" species:237561 "Candida albicans SC5314" [GO:0004338 "glucan exo-1,3-beta-glucosidase activity" evidence=IMP;IDA] [GO:0005576 "extracellular region" evidence=IDA] [GO:0006073 "cellular glucan metabolic process" evidence=IMP] [GO:0009986 "cell surface" evidence=IDA] [GO:0031505 "fungal-type cell wall organization" evidence=IMP] [GO:0031589 "cell-substrate adhesion" evidence=IDA] [GO:0050839 "cell adhesion molecule binding" evidence=IDA] InterPro:IPR001547 InterPro:IPR013781 InterPro:IPR018087 Pfam:PF00150 PROSITE:PS00659 GO:GO:0005576 EMBL:AACQ01000015 GO:GO:0009986 GO:GO:0043169 InterPro:IPR017853 Gene3D:G3DSA:3.20.20.80 SUPFAM:SSF51445 CAZy:GH5 GO:GO:0031505 GO:GO:0050839 GO:GO:0031589 EMBL:AL033497 KO:K01210 GO:GO:0004338 eggNOG:COG2730 GO:GO:0006073 EMBL:X56556 PIR:A47702 PIR:T52149 RefSeq:XP_721488.1 PDB:1CZ1 PDB:1EQC PDB:1EQP PDB:2PB1 PDB:2PBO PDB:2PC8 PDB:2PF0 PDB:3N9K PDB:3O6A PDBsum:1CZ1 PDBsum:1EQC PDBsum:1EQP PDBsum:2PB1 PDBsum:2PBO PDBsum:2PC8 PDBsum:2PF0 PDBsum:3N9K PDBsum:3O6A ProteinModelPortal:P29717 SMR:P29717 STRING:P29717 GeneID:3636837 KEGG:cal:CaO19.2990 PhylomeDB:P29717 PMAP-CutDB:P29717 Uniprot:P29717
Root	Isotig04856	0	35	-6.258	3.45E-10	UNIPROTKB|P29717 - symbol:XOG1 "Glucan 1,3-beta-glucosidase" species:237561 "Candida albicans SC5314" [GO:0004338 "glucan exo-1,3-beta-glucosidase activity" evidence=IMP;IDA] [GO:0005576 "extracellular region" evidence=IDA] [GO:0006073 "cellular glucan metabolic process" evidence=IMP] [GO:0009986 "cell surface" evidence=IDA] [GO:0031505 "fungal-type cell wall organization" evidence=IMP] [GO:0031589 "cell-substrate adhesion" evidence=IDA] [GO:0050839 "cell adhesion molecule binding" evidence=IDA] InterPro:IPR001547 InterPro:IPR013781 InterPro:IPR018087 Pfam:PF00150 PROSITE:PS00659 GO:GO:0005576 EMBL:AACQ01000015 GO:GO:0009986 GO:GO:0043169 InterPro:IPR017853 Gene3D:G3DSA:3.20.20.80 SUPFAM:SSF51445 CAZy:GH5 GO:GO:0031505 GO:GO:0050839 GO:GO:0031589 EMBL:AL033497 KO:K01210 GO:GO:0004338 eggNOG:COG2730 GO:GO:0006073 EMBL:X56556 PIR:A47702 PIR:T52149 RefSeq:XP_721488.1 PDB:1CZ1 PDB:1EQC PDB:1EQP PDB:2PB1 PDB:2PBO PDB:2PC8 PDB:2PF0 PDB:3N9K PDB:3O6A PDBsum:1CZ1 PDBsum:1EQC PDBsum:1EQP PDBsum:2PB1 PDBsum:2PBO PDBsum:2PC8 PDBsum:2PF0 PDBsum:3N9K PDBsum:3O6A ProteinModelPortal:P29717 SMR:P29717 STRING:P29717 GeneID:3636837 KEGG:cal:CaO19.2990 PhylomeDB:P29717 PMAP-CutDB:P29717 Uniprot:P29717
Root	Isotig04857	0	15	-5.036	4.38E-05	No hit
Root	Isotig04879	30	6	2.193	8.18E-05	TAIR|locus:2009650 - symbol:DJ1B "AT1G53280" species:3702 "Arabidopsis thaliana" [GO:0008150 "biological_process" evidence=ND] [GO:0009507 "chloroplast" evidence=IDA] [GO:0009570 "chloroplast stroma" evidence=IDA] INTERPRO:IPR002818 EMBL:CP002684 GO:GO:0009570 EMBL:AC008007 UniGene:At.66897 Pfam:PF01965 HSSP:Q99497 InterPro:IPR006287 TIGRFAMs:TIGR01383 KO:K03152 ProtClustDB:CLSN2685087 EMBL:AY056268 EMBL:AY091184 IPI:IPI00532024 RefSeq:NP_564626.1 UniGene:At.24375 ProteinModelPortal:Q9MAH3 SMR:Q9MAH3 IntAct:Q9MAH3 PRIDE:Q9MAH3 EnsemblPlants:AT1G53280.1

Tissue	Identification	# of reads (stress)	# of reads (control)	Fold Change	p-value	Description
						GeneID:841762 KEGG:ath:AT1G53280 TAIR:At1g53280 InParanoid:Q9MAH3 OMA:MIIDILR PhylomeDB:Q9MAH3 ArrayExpress:Q9MAH3 Genevestigator:Q9MAH3 Uniprot:Q9MAH3
Root	Isotig04880	29	7	1.922	0.000375107	TAIR|locus:2009650 - symbol:DJ1B "AT1G53280" species:3702 "Arabidopsis thaliana" [GO:0008150 "biological_process" evidence=ND] [GO:0009507 "chloroplast" evidence=IDA] [GO:0009570 "chloroplast stroma" evidence=IDA] INTERPRO:IPR002818 EMBL:CP002684 GO:GO:0009570 EMBL:AC008007 UniGene:At.66897 Pfam:PF01965 HSSP:Q99497 InterPro:IPR006287 TIGRFAMs:TIGR01383 KO:K03152 ProtClustDB:CLSN2685087 EMBL:AY056268 EMBL:AY091184 IPI:IPI00532024 RefSeq:NP_564626.1 UniGene:At.24375 ProteinModelPortal:Q9MAH3 SMR:Q9MAH3 IntAct:Q9MAH3 PRIDE:Q9MAH3 EnsemblPlants:AT1G53280.1 GeneID:841762 KEGG:ath:AT1G53280 TAIR:At1g53280 InParanoid:Q9MAH3 OMA:MIIDILR PhylomeDB:Q9MAH3 ArrayExpress:Q9MAH3 Genevestigator:Q9MAH3 Uniprot:Q9MAH3
Root	Isotig04881	29	6	2.144	0.000134201	TAIR|locus:2009650 - symbol:DJ1B "AT1G53280" species:3702 "Arabidopsis thaliana" [GO:0008150 "biological_process" evidence=ND] [GO:0009507 "chloroplast" evidence=IDA] [GO:0009570 "chloroplast stroma" evidence=IDA] INTERPRO:IPR002818 EMBL:CP002684 GO:GO:0009570 EMBL:AC008007 UniGene:At.66897 Pfam:PF01965 HSSP:Q99497 InterPro:IPR006287 TIGRFAMs:TIGR01383 KO:K03152 ProtClustDB:CLSN2685087 EMBL:AY056268 EMBL:AY091184 IPI:IPI00532024 RefSeq:NP_564626.1 UniGene:At.24375 ProteinModelPortal:Q9MAH3 SMR:Q9MAH3 IntAct:Q9MAH3 PRIDE:Q9MAH3 EnsemblPlants:AT1G53280.1 GeneID:841762 KEGG:ath:AT1G53280 TAIR:At1g53280 InParanoid:Q9MAH3 OMA:MIIDILR PhylomeDB:Q9MAH3 ArrayExpress:Q9MAH3 Genevestigator:Q9MAH3 Uniprot:Q9MAH3
Root	Isotig04890	7	24	-1.907	0.000762598	TAIR|locus:2172214 - symbol:TUB3 "tubulin beta chain 3" species:3702 "Arabidopsis thaliana" [GO:0003924 "GTPase activity" evidence=IEA] [GO:0005198 "structural molecule activity" evidence=IEA;ISS] [GO:0005525 "GTP binding" evidence=IEA] [GO:0045298 "tubulin complex" evidence=TAS] [GO:0005618 "cell wall" evidence=IDA] [GO:0009506 "plasmodesma" evidence=IDA] [GO:0005794 "Golgi apparatus" evidence=IDA] InterPro:IPR000217 InterPro:IPR002453 InterPro:IPR003008 InterPro:IPR008280 InterPro:IPR017975 InterPro:IPR018316 Pfam:PF00091 Pfam:PF03953 PRINTS:PR01161 PRINTS:PR01163 PROSITE:PS00227 SMART:SM00864 SMART:SM00865 GO:GO:0005525 GO:GO:0005886 GO:GO:0009506 GO:GO:0046686 GO:GO:0005737 EMBL:CP002688 GenomeReviews:BA000015_GR GO:GO:0005618 GO:GO:0005515 GO:GO:0005730 GO:GO:0005198 GO:GO:0003924 GO:GO:0009651 GO:GO:0005874 GO:GO:0051258 Gene3D:G3DSA:3.40.50.1440 SUPFAM:SSF52490 GO:GO:0007018 EMBL:AB009053 EMBL:AB020751 Gene3D:G3DSA:3.30.1330.20 SUPFAM:SSF55307 GO:GO:0045298 eggNOG:COG5023 HOGENOM:HBG750007 InterPro:IPR023123 Gene3D:G3DSA:1.10.287.600 PANTHER:PTHR11588 InterPro:IPR013838 KO:K07375 ProtClustDB:PLN00220 PROSITE:PS00228 OMA:ADEMGEY EMBL:M84700 EMBL:M84701 EMBL:AK117431 EMBL:AY056411 EMBL:AY060550 EMBL:AY062614 EMBL:AY062742 EMBL:AY128785 EMBL:BT002587 EMBL:AY087859 IPI:IPI00525001 PIR:JQ1587 RefSeq:NP_568959.1 RefSeq:NP_568960.1 UniGene:At.24202 UniGene:At.49208 ProteinModelPortal:P29512 SMR:P29512 IntAct:P29512 STRING:P29512 PRIDE:P29512 EnsemblPlants:AT5G62690.1 EnsemblPlants:AT5G62700.1 GeneID:836390 GeneID:836391 KEGG:ath:AT5G62690 KEGG:ath:AT5G62700 TAIR:At5g62690 TAIR:At5g62700 InParanoid:P29512 PhylomeDB:P29512 ArrayExpress:P29512 Genevestigator:P29512 GermOnline:AT5G62690 Uniprot:P29512
Root	Isotig04894	5	21	-2.199	0.000555521	TAIR|locus:2175088 - symbol:ALP "AT5G60360" species:3702 "Arabidopsis thaliana" [GO:0006508 "proteolysis" evidence=ISS] [GO:0008234 "cysteine-type peptidase activity" evidence=ISS] [GO:0009723 "response to ethylene stimulus" evidence=IEP] [GO:0005773 "vacuole" evidence=IDA] [GO:0005829 "cytosol" evidence=RCA] [GO:0007568 "aging" evidence=IEP] InterPro:IPR000668 InterPro:IPR013128 Pfam:PF00112 PRINTS:PR00705 SMART:SM00645 InterPro:IPR000169 GO:GO:0005773 EMBL:CP002688 GenomeReviews:BA000015_GR GO:GO:0007568 eggNOG:COG4870 HOGENOM:HBG746690 GO:GO:0004197 GO:GO:0006508 InterPro:IPR013201 PANTHER:PTHR12411 Pfam:PF08246 SMART:SM00848 PROSITE:PS00640 PROSITE:PS00139 PROSITE:PS00639 EMBL:AB011483 KO:K01366 ProtClustDB:CLSN2689015 EMBL:AF233883 EMBL:AF083703 EMBL:AF360273 EMBL:BT000673 EMBL:BT000674 EMBL:BT000676 EMBL:AY088662 IPI:IPI00537494 PIR:PQ0650 RefSeq:NP_568921.1 UniGene:At.25414 UniGene:At.73165 ProteinModelPortal:Q8H166 SMR:Q8H166 IntAct:Q8H166 STRING:Q8H166 MEROPS:C01.A08 PRIDE:Q8H166 EnsemblPlants:AT5G60360.1 GeneID:836158 KEGG:ath:AT5G60360 GeneFarm:5031 TAIR:At5g60360 InParanoid:Q8H166 OMA:CGSTPMD PhylomeDB:Q8H166 Genevestigator:Q8H166 Uniprot:Q8H166
Root	Isotig04908	5	24	-2.392	0.000103464	TAIR|locus:2035619 - symbol:AT1G32780 species:3702 "Arabidopsis thaliana" [GO:0008270 "zinc ion binding" evidence=IEA] [GO:0016491 "oxidoreductase activity" evidence=IEA] [GO:0055114 "oxidation-reduction process"

Tissue	Identification	# of reads (stress)	# of reads (control)	Fold Change	p-value	Description
						evidence=IEA] InterPro:IPR002085 InterPro:IPR002328 InterPro:IPR013149 InterPro:IPR013154 InterPro:IPR016040 Pfam:PF00107 Pfam:PF08240 PROSITE:PS00059 EMBL:CP002684 GenomeReviews:CT485782_GR GO:GO:0005737 GO:GO:0000166 Gene3D:G3DSA:3.40.50.720 GO:GO:0008270 HOGENOM:HBG753318 InterPro:IPR011032 PANTHER:PTHR11695 SUPFAM:SSF50129 eggNOG:COG1062 GO:GO:0004022 KO:K00001 EMBL:AC017118 EMBL:BT029769 EMBL:AY087044 IPI:IPI00541581 RefSeq:NP_564409.1 UniGene:At.40067 ProteinModelPortal:A1L4Y2 SMR:A1L4Y2 STRING:A1L4Y2 PRIDE:A1L4Y2 EnsemblPlants:AT1G32780.1 GeneID:840172 KEGG:ath:AT1G32780 TAIR:At1g32780 InParanoid:A1L4Y2 OMA:TCKAAVV PhylomeDB:A1L4Y2 ProtClustDB:PLN02740 Genevestigator:A1L4Y2 Uniprot:A1L4Y2
Root	Isotig04909	5	26	-2.507	3.30E-05	TAIR|locus:2035619 - symbol:AT1G32780 species:3702 "Arabidopsis thaliana" [GO:0008270 "zinc ion binding" evidence=IEA] [GO:0016491 "oxidoreductase activity" evidence=IEA] [GO:0055114 "oxidation-reduction process" evidence=IEA] InterPro:IPR002085 InterPro:IPR002328 InterPro:IPR013149 InterPro:IPR013154 InterPro:IPR016040 Pfam:PF00107 Pfam:PF08240 PROSITE:PS00059 EMBL:CP002684 GenomeReviews:CT485782_GR GO:GO:0005737 GO:GO:0000166 Gene3D:G3DSA:3.40.50.720 GO:GO:0008270 HOGENOM:HBG753318 InterPro:IPR011032 PANTHER:PTHR11695 SUPFAM:SSF50129 eggNOG:COG1062 GO:GO:0004022 KO:K00001 EMBL:AC017118 EMBL:BT029769 EMBL:AY087044 IPI:IPI00541581 RefSeq:NP_564409.1 UniGene:At.40067 ProteinModelPortal:A1L4Y2 SMR:A1L4Y2 STRING:A1L4Y2 PRIDE:A1L4Y2 EnsemblPlants:AT1G32780.1 GeneID:840172 KEGG:ath:AT1G32780 TAIR:At1g32780 InParanoid:A1L4Y2 OMA:TCKAAVV PhylomeDB:A1L4Y2 ProtClustDB:PLN02740 Genevestigator:A1L4Y2 Uniprot:A1L4Y2
Root	Isotig04910	5	26	-2.507	3.30E-05	TAIR|locus:2035619 - symbol:AT1G32780 species:3702 "Arabidopsis thaliana" [GO:0008270 "zinc ion binding" evidence=IEA] [GO:0016491 "oxidoreductase activity" evidence=IEA] [GO:0055114 "oxidation-reduction process" evidence=IEA] InterPro:IPR002085 InterPro:IPR002328 InterPro:IPR013149 InterPro:IPR013154 InterPro:IPR016040 Pfam:PF00107 Pfam:PF08240 PROSITE:PS00059 EMBL:CP002684 GenomeReviews:CT485782_GR GO:GO:0005737 GO:GO:0000166 Gene3D:G3DSA:3.40.50.720 GO:GO:0008270 HOGENOM:HBG753318 InterPro:IPR011032 PANTHER:PTHR11695 SUPFAM:SSF50129 eggNOG:COG1062 GO:GO:0004022 KO:K00001 EMBL:AC017118 EMBL:BT029769 EMBL:AY087044 IPI:IPI00541581 RefSeq:NP_564409.1 UniGene:At.40067 ProteinModelPortal:A1L4Y2 SMR:A1L4Y2 STRING:A1L4Y2 PRIDE:A1L4Y2 EnsemblPlants:AT1G32780.1 GeneID:840172 KEGG:ath:AT1G32780 TAIR:At1g32780 InParanoid:A1L4Y2 OMA:TCKAAVV PhylomeDB:A1L4Y2 ProtClustDB:PLN02740 Genevestigator:A1L4Y2 Uniprot:A1L4Y2
Root	Isotig04915	9	27	-1.714	0.000905257	TAIR|locus:505006345 - symbol:AT3G14205 "AT3G14205" species:3702 "Arabidopsis thaliana" [GO:0004439 "phosphatidylinositol-4,5-bisphosphate 5-phosphatase activity" evidence=ISS] [GO:0008150 "biological_process" evidence=ND] InterPro:IPR002013 Pfam:PF02383 PROSITE:PS50275 EMBL:CP002686 GO:GO:0042578 OMA:IESEICV EMBL:AY050432 EMBL:AY093999 EMBL:AY227245 IPI:IPI00525545 RefSeq:NP_566481.1 UniGene:At.26576 ProteinModelPortal:Q94A27 SMR:Q94A27 PRIDE:Q94A27 EnsemblPlants:AT3G14205.1 GeneID:820638 KEGG:ath:AT3G14205 TAIR:At3g14205 InParanoid:Q94A27 PhylomeDB:Q94A27 ProtClustDB:CLSN2914997 Genevestigator:Q94A27 Uniprot:Q94A27
Root	Isotig04920	29	3	3.144	2.49E-06	TAIR|locus:2121363 - symbol:AAC3 "AT4G28390" species:3702 "Arabidopsis thaliana" [GO:0005471 "ATP:ADP antiporter activity" evidence=ISS;IDA] [GO:0005743 "mitochondrial inner membrane" evidence=ISS] [GO:0006810 "transport" evidence=ISS] [GO:0006839 "mitochondrial transport" evidence=ISS] [GO:0015865 "purine nucleotide transport" evidence=IDA] [GO:0016020 "membrane" evidence=IDA] [GO:0009941 "chloroplast envelope" evidence=IDA] [GO:0005739 "mitochondrion" evidence=IDA] InterPro:IPR002067 InterPro:IPR002113 PRINTS:PR00926 PRINTS:PR00927 Pfam:PF00153 GO:GO:0016021 EMBL:CP002687 GenomeReviews:CT486007_GR GO:GO:0005488 GO:GO:0005743 GO:GO:0009941 eggNOG:NOG238123 HOGENOM:HBG610399 KO:K05863 ProtClustDB:CLSN2684949 GO:GO:0005471 GO:GO:0015865 InterPro:IPR018108 InterPro:IPR023395 Gene3D:G3DSA:1.50.40.10 SUPFAM:SSF103506 PROSITE:PS50920 OMA:QREFNGL EMBL:AL021749 EMBL:AL161572 EMBL:AK118017 EMBL:AY088151 IPI:IPI00517177 PIR:T04608 RefSeq:NP_194568.1 UniGene:At.44823 UniGene:At.69694 HSSP:P02722 ProteinModelPortal:O49447 SMR:O49447 STRING:O49447 PRIDE:O49447 GeneID:828955 KEGG:ath:AT4G28390 GeneFarm:3748 TAIR:At4g28390 InParanoid:O49447 PhylomeDB:O49447 ArrayExpress:O49447 Genevestigator:O49447 Uniprot:O49447

Tissue	Identification	# of reads (stress)	# of reads (control)	Fold Change	p-value	Description
Root	Isotig04921	26	3	2.987	1.35E-05	TAIR|locus:2121363 - symbol:AAC3 "AT4G28390" species:3702 "Arabidopsis thaliana" [GO:0005471 "ATP:ADP antiporter activity" evidence=ISS;IDA] [GO:0005743 "mitochondrial inner membrane" evidence=ISS] [GO:0006810 "transport" evidence=ISS] [GO:0006839 "mitochondrial transport" evidence=ISS] [GO:0015865 "purine nucleotide transport" evidence=IDA] [GO:0016020 "membrane" evidence=IDA] [GO:0009941 "chloroplast envelope" evidence=IDA] [GO:0005739 "mitochondrion" evidence=IDA] InterPro:IPR002067 InterPro:IPR002113 PRINTS:PR00926 PRINTS:PR00927 Pfam:PF00153 GO:GO:0016021 EMBL:CP002687 GenomeReviews:CT486007_GR GO:GO:0005488 GO:GO:0005743 GO:GO:0009941 eggNOG:NOG238123 HOGENOM:HBG610399 KO:K05863 ProtClustDB:CLSN2684949 GO:GO:0005471 GO:GO:0015865 InterPro:IPR018108 InterPro:IPR023395 Gene3D:G3DSA:1.50.40.10 SUPFAM:SSF103506 PROSITE:PS50920 OMA:QREFNGL EMBL:AL021749 EMBL:AL161572 EMBL:AK118017 EMBL:AY088151 IPI:IPI00517177 PIR:T04608 RefSeq:NP_194568.1 UniGene:At.44823 UniGene:At.69694 HSSP:P02722 ProteinModelPortal:O49447 SMR:O49447 STRING:O49447 PRIDE:O49447 GeneID:828955 KEGG:ath:AT4G28390 GeneFarm:3748 TAIR:At4g28390 InParanoid:O49447 PhylomeDB:O49447 ArrayExpress:O49447 Genevestigator:O49447 Uniprot:O49447
Root	Isotig04922	27	3	3.041	7.69E-06	TAIR|locus:2121363 - symbol:AAC3 "AT4G28390" species:3702 "Arabidopsis thaliana" [GO:0005471 "ATP:ADP antiporter activity" evidence=ISS;IDA] [GO:0005743 "mitochondrial inner membrane" evidence=ISS] [GO:0006810 "transport" evidence=ISS] [GO:0006839 "mitochondrial transport" evidence=ISS] [GO:0015865 "purine nucleotide transport" evidence=IDA] [GO:0016020 "membrane" evidence=IDA] [GO:0009941 "chloroplast envelope" evidence=IDA] [GO:0005739 "mitochondrion" evidence=IDA] InterPro:IPR002067 InterPro:IPR002113 PRINTS:PR00926 PRINTS:PR00927 Pfam:PF00153 GO:GO:0016021 EMBL:CP002687 GenomeReviews:CT486007_GR GO:GO:0005488 GO:GO:0005743 GO:GO:0009941 eggNOG:NOG238123 HOGENOM:HBG610399 KO:K05863 ProtClustDB:CLSN2684949 GO:GO:0005471 GO:GO:0015865 InterPro:IPR018108 InterPro:IPR023395 Gene3D:G3DSA:1.50.40.10 SUPFAM:SSF103506 PROSITE:PS50920 OMA:QREFNGL EMBL:AL021749 EMBL:AL161572 EMBL:AK118017 EMBL:AY088151 IPI:IPI00517177 PIR:T04608 RefSeq:NP_194568.1 UniGene:At.44823 UniGene:At.69694 HSSP:P02722 ProteinModelPortal:O49447 SMR:O49447 STRING:O49447 PRIDE:O49447 GeneID:828955 KEGG:ath:AT4G28390 GeneFarm:3748 TAIR:At4g28390 InParanoid:O49447 PhylomeDB:O49447 ArrayExpress:O49447 Genevestigator:O49447 Uniprot:O49447
Root	Isotig04923	37	2	4.080	4.39E-09	TAIR|locus:2027317 - symbol:AT1G11820 species:3702 "Arabidopsis thaliana" [GO:0003824 "catalytic activity" evidence=IEA] [GO:0004553 "hydrolase activity, hydrolyzing O-glycosyl compounds" evidence=IEA;ISS] [GO:0005975 "carbohydrate metabolic process" evidence=IEA] [GO:0043169 "cation binding" evidence=IEA] InterPro:IPR000490 InterPro:IPR013781 Pfam:PF00332 PROSITE:PS00587 EMBL:CP002684 GenomeReviews:CT485782_GR GO:GO:0005886 GO:GO:0006952 GO:GO:0043169 InterPro:IPR017853 Gene3D:G3DSA:3.20.20.80 SUPFAM:SSF51445 GO:GO:0005975 GO:GO:0031225 EMBL:AC002131 EMBL:AC007296 CAZy:GH17 GO:GO:0042973 HOGENOM:HBG605194 CAZy:CBM43 InterPro:IPR012946 Pfam:PF07983 SMART:SM00768 EMBL:BT004271 IPI:IPI00548319 PIR:E86252 RefSeq:NP_001184967.1 UniGene:At.10643 ProteinModelPortal:O65399 SMR:O65399 PRIDE:O65399 GeneID:837730 KEGG:ath:AT1G11820 TAIR:At1g11820 eggNOG:NOG329755 InParanoid:O65399 PhylomeDB:O65399 Genevestigator:O65399 Uniprot:O65399
Root	Isotig04924	41	2	4.229	4.38E-10	TAIR|locus:2092855 - symbol:AT3G13560 species:3702 "Arabidopsis thaliana" [GO:0003824 "catalytic activity" evidence=IEA] [GO:0004553 "hydrolase activity, hydrolyzing O-glycosyl compounds" evidence=IEA;ISS] [GO:0005975 "carbohydrate metabolic process" evidence=IEA] [GO:0043169 "cation binding" evidence=IEA] [GO:0031225 "anchored to membrane" evidence=TAS] [GO:0046658 "anchored to plasma membrane" evidence=IDA] [GO:0005886 "plasma membrane" evidence=IDA] InterPro:IPR000490 InterPro:IPR013781 Pfam:PF00332 PROSITE:PS00587 EMBL:CP002686 GenomeReviews:BA000014_GR GO:GO:0006952 GO:GO:0043169 InterPro:IPR017853 Gene3D:G3DSA:3.20.20.80 SUPFAM:SSF51445 GO:GO:0005975 EMBL:AP000603 GO:GO:0046658 CAZy:GH17 GO:GO:0042973 HOGENOM:HBG605194 CAZy:CBM43 InterPro:IPR012946 Pfam:PF07983 SMART:SM00768 EMBL:AY034940 EMBL:AY063117 IPI:IPI00537250 RefSeq:NP_187965.1 RefSeq:NP_974302.1 RefSeq:NP_974303.1 UniGene:At.8269 ProteinModelPortal:Q94CD8 SMR:Q94CD8 STRING:Q94CD8 PRIDE:Q94CD8 EnsemblPlants:AT3G13560.1 EnsemblPlants:AT3G13560.2 EnsemblPlants:AT3G13560.3 GeneID:820558 KEGG:ath:AT3G13560 TAIR:At3g13560 eggNOG:NOG310730

Tissue	Identification	# of reads (stress)	# of reads (control)	Fold Change	p-value	Description
						InParanoid:Q94CD8 OMA:IQPGQPC PhylomeDB:Q94CD8 ProtClustDB:CLSN2684782 ArrayExpress:Q94CD8 Genevestigator:Q94CD8 GermOnline:AT3G13560 Uniprot:Q94CD8
Root	Isotig04926	2	16	-3.129	0.000262867	TAIR|locus:2166086 - symbol:sks3 "SKU5 similar 3" species:3702 "Arabidopsis thaliana" [GO:0005507 "copper ion binding" evidence=IEA;ISS] [GO:0016491 "oxidoreductase activity" evidence=IEA] [GO:0055114 "oxidation-reduction process" evidence=IEA] [GO:0005886 "plasma membrane" evidence=IDA] [GO:0009506 "plasmodesma" evidence=IDA] InterPro:IPR001117 InterPro:IPR011706 InterPro:IPR011707 Pfam:PF00394 Pfam:PF07731 Pfam:PF07732 GO:GO:0005886 GO:GO:0009506 GenomeReviews:BA000015_GR GO:GO:0016491 GO:GO:0005507 InterPro:IPR008972 Gene3D:G3DSA:2.60.40.420 SUPFAM:SSF49503 EMBL:AB020745 eggNOG:COG2132 HOGENOM:HBG316961 IPI:IPI00536545 ProteinModelPortal:Q9LV68 SMR:Q9LV68 PRIDE:Q9LV68 TAIR:At5g48450 InParanoid:Q9LV68 PhylomeDB:Q9LV68 Genevestigator:Q9LV68 Uniprot:Q9LV68
Root	Isotig04932	81	0	7.211	4.65E-19	TAIR|locus:2040646 - symbol:ChlAKR "AT2G37770" species:3702 "Arabidopsis thaliana" [GO:0004033 "aldo-keto reductase (NADP) activity" evidence=IDA] [GO:0009409 "response to cold" evidence=IEP] [GO:0009414 "response to water deprivation" evidence=IEP] [GO:0009651 "response to salt stress" evidence=IEP] [GO:0016229 "steroid dehydrogenase activity" evidence=IDA] [GO:0070401 "NADP+ binding" evidence=IDA] [GO:0008106 "alcohol dehydrogenase (NADP+) activity" evidence=IDA] [GO:0009507 "chloroplast" evidence=IDA] [GO:0055114 "oxidation-reduction process" evidence=IDA] InterPro:IPR018170 InterPro:IPR020471 PIRSF:PIRSF000097 PRINTS:PR00069 PROSITE:PS00062 PROSITE:PS00063 PROSITE:PS00798 InterPro:IPR001395 EMBL:AC004684 Pfam:PF00248 GO:GO:0009507 EMBL:CP002685 GenomeReviews:CT485783_GR eggNOG:COG0656 GO:GO:0008106 InterPro:IPR023210 Gene3D:G3DSA:3.20.20.100 PANTHER:PTHR11732 SUPFAM:SSF51430 HOGENOM:HBG605727 GO:GO:0009409 GO:GO:0009651 GO:GO:0009414 GO:GO:0009636 GO:GO:0070401 GO:GO:0016229 EMBL:DQ837654 EMBL:BX820913 EMBL:BT004098 IPI:IPI00541607 IPI:IPI00657326 PIR:T02543 RefSeq:NP_001031505.1 RefSeq:NP_181313.3 UniGene:At.12803 PDB:3H7U PDBsum:3H7U ProteinModelPortal:Q0PGJ6 SMR:Q0PGJ6 STRING:Q0PGJ6 PRIDE:Q0PGJ6 EnsemblPlants:AT2G37770.2 GeneID:818354 KEGG:ath:AT2G37770 TAIR:At2g37770 InParanoid:Q2V420 OMA:VDIPSTW PhylomeDB:Q0PGJ6 ProtClustDB:CLSN2681439 BioCyc:MetaCyc:AT2G37770-MONOMER Genevestigator:Q0PGJ6 Uniprot:Q0PGJ6
Root	Isotig04933	78	0	7.156	1.79E-18	TAIR|locus:2040646 - symbol:ChlAKR "AT2G37770" species:3702 "Arabidopsis thaliana" [GO:0004033 "aldo-keto reductase (NADP) activity" evidence=IDA] [GO:0009409 "response to cold" evidence=IEP] [GO:0009414 "response to water deprivation" evidence=IEP] [GO:0009651 "response to salt stress" evidence=IEP] [GO:0016229 "steroid dehydrogenase activity" evidence=IDA] [GO:0070401 "NADP+ binding" evidence=IDA] [GO:0008106 "alcohol dehydrogenase (NADP+) activity" evidence=IDA] [GO:0009507 "chloroplast" evidence=IDA] [GO:0055114 "oxidation-reduction process" evidence=IDA] InterPro:IPR018170 InterPro:IPR020471 PIRSF:PIRSF000097 PRINTS:PR00069 PROSITE:PS00062 PROSITE:PS00063 PROSITE:PS00798 InterPro:IPR001395 EMBL:AC004684 Pfam:PF00248 GO:GO:0009507 EMBL:CP002685 GenomeReviews:CT485783_GR eggNOG:COG0656 GO:GO:0008106 InterPro:IPR023210 Gene3D:G3DSA:3.20.20.100 PANTHER:PTHR11732 SUPFAM:SSF51430 HOGENOM:HBG605727 GO:GO:0009409 GO:GO:0009651 GO:GO:0009414 GO:GO:0009636 GO:GO:0070401 GO:GO:0016229 EMBL:DQ837654 EMBL:BX820913 EMBL:BT004098 IPI:IPI00541607 IPI:IPI00657326 PIR:T02543 RefSeq:NP_001031505.1 RefSeq:NP_181313.3 UniGene:At.12803 PDB:3H7U PDBsum:3H7U ProteinModelPortal:Q0PGJ6 SMR:Q0PGJ6 STRING:Q0PGJ6 PRIDE:Q0PGJ6 EnsemblPlants:AT2G37770.2 GeneID:818354 KEGG:ath:AT2G37770 TAIR:At2g37770 InParanoid:Q2V420 OMA:VDIPSTW PhylomeDB:Q0PGJ6 ProtClustDB:CLSN2681439 BioCyc:MetaCyc:AT2G37770-MONOMER Genevestigator:Q0PGJ6 Uniprot:Q0PGJ6
Root	Isotig04934	81	0	7.211	4.65E-19	TAIR|locus:2040646 - symbol:ChlAKR "AT2G37770" species:3702 "Arabidopsis thaliana" [GO:0004033 "aldo-keto reductase (NADP) activity" evidence=IDA] [GO:0009409 "response to cold" evidence=IEP] [GO:0009414 "response to water deprivation" evidence=IEP] [GO:0009651 "response to salt stress" evidence=IEP] [GO:0016229 "steroid dehydrogenase activity" evidence=IDA] [GO:0070401 "NADP+ binding" evidence=IDA] [GO:0008106 "alcohol dehydrogenase (NADP+) activity" evidence=IDA] [GO:0009507 "chloroplast" evidence=IDA] [GO:0055114 "oxidation-reduction process" evidence=IDA] InterPro:IPR018170 InterPro:IPR020471 PIRSF:PIRSF000097 PRINTS:PR00069 PROSITE:PS00062 PROSITE:PS00063 PROSITE:PS00798 InterPro:IPR001395 EMBL:AC004684 Pfam:PF00248 GO:GO:0009507 EMBL:CP002685 GenomeReviews:CT485783_GR eggNOG:COG0656 GO:GO:0008106 InterPro:IPR023210 Gene3D:G3DSA:3.20.20.100 PANTHER:PTHR11732

Tissue	Identification	# of reads (stress)	# of reads (control)	Fold Change	p-value	Description
						SUPFAM:SSF51430 HOGENOM:HBG605727 GO:GO:0009409 GO:GO:0009651 GO:GO:0009414 GO:GO:0009636 GO:GO:0070401 GO:GO:0016229 EMBL:DQ837654 EMBL:BX820913 EMBL:BT004098 IPI:IPI00541607 IPI:IPI00657326 PIR:T02543 RefSeq:NP_001031505.1 RefSeq:NP_181313.3 UniGene:At.12803 PDB:3H7U PDBsum:3H7U ProteinModelPortal:Q0PGJ6 SMR:Q0PGJ6 STRING:Q0PGJ6 PRIDE:Q0PGJ6 EnsemblPlants:AT2G37770.2 GeneID:818354 KEGG:ath:AT2G37770 TAIR:At2g37770 InParanoid:Q2V420 OMA:VDIPSTW PhylomeDB:Q0PGJ6 ProtClustDB:CLSN2681439 BioCyc:MetaCyc:AT2G37770-MONOMER Genevestigator:Q0PGJ6 Uniprot:Q0PGJ6
Root	Isotig04953	5	21	-2.199	0.000555521	TAIR|locus:2025687 - symbol:WRKY40 "AT1G80840" species:3702 "Arabidopsis thaliana" [GO:0003700 "sequence-specific DNA binding transcription factor activity" evidence=ISS;IDA] [GO:0006355 "regulation of transcription, DNA-dependent" evidence=ISS] [GO:0031347 "regulation of defense response" evidence=IMP] [GO:0009751 "response to salicylic acid stimulus" evidence=IEP] [GO:0042742 "defense response to bacterium" evidence=IEP] [GO:0005634 "nucleus" evidence=IDA] [GO:0050832 "defense response to fungus" evidence=IEP] [GO:0050691 "regulation of defense response to virus by host" evidence=IGI] [GO:0010200 "response to chitin" evidence=IEP] [GO:0009611 "response to wounding" evidence=IEP] [GO:0005515 "protein binding" evidence=IPI] [GO:0002237 "response to molecule of bacterial origin" evidence=IMP] InterPro:IPR003657 Pfam:PF03106 PROSITE:PS50811 SMART:SM00774 EMBL:CP002684 GenomeReviews:CT485782_GR GO:GO:0005634 GO:GO:0042742 GO:GO:0005515 GO:GO:0009611 GO:GO:0050832 GO:GO:0043565 GO:GO:0003700 GO:GO:0006351 GO:GO:0009751 GO:GO:0010200 EMBL:AC011713 GO:GO:0050691 Gene3D:G3DSA:2.20.25.80 SUPFAM:SSF118290 HOGENOM:HBG748409 EMBL:AF480149 EMBL:BT001914 IPI:IPI00522578 PIR:A96841 RefSeq:NP_178199.1 UniGene:At.28188 ProteinModelPortal:Q9SAH7 SMR:Q9SAH7 IntAct:Q9SAH7 STRING:Q9SAH7 PRIDE:Q9SAH7 EnsemblPlants:AT1G80840.1 GeneID:844423 KEGG:ath:AT1G80840 TAIR:At1g80840 eggNOG:NOG310042 InParanoid:Q9SAH7 OMA:SNITERD PhylomeDB:Q9SAH7 ProtClustDB:CLSN2914521 ArrayExpress:Q9SAH7 Genevestigator:Q9SAH7 GermOnline:AT1G80840 Uniprot:Q9SAH7
Root	Isotig04954	5	21	-2.199	0.000555521	TAIR|locus:2025687 - symbol:WRKY40 "AT1G80840" species:3702 "Arabidopsis thaliana" [GO:0003700 "sequence-specific DNA binding transcription factor activity" evidence=ISS;IDA] [GO:0006355 "regulation of transcription, DNA-dependent" evidence=ISS] [GO:0031347 "regulation of defense response" evidence=IMP] [GO:0009751 "response to salicylic acid stimulus" evidence=IEP] [GO:0042742 "defense response to bacterium" evidence=IEP] [GO:0005634 "nucleus" evidence=IDA] [GO:0050832 "defense response to fungus" evidence=IEP] [GO:0050691 "regulation of defense response to virus by host" evidence=IGI] [GO:0010200 "response to chitin" evidence=IEP] [GO:0009611 "response to wounding" evidence=IEP] [GO:0005515 "protein binding" evidence=IPI] [GO:0002237 "response to molecule of bacterial origin" evidence=IMP] InterPro:IPR003657 Pfam:PF03106 PROSITE:PS50811 SMART:SM00774 EMBL:CP002684 GenomeReviews:CT485782_GR GO:GO:0005634 GO:GO:0042742 GO:GO:0005515 GO:GO:0009611 GO:GO:0050832 GO:GO:0043565 GO:GO:0003700 GO:GO:0006351 GO:GO:0009751 GO:GO:0010200 EMBL:AC011713 GO:GO:0050691 Gene3D:G3DSA:2.20.25.80 SUPFAM:SSF118290 HOGENOM:HBG748409 EMBL:AF480149 EMBL:BT001914 IPI:IPI00522578 PIR:A96841 RefSeq:NP_178199.1 UniGene:At.28188 ProteinModelPortal:Q9SAH7 SMR:Q9SAH7 IntAct:Q9SAH7 STRING:Q9SAH7 PRIDE:Q9SAH7 EnsemblPlants:AT1G80840.1 GeneID:844423 KEGG:ath:AT1G80840 TAIR:At1g80840 eggNOG:NOG310042 InParanoid:Q9SAH7 OMA:SNITERD PhylomeDB:Q9SAH7 ProtClustDB:CLSN2914521 ArrayExpress:Q9SAH7 Genevestigator:Q9SAH7 GermOnline:AT1G80840 Uniprot:Q9SAH7
Root	Isotig04969	26	2	3.571	2.56E-06	TAIR|locus:2056700 - symbol:ARPN "plantacyanin" species:3702 "Arabidopsis thaliana" [GO:0005507 "copper ion binding" evidence=IEA;ISS] [GO:0009055 "electron carrier activity" evidence=IEA] [GO:0009856 "pollination" evidence=IMP] [GO:0048196 "plant extracellular matrix" evidence=TAS] [GO:0048653 "anther development" evidence=IMP] [GO:0048046 "apoplast" evidence=IDA] InterPro:IPR003245 Pfam:PF02298 ProDom:PD003122 PROSITE:PS00196 PROSITE:PS51485 EMBL:AC004138 GO:GO:0048046 EMBL:CP002685 GenomeReviews:CT485783_GR GO:GO:0009055 GO:GO:0006810 GO:GO:0005507 GO:GO:0022900 GO:GO:0048653 EMBL:AC002521 InterPro:IPR008972 Gene3D:G3DSA:2.60.40.420 SUPFAM:SSF49503 EMBL:U76297 EMBL:AF325063 EMBL:AY064141 EMBL:AY097407 EMBL:AY084407 IPI:IPI00540939 PIR:F84441 PIR:T00843 RefSeq:NP_178388.1 UniGene:At.23668 ProteinModelPortal:Q8LG89 SMR:Q8LG89 PRIDE:Q8LG89 ProMEX:Q8LG89 EnsemblPlants:AT2G02850.1 GeneID:814816 KEGG:ath:AT2G02850 TAIR:At2g02850 eggNOG:NOG271220 HOGENOM:HBG748847 InParanoid:Q8LG89 OMA:SWSARAI PhylomeDB:Q8LG89

Tissue	Identification	# of reads (stress)	# of reads (control)	Fold Change	p-value	Description
						ProtClustDB:CLSN2913208 ArrayExpress:Q8LG89 Genevestigator:Q8LG89 GermOnline:AT2G02850 GO:GO:0048196 GO:GO:0009856 Uniprot:Q8LG89
Root	Isotig04970	20	2	3.193	8.23E-05	TAIR|locus:2056700 - symbol:ARPN "plantacyanin" species:3702 "Arabidopsis thaliana" [GO:0005507 "copper ion binding" evidence=IEA;ISS] [GO:0009055 "electron carrier activity" evidence=IEA] [GO:0009856 "pollination" evidence=IMP] [GO:0048196 "plant extracellular matrix" evidence=TAS] [GO:0048653 "anther development" evidence=IMP] [GO:0048046 "apoplast" evidence=IDA] InterPro:IPR003245 Pfam:PF02298 ProDom:PD003122 PROSITE:PS00196 PROSITE:PS51485 EMBL:AC004138 GO:GO:0048046 EMBL:CP002685 GenomeReviews:CT485783_GR GO:GO:0009055 GO:GO:0006810 GO:GO:0005507 GO:GO:0022900 GO:GO:0048653 EMBL:AC002521 InterPro:IPR008972 Gene3D:G3DSA:2.60.40.420 SUPFAM:SSF49503 EMBL:U76297 EMBL:AF325063 EMBL:AY064141 EMBL:AY097407 EMBL:AY084407 IPI:IPI00540939 PIR:F84441 PIR:T00843 RefSeq:NP_178388.1 UniGene:At.23668 ProteinModelPortal:Q8LG89 SMR:Q8LG89 PRIDE:Q8LG89 ProMEX:Q8LG89 EnsemblPlants:AT2G02850.1 GeneID:814816 KEGG:ath:AT2G02850 TAIR:At2g02850 eggNOG:NOG271220 HOGENOM:HBG748847 InParanoid:Q8LG89 OMA:SWSARAI PhylomeDB:Q8LG89 ProtClustDB:CLSN2913208 ArrayExpress:Q8LG89 Genevestigator:Q8LG89 GermOnline:AT2G02850 GO:GO:0048196 GO:GO:0009856 Uniprot:Q8LG89
Root	Isotig04980	317	0	9.179	2.01E-57	TIGR_CMR|BA_1222 - symbol:BA_1222 "conserved hypothetical protein" species:198094 "Bacillus anthracis str. Ames" [GO:0003674 "molecular_function" evidence=ND] [GO:0005575 "cellular_component" evidence=ND] [GO:0008150 "biological_process" evidence=ND] EMBL:AE016879 EMBL:AE017334 GenomeReviews:AE016879_GR GenomeReviews:AE017334_GR InterPro:IPR008160 Pfam:PF01391 EMBL:AB281483 EMBL:AJ516936 RefSeq:NP_843695.1 RefSeq:YP_017836.1 PDB:2R6Q PDBsum:2R6Q ProteinModelPortal:Q81JD7 SMR:Q81JD7 IntAct:Q81JD7 EnsemblBacteria:EBBACT00000011819 EnsemblBacteria:EBBACT00000016984 GeneID:1084744 GeneID:2814858 KEGG:ban:BA_1222 KEGG:bar:GBAA_1222 PATRIC:18780046 TIGR:BA_1222 TIGR:GBAA1222 HOGENOM:HBG342646 OMA:SDGLNPD ProtClustDB:CLSK824721 Uniprot:Q81JD7
Root	Isotig04981	318	1	8.184	2.01E-64	TIGR_CMR|BA_1222 - symbol:BA_1222 "conserved hypothetical protein" species:198094 "Bacillus anthracis str. Ames" [GO:0003674 "molecular_function" evidence=ND] [GO:0005575 "cellular_component" evidence=ND] [GO:0008150 "biological_process" evidence=ND] EMBL:AE016879 EMBL:AE017334 GenomeReviews:AE016879_GR GenomeReviews:AE017334_GR InterPro:IPR008160 Pfam:PF01391 EMBL:AB281483 EMBL:AJ516936 RefSeq:NP_843695.1 RefSeq:YP_017836.1 PDB:2R6Q PDBsum:2R6Q ProteinModelPortal:Q81JD7 SMR:Q81JD7 IntAct:Q81JD7 EnsemblBacteria:EBBACT00000011819 EnsemblBacteria:EBBACT00000016984 GeneID:1084744 GeneID:2814858 KEGG:ban:BA_1222 KEGG:bar:GBAA_1222 PATRIC:18780046 TIGR:BA_1222 TIGR:GBAA1222 HOGENOM:HBG342646 OMA:SDGLNPD ProtClustDB:CLSK824721 Uniprot:Q81JD7
Root	Isotig04982	317	1	8.179	2.96E-64	TIGR_CMR|BA_1222 - symbol:BA_1222 "conserved hypothetical protein" species:198094 "Bacillus anthracis str. Ames" [GO:0003674 "molecular_function" evidence=ND] [GO:0005575 "cellular_component" evidence=ND] [GO:0008150 "biological_process" evidence=ND] EMBL:AE016879 EMBL:AE017334 GenomeReviews:AE016879_GR GenomeReviews:AE017334_GR InterPro:IPR008160 Pfam:PF01391 EMBL:AB281483 EMBL:AJ516936 RefSeq:NP_843695.1 RefSeq:YP_017836.1 PDB:2R6Q PDBsum:2R6Q ProteinModelPortal:Q81JD7 SMR:Q81JD7 IntAct:Q81JD7 EnsemblBacteria:EBBACT00000011819 EnsemblBacteria:EBBACT00000016984 GeneID:1084744 GeneID:2814858 KEGG:ban:BA_1222 KEGG:bar:GBAA_1222 PATRIC:18780046 TIGR:BA_1222 TIGR:GBAA1222 HOGENOM:HBG342646 OMA:SDGLNPD ProtClustDB:CLSK824721 Uniprot:Q81JD7
Root	Isotig04986	0	12	-4.714	0.000297064	TAIR|locus:2200111 - symbol:AT1G11340 species:3702 "Arabidopsis thaliana" [GO:0004672 "protein kinase activity" evidence=IEA] [GO:0004674 "protein serine/threonine kinase activity" evidence=IEA] [GO:0005524 "ATP binding" evidence=IEA] [GO:0006468 "protein phosphorylation" evidence=IEA] [GO:0016301 "kinase activity" evidence=ISS] [GO:0030246 "carbohydrate binding" evidence=ISS] [GO:0048544 "recognition of pollen" evidence=IEA] InterPro:IPR000719 InterPro:IPR000742 InterPro:IPR000858 InterPro:IPR001245 InterPro:IPR006210 InterPro:IPR008271 InterPro:IPR011009 InterPro:IPR024171 Pfam:PF00954 Pfam:PF07714 PIRSF:PIRSF000641 PROSITE:PS00107 PROSITE:PS00108 PROSITE:PS50011 PROSITE:PS50026 SMART:SM00181 InterPro:IPR001480 GO:GO:0016021 EMBL:CP002684 GenomeReviews:CT485782_GR GO:GO:0005524 eggNOG:COG0515 GO:GO:0004872 GO:GO:0005529 SUPFAM:SSF56112 GO:GO:0004674 HOGENOM:HBG755340 GO:GO:0005516 PROSITE:PS00022 PROSITE:PS01186 GO:GO:0044459

Tissue	Identification	# of reads (stress)	# of reads (control)	Fold Change	p-value	Description
						GO:GO:0048544 InterPro:IPR013227 InterPro:IPR003609 Gene3D:G3DSA:2.90.10.10 Pfam:PF01453 Pfam:PF08276 SMART:SM00108 SMART:SM00473 SUPFAM:SSF51110 PROSITE:PS50927 PROSITE:PS50948 EMBL:AC011661 EMBL:AC007259 HSSP:P00523 EMBL:AF084035 IPI:IPI00520959 PIR:E86247 ProteinModelPortal:Q9ZT07 SMR:Q9ZT07 PRIDE:Q9ZT07 GeneFarm:2670 TAIR:At1g11340 InParanoid:Q9LQ00 PhylomeDB:Q9ZT07 Genevestigator:Q9ZT07 Uniprot:Q9ZT07
Root	Isotig04987	0	11	-4.588	0.000569522	TAIR|locus:2200111 - symbol:AT1G11340 species:3702 "Arabidopsis thaliana" [GO:0004672 "protein kinase activity" evidence=IEA] [GO:0004674 "protein serine/threonine kinase activity" evidence=IEA] [GO:0005524 "ATP binding" evidence=IEA] [GO:0006468 "protein phosphorylation" evidence=IEA] [GO:0016301 "kinase activity" evidence=ISS] [GO:0030246 "carbohydrate binding" evidence=ISS] [GO:0048544 "recognition of pollen" evidence=IEA] InterPro:IPR000719 InterPro:IPR000742 InterPro:IPR000858 InterPro:IPR001245 InterPro:IPR006210 InterPro:IPR008271 InterPro:IPR011009 InterPro:IPR024171 Pfam:PF00954 Pfam:PF07714 PIRSF:PIRSF000641 PROSITE:PS00107 PROSITE:PS00108 PROSITE:PS50011 PROSITE:PS50026 SMART:SM00181 InterPro:IPR001480 GO:GO:0016021 EMBL:CP002684 GenomeReviews:CT485782_GR GO:GO:0005524 eggNOG:COG0515 GO:GO:0004872 GO:GO:0005529 SUPFAM:SSF56112 GO:GO:0004674 HOGENOM:HBG755340 GO:GO:0005516 PROSITE:PS00022 PROSITE:PS01186 GO:GO:0044459 GO:GO:0048544 InterPro:IPR013227 InterPro:IPR003609 Gene3D:G3DSA:2.90.10.10 Pfam:PF01453 Pfam:PF08276 SMART:SM00108 SMART:SM00473 SUPFAM:SSF51110 PROSITE:PS50927 PROSITE:PS50948 EMBL:AC011661 EMBL:AC007259 HSSP:P00523 EMBL:AF084035 IPI:IPI00520959 PIR:E86247 ProteinModelPortal:Q9ZT07 SMR:Q9ZT07 PRIDE:Q9ZT07 GeneFarm:2670 TAIR:At1g11340 InParanoid:Q9LQ00 PhylomeDB:Q9ZT07 Genevestigator:Q9ZT07 Uniprot:Q9ZT07
Root	Isotig04995	17	2	2.958	0.000459627	TAIR|locus:2206825 - symbol:AT1G73480 "AT1G73480" species:3702 "Arabidopsis thaliana" [GO:0016787 "hydrolase activity" evidence=ISS] EMBL:CP002684 GenomeReviews:CT485782_GR GO:GO:0016787 HOGENOM:HBG748003 InterPro:IPR022742 Pfam:PF12146 EMBL:AY045929 EMBL:BT001959 IPI:IPI00526340 RefSeq:NP_565066.1 UniGene:At.23620 ProteinModelPortal:Q94AM5 SMR:Q94AM5 MEROPS:S33.A12 PRIDE:Q94AM5 EnsemblPlants:AT1G73480.1 GeneID:843683 KEGG:ath:AT1G73480 TAIR:At1g73480 InParanoid:Q94AM5 OMA:VQPSHPI PhylomeDB:Q94AM5 ProtClustDB:CLSN2917439 ArrayExpress:Q94AM5 Genevestigator:Q94AM5 Uniprot:Q94AM5
Root	Isotig04996	18	2	3.041	0.000259481	TAIR|locus:2206825 - symbol:AT1G73480 "AT1G73480" species:3702 "Arabidopsis thaliana" [GO:0016787 "hydrolase activity" evidence=ISS] EMBL:CP002684 GenomeReviews:CT485782_GR GO:GO:0016787 HOGENOM:HBG748003 InterPro:IPR022742 Pfam:PF12146 EMBL:AY045929 EMBL:BT001959 IPI:IPI00526340 RefSeq:NP_565066.1 UniGene:At.23620 ProteinModelPortal:Q94AM5 SMR:Q94AM5 MEROPS:S33.A12 PRIDE:Q94AM5 EnsemblPlants:AT1G73480.1 GeneID:843683 KEGG:ath:AT1G73480 TAIR:At1g73480 InParanoid:Q94AM5 OMA:VQPSHPI PhylomeDB:Q94AM5 ProtClustDB:CLSN2917439 ArrayExpress:Q94AM5 Genevestigator:Q94AM5 Uniprot:Q94AM5
Root	Isotig04997	17	2	2.958	0.000459627	TAIR|locus:2206825 - symbol:AT1G73480 "AT1G73480" species:3702 "Arabidopsis thaliana" [GO:0016787 "hydrolase activity" evidence=ISS] EMBL:CP002684 GenomeReviews:CT485782_GR GO:GO:0016787 HOGENOM:HBG748003 InterPro:IPR022742 Pfam:PF12146 EMBL:AY045929 EMBL:BT001959 IPI:IPI00526340 RefSeq:NP_565066.1 UniGene:At.23620 ProteinModelPortal:Q94AM5 SMR:Q94AM5 MEROPS:S33.A12 PRIDE:Q94AM5 EnsemblPlants:AT1G73480.1 GeneID:843683 KEGG:ath:AT1G73480 TAIR:At1g73480 InParanoid:Q94AM5 OMA:VQPSHPI PhylomeDB:Q94AM5 ProtClustDB:CLSN2917439 ArrayExpress:Q94AM5 Genevestigator:Q94AM5 Uniprot:Q94AM5
Root	Isotig05001	2	26	-3.829	4.59E-07	TAIR|locus:2032075 - symbol:SNRK2-8 "SNF1-RELATED PROTEIN KINASE 2-8" species:3702 "Arabidopsis thaliana" [GO:0004672 "protein kinase activity" evidence=IEA] [GO:0004674 "protein serine/threonine kinase activity" evidence=IEA] [GO:0005524 "ATP binding" evidence=IEA] [GO:0016301 "kinase activity" evidence=ISS] [GO:0080167 "response to karrikin" evidence=IEP] [GO:0005634 "nucleus" evidence=IDA] [GO:0005737 "cytoplasm" evidence=IDA] [GO:0006970 "response to osmotic stress" evidence=IGI] InterPro:IPR000719 InterPro:IPR002290 InterPro:IPR008271 InterPro:IPR011009 InterPro:IPR017441 Pfam:PF00069 PROSITE:PS00107 PROSITE:PS00108 PROSITE:PS50011 SMART:SM00220 EMBL:CP002684 GenomeReviews:CT485782_GR GO:GO:0005524 GO:GO:0005634 EMBL:AC013430 GO:GO:0005737 eggNOG:COG0515 SUPFAM:SSF56112 GO:GO:0004674 HOGENOM:HBG755340 GO:GO:0080167 HSSP:P06782 UniGene:At.48390 UniGene:At.70998 KO:K14498 EMBL:AF411782 EMBL:BT020608 EMBL:AY088883 IPI:IPI00530546 RefSeq:NP_001077839.1 RefSeq:NP_974170.1 ProteinModelPortal:Q9M9E9

Tissue	Identification	# of reads (stress)	# of reads (control)	Fold Change	p-value	Description
						SMR:Q9M9E9 DIP:DIP-48987N STRING:Q9M9E9 PRIDE:Q9M9E9 EnsemblPlants:AT1G78290.2 EnsemblPlants:AT1G78290.3 GeneID:844164 KEGG:ath:AT1G78290 TAIR:At1g78290 OMA:ERYEIIK PhylomeDB:Q9M9E9 ProtClustDB:CLSN2681301 Genevestigator:Q9M9E9 Uniprot:Q9M9E9
Root	Isotig05002	2	24	-3.714	1.64E-06	TAIR|locus:2032075 - symbol:SNRK2-8 "SNF1-RELATED PROTEIN KINASE 2-8" species:3702 "Arabidopsis thaliana" [GO:0004672 "protein kinase activity" evidence=IEA] [GO:0004674 "protein serine/threonine kinase activity" evidence=IEA] [GO:0005524 "ATP binding" evidence=IEA] [GO:0016301 "kinase activity" evidence=ISS] [GO:0080167 "response to karrikin" evidence=IEP] [GO:0005634 "nucleus" evidence=IDA] [GO:0005737 "cytoplasm" evidence=IDA] [GO:0006970 "response to osmotic stress" evidence=IGI] InterPro:IPR000719 InterPro:IPR002290 InterPro:IPR008271 InterPro:IPR011009 InterPro:IPR017441 Pfam:PF00069 PROSITE:PS00107 PROSITE:PS00108 PROSITE:PS50011 SMART:SM00220 EMBL:CP002684 GenomeReviews:CT485782_GR GO:GO:0005524 GO:GO:0005634 EMBL:AC013430 GO:GO:0005737 eggNOG:COG0515 SUPFAM:SSF56112 GO:GO:0004674 HOGENOM:HBG755340 GO:GO:0080167 HSSP:P06782 UniGene:At.48390 UniGene:At.70998 KO:K14498 EMBL:AF411782 EMBL:BT020608 EMBL:AY088883 IPI:IPI00530546 RefSeq:NP_001077839.1 RefSeq:NP_974170.1 ProteinModelPortal:Q9M9E9 SMR:Q9M9E9 DIP:DIP-48987N STRING:Q9M9E9 PRIDE:Q9M9E9 EnsemblPlants:AT1G78290.2 EnsemblPlants:AT1G78290.3 GeneID:844164 KEGG:ath:AT1G78290 TAIR:At1g78290 OMA:ERYEIIK PhylomeDB:Q9M9E9 ProtClustDB:CLSN2681301 Genevestigator:Q9M9E9 Uniprot:Q9M9E9
Root	Isotig05003	2	24	-3.714	1.64E-06	TAIR|locus:2032075 - symbol:SNRK2-8 "SNF1-RELATED PROTEIN KINASE 2-8" species:3702 "Arabidopsis thaliana" [GO:0004672 "protein kinase activity" evidence=IEA] [GO:0004674 "protein serine/threonine kinase activity" evidence=IEA] [GO:0005524 "ATP binding" evidence=IEA] [GO:0016301 "kinase activity" evidence=ISS] [GO:0080167 "response to karrikin" evidence=IEP] [GO:0005634 "nucleus" evidence=IDA] [GO:0005737 "cytoplasm" evidence=IDA] [GO:0006970 "response to osmotic stress" evidence=IGI] InterPro:IPR000719 InterPro:IPR002290 InterPro:IPR008271 InterPro:IPR011009 InterPro:IPR017441 Pfam:PF00069 PROSITE:PS00107 PROSITE:PS00108 PROSITE:PS50011 SMART:SM00220 EMBL:CP002684 GenomeReviews:CT485782_GR GO:GO:0005524 GO:GO:0005634 EMBL:AC013430 GO:GO:0005737 eggNOG:COG0515 SUPFAM:SSF56112 GO:GO:0004674 HOGENOM:HBG755340 GO:GO:0080167 HSSP:P06782 UniGene:At.48390 UniGene:At.70998 KO:K14498 EMBL:AF411782 EMBL:BT020608 EMBL:AY088883 IPI:IPI00530546 RefSeq:NP_001077839.1 RefSeq:NP_974170.1 ProteinModelPortal:Q9M9E9 SMR:Q9M9E9 DIP:DIP-48987N STRING:Q9M9E9 PRIDE:Q9M9E9 EnsemblPlants:AT1G78290.2 EnsemblPlants:AT1G78290.3 GeneID:844164 KEGG:ath:AT1G78290 TAIR:At1g78290 OMA:ERYEIIK PhylomeDB:Q9M9E9 ProtClustDB:CLSN2681301 Genevestigator:Q9M9E9 Uniprot:Q9M9E9
Root	Isotig05007	1	12	-3.714	0.000699736	TAIR|locus:2043699 - symbol:CYP76C1 ""cytochrome P450, family 76, subfamily C, polypeptide 1"" species:3702 "Arabidopsis thaliana" [GO:0004497 "monooxygenase activity" evidence=IEA] [GO:0005506 "iron ion binding" evidence=IEA] [GO:0009055 "electron carrier activity" evidence=IEA] [GO:0020037 "heme binding" evidence=IEA] [GO:0055114 "oxidation-reduction process" evidence=IEA] InterPro:IPR001128 InterPro:IPR002401 InterPro:IPR017972 Pfam:PF00067 PRINTS:PR00385 PRINTS:PR00463 PROSITE:PS00086 GO:GO:0016021 EMBL:CP002685 GenomeReviews:CT485783_GR GO:GO:0009055 GO:GO:0004497 eggNOG:COG2124 GO:GO:0020037 Gene3D:G3DSA:1.10.630.10 SUPFAM:SSF48264 EMBL:AC003680 GO:GO:0016705 HOGENOM:HBG749920 EMBL:D78600 EMBL:AY078939 EMBL:AY124874 EMBL:AY085090 IPI:IPI00519972 IPI:IPI00548813 PIR:T00869 PIR:T52168 RefSeq:NP_850439.1 RefSeq:NP_850440.1 UniGene:At.334 UniGene:At.67815 ProteinModelPortal:O64636 SMR:O64636 PRIDE:O64636 EnsemblPlants:AT2G45560.1 GeneID:819164 KEGG:ath:AT2G45560 GeneFarm:1463 TAIR:At2g45560 InParanoid:O64636 OMA:SISDIEH PhylomeDB:O64636 ProtClustDB:CLSN2679301 ArrayExpress:O64636 Genevestigator:O64636 GermOnline:AT2G45560 Uniprot:O64636
Root	Isotig05019	35	3	3.415	8.27E-08	TAIR|locus:2132644 - symbol:AT4G22740 "AT4G22740" species:3702 "Arabidopsis thaliana" [GO:0003674 "molecular_function" evidence=ND] [GO:0005886 "plasma membrane" evidence=IDA] GO:GO:0005886 EMBL:CP002687 InterPro:IPR019376 Pfam:PF10248 UniGene:At.22243 UniGene:At.32505 EMBL:AY054243 EMBL:AY080635 EMBL:AY117203 IPI:IPI00525351 RefSeq:NP_567666.1 RefSeq:NP_849421.1 PRIDE:Q940N4 ProMEX:Q940N4 EnsemblPlants:AT4G22740.1 EnsemblPlants:AT4G22740.2 GeneID:828370 KEGG:ath:AT4G22740 TAIR:At4g22740 InParanoid:Q940N4 OMA:VNAERRN PhylomeDB:Q940N4 ProtClustDB:CLSN2689561 Genevestigator:Q940N4 Uniprot:Q940N4

Tissue	Identification	# of reads (stress)	# of reads (control)	Fold Change	p-value	Description
Root	Isotig05020	42	5	2.941	4.00E-08	TAIR|locus:2132644 - symbol:AT4G22740 "AT4G22740" species:3702 "Arabidopsis thaliana" [GO:0003674 "molecular_function" evidence=ND] [GO:0005886 "plasma membrane" evidence=IDA] GO:GO:0005886 EMBL:CP002687 InterPro:IPR019376 Pfam:PF10248 UniGene:At.22243 UniGene:At.32505 EMBL:AY054243 EMBL:AY080635 EMBL:AY117203 IPI:IPI00525351 RefSeq:NP_567666.1 RefSeq:NP_849421.1 PRIDE:Q940N4 ProMEX:Q940N4 EnsemblPlants:AT4G22740.1 EnsemblPlants:AT4G22740.2 GeneID:828370 KEGG:ath:AT4G22740 TAIR:At4g22740 InParanoid:Q940N4 OMA:VNAERRN PhylomeDB:Q940N4 ProtClustDB:CLSN2689561 Genevestigator:Q940N4 Uniprot:Q940N4
Root	Isotig05022	21	0	5.263	3.38E-06	TAIR|locus:2195623 - symbol:AT1G60420 species:3702 "Arabidopsis thaliana" [GO:0016209 "antioxidant activity" evidence=IEA] [GO:0016491 "oxidoreductase activity" evidence=IEA] [GO:0046686 "response to cadmium ion" evidence=IEP] [GO:0009860 "pollen tube growth" evidence=IMP] [GO:0010183 "pollen tube guidance" evidence=IMP] [GO:0080092 "regulation of pollen tube growth" evidence=IMP] [GO:0005829 "cytosol" evidence=IDA] InterPro:IPR011424 InterPro:IPR017937 Pfam:PF07649 PROSITE:PS00194 EMBL:CP002684 GenomeReviews:CT485782_GR GO:GO:0005829 GO:GO:0046686 GO:GO:0009860 InterPro:IPR012336 Gene3D:G3DSA:3.40.30.10 SUPFAM:SSF52833 GO:GO:0045454 PROSITE:PS51352 GO:GO:0080092 GO:GO:0010183 eggNOG:COG0526 GO:GO:0047134 EMBL:AC004473 EMBL:AY065433 EMBL:AY117231 EMBL:AY087396 IPI:IPI00537432 PIR:T02292 RefSeq:NP_564756.1 UniGene:At.10685 UniGene:At.64112 PDB:1V5N PDBsum:1V5N ProteinModelPortal:O80763 SMR:O80763 IntAct:O80763 PRIDE:O80763 EnsemblPlants:AT1G60420.1 GeneID:842337 KEGG:ath:AT1G60420 TAIR:At1g60420 HOGENOM:HBG594354 InParanoid:O80763 OMA:QMPWLAL PhylomeDB:O80763 ProtClustDB:CLSN2917334 ArrayExpress:O80763 Genevestigator:O80763 Uniprot:O80763
Root	Isotig05023	16	0	4.871	5.89E-05	TAIR|locus:2195623 - symbol:AT1G60420 species:3702 "Arabidopsis thaliana" [GO:0016209 "antioxidant activity" evidence=IEA] [GO:0016491 "oxidoreductase activity" evidence=IEA] [GO:0046686 "response to cadmium ion" evidence=IEP] [GO:0009860 "pollen tube growth" evidence=IMP] [GO:0010183 "pollen tube guidance" evidence=IMP] [GO:0080092 "regulation of pollen tube growth" evidence=IMP] [GO:0005829 "cytosol" evidence=IDA] InterPro:IPR011424 InterPro:IPR017937 Pfam:PF07649 PROSITE:PS00194 EMBL:CP002684 GenomeReviews:CT485782_GR GO:GO:0005829 GO:GO:0046686 GO:GO:0009860 InterPro:IPR012336 Gene3D:G3DSA:3.40.30.10 SUPFAM:SSF52833 GO:GO:0045454 PROSITE:PS51352 GO:GO:0080092 GO:GO:0010183 eggNOG:COG0526 GO:GO:0047134 EMBL:AC004473 EMBL:AY065433 EMBL:AY117231 EMBL:AY087396 IPI:IPI00537432 PIR:T02292 RefSeq:NP_564756.1 UniGene:At.10685 UniGene:At.64112 PDB:1V5N PDBsum:1V5N ProteinModelPortal:O80763 SMR:O80763 IntAct:O80763 PRIDE:O80763 EnsemblPlants:AT1G60420.1 GeneID:842337 KEGG:ath:AT1G60420 TAIR:At1g60420 HOGENOM:HBG594354 InParanoid:O80763 OMA:QMPWLAL PhylomeDB:O80763 ProtClustDB:CLSN2917334 ArrayExpress:O80763 Genevestigator:O80763 Uniprot:O80763
Root	Isotig05037	105	51	0.913	0.000128333	TAIR|locus:2061231 - symbol:AT2G40010 "AT2G40010" species:3702 "Arabidopsis thaliana" [GO:0003735 "structural constituent of ribosome" evidence=ISS] [GO:0005840 "ribosome" evidence=ISS] [GO:0006412 "translation" evidence=ISS] [GO:0006414 "translational elongation" evidence=IEA;ISS] [GO:0042254 "ribosome biogenesis" evidence=IEA] [GO:0022626 "cytosolic ribosome" evidence=IDA] [GO:0005515 "protein binding" evidence=IPI] [GO:0005794 "Golgi apparatus" evidence=RCA] InterPro:IPR001790 InterPro:IPR001813 Pfam:PF00428 Pfam:PF00466 EMBL:CP002685 GenomeReviews:CT485783_GR GO:GO:0005515 GO:GO:0003735 GO:GO:0022626 GO:GO:0042254 EMBL:AF002109 GO:GO:0006414 eggNOG:COG0244 EMBL:BT001919 IPI:IPI00525504 PIR:B84824 RefSeq:NP_181530.1 UniGene:At.28353 UniGene:At.49578 ProteinModelPortal:O04204 SMR:O04204 IntAct:O04204 STRING:O04204 PRIDE:O04204 EnsemblPlants:AT2G40010.1 GeneID:818589 KEGG:ath:AT2G40010 TAIR:At2g40010 HOGENOM:HBG601294 InParanoid:O04204 KO:K02941 OMA:DYEKLLP PhylomeDB:O04204 ProtClustDB:CLSN2683388 ArrayExpress:O04204 Genevestigator:O04204 GermOnline:AT2G40010 Uniprot:O04204
Root	Isotig05038	103	50	0.914	0.000147131	TAIR|locus:2061231 - symbol:AT2G40010 "AT2G40010" species:3702 "Arabidopsis thaliana" [GO:0003735 "structural constituent of ribosome" evidence=ISS] [GO:0005840 "ribosome" evidence=ISS] [GO:0006412 "translation" evidence=ISS] [GO:0006414 "translational elongation" evidence=IEA;ISS] [GO:0042254 "ribosome biogenesis" evidence=IEA] [GO:0022626 "cytosolic ribosome" evidence=IDA] [GO:0005515 "protein binding" evidence=IPI] [GO:0005794 "Golgi apparatus" evidence=RCA] InterPro:IPR001790 InterPro:IPR001813 Pfam:PF00428 Pfam:PF00466 EMBL:CP002685 GenomeReviews:CT485783_GR GO:GO:0005515 GO:GO:0003735 GO:GO:0022626 GO:GO:0042254 EMBL:AF002109 GO:GO:0006414 eggNOG:COG0244

Tissue	Identification	# of reads (stress)	# of reads (control)	Fold Change	p-value	Description
						EMBL:BT001919 IPI:IPI00525504 PIR:B84824 RefSeq:NP_181530.1 UniGene:At.28353 UniGene:At.49578 ProteinModelPortal:O04204 SMR:O04204 IntAct:O04204 STRING:O04204 PRIDE:O04204 EnsemblPlants:AT2G40010.1 GeneID:818589 KEGG:ath:AT2G40010 TAIR:At2g40010 HOGENOM:HBG601294 InParanoid:O04204 KO:K02941 OMA:DYEKLLP PhylomeDB:O04204 ProtClustDB:CLSN2683388 ArrayExpress:O04204 Genevestigator:O04204 GermOnline:AT2G40010 Uniprot:O04204
Root	Isotig05039	98	51	0.813	0.000797392	TAIR|locus:2061231 - symbol:AT2G40010 "AT2G40010" species:3702 "Arabidopsis thaliana" [GO:0003735 "structural constituent of ribosome" evidence=ISS] [GO:0005840 "ribosome" evidence=ISS] [GO:0006412 "translation" evidence=ISS] [GO:0006414 "translational elongation" evidence=IEA;ISS] [GO:0042254 "ribosome biogenesis" evidence=IEA] [GO:0022626 "cytosolic ribosome" evidence=IDA] [GO:0005515 "protein binding" evidence=IPI] [GO:0005794 "Golgi apparatus" evidence=RCA] InterPro:IPR001790 InterPro:IPR001813 Pfam:PF00428 Pfam:PF00466 EMBL:CP002685 GenomeReviews:CT485783_GR GO:GO:0005515 GO:GO:0003735 GO:GO:0022626 GO:GO:0042254 EMBL:AF002109 GO:GO:0006414 eggNOG:COG0244 EMBL:BT001919 IPI:IPI00525504 PIR:B84824 RefSeq:NP_181530.1 UniGene:At.28353 UniGene:At.49578 ProteinModelPortal:O04204 SMR:O04204 IntAct:O04204 STRING:O04204 PRIDE:O04204 EnsemblPlants:AT2G40010.1 GeneID:818589 KEGG:ath:AT2G40010 TAIR:At2g40010 HOGENOM:HBG601294 InParanoid:O04204 KO:K02941 OMA:DYEKLLP PhylomeDB:O04204 ProtClustDB:CLSN2683388 ArrayExpress:O04204 Genevestigator:O04204 GermOnline:AT2G40010 Uniprot:O04204
Root	Isotig05055	5	28	-2.614	1.03E-05	TAIR|locus:2172309 - symbol:GDH1 "AT5G18170" species:3702 "Arabidopsis thaliana" [GO:0016491 "oxidoreductase activity" evidence=ISS] [GO:0005739 "mitochondrion" evidence=IDA] [GO:0046686 "response to cadmium ion" evidence=IEP] [GO:0005524 "ATP binding" evidence=IDA] [GO:0009651 "response to salt stress" evidence=IEP] [GO:0005507 "copper ion binding" evidence=IDA] [GO:0008270 "zinc ion binding" evidence=IDA] [GO:0050897 "cobalt ion binding" evidence=IDA] [GO:0004353 "glutamate dehydrogenase [NAD(P)+] activity" evidence=IDA;IMP] [GO:0006807 "nitrogen compound metabolic process" evidence=IMP] [GO:0009646 "response to absence of light" evidence=IEP] InterPro:IPR006095 InterPro:IPR006096 InterPro:IPR006097 InterPro:IPR014362 InterPro:IPR016040 Pfam:PF00208 Pfam:PF02812 PIRSF:PIRSF000185 PRINTS:PR00082 PROSITE:PS00074 SMART:SM00839 GO:GO:0005739 GO:GO:0005524 GO:GO:0046686 EMBL:CP002688 GenomeReviews:BA000015_GR Gene3D:G3DSA:3.40.50.720 GO:GO:0008270 GO:GO:0005507 GO:GO:0006520 GO:GO:0009651 GO:GO:0009646 GO:GO:0050897 EMBL:AB012246 EMBL:U37771 EMBL:U53527 IPI:IPI00531762 PIR:S71217 RefSeq:NP_197318.1 UniGene:At.23609 ProteinModelPortal:Q43314 SMR:Q43314 IntAct:Q43314 STRING:Q43314 PRIDE:Q43314 ProMEX:Q43314 EnsemblPlants:AT5G18170.1 GeneID:831935 KEGG:ath:AT5G18170 GeneFarm:2257 TAIR:At5g18170 eggNOG:COG0334 HOGENOM:HBG590661 InParanoid:Q43314 KO:K00261 OMA:NALYDFH PhylomeDB:Q43314 ProtClustDB:PLN02477 ArrayExpress:Q43314 Genevestigator:Q43314 GermOnline:AT5G18170 GO:GO:0004353 Uniprot:Q43314
Root	Isotig05056	5	22	-2.266	0.000318925	TAIR|locus:2079364 - symbol:GDH3 "glutamate dehydrogenase 3" species:3702 "Arabidopsis thaliana" [GO:0003824 "catalytic activity" evidence=IEA] [GO:0016491 "oxidoreductase activity" evidence=IEA;ISS] [GO:0016639 "oxidoreductase activity, acting on the CH-NH2 group of donors, NAD or NADP as acceptor" evidence=IEA] [GO:0005739 "mitochondrion" evidence=IDA] [GO:0051171 "regulation of nitrogen compound metabolic process" evidence=IEP] InterPro:IPR006095 InterPro:IPR006096 InterPro:IPR006097 InterPro:IPR014362 InterPro:IPR016040 Pfam:PF00208 Pfam:PF02812 PIRSF:PIRSF000185 PRINTS:PR00082 PROSITE:PS00074 SMART:SM00839 GO:GO:0005739 EMBL:CP002686 GenomeReviews:BA000014_GR GO:GO:0000166 Gene3D:G3DSA:3.40.50.720 GO:GO:0006520 EMBL:AC009540 eggNOG:COG0334 HOGENOM:HBG590661 KO:K00261 ProtClustDB:PLN02477 GO:GO:0004353 EMBL:AC011698 IPI:IPI00538151 RefSeq:NP_187041.1 UniGene:At.27423 UniGene:At.53173 ProteinModelPortal:Q9S7A0 SMR:Q9S7A0 STRING:Q9S7A0 PRIDE:Q9S7A0 ProMEX:Q9S7A0 EnsemblPlants:AT3G03910.1 GeneID:821072 KEGG:ath:AT3G03910 GeneFarm:2267 TAIR:At3g03910 InParanoid:Q9S7A0 OMA:VPTHEEE PhylomeDB:Q9S7A0 ArrayExpress:Q9S7A0 Genevestigator:Q9S7A0 GermOnline:AT3G03910 GO:GO:0051171 Uniprot:Q9S7A0
Root	Isotig05057	5	22	-2.266	0.000318925	TAIR|locus:2079364 - symbol:GDH3 "glutamate dehydrogenase 3" species:3702 "Arabidopsis thaliana" [GO:0003824 "catalytic activity" evidence=IEA] [GO:0016491 "oxidoreductase activity" evidence=IEA;ISS] [GO:0016639 "oxidoreductase activity, acting on the CH-NH2 group of donors, NAD or NADP as acceptor" evidence=IEA] [GO:0005739 "mitochondrion" evidence=IDA] [GO:0051171 "regulation of nitrogen compound metabolic process" evidence=IEP] InterPro:IPR006095 InterPro:IPR006096 InterPro:IPR006097

Tissue	Identification	# of reads (stress)	# of reads (control)	Fold Change	p-value	Description
						InterPro:IPR014362 InterPro:IPR016040 Pfam:PF00208 Pfam:PF02812 PIRSF:PIRSF000185 PRINTS:PR00082 PROSITE:PS00074 SMART:SM00839 GO:GO:0005739 EMBL:CP002686 GenomeReviews:BA000014_GR GO:GO:0000166 Gene3D:G3DSA:3.40.50.720 GO:GO:0006520 EMBL:AC009540 eggNOG:COG0334 HOGENOM:HBG590661 KO:K00261 ProtClustDB:PLN02477 GO:GO:0004353 EMBL:AC011698 IPI:IPI00538151 RefSeq:NP_187041.1 UniGene:At.27423 UniGene:At.53173 ProteinModelPortal:Q9S7A0 SMR:Q9S7A0 STRING:Q9S7A0 PRIDE:Q9S7A0 ProMEX:Q9S7A0 EnsemblPlants:AT3G03910.1 GeneID:821072 KEGG:ath:AT3G03910 GeneFarm:2267 TAIR:At3g03910 InParanoid:Q9S7A0 OMA:VPTHEEE PhylomeDB:Q9S7A0 ArrayExpress:Q9S7A0 Genevestigator:Q9S7A0 GermOnline:AT3G03910 GO:GO:0051171 Uniprot:Q9S7A0
Root	Isotig05061	36	9	1.871	9.91E-05	UNIPROTKB|P83304 - symbol:P83304 "Mannose/glucose-specific lectin" species:185447 "Parkia platycephala" [GO:0000771 "agglutination involved in conjugation" evidence=IDA] [GO:0005536 "glucose binding" evidence=IDA] [GO:0005537 "mannose binding" evidence=IDA] [GO:0005575 "cellular_component" evidence=ND] GO:GO:0005537 GO:GO:0005536 InterPro:IPR001229 Gene3D:G3DSA:2.100.10.30 Pfam:PF01419 SMART:SM00915 SUPFAM:SSF51101 GO:GO:0000771 PDB:1ZGR PDB:1ZGS PDBsum:1ZGR PDBsum:1ZGS ProteinModelPortal:P83304 SMR:P83304 Uniprot:P83304
Root	Isotig05062	44	4	3.330	2.70E-09	TAIR|locus:2154885 - symbol:AT5G66540 "AT5G66540" species:3702 "Arabidopsis thaliana" [GO:0003674 "molecular_function" evidence=ND] [GO:0006364 "rRNA processing" evidence=IEA] [GO:0005730 "nucleolus" evidence=IDA] [GO:0005634 "nucleus" evidence=IDA] [GO:0005829 "cytosol" evidence=IDA] InterPro:IPR012173 PIRSF:PIRSF017300 GO:GO:0005829 EMBL:CP002688 GenomeReviews:BA000015_GR GO:GO:0005730 GO:GO:0030529 GO:GO:0006364 EMBL:AB013389 eggNOG:COG5384 KO:K14559 InterPro:IPR007151 PANTHER:PTHR17039 Pfam:PF04006 EMBL:AF389279 EMBL:AY149928 IPI:IPI00539000 RefSeq:NP_569037.1 UniGene:At.8562 STRING:Q9FJY5 PRIDE:Q9FJY5 EnsemblPlants:AT5G66540.1 GeneID:836786 KEGG:ath:AT5G66540 TAIR:At5g66540 HOGENOM:HBG317308 InParanoid:Q9FJY5 OMA:FKAESAN PhylomeDB:Q9FJY5 ProtClustDB:CLSN2690096 Genevestigator:Q9FJY5 Uniprot:Q9FJY5
Root	Isotig05063	34	6	2.374	1.09E-05	UNIPROTKB|P83304 - symbol:P83304 "Mannose/glucose-specific lectin" species:185447 "Parkia platycephala" [GO:0000771 "agglutination involved in conjugation" evidence=IDA] [GO:0005536 "glucose binding" evidence=IDA] [GO:0005537 "mannose binding" evidence=IDA] [GO:0005575 "cellular_component" evidence=ND] GO:GO:0005537 GO:GO:0005536 InterPro:IPR001229 Gene3D:G3DSA:2.100.10.30 Pfam:PF01419 SMART:SM00915 SUPFAM:SSF51101 GO:GO:0000771 PDB:1ZGR PDB:1ZGS PDBsum:1ZGR PDBsum:1ZGS ProteinModelPortal:P83304 SMR:P83304 Uniprot:P83304
Root	Isotig05067	130	22	2.434	2.57E-18	TAIR|locus:2025162 - symbol:DHAR2 "AT1G75270" species:3702 "Arabidopsis thaliana" [GO:0045174 "glutathione dehydrogenase (ascorbate) activity" evidence=ISS;IDA] [GO:0010731 "protein glutathionylation" evidence=IDA] [GO:0043295 "glutathione binding" evidence=IDA] [GO:0005886 "plasma membrane" evidence=IDA] [GO:0005829 "cytosol" evidence=IDA] InterPro:IPR004045 PROSITE:PS50404 EMBL:CP002684 GenomeReviews:CT485782_GR GO:GO:0005829 GO:GO:0005886 GO:GO:0006950 GO:GO:0009636 InterPro:IPR012336 Gene3D:G3DSA:3.40.30.10 SUPFAM:SSF52833 InterPro:IPR010987 Gene3D:G3DSA:1.20.1050.10 SUPFAM:SSF47616 PROSITE:PS50405 InterPro:IPR017933 HSSP:Q9ZVQ3 GO:GO:0045174 GO:GO:0004364 GO:GO:0010731 EMBL:AY074785 EMBL:AC025814 EMBL:AY140019 EMBL:BT006257 EMBL:AY087460 IPI:IPI00542474 PIR:B96783 RefSeq:NP_177662.1 UniGene:At.27979 ProteinModelPortal:Q9FRL8 SMR:Q9FRL8 IntAct:Q9FRL8 STRING:Q9FRL8 PRIDE:Q9FRL8 EnsemblPlants:AT1G75270.1 GeneID:843864 KEGG:ath:AT1G75270 TAIR:At1g75270 eggNOG:COG0625 InParanoid:Q9FRL8 OMA:IDVFAKF PhylomeDB:Q9FRL8 ProtClustDB:CLSN2914231 ArrayExpress:Q9FRL8 Genevestigator:Q9FRL8 GO:GO:0043295 Uniprot:Q9FRL8
Root	Isotig05068	107	19	2.365	6.89E-15	TAIR|locus:2025162 - symbol:DHAR2 "AT1G75270" species:3702 "Arabidopsis thaliana" [GO:0045174 "glutathione dehydrogenase (ascorbate) activity" evidence=ISS;IDA] [GO:0010731 "protein glutathionylation" evidence=IDA] [GO:0043295 "glutathione binding" evidence=IDA] [GO:0005886 "plasma membrane" evidence=IDA] [GO:0005829 "cytosol" evidence=IDA] InterPro:IPR004045 PROSITE:PS50404 EMBL:CP002684 GenomeReviews:CT485782_GR GO:GO:0005829 GO:GO:0005886 GO:GO:0006950 GO:GO:0009636 InterPro:IPR012336 Gene3D:G3DSA:3.40.30.10 SUPFAM:SSF52833 InterPro:IPR010987 Gene3D:G3DSA:1.20.1050.10 SUPFAM:SSF47616 PROSITE:PS50405 InterPro:IPR017933 HSSP:Q9ZVQ3 GO:GO:0045174 GO:GO:0004364 GO:GO:0010731 EMBL:AY074785 EMBL:AC025814 EMBL:AY140019 EMBL:BT006257 EMBL:AY087460 IPI:IPI00542474 PIR:B96783 RefSeq:NP_177662.1 UniGene:At.27979

Tissue	Identification	# of reads (stress)	# of reads (control)	Fold Change	p-value	Description
						ProteinModelPortal:Q9FRL8 SMR:Q9FRL8 IntAct:Q9FRL8 STRING:Q9FRL8 PRIDE:Q9FRL8 EnsemblPlants:AT1G75270.1 GeneID:843864 KEGG:ath:AT1G75270 TAIR:At1g75270 eggNOG:COG0625 InParanoid:Q9FRL8 OMA:IDVFAKF PhylomeDB:Q9FRL8 ProtClustDB:CLSN2914231 ArrayExpress:Q9FRL8 Genevestigator:Q9FRL8 GO:GO:0043295 Uniprot:Q9FRL8
Root	Isotig05069	111	19	2.418	9.42E-16	TAIR|locus:2025162 - symbol:DHAR2 "AT1G75270" species:3702 "Arabidopsis thaliana" [GO:0045174 "glutathione dehydrogenase (ascorbate) activity" evidence=ISS;IDA] [GO:0010731 "protein glutathionylation" evidence=IDA] [GO:0043295 "glutathione binding" evidence=IDA] [GO:0005886 "plasma membrane" evidence=IDA] [GO:0005829 "cytosol" evidence=IDA] InterPro:IPR004045 PROSITE:PS50404 EMBL:CP002684 GenomeReviews:CT485782_GR GO:GO:0005829 GO:GO:0005886 GO:GO:0006950 GO:GO:0009636 InterPro:IPR012336 Gene3D:G3DSA:3.40.30.10 SUPFAM:SSF52833 InterPro:IPR010987 Gene3D:G3DSA:1.20.1050.10 SUPFAM:SSF47616 PROSITE:PS50405 InterPro:IPR017933 HSSP:Q9ZVQ3 GO:GO:0045174 GO:GO:0004364 GO:GO:0010731 EMBL:AY074785 EMBL:AC025814 EMBL:AY140019 EMBL:BT006257 EMBL:AY087460 IPI:IPI00542474 PIR:B96783 RefSeq:NP_177662.1 UniGene:At.27979 ProteinModelPortal:Q9FRL8 SMR:Q9FRL8 IntAct:Q9FRL8 STRING:Q9FRL8 PRIDE:Q9FRL8 EnsemblPlants:AT1G75270.1 GeneID:843864 KEGG:ath:AT1G75270 TAIR:At1g75270 eggNOG:COG0625 InParanoid:Q9FRL8 OMA:IDVFAKF PhylomeDB:Q9FRL8 ProtClustDB:CLSN2914231 ArrayExpress:Q9FRL8 Genevestigator:Q9FRL8 GO:GO:0043295 Uniprot:Q9FRL8
Root	Isotig05073	68	18	1.789	2.13E-07	TAIR|locus:2128238 - symbol:BTI2 "AT4G11220" species:3702 "Arabidopsis thaliana" [GO:0005783 "endoplasmic reticulum" evidence=ISS] [GO:0005886 "plasma membrane" evidence=IDA] [GO:0005789 "endoplasmic reticulum membrane" evidence=IDA] [GO:0071458 "integral to cytosolic side of endoplasmic reticulum membrane" evidence=IDA] [GO:0071782 "endoplasmic reticulum tubular network" evidence=IDA] [GO:0071786 "endoplasmic reticulum tubular network organization" evidence=IMP] [GO:0005794 "Golgi apparatus" evidence=IDA] InterPro:IPR003388 Pfam:PF02453 PROSITE:PS50845 GO:GO:0005886 EMBL:CP002687 GenomeReviews:CT486007_GR EMBL:AL096882 EMBL:AL161531 GO:GO:0071458 PANTHER:PTHR10994 GO:GO:0071786 GO:GO:0071782 eggNOG:NOG303514 ProtClustDB:CLSN2685988 EMBL:AY034901 EMBL:AY048273 EMBL:AY057606 EMBL:BT002368 EMBL:AY086294 IPI:IPI00525362 PIR:T13013 RefSeq:NP_192861.1 UniGene:At.3649 ProteinModelPortal:Q9SUT9 SMR:Q9SUT9 IntAct:Q9SUT9 STRING:Q9SUT9 PRIDE:Q9SUT9 EnsemblPlants:AT4G11220.1 GeneID:826724 KEGG:ath:AT4G11220 TAIR:At4g11220 HOGENOM:HBG596795 InParanoid:Q9SUT9 OMA:HEESSPN PhylomeDB:Q9SUT9 Genevestigator:Q9SUT9 Uniprot:Q9SUT9
Root	Isotig05074	68	18	1.789	2.13E-07	TAIR|locus:2128238 - symbol:BTI2 "AT4G11220" species:3702 "Arabidopsis thaliana" [GO:0005783 "endoplasmic reticulum" evidence=ISS] [GO:0005886 "plasma membrane" evidence=IDA] [GO:0005789 "endoplasmic reticulum membrane" evidence=IDA] [GO:0071458 "integral to cytosolic side of endoplasmic reticulum membrane" evidence=IDA] [GO:0071782 "endoplasmic reticulum tubular network" evidence=IDA] [GO:0071786 "endoplasmic reticulum tubular network organization" evidence=IMP] [GO:0005794 "Golgi apparatus" evidence=IDA] InterPro:IPR003388 Pfam:PF02453 PROSITE:PS50845 GO:GO:0005886 EMBL:CP002687 GenomeReviews:CT486007_GR EMBL:AL096882 EMBL:AL161531 GO:GO:0071458 PANTHER:PTHR10994 GO:GO:0071786 GO:GO:0071782 eggNOG:NOG303514 ProtClustDB:CLSN2685988 EMBL:AY034901 EMBL:AY048273 EMBL:AY057606 EMBL:BT002368 EMBL:AY086294 IPI:IPI00525362 PIR:T13013 RefSeq:NP_192861.1 UniGene:At.3649 ProteinModelPortal:Q9SUT9 SMR:Q9SUT9 IntAct:Q9SUT9 STRING:Q9SUT9 PRIDE:Q9SUT9 EnsemblPlants:AT4G11220.1 GeneID:826724 KEGG:ath:AT4G11220 TAIR:At4g11220 HOGENOM:HBG596795 InParanoid:Q9SUT9 OMA:HEESSPN PhylomeDB:Q9SUT9 Genevestigator:Q9SUT9 Uniprot:Q9SUT9
Root	Isotig05075	61	9	2.632	4.18E-10	TAIR|locus:2128238 - symbol:BTI2 "AT4G11220" species:3702 "Arabidopsis thaliana" [GO:0005783 "endoplasmic reticulum" evidence=ISS] [GO:0005886 "plasma membrane" evidence=IDA] [GO:0005789 "endoplasmic reticulum membrane" evidence=IDA] [GO:0071458 "integral to cytosolic side of endoplasmic reticulum membrane" evidence=IDA] [GO:0071782 "endoplasmic reticulum tubular network" evidence=IDA] [GO:0071786 "endoplasmic reticulum tubular network organization" evidence=IMP] [GO:0005794 "Golgi apparatus" evidence=IDA] InterPro:IPR003388 Pfam:PF02453 PROSITE:PS50845 GO:GO:0005886 EMBL:CP002687 GenomeReviews:CT486007_GR EMBL:AL096882 EMBL:AL161531 GO:GO:0071458 PANTHER:PTHR10994 GO:GO:0071786 GO:GO:0071782 eggNOG:NOG303514 ProtClustDB:CLSN2685988 EMBL:AY034901 EMBL:AY048273 EMBL:AY057606 EMBL:BT002368 EMBL:AY086294 IPI:IPI00525362 PIR:T13013

Tissue	Identification	# of reads (stress)	# of reads (control)	Fold Change	p-value	Description
						RefSeq:NP_192861.1 UniGene:At.3649 ProteinModelPortal:Q9SUT9 SMR:Q9SUT9 IntAct:Q9SUT9 STRING:Q9SUT9 PRIDE:Q9SUT9 EnsemblPlants:AT4G11220.1 GeneID:826724 KEGG:ath:AT4G11220 TAIR:At4g11220 HOGENOM:HBG596795 InParanoid:Q9SUT9 OMA:HEESSPN PhylomeDB:Q9SUT9 Genevestigator:Q9SUT9 Uniprot:Q9SUT9
Root	Isotig05082	7	28	-2.129	9.37E-05	TAIR|locus:2154508 - symbol:AT5G58900 "AT5G58900" species:3702 "Arabidopsis thaliana" [GO:0003677 "DNA binding" evidence=ISS] [GO:0006355 "regulation of transcription, DNA-dependent" evidence=IEA] [GO:0003700 "sequence-specific DNA binding transcription factor activity" evidence=ISS] InterPro:IPR001005 InterPro:IPR009057 Pfam:PF00249 SMART:SM00717 GO:GO:0005634 EMBL:CP002688 GenomeReviews:BA000015_GR GO:GO:0006355 GO:GO:0003677 Gene3D:G3DSA:1.10.10.60 SUPFAM:SSF46689 InterPro:IPR017884 PROSITE:PS51293 InterPro:IPR017930 InterPro:IPR006447 TIGRFAMs:TIGR01557 PROSITE:PS51294 EMBL:AB016885 HOGENOM:HBG591154 EMBL:BT005473 EMBL:AY519533 EMBL:AK118891 IPI:IPI00547099 RefSeq:NP_200698.1 UniGene:At.7757 ProteinModelPortal:Q9FIL9 SMR:Q9FIL9 PRIDE:Q9FIL9 DNASU:836007 EnsemblPlants:AT5G58900.1 GeneID:836007 KEGG:ath:AT5G58900 TAIR:At5g58900 eggNOG:NOG325121 InParanoid:Q9FIL9 OMA:PFTLDWA PhylomeDB:Q9FIL9 ProtClustDB:CLSN2914846 ArrayExpress:Q9FIL9 Genevestigator:Q9FIL9 Uniprot:Q9FIL9
Root	Isotig05109	8	33	-2.173	1.74E-05	TAIR|locus:2125239 - symbol:WRKY11 "AT4G31550" species:3702 "Arabidopsis thaliana" [GO:0003700 "sequence-specific DNA binding transcription factor activity" evidence=ISS] [GO:0005634 "nucleus" evidence=IC] [GO:0006355 "regulation of transcription, DNA-dependent" evidence=ISS] [GO:0005516 "calmodulin binding" evidence=IDA] [GO:0042742 "defense response to bacterium" evidence=IEP] [GO:0010200 "response to chitin" evidence=IEP] InterPro:IPR003657 Pfam:PF03106 PROSITE:PS50811 SMART:SM00774 EMBL:CP002687 GenomeReviews:CT486007_GR GO:GO:0005634 GO:GO:0042742 GO:GO:0043565 GO:GO:0003700 GO:GO:0006351 GO:GO:0005516 GO:GO:0010200 EMBL:AL080283 EMBL:AL161579 Gene3D:G3DSA:2.20.25.80 SUPFAM:SSF118290 EMBL:AF404856 EMBL:AY059731 EMBL:AY150394 EMBL:BT001100 EMBL:AY084855 IPI:IPI00516622 IPI:IPI00531377 PIR:T10685 RefSeq:NP_567878.2 RefSeq:NP_849559.1 UniGene:At.24683 ProteinModelPortal:Q9SV15 SMR:Q9SV15 IntAct:Q9SV15 MINT:MINT-1339027 STRING:Q9SV15 EnsemblPlants:AT4G31550.1 GeneID:829282 KEGG:ath:AT4G31550 TAIR:At4g31550 eggNOG:NOG325238 HOGENOM:HBG595203 InParanoid:Q9SV15 OMA:AMQENIS PhylomeDB:Q9SV15 ProtClustDB:CLSN2688388 ArrayExpress:Q9SV15 Genevestigator:Q9SV15 InterPro:IPR018872 Pfam:PF10533 Uniprot:Q9SV15
Root	Isotig05110	9	32	-1.959	7.57E-05	TAIR|locus:2125239 - symbol:WRKY11 "AT4G31550" species:3702 "Arabidopsis thaliana" [GO:0003700 "sequence-specific DNA binding transcription factor activity" evidence=ISS] [GO:0005634 "nucleus" evidence=IC] [GO:0006355 "regulation of transcription, DNA-dependent" evidence=ISS] [GO:0005516 "calmodulin binding" evidence=IDA] [GO:0042742 "defense response to bacterium" evidence=IEP] [GO:0010200 "response to chitin" evidence=IEP] InterPro:IPR003657 Pfam:PF03106 PROSITE:PS50811 SMART:SM00774 EMBL:CP002687 GenomeReviews:CT486007_GR GO:GO:0005634 GO:GO:0042742 GO:GO:0043565 GO:GO:0003700 GO:GO:0006351 GO:GO:0005516 GO:GO:0010200 EMBL:AL080283 EMBL:AL161579 Gene3D:G3DSA:2.20.25.80 SUPFAM:SSF118290 EMBL:AF404856 EMBL:AY059731 EMBL:AY150394 EMBL:BT001100 EMBL:AY084855 IPI:IPI00516622 IPI:IPI00531377 PIR:T10685 RefSeq:NP_567878.2 RefSeq:NP_849559.1 UniGene:At.24683 ProteinModelPortal:Q9SV15 SMR:Q9SV15 IntAct:Q9SV15 MINT:MINT-1339027 STRING:Q9SV15 EnsemblPlants:AT4G31550.1 GeneID:829282 KEGG:ath:AT4G31550 TAIR:At4g31550 eggNOG:NOG325238 HOGENOM:HBG595203 InParanoid:Q9SV15 OMA:AMQENIS PhylomeDB:Q9SV15 ProtClustDB:CLSN2688388 ArrayExpress:Q9SV15 Genevestigator:Q9SV15 InterPro:IPR018872 Pfam:PF10533 Uniprot:Q9SV15
Root	Isotig05111	6	24	-2.129	0.00029855	TAIR|locus:2125239 - symbol:WRKY11 "AT4G31550" species:3702 "Arabidopsis thaliana" [GO:0003700 "sequence-specific DNA binding transcription factor activity" evidence=ISS] [GO:0005634 "nucleus" evidence=IC] [GO:0006355 "regulation of transcription, DNA-dependent" evidence=ISS] [GO:0005516 "calmodulin binding" evidence=IDA] [GO:0042742 "defense response to bacterium" evidence=IEP] [GO:0010200 "response to chitin" evidence=IEP] InterPro:IPR003657 Pfam:PF03106 PROSITE:PS50811 SMART:SM00774 EMBL:CP002687 GenomeReviews:CT486007_GR GO:GO:0005634 GO:GO:0042742 GO:GO:0043565 GO:GO:0003700 GO:GO:0006351 GO:GO:0005516 GO:GO:0010200 EMBL:AL080283 EMBL:AL161579 Gene3D:G3DSA:2.20.25.80 SUPFAM:SSF118290 EMBL:AF404856 EMBL:AY059731 EMBL:AY150394 EMBL:BT001100 EMBL:AY084855 IPI:IPI00516622 IPI:IPI00531377 PIR:T10685 RefSeq:NP_567878.2 RefSeq:NP_849559.1 UniGene:At.24683 ProteinModelPortal:Q9SV15 SMR:Q9SV15 IntAct:Q9SV15 MINT:MINT-1339027 STRING:Q9SV15 EnsemblPlants:AT4G31550.1 GeneID:829282 KEGG:ath:AT4G31550 TAIR:At4g31550 eggNOG:NOG325238

Tissue	Identification	# of reads (stress)	# of reads (control)	Fold Change	p-value	Description
						HOGENOM:HBG595203 InParanoid:Q9SV15 OMA:AMQENIS PhylomeDB:Q9SV15 ProtClustDB:CLSN2688388 ArrayExpress:Q9SV15 Genevestigator:Q9SV15 InterPro:IPR018872 Pfam:PF10533 Uniprot:Q9SV15
Root	Isotig05116	2	16	-3.129	0.000262867	TAIR|locus:2018027 - symbol:SLAH1 "AT1G62280" species:3702 "Arabidopsis thaliana" [GO:0005215 "transporter activity" evidence=ISS] [GO:0016021 "integral to membrane" evidence=ISS] [GO:0005886 "plasma membrane" evidence=IDA] [GO:0006873 "cellular ion homeostasis" evidence=IMP] InterPro:IPR004695 Pfam:PF03595 GO:GO:0016021 EMBL:CP002684 GenomeReviews:CT485782_GR GO:GO:0005886 GO:GO:0006811 GO:GO:0055085 EMBL:AC000375 EMBL:BT004052 EMBL:BT021090 IPI:IPI00517394 PIR:D96649 RefSeq:NP_176418.2 UniGene:At.36280 EnsemblPlants:AT1G62280.1 GeneID:842525 KEGG:ath:AT1G62280 TAIR:At1g62280 eggNOG:NOG130260 InParanoid:Q5E930 OMA:WWAVSFP PhylomeDB:Q5E930 ProtClustDB:CLSN2680771 ArrayExpress:Q84WB2 Genevestigator:Q5E930 Uniprot:Q5E930
Root	Isotig05124	12	34	-1.631	0.000323514	TAIR|locus:2047057 - symbol:SYTA "synaptotagmin A" species:3702 "Arabidopsis thaliana" [GO:0003674 "molecular_function" evidence=ND] [GO:0006612 "protein targeting to membrane" evidence=ISS] [GO:0005773 "vacuole" evidence=IDA] [GO:0005886 "plasma membrane" evidence=IDA] [GO:0016020 "membrane" evidence=IDA] [GO:0001778 "plasma membrane repair" evidence=IMP] [GO:0009409 "response to cold" evidence=IMP] [GO:0009898 "internal side of plasma membrane" evidence=IDA] [GO:0005768 "endosome" evidence=IDA] [GO:0006897 "endocytosis" evidence=IMP] [GO:0009615 "response to virus" evidence=IPI] [GO:0032456 "endocytic recycling" evidence=IMP] [GO:0046740 "spread of virus in host, cell to cell" evidence=IMP] [GO:0009506 "plasmodesma" evidence=IDA] InterPro:IPR000008 InterPro:IPR008973 Pfam:PF00168 SMART:SM00239 GO:GO:0009506 GO:GO:0005773 EMBL:CP002685 GenomeReviews:CT485783_GR GO:GO:0005768 GO:GO:0006897 GO:GO:0009409 GO:GO:0009615 InterPro:IPR018029 SUPFAM:SSF49562 PROSITE:PS50004 eggNOG:COG5038 GO:GO:0032456 GO:GO:0009898 EMBL:AC006234 EMBL:AC006264 InterPro:IPR020477 PRINTS:PR00360 HSSP:P47709 GO:GO:0046740 GO:GO:0001778 HOGENOM:HBG316820 ProtClustDB:CLSN2688294 EMBL:AY045836 EMBL:AY087925 EMBL:BT004371 EMBL:AB102951 EMBL:AJ617630 IPI:IPI00517829 PIR:G84595 RefSeq:NP_565495.1 UniGene:At.24916 ProteinModelPortal:Q9SKR2 SMR:Q9SKR2 STRING:Q9SKR2 PRIDE:Q9SKR2 EnsemblPlants:AT2G20990.1 GeneID:816633 KEGG:ath:AT2G20990 TAIR:At2g20990 InParanoid:Q9SKR2 OMA:EYMWPYL PhylomeDB:Q9SKR2 ArrayExpress:Q9SKR2 Genevestigator:Q9SKR2 Uniprot:Q9SKR2
Root	Isotig05125	11	41	-2.027	4.63E-06	TAIR|locus:2047057 - symbol:SYTA "synaptotagmin A" species:3702 "Arabidopsis thaliana" [GO:0003674 "molecular_function" evidence=ND] [GO:0006612 "protein targeting to membrane" evidence=ISS] [GO:0005773 "vacuole" evidence=IDA] [GO:0005886 "plasma membrane" evidence=IDA] [GO:0016020 "membrane" evidence=IDA] [GO:0001778 "plasma membrane repair" evidence=IMP] [GO:0009409 "response to cold" evidence=IMP] [GO:0009898 "internal side of plasma membrane" evidence=IDA] [GO:0005768 "endosome" evidence=IDA] [GO:0006897 "endocytosis" evidence=IMP] [GO:0009615 "response to virus" evidence=IPI] [GO:0032456 "endocytic recycling" evidence=IMP] [GO:0046740 "spread of virus in host, cell to cell" evidence=IMP] [GO:0009506 "plasmodesma" evidence=IDA] InterPro:IPR000008 InterPro:IPR008973 Pfam:PF00168 SMART:SM00239 GO:GO:0009506 GO:GO:0005773 EMBL:CP002685 GenomeReviews:CT485783_GR GO:GO:0005768 GO:GO:0006897 GO:GO:0009409 GO:GO:0009615 InterPro:IPR018029 SUPFAM:SSF49562 PROSITE:PS50004 eggNOG:COG5038 GO:GO:0032456 GO:GO:0009898 EMBL:AC006234 EMBL:AC006264 InterPro:IPR020477 PRINTS:PR00360 HSSP:P47709 GO:GO:0046740 GO:GO:0001778 HOGENOM:HBG316820 ProtClustDB:CLSN2688294 EMBL:AY045836 EMBL:AY087925 EMBL:BT004371 EMBL:AB102951 EMBL:AJ617630 IPI:IPI00517829 PIR:G84595 RefSeq:NP_565495.1 UniGene:At.24916 ProteinModelPortal:Q9SKR2 SMR:Q9SKR2 STRING:Q9SKR2 PRIDE:Q9SKR2 EnsemblPlants:AT2G20990.1 GeneID:816633 KEGG:ath:AT2G20990 TAIR:At2g20990 InParanoid:Q9SKR2 OMA:EYMWPYL PhylomeDB:Q9SKR2 ArrayExpress:Q9SKR2 Genevestigator:Q9SKR2 Uniprot:Q9SKR2
Root	Isotig05126	11	42	-2.062	2.76E-06	TAIR|locus:2047057 - symbol:SYTA "synaptotagmin A" species:3702 "Arabidopsis thaliana" [GO:0003674 "molecular_function" evidence=ND] [GO:0006612 "protein targeting to membrane" evidence=ISS] [GO:0005773 "vacuole" evidence=IDA] [GO:0005886 "plasma membrane" evidence=IDA] [GO:0016020 "membrane" evidence=IDA] [GO:0001778 "plasma membrane repair" evidence=IMP] [GO:0009409 "response to cold" evidence=IMP] [GO:0009898 "internal side of plasma membrane" evidence=IDA] [GO:0005768 "endosome" evidence=IDA] [GO:0006897 "endocytosis" evidence=IMP] [GO:0009615 "response to virus" evidence=IPI] [GO:0032456 "endocytic recycling" evidence=IMP] [GO:0046740 "spread of virus in host, cell to cell" evidence=IMP] [GO:0009506 "plasmodesma" evidence=IDA] InterPro:IPR000008 InterPro:IPR008973 Pfam:PF00168

Tissue	Identification	# of reads (stress)	# of reads (control)	Fold Change	p-value	Description
						SMART:SM00239 GO:GO:0009506 GO:GO:0005773 EMBL:CP002685 GenomeReviews:CT485783_GR GO:GO:0005768 GO:GO:0006897 GO:GO:0009409 GO:GO:0009615 InterPro:IPR018029 SUPFAM:SSF49562 PROSITE:PS50004 eggNOG:COG5038 GO:GO:0032456 GO:GO:0009898 EMBL:AC006234 EMBL:AC006264 InterPro:IPR020477 PRINTS:PR00360 HSSP:P47709 GO:GO:0046740 GO:GO:0001778 HOGENOM:HBG316820 ProtClustDB:CLSN2688294 EMBL:AY045836 EMBL:AY087925 EMBL:BT004371 EMBL:AB102951 EMBL:AJ617630 IPI:IPI00517829 PIR:G84595 RefSeq:NP_565495.1 UniGene:At.24916 ProteinModelPortal:Q9SKR2 SMR:Q9SKR2 STRING:Q9SKR2 PRIDE:Q9SKR2 EnsemblPlants:AT2G20990.1 GeneID:816633 KEGG:ath:AT2G20990 TAIR:At2g20990 InParanoid:Q9SKR2 OMA:EYMWPYL PhylomeDB:Q9SKR2 ArrayExpress:Q9SKR2 Genevestigator:Q9SKR2 Uniprot:Q9SKR2
Root	Isotig05133	0	11	-4.588	0.000569522	TAIR|locus:2180454 - symbol:CPN20 "AT5G20720" species:3702 "Arabidopsis thaliana" [GO:0009507 "chloroplast" evidence=ISS;IDA] [GO:0005516 "calmodulin binding" evidence=TAS] [GO:0009579 "thylakoid" evidence=IDA] [GO:0009941 "chloroplast envelope" evidence=IDA] [GO:0009409 "response to cold" evidence=IEP] [GO:0009535 "chloroplast thylakoid membrane" evidence=IDA] [GO:0009570 "chloroplast stroma" evidence=IDA] [GO:0046686 "response to cadmium ion" evidence=IEP] [GO:0005739 "mitochondrion" evidence=IDA] [GO:0048046 "apoplast" evidence=IDA] [GO:0005507 "copper ion binding" evidence=IDA] [GO:0005829 "cytosol" evidence=RCA] InterPro:IPR018369 InterPro:IPR020818 Pfam:PF00166 PRINTS:PR00297 PROSITE:PS00681 SMART:SM00883 GO:GO:0005739 GO:GO:0005524 GO:GO:0048046 GO:GO:0009570 GO:GO:0046686 EMBL:CP002688 GenomeReviews:BA000015_GR GO:GO:0006457 GO:GO:0009941 GO:GO:0005507 GO:GO:0009409 InterPro:IPR011032 SUPFAM:SSF50129 GO:GO:0009535 EMBL:AF296832 UniGene:At.49045 UniGene:At.59645 eggNOG:COG0234 Gene3D:G3DSA:2.30.33.40 PANTHER:PTHR10772 EMBL:AJ010818 EMBL:AF268068 EMBL:AF059037 EMBL:AB007130 EMBL:AY062971 EMBL:AY034979 EMBL:AF428366 EMBL:AF428339 IPI:IPI00537354 PIR:T52122 PIR:T52613 RefSeq:NP_001190350.1 RefSeq:NP_197572.1 RefSeq:NP_851045.1 ProteinModelPortal:O65282 SMR:O65282 IntAct:O65282 STRING:O65282 SWISS-2DPAGE:O65282 PRIDE:O65282 ProMEX:O65282 EnsemblPlants:AT5G20720.1 EnsemblPlants:AT5G20720.2 EnsemblPlants:AT5G20720.3 GeneID:832195 KEGG:ath:AT5G20720 TAIR:At5g20720 HOGENOM:HBG606344 InParanoid:O65282 OMA:RVCSSRR PhylomeDB:O65282 ProtClustDB:CLSN2687389 ArrayExpress:O65282 Genevestigator:O65282 GermOnline:AT5G20720 InterPro:IPR017416 PIRSF:PIRSF038157 Uniprot:O65282
Root	Isotig05134	0	11	-4.588	0.000569522	TAIR|locus:2180454 - symbol:CPN20 "AT5G20720" species:3702 "Arabidopsis thaliana" [GO:0009507 "chloroplast" evidence=ISS;IDA] [GO:0005516 "calmodulin binding" evidence=TAS] [GO:0009579 "thylakoid" evidence=IDA] [GO:0009941 "chloroplast envelope" evidence=IDA] [GO:0009409 "response to cold" evidence=IEP] [GO:0009535 "chloroplast thylakoid membrane" evidence=IDA] [GO:0009570 "chloroplast stroma" evidence=IDA] [GO:0046686 "response to cadmium ion" evidence=IEP] [GO:0005739 "mitochondrion" evidence=IDA] [GO:0048046 "apoplast" evidence=IDA] [GO:0005507 "copper ion binding" evidence=IDA] [GO:0005829 "cytosol" evidence=RCA] InterPro:IPR018369 InterPro:IPR020818 Pfam:PF00166 PRINTS:PR00297 PROSITE:PS00681 SMART:SM00883 GO:GO:0005739 GO:GO:0005524 GO:GO:0048046 GO:GO:0009570 GO:GO:0046686 EMBL:CP002688 GenomeReviews:BA000015_GR GO:GO:0006457 GO:GO:0009941 GO:GO:0005507 GO:GO:0009409 InterPro:IPR011032 SUPFAM:SSF50129 GO:GO:0009535 EMBL:AF296832 UniGene:At.49045 UniGene:At.59645 eggNOG:COG0234 Gene3D:G3DSA:2.30.33.40 PANTHER:PTHR10772 EMBL:AJ010818 EMBL:AF268068 EMBL:AF059037 EMBL:AB007130 EMBL:AY062971 EMBL:AY034979 EMBL:AF428366 EMBL:AF428339 IPI:IPI00537354 PIR:T52122 PIR:T52613 RefSeq:NP_001190350.1 RefSeq:NP_197572.1 RefSeq:NP_851045.1 ProteinModelPortal:O65282 SMR:O65282 IntAct:O65282 STRING:O65282 SWISS-2DPAGE:O65282 PRIDE:O65282 ProMEX:O65282 EnsemblPlants:AT5G20720.1 EnsemblPlants:AT5G20720.2 EnsemblPlants:AT5G20720.3 GeneID:832195 KEGG:ath:AT5G20720 TAIR:At5g20720 HOGENOM:HBG606344 InParanoid:O65282 OMA:RVCSSRR PhylomeDB:O65282 ProtClustDB:CLSN2687389 ArrayExpress:O65282 Genevestigator:O65282 GermOnline:AT5G20720 InterPro:IPR017416 PIRSF:PIRSF038157 Uniprot:O65282
Root	Isotig05135	0	11	-4.588	0.000569522	TAIR|locus:2180454 - symbol:CPN20 "AT5G20720" species:3702 "Arabidopsis thaliana" [GO:0009507 "chloroplast" evidence=ISS;IDA] [GO:0005516 "calmodulin binding" evidence=TAS] [GO:0009579 "thylakoid" evidence=IDA] [GO:0009941 "chloroplast envelope" evidence=IDA] [GO:0009409 "response to cold" evidence=IEP] [GO:0009535 "chloroplast thylakoid membrane" evidence=IDA] [GO:0009570 "chloroplast stroma" evidence=IDA] [GO:0046686 "response to cadmium ion" evidence=IEP] [GO:0005739 "mitochondrion" evidence=IDA] [GO:0048046 "apoplast" evidence=IDA] [GO:0005507 "copper ion binding" evidence=IDA] [GO:0005829 "cytosol" evidence=RCA] InterPro:IPR018369 InterPro:IPR020818 Pfam:PF00166 PRINTS:PR00297 PROSITE:PS00681 SMART:SM00883

Tissue	Identification	# of reads (stress)	# of reads (control)	Fold Change	p-value	Description
						GO:GO:0005739 GO:GO:0005524 GO:GO:0048046 GO:GO:0009570 GO:GO:0046686 EMBL:CP002688 GenomeReviews:BA000015_GR GO:GO:0006457 GO:GO:0009941 GO:GO:0005507 GO:GO:0009409 InterPro:IPR011032 SUPFAM:SSF50129 GO:GO:0009535 EMBL:AF296832 UniGene:At.49045 UniGene:At.59645 eggNOG:COG0234 Gene3D:G3DSA:2.30.33.40 PANTHER:PTHR10772 EMBL:AJ010818 EMBL:AF268068 EMBL:AF059037 EMBL:AB007130 EMBL:AY062971 EMBL:AY034979 EMBL:AF428366 EMBL:AF428339 IPI:IPI00537354 PIR:T52122 PIR:T52613 RefSeq:NP_001190350.1 RefSeq:NP_197572.1 RefSeq:NP_851045.1 ProteinModelPortal:O65282 SMR:O65282 IntAct:O65282 STRING:O65282 SWISS-2DPAGE:O65282 PRIDE:O65282 ProMEX:O65282 EnsemblPlants:AT5G20720.1 EnsemblPlants:AT5G20720.2 EnsemblPlants:AT5G20720.3 GeneID:832195 KEGG:ath:AT5G20720 TAIR:At5g20720 HOGENOM:HBG606344 InParanoid:O65282 OMA:RVCSSRR PhylomeDB:O65282 ProtClustDB:CLSN2687389 ArrayExpress:O65282 Genevestigator:O65282 GermOnline:AT5G20720 InterPro:IPR017416 PIRSF:PIRSF038157 Uniprot:O65282
Root	Isotig05160	11	85	-3.079	6.87E-17	TAIR|locus:2126026 - symbol:PIP1;4 "AT4G00430" species:3702 "Arabidopsis thaliana" [GO:0006810 "transport" evidence=ISS] [GO:0015250 "water channel activity" evidence=ISS] [GO:0016020 "membrane" evidence=ISS;IDA] [GO:0005886 "plasma membrane" evidence=IDA] [GO:0009414 "response to water deprivation" evidence=IEP] InterPro:IPR000425 InterPro:IPR012269 InterPro:IPR022357 Pfam:PF00230 PRINTS:PR00783 PROSITE:PS00221 GO:GO:0016021 GO:GO:0005886 EMBL:CP002687 GenomeReviews:CT486007_GR GO:GO:0055085 GO:GO:0009414 GO:GO:0005215 GO:GO:0006833 EMBL:AF013293 EMBL:AF195115 EMBL:AL161471 InterPro:IPR023271 Gene3D:G3DSA:1.20.1080.10 SUPFAM:SSF81338 eggNOG:COG0580 HOGENOM:HBG705794 PANTHER:PTHR19139 TIGRFAMs:TIGR00861 OMA:ITHNFKD KO:K09872 ProtClustDB:CLSN2682869 UniGene:At.20518 UniGene:At.20892 EMBL:D26609 EMBL:D85192 EMBL:AY099825 EMBL:AY120785 EMBL:BT000330 EMBL:BT006313 IPI:IPI00521990 PIR:T01528 RefSeq:NP_567178.1 ProteinModelPortal:Q39196 SMR:Q39196 IntAct:Q39196 STRING:Q39196 PRIDE:Q39196 EnsemblPlants:AT4G00430.1 GeneID:827956 KEGG:ath:AT4G00430 GeneFarm:2265 TAIR:At4g00430 InParanoid:Q39196 PhylomeDB:Q39196 ArrayExpress:Q39196 Genevestigator:Q39196 Uniprot:Q39196
Root	Isotig05161	7	31	-2.276	1.84E-05	TAIR|locus:2126026 - symbol:PIP1;4 "AT4G00430" species:3702 "Arabidopsis thaliana" [GO:0006810 "transport" evidence=ISS] [GO:0015250 "water channel activity" evidence=ISS] [GO:0016020 "membrane" evidence=ISS;IDA] [GO:0005886 "plasma membrane" evidence=IDA] [GO:0009414 "response to water deprivation" evidence=IEP] InterPro:IPR000425 InterPro:IPR012269 InterPro:IPR022357 Pfam:PF00230 PRINTS:PR00783 PROSITE:PS00221 GO:GO:0016021 GO:GO:0005886 EMBL:CP002687 GenomeReviews:CT486007_GR GO:GO:0055085 GO:GO:0009414 GO:GO:0005215 GO:GO:0006833 EMBL:AF013293 EMBL:AF195115 EMBL:AL161471 InterPro:IPR023271 Gene3D:G3DSA:1.20.1080.10 SUPFAM:SSF81338 eggNOG:COG0580 HOGENOM:HBG705794 PANTHER:PTHR19139 TIGRFAMs:TIGR00861 OMA:ITHNFKD KO:K09872 ProtClustDB:CLSN2682869 UniGene:At.20518 UniGene:At.20892 EMBL:D26609 EMBL:D85192 EMBL:AY099825 EMBL:AY120785 EMBL:BT000330 EMBL:BT006313 IPI:IPI00521990 PIR:T01528 RefSeq:NP_567178.1 ProteinModelPortal:Q39196 SMR:Q39196 IntAct:Q39196 STRING:Q39196 PRIDE:Q39196 EnsemblPlants:AT4G00430.1 GeneID:827956 KEGG:ath:AT4G00430 GeneFarm:2265 TAIR:At4g00430 InParanoid:Q39196 PhylomeDB:Q39196 ArrayExpress:Q39196 Genevestigator:Q39196 Uniprot:Q39196
Root	Isotig05172	31	0	5.825	1.45E-08	TAIR|locus:2035109 - symbol:LEA7 "AT1G52690" species:3702 "Arabidopsis thaliana" [GO:0003674 "molecular_function" evidence=ND] [GO:0005575 "cellular_component" evidence=ND] [GO:0009793 "embryo development ending in seed dormancy" evidence=ISS] EMBL:CP002684 EMBL:AC008016 EMBL:AF385732 EMBL:AY093968 EMBL:AY087466 EMBL:BT000737 EMBL:BT000739 EMBL:X91919 IPI:IPI00526817 PIR:H96567 RefSeq:NP_175678.1 RefSeq:NP_974009.1 UniGene:At.21275 STRING:Q96270 PRIDE:Q96270 ProMEX:Q96270 DNASU:841701 EnsemblPlants:AT1G52690.1 EnsemblPlants:AT1G52690.2 GeneID:841701 KEGG:ath:AT1G52690 TAIR:At1g52690 InParanoid:Q96270 OMA:MASHQEQ PhylomeDB:Q96270 ProtClustDB:CLSN2679745 ArrayExpress:Q96270 Genevestigator:Q96270 Uniprot:Q96270
Root	Isotig05173	22	0	5.330	1.93E-06	TAIR|locus:2035109 - symbol:LEA7 "AT1G52690" species:3702 "Arabidopsis thaliana" [GO:0003674 "molecular_function" evidence=ND] [GO:0005575 "cellular_component" evidence=ND] [GO:0009793 "embryo development ending in seed dormancy" evidence=ISS] EMBL:CP002684 EMBL:AC008016 EMBL:AF385732 EMBL:AY093968 EMBL:AY087466 EMBL:BT000737 EMBL:BT000739 EMBL:X91919 IPI:IPI00526817 PIR:H96567 RefSeq:NP_175678.1 RefSeq:NP_974009.1 UniGene:At.21275 STRING:Q96270 PRIDE:Q96270 ProMEX:Q96270 DNASU:841701 EnsemblPlants:AT1G52690.1 EnsemblPlants:AT1G52690.2 GeneID:841701

Tissue	Identification	# of reads (stress)	# of reads (control)	Fold Change	p-value	Description
						KEGG:ath:AT1G52690 TAIR:At1g52690 InParanoid:Q96270 OMA:MASHQEQ PhylomeDB:Q96270 ProtClustDB:CLSN2679745 ArrayExpress:Q96270 Genevestigator:Q96270 Uniprot:Q96270
Root	Isotig05174	18	0	5.041	1.86E-05	TAIR|locus:2035109 - symbol:LEA7 "AT1G52690" species:3702 "Arabidopsis thaliana" [GO:0003674 "molecular_function" evidence=ND] [GO:0005575 "cellular_component" evidence=ND] [GO:0009793 "embryo development ending in seed dormancy" evidence=ISS] EMBL:CP002684 EMBL:AC008016 EMBL:AF385732 EMBL:AY093968 EMBL:AY087466 EMBL:BT000737 EMBL:BT000739 EMBL:X91919 IPI:IPI00526817 PIR:H96567 RefSeq:NP_175678.1 RefSeq:NP_974009.1 UniGene:At.21275 STRING:Q96270 PRIDE:Q96270 ProMEX:Q96270 DNASU:841701 EnsemblPlants:AT1G52690.1 EnsemblPlants:AT1G52690.2 GeneID:841701 KEGG:ath:AT1G52690 TAIR:At1g52690 InParanoid:Q96270 OMA:MASHQEQ PhylomeDB:Q96270 ProtClustDB:CLSN2679745 ArrayExpress:Q96270 Genevestigator:Q96270 Uniprot:Q96270
Root	Isotig05179	30	7	1.971	0.000234532	No hit
Root	Isotig05181	32	0	5.871	8.51E-09	TAIR|locus:2010419 - symbol:AT1G27990 "AT1G27990" species:3702 "Arabidopsis thaliana" [GO:0003674 "molecular_function" evidence=ND] [GO:0005575 "cellular_component" evidence=ND] [GO:0008150 "biological_process" evidence=ND] EMBL:CP002684 GenomeReviews:CT485782_GR EMBL:AC069471 UniGene:At.66881 EMBL:AK317213 IPI:IPI00523350 PIR:D86405 RefSeq:NP_174120.1 PRIDE:Q9C7F4 EnsemblPlants:AT1G27990.1 GeneID:839692 KEGG:ath:AT1G27990 TAIR:At1g27990 HOGENOM:HBG319949 InParanoid:Q9C7F4 OMA:IPRFLPR PhylomeDB:Q9C7F4 ProtClustDB:CLSN2679353 Genevestigator:Q9C7F4 Uniprot:Q9C7F4
Root	Isotig05182	33	0	5.915	5.02E-09	TAIR|locus:2010419 - symbol:AT1G27990 "AT1G27990" species:3702 "Arabidopsis thaliana" [GO:0003674 "molecular_function" evidence=ND] [GO:0005575 "cellular_component" evidence=ND] [GO:0008150 "biological_process" evidence=ND] EMBL:CP002684 GenomeReviews:CT485782_GR EMBL:AC069471 UniGene:At.66881 EMBL:AK317213 IPI:IPI00523350 PIR:D86405 RefSeq:NP_174120.1 PRIDE:Q9C7F4 EnsemblPlants:AT1G27990.1 GeneID:839692 KEGG:ath:AT1G27990 TAIR:At1g27990 HOGENOM:HBG319949 InParanoid:Q9C7F4 OMA:IPRFLPR PhylomeDB:Q9C7F4 ProtClustDB:CLSN2679353 Genevestigator:Q9C7F4 Uniprot:Q9C7F4
Root	Isotig05183	31	0	5.825	1.45E-08	TAIR|locus:2010419 - symbol:AT1G27990 "AT1G27990" species:3702 "Arabidopsis thaliana" [GO:0003674 "molecular_function" evidence=ND] [GO:0005575 "cellular_component" evidence=ND] [GO:0008150 "biological_process" evidence=ND] EMBL:CP002684 GenomeReviews:CT485782_GR EMBL:AC069471 UniGene:At.66881 EMBL:AK317213 IPI:IPI00523350 PIR:D86405 RefSeq:NP_174120.1 PRIDE:Q9C7F4 EnsemblPlants:AT1G27990.1 GeneID:839692 KEGG:ath:AT1G27990 TAIR:At1g27990 HOGENOM:HBG319949 InParanoid:Q9C7F4 OMA:IPRFLPR PhylomeDB:Q9C7F4 ProtClustDB:CLSN2679353 Genevestigator:Q9C7F4 Uniprot:Q9C7F4
Root	Isotig05188	12	0	4.456	0.000626491	TAIR|locus:2062754 - symbol:HB-7 "homeobox 7" species:3702 "Arabidopsis thaliana" [GO:0005634 "nucleus" evidence=IEA;ISS] [GO:0006355 "regulation of transcription, DNA-dependent" evidence=ISS] [GO:0003700 "sequence-specific DNA binding transcription factor activity" evidence=ISS] [GO:0045893 "positive regulation of transcription, DNA-dependent" evidence=IDA] [GO:0009414 "response to water deprivation" evidence=IEP] [GO:0009737 "response to abscisic acid stimulus" evidence=IEP] [GO:0009738 "abscisic acid mediated signaling pathway" evidence=IGI] InterPro:IPR000047 InterPro:IPR001356 InterPro:IPR003106 InterPro:IPR009057 InterPro:IPR017970 Pfam:PF00046 Pfam:PF02183 PRINTS:PR00031 PROSITE:PS00027 PROSITE:PS50071 SMART:SM00389 GO:GO:0005634 EMBL:CP002685 GenomeReviews:CT485783_GR GO:GO:0045893 GO:GO:0009738 GO:GO:0003700 GO:GO:0006351 GO:GO:0009414 Gene3D:G3DSA:1.10.10.60 SUPFAM:SSF46689 GO:GO:0000976 eggNOG:NOG252905 HOGENOM:HBG593038 ProtClustDB:CLSN2683559 EMBL:X67032 EMBL:AC005819 EMBL:AY045826 EMBL:AY091364 IPI:IPI00544746 PIR:H84905 PIR:S47137 RefSeq:NP_182191.1 UniGene:At.136 ProteinModelPortal:P46897 SMR:P46897 STRING:P46897 EnsemblPlants:AT2G46680.1 GeneID:819280 KEGG:ath:AT2G46680 GeneFarm:3966 TAIR:At2g46680 InParanoid:P46897 OMA:ALVIQLQ PhylomeDB:P46897 ArrayExpress:P46897 Genevestigator:P46897 Uniprot:P46897
Root	Isotig05205	54	144	-1.544	1.15E-12	UNIPROTKB|Q43472 - symbol:blt801 "Glycine-rich RNA-binding protein blt801" species:4513 "Hordeum vulgare" [GO:0003697 "single-stranded DNA binding" evidence=IDA] [GO:0003723 "RNA binding" evidence=IDA] [GO:0009409 "response to cold" evidence=IDA] [GO:0009737 "response to abscisic acid stimulus" evidence=IDA] InterPro:IPR000504 InterPro:IPR012677 Pfam:PF00076 PROSITE:PS50102 SMART:SM00360 GO:GO:0009737 GO:GO:0000166 GO:GO:0006397 Gene3D:G3DSA:3.30.70.330 GO:GO:0006351 GO:GO:0009409

Tissue	Identification	# of reads (stress)	# of reads (control)	Fold Change	p-value	Description
						GO:GO:0003723 GO:GO:0003697 EMBL:U49482 PIR:S71453 HSSP:P11940 ProteinModelPortal:Q43472 Gramene:Q43472 Genevestigator:Q43472 Uniprot:Q43472
Root	Isotig05206	57	202	-1.954	3.13E-23	UNIPROTKB|Q43472 - symbol:blt801 "Glycine-rich RNA-binding protein blt801" species:4513 "Hordeum vulgare" [GO:0003697 "single-stranded DNA binding" evidence=IDA] [GO:0003723 "RNA binding" evidence=IDA] [GO:0009409 "response to cold" evidence=IDA] [GO:0009737 "response to abscisic acid stimulus" evidence=IDA] InterPro:IPR000504 InterPro:IPR012677 Pfam:PF00076 PROSITE:PS50102 SMART:SM00360 GO:GO:0009737 GO:GO:0000166 GO:GO:0006397 Gene3D:G3DSA:3.30.70.330 GO:GO:0006351 GO:GO:0009409 GO:GO:0003723 GO:GO:0003697 EMBL:U49482 PIR:S71453 HSSP:P11940 ProteinModelPortal:Q43472 Gramene:Q43472 Genevestigator:Q43472 Uniprot:Q43472
Root	Isotig05207	28	70	-1.451	2.20E-06	UNIPROTKB|Q43472 - symbol:blt801 "Glycine-rich RNA-binding protein blt801" species:4513 "Hordeum vulgare" [GO:0003697 "single-stranded DNA binding" evidence=IDA] [GO:0003723 "RNA binding" evidence=IDA] [GO:0009409 "response to cold" evidence=IDA] [GO:0009737 "response to abscisic acid stimulus" evidence=IDA] InterPro:IPR000504 InterPro:IPR012677 Pfam:PF00076 PROSITE:PS50102 SMART:SM00360 GO:GO:0009737 GO:GO:0000166 GO:GO:0006397 Gene3D:G3DSA:3.30.70.330 GO:GO:0006351 GO:GO:0009409 GO:GO:0003723 GO:GO:0003697 EMBL:U49482 PIR:S71453 HSSP:P11940 ProteinModelPortal:Q43472 Gramene:Q43472 Genevestigator:Q43472 Uniprot:Q43472
Root	Isotig05211	6	56	-3.351	1.97E-12	TAIR|locus:2064332 - symbol:AT2G30620 "AT2G30620" species:3702 "Arabidopsis thaliana" [GO:0000786 "nucleosome" evidence=ISS] [GO:0003677 "DNA binding" evidence=IEA;ISS] [GO:0005634 "nucleus" evidence=ISS] [GO:0006334 "nucleosome assembly" evidence=IEA;ISS] [GO:0005730 "nucleolus" evidence=IDA] InterPro:IPR005818 InterPro:IPR005819 Pfam:PF00538 PRINTS:PR00624 PROSITE:PS51504 SMART:SM00526 EMBL:CP002685 GenomeReviews:CT485783_GR GO:GO:0005730 GO:GO:0003677 InterPro:IPR011991 Gene3D:G3DSA:1.10.10.10 GO:GO:0006334 GO:GO:0000786 EMBL:U93215 KO:K11275 eggNOG:NOG126832 EMBL:X62459 EMBL:AF360211 EMBL:AY040059 EMBL:AY085789 IPI:IPI00532307 PIR:S19699 RefSeq:NP_180620.1 UniGene:At.20406 ProteinModelPortal:P26569 STRING:P26569 PRIDE:P26569 EnsemblPlants:AT2G30620.1 GeneID:817612 KEGG:ath:AT2G30620 TAIR:At2g30620 HOGENOM:HBG184949 InParanoid:P26569 OMA:HRTPSSH PhylomeDB:P26569 ProtClustDB:CLSN2912930 ArrayExpress:P26569 Genevestigator:P26569 GermOnline:AT2G30620 Uniprot:P26569
Root	Isotig05212	5	54	-3.562	1.44E-12	TAIR|locus:2064332 - symbol:AT2G30620 "AT2G30620" species:3702 "Arabidopsis thaliana" [GO:0000786 "nucleosome" evidence=ISS] [GO:0003677 "DNA binding" evidence=IEA;ISS] [GO:0005634 "nucleus" evidence=ISS] [GO:0006334 "nucleosome assembly" evidence=IEA;ISS] [GO:0005730 "nucleolus" evidence=IDA] InterPro:IPR005818 InterPro:IPR005819 Pfam:PF00538 PRINTS:PR00624 PROSITE:PS51504 SMART:SM00526 EMBL:CP002685 GenomeReviews:CT485783_GR GO:GO:0005730 GO:GO:0003677 InterPro:IPR011991 Gene3D:G3DSA:1.10.10.10 GO:GO:0006334 GO:GO:0000786 EMBL:U93215 KO:K11275 eggNOG:NOG126832 EMBL:X62459 EMBL:AF360211 EMBL:AY040059 EMBL:AY085789 IPI:IPI00532307 PIR:S19699 RefSeq:NP_180620.1 UniGene:At.20406 ProteinModelPortal:P26569 STRING:P26569 PRIDE:P26569 EnsemblPlants:AT2G30620.1 GeneID:817612 KEGG:ath:AT2G30620 TAIR:At2g30620 HOGENOM:HBG184949 InParanoid:P26569 OMA:HRTPSSH PhylomeDB:P26569 ProtClustDB:CLSN2912930 ArrayExpress:P26569 Genevestigator:P26569 GermOnline:AT2G30620 Uniprot:P26569
Root	Isotig05213	5	54	-3.562	1.44E-12	TAIR|locus:2064332 - symbol:AT2G30620 "AT2G30620" species:3702 "Arabidopsis thaliana" [GO:0000786 "nucleosome" evidence=ISS] [GO:0003677 "DNA binding" evidence=IEA;ISS] [GO:0005634 "nucleus" evidence=ISS] [GO:0006334 "nucleosome assembly" evidence=IEA;ISS] [GO:0005730 "nucleolus" evidence=IDA] InterPro:IPR005818 InterPro:IPR005819 Pfam:PF00538 PRINTS:PR00624 PROSITE:PS51504 SMART:SM00526 EMBL:CP002685 GenomeReviews:CT485783_GR GO:GO:0005730 GO:GO:0003677 InterPro:IPR011991 Gene3D:G3DSA:1.10.10.10 GO:GO:0006334 GO:GO:0000786 EMBL:U93215 KO:K11275 eggNOG:NOG126832 EMBL:X62459 EMBL:AF360211 EMBL:AY040059 EMBL:AY085789 IPI:IPI00532307 PIR:S19699 RefSeq:NP_180620.1 UniGene:At.20406 ProteinModelPortal:P26569 STRING:P26569 PRIDE:P26569 EnsemblPlants:AT2G30620.1 GeneID:817612 KEGG:ath:AT2G30620 TAIR:At2g30620 HOGENOM:HBG184949 InParanoid:P26569 OMA:HRTPSSH PhylomeDB:P26569 ProtClustDB:CLSN2912930 ArrayExpress:P26569 Genevestigator:P26569 GermOnline:AT2G30620 Uniprot:P26569
Root	Isotig05235	0	64	-7.129	9.20E-17	TAIR|locus:2168953 - symbol:TIP2;3 "AT5G47450" species:3702 "Arabidopsis thaliana" [GO:0006810 "transport" evidence=ISS] [GO:0015250 "water channel activity" evidence=ISS] [GO:0016020 "membrane" evidence=ISS] [GO:0009705 "plant-type vacuole membrane" evidence=IDA] [GO:0015200 "methylammonium transmembrane

Tissue	Identification	# of reads (stress)	# of reads (control)	Fold Change	p-value	Description
						transporter activity" evidence=IDA] [GO:0051739 "ammonia transmembrane transporter activity" evidence=IGI] [GO:0042807 "central vacuole" evidence=IDA] InterPro:IPR000425 InterPro:IPR012269 InterPro:IPR022357 Pfam:PF00230 PRINTS:PR00783 PROSITE:PS00221 GO:GO:0016021 EMBL:CP002688 GenomeReviews:BA000015_GR GO:GO:0009705 EMBL:AB025628 GO:GO:0006833 GO:GO:0015200 GO:GO:0051739 InterPro:IPR023271 Gene3D:G3DSA:1.20.1080.10 SUPFAM:SSF81338 eggNOG:COG0580 HOGENOM:HBG705794 PANTHER:PTHR19139 TIGRFAMs:TIGR00861 KO:K09873 GO:GO:0042807 ProtClustDB:PLN00166 EMBL:BT011212 EMBL:BT011663 EMBL:AK229341 IPI:IPI00541642 RefSeq:NP_199556.1 UniGene:At.43789 ProteinModelPortal:Q9FGL2 SMR:Q9FGL2 STRING:Q9FGL2 TCDB:1.A.8.10.4 PRIDE:Q9FGL2 EnsemblPlants:AT5G47450.1 GeneID:834794 KEGG:ath:AT5G47450 GeneFarm:4858 TAIR:At5g47450 InParanoid:Q9FGL2 OMA:GHIVACL PhylomeDB:Q9FGL2 ArrayExpress:Q9FGL2 Genevestigator:Q9FGL2 GermOnline:AT5G47450 Uniprot:Q9FGL2
Root	Isotig05236	0	64	-7.129	9.20E-17	TAIR|locus:2168953 - symbol:TIP2;3 "AT5G47450" species:3702 "Arabidopsis thaliana" [GO:0006810 "transport" evidence=ISS] [GO:0015250 "water channel activity" evidence=ISS] [GO:0016020 "membrane" evidence=ISS] [GO:0009705 "plant-type vacuole membrane" evidence=IDA] [GO:0015200 "methylammonium transmembrane transporter activity" evidence=IDA] [GO:0051739 "ammonia transmembrane transporter activity" evidence=IGI] [GO:0042807 "central vacuole" evidence=IDA] InterPro:IPR000425 InterPro:IPR012269 InterPro:IPR022357 Pfam:PF00230 PRINTS:PR00783 PROSITE:PS00221 GO:GO:0016021 EMBL:CP002688 GenomeReviews:BA000015_GR GO:GO:0009705 EMBL:AB025628 GO:GO:0006833 GO:GO:0015200 GO:GO:0051739 InterPro:IPR023271 Gene3D:G3DSA:1.20.1080.10 SUPFAM:SSF81338 eggNOG:COG0580 HOGENOM:HBG705794 PANTHER:PTHR19139 TIGRFAMs:TIGR00861 KO:K09873 GO:GO:0042807 ProtClustDB:PLN00166 EMBL:BT011212 EMBL:BT011663 EMBL:AK229341 IPI:IPI00541642 RefSeq:NP_199556.1 UniGene:At.43789 ProteinModelPortal:Q9FGL2 SMR:Q9FGL2 STRING:Q9FGL2 TCDB:1.A.8.10.4 PRIDE:Q9FGL2 EnsemblPlants:AT5G47450.1 GeneID:834794 KEGG:ath:AT5G47450 GeneFarm:4858 TAIR:At5g47450 InParanoid:Q9FGL2 OMA:GHIVACL PhylomeDB:Q9FGL2 ArrayExpress:Q9FGL2 Genevestigator:Q9FGL2 GermOnline:AT5G47450 Uniprot:Q9FGL2
Root	Isotig05237	0	43	-6.555	4.45E-12	TAIR|locus:2168953 - symbol:TIP2;3 "AT5G47450" species:3702 "Arabidopsis thaliana" [GO:0006810 "transport" evidence=ISS] [GO:0015250 "water channel activity" evidence=ISS] [GO:0016020 "membrane" evidence=ISS] [GO:0009705 "plant-type vacuole membrane" evidence=IDA] [GO:0015200 "methylammonium transmembrane transporter activity" evidence=IDA] [GO:0051739 "ammonia transmembrane transporter activity" evidence=IGI] [GO:0042807 "central vacuole" evidence=IDA] InterPro:IPR000425 InterPro:IPR012269 InterPro:IPR022357 Pfam:PF00230 PRINTS:PR00783 PROSITE:PS00221 GO:GO:0016021 EMBL:CP002688 GenomeReviews:BA000015_GR GO:GO:0009705 EMBL:AB025628 GO:GO:0006833 GO:GO:0015200 GO:GO:0051739 InterPro:IPR023271 Gene3D:G3DSA:1.20.1080.10 SUPFAM:SSF81338 eggNOG:COG0580 HOGENOM:HBG705794 PANTHER:PTHR19139 TIGRFAMs:TIGR00861 KO:K09873 GO:GO:0042807 ProtClustDB:PLN00166 EMBL:BT011212 EMBL:BT011663 EMBL:AK229341 IPI:IPI00541642 RefSeq:NP_199556.1 UniGene:At.43789 ProteinModelPortal:Q9FGL2 SMR:Q9FGL2 STRING:Q9FGL2 TCDB:1.A.8.10.4 PRIDE:Q9FGL2 EnsemblPlants:AT5G47450.1 GeneID:834794 KEGG:ath:AT5G47450 GeneFarm:4858 TAIR:At5g47450 InParanoid:Q9FGL2 OMA:GHIVACL PhylomeDB:Q9FGL2 ArrayExpress:Q9FGL2 Genevestigator:Q9FGL2 GermOnline:AT5G47450 Uniprot:Q9FGL2
Root	Isotig05260	5	20	-2.129	0.000961902	TAIR|locus:2032574 - symbol:GRX480 "AT1G28480" species:3702 "Arabidopsis thaliana" [GO:0005575 "cellular_component" evidence=ND] [GO:0005515 "protein binding" evidence=IPI] [GO:0009751 "response to salicylic acid stimulus" evidence=IEP] [GO:0009863 "salicylic acid mediated signaling pathway" evidence=IMP] [GO:0009867 "jasmonic acid mediated signaling pathway" evidence=IMP] InterPro:IPR002109 Pfam:PF00462 PROSITE:PS00195 PROSITE:PS51354 EMBL:CP002684 GenomeReviews:CT485782_GR GO:GO:0005634 GO:GO:0005737 GO:GO:0005515 GO:GO:0009055 GO:GO:0006810 GO:GO:0022900 InterPro:IPR012336 Gene3D:G3DSA:3.40.30.10 SUPFAM:SSF52833 GO:GO:0045454 GO:GO:0015035 GO:GO:0009867 GO:GO:0009863 EMBL:AC010155 HOGENOM:HBG728471 HSSP:Q9CQM9 InterPro:IPR011905 TIGRFAMs:TIGR02189 EMBL:FJ611918 EMBL:AF325030 EMBL:AK229369 EMBL:BT029336 IPI:IPI00535123 PIR:H86410 RefSeq:NP_174170.1 UniGene:At.11829 ProteinModelPortal:Q9SGP6 SMR:Q9SGP6 IntAct:Q9SGP6 STRING:Q9SGP6 EnsemblPlants:AT1G28480.1 GeneID:839748 KEGG:ath:AT1G28480 TAIR:At1g28480 eggNOG:NOG318179 InParanoid:Q9SGP6 OMA:MVVEENA PhylomeDB:Q9SGP6 ProtClustDB:CLSN2913511 Genevestigator:Q9SGP6 GermOnline:AT1G28480 Uniprot:Q9SGP6

Tissue	Identification	# of reads (stress)	# of reads (control)	Fold Change	p-value	Description
Root	Isotig05273	5	24	-2.392	0.000103464	TAIR|locus:2092339 - symbol:ERF7 "AT3G20310" species:3702 "Arabidopsis thaliana" [GO:0003700 "sequence-specific DNA binding transcription factor activity" evidence=ISS;TAS] [GO:0006355 "regulation of transcription, DNA-dependent" evidence=TAS] [GO:0003677 "DNA binding" evidence=TAS] [GO:0005634 "nucleus" evidence=IC;IDA] [GO:0005515 "protein binding" evidence=IPI] [GO:0009414 "response to water deprivation" evidence=IMP] [GO:0009737 "response to abscisic acid stimulus" evidence=IMP] [GO:0043565 "sequence-specific DNA binding" evidence=IDA] [GO:0045892 "negative regulation of transcription, DNA-dependent" evidence=IDA] [GO:0009873 "ethylene mediated signaling pathway" evidence=TAS] InterPro:IPR001471 InterPro:IPR016177 Pfam:PF00847 PRINTS:PR00367 PROSITE:PS51032 SMART:SM00380 GO:GO:0009737 GO:GO:0005634 EMBL:CP002686 GenomeReviews:BA000014_GR GO:GO:0045892 GO:GO:0005515 GO:GO:0006952 GO:GO:0009873 GO:GO:0043565 GO:GO:0003700 GO:GO:0006351 GO:GO:0009414 KO:K09286 Gene3D:G3DSA:3.30.730.10 SUPFAM:SSF54171 EMBL:AB024036 HOGENOM:HBG744062 ProtClustDB:CLSN2682907 EMBL:AB032201 EMBL:AY037254 EMBL:AY094001 EMBL:AY085023 EMBL:AK228460 IPI:IPI00537654 RefSeq:NP_188666.1 UniGene:At.20161 ProteinModelPortal:Q9LDE4 SMR:Q9LDE4 IntAct:Q9LDE4 STRING:Q9LDE4 PRIDE:Q9LDE4 EnsemblPlants:AT3G20310.1 GeneID:821575 KEGG:ath:AT3G20310 TAIR:At3g20310 eggNOG:NOG322216 InParanoid:Q9LDE4 OMA:VAPEDCH PhylomeDB:Q9LDE4 ArrayExpress:Q9LDE4 Genevestigator:Q9LDE4 GermOnline:AT3G20310 Uniprot:Q9LDE4
Root	Isotig05274	5	24	-2.392	0.000103464	TAIR|locus:2092339 - symbol:ERF7 "AT3G20310" species:3702 "Arabidopsis thaliana" [GO:0003700 "sequence-specific DNA binding transcription factor activity" evidence=ISS;TAS] [GO:0006355 "regulation of transcription, DNA-dependent" evidence=TAS] [GO:0003677 "DNA binding" evidence=TAS] [GO:0005634 "nucleus" evidence=IC;IDA] [GO:0005515 "protein binding" evidence=IPI] [GO:0009414 "response to water deprivation" evidence=IMP] [GO:0009737 "response to abscisic acid stimulus" evidence=IMP] [GO:0043565 "sequence-specific DNA binding" evidence=IDA] [GO:0045892 "negative regulation of transcription, DNA-dependent" evidence=IDA] [GO:0009873 "ethylene mediated signaling pathway" evidence=TAS] InterPro:IPR001471 InterPro:IPR016177 Pfam:PF00847 PRINTS:PR00367 PROSITE:PS51032 SMART:SM00380 GO:GO:0009737 GO:GO:0005634 EMBL:CP002686 GenomeReviews:BA000014_GR GO:GO:0045892 GO:GO:0005515 GO:GO:0006952 GO:GO:0009873 GO:GO:0043565 GO:GO:0003700 GO:GO:0006351 GO:GO:0009414 KO:K09286 Gene3D:G3DSA:3.30.730.10 SUPFAM:SSF54171 EMBL:AB024036 HOGENOM:HBG744062 ProtClustDB:CLSN2682907 EMBL:AB032201 EMBL:AY037254 EMBL:AY094001 EMBL:AY085023 EMBL:AK228460 IPI:IPI00537654 RefSeq:NP_188666.1 UniGene:At.20161 ProteinModelPortal:Q9LDE4 SMR:Q9LDE4 IntAct:Q9LDE4 STRING:Q9LDE4 PRIDE:Q9LDE4 EnsemblPlants:AT3G20310.1 GeneID:821575 KEGG:ath:AT3G20310 TAIR:At3g20310 eggNOG:NOG322216 InParanoid:Q9LDE4 OMA:VAPEDCH PhylomeDB:Q9LDE4 ArrayExpress:Q9LDE4 Genevestigator:Q9LDE4 GermOnline:AT3G20310 Uniprot:Q9LDE4
Root	Isotig05275	3	22	-3.003	2.69E-05	TAIR|locus:2092339 - symbol:ERF7 "AT3G20310" species:3702 "Arabidopsis thaliana" [GO:0003700 "sequence-specific DNA binding transcription factor activity" evidence=ISS;TAS] [GO:0006355 "regulation of transcription, DNA-dependent" evidence=TAS] [GO:0003677 "DNA binding" evidence=TAS] [GO:0005634 "nucleus" evidence=IC;IDA] [GO:0005515 "protein binding" evidence=IPI] [GO:0009414 "response to water deprivation" evidence=IMP] [GO:0009737 "response to abscisic acid stimulus" evidence=IMP] [GO:0043565 "sequence-specific DNA binding" evidence=IDA] [GO:0045892 "negative regulation of transcription, DNA-dependent" evidence=IDA] [GO:0009873 "ethylene mediated signaling pathway" evidence=TAS] InterPro:IPR001471 InterPro:IPR016177 Pfam:PF00847 PRINTS:PR00367 PROSITE:PS51032 SMART:SM00380 GO:GO:0009737 GO:GO:0005634 EMBL:CP002686 GenomeReviews:BA000014_GR GO:GO:0045892 GO:GO:0005515 GO:GO:0006952 GO:GO:0009873 GO:GO:0043565 GO:GO:0003700 GO:GO:0006351 GO:GO:0009414 KO:K09286 Gene3D:G3DSA:3.30.730.10 SUPFAM:SSF54171 EMBL:AB024036 HOGENOM:HBG744062 ProtClustDB:CLSN2682907 EMBL:AB032201 EMBL:AY037254 EMBL:AY094001 EMBL:AY085023 EMBL:AK228460 IPI:IPI00537654 RefSeq:NP_188666.1 UniGene:At.20161 ProteinModelPortal:Q9LDE4 SMR:Q9LDE4 IntAct:Q9LDE4 STRING:Q9LDE4 PRIDE:Q9LDE4 EnsemblPlants:AT3G20310.1 GeneID:821575 KEGG:ath:AT3G20310 TAIR:At3g20310 eggNOG:NOG322216 InParanoid:Q9LDE4 OMA:VAPEDCH PhylomeDB:Q9LDE4 ArrayExpress:Q9LDE4 Genevestigator:Q9LDE4 GermOnline:AT3G20310 Uniprot:Q9LDE4
Root	Isotig05276	257	6	5.292	1.85E-59	TAIR|locus:2123919 - symbol:CFIM-25 "AT4G29820" species:3702 "Arabidopsis thaliana" [GO:0005575 "cellular_component" evidence=ND] [GO:0008150 "biological_process" evidence=ND] InterPro:IPR000086 InterPro:IPR015797 InterPro:IPR016706 PIRSF:PIRSF017888 EMBL:CP002687 GenomeReviews:CT486007_GR GO:GO:0005515 Gene3D:G3DSA:3.90.79.10 SUPFAM:SSF55811 GO:GO:0016787 PANTHER:PTHR13047

Tissue	Identification	# of reads (stress)	# of reads (control)	Fold Change	p-value	Description
						HOGENOM:HBG629877 ProtClustDB:CLSN2689678 EMBL:AY048229 EMBL:AY091704 EMBL:AY085984 IPI:IPI00532079 RefSeq:NP_567835.1 UniGene:At.26190 ProteinModelPortal:Q94AF0 IntAct:Q94AF0 STRING:Q94AF0 PRIDE:Q94AF0 EnsemblPlants:AT4G29820.1 GeneID:829104 KEGG:ath:AT4G29820 TAIR:At4g29820 eggNOG:NOG303614 InParanoid:Q94AF0 OMA:GECIGMW PhylomeDB:Q94AF0 Genevestigator:Q94AF0 Uniprot:Q94AF0
Root	Isotig05277	257	6	5.292	1.85E-59	TAIR|locus:2123919 - symbol:CFIM-25 "AT4G29820" species:3702 "Arabidopsis thaliana" [GO:0005575 "cellular_component" evidence=ND] [GO:0008150 "biological_process" evidence=ND] InterPro:IPR000086 InterPro:IPR015797 InterPro:IPR016706 PIRSF:PIRSF017888 EMBL:CP002687 GenomeReviews:CT486007_GR GO:GO:0005515 Gene3D:G3DSA:3.90.79.10 SUPFAM:SSF55811 GO:GO:0016787 PANTHER:PTHR13047 HOGENOM:HBG629877 ProtClustDB:CLSN2689678 EMBL:AY048229 EMBL:AY091704 EMBL:AY085984 IPI:IPI00532079 RefSeq:NP_567835.1 UniGene:At.26190 ProteinModelPortal:Q94AF0 IntAct:Q94AF0 STRING:Q94AF0 PRIDE:Q94AF0 EnsemblPlants:AT4G29820.1 GeneID:829104 KEGG:ath:AT4G29820 TAIR:At4g29820 eggNOG:NOG303614 InParanoid:Q94AF0 OMA:GECIGMW PhylomeDB:Q94AF0 Genevestigator:Q94AF0 Uniprot:Q94AF0
Root	Isotig05291	1	21	-4.521	2.07E-06	TAIR|locus:2009130 - symbol:AT1G06620 "AT1G06620" species:3702 "Arabidopsis thaliana" [GO:0005575 "cellular_component" evidence=ND] [GO:0008150 "biological_process" evidence=ND] [GO:0016706 "oxidoreductase activity, acting on paired donors, with incorporation or reduction of molecular oxygen, 2-oxoglutarate as one donor, and incorporation of one atom each of oxygen into both donors" evidence=ISS] InterPro:IPR005123 Pfam:PF03171 PROSITE:PS51471 EMBL:CP002684 GenomeReviews:CT485782_GR GO:GO:0046872 EMBL:AC007592 EMBL:BT006430 EMBL:AK227676 IPI:IPI00533375 PIR:D86201 RefSeq:NP_172147.2 UniGene:At.42332 HSSP:Q08506 ProteinModelPortal:Q84MB3 SMR:Q84MB3 PRIDE:Q84MB3 EnsemblPlants:AT1G06620.1 GeneID:837171 KEGG:ath:AT1G06620 TAIR:At1g06620 eggNOG:COG3491 HOGENOM:HBG749762 InParanoid:Q84MB3 OMA:CEMMIEY PhylomeDB:Q84MB3 ProtClustDB:CLSN2685246 Genevestigator:Q84MB3 GO:GO:0016706 Uniprot:Q84MB3
Root	Isotig05292	1	17	-4.216	2.69E-05	TAIR|locus:2009130 - symbol:AT1G06620 "AT1G06620" species:3702 "Arabidopsis thaliana" [GO:0005575 "cellular_component" evidence=ND] [GO:0008150 "biological_process" evidence=ND] [GO:0016706 "oxidoreductase activity, acting on paired donors, with incorporation or reduction of molecular oxygen, 2-oxoglutarate as one donor, and incorporation of one atom each of oxygen into both donors" evidence=ISS] InterPro:IPR005123 Pfam:PF03171 PROSITE:PS51471 EMBL:CP002684 GenomeReviews:CT485782_GR GO:GO:0046872 EMBL:AC007592 EMBL:BT006430 EMBL:AK227676 IPI:IPI00533375 PIR:D86201 RefSeq:NP_172147.2 UniGene:At.42332 HSSP:Q08506 ProteinModelPortal:Q84MB3 SMR:Q84MB3 PRIDE:Q84MB3 EnsemblPlants:AT1G06620.1 GeneID:837171 KEGG:ath:AT1G06620 TAIR:At1g06620 eggNOG:COG3491 HOGENOM:HBG749762 InParanoid:Q84MB3 OMA:CEMMIEY PhylomeDB:Q84MB3 ProtClustDB:CLSN2685246 Genevestigator:Q84MB3 GO:GO:0016706 Uniprot:Q84MB3
Root	Isotig05318	1	28	-4.936	2.57E-08	TAIR|locus:2086445 - symbol:NQR "AT3G27890" species:3702 "Arabidopsis thaliana" [GO:0008752 "FMN reductase activity" evidence=ISS] [GO:0005886 "plasma membrane" evidence=IDA] [GO:0042742 "defense response to bacterium" evidence=IEP] [GO:0009651 "response to salt stress" evidence=IEP] [GO:0009507 "chloroplast" evidence=IDA] [GO:0009570 "chloroplast stroma" evidence=IDA] [GO:0005829 "cytosol" evidence=IDA] Pfam:PF03358 GO:GO:0005829 GO:GO:0005886 GO:GO:0009570 GO:GO:0042742 EMBL:CP002686 GenomeReviews:BA000014_GR GO:GO:0009651 EMBL:AP000371 GO:GO:0003955 eggNOG:COG0431 HOGENOM:HBG697130 InterPro:IPR005025 EMBL:AF145234 IPI:IPI00522027 RefSeq:NP_189427.1 UniGene:At.5735 ProteinModelPortal:Q9LK88 SMR:Q9LK88 PRIDE:Q9LK88 EnsemblPlants:AT3G27890.1 GeneID:822411 KEGG:ath:AT3G27890 TAIR:At3g27890 InParanoid:Q9LK88 OMA:ASPEYNF PhylomeDB:Q9LK88 ProtClustDB:CLSN2685191 Genevestigator:Q9LK88 GermOnline:AT3G27890 Uniprot:Q9LK88
Root	Isotig05319	1	18	-4.299	1.41E-05	TAIR|locus:2086445 - symbol:NQR "AT3G27890" species:3702 "Arabidopsis thaliana" [GO:0008752 "FMN reductase activity" evidence=ISS] [GO:0005886 "plasma membrane" evidence=IDA] [GO:0042742 "defense response to bacterium" evidence=IEP] [GO:0009651 "response to salt stress" evidence=IEP] [GO:0009507 "chloroplast" evidence=IDA] [GO:0009570 "chloroplast stroma" evidence=IDA] [GO:0005829 "cytosol" evidence=IDA] Pfam:PF03358 GO:GO:0005829 GO:GO:0005886 GO:GO:0009570 GO:GO:0042742 EMBL:CP002686 GenomeReviews:BA000014_GR GO:GO:0009651 EMBL:AP000371 GO:GO:0003955 eggNOG:COG0431 HOGENOM:HBG697130 InterPro:IPR005025 EMBL:AF145234 IPI:IPI00522027

Tissue	Identification	# of reads (stress)	# of reads (control)	Fold Change	p-value	Description
						RefSeq:NP_189427.1 UniGene:At.5735 ProteinModelPortal:Q9LK88 SMR:Q9LK88 PRIDE:Q9LK88 EnsemblPlants:AT3G27890.1 GeneID:822411 KEGG:ath:AT3G27890 TAIR:At3g27890 InParanoid:Q9LK88 OMA:ASPEYNF PhylomeDB:Q9LK88 ProtClustDB:CLSN2685191 Genevestigator:Q9LK88 GermOnline:AT3G27890 Uniprot:Q9LK88
Root	Isotig05320	1	18	-4.299	1.41E-05	TAIR|locus:2086445 - symbol:NQR "AT3G27890" species:3702 "Arabidopsis thaliana" [GO:0008752 "FMN reductase activity" evidence=ISS] [GO:0005886 "plasma membrane" evidence=IDA] [GO:0042742 "defense response to bacterium" evidence=IEP] [GO:0009651 "response to salt stress" evidence=IEP] [GO:0009507 "chloroplast" evidence=IDA] [GO:0009570 "chloroplast stroma" evidence=IDA] [GO:0005829 "cytosol" evidence=IDA] Pfam:PF03358 GO:GO:0005829 GO:GO:0005886 GO:GO:0009570 GO:GO:0042742 EMBL:CP002686 GenomeReviews:BA000014_GR GO:GO:0009651 EMBL:AP000371 GO:GO:0003955 eggNOG:COG0431 HOGENOM:HBG697130 InterPro:IPR005025 EMBL:AF145234 IPI:IPI00522027 RefSeq:NP_189427.1 UniGene:At.5735 ProteinModelPortal:Q9LK88 SMR:Q9LK88 PRIDE:Q9LK88 EnsemblPlants:AT3G27890.1 GeneID:822411 KEGG:ath:AT3G27890 TAIR:At3g27890 InParanoid:Q9LK88 OMA:ASPEYNF PhylomeDB:Q9LK88 ProtClustDB:CLSN2685191 Genevestigator:Q9LK88 GermOnline:AT3G27890 Uniprot:Q9LK88
Root	Isotig05321	0	16	-5.129	2.34E-05	TAIR|locus:2195733 - symbol:BAN "AT1G61720" species:3702 "Arabidopsis thaliana" [GO:0033729 "anthocyanidin reductase activity" evidence=IDA] [GO:0009964 "negative regulation of flavonoid biosynthetic process" evidence=IMP] [GO:0016491 "oxidoreductase activity" evidence=ISS] InterPro:IPR001509 InterPro:IPR016040 Pfam:PF01370 EMBL:CP002684 GenomeReviews:CT485782_GR eggNOG:COG0451 GO:GO:0000166 Gene3D:G3DSA:3.40.50.720 HOGENOM:HBG755066 GO:GO:0050662 EMBL:AF092912 EMBL:AC005882 EMBL:DQ446384 EMBL:AK175960 IPI:IPI00523362 PIR:H96642 RefSeq:NP_176365.1 UniGene:At.11057 ProteinModelPortal:Q9SEV0 SMR:Q9SEV0 STRING:Q9SEV0 PRIDE:Q9SEV0 EnsemblPlants:AT1G61720.1 GeneID:842469 KEGG:ath:AT1G61720 TAIR:At1g61720 InParanoid:Q9SEV0 KO:K08695 OMA:ICCAYNT PhylomeDB:Q9SEV0 ProtClustDB:PLN00198 Genevestigator:Q9SEV0 GO:GO:0033729 GO:GO:0009813 GO:GO:0009964 Uniprot:Q9SEV0
Root	Isotig05322	0	18	-5.299	6.77E-06	TAIR|locus:2195733 - symbol:BAN "AT1G61720" species:3702 "Arabidopsis thaliana" [GO:0033729 "anthocyanidin reductase activity" evidence=IDA] [GO:0009964 "negative regulation of flavonoid biosynthetic process" evidence=IMP] [GO:0016491 "oxidoreductase activity" evidence=ISS] InterPro:IPR001509 InterPro:IPR016040 Pfam:PF01370 EMBL:CP002684 GenomeReviews:CT485782_GR eggNOG:COG0451 GO:GO:0000166 Gene3D:G3DSA:3.40.50.720 HOGENOM:HBG755066 GO:GO:0050662 EMBL:AF092912 EMBL:AC005882 EMBL:DQ446384 EMBL:AK175960 IPI:IPI00523362 PIR:H96642 RefSeq:NP_176365.1 UniGene:At.11057 ProteinModelPortal:Q9SEV0 SMR:Q9SEV0 STRING:Q9SEV0 PRIDE:Q9SEV0 EnsemblPlants:AT1G61720.1 GeneID:842469 KEGG:ath:AT1G61720 TAIR:At1g61720 InParanoid:Q9SEV0 KO:K08695 OMA:ICCAYNT PhylomeDB:Q9SEV0 ProtClustDB:PLN00198 Genevestigator:Q9SEV0 GO:GO:0033729 GO:GO:0009813 GO:GO:0009964 Uniprot:Q9SEV0
Root	Isotig05325	1	20	-4.451	3.92E-06	No hit
Root	Isotig05326	1	20	-4.451	3.92E-06	No hit
Root	Isotig05333	13	141	-3.568	2.37E-30	TAIR|locus:2057906 - symbol:GAMMA-TIP "AT2G36830" species:3702 "Arabidopsis thaliana" [GO:0006810 "transport" evidence=ISS] [GO:0009740 "gibberellic acid mediated signaling pathway" evidence=TAS] [GO:0015250 "water channel activity" evidence=ISS;IDA] [GO:0016020 "membrane" evidence=ISS] [GO:0009705 "plant-type vacuole membrane" evidence=IDA] [GO:0009941 "chloroplast envelope" evidence=IDA] [GO:0015204 "urea transmembrane transporter activity" evidence=IGI] [GO:0015840 "urea transport" evidence=IGI] [GO:0005773 "vacuole" evidence=IDA] [GO:0080170 "hydrogen peroxide transmembrane transport" evidence=IDA] [GO:0005774 "vacuolar membrane" evidence=IDA] [GO:0000326 "protein storage vacuole" evidence=IDA] [GO:0042807 "central vacuole" evidence=IDA] [GO:0006833 "water transport" evidence=IDA] InterPro:IPR000425 InterPro:IPR022357 Pfam:PF00230 PRINTS:PR00783 PROSITE:PS00221 GO:GO:0016021 EMBL:CP002685 GenomeReviews:CT485783_GR GO:GO:0009941 EMBL:AC006922 GO:GO:0009705 InterPro:IPR023271 Gene3D:G3DSA:1.20.1080.10 SUPFAM:SSF81338 eggNOG:COG0580 HOGENOM:HBG705794 PANTHER:PTHR19139 GO:GO:0015250 GO:GO:0000326 GO:GO:0015204 GO:GO:0080170 EMBL:X54854 EMBL:X63552 EMBL:M84344 EMBL:X72581 EMBL:AF370172 EMBL:AY059134 EMBL:AY087558 EMBL:Z18124 EMBL:Z18771 EMBL:Z26215 IPI:IPI00531328 PIR:S13718 PIR:S22202 RefSeq:NP_181221.1 UniGene:At.25221 UniGene:At.43252 UniGene:At.67051 ProteinModelPortal:P25818 SMR:P25818 IntAct:P25818 STRING:P25818

Tissue	Identification	# of reads (stress)	# of reads (control)	Fold Change	p-value	Description
						TCDB:1.A.8.10.3 PRIDE:P25818 EnsemblPlants:AT2G36830.1 GeneID:818255 KEGG:ath:AT2G36830 GeneFarm:4855 TAIR:At2g36830 InParanoid:P25818 KO:K09873 OMA:INTTHEQ PhylomeDB:P25818 ProtClustDB:PLN00027 ArrayExpress:P25818 Genevestigator:P25818 GermOnline:AT2G36830 GO:GO:0042807 Uniprot:P25818
Root	Isotig05334	17	119	-2.936	4.58E-22	TAIR|locus:2057906 - symbol:GAMMA-TIP "AT2G36830" species:3702 "Arabidopsis thaliana" [GO:0006810 "transport" evidence=ISS] [GO:0009740 "gibberellic acid mediated signaling pathway" evidence=TAS] [GO:0015250 "water channel activity" evidence=ISS;IDA] [GO:0016020 "membrane" evidence=ISS] [GO:0009705 "plant-type vacuole membrane" evidence=IDA] [GO:0009941 "chloroplast envelope" evidence=IDA] [GO:0015204 "urea transmembrane transporter activity" evidence=IGI] [GO:0015840 "urea transport" evidence=IGI] [GO:0005773 "vacuole" evidence=IDA] [GO:0080170 "hydrogen peroxide transmembrane transport" evidence=IDA] [GO:0005774 "vacuolar membrane" evidence=IDA] [GO:0000326 "protein storage vacuole" evidence=IDA] [GO:0042807 "central vacuole" evidence=IDA] [GO:0006833 "water transport" evidence=IDA] InterPro:IPR000425 InterPro:IPR022357 Pfam:PF00230 PRINTS:PR00783 PROSITE:PS00221 GO:GO:0016021 EMBL:CP002685 GenomeReviews:CT485783_GR GO:GO:0009941 EMBL:AC006922 GO:GO:0009705 InterPro:IPR023271 Gene3D:G3DSA:1.20.1080.10 SUPFAM:SSF81338 eggNOG:COG0580 HOGENOM:HBG705794 PANTHER:PTHR19139 GO:GO:0015250 GO:GO:0000326 GO:GO:0015204 GO:GO:0080170 EMBL:X54854 EMBL:X63552 EMBL:M84344 EMBL:X72581 EMBL:AF370172 EMBL:AY059134 EMBL:AY087558 EMBL:Z18124 EMBL:Z18771 EMBL:Z26215 IPI:IPI00531328 PIR:S13718 PIR:S22202 RefSeq:NP_181221.1 UniGene:At.25221 UniGene:At.43252 UniGene:At.67051 ProteinModelPortal:P25818 SMR:P25818 IntAct:P25818 STRING:P25818 TCDB:1.A.8.10.3 PRIDE:P25818 EnsemblPlants:AT2G36830.1 GeneID:818255 KEGG:ath:AT2G36830 GeneFarm:4855 TAIR:At2g36830 InParanoid:P25818 KO:K09873 OMA:INTTHEQ PhylomeDB:P25818 ProtClustDB:PLN00027 ArrayExpress:P25818 Genevestigator:P25818 GermOnline:AT2G36830 GO:GO:0042807 Uniprot:P25818
Root	Isotig05339	0	22	-5.588	6.01E-07	TAIR|locus:2195301 - symbol:NAS3 "AT1G09240" species:3702 "Arabidopsis thaliana" [GO:0030410 "nicotianamine synthase activity" evidence=IDA] [GO:0030418 "nicotianamine biosynthetic process" evidence=IDA] InterPro:IPR004298 Pfam:PF03059 PROSITE:PS51142 EMBL:CP002684 GenomeReviews:CT485782_GR EMBL:AC003114 HOGENOM:HBG594037 KO:K05953 ProtClustDB:PLN03075 GO:GO:0030410 GO:GO:0030418 EMBL:AB021936 EMBL:AY140031 EMBL:BT008817 EMBL:AY087620 IPI:IPI00518527 PIR:C86225 RefSeq:NP_172395.1 UniGene:At.42234 ProteinModelPortal:O80483 SMR:O80483 STRING:O80483 PRIDE:O80483 EnsemblPlants:AT1G09240.1 GeneID:837444 KEGG:ath:AT1G09240 TAIR:At1g09240 eggNOG:NOG139408 InParanoid:O80483 OMA:DISQRMF PhylomeDB:O80483 ArrayExpress:O80483 Genevestigator:O80483 GermOnline:AT1G09240 Uniprot:O80483
Root	Isotig05340	0	19	-5.377	3.67E-06	TAIR|locus:2195301 - symbol:NAS3 "AT1G09240" species:3702 "Arabidopsis thaliana" [GO:0030410 "nicotianamine synthase activity" evidence=IDA] [GO:0030418 "nicotianamine biosynthetic process" evidence=IDA] InterPro:IPR004298 Pfam:PF03059 PROSITE:PS51142 EMBL:CP002684 GenomeReviews:CT485782_GR EMBL:AC003114 HOGENOM:HBG594037 KO:K05953 ProtClustDB:PLN03075 GO:GO:0030410 GO:GO:0030418 EMBL:AB021936 EMBL:AY140031 EMBL:BT008817 EMBL:AY087620 IPI:IPI00518527 PIR:C86225 RefSeq:NP_172395.1 UniGene:At.42234 ProteinModelPortal:O80483 SMR:O80483 STRING:O80483 PRIDE:O80483 EnsemblPlants:AT1G09240.1 GeneID:837444 KEGG:ath:AT1G09240 TAIR:At1g09240 eggNOG:NOG139408 InParanoid:O80483 OMA:DISQRMF PhylomeDB:O80483 ArrayExpress:O80483 Genevestigator:O80483 GermOnline:AT1G09240 Uniprot:O80483
Root	Isotig05341	0	18	-5.299	6.77E-06	TAIR|locus:2195301 - symbol:NAS3 "AT1G09240" species:3702 "Arabidopsis thaliana" [GO:0030410 "nicotianamine synthase activity" evidence=IDA] [GO:0030418 "nicotianamine biosynthetic process" evidence=IDA] InterPro:IPR004298 Pfam:PF03059 PROSITE:PS51142 EMBL:CP002684 GenomeReviews:CT485782_GR EMBL:AC003114 HOGENOM:HBG594037 KO:K05953 ProtClustDB:PLN03075 GO:GO:0030410 GO:GO:0030418 EMBL:AB021936 EMBL:AY140031 EMBL:BT008817 EMBL:AY087620 IPI:IPI00518527 PIR:C86225 RefSeq:NP_172395.1 UniGene:At.42234 ProteinModelPortal:O80483 SMR:O80483 STRING:O80483 PRIDE:O80483 EnsemblPlants:AT1G09240.1 GeneID:837444 KEGG:ath:AT1G09240 TAIR:At1g09240 eggNOG:NOG139408 InParanoid:O80483 OMA:DISQRMF PhylomeDB:O80483 ArrayExpress:O80483 Genevestigator:O80483 GermOnline:AT1G09240 Uniprot:O80483
Root	Isotig05354	1	12	-3.714	0.000699736	TAIR|locus:2024122 - symbol:FLA9 "AT1G03870" species:3702 "Arabidopsis thaliana" [GO:0031225 "anchored to membrane" evidence=TAS] [GO:0046658 "anchored to plasma membrane" evidence=IDA] [GO:0005886 "plasma

Tissue	Identification	# of reads (stress)	# of reads (control)	Fold Change	p-value	Description
						membrane" evidence=IDA] Pfam:PF02469 InterPro:IPR000782 EMBL:CP002684 GenomeReviews:CT485782_GR EMBL:AC003027 GO:GO:0046658 Gene3D:G3DSA:2.30.180.10 SMART:SM00554 SUPFAM:SSF82153 PROSITE:PS50213 HOGENOM:HBG750250 ProtClustDB:CLSN2687227 EMBL:AF333974 EMBL:AF083781 EMBL:AY087938 EMBL:AK227010 IPI:IPI00540701 PIR:D86169 RefSeq:NP_563692.1 UniGene:At.23966 UniGene:At.73148 ProteinModelPortal:Q9ZWA8 SMR:Q9ZWA8 PRIDE:Q9ZWA8 EnsemblPlants:AT1G03870.1 GeneID:839384 KEGG:ath:AT1G03870 GeneFarm:3781 TAIR:At1g03870 eggNOG:NOG299573 InParanoid:Q9ZWA8 OMA:VNSSSEG PhylomeDB:Q9ZWA8 Genevestigator:Q9ZWA8 GermOnline:AT1G03870 Uniprot:Q9ZWA8
Root	Isotig05355	1	12	-3.714	0.000699736	TAIR|locus:2024122 - symbol:FLA9 "AT1G03870" species:3702 "Arabidopsis thaliana" [GO:0031225 "anchored to membrane" evidence=TAS] [GO:0046658 "anchored to plasma membrane" evidence=IDA] [GO:0005886 "plasma membrane" evidence=IDA] Pfam:PF02469 InterPro:IPR000782 EMBL:CP002684 GenomeReviews:CT485782_GR EMBL:AC003027 GO:GO:0046658 Gene3D:G3DSA:2.30.180.10 SMART:SM00554 SUPFAM:SSF82153 PROSITE:PS50213 HOGENOM:HBG750250 ProtClustDB:CLSN2687227 EMBL:AF333974 EMBL:AF083781 EMBL:AY087938 EMBL:AK227010 IPI:IPI00540701 PIR:D86169 RefSeq:NP_563692.1 UniGene:At.23966 UniGene:At.73148 ProteinModelPortal:Q9ZWA8 SMR:Q9ZWA8 PRIDE:Q9ZWA8 EnsemblPlants:AT1G03870.1 GeneID:839384 KEGG:ath:AT1G03870 GeneFarm:3781 TAIR:At1g03870 eggNOG:NOG299573 InParanoid:Q9ZWA8 OMA:VNSSSEG PhylomeDB:Q9ZWA8 Genevestigator:Q9ZWA8 GermOnline:AT1G03870 Uniprot:Q9ZWA8
Root	Isotig05366	6	25	-2.188	0.000173532	TAIR|locus:2038593 - symbol:FD3 "ferredoxin 3" species:3702 "Arabidopsis thaliana" [GO:0009055 "electron carrier activity" evidence=IEA;ISS] [GO:0022900 "electron transport chain" evidence=IEA] [GO:0051536 "iron-sulfur cluster binding" evidence=IEA] [GO:0051537 "2 iron, 2 sulfur cluster binding" evidence=IEA] InterPro:IPR001041 InterPro:IPR006058 InterPro:IPR010241 Pfam:PF00111 PROSITE:PS00197 PROSITE:PS51085 GO:GO:0009507 EMBL:CP002685 GenomeReviews:CT485783_GR GO:GO:0009055 GO:GO:0046872 GO:GO:0006810 GO:GO:0022900 GO:GO:0051537 InterPro:IPR012675 Gene3D:G3DSA:3.10.20.30 SUPFAM:SSF54292 eggNOG:COG0633 EMBL:AC006232 TIGRFAMs:TIGR02008 HOGENOM:HBG748855 KO:K02639 EMBL:AY086622 EMBL:BT004187 EMBL:BT005393 IPI:IPI00548374 PIR:G84673 RefSeq:NP_180320.1 UniGene:At.38754 HSSP:P00248 ProteinModelPortal:Q9ZQG8 SMR:Q9ZQG8 STRING:Q9ZQG8 PRIDE:Q9ZQG8 EnsemblPlants:AT2G27510.1 GeneID:817297 KEGG:ath:AT2G27510 TAIR:At2g27510 InParanoid:Q9ZQG8 OMA:YPQSDCV PhylomeDB:Q9ZQG8 ProtClustDB:CLSN2913085 ArrayExpress:Q9ZQG8 Genevestigator:Q9ZQG8 Uniprot:Q9ZQG8
Root	Isotig05367	6	25	-2.188	0.000173532	TAIR|locus:2038593 - symbol:FD3 "ferredoxin 3" species:3702 "Arabidopsis thaliana" [GO:0009055 "electron carrier activity" evidence=IEA;ISS] [GO:0022900 "electron transport chain" evidence=IEA] [GO:0051536 "iron-sulfur cluster binding" evidence=IEA] [GO:0051537 "2 iron, 2 sulfur cluster binding" evidence=IEA] InterPro:IPR001041 InterPro:IPR006058 InterPro:IPR010241 Pfam:PF00111 PROSITE:PS00197 PROSITE:PS51085 GO:GO:0009507 EMBL:CP002685 GenomeReviews:CT485783_GR GO:GO:0009055 GO:GO:0046872 GO:GO:0006810 GO:GO:0022900 GO:GO:0051537 InterPro:IPR012675 Gene3D:G3DSA:3.10.20.30 SUPFAM:SSF54292 eggNOG:COG0633 EMBL:AC006232 TIGRFAMs:TIGR02008 HOGENOM:HBG748855 KO:K02639 EMBL:AY086622 EMBL:BT004187 EMBL:BT005393 IPI:IPI00548374 PIR:G84673 RefSeq:NP_180320.1 UniGene:At.38754 HSSP:P00248 ProteinModelPortal:Q9ZQG8 SMR:Q9ZQG8 STRING:Q9ZQG8 PRIDE:Q9ZQG8 EnsemblPlants:AT2G27510.1 GeneID:817297 KEGG:ath:AT2G27510 TAIR:At2g27510 InParanoid:Q9ZQG8 OMA:YPQSDCV PhylomeDB:Q9ZQG8 ProtClustDB:CLSN2913085 ArrayExpress:Q9ZQG8 Genevestigator:Q9ZQG8 Uniprot:Q9ZQG8
Root	Isotig05411	1	23	-4.653	5.84E-07	TAIR|locus:505006681 - symbol:DIR1 "AT5G48485" species:3702 "Arabidopsis thaliana" [GO:0006869 "lipid transport" evidence=ISS] [GO:0008289 "lipid binding" evidence=ISS] [GO:0005319 "lipid transporter activity" evidence=ISS] [GO:0009862 "systemic acquired resistance, salicylic acid mediated signaling pathway" evidence=IMP] [GO:0005504 "fatty acid binding" evidence=IDA] [GO:0008270 "zinc ion binding" evidence=IDA] InterPro:IPR013770 GO:GO:0048046 EMBL:CP002688 GenomeReviews:BA000015_GR InterPro:IPR016140 SMART:SM00499 SUPFAM:SSF47699 GO:GO:0008270 GO:GO:0006869 GO:GO:0005504 Gene3D:G3DSA:1.10.110.10 EMBL:AB020745 GO:GO:0009862 EMBL:AF342726 EMBL:AY062857 EMBL:BT006510 EMBL:AY085224 IPI:IPI00525511 RefSeq:NP_568699.1 UniGene:At.27209 PDB:2RKN PDBsum:2RKN ProteinModelPortal:Q8W453 SMR:Q8W453 PRIDE:Q8W453 EnsemblPlants:AT5G48485.1 GeneID:834904 KEGG:ath:AT5G48485 TAIR:At5g48485 eggNOG:NOG297830 InParanoid:Q8W453

Tissue	Identification	# of reads (stress)	# of reads (control)	Fold Change	p-value	Description
						OMA:NECKPAV PhylomeDB:Q8W453 ProtClustDB:CLSN2686599 Genevestigator:Q8W453 Pfam:PF14368 Uniprot:Q8W453
Root	Isotig05412	1	23	-4.653	5.84E-07	TAIR|locus:505006681 - symbol:DIR1 "AT5G48485" species:3702 "Arabidopsis thaliana" [GO:0006869 "lipid transport" evidence=ISS] [GO:0008289 "lipid binding" evidence=ISS] [GO:0005319 "lipid transporter activity" evidence=ISS] [GO:0009862 "systemic acquired resistance, salicylic acid mediated signaling pathway" evidence=IMP] [GO:0005504 "fatty acid binding" evidence=IDA] [GO:0008270 "zinc ion binding" evidence=IDA] InterPro:IPR013770 GO:GO:0048046 EMBL:CP002688 GenomeReviews:BA000015_GR InterPro:IPR016140 SMART:SM00499 SUPFAM:SSF47699 GO:GO:0008270 GO:GO:0006869 GO:GO:0005504 Gene3D:G3DSA:1.10.110.10 EMBL:AB020745 GO:GO:0009862 EMBL:AF342726 EMBL:AY062857 EMBL:BT006510 EMBL:AY085224 IPI:IPI00525511 RefSeq:NP_568699.1 UniGene:At.27209 PDB:2RKN PDBsum:2RKN ProteinModelPortal:Q8W453 SMR:Q8W453 PRIDE:Q8W453 EnsemblPlants:AT5G48485.1 GeneID:834904 KEGG:ath:AT5G48485 TAIR:At5g48485 eggNOG:NOG297830 InParanoid:Q8W453 OMA:NECKPAV PhylomeDB:Q8W453 ProtClustDB:CLSN2686599 Genevestigator:Q8W453 Pfam:PF14368 Uniprot:Q8W453
Root	Isotig05435	5	21	-2.199	0.000555521	TAIR|locus:2181823 - symbol:AT5G02570 species:3702 "Arabidopsis thaliana" [GO:0003677 "DNA binding" evidence=IEA;ISS] [GO:0006334 "nucleosome assembly" evidence=IEA] [GO:0005730 "nucleolus" evidence=IDA] InterPro:IPR000558 InterPro:IPR007125 InterPro:IPR009072 Pfam:PF00125 PRINTS:PR00621 PROSITE:PS00357 SMART:SM00427 EMBL:CP002688 GenomeReviews:BA000015_GR GO:GO:0005730 GO:GO:0003677 Gene3D:G3DSA:1.10.20.10 SUPFAM:SSF47113 GO:GO:0006334 GO:GO:0000786 HOGENOM:HBG715487 EMBL:AL162971 eggNOG:NOG289161 KO:K11252 PANTHER:PTHR23428 OMA:RMPEPAK IPI:IPI00527484 PIR:T48278 RefSeq:NP_195877.1 UniGene:At.65481 ProteinModelPortal:Q9LZ45 SMR:Q9LZ45 PRIDE:Q9LZ45 EnsemblPlants:AT5G02570.1 GeneID:831878 KEGG:ath:AT5G02570 TAIR:At5g02570 InParanoid:Q9LZ45 PhylomeDB:Q9LZ45 ProtClustDB:CLSN2916781 Genevestigator:Q9LZ45 GermOnline:AT5G02570 Uniprot:Q9LZ45
Root	Isotig05436	5	21	-2.199	0.000555521	TAIR|locus:2181823 - symbol:AT5G02570 species:3702 "Arabidopsis thaliana" [GO:0003677 "DNA binding" evidence=IEA;ISS] [GO:0006334 "nucleosome assembly" evidence=IEA] [GO:0005730 "nucleolus" evidence=IDA] InterPro:IPR000558 InterPro:IPR007125 InterPro:IPR009072 Pfam:PF00125 PRINTS:PR00621 PROSITE:PS00357 SMART:SM00427 EMBL:CP002688 GenomeReviews:BA000015_GR GO:GO:0005730 GO:GO:0003677 Gene3D:G3DSA:1.10.20.10 SUPFAM:SSF47113 GO:GO:0006334 GO:GO:0000786 HOGENOM:HBG715487 EMBL:AL162971 eggNOG:NOG289161 KO:K11252 PANTHER:PTHR23428 OMA:RMPEPAK IPI:IPI00527484 PIR:T48278 RefSeq:NP_195877.1 UniGene:At.65481 ProteinModelPortal:Q9LZ45 SMR:Q9LZ45 PRIDE:Q9LZ45 EnsemblPlants:AT5G02570.1 GeneID:831878 KEGG:ath:AT5G02570 TAIR:At5g02570 InParanoid:Q9LZ45 PhylomeDB:Q9LZ45 ProtClustDB:CLSN2916781 Genevestigator:Q9LZ45 GermOnline:AT5G02570 Uniprot:Q9LZ45
Root	Isotig05437	5	21	-2.199	0.000555521	TAIR|locus:2181823 - symbol:AT5G02570 species:3702 "Arabidopsis thaliana" [GO:0003677 "DNA binding" evidence=IEA;ISS] [GO:0006334 "nucleosome assembly" evidence=IEA] [GO:0005730 "nucleolus" evidence=IDA] InterPro:IPR000558 InterPro:IPR007125 InterPro:IPR009072 Pfam:PF00125 PRINTS:PR00621 PROSITE:PS00357 SMART:SM00427 EMBL:CP002688 GenomeReviews:BA000015_GR GO:GO:0005730 GO:GO:0003677 Gene3D:G3DSA:1.10.20.10 SUPFAM:SSF47113 GO:GO:0006334 GO:GO:0000786 HOGENOM:HBG715487 EMBL:AL162971 eggNOG:NOG289161 KO:K11252 PANTHER:PTHR23428 OMA:RMPEPAK IPI:IPI00527484 PIR:T48278 RefSeq:NP_195877.1 UniGene:At.65481 ProteinModelPortal:Q9LZ45 SMR:Q9LZ45 PRIDE:Q9LZ45 EnsemblPlants:AT5G02570.1 GeneID:831878 KEGG:ath:AT5G02570 TAIR:At5g02570 InParanoid:Q9LZ45 PhylomeDB:Q9LZ45 ProtClustDB:CLSN2916781 Genevestigator:Q9LZ45 GermOnline:AT5G02570 Uniprot:Q9LZ45
Root	Isotig05506	27	6	2.041	0.000356109	TAIR|locus:2014736 - symbol:AT1G35660 "AT1G35660" species:3702 "Arabidopsis thaliana" [GO:0003674 "molecular_function" evidence=ND] [GO:0008150 "biological_process" evidence=ND] [GO:0005886 "plasma membrane" evidence=IDA] GenomeReviews:CT485782_GR GO:GO:0005886 EMBL:AC007887 eggNOG:NOG79617 IPI:IPI00544757 PIR:D86478 ProteinModelPortal:Q9LQH4 PRIDE:Q9LQH4 TAIR:At1g35660 HOGENOM:HBG591637 InParanoid:Q9LQH4 PhylomeDB:Q9LQH4 Genevestigator:Q9LQH4 Uniprot:Q9LQH4
Root	Isotig05507	26	6	1.987	0.000575837	TAIR|locus:2014736 - symbol:AT1G35660 "AT1G35660" species:3702 "Arabidopsis thaliana" [GO:0003674 "molecular_function" evidence=ND] [GO:0008150 "biological_process" evidence=ND] [GO:0005886 "plasma membrane" evidence=IDA] GenomeReviews:CT485782_GR GO:GO:0005886 EMBL:AC007887 eggNOG:NOG79617 IPI:IPI00544757 PIR:D86478 ProteinModelPortal:Q9LQH4 PRIDE:Q9LQH4 TAIR:At1g35660 HOGENOM:HBG591637 InParanoid:Q9LQH4 PhylomeDB:Q9LQH4 Genevestigator:Q9LQH4 Uniprot:Q9LQH4

Tissue	Identification	# of reads (stress)	# of reads (control)	Fold Change	p-value	Description
Root	Isotig05530	0	23	-5.653	3.32E-07	TAIR|locus:2195850 - symbol:AT1G51860 species:3702 "Arabidopsis thaliana" [GO:0006468 "protein phosphorylation" evidence=IEA] [GO:0016301 "kinase activity" evidence=ISS] Pfam:PF00560 InterPro:IPR001611 InterPro:IPR000719 InterPro:IPR001245 InterPro:IPR008271 InterPro:IPR011009 InterPro:IPR017441 Pfam:PF07714 PROSITE:PS00107 PROSITE:PS00108 PROSITE:PS50011 GO:GO:0016021 EMBL:CP002684 GenomeReviews:CT485782_GR GO:GO:0005524 eggNOG:COG0515 GO:GO:0004872 SUPFAM:SSF56112 GO:GO:0004674 HOGENOM:HBG755340 InterPro:IPR024788 Pfam:PF12819 EMBL:AC015448 EMBL:FJ708653 IPI:IPI00532293 PIR:A96558 RefSeq:NP_175598.1 UniGene:At.52143 ProteinModelPortal:C0LGG4 SMR:C0LGG4 PRIDE:C0LGG4 EnsemblPlants:AT1G51860.1 GeneID:841613 KEGG:ath:AT1G51860 GeneFarm:343 TAIR:At1g51860 InParanoid:Q9FZB3 OMA:ERPHIND PhylomeDB:C0LGG4 ProtClustDB:CLSN2679439 Uniprot:C0LGG4
Root	Isotig05531	0	16	-5.129	2.34E-05	TAIR|locus:2089483 - symbol:AT3G21340 species:3702 "Arabidopsis thaliana" [GO:0006468 "protein phosphorylation" evidence=IEA] [GO:0016301 "kinase activity" evidence=ISS] InterPro:IPR000719 InterPro:IPR008271 InterPro:IPR011009 InterPro:IPR017441 Pfam:PF00069 PROSITE:PS00107 PROSITE:PS00108 PROSITE:PS50011 GO:GO:0016021 GO:GO:0005886 GO:GO:0005524 EMBL:CP002686 GenomeReviews:BA000014_GR eggNOG:COG0515 GO:GO:0004714 SUPFAM:SSF56112 GO:GO:0004674 HOGENOM:HBG755340 EMBL:AP001305 InterPro:IPR024788 Pfam:PF12819 EMBL:FJ708725 IPI:IPI00518645 RefSeq:NP_188771.2 UniGene:At.38075 ProteinModelPortal:Q9LIG2 SMR:Q9LIG2 IntAct:Q9LIG2 PRIDE:Q9LIG2 EnsemblPlants:AT3G21340.1 GeneID:821688 KEGG:ath:AT3G21340 GeneFarm:418 TAIR:At3g21340 InParanoid:Q9LIG2 PhylomeDB:Q9LIG2 Genevestigator:Q9LIG2 Uniprot:Q9LIG2
Root	Isotig05533	5	22	-2.266	0.000318925	TAIR|locus:2064712 - symbol:RIPK "AT2G05940" species:3702 "Arabidopsis thaliana" [GO:0005575 "cellular_component" evidence=ND] [GO:0016301 "kinase activity" evidence=ISS] [GO:0004674 "protein serine/threonine kinase activity" evidence=IDA] [GO:0042742 "defense response to bacterium" evidence=IMP] InterPro:IPR000719 InterPro:IPR001245 InterPro:IPR008271 InterPro:IPR011009 Pfam:PF07714 PROSITE:PS00108 PROSITE:PS50011 GO:GO:0005524 GO:GO:0042742 EMBL:CP002685 GenomeReviews:CT485783_GR eggNOG:COG0515 SUPFAM:SSF56112 GO:GO:0004674 HOGENOM:HBG755340 EMBL:AC005970 EMBL:AY056245 EMBL:AY113963 IPI:IPI00547996 PIR:C84473 RefSeq:NP_178651.1 UniGene:At.23011 ProteinModelPortal:Q9ZUF4 SMR:Q9ZUF4 STRING:Q9ZUF4 PRIDE:Q9ZUF4 EnsemblPlants:AT2G05940.1 GeneID:815147 KEGG:ath:AT2G05940 TAIR:At2g05940 InParanoid:Q9ZUF4 OMA:MLNDPRK PhylomeDB:Q9ZUF4 ProtClustDB:CLSN2912989 ArrayExpress:Q9ZUF4 Genevestigator:Q9ZUF4 Uniprot:Q9ZUF4
Root	Isotig05534	5	22	-2.266	0.000318925	TAIR|locus:2064712 - symbol:RIPK "AT2G05940" species:3702 "Arabidopsis thaliana" [GO:0005575 "cellular_component" evidence=ND] [GO:0016301 "kinase activity" evidence=ISS] [GO:0004674 "protein serine/threonine kinase activity" evidence=IDA] [GO:0042742 "defense response to bacterium" evidence=IMP] InterPro:IPR000719 InterPro:IPR001245 InterPro:IPR008271 InterPro:IPR011009 Pfam:PF07714 PROSITE:PS00108 PROSITE:PS50011 GO:GO:0005524 GO:GO:0042742 EMBL:CP002685 GenomeReviews:CT485783_GR eggNOG:COG0515 SUPFAM:SSF56112 GO:GO:0004674 HOGENOM:HBG755340 EMBL:AC005970 EMBL:AY056245 EMBL:AY113963 IPI:IPI00547996 PIR:C84473 RefSeq:NP_178651.1 UniGene:At.23011 ProteinModelPortal:Q9ZUF4 SMR:Q9ZUF4 STRING:Q9ZUF4 PRIDE:Q9ZUF4 EnsemblPlants:AT2G05940.1 GeneID:815147 KEGG:ath:AT2G05940 TAIR:At2g05940 InParanoid:Q9ZUF4 OMA:MLNDPRK PhylomeDB:Q9ZUF4 ProtClustDB:CLSN2912989 ArrayExpress:Q9ZUF4 Genevestigator:Q9ZUF4 Uniprot:Q9ZUF4
Root	Isotig05535	5	22	-2.266	0.000318925	TAIR|locus:2064712 - symbol:RIPK "AT2G05940" species:3702 "Arabidopsis thaliana" [GO:0005575 "cellular_component" evidence=ND] [GO:0016301 "kinase activity" evidence=ISS] [GO:0004674 "protein serine/threonine kinase activity" evidence=IDA] [GO:0042742 "defense response to bacterium" evidence=IMP] InterPro:IPR000719 InterPro:IPR001245 InterPro:IPR008271 InterPro:IPR011009 Pfam:PF07714 PROSITE:PS00108 PROSITE:PS50011 GO:GO:0005524 GO:GO:0042742 EMBL:CP002685 GenomeReviews:CT485783_GR eggNOG:COG0515 SUPFAM:SSF56112 GO:GO:0004674 HOGENOM:HBG755340 EMBL:AC005970 EMBL:AY056245 EMBL:AY113963 IPI:IPI00547996 PIR:C84473 RefSeq:NP_178651.1 UniGene:At.23011 ProteinModelPortal:Q9ZUF4 SMR:Q9ZUF4 STRING:Q9ZUF4 PRIDE:Q9ZUF4 EnsemblPlants:AT2G05940.1 GeneID:815147 KEGG:ath:AT2G05940 TAIR:At2g05940 InParanoid:Q9ZUF4 OMA:MLNDPRK PhylomeDB:Q9ZUF4 ProtClustDB:CLSN2912989 ArrayExpress:Q9ZUF4 Genevestigator:Q9ZUF4 Uniprot:Q9ZUF4

Tissue	Identification	# of reads (stress)	# of reads (control)	Fold Change	p-value	Description
Root	Isotig05536	2	78	-5.414	6.38E-21	TAIR|locus:2168988 - symbol:TDT "AT5G47560" species:3702 "Arabidopsis thaliana" [GO:0006814 "sodium ion transport" evidence=ISS] [GO:0006835 "dicarboxylic acid transport" evidence=ISS] [GO:0017153 "sodium:dicarboxylate symporter activity" evidence=ISS] [GO:0005773 "vacuole" evidence=IDA] [GO:0015140 "malate transmembrane transporter activity" evidence=IMP;IDA] [GO:0015743 "malate transport" evidence=TAS] [GO:0051453 "regulation of intracellular pH" evidence=TAS] InterPro:IPR001898 Pfam:PF00939 GO:GO:0016021 GO:GO:0005774 EMBL:CP002688 GenomeReviews:BA000015_GR EMBL:AB025628 GO:GO:0006814 GO:GO:0015140 GO:GO:0051453 eggNOG:COG0471 TIGRFAMs:TIGR00785 KO:K14445 HOGENOM:HBG616159 EMBL:AJ223445 EMBL:AB043024 EMBL:AY072145 EMBL:AY133792 EMBL:AY084408 IPI:IPI00534224 RefSeq:NP_199567.1 UniGene:At.21038 UniGene:At.68515 UniGene:At.71618 STRING:Q8LG88 TCDB:2.A.47.1.6 PRIDE:Q8LG88 EnsemblPlants:AT5G47560.1 GeneID:834806 KEGG:ath:AT5G47560 GeneFarm:4980 TAIR:At5g47560 InParanoid:Q8LG88 OMA:TECMPLA PhylomeDB:Q8LG88 ProtClustDB:CLSN2686388 ArrayExpress:Q9MAW4 Genevestigator:Q8LG88 GermOnline:AT5G47560 Uniprot:Q8LG88
Root	Isotig05537	2	77	-5.396	1.15E-20	TAIR|locus:2168988 - symbol:TDT "AT5G47560" species:3702 "Arabidopsis thaliana" [GO:0006814 "sodium ion transport" evidence=ISS] [GO:0006835 "dicarboxylic acid transport" evidence=ISS] [GO:0017153 "sodium:dicarboxylate symporter activity" evidence=ISS] [GO:0005773 "vacuole" evidence=IDA] [GO:0015140 "malate transmembrane transporter activity" evidence=IMP;IDA] [GO:0015743 "malate transport" evidence=TAS] [GO:0051453 "regulation of intracellular pH" evidence=TAS] InterPro:IPR001898 Pfam:PF00939 GO:GO:0016021 GO:GO:0005774 EMBL:CP002688 GenomeReviews:BA000015_GR EMBL:AB025628 GO:GO:0006814 GO:GO:0015140 GO:GO:0051453 eggNOG:COG0471 TIGRFAMs:TIGR00785 KO:K14445 HOGENOM:HBG616159 EMBL:AJ223445 EMBL:AB043024 EMBL:AY072145 EMBL:AY133792 EMBL:AY084408 IPI:IPI00534224 RefSeq:NP_199567.1 UniGene:At.21038 UniGene:At.68515 UniGene:At.71618 STRING:Q8LG88 TCDB:2.A.47.1.6 PRIDE:Q8LG88 EnsemblPlants:AT5G47560.1 GeneID:834806 KEGG:ath:AT5G47560 GeneFarm:4980 TAIR:At5g47560 InParanoid:Q8LG88 OMA:TECMPLA PhylomeDB:Q8LG88 ProtClustDB:CLSN2686388 ArrayExpress:Q9MAW4 Genevestigator:Q8LG88 GermOnline:AT5G47560 Uniprot:Q8LG88
Root	Isotig05538	2	64	-5.129	2.68E-17	TAIR|locus:2168988 - symbol:TDT "AT5G47560" species:3702 "Arabidopsis thaliana" [GO:0006814 "sodium ion transport" evidence=ISS] [GO:0006835 "dicarboxylic acid transport" evidence=ISS] [GO:0017153 "sodium:dicarboxylate symporter activity" evidence=ISS] [GO:0005773 "vacuole" evidence=IDA] [GO:0015140 "malate transmembrane transporter activity" evidence=IMP;IDA] [GO:0015743 "malate transport" evidence=TAS] [GO:0051453 "regulation of intracellular pH" evidence=TAS] InterPro:IPR001898 Pfam:PF00939 GO:GO:0016021 GO:GO:0005774 EMBL:CP002688 GenomeReviews:BA000015_GR EMBL:AB025628 GO:GO:0006814 GO:GO:0015140 GO:GO:0051453 eggNOG:COG0471 TIGRFAMs:TIGR00785 KO:K14445 HOGENOM:HBG616159 EMBL:AJ223445 EMBL:AB043024 EMBL:AY072145 EMBL:AY133792 EMBL:AY084408 IPI:IPI00534224 RefSeq:NP_199567.1 UniGene:At.21038 UniGene:At.68515 UniGene:At.71618 STRING:Q8LG88 TCDB:2.A.47.1.6 PRIDE:Q8LG88 EnsemblPlants:AT5G47560.1 GeneID:834806 KEGG:ath:AT5G47560 GeneFarm:4980 TAIR:At5g47560 InParanoid:Q8LG88 OMA:TECMPLA PhylomeDB:Q8LG88 ProtClustDB:CLSN2686388 ArrayExpress:Q9MAW4 Genevestigator:Q8LG88 GermOnline:AT5G47560 Uniprot:Q8LG88
Root	Isotig05542	54	7	2.819	1.10E-09	TAIR|locus:2005543 - symbol:TSB2 "AT4G27070" species:3702 "Arabidopsis thaliana" [GO:0000162 "tryptophan biosynthetic process" evidence=TAS] [GO:0004834 "tryptophan synthase activity" evidence=ISS;IMP] [GO:0009507 "chloroplast" evidence=IDA] [GO:0009570 "chloroplast stroma" evidence=IDA] [GO:0009941 "chloroplast envelope" evidence=IDA] InterPro:IPR001926 InterPro:IPR006653 InterPro:IPR006654 InterPro:IPR023026 Pfam:PF00291 PIRSF:PIRSF001413 PROSITE:PS00168 EMBL:CP002687 GenomeReviews:CT486007_GR GO:GO:0009570 GO:GO:0030170 SUPFAM:SSF53686 EMBL:AL035680 EMBL:AL161566 GO:GO:0009941 EMBL:M81620 EMBL:BT003144 EMBL:AY084334 IPI:IPI00547656 PIR:JQ1073 PIR:T06037 RefSeq:NP_194437.1 UniGene:At.327 ProteinModelPortal:P25269 SMR:P25269 STRING:P25269 PRIDE:P25269 EnsemblPlants:AT4G27070.1 GeneID:828815 KEGG:ath:AT4G27070 TAIR:At4g27070 eggNOG:COG0133 HOGENOM:HBG303148 InParanoid:P25269 KO:K01696 OMA:IMVINLS PhylomeDB:P25269 ProtClustDB:PLN02618 ArrayExpress:P25269 Genevestigator:P25269 GermOnline:AT4G27070 GO:GO:0004834 PANTHER:PTHR10314:SF3 TIGRFAMs:TIGR00263 Uniprot:P25269

Tissue	Identification	# of reads (stress)	# of reads (control)	Fold Change	p-value	Description
Root	Isotig05543	49	8	2.486	5.83E-08	TAIR|locus:2005543 - symbol:TSB2 "AT4G27070" species:3702 "Arabidopsis thaliana" [GO:0000162 "tryptophan biosynthetic process" evidence=TAS] [GO:0004834 "tryptophan synthase activity" evidence=ISS;IMP] [GO:0009507 "chloroplast" evidence=IDA] [GO:0009570 "chloroplast stroma" evidence=IDA] [GO:0009941 "chloroplast envelope" evidence=IDA] InterPro:IPR001926 InterPro:IPR006653 InterPro:IPR006654 InterPro:IPR023026 Pfam:PF00291 PIRSF:PIRSF001413 PROSITE:PS00168 EMBL:CP002687 GenomeReviews:CT486007_GR GO:GO:0009570 GO:GO:0030170 SUPFAM:SSF53686 EMBL:AL035680 EMBL:AL161566 GO:GO:0009941 EMBL:M81620 EMBL:BT003144 EMBL:AY084334 IPI:IPI00547656 PIR:JQ1073 PIR:T06037 RefSeq:NP_194437.1 UniGene:At.327 ProteinModelPortal:P25269 SMR:P25269 STRING:P25269 PRIDE:P25269 EnsemblPlants:AT4G27070.1 GeneID:828815 KEGG:ath:AT4G27070 TAIR:At4g27070 eggNOG:COG0133 HOGENOM:HBG303148 InParanoid:P25269 KO:K01696 OMA:IMVINLS PhylomeDB:P25269 ProtClustDB:PLN02618 ArrayExpress:P25269 Genevestigator:P25269 GermOnline:AT4G27070 GO:GO:0004834 PANTHER:PTHR10314:SF3 TIGRFAMs:TIGR00263 Uniprot:P25269
Root	Isotig05544	41	7	2.421	1.02E-06	TAIR|locus:2005543 - symbol:TSB2 "AT4G27070" species:3702 "Arabidopsis thaliana" [GO:0000162 "tryptophan biosynthetic process" evidence=TAS] [GO:0004834 "tryptophan synthase activity" evidence=ISS;IMP] [GO:0009507 "chloroplast" evidence=IDA] [GO:0009570 "chloroplast stroma" evidence=IDA] [GO:0009941 "chloroplast envelope" evidence=IDA] InterPro:IPR001926 InterPro:IPR006653 InterPro:IPR006654 InterPro:IPR023026 Pfam:PF00291 PIRSF:PIRSF001413 PROSITE:PS00168 EMBL:CP002687 GenomeReviews:CT486007_GR GO:GO:0009570 GO:GO:0030170 SUPFAM:SSF53686 EMBL:AL035680 EMBL:AL161566 GO:GO:0009941 EMBL:M81620 EMBL:BT003144 EMBL:AY084334 IPI:IPI00547656 PIR:JQ1073 PIR:T06037 RefSeq:NP_194437.1 UniGene:At.327 ProteinModelPortal:P25269 SMR:P25269 STRING:P25269 PRIDE:P25269 EnsemblPlants:AT4G27070.1 GeneID:828815 KEGG:ath:AT4G27070 TAIR:At4g27070 eggNOG:COG0133 HOGENOM:HBG303148 InParanoid:P25269 KO:K01696 OMA:IMVINLS PhylomeDB:P25269 ProtClustDB:PLN02618 ArrayExpress:P25269 Genevestigator:P25269 GermOnline:AT4G27070 GO:GO:0004834 PANTHER:PTHR10314:SF3 TIGRFAMs:TIGR00263 Uniprot:P25269
Root	Isotig05560	32	1	4.871	1.34E-08	TAIR|locus:2030230 - symbol:HAB1 "AT1G72770" species:3702 "Arabidopsis thaliana" [GO:0004722 "protein serine/threonine phosphatase activity" evidence=ISS;IDA] [GO:0006470 "protein dephosphorylation" evidence=IEA] [GO:0008287 "protein serine/threonine phosphatase complex" evidence=IEA] [GO:0005515 "protein binding" evidence=IPI] InterPro:IPR000222 InterPro:IPR001932 Pfam:PF00481 PROSITE:PS01032 SMART:SM00331 SMART:SM00332 EMBL:CP002684 GenomeReviews:CT485782_GR GO:GO:0005634 GO:GO:0005737 GO:GO:0005515 GO:GO:0006470 GO:GO:0009738 GO:GO:0004722 GO:GO:0046872 EMBL:AC010926 eggNOG:COG0631 Gene3D:G3DSA:3.60.40.10 SUPFAM:SSF81606 GO:GO:0008287 InterPro:IPR015655 PANTHER:PTHR13832 HOGENOM:HBG747569 ProtClustDB:CLSN2679602 EMBL:AJ003119 EMBL:BT015409 EMBL:AK230171 EMBL:AK318665 IPI:IPI00538917 IPI:IPI00846864 PIR:F96752 RefSeq:NP_001077815.1 RefSeq:NP_001185385.1 RefSeq:NP_177421.1 UniGene:At.46635 UniGene:At.67356 PDB:3KB3 PDB:3NMT PDB:3QN1 PDB:3RT0 PDBsum:3KB3 PDBsum:3NMT PDBsum:3QN1 PDBsum:3RT0 ProteinModelPortal:Q9CAJ0 SMR:Q9CAJ0 DIP:DIP-48988N IntAct:Q9CAJ0 STRING:Q9CAJ0 PRIDE:Q9CAJ0 EnsemblPlants:AT1G72770.1 EnsemblPlants:AT1G72770.3 GeneID:843609 KEGG:ath:AT1G72770 TAIR:At1g72770 InParanoid:Q9CAJ0 OMA:YARIENA PhylomeDB:Q9CAJ0 Genevestigator:Q9CAJ0 Uniprot:Q9CAJ0
Root	Isotig05561	23	0	5.395	1.11E-06	TAIR|locus:2007943 - symbol:HAB2 "homology to ABI2" species:3702 "Arabidopsis thaliana" [GO:0003824 "catalytic activity" evidence=IEA] [GO:0004722 "protein serine/threonine phosphatase activity" evidence=IEA;ISS] [GO:0006470 "protein dephosphorylation" evidence=IEA] InterPro:IPR000222 InterPro:IPR001932 Pfam:PF00481 PROSITE:PS01032 SMART:SM00331 SMART:SM00332 EMBL:CP002684 GenomeReviews:CT485782_GR GO:GO:0006470 GO:GO:0009738 GO:GO:0004722 GO:GO:0046872 EMBL:AC022492 eggNOG:COG0631 Gene3D:G3DSA:3.60.40.10 SUPFAM:SSF81606 GO:GO:0008287 InterPro:IPR015655 PANTHER:PTHR13832 HSSP:P35813 HOGENOM:HBG747569 KO:K14497 EMBL:AK118656 IPI:IPI00532752 RefSeq:NP_173199.2 UniGene:At.41827 ProteinModelPortal:Q9LNP9 SMR:Q9LNP9 DIP:DIP-48989N PRIDE:Q9LNP9 EnsemblPlants:AT1G17550.1 GeneID:838330 KEGG:ath:AT1G17550 TAIR:At1g17550 InParanoid:Q8GWS8 OMA:RRILAWH PhylomeDB:Q9LNP9 ProtClustDB:CLSN2679602 Genevestigator:Q9LNP9 Uniprot:Q9LNP9
Root	Isotig05562	20	1	4.193	1.37E-05	TAIR|locus:2030230 - symbol:HAB1 "AT1G72770" species:3702 "Arabidopsis thaliana" [GO:0004722 "protein serine/threonine phosphatase activity" evidence=ISS;IDA] [GO:0006470 "protein dephosphorylation" evidence=IEA] [GO:0008287 "protein serine/threonine phosphatase complex" evidence=IEA] [GO:0005515 "protein binding" evidence=IPI] InterPro:IPR000222 InterPro:IPR001932 Pfam:PF00481 PROSITE:PS01032 SMART:SM00331

Tissue	Identification	# of reads (stress)	# of reads (control)	Fold Change	p-value	Description
						SMART:SM00332 EMBL:CP002684 GenomeReviews:CT485782_GR GO:GO:0005634 GO:GO:0005737 GO:GO:0005515 GO:GO:0006470 GO:GO:0009738 GO:GO:0004722 GO:GO:0046872 EMBL:AC010926 eggNOG:COG0631 Gene3D:G3DSA:3.60.40.10 SUPFAM:SSF81606 GO:GO:0008287 InterPro:IPR015655 PANTHER:PTHR13832 HOGENOM:HBG747569 ProtClustDB:CLSN2679602 EMBL:AJ003119 EMBL:BT015409 EMBL:AK230171 EMBL:AK318665 IPI:IPI00538917 IPI:IPI00846864 PIR:F96752 RefSeq:NP_001077815.1 RefSeq:NP_001185385.1 RefSeq:NP_177421.1 UniGene:At.46635 UniGene:At.67356 PDB:3KB3 PDB:3NMT PDB:3QN1 PDB:3RT0 PDBsum:3KB3 PDBsum:3NMT PDBsum:3QN1 PDBsum:3RT0 ProteinModelPortal:Q9CAJ0 SMR:Q9CAJ0 DIP:DIP-48988N IntAct:Q9CAJ0 STRING:Q9CAJ0 PRIDE:Q9CAJ0 EnsemblPlants:AT1G72770.1 EnsemblPlants:AT1G72770.3 GeneID:843609 KEGG:ath:AT1G72770 TAIR:At1g72770 InParanoid:Q9CAJ0 OMA:YARIENA PhylomeDB:Q9CAJ0 Genevestigator:Q9CAJ0 Uniprot:Q9CAJ0
Root	Isotig05574	9	39	-2.244	1.90E-06	No hit
Root	Isotig05581	9	27	-1.714	0.000905257	No hit
Root	Isotig05583	4	20	-2.451	0.000325095	No hit
Root	Isotig05613	13	1	3.571	0.000882483	TAIR|locus:2091117 - symbol:IBM1 "AT3G07610" species:3702 "Arabidopsis thaliana" [GO:0003700 "sequence-specific DNA binding transcription factor activity" evidence=ISS] [GO:0005575 "cellular_component" evidence=ND] [GO:0009555 "pollen development" evidence=IMP] [GO:0009908 "flower development" evidence=IMP] [GO:0032454 "histone demethylase activity (H3-K9 specific)" evidence=ISS] [GO:0032776 "DNA methylation on cytosine" evidence=IGI;IMP] [GO:0033169 "histone H3-K9 demethylation" evidence=IGI;IMP] [GO:0048366 "leaf development" evidence=IMP] InterPro:IPR003347 Pfam:PF02373 PROSITE:PS51184 SMART:SM00558 EMBL:CP002686 GenomeReviews:BA000014_GR GO:GO:0009555 GO:GO:0048366 GO:GO:0009908 EMBL:AC009176 GO:GO:0033169 InterPro:IPR013129 HOGENOM:HBG633312 IPI:IPI00530050 RefSeq:NP_187418.1 UniGene:At.18334 ProteinModelPortal:Q9SSE9 STRING:Q9SSE9 PRIDE:Q9SSE9 EnsemblPlants:AT3G07610.1 GeneID:819952 KEGG:ath:AT3G07610 TAIR:At3g07610 InParanoid:Q9SSE9 OMA:PDDKPKG PhylomeDB:Q9SSE9 ProtClustDB:CLSN2684849 Genevestigator:Q9SSE9 GO:GO:0032776 Uniprot:Q9SSE9
Root	Isotig05614	15	1	3.778	0.000266457	TAIR|locus:2091117 - symbol:IBM1 "AT3G07610" species:3702 "Arabidopsis thaliana" [GO:0003700 "sequence-specific DNA binding transcription factor activity" evidence=ISS] [GO:0005575 "cellular_component" evidence=ND] [GO:0009555 "pollen development" evidence=IMP] [GO:0009908 "flower development" evidence=IMP] [GO:0032454 "histone demethylase activity (H3-K9 specific)" evidence=ISS] [GO:0032776 "DNA methylation on cytosine" evidence=IGI;IMP] [GO:0033169 "histone H3-K9 demethylation" evidence=IGI;IMP] [GO:0048366 "leaf development" evidence=IMP] InterPro:IPR003347 Pfam:PF02373 PROSITE:PS51184 SMART:SM00558 EMBL:CP002686 GenomeReviews:BA000014_GR GO:GO:0009555 GO:GO:0048366 GO:GO:0009908 EMBL:AC009176 GO:GO:0033169 InterPro:IPR013129 HOGENOM:HBG633312 IPI:IPI00530050 RefSeq:NP_187418.1 UniGene:At.18334 ProteinModelPortal:Q9SSE9 STRING:Q9SSE9 PRIDE:Q9SSE9 EnsemblPlants:AT3G07610.1 GeneID:819952 KEGG:ath:AT3G07610 TAIR:At3g07610 InParanoid:Q9SSE9 OMA:PDDKPKG PhylomeDB:Q9SSE9 ProtClustDB:CLSN2684849 Genevestigator:Q9SSE9 GO:GO:0032776 Uniprot:Q9SSE9
Root	Isotig05634	15	44	-1.682	2.92E-05	TAIR|locus:2043844 - symbol:SPX2 "AT2G26660" species:3702 "Arabidopsis thaliana" [GO:0003674 "molecular_function" evidence=ND] [GO:0005634 "nucleus" evidence=IDA] [GO:0016036 "cellular response to phosphate starvation" evidence=IEP] GO:GO:0005634 EMBL:CP002685 GenomeReviews:CT485783_GR GO:GO:0016036 EMBL:AC003105 InterPro:IPR004331 Pfam:PF03105 PROSITE:PS51382 ProtClustDB:CLSN2683415 EMBL:AK118537 EMBL:BT003688 IPI:IPI00529925 PIR:C84663 RefSeq:NP_180234.1 UniGene:At.38863 PRIDE:O48781 EnsemblPlants:AT2G26660.1 GeneID:817207 KEGG:ath:AT2G26660 TAIR:At2g26660 eggNOG:NOG311509 HOGENOM:HBG593729 InParanoid:O48781 OMA:EMINIKK PhylomeDB:O48781 Genevestigator:O48781 Uniprot:O48781
Root	Isotig05635	10	35	-1.936	4.01E-05	TAIR|locus:2149254 - symbol:SPX1 "AT5G20150" species:3702 "Arabidopsis thaliana" [GO:0003674 "molecular_function" evidence=ND] [GO:0005634 "nucleus" evidence=IDA] [GO:0016036 "cellular response to phosphate starvation" evidence=IEP] [GO:0080040 "positive regulation of cellular response to phosphate starvation" evidence=IMP] GO:GO:0005634 EMBL:CP002688 GenomeReviews:BA000015_GR EMBL:AF296825 InterPro:IPR004331 Pfam:PF03105 PROSITE:PS51382 EMBL:AY075605 EMBL:BT000868 EMBL:AY087206 IPI:IPI00537462 RefSeq:NP_197515.1 UniGene:At.19659 IntAct:Q8LBH4 STRING:Q8LBH4 PRIDE:Q8LBH4 DNASU:832137 EnsemblPlants:AT5G20150.1 GeneID:832137 KEGG:ath:AT5G20150 TAIR:At5g20150

Tissue	Identification	# of reads (stress)	# of reads (control)	Fold Change	p-value	Description
						eggNOG:NOG289536 InParanoid:Q8RY68 OMA:NGLDETW PhylomeDB:Q8LBH4 ProtClustDB:CLSN2683415 Genevestigator:Q8LBH4 Uniprot:Q8LBH4
Root	Isotig05636	14	40	-1.644	8.83E-05	TAIR|locus:2043844 - symbol:SPX2 "AT2G26660" species:3702 "Arabidopsis thaliana" [GO:0003674 "molecular_function" evidence=ND] [GO:0005634 "nucleus" evidence=IDA] [GO:0016036 "cellular response to phosphate starvation" evidence=IEP] GO:GO:0005634 EMBL:CP002685 GenomeReviews:CT485783_GR GO:GO:0016036 EMBL:AC003105 InterPro:IPR004331 Pfam:PF03105 PROSITE:PS51382 ProtClustDB:CLSN2683415 EMBL:AK118537 EMBL:BT003688 IPI:IPI00529925 PIR:C84663 RefSeq:NP_180234.1 UniGene:At.38863 PRIDE:O48781 EnsemblPlants:AT2G26660.1 GeneID:817207 KEGG:ath:AT2G26660 TAIR:At2g26660 eggNOG:NOG311509 HOGENOM:HBG593729 InParanoid:O48781 OMA:EMINIKK PhylomeDB:O48781 Genevestigator:O48781 Uniprot:O48781
Root	Isotig05649	2	22	-3.588	5.84E-06	TAIR|locus:2181082 - symbol:AT5G01750 "AT5G01750" species:3702 "Arabidopsis thaliana" [GO:0003674 "molecular_function" evidence=ND] [GO:0008150 "biological_process" evidence=ND] [GO:0009507 "chloroplast" evidence=IDA] INTERPRO:IPR000007 EMBL:CP002688 GenomeReviews:BA000015_GR GO:GO:0009507 EMBL:AL162351 InterPro:IPR007612 Pfam:PF04525 SUPFAM:SSF54518 HOGENOM:HBG595263 EMBL:AF372877 EMBL:AY142034 EMBL:AY085369 IPI:IPI00516693 IPI:IPI00533737 PIR:T48196 RefSeq:NP_568095.1 RefSeq:NP_850751.1 UniGene:At.21392 PDB:1ZXU PDB:2Q4M PDBsum:1ZXU PDBsum:2Q4M ProteinModelPortal:Q9LZX1 SMR:Q9LZX1 IntAct:Q9LZX1 STRING:Q9LZX1 PRIDE:Q9LZX1 DNASU:831684 EnsemblPlants:AT5G01750.2 GeneID:831684 KEGG:ath:AT5G01750 TAIR:At5g01750 eggNOG:NOG242955 InParanoid:Q9LZX1 OMA:QMHRKHT PhylomeDB:Q9LZX1 ProtClustDB:CLSN2689401 Genevestigator:Q9LZX1 GermOnline:AT5G01750 Uniprot:Q9LZX1
Root	Isotig05650	2	22	-3.588	5.84E-06	TAIR|locus:2181082 - symbol:AT5G01750 "AT5G01750" species:3702 "Arabidopsis thaliana" [GO:0003674 "molecular_function" evidence=ND] [GO:0008150 "biological_process" evidence=ND] [GO:0009507 "chloroplast" evidence=IDA] INTERPRO:IPR000007 EMBL:CP002688 GenomeReviews:BA000015_GR GO:GO:0009507 EMBL:AL162351 InterPro:IPR007612 Pfam:PF04525 SUPFAM:SSF54518 HOGENOM:HBG595263 EMBL:AF372877 EMBL:AY142034 EMBL:AY085369 IPI:IPI00516693 IPI:IPI00533737 PIR:T48196 RefSeq:NP_568095.1 RefSeq:NP_850751.1 UniGene:At.21392 PDB:1ZXU PDB:2Q4M PDBsum:1ZXU PDBsum:2Q4M ProteinModelPortal:Q9LZX1 SMR:Q9LZX1 IntAct:Q9LZX1 STRING:Q9LZX1 PRIDE:Q9LZX1 DNASU:831684 EnsemblPlants:AT5G01750.2 GeneID:831684 KEGG:ath:AT5G01750 TAIR:At5g01750 eggNOG:NOG242955 InParanoid:Q9LZX1 OMA:QMHRKHT PhylomeDB:Q9LZX1 ProtClustDB:CLSN2689401 Genevestigator:Q9LZX1 GermOnline:AT5G01750 Uniprot:Q9LZX1
Root	Isotig05651	2	22	-3.588	5.84E-06	TAIR|locus:2181082 - symbol:AT5G01750 "AT5G01750" species:3702 "Arabidopsis thaliana" [GO:0003674 "molecular_function" evidence=ND] [GO:0008150 "biological_process" evidence=ND] [GO:0009507 "chloroplast" evidence=IDA] INTERPRO:IPR000007 EMBL:CP002688 GenomeReviews:BA000015_GR GO:GO:0009507 EMBL:AL162351 InterPro:IPR007612 Pfam:PF04525 SUPFAM:SSF54518 HOGENOM:HBG595263 EMBL:AF372877 EMBL:AY142034 EMBL:AY085369 IPI:IPI00516693 IPI:IPI00533737 PIR:T48196 RefSeq:NP_568095.1 RefSeq:NP_850751.1 UniGene:At.21392 PDB:1ZXU PDB:2Q4M PDBsum:1ZXU PDBsum:2Q4M ProteinModelPortal:Q9LZX1 SMR:Q9LZX1 IntAct:Q9LZX1 STRING:Q9LZX1 PRIDE:Q9LZX1 DNASU:831684 EnsemblPlants:AT5G01750.2 GeneID:831684 KEGG:ath:AT5G01750 TAIR:At5g01750 eggNOG:NOG242955 InParanoid:Q9LZX1 OMA:QMHRKHT PhylomeDB:Q9LZX1 ProtClustDB:CLSN2689401 Genevestigator:Q9LZX1 GermOnline:AT5G01750 Uniprot:Q9LZX1
Root	Isotig05658	0	83	-7.504	9.65E-21	TAIR|locus:2009769 - symbol:AT1G65870 "AT1G65870" species:3702 "Arabidopsis thaliana" [GO:0003674 "molecular_function" evidence=ND] [GO:0006952 "defense response" evidence=ISS] [GO:0009807 "lignan biosynthetic process" evidence=ISS] [GO:0005618 "cell wall" evidence=IDA] Pfam:PF03018 EMBL:CP002684 GenomeReviews:CT485782_GR GO:GO:0005618 EMBL:AC009513 InterPro:IPR004265 HOGENOM:HBG605442 IPI:IPI00539166 PIR:G96682 RefSeq:NP_176762.1 UniGene:At.66110 EnsemblPlants:AT1G65870.1 GeneID:842898 KEGG:ath:AT1G65870 TAIR:At1g65870 eggNOG:NOG288717 InParanoid:Q9SS03 OMA:ATVEYNI PhylomeDB:Q9SS03 Genevestigator:Q9SS03 Uniprot:Q9SS03
Root	Isotig05659	0	20	-5.451	2.00E-06	TAIR|locus:2009769 - symbol:AT1G65870 "AT1G65870" species:3702 "Arabidopsis thaliana" [GO:0003674 "molecular_function" evidence=ND] [GO:0006952 "defense response" evidence=ISS] [GO:0009807 "lignan biosynthetic process" evidence=ISS] [GO:0005618 "cell wall" evidence=IDA] Pfam:PF03018 EMBL:CP002684 GenomeReviews:CT485782_GR GO:GO:0005618 EMBL:AC009513 InterPro:IPR004265 HOGENOM:HBG605442 IPI:IPI00539166 PIR:G96682 RefSeq:NP_176762.1 UniGene:At.66110 EnsemblPlants:AT1G65870.1

Tissue	Identification	# of reads (stress)	# of reads (control)	Fold Change	p-value	Description
						GeneID:842898 KEGG:ath:AT1G65870 TAIR:At1g65870 eggNOG:NOG288717 InParanoid:Q9SS03 OMA:ATVEYNI PhylomeDB:Q9SS03 Genevestigator:Q9SS03 Uniprot:Q9SS03
Root	Isotig05660	0	85	-7.538	3.78E-21	TAIR|locus:2009769 - symbol:AT1G65870 "AT1G65870" species:3702 "Arabidopsis thaliana" [GO:0003674 "molecular_function" evidence=ND] [GO:0006952 "defense response" evidence=ISS] [GO:0009807 "lignan biosynthetic process" evidence=ISS] [GO:0005618 "cell wall" evidence=IDA] Pfam:PF03018 EMBL:CP002684 GenomeReviews:CT485782_GR GO:GO:0005618 EMBL:AC009513 InterPro:IPR004265 HOGENOM:HBG605442 IPI:IPI00539166 PIR:G96682 RefSeq:NP_176762.1 UniGene:At.66110 EnsemblPlants:AT1G65870.1 GeneID:842898 KEGG:ath:AT1G65870 TAIR:At1g65870 eggNOG:NOG288717 InParanoid:Q9SS03 OMA:ATVEYNI PhylomeDB:Q9SS03 Genevestigator:Q9SS03 Uniprot:Q9SS03
Root	Isotig05712	0	19	-5.377	3.67E-06	TAIR|locus:4010713753 - symbol:AT3G17675 species:3702 "Arabidopsis thaliana" [GO:0005507 "copper ion binding" evidence=IEA] [GO:0009055 "electron carrier activity" evidence=IEA] InterPro:IPR003245 Pfam:PF02298 ProDom:PD003122 PROSITE:PS51485 GenomeReviews:BA000014_GR GO:GO:0009055 GO:GO:0005507 EMBL:AB022219 InterPro:IPR008972 Gene3D:G3DSA:2.60.40.420 SUPFAM:SSF49503 HOGENOM:HBG748847 HSSP:P29602 IPI:IPI00534093 ProteinModelPortal:Q9LUM8 SMR:Q9LUM8 PRIDE:Q9LUM8 TAIR:At3g17675 eggNOG:NOG261136 PhylomeDB:Q9LUM8 Genevestigator:Q9LUM8 Uniprot:Q9LUM8
Root	Isotig05713	0	18	-5.299	6.77E-06	TAIR|locus:4010713753 - symbol:AT3G17675 species:3702 "Arabidopsis thaliana" [GO:0005507 "copper ion binding" evidence=IEA] [GO:0009055 "electron carrier activity" evidence=IEA] InterPro:IPR003245 Pfam:PF02298 ProDom:PD003122 PROSITE:PS51485 GenomeReviews:BA000014_GR GO:GO:0009055 GO:GO:0005507 EMBL:AB022219 InterPro:IPR008972 Gene3D:G3DSA:2.60.40.420 SUPFAM:SSF49503 HOGENOM:HBG748847 HSSP:P29602 IPI:IPI00534093 ProteinModelPortal:Q9LUM8 SMR:Q9LUM8 PRIDE:Q9LUM8 TAIR:At3g17675 eggNOG:NOG261136 PhylomeDB:Q9LUM8 Genevestigator:Q9LUM8 Uniprot:Q9LUM8
Root	Isotig05714	0	18	-5.299	6.77E-06	TAIR|locus:4010713753 - symbol:AT3G17675 species:3702 "Arabidopsis thaliana" [GO:0005507 "copper ion binding" evidence=IEA] [GO:0009055 "electron carrier activity" evidence=IEA] InterPro:IPR003245 Pfam:PF02298 ProDom:PD003122 PROSITE:PS51485 GenomeReviews:BA000014_GR GO:GO:0009055 GO:GO:0005507 EMBL:AB022219 InterPro:IPR008972 Gene3D:G3DSA:2.60.40.420 SUPFAM:SSF49503 HOGENOM:HBG748847 HSSP:P29602 IPI:IPI00534093 ProteinModelPortal:Q9LUM8 SMR:Q9LUM8 PRIDE:Q9LUM8 TAIR:At3g17675 eggNOG:NOG261136 PhylomeDB:Q9LUM8 Genevestigator:Q9LUM8 Uniprot:Q9LUM8
Root	Isotig05718	0	16	-5.129	2.34E-05	TAIR|locus:2143651 - symbol:HTA7 "histone H2A 7" species:3702 "Arabidopsis thaliana" [GO:0003677 "DNA binding" evidence=IEA;ISS] [GO:0006334 "nucleosome assembly" evidence=IEA] [GO:0005730 "nucleolus" evidence=IDA] InterPro:IPR002119 InterPro:IPR007125 InterPro:IPR009072 Pfam:PF00125 PRINTS:PR00620 PROSITE:PS00046 SMART:SM00414 EMBL:CP002688 GenomeReviews:BA000015_GR GO:GO:0005730 GO:GO:0003677 Gene3D:G3DSA:1.10.20.10 SUPFAM:SSF47113 EMBL:AC007478 GO:GO:0006334 GO:GO:0000786 EMBL:AC069556 eggNOG:COG5262 HOGENOM:HBG610736 KO:K11251 EMBL:AF385711 EMBL:AY078019 IPI:IPI00533679 RefSeq:NP_198119.1 UniGene:At.19946 ProteinModelPortal:Q94F49 SMR:Q94F49 STRING:Q94F49 PRIDE:Q94F49 EnsemblPlants:AT5G27670.1 GeneID:832829 KEGG:ath:AT5G27670 TAIR:At5g27670 InParanoid:Q94F49 OMA:CLAIRND PhylomeDB:Q94F49 ProtClustDB:CLSN2916652 Genevestigator:Q94F49 GermOnline:AT5G27670 Uniprot:Q94F49
Root	Isotig05719	0	16	-5.129	2.34E-05	TAIR|locus:2143651 - symbol:HTA7 "histone H2A 7" species:3702 "Arabidopsis thaliana" [GO:0003677 "DNA binding" evidence=IEA;ISS] [GO:0006334 "nucleosome assembly" evidence=IEA] [GO:0005730 "nucleolus" evidence=IDA] InterPro:IPR002119 InterPro:IPR007125 InterPro:IPR009072 Pfam:PF00125 PRINTS:PR00620 PROSITE:PS00046 SMART:SM00414 EMBL:CP002688 GenomeReviews:BA000015_GR GO:GO:0005730 GO:GO:0003677 Gene3D:G3DSA:1.10.20.10 SUPFAM:SSF47113 EMBL:AC007478 GO:GO:0006334 GO:GO:0000786 EMBL:AC069556 eggNOG:COG5262 HOGENOM:HBG610736 KO:K11251 EMBL:AF385711 EMBL:AY078019 IPI:IPI00533679 RefSeq:NP_198119.1 UniGene:At.19946 ProteinModelPortal:Q94F49 SMR:Q94F49 STRING:Q94F49 PRIDE:Q94F49 EnsemblPlants:AT5G27670.1 GeneID:832829 KEGG:ath:AT5G27670 TAIR:At5g27670 InParanoid:Q94F49 OMA:CLAIRND PhylomeDB:Q94F49 ProtClustDB:CLSN2916652 Genevestigator:Q94F49 GermOnline:AT5G27670 Uniprot:Q94F49
Root	Isotig05720	0	17	-5.216	1.26E-05	TAIR|locus:2143651 - symbol:HTA7 "histone H2A 7" species:3702 "Arabidopsis thaliana" [GO:0003677 "DNA binding" evidence=IEA;ISS] [GO:0006334 "nucleosome assembly" evidence=IEA] [GO:0005730 "nucleolus" evidence=IDA] InterPro:IPR002119 InterPro:IPR007125 InterPro:IPR009072 Pfam:PF00125 PRINTS:PR00620 PROSITE:PS00046 SMART:SM00414 EMBL:CP002688 GenomeReviews:BA000015_GR GO:GO:0005730 GO:GO:0003677 Gene3D:G3DSA:1.10.20.10 SUPFAM:SSF47113 EMBL:AC007478 GO:GO:0006334

Tissue	Identification	# of reads (stress)	# of reads (control)	Fold Change	p-value	Description
						GO:GO:0000786 EMBL:AC069556 eggNOG:COG5262 HOGENOM:HBG610736 KO:K11251 EMBL:AF385711 EMBL:AY078019 IPI:IPI00533679 RefSeq:NP_198119.1 UniGene:At.19946 ProteinModelPortal:Q94F49 SMR:Q94F49 STRING:Q94F49 PRIDE:Q94F49 EnsemblPlants:AT5G27670.1 GeneID:832829 KEGG:ath:AT5G27670 TAIR:At5g27670 InParanoid:Q94F49 OMA:CLAIRND PhylomeDB:Q94F49 ProtClustDB:CLSN2916652 Genevestigator:Q94F49 GermOnline:AT5G27670 Uniprot:Q94F49
Root	Isotig05744	34	10	1.637	0.000568537	TAIR|locus:2005614 - symbol:XRN3 "AT1G75660" species:3702 "Arabidopsis thaliana" [GO:0004534 "5'-3' exoribonuclease activity" evidence=IGI;ISS] [GO:0005634 "nucleus" evidence=IDA] [GO:0010587 "miRNA catabolic process" evidence=IMP] InterPro:IPR001878 InterPro:IPR004859 InterPro:IPR017151 Pfam:PF00098 Pfam:PF03159 PIRSF:PIRSF037239 PROSITE:PS50158 SMART:SM00343 EMBL:CP002684 GenomeReviews:CT485782_GR GO:GO:0005634 GO:GO:0008270 GO:GO:0006397 GO:GO:0003676 Gene3D:G3DSA:4.10.60.10 GO:GO:0010587 EMBL:AC006434 eggNOG:COG5049 GO:GO:0004534 HOGENOM:HBG592534 KO:K12619 EMBL:AF286719 EMBL:AY136383 EMBL:BT000175 IPI:IPI00542756 IPI:IPI00903453 PIR:D96786 RefSeq:NP_565114.1 UniGene:At.23685 ProteinModelPortal:Q9FQ03 SMR:Q9FQ03 STRING:Q9FQ03 PRIDE:Q9FQ03 EnsemblPlants:AT1G75660.1 GeneID:843900 KEGG:ath:AT1G75660 TAIR:At1g75660 InParanoid:Q9FQ03 OMA:ILREVVY PhylomeDB:Q9FQ03 ArrayExpress:Q8L7A3 Genevestigator:Q9FQ03 Uniprot:Q9FQ03
Root	Isotig05745	34	10	1.637	0.000568537	TAIR|locus:2005614 - symbol:XRN3 "AT1G75660" species:3702 "Arabidopsis thaliana" [GO:0004534 "5'-3' exoribonuclease activity" evidence=IGI;ISS] [GO:0005634 "nucleus" evidence=IDA] [GO:0010587 "miRNA catabolic process" evidence=IMP] InterPro:IPR001878 InterPro:IPR004859 InterPro:IPR017151 Pfam:PF00098 Pfam:PF03159 PIRSF:PIRSF037239 PROSITE:PS50158 SMART:SM00343 EMBL:CP002684 GenomeReviews:CT485782_GR GO:GO:0005634 GO:GO:0008270 GO:GO:0006397 GO:GO:0003676 Gene3D:G3DSA:4.10.60.10 GO:GO:0010587 EMBL:AC006434 eggNOG:COG5049 GO:GO:0004534 HOGENOM:HBG592534 KO:K12619 EMBL:AF286719 EMBL:AY136383 EMBL:BT000175 IPI:IPI00542756 IPI:IPI00903453 PIR:D96786 RefSeq:NP_565114.1 UniGene:At.23685 ProteinModelPortal:Q9FQ03 SMR:Q9FQ03 STRING:Q9FQ03 PRIDE:Q9FQ03 EnsemblPlants:AT1G75660.1 GeneID:843900 KEGG:ath:AT1G75660 TAIR:At1g75660 InParanoid:Q9FQ03 OMA:ILREVVY PhylomeDB:Q9FQ03 ArrayExpress:Q8L7A3 Genevestigator:Q9FQ03 Uniprot:Q9FQ03
Root	Isotig05794	6	38	-2.792	1.04E-07	TAIR|locus:2827943 - symbol:STY8 "AT2G17700" species:3702 "Arabidopsis thaliana" [GO:0004672 "protein kinase activity" evidence=ISS] [GO:0004712 "protein serine/threonine/tyrosine kinase activity" evidence=ISS] [GO:0005829 "cytosol" evidence=IDA] [GO:0004674 "protein serine/threonine kinase activity" evidence=IDA] [GO:0009658 "chloroplast organization" evidence=IGI] InterPro:IPR000719 InterPro:IPR001245 InterPro:IPR002912 InterPro:IPR008271 InterPro:IPR011009 Pfam:PF01842 Pfam:PF07714 PRINTS:PR00109 PROSITE:PS00108 PROSITE:PS50011 GO:GO:0005829 GO:GO:0005524 EMBL:CP002685 GenomeReviews:CT485783_GR eggNOG:COG0515 SUPFAM:SSF56112 GO:GO:0004674 HOGENOM:HBG755340 GO:GO:0016597 GO:GO:0009658 HSSP:P41240 EMBL:AY070086 EMBL:AY096470 IPI:IPI00530122 PIR:D84555 PIR:T08864 RefSeq:NP_179361.1 UniGene:At.14679 ProteinModelPortal:O22558 SMR:O22558 PRIDE:O22558 EnsemblPlants:AT2G17700.1 GeneID:816278 KEGG:ath:AT2G17700 TAIR:At2g17700 InParanoid:O22558 OMA:DESESCG PhylomeDB:O22558 ProtClustDB:CLSN2683904 ArrayExpress:O22558 Genevestigator:O22558 Uniprot:O22558
Root	Isotig05795	6	38	-2.792	1.04E-07	TAIR|locus:2827943 - symbol:STY8 "AT2G17700" species:3702 "Arabidopsis thaliana" [GO:0004672 "protein kinase activity" evidence=ISS] [GO:0004712 "protein serine/threonine/tyrosine kinase activity" evidence=ISS] [GO:0005829 "cytosol" evidence=IDA] [GO:0004674 "protein serine/threonine kinase activity" evidence=IDA] [GO:0009658 "chloroplast organization" evidence=IGI] InterPro:IPR000719 InterPro:IPR001245 InterPro:IPR002912 InterPro:IPR008271 InterPro:IPR011009 Pfam:PF01842 Pfam:PF07714 PRINTS:PR00109 PROSITE:PS00108 PROSITE:PS50011 GO:GO:0005829 GO:GO:0005524 EMBL:CP002685 GenomeReviews:CT485783_GR eggNOG:COG0515 SUPFAM:SSF56112 GO:GO:0004674 HOGENOM:HBG755340 GO:GO:0016597 GO:GO:0009658 HSSP:P41240 EMBL:AY070086 EMBL:AY096470 IPI:IPI00530122 PIR:D84555 PIR:T08864 RefSeq:NP_179361.1 UniGene:At.14679 ProteinModelPortal:O22558 SMR:O22558 PRIDE:O22558 EnsemblPlants:AT2G17700.1 GeneID:816278 KEGG:ath:AT2G17700 TAIR:At2g17700 InParanoid:O22558 OMA:DESESCG PhylomeDB:O22558 ProtClustDB:CLSN2683904 ArrayExpress:O22558 Genevestigator:O22558 Uniprot:O22558

Tissue	Identification	# of reads (stress)	# of reads (control)	Fold Change	p-value	Description
Root	Isotig05862	65	0	6.893	6.96E-16	TAIR|locus:2156652 - symbol:LTI65 "AT5G52300" species:3702 "Arabidopsis thaliana" [GO:0003674 "molecular_function" evidence=ND] [GO:0005575 "cellular_component" evidence=ND] [GO:0009738 "abscisic acid mediated signaling pathway" evidence=IEP] [GO:0009414 "response to water deprivation" evidence=IEP] [GO:0009651 "response to salt stress" evidence=IEP] [GO:0009737 "response to abscisic acid stimulus" evidence=IEP] [GO:0010150 "leaf senescence" evidence=IMP] [GO:0009409 "response to cold" evidence=IEP] EMBL:CP002688 GenomeReviews:BA000015_GR GO:GO:0009738 GO:GO:0010150 GO:GO:0009409 GO:GO:0009651 GO:GO:0009414 EMBL:AB019226 EMBL:X67670 EMBL:D13044 EMBL:AY081282 EMBL:AY128731 EMBL:Z34014 IPI:IPI00526103 PIR:S30153 RefSeq:NP_200043.2 UniGene:At.43012 STRING:Q04980 PRIDE:Q04980 EnsemblPlants:AT5G52300.1 GeneID:835306 KEGG:ath:AT5G52300 TAIR:At5g52300 eggNOG:NOG281420 HOGENOM:HBG027913 InParanoid:Q04980 OMA:APVYESS PhylomeDB:Q04980 ProtClustDB:PLN03021 ArrayExpress:Q04980 Genevestigator:Q04980 InterPro:IPR012418 Pfam:PF07918 Uniprot:Q04980
Root	Isotig05863	72	0	7.041	2.73E-17	TAIR|locus:2156652 - symbol:LTI65 "AT5G52300" species:3702 "Arabidopsis thaliana" [GO:0003674 "molecular_function" evidence=ND] [GO:0005575 "cellular_component" evidence=ND] [GO:0009738 "abscisic acid mediated signaling pathway" evidence=IEP] [GO:0009414 "response to water deprivation" evidence=IEP] [GO:0009651 "response to salt stress" evidence=IEP] [GO:0009737 "response to abscisic acid stimulus" evidence=IEP] [GO:0010150 "leaf senescence" evidence=IMP] [GO:0009409 "response to cold" evidence=IEP] EMBL:CP002688 GenomeReviews:BA000015_GR GO:GO:0009738 GO:GO:0010150 GO:GO:0009409 GO:GO:0009651 GO:GO:0009414 EMBL:AB019226 EMBL:X67670 EMBL:D13044 EMBL:AY081282 EMBL:AY128731 EMBL:Z34014 IPI:IPI00526103 PIR:S30153 RefSeq:NP_200043.2 UniGene:At.43012 STRING:Q04980 PRIDE:Q04980 EnsemblPlants:AT5G52300.1 GeneID:835306 KEGG:ath:AT5G52300 TAIR:At5g52300 eggNOG:NOG281420 HOGENOM:HBG027913 InParanoid:Q04980 OMA:APVYESS PhylomeDB:Q04980 ProtClustDB:PLN03021 ArrayExpress:Q04980 Genevestigator:Q04980 InterPro:IPR012418 Pfam:PF07918 Uniprot:Q04980
Root	Isotig05870	2	30	-4.036	3.63E-08	UNIPROTKB|Q70KG3 - symbol:RAFTIN1B "Protein RAFTIN 1B" species:4565 "Triticum aestivum" [GO:0009555 "pollen development" evidence=ISS] [GO:0043668 "exine" evidence=IDA] GO:GO:0009555 InterPro:IPR004873 Pfam:PF03181 PROSITE:PS51277 GO:GO:0043668 EMBL:AJ575664 EMBL:AJ575665 UniGene:Ta.4176 Gramene:Q70KG3 Uniprot:Q70KG3
Root	Isotig05871	2	35	-4.258	1.54E-09	UNIPROTKB|Q70KG3 - symbol:RAFTIN1B "Protein RAFTIN 1B" species:4565 "Triticum aestivum" [GO:0009555 "pollen development" evidence=ISS] [GO:0043668 "exine" evidence=IDA] GO:GO:0009555 InterPro:IPR004873 Pfam:PF03181 PROSITE:PS51277 GO:GO:0043668 EMBL:AJ575664 EMBL:AJ575665 UniGene:Ta.4176 Gramene:Q70KG3 Uniprot:Q70KG3
Root	Isotig05876	4	86	-4.555	6.56E-22	TAIR|locus:2053654 - symbol:NIR1 "AT2G15620" species:3702 "Arabidopsis thaliana" [GO:0047889 "ferredoxin-nitrate reductase activity" evidence=ISS] [GO:0005739 "mitochondrion" evidence=IDA] [GO:0009507 "chloroplast" evidence=IDA] [GO:0009570 "chloroplast stroma" evidence=IDA] [GO:0016020 "membrane" evidence=IDA] [GO:0048046 "apoplast" evidence=IDA] [GO:0010167 "response to nitrate" evidence=IEP] [GO:0005829 "cytosol" evidence=RCA] [GO:0050421 "nitrite reductase (NO-forming) activity" evidence=ISS] InterPro:IPR005117 InterPro:IPR006066 InterPro:IPR006067 Pfam:PF01077 Pfam:PF03460 PRINTS:PR00397 PROSITE:PS00365 GO:GO:0005739 GO:GO:0048046 GO:GO:0009570 EMBL:CP002685 GenomeReviews:CT485783_GR GO:GO:0016020 GO:GO:0006810 GO:GO:0051539 GO:GO:0020037 GO:GO:0022900 GO:GO:0042128 GO:GO:0010167 EMBL:AC006248 eggNOG:COG0155 Gene3D:G3DSA:3.90.480.10 SUPFAM:SSF55124 EMBL:D14824 EMBL:AB006032 EMBL:AF360320 EMBL:AY045608 EMBL:AY093995 EMBL:AY142685 EMBL:BT000685 EMBL:BT000686 IPI:IPI00533476 PIR:C84531 RefSeq:NP_179164.1 UniGene:At.21870 ProteinModelPortal:Q39161 SMR:Q39161 IntAct:Q39161 STRING:Q39161 PRIDE:Q39161 EnsemblPlants:AT2G15620.1 GeneID:816055 KEGG:ath:AT2G15620 GeneFarm:4362 TAIR:At2g15620 HOGENOM:HBG578916 InParanoid:Q39161 KO:K00366 OMA:DKYFMLR PhylomeDB:Q39161 ProtClustDB:PLN02431 ArrayExpress:Q39161 Genevestigator:Q39161 GermOnline:AT2G15620 GO:GO:0048307 Uniprot:Q39161
Root	Isotig05877	4	80	-4.451	2.67E-20	TAIR|locus:2053654 - symbol:NIR1 "AT2G15620" species:3702 "Arabidopsis thaliana" [GO:0047889 "ferredoxin-nitrate reductase activity" evidence=ISS] [GO:0005739 "mitochondrion" evidence=IDA] [GO:0009507 "chloroplast" evidence=IDA] [GO:0009570 "chloroplast stroma" evidence=IDA] [GO:0016020 "membrane" evidence=IDA] [GO:0048046 "apoplast" evidence=IDA] [GO:0010167 "response to nitrate" evidence=IEP] [GO:0005829 "cytosol"

Tissue	Identification	# of reads (stress)	# of reads (control)	Fold Change	p-value	Description
						evidence=RCA] [GO:0050421 "nitrite reductase (NO-forming) activity" evidence=ISS] InterPro:IPR005117 InterPro:IPR006066 InterPro:IPR006067 Pfam:PF01077 Pfam:PF03460 PRINTS:PR00397 PROSITE:PS00365 GO:GO:0005739 GO:GO:0048046 GO:GO:0009570 EMBL:CP002685 GenomeReviews:CT485783_GR GO:GO:0016020 GO:GO:0006810 GO:GO:0051539 GO:GO:0020037 GO:GO:0022900 GO:GO:0042128 GO:GO:0010167 EMBL:AC006248 eggNOG:COG0155 Gene3D:G3DSA:3.90.480.10 SUPFAM:SSF55124 EMBL:D14824 EMBL:AB006032 EMBL:AF360320 EMBL:AY045608 EMBL:AY093995 EMBL:AY142685 EMBL:BT000685 EMBL:BT000686 IPI:IPI00533476 PIR:C84531 RefSeq:NP_179164.1 UniGene:At.21870 ProteinModelPortal:Q39161 SMR:Q39161 IntAct:Q39161 STRING:Q39161 PRIDE:Q39161 EnsemblPlants:AT2G15620.1 GeneID:816055 KEGG:ath:AT2G15620 GeneFarm:4362 TAIR:At2g15620 HOGENOM:HBG578916 InParanoid:Q39161 KO:K00366 OMA:DKYFMLR PhylomeDB:Q39161 ProtClustDB:PLN02431 ArrayExpress:Q39161 Genevestigator:Q39161 GermOnline:AT2G15620 GO:GO:0048307 Uniprot:Q39161
Root	Isotig05884	36	7	2.234	1.28E-05	TAIR|locus:2155806 - symbol:FIP1[V] "AT5G58040" species:3702 "Arabidopsis thaliana" [GO:0005575 "cellular_component" evidence=ND] [GO:0008150 "biological_process" evidence=ND] [GO:0003723 "RNA binding" evidence=IDA] [GO:0005515 "protein binding" evidence=IPI] GenomeReviews:BA000015_GR GO:GO:0042802 GO:GO:0003723 EMBL:AB024029 InterPro:IPR007854 Pfam:PF05182 IPI:IPI00524599 IntAct:Q9FGU0 STRING:Q9FGU0 PRIDE:Q9FGU0 TAIR:At5g58040 HOGENOM:HBG595008 InParanoid:Q9FGU0 PhylomeDB:Q9FGU0 Genevestigator:Q9FGU0 Uniprot:Q9FGU0
Root	Isotig05885	6	23	-2.068	0.000510704	No hit
Root	Isotig05886	6	23	-2.068	0.000510704	No hit
Root	Isotig05911	57	20	1.382	8.22E-05	TAIR|locus:2037863 - symbol:MBD10 "AT1G15340" species:3702 "Arabidopsis thaliana" [GO:0003677 "DNA binding" evidence=ISS] [GO:0008150 "biological_process" evidence=ND] [GO:0008327 "methyl-CpG binding" evidence=ISS] [GO:0005634 "nucleus" evidence=IDA] [GO:0005829 "cytosol" evidence=IDA] InterPro:IPR001739 InterPro:IPR016177 Pfam:PF01429 PROSITE:PS50982 EMBL:CP002684 GenomeReviews:CT485782_GR GO:GO:0005829 GO:GO:0005634 GO:GO:0006355 GO:GO:0003677 GO:GO:0006351 EMBL:AC007591 SUPFAM:SSF54171 Gene3D:G3DSA:3.30.890.10 EMBL:AY094439 EMBL:BT020367 EMBL:AK230256 IPI:IPI00519005 IPI:IPI00785822 PIR:G86287 RefSeq:NP_001185003.1 RefSeq:NP_563971.1 UniGene:At.27594 UniGene:At.70811 ProteinModelPortal:Q9XI36 SMR:Q9XI36 STRING:Q9XI36 PRIDE:Q9XI36 ProMEX:Q9XI36 EnsemblPlants:AT1G15340.1 GeneID:838103 KEGG:ath:AT1G15340 TAIR:At1g15340 eggNOG:NOG328305 InParanoid:Q9XI36 OMA:VTTEANG PhylomeDB:Q9XI36 ProtClustDB:CLSN2917009 Genevestigator:Q9XI36 Uniprot:Q9XI36
Root	Isotig05912	61	20	1.480	1.80E-05	TAIR|locus:2037863 - symbol:MBD10 "AT1G15340" species:3702 "Arabidopsis thaliana" [GO:0003677 "DNA binding" evidence=ISS] [GO:0008150 "biological_process" evidence=ND] [GO:0008327 "methyl-CpG binding" evidence=ISS] [GO:0005634 "nucleus" evidence=IDA] [GO:0005829 "cytosol" evidence=IDA] InterPro:IPR001739 InterPro:IPR016177 Pfam:PF01429 PROSITE:PS50982 EMBL:CP002684 GenomeReviews:CT485782_GR GO:GO:0005829 GO:GO:0005634 GO:GO:0006355 GO:GO:0003677 GO:GO:0006351 EMBL:AC007591 SUPFAM:SSF54171 Gene3D:G3DSA:3.30.890.10 EMBL:AY094439 EMBL:BT020367 EMBL:AK230256 IPI:IPI00519005 IPI:IPI00785822 PIR:G86287 RefSeq:NP_001185003.1 RefSeq:NP_563971.1 UniGene:At.27594 UniGene:At.70811 ProteinModelPortal:Q9XI36 SMR:Q9XI36 STRING:Q9XI36 PRIDE:Q9XI36 ProMEX:Q9XI36 EnsemblPlants:AT1G15340.1 GeneID:838103 KEGG:ath:AT1G15340 TAIR:At1g15340 eggNOG:NOG328305 InParanoid:Q9XI36 OMA:VTTEANG PhylomeDB:Q9XI36 ProtClustDB:CLSN2917009 Genevestigator:Q9XI36 Uniprot:Q9XI36
Root	Isotig05948	0	46	-6.653	9.06E-13	UNIPROTKB|P47787 - symbol:TBXAS1 "Thromboxane-A synthase" species:9823 "Sus scrofa" [GO:0001516 "prostaglandin biosynthetic process" evidence=IEA] [GO:0004497 "monooxygenase activity" evidence=IEA] [GO:0004796 "thromboxane-A synthase activity" evidence=IEA] [GO:0005789 "endoplasmic reticulum membrane" evidence=IEA] [GO:0009055 "electron carrier activity" evidence=IEA] [GO:0016021 "integral to membrane" evidence=IEA] [GO:0016705 "oxidoreductase activity, acting on paired donors, with incorporation or reduction of molecular oxygen" evidence=IEA] [GO:0020037 "heme binding" evidence=IEA] InterPro:IPR001128 InterPro:IPR002401 InterPro:IPR017972 Pfam:PF00067 PRINTS:PR00385 PRINTS:PR00463 PROSITE:PS00086 GO:GO:0016021 GO:GO:0005789 GO:GO:0009055 GO:GO:0004497 GO:GO:0020037 Gene3D:G3DSA:1.10.630.10 SUPFAM:SSF48264 GO:GO:0016705 HOVERGEN:HBG108567 GO:GO:0001516 CTD:6916 KO:K01832 OrthoDB:EOG415GDB GO:GO:0004796 EMBL:L13128 RefSeq:NP_999211.1

Tissue	Identification	# of reads (stress)	# of reads (control)	Fold Change	p-value	Description
						UniGene:Ssc.158 ProteinModelPortal:P47787 STRING:P47787 GeneID:397112 KEGG:ssc:397112 eggNOG:NOG308437 Uniprot:P47787
Root	Isotig05949	0	46	-6.653	9.06E-13	UNIPROTKB|P47787 - symbol:TBXAS1 "Thromboxane-A synthase" species:9823 "Sus scrofa" [GO:0001516 "prostaglandin biosynthetic process" evidence=IEA] [GO:0004497 "monooxygenase activity" evidence=IEA] [GO:0004796 "thromboxane-A synthase activity" evidence=IEA] [GO:0005789 "endoplasmic reticulum membrane" evidence=IEA] [GO:0009055 "electron carrier activity" evidence=IEA] [GO:0016021 "integral to membrane" evidence=IEA] [GO:0016705 "oxidoreductase activity, acting on paired donors, with incorporation or reduction of molecular oxygen" evidence=IEA] [GO:0020037 "heme binding" evidence=IEA] InterPro:IPR001128 InterPro:IPR002401 InterPro:IPR017972 Pfam:PF00067 PRINTS:PR00385 PRINTS:PR00463 PROSITE:PS00086 GO:GO:0016021 GO:GO:0005789 GO:GO:0009055 GO:GO:0004497 GO:GO:0020037 Gene3D:G3DSA:1.10.630.10 SUPFAM:SSF48264 GO:GO:0016705 HOVERGEN:HBG108567 GO:GO:0001516 CTD:6916 KO:K01832 OrthoDB:EOG415GDB GO:GO:0004796 EMBL:L13128 RefSeq:NP_999211.1 UniGene:Ssc.158 ProteinModelPortal:P47787 STRING:P47787 GeneID:397112 KEGG:ssc:397112 eggNOG:NOG308437 Uniprot:P47787
Root	Isotig05988	0	77	-7.396	1.66E-19	TAIR|locus:2183730 - symbol:AT5G10770 "AT5G10770" species:3702 "Arabidopsis thaliana" [GO:0003677 "DNA binding" evidence=ISS] [GO:0004190 "aspartic-type endopeptidase activity" evidence=IEA] [GO:0006508 "proteolysis" evidence=IEA] InterPro:IPR001461 InterPro:IPR009007 Pfam:PF00026 PRINTS:PR00792 EMBL:CP002688 GO:GO:0006508 GO:GO:0004190 InterPro:IPR021109 SUPFAM:SSF50630 Gene3D:G3DSA:2.40.70.10 PANTHER:PTHR13683 HSSP:P00799 EMBL:AY075656 EMBL:BT001004 IPI:IPI00543783 RefSeq:NP_196638.2 UniGene:At.32336 ProteinModelPortal:Q8S9J6 MEROPS:A01.A15 PRIDE:Q8S9J6 EnsemblPlants:AT5G10770.1 GeneID:830944 KEGG:ath:AT5G10770 TAIR:At5g10770 InParanoid:Q8S9J6 OMA:LEVVYDG PhylomeDB:Q8S9J6 ProtClustDB:CLSN2686424 Genevestigator:Q8S9J6 Uniprot:Q8S9J6
Root	Isotig05989	0	78	-7.414	1.03E-19	TAIR|locus:2183730 - symbol:AT5G10770 "AT5G10770" species:3702 "Arabidopsis thaliana" [GO:0003677 "DNA binding" evidence=ISS] [GO:0004190 "aspartic-type endopeptidase activity" evidence=IEA] [GO:0006508 "proteolysis" evidence=IEA] InterPro:IPR001461 InterPro:IPR009007 Pfam:PF00026 PRINTS:PR00792 EMBL:CP002688 GO:GO:0006508 GO:GO:0004190 InterPro:IPR021109 SUPFAM:SSF50630 Gene3D:G3DSA:2.40.70.10 PANTHER:PTHR13683 HSSP:P00799 EMBL:AY075656 EMBL:BT001004 IPI:IPI00543783 RefSeq:NP_196638.2 UniGene:At.32336 ProteinModelPortal:Q8S9J6 MEROPS:A01.A15 PRIDE:Q8S9J6 EnsemblPlants:AT5G10770.1 GeneID:830944 KEGG:ath:AT5G10770 TAIR:At5g10770 InParanoid:Q8S9J6 OMA:LEVVYDG PhylomeDB:Q8S9J6 ProtClustDB:CLSN2686424 Genevestigator:Q8S9J6 Uniprot:Q8S9J6
Root	Isotig06010	0	28	-5.936	1.79E-08	TAIR|locus:2181950 - symbol:AT5G11650 "AT5G11650" species:3702 "Arabidopsis thaliana" [GO:0016787 "hydrolase activity" evidence=ISS] EMBL:CP002688 GO:GO:0016787 EMBL:AL163814 InterPro:IPR022742 Pfam:PF12146 EMBL:BT026426 EMBL:AK229263 IPI:IPI00517172 PIR:T48524 RefSeq:NP_196726.1 UniGene:At.32229 ProteinModelPortal:Q9LYG5 SMR:Q9LYG5 IntAct:Q9LYG5 STRING:Q9LYG5 MEROPS:S33.A11 PRIDE:Q9LYG5 ProMEX:Q9LYG5 EnsemblPlants:AT5G11650.1 GeneID:831037 KEGG:ath:AT5G11650 TAIR:At5g11650 InParanoid:Q9LYG5 OMA:RAWVERF PhylomeDB:Q9LYG5 ProtClustDB:PLN02652 Genevestigator:Q9LYG5 Uniprot:Q9LYG5
Root	Isotig06011	0	28	-5.936	1.79E-08	TAIR|locus:2181950 - symbol:AT5G11650 "AT5G11650" species:3702 "Arabidopsis thaliana" [GO:0016787 "hydrolase activity" evidence=ISS] EMBL:CP002688 GO:GO:0016787 EMBL:AL163814 InterPro:IPR022742 Pfam:PF12146 EMBL:BT026426 EMBL:AK229263 IPI:IPI00517172 PIR:T48524 RefSeq:NP_196726.1 UniGene:At.32229 ProteinModelPortal:Q9LYG5 SMR:Q9LYG5 IntAct:Q9LYG5 STRING:Q9LYG5 MEROPS:S33.A11 PRIDE:Q9LYG5 ProMEX:Q9LYG5 EnsemblPlants:AT5G11650.1 GeneID:831037 KEGG:ath:AT5G11650 TAIR:At5g11650 InParanoid:Q9LYG5 OMA:RAWVERF PhylomeDB:Q9LYG5 ProtClustDB:PLN02652 Genevestigator:Q9LYG5 Uniprot:Q9LYG5
Root	Isotig06048	3	17	-2.631	0.00056383	TAIR|locus:2061748 - symbol:IRX9 "AT2G37090" species:3702 "Arabidopsis thaliana" [GO:0016757 "transferase activity, transferring glycosyl groups" evidence=ISS] [GO:0009834 "secondary cell wall biogenesis" evidence=IMP] [GO:0010417 "glucuronoxylan biosynthetic process" evidence=IGI] [GO:0005794 "Golgi apparatus" evidence=IDA] [GO:0010413 "glucuronoxylan metabolic process" evidence=IMP] [GO:0042285 "xylosyltransferase activity" evidence=IMP] InterPro:IPR005027 Pfam:PF03360 GO:GO:0016021 EMBL:CP002685 GenomeReviews:CT485783_GR GO:GO:0000139 GO:GO:0007047 CAZy:GT43 GO:GO:0015018

Tissue	Identification	# of reads (stress)	# of reads (control)	Fold Change	p-value	Description
						PANTHER:PTHR10896 GO:GO:0009834 EMBL:AC006260 GO:GO:0010417 GO:GO:0042285 EMBL:BT015044 EMBL:BT015838 IPI:IPI00532147 PIR:D84788 RefSeq:NP_181246.1 UniGene:At.26964 UniGene:At.67134 ProteinModelPortal:Q9ZQC6 SMR:Q9ZQC6 STRING:Q9ZQC6 PRIDE:Q9ZQC6 EnsemblPlants:AT2G37090.1 GeneID:818285 KEGG:ath:AT2G37090 TAIR:At2g37090 eggNOG:NOG308705 HOGENOM:HBG600303 InParanoid:Q9ZQC6 OMA:FSLCFVM PhylomeDB:Q9ZQC6 ProtClustDB:PLN02458 ArrayExpress:Q9ZQC6 Genevestigator:Q9ZQC6 Uniprot:Q9ZQC6
Root	Isotig06049	3	17	-2.631	0.00056383	TAIR|locus:2061748 - symbol:IRX9 "AT2G37090" species:3702 "Arabidopsis thaliana" [GO:0016757 "transferase activity, transferring glycosyl groups" evidence=ISS] [GO:0009834 "secondary cell wall biogenesis" evidence=IMP] [GO:0010417 "glucuronoxylan biosynthetic process" evidence=IGI] [GO:0005794 "Golgi apparatus" evidence=IDA] [GO:0010413 "glucuronoxylan metabolic process" evidence=IMP] [GO:0042285 "xylosyltransferase activity" evidence=IMP] InterPro:IPR005027 Pfam:PF03360 GO:GO:0016021 EMBL:CP002685 GenomeReviews:CT485783_GR GO:GO:0000139 GO:GO:0007047 CAZy:GT43 GO:GO:0015018 PANTHER:PTHR10896 GO:GO:0009834 EMBL:AC006260 GO:GO:0010417 GO:GO:0042285 EMBL:BT015044 EMBL:BT015838 IPI:IPI00532147 PIR:D84788 RefSeq:NP_181246.1 UniGene:At.26964 UniGene:At.67134 ProteinModelPortal:Q9ZQC6 SMR:Q9ZQC6 STRING:Q9ZQC6 PRIDE:Q9ZQC6 EnsemblPlants:AT2G37090.1 GeneID:818285 KEGG:ath:AT2G37090 TAIR:At2g37090 eggNOG:NOG308705 HOGENOM:HBG600303 InParanoid:Q9ZQC6 OMA:FSLCFVM PhylomeDB:Q9ZQC6 ProtClustDB:PLN02458 ArrayExpress:Q9ZQC6 Genevestigator:Q9ZQC6 Uniprot:Q9ZQC6
Root	Isotig06054	0	15	-5.036	4.38E-05	TAIR|locus:2092950 - symbol:AT3G20980 "AT3G20980" species:3702 "Arabidopsis thaliana" [GO:0003674 "molecular_function" evidence=ND] [GO:0005575 "cellular_component" evidence=ND] [GO:0008150 "biological_process" evidence=ND] GenomeReviews:BA000014_GR EMBL:AP000604 eggNOG:KOG0017 IPI:IPI01020495 PRIDE:Q9LJD3 TAIR:At3g20980 InParanoid:Q9LJD3 PhylomeDB:Q9LJD3 Genevestigator:Q9LJD3 Uniprot:Q9LJD3
Root	Isotig06055	0	12	-4.714	0.000297064	TAIR|locus:2092950 - symbol:AT3G20980 "AT3G20980" species:3702 "Arabidopsis thaliana" [GO:0003674 "molecular_function" evidence=ND] [GO:0005575 "cellular_component" evidence=ND] [GO:0008150 "biological_process" evidence=ND] GenomeReviews:BA000014_GR EMBL:AP000604 eggNOG:KOG0017 IPI:IPI01020495 PRIDE:Q9LJD3 TAIR:At3g20980 InParanoid:Q9LJD3 PhylomeDB:Q9LJD3 Genevestigator:Q9LJD3 Uniprot:Q9LJD3
Root	Isotig06056	3	66	-4.588	3.16E-17	TAIR|locus:2150763 - symbol:AGAL2 "alpha-galactosidase 2" species:3702 "Arabidopsis thaliana" [GO:0003824 "catalytic activity" evidence=IEA] [GO:0004553 "hydrolase activity, hydrolyzing O-glycosyl compounds" evidence=IEA] [GO:0004557 "alpha-galactosidase activity" evidence=ISS;IBA] [GO:0005737 "cytoplasm" evidence=IBA] [GO:0009311 "oligosaccharide metabolic process" evidence=IBA] [GO:0016139 "glycoside catabolic process" evidence=IBA] [GO:0046477 "glycosylceramide catabolic process" evidence=IBA] [GO:0009505 "plant-type cell wall" evidence=IDA] [GO:0009911 "positive regulation of flower development" evidence=IMP] [GO:0009965 "leaf morphogenesis" evidence=IMP] InterPro:IPR000111 InterPro:IPR002241 InterPro:IPR013780 InterPro:IPR013785 Pfam:PF02065 PRINTS:PR00740 PROSITE:PS00512 EMBL:CP002688 GenomeReviews:BA000015_GR Gene3D:G3DSA:3.20.20.70 GO:GO:0043169 InterPro:IPR017853 SUPFAM:SSF51445 GO:GO:0005975 Gene3D:G3DSA:2.60.40.1180 GO:GO:0004553 GO:GO:0009505 GO:GO:0009911 CAZy:GH27 KO:K07407 HOGENOM:HBG424315 OMA:DMMEVGN GO:GO:0009965 UniGene:At.32587 UniGene:At.48997 EMBL:AY090238 EMBL:AY085529 EMBL:BT000619 IPI:IPI00547726 RefSeq:NP_568193.1 HSSP:Q9FXT4 ProteinModelPortal:Q8RX86 SMR:Q8RX86 PRIDE:Q8RX86 EnsemblPlants:AT5G08370.1 GeneID:830735 KEGG:ath:AT5G08370 TAIR:At5g08370 InParanoid:Q8RX86 PhylomeDB:Q8RX86 ProtClustDB:PLN02808 Genevestigator:Q8RX86 Uniprot:Q8RX86
Root	Isotig06057	3	70	-4.673	2.69E-18	TAIR|locus:2150763 - symbol:AGAL2 "alpha-galactosidase 2" species:3702 "Arabidopsis thaliana" [GO:0003824 "catalytic activity" evidence=IEA] [GO:0004553 "hydrolase activity, hydrolyzing O-glycosyl compounds" evidence=IEA] [GO:0004557 "alpha-galactosidase activity" evidence=ISS;IBA] [GO:0005737 "cytoplasm" evidence=IBA] [GO:0009311 "oligosaccharide metabolic process" evidence=IBA] [GO:0016139 "glycoside catabolic process" evidence=IBA] [GO:0046477 "glycosylceramide catabolic process" evidence=IBA] [GO:0009505 "plant-type cell wall" evidence=IDA] [GO:0009911 "positive regulation of flower development" evidence=IMP] [GO:0009965 "leaf morphogenesis" evidence=IMP] InterPro:IPR000111 InterPro:IPR002241 InterPro:IPR013780 InterPro:IPR013785 Pfam:PF02065 PRINTS:PR00740 PROSITE:PS00512 EMBL:CP002688 GenomeReviews:BA000015_GR Gene3D:G3DSA:3.20.20.70 GO:GO:0043169 InterPro:IPR017853 SUPFAM:SSF51445 GO:GO:0005975

Tissue	Identification	# of reads (stress)	# of reads (control)	Fold Change	p-value	Description
						Gene3D:G3DSA:2.60.40.1180 GO:GO:0004553 GO:GO:0009505 GO:GO:0009911 CAZy:GH27 KO:K07407 HOGENOM:HBG424315 OMA:DMMEVGN GO:GO:0009965 UniGene:At.32587 UniGene:At.48997 EMBL:AY090238 EMBL:AY085529 EMBL:BT000619 IPI:IPI00547726 RefSeq:NP_568193.1 HSSP:Q9FXT4 ProteinModelPortal:Q8RX86 SMR:Q8RX86 PRIDE:Q8RX86 EnsemblPlants:AT5G08370.1 GeneID:830735 KEGG:ath:AT5G08370 TAIR:At5g08370 InParanoid:Q8RX86 PhylomeDB:Q8RX86 ProtClustDB:PLN02808 Genevestigator:Q8RX86 Uniprot:Q8RX86
Root	Isotig06064	17	163	-3.390	1.66E-33	TAIR|locus:2084031 - symbol:PIP2A "AT3G53420" species:3702 "Arabidopsis thaliana" [GO:0006810 "transport" evidence=ISS] [GO:0015250 "water channel activity" evidence=ISS;IDA] [GO:0016020 "membrane" evidence=ISS;IDA] [GO:0009507 "chloroplast" evidence=IDA] [GO:0005886 "plasma membrane" evidence=IDA;TAS] [GO:0005773 "vacuole" evidence=IDA] [GO:0009414 "response to water deprivation" evidence=IEP] [GO:0080170 "hydrogen peroxide transmembrane transport" evidence=IDA] [GO:0031625 "ubiquitin protein ligase binding" evidence=IPI] [GO:0009737 "response to abscisic acid stimulus" evidence=IDA] [GO:0009506 "plasmodesma" evidence=IDA] [GO:0006833 "water transport" evidence=IDA] InterPro:IPR000425 InterPro:IPR012269 InterPro:IPR022357 Pfam:PF00230 PRINTS:PR00783 PROSITE:PS00221 GO:GO:0016021 GO:GO:0005886 GO:GO:0009506 GO:GO:0009737 GO:GO:0005773 GO:GO:0009507 EMBL:CP002686 GenomeReviews:BA000014_GR EMBL:AL132966 GO:GO:0009414 GO:GO:0031625 InterPro:IPR023271 Gene3D:G3DSA:1.20.1080.10 SUPFAM:SSF81338 eggNOG:COG0580 HOGENOM:HBG705794 PANTHER:PTHR19139 TIGRFAMs:TIGR00861 GO:GO:0015250 GO:GO:0080170 KO:K09872 EMBL:X75883 EMBL:AY039579 EMBL:AY044327 EMBL:AY056085 EMBL:AF428426 EMBL:AY072374 EMBL:AY087854 IPI:IPI00532482 PIR:S44084 RefSeq:NP_001030851.1 RefSeq:NP_190910.1 UniGene:At.47609 ProteinModelPortal:P43286 SMR:P43286 MINT:MINT-6951259 STRING:P43286 TCDB:1.A.8.11.4 PRIDE:P43286 ProMEX:P43286 EnsemblPlants:AT3G53420.1 EnsemblPlants:AT3G53420.2 GeneID:824510 KEGG:ath:AT3G53420 GeneFarm:4866 TAIR:At3g53420 InParanoid:P43286 OMA:VFMAITI PhylomeDB:P43286 ProtClustDB:CLSN2683965 ArrayExpress:P43286 Genevestigator:P43286 GermOnline:AT3G53420 Uniprot:P43286
Root	Isotig06065	17	163	-3.390	1.66E-33	TAIR|locus:2084031 - symbol:PIP2A "AT3G53420" species:3702 "Arabidopsis thaliana" [GO:0006810 "transport" evidence=ISS] [GO:0015250 "water channel activity" evidence=ISS;IDA] [GO:0016020 "membrane" evidence=ISS;IDA] [GO:0009507 "chloroplast" evidence=IDA] [GO:0005886 "plasma membrane" evidence=IDA;TAS] [GO:0005773 "vacuole" evidence=IDA] [GO:0009414 "response to water deprivation" evidence=IEP] [GO:0080170 "hydrogen peroxide transmembrane transport" evidence=IDA] [GO:0031625 "ubiquitin protein ligase binding" evidence=IPI] [GO:0009737 "response to abscisic acid stimulus" evidence=IDA] [GO:0009506 "plasmodesma" evidence=IDA] [GO:0006833 "water transport" evidence=IDA] InterPro:IPR000425 InterPro:IPR012269 InterPro:IPR022357 Pfam:PF00230 PRINTS:PR00783 PROSITE:PS00221 GO:GO:0016021 GO:GO:0005886 GO:GO:0009506 GO:GO:0009737 GO:GO:0005773 GO:GO:0009507 EMBL:CP002686 GenomeReviews:BA000014_GR EMBL:AL132966 GO:GO:0009414 GO:GO:0031625 InterPro:IPR023271 Gene3D:G3DSA:1.20.1080.10 SUPFAM:SSF81338 eggNOG:COG0580 HOGENOM:HBG705794 PANTHER:PTHR19139 TIGRFAMs:TIGR00861 GO:GO:0015250 GO:GO:0080170 KO:K09872 EMBL:X75883 EMBL:AY039579 EMBL:AY044327 EMBL:AY056085 EMBL:AF428426 EMBL:AY072374 EMBL:AY087854 IPI:IPI00532482 PIR:S44084 RefSeq:NP_001030851.1 RefSeq:NP_190910.1 UniGene:At.47609 ProteinModelPortal:P43286 SMR:P43286 MINT:MINT-6951259 STRING:P43286 TCDB:1.A.8.11.4 PRIDE:P43286 ProMEX:P43286 EnsemblPlants:AT3G53420.1 EnsemblPlants:AT3G53420.2 GeneID:824510 KEGG:ath:AT3G53420 GeneFarm:4866 TAIR:At3g53420 InParanoid:P43286 OMA:VFMAITI PhylomeDB:P43286 ProtClustDB:CLSN2683965 ArrayExpress:P43286 Genevestigator:P43286 GermOnline:AT3G53420 Uniprot:P43286
Root	Isotig06082	15	0	4.778	0.000105609	TAIR|locus:2158544 - symbol:AT5G63350 "AT5G63350" species:3702 "Arabidopsis thaliana" [GO:0008150 "biological_process" evidence=ND] [GO:0005515 "protein binding" evidence=IPI] EMBL:CP002688 GenomeReviews:BA000015_GR GO:GO:0005515 EMBL:AB023035 EMBL:BT002821 EMBL:BT004383 IPI:IPI00523793 RefSeq:NP_201140.1 UniGene:At.29001 IntAct:Q9FGW7 PRIDE:Q9FGW7 EnsemblPlants:AT5G63350.1 GeneID:836455 KEGG:ath:AT5G63350 TAIR:At5g63350 eggNOG:NOG314236 HOGENOM:HBG745108 InParanoid:Q9FGW7 OMA:DINIPME PhylomeDB:Q9FGW7 ProtClustDB:CLSN2687559 Genevestigator:Q9FGW7 Uniprot:Q9FGW7

Tissue	Identification	# of reads (stress)	# of reads (control)	Fold Change	p-value	Description
Root	Isotig06083	15	0	4.778	0.000105609	TAIR|locus:2158544 - symbol:AT5G63350 "AT5G63350" species:3702 "Arabidopsis thaliana" [GO:0008150 "biological_process" evidence=ND] [GO:0005515 "protein binding" evidence=IPI] EMBL:CP002688 GenomeReviews:BA000015_GR GO:GO:0005515 EMBL:AB023035 EMBL:BT002821 EMBL:BT004383 IPI:IPI00523793 RefSeq:NP_201140.1 UniGene:At.29001 IntAct:Q9FGW7 PRIDE:Q9FGW7 EnsemblPlants:AT5G63350.1 GeneID:836455 KEGG:ath:AT5G63350 TAIR:At5g63350 eggNOG:NOG314236 HOGENOM:HBG745108 InParanoid:Q9FGW7 OMA:DINIPME PhylomeDB:Q9FGW7 ProtClustDB:CLSN2687559 Genevestigator:Q9FGW7 Uniprot:Q9FGW7
Root	Isotig06108	14	1	3.678	0.000484579	TAIR|locus:2055155 - symbol:AT2G14520 "AT2G14520" species:3702 "Arabidopsis thaliana" [GO:0003674 "molecular_function" evidence=ND] [GO:0008150 "biological_process" evidence=ND] InterPro:IPR000644 Pfam:PF00571 PROSITE:PS51371 GO:GO:0016021 EMBL:CP002685 GenomeReviews:CT485783_GR eggNOG:COG1253 InterPro:IPR002550 Pfam:PF01595 EMBL:AC006067 HOGENOM:HBG317049 EMBL:AY080819 IPI:IPI01019762 PIR:B84518 RefSeq:NP_179058.3 UniGene:At.40534 ProteinModelPortal:Q9ZQR4 SMR:Q9ZQR4 PRIDE:Q9ZQR4 GeneID:815939 KEGG:ath:AT2G14520 TAIR:At2g14520 InParanoid:Q9ZQR4 PhylomeDB:Q9ZQR4 ArrayExpress:Q9ZQR4 Genevestigator:Q9ZQR4 Uniprot:Q9ZQR4
Root	Isotig06109	14	1	3.678	0.000484579	TAIR|locus:2055155 - symbol:AT2G14520 "AT2G14520" species:3702 "Arabidopsis thaliana" [GO:0003674 "molecular_function" evidence=ND] [GO:0008150 "biological_process" evidence=ND] InterPro:IPR000644 Pfam:PF00571 PROSITE:PS51371 GO:GO:0016021 EMBL:CP002685 GenomeReviews:CT485783_GR eggNOG:COG1253 InterPro:IPR002550 Pfam:PF01595 EMBL:AC006067 HOGENOM:HBG317049 EMBL:AY080819 IPI:IPI01019762 PIR:B84518 RefSeq:NP_179058.3 UniGene:At.40534 ProteinModelPortal:Q9ZQR4 SMR:Q9ZQR4 PRIDE:Q9ZQR4 GeneID:815939 KEGG:ath:AT2G14520 TAIR:At2g14520 InParanoid:Q9ZQR4 PhylomeDB:Q9ZQR4 ArrayExpress:Q9ZQR4 Genevestigator:Q9ZQR4 Uniprot:Q9ZQR4
Root	Isotig06112	0	18	-5.299	6.77E-06	TAIR|locus:2183229 - symbol:AT5G19740 species:3702 "Arabidopsis thaliana" [GO:0006508 "proteolysis" evidence=IEA;ISS] [GO:0016805 "dipeptidase activity" evidence=ISS] [GO:0005773 "vacuole" evidence=IDA] InterPro:IPR007484 Pfam:PF04389 InterPro:IPR003137 Pfam:PF02225 GO:GO:0005773 EMBL:CP002688 GenomeReviews:BA000015_GR GO:GO:0006508 GO:GO:0008233 eggNOG:NOG74799 HOGENOM:HBG713441 InterPro:IPR007365 Gene3D:G3DSA:1.20.930.40 Pfam:PF04253 SUPFAM:SSF47672 EMBL:BT008323 IPI:IPI00537808 RefSeq:NP_197475.2 UniGene:At.31257 HSSP:P80561 ProteinModelPortal:Q7Y228 SMR:Q7Y228 MEROPS:M28.A02 PRIDE:Q7Y228 EnsemblPlants:AT5G19740.1 GeneID:832094 KEGG:ath:AT5G19740 TAIR:At5g19740 InParanoid:Q7Y228 OMA:FLQHLGI PhylomeDB:Q7Y228 ProtClustDB:CLSN2918628 Genevestigator:Q7Y228 Uniprot:Q7Y228
Root	Isotig06113	0	18	-5.299	6.77E-06	TAIR|locus:2183229 - symbol:AT5G19740 species:3702 "Arabidopsis thaliana" [GO:0006508 "proteolysis" evidence=IEA;ISS] [GO:0016805 "dipeptidase activity" evidence=ISS] [GO:0005773 "vacuole" evidence=IDA] InterPro:IPR007484 Pfam:PF04389 InterPro:IPR003137 Pfam:PF02225 GO:GO:0005773 EMBL:CP002688 GenomeReviews:BA000015_GR GO:GO:0006508 GO:GO:0008233 eggNOG:NOG74799 HOGENOM:HBG713441 InterPro:IPR007365 Gene3D:G3DSA:1.20.930.40 Pfam:PF04253 SUPFAM:SSF47672 EMBL:BT008323 IPI:IPI00537808 RefSeq:NP_197475.2 UniGene:At.31257 HSSP:P80561 ProteinModelPortal:Q7Y228 SMR:Q7Y228 MEROPS:M28.A02 PRIDE:Q7Y228 EnsemblPlants:AT5G19740.1 GeneID:832094 KEGG:ath:AT5G19740 TAIR:At5g19740 InParanoid:Q7Y228 OMA:FLQHLGI PhylomeDB:Q7Y228 ProtClustDB:CLSN2918628 Genevestigator:Q7Y228 Uniprot:Q7Y228
Root	Isotig06144	18	1	4.041	4.47E-05	TAIR|locus:2054809 - symbol:AT2G20560 species:3702 "Arabidopsis thaliana" [GO:0006457 "protein folding" evidence=IEA;ISS] [GO:0031072 "heat shock protein binding" evidence=IEA] [GO:0051082 "unfolded protein binding" evidence=IEA] InterPro:IPR001623 InterPro:IPR002939 InterPro:IPR003095 InterPro:IPR008971 InterPro:IPR018253 Pfam:PF00226 Pfam:PF01556 PRINTS:PR00625 PROSITE:PS00636 PROSITE:PS50076 SMART:SM00271 EMBL:CP002685 GenomeReviews:CT485783_GR GO:GO:0006457 GO:GO:0006950 GO:GO:0051082 eggNOG:COG0484 HOGENOM:HBG635315 GO:GO:0031072 Gene3D:G3DSA:1.10.287.110 SUPFAM:SSF46565 SUPFAM:SSF49493 HSSP:P25685 EMBL:AC007109 ProtClustDB:CLSN2683325 EMBL:AY057555 EMBL:AY086509 EMBL:AY133644 IPI:IPI00547861 PIR:G84590 RefSeq:NP_179646.1 UniGene:At.26315 ProteinModelPortal:Q9SIL3 SMR:Q9SIL3 STRING:Q9SIL3 PRIDE:Q9SIL3 EnsemblPlants:AT2G20560.1 GeneID:816578 KEGG:ath:AT2G20560 TAIR:At2g20560 InParanoid:Q9SIL3 OMA:TKKMRIS PhylomeDB:Q9SIL3 ArrayExpress:Q9SIL3 Genevestigator:Q9SIL3 Uniprot:Q9SIL3

Tissue	Identification	# of reads (stress)	# of reads (control)	Fold Change	p-value	Description
Root	Isotig06145	18	1	4.041	4.47E-05	TAIR|locus:2097638 - symbol:AT3G08910 "AT3G08910" species:3702 "Arabidopsis thaliana" [GO:0005575 "cellular_component" evidence=ND] [GO:0006457 "protein folding" evidence=IEA;ISS] [GO:0031072 "heat shock protein binding" evidence=IEA] [GO:0051082 "unfolded protein binding" evidence=IEA] InterPro:IPR001623 InterPro:IPR002939 InterPro:IPR003095 InterPro:IPR008971 InterPro:IPR018253 Pfam:PF00226 Pfam:PF01556 PRINTS:PR00625 PROSITE:PS00636 PROSITE:PS50076 SMART:SM00271 EMBL:CP002686 GO:GO:0006457 GO:GO:0006950 GO:GO:0051082 EMBL:AC010871 GO:GO:0031072 Gene3D:G3DSA:1.10.287.110 SUPFAM:SSF46565 SUPFAM:SSF49493 HSSP:P25685 EMBL:BT044615 IPI:IPI00530981 RefSeq:NP_187503.1 UniGene:At.40199 ProteinModelPortal:Q9SR91 SMR:Q9SR91 PRIDE:Q9SR91 EnsemblPlants:AT3G08910.1 GeneID:820040 KEGG:ath:AT3G08910 TAIR:At3g08910 InParanoid:Q9SR91 OMA:FRFNGRS PhylomeDB:Q9SR91 ProtClustDB:CLSN2915586 Genevestigator:Q9SR91 Uniprot:Q9SR91
Root	Isotig06176	7	57	-3.155	4.72E-12	TAIR|locus:2122829 - symbol:NIP5;1 "AT4G10380" species:3702 "Arabidopsis thaliana" [GO:0006810 "transport" evidence=ISS] [GO:0015250 "water channel activity" evidence=ISS;IDA] [GO:0016020 "membrane" evidence=ISS] [GO:0005886 "plasma membrane" evidence=IDA] [GO:0010036 "response to boron-containing substance" evidence=IEP;IMP] [GO:0046713 "borate transport" evidence=IDA] [GO:0046715 "borate transmembrane transporter activity" evidence=IDA] [GO:0080138 "borate uptake transmembrane transporter activity" evidence=IDA] [GO:0080029 "cellular response to boron-containing substance levels" evidence=IEP] [GO:0015105 "arsenite transmembrane transporter activity" evidence=IDA] [GO:0015700 "arsenite transport" evidence=IDA] [GO:0046685 "response to arsenic-containing substance" evidence=IMP] [GO:0016328 "lateral plasma membrane" evidence=IDA] InterPro:IPR000425 InterPro:IPR022357 Pfam:PF00230 PRINTS:PR00783 PROSITE:PS00221 GO:GO:0016021 EMBL:CP002687 GenomeReviews:CT486007_GR GO:GO:0046685 GO:GO:0016328 InterPro:IPR023271 Gene3D:G3DSA:1.20.1080.10 SUPFAM:SSF81338 eggNOG:COG0580 HOGENOM:HBG705794 PANTHER:PTHR19139 GO:GO:0015250 GO:GO:0015105 GO:GO:0046715 GO:GO:0080029 EMBL:AL049488 EMBL:AL161517 KO:K09874 EMBL:AY087560 IPI:IPI00536883 PIR:T04053 RefSeq:NP_192776.1 UniGene:At.33633 ProteinModelPortal:Q9SV84 SMR:Q9SV84 TCDB:1.A.8.12.3 EnsemblPlants:AT4G10380.1 GeneID:826630 KEGG:ath:AT4G10380 GeneFarm:4878 TAIR:At4g10380 InParanoid:Q9SV84 OMA:PRCKCLP PhylomeDB:Q9SV84 ProtClustDB:PLN00026 ArrayExpress:Q9SV84 Genevestigator:Q9SV84 GermOnline:AT4G10380 GO:GO:0080138 Uniprot:Q9SV84
Root	Isotig06177	7	57	-3.155	4.72E-12	TAIR|locus:2122829 - symbol:NIP5;1 "AT4G10380" species:3702 "Arabidopsis thaliana" [GO:0006810 "transport" evidence=ISS] [GO:0015250 "water channel activity" evidence=ISS;IDA] [GO:0016020 "membrane" evidence=ISS] [GO:0005886 "plasma membrane" evidence=IDA] [GO:0010036 "response to boron-containing substance" evidence=IEP;IMP] [GO:0046713 "borate transport" evidence=IDA] [GO:0046715 "borate transmembrane transporter activity" evidence=IDA] [GO:0080138 "borate uptake transmembrane transporter activity" evidence=IDA] [GO:0080029 "cellular response to boron-containing substance levels" evidence=IEP] [GO:0015105 "arsenite transmembrane transporter activity" evidence=IDA] [GO:0015700 "arsenite transport" evidence=IDA] [GO:0046685 "response to arsenic-containing substance" evidence=IMP] [GO:0016328 "lateral plasma membrane" evidence=IDA] InterPro:IPR000425 InterPro:IPR022357 Pfam:PF00230 PRINTS:PR00783 PROSITE:PS00221 GO:GO:0016021 EMBL:CP002687 GenomeReviews:CT486007_GR GO:GO:0046685 GO:GO:0016328 InterPro:IPR023271 Gene3D:G3DSA:1.20.1080.10 SUPFAM:SSF81338 eggNOG:COG0580 HOGENOM:HBG705794 PANTHER:PTHR19139 GO:GO:0015250 GO:GO:0015105 GO:GO:0046715 GO:GO:0080029 EMBL:AL049488 EMBL:AL161517 KO:K09874 EMBL:AY087560 IPI:IPI00536883 PIR:T04053 RefSeq:NP_192776.1 UniGene:At.33633 ProteinModelPortal:Q9SV84 SMR:Q9SV84 TCDB:1.A.8.12.3 EnsemblPlants:AT4G10380.1 GeneID:826630 KEGG:ath:AT4G10380 GeneFarm:4878 TAIR:At4g10380 InParanoid:Q9SV84 OMA:PRCKCLP PhylomeDB:Q9SV84 ProtClustDB:PLN00026 ArrayExpress:Q9SV84 Genevestigator:Q9SV84 GermOnline:AT4G10380 GO:GO:0080138 Uniprot:Q9SV84
Root	Isotig06181	0	11	-4.588	0.000569522	TAIR|locus:2207210 - symbol:RCI3 "AT1G05260" species:3702 "Arabidopsis thaliana" [GO:0004601 "peroxidase activity" evidence=ISS;IDA] [GO:0005783 "endoplasmic reticulum" evidence=ISS] [GO:0009269 "response to desiccation" evidence=IMP] [GO:0009409 "response to cold" evidence=IEP] [GO:0042538 "hyperosmotic salinity response" evidence=IMP] InterPro:IPR000823 InterPro:IPR002016 InterPro:IPR010255 InterPro:IPR019793 InterPro:IPR019794 Pfam:PF00141 PRINTS:PR00458 PRINTS:PR00461 PROSITE:PS00435 PROSITE:PS00436 PROSITE:PS50873 EMBL:CP002684 GenomeReviews:CT485782_GR GO:GO:0005576 GO:GO:0009409 GO:GO:0020037 GO:GO:0004601 GO:GO:0009269 GO:GO:0042744 HOGENOM:HBG597790 SUPFAM:SSF48113 GO:GO:0042538 EMBL:AC000098 KO:K00430 eggNOG:NOG242082

Tissue	Identification	# of reads (stress)	# of reads (control)	Fold Change	p-value	Description
						ProtClustDB:CLSN2681995 EMBL:U97684 EMBL:BT004817 EMBL:AY084678 IPI:IPI00521040 PIR:B86187 RefSeq:NP_172018.1 UniGene:At.23986 ProteinModelPortal:O23044 SMR:O23044 IntAct:O23044 STRING:O23044 PeroxiBase:79 PRIDE:O23044 EnsemblPlants:AT1G05260.1 GeneID:837028 KEGG:ath:AT1G05260 GeneFarm:1833 TAIR:At1g05260 InParanoid:O23044 OMA:ANIPPPT PhylomeDB:O23044 ArrayExpress:O23044 Genevestigator:O23044 GermOnline:AT1G05260 Uniprot:O23044
Root	Isotig06182	0	11	-4.588	0.000569522	TAIR|locus:2128308 - symbol:AT4G11290 species:3702 "Arabidopsis thaliana" [GO:0004601 "peroxidase activity" evidence=IEA;ISS] [GO:0006979 "response to oxidative stress" evidence=IEA] [GO:0020037 "heme binding" evidence=IEA] [GO:0055114 "oxidation-reduction process" evidence=IEA] InterPro:IPR000823 InterPro:IPR002016 InterPro:IPR010255 InterPro:IPR019793 InterPro:IPR019794 Pfam:PF00141 PRINTS:PR00458 PRINTS:PR00461 PROSITE:PS00435 PROSITE:PS00436 PROSITE:PS50873 EMBL:CP002687 GenomeReviews:CT486007_GR GO:GO:0005576 EMBL:AL096882 EMBL:AL161531 GO:GO:0020037 GO:GO:0004601 GO:GO:0042744 HOGENOM:HBG597790 SUPFAM:SSF48113 KO:K00430 eggNOG:NOG242082 ProtClustDB:CLSN2681995 EMBL:X98805 IPI:IPI00531288 PIR:T13020 RefSeq:NP_192868.1 UniGene:At.22642 ProteinModelPortal:Q9SUT2 SMR:Q9SUT2 STRING:Q9SUT2 PeroxiBase:205 PRIDE:Q9SUT2 EnsemblPlants:AT4G11290.1 GeneID:826731 KEGG:ath:AT4G11290 GeneFarm:1868 TAIR:At4g11290 InParanoid:Q9SUT2 OMA:QLKMGFY PhylomeDB:Q9SUT2 ArrayExpress:Q9SUT2 Genevestigator:Q9SUT2 GermOnline:AT4G11290 Uniprot:Q9SUT2
Root	Isotig06236	0	19	-5.377	3.67E-06	TAIR|locus:2040939 - symbol:CYP710A1 "AT2G34500" species:3702 "Arabidopsis thaliana" [GO:0019825 "oxygen binding" evidence=ISS] [GO:0055114 "oxidation-reduction process" evidence=IEA] [GO:0000249 "C-22 sterol desaturase activity" evidence=IDA] InterPro:IPR001128 InterPro:IPR002401 InterPro:IPR017972 Pfam:PF00067 PRINTS:PR00385 PRINTS:PR00463 PROSITE:PS00086 GO:GO:0016021 EMBL:CP002685 GenomeReviews:CT485783_GR GO:GO:0009055 GO:GO:0004497 GO:GO:0016126 eggNOG:COG2124 GO:GO:0020037 Gene3D:G3DSA:1.10.630.10 SUPFAM:SSF48264 HOGENOM:HBG592092 OMA:CLGQTYA EMBL:AB219423 EMBL:AC004077 EMBL:AC004481 EMBL:BT002069 EMBL:BT010554 IPI:IPI00537608 PIR:T02337 RefSeq:NP_180997.1 UniGene:At.27806 UniGene:At.67747 ProteinModelPortal:O64697 SMR:O64697 STRING:O64697 PRIDE:O64697 GeneID:818013 KEGG:ath:AT2G34500 GeneFarm:1168 TAIR:At2g34500 InParanoid:O64697 KO:K09832 PhylomeDB:O64697 ProtClustDB:CLSN2683644 BioCyc:ARA:AT2G34500-MONOMER BioCyc:MetaCyc:AT2G34500-MONOMER ArrayExpress:O64697 Genevestigator:O64697 GO:GO:0000249 Uniprot:O64697
Root	Isotig06237	0	19	-5.377	3.67E-06	TAIR|locus:2040939 - symbol:CYP710A1 "AT2G34500" species:3702 "Arabidopsis thaliana" [GO:0019825 "oxygen binding" evidence=ISS] [GO:0055114 "oxidation-reduction process" evidence=IEA] [GO:0000249 "C-22 sterol desaturase activity" evidence=IDA] InterPro:IPR001128 InterPro:IPR002401 InterPro:IPR017972 Pfam:PF00067 PRINTS:PR00385 PRINTS:PR00463 PROSITE:PS00086 GO:GO:0016021 EMBL:CP002685 GenomeReviews:CT485783_GR GO:GO:0009055 GO:GO:0004497 GO:GO:0016126 eggNOG:COG2124 GO:GO:0020037 Gene3D:G3DSA:1.10.630.10 SUPFAM:SSF48264 HOGENOM:HBG592092 OMA:CLGQTYA EMBL:AB219423 EMBL:AC004077 EMBL:AC004481 EMBL:BT002069 EMBL:BT010554 IPI:IPI00537608 PIR:T02337 RefSeq:NP_180997.1 UniGene:At.27806 UniGene:At.67747 ProteinModelPortal:O64697 SMR:O64697 STRING:O64697 PRIDE:O64697 GeneID:818013 KEGG:ath:AT2G34500 GeneFarm:1168 TAIR:At2g34500 InParanoid:O64697 KO:K09832 PhylomeDB:O64697 ProtClustDB:CLSN2683644 BioCyc:ARA:AT2G34500-MONOMER BioCyc:MetaCyc:AT2G34500-MONOMER ArrayExpress:O64697 Genevestigator:O64697 GO:GO:0000249 Uniprot:O64697
Root	Isotig06238	2	23	-3.653	3.09E-06	TAIR|locus:2132303 - symbol:UNE12 "AT4G02590" species:3702 "Arabidopsis thaliana" [GO:0003677 "DNA binding" evidence=ISS] [GO:0005634 "nucleus" evidence=IEA] [GO:0003700 "sequence-specific DNA binding transcription factor activity" evidence=ISS] [GO:0006355 "regulation of transcription, DNA-dependent" evidence=TAS] [GO:0009567 "double fertilization forming a zygote and endosperm" evidence=IMP] [GO:0031347 "regulation of defense response" evidence=IMP] InterPro:IPR011598 Pfam:PF00010 GO:GO:0007275 EMBL:CP002687 GenomeReviews:CT486007_GR GO:GO:0005634 GO:GO:0006355 GO:GO:0003677 GO:GO:0006351 Gene3D:G3DSA:4.10.280.10 SMART:SM00353 SUPFAM:SSF47459 PROSITE:PS50888 HOGENOM:HBG602703 ProtClustDB:CLSN2687635 EMBL:AC002330 EMBL:AL161494 GO:GO:0031347 GO:GO:0009567 EMBL:AF488592 EMBL:AF367328 EMBL:AY143951 EMBL:AY088218 IPI:IPI00545077 IPI:IPI00892352 PIR:T01090 RefSeq:NP_001031577.1 RefSeq:NP_001118919.1 RefSeq:NP_567245.1 UniGene:At.20028 ProteinModelPortal:O22768 SMR:O22768 IntAct:O22768 PRIDE:O22768 EnsemblPlants:AT4G02590.1 EnsemblPlants:AT4G02590.2 GeneID:828126 KEGG:ath:AT4G02590

Tissue	Identification	# of reads (stress)	# of reads (control)	Fold Change	p-value	Description
						TAIR:At4g02590 eggNOG:euNOG02041 InParanoid:O22768 OMA:FHGQPMQ PhylomeDB:O22768 ArrayExpress:O22768 Genevestigator:O22768 Uniprot:O22768
Root	Isotig06239	2	21	-3.521	1.10E-05	TAIR|locus:2132303 - symbol:UNE12 "AT4G02590" species:3702 "Arabidopsis thaliana" [GO:0003677 "DNA binding" evidence=ISS] [GO:0005634 "nucleus" evidence=IEA] [GO:0003700 "sequence-specific DNA binding transcription factor activity" evidence=ISS] [GO:0006355 "regulation of transcription, DNA-dependent" evidence=TAS] [GO:0009567 "double fertilization forming a zygote and endosperm" evidence=IMP] [GO:0031347 "regulation of defense response" evidence=IMP] InterPro:IPR011598 Pfam:PF00010 GO:GO:0007275 EMBL:CP002687 GenomeReviews:CT486007_GR GO:GO:0005634 GO:GO:0006355 GO:GO:0003677 GO:GO:0006351 Gene3D:G3DSA:4.10.280.10 SMART:SM00353 SUPFAM:SSF47459 PROSITE:PS50888 HOGENOM:HBG602703 ProtClustDB:CLSN2687635 EMBL:AC002330 EMBL:AL161494 GO:GO:0031347 GO:GO:0009567 EMBL:AF488592 EMBL:AF367328 EMBL:AY143951 EMBL:AY088218 IPI:IPI00545077 IPI:IPI00892352 PIR:T01090 RefSeq:NP_001031577.1 RefSeq:NP_001118919.1 RefSeq:NP_567245.1 UniGene:At.20028 ProteinModelPortal:O22768 SMR:O22768 IntAct:O22768 PRIDE:O22768 EnsemblPlants:AT4G02590.1 EnsemblPlants:AT4G02590.2 GeneID:828126 KEGG:ath:AT4G02590 TAIR:At4g02590 eggNOG:euNOG02041 InParanoid:O22768 OMA:FHGQPMQ PhylomeDB:O22768 ArrayExpress:O22768 Genevestigator:O22768 Uniprot:O22768
Root	Isotig06240	112	45	1.187	9.44E-07	TAIR|locus:2149194 - symbol:AT5G20290 "AT5G20290" species:3702 "Arabidopsis thaliana" [GO:0000462 "maturation of SSU-rRNA from tricistronic rRNA transcript (SSU-rRNA, 5.8S rRNA, LSU-rRNA)" evidence=IBA] [GO:0003735 "structural constituent of ribosome" evidence=ISS;IBA] [GO:0005840 "ribosome" evidence=ISS] [GO:0006412 "translation" evidence=ISS] [GO:0006414 "translational elongation" evidence=IBA] [GO:0042254 "ribosome biogenesis" evidence=ISS] [GO:0022627 "cytosolic small ribosomal subunit" evidence=ISS;IDA] [GO:0009507 "chloroplast" evidence=IDA] [GO:0005730 "nucleolus" evidence=IDA] [GO:0022626 "cytosolic ribosome" evidence=IDA] [GO:0005618 "cell wall" evidence=IDA] [GO:0005886 "plasma membrane" evidence=IDA] [GO:0016020 "membrane" evidence=IDA] [GO:0005829 "cytosol" evidence=IDA] [GO:0009506 "plasmodesma" evidence=IDA] [GO:0005794 "Golgi apparatus" evidence=RCA] InterPro:IPR001047 InterPro:IPR018283 PROSITE:PS01193 Pfam:PF01201 PANTHER:PTHR10394 GO:GO:0005886 GO:GO:0009506 EMBL:CP002688 GenomeReviews:BA000015_GR GO:GO:0005618 GO:GO:0009507 GO:GO:0005730 GO:GO:0006412 GO:GO:0003735 EMBL:AF296825 GO:GO:0022627 eggNOG:COG2007 InterPro:IPR022309 UniGene:At.24020 EMBL:AY050937 EMBL:AY052338 EMBL:AY061909 EMBL:AY091172 EMBL:AK226280 EMBL:AY086963 IPI:IPI00524577 RefSeq:NP_197529.1 UniGene:At.24724 ProteinModelPortal:Q93VG5 SMR:Q93VG5 IntAct:Q93VG5 STRING:Q93VG5 PRIDE:Q93VG5 EnsemblPlants:AT5G20290.1 GeneID:832151 KEGG:ath:AT5G20290 TAIR:At5g20290 HOGENOM:HBG745393 InParanoid:Q93VG5 KO:K02995 OMA:AWASEGC PhylomeDB:Q93VG5 ProtClustDB:CLSN2687206 ArrayExpress:Q93VG5 Genevestigator:Q93VG5 GermOnline:AT5G20290 TIGRFAMs:TIGR00307 Uniprot:Q93VG5
Root	Isotig06241	112	45	1.187	9.44E-07	TAIR|locus:2149194 - symbol:AT5G20290 "AT5G20290" species:3702 "Arabidopsis thaliana" [GO:0000462 "maturation of SSU-rRNA from tricistronic rRNA transcript (SSU-rRNA, 5.8S rRNA, LSU-rRNA)" evidence=IBA] [GO:0003735 "structural constituent of ribosome" evidence=ISS;IBA] [GO:0005840 "ribosome" evidence=ISS] [GO:0006412 "translation" evidence=ISS] [GO:0006414 "translational elongation" evidence=IBA] [GO:0042254 "ribosome biogenesis" evidence=ISS] [GO:0022627 "cytosolic small ribosomal subunit" evidence=ISS;IDA] [GO:0009507 "chloroplast" evidence=IDA] [GO:0005730 "nucleolus" evidence=IDA] [GO:0022626 "cytosolic ribosome" evidence=IDA] [GO:0005618 "cell wall" evidence=IDA] [GO:0005886 "plasma membrane" evidence=IDA] [GO:0016020 "membrane" evidence=IDA] [GO:0005829 "cytosol" evidence=IDA] [GO:0009506 "plasmodesma" evidence=IDA] [GO:0005794 "Golgi apparatus" evidence=RCA] InterPro:IPR001047 InterPro:IPR018283 PROSITE:PS01193 Pfam:PF01201 PANTHER:PTHR10394 GO:GO:0005886 GO:GO:0009506 EMBL:CP002688 GenomeReviews:BA000015_GR GO:GO:0005618 GO:GO:0009507 GO:GO:0005730 GO:GO:0006412 GO:GO:0003735 EMBL:AF296825 GO:GO:0022627 eggNOG:COG2007 InterPro:IPR022309 UniGene:At.24020 EMBL:AY050937 EMBL:AY052338 EMBL:AY061909 EMBL:AY091172 EMBL:AK226280 EMBL:AY086963 IPI:IPI00524577 RefSeq:NP_197529.1 UniGene:At.24724 ProteinModelPortal:Q93VG5 SMR:Q93VG5 IntAct:Q93VG5 STRING:Q93VG5 PRIDE:Q93VG5 EnsemblPlants:AT5G20290.1 GeneID:832151 KEGG:ath:AT5G20290 TAIR:At5g20290 HOGENOM:HBG745393 InParanoid:Q93VG5 KO:K02995 OMA:AWASEGC PhylomeDB:Q93VG5 ProtClustDB:CLSN2687206 ArrayExpress:Q93VG5 Genevestigator:Q93VG5 GermOnline:AT5G20290 TIGRFAMs:TIGR00307 Uniprot:Q93VG5

Tissue	Identification	# of reads (stress)	# of reads (control)	Fold Change	p-value	Description
Root	Isotig06244	25	77	-1.752	1.26E-08	No hit
Root	Isotig06245	23	66	-1.650	4.42E-07	No hit
Root	Isotig06343	18	0	5.041	1.86E-05	DICTYBASE|DDB_G0267846 - symbol:expl1 "expansin-like protein" species:44689 "Dictyostelium discoideum" [GO:0003674 "molecular_function" evidence=ND] [GO:0005575 "cellular_component" evidence=ND] [GO:0008150 "biological_process" evidence=ND] dictyBase:DDB_G0267846 GO:GO:0016021 GenomeReviews:CM000150_GR EMBL:AAFI02000003 InterPro:IPR014733 InterPro:IPR009009 InterPro:IPR007112 Gene3D:G3DSA:2.40.40.10 Pfam:PF03330 SMART:SM00837 SUPFAM:SSF50685 PROSITE:PS50842 InterPro:IPR007117 Gene3D:G3DSA:2.60.40.760 SUPFAM:SSF49590 RefSeq:XP_647352.1 HSSP:P43213 ProteinModelPortal:Q55G31 EnsemblProtists:DDB0231629 GeneID:8616163 KEGG:ddi:DDB_G0267846 eggNOG:COG4305 HOGENOM:HBG077223 InParanoid:Q55G31 OMA:ISKSETC ProtClustDB:CLSZ2430666 Uniprot:Q55G31
Root	Isotig06344	18	0	5.041	1.86E-05	DICTYBASE|DDB_G0267846 - symbol:expl1 "expansin-like protein" species:44689 "Dictyostelium discoideum" [GO:0003674 "molecular_function" evidence=ND] [GO:0005575 "cellular_component" evidence=ND] [GO:0008150 "biological_process" evidence=ND] dictyBase:DDB_G0267846 GO:GO:0016021 GenomeReviews:CM000150_GR EMBL:AAFI02000003 InterPro:IPR014733 InterPro:IPR009009 InterPro:IPR007112 Gene3D:G3DSA:2.40.40.10 Pfam:PF03330 SMART:SM00837 SUPFAM:SSF50685 PROSITE:PS50842 InterPro:IPR007117 Gene3D:G3DSA:2.60.40.760 SUPFAM:SSF49590 RefSeq:XP_647352.1 HSSP:P43213 ProteinModelPortal:Q55G31 EnsemblProtists:DDB0231629 GeneID:8616163 KEGG:ddi:DDB_G0267846 eggNOG:COG4305 HOGENOM:HBG077223 InParanoid:Q55G31 OMA:ISKSETC ProtClustDB:CLSZ2430666 Uniprot:Q55G31
Root	Isotig06361	2	14	-2.936	0.000925454	TAIR|locus:2168656 - symbol:YSL3 "AT5G53550" species:3702 "Arabidopsis thaliana" [GO:0006857 "oligopeptide transport" evidence=ISS] [GO:0015198 "oligopeptide transporter activity" evidence=ISS] [GO:0009624 "response to nematode" evidence=IEP] [GO:0003006 "developmental process involved in reproduction" evidence=IGI] [GO:0005886 "plasma membrane" evidence=IDA] [GO:0048316 "seed development" evidence=IGI] [GO:0051980 "iron-nicotianamine transmembrane transporter activity" evidence=IGI] InterPro:IPR004813 Pfam:PF03169 GO:GO:0016021 GO:GO:0005886 EMBL:CP002688 GenomeReviews:BA000015_GR GO:GO:0009624 GO:GO:0048316 GO:GO:0055072 EMBL:AB015476 TIGRFAMs:TIGR00728 eggNOG:COG1297 HOGENOM:HBG606963 GO:GO:0051980 EMBL:DQ385057 EMBL:AK226687 IPI:IPI00523096 RefSeq:NP_001190532.1 RefSeq:NP_200167.2 UniGene:At.49426 TCDB:2.A.67.2.4 EnsemblPlants:AT5G53550.1 EnsemblPlants:AT5G53550.2 GeneID:835437 KEGG:ath:AT5G53550 TAIR:At5g53550 InParanoid:Q2EF88 PhylomeDB:Q2EF88 ProtClustDB:CLSN2681646 Genevestigator:Q2EF88 Uniprot:Q2EF88
Root	Isotig06424	0	26	-5.829	5.70E-08	No hit
Root	Isotig06425	0	25	-5.773	1.02E-07	No hit
Root	Isotig06619	39	11	1.697	0.000152266	TAIR|locus:2149179 - symbol:SPS1F "sucrose phosphate synthase 1F" species:3702 "Arabidopsis thaliana" [GO:0005985 "sucrose metabolic process" evidence=IEA] [GO:0009058 "biosynthetic process" evidence=IEA] [GO:0016757 "transferase activity, transferring glycosyl groups" evidence=ISS] [GO:0046524 "sucrose-phosphate synthase activity" evidence=RCA;IDA] [GO:0005886 "plasma membrane" evidence=IDA] [GO:0005829 "cytosol" evidence=IDA] [GO:0009506 "plasmodesma" evidence=IDA] InterPro:IPR001296 InterPro:IPR012819 Pfam:PF00534 Pfam:PF05116 GO:GO:0005829 GO:GO:0005886 GO:GO:0009506 EMBL:CP002688 GO:GO:0009058 eggNOG:COG0438 CAZy:GT4 GO:GO:0005985 InterPro:IPR006380 EMBL:AY039911 EMBL:AY079334 EMBL:AK230113 IPI:IPI00528074 RefSeq:NP_197528.1 UniGene:At.22681 ProteinModelPortal:Q94BT0 SMR:Q94BT0 IntAct:Q94BT0 STRING:Q94BT0 PRIDE:Q94BT0 ProMEX:Q94BT0 EnsemblPlants:AT5G20280.1 GeneID:832150 KEGG:ath:AT5G20280 TAIR:At5g20280 InParanoid:Q94BT0 KO:K00696 OMA:ESGDTDY PhylomeDB:Q94BT0 ProtClustDB:CLSN2687205 ArrayExpress:Q94BT0 Genevestigator:Q94BT0 GO:GO:0046524 TIGRFAMs:TIGR02468 Uniprot:Q94BT0
Root	Isotig06620	44	11	1.871	1.68E-05	TAIR|locus:2149179 - symbol:SPS1F "sucrose phosphate synthase 1F" species:3702 "Arabidopsis thaliana" [GO:0005985 "sucrose metabolic process" evidence=IEA] [GO:0009058 "biosynthetic process" evidence=IEA] [GO:0016757 "transferase activity, transferring glycosyl groups" evidence=ISS] [GO:0046524 "sucrose-phosphate synthase activity" evidence=RCA;IDA] [GO:0005886 "plasma membrane" evidence=IDA] [GO:0005829 "cytosol" evidence=IDA] [GO:0009506 "plasmodesma" evidence=IDA] InterPro:IPR001296 InterPro:IPR012819 Pfam:PF00534 Pfam:PF05116 GO:GO:0005829 GO:GO:0005886 GO:GO:0009506 EMBL:CP002688 GO:GO:0009058 eggNOG:COG0438 CAZy:GT4 GO:GO:0005985 InterPro:IPR006380 EMBL:AY039911

Tissue	Identification	# of reads (stress)	# of reads (control)	Fold Change	p-value	Description
						EMBL:AY079334 EMBL:AK230113 IPI:IPI00528074 RefSeq:NP_197528.1 UniGene:At.22681 ProteinModelPortal:Q94BT0 SMR:Q94BT0 IntAct:Q94BT0 STRING:Q94BT0 PRIDE:Q94BT0 ProMEX:Q94BT0 EnsemblPlants:AT5G20280.1 GeneID:832150 KEGG:ath:AT5G20280 TAIR:At5g20280 InParanoid:Q94BT0 KO:K00696 OMA:ESGDTDY PhylomeDB:Q94BT0 ProtClustDB:CLSN2687205 ArrayExpress:Q94BT0 Genevestigator:Q94BT0 GO:GO:0046524 TIGRFAMs:TIGR02468 Uniprot:Q94BT0
Root	Isotig06621	31	6	2.240	4.97E-05	TAIR|locus:2156030 - symbol:OXP1 "AT5G37830" species:3702 "Arabidopsis thaliana" [GO:0016787 "hydrolase activity" evidence=ISS] [GO:0005737 "cytoplasm" evidence=NAS] [GO:0006751 "glutathione catabolic process" evidence=IMP] [GO:0017168 "5-oxoprolinase (ATP-hydrolyzing) activity" evidence=IMP] [GO:0005829 "cytosol" evidence=IDA] [GO:0009506 "plasmodesma" evidence=IDA] InterPro:IPR002821 InterPro:IPR003692 Pfam:PF01968 Pfam:PF02538 Pfam:PF05378 GO:GO:0005829 GO:GO:0009506 GO:GO:0005524 EMBL:CP002688 GenomeReviews:BA000015_GR InterPro:IPR008040 eggNOG:COG0146 EMBL:AB016873 GO:GO:0006751 EMBL:AY102096 EMBL:BT004510 EMBL:AK228904 IPI:IPI00543587 RefSeq:NP_198599.1 UniGene:At.30461 ProteinModelPortal:Q9FIZ7 STRING:Q9FIZ7 PRIDE:Q9FIZ7 EnsemblPlants:AT5G37830.1 GeneID:833761 KEGG:ath:AT5G37830 TAIR:At5g37830 InParanoid:Q9FIZ7 KO:K01469 OMA:PLTVTDC PhylomeDB:Q9FIZ7 ProtClustDB:PLN02666 Genevestigator:Q9FIZ7 GO:GO:0017168 Uniprot:Q9FIZ7
Root	Isotig06622	32	6	2.286	3.01E-05	TAIR|locus:2156030 - symbol:OXP1 "AT5G37830" species:3702 "Arabidopsis thaliana" [GO:0016787 "hydrolase activity" evidence=ISS] [GO:0005737 "cytoplasm" evidence=NAS] [GO:0006751 "glutathione catabolic process" evidence=IMP] [GO:0017168 "5-oxoprolinase (ATP-hydrolyzing) activity" evidence=IMP] [GO:0005829 "cytosol" evidence=IDA] [GO:0009506 "plasmodesma" evidence=IDA] InterPro:IPR002821 InterPro:IPR003692 Pfam:PF01968 Pfam:PF02538 Pfam:PF05378 GO:GO:0005829 GO:GO:0009506 GO:GO:0005524 EMBL:CP002688 GenomeReviews:BA000015_GR InterPro:IPR008040 eggNOG:COG0146 EMBL:AB016873 GO:GO:0006751 EMBL:AY102096 EMBL:BT004510 EMBL:AK228904 IPI:IPI00543587 RefSeq:NP_198599.1 UniGene:At.30461 ProteinModelPortal:Q9FIZ7 STRING:Q9FIZ7 PRIDE:Q9FIZ7 EnsemblPlants:AT5G37830.1 GeneID:833761 KEGG:ath:AT5G37830 TAIR:At5g37830 InParanoid:Q9FIZ7 KO:K01469 OMA:PLTVTDC PhylomeDB:Q9FIZ7 ProtClustDB:PLN02666 Genevestigator:Q9FIZ7 GO:GO:0017168 Uniprot:Q9FIZ7
Root	Isotig06630	32	9	1.701	0.000589296	UNIPROTKB|E1B7V8 - symbol:PLEKHA4 "Uncharacterized protein" species:9913 "Bos taurus" [GO:0005543 "phospholipid binding" evidence=IEA] Pfam:PF00169 InterPro:IPR001849 PROSITE:PS50003 SMART:SM00233 InterPro:IPR011993 Gene3D:G3DSA:2.30.29.30 GeneTree:ENSGT00530000063012 EMBL:DAAA02047467 IPI:IPI00923864 Ensembl:ENSBTAT00000061135 Uniprot:E1B7V8
Root	Isotig06643	2	31	-4.083	1.93E-08	TAIR|locus:2100367 - symbol:ABCA2 "AT3G47730" species:3702 "Arabidopsis thaliana" [GO:0005215 "transporter activity" evidence=ISS] [GO:0042626 "ATPase activity, coupled to transmembrane movement of substances" evidence=ISS] InterPro:IPR003439 InterPro:IPR003593 InterPro:IPR017871 Pfam:PF00005 PROSITE:PS00211 PROSITE:PS50893 SMART:SM00382 GO:GO:0016021 GO:GO:0005524 EMBL:CP002686 GenomeReviews:BA000014_GR GO:GO:0006810 eggNOG:COG1131 GO:GO:0016887 EMBL:AL049746 EMBL:BT004197 EMBL:BT005456 IPI:IPI00534264 PIR:T07712 RefSeq:NP_190357.2 UniGene:At.23005 ProteinModelPortal:Q84K47 SMR:Q84K47 PRIDE:Q84K47 EnsemblPlants:AT3G47730.1 GeneID:823927 KEGG:ath:AT3G47730 TAIR:At3g47730 HOGENOM:HBG317214 InParanoid:Q84K47 OMA:SLCISGH PhylomeDB:Q84K47 ProtClustDB:CLSN2686937 ArrayExpress:Q84K47 Genevestigator:Q84K47 GermOnline:AT3G47730 Uniprot:Q84K47
Root	Isotig06644	4	19	-2.377	0.000578515	TAIR|locus:2100367 - symbol:ABCA2 "AT3G47730" species:3702 "Arabidopsis thaliana" [GO:0005215 "transporter activity" evidence=ISS] [GO:0042626 "ATPase activity, coupled to transmembrane movement of substances" evidence=ISS] InterPro:IPR003439 InterPro:IPR003593 InterPro:IPR017871 Pfam:PF00005 PROSITE:PS00211 PROSITE:PS50893 SMART:SM00382 GO:GO:0016021 GO:GO:0005524 EMBL:CP002686 GenomeReviews:BA000014_GR GO:GO:0006810 eggNOG:COG1131 GO:GO:0016887 EMBL:AL049746 EMBL:BT004197 EMBL:BT005456 IPI:IPI00534264 PIR:T07712 RefSeq:NP_190357.2 UniGene:At.23005 ProteinModelPortal:Q84K47 SMR:Q84K47 PRIDE:Q84K47 EnsemblPlants:AT3G47730.1 GeneID:823927 KEGG:ath:AT3G47730 TAIR:At3g47730 HOGENOM:HBG317214 InParanoid:Q84K47 OMA:SLCISGH PhylomeDB:Q84K47 ProtClustDB:CLSN2686937 ArrayExpress:Q84K47 Genevestigator:Q84K47 GermOnline:AT3G47730 Uniprot:Q84K47
Root	Isotig06647	4	21	-2.521	0.000181802	TAIR|locus:2019863 - symbol:AT1G79620 "AT1G79620" species:3702 "Arabidopsis thaliana" [GO:0004674 "protein serine/threonine kinase activity" evidence=ISS] [GO:0005524 "ATP binding" evidence=ISS] [GO:0006468 "protein phosphorylation" evidence=IEA;ISS] [GO:0007169 "transmembrane receptor protein tyrosine kinase signaling

Tissue	Identification	# of reads (stress)	# of reads (control)	Fold Change	p-value	Description
						pathway" evidence=ISS] [GO:0016301 "kinase activity" evidence=ISS] Pfam:PF00560 InterPro:IPR001611 InterPro:IPR000719 InterPro:IPR001245 InterPro:IPR008271 InterPro:IPR011009 InterPro:IPR017441 Pfam:PF07714 PROSITE:PS00107 PROSITE:PS00108 PROSITE:PS50011 GO:GO:0016021 EMBL:CP002684 GenomeReviews:CT485782_GR GO:GO:0005524 eggNOG:COG0515 GO:GO:0004872 SUPFAM:SSF56112 GO:GO:0004674 HSSP:P36897 InterPro:IPR013210 Pfam:PF08263 HOGENOM:HBG316990 EMBL:FJ708684 EMBL:AK117863 IPI:IPI00517320 RefSeq:NP_178080.2 UniGene:At.46098 ProteinModelPortal:Q8GY50 SMR:Q8GY50 PRIDE:Q8GY50 EnsemblPlants:AT1G79620.1 GeneID:844301 KEGG:ath:AT1G79620 TAIR:At1g79620 InParanoid:Q8GY50 OMA:YFIASPY PhylomeDB:Q8GY50 ProtClustDB:CLSN2680668 Genevestigator:Q8GY50 Uniprot:Q8GY50
Root	Isotig06648	4	21	-2.521	0.000181802	TAIR|locus:2019863 - symbol:AT1G79620 "AT1G79620" species:3702 "Arabidopsis thaliana" [GO:0004674 "protein serine/threonine kinase activity" evidence=ISS] [GO:0005524 "ATP binding" evidence=ISS] [GO:0006468 "protein phosphorylation" evidence=IEA;ISS] [GO:0007169 "transmembrane receptor protein tyrosine kinase signaling pathway" evidence=ISS] [GO:0016301 "kinase activity" evidence=ISS] Pfam:PF00560 InterPro:IPR001611 InterPro:IPR000719 InterPro:IPR001245 InterPro:IPR008271 InterPro:IPR011009 InterPro:IPR017441 Pfam:PF07714 PROSITE:PS00107 PROSITE:PS00108 PROSITE:PS50011 GO:GO:0016021 EMBL:CP002684 GenomeReviews:CT485782_GR GO:GO:0005524 eggNOG:COG0515 GO:GO:0004872 SUPFAM:SSF56112 GO:GO:0004674 HSSP:P36897 InterPro:IPR013210 Pfam:PF08263 HOGENOM:HBG316990 EMBL:FJ708684 EMBL:AK117863 IPI:IPI00517320 RefSeq:NP_178080.2 UniGene:At.46098 ProteinModelPortal:Q8GY50 SMR:Q8GY50 PRIDE:Q8GY50 EnsemblPlants:AT1G79620.1 GeneID:844301 KEGG:ath:AT1G79620 TAIR:At1g79620 InParanoid:Q8GY50 OMA:YFIASPY PhylomeDB:Q8GY50 ProtClustDB:CLSN2680668 Genevestigator:Q8GY50 Uniprot:Q8GY50
Root	Isotig06659	12	61	-2.475	2.75E-10	TAIR|locus:2007760 - symbol:AGO1 "AT1G48410" species:3702 "Arabidopsis thaliana" [GO:0016441 "posttranscriptional gene silencing" evidence=IEP] [GO:0035195 "gene silencing by miRNA" evidence=IGI;IEP;IMP] [GO:0009733 "response to auxin stimulus" evidence=IGI] [GO:0009850 "auxin metabolic process" evidence=IGI] [GO:0010218 "response to far red light" evidence=IGI] [GO:0048830 "adventitious root development" evidence=IMP] [GO:0004521 "endoribonuclease activity" evidence=IDA] [GO:0035197 "siRNA binding" evidence=IPI] [GO:0035198 "miRNA binding" evidence=IPI] [GO:0009616 "virus induced gene silencing" evidence=IDA] [GO:0005634 "nucleus" evidence=IDA] [GO:0005737 "cytoplasm" evidence=IDA;TAS] [GO:0016246 "RNA interference" evidence=IMP] [GO:0005515 "protein binding" evidence=IPI] [GO:0005829 "cytosol" evidence=IDA] [GO:0048864 "stem cell development" evidence=IGI] [GO:0045087 "innate immune response" evidence=IMP] [GO:0000300 "peripheral to membrane of membrane fraction" evidence=IDA] [GO:0009965 "leaf morphogenesis" evidence=IMP] [GO:0009793 "embryo development ending in seed dormancy" evidence=IGI] InterPro:IPR003100 InterPro:IPR003165 InterPro:IPR012337 Pfam:PF02170 Pfam:PF02171 PROSITE:PS50821 PROSITE:PS50822 SMART:SM00949 SMART:SM00950 EMBL:CP002684 GenomeReviews:CT485782_GR GO:GO:0005829 GO:GO:0005634 GO:GO:0005515 GO:GO:0009733 GO:GO:0006417 GO:GO:0016246 GO:GO:0006355 GO:GO:0006351 GO:GO:0010218 GO:GO:0009793 GO:GO:0030529 GO:GO:0009850 SUPFAM:SSF53098 GO:GO:0009965 eggNOG:NOG279895 HOGENOM:HBG717005 KO:K11596 GO:GO:0035198 InterPro:IPR014811 Pfam:PF08699 SUPFAM:SSF101690 EMBL:U91995 EMBL:AC007932 EMBL:AC020889 EMBL:BT000941 EMBL:AK227868 IPI:IPI00522985 IPI:IPI00523962 RefSeq:NP_001185169.1 RefSeq:NP_175274.1 RefSeq:NP_849784.1 UniGene:At.21124 ProteinModelPortal:O04379 SMR:O04379 STRING:O04379 PRIDE:O04379 EnsemblPlants:AT1G48410.1 GeneID:841262 KEGG:ath:AT1G48410 TAIR:At1g48410 InParanoid:O04379 OMA:RANHFQI PhylomeDB:O04379 ProtClustDB:CLSN2693050 ArrayExpress:O04379 Genevestigator:O04379 GO:GO:0004521 GO:GO:0035197 GO:GO:0048830 GO:GO:0035195 GO:GO:0048864 GO:GO:0009616 InterPro:IPR024357 Pfam:PF12764 Uniprot:O04379
Root	Isotig06660	12	62	-2.498	1.57E-10	TAIR|locus:2007760 - symbol:AGO1 "AT1G48410" species:3702 "Arabidopsis thaliana" [GO:0016441 "posttranscriptional gene silencing" evidence=IEP] [GO:0035195 "gene silencing by miRNA" evidence=IGI;IEP;IMP] [GO:0009733 "response to auxin stimulus" evidence=IGI] [GO:0009850 "auxin metabolic process" evidence=IGI] [GO:0010218 "response to far red light" evidence=IGI] [GO:0048830 "adventitious root development" evidence=IMP] [GO:0004521 "endoribonuclease activity" evidence=IDA] [GO:0035197 "siRNA binding" evidence=IPI] [GO:0035198 "miRNA binding" evidence=IPI] [GO:0009616 "virus induced gene silencing" evidence=IDA] [GO:0005634 "nucleus" evidence=IDA] [GO:0005737 "cytoplasm" evidence=IDA;TAS] [GO:0016246 "RNA interference" evidence=IMP] [GO:0005515 "protein binding" evidence=IPI] [GO:0005829 "cytosol" evidence=IDA] [GO:0048864 "stem cell

Tissue	Identification	# of reads (stress)	# of reads (control)	Fold Change	p-value	Description
						development" evidence=IGI] [GO:0045087 "innate immune response" evidence=IMP] [GO:0000300 "peripheral to membrane of membrane fraction" evidence=IDA] [GO:0009965 "leaf morphogenesis" evidence=IMP] [GO:0009793 "embryo development ending in seed dormancy" evidence=IGI] InterPro:IPR003100 InterPro:IPR003165 InterPro:IPR012337 Pfam:PF02170 Pfam:PF02171 PROSITE:PS50821 PROSITE:PS50822 SMART:SM00949 SMART:SM00950 EMBL:CP002684 GenomeReviews:CT485782_GR GO:GO:0005829 GO:GO:0005634 GO:GO:0005515 GO:GO:0009733 GO:GO:0006417 GO:GO:0016246 GO:GO:0006355 GO:GO:0006351 GO:GO:0010218 GO:GO:0009793 GO:GO:0030529 GO:GO:0009850 SUPFAM:SSF53098 GO:GO:0009965 eggNOG:NOG279895 HOGENOM:HBG717005 KO:K11596 GO:GO:0035198 InterPro:IPR014811 Pfam:PF08699 SUPFAM:SSF101690 EMBL:U91995 EMBL:AC007932 EMBL:AC020889 EMBL:BT000941 EMBL:AK227868 IPI:IPI00522985 IPI:IPI00523962 RefSeq:NP_001185169.1 RefSeq:NP_175274.1 RefSeq:NP_849784.1 UniGene:At.21124 ProteinModelPortal:O04379 SMR:O04379 STRING:O04379 PRIDE:O04379 EnsemblPlants:AT1G48410.1 GeneID:841262 KEGG:ath:AT1G48410 TAIR:At1g48410 InParanoid:O04379 OMA:RANHFQI PhylomeDB:O04379 ProtClustDB:CLSN2693050 ArrayExpress:O04379 Genevestigator:O04379 GO:GO:0004521 GO:GO:0035197 GO:GO:0048830 GO:GO:0035195 GO:GO:0048864 GO:GO:0009616 InterPro:IPR024357 Pfam:PF12764 Uniprot:O04379
Root	Isotig06663	15	38	-1.470	0.00042777	TAIR|locus:2182397 - symbol:AT5G35160 "AT5G35160" species:3702 "Arabidopsis thaliana" [GO:0016021 "integral to membrane" evidence=ISS] [GO:0005794 "Golgi apparatus" evidence=IDA] [GO:0005829 "cytosol" evidence=RCA] InterPro:IPR004240 Pfam:PF02990 GO:GO:0016021 EMBL:CP002688 GO:GO:0005794 PANTHER:PTHR10766 EMBL:AP000421 IPI:IPI00992767 RefSeq:NP_001190420.1 UniGene:At.1657 STRING:Q9FYQ8 PRIDE:Q9FYQ8 EnsemblPlants:AT5G35160.2 GeneID:833470 KEGG:ath:AT5G35160 TAIR:At5g35160 InParanoid:Q9FYQ8 PhylomeDB:Q9FYQ8 Genevestigator:Q9FYQ8 Uniprot:Q9FYQ8
Root	Isotig06667	19	48	-1.466	7.79E-05	TAIR|locus:2123777 - symbol:GLDP1 "AT4G33010" species:3702 "Arabidopsis thaliana" [GO:0004375 "glycine dehydrogenase (decarboxylating) activity" evidence=ISS] [GO:0005960 "glycine cleavage complex" evidence=ISS] [GO:0019464 "glycine decarboxylation via glycine cleavage system" evidence=ISS] [GO:0009941 "chloroplast envelope" evidence=IDA] [GO:0005739 "mitochondrion" evidence=IDA] [GO:0006546 "glycine catabolic process" evidence=IMP] [GO:0009507 "chloroplast" evidence=IDA] [GO:0048046 "apoplast" evidence=IDA] [GO:0005515 "protein binding" evidence=IPI] [GO:0046686 "response to cadmium ion" evidence=IEP] [GO:0009534 "chloroplast thylakoid" evidence=IDA] [GO:0009570 "chloroplast stroma" evidence=IDA] InterPro:IPR003437 InterPro:IPR015421 InterPro:IPR020580 InterPro:IPR020581 Pfam:PF02347 GO:GO:0005739 EMBL:CP002687 GenomeReviews:CT486007_GR GO:GO:0048046 GO:GO:0009570 GO:GO:0046686 GO:GO:0005515 GO:GO:0030170 InterPro:IPR015424 Gene3D:G3DSA:3.40.640.10 SUPFAM:SSF53383 GO:GO:0009941 GO:GO:0009534 EMBL:AL031804 EMBL:AL161582 GO:GO:0006546 GO:GO:0004375 UniGene:At.24550 HOGENOM:HBG286723 KO:K00281 ProtClustDB:PLN02414 PANTHER:PTHR11773 TIGRFAMs:TIGR00461 EMBL:AY063903 EMBL:AY091186 EMBL:AY042800 EMBL:AY128922 EMBL:AY065004 EMBL:BT001132 EMBL:BT000446 IPI:IPI00536025 PIR:T05309 RefSeq:NP_195027.1 UniGene:At.22214 ProteinModelPortal:Q94B78 SMR:Q94B78 IntAct:Q94B78 STRING:Q94B78 PRIDE:Q94B78 ProMEX:Q94B78 EnsemblPlants:AT4G33010.1 GeneID:829438 KEGG:ath:AT4G33010 GeneFarm:1737 TAIR:At4g33010 InParanoid:Q94B78 OMA:VPMSEYQ PhylomeDB:Q94B78 ArrayExpress:Q94B78 Genevestigator:Q94B78 GermOnline:AT4G33010 Uniprot:Q94B78
Root	Isotig06668	19	51	-1.553	2.13E-05	TAIR|locus:2123777 - symbol:GLDP1 "AT4G33010" species:3702 "Arabidopsis thaliana" [GO:0004375 "glycine dehydrogenase (decarboxylating) activity" evidence=ISS] [GO:0005960 "glycine cleavage complex" evidence=ISS] [GO:0019464 "glycine decarboxylation via glycine cleavage system" evidence=ISS] [GO:0009941 "chloroplast envelope" evidence=IDA] [GO:0005739 "mitochondrion" evidence=IDA] [GO:0006546 "glycine catabolic process" evidence=IMP] [GO:0009507 "chloroplast" evidence=IDA] [GO:0048046 "apoplast" evidence=IDA] [GO:0005515 "protein binding" evidence=IPI] [GO:0046686 "response to cadmium ion" evidence=IEP] [GO:0009534 "chloroplast thylakoid" evidence=IDA] [GO:0009570 "chloroplast stroma" evidence=IDA] InterPro:IPR003437 InterPro:IPR015421 InterPro:IPR020580 InterPro:IPR020581 Pfam:PF02347 GO:GO:0005739 EMBL:CP002687 GenomeReviews:CT486007_GR GO:GO:0048046 GO:GO:0009570 GO:GO:0046686 GO:GO:0005515 GO:GO:0030170 InterPro:IPR015424 Gene3D:G3DSA:3.40.640.10 SUPFAM:SSF53383 GO:GO:0009941 GO:GO:0009534 EMBL:AL031804 EMBL:AL161582 GO:GO:0006546 GO:GO:0004375 UniGene:At.24550 HOGENOM:HBG286723 KO:K00281 ProtClustDB:PLN02414 PANTHER:PTHR11773 TIGRFAMs:TIGR00461 EMBL:AY063903 EMBL:AY091186 EMBL:AY042800 EMBL:AY128922 EMBL:AY065004 EMBL:BT001132

Tissue	Identification	# of reads (stress)	# of reads (control)	Fold Change	p-value	Description
						EMBL:BT000446 IPI:IPI00536025 PIR:T05309 RefSeq:NP_195027.1 UniGene:At.22214 ProteinModelPortal:Q94B78 SMR:Q94B78 IntAct:Q94B78 STRING:Q94B78 PRIDE:Q94B78 ProMEX:Q94B78 EnsemblPlants:AT4G33010.1 GeneID:829438 KEGG:ath:AT4G33010 GeneFarm:1737 TAIR:At4g33010 InParanoid:Q94B78 OMA:VPMSEYQ PhylomeDB:Q94B78 ArrayExpress:Q94B78 Genevestigator:Q94B78 GermOnline:AT4G33010 Uniprot:Q94B78
Root	Isotig06683	15	44	-1.682	2.92E-05	TAIR|locus:2011030 - symbol:LOX1 "AT1G55020" species:3702 "Arabidopsis thaliana" [GO:0005575 "cellular_component" evidence=ND] [GO:0010386 "lateral root primordium development" evidence=IMP] [GO:0048364 "root development" evidence=IMP] [GO:0016165 "lipoxygenase activity" evidence=ISS;IMP;IDA] [GO:0006952 "defense response" evidence=TAS] [GO:0009611 "response to wounding" evidence=TAS] [GO:0009695 "jasmonic acid biosynthetic process" evidence=TAS] [GO:0009737 "response to abscisic acid stimulus" evidence=IEP] [GO:0009753 "response to jasmonic acid stimulus" evidence=IEP] [GO:0009816 "defense response to bacterium, incompatible interaction" evidence=IEP] [GO:0030397 "membrane disassembly" evidence=TAS] [GO:0040007 "growth" evidence=TAS] InterPro:IPR000907 InterPro:IPR001024 InterPro:IPR001246 InterPro:IPR013819 InterPro:IPR020833 InterPro:IPR020834 Pfam:PF00305 Pfam:PF01477 PRINTS:PR00087 PRINTS:PR00468 PROSITE:PS00081 PROSITE:PS00711 PROSITE:PS50095 PROSITE:PS51393 SMART:SM00308 EMBL:CP002684 GenomeReviews:CT485782_GR GO:GO:0009737 GO:GO:0005737 GO:GO:0009753 GO:GO:0005506 GO:GO:0031408 GO:GO:0010386 EMBL:AC064840 EMBL:AC069144 InterPro:IPR008976 Gene3D:G3DSA:2.60.60.20 SUPFAM:SSF49723 GO:GO:0016165 PANTHER:PTHR11771 SUPFAM:SSF48484 eggNOG:NOG69653 EMBL:L04637 EMBL:U01843 EMBL:AY093104 EMBL:BT010358 IPI:IPI00547307 PIR:JQ2267 RefSeq:NP_175900.1 UniGene:At.19984 UniGene:At.67309 ProteinModelPortal:Q06327 SMR:Q06327 STRING:Q06327 PRIDE:Q06327 EnsemblPlants:AT1G55020.1 GeneID:841944 KEGG:ath:AT1G55020 TAIR:At1g55020 HOGENOM:HBG749429 InParanoid:Q06327 KO:K15718 OMA:TPKEFDS PhylomeDB:Q06327 ProtClustDB:PLN02337 ArrayExpress:Q06327 Genevestigator:Q06327 GermOnline:AT1G55020 Uniprot:Q06327
Root	Isotig06684	15	43	-1.648	4.63E-05	TAIR|locus:2011030 - symbol:LOX1 "AT1G55020" species:3702 "Arabidopsis thaliana" [GO:0005575 "cellular_component" evidence=ND] [GO:0010386 "lateral root primordium development" evidence=IMP] [GO:0048364 "root development" evidence=IMP] [GO:0016165 "lipoxygenase activity" evidence=ISS;IMP;IDA] [GO:0006952 "defense response" evidence=TAS] [GO:0009611 "response to wounding" evidence=TAS] [GO:0009695 "jasmonic acid biosynthetic process" evidence=TAS] [GO:0009737 "response to abscisic acid stimulus" evidence=IEP] [GO:0009753 "response to jasmonic acid stimulus" evidence=IEP] [GO:0009816 "defense response to bacterium, incompatible interaction" evidence=IEP] [GO:0030397 "membrane disassembly" evidence=TAS] [GO:0040007 "growth" evidence=TAS] InterPro:IPR000907 InterPro:IPR001024 InterPro:IPR001246 InterPro:IPR013819 InterPro:IPR020833 InterPro:IPR020834 Pfam:PF00305 Pfam:PF01477 PRINTS:PR00087 PRINTS:PR00468 PROSITE:PS00081 PROSITE:PS00711 PROSITE:PS50095 PROSITE:PS51393 SMART:SM00308 EMBL:CP002684 GenomeReviews:CT485782_GR GO:GO:0009737 GO:GO:0005737 GO:GO:0009753 GO:GO:0005506 GO:GO:0031408 GO:GO:0010386 EMBL:AC064840 EMBL:AC069144 InterPro:IPR008976 Gene3D:G3DSA:2.60.60.20 SUPFAM:SSF49723 GO:GO:0016165 PANTHER:PTHR11771 SUPFAM:SSF48484 eggNOG:NOG69653 EMBL:L04637 EMBL:U01843 EMBL:AY093104 EMBL:BT010358 IPI:IPI00547307 PIR:JQ2267 RefSeq:NP_175900.1 UniGene:At.19984 UniGene:At.67309 ProteinModelPortal:Q06327 SMR:Q06327 STRING:Q06327 PRIDE:Q06327 EnsemblPlants:AT1G55020.1 GeneID:841944 KEGG:ath:AT1G55020 TAIR:At1g55020 HOGENOM:HBG749429 InParanoid:Q06327 KO:K15718 OMA:TPKEFDS PhylomeDB:Q06327 ProtClustDB:PLN02337 ArrayExpress:Q06327 Genevestigator:Q06327 GermOnline:AT1G55020 Uniprot:Q06327
Root	Isotig06693	4	31	-3.083	4.53E-07	TAIR|locus:2133995 - symbol:AK-HSDH II "AT4G19710" species:3702 "Arabidopsis thaliana" [GO:0004072 "aspartate kinase activity" evidence=IDA] [GO:0004412 "homoserine dehydrogenase activity" evidence=IDA] [GO:0009507 "chloroplast" evidence=IDA] [GO:0009570 "chloroplast stroma" evidence=IDA] [GO:0009067 "aspartate family amino acid biosynthetic process" evidence=TAS] InterPro:IPR001048 InterPro:IPR001341 InterPro:IPR001342 InterPro:IPR002912 InterPro:IPR005106 InterPro:IPR011147 InterPro:IPR016040 InterPro:IPR018042 InterPro:IPR019811 Pfam:PF00696 Pfam:PF00742 Pfam:PF01842 Pfam:PF03447 PIRSF:PIRSF000727 PROSITE:PS00324 PROSITE:PS01042 GO:GO:0005524 EMBL:CP002687 GenomeReviews:CT486007_GR GO:GO:0009570 Gene3D:G3DSA:3.40.50.720 GO:GO:0050661 GO:GO:0016597 eggNOG:COG0527 HOGENOM:HBG298754 KO:K12524 OMA:GANEMNI GO:GO:0004072 GO:GO:0004412

Tissue	Identification	# of reads (stress)	# of reads (control)	Fold Change	p-value	Description
						Gene3D:G3DSA:3.40.1160.10 SUPFAM:SSF53633 TIGRFAMs:TIGR00657 GO:GO:0009086 ProtClustDB:CLSN2682367 EMBL:AL024486 EMBL:AL161551 EMBL:BX827863 IPI:IPI00521909 IPI:IPI00539074 PIR:T04752 RefSeq:NP_193706.2 RefSeq:NP_974576.1 UniGene:At.32787 ProteinModelPortal:O81852 SMR:O81852 STRING:O81852 PRIDE:O81852 EnsemblPlants:AT4G19710.2 GeneID:827715 KEGG:ath:AT4G19710 TAIR:At4g19710 InParanoid:O81852 PhylomeDB:O81852 BRENDA:1.1.1.3 ArrayExpress:O81852 Genevestigator:O81852 GermOnline:AT4G19710 Uniprot:O81852
Root	Isotig06694	4	32	-3.129	2.46E-07	TAIR|locus:2133995 - symbol:AK-HSDH II "AT4G19710" species:3702 "Arabidopsis thaliana" [GO:0004072 "aspartate kinase activity" evidence=IDA] [GO:0004412 "homoserine dehydrogenase activity" evidence=IDA] [GO:0009507 "chloroplast" evidence=IDA] [GO:0009570 "chloroplast stroma" evidence=IDA] [GO:0009067 "aspartate family amino acid biosynthetic process" evidence=TAS] InterPro:IPR001048 InterPro:IPR001341 InterPro:IPR001342 InterPro:IPR002912 InterPro:IPR005106 InterPro:IPR011147 InterPro:IPR016040 InterPro:IPR018042 InterPro:IPR019811 Pfam:PF00696 Pfam:PF00742 Pfam:PF01842 Pfam:PF03447 PIRSF:PIRSF000727 PROSITE:PS00324 PROSITE:PS01042 GO:GO:0005524 EMBL:CP002687 GenomeReviews:CT486007_GR GO:GO:0009570 Gene3D:G3DSA:3.40.50.720 GO:GO:0050661 GO:GO:0016597 eggNOG:COG0527 HOGENOM:HBG298754 KO:K12524 OMA:GANEMNI GO:GO:0004072 GO:GO:0004412 Gene3D:G3DSA:3.40.1160.10 SUPFAM:SSF53633 TIGRFAMs:TIGR00657 GO:GO:0009086 ProtClustDB:CLSN2682367 EMBL:AL024486 EMBL:AL161551 EMBL:BX827863 IPI:IPI00521909 IPI:IPI00539074 PIR:T04752 RefSeq:NP_193706.2 RefSeq:NP_974576.1 UniGene:At.32787 ProteinModelPortal:O81852 SMR:O81852 STRING:O81852 PRIDE:O81852 EnsemblPlants:AT4G19710.2 GeneID:827715 KEGG:ath:AT4G19710 TAIR:At4g19710 InParanoid:O81852 PhylomeDB:O81852 BRENDA:1.1.1.3 ArrayExpress:O81852 Genevestigator:O81852 GermOnline:AT4G19710 Uniprot:O81852
Root	Isotig06697	68	6	3.374	1.04E-13	TAIR|locus:2175579 - symbol:ACA8 "AT5G57110" species:3702 "Arabidopsis thaliana" [GO:0005388 "calcium-transporting ATPase activity" evidence=ISS;IDA] [GO:0005886 "plasma membrane" evidence=IDA] [GO:0005516 "calmodulin binding" evidence=TAS] [GO:0043621 "protein self-association" evidence=IDA] [GO:0005515 "protein binding" evidence=IPI] [GO:0009624 "response to nematode" evidence=IEP] [GO:0016020 "membrane" evidence=IDA] [GO:0009506 "plasmodesma" evidence=IDA] InterPro:IPR000695 InterPro:IPR001757 InterPro:IPR005834 InterPro:IPR006408 InterPro:IPR008250 InterPro:IPR024750 Pfam:PF00122 Pfam:PF00702 Pfam:PF12515 PRINTS:PR00119 PRINTS:PR00120 InterPro:IPR018303 InterPro:IPR004014 InterPro:IPR006068 Pfam:PF00690 Pfam:PF00689 Prosite:PS00154 GO:GO:0016021 GO:GO:0005886 GO:GO:0009506 GO:GO:0005524 EMBL:CP002688 GenomeReviews:BA000015_GR GO:GO:0046872 InterPro:IPR023214 SUPFAM:SSF56784 GO:GO:0006754 GO:GO:0009624 GO:GO:0005516 GO:GO:0043621 eggNOG:COG0474 HOGENOM:HBG456486 GO:GO:0005388 InterPro:IPR023300 InterPro:IPR023299 InterPro:IPR023298 Gene3D:G3DSA:2.70.150.10 Gene3D:G3DSA:3.40.1110.10 Gene3D:G3DSA:1.20.1110.10 SMART:SM00831 TIGRFAMs:TIGR01517 TIGRFAMs:TIGR01494 EMBL:AJ249352 EMBL:AB023042 EMBL:AY069869 IPI:IPI00540817 PIR:T52654 RefSeq:NP_200521.3 RefSeq:NP_851200.1 UniGene:At.9676 ProteinModelPortal:Q9LF79 SMR:Q9LF79 IntAct:Q9LF79 TCDB:3.A.3.2.10 PRIDE:Q9LF79 EnsemblPlants:AT5G57110.1 EnsemblPlants:AT5G57110.2 GeneID:835815 KEGG:ath:AT5G57110 TAIR:At5g57110 InParanoid:Q9LF79 OMA:VAFVGAC PhylomeDB:Q9LF79 ProtClustDB:CLSN2685405 ArrayExpress:Q9LF79 Genevestigator:Q9LF79 GermOnline:AT5G57110 Uniprot:Q9LF79
Root	Isotig06698	35	2	4.000	1.39E-08	TAIR|locus:2175579 - symbol:ACA8 "AT5G57110" species:3702 "Arabidopsis thaliana" [GO:0005388 "calcium-transporting ATPase activity" evidence=ISS;IDA] [GO:0005886 "plasma membrane" evidence=IDA] [GO:0005516 "calmodulin binding" evidence=TAS] [GO:0043621 "protein self-association" evidence=IDA] [GO:0005515 "protein binding" evidence=IPI] [GO:0009624 "response to nematode" evidence=IEP] [GO:0016020 "membrane" evidence=IDA] [GO:0009506 "plasmodesma" evidence=IDA] InterPro:IPR000695 InterPro:IPR001757 InterPro:IPR005834 InterPro:IPR006408 InterPro:IPR008250 InterPro:IPR024750 Pfam:PF00122 Pfam:PF00702 Pfam:PF12515 PRINTS:PR00119 PRINTS:PR00120 InterPro:IPR018303 InterPro:IPR004014 InterPro:IPR006068 Pfam:PF00690 Pfam:PF00689 Prosite:PS00154 GO:GO:0016021 GO:GO:0005886 GO:GO:0009506 GO:GO:0005524 EMBL:CP002688 GenomeReviews:BA000015_GR GO:GO:0046872 InterPro:IPR023214 SUPFAM:SSF56784 GO:GO:0006754 GO:GO:0009624 GO:GO:0005516 GO:GO:0043621 eggNOG:COG0474 HOGENOM:HBG456486 GO:GO:0005388 InterPro:IPR023300 InterPro:IPR023299 InterPro:IPR023298 Gene3D:G3DSA:2.70.150.10 Gene3D:G3DSA:3.40.1110.10 Gene3D:G3DSA:1.20.1110.10 SMART:SM00831 TIGRFAMs:TIGR01517 TIGRFAMs:TIGR01494 EMBL:AJ249352 EMBL:AB023042 EMBL:AY069869

Tissue	Identification	# of reads (stress)	# of reads (control)	Fold Change	p-value	Description
						IPI:IPI00540817 PIR:T52654 RefSeq:NP_200521.3 RefSeq:NP_851200.1 UniGene:At.9676 ProteinModelPortal:Q9LF79 SMR:Q9LF79 IntAct:Q9LF79 TCDB:3.A.3.2.10 PRIDE:Q9LF79 EnsemblPlants:AT5G57110.1 EnsemblPlants:AT5G57110.2 GeneID:835815 KEGG:ath:AT5G57110 TAIR:At5g57110 InParanoid:Q9LF79 OMA:VAFVGAC PhylomeDB:Q9LF79 ProtClustDB:CLSN2685405 ArrayExpress:Q9LF79 Genevestigator:Q9LF79 GermOnline:AT5G57110 Uniprot:Q9LF79
Root	Isotig06705	12	32	-1.544	0.000803022	TAIR|locus:2025991 - symbol:ARF1 "AT1G59750" species:3702 "Arabidopsis thaliana" [GO:0005634 "nucleus" evidence=ISS] [GO:0003677 "DNA binding" evidence=IDA;IPI] [GO:0003700 "sequence-specific DNA binding transcription factor activity" evidence=ISS;TAS] [GO:0010150 "leaf senescence" evidence=IGI] [GO:0045892 "negative regulation of transcription, DNA-dependent" evidence=IEP] [GO:0005515 "protein binding" evidence=IPI] [GO:0006355 "regulation of transcription, DNA-dependent" evidence=TAS] [GO:0009733 "response to auxin stimulus" evidence=IEP] InterPro:IPR003311 InterPro:IPR003340 InterPro:IPR010525 InterPro:IPR011525 Pfam:PF02309 Pfam:PF02362 Pfam:PF06507 PROSITE:PS50863 PROSITE:PS50962 SMART:SM01019 EMBL:CP002684 GenomeReviews:CT485782_GR GO:GO:0005634 GO:GO:0045892 GO:GO:0009734 GO:GO:0003677 GO:GO:0006351 GO:GO:0010150 EMBL:AC007258 GO:GO:0046983 InterPro:IPR015300 Gene3D:G3DSA:2.40.330.10 SUPFAM:SSF101936 EMBL:U83245 EMBL:AY133723 EMBL:BT002748 IPI:IPI00518211 IPI:IPI00520405 PIR:D96621 RefSeq:NP_001031208.1 RefSeq:NP_176184.1 RefSeq:NP_849830.1 UniGene:At.74832 ProteinModelPortal:Q8L7G0 SMR:Q8L7G0 IntAct:Q8L7G0 STRING:Q8L7G0 PRIDE:Q8L7G0 EnsemblPlants:AT1G59750.1 EnsemblPlants:AT1G59750.3 GeneID:842268 KEGG:ath:AT1G59750 GeneFarm:3363 TAIR:At1g59750 eggNOG:NOG257828 HOGENOM:HBG746399 InParanoid:Q8L7G0 KO:K14486 OMA:NEFCGMV PhylomeDB:Q8L7G0 ProtClustDB:CLSN2679699 ArrayExpress:Q8L7G0 Genevestigator:Q8L7G0 GermOnline:AT1G59750 Uniprot:Q8L7G0
Root	Isotig06706	12	32	-1.544	0.000803022	TAIR|locus:2025991 - symbol:ARF1 "AT1G59750" species:3702 "Arabidopsis thaliana" [GO:0005634 "nucleus" evidence=ISS] [GO:0003677 "DNA binding" evidence=IDA;IPI] [GO:0003700 "sequence-specific DNA binding transcription factor activity" evidence=ISS;TAS] [GO:0010150 "leaf senescence" evidence=IGI] [GO:0045892 "negative regulation of transcription, DNA-dependent" evidence=IEP] [GO:0005515 "protein binding" evidence=IPI] [GO:0006355 "regulation of transcription, DNA-dependent" evidence=TAS] [GO:0009733 "response to auxin stimulus" evidence=IEP] InterPro:IPR003311 InterPro:IPR003340 InterPro:IPR010525 InterPro:IPR011525 Pfam:PF02309 Pfam:PF02362 Pfam:PF06507 PROSITE:PS50863 PROSITE:PS50962 SMART:SM01019 EMBL:CP002684 GenomeReviews:CT485782_GR GO:GO:0005634 GO:GO:0045892 GO:GO:0009734 GO:GO:0003677 GO:GO:0006351 GO:GO:0010150 EMBL:AC007258 GO:GO:0046983 InterPro:IPR015300 Gene3D:G3DSA:2.40.330.10 SUPFAM:SSF101936 EMBL:U83245 EMBL:AY133723 EMBL:BT002748 IPI:IPI00518211 IPI:IPI00520405 PIR:D96621 RefSeq:NP_001031208.1 RefSeq:NP_176184.1 RefSeq:NP_849830.1 UniGene:At.74832 ProteinModelPortal:Q8L7G0 SMR:Q8L7G0 IntAct:Q8L7G0 STRING:Q8L7G0 PRIDE:Q8L7G0 EnsemblPlants:AT1G59750.1 EnsemblPlants:AT1G59750.3 GeneID:842268 KEGG:ath:AT1G59750 GeneFarm:3363 TAIR:At1g59750 eggNOG:NOG257828 HOGENOM:HBG746399 InParanoid:Q8L7G0 KO:K14486 OMA:NEFCGMV PhylomeDB:Q8L7G0 ProtClustDB:CLSN2679699 ArrayExpress:Q8L7G0 Genevestigator:Q8L7G0 GermOnline:AT1G59750 Uniprot:Q8L7G0
Root	Isotig06707	69	4	3.980	1.80E-15	TAIR|locus:2155894 - symbol:SUS2 "AT5G49190" species:3702 "Arabidopsis thaliana" [GO:0005986 "sucrose biosynthetic process" evidence=ISS] [GO:0008194 "UDP-glycosyltransferase activity" evidence=ISS] [GO:0016157 "sucrose synthase activity" evidence=ISS;IMP] [GO:0016757 "transferase activity, transferring glycosyl groups" evidence=ISS] [GO:0001666 "response to hypoxia" evidence=IEP] [GO:0009505 "plant-type cell wall" evidence=IDA] [GO:0016020 "membrane" evidence=IDA] [GO:0005829 "cytosol" evidence=IDA] [GO:0005982 "starch metabolic process" evidence=IMP] [GO:0005985 "sucrose metabolic process" evidence=IMP] [GO:0010431 "seed maturation" evidence=IMP] InterPro:IPR000368 InterPro:IPR001296 InterPro:IPR012820 Pfam:PF00534 Pfam:PF00862 GO:GO:0005829 EMBL:CP002688 GenomeReviews:BA000015_GR GO:GO:0016020 GO:GO:0001666 GO:GO:0009058 GO:GO:0009505 eggNOG:COG0438 GO:GO:0010431 CAZy:GT4 EMBL:AB016872 GO:GO:0005985 GO:GO:0005982 HOGENOM:HBG286768 GO:GO:0016157 PANTHER:PTHR12526:SF27 TIGRFAMs:TIGR02470 EMBL:X60987 IPI:IPI00539012 PIR:S19125 RefSeq:NP_199730.1 UniGene:At.8597 ProteinModelPortal:Q00917 STRING:Q00917 PRIDE:Q00917 GeneID:834978 KEGG:ath:AT5G49190 TAIR:At5g49190 InParanoid:Q00917 PhylomeDB:Q00917 ArrayExpress:Q00917 Genevestigator:Q00917 GermOnline:AT5G49190 Uniprot:Q00917

Tissue	Identification	# of reads (stress)	# of reads (control)	Fold Change	p-value	Description
Root	Isotig06708	62	4	3.825	9.77E-14	TAIR|locus:2155894 - symbol:SUS2 "AT5G49190" species:3702 "Arabidopsis thaliana" [GO:0005986 "sucrose biosynthetic process" evidence=ISS] [GO:0008194 "UDP-glycosyltransferase activity" evidence=ISS] [GO:0016157 "sucrose synthase activity" evidence=ISS;IMP] [GO:0016757 "transferase activity, transferring glycosyl groups" evidence=ISS] [GO:0001666 "response to hypoxia" evidence=IEP] [GO:0009505 "plant-type cell wall" evidence=IDA] [GO:0016020 "membrane" evidence=IDA] [GO:0005829 "cytosol" evidence=IDA] [GO:0005982 "starch metabolic process" evidence=IMP] [GO:0005985 "sucrose metabolic process" evidence=IMP] [GO:0010431 "seed maturation" evidence=IMP] InterPro:IPR000368 InterPro:IPR001296 InterPro:IPR012820 Pfam:PF00534 Pfam:PF00862 GO:GO:0005829 EMBL:CP002688 GenomeReviews:BA000015_GR GO:GO:0016020 GO:GO:0001666 GO:GO:0009058 GO:GO:0009505 eggNOG:COG0438 GO:GO:0010431 CAZy:GT4 EMBL:AB016872 GO:GO:0005985 GO:GO:0005982 HOGENOM:HBG286768 GO:GO:0016157 PANTHER:PTHR12526:SF27 TIGRFAMs:TIGR02470 EMBL:X60987 IPI:IPI00539012 PIR:S19125 RefSeq:NP_199730.1 UniGene:At.8597 ProteinModelPortal:Q00917 STRING:Q00917 PRIDE:Q00917 GeneID:834978 KEGG:ath:AT5G49190 TAIR:At5g49190 InParanoid:Q00917 PhylomeDB:Q00917 ArrayExpress:Q00917 Genevestigator:Q00917 GermOnline:AT5G49190 Uniprot:Q00917
Root	Isotig06711	3	32	-3.544	5.33E-08	TAIR|locus:2117343 - symbol:AT4G25160 species:3702 "Arabidopsis thaliana" [GO:0000151 "ubiquitin ligase complex" evidence=IEA] [GO:0006468 "protein phosphorylation" evidence=IEA] [GO:0006950 "response to stress" evidence=IEA] [GO:0016301 "kinase activity" evidence=ISS] [GO:0016567 "protein ubiquitination" evidence=IEA] [GO:0005515 "protein binding" evidence=IPI] InterPro:IPR000719 InterPro:IPR001245 InterPro:IPR003613 InterPro:IPR006016 InterPro:IPR008271 InterPro:IPR011009 InterPro:IPR017441 Pfam:PF00582 Pfam:PF04564 Pfam:PF07714 PROSITE:PS00107 PROSITE:PS00108 PROSITE:PS50011 SMART:SM00504 GO:GO:0005524 EMBL:CP002687 GenomeReviews:CT486007_GR GO:GO:0005515 GO:GO:0006950 eggNOG:COG0515 SUPFAM:SSF56112 GO:GO:0004674 InterPro:IPR013083 Gene3D:G3DSA:3.30.40.10 EMBL:AL161562 GO:GO:0004842 InterPro:IPR014729 Gene3D:G3DSA:3.40.50.620 GO:GO:0000151 EMBL:AL035523 EMBL:AL035396 HSSP:Q8VZ40 HOGENOM:HBG590743 EMBL:BX828107 IPI:IPI00540343 PIR:T05537 RefSeq:NP_194246.2 UniGene:At.32287 ProteinModelPortal:Q9SW11 SMR:Q9SW11 IntAct:Q9SW11 PRIDE:Q9SW11 EnsemblPlants:AT4G25160.1 GeneID:828619 KEGG:ath:AT4G25160 GeneFarm:2015 TAIR:At4g25160 InParanoid:Q9SW11 OMA:FQQELEI PhylomeDB:Q9SW11 ProtClustDB:CLSN2918598 ArrayExpress:Q9SW11 Genevestigator:Q9SW11 Uniprot:Q9SW11
Root	Isotig06712	3	32	-3.544	5.33E-08	TAIR|locus:2117343 - symbol:AT4G25160 species:3702 "Arabidopsis thaliana" [GO:0000151 "ubiquitin ligase complex" evidence=IEA] [GO:0006468 "protein phosphorylation" evidence=IEA] [GO:0006950 "response to stress" evidence=IEA] [GO:0016301 "kinase activity" evidence=ISS] [GO:0016567 "protein ubiquitination" evidence=IEA] [GO:0005515 "protein binding" evidence=IPI] InterPro:IPR000719 InterPro:IPR001245 InterPro:IPR003613 InterPro:IPR006016 InterPro:IPR008271 InterPro:IPR011009 InterPro:IPR017441 Pfam:PF00582 Pfam:PF04564 Pfam:PF07714 PROSITE:PS00107 PROSITE:PS00108 PROSITE:PS50011 SMART:SM00504 GO:GO:0005524 EMBL:CP002687 GenomeReviews:CT486007_GR GO:GO:0005515 GO:GO:0006950 eggNOG:COG0515 SUPFAM:SSF56112 GO:GO:0004674 InterPro:IPR013083 Gene3D:G3DSA:3.30.40.10 EMBL:AL161562 GO:GO:0004842 InterPro:IPR014729 Gene3D:G3DSA:3.40.50.620 GO:GO:0000151 EMBL:AL035523 EMBL:AL035396 HSSP:Q8VZ40 HOGENOM:HBG590743 EMBL:BX828107 IPI:IPI00540343 PIR:T05537 RefSeq:NP_194246.2 UniGene:At.32287 ProteinModelPortal:Q9SW11 SMR:Q9SW11 IntAct:Q9SW11 PRIDE:Q9SW11 EnsemblPlants:AT4G25160.1 GeneID:828619 KEGG:ath:AT4G25160 GeneFarm:2015 TAIR:At4g25160 InParanoid:Q9SW11 OMA:FQQELEI PhylomeDB:Q9SW11 ProtClustDB:CLSN2918598 ArrayExpress:Q9SW11 Genevestigator:Q9SW11 Uniprot:Q9SW11
Root	Isotig06715	31	8	1.825	0.000382406	TAIR|locus:2035893 - symbol:AT1G42440 "AT1G42440" species:3702 "Arabidopsis thaliana" [GO:0003674 "molecular_function" evidence=ND] [GO:0005634 "nucleus" evidence=IEA] [GO:0042254 "ribosome biogenesis" evidence=IEA] InterPro:IPR012948 Pfam:PF08142 SMART:SM00785 EMBL:CP002684 GenomeReviews:CT485782_GR GO:GO:0005634 GO:GO:0042254 InterPro:IPR007034 Pfam:PF04950 eggNOG:COG5177 HOGENOM:HBG330634 KO:K14799 OMA:DAEQTWP EMBL:AF361826 EMBL:AY133551 EMBL:BT000755 IPI:IPI00527453 RefSeq:NP_564480.1 UniGene:At.19120 UniGene:At.67019 ProteinModelPortal:Q9ASU6 IntAct:Q9ASU6 STRING:Q9ASU6 PRIDE:Q9ASU6 EnsemblPlants:AT1G42440.1 GeneID:840848 KEGG:ath:AT1G42440 TAIR:At1g42440 InParanoid:Q9ASU6 PhylomeDB:Q9ASU6 ProtClustDB:CLSN2688391 ArrayExpress:Q9ASU6 Genevestigator:Q9ASU6 Uniprot:Q9ASU6

Tissue	Identification	# of reads (stress)	# of reads (control)	Fold Change	p-value	Description
Root	Isotig06716	31	8	1.825	0.000382406	TAIR|locus:2035893 - symbol:AT1G42440 "AT1G42440" species:3702 "Arabidopsis thaliana" [GO:0003674 "molecular_function" evidence=ND] [GO:0005634 "nucleus" evidence=IEA] [GO:0042254 "ribosome biogenesis" evidence=IEA] InterPro:IPR012948 Pfam:PF08142 SMART:SM00785 EMBL:CP002684 GenomeReviews:CT485782_GR GO:GO:0005634 GO:GO:0042254 InterPro:IPR007034 Pfam:PF04950 eggNOG:COG5177 HOGENOM:HBG330634 KO:K14799 OMA:DAEQTWP EMBL:AF361826 EMBL:AY133551 EMBL:BT000755 IPI:IPI00527453 RefSeq:NP_564480.1 UniGene:At.19120 UniGene:At.67019 ProteinModelPortal:Q9ASU6 IntAct:Q9ASU6 STRING:Q9ASU6 PRIDE:Q9ASU6 EnsemblPlants:AT1G42440.1 GeneID:840848 KEGG:ath:AT1G42440 TAIR:At1g42440 InParanoid:Q9ASU6 PhylomeDB:Q9ASU6 ProtClustDB:CLSN2688391 ArrayExpress:Q9ASU6 Genevestigator:Q9ASU6 Uniprot:Q9ASU6
Root	Isotig06719	162	15	3.304	5.50E-30	No hit
Root	Isotig06720	87	10	2.992	1.60E-15	No hit
Root	Isotig06723	13	34	-1.516	0.00065265	TAIR|locus:2175851 - symbol:HDS "AT5G60600" species:3702 "Arabidopsis thaliana" [GO:0008299 "isoprenoid biosynthetic process" evidence=ISS] [GO:0046429 "4-hydroxy-3-methylbut-2-en-1-yl diphosphate synthase activity" evidence=IGI;ISS;IDA] [GO:0009507 "chloroplast" evidence=IDA] [GO:0009941 "chloroplast envelope" evidence=IDA] [GO:0019288 "isopentenyl diphosphate biosynthetic process, mevalonate-independent pathway" evidence=TAS] [GO:0051539 "4 iron, 4 sulfur cluster binding" evidence=IDA] [GO:0009617 "response to bacterium" evidence=IMP] [GO:0009862 "systemic acquired resistance, salicylic acid mediated signaling pathway" evidence=IMP] [GO:0009570 "chloroplast stroma" evidence=IDA] InterPro:IPR004588 InterPro:IPR017178 Pfam:PF04551 PIRSF:PIRSF037336 GO:GO:0009570 GenomeReviews:BA000015_GR GO:GO:0009617 GO:GO:0005506 GO:GO:0009941 GO:GO:0051539 GO:GO:0009862 GO:GO:0019288 GO:GO:0016114 HOGENOM:HBG335271 GO:GO:0046429 HAMAP:MF_00159 TIGRFAMs:TIGR00612 EMBL:AY094472 IPI:IPI00530209 ProteinModelPortal:Q8LPQ4 STRING:Q8LPQ4 PRIDE:Q8LPQ4 TAIR:At5g60600 InParanoid:Q8LPQ4 PhylomeDB:Q8LPQ4 Genevestigator:Q8LPQ4 Uniprot:Q8LPQ4
Root	Isotig06725	20	2	3.193	8.23E-05	TAIR|locus:2142788 - symbol:DGK1 "AT5G07920" species:3702 "Arabidopsis thaliana" [GO:0004143 "diacylglycerol kinase activity" evidence=ISS;TAS] [GO:0007205 "activation of protein kinase C activity by G-protein coupled receptor protein signaling pathway" evidence=IEA;ISS] [GO:0035556 "intracellular signal transduction" evidence=IEA] [GO:0005509 "calcium ion binding" evidence=ISS] InterPro:IPR000756 InterPro:IPR001206 InterPro:IPR002219 Pfam:PF00130 Pfam:PF00609 Pfam:PF00781 PROSITE:PS00479 PROSITE:PS50081 PROSITE:PS50146 SMART:SM00045 SMART:SM00046 SMART:SM00109 GO:GO:0016021 GO:GO:0005524 EMBL:CP002688 GenomeReviews:BA000015_GR GO:GO:0035556 GO:GO:0046872 GO:GO:0007205 InterPro:IPR013083 Gene3D:G3DSA:3.30.40.10 GO:GO:0004143 EMBL:AL133421 EMBL:AB005249 EMBL:D63787 EMBL:BT004148 IPI:IPI00527797 PIR:S71467 RefSeq:NP_196409.1 UniGene:At.260 ProteinModelPortal:Q39017 SMR:Q39017 STRING:Q39017 PRIDE:Q39017 EnsemblPlants:AT5G07920.1 GeneID:830686 KEGG:ath:AT5G07920 TAIR:At5g07920 eggNOG:NOG47311 HOGENOM:HBG317349 InParanoid:Q39017 KO:K00901 OMA:VDLWQNE PhylomeDB:Q39017 ProtClustDB:CLSN2687236 ArrayExpress:Q39017 Genevestigator:Q39017 GermOnline:AT5G07920 Uniprot:Q39017
Root	Isotig06726	20	2	3.193	8.23E-05	TAIR|locus:2142788 - symbol:DGK1 "AT5G07920" species:3702 "Arabidopsis thaliana" [GO:0004143 "diacylglycerol kinase activity" evidence=ISS;TAS] [GO:0007205 "activation of protein kinase C activity by G-protein coupled receptor protein signaling pathway" evidence=IEA;ISS] [GO:0035556 "intracellular signal transduction" evidence=IEA] [GO:0005509 "calcium ion binding" evidence=ISS] InterPro:IPR000756 InterPro:IPR001206 InterPro:IPR002219 Pfam:PF00130 Pfam:PF00609 Pfam:PF00781 PROSITE:PS00479 PROSITE:PS50081 PROSITE:PS50146 SMART:SM00045 SMART:SM00046 SMART:SM00109 GO:GO:0016021 GO:GO:0005524 EMBL:CP002688 GenomeReviews:BA000015_GR GO:GO:0035556 GO:GO:0046872 GO:GO:0007205 InterPro:IPR013083 Gene3D:G3DSA:3.30.40.10 GO:GO:0004143 EMBL:AL133421 EMBL:AB005249 EMBL:D63787 EMBL:BT004148 IPI:IPI00527797 PIR:S71467 RefSeq:NP_196409.1 UniGene:At.260 ProteinModelPortal:Q39017 SMR:Q39017 STRING:Q39017 PRIDE:Q39017 EnsemblPlants:AT5G07920.1 GeneID:830686 KEGG:ath:AT5G07920 TAIR:At5g07920 eggNOG:NOG47311 HOGENOM:HBG317349 InParanoid:Q39017 KO:K00901 OMA:VDLWQNE PhylomeDB:Q39017 ProtClustDB:CLSN2687236 ArrayExpress:Q39017 Genevestigator:Q39017 GermOnline:AT5G07920 Uniprot:Q39017
Root	Isotig06743	7	33	-2.366	6.09E-06	TAIR|locus:2050669 - symbol:AT2G45910 species:3702 "Arabidopsis thaliana" [GO:0000151 "ubiquitin ligase complex" evidence=IEA] [GO:0004672 "protein kinase activity" evidence=IEA] [GO:0004674 "protein serine/threonine kinase activity" evidence=IEA] [GO:0004842 "ubiquitin-protein ligase activity" evidence=IEA]

Tissue	Identification	# of reads (stress)	# of reads (control)	Fold Change	p-value	Description
						[GO:0005524 "ATP binding" evidence=IEA] [GO:0006468 "protein phosphorylation" evidence=IEA] [GO:0006950 "response to stress" evidence=IEA] [GO:0016301 "kinase activity" evidence=ISS] [GO:0016567 "protein ubiquitination" evidence=IEA] InterPro:IPR000719 InterPro:IPR003613 InterPro:IPR006016 InterPro:IPR008271 InterPro:IPR011009 InterPro:IPR017441 Pfam:PF00069 Pfam:PF00582 Pfam:PF04564 PROSITE:PS00107 PROSITE:PS00108 PROSITE:PS50011 SMART:SM00504 GO:GO:0005524 EMBL:CP002685 GenomeReviews:CT485783_GR GO:GO:0006950 eggNOG:COG0515 SUPFAM:SSF56112 GO:GO:0004674 HOGENOM:HBG755340 InterPro:IPR013083 Gene3D:G3DSA:3.30.40.10 GO:GO:0004842 InterPro:IPR014729 Gene3D:G3DSA:3.40.50.620 GO:GO:0000151 EMBL:AC004665 HSSP:Q8VZ40 EMBL:BT002512 EMBL:AK227123 IPI:IPI00542046 PIR:T02456 RefSeq:NP_182115.2 UniGene:At.36571 ProteinModelPortal:Q8GUH1 SMR:Q8GUH1 PRIDE:Q8GUH1 EnsemblPlants:AT2G45910.1 GeneID:819199 KEGG:ath:AT2G45910 GeneFarm:1984 TAIR:At2g45910 InParanoid:Q8GUH1 OMA:SQMIPLM PhylomeDB:Q8GUH1 ProtClustDB:CLSN2680270 Genevestigator:Q8GUH1 Uniprot:Q8GUH1
Root	Isotig06744	8	33	-2.173	1.74E-05	TAIR|locus:2050669 - symbol:AT2G45910 species:3702 "Arabidopsis thaliana" [GO:0000151 "ubiquitin ligase complex" evidence=IEA] [GO:0004672 "protein kinase activity" evidence=IEA] [GO:0004674 "protein serine/threonine kinase activity" evidence=IEA] [GO:0004842 "ubiquitin-protein ligase activity" evidence=IEA] [GO:0005524 "ATP binding" evidence=IEA] [GO:0006468 "protein phosphorylation" evidence=IEA] [GO:0006950 "response to stress" evidence=IEA] [GO:0016301 "kinase activity" evidence=ISS] [GO:0016567 "protein ubiquitination" evidence=IEA] InterPro:IPR000719 InterPro:IPR003613 InterPro:IPR006016 InterPro:IPR008271 InterPro:IPR011009 InterPro:IPR017441 Pfam:PF00069 Pfam:PF00582 Pfam:PF04564 PROSITE:PS00107 PROSITE:PS00108 PROSITE:PS50011 SMART:SM00504 GO:GO:0005524 EMBL:CP002685 GenomeReviews:CT485783_GR GO:GO:0006950 eggNOG:COG0515 SUPFAM:SSF56112 GO:GO:0004674 HOGENOM:HBG755340 InterPro:IPR013083 Gene3D:G3DSA:3.30.40.10 GO:GO:0004842 InterPro:IPR014729 Gene3D:G3DSA:3.40.50.620 GO:GO:0000151 EMBL:AC004665 HSSP:Q8VZ40 EMBL:BT002512 EMBL:AK227123 IPI:IPI00542046 PIR:T02456 RefSeq:NP_182115.2 UniGene:At.36571 ProteinModelPortal:Q8GUH1 SMR:Q8GUH1 PRIDE:Q8GUH1 EnsemblPlants:AT2G45910.1 GeneID:819199 KEGG:ath:AT2G45910 GeneFarm:1984 TAIR:At2g45910 InParanoid:Q8GUH1 OMA:SQMIPLM PhylomeDB:Q8GUH1 ProtClustDB:CLSN2680270 Genevestigator:Q8GUH1 Uniprot:Q8GUH1
Root	Isotig06745	21	59	-1.619	2.46E-06	TAIR|locus:2198656 - symbol:ATSBT5.2 "AT1G20160" species:3702 "Arabidopsis thaliana" [GO:0004252 "serine-type endopeptidase activity" evidence=IEA;IBA] [GO:0005618 "cell wall" evidence=IBA] [GO:0006508 "proteolysis" evidence=ISS] [GO:0008152 "metabolic process" evidence=IBA] [GO:0042802 "identical protein binding" evidence=IEA] [GO:0048046 "apoplast" evidence=IDA] InterPro:IPR000209 InterPro:IPR010259 Pfam:PF00082 Pfam:PF05922 InterPro:IPR003137 Prosite:PS00137 Prosite:PS00138 PANTHER:PTHR10795 Pfam:PF02225 EMBL:CP002684 GO:GO:0048046 GO:GO:0042802 GO:GO:0006508 GO:GO:0004252 GO:GO:0043086 InterPro:IPR022398 InterPro:IPR023828 InterPro:IPR015500 Gene3D:G3DSA:3.40.50.200 PRINTS:PR00723 SUPFAM:SSF52743 EMBL:AC022472 HSSP:Q45670 EMBL:AK228874 IPI:IPI00518804 PIR:D86335 RefSeq:NP_564107.1 UniGene:At.24841 ProteinModelPortal:Q9LNU1 SMR:Q9LNU1 MEROPS:S08.A22 PRIDE:Q9LNU1 ProMEX:Q9LNU1 EnsemblPlants:AT1G20160.1 GeneID:838606 KEGG:ath:AT1G20160 TAIR:At1g20160 InParanoid:Q9LNU1 OMA:GGNKVIK PhylomeDB:Q9LNU1 ProtClustDB:CLSN2687878 Genevestigator:Q9LNU1 Uniprot:Q9LNU1
Root	Isotig06746	21	59	-1.619	2.46E-06	TAIR|locus:2198656 - symbol:ATSBT5.2 "AT1G20160" species:3702 "Arabidopsis thaliana" [GO:0004252 "serine-type endopeptidase activity" evidence=IEA;IBA] [GO:0005618 "cell wall" evidence=IBA] [GO:0006508 "proteolysis" evidence=ISS] [GO:0008152 "metabolic process" evidence=IBA] [GO:0042802 "identical protein binding" evidence=IEA] [GO:0048046 "apoplast" evidence=IDA] InterPro:IPR000209 InterPro:IPR010259 Pfam:PF00082 Pfam:PF05922 InterPro:IPR003137 Prosite:PS00137 Prosite:PS00138 PANTHER:PTHR10795 Pfam:PF02225 EMBL:CP002684 GO:GO:0048046 GO:GO:0042802 GO:GO:0006508 GO:GO:0004252 GO:GO:0043086 InterPro:IPR022398 InterPro:IPR023828 InterPro:IPR015500 Gene3D:G3DSA:3.40.50.200 PRINTS:PR00723 SUPFAM:SSF52743 EMBL:AC022472 HSSP:Q45670 EMBL:AK228874 IPI:IPI00518804 PIR:D86335 RefSeq:NP_564107.1 UniGene:At.24841 ProteinModelPortal:Q9LNU1 SMR:Q9LNU1 MEROPS:S08.A22 PRIDE:Q9LNU1 ProMEX:Q9LNU1 EnsemblPlants:AT1G20160.1 GeneID:838606 KEGG:ath:AT1G20160 TAIR:At1g20160 InParanoid:Q9LNU1 OMA:GGNKVIK PhylomeDB:Q9LNU1 ProtClustDB:CLSN2687878 Genevestigator:Q9LNU1 Uniprot:Q9LNU1

Tissue	Identification	# of reads (stress)	# of reads (control)	Fold Change	p-value	Description
Root	Isotig06753	18	2	3.041	0.000259481	TAIR|locus:2125541 - symbol:AT4G03200 species:3702 "Arabidopsis thaliana" [GO:0003824 "catalytic activity" evidence=IEA] [GO:0009507 "chloroplast" evidence=IDA] InterPro:IPR008928 InterPro:IPR012341 GenomeReviews:CT486007_GR GO:GO:0009507 GO:GO:0003824 Gene3D:G3DSA:1.50.10.10 SUPFAM:SSF48208 InterPro:IPR012336 Gene3D:G3DSA:3.40.30.10 SUPFAM:SSF52833 InterPro:IPR004879 InterPro:IPR024705 Pfam:PF03190 PIRSF:PIRSF006402 HOGENOM:HBG756603 EMBL:AY062530 EMBL:BT010343 IPI:IPI00528599 ProteinModelPortal:Q8W4J1 SMR:Q8W4J1 STRING:Q8W4J1 PRIDE:Q8W4J1 TAIR:At4g03200 InParanoid:Q8W4J1 PhylomeDB:Q8W4J1 Genevestigator:Q8W4J1 Uniprot:Q8W4J1
Root	Isotig06754	18	2	3.041	0.000259481	TAIR|locus:2125541 - symbol:AT4G03200 species:3702 "Arabidopsis thaliana" [GO:0003824 "catalytic activity" evidence=IEA] [GO:0009507 "chloroplast" evidence=IDA] InterPro:IPR008928 InterPro:IPR012341 GenomeReviews:CT486007_GR GO:GO:0009507 GO:GO:0003824 Gene3D:G3DSA:1.50.10.10 SUPFAM:SSF48208 InterPro:IPR012336 Gene3D:G3DSA:3.40.30.10 SUPFAM:SSF52833 InterPro:IPR004879 InterPro:IPR024705 Pfam:PF03190 PIRSF:PIRSF006402 HOGENOM:HBG756603 EMBL:AY062530 EMBL:BT010343 IPI:IPI00528599 ProteinModelPortal:Q8W4J1 SMR:Q8W4J1 STRING:Q8W4J1 PRIDE:Q8W4J1 TAIR:At4g03200 InParanoid:Q8W4J1 PhylomeDB:Q8W4J1 Genevestigator:Q8W4J1 Uniprot:Q8W4J1
Root	Isotig06757	23	5	2.073	0.000873448	TAIR|locus:2152965 - symbol:AT5G41770 species:3702 "Arabidopsis thaliana" [GO:0005622 "intracellular" evidence=IEA;ISS] [GO:0006396 "RNA processing" evidence=IEA;ISS] InterPro:IPR003107 InterPro:IPR011990 InterPro:IPR013026 Pfam:PF02184 PROSITE:PS50293 SMART:SM00386 GenomeReviews:BA000015_GR GO:GO:0005488 Gene3D:G3DSA:1.25.40.10 GO:GO:0006396 GO:GO:0005622 eggNOG:NOG327505 HOGENOM:HBG611628 EMBL:BT002040 IPI:IPI00530971 ProteinModelPortal:Q8GTX9 SMR:Q8GTX9 IntAct:Q8GTX9 STRING:Q8GTX9 PRIDE:Q8GTX9 TAIR:At5g41770 InParanoid:Q8GTX9 PhylomeDB:Q8GTX9 ArrayExpress:Q8GTX9 Genevestigator:Q8GTX9 Uniprot:Q8GTX9
Root	Isotig06758	23	5	2.073	0.000873448	TAIR|locus:2152965 - symbol:AT5G41770 species:3702 "Arabidopsis thaliana" [GO:0005622 "intracellular" evidence=IEA;ISS] [GO:0006396 "RNA processing" evidence=IEA;ISS] InterPro:IPR003107 InterPro:IPR011990 InterPro:IPR013026 Pfam:PF02184 PROSITE:PS50293 SMART:SM00386 GenomeReviews:BA000015_GR GO:GO:0005488 Gene3D:G3DSA:1.25.40.10 GO:GO:0006396 GO:GO:0005622 eggNOG:NOG327505 HOGENOM:HBG611628 EMBL:BT002040 IPI:IPI00530971 ProteinModelPortal:Q8GTX9 SMR:Q8GTX9 IntAct:Q8GTX9 STRING:Q8GTX9 PRIDE:Q8GTX9 TAIR:At5g41770 InParanoid:Q8GTX9 PhylomeDB:Q8GTX9 ArrayExpress:Q8GTX9 Genevestigator:Q8GTX9 Uniprot:Q8GTX9
Root	Isotig06761	25	0	5.515	3.68E-07	No hit
Root	Isotig06762	25	0	5.515	3.68E-07	No hit
Root	Isotig06765	1	24	-4.714	3.11E-07	TIGR_CMR|GSU_0686 - symbol:GSU_0686 "deoxyxylulose-5-phosphate synthase" species:243231 "Geobacter sulfurreducens PCA" [GO:0008299 "isoprenoid biosynthetic process" evidence=ISS] [GO:0008615 "pyridoxine biosynthetic process" evidence=ISS] [GO:0008661 "1-deoxy-D-xylulose-5-phosphate synthase activity" evidence=ISS] [GO:0009228 "thiamine biosynthetic process" evidence=ISS] HAMAP:MF_00315 InterPro:IPR005476 InterPro:IPR005477 InterPro:IPR009014 InterPro:IPR015941 Pfam:PF02780 InterPro:IPR005475 Pfam:PF02779 InterPro:IPR005474 EMBL:AE017180 GenomeReviews:AE017180_GR GO:GO:0009228 SMART:SM00861 RefSeq:NP_951743.1 ProteinModelPortal:Q74FC3 GeneID:2685342 KEGG:gsu:GSU0686 PATRIC:22024127 TIGR:GSU0686 eggNOG:COG1154 HOGENOM:HBG571647 KO:K01662 OMA:DPILYHG PhylomeDB:Q74FC3 ProtClustDB:PRK05444 BioCyc:GSUL243231:GSU_0686-MONOMER GO:GO:0008661 GO:GO:0016114 InterPro:IPR020826 Gene3D:G3DSA:3.40.50.920 SUPFAM:SSF52922 TIGRFAMs:TIGR00204 PROSITE:PS00801 PROSITE:PS00802 Uniprot:Q74FC3
Root	Isotig06766	1	19	-4.377	7.43E-06	TAIR|locus:2130374 - symbol:CLA1 "AT4G15560" species:3702 "Arabidopsis thaliana" [GO:0008661 "1-deoxy-D-xylulose-5-phosphate synthase activity" evidence=IDA] [GO:0015995 "chlorophyll biosynthetic process" evidence=IMP] [GO:0019288 "isopentenyl diphosphate biosynthetic process, mevalonate-independent pathway" evidence=TAS] [GO:0009507 "chloroplast" evidence=IDA] InterPro:IPR005476 InterPro:IPR005477 InterPro:IPR009014 InterPro:IPR015941 Pfam:PF02780 InterPro:IPR005475 Pfam:PF02779 InterPro:IPR005474 EMBL:CP002687 GenomeReviews:CT486007_GR GO:GO:0009507 GO:GO:0015995 GO:GO:0009228 EMBL:AL161542 EMBL:Z97339 SMART:SM00861 GO:GO:0019288 eggNOG:COG1154 HOGENOM:HBG571647 KO:K01662 OMA:DPILYHG GO:GO:0008661 GO:GO:0016114 InterPro:IPR020826 Gene3D:G3DSA:3.40.50.920 SUPFAM:SSF52922 TIGRFAMs:TIGR00204 PROSITE:PS00801 PROSITE:PS00802 EMBL:U27099 EMBL:Y14333 EMBL:BT002340 IPI:IPI00516684 PIR:H85171 PIR:T52289 RefSeq:NP_193291.1 UniGene:At.23240 ProteinModelPortal:Q38854 SMR:Q38854 STRING:Q38854 PRIDE:Q38854 EnsemblPlants:AT4G15560.1

Tissue	Identification	# of reads (stress)	# of reads (control)	Fold Change	p-value	Description
						GeneID:827230 KEGG:ath:AT4G15560 TAIR:At4g15560 InParanoid:Q38854 PhylomeDB:Q38854 ProtClustDB:PLN02582 BioCyc:ARA:AT4G15560-MONOMER BioCyc:MetaCyc:AT4G15560-MONOMER Genevestigator:Q38854 GermOnline:AT4G15560 Uniprot:Q38854
Root	Isotig06771	5	24	-2.392	0.000103464	TAIR|locus:2007760 - symbol:AGO1 "AT1G48410" species:3702 "Arabidopsis thaliana" [GO:0016441 "posttranscriptional gene silencing" evidence=IEP] [GO:0035195 "gene silencing by miRNA" evidence=IGI;IEP;IMP] [GO:0009733 "response to auxin stimulus" evidence=IGI] [GO:0009850 "auxin metabolic process" evidence=IGI] [GO:0010218 "response to far red light" evidence=IGI] [GO:0048830 "adventitious root development" evidence=IMP] [GO:0004521 "endoribonuclease activity" evidence=IDA] [GO:0035197 "siRNA binding" evidence=IPI] [GO:0035198 "miRNA binding" evidence=IPI] [GO:0009616 "virus induced gene silencing" evidence=IDA] [GO:0005634 "nucleus" evidence=IDA] [GO:0005737 "cytoplasm" evidence=IDA;TAS] [GO:0016246 "RNA interference" evidence=IMP] [GO:0005515 "protein binding" evidence=IPI] [GO:0005829 "cytosol" evidence=IDA] [GO:0048864 "stem cell development" evidence=IGI] [GO:0045087 "innate immune response" evidence=IMP] [GO:0000300 "peripheral to membrane of membrane fraction" evidence=IDA] [GO:0009965 "leaf morphogenesis" evidence=IMP] [GO:0009793 "embryo development ending in seed dormancy" evidence=IGI] InterPro:IPR003100 InterPro:IPR003165 InterPro:IPR012337 Pfam:PF02170 Pfam:PF02171 PROSITE:PS50821 PROSITE:PS50822 SMART:SM00949 SMART:SM00950 EMBL:CP002684 GenomeReviews:CT485782_GR GO:GO:0005829 GO:GO:0005634 GO:GO:0005515 GO:GO:0009733 GO:GO:0006417 GO:GO:0016246 GO:GO:0006355 GO:GO:0006351 GO:GO:0010218 GO:GO:0009793 GO:GO:0030529 GO:GO:0009850 SUPFAM:SSF53098 GO:GO:0009965 eggNOG:NOG279895 HOGENOM:HBG717005 KO:K11596 GO:GO:0035198 InterPro:IPR014811 Pfam:PF08699 SUPFAM:SSF101690 EMBL:U91995 EMBL:AC007932 EMBL:AC020889 EMBL:BT000941 EMBL:AK227868 IPI:IPI00522985 IPI:IPI00523962 RefSeq:NP_001185169.1 RefSeq:NP_175274.1 RefSeq:NP_849784.1 UniGene:At.21124 ProteinModelPortal:O04379 SMR:O04379 STRING:O04379 PRIDE:O04379 EnsemblPlants:AT1G48410.1 GeneID:841262 KEGG:ath:AT1G48410 TAIR:At1g48410 InParanoid:O04379 OMA:RANHFQI PhylomeDB:O04379 ProtClustDB:CLSN2693050 ArrayExpress:O04379 Genevestigator:O04379 GO:GO:0004521 GO:GO:0035197 GO:GO:0048830 GO:GO:0035195 GO:GO:0048864 GO:GO:0009616 InterPro:IPR024357 Pfam:PF12764 Uniprot:O04379
Root	Isotig06772	2	23	-3.653	3.09E-06	TAIR|locus:2007760 - symbol:AGO1 "AT1G48410" species:3702 "Arabidopsis thaliana" [GO:0016441 "posttranscriptional gene silencing" evidence=IEP] [GO:0035195 "gene silencing by miRNA" evidence=IGI;IEP;IMP] [GO:0009733 "response to auxin stimulus" evidence=IGI] [GO:0009850 "auxin metabolic process" evidence=IGI] [GO:0010218 "response to far red light" evidence=IGI] [GO:0048830 "adventitious root development" evidence=IMP] [GO:0004521 "endoribonuclease activity" evidence=IDA] [GO:0035197 "siRNA binding" evidence=IPI] [GO:0035198 "miRNA binding" evidence=IPI] [GO:0009616 "virus induced gene silencing" evidence=IDA] [GO:0005634 "nucleus" evidence=IDA] [GO:0005737 "cytoplasm" evidence=IDA;TAS] [GO:0016246 "RNA interference" evidence=IMP] [GO:0005515 "protein binding" evidence=IPI] [GO:0005829 "cytosol" evidence=IDA] [GO:0048864 "stem cell development" evidence=IGI] [GO:0045087 "innate immune response" evidence=IMP] [GO:0000300 "peripheral to membrane of membrane fraction" evidence=IDA] [GO:0009965 "leaf morphogenesis" evidence=IMP] [GO:0009793 "embryo development ending in seed dormancy" evidence=IGI] InterPro:IPR003100 InterPro:IPR003165 InterPro:IPR012337 Pfam:PF02170 Pfam:PF02171 PROSITE:PS50821 PROSITE:PS50822 SMART:SM00949 SMART:SM00950 EMBL:CP002684 GenomeReviews:CT485782_GR GO:GO:0005829 GO:GO:0005634 GO:GO:0005515 GO:GO:0009733 GO:GO:0006417 GO:GO:0016246 GO:GO:0006355 GO:GO:0006351 GO:GO:0010218 GO:GO:0009793 GO:GO:0030529 GO:GO:0009850 SUPFAM:SSF53098 GO:GO:0009965 eggNOG:NOG279895 HOGENOM:HBG717005 KO:K11596 GO:GO:0035198 InterPro:IPR014811 Pfam:PF08699 SUPFAM:SSF101690 EMBL:U91995 EMBL:AC007932 EMBL:AC020889 EMBL:BT000941 EMBL:AK227868 IPI:IPI00522985 IPI:IPI00523962 RefSeq:NP_001185169.1 RefSeq:NP_175274.1 RefSeq:NP_849784.1 UniGene:At.21124 ProteinModelPortal:O04379 SMR:O04379 STRING:O04379 PRIDE:O04379 EnsemblPlants:AT1G48410.1 GeneID:841262 KEGG:ath:AT1G48410 TAIR:At1g48410 InParanoid:O04379 OMA:RANHFQI PhylomeDB:O04379 ProtClustDB:CLSN2693050 ArrayExpress:O04379 Genevestigator:O04379 GO:GO:0004521 GO:GO:0035197 GO:GO:0048830 GO:GO:0035195 GO:GO:0048864 GO:GO:0009616 InterPro:IPR024357 Pfam:PF12764 Uniprot:O04379
Root	Isotig06782	5	22	-2.266	0.000318925	TAIR|locus:2199512 - symbol:ATG18H "AT1G54710" species:3702 "Arabidopsis thaliana" [GO:0003674 "molecular_function" evidence=ND] [GO:0042594 "response to starvation" evidence=IEP] InterPro:IPR017986 InterPro:IPR001680 InterPro:IPR015943 Pfam:PF00400 SMART:SM00320 EMBL:CP002684

Tissue	Identification	# of reads (stress)	# of reads (control)	Fold Change	p-value	Description
						Gene3D:G3DSA:2.130.10.10 SUPFAM:SSF50978 GO:GO:0042594 eggNOG:NOG321894 InterPro:IPR022175 Pfam:PF12490 ProtClustDB:CLSN2686780 EMBL:AY142626 IPI:IPI00534724 RefSeq:NP_564664.1 UniGene:At.21107 UniGene:At.52203 UniGene:At.64867 ProteinModelPortal:Q8H1Q5 SMR:Q8H1Q5 PRIDE:Q8H1Q5 EnsemblPlants:AT1G54710.1 GeneID:841912 KEGG:ath:AT1G54710 TAIR:At1g54710 InParanoid:Q8H1Q5 OMA:HRGMTSA PhylomeDB:Q8H1Q5 Genevestigator:Q8H1Q5 Uniprot:Q8H1Q5
Root	Isotig06787	44	96	-1.255	7.06E-07	TAIR|locus:2057981 - symbol:PAL1 "PHE ammonia lyase 1" species:3702 "Arabidopsis thaliana" [GO:0005737 "cytoplasm" evidence=IEA] [GO:0009699 "phenylpropanoid biosynthetic process" evidence=TAS] [GO:0045548 "phenylalanine ammonia-lyase activity" evidence=ISS;IDA;TAS] [GO:0006979 "response to oxidative stress" evidence=IEP] [GO:0080167 "response to karrikin" evidence=IEP] [GO:0009555 "pollen development" evidence=IMP] [GO:0009819 "drought recovery" evidence=IMP] [GO:0010224 "response to UV-B" evidence=IMP] [GO:0046244 "salicylic acid catabolic process" evidence=IMP] [GO:0046274 "lignin catabolic process" evidence=IMP] [GO:0006952 "defense response" evidence=TAS] [GO:0009611 "response to wounding" evidence=TAS] InterPro:IPR001106 InterPro:IPR005922 InterPro:IPR008948 InterPro:IPR022313 Pfam:PF00221 PROSITE:PS00488 GO:GO:0005737 EMBL:CP002685 GenomeReviews:CT485783_GR GO:GO:0006979 GO:GO:0009058 GO:GO:0080167 EMBL:AC006922 GO:GO:0009555 SUPFAM:SSF48557 GO:GO:0006559 InterPro:IPR024083 Gene3D:G3DSA:1.10.275.10 GO:GO:0010224 eggNOG:COG2986 GO:GO:0046274 EMBL:L33677 EMBL:AY303128 EMBL:AY045919 EMBL:AY079363 EMBL:BT003330 EMBL:X62747 IPI:IPI00519512 PIR:G84787 PIR:S52990 RefSeq:NP_181241.1 UniGene:At.21614 UniGene:At.22705 ProteinModelPortal:P35510 SMR:P35510 IntAct:P35510 STRING:P35510 PRIDE:P35510 EnsemblPlants:AT2G37040.1 GeneID:818280 KEGG:ath:AT2G37040 TAIR:At2g37040 HOGENOM:HBG318028 InParanoid:P35510 KO:K10775 OMA:QRGADFL PhylomeDB:P35510 ProtClustDB:PLN02457 BRENDA:4.3.1.24 Genevestigator:P35510 GermOnline:AT2G37040 GO:GO:0045548 GO:GO:0009819 GO:GO:0046244 InterPro:IPR023144 Gene3D:G3DSA:1.10.274.20 TIGRFAMs:TIGR01226 Uniprot:P35510
Root	Isotig06796	9	39	-2.244	1.90E-06	TAIR|locus:2012763 - symbol:ADL6 "AT1G10290" species:3702 "Arabidopsis thaliana" [GO:0003924 "GTPase activity" evidence=ISS] [GO:0005794 "Golgi apparatus" evidence=IDA] [GO:0006896 "Golgi to vacuole transport" evidence=IMP] [GO:0005886 "plasma membrane" evidence=IDA] [GO:0005774 "vacuolar membrane" evidence=IDA] [GO:0016020 "membrane" evidence=IDA] [GO:0009506 "plasmodesma" evidence=IDA] Pfam:PF00169 InterPro:IPR000375 InterPro:IPR001401 InterPro:IPR001849 InterPro:IPR003130 Pfam:PF00350 Pfam:PF01031 Pfam:PF02212 PROSITE:PS50003 SMART:SM00053 SMART:SM00233 GO:GO:0005525 EMBL:CP002684 GenomeReviews:CT485782_GR GO:GO:0005829 GO:GO:0005886 GO:GO:0009506 GO:GO:0005774 GO:GO:0000139 InterPro:IPR011993 Gene3D:G3DSA:2.30.29.30 GO:GO:0003924 GO:GO:0008289 GO:GO:0005874 GO:GO:0006896 eggNOG:COG0699 InterPro:IPR022812 PRINTS:PR00195 PROSITE:PS00410 EMBL:AC005489 GO:GO:0009524 InterPro:IPR020850 PROSITE:PS51388 InterPro:IPR019762 EMBL:AF180732 EMBL:AK221193 EMBL:AK228995 IPI:IPI00539942 PIR:B86237 RefSeq:NP_172500.1 UniGene:At.11881 ProteinModelPortal:Q9SE83 SMR:Q9SE83 STRING:Q9SE83 PRIDE:Q9SE83 EnsemblPlants:AT1G10290.1 GeneID:837568 KEGG:ath:AT1G10290 GeneFarm:4740 TAIR:At1g10290 HOGENOM:HBG593279 InParanoid:Q9SE83 OMA:ESTRTIG PhylomeDB:Q9SE83 ProtClustDB:CLSN2679567 BRENDA:3.6.5.5 ArrayExpress:Q9SE83 Genevestigator:Q9SE83 GermOnline:AT1G10290 Uniprot:Q9SE83
Root	Isotig06805	1	30	-5.036	7.50E-09	TAIR|locus:2019693 - symbol:ABCG39 "AT1G66950" species:3702 "Arabidopsis thaliana" [GO:0042626 "ATPase activity, coupled to transmembrane movement of substances" evidence=ISS] [GO:0006855 "drug transmembrane transport" evidence=ISS;IMP] [GO:0000302 "response to reactive oxygen species" evidence=IEP] [GO:0005886 "plasma membrane" evidence=IDA] InterPro:IPR003439 InterPro:IPR003593 InterPro:IPR013525 Pfam:PF00005 Pfam:PF01061 PROSITE:PS00211 PROSITE:PS50893 SMART:SM00382 GO:GO:0016021 EMBL:CP002684 GenomeReviews:CT485782_GR GO:GO:0005886 GO:GO:0005524 eggNOG:COG1131 GO:GO:0016887 HOGENOM:HBG750535 InterPro:IPR013581 Pfam:PF08370 ProtClustDB:CLSN2690483 EMBL:AC007152 EMBL:AC083891 EMBL:BK001010 IPI:IPI00531524 PIR:D96693 RefSeq:NP_176867.2 UniGene:At.35712 ProteinModelPortal:Q7PC84 SMR:Q7PC84 PRIDE:Q7PC84 EnsemblPlants:AT1G66950.1 GeneID:843013 KEGG:ath:AT1G66950 TAIR:At1g66950 InParanoid:Q7PC84 OMA:AKFLWFY PhylomeDB:Q7PC84 ArrayExpress:Q7PC84 Genevestigator:Q7PC84 GermOnline:AT1G66950 GO:GO:0006855 GO:GO:0000302 Uniprot:Q7PC84

Tissue	Identification	# of reads (stress)	# of reads (control)	Fold Change	p-value	Description
Root	Isotig06806	1	26	-4.829	8.91E-08	TAIR|locus:2019693 - symbol:ABCG39 "AT1G66950" species:3702 "Arabidopsis thaliana" [GO:0042626 "ATPase activity, coupled to transmembrane movement of substances" evidence=ISS] [GO:0006855 "drug transmembrane transport" evidence=ISS;IMP] [GO:0000302 "response to reactive oxygen species" evidence=IEP] [GO:0005886 "plasma membrane" evidence=IDA] InterPro:IPR003439 InterPro:IPR003593 InterPro:IPR013525 Pfam:PF00005 Pfam:PF01061 PROSITE:PS00211 PROSITE:PS50893 SMART:SM00382 GO:GO:0016021 EMBL:CP002684 GenomeReviews:CT485782_GR GO:GO:0005886 GO:GO:0005524 eggNOG:COG1131 GO:GO:0016887 HOGENOM:HBG750535 InterPro:IPR013581 Pfam:PF08370 ProtClustDB:CLSN2690483 EMBL:AC007152 EMBL:AC083891 EMBL:BK001010 IPI:IPI00531524 PIR:D96693 RefSeq:NP_176867.2 UniGene:At.35712 ProteinModelPortal:Q7PC84 SMR:Q7PC84 PRIDE:Q7PC84 EnsemblPlants:AT1G66950.1 GeneID:843013 KEGG:ath:AT1G66950 TAIR:At1g66950 InParanoid:Q7PC84 OMA:AKFLWFY PhylomeDB:Q7PC84 ArrayExpress:Q7PC84 Genevestigator:Q7PC84 GermOnline:AT1G66950 GO:GO:0006855 GO:GO:0000302 Uniprot:Q7PC84
Root	Isotig06813	3	28	-3.351	6.51E-07	TAIR|locus:2145643 - symbol:RGLG2 "AT5G14420" species:3702 "Arabidopsis thaliana" [GO:0004842 "ubiquitin-protein ligase activity" evidence=IDA] [GO:0005886 "plasma membrane" evidence=IDA] [GO:0009690 "cytokinin metabolic process" evidence=IGI] [GO:0009850 "auxin metabolic process" evidence=IGI] [GO:0005634 "nucleus" evidence=IDA] [GO:0080148 "negative regulation of response to water deprivation" evidence=IGI] InterPro:IPR001841 InterPro:IPR002035 PROSITE:PS50089 PROSITE:PS50234 SMART:SM00184 SMART:SM00327 Prosite:PS00518 GO:GO:0005886 GO:GO:0005634 EMBL:CP002688 GenomeReviews:BA000015_GR GO:GO:0008270 InterPro:IPR013083 Gene3D:G3DSA:3.30.40.10 GO:GO:0009850 EMBL:AL163817 GO:GO:0004842 InterPro:IPR010734 Pfam:PF07002 GO:GO:0009690 GO:GO:0080148 eggNOG:NOG278119 HOGENOM:HBG746179 EMBL:DQ086862 EMBL:AY099597 EMBL:BT000249 IPI:IPI00535253 PIR:T48615 RefSeq:NP_196946.1 RefSeq:NP_850818.1 RefSeq:NP_974779.1 RefSeq:NP_974780.1 UniGene:At.5177 ProteinModelPortal:Q9LY87 SMR:Q9LY87 STRING:Q9LY87 PRIDE:Q9LY87 EnsemblPlants:AT5G14420.1 EnsemblPlants:AT5G14420.2 EnsemblPlants:AT5G14420.3 EnsemblPlants:AT5G14420.4 GeneID:831293 KEGG:ath:AT5G14420 TAIR:At5g14420 InParanoid:Q9LY87 OMA:EMATTIV PhylomeDB:Q9LY87 ProtClustDB:CLSN2687222 ArrayExpress:Q9LY87 Genevestigator:Q9LY87 Uniprot:Q9LY87
Root	Isotig06817	13	41	-1.786	2.57E-05	TAIR|locus:2005496 - symbol:AUX1 "AT2G38120" species:3702 "Arabidopsis thaliana" [GO:0005215 "transporter activity" evidence=ISS] [GO:0006865 "amino acid transport" evidence=ISS] [GO:0009723 "response to ethylene stimulus" evidence=NAS] [GO:0009733 "response to auxin stimulus" evidence=NAS] [GO:0015171 "amino acid transmembrane transporter activity" evidence=ISS] [GO:0016020 "membrane" evidence=ISS] [GO:0009986 "cell surface" evidence=IDA] [GO:0009624 "response to nematode" evidence=IEP] [GO:0010328 "auxin influx transmembrane transporter activity" evidence=IDA] [GO:0005886 "plasma membrane" evidence=IDA] [GO:0009926 "auxin polar transport" evidence=TAS] [GO:0001736 "establishment of planar polarity" evidence=IGI] [GO:0048765 "root hair cell differentiation" evidence=IGI] [GO:0005768 "endosome" evidence=IDA] [GO:0005794 "Golgi apparatus" evidence=IDA] [GO:0010311 "lateral root formation" evidence=IGI;IMP] [GO:0010011 "auxin binding" evidence=IDA] [GO:0048829 "root cap development" evidence=IMP] [GO:0009958 "positive gravitropism" evidence=IMP] GO:GO:0016021 GO:GO:0005886 EMBL:CP002685 GenomeReviews:CT485783_GR GO:GO:0009986 GO:GO:0009734 GO:GO:0009926 GO:GO:0005794 GO:GO:0005768 GO:GO:0015293 GO:GO:0009624 InterPro:IPR013057 Pfam:PF01490 GO:GO:0006865 GO:GO:0009958 GO:GO:0010011 GO:GO:0010311 GO:GO:0048829 EMBL:AC003028 EMBL:X98772 EMBL:AY054488 EMBL:AY093300 EMBL:AY087092 IPI:IPI00525677 PIR:T01244 RefSeq:NP_565882.1 UniGene:At.24295 ProteinModelPortal:Q96247 STRING:Q96247 TCDB:2.A.18.1.1 PRIDE:Q96247 EnsemblPlants:AT2G38120.1 GeneID:818390 KEGG:ath:AT2G38120 TAIR:At2g38120 eggNOG:NOG319329 HOGENOM:HBG320564 InParanoid:Q96247 KO:K13946 OMA:ASARMNA PhylomeDB:Q96247 ProtClustDB:PLN03074 ArrayExpress:Q96247 Genevestigator:Q96247 GermOnline:AT2G38120 GO:GO:0010328 Uniprot:Q96247
Root	Isotig06818	8	33	-2.173	1.74E-05	TAIR|locus:2005496 - symbol:AUX1 "AT2G38120" species:3702 "Arabidopsis thaliana" [GO:0005215 "transporter activity" evidence=ISS] [GO:0006865 "amino acid transport" evidence=ISS] [GO:0009723 "response to ethylene stimulus" evidence=NAS] [GO:0009733 "response to auxin stimulus" evidence=NAS] [GO:0015171 "amino acid transmembrane transporter activity" evidence=ISS] [GO:0016020 "membrane" evidence=ISS] [GO:0009986 "cell surface" evidence=IDA] [GO:0009624 "response to nematode" evidence=IEP] [GO:0010328 "auxin influx transmembrane transporter activity" evidence=IDA] [GO:0005886 "plasma membrane" evidence=IDA] [GO:0009926

Tissue	Identification	# of reads (stress)	# of reads (control)	Fold Change	p-value	Description
						"auxin polar transport" evidence=TAS] [GO:0001736 "establishment of planar polarity" evidence=IGI] [GO:0048765 "root hair cell differentiation" evidence=IGI] [GO:0005768 "endosome" evidence=IDA] [GO:0005794 "Golgi apparatus" evidence=IDA] [GO:0010311 "lateral root formation" evidence=IGI;IMP] [GO:0010011 "auxin binding" evidence=IDA] [GO:0048829 "root cap development" evidence=IMP] [GO:0009958 "positive gravitropism" evidence=IMP] GO:GO:0016021 GO:GO:0005886 EMBL:CP002685 GenomeReviews:CT485783_GR GO:GO:0009986 GO:GO:0009734 GO:GO:0009926 GO:GO:0005794 GO:GO:0005768 GO:GO:0015293 GO:GO:0009624 InterPro:IPR013057 Pfam:PF01490 GO:GO:0006865 GO:GO:0009958 GO:GO:0010011 GO:GO:0010311 GO:GO:0048829 EMBL:AC003028 EMBL:X98772 EMBL:AY054488 EMBL:AY093300 EMBL:AY087092 IPI:IPI00525677 PIR:T01244 RefSeq:NP_565882.1 UniGene:At.24295 ProteinModelPortal:Q96247 STRING:Q96247 TCDB:2.A.18.1.1 PRIDE:Q96247 EnsemblPlants:AT2G38120.1 GeneID:818390 KEGG:ath:AT2G38120 TAIR:At2g38120 eggNOG:NOG319329 HOGENOM:HBG320564 InParanoid:Q96247 KO:K13946 OMA:ASARMNA PhylomeDB:Q96247 ProtClustDB:PLN03074 ArrayExpress:Q96247 Genevestigator:Q96247 GermOnline:AT2G38120 GO:GO:0010328 Uniprot:Q96247
Root	Isotig06821	66	0	6.915	4.37E-16	TAIR|locus:2084756 - symbol:SUS4 "AT3G43190" species:3702 "Arabidopsis thaliana" [GO:0005986 "sucrose biosynthetic process" evidence=ISS] [GO:0008194 "UDP-glycosyltransferase activity" evidence=ISS] [GO:0016157 "sucrose synthase activity" evidence=IGI;ISS;IDA] [GO:0016757 "transferase activity, transferring glycosyl groups" evidence=ISS] [GO:0005773 "vacuole" evidence=IDA] [GO:0005886 "plasma membrane" evidence=IDA] [GO:0016020 "membrane" evidence=IDA] [GO:0005829 "cytosol" evidence=IDA] InterPro:IPR000368 InterPro:IPR001296 InterPro:IPR012820 Pfam:PF00534 Pfam:PF00862 GO:GO:0005829 GO:GO:0005886 GO:GO:0005773 EMBL:CP002686 GO:GO:0009058 CAZy:GT4 EMBL:AL353871 GO:GO:0005985 KO:K00695 ProtClustDB:PLN00142 GO:GO:0016157 PANTHER:PTHR12526:SF27 TIGRFAMs:TIGR02470 IPI:IPI00540190 PIR:T49233 RefSeq:NP_566865.2 UniGene:At.1720 ProteinModelPortal:Q9LXL5 SMR:Q9LXL5 STRING:Q9LXL5 PRIDE:Q9LXL5 EnsemblPlants:AT3G43190.1 GeneID:823393 KEGG:ath:AT3G43190 TAIR:At3g43190 InParanoid:Q9LXL5 OMA:KAEEYLM PhylomeDB:Q9LXL5 Genevestigator:Q9LXL5 Uniprot:Q9LXL5
Root	Isotig06822	62	0	6.825	2.85E-15	TAIR|locus:2084756 - symbol:SUS4 "AT3G43190" species:3702 "Arabidopsis thaliana" [GO:0005986 "sucrose biosynthetic process" evidence=ISS] [GO:0008194 "UDP-glycosyltransferase activity" evidence=ISS] [GO:0016157 "sucrose synthase activity" evidence=IGI;ISS;IDA] [GO:0016757 "transferase activity, transferring glycosyl groups" evidence=ISS] [GO:0005773 "vacuole" evidence=IDA] [GO:0005886 "plasma membrane" evidence=IDA] [GO:0016020 "membrane" evidence=IDA] [GO:0005829 "cytosol" evidence=IDA] InterPro:IPR000368 InterPro:IPR001296 InterPro:IPR012820 Pfam:PF00534 Pfam:PF00862 GO:GO:0005829 GO:GO:0005886 GO:GO:0005773 EMBL:CP002686 GO:GO:0009058 CAZy:GT4 EMBL:AL353871 GO:GO:0005985 KO:K00695 ProtClustDB:PLN00142 GO:GO:0016157 PANTHER:PTHR12526:SF27 TIGRFAMs:TIGR02470 IPI:IPI00540190 PIR:T49233 RefSeq:NP_566865.2 UniGene:At.1720 ProteinModelPortal:Q9LXL5 SMR:Q9LXL5 STRING:Q9LXL5 PRIDE:Q9LXL5 EnsemblPlants:AT3G43190.1 GeneID:823393 KEGG:ath:AT3G43190 TAIR:At3g43190 InParanoid:Q9LXL5 OMA:KAEEYLM PhylomeDB:Q9LXL5 Genevestigator:Q9LXL5 Uniprot:Q9LXL5
Root	Isotig06825	41	0	6.229	8.08E-11	UNIPROTKB|Q43848 - symbol:Q43848 "Transketolase, chloroplastic" species:4113 "Solanum tuberosum" [GO:0004802 "transketolase activity" evidence=ISS] [GO:0009535 "chloroplast thylakoid membrane" evidence=ISS] InterPro:IPR005476 InterPro:IPR005478 InterPro:IPR009014 InterPro:IPR015941 Pfam:PF02780 InterPro:IPR005475 Pfam:PF00456 Pfam:PF02779 InterPro:IPR005474 GO:GO:0046872 GO:GO:0009535 SMART:SM00861 InterPro:IPR020826 Gene3D:G3DSA:3.40.50.920 SUPFAM:SSF52922 PROSITE:PS00801 PROSITE:PS00802 GO:GO:0004802 TIGRFAMs:TIGR00232 EMBL:Z50099 PIR:S58083 ProteinModelPortal:Q43848 SMR:Q43848 PRIDE:Q43848 Uniprot:Q43848
Root	Isotig06826	45	0	6.363	1.08E-11	UNIPROTKB|Q43848 - symbol:Q43848 "Transketolase, chloroplastic" species:4113 "Solanum tuberosum" [GO:0004802 "transketolase activity" evidence=ISS] [GO:0009535 "chloroplast thylakoid membrane" evidence=ISS] InterPro:IPR005476 InterPro:IPR005478 InterPro:IPR009014 InterPro:IPR015941 Pfam:PF02780 InterPro:IPR005475 Pfam:PF00456 Pfam:PF02779 InterPro:IPR005474 GO:GO:0046872 GO:GO:0009535 SMART:SM00861 InterPro:IPR020826 Gene3D:G3DSA:3.40.50.920 SUPFAM:SSF52922 PROSITE:PS00801 PROSITE:PS00802 GO:GO:0004802 TIGRFAMs:TIGR00232 EMBL:Z50099 PIR:S58083 ProteinModelPortal:Q43848 SMR:Q43848 PRIDE:Q43848 Uniprot:Q43848
Root	Isotig06827	26	60	-1.335	3.86E-05	TAIR|locus:2121894 - symbol:ATR1 "AT4G24520" species:3702 "Arabidopsis thaliana" [GO:0009737 "response to abscisic acid stimulus" evidence=IDA] [GO:0005829 "cytosol" evidence=IDA] [GO:0003958 "NADPH-hemoprotein reductase activity" evidence=IDA] [GO:0005783 "endoplasmic reticulum" evidence=IDA] [GO:0009698

Tissue	Identification	# of reads (stress)	# of reads (control)	Fold Change	p-value	Description
						"phenylpropanoid metabolic process" evidence=IDA] [GO:0006979 "response to oxidative stress" evidence=IEP] InterPro:IPR001094 InterPro:IPR001433 InterPro:IPR001709 InterPro:IPR003097 InterPro:IPR008254 InterPro:IPR017927 InterPro:IPR017938 InterPro:IPR023173 InterPro:IPR023208 Pfam:PF00175 Pfam:PF00258 Pfam:PF00667 PIRSF:PIRSF000208 PRINTS:PR00369 PRINTS:PR00371 PROSITE:PS50902 PROSITE:PS51384 GO:GO:0005829 GO:GO:0009737 EMBL:CP002687 GO:GO:0006979 GO:GO:0005789 GO:GO:0005506 GO:GO:0009698 EMBL:AL161561 EMBL:AL035356 SUPFAM:SSF63380 GO:GO:0010181 Gene3D:G3DSA:1.20.990.10 OMA:KIQPRYY HSSP:P00388 GO:GO:0003958 KO:K00327 EMBL:AY054688 EMBL:BT008426 IPI:IPI00541592 PIR:T05582 RefSeq:NP_194183.1 UniGene:At.144 UniGene:At.70123 ProteinModelPortal:Q9SB48 SMR:Q9SB48 STRING:Q9SB48 PRIDE:Q9SB48 EnsemblPlants:AT4G24520.1 GeneID:828554 KEGG:ath:AT4G24520 TAIR:At4g24520 InParanoid:Q9SB48 PhylomeDB:Q9SB48 ProtClustDB:CLSN2685426 ArrayExpress:Q9SB48 Genevestigator:Q9SB48 Uniprot:Q9SB48
Root	Isotig06828	26	61	-1.359	2.59E-05	TAIR|locus:2121894 - symbol:ATR1 "AT4G24520" species:3702 "Arabidopsis thaliana" [GO:0009737 "response to abscisic acid stimulus" evidence=IDA] [GO:0005829 "cytosol" evidence=IDA] [GO:0003958 "NADPH-hemoprotein reductase activity" evidence=IDA] [GO:0005783 "endoplasmic reticulum" evidence=IDA] [GO:0009698 "phenylpropanoid metabolic process" evidence=IDA] [GO:0006979 "response to oxidative stress" evidence=IEP] InterPro:IPR001094 InterPro:IPR001433 InterPro:IPR001709 InterPro:IPR003097 InterPro:IPR008254 InterPro:IPR017927 InterPro:IPR017938 InterPro:IPR023173 InterPro:IPR023208 Pfam:PF00175 Pfam:PF00258 Pfam:PF00667 PIRSF:PIRSF000208 PRINTS:PR00369 PRINTS:PR00371 PROSITE:PS50902 PROSITE:PS51384 GO:GO:0005829 GO:GO:0009737 EMBL:CP002687 GO:GO:0006979 GO:GO:0005789 GO:GO:0005506 GO:GO:0009698 EMBL:AL161561 EMBL:AL035356 SUPFAM:SSF63380 GO:GO:0010181 Gene3D:G3DSA:1.20.990.10 OMA:KIQPRYY HSSP:P00388 GO:GO:0003958 KO:K00327 EMBL:AY054688 EMBL:BT008426 IPI:IPI00541592 PIR:T05582 RefSeq:NP_194183.1 UniGene:At.144 UniGene:At.70123 ProteinModelPortal:Q9SB48 SMR:Q9SB48 STRING:Q9SB48 PRIDE:Q9SB48 EnsemblPlants:AT4G24520.1 GeneID:828554 KEGG:ath:AT4G24520 TAIR:At4g24520 InParanoid:Q9SB48 PhylomeDB:Q9SB48 ProtClustDB:CLSN2685426 ArrayExpress:Q9SB48 Genevestigator:Q9SB48 Uniprot:Q9SB48
Root	Isotig06829	10	29	-1.665	0.000753212	TAIR|locus:2036778 - symbol:GPT2 "AT1G61800" species:3702 "Arabidopsis thaliana" [GO:0015152 "glucose-6-phosphate transmembrane transporter activity" evidence=ISS;IDA] [GO:0015297 "antiporter activity" evidence=ISS] [GO:0015712 "hexose phosphate transport" evidence=ISS] [GO:0005315 "inorganic phosphate transmembrane transporter activity" evidence=IDA] [GO:0007276 "gamete generation" evidence=IMP] [GO:0015120 "phosphoglycerate transmembrane transporter activity" evidence=IDA] [GO:0015713 "phosphoglycerate transport" evidence=IDA] [GO:0015714 "phosphoenolpyruvate transport" evidence=IDA] [GO:0015760 "glucose-6-phosphate transport" evidence=IDA] [GO:0035436 "triose phosphate transmembrane transport" evidence=IDA] [GO:0071917 "triose-phosphate transmembrane transporter activity" evidence=IDA] [GO:0009744 "response to sucrose stimulus" evidence=IEP] [GO:0009749 "response to glucose stimulus" evidence=IEP] [GO:0005774 "vacuolar membrane" evidence=IDA] [GO:0009624 "response to nematode" evidence=IEP] [GO:0009643 "photosynthetic acclimation" evidence=IMP] [GO:0010109 "regulation of photosynthesis" evidence=IMP] [GO:0015979 "photosynthesis" evidence=IMP] [GO:0080167 "response to karrikin" evidence=IEP] InterPro:IPR000620 InterPro:IPR004696 Pfam:PF00892 Pfam:PF03151 GO:GO:0016021 EMBL:CP002684 GenomeReviews:CT485782_GR GO:GO:0005774 GO:GO:0009624 GO:GO:0080167 GO:GO:0005315 GO:GO:0031969 GO:GO:0009749 GO:GO:0009744 GO:GO:0015979 EMBL:AC004392 InterPro:IPR004853 GO:GO:0015152 eggNOG:NOG312636 HOGENOM:HBG745150 KO:K15283 GO:GO:0015120 GO:GO:0071917 GO:GO:0015714 TIGRFAMs:TIGR00817 EMBL:AY042874 EMBL:AY081479 IPI:IPI00535228 PIR:T02126 RefSeq:NP_564785.1 UniGene:At.10733 STRING:Q94B38 PRIDE:Q94B38 EnsemblPlants:AT1G61800.1 GeneID:842477 KEGG:ath:AT1G61800 TAIR:At1g61800 InParanoid:Q94B38 OMA:PDEQSAQ ArrayExpress:Q94B38 Genevestigator:Q94B38 GermOnline:AT1G61800 GO:GO:0009643 GO:GO:0010109 Uniprot:Q94B38
Root	Isotig06831	2	19	-3.377	3.93E-05	TAIR|locus:2088500 - symbol:RLK902 "AT3G17840" species:3702 "Arabidopsis thaliana" [GO:0004674 "protein serine/threonine kinase activity" evidence=ISS] [GO:0005524 "ATP binding" evidence=ISS] [GO:0006468 "protein phosphorylation" evidence=ISS] [GO:0007169 "transmembrane receptor protein tyrosine kinase signaling pathway" evidence=ISS] [GO:0016301 "kinase activity" evidence=ISS] [GO:0005886 "plasma membrane" evidence=IDA] [GO:0009506 "plasmodesma" evidence=IDA] InterPro:IPR000719 InterPro:IPR011009 InterPro:IPR017441 Pfam:PF00069 PROSITE:PS00107 PROSITE:PS50011 GO:GO:0016021 GO:GO:0005886 GO:GO:0009506 GO:GO:0005524 EMBL:CP002686 GenomeReviews:BA000014_GR eggNOG:COG0515 GO:GO:0004872

Tissue	Identification	# of reads (stress)	# of reads (control)	Fold Change	p-value	Description
						SUPFAM:SSF56112 HOGENOM:HBG755340 GO:GO:0004672 InterPro:IPR013210 Pfam:PF08263 EMBL:AB019230 EMBL:AY095994 EMBL:BT002283 EMBL:AY086189 IPI:IPI00521331 RefSeq:NP_566589.1 UniGene:At.38651 ProteinModelPortal:Q9LVI6 SMR:Q9LVI6 IntAct:Q9LVI6 STRING:Q9LVI6 PRIDE:Q9LVI6 EnsemblPlants:AT3G17840.1 GeneID:821053 KEGG:ath:AT3G17840 GeneFarm:530 TAIR:At3g17840 InParanoid:Q9LVI6 OMA:YYYSGDE PhylomeDB:Q9LVI6 ProtClustDB:CLSN2688502 Genevestigator:Q9LVI6 Uniprot:Q9LVI6
Root	Isotig06832	2	19	-3.377	3.93E-05	TAIR|locus:2088500 - symbol:RLK902 "AT3G17840" species:3702 "Arabidopsis thaliana" [GO:0004674 "protein serine/threonine kinase activity" evidence=ISS] [GO:0005524 "ATP binding" evidence=ISS] [GO:0006468 "protein phosphorylation" evidence=ISS] [GO:0007169 "transmembrane receptor protein tyrosine kinase signaling pathway" evidence=ISS] [GO:0016301 "kinase activity" evidence=ISS] [GO:0005886 "plasma membrane" evidence=IDA] [GO:0009506 "plasmodesma" evidence=IDA] InterPro:IPR000719 InterPro:IPR011009 InterPro:IPR017441 Pfam:PF00069 PROSITE:PS00107 PROSITE:PS50011 GO:GO:0016021 GO:GO:0005886 GO:GO:0009506 GO:GO:0005524 EMBL:CP002686 GenomeReviews:BA000014_GR eggNOG:COG0515 GO:GO:0004872 SUPFAM:SSF56112 HOGENOM:HBG755340 GO:GO:0004672 InterPro:IPR013210 Pfam:PF08263 EMBL:AB019230 EMBL:AY095994 EMBL:BT002283 EMBL:AY086189 IPI:IPI00521331 RefSeq:NP_566589.1 UniGene:At.38651 ProteinModelPortal:Q9LVI6 SMR:Q9LVI6 IntAct:Q9LVI6 STRING:Q9LVI6 PRIDE:Q9LVI6 EnsemblPlants:AT3G17840.1 GeneID:821053 KEGG:ath:AT3G17840 GeneFarm:530 TAIR:At3g17840 InParanoid:Q9LVI6 OMA:YYYSGDE PhylomeDB:Q9LVI6 ProtClustDB:CLSN2688502 Genevestigator:Q9LVI6 Uniprot:Q9LVI6
Root	Isotig06839	8	28	-1.936	0.000239276	TAIR|locus:2091960 - symbol:AT3G26935 "AT3G26935" species:3702 "Arabidopsis thaliana" [GO:0008150 "biological_process" evidence=ND] [GO:0008270 "zinc ion binding" evidence=IEA] [GO:0005886 "plasma membrane" evidence=IDA] InterPro:IPR001594 Pfam:PF01529 PROSITE:PS50216 GO:GO:0016021 GO:GO:0005886 EMBL:CP002686 GenomeReviews:BA000014_GR GO:GO:0008270 GO:GO:0016746 EMBL:AB026649 EMBL:AP000602 eggNOG:COG5273 HOGENOM:HBG744542 EMBL:AK228693 IPI:IPI00526912 RefSeq:NP_850638.1 UniGene:At.47119 STRING:Q0WQK2 PRIDE:Q0WQK2 EnsemblPlants:AT3G26935.1 GeneID:822311 KEGG:ath:AT3G26935 TAIR:At3g26935 InParanoid:Q0WQK2 OMA:AVGDIEM PhylomeDB:Q0WQK2 ProtClustDB:CLSN2918228 Genevestigator:Q0WQK2 Uniprot:Q0WQK2
Root	Isotig06845	17	51	-1.714	5.10E-06	TAIR|locus:2156789 - symbol:CEV1 "AT5G05170" species:3702 "Arabidopsis thaliana" [GO:0016757 "transferase activity, transferring glycosyl groups" evidence=ISS] [GO:0016759 "cellulose synthase activity" evidence=ISS;IMP] [GO:0005886 "plasma membrane" evidence=IDA;TAS] [GO:0009834 "secondary cell wall biogenesis" evidence=IMP] [GO:0009833 "primary cell wall biogenesis" evidence=IMP;TAS] [GO:0030244 "cellulose biosynthetic process" evidence=IMP] [GO:0009809 "lignin biosynthetic process" evidence=IGI] [GO:0043255 "regulation of carbohydrate biosynthetic process" evidence=IGI] [GO:0006952 "defense response" evidence=TAS] [GO:0005794 "Golgi apparatus" evidence=IDA] [GO:0009506 "plasmodesma" evidence=IDA] InterPro:IPR001841 InterPro:IPR005150 Pfam:PF03552 PROSITE:PS50089 SMART:SM00184 Prosite:PS00518 GO:GO:0016021 GO:GO:0005886 GO:GO:0009506 EMBL:CP002688 GenomeReviews:BA000015_GR GO:GO:0006952 GO:GO:0005794 GO:GO:0008270 InterPro:IPR013083 Gene3D:G3DSA:3.30.40.10 GO:GO:0007047 CAZy:GT2 eggNOG:COG1215 GO:GO:0016760 GO:GO:0030244 HOGENOM:HBG744549 KO:K10999 GO:GO:0009833 EMBL:AF027174 EMBL:AB018111 EMBL:AY045960 EMBL:BT002335 EMBL:AK230097 IPI:IPI00528019 PIR:T52054 RefSeq:NP_196136.1 UniGene:At.24338 ProteinModelPortal:Q941L0 SMR:Q941L0 DIP:DIP-46437N IntAct:Q941L0 STRING:Q941L0 PRIDE:Q941L0 EnsemblPlants:AT5G05170.1 GeneID:830399 KEGG:ath:AT5G05170 GeneFarm:5086 TAIR:At5g05170 InParanoid:Q941L0 OMA:DVNQSPN PhylomeDB:Q941L0 ProtClustDB:PLN02638 Genevestigator:Q941L0 GermOnline:AT5G05170 Uniprot:Q941L0
Root	Isotig06846	8	29	-1.987	0.000143108	TAIR|locus:2156789 - symbol:CEV1 "AT5G05170" species:3702 "Arabidopsis thaliana" [GO:0016757 "transferase activity, transferring glycosyl groups" evidence=ISS] [GO:0016759 "cellulose synthase activity" evidence=ISS;IMP] [GO:0005886 "plasma membrane" evidence=IDA;TAS] [GO:0009834 "secondary cell wall biogenesis" evidence=IMP] [GO:0009833 "primary cell wall biogenesis" evidence=IMP;TAS] [GO:0030244 "cellulose biosynthetic process" evidence=IMP] [GO:0009809 "lignin biosynthetic process" evidence=IGI] [GO:0043255 "regulation of carbohydrate biosynthetic process" evidence=IGI] [GO:0006952 "defense response" evidence=TAS] [GO:0005794 "Golgi apparatus" evidence=IDA] [GO:0009506 "plasmodesma" evidence=IDA] InterPro:IPR001841 InterPro:IPR005150 Pfam:PF03552 PROSITE:PS50089 SMART:SM00184 Prosite:PS00518 GO:GO:0016021 GO:GO:0005886 GO:GO:0009506 EMBL:CP002688 GenomeReviews:BA000015_GR GO:GO:0006952

Tissue	Identification	# of reads (stress)	# of reads (control)	Fold Change	p-value	Description
						GO:GO:0005794 GO:GO:0008270 InterPro:IPR013083 Gene3D:G3DSA:3.30.40.10 GO:GO:0007047 CAZy:GT2 eggNOG:COG1215 GO:GO:0016760 GO:GO:0030244 HOGENOM:HBG744549 KO:K10999 GO:GO:0009833 EMBL:AF027174 EMBL:AB018111 EMBL:AY045960 EMBL:BT002335 EMBL:AK230097 IPI:IPI00528019 PIR:T52054 RefSeq:NP_196136.1 UniGene:At.24338 ProteinModelPortal:Q941L0 SMR:Q941L0 DIP:DIP-46437N IntAct:Q941L0 STRING:Q941L0 PRIDE:Q941L0 EnsemblPlants:AT5G05170.1 GeneID:830399 KEGG:ath:AT5G05170 GeneFarm:5086 TAIR:At5g05170 InParanoid:Q941L0 OMA:DVNQSPN PhylomeDB:Q941L0 ProtClustDB:PLN02638 Genevestigator:Q941L0 GermOnline:AT5G05170 Uniprot:Q941L0
Root	Isotig06849	8	29	-1.987	0.000143108	TAIR|locus:2129650 - symbol:AT4G14350 "AT4G14350" species:3702 "Arabidopsis thaliana" [GO:0004672 "protein kinase activity" evidence=ISS] [GO:0005524 "ATP binding" evidence=ISS] [GO:0006468 "protein phosphorylation" evidence=IEA;ISS] [GO:0016301 "kinase activity" evidence=ISS] [GO:0005634 "nucleus" evidence=IDA] [GO:0005737 "cytoplasm" evidence=IDA] [GO:0009524 "phragmoplast" evidence=IDA] [GO:0005886 "plasma membrane" evidence=IDA] [GO:0005829 "cytosol" evidence=IDA] InterPro:IPR000719 InterPro:IPR000961 InterPro:IPR002290 InterPro:IPR008271 InterPro:IPR011009 InterPro:IPR017441 InterPro:IPR017892 Pfam:PF00069 Pfam:PF00433 PROSITE:PS00107 PROSITE:PS00108 PROSITE:PS50011 PROSITE:PS51285 SMART:SM00133 SMART:SM00220 GO:GO:0005829 GO:GO:0005886 GO:GO:0005524 EMBL:CP002687 GO:GO:0005634 SUPFAM:SSF56112 GO:GO:0004674 GO:GO:0009524 HSSP:P31751 OMA:IFPPEIP EMBL:AY128279 EMBL:BT000628 IPI:IPI00546199 RefSeq:NP_193171.2 RefSeq:NP_849380.1 UniGene:At.23938 UniGene:At.68891 ProteinModelPortal:Q8L7S7 SMR:Q8L7S7 PRIDE:Q8L7S7 EnsemblPlants:AT4G14350.1 EnsemblPlants:AT4G14350.2 GeneID:827078 KEGG:ath:AT4G14350 TAIR:At4g14350 InParanoid:Q8L7S7 PhylomeDB:Q8L7S7 ProtClustDB:CLSN2690563 Genevestigator:Q8L7S7 Uniprot:Q8L7S7
Root	Isotig06850	8	29	-1.987	0.000143108	TAIR|locus:2129650 - symbol:AT4G14350 "AT4G14350" species:3702 "Arabidopsis thaliana" [GO:0004672 "protein kinase activity" evidence=ISS] [GO:0005524 "ATP binding" evidence=ISS] [GO:0006468 "protein phosphorylation" evidence=IEA;ISS] [GO:0016301 "kinase activity" evidence=ISS] [GO:0005634 "nucleus" evidence=IDA] [GO:0005737 "cytoplasm" evidence=IDA] [GO:0009524 "phragmoplast" evidence=IDA] [GO:0005886 "plasma membrane" evidence=IDA] [GO:0005829 "cytosol" evidence=IDA] InterPro:IPR000719 InterPro:IPR000961 InterPro:IPR002290 InterPro:IPR008271 InterPro:IPR011009 InterPro:IPR017441 InterPro:IPR017892 Pfam:PF00069 Pfam:PF00433 PROSITE:PS00107 PROSITE:PS00108 PROSITE:PS50011 PROSITE:PS51285 SMART:SM00133 SMART:SM00220 GO:GO:0005829 GO:GO:0005886 GO:GO:0005524 EMBL:CP002687 GO:GO:0005634 SUPFAM:SSF56112 GO:GO:0004674 GO:GO:0009524 HSSP:P31751 OMA:IFPPEIP EMBL:AY128279 EMBL:BT000628 IPI:IPI00546199 RefSeq:NP_193171.2 RefSeq:NP_849380.1 UniGene:At.23938 UniGene:At.68891 ProteinModelPortal:Q8L7S7 SMR:Q8L7S7 PRIDE:Q8L7S7 EnsemblPlants:AT4G14350.1 EnsemblPlants:AT4G14350.2 GeneID:827078 KEGG:ath:AT4G14350 TAIR:At4g14350 InParanoid:Q8L7S7 PhylomeDB:Q8L7S7 ProtClustDB:CLSN2690563 Genevestigator:Q8L7S7 Uniprot:Q8L7S7
Root	Isotig06853	13	42	-1.821	1.59E-05	TAIR|locus:2103172 - symbol:ASD1 "AT3G10740" species:3702 "Arabidopsis thaliana" [GO:0016798 "hydrolase activity, acting on glycosyl bonds" evidence=ISS] [GO:0009044 "xylan 1,4-beta-xylosidase activity" evidence=IDA] [GO:0045493 "xylan catabolic process" evidence=IDA] [GO:0046556 "alpha-N-arabinofuranosidase activity" evidence=IDA] [GO:0005773 "vacuole" evidence=IDA] [GO:0009505 "plant-type cell wall" evidence=IDA] [GO:0048046 "apoplast" evidence=IDA] InterPro:IPR010720 Pfam:PF06964 SMART:SM00813 GO:GO:0048046 GO:GO:0005773 EMBL:CP002686 GenomeReviews:BA000014_GR GO:GO:0045493 InterPro:IPR017853 SUPFAM:SSF51445 GO:GO:0005578 EMBL:AC011708 CAZy:GH51 GO:GO:0046556 GO:GO:0046373 eggNOG:COG3534 GO:GO:0009505 EMBL:AY243509 EMBL:AF372949 EMBL:AY143944 EMBL:AK222175 EMBL:AK220766 IPI:IPI00526411 RefSeq:NP_187685.1 UniGene:At.20271 ProteinModelPortal:Q9SG80 SMR:Q9SG80 STRING:Q9SG80 PRIDE:Q9SG80 EnsemblPlants:AT3G10740.1 GeneID:820243 KEGG:ath:AT3G10740 TAIR:At3g10740 InParanoid:Q9SG80 OMA:LEYLGIG PhylomeDB:Q9SG80 ProtClustDB:CLSN2684036 Genevestigator:Q9SG80 GO:GO:0009044 Uniprot:Q9SG80
Root	Isotig06854	12	34	-1.631	0.000323514	TAIR|locus:2103172 - symbol:ASD1 "AT3G10740" species:3702 "Arabidopsis thaliana" [GO:0016798 "hydrolase activity, acting on glycosyl bonds" evidence=ISS] [GO:0009044 "xylan 1,4-beta-xylosidase activity" evidence=IDA] [GO:0045493 "xylan catabolic process" evidence=IDA] [GO:0046556 "alpha-N-arabinofuranosidase activity" evidence=IDA] [GO:0005773 "vacuole" evidence=IDA] [GO:0009505 "plant-type cell wall" evidence=IDA] [GO:0048046 "apoplast" evidence=IDA] InterPro:IPR010720 Pfam:PF06964 SMART:SM00813 GO:GO:0048046 GO:GO:0005773 EMBL:CP002686 GenomeReviews:BA000014_GR GO:GO:0045493 InterPro:IPR017853 SUPFAM:SSF51445 GO:GO:0005578 EMBL:AC011708 CAZy:GH51 GO:GO:0046556 GO:GO:0046373

Tissue	Identification	# of reads (stress)	# of reads (control)	Fold Change	p-value	Description
						eggNOG:COG3534 GO:GO:0009505 EMBL:AY243509 EMBL:AF372949 EMBL:AY143944 EMBL:AK222175 EMBL:AK220766 IPI:IPI00526411 RefSeq:NP_187685.1 UniGene:At.20271 ProteinModelPortal:Q9SG80 SMR:Q9SG80 STRING:Q9SG80 PRIDE:Q9SG80 EnsemblPlants:AT3G10740.1 GeneID:820243 KEGG:ath:AT3G10740 TAIR:At3g10740 InParanoid:Q9SG80 OMA:LEYLGIG PhylomeDB:Q9SG80 ProtClustDB:CLSN2684036 Genevestigator:Q9SG80 GO:GO:0009044 Uniprot:Q9SG80
Root	Isotig06875	17	50	-1.685	8.10E-06	TAIR|locus:2026177 - symbol:AT1G62660 species:3702 "Arabidopsis thaliana" [GO:0004553 "hydrolase activity, hydrolyzing O-glycosyl compounds" evidence=IEA;ISS] [GO:0071370 "cellular response to gibberellin stimulus" evidence=IEP] [GO:0000325 "plant-type vacuole" evidence=IDA] [GO:0005773 "vacuole" evidence=IDA] [GO:0009617 "response to bacterium" evidence=IEP] InterPro:IPR001362 InterPro:IPR018053 InterPro:IPR021792 Pfam:PF11837 PROSITE:PS00609 SMART:SM00640 EMBL:CP002684 GO:GO:0005975 InterPro:IPR008985 SUPFAM:SSF49899 InterPro:IPR023296 SUPFAM:SSF75005 CAZy:GH32 KO:K01193 GO:GO:0004575 InterPro:IPR013189 InterPro:IPR013148 Pfam:PF08244 Pfam:PF00251 ProtClustDB:CLSN2687777 EMBL:AY048269 EMBL:AY062481 EMBL:AY063904 EMBL:AY114066 EMBL:X99111 IPI:IPI00517592 PIR:S71268 RefSeq:NP_564798.1 UniGene:At.24113 UniGene:At.67066 ProteinModelPortal:Q43348 SMR:Q43348 STRING:Q43348 PRIDE:Q43348 ProMEX:Q43348 EnsemblPlants:AT1G62660.1 GeneID:842563 KEGG:ath:AT1G62660 TAIR:At1g62660 InParanoid:Q43348 OMA:HSIVEGF PhylomeDB:Q43348 ArrayExpress:Q43348 Genevestigator:Q43348 Uniprot:Q43348
Root	Isotig06876	12	47	-2.099	5.30E-07	TAIR|locus:2026177 - symbol:AT1G62660 species:3702 "Arabidopsis thaliana" [GO:0004553 "hydrolase activity, hydrolyzing O-glycosyl compounds" evidence=IEA;ISS] [GO:0071370 "cellular response to gibberellin stimulus" evidence=IEP] [GO:0000325 "plant-type vacuole" evidence=IDA] [GO:0005773 "vacuole" evidence=IDA] [GO:0009617 "response to bacterium" evidence=IEP] InterPro:IPR001362 InterPro:IPR018053 InterPro:IPR021792 Pfam:PF11837 PROSITE:PS00609 SMART:SM00640 EMBL:CP002684 GO:GO:0005975 InterPro:IPR008985 SUPFAM:SSF49899 InterPro:IPR023296 SUPFAM:SSF75005 CAZy:GH32 KO:K01193 GO:GO:0004575 InterPro:IPR013189 InterPro:IPR013148 Pfam:PF08244 Pfam:PF00251 ProtClustDB:CLSN2687777 EMBL:AY048269 EMBL:AY062481 EMBL:AY063904 EMBL:AY114066 EMBL:X99111 IPI:IPI00517592 PIR:S71268 RefSeq:NP_564798.1 UniGene:At.24113 UniGene:At.67066 ProteinModelPortal:Q43348 SMR:Q43348 STRING:Q43348 PRIDE:Q43348 ProMEX:Q43348 EnsemblPlants:AT1G62660.1 GeneID:842563 KEGG:ath:AT1G62660 TAIR:At1g62660 InParanoid:Q43348 OMA:HSIVEGF PhylomeDB:Q43348 ArrayExpress:Q43348 Genevestigator:Q43348 Uniprot:Q43348
Root	Isotig06893	12	50	-2.188	1.10E-07	TAIR|locus:2080722 - symbol:SPY "AT3G11540" species:3702 "Arabidopsis thaliana" [GO:0016757 "transferase activity, transferring glycosyl groups" evidence=ISS] [GO:0016262 "protein N-acetylglucosaminyltransferase activity" evidence=TAS] [GO:0009938 "negative regulation of gibberellic acid mediated signaling pathway" evidence=IGI] [GO:0005634 "nucleus" evidence=IDA;TAS] [GO:0005737 "cytoplasm" evidence=IDA] [GO:0009740 "gibberellic acid mediated signaling pathway" evidence=TAS] [GO:0005515 "protein binding" evidence=IPI] [GO:0009736 "cytokinin mediated signaling pathway" evidence=IMP] [GO:0005829 "cytosol" evidence=IDA] [GO:2000377 "regulation of reactive oxygen species metabolic process" evidence=IMP] InterPro:IPR001440 InterPro:IPR011990 InterPro:IPR013026 InterPro:IPR019734 Pfam:PF00515 PROSITE:PS50005 PROSITE:PS50293 SMART:SM00028 GO:GO:0005829 GO:GO:0005634 EMBL:CP002686 GenomeReviews:BA000014_GR GO:GO:0005515 GO:GO:0030154 GO:GO:0048511 GO:GO:0009736 InterPro:IPR006597 Gene3D:G3DSA:1.25.40.10 SMART:SM00671 GO:GO:0009908 GO:GO:0016262 EMBL:AC008153 GO:GO:0009740 GO:GO:2000377 GO:GO:0009938 CAZy:GT41 eggNOG:COG3914 EMBL:U62135 EMBL:AK220931 EMBL:AK221192 EMBL:AK221314 IPI:IPI00544177 RefSeq:NP_187761.1 UniGene:At.17656 ProteinModelPortal:Q96301 SMR:Q96301 IntAct:Q96301 STRING:Q96301 PRIDE:Q96301 ProMEX:Q96301 EnsemblPlants:AT3G11540.1 GeneID:820327 KEGG:ath:AT3G11540 GeneFarm:5156 TAIR:At3g11540 HOGENOM:HBG684498 InParanoid:Q96301 OMA:GVPCVTM PhylomeDB:Q96301 ProtClustDB:CLSN2684418 Genevestigator:Q96301 Uniprot:Q96301
Root	Isotig06894	15	54	-1.977	2.32E-07	TAIR|locus:2080722 - symbol:SPY "AT3G11540" species:3702 "Arabidopsis thaliana" [GO:0016757 "transferase activity, transferring glycosyl groups" evidence=ISS] [GO:0016262 "protein N-acetylglucosaminyltransferase activity" evidence=TAS] [GO:0009938 "negative regulation of gibberellic acid mediated signaling pathway" evidence=IGI] [GO:0005634 "nucleus" evidence=IDA;TAS] [GO:0005737 "cytoplasm" evidence=IDA] [GO:0009740 "gibberellic acid mediated signaling pathway" evidence=TAS] [GO:0005515 "protein binding" evidence=IPI] [GO:0009736 "cytokinin mediated signaling pathway" evidence=IMP] [GO:0005829 "cytosol" evidence=IDA] [GO:2000377 "regulation of

Tissue	Identification	# of reads (stress)	# of reads (control)	Fold Change	p-value	Description
						reactive oxygen species metabolic process" evidence=IMP] InterPro:IPR001440 InterPro:IPR011990 InterPro:IPR013026 InterPro:IPR019734 Pfam:PF00515 PROSITE:PS50005 PROSITE:PS50293 SMART:SM00028 GO:GO:0005829 GO:GO:0005634 EMBL:CP002686 GenomeReviews:BA000014_GR GO:GO:0005515 GO:GO:0030154 GO:GO:0048511 GO:GO:0009736 InterPro:IPR006597 Gene3D:G3DSA:1.25.40.10 SMART:SM00671 GO:GO:0009908 GO:GO:0016262 EMBL:AC008153 GO:GO:0009740 GO:GO:2000377 GO:GO:0009938 CAZy:GT41 eggNOG:COG3914 EMBL:U62135 EMBL:AK220931 EMBL:AK221192 EMBL:AK221314 IPI:IPI00544177 RefSeq:NP_187761.1 UniGene:At.17656 ProteinModelPortal:Q96301 SMR:Q96301 IntAct:Q96301 STRING:Q96301 PRIDE:Q96301 ProMEX:Q96301 EnsemblPlants:AT3G11540.1 GeneID:820327 KEGG:ath:AT3G11540 GeneFarm:5156 TAIR:At3g11540 HOGENOM:HBG684498 InParanoid:Q96301 OMA:GVPCVTM PhylomeDB:Q96301 ProtClustDB:CLSN2684418 Genevestigator:Q96301 Uniprot:Q96301
Root	Isotig06895	35	2	4.000	1.39E-08	TAIR|locus:2076086 - symbol:BAM1 "AT3G23920" species:3702 "Arabidopsis thaliana" [GO:0016161 "beta-amylase activity" evidence=ISS;IDA] [GO:0005983 "starch catabolic process" evidence=IMP] [GO:0005634 "nucleus" evidence=IDA] [GO:0005829 "cytosol" evidence=IDA] [GO:0009414 "response to water deprivation" evidence=IEP] BRENDA:3.2.1.2 InterPro:IPR001371 InterPro:IPR001554 InterPro:IPR013781 InterPro:IPR018238 Pfam:PF01373 PRINTS:PR00750 PRINTS:PR00842 PROSITE:PS00506 PROSITE:PS00679 GO:GO:0005829 GO:GO:0005634 GO:GO:0009507 EMBL:CP002686 GenomeReviews:BA000014_GR GO:GO:0043169 InterPro:IPR017853 Gene3D:G3DSA:3.20.20.80 SUPFAM:SSF51445 GO:GO:0009414 GO:GO:0005983 EMBL:AP001297 EMBL:AF367293 EMBL:AY074393 EMBL:AY078046 EMBL:AY096517 EMBL:AK226274 IPI:IPI00518242 RefSeq:NP_189034.1 UniGene:At.8278 HSSP:P16098 ProteinModelPortal:Q9LIR6 SMR:Q9LIR6 STRING:Q9LIR6 CAZy:GH14 PRIDE:Q9LIR6 EnsemblPlants:AT3G23920.1 GeneID:821975 KEGG:ath:AT3G23920 TAIR:At3g23920 eggNOG:NOG77898 InParanoid:Q9LIR6 KO:K01177 OMA:DKDQDLA PhylomeDB:Q9LIR6 ProtClustDB:PLN00197 ArrayExpress:Q9LIR6 Genevestigator:Q9LIR6 GO:GO:0016161 Uniprot:Q9LIR6
Root	Isotig06896	33	2	3.915	4.43E-08	TAIR|locus:2076086 - symbol:BAM1 "AT3G23920" species:3702 "Arabidopsis thaliana" [GO:0016161 "beta-amylase activity" evidence=ISS;IDA] [GO:0005983 "starch catabolic process" evidence=IMP] [GO:0005634 "nucleus" evidence=IDA] [GO:0005829 "cytosol" evidence=IDA] [GO:0009414 "response to water deprivation" evidence=IEP] BRENDA:3.2.1.2 InterPro:IPR001371 InterPro:IPR001554 InterPro:IPR013781 InterPro:IPR018238 Pfam:PF01373 PRINTS:PR00750 PRINTS:PR00842 PROSITE:PS00506 PROSITE:PS00679 GO:GO:0005829 GO:GO:0005634 GO:GO:0009507 EMBL:CP002686 GenomeReviews:BA000014_GR GO:GO:0043169 InterPro:IPR017853 Gene3D:G3DSA:3.20.20.80 SUPFAM:SSF51445 GO:GO:0009414 GO:GO:0005983 EMBL:AP001297 EMBL:AF367293 EMBL:AY074393 EMBL:AY078046 EMBL:AY096517 EMBL:AK226274 IPI:IPI00518242 RefSeq:NP_189034.1 UniGene:At.8278 HSSP:P16098 ProteinModelPortal:Q9LIR6 SMR:Q9LIR6 STRING:Q9LIR6 CAZy:GH14 PRIDE:Q9LIR6 EnsemblPlants:AT3G23920.1 GeneID:821975 KEGG:ath:AT3G23920 TAIR:At3g23920 eggNOG:NOG77898 InParanoid:Q9LIR6 KO:K01177 OMA:DKDQDLA PhylomeDB:Q9LIR6 ProtClustDB:PLN00197 ArrayExpress:Q9LIR6 Genevestigator:Q9LIR6 GO:GO:0016161 Uniprot:Q9LIR6
Root	Isotig06907	7	33	-2.366	6.09E-06	TAIR|locus:2175143 - symbol:AT5G60570 "AT5G60570" species:3702 "Arabidopsis thaliana" [GO:0003674 "molecular_function" evidence=ND] [GO:0008150 "biological_process" evidence=ND] [GO:0005634 "nucleus" evidence=IDA] InterPro:IPR006652 Pfam:PF01344 PROSITE:PS50181 SMART:SM00612 GO:GO:0005634 EMBL:CP002688 GenomeReviews:BA000015_GR InterPro:IPR015916 Gene3D:G3DSA:2.130.10.80 EMBL:AB011483 eggNOG:NOG312724 EMBL:AK221729 EMBL:BT029765 IPI:IPI00533154 RefSeq:NP_200865.1 UniGene:At.43004 ProteinModelPortal:Q9FKJ0 SMR:Q9FKJ0 IntAct:Q9FKJ0 PRIDE:Q9FKJ0 EnsemblPlants:AT5G60570.1 GeneID:836178 KEGG:ath:AT5G60570 TAIR:At5g60570 InParanoid:Q9FKJ0 OMA:SSSGRWE PhylomeDB:Q9FKJ0 ProtClustDB:CLSN2916403 Genevestigator:Q9FKJ0 Uniprot:Q9FKJ0
Root	Isotig06908	6	31	-2.498	6.06E-06	TAIR|locus:2175143 - symbol:AT5G60570 "AT5G60570" species:3702 "Arabidopsis thaliana" [GO:0003674 "molecular_function" evidence=ND] [GO:0008150 "biological_process" evidence=ND] [GO:0005634 "nucleus" evidence=IDA] InterPro:IPR006652 Pfam:PF01344 PROSITE:PS50181 SMART:SM00612 GO:GO:0005634 EMBL:CP002688 GenomeReviews:BA000015_GR InterPro:IPR015916 Gene3D:G3DSA:2.130.10.80 EMBL:AB011483 eggNOG:NOG312724 EMBL:AK221729 EMBL:BT029765 IPI:IPI00533154 RefSeq:NP_200865.1 UniGene:At.43004 ProteinModelPortal:Q9FKJ0 SMR:Q9FKJ0 IntAct:Q9FKJ0 PRIDE:Q9FKJ0 EnsemblPlants:AT5G60570.1 GeneID:836178 KEGG:ath:AT5G60570 TAIR:At5g60570 InParanoid:Q9FKJ0 OMA:SSSGRWE PhylomeDB:Q9FKJ0 ProtClustDB:CLSN2916403 Genevestigator:Q9FKJ0 Uniprot:Q9FKJ0

Tissue	Identification	# of reads (stress)	# of reads (control)	Fold Change	p-value	Description
Root	Isotig06911	21	4	2.263	0.000779124	TAIR|locus:2138947 - symbol:ABCI8 "AT4G04770" species:3702 "Arabidopsis thaliana" [GO:0005215 "transporter activity" evidence=ISS] [GO:0009507 "chloroplast" evidence=IDA] [GO:0042626 "ATPase activity, coupled to transmembrane movement of substances" evidence=ISS;IDA] [GO:2000030 "regulation of response to red or far red light" evidence=IMP] [GO:0005515 "protein binding" evidence=IPI] [GO:0006879 "cellular iron ion homeostasis" evidence=IDA;TAS] [GO:0016226 "iron-sulfur cluster assembly" evidence=IGI] [GO:0009570 "chloroplast stroma" evidence=IDA] InterPro:IPR000825 InterPro:IPR010231 Pfam:PF01458 EMBL:CP002687 GenomeReviews:CT486007_GR GO:GO:0009570 GO:GO:0005515 GO:GO:0042626 GO:GO:0006879 GO:GO:0016226 eggNOG:COG0719 HOGENOM:HBG711081 EMBL:AF118223 EMBL:AL161501 EMBL:AY052195 EMBL:AY056410 EMBL:AY142069 EMBL:BK001497 IPI:IPI00526234 PIR:A85060 RefSeq:NP_192386.1 UniGene:At.4040 UniGene:At.67960 ProteinModelPortal:Q9ZS97 IntAct:Q9ZS97 STRING:Q9ZS97 PRIDE:Q9ZS97 EnsemblPlants:AT4G04770.1 GeneID:825814 KEGG:ath:AT4G04770 GeneFarm:2674 TAIR:At4g04770 InParanoid:Q9ZS97 KO:K07033 OMA:CTAPMFD PhylomeDB:Q9ZS97 ProtClustDB:CLSN2685558 ArrayExpress:Q9ZS97 Genevestigator:Q9ZS97 GermOnline:AT4G04770 GO:GO:2000030 TIGRFAMs:TIGR01980 Uniprot:Q9ZS97
Root	Isotig06943	12	62	-2.498	1.57E-10	TAIR|locus:2157022 - symbol:GH9A1 "AT5G49720" species:3702 "Arabidopsis thaliana" [GO:0004553 "hydrolase activity, hydrolyzing O-glycosyl compounds" evidence=ISS] [GO:0009504 "cell plate" evidence=IDA] [GO:0009826 "unidimensional cell growth" evidence=IMP] [GO:0009735 "response to cytokinin stimulus" evidence=IMP] [GO:0048367 "shoot development" evidence=IMP] [GO:0030244 "cellulose biosynthetic process" evidence=IMP;TAS] [GO:0005886 "plasma membrane" evidence=IDA] [GO:0005769 "early endosome" evidence=IDA] [GO:0005794 "Golgi apparatus" evidence=IDA] [GO:0042538 "hyperosmotic salinity response" evidence=IMP] [GO:0043622 "cortical microtubule organization" evidence=IMP] [GO:0008810 "cellulase activity" evidence=ISS] InterPro:IPR001701 InterPro:IPR008928 InterPro:IPR012341 InterPro:IPR018221 Pfam:PF00759 PROSITE:PS00592 PROSITE:PS00698 GO:GO:0016021 GO:GO:0005886 EMBL:CP002688 GenomeReviews:BA000015_GR GO:GO:0009735 GO:GO:0005794 GO:GO:0030245 GO:GO:0009826 GO:GO:0007047 Gene3D:G3DSA:1.50.10.10 SUPFAM:SSF48208 GO:GO:0048367 GO:GO:0005769 GO:GO:0042538 GO:GO:0043622 UniGene:At.23416 GO:GO:0009504 GO:GO:0030244 GO:GO:0008810 CAZy:GH9 EMBL:AB025613 HSSP:P26221 HOGENOM:HBG746347 ProtClustDB:CLSN2679649 EMBL:U37702 EMBL:AF073875 EMBL:AF074092 EMBL:AF074375 EMBL:AY037218 EMBL:BT002221 EMBL:AY086165 EMBL:AK221941 EMBL:AK222193 IPI:IPI00523242 PIR:S71215 RefSeq:NP_199783.1 UniGene:At.21098 ProteinModelPortal:Q38890 SMR:Q38890 STRING:Q38890 PRIDE:Q38890 EnsemblPlants:AT5G49720.1 GeneID:835035 KEGG:ath:AT5G49720 TAIR:At5g49720 eggNOG:NOG257354 InParanoid:Q38890 OMA:DEFIWGG PhylomeDB:Q38890 Genevestigator:Q38890 GermOnline:AT5G49720 Uniprot:Q38890
Root	Isotig06944	12	60	-2.451	4.79E-10	TAIR|locus:2157022 - symbol:GH9A1 "AT5G49720" species:3702 "Arabidopsis thaliana" [GO:0004553 "hydrolase activity, hydrolyzing O-glycosyl compounds" evidence=ISS] [GO:0009504 "cell plate" evidence=IDA] [GO:0009826 "unidimensional cell growth" evidence=IMP] [GO:0009735 "response to cytokinin stimulus" evidence=IMP] [GO:0048367 "shoot development" evidence=IMP] [GO:0030244 "cellulose biosynthetic process" evidence=IMP;TAS] [GO:0005886 "plasma membrane" evidence=IDA] [GO:0005769 "early endosome" evidence=IDA] [GO:0005794 "Golgi apparatus" evidence=IDA] [GO:0042538 "hyperosmotic salinity response" evidence=IMP] [GO:0043622 "cortical microtubule organization" evidence=IMP] [GO:0008810 "cellulase activity" evidence=ISS] InterPro:IPR001701 InterPro:IPR008928 InterPro:IPR012341 InterPro:IPR018221 Pfam:PF00759 PROSITE:PS00592 PROSITE:PS00698 GO:GO:0016021 GO:GO:0005886 EMBL:CP002688 GenomeReviews:BA000015_GR GO:GO:0009735 GO:GO:0005794 GO:GO:0030245 GO:GO:0009826 GO:GO:0007047 Gene3D:G3DSA:1.50.10.10 SUPFAM:SSF48208 GO:GO:0048367 GO:GO:0005769 GO:GO:0042538 GO:GO:0043622 UniGene:At.23416 GO:GO:0009504 GO:GO:0030244 GO:GO:0008810 CAZy:GH9 EMBL:AB025613 HSSP:P26221 HOGENOM:HBG746347 ProtClustDB:CLSN2679649 EMBL:U37702 EMBL:AF073875 EMBL:AF074092 EMBL:AF074375 EMBL:AY037218 EMBL:BT002221 EMBL:AY086165 EMBL:AK221941 EMBL:AK222193 IPI:IPI00523242 PIR:S71215 RefSeq:NP_199783.1 UniGene:At.21098 ProteinModelPortal:Q38890 SMR:Q38890 STRING:Q38890 PRIDE:Q38890 EnsemblPlants:AT5G49720.1 GeneID:835035 KEGG:ath:AT5G49720 TAIR:At5g49720 eggNOG:NOG257354 InParanoid:Q38890 OMA:DEFIWGG PhylomeDB:Q38890 Genevestigator:Q38890 GermOnline:AT5G49720 Uniprot:Q38890
Root	Isotig06957	28	6	2.093	0.000219123	ZFIN|ZDB-GENE-030131-1473 - symbol:ubr3 "ubiquitin protein ligase E3 component n-recognin 3" species:7955 "Danio rerio" [GO:0008270 "zinc ion binding" evidence=IEA] [GO:0046872 "metal ion binding" evidence=IEA]

Tissue	Identification	# of reads (stress)	# of reads (control)	Fold Change	p-value	Description
						InterPro:IPR001841 PROSITE:PS50089 SMART:SM00184 ZFIN:ZDB-GENE-030131-1473 GO:GO:0008270 InterPro:IPR013083 Gene3D:G3DSA:3.30.40.10 GeneTree:ENSGT00530000063055 EMBL:CU639483 EMBL:CU986282 EMBL:FP089512 IPI:IPI00897370 Ensembl:ENSDART00000129091 Bgee:F1QJX5 Uniprot:F1QJX5
Root	Isotig06958	28	5	2.356	7.04E-05	ZFIN|ZDB-GENE-030131-1473 - symbol:ubr3 "ubiquitin protein ligase E3 component n-recognin 3" species:7955 "Danio rerio" [GO:0008270 "zinc ion binding" evidence=IEA] [GO:0046872 "metal ion binding" evidence=IEA] InterPro:IPR001841 PROSITE:PS50089 SMART:SM00184 ZFIN:ZDB-GENE-030131-1473 GO:GO:0008270 InterPro:IPR013083 Gene3D:G3DSA:3.30.40.10 GeneTree:ENSGT00530000063055 EMBL:CU639483 EMBL:CU986282 EMBL:FP089512 IPI:IPI00897370 Ensembl:ENSDART00000129091 Bgee:F1QJX5 Uniprot:F1QJX5
Root	Isotig06963	7	24	-1.907	0.000762598	TAIR|locus:2149104 - symbol:ACS "AT5G36880" species:3702 "Arabidopsis thaliana" [GO:0008152 "metabolic process" evidence=ISS] [GO:0009507 "chloroplast" evidence=IDA] [GO:0009570 "chloroplast stroma" evidence=IDA] [GO:0005829 "cytosol" evidence=RCA;IDA] [GO:0003987 "acetate-CoA ligase activity" evidence=IMP] [GO:0006083 "acetate metabolic process" evidence=IMP] InterPro:IPR000873 InterPro:IPR011904 Pfam:PF00501 Prosite:PS00455 GO:GO:0005829 GO:GO:0009570 EMBL:CP002688 HOGENOM:HBG547964 InterPro:IPR020845 InterPro:IPR025110 Pfam:PF13193 eggNOG:COG0365 InterPro:IPR024597 Pfam:PF11930 GO:GO:0016208 GO:GO:0006083 KO:K01895 GO:GO:0003987 PANTHER:PTHR24095:SF42 TIGRFAMs:TIGR02188 EMBL:AF036618 EMBL:AB025605 EMBL:AY045880 EMBL:BT002371 EMBL:AK317115 IPI:IPI00846476 RefSeq:NP_001031974.2 RefSeq:NP_198504.1 UniGene:At.21024 ProteinModelPortal:B9DGD6 STRING:B9DGD6 PRIDE:O49063 EnsemblPlants:AT5G36880.2 GeneID:833655 KEGG:ath:AT5G36880 TAIR:At5g36880 PhylomeDB:B9DGD6 ProtClustDB:PLN02654 Genevestigator:B9DGD6 Uniprot:B9DGD6
Root	Isotig06964	6	24	-2.129	0.00029855	TAIR|locus:2149104 - symbol:ACS "AT5G36880" species:3702 "Arabidopsis thaliana" [GO:0008152 "metabolic process" evidence=ISS] [GO:0009507 "chloroplast" evidence=IDA] [GO:0009570 "chloroplast stroma" evidence=IDA] [GO:0005829 "cytosol" evidence=RCA;IDA] [GO:0003987 "acetate-CoA ligase activity" evidence=IMP] [GO:0006083 "acetate metabolic process" evidence=IMP] InterPro:IPR000873 InterPro:IPR011904 Pfam:PF00501 Prosite:PS00455 GO:GO:0005829 GO:GO:0009570 EMBL:CP002688 HOGENOM:HBG547964 InterPro:IPR020845 InterPro:IPR025110 Pfam:PF13193 eggNOG:COG0365 InterPro:IPR024597 Pfam:PF11930 GO:GO:0016208 GO:GO:0006083 KO:K01895 GO:GO:0003987 PANTHER:PTHR24095:SF42 TIGRFAMs:TIGR02188 EMBL:AF036618 EMBL:AB025605 EMBL:AY045880 EMBL:BT002371 EMBL:AK317115 IPI:IPI00846476 RefSeq:NP_001031974.2 RefSeq:NP_198504.1 UniGene:At.21024 ProteinModelPortal:B9DGD6 STRING:B9DGD6 PRIDE:O49063 EnsemblPlants:AT5G36880.2 GeneID:833655 KEGG:ath:AT5G36880 TAIR:At5g36880 PhylomeDB:B9DGD6 ProtClustDB:PLN02654 Genevestigator:B9DGD6 Uniprot:B9DGD6
Root	Isotig06965	26	5	2.250	0.000195402	TAIR|locus:2034235 - symbol:NTT1 "AT1G80300" species:3702 "Arabidopsis thaliana" [GO:0005471 "ATP:ADP antiporter activity" evidence=ISS] [GO:0006810 "transport" evidence=IEA] [GO:0009941 "chloroplast envelope" evidence=IDA] [GO:0016020 "membrane" evidence=IDA] InterPro:IPR004667 Pfam:PF03219 GO:GO:0016021 EMBL:CP002684 GenomeReviews:CT485782_GR GO:GO:0005524 GO:GO:0031969 GO:GO:0005471 EMBL:AC018848 EMBL:Z49227 EMBL:AF428316 EMBL:AY045903 IPI:IPI00527573 PIR:E96834 PIR:S68205 RefSeq:NP_178146.1 UniGene:At.23897 UniGene:At.72668 ProteinModelPortal:Q39002 IntAct:Q39002 STRING:Q39002 TCDB:2.A.12.2.1 PRIDE:Q39002 EnsemblPlants:AT1G80300.1 GeneID:844370 KEGG:ath:AT1G80300 GeneFarm:3067 TAIR:At1g80300 eggNOG:COG3202 HOGENOM:HBG586333 InParanoid:Q39002 OMA:AYPNFKW PhylomeDB:Q39002 ProtClustDB:CLSN2679486 ArrayExpress:Q39002 Genevestigator:Q39002 GermOnline:AT1G80300 TIGRFAMs:TIGR00769 Uniprot:Q39002
Root	Isotig06966	27	5	2.304	0.000117535	TAIR|locus:2034235 - symbol:NTT1 "AT1G80300" species:3702 "Arabidopsis thaliana" [GO:0005471 "ATP:ADP antiporter activity" evidence=ISS] [GO:0006810 "transport" evidence=IEA] [GO:0009941 "chloroplast envelope" evidence=IDA] [GO:0016020 "membrane" evidence=IDA] InterPro:IPR004667 Pfam:PF03219 GO:GO:0016021 EMBL:CP002684 GenomeReviews:CT485782_GR GO:GO:0005524 GO:GO:0031969 GO:GO:0005471 EMBL:AC018848 EMBL:Z49227 EMBL:AF428316 EMBL:AY045903 IPI:IPI00527573 PIR:E96834 PIR:S68205 RefSeq:NP_178146.1 UniGene:At.23897 UniGene:At.72668 ProteinModelPortal:Q39002 IntAct:Q39002 STRING:Q39002 TCDB:2.A.12.2.1 PRIDE:Q39002 EnsemblPlants:AT1G80300.1 GeneID:844370 KEGG:ath:AT1G80300 GeneFarm:3067 TAIR:At1g80300 eggNOG:COG3202 HOGENOM:HBG586333 InParanoid:Q39002 OMA:AYPNFKW PhylomeDB:Q39002 ProtClustDB:CLSN2679486 ArrayExpress:Q39002 Genevestigator:Q39002 GermOnline:AT1G80300 TIGRFAMs:TIGR00769 Uniprot:Q39002

Tissue	Identification	# of reads (stress)	# of reads (control)	Fold Change	p-value	Description
Root	Isotig06980	46	14	1.587	8.82E-05	TAIR|locus:2154094 - symbol:ALDH12A1 "AT5G62530" species:3702 "Arabidopsis thaliana" [GO:0004028 "3-chloroallyl aldehyde dehydrogenase activity" evidence=ISS] [GO:0003842 "1-pyrroline-5-carboxylate dehydrogenase activity" evidence=IGI;IMP] [GO:0005739 "mitochondrion" evidence=IDA] [GO:0006560 "proline metabolic process" evidence=TAS] [GO:0009507 "chloroplast" evidence=IDA] [GO:0010133 "proline catabolic process to glutamate" evidence=IMP] [GO:0009651 "response to salt stress" evidence=IEP] [GO:0072593 "reactive oxygen species metabolic process" evidence=IMP] [GO:0008270 "zinc ion binding" evidence=IDA] [GO:0050897 "cobalt ion binding" evidence=IDA] InterPro:IPR015590 InterPro:IPR016160 InterPro:IPR016161 InterPro:IPR016162 InterPro:IPR016163 Pfam:PF00171 PROSITE:PS00070 PROSITE:PS00687 EMBL:CP002688 GenomeReviews:BA000015_GR GO:GO:0009507 GO:GO:0005759 GO:GO:0008270 GO:GO:0016620 Gene3D:G3DSA:3.40.309.10 Gene3D:G3DSA:3.40.605.10 SUPFAM:SSF53720 GO:GO:0009651 GO:GO:0050897 GO:GO:0072593 EMBL:AY039787 EMBL:AB015469 EMBL:AY065072 EMBL:BT000746 EMBL:BT010391 IPI:IPI00520602 RefSeq:NP_568955.1 UniGene:At.75205 UniGene:At.9309 ProteinModelPortal:Q8VZC3 IntAct:Q8VZC3 STRING:Q8VZC3 PRIDE:Q8VZC3 EnsemblPlants:AT5G62530.1 GeneID:836373 KEGG:ath:AT5G62530 GeneFarm:4315 TAIR:At5g62530 eggNOG:COG4230 HOGENOM:HBG317230 InParanoid:Q8VZC3 KO:K00294 OMA:DAYACSG PhylomeDB:Q8VZC3 ProtClustDB:CLSN2690065 ArrayExpress:Q8VZC3 Genevestigator:Q8VZC3 GermOnline:AT5G62530 GO:GO:0003842 GO:GO:0010133 Uniprot:Q8VZC3
Root	Isotig06997	7	39	-2.607	2.03E-07	TAIR|locus:2044340 - symbol:AT2G46750 species:3702 "Arabidopsis thaliana" [GO:0003824 "catalytic activity" evidence=IEA] [GO:0003885 "D-arabinono-1,4-lactone oxidase activity" evidence=IEA] [GO:0016020 "membrane" evidence=IEA] [GO:0016491 "oxidoreductase activity" evidence=IEA] [GO:0050660 "flavin adenine dinucleotide binding" evidence=IEA] [GO:0055114 "oxidation-reduction process" evidence=IEA] InterPro:IPR006094 InterPro:IPR007173 InterPro:IPR016166 InterPro:IPR016168 Pfam:PF01565 Pfam:PF04030 PROSITE:PS51387 EMBL:CP002685 GO:GO:0016020 GO:GO:0050660 GO:GO:0008762 Gene3D:G3DSA:3.30.465.20 SUPFAM:SSF56176 GO:GO:0003885 ProtClustDB:CLSN2683562 InterPro:IPR010030 TIGRFAMs:TIGR01677 EMBL:BT010596 IPI:IPI00538609 RefSeq:NP_182198.2 UniGene:At.36462 ProteinModelPortal:Q6NQ66 PRIDE:Q6NQ66 EnsemblPlants:AT2G46750.1 GeneID:819288 KEGG:ath:AT2G46750 TAIR:At2g46750 InParanoid:Q6NQ66 OMA:LELHYGI PhylomeDB:Q6NQ66 Genevestigator:Q6NQ66 Uniprot:Q6NQ66
Root	Isotig07001	83	0	7.246	1.90E-19	No hit
Root	Isotig07002	83	0	7.246	1.90E-19	No hit
Root	Isotig07019	36	7	2.234	1.28E-05	TAIR|locus:2075845 - symbol:SCPL49 "SERINE CARBOXYPEPTIDASE-LIKE 49" species:3702 "Arabidopsis thaliana" [GO:0004185 "serine-type carboxypeptidase activity" evidence=IEA;ISS] [GO:0006508 "proteolysis" evidence=IEA;ISS] [GO:0005773 "vacuole" evidence=IDA] [GO:0005829 "cytosol" evidence=IDA] InterPro:IPR001563 InterPro:IPR018202 Pfam:PF00450 PRINTS:PR00724 PROSITE:PS00131 PROSITE:PS00560 GO:GO:0005829 GO:GO:0005773 EMBL:CP002686 GenomeReviews:BA000014_GR GO:GO:0005576 GO:GO:0006508 EMBL:AC011560 GO:GO:0004185 eggNOG:COG2939 KO:K13289 PANTHER:PTHR11802 HOGENOM:HBG588032 ProtClustDB:CLSN2684010 EMBL:M81130 EMBL:AY091767 EMBL:AY149954 EMBL:Z25955 EMBL:Z26528 IPI:IPI00547765 RefSeq:NP_187652.1 UniGene:At.20528 UniGene:At.45878 UniGene:At.72674 ProteinModelPortal:P32826 SMR:P32826 STRING:P32826 MEROPS:S10.A45 PRIDE:P32826 EnsemblPlants:AT3G10410.1 GeneID:820205 KEGG:ath:AT3G10410 TAIR:At3g10410 InParanoid:P32826 OMA:AYPDYAL PhylomeDB:P32826 ArrayExpress:P32826 Genevestigator:P32826 GermOnline:AT3G10410 Uniprot:P32826
Root	Isotig07020	34	7	2.151	3.42E-05	TAIR|locus:2075845 - symbol:SCPL49 "SERINE CARBOXYPEPTIDASE-LIKE 49" species:3702 "Arabidopsis thaliana" [GO:0004185 "serine-type carboxypeptidase activity" evidence=IEA;ISS] [GO:0006508 "proteolysis" evidence=IEA;ISS] [GO:0005773 "vacuole" evidence=IDA] [GO:0005829 "cytosol" evidence=IDA] InterPro:IPR001563 InterPro:IPR018202 Pfam:PF00450 PRINTS:PR00724 PROSITE:PS00131 PROSITE:PS00560 GO:GO:0005829 GO:GO:0005773 EMBL:CP002686 GenomeReviews:BA000014_GR GO:GO:0005576 GO:GO:0006508 EMBL:AC011560 GO:GO:0004185 eggNOG:COG2939 KO:K13289 PANTHER:PTHR11802 HOGENOM:HBG588032 ProtClustDB:CLSN2684010 EMBL:M81130 EMBL:AY091767 EMBL:AY149954 EMBL:Z25955 EMBL:Z26528 IPI:IPI00547765 RefSeq:NP_187652.1 UniGene:At.20528 UniGene:At.45878 UniGene:At.72674 ProteinModelPortal:P32826 SMR:P32826 STRING:P32826 MEROPS:S10.A45 PRIDE:P32826 EnsemblPlants:AT3G10410.1 GeneID:820205 KEGG:ath:AT3G10410 TAIR:At3g10410 InParanoid:P32826

Tissue	Identification	# of reads (stress)	# of reads (control)	Fold Change	p-value	Description
						OMA:AYPDYAL PhylomeDB:P32826 ArrayExpress:P32826 Genevestigator:P32826 GermOnline:AT3G10410 Uniprot:P32826
Root	Isotig07025	52	20	1.250	0.000501794	TAIR|locus:2076371 - symbol:AT3G58610 "AT3G58610" species:3702 "Arabidopsis thaliana" [GO:0004455 "ketol-acid reductoisomerase activity" evidence=ISS] [GO:0009082 "branched chain family amino acid biosynthetic process" evidence=ISS] [GO:0009941 "chloroplast envelope" evidence=IDA] [GO:0005739 "mitochondrion" evidence=IDA] [GO:0009507 "chloroplast" evidence=IDA] [GO:0009570 "chloroplast stroma" evidence=IDA] [GO:0005618 "cell wall" evidence=IDA] [GO:0046686 "response to cadmium ion" evidence=IEP] [GO:0048046 "apoplast" evidence=IDA] [GO:0005507 "copper ion binding" evidence=IDA] [GO:0005829 "cytosol" evidence=RCA] InterPro:IPR000506 InterPro:IPR008927 InterPro:IPR013023 InterPro:IPR013116 InterPro:IPR013328 InterPro:IPR016040 InterPro:IPR016206 Pfam:PF01450 Pfam:PF07991 PIRSF:PIRSF000118 GO:GO:0005739 GO:GO:0048046 GO:GO:0009570 GO:GO:0046686 GO:GO:0005618 EMBL:CP002686 GenomeReviews:BA000014_GR GO:GO:0000166 Gene3D:G3DSA:3.40.50.720 Gene3D:G3DSA:1.10.1040.10 SUPFAM:SSF48179 GO:GO:0009941 GO:GO:0005507 GO:GO:0050662 GO:GO:0009082 EMBL:AL137082 UniGene:At.480 EMBL:X68150 EMBL:X69880 EMBL:AF324671 EMBL:AF329500 EMBL:AY062094 EMBL:AY065398 EMBL:AY096556 EMBL:BT000669 EMBL:BT000822 IPI:IPI00533630 PIR:S30145 PIR:T45681 RefSeq:NP_001078309.1 RefSeq:NP_001190127.1 RefSeq:NP_191420.1 UniGene:At.46637 UniGene:At.69012 ProteinModelPortal:Q05758 SMR:Q05758 STRING:Q05758 PRIDE:Q05758 EnsemblPlants:AT3G58610.1 EnsemblPlants:AT3G58610.2 EnsemblPlants:AT3G58610.3 GeneID:825030 KEGG:ath:AT3G58610 GeneFarm:4243 TAIR:At3g58610 eggNOG:COG0059 HOGENOM:HBG743920 InParanoid:Q05758 KO:K00053 OMA:EAYSAAY PhylomeDB:Q05758 ProtClustDB:CLSN2684737 ArrayExpress:Q05758 Genevestigator:Q05758 GermOnline:AT3G58610 GO:GO:0004455 PANTHER:PTHR21371 Uniprot:Q05758
Root	Isotig07026	52	20	1.250	0.000501794	TAIR|locus:2076371 - symbol:AT3G58610 "AT3G58610" species:3702 "Arabidopsis thaliana" [GO:0004455 "ketol-acid reductoisomerase activity" evidence=ISS] [GO:0009082 "branched chain family amino acid biosynthetic process" evidence=ISS] [GO:0009941 "chloroplast envelope" evidence=IDA] [GO:0005739 "mitochondrion" evidence=IDA] [GO:0009507 "chloroplast" evidence=IDA] [GO:0009570 "chloroplast stroma" evidence=IDA] [GO:0005618 "cell wall" evidence=IDA] [GO:0046686 "response to cadmium ion" evidence=IEP] [GO:0048046 "apoplast" evidence=IDA] [GO:0005507 "copper ion binding" evidence=IDA] [GO:0005829 "cytosol" evidence=RCA] InterPro:IPR000506 InterPro:IPR008927 InterPro:IPR013023 InterPro:IPR013116 InterPro:IPR013328 InterPro:IPR016040 InterPro:IPR016206 Pfam:PF01450 Pfam:PF07991 PIRSF:PIRSF000118 GO:GO:0005739 GO:GO:0048046 GO:GO:0009570 GO:GO:0046686 GO:GO:0005618 EMBL:CP002686 GenomeReviews:BA000014_GR GO:GO:0000166 Gene3D:G3DSA:3.40.50.720 Gene3D:G3DSA:1.10.1040.10 SUPFAM:SSF48179 GO:GO:0009941 GO:GO:0005507 GO:GO:0050662 GO:GO:0009082 EMBL:AL137082 UniGene:At.480 EMBL:X68150 EMBL:X69880 EMBL:AF324671 EMBL:AF329500 EMBL:AY062094 EMBL:AY065398 EMBL:AY096556 EMBL:BT000669 EMBL:BT000822 IPI:IPI00533630 PIR:S30145 PIR:T45681 RefSeq:NP_001078309.1 RefSeq:NP_001190127.1 RefSeq:NP_191420.1 UniGene:At.46637 UniGene:At.69012 ProteinModelPortal:Q05758 SMR:Q05758 STRING:Q05758 PRIDE:Q05758 EnsemblPlants:AT3G58610.1 EnsemblPlants:AT3G58610.2 EnsemblPlants:AT3G58610.3 GeneID:825030 KEGG:ath:AT3G58610 GeneFarm:4243 TAIR:At3g58610 eggNOG:COG0059 HOGENOM:HBG743920 InParanoid:Q05758 KO:K00053 OMA:EAYSAAY PhylomeDB:Q05758 ProtClustDB:CLSN2684737 ArrayExpress:Q05758 Genevestigator:Q05758 GermOnline:AT3G58610 GO:GO:0004455 PANTHER:PTHR21371 Uniprot:Q05758
Root	Isotig07033	28	56	-1.129	0.000494096	TAIR|locus:2007357 - symbol:NPC1 "non-specific phospholipase C1" species:3702 "Arabidopsis thaliana" [GO:0016788 "hydrolase activity, acting on ester bonds" evidence=IEA;ISS] InterPro:IPR007312 Pfam:PF04185 EMBL:CP002684 GenomeReviews:CT485782_GR GO:GO:0016788 eggNOG:COG3511 KO:K01114 ProtClustDB:CLSN2683426 EMBL:AY124005 EMBL:BT020583 EMBL:AB084291 IPI:IPI00522350 RefSeq:NP_172203.2 UniGene:At.43894 ProteinModelPortal:Q8L7Y9 STRING:Q8L7Y9 PRIDE:Q8L7Y9 EnsemblPlants:AT1G07230.1 GeneID:837234 KEGG:ath:AT1G07230 TAIR:At1g07230 InParanoid:Q8L7Y9 PhylomeDB:Q8L7Y9 Genevestigator:Q8L7Y9 Uniprot:Q8L7Y9
Root	Isotig07034	28	56	-1.129	0.000494096	TAIR|locus:2007357 - symbol:NPC1 "non-specific phospholipase C1" species:3702 "Arabidopsis thaliana" [GO:0016788 "hydrolase activity, acting on ester bonds" evidence=IEA;ISS] InterPro:IPR007312 Pfam:PF04185 EMBL:CP002684 GenomeReviews:CT485782_GR GO:GO:0016788 eggNOG:COG3511 KO:K01114 ProtClustDB:CLSN2683426 EMBL:AY124005 EMBL:BT020583 EMBL:AB084291 IPI:IPI00522350 RefSeq:NP_172203.2 UniGene:At.43894 ProteinModelPortal:Q8L7Y9 STRING:Q8L7Y9 PRIDE:Q8L7Y9

Tissue	Identification	# of reads (stress)	# of reads (control)	Fold Change	p-value	Description
						EnsemblPlants:AT1G07230.1 GeneID:837234 KEGG:ath:AT1G07230 TAIR:At1g07230 InParanoid:Q8L7Y9 PhylomeDB:Q8L7Y9 Genevestigator:Q8L7Y9 Uniprot:Q8L7Y9
Root	Isotig07037	5	24	-2.392	0.000103464	TAIR|locus:2084500 - symbol:AT3G53960 "AT3G53960" species:3702 "Arabidopsis thaliana" [GO:0005215 "transporter activity" evidence=ISS] [GO:0006857 "oligopeptide transport" evidence=IEA;ISS] [GO:0016020 "membrane" evidence=ISS] [GO:0005886 "plasma membrane" evidence=IDA] InterPro:IPR000109 InterPro:IPR018456 Pfam:PF00854 PROSITE:PS01022 PROSITE:PS01023 GO:GO:0016021 GO:GO:0005886 EMBL:CP002686 GenomeReviews:BA000014_GR GO:GO:0005215 InterPro:IPR016196 SUPFAM:SSF103473 EMBL:AL132960 eggNOG:COG3104 OMA:GAFLMAV GO:GO:0006857 EMBL:AF361587 IPI:IPI00532128 PIR:T45936 RefSeq:NP_190964.2 UniGene:At.27522 ProteinModelPortal:Q9M331 PRIDE:Q9M331 EnsemblPlants:AT3G53960.1 GeneID:824563 KEGG:ath:AT3G53960 TAIR:At3g53960 InParanoid:Q9M331 PhylomeDB:Q9M331 Genevestigator:Q9M331 Uniprot:Q9M331
Root	Isotig07038	5	21	-2.199	0.000555521	TAIR|locus:2084500 - symbol:AT3G53960 "AT3G53960" species:3702 "Arabidopsis thaliana" [GO:0005215 "transporter activity" evidence=ISS] [GO:0006857 "oligopeptide transport" evidence=IEA;ISS] [GO:0016020 "membrane" evidence=ISS] [GO:0005886 "plasma membrane" evidence=IDA] InterPro:IPR000109 InterPro:IPR018456 Pfam:PF00854 PROSITE:PS01022 PROSITE:PS01023 GO:GO:0016021 GO:GO:0005886 EMBL:CP002686 GenomeReviews:BA000014_GR GO:GO:0005215 InterPro:IPR016196 SUPFAM:SSF103473 EMBL:AL132960 eggNOG:COG3104 OMA:GAFLMAV GO:GO:0006857 EMBL:AF361587 IPI:IPI00532128 PIR:T45936 RefSeq:NP_190964.2 UniGene:At.27522 ProteinModelPortal:Q9M331 PRIDE:Q9M331 EnsemblPlants:AT3G53960.1 GeneID:824563 KEGG:ath:AT3G53960 TAIR:At3g53960 InParanoid:Q9M331 PhylomeDB:Q9M331 Genevestigator:Q9M331 Uniprot:Q9M331
Root	Isotig07041	270	365	-0.564	9.51E-07	TAIR|locus:2094473 - symbol:AT3G25570 species:3702 "Arabidopsis thaliana" [GO:0004014 "adenosylmethionine decarboxylase activity" evidence=IEA;ISS] [GO:0005575 "cellular_component" evidence=ND] [GO:0006597 "spermine biosynthetic process" evidence=IEA] [GO:0008295 "spermidine biosynthetic process" evidence=IEA] InterPro:IPR001985 InterPro:IPR016067 InterPro:IPR018166 InterPro:IPR018167 Pfam:PF01536 PIRSF:PIRSF001355 PROSITE:PS01336 EMBL:CP002686 GenomeReviews:BA000014_GR eggNOG:NOG77566 HOGENOM:HBG610629 KO:K01611 ProtClustDB:PLN02524 GO:GO:0004014 GO:GO:0008295 GO:GO:0006597 Gene3D:G3DSA:3.60.90.10 PANTHER:PTHR11570 SUPFAM:SSF56276 TIGRFAMs:TIGR00535 HSSP:P17707 OMA:TSELLMY EMBL:AB025639 EMBL:BT012654 IPI:IPI00546639 RefSeq:NP_001189972.1 RefSeq:NP_189184.1 UniGene:At.49653 ProteinModelPortal:Q9LSU6 SMR:Q9LSU6 STRING:Q9LSU6 PRIDE:Q9LSU6 EnsemblPlants:AT3G25570.1 EnsemblPlants:AT3G25570.2 GeneID:822144 KEGG:ath:AT3G25570 TAIR:At3g25570 InParanoid:Q9LSU6 PhylomeDB:Q9LSU6 Genevestigator:Q9LSU6 Uniprot:Q9LSU6
Root	Isotig07042	237	317	-0.549	8.38E-06	TAIR|locus:2094473 - symbol:AT3G25570 species:3702 "Arabidopsis thaliana" [GO:0004014 "adenosylmethionine decarboxylase activity" evidence=IEA;ISS] [GO:0005575 "cellular_component" evidence=ND] [GO:0006597 "spermine biosynthetic process" evidence=IEA] [GO:0008295 "spermidine biosynthetic process" evidence=IEA] InterPro:IPR001985 InterPro:IPR016067 InterPro:IPR018166 InterPro:IPR018167 Pfam:PF01536 PIRSF:PIRSF001355 PROSITE:PS01336 EMBL:CP002686 GenomeReviews:BA000014_GR eggNOG:NOG77566 HOGENOM:HBG610629 KO:K01611 ProtClustDB:PLN02524 GO:GO:0004014 GO:GO:0008295 GO:GO:0006597 Gene3D:G3DSA:3.60.90.10 PANTHER:PTHR11570 SUPFAM:SSF56276 TIGRFAMs:TIGR00535 HSSP:P17707 OMA:TSELLMY EMBL:AB025639 EMBL:BT012654 IPI:IPI00546639 RefSeq:NP_001189972.1 RefSeq:NP_189184.1 UniGene:At.49653 ProteinModelPortal:Q9LSU6 SMR:Q9LSU6 STRING:Q9LSU6 PRIDE:Q9LSU6 EnsemblPlants:AT3G25570.1 EnsemblPlants:AT3G25570.2 GeneID:822144 KEGG:ath:AT3G25570 TAIR:At3g25570 InParanoid:Q9LSU6 PhylomeDB:Q9LSU6 Genevestigator:Q9LSU6 Uniprot:Q9LSU6
Root	Isotig07043	13	38	-1.676	0.000106259	TAIR|locus:2059456 - symbol:ALDH6B2 "AT2G14170" species:3702 "Arabidopsis thaliana" [GO:0004028 "3-chloroallyl aldehyde dehydrogenase activity" evidence=ISS] [GO:0005739 "mitochondrion" evidence=IDA] [GO:0006979 "response to oxidative stress" evidence=IDA] [GO:0005507 "copper ion binding" evidence=IDA] InterPro:IPR010061 InterPro:IPR015590 InterPro:IPR016160 InterPro:IPR016161 InterPro:IPR016162 InterPro:IPR016163 Pfam:PF00171 PROSITE:PS00070 PROSITE:PS00687 GO:GO:0005739 EMBL:CP002685 GenomeReviews:CT485783_GR GO:GO:0006979 eggNOG:COG1012 Gene3D:G3DSA:3.40.309.10 Gene3D:G3DSA:3.40.605.10 SUPFAM:SSF53720 HOGENOM:HBG752218 GO:GO:0005507 KO:K00140 GO:GO:0004491 PANTHER:PTHR11699:SF27 TIGRFAMs:TIGR01722 EMBL:AC007197 EMBL:AK230004

Tissue	Identification	# of reads (stress)	# of reads (control)	Fold Change	p-value	Description
						IPI:IPI00518167 PIR:H84514 RefSeq:NP_179032.1 UniGene:At.43851 ProteinModelPortal:Q0WM29 SMR:Q0WM29 STRING:Q0WM29 PRIDE:Q0WM29 EnsemblPlants:AT2G14170.1 GeneID:815903 KEGG:ath:AT2G14170 GeneFarm:4385 TAIR:At2g14170 InParanoid:Q0WM29 OMA:IISNVKP PhylomeDB:Q0WM29 ProtClustDB:PLN02419 ArrayExpress:Q9SI43 Genevestigator:Q0WM29 Uniprot:Q0WM29
Root	Isotig07044	13	39	-1.714	6.66E-05	TAIR|locus:2059456 - symbol:ALDH6B2 "AT2G14170" species:3702 "Arabidopsis thaliana" [GO:0004028 "3-chloroallyl aldehyde dehydrogenase activity" evidence=ISS] [GO:0005739 "mitochondrion" evidence=IDA] [GO:0006979 "response to oxidative stress" evidence=IDA] [GO:0005507 "copper ion binding" evidence=IDA] InterPro:IPR010061 InterPro:IPR015590 InterPro:IPR016160 InterPro:IPR016161 InterPro:IPR016162 InterPro:IPR016163 Pfam:PF00171 PROSITE:PS00070 PROSITE:PS00687 GO:GO:0005739 EMBL:CP002685 GenomeReviews:CT485783_GR GO:GO:0006979 eggNOG:COG1012 Gene3D:G3DSA:3.40.309.10 Gene3D:G3DSA:3.40.605.10 SUPFAM:SSF53720 HOGENOM:HBG752218 GO:GO:0005507 KO:K00140 GO:GO:0004491 PANTHER:PTHR11699:SF27 TIGRFAMs:TIGR01722 EMBL:AC007197 EMBL:AK230004 IPI:IPI00518167 PIR:H84514 RefSeq:NP_179032.1 UniGene:At.43851 ProteinModelPortal:Q0WM29 SMR:Q0WM29 STRING:Q0WM29 PRIDE:Q0WM29 EnsemblPlants:AT2G14170.1 GeneID:815903 KEGG:ath:AT2G14170 GeneFarm:4385 TAIR:At2g14170 InParanoid:Q0WM29 OMA:IISNVKP PhylomeDB:Q0WM29 ProtClustDB:PLN02419 ArrayExpress:Q9SI43 Genevestigator:Q0WM29 Uniprot:Q0WM29
Root	Isotig07053	11	33	-1.714	0.000243809	TAIR|locus:2087418 - symbol:CCD1 "AT3G63520" species:3702 "Arabidopsis thaliana" [GO:0009414 "response to water deprivation" evidence=IEP] [GO:0016118 "carotenoid catabolic process" evidence=IDA] [GO:0045549 "9-cis-epoxycarotenoid dioxygenase activity" evidence=IDA] [GO:0005773 "vacuole" evidence=IDA] [GO:0005737 "cytoplasm" evidence=IDA] [GO:0016121 "carotene catabolic process" evidence=IMP] [GO:0016124 "xanthophyll catabolic process" evidence=IMP] [GO:0005774 "vacuolar membrane" evidence=IDA] [GO:0005886 "plasma membrane" evidence=IDA] [GO:0009506 "plasmodesma" evidence=IDA] [GO:0005794 "Golgi apparatus" evidence=IDA] GO:GO:0005886 GO:GO:0009506 GO:GO:0005774 EMBL:CP002686 GenomeReviews:BA000014_GR GO:GO:0006950 GO:GO:0046872 EMBL:AL163818 eggNOG:COG3670 InterPro:IPR004294 PANTHER:PTHR10543 Pfam:PF03055 GO:GO:0016121 EMBL:AJ005813 EMBL:BT000436 EMBL:BT002102 IPI:IPI00517969 PIR:T49193 PIR:T51734 RefSeq:NP_191911.1 UniGene:At.22615 UniGene:At.43820 ProteinModelPortal:O65572 SMR:O65572 STRING:O65572 PRIDE:O65572 EnsemblPlants:AT3G63520.1 GeneID:825527 KEGG:ath:AT3G63520 TAIR:At3g63520 HOGENOM:HBG559594 InParanoid:O65572 KO:K11159 OMA:IKFDLHA PhylomeDB:O65572 ProtClustDB:PLN02491 ArrayExpress:Q8GRI2 Genevestigator:O65572 GO:GO:0045549 GO:GO:0016124 Uniprot:O65572
Root	Isotig07054	11	30	-1.576	0.000984585	TAIR|locus:2087418 - symbol:CCD1 "AT3G63520" species:3702 "Arabidopsis thaliana" [GO:0009414 "response to water deprivation" evidence=IEP] [GO:0016118 "carotenoid catabolic process" evidence=IDA] [GO:0045549 "9-cis-epoxycarotenoid dioxygenase activity" evidence=IDA] [GO:0005773 "vacuole" evidence=IDA] [GO:0005737 "cytoplasm" evidence=IDA] [GO:0016121 "carotene catabolic process" evidence=IMP] [GO:0016124 "xanthophyll catabolic process" evidence=IMP] [GO:0005774 "vacuolar membrane" evidence=IDA] [GO:0005886 "plasma membrane" evidence=IDA] [GO:0009506 "plasmodesma" evidence=IDA] [GO:0005794 "Golgi apparatus" evidence=IDA] GO:GO:0005886 GO:GO:0009506 GO:GO:0005774 EMBL:CP002686 GenomeReviews:BA000014_GR GO:GO:0006950 GO:GO:0046872 EMBL:AL163818 eggNOG:COG3670 InterPro:IPR004294 PANTHER:PTHR10543 Pfam:PF03055 GO:GO:0016121 EMBL:AJ005813 EMBL:BT000436 EMBL:BT002102 IPI:IPI00517969 PIR:T49193 PIR:T51734 RefSeq:NP_191911.1 UniGene:At.22615 UniGene:At.43820 ProteinModelPortal:O65572 SMR:O65572 STRING:O65572 PRIDE:O65572 EnsemblPlants:AT3G63520.1 GeneID:825527 KEGG:ath:AT3G63520 TAIR:At3g63520 HOGENOM:HBG559594 InParanoid:O65572 KO:K11159 OMA:IKFDLHA PhylomeDB:O65572 ProtClustDB:PLN02491 ArrayExpress:Q8GRI2 Genevestigator:O65572 GO:GO:0045549 GO:GO:0016124 Uniprot:O65572
Root	Isotig07057	39	8	2.156	8.81E-06	TAIR|locus:2201036 - symbol:NTMC2T2.1 "AT1G05500" species:3702 "Arabidopsis thaliana" [GO:0003674 "molecular_function" evidence=ND] [GO:0008150 "biological_process" evidence=ND] [GO:0005886 "plasma membrane" evidence=IDA] [GO:0012505 "endomembrane system" evidence=IDA] [GO:0009506 "plasmodesma" evidence=IDA] InterPro:IPR000008 InterPro:IPR008973 Pfam:PF00168 SMART:SM00239 EMBL:CP002684 GenomeReviews:CT485782_GR GO:GO:0005886 GO:GO:0009506 GO:GO:0012505 InterPro:IPR018029 SUPFAM:SSF49562 PROSITE:PS50004 eggNOG:COG5038 InterPro:IPR020477 PRINTS:PR00360 HSSP:P04410 ProtClustDB:CLSN2697228 EMBL:AY140038 EMBL:BT008907 EMBL:AK228581 IPI:IPI00548718 RefSeq:NP_172041.2 UniGene:At.27942 ProteinModelPortal:Q8L706 SMR:Q8L706 PRIDE:Q8L706

Tissue	Identification	# of reads (stress)	# of reads (control)	Fold Change	p-value	Description
						EnsemblPlants:AT1G05500.1 GeneID:837052 KEGG:ath:AT1G05500 TAIR:At1g05500 HOGENOM:HBG320331 InParanoid:Q8L706 PhylomeDB:Q8L706 ArrayExpress:Q8L706 Genevestigator:Q8L706 Uniprot:Q8L706
Root	Isotig07058	41	9	2.059	9.66E-06	TAIR|locus:2201036 - symbol:NTMC2T2.1 "AT1G05500" species:3702 "Arabidopsis thaliana" [GO:0003674 "molecular_function" evidence=ND] [GO:0008150 "biological_process" evidence=ND] [GO:0005886 "plasma membrane" evidence=IDA] [GO:0012505 "endomembrane system" evidence=IDA] [GO:0009506 "plasmodesma" evidence=IDA] InterPro:IPR000008 InterPro:IPR008973 Pfam:PF00168 SMART:SM00239 EMBL:CP002684 GenomeReviews:CT485782_GR GO:GO:0005886 GO:GO:0009506 GO:GO:0012505 InterPro:IPR018029 SUPFAM:SSF49562 PROSITE:PS50004 eggNOG:COG5038 InterPro:IPR020477 PRINTS:PR00360 HSSP:P04410 ProtClustDB:CLSN2697228 EMBL:AY140038 EMBL:BT008907 EMBL:AK228581 IPI:IPI00548718 RefSeq:NP_172041.2 UniGene:At.27942 ProteinModelPortal:Q8L706 SMR:Q8L706 PRIDE:Q8L706 EnsemblPlants:AT1G05500.1 GeneID:837052 KEGG:ath:AT1G05500 TAIR:At1g05500 HOGENOM:HBG320331 InParanoid:Q8L706 PhylomeDB:Q8L706 ArrayExpress:Q8L706 Genevestigator:Q8L706 Uniprot:Q8L706
Root	Isotig07067	29	7	1.922	0.000375107	SGD|S000028422 - symbol:TAR1 "Mitochondrial protein potentially involved in respiratory metabolism" species:4932 "Saccharomyces cerevisiae" [GO:0003674 "molecular_function" evidence=ND] [GO:0043457 "regulation of cellular respiration" evidence=IGI] [GO:0005739 "mitochondrion" evidence=IEA;IMP] SGD:S000028422 GO:GO:0005739 EMBL:BK006945 EMBL:U53879 GO:GO:0043457 EMBL:Z73326 EMBL:AF479964 RefSeq:NP_690845.1 ProteinModelPortal:Q8TGM6 STRING:Q8TGM6 EnsemblFungi:YLR154W-C GeneID:1466405 KEGG:sce:YLR154W-C CYGD:YLR154w-c eggNOG:NOG79861 OMA:SCSKEHR OrthoDB:EOG466ZX8 NextBio:967146 Genevestigator:Q8TGM6 GermOnline:YLR154W-C Uniprot:Q8TGM6
Root	Isotig07068	23	3	2.810	7.16E-05	No hit
Root	Isotig07075	25	1	4.515	7.40E-07	TAIR|locus:2009580 - symbol:AT1G53210 species:3702 "Arabidopsis thaliana" [GO:0005509 "calcium ion binding" evidence=IEA;ISS] [GO:0055085 "transmembrane transport" evidence=IEA] [GO:0016020 "membrane" evidence=ISS;IDA] [GO:0005773 "vacuole" evidence=IDA] [GO:0000325 "plant-type vacuole" evidence=IDA] [GO:0005774 "vacuolar membrane" evidence=IDA] [GO:0009506 "plasmodesma" evidence=IDA] InterPro:IPR002048 InterPro:IPR004837 InterPro:IPR011992 Pfam:PF01699 SMART:SM00054 Prosite:PS00018 GO:GO:0016021 EMBL:CP002684 GO:GO:0009506 GO:GO:0005774 GO:GO:0005509 InterPro:IPR018247 InterPro:IPR018249 Gene3D:G3DSA:1.10.238.10 PROSITE:PS50222 GO:GO:0055085 GO:GO:0000325 EMBL:AY099550 IPI:IPI00521295 RefSeq:NP_564623.2 UniGene:At.23754 ProteinModelPortal:Q8L636 SMR:Q8L636 PRIDE:Q8L636 EnsemblPlants:AT1G53210.1 GeneID:841755 KEGG:ath:AT1G53210 TAIR:At1g53210 InParanoid:Q8L636 OMA:CEQTYGF PhylomeDB:Q8L636 ProtClustDB:CLSN2680479 Genevestigator:Q8L636 Uniprot:Q8L636
Root	Isotig07076	25	1	4.515	7.40E-07	TAIR|locus:2009580 - symbol:AT1G53210 species:3702 "Arabidopsis thaliana" [GO:0005509 "calcium ion binding" evidence=IEA;ISS] [GO:0055085 "transmembrane transport" evidence=IEA] [GO:0016020 "membrane" evidence=ISS;IDA] [GO:0005773 "vacuole" evidence=IDA] [GO:0000325 "plant-type vacuole" evidence=IDA] [GO:0005774 "vacuolar membrane" evidence=IDA] [GO:0009506 "plasmodesma" evidence=IDA] InterPro:IPR002048 InterPro:IPR004837 InterPro:IPR011992 Pfam:PF01699 SMART:SM00054 Prosite:PS00018 GO:GO:0016021 EMBL:CP002684 GO:GO:0009506 GO:GO:0005774 GO:GO:0005509 InterPro:IPR018247 InterPro:IPR018249 Gene3D:G3DSA:1.10.238.10 PROSITE:PS50222 GO:GO:0055085 GO:GO:0000325 EMBL:AY099550 IPI:IPI00521295 RefSeq:NP_564623.2 UniGene:At.23754 ProteinModelPortal:Q8L636 SMR:Q8L636 PRIDE:Q8L636 EnsemblPlants:AT1G53210.1 GeneID:841755 KEGG:ath:AT1G53210 TAIR:At1g53210 InParanoid:Q8L636 OMA:CEQTYGF PhylomeDB:Q8L636 ProtClustDB:CLSN2680479 Genevestigator:Q8L636 Uniprot:Q8L636
Root	Isotig07107	43	1	5.297	2.89E-11	TAIR|locus:2164047 - symbol:AT5G53770 "AT5G53770" species:3702 "Arabidopsis thaliana" [GO:0003676 "nucleic acid binding" evidence=ISS] [GO:0005575 "cellular_component" evidence=ND] [GO:0008150 "biological_process" evidence=ND] [GO:0016779 "nucleotidyltransferase activity" evidence=IEA] InterPro:IPR002934 Pfam:PF01909 Pfam:PF03828 EMBL:CP002688 eggNOG:COG5260 InterPro:IPR002058 GO:GO:0016779 EMBL:BT002764 IPI:IPI00522291 RefSeq:NP_568798.1 UniGene:At.6981 ProteinModelPortal:Q84WU1 SMR:Q84WU1 PRIDE:Q84WU1 EnsemblPlants:AT5G53770.1 GeneID:835458 KEGG:ath:AT5G53770 TAIR:At5g53770 InParanoid:Q84WU1 OMA:IVDFCDF PhylomeDB:Q84WU1 ProtClustDB:CLSN2690011 ArrayExpress:Q84WU1 Genevestigator:Q84WU1 Uniprot:Q84WU1
Root	Isotig07108	43	1	5.297	2.89E-11	TAIR|locus:2164047 - symbol:AT5G53770 "AT5G53770" species:3702 "Arabidopsis thaliana" [GO:0003676 "nucleic acid binding" evidence=ISS] [GO:0005575 "cellular_component" evidence=ND] [GO:0008150 "biological_process"

Tissue	Identification	# of reads (stress)	# of reads (control)	Fold Change	p-value	Description
						evidence=ND] [GO:0016779 "nucleotidyltransferase activity" evidence=IEA] InterPro:IPR002934 Pfam:PF01909 Pfam:PF03828 EMBL:CP002688 eggNOG:COG5260 InterPro:IPR002058 GO:GO:0016779 EMBL:BT002764 IPI:IPI00522291 RefSeq:NP_568798.1 UniGene:At.6981 ProteinModelPortal:Q84WU1 SMR:Q84WU1 PRIDE:Q84WU1 EnsemblPlants:AT5G53770.1 GeneID:835458 KEGG:ath:AT5G53770 TAIR:At5g53770 InParanoid:Q84WU1 OMA:IVDFCDF PhylomeDB:Q84WU1 ProtClustDB:CLSN2690011 ArrayExpress:Q84WU1 Genevestigator:Q84WU1 Uniprot:Q84WU1
Root	Isotig07109	1	20	-4.451	3.92E-06	TAIR|locus:2171402 - symbol:AAE5 "acyl activating enzyme 5" species:3702 "Arabidopsis thaliana" [GO:0003824 "catalytic activity" evidence=IEA] [GO:0008152 "metabolic process" evidence=IEA] [GO:0005777 "peroxisome" evidence=IDA] InterPro:IPR000873 Pfam:PF00501 Prosite:PS00455 EMBL:CP002688 GO:GO:0005777 InterPro:IPR020845 InterPro:IPR025110 Pfam:PF13193 HSSP:P08659 GO:GO:0003824 EMBL:AF503764 EMBL:AB005242 IPI:IPI00539798 RefSeq:NP_197141.1 UniGene:At.28787 UniGene:At.43181 ProteinModelPortal:Q9FFE6 SMR:Q9FFE6 STRING:Q9FFE6 PRIDE:Q9FFE6 EnsemblPlants:AT5G16370.1 GeneID:831498 KEGG:ath:AT5G16370 TAIR:At5g16370 InParanoid:Q9FFE6 OMA:SCAWKPQ PhylomeDB:Q9FFE6 ProtClustDB:CLSN2913637 Genevestigator:Q9FFE6 Uniprot:Q9FFE6
Root	Isotig07110	1	20	-4.451	3.92E-06	TAIR|locus:2171402 - symbol:AAE5 "acyl activating enzyme 5" species:3702 "Arabidopsis thaliana" [GO:0003824 "catalytic activity" evidence=IEA] [GO:0008152 "metabolic process" evidence=IEA] [GO:0005777 "peroxisome" evidence=IDA] InterPro:IPR000873 Pfam:PF00501 Prosite:PS00455 EMBL:CP002688 GO:GO:0005777 InterPro:IPR020845 InterPro:IPR025110 Pfam:PF13193 HSSP:P08659 GO:GO:0003824 EMBL:AF503764 EMBL:AB005242 IPI:IPI00539798 RefSeq:NP_197141.1 UniGene:At.28787 UniGene:At.43181 ProteinModelPortal:Q9FFE6 SMR:Q9FFE6 STRING:Q9FFE6 PRIDE:Q9FFE6 EnsemblPlants:AT5G16370.1 GeneID:831498 KEGG:ath:AT5G16370 TAIR:At5g16370 InParanoid:Q9FFE6 OMA:SCAWKPQ PhylomeDB:Q9FFE6 ProtClustDB:CLSN2913637 Genevestigator:Q9FFE6 Uniprot:Q9FFE6
Root	Isotig07113	0	14	-4.936	8.24E-05	TAIR|locus:2132609 - symbol:ACR7 "ACT domain repeat 7" species:3702 "Arabidopsis thaliana" [GO:0008152 "metabolic process" evidence=IEA] [GO:0005829 "cytosol" evidence=ISS] [GO:0016597 "amino acid binding" evidence=ISS] InterPro:IPR002912 Pfam:PF01842 EMBL:CP002687 GO:GO:0016597 GO:GO:0008152 EMBL:AF528063 EMBL:BT029176 IPI:IPI00537192 RefSeq:NP_194009.2 UniGene:At.32498 ProteinModelPortal:Q8LJW1 PRIDE:Q8LJW1 EnsemblPlants:AT4G22780.1 GeneID:828377 KEGG:ath:AT4G22780 TAIR:At4g22780 InParanoid:Q8LJW1 OMA:VLADLEC PhylomeDB:Q8LJW1 ProtClustDB:CLSN2682266 Genevestigator:Q8LJW1 Uniprot:Q8LJW1
Root	Isotig07114	0	14	-4.936	8.24E-05	TAIR|locus:2132609 - symbol:ACR7 "ACT domain repeat 7" species:3702 "Arabidopsis thaliana" [GO:0008152 "metabolic process" evidence=IEA] [GO:0005829 "cytosol" evidence=ISS] [GO:0016597 "amino acid binding" evidence=ISS] InterPro:IPR002912 Pfam:PF01842 EMBL:CP002687 GO:GO:0016597 GO:GO:0008152 EMBL:AF528063 EMBL:BT029176 IPI:IPI00537192 RefSeq:NP_194009.2 UniGene:At.32498 ProteinModelPortal:Q8LJW1 PRIDE:Q8LJW1 EnsemblPlants:AT4G22780.1 GeneID:828377 KEGG:ath:AT4G22780 TAIR:At4g22780 InParanoid:Q8LJW1 OMA:VLADLEC PhylomeDB:Q8LJW1 ProtClustDB:CLSN2682266 Genevestigator:Q8LJW1 Uniprot:Q8LJW1
Root	Isotig07123	4	28	-2.936	2.81E-06	TAIR|locus:2039270 - symbol:FLA16 "AT2G35860" species:3702 "Arabidopsis thaliana" [GO:0007155 "cell adhesion" evidence=ISS] Pfam:PF02469 InterPro:IPR000782 EMBL:CP002685 GenomeReviews:CT485783_GR GO:GO:0005576 EMBL:AC007017 Gene3D:G3DSA:2.30.180.10 SMART:SM00554 SUPFAM:SSF82153 PROSITE:PS50213 HOGENOM:HBG595175 ProtClustDB:CLSN2915491 EMBL:AY093189 EMBL:BT006590 IPI:IPI00537255 PIR:H84773 RefSeq:NP_850253.1 UniGene:At.27800 ProteinModelPortal:Q8RWC5 SMR:Q8RWC5 STRING:Q8RWC5 PRIDE:Q8RWC5 EnsemblPlants:AT2G35860.1 GeneID:818159 KEGG:ath:AT2G35860 GeneFarm:3813 TAIR:At2g35860 eggNOG:NOG302692 InParanoid:Q8RWC5 OMA:ATEIKPA PhylomeDB:Q8RWC5 Genevestigator:Q8RWC5 GermOnline:AT2G35860 Uniprot:Q8RWC5
Root	Isotig07124	3	29	-3.402	3.49E-07	TAIR|locus:2164290 - symbol:FLA17 "AT5G06390" species:3702 "Arabidopsis thaliana" [GO:0003674 "molecular_function" evidence=ND] [GO:0007155 "cell adhesion" evidence=ISS] [GO:0005773 "vacuole" evidence=IDA] Pfam:PF02469 InterPro:IPR000782 GO:GO:0005773 EMBL:CP002688 GenomeReviews:BA000015_GR GO:GO:0005576 EMBL:AB006700 Gene3D:G3DSA:2.30.180.10 SMART:SM00554 SUPFAM:SSF82153 PROSITE:PS50213 HOGENOM:HBG595175 eggNOG:NOG302692 EMBL:BT015342 EMBL:BT020253 IPI:IPI00521755 RefSeq:NP_196257.2 UniGene:At.32865 ProteinModelPortal:Q66GR0 SMR:Q66GR0 PRIDE:Q66GR0 EnsemblPlants:AT5G06390.1 GeneID:830527

Tissue	Identification	# of reads (stress)	# of reads (control)	Fold Change	p-value	Description
						KEGG:ath:AT5G06390 GeneFarm:3814 TAIR:At5g06390 InParanoid:Q66GR0 OMA:IQSAMAP PhylomeDB:Q66GR0 ProtClustDB:CLSN2914955 Genevestigator:Q66GR0 Uniprot:Q66GR0
Root	Isotig07131	5	23	-2.331	0.000182103	No hit
Root	Isotig07149	4	24	-2.714	3.10E-05	TAIR|locus:2015791 - symbol:GlcNAc1pUT1 "N-acetylglucosamine-1-phosphate uridylyltransferase 1" species:3702 "Arabidopsis thaliana" [GO:0005886 "plasma membrane" evidence=IDA] [GO:0003977 "UDP-N-acetylglucosamine diphosphorylase activity" evidence=IDA] [GO:0006047 "UDP-N-acetylglucosamine metabolic process" evidence=IDA] [GO:0019276 "UDP-N-acetylgalactosamine metabolic process" evidence=IDA] [GO:0052630 "UDP-N-acetylgalactosamine diphosphorylase activity" evidence=IDA] [GO:0005829 "cytosol" evidence=IDA] InterPro:IPR002618 Pfam:PF01704 EMBL:CP002684 GenomeReviews:CT485782_GR GO:GO:0005829 GO:GO:0005886 GO:GO:0019276 GO:GO:0006047 GO:GO:0003977 HSSP:Q16222 eggNOG:COG4284 HOGENOM:HBG527090 KO:K00972 PANTHER:PTHR11952 ProtClustDB:PLN02435 GO:GO:0052630 OMA:YFDIFRY EMBL:AY053411 IPI:IPI00517047 RefSeq:NP_564372.3 UniGene:At.15935 ProteinModelPortal:Q940S3 SMR:Q940S3 STRING:Q940S3 PRIDE:Q940S3 EnsemblPlants:AT1G31070.2 GeneID:839993 KEGG:ath:AT1G31070 TAIR:At1g31070 InParanoid:Q940S3 PhylomeDB:Q940S3 Genevestigator:Q940S3 Uniprot:Q940S3
Root	Isotig07150	4	24	-2.714	3.10E-05	TAIR|locus:2015791 - symbol:GlcNAc1pUT1 "N-acetylglucosamine-1-phosphate uridylyltransferase 1" species:3702 "Arabidopsis thaliana" [GO:0005886 "plasma membrane" evidence=IDA] [GO:0003977 "UDP-N-acetylglucosamine diphosphorylase activity" evidence=IDA] [GO:0006047 "UDP-N-acetylglucosamine metabolic process" evidence=IDA] [GO:0019276 "UDP-N-acetylgalactosamine metabolic process" evidence=IDA] [GO:0052630 "UDP-N-acetylgalactosamine diphosphorylase activity" evidence=IDA] [GO:0005829 "cytosol" evidence=IDA] InterPro:IPR002618 Pfam:PF01704 EMBL:CP002684 GenomeReviews:CT485782_GR GO:GO:0005829 GO:GO:0005886 GO:GO:0019276 GO:GO:0006047 GO:GO:0003977 HSSP:Q16222 eggNOG:COG4284 HOGENOM:HBG527090 KO:K00972 PANTHER:PTHR11952 ProtClustDB:PLN02435 GO:GO:0052630 OMA:YFDIFRY EMBL:AY053411 IPI:IPI00517047 RefSeq:NP_564372.3 UniGene:At.15935 ProteinModelPortal:Q940S3 SMR:Q940S3 STRING:Q940S3 PRIDE:Q940S3 EnsemblPlants:AT1G31070.2 GeneID:839993 KEGG:ath:AT1G31070 TAIR:At1g31070 InParanoid:Q940S3 PhylomeDB:Q940S3 Genevestigator:Q940S3 Uniprot:Q940S3
Root	Isotig07151	4	31	-3.083	4.53E-07	TAIR|locus:2147117 - symbol:AT5G20950 species:3702 "Arabidopsis thaliana" [GO:0004553 "hydrolase activity, hydrolyzing O-glycosyl compounds" evidence=IEA;ISS] [GO:0005975 "carbohydrate metabolic process" evidence=IEA;ISS] [GO:0005618 "cell wall" evidence=IDA] [GO:0009505 "plant-type cell wall" evidence=IDA] [GO:0016020 "membrane" evidence=IDA] [GO:0005829 "cytosol" evidence=RCA] [GO:0009506 "plasmodesma" evidence=IDA] InterPro:IPR001764 InterPro:IPR002772 Pfam:PF00933 Pfam:PF01915 PRINTS:PR00133 GO:GO:0009506 EMBL:CP002688 GO:GO:0016020 InterPro:IPR017853 SUPFAM:SSF51445 GO:GO:0005975 GO:GO:0004553 GO:GO:0009505 Gene3D:G3DSA:3.40.50.1700 Gene3D:G3DSA:3.20.20.300 SUPFAM:SSF52279 CAZy:GH3 HSSP:Q9XEI3 EMBL:AF462808 EMBL:AY091027 EMBL:AY142679 IPI:IPI00528669 RefSeq:NP_197595.2 RefSeq:NP_851048.1 UniGene:At.23560 ProteinModelPortal:Q8W112 SMR:Q8W112 PRIDE:Q8W112 EnsemblPlants:AT5G20950.1 EnsemblPlants:AT5G20950.2 GeneID:832220 KEGG:ath:AT5G20950 TAIR:At5g20950 InParanoid:Q8W112 OMA:THNGINE PhylomeDB:Q8W112 ProtClustDB:CLSN2690178 ArrayExpress:Q8W112 Genevestigator:Q8W112 Uniprot:Q8W112
Root	Isotig07152	4	23	-2.653	5.61E-05	TAIR|locus:2147117 - symbol:AT5G20950 species:3702 "Arabidopsis thaliana" [GO:0004553 "hydrolase activity, hydrolyzing O-glycosyl compounds" evidence=IEA;ISS] [GO:0005975 "carbohydrate metabolic process" evidence=IEA;ISS] [GO:0005618 "cell wall" evidence=IDA] [GO:0009505 "plant-type cell wall" evidence=IDA] [GO:0016020 "membrane" evidence=IDA] [GO:0005829 "cytosol" evidence=RCA] [GO:0009506 "plasmodesma" evidence=IDA] InterPro:IPR001764 InterPro:IPR002772 Pfam:PF00933 Pfam:PF01915 PRINTS:PR00133 GO:GO:0009506 EMBL:CP002688 GO:GO:0016020 InterPro:IPR017853 SUPFAM:SSF51445 GO:GO:0005975 GO:GO:0004553 GO:GO:0009505 Gene3D:G3DSA:3.40.50.1700 Gene3D:G3DSA:3.20.20.300 SUPFAM:SSF52279 CAZy:GH3 HSSP:Q9XEI3 EMBL:AF462808 EMBL:AY091027 EMBL:AY142679 IPI:IPI00528669 RefSeq:NP_197595.2 RefSeq:NP_851048.1 UniGene:At.23560 ProteinModelPortal:Q8W112 SMR:Q8W112 PRIDE:Q8W112 EnsemblPlants:AT5G20950.1 EnsemblPlants:AT5G20950.2 GeneID:832220 KEGG:ath:AT5G20950 TAIR:At5g20950 InParanoid:Q8W112 OMA:THNGINE PhylomeDB:Q8W112 ProtClustDB:CLSN2690178 ArrayExpress:Q8W112 Genevestigator:Q8W112 Uniprot:Q8W112

Tissue	Identification	# of reads (stress)	# of reads (control)	Fold Change	p-value	Description
Root	Isotig07165	2	17	-3.216	0.000139706	TAIR|locus:2167240 - symbol:GAD "AT5G17330" species:3702 "Arabidopsis thaliana" [GO:0005516 "calmodulin binding" evidence=IDA;TAS;IPI] [GO:0046686 "response to cadmium ion" evidence=IEP] [GO:0005829 "cytosol" evidence=IDA] [GO:0004351 "glutamate decarboxylase activity" evidence=IDA] InterPro:IPR002129 InterPro:IPR010107 InterPro:IPR015421 Pfam:PF00282 PROSITE:PS00392 GO:GO:0005829 GO:GO:0046686 EMBL:CP002688 GenomeReviews:BA000015_GR GO:GO:0030170 InterPro:IPR015424 Gene3D:G3DSA:3.40.640.10 SUPFAM:SSF53383 GO:GO:0006536 GO:GO:0005516 EMBL:AB005238 eggNOG:COG0076 PANTHER:PTHR11999 EMBL:U10034 EMBL:AY094464 EMBL:BT001047 IPI:IPI00530557 RefSeq:NP_197235.1 UniGene:At.25228 PDB:3HBX PDBsum:3HBX ProteinModelPortal:Q42521 SMR:Q42521 STRING:Q42521 PRIDE:Q42521 EnsemblPlants:AT5G17330.1 GeneID:831599 KEGG:ath:AT5G17330 TAIR:At5g17330 HOGENOM:HBG365574 InParanoid:Q42521 KO:K01580 OMA:RRWQNKM PhylomeDB:Q42521 ProtClustDB:CLSN2683665 ArrayExpress:Q42521 Genevestigator:Q42521 GermOnline:AT5G17330 GO:GO:0004351 PANTHER:PTHR11999:SF1 TIGRFAMs:TIGR01788 Uniprot:Q42521
Root	Isotig07166	2	17	-3.216	0.000139706	TAIR|locus:2167240 - symbol:GAD "AT5G17330" species:3702 "Arabidopsis thaliana" [GO:0005516 "calmodulin binding" evidence=IDA;TAS;IPI] [GO:0046686 "response to cadmium ion" evidence=IEP] [GO:0005829 "cytosol" evidence=IDA] [GO:0004351 "glutamate decarboxylase activity" evidence=IDA] InterPro:IPR002129 InterPro:IPR010107 InterPro:IPR015421 Pfam:PF00282 PROSITE:PS00392 GO:GO:0005829 GO:GO:0046686 EMBL:CP002688 GenomeReviews:BA000015_GR GO:GO:0030170 InterPro:IPR015424 Gene3D:G3DSA:3.40.640.10 SUPFAM:SSF53383 GO:GO:0006536 GO:GO:0005516 EMBL:AB005238 eggNOG:COG0076 PANTHER:PTHR11999 EMBL:U10034 EMBL:AY094464 EMBL:BT001047 IPI:IPI00530557 RefSeq:NP_197235.1 UniGene:At.25228 PDB:3HBX PDBsum:3HBX ProteinModelPortal:Q42521 SMR:Q42521 STRING:Q42521 PRIDE:Q42521 EnsemblPlants:AT5G17330.1 GeneID:831599 KEGG:ath:AT5G17330 TAIR:At5g17330 HOGENOM:HBG365574 InParanoid:Q42521 KO:K01580 OMA:RRWQNKM PhylomeDB:Q42521 ProtClustDB:CLSN2683665 ArrayExpress:Q42521 Genevestigator:Q42521 GermOnline:AT5G17330 GO:GO:0004351 PANTHER:PTHR11999:SF1 TIGRFAMs:TIGR01788 Uniprot:Q42521
Root	Isotig07185	0	14	-4.936	8.24E-05	TAIR|locus:2148171 - symbol:CSLD2 "AT5G16910" species:3702 "Arabidopsis thaliana" [GO:0000271 "polysaccharide biosynthetic process" evidence=ISS] [GO:0009832 "plant-type cell wall biogenesis" evidence=ISS] [GO:0016757 "transferase activity, transferring glycosyl groups" evidence=ISS] [GO:0016759 "cellulose synthase activity" evidence=ISS] [GO:0005794 "Golgi apparatus" evidence=IDA] [GO:0005886 "plasma membrane" evidence=IDA] [GO:0030173 "integral to Golgi membrane" evidence=IDA] [GO:0009409 "response to cold" evidence=IEP] [GO:0048767 "root hair elongation" evidence=IMP] InterPro:IPR005150 Pfam:PF03552 GO:GO:0005886 EMBL:CP002688 GenomeReviews:BA000015_GR GO:GO:0030173 GO:GO:0009409 GO:GO:0048767 InterPro:IPR013083 Gene3D:G3DSA:3.30.40.10 GO:GO:0007047 CAZy:GT2 eggNOG:COG1215 GO:GO:0016760 GO:GO:0030244 HOGENOM:HBG744549 ProtClustDB:PLN02248 EMBL:AL391141 IPI:IPI00532872 PIR:T51546 RefSeq:NP_197193.1 UniGene:At.20318 ProteinModelPortal:Q9LFL0 SMR:Q9LFL0 PRIDE:Q9LFL0 EnsemblPlants:AT5G16910.1 GeneID:831554 KEGG:ath:AT5G16910 TAIR:At5g16910 InParanoid:Q9LFL0 OMA:WRIKHQN PhylomeDB:Q9LFL0 Genevestigator:Q9LFL0 Uniprot:Q9LFL0
Root	Isotig07187	28	6	2.093	0.000219123	TAIR|locus:2091618 - symbol:AT3G22270 "AT3G22270" species:3702 "Arabidopsis thaliana" [GO:0003674 "molecular_function" evidence=ND] [GO:0005575 "cellular_component" evidence=ND] [GO:0008150 "biological_process" evidence=ND] GenomeReviews:BA000014_GR HOGENOM:HBG318375 EMBL:AK228960 IPI:IPI00533404 ProteinModelPortal:Q0WPV0 PRIDE:Q0WPV0 TAIR:At3g22270 eggNOG:NOG323978 InParanoid:Q0WPV0 PhylomeDB:Q0WPV0 Genevestigator:Q0WPV0 Uniprot:Q0WPV0
Root	Isotig07188	27	5	2.304	0.000117535	TAIR|locus:2091618 - symbol:AT3G22270 "AT3G22270" species:3702 "Arabidopsis thaliana" [GO:0003674 "molecular_function" evidence=ND] [GO:0005575 "cellular_component" evidence=ND] [GO:0008150 "biological_process" evidence=ND] GenomeReviews:BA000014_GR HOGENOM:HBG318375 EMBL:AK228960 IPI:IPI00533404 ProteinModelPortal:Q0WPV0 PRIDE:Q0WPV0 TAIR:At3g22270 eggNOG:NOG323978 InParanoid:Q0WPV0 PhylomeDB:Q0WPV0 Genevestigator:Q0WPV0 Uniprot:Q0WPV0
Root	Isotig07193	7	28	-2.129	9.37E-05	TAIR|locus:2118791 - symbol:DGK7 "AT4G30340" species:3702 "Arabidopsis thaliana" [GO:0004143 "diacylglycerol kinase activity" evidence=ISS;IDA] [GO:0007205 "activation of protein kinase C activity by G-protein coupled receptor protein signaling pathway" evidence=ISS] [GO:0048364 "root development" evidence=IMP] [GO:0048366 "leaf development" evidence=IMP] [GO:0005886 "plasma membrane" evidence=IDA] InterPro:IPR000756 InterPro:IPR001206 Pfam:PF00609 Pfam:PF00781 PROSITE:PS50146 SMART:SM00045 SMART:SM00046 GO:GO:0005886 GenomeReviews:CT486007_GR GO:GO:0007205 GO:GO:0048364

Tissue	Identification	# of reads (stress)	# of reads (control)	Fold Change	p-value	Description
						GO:GO:0048366 GO:GO:0004143 eggNOG:NOG47311 EMBL:DQ350135 IPI:IPI00538842 ProteinModelPortal:B0KYU1 PRIDE:B0KYU1 TAIR:At4g30340 HOGENOM:HBG317142 PhylomeDB:B0KYU1 Genevestigator:B0KYU1 InterPro:IPR016961 PIRSF:PIRSF030829 Uniprot:B0KYU1
Root	Isotig07194	7	28	-2.129	9.37E-05	TAIR|locus:2118791 - symbol:DGK7 "AT4G30340" species:3702 "Arabidopsis thaliana" [GO:0004143 "diacylglycerol kinase activity" evidence=ISS;IDA] [GO:0007205 "activation of protein kinase C activity by G-protein coupled receptor protein signaling pathway" evidence=ISS] [GO:0048364 "root development" evidence=IMP] [GO:0048366 "leaf development" evidence=IMP] [GO:0005886 "plasma membrane" evidence=IDA] InterPro:IPR000756 InterPro:IPR001206 Pfam:PF00609 Pfam:PF00781 PROSITE:PS50146 SMART:SM00045 SMART:SM00046 GO:GO:0005886 GenomeReviews:CT486007_GR GO:GO:0007205 GO:GO:0048364 GO:GO:0048366 GO:GO:0004143 eggNOG:NOG47311 EMBL:DQ350135 IPI:IPI00538842 ProteinModelPortal:B0KYU1 PRIDE:B0KYU1 TAIR:At4g30340 HOGENOM:HBG317142 PhylomeDB:B0KYU1 Genevestigator:B0KYU1 InterPro:IPR016961 PIRSF:PIRSF030829 Uniprot:B0KYU1
Root	Isotig07217	38	7	2.312	4.70E-06	TAIR|locus:2089521 - symbol:CYP72A15 ""cytochrome P450, family 72, subfamily A, polypeptide 15"" species:3702 "Arabidopsis thaliana" [GO:0004497 "monooxygenase activity" evidence=IEA] [GO:0005506 "iron ion binding" evidence=IEA] [GO:0009055 "electron carrier activity" evidence=IEA] [GO:0019825 "oxygen binding" evidence=ISS] [GO:0020037 "heme binding" evidence=IEA] InterPro:IPR001128 InterPro:IPR002401 Pfam:PF00067 PRINTS:PR00385 PRINTS:PR00463 EMBL:CP002686 GenomeReviews:BA000014_GR GO:GO:0009055 GO:GO:0004497 eggNOG:COG2124 GO:GO:0020037 Gene3D:G3DSA:1.10.630.10 SUPFAM:SSF48264 GO:GO:0016705 HSSP:P14779 HOGENOM:HBG715383 EMBL:AB023038 EMBL:AY050827 EMBL:AY096748 IPI:IPI00542070 RefSeq:NP_188087.1 UniGene:At.19957 UniGene:At.23975 ProteinModelPortal:Q9LUC5 SMR:Q9LUC5 STRING:Q9LUC5 PRIDE:Q9LUC5 EnsemblPlants:AT3G14690.1 GeneID:820697 KEGG:ath:AT3G14690 TAIR:At3g14690 InParanoid:Q9LUC5 OMA:TVITIHP PhylomeDB:Q9LUC5 ProtClustDB:CLSN2684942 ArrayExpress:Q9LUC5 Genevestigator:Q9LUC5 Uniprot:Q9LUC5
Root	Isotig07218	38	5	2.797	3.54E-07	TAIR|locus:2089521 - symbol:CYP72A15 ""cytochrome P450, family 72, subfamily A, polypeptide 15"" species:3702 "Arabidopsis thaliana" [GO:0004497 "monooxygenase activity" evidence=IEA] [GO:0005506 "iron ion binding" evidence=IEA] [GO:0009055 "electron carrier activity" evidence=IEA] [GO:0019825 "oxygen binding" evidence=ISS] [GO:0020037 "heme binding" evidence=IEA] InterPro:IPR001128 InterPro:IPR002401 Pfam:PF00067 PRINTS:PR00385 PRINTS:PR00463 EMBL:CP002686 GenomeReviews:BA000014_GR GO:GO:0009055 GO:GO:0004497 eggNOG:COG2124 GO:GO:0020037 Gene3D:G3DSA:1.10.630.10 SUPFAM:SSF48264 GO:GO:0016705 HSSP:P14779 HOGENOM:HBG715383 EMBL:AB023038 EMBL:AY050827 EMBL:AY096748 IPI:IPI00542070 RefSeq:NP_188087.1 UniGene:At.19957 UniGene:At.23975 ProteinModelPortal:Q9LUC5 SMR:Q9LUC5 STRING:Q9LUC5 PRIDE:Q9LUC5 EnsemblPlants:AT3G14690.1 GeneID:820697 KEGG:ath:AT3G14690 TAIR:At3g14690 InParanoid:Q9LUC5 OMA:TVITIHP PhylomeDB:Q9LUC5 ProtClustDB:CLSN2684942 ArrayExpress:Q9LUC5 Genevestigator:Q9LUC5 Uniprot:Q9LUC5
Root	Isotig07225	5	47	-3.362	1.09E-10	TAIR|locus:2166645 - symbol:AT5G38710 species:3702 "Arabidopsis thaliana" [GO:0004657 "proline dehydrogenase activity" evidence=IEA;IGI;ISS] [GO:0006537 "glutamate biosynthetic process" evidence=IEA;ISS] [GO:0006562 "proline catabolic process" evidence=IEA;IGI;ISS] [GO:0055114 "oxidation-reduction process" evidence=IEA] [GO:0005739 "mitochondrion" evidence=IDA] [GO:0006970 "response to osmotic stress" evidence=IEP] [GO:0009414 "response to water deprivation" evidence=IEP] InterPro:IPR002872 InterPro:IPR015659 Pfam:PF01619 GO:GO:0005739 EMBL:CP002688 GenomeReviews:BA000015_GR GO:GO:0009414 GO:GO:0006970 GO:GO:0006537 GO:GO:0006562 EMBL:AB011478 eggNOG:COG0506 KO:K00318 ProtClustDB:PLN02681 GO:GO:0004657 PANTHER:PTHR13914 EMBL:BT012572 EMBL:AK221601 IPI:IPI00544454 RefSeq:NP_198687.1 UniGene:At.50494 ProteinModelPortal:Q6NKX1 PRIDE:Q6NKX1 EnsemblPlants:AT5G38710.1 GeneID:833862 KEGG:ath:AT5G38710 TAIR:At5g38710 InParanoid:Q6NKX1 OMA:GQTGREL PhylomeDB:Q6NKX1 BioCyc:MetaCyc:AT5G38710-MONOMER Genevestigator:Q6NKX1 Uniprot:Q6NKX1
Root	Isotig07226	4	47	-3.684	2.25E-11	TAIR|locus:2166645 - symbol:AT5G38710 species:3702 "Arabidopsis thaliana" [GO:0004657 "proline dehydrogenase activity" evidence=IEA;IGI;ISS] [GO:0006537 "glutamate biosynthetic process" evidence=IEA;ISS] [GO:0006562 "proline catabolic process" evidence=IEA;IGI;ISS] [GO:0055114 "oxidation-reduction process" evidence=IEA] [GO:0005739 "mitochondrion" evidence=IDA] [GO:0006970 "response to osmotic stress" evidence=IEP] [GO:0009414 "response to water deprivation" evidence=IEP] InterPro:IPR002872 InterPro:IPR015659 Pfam:PF01619 GO:GO:0005739 EMBL:CP002688 GenomeReviews:BA000015_GR

Tissue	Identification	# of reads (stress)	# of reads (control)	Fold Change	p-value	Description
						GO:GO:0009414 GO:GO:0006970 GO:GO:0006537 GO:GO:0006562 EMBL:AB011478 eggNOG:COG0506 KO:K00318 ProtClustDB:PLN02681 GO:GO:0004657 PANTHER:PTHR13914 EMBL:BT012572 EMBL:AK221601 IPI:IPI00544454 RefSeq:NP_198687.1 UniGene:At.50494 ProteinModelPortal:Q6NKX1 PRIDE:Q6NKX1 EnsemblPlants:AT5G38710.1 GeneID:833862 KEGG:ath:AT5G38710 TAIR:At5g38710 InParanoid:Q6NKX1 OMA:GQTGREL PhylomeDB:Q6NKX1 BioCyc:MetaCyc:AT5G38710-MONOMER Genevestigator:Q6NKX1 Uniprot:Q6NKX1
Root	Isotig07229	0	20	-5.451	2.00E-06	TAIR|locus:2161710 - symbol:AT5G55180 species:3702 "Arabidopsis thaliana" [GO:0003824 "catalytic activity" evidence=IEA] [GO:0004553 "hydrolase activity, hydrolyzing O-glycosyl compounds" evidence=IEA;ISS] [GO:0005975 "carbohydrate metabolic process" evidence=IEA] [GO:0043169 "cation binding" evidence=IEA] InterPro:IPR000490 InterPro:IPR013781 Pfam:PF00332 PROSITE:PS00587 GO:GO:0043169 InterPro:IPR017853 Gene3D:G3DSA:3.20.20.80 SUPFAM:SSF51445 GO:GO:0005975 GO:GO:0004553 CAZy:GH17 EMBL:AB010071 HSSP:P15737 CAZy:CBM43 InterPro:IPR012946 Pfam:PF07983 SMART:SM00768 IPI:IPI00531642 ProteinModelPortal:Q9FLP4 SMR:Q9FLP4 PRIDE:Q9FLP4 TAIR:At5g55180 PhylomeDB:Q9FLP4 Genevestigator:Q9FLP4 Uniprot:Q9FLP4
Root	Isotig07230	0	16	-5.129	2.34E-05	TAIR|locus:2161710 - symbol:AT5G55180 species:3702 "Arabidopsis thaliana" [GO:0003824 "catalytic activity" evidence=IEA] [GO:0004553 "hydrolase activity, hydrolyzing O-glycosyl compounds" evidence=IEA;ISS] [GO:0005975 "carbohydrate metabolic process" evidence=IEA] [GO:0043169 "cation binding" evidence=IEA] InterPro:IPR000490 InterPro:IPR013781 Pfam:PF00332 PROSITE:PS00587 GO:GO:0043169 InterPro:IPR017853 Gene3D:G3DSA:3.20.20.80 SUPFAM:SSF51445 GO:GO:0005975 GO:GO:0004553 CAZy:GH17 EMBL:AB010071 HSSP:P15737 CAZy:CBM43 InterPro:IPR012946 Pfam:PF07983 SMART:SM00768 IPI:IPI00531642 ProteinModelPortal:Q9FLP4 SMR:Q9FLP4 PRIDE:Q9FLP4 TAIR:At5g55180 PhylomeDB:Q9FLP4 Genevestigator:Q9FLP4 Uniprot:Q9FLP4
Root	Isotig07231	23	99	-2.235	3.72E-14	RGD|727924 - symbol:LOC257642 "rRNA promoter binding protein" species:10116 "Rattus norvegicus" [GO:0003690 "double-stranded DNA binding" evidence=IDA] [GO:0003700 "sequence-specific DNA binding transcription factor activity" evidence=IDA] [GO:0008134 "transcription factor binding" evidence=IDA] [GO:0008283 "cell proliferation" evidence=IDA] [GO:0009303 "rRNA transcription" evidence=IDA] RGD:727924 GO:GO:0008134 GO:GO:0008283 GO:GO:0003700 GO:GO:0003690 GO:GO:0009303 EMBL:U77931 IPI:IPI00195015 RefSeq:NP_671477.1 GeneID:257642 KEGG:rno:257642 NextBio:624170 Genevestigator:Q99JC0 Uniprot:Q99JC0
Root	Isotig07232	23	98	-2.220	6.29E-14	RGD|727924 - symbol:LOC257642 "rRNA promoter binding protein" species:10116 "Rattus norvegicus" [GO:0003690 "double-stranded DNA binding" evidence=IDA] [GO:0003700 "sequence-specific DNA binding transcription factor activity" evidence=IDA] [GO:0008134 "transcription factor binding" evidence=IDA] [GO:0008283 "cell proliferation" evidence=IDA] [GO:0009303 "rRNA transcription" evidence=IDA] RGD:727924 GO:GO:0008134 GO:GO:0008283 GO:GO:0003700 GO:GO:0003690 GO:GO:0009303 EMBL:U77931 IPI:IPI00195015 RefSeq:NP_671477.1 GeneID:257642 KEGG:rno:257642 NextBio:624170 Genevestigator:Q99JC0 Uniprot:Q99JC0
Root	Isotig07235	4	20	-2.451	0.000325095	TAIR|locus:2064402 - symbol:C4H "AT2G30490" species:3702 "Arabidopsis thaliana" [GO:0009505 "plant-type cell wall" evidence=IDA] [GO:0005783 "endoplasmic reticulum" evidence=IDA;TAS] [GO:0005774 "vacuolar membrane" evidence=IDA] [GO:0005886 "plasma membrane" evidence=IDA] [GO:0016020 "membrane" evidence=IDA] [GO:0009555 "pollen development" evidence=IMP] [GO:0009698 "phenylpropanoid metabolic process" evidence=IMP] [GO:0009808 "lignin metabolic process" evidence=IMP] [GO:0032502 "developmental process" evidence=IMP] [GO:0040007 "growth" evidence=IMP] [GO:0080167 "response to karrikin" evidence=IEP] [GO:0009506 "plasmodesma" evidence=IDA] [GO:0005794 "Golgi apparatus" evidence=IDA] [GO:0009416 "response to light stimulus" evidence=IEP] [GO:0009611 "response to wounding" evidence=IMP] [GO:0009699 "phenylpropanoid biosynthetic process" evidence=TAS] [GO:0016710 "trans-cinnamate 4-monooxygenase activity" evidence=IDA] InterPro:IPR001128 InterPro:IPR002401 InterPro:IPR017972 Pfam:PF00067 PRINTS:PR00385 PRINTS:PR00463 PROSITE:PS00086 GO:GO:0005783 GO:GO:0016021 GO:GO:0005886 GO:GO:0009506 GO:GO:0005774 GO:GO:0040007 EMBL:CP002685 GenomeReviews:CT485783_GR GO:GO:0009055 GO:GO:0080167 GO:GO:0009555 eggNOG:COG2124 GO:GO:0020037 Gene3D:G3DSA:1.10.630.10 SUPFAM:SSF48264 GO:GO:0009505 HOGENOM:HBG749920 GO:GO:0009808 EMBL:U93215 EMBL:U71081 EMBL:U71080 EMBL:D78596 EMBL:AY065145 EMBL:BT008875 IPI:IPI00525264 PIR:A84709 RefSeq:NP_180607.1 UniGene:At.23450 ProteinModelPortal:P92994 SMR:P92994 IntAct:P92994 STRING:P92994 PRIDE:P92994 EnsemblPlants:AT2G30490.1 GeneID:817599 KEGG:ath:AT2G30490 GeneFarm:1333

Tissue	Identification	# of reads (stress)	# of reads (control)	Fold Change	p-value	Description
						TAIR:At2g30490 InParanoid:P92994 KO:K00487 OMA:GITIGRM PhylomeDB:P92994 ProtClustDB:PLN02394 ArrayExpress:P92994 Genevestigator:P92994 GermOnline:AT2G30490 GO:GO:0016710 Uniprot:P92994
Root	Isotig07236	4	21	-2.521	0.000181802	TAIR|locus:2064402 - symbol:C4H "AT2G30490" species:3702 "Arabidopsis thaliana" [GO:0009505 "plant-type cell wall" evidence=IDA] [GO:0005783 "endoplasmic reticulum" evidence=IDA;TAS] [GO:0005774 "vacuolar membrane" evidence=IDA] [GO:0005886 "plasma membrane" evidence=IDA] [GO:0016020 "membrane" evidence=IDA] [GO:0009555 "pollen development" evidence=IMP] [GO:0009698 "phenylpropanoid metabolic process" evidence=IMP] [GO:0009808 "lignin metabolic process" evidence=IMP] [GO:0032502 "developmental process" evidence=IMP] [GO:0040007 "growth" evidence=IMP] [GO:0080167 "response to karrikin" evidence=IEP] [GO:0009506 "plasmodesma" evidence=IDA] [GO:0005794 "Golgi apparatus" evidence=IDA] [GO:0009416 "response to light stimulus" evidence=IEP] [GO:0009611 "response to wounding" evidence=IMP] [GO:0009699 "phenylpropanoid biosynthetic process" evidence=TAS] [GO:0016710 "trans-cinnamate 4-monooxygenase activity" evidence=IDA] InterPro:IPR001128 InterPro:IPR002401 InterPro:IPR017972 Pfam:PF00067 PRINTS:PR00385 PRINTS:PR00463 PROSITE:PS00086 GO:GO:0005783 GO:GO:0016021 GO:GO:0005886 GO:GO:0009506 GO:GO:0005774 GO:GO:0040007 EMBL:CP002685 GenomeReviews:CT485783_GR GO:GO:0009055 GO:GO:0080167 GO:GO:0009555 eggNOG:COG2124 GO:GO:0020037 Gene3D:G3DSA:1.10.630.10 SUPFAM:SSF48264 GO:GO:0009505 HOGENOM:HBG749920 GO:GO:0009808 EMBL:U93215 EMBL:U71081 EMBL:U71080 EMBL:D78596 EMBL:AY065145 EMBL:BT008875 IPI:IPI00525264 PIR:A84709 RefSeq:NP_180607.1 UniGene:At.23450 ProteinModelPortal:P92994 SMR:P92994 IntAct:P92994 STRING:P92994 PRIDE:P92994 EnsemblPlants:AT2G30490.1 GeneID:817599 KEGG:ath:AT2G30490 GeneFarm:1333 TAIR:At2g30490 InParanoid:P92994 KO:K00487 OMA:GITIGRM PhylomeDB:P92994 ProtClustDB:PLN02394 ArrayExpress:P92994 Genevestigator:P92994 GermOnline:AT2G30490 GO:GO:0016710 Uniprot:P92994
Root	Isotig07239	0	17	-5.216	1.26E-05	No hit
Root	Isotig07240	0	13	-4.829	0.000155955	TAIR|locus:2097380 - symbol:AT3G49720 "AT3G49720" species:3702 "Arabidopsis thaliana" [GO:0003674 "molecular_function" evidence=ND] [GO:0008150 "biological_process" evidence=ND] [GO:0009535 "chloroplast thylakoid membrane" evidence=IDA] [GO:0005794 "Golgi apparatus" evidence=IDA] [GO:0005774 "vacuolar membrane" evidence=IDA] [GO:0016020 "membrane" evidence=IDA] [GO:0005886 "plasma membrane" evidence=IDA] GO:GO:0016021 GO:GO:0005886 GO:GO:0005774 EMBL:CP002686 GenomeReviews:BA000014_GR GO:GO:0000139 EMBL:AL132965 GO:GO:0009535 UniGene:At.22445 EMBL:AY087451 EMBL:AY059938 EMBL:AY114597 IPI:IPI00546242 PIR:T46038 RefSeq:NP_001078265.1 RefSeq:NP_566924.1 STRING:Q9M2Y6 PRIDE:Q9M2Y6 EnsemblPlants:AT3G49720.1 EnsemblPlants:AT3G49720.2 GeneID:824134 KEGG:ath:AT3G49720 TAIR:At3g49720 eggNOG:NOG327425 HOGENOM:HBG317293 InParanoid:Q9M2Y6 OMA:IEDADSH PhylomeDB:Q9M2Y6 ProtClustDB:CLSN2689079 ArrayExpress:Q93YM8 Genevestigator:Q9M2Y6 Uniprot:Q9M2Y6
Root	Isotig07248	12	0	4.456	0.000626491	TAIR|locus:2095833 - symbol:AT3G58030 species:3702 "Arabidopsis thaliana" [GO:0008270 "zinc ion binding" evidence=IEA;ISS] InterPro:IPR001841 PROSITE:PS50089 SMART:SM00184 Prosite:PS00518 EMBL:CP002686 GenomeReviews:BA000014_GR GO:GO:0008270 InterPro:IPR013083 Gene3D:G3DSA:3.30.40.10 InterPro:IPR017907 HOGENOM:HBG749996 ProtClustDB:CLSN2683808 EMBL:AY081289 EMBL:BT006585 IPI:IPI00546255 RefSeq:NP_001190122.1 RefSeq:NP_191362.2 RefSeq:NP_974453.1 RefSeq:NP_974454.1 UniGene:At.34773 ProteinModelPortal:Q8RXF2 SMR:Q8RXF2 PRIDE:Q8RXF2 EnsemblPlants:AT3G58030.1 EnsemblPlants:AT3G58030.2 EnsemblPlants:AT3G58030.3 EnsemblPlants:AT3G58030.4 GeneID:824972 KEGG:ath:AT3G58030 TAIR:At3g58030 eggNOG:NOG244906 InParanoid:Q8RXF2 OMA:HARRIES PhylomeDB:Q8RXF2 Genevestigator:Q8RXF2 Uniprot:Q8RXF2
Root	Isotig07259	49	7	2.678	1.58E-08	RGD|727924 - symbol:LOC257642 "rRNA promoter binding protein" species:10116 "Rattus norvegicus" [GO:0003690 "double-stranded DNA binding" evidence=IDA] [GO:0003700 "sequence-specific DNA binding transcription factor activity" evidence=IDA] [GO:0008134 "transcription factor binding" evidence=IDA] [GO:0008283 "cell proliferation" evidence=IDA] [GO:0009303 "rRNA transcription" evidence=IDA] RGD:727924 GO:GO:0008134 GO:GO:0008283 GO:GO:0003700 GO:GO:0003690 GO:GO:0009303 EMBL:U77931 IPI:IPI00195015 RefSeq:NP_671477.1 GeneID:257642 KEGG:rno:257642 NextBio:624170 Genevestigator:Q99JC0 Uniprot:Q99JC0
Root	Isotig07281	92	0	7.395	3.60E-21	TAIR|locus:2092990 - symbol:AT3G21000 "AT3G21000" species:3702 "Arabidopsis thaliana" [GO:0003674 "molecular_function" evidence=ND] [GO:0005575 "cellular_component" evidence=ND] [GO:0008150 "biological_process" evidence=ND] EMBL:CP002686 EMBL:DQ446681 IPI:IPI00522289 RefSeq:NP_188737.1

Tissue	Identification	# of reads (stress)	# of reads (control)	Fold Change	p-value	Description
						UniGene:At.53413 PRIDE:Q1PEN6 EnsemblPlants:AT3G21000.1 GeneID:821651 KEGG:ath:AT3G21000 TAIR:At3g21000 InParanoid:Q1PEN6 OMA:ATANIRD Genevestigator:Q1PEN6 Uniprot:Q1PEN6
Root	Isotig07282	88	0	7.330	2.08E-20	TAIR|locus:2092990 - symbol:AT3G21000 "AT3G21000" species:3702 "Arabidopsis thaliana" [GO:0003674 "molecular_function" evidence=ND] [GO:0005575 "cellular_component" evidence=ND] [GO:0008150 "biological_process" evidence=ND] EMBL:CP002686 EMBL:DQ446681 IPI:IPI00522289 RefSeq:NP_188737.1 UniGene:At.53413 PRIDE:Q1PEN6 EnsemblPlants:AT3G21000.1 GeneID:821651 KEGG:ath:AT3G21000 TAIR:At3g21000 InParanoid:Q1PEN6 OMA:ATANIRD Genevestigator:Q1PEN6 Uniprot:Q1PEN6
Root	Isotig07301	16	0	4.871	5.89E-05	TAIR|locus:2129351 - symbol:SBP1 "selenium-binding protein 1" species:3702 "Arabidopsis thaliana" [GO:0008430 "selenium binding" evidence=IEA;ISS] [GO:0046686 "response to cadmium ion" evidence=IEP;IDA] [GO:0000103 "sulfate assimilation" evidence=IEP] [GO:0042542 "response to hydrogen peroxide" evidence=IDA] [GO:0071291 "cellular response to selenium ion" evidence=IDA] [GO:0005829 "cytosol" evidence=IDA] [GO:0009506 "plasmodesma" evidence=IDA] InterPro:IPR008826 InterPro:IPR015943 Pfam:PF05694 GO:GO:0005829 GO:GO:0009506 EMBL:CP002687 GenomeReviews:CT486007_GR GO:GO:0046686 Gene3D:G3DSA:2.130.10.10 EMBL:Z97335 GO:GO:0042542 GO:GO:0000103 GO:GO:0008430 EMBL:AL161537 eggNOG:NOG84363 PANTHER:PTHR23300 HOGENOM:HBG318348 EMBL:AY062634 EMBL:AY128727 EMBL:Z27257 EMBL:AJ401229 IPI:IPI00543374 PIR:D71401 RefSeq:NP_001190723.1 RefSeq:NP_193139.1 UniGene:At.4249 ProteinModelPortal:O23264 SMR:O23264 PRIDE:O23264 EnsemblPlants:AT4G14030.1 EnsemblPlants:AT4G14030.2 GeneID:827039 KEGG:ath:AT4G14030 TAIR:At4g14030 InParanoid:O23264 OMA:MATETEV PhylomeDB:O23264 ProtClustDB:CLSN2684339 ArrayExpress:O23264 Genevestigator:O23264 GermOnline:AT4G14030 GO:GO:0071291 Uniprot:O23264
Root	Isotig07302	16	0	4.871	5.89E-05	TAIR|locus:2129351 - symbol:SBP1 "selenium-binding protein 1" species:3702 "Arabidopsis thaliana" [GO:0008430 "selenium binding" evidence=IEA;ISS] [GO:0046686 "response to cadmium ion" evidence=IEP;IDA] [GO:0000103 "sulfate assimilation" evidence=IEP] [GO:0042542 "response to hydrogen peroxide" evidence=IDA] [GO:0071291 "cellular response to selenium ion" evidence=IDA] [GO:0005829 "cytosol" evidence=IDA] [GO:0009506 "plasmodesma" evidence=IDA] InterPro:IPR008826 InterPro:IPR015943 Pfam:PF05694 GO:GO:0005829 GO:GO:0009506 EMBL:CP002687 GenomeReviews:CT486007_GR GO:GO:0046686 Gene3D:G3DSA:2.130.10.10 EMBL:Z97335 GO:GO:0042542 GO:GO:0000103 GO:GO:0008430 EMBL:AL161537 eggNOG:NOG84363 PANTHER:PTHR23300 HOGENOM:HBG318348 EMBL:AY062634 EMBL:AY128727 EMBL:Z27257 EMBL:AJ401229 IPI:IPI00543374 PIR:D71401 RefSeq:NP_001190723.1 RefSeq:NP_193139.1 UniGene:At.4249 ProteinModelPortal:O23264 SMR:O23264 PRIDE:O23264 EnsemblPlants:AT4G14030.1 EnsemblPlants:AT4G14030.2 GeneID:827039 KEGG:ath:AT4G14030 TAIR:At4g14030 InParanoid:O23264 OMA:MATETEV PhylomeDB:O23264 ProtClustDB:CLSN2684339 ArrayExpress:O23264 Genevestigator:O23264 GermOnline:AT4G14030 GO:GO:0071291 Uniprot:O23264
Root	Isotig07315	7	31	-2.276	1.84E-05	TAIR|locus:3439219 - symbol:TUA6 "AT4G14960" species:3702 "Arabidopsis thaliana" [GO:0005874 "microtubule" evidence=IDA] [GO:0045298 "tubulin complex" evidence=IDA] [GO:0005773 "vacuole" evidence=IDA] [GO:0005200 "structural constituent of cytoskeleton" evidence=ISS] [GO:0005730 "nucleolus" evidence=IDA] [GO:0000226 "microtubule cytoskeleton organization" evidence=IMP] [GO:0005618 "cell wall" evidence=IDA] [GO:0016020 "membrane" evidence=IDA] [GO:0005886 "plasma membrane" evidence=IDA] [GO:0009651 "response to salt stress" evidence=IEP] [GO:0005829 "cytosol" evidence=IDA] [GO:0005515 "protein binding" evidence=IPI] [GO:0071258 "cellular response to gravity" evidence=IMP] [GO:0009506 "plasmodesma" evidence=IDA] InterPro:IPR000217 InterPro:IPR002452 InterPro:IPR003008 InterPro:IPR008280 InterPro:IPR017975 InterPro:IPR018316 Pfam:PF00091 Pfam:PF03953 PRINTS:PR01161 PRINTS:PR01162 PROSITE:PS00227 SMART:SM00864 SMART:SM00865 GO:GO:0005525 GO:GO:0005829 GO:GO:0005886 GO:GO:0009506 EMBL:CP002687 GenomeReviews:CT486007_GR GO:GO:0005773 GO:GO:0005618 GO:GO:0005515 GO:GO:0005730 GO:GO:0005198 GO:GO:0003924 GO:GO:0009651 EMBL:AL161540 GO:GO:0000226 GO:GO:0005874 GO:GO:0051258 Gene3D:G3DSA:3.40.50.1440 SUPFAM:SSF52490 GO:GO:0007018 EMBL:Z97337 Gene3D:G3DSA:3.30.1330.20 SUPFAM:SSF55307 GO:GO:0045298 eggNOG:COG5023 GO:GO:0071258 HOGENOM:HBG750007 KO:K07374 InterPro:IPR023123 Gene3D:G3DSA:1.10.287.600 PANTHER:PTHR11588 ProtClustDB:PLN00221 UniGene:At.22368 UniGene:At.48153 EMBL:M84699 EMBL:AY058858 EMBL:AY065347 EMBL:AY079036 EMBL:AY117174 IPI:IPI00520226 PIR:JQ1597 RefSeq:NP_193232.1 ProteinModelPortal:P29511 SMR:P29511 IntAct:P29511 STRING:P29511 PRIDE:P29511

Tissue	Identification	# of reads (stress)	# of reads (control)	Fold Change	p-value	Description
						EnsemblPlants:AT4G14960.2 GeneID:827154 KEGG:ath:AT4G14960 TAIR:At4g14960 InParanoid:P29511 OMA:MPSDKAI PhylomeDB:P29511 ArrayExpress:P29511 Genevestigator:P29511 Uniprot:P29511
Root	Isotig07316	5	34	-2.895	2.97E-07	TAIR|locus:3439219 - symbol:TUA6 "AT4G14960" species:3702 "Arabidopsis thaliana" [GO:0005874 "microtubule" evidence=IDA] [GO:0045298 "tubulin complex" evidence=IDA] [GO:0005773 "vacuole" evidence=IDA] [GO:0005200 "structural constituent of cytoskeleton" evidence=ISS] [GO:0005730 "nucleolus" evidence=ISS] [GO:0000226 "microtubule cytoskeleton organization" evidence=IMP] [GO:0005618 "cell wall" evidence=IDA] [GO:0016020 "membrane" evidence=IDA] [GO:0005886 "plasma membrane" evidence=IDA] [GO:0009651 "response to salt stress" evidence=IEP] [GO:0005829 "cytosol" evidence=IDA] [GO:0005515 "protein binding" evidence=IPI] [GO:0071258 "cellular response to gravity" evidence=IMP] [GO:0009506 "plasmodesma" evidence=IDA] InterPro:IPR000217 InterPro:IPR002452 InterPro:IPR003008 InterPro:IPR008280 InterPro:IPR017975 InterPro:IPR018316 Pfam:PF00091 Pfam:PF03953 PRINTS:PR01161 PRINTS:PR01162 PROSITE:PS00227 SMART:SM00864 SMART:SM00865 GO:GO:0005525 GO:GO:0005829 GO:GO:0005886 GO:GO:0009506 EMBL:CP002687 GenomeReviews:CT486007_GR GO:GO:0005773 GO:GO:0005618 GO:GO:0005515 GO:GO:0005730 GO:GO:0005198 GO:GO:0003924 GO:GO:0009651 EMBL:AL161540 GO:GO:0000226 GO:GO:0005874 GO:GO:0051258 Gene3D:G3DSA:3.40.50.1440 SUPFAM:SSF52490 GO:GO:0007018 EMBL:Z97337 Gene3D:G3DSA:3.30.1330.20 SUPFAM:SSF55307 GO:GO:0045298 eggNOG:COG5023 GO:GO:0071258 HOGENOM:HBG750007 KO:K07374 InterPro:IPR023123 Gene3D:G3DSA:1.10.287.600 PANTHER:PTHR11588 ProtClustDB:PLN00221 UniGene:At.22368 UniGene:At.48153 EMBL:M84699 EMBL:AY058858 EMBL:AY065347 EMBL:AY079036 EMBL:AY117174 IPI:IPI00520226 PIR:JQ1597 RefSeq:NP_193232.1 ProteinModelPortal:P29511 SMR:P29511 IntAct:P29511 STRING:P29511 PRIDE:P29511 EnsemblPlants:AT4G14960.2 GeneID:827154 KEGG:ath:AT4G14960 TAIR:At4g14960 InParanoid:P29511 OMA:MPSDKAI PhylomeDB:P29511 ArrayExpress:P29511 Genevestigator:P29511 Uniprot:P29511
Root	Isotig07323	18	0	5.041	1.86E-05	TAIR|locus:2014109 - symbol:EBS5 "AT1G18260" species:3702 "Arabidopsis thaliana" [GO:0005783 "endoplasmic reticulum" evidence=IDA] [GO:0016020 "membrane" evidence=IDA] [GO:0030433 "ER-associated protein catabolic process" evidence=IMP] [GO:0042538 "hyperosmotic salinity response" evidence=IMP] InterPro:IPR011990 GO:GO:0005783 EMBL:CP002684 GO:GO:0005488 GO:GO:0016020 KO:K07126 InterPro:IPR006597 Gene3D:G3DSA:1.25.40.10 Pfam:PF08238 SMART:SM00671 GO:GO:0030433 GO:GO:0042538 EMBL:AC069551 ProtClustDB:CLSN2679872 EMBL:AB189471 IPI:IPI00542104 RefSeq:NP_564049.1 UniGene:At.14948 UniGene:At.67114 ProteinModelPortal:Q9LM25 SMR:Q9LM25 PRIDE:Q9LM25 EnsemblPlants:AT1G18260.1 GeneID:838406 KEGG:ath:AT1G18260 TAIR:At1g18260 InParanoid:Q9LM25 OMA:HGQGLPF PhylomeDB:Q9LM25 Genevestigator:Q9LM25 Uniprot:Q9LM25
Root	Isotig07324	18	0	5.041	1.86E-05	TAIR|locus:2014109 - symbol:EBS5 "AT1G18260" species:3702 "Arabidopsis thaliana" [GO:0005783 "endoplasmic reticulum" evidence=IDA] [GO:0016020 "membrane" evidence=IDA] [GO:0030433 "ER-associated protein catabolic process" evidence=IMP] [GO:0042538 "hyperosmotic salinity response" evidence=IMP] InterPro:IPR011990 GO:GO:0005783 EMBL:CP002684 GO:GO:0005488 GO:GO:0016020 KO:K07126 InterPro:IPR006597 Gene3D:G3DSA:1.25.40.10 Pfam:PF08238 SMART:SM00671 GO:GO:0030433 GO:GO:0042538 EMBL:AC069551 ProtClustDB:CLSN2679872 EMBL:AB189471 IPI:IPI00542104 RefSeq:NP_564049.1 UniGene:At.14948 UniGene:At.67114 ProteinModelPortal:Q9LM25 SMR:Q9LM25 PRIDE:Q9LM25 EnsemblPlants:AT1G18260.1 GeneID:838406 KEGG:ath:AT1G18260 TAIR:At1g18260 InParanoid:Q9LM25 OMA:HGQGLPF PhylomeDB:Q9LM25 Genevestigator:Q9LM25 Uniprot:Q9LM25
Root	Isotig07347	32	6	2.286	3.01E-05	TAIR|locus:2016254 - symbol:GLYI7 "AT1G80160" species:3702 "Arabidopsis thaliana" [GO:0004462 "lactoylglutathione lyase activity" evidence=ISS] [GO:0005975 "carbohydrate metabolic process" evidence=ISS] EMBL:AC009322 IPI:IPI00532356 PIR:C96833 ProteinModelPortal:Q9SSC3 SMR:Q9SSC3 STRING:Q9SSC3 PRIDE:Q9SSC3 TAIR:At1g80160 InParanoid:Q9SSC3 PhylomeDB:Q9SSC3 Genevestigator:Q9SSC3 Uniprot:Q9SSC3
Root	Isotig07348	32	5	2.549	8.76E-06	TAIR|locus:2016254 - symbol:GLYI7 "AT1G80160" species:3702 "Arabidopsis thaliana" [GO:0004462 "lactoylglutathione lyase activity" evidence=ISS] [GO:0005975 "carbohydrate metabolic process" evidence=ISS] EMBL:AC009322 IPI:IPI00532356 PIR:C96833 ProteinModelPortal:Q9SSC3 SMR:Q9SSC3 STRING:Q9SSC3 PRIDE:Q9SSC3 TAIR:At1g80160 InParanoid:Q9SSC3 PhylomeDB:Q9SSC3 Genevestigator:Q9SSC3 Uniprot:Q9SSC3
Root	Isotig07355	0	33	-6.173	1.05E-09	TAIR|locus:2141201 - symbol:CRK29 "AT4G21410" species:3702 "Arabidopsis thaliana" [GO:0016301 "kinase activity" evidence=ISS] [GO:0005773 "vacuole" evidence=IDA] [GO:0009737 "response to abscisic acid stimulus"

Tissue	Identification	# of reads (stress)	# of reads (control)	Fold Change	p-value	Description
						evidence=IEP] [GO:0005886 "plasma membrane" evidence=IDA] InterPro:IPR000719 InterPro:IPR008271 InterPro:IPR011009 InterPro:IPR017441 Pfam:PF00069 PROSITE:PS00107 PROSITE:PS00108 PROSITE:PS50011 GO:GO:0016021 GO:GO:0005886 GO:GO:0005524 GO:GO:0009737 EMBL:CP002687 GenomeReviews:CT486007_GR GO:GO:0005773 eggNOG:COG0515 GO:GO:0004872 SUPFAM:SSF56112 GO:GO:0004674 HOGENOM:HBG755340 EMBL:AL161555 EMBL:AL022603 EMBL:AL031187 InterPro:IPR002902 Pfam:PF01657 PROSITE:PS51473 ProtClustDB:CLSN2685554 EMBL:AY074844 IPI:IPI00516505 PIR:T05149 RefSeq:NP_193872.2 UniGene:At.43984 ProteinModelPortal:Q8S9L6 SMR:Q8S9L6 PRIDE:Q8S9L6 EnsemblPlants:AT4G21410.1 GeneID:827893 KEGG:ath:AT4G21410 GeneFarm:237 TAIR:At4g21410 InParanoid:Q8S9L6 OMA:ISANRDD PhylomeDB:Q8S9L6 ArrayExpress:O65406 Genevestigator:Q8S9L6 Uniprot:Q8S9L6
Root	Isotig07356	0	12	-4.714	0.000297064	TAIR|locus:2115728 - symbol:CRK25 "cysteine-rich RLK (RECEPTOR-like protein kinase) 25" species:3702 "Arabidopsis thaliana" [GO:0006468 "protein phosphorylation" evidence=IEA] [GO:0016301 "kinase activity" evidence=ISS] InterPro:IPR000719 InterPro:IPR001245 InterPro:IPR008271 InterPro:IPR011009 InterPro:IPR017441 Pfam:PF07714 PROSITE:PS00107 PROSITE:PS00108 PROSITE:PS50011 GO:GO:0016021 GO:GO:0005524 EMBL:CP002687 GenomeReviews:CT486007_GR eggNOG:COG0515 GO:GO:0004872 SUPFAM:SSF56112 GO:GO:0004674 HOGENOM:HBG755340 EMBL:AL161503 InterPro:IPR002902 Pfam:PF01657 PROSITE:PS51473 HSSP:P19525 EMBL:AC012477 IPI:IPI00521065 PIR:D85065 RefSeq:NP_192429.1 UniGene:At.33905 ProteinModelPortal:Q9M0X5 SMR:Q9M0X5 PRIDE:Q9M0X5 EnsemblPlants:AT4G05200.1 GeneID:825868 KEGG:ath:AT4G05200 GeneFarm:203 TAIR:At4g05200 InParanoid:Q9M0X5 OMA:LMETEIA PhylomeDB:Q9M0X5 ArrayExpress:Q9M0X5 Genevestigator:Q9M0X5 Uniprot:Q9M0X5
Root	Isotig07357	0	15	-5.036	4.38E-05	TAIR|locus:2126540 - symbol:AT4G29990 species:3702 "Arabidopsis thaliana" [GO:0004672 "protein kinase activity" evidence=IEA;ISS] [GO:0004674 "protein serine/threonine kinase activity" evidence=IEA] [GO:0005524 "ATP binding" evidence=IEA;ISS] [GO:0006468 "protein phosphorylation" evidence=IEA;ISS] InterPro:IPR000719 InterPro:IPR008271 InterPro:IPR011009 InterPro:IPR017441 Pfam:PF00069 PROSITE:PS00107 PROSITE:PS00108 PROSITE:PS50011 GO:GO:0016021 GO:GO:0005524 EMBL:CP002687 GO:GO:0004872 SUPFAM:SSF56112 GO:GO:0004674 EMBL:AL161576 EMBL:AL078464 InterPro:IPR024788 Pfam:PF12819 HSSP:Q62838 ProtClustDB:CLSN2683161 IPI:IPI00537051 PIR:D85350 RefSeq:NP_194728.1 UniGene:At.5387 ProteinModelPortal:Q9SZV2 SMR:Q9SZV2 PRIDE:Q9SZV2 EnsemblPlants:AT4G29990.1 GeneID:829122 KEGG:ath:AT4G29990 TAIR:At4g29990 InParanoid:Q9SZV2 OMA:INETAIM PhylomeDB:Q9SZV2 ArrayExpress:Q9SZV2 Genevestigator:Q9SZV2 Uniprot:Q9SZV2
Root	Isotig07358	0	16	-5.129	2.34E-05	TAIR|locus:2126540 - symbol:AT4G29990 species:3702 "Arabidopsis thaliana" [GO:0004672 "protein kinase activity" evidence=IEA;ISS] [GO:0004674 "protein serine/threonine kinase activity" evidence=IEA] [GO:0005524 "ATP binding" evidence=IEA;ISS] [GO:0006468 "protein phosphorylation" evidence=IEA;ISS] InterPro:IPR000719 InterPro:IPR008271 InterPro:IPR011009 InterPro:IPR017441 Pfam:PF00069 PROSITE:PS00107 PROSITE:PS00108 PROSITE:PS50011 GO:GO:0016021 GO:GO:0005524 EMBL:CP002687 GO:GO:0004872 SUPFAM:SSF56112 GO:GO:0004674 EMBL:AL161576 EMBL:AL078464 InterPro:IPR024788 Pfam:PF12819 HSSP:Q62838 ProtClustDB:CLSN2683161 IPI:IPI00537051 PIR:D85350 RefSeq:NP_194728.1 UniGene:At.5387 ProteinModelPortal:Q9SZV2 SMR:Q9SZV2 PRIDE:Q9SZV2 EnsemblPlants:AT4G29990.1 GeneID:829122 KEGG:ath:AT4G29990 TAIR:At4g29990 InParanoid:Q9SZV2 OMA:INETAIM PhylomeDB:Q9SZV2 ArrayExpress:Q9SZV2 Genevestigator:Q9SZV2 Uniprot:Q9SZV2
Root	Isotig07379	24	57	-1.377	4.02E-05	TAIR|locus:2061136 - symbol:COI1 "AT2G39940" species:3702 "Arabidopsis thaliana" [GO:0006511 "ubiquitin-dependent protein catabolic process" evidence=TAS] [GO:0009861 "jasmonic acid and ethylene-dependent systemic resistance" evidence=TAS] [GO:0005515 "protein binding" evidence=IPI] [GO:0009867 "jasmonic acid mediated signaling pathway" evidence=IGI;TAS] [GO:0009625 "response to insect" evidence=IMP] [GO:0009611 "response to wounding" evidence=IMP] [GO:0009753 "response to jasmonic acid stimulus" evidence=IMP] [GO:0019005 "SCF ubiquitin ligase complex" evidence=IPI] [GO:0031348 "negative regulation of defense response" evidence=IMP] [GO:0010118 "stomatal movement" evidence=IMP] [GO:0042742 "defense response to bacterium" evidence=IMP] [GO:0050832 "defense response to fungus" evidence=IMP] [GO:0031146 "SCF-dependent proteasomal ubiquitin-dependent protein catabolic process" evidence=IMP] [GO:0009641 "shade avoidance" evidence=IMP] [GO:0009909 "regulation of flower development" evidence=IMP] [GO:0010218 "response to far red light" evidence=IMP] [GO:0004842 "ubiquitin-protein ligase activity" evidence=ISS] [GO:0006952 "defense

Tissue	Identification	# of reads (stress)	# of reads (control)	Fold Change	p-value	Description
						response" evidence=TAS] PROSITE:PS50181 GO:GO:0042742 EMBL:CP002685 GenomeReviews:CT485783_GR GO:GO:0005515 GO:GO:0031348 GO:GO:0050832 GO:GO:0010218 GO:GO:0010118 GO:GO:0019005 GO:GO:0031146 HOGENOM:HBG319412 GO:GO:0009625 GO:GO:0009909 GO:GO:0009641 GO:GO:0009867 EMBL:AF002109 EMBL:AF036340 EMBL:EF470606 EMBL:EF470607 EMBL:EF470608 EMBL:EF470609 EMBL:EF470610 EMBL:EF470611 EMBL:EF470612 EMBL:EF470613 EMBL:EF470614 EMBL:EF470615 EMBL:EF470616 EMBL:EF470617 EMBL:EF470619 EMBL:EF470620 EMBL:EF470621 EMBL:EF470622 EMBL:EF470623 EMBL:EF470624 EMBL:AY045625 EMBL:AY133556 IPI:IPI00516922 PIR:T52139 RefSeq:NP_565919.1 UniGene:At.20831 UniGene:At.71018 PDB:3OGK PDB:3OGL PDB:3OGM PDBsum:3OGK PDBsum:3OGL PDBsum:3OGM ProteinModelPortal:O04197 SMR:O04197 DIP:DIP-31324N IntAct:O04197 STRING:O04197 PRIDE:O04197 EnsemblPlants:AT2G39940.1 GeneID:818581 KEGG:ath:AT2G39940 GeneFarm:4758 TAIR:At2g39940 eggNOG:NOG262527 InParanoid:O04197 KO:K13463 OMA:ARNCRSL PhylomeDB:O04197 ProtClustDB:CLSN2688817 ArrayExpress:O04197 Genevestigator:O04197 GermOnline:AT2G39940 GO:GO:0009861 Uniprot:O04197
Root	Isotig07386	1	12	-3.714	0.000699736	TAIR|locus:2036504 - symbol:ATB2 species:3702 "Arabidopsis thaliana" [GO:0016491 "oxidoreductase activity" evidence=IEA] [GO:0046686 "response to cadmium ion" evidence=IEP] [GO:0005829 "cytosol" evidence=IDA] InterPro:IPR020471 PRINTS:PR00069 PROSITE:PS00062 PROSITE:PS00063 PROSITE:PS00798 InterPro:IPR001395 Pfam:PF00248 EMBL:CP002684 GO:GO:0005829 GO:GO:0046686 InterPro:IPR023210 Gene3D:G3DSA:3.20.20.100 PANTHER:PTHR11732 SUPFAM:SSF51430 GO:GO:0016491 eggNOG:COG0667 EMBL:AC002292 HSSP:P38918 ProtClustDB:CLSN2681811 EMBL:AJ608277 EMBL:AF057715 EMBL:AY056440 EMBL:AY099655 EMBL:BT000251 EMBL:Z26233 IPI:IPI00523400 IPI:IPI00533262 PIR:E96632 RefSeq:NP_564761.1 UniGene:At.22690 ProteinModelPortal:Q93ZN2 SMR:Q93ZN2 STRING:Q93ZN2 ANU-2DPAGE:Q9SEX1 PRIDE:Q93ZN2 EnsemblPlants:AT1G60710.1 GeneID:842365 KEGG:ath:AT1G60710 TAIR:At1g60710 InParanoid:Q93ZN2 OMA:ELGINFL PhylomeDB:Q93ZN2 ArrayExpress:Q93ZN2 Genevestigator:Q93ZN2 Uniprot:Q93ZN2
Root	Isotig07391	5	26	-2.507	3.30E-05	TAIR|locus:2080432 - symbol:AT3G59940 "AT3G59940" species:3702 "Arabidopsis thaliana" [GO:0003674 "molecular_function" evidence=ND] [GO:0005575 "cellular_component" evidence=ND] [GO:0008150 "biological_process" evidence=ND] InterPro:IPR001810 InterPro:IPR006652 Pfam:PF01344 PROSITE:PS50181 SMART:SM00256 SMART:SM00612 GO:GO:0005634 EMBL:CP002686 GenomeReviews:BA000014_GR SUPFAM:SSF81383 InterPro:IPR015916 Gene3D:G3DSA:2.130.10.80 EMBL:AL138647 UniGene:At.1165 UniGene:At.48785 HOGENOM:HBG593750 eggNOG:NOG282025 ProtClustDB:CLSN2685021 EMBL:AY074382 EMBL:AY081325 EMBL:AY091240 EMBL:AY088575 IPI:IPI00534118 PIR:T47818 RefSeq:NP_191553.1 ProteinModelPortal:Q9M1Y1 SMR:Q9M1Y1 IntAct:Q9M1Y1 STRING:Q9M1Y1 PRIDE:Q9M1Y1 EnsemblPlants:AT3G59940.1 GeneID:825164 KEGG:ath:AT3G59940 TAIR:At3g59940 InParanoid:Q9M1Y1 OMA:CFIDSER PhylomeDB:Q9M1Y1 ArrayExpress:Q9M1Y1 Genevestigator:Q9M1Y1 Uniprot:Q9M1Y1
Root	Isotig07392	5	26	-2.507	3.30E-05	TAIR|locus:2080432 - symbol:AT3G59940 "AT3G59940" species:3702 "Arabidopsis thaliana" [GO:0003674 "molecular_function" evidence=ND] [GO:0005575 "cellular_component" evidence=ND] [GO:0008150 "biological_process" evidence=ND] InterPro:IPR001810 InterPro:IPR006652 Pfam:PF01344 PROSITE:PS50181 SMART:SM00256 SMART:SM00612 GO:GO:0005634 EMBL:CP002686 GenomeReviews:BA000014_GR SUPFAM:SSF81383 InterPro:IPR015916 Gene3D:G3DSA:2.130.10.80 EMBL:AL138647 UniGene:At.1165 UniGene:At.48785 HOGENOM:HBG593750 eggNOG:NOG282025 ProtClustDB:CLSN2685021 EMBL:AY074382 EMBL:AY081325 EMBL:AY091240 EMBL:AY088575 IPI:IPI00534118 PIR:T47818 RefSeq:NP_191553.1 ProteinModelPortal:Q9M1Y1 SMR:Q9M1Y1 IntAct:Q9M1Y1 STRING:Q9M1Y1 PRIDE:Q9M1Y1 EnsemblPlants:AT3G59940.1 GeneID:825164 KEGG:ath:AT3G59940 TAIR:At3g59940 InParanoid:Q9M1Y1 OMA:CFIDSER PhylomeDB:Q9M1Y1 ArrayExpress:Q9M1Y1 Genevestigator:Q9M1Y1 Uniprot:Q9M1Y1
Root	Isotig07393	804	976	-0.409	2.35E-09	No hit
Root	Isotig07394	752	909	-0.403	1.33E-08	No hit
Root	Isotig07399	18	0	5.041	1.86E-05	TAIR|locus:2029486 - symbol:AT1G78070 "AT1G78070" species:3702 "Arabidopsis thaliana" [GO:0003674 "molecular_function" evidence=ND] [GO:0008150 "biological_process" evidence=ND] [GO:0080008 "CUL4 RING ubiquitin ligase complex" evidence=ISS] InterPro:IPR017986 InterPro:IPR001680 InterPro:IPR015943 Pfam:PF00400 PROSITE:PS50082 PROSITE:PS50294 SMART:SM00320 EMBL:CP002684 InterPro:IPR019775 Gene3D:G3DSA:2.130.10.10 PROSITE:PS00678 EMBL:AF372951 EMBL:BT002231 IPI:IPI00528231 RefSeq:NP_565168.4 UniGene:At.19130 ProteinModelPortal:Q94JT6 SMR:Q94JT6 IntAct:Q94JT6

Tissue	Identification	# of reads (stress)	# of reads (control)	Fold Change	p-value	Description
						STRING:Q94JT6 EnsemblPlants:AT1G78070.1 GeneID:844142 KEGG:ath:AT1G78070 TAIR:At1g78070 InParanoid:Q94JT6 PhylomeDB:Q94JT6 ProtClustDB:CLSN2714470 Genevestigator:Q94JT6 Uniprot:Q94JT6
Root	Isotig07400	18	0	5.041	1.86E-05	TAIR|locus:2029486 - symbol:AT1G78070 "AT1G78070" species:3702 "Arabidopsis thaliana" [GO:0003674 "molecular_function" evidence=ND] [GO:0008150 "biological_process" evidence=ND] [GO:0080008 "CUL4 RING ubiquitin ligase complex" evidence=ISS] InterPro:IPR017986 InterPro:IPR001680 InterPro:IPR015943 Pfam:PF00400 PROSITE:PS50082 PROSITE:PS50294 SMART:SM00320 EMBL:CP002684 InterPro:IPR019775 Gene3D:G3DSA:2.130.10.10 PROSITE:PS00678 EMBL:AF372951 EMBL:BT002231 IPI:IPI00528231 RefSeq:NP_565168.4 UniGene:At.19130 ProteinModelPortal:Q94JT6 SMR:Q94JT6 IntAct:Q94JT6 STRING:Q94JT6 EnsemblPlants:AT1G78070.1 GeneID:844142 KEGG:ath:AT1G78070 TAIR:At1g78070 InParanoid:Q94JT6 PhylomeDB:Q94JT6 ProtClustDB:CLSN2714470 Genevestigator:Q94JT6 Uniprot:Q94JT6
Root	Isotig07423	16	0	4.871	5.89E-05	TAIR|locus:2176501 - symbol:AT5G43400 "AT5G43400" species:3702 "Arabidopsis thaliana" [GO:0003674 "molecular_function" evidence=ND] [GO:0008150 "biological_process" evidence=ND] EMBL:CP002688 GenomeReviews:BA000015_GR EMBL:AB025638 InterPro:IPR024553 Pfam:PF11443 eggNOG:NOG75724 HOGENOM:HBG589557 InterPro:IPR011205 PIRSF:PIRSF015417 EMBL:AY048243 EMBL:BT002275 IPI:IPI00546502 RefSeq:NP_199153.1 UniGene:At.7153 ProteinModelPortal:Q94AE4 IntAct:Q94AE4 PRIDE:Q94AE4 EnsemblPlants:AT5G43400.1 GeneID:834358 KEGG:ath:AT5G43400 TAIR:At5g43400 InParanoid:Q94AE4 OMA:EDRILEN PhylomeDB:Q94AE4 ProtClustDB:CLSN2686815 Genevestigator:Q94AE4 Uniprot:Q94AE4
Root	Isotig07427	2	24	-3.714	1.64E-06	TAIR|locus:2170773 - symbol:DXR "AT5G62790" species:3702 "Arabidopsis thaliana" [GO:0008299 "isoprenoid biosynthetic process" evidence=TAS] [GO:0009507 "chloroplast" evidence=IDA] [GO:0019288 "isopentenyl diphosphate biosynthetic process, mevalonate-independent pathway" evidence=IDA;TAS] [GO:0030604 "1-deoxy-D-xylulose-5-phosphate reductoisomerase activity" evidence=IDA] [GO:0046686 "response to cadmium ion" evidence=IEP] [GO:0009570 "chloroplast stroma" evidence=IDA] InterPro:IPR003821 InterPro:IPR013512 InterPro:IPR013644 InterPro:IPR016040 Pfam:PF02670 Pfam:PF08436 PIRSF:PIRSF006205 GO:GO:0009570 GO:GO:0046686 EMBL:CP002688 GenomeReviews:BA000015_GR Gene3D:G3DSA:3.40.50.720 GO:GO:0046872 GO:GO:0070402 EMBL:AB009053 eggNOG:COG0743 HOGENOM:HBG430762 KO:K00099 GO:GO:0030604 TIGRFAMs:TIGR00243 OMA:IHSMVEY EMBL:AF148852 EMBL:AY045634 EMBL:AY050802 EMBL:AY054682 EMBL:AY081453 EMBL:AY091405 EMBL:AY098952 EMBL:AY084775 EMBL:AJ242588 IPI:IPI00519417 PIR:T52570 RefSeq:NP_201085.1 UniGene:At.23021 ProteinModelPortal:Q9XFS9 SMR:Q9XFS9 STRING:Q9XFS9 PRIDE:Q9XFS9 EnsemblPlants:AT5G62790.1 GeneID:836400 KEGG:ath:AT5G62790 TAIR:At5g62790 InParanoid:Q9XFS9 PhylomeDB:Q9XFS9 ProtClustDB:PLN02696 BioCyc:ARA:AT5G62790-MONOMER BioCyc:MetaCyc:AT5G62790-MONOMER BRENDA:1.1.1.267 ArrayExpress:Q9XFS9 Genevestigator:Q9XFS9 GermOnline:AT5G62790 GO:GO:0019288 Uniprot:Q9XFS9
Root	Isotig07428	2	24	-3.714	1.64E-06	TAIR|locus:2170773 - symbol:DXR "AT5G62790" species:3702 "Arabidopsis thaliana" [GO:0008299 "isoprenoid biosynthetic process" evidence=TAS] [GO:0009507 "chloroplast" evidence=IDA] [GO:0019288 "isopentenyl diphosphate biosynthetic process, mevalonate-independent pathway" evidence=IDA;TAS] [GO:0030604 "1-deoxy-D-xylulose-5-phosphate reductoisomerase activity" evidence=IDA] [GO:0046686 "response to cadmium ion" evidence=IEP] [GO:0009570 "chloroplast stroma" evidence=IDA] InterPro:IPR003821 InterPro:IPR013512 InterPro:IPR013644 InterPro:IPR016040 Pfam:PF02670 Pfam:PF08436 PIRSF:PIRSF006205 GO:GO:0009570 GO:GO:0046686 EMBL:CP002688 GenomeReviews:BA000015_GR Gene3D:G3DSA:3.40.50.720 GO:GO:0046872 GO:GO:0070402 EMBL:AB009053 eggNOG:COG0743 HOGENOM:HBG430762 KO:K00099 GO:GO:0030604 TIGRFAMs:TIGR00243 OMA:IHSMVEY EMBL:AF148852 EMBL:AY045634 EMBL:AY050802 EMBL:AY054682 EMBL:AY081453 EMBL:AY091405 EMBL:AY098952 EMBL:AY084775 EMBL:AJ242588 IPI:IPI00519417 PIR:T52570 RefSeq:NP_201085.1 UniGene:At.23021 ProteinModelPortal:Q9XFS9 SMR:Q9XFS9 STRING:Q9XFS9 PRIDE:Q9XFS9 EnsemblPlants:AT5G62790.1 GeneID:836400 KEGG:ath:AT5G62790 TAIR:At5g62790 InParanoid:Q9XFS9 PhylomeDB:Q9XFS9 ProtClustDB:PLN02696 BioCyc:ARA:AT5G62790-MONOMER BioCyc:MetaCyc:AT5G62790-MONOMER BRENDA:1.1.1.267 ArrayExpress:Q9XFS9 Genevestigator:Q9XFS9 GermOnline:AT5G62790 GO:GO:0019288 Uniprot:Q9XFS9
Root	Isotig07431	4	41	-3.487	9.45E-10	TAIR|locus:505006632 - symbol:TUB8 "AT5G23860" species:3702 "Arabidopsis thaliana" [GO:0005200 "structural constituent of cytoskeleton" evidence=ISS] [GO:0007017 "microtubule-based process" evidence=ISS] [GO:0045298 "tubulin complex" evidence=ISS] [GO:0016020 "membrane" evidence=IDA] [GO:0009651 "response to salt stress" evidence=IEP] [GO:0005515 "protein binding" evidence=IPI] [GO:0005794 "Golgi apparatus" evidence=IDA]

Tissue	Identification	# of reads (stress)	# of reads (control)	Fold Change	p-value	Description
						InterPro:IPR000217 InterPro:IPR002453 InterPro:IPR003008 InterPro:IPR008280 InterPro:IPR017975 InterPro:IPR018316 Pfam:PF00091 Pfam:PF03953 PRINTS:PR01161 PRINTS:PR01163 PROSITE:PS00227 SMART:SM00864 SMART:SM00865 GO:GO:0005525 GO:GO:0005737 EMBL:CP002688 GenomeReviews:BA000015_GR GO:GO:0005515 GO:GO:0016020 GO:GO:0005198 GO:GO:0003924 EMBL:AB005244 GO:GO:0009651 GO:GO:0005874 GO:GO:0051258 Gene3D:G3DSA:3.40.50.1440 SUPFAM:SSF52490 GO:GO:0007018 Gene3D:G3DSA:3.30.1330.20 SUPFAM:SSF55307 eggNOG:COG5023 HOGENOM:HBG750007 InterPro:IPR023123 Gene3D:G3DSA:1.10.287.600 InterPro:IPR013838 KO:K07375 ProtClustDB:PLN00220 PROSITE:PS00228 OMA:RYQGEND EMBL:M84705 EMBL:AY054693 EMBL:AY081473 IPI:IPI00539093 PIR:JQ1592 RefSeq:NP_001190373.1 RefSeq:NP_568437.1 UniGene:At.330 UniGene:At.67084 ProteinModelPortal:P29516 SMR:P29516 IntAct:P29516 STRING:P29516 PRIDE:P29516 EnsemblPlants:AT5G23860.1 EnsemblPlants:AT5G23860.2 GeneID:832451 KEGG:ath:AT5G23860 TAIR:At5g23860 InParanoid:P29516 PhylomeDB:P29516 Genevestigator:P29516 GermOnline:AT5G23860 Uniprot:P29516
Root	Isotig07432	4	42	-3.521	5.08E-10	TAIR|locus:505006632 - symbol:TUB8 "AT5G23860" species:3702 "Arabidopsis thaliana" [GO:0005200 "structural constituent of cytoskeleton" evidence=ISS] [GO:0007017 "microtubule-based process" evidence=ISS] [GO:0045298 "tubulin complex" evidence=ISS] [GO:0016020 "membrane" evidence=IDA] [GO:0009651 "response to salt stress" evidence=IEP] [GO:0005515 "protein binding" evidence=IPI] [GO:0005794 "Golgi apparatus" evidence=IDA] InterPro:IPR000217 InterPro:IPR002453 InterPro:IPR003008 InterPro:IPR008280 InterPro:IPR017975 InterPro:IPR018316 Pfam:PF00091 Pfam:PF03953 PRINTS:PR01161 PRINTS:PR01163 PROSITE:PS00227 SMART:SM00864 SMART:SM00865 GO:GO:0005525 GO:GO:0005737 EMBL:CP002688 GenomeReviews:BA000015_GR GO:GO:0005515 GO:GO:0016020 GO:GO:0005198 GO:GO:0003924 EMBL:AB005244 GO:GO:0009651 GO:GO:0005874 GO:GO:0051258 Gene3D:G3DSA:3.40.50.1440 SUPFAM:SSF52490 GO:GO:0007018 Gene3D:G3DSA:3.30.1330.20 SUPFAM:SSF55307 eggNOG:COG5023 HOGENOM:HBG750007 InterPro:IPR023123 Gene3D:G3DSA:1.10.287.600 InterPro:IPR013838 KO:K07375 ProtClustDB:PLN00220 PROSITE:PS00228 OMA:RYQGEND EMBL:M84705 EMBL:AY054693 EMBL:AY081473 IPI:IPI00539093 PIR:JQ1592 RefSeq:NP_001190373.1 RefSeq:NP_568437.1 UniGene:At.330 UniGene:At.67084 ProteinModelPortal:P29516 SMR:P29516 IntAct:P29516 STRING:P29516 PRIDE:P29516 EnsemblPlants:AT5G23860.1 EnsemblPlants:AT5G23860.2 GeneID:832451 KEGG:ath:AT5G23860 TAIR:At5g23860 InParanoid:P29516 PhylomeDB:P29516 Genevestigator:P29516 GermOnline:AT5G23860 Uniprot:P29516
Root	Isotig07439	0	27	-5.884	3.19E-08	TAIR|locus:2173219 - symbol:PAO1 "polyamine oxidase 1" species:3702 "Arabidopsis thaliana" [GO:0055114 "oxidation-reduction process" evidence=IEA] [GO:0046592 "polyamine oxidase activity" evidence=IDA] [GO:0050660 "flavin adenine dinucleotide binding" evidence=IDA] InterPro:IPR001613 InterPro:IPR002937 Pfam:PF01593 PRINTS:PR00757 EMBL:CP002688 GenomeReviews:BA000015_GR GO:GO:0050660 EMBL:AB006704 eggNOG:COG1231 GO:GO:0046592 EMBL:AK118627 EMBL:BT026370 IPI:IPI00526970 RefSeq:NP_196874.1 UniGene:At.49017 UniGene:At.6477 HSSP:O64411 ProteinModelPortal:Q9FNA2 SMR:Q9FNA2 STRING:Q9FNA2 PRIDE:Q9FNA2 EnsemblPlants:AT5G13700.1 GeneID:831215 KEGG:ath:AT5G13700 TAIR:At5g13700 HOGENOM:HBG325057 InParanoid:Q9FNA2 KO:K13366 OMA:EPISTYV PhylomeDB:Q9FNA2 ProtClustDB:PLN02676 BioCyc:ARA:AT5G13700-MONOMER BioCyc:MetaCyc:AT5G13700-MONOMER Genevestigator:Q9FNA2 Uniprot:Q9FNA2
Root	Isotig07440	0	24	-5.714	1.84E-07	TAIR|locus:2173219 - symbol:PAO1 "polyamine oxidase 1" species:3702 "Arabidopsis thaliana" [GO:0055114 "oxidation-reduction process" evidence=IEA] [GO:0046592 "polyamine oxidase activity" evidence=IDA] [GO:0050660 "flavin adenine dinucleotide binding" evidence=IDA] InterPro:IPR001613 InterPro:IPR002937 Pfam:PF01593 PRINTS:PR00757 EMBL:CP002688 GenomeReviews:BA000015_GR GO:GO:0050660 EMBL:AB006704 eggNOG:COG1231 GO:GO:0046592 EMBL:AK118627 EMBL:BT026370 IPI:IPI00526970 RefSeq:NP_196874.1 UniGene:At.49017 UniGene:At.6477 HSSP:O64411 ProteinModelPortal:Q9FNA2 SMR:Q9FNA2 STRING:Q9FNA2 PRIDE:Q9FNA2 EnsemblPlants:AT5G13700.1 GeneID:831215 KEGG:ath:AT5G13700 TAIR:At5g13700 HOGENOM:HBG325057 InParanoid:Q9FNA2 KO:K13366 OMA:EPISTYV PhylomeDB:Q9FNA2 ProtClustDB:PLN02676 BioCyc:ARA:AT5G13700-MONOMER BioCyc:MetaCyc:AT5G13700-MONOMER Genevestigator:Q9FNA2 Uniprot:Q9FNA2
Root	Isotig07441	30	0	5.778	2.46E-08	TAIR|locus:2179867 - symbol:SAG29 "senescence-associated gene 29" species:3702 "Arabidopsis thaliana" [GO:0016020 "membrane" evidence=IEA;ISS] [GO:0016021 "integral to membrane" evidence=IEA] [GO:0010150

Tissue	Identification	# of reads (stress)	# of reads (control)	Fold Change	p-value	Description
						"leaf senescence" evidence=IEP;IMP] [GO:0071446 "cellular response to salicylic acid stimulus" evidence=IEP] [GO:0005887 "integral to plasma membrane" evidence=IDA] [GO:0071215 "cellular response to abscisic acid stimulus" evidence=IEP] [GO:0071470 "cellular response to osmotic stress" evidence=IMP] [GO:0051119 "sugar transmembrane transporter activity" evidence=ISS] InterPro:IPR004316 Pfam:PF03083 EMBL:CP002688 GenomeReviews:BA000015_GR GO:GO:0005887 GO:GO:0010150 EMBL:AL391711 GO:GO:0071470 GO:GO:0071215 GO:GO:0071446 GO:GO:0051119 PANTHER:PTHR10791 KO:K15382 eggNOG:NOG308353 EMBL:AY045949 EMBL:AY113934 EMBL:AY087836 EMBL:AF118824 IPI:IPI00530310 RefSeq:NP_196821.1 UniGene:At.46857 TCDB:9.A.58.1.3 EnsemblPlants:AT5G13170.1 GeneID:831156 KEGG:ath:AT5G13170 TAIR:At5g13170 InParanoid:Q9FY94 OMA:WFAYGLF PhylomeDB:Q9FY94 ProtClustDB:CLSN2916510 ArrayExpress:Q9FY94 Genevestigator:Q9FY94 Uniprot:Q9FY94
Root	Isotig07442	30	0	5.778	2.46E-08	TAIR|locus:2179867 - symbol:SAG29 "senescence-associated gene 29" species:3702 "Arabidopsis thaliana" [GO:0016020 "membrane" evidence=IEA;ISS] [GO:0016021 "integral to membrane" evidence=IEA] [GO:0010150 "leaf senescence" evidence=IEP;IMP] [GO:0071446 "cellular response to salicylic acid stimulus" evidence=IEP] [GO:0005887 "integral to plasma membrane" evidence=IDA] [GO:0071215 "cellular response to abscisic acid stimulus" evidence=IEP] [GO:0071470 "cellular response to osmotic stress" evidence=IMP] [GO:0051119 "sugar transmembrane transporter activity" evidence=ISS] InterPro:IPR004316 Pfam:PF03083 EMBL:CP002688 GenomeReviews:BA000015_GR GO:GO:0005887 GO:GO:0010150 EMBL:AL391711 GO:GO:0071470 GO:GO:0071215 GO:GO:0071446 GO:GO:0051119 PANTHER:PTHR10791 KO:K15382 eggNOG:NOG308353 EMBL:AY045949 EMBL:AY113934 EMBL:AY087836 EMBL:AF118824 IPI:IPI00530310 RefSeq:NP_196821.1 UniGene:At.46857 TCDB:9.A.58.1.3 EnsemblPlants:AT5G13170.1 GeneID:831156 KEGG:ath:AT5G13170 TAIR:At5g13170 InParanoid:Q9FY94 OMA:WFAYGLF PhylomeDB:Q9FY94 ProtClustDB:CLSN2916510 ArrayExpress:Q9FY94 Genevestigator:Q9FY94 Uniprot:Q9FY94
Root	Isotig07449	33	7	2.108	5.57E-05	TAIR|locus:2026242 - symbol:BETA-VPE "AT1G62710" species:3702 "Arabidopsis thaliana" [GO:0004197 "cysteine-type endopeptidase activity" evidence=ISS] [GO:0006508 "proteolysis" evidence=ISS] [GO:0006624 "vacuolar protein processing" evidence=ISS] InterPro:IPR001096 Pfam:PF01650 PIRSF:PIRSF019663 PRINTS:PR00776 EMBL:CP002684 GenomeReviews:CT485782_GR GO:GO:0005773 GO:GO:0004197 GO:GO:0006508 EMBL:AC007190 eggNOG:COG5206 PANTHER:PTHR12000 KO:K01369 HOGENOM:HBG318720 ProtClustDB:CLSN2682340 EMBL:D61394 EMBL:AY059156 EMBL:AF367254 IPI:IPI00519485 PIR:C96652 PIR:S60050 RefSeq:NP_176458.1 UniGene:At.16374 ProteinModelPortal:Q39044 STRING:Q39044 MEROPS:C13.001 PRIDE:Q39044 EnsemblPlants:AT1G62710.1 GeneID:842569 KEGG:ath:AT1G62710 TAIR:At1g62710 InParanoid:Q39044 OMA:PPSEYIT PhylomeDB:Q39044 ArrayExpress:Q39044 Genevestigator:Q39044 GermOnline:AT1G62710 Uniprot:Q39044
Root	Isotig07450	35	7	2.193	2.09E-05	TAIR|locus:2123782 - symbol:GAMMA-VPE "AT4G32940" species:3702 "Arabidopsis thaliana" [GO:0004197 "cysteine-type endopeptidase activity" evidence=ISS;IDA] [GO:0006508 "proteolysis" evidence=ISS] [GO:0000323 "lytic vacuole" evidence=IDA] [GO:0006624 "vacuolar protein processing" evidence=ISS] InterPro:IPR001096 Pfam:PF01650 PIRSF:PIRSF019663 PRINTS:PR00776 EMBL:CP002687 GenomeReviews:CT486007_GR GO:GO:0004197 GO:GO:0006508 EMBL:AL031804 EMBL:AL161582 eggNOG:COG5206 PANTHER:PTHR12000 KO:K01369 HOGENOM:HBG318720 GO:GO:0000323 ProtClustDB:CLSN2682340 EMBL:D61395 EMBL:AF370160 EMBL:AF424619 EMBL:AY059104 EMBL:AY133531 IPI:IPI00537536 PIR:T05302 RefSeq:NP_195020.1 UniGene:At.21567 UniGene:At.48929 UniGene:At.75206 ProteinModelPortal:Q39119 STRING:Q39119 MEROPS:C13.006 PRIDE:Q39119 EnsemblPlants:AT4G32940.1 GeneID:829431 KEGG:ath:AT4G32940 TAIR:At4g32940 InParanoid:Q39119 OMA:ICHAYQL PhylomeDB:Q39119 Genevestigator:Q39119 Uniprot:Q39119
Root	Isotig07453	0	17	-5.216	1.26E-05	TAIR|locus:2064945 - symbol:AT2G38970 "AT2G38970" species:3702 "Arabidopsis thaliana" [GO:0004842 "ubiquitin-protein ligase activity" evidence=ISS] [GO:0008270 "zinc ion binding" evidence=IEA;ISS] InterPro:IPR001841 InterPro:IPR002035 PROSITE:PS50089 PROSITE:PS50234 SMART:SM00184 SMART:SM00327 GenomeReviews:CT485783_GR GO:GO:0008270 InterPro:IPR013083 Gene3D:G3DSA:3.30.40.10 eggNOG:COG2304 EMBL:AY075691 IPI:IPI00529574 ProteinModelPortal:Q8S9I2 SMR:Q8S9I2 PRIDE:Q8S9I2 TAIR:At2g38970 HOGENOM:HBG317190 InParanoid:Q8S9I2 PhylomeDB:Q8S9I2 Genevestigator:Q8S9I2 Uniprot:Q8S9I2
Root	Isotig07454	0	14	-4.936	8.24E-05	TAIR|locus:2175796 - symbol:AT5G60710 "AT5G60710" species:3702 "Arabidopsis thaliana" [GO:0004842 "ubiquitin-protein ligase activity" evidence=ISS] [GO:0008270 "zinc ion binding" evidence=IEA;ISS] [GO:0005886

Tissue	Identification	# of reads (stress)	# of reads (control)	Fold Change	p-value	Description
						"plasma membrane" evidence=IDA] InterPro:IPR001841 InterPro:IPR002035 Pfam:PF00092 PROSITE:PS50089 PROSITE:PS50234 SMART:SM00184 SMART:SM00327 GO:GO:0005886 EMBL:CP002688 GO:GO:0008270 EMBL:AB005246 InterPro:IPR013083 Gene3D:G3DSA:3.30.40.10 IPI:IPI00533797 RefSeq:NP_200879.1 UniGene:At.21965 ProteinModelPortal:Q9FF49 SMR:Q9FF49 PRIDE:Q9FF49 EnsemblPlants:AT5G60710.1 GeneID:836192 KEGG:ath:AT5G60710 TAIR:At5g60710 InParanoid:Q9FF49 OMA:QGHAIFT PhylomeDB:Q9FF49 Genevestigator:Q9FF49 Uniprot:Q9FF49
Root	Isotig07475	1	26	-4.829	8.91E-08	UNIPROTKB|Q9ST02 - symbol:naat-A "Nicotianamine aminotransferase A" species:4513 "Hordeum vulgare" [GO:0033855 "nicotianamine aminotransferase activity" evidence=IDA] InterPro:IPR001176 InterPro:IPR004839 InterPro:IPR005958 InterPro:IPR015421 InterPro:IPR015422 InterPro:IPR021178 Pfam:PF00155 PIRSF:PIRSF000517 PRINTS:PR00753 PROSITE:PS00105 GO:GO:0016847 GO:GO:0030170 InterPro:IPR015424 Gene3D:G3DSA:3.40.640.10 Gene3D:G3DSA:3.90.1150.10 SUPFAM:SSF53383 TIGRFAMs:TIGR01265 EMBL:AB024006 EMBL:D88273 ProteinModelPortal:Q9ST02 Gramene:Q9ST02 BRENDA:2.6.1.80 Genevestigator:Q9ST02 GO:GO:0033855 Uniprot:Q9ST02
Root	Isotig07476	1	26	-4.829	8.91E-08	UNIPROTKB|Q9ST02 - symbol:naat-A "Nicotianamine aminotransferase A" species:4513 "Hordeum vulgare" [GO:0033855 "nicotianamine aminotransferase activity" evidence=IDA] InterPro:IPR001176 InterPro:IPR004839 InterPro:IPR005958 InterPro:IPR015421 InterPro:IPR015422 InterPro:IPR021178 Pfam:PF00155 PIRSF:PIRSF000517 PRINTS:PR00753 PROSITE:PS00105 GO:GO:0016847 GO:GO:0030170 InterPro:IPR015424 Gene3D:G3DSA:3.40.640.10 Gene3D:G3DSA:3.90.1150.10 SUPFAM:SSF53383 TIGRFAMs:TIGR01265 EMBL:AB024006 EMBL:D88273 ProteinModelPortal:Q9ST02 Gramene:Q9ST02 BRENDA:2.6.1.80 Genevestigator:Q9ST02 GO:GO:0033855 Uniprot:Q9ST02
Root	Isotig07509	3	43	-3.970	5.33E-11	TAIR|locus:2178067 - symbol:AATP1 "AT5G40010" species:3702 "Arabidopsis thaliana" [GO:0005524 "ATP binding" evidence=ISS] [GO:0016887 "ATPase activity" evidence=ISS;IDA] [GO:0005739 "mitochondrion" evidence=IDA] [GO:0010154 "fruit development" evidence=IMP] [GO:0010431 "seed maturation" evidence=IMP] InterPro:IPR003593 InterPro:IPR003959 InterPro:IPR003960 Pfam:PF00004 PROSITE:PS00674 SMART:SM00382 GO:GO:0005739 GO:GO:0005524 EMBL:CP002688 GenomeReviews:BA000015_GR GO:GO:0016887 eggNOG:COG0465 GO:GO:0010431 EMBL:AB010077 HOGENOM:HBG744315 IPI:IPI00538441 RefSeq:NP_198817.1 UniGene:At.65609 ProteinModelPortal:Q9FLD5 SMR:Q9FLD5 PRIDE:Q9FLD5 EnsemblPlants:AT5G40010.1 GeneID:833998 KEGG:ath:AT5G40010 TAIR:At5g40010 InParanoid:Q9FLD5 OMA:LRLESHH PhylomeDB:Q9FLD5 ProtClustDB:CLSN2685235 Genevestigator:Q9FLD5 Uniprot:Q9FLD5
Root	Isotig07510	3	32	-3.544	5.33E-08	TAIR|locus:2178067 - symbol:AATP1 "AT5G40010" species:3702 "Arabidopsis thaliana" [GO:0005524 "ATP binding" evidence=ISS] [GO:0016887 "ATPase activity" evidence=ISS;IDA] [GO:0005739 "mitochondrion" evidence=IDA] [GO:0010154 "fruit development" evidence=IMP] [GO:0010431 "seed maturation" evidence=IMP] InterPro:IPR003593 InterPro:IPR003959 InterPro:IPR003960 Pfam:PF00004 PROSITE:PS00674 SMART:SM00382 GO:GO:0005739 GO:GO:0005524 EMBL:CP002688 GenomeReviews:BA000015_GR GO:GO:0016887 eggNOG:COG0465 GO:GO:0010431 EMBL:AB010077 HOGENOM:HBG744315 IPI:IPI00538441 RefSeq:NP_198817.1 UniGene:At.65609 ProteinModelPortal:Q9FLD5 SMR:Q9FLD5 PRIDE:Q9FLD5 EnsemblPlants:AT5G40010.1 GeneID:833998 KEGG:ath:AT5G40010 TAIR:At5g40010 InParanoid:Q9FLD5 OMA:LRLESHH PhylomeDB:Q9FLD5 ProtClustDB:CLSN2685235 Genevestigator:Q9FLD5 Uniprot:Q9FLD5
Root	Isotig07514	24	53	-1.272	0.000195269	TAIR|locus:2044851 - symbol:LOS2 "AT2G36530" species:3702 "Arabidopsis thaliana" [GO:0004634 "phosphopyruvate hydratase activity" evidence=ISS;IDA] [GO:0003677 "DNA binding" evidence=IDA] [GO:0005634 "nucleus" evidence=IDA] [GO:0005737 "cytoplasm" evidence=IDA] [GO:0009409 "response to cold" evidence=IMP] [GO:0009416 "response to light stimulus" evidence=IMP] [GO:0005739 "mitochondrion" evidence=IDA] [GO:0005740 "mitochondrial envelope" evidence=IDA] [GO:0046686 "response to cadmium ion" evidence=IEP] [GO:0005507 "copper ion binding" evidence=IDA] [GO:0005886 "plasma membrane" evidence=IDA] [GO:0016020 "membrane" evidence=IDA] [GO:0009651 "response to salt stress" evidence=IEP] [GO:0009507 "chloroplast" evidence=IDA] [GO:0048046 "apoplast" evidence=IDA] [GO:0009737 "response to abscisic acid stimulus" evidence=IEP] [GO:0005829 "cytosol" evidence=IDA] [GO:0009506 "plasmodesma" evidence=IDA] InterPro:IPR000941 InterPro:IPR020809 InterPro:IPR020810 InterPro:IPR020811 Pfam:PF00113 Pfam:PF03952 PIRSF:PIRSF001400 PRINTS:PR00148 PROSITE:PS00164 PANTHER:PTHR11902 GO:GO:0005886 GO:GO:0009506 GO:GO:0009737 GO:GO:0048046 GO:GO:0005634 GO:GO:0046686 GO:GO:0009507 EMBL:CP002685 GenomeReviews:CT485783_GR GO:GO:0006355 GO:GO:0003677 GO:GO:0000287 GO:GO:0006351 GO:GO:0005507 GO:GO:0009409 GO:GO:0009651 EMBL:AC006919 GO:GO:0005740

Tissue	Identification	# of reads (stress)	# of reads (control)	Fold Change	p-value	Description
						GO:GO:0009416 GO:GO:0006096 eggNOG:COG0148 HOGENOM:HBG726599 KO:K01689 GO:GO:0000015 GO:GO:0004634 TIGRFAMs:TIGR01060 ProtClustDB:PLN00191 EMBL:X58107 EMBL:AF424603 EMBL:AY054253 EMBL:AY072095 EMBL:AY092986 EMBL:AY150418 IPI:IPI00526310 PIR:JQ1187 RefSeq:NP_181192.1 UniGene:At.24124 UniGene:At.25354 UniGene:At.74012 ProteinModelPortal:P25696 SMR:P25696 STRING:P25696 SWISS-2DPAGE:P25696 PRIDE:P25696 ProMEX:P25696 EnsemblPlants:AT2G36530.1 GeneID:818226 KEGG:ath:AT2G36530 TAIR:At2g36530 InParanoid:P25696 OMA:GELYKNF PhylomeDB:P25696 BioCyc:ARA:AT2G36530-MONOMER BioCyc:MetaCyc:AT2G36530-MONOMER Genevestigator:P25696 GermOnline:AT2G36530 Uniprot:P25696
Root	Isotig07533	2	16	-3.129	0.000262867	TAIR|locus:2055165 - symbol:AT2G14510 species:3702 "Arabidopsis thaliana" [GO:0006468 "protein phosphorylation" evidence=IEA] [GO:0016301 "kinase activity" evidence=ISS] InterPro:IPR000719 InterPro:IPR001245 InterPro:IPR008271 InterPro:IPR011009 InterPro:IPR017441 Pfam:PF07714 PROSITE:PS00107 PROSITE:PS00108 PROSITE:PS50011 GO:GO:0016021 GO:GO:0005886 GO:GO:0005524 EMBL:CP002685 GenomeReviews:CT485783_GR eggNOG:COG0515 GO:GO:0004872 SUPFAM:SSF56112 GO:GO:0004674 HOGENOM:HBG755340 InterPro:IPR024788 Pfam:PF12819 EMBL:AC006067 ProtClustDB:CLSN2683452 EMBL:FJ708693 IPI:IPI00521096 PIR:A84518 RefSeq:NP_179057.1 UniGene:At.52739 ProteinModelPortal:Q9ZQR3 SMR:Q9ZQR3 PRIDE:Q9ZQR3 EnsemblPlants:AT2G14510.1 GeneID:815938 KEGG:ath:AT2G14510 GeneFarm:454 TAIR:At2g14510 InParanoid:Q9ZQR3 PhylomeDB:Q9ZQR3 ArrayExpress:Q9ZQR3 Genevestigator:Q9ZQR3 Uniprot:Q9ZQR3
Root	Isotig07534	1	15	-4.036	9.83E-05	TAIR|locus:2017537 - symbol:AT1G51810 species:3702 "Arabidopsis thaliana" [GO:0004672 "protein kinase activity" evidence=IEA] [GO:0004674 "protein serine/threonine kinase activity" evidence=IEA] [GO:0005524 "ATP binding" evidence=IEA] [GO:0006468 "protein phosphorylation" evidence=IEA] [GO:0016301 "kinase activity" evidence=ISS] InterPro:IPR000719 InterPro:IPR008271 InterPro:IPR011009 InterPro:IPR017441 Pfam:PF00069 PROSITE:PS00107 PROSITE:PS00108 PROSITE:PS50011 GO:GO:0016021 EMBL:CP002684 GenomeReviews:CT485782_GR GO:GO:0005524 EMBL:AC025294 eggNOG:COG0515 GO:GO:0004872 SUPFAM:SSF56112 GO:GO:0004674 HSSP:P36897 InterPro:IPR024788 Pfam:PF12819 EMBL:AC015448 EMBL:DQ459169 EMBL:FJ708651 IPI:IPI00546436 IPI:IPI00953809 PIR:D96557 RefSeq:NP_175593.2 UniGene:At.52140 ProteinModelPortal:Q9FZB8 SMR:Q9FZB8 PRIDE:Q9FZB8 GeneID:841608 KEGG:ath:AT1G51810 GeneFarm:337 TAIR:At1g51810 InParanoid:Q9C8I8 PhylomeDB:Q9FZB8 Uniprot:Q9FZB8
Root	Isotig07543	66	9	2.745	3.00E-11	TAIR|locus:2025237 - symbol:ADH1 "AT1G77120" species:3702 "Arabidopsis thaliana" [GO:0045333 "cellular respiration" evidence=TAS] [GO:0006970 "response to osmotic stress" evidence=IGI] [GO:0001666 "response to hypoxia" evidence=IEP] [GO:0046686 "response to cadmium ion" evidence=IEP] [GO:0005886 "plasma membrane" evidence=IDA] [GO:0009651 "response to salt stress" evidence=IEP] [GO:0005829 "cytosol" evidence=IDA] [GO:0005794 "Golgi apparatus" evidence=RCA] [GO:0004022 "alcohol dehydrogenase (NAD) activity" evidence=ISS;IMP] InterPro:IPR002085 InterPro:IPR002328 InterPro:IPR013149 InterPro:IPR013154 InterPro:IPR016040 Pfam:PF00107 Pfam:PF08240 PROSITE:PS00059 EMBL:CP002684 GenomeReviews:CT485782_GR GO:GO:0005829 GO:GO:0005886 GO:GO:0046686 GO:GO:0000166 Gene3D:G3DSA:3.40.50.720 GO:GO:0008270 GO:GO:0001666 GO:GO:0009651 InterPro:IPR011032 PANTHER:PTHR11695 SUPFAM:SSF50129 eggNOG:COG1062 GO:GO:0004022 EMBL:M12196 EMBL:X77943 EMBL:D84240 EMBL:D84241 EMBL:D84242 EMBL:D84243 EMBL:D84244 EMBL:D84245 EMBL:D84246 EMBL:D84247 EMBL:D84248 EMBL:D84249 EMBL:D63460 EMBL:D63461 EMBL:D63462 EMBL:D63463 EMBL:D63464 EMBL:AF110456 EMBL:AB048394 EMBL:AB048395 EMBL:AY536888 EMBL:AC002291 EMBL:AY045612 EMBL:AY090330 EMBL:AY088010 IPI:IPI00539119 PIR:A23815 RefSeq:NP_177837.1 UniGene:At.22653 UniGene:At.64099 ProteinModelPortal:P06525 SMR:P06525 STRING:P06525 PRIDE:P06525 EnsemblPlants:AT1G77120.1 GeneID:844047 KEGG:ath:AT1G77120 TAIR:At1g77120 InParanoid:P06525 KO:K00001 OMA:ECTGSVQ PhylomeDB:P06525 ProtClustDB:CLSN2717044 ArrayExpress:P06525 Genevestigator:P06525 Uniprot:P06525
Root	Isotig07544	63	9	2.678	1.46E-10	TAIR|locus:2025237 - symbol:ADH1 "AT1G77120" species:3702 "Arabidopsis thaliana" [GO:0045333 "cellular respiration" evidence=TAS] [GO:0006970 "response to osmotic stress" evidence=IGI] [GO:0001666 "response to hypoxia" evidence=IEP] [GO:0046686 "response to cadmium ion" evidence=IEP] [GO:0005886 "plasma membrane" evidence=IDA] [GO:0009651 "response to salt stress" evidence=IEP] [GO:0005829 "cytosol" evidence=IDA] [GO:0005794 "Golgi apparatus" evidence=RCA] [GO:0004022 "alcohol dehydrogenase (NAD) activity" evidence=ISS;IMP] InterPro:IPR002085 InterPro:IPR002328 InterPro:IPR013149 InterPro:IPR013154

Tissue	Identification	# of reads (stress)	# of reads (control)	Fold Change	p-value	Description
						InterPro:IPR016040 Pfam:PF00107 Pfam:PF08240 PROSITE:PS00059 EMBL:CP002684 GenomeReviews:CT485782_GR GO:GO:0005829 GO:GO:0005886 GO:GO:0046686 GO:GO:0000166 Gene3D:G3DSA:3.40.50.720 GO:GO:0008270 GO:GO:0001666 GO:GO:0009651 InterPro:IPR011032 PANTHER:PTHR11695 SUPFAM:SSF50129 eggNOG:COG1062 GO:GO:0004022 EMBL:M12196 EMBL:X77943 EMBL:D84240 EMBL:D84241 EMBL:D84242 EMBL:D84243 EMBL:D84244 EMBL:D84245 EMBL:D84246 EMBL:D84247 EMBL:D84248 EMBL:D84249 EMBL:D63460 EMBL:D63461 EMBL:D63462 EMBL:D63463 EMBL:D63464 EMBL:AF110456 EMBL:AB048394 EMBL:AB048395 EMBL:AY536888 EMBL:AC002291 EMBL:AY045612 EMBL:AY090330 EMBL:AY088010 IPI:IPI00539119 PIR:A23815 RefSeq:NP_177837.1 UniGene:At.22653 UniGene:At.64099 ProteinModelPortal:P06525 SMR:P06525 STRING:P06525 PRIDE:P06525 EnsemblPlants:AT1G77120.1 GeneID:844047 KEGG:ath:AT1G77120 TAIR:At1g77120 InParanoid:P06525 KO:K00001 OMA:ECTGSVQ PhylomeDB:P06525 ProtClustDB:CLSN2717044 ArrayExpress:P06525 Genevestigator:P06525 Uniprot:P06525
Root	Isotig07554	10	29	-1.665	0.000753212	TAIR|locus:2039037 - symbol:AT2G20420 "AT2G20420" species:3702 "Arabidopsis thaliana" [GO:0004776 "succinate-CoA ligase (GDP-forming) activity" evidence=ISS] [GO:0008152 "metabolic process" evidence=ISS] [GO:0005739 "mitochondrion" evidence=IDA] [GO:0046686 "response to cadmium ion" evidence=IEP] [GO:0005524 "ATP binding" evidence=IDA] [GO:0005507 "copper ion binding" evidence=IDA] [GO:0005794 "Golgi apparatus" evidence=RCA] InterPro:IPR005809 InterPro:IPR005811 InterPro:IPR011761 InterPro:IPR013815 InterPro:IPR013816 Pfam:PF00549 PIRSF:PIRSF001554 PROSITE:PS50975 Prosite:PS01217 GO:GO:0005739 GO:GO:0005524 GO:GO:0046686 EMBL:CP002685 GenomeReviews:CT485783_GR GO:GO:0005507 Gene3D:G3DSA:3.30.1490.20 Gene3D:G3DSA:3.30.470.20 GO:GO:0006099 InterPro:IPR017866 InterPro:IPR016102 Gene3D:G3DSA:3.40.50.261 eggNOG:COG0045 InterPro:IPR013650 Pfam:PF08442 SUPFAM:SSF52210 GO:GO:0004775 EMBL:AC006569 KO:K01900 PANTHER:PTHR11815 TIGRFAMs:TIGR01016 HOGENOM:HBG315432 EMBL:AJ001808 EMBL:AY099707 EMBL:AY128889 IPI:IPI00532524 PIR:T51809 RefSeq:NP_179632.1 UniGene:At.21151 ProteinModelPortal:O82662 SMR:O82662 STRING:O82662 PRIDE:O82662 EnsemblPlants:AT2G20420.1 GeneID:816561 KEGG:ath:AT2G20420 GeneFarm:2004 TAIR:At2g20420 InParanoid:O82662 OMA:MIIKVPI PhylomeDB:O82662 ProtClustDB:PLN00124 ArrayExpress:O82662 Genevestigator:O82662 GermOnline:AT2G20420 Uniprot:O82662
Root	Isotig07565	78	133	-0.899	8.63E-06	TAIR|locus:2141385 - symbol:SAM-2 "AT4G01850" species:3702 "Arabidopsis thaliana" [GO:0004478 "methionine adenosyltransferase activity" evidence=ISS] [GO:0006556 "S-adenosylmethionine biosynthetic process" evidence=ISS] [GO:0005730 "nucleolus" evidence=IDA] [GO:0005618 "cell wall" evidence=IDA] [GO:0005507 "copper ion binding" evidence=IDA] [GO:0071281 "cellular response to iron ion" evidence=IEP] [GO:0005829 "cytosol" evidence=IDA;RCA] [GO:0070062 "extracellular vesicular exosome" evidence=IDA] [GO:0009506 "plasmodesma" evidence=IDA] InterPro:IPR002133 InterPro:IPR022628 InterPro:IPR022629 InterPro:IPR022630 InterPro:IPR022631 InterPro:IPR022636 Pfam:PF00438 Pfam:PF02772 Pfam:PF02773 PIRSF:PIRSF000497 PROSITE:PS00376 PROSITE:PS00377 GO:GO:0005829 GO:GO:0009506 GO:GO:0005524 EMBL:CP002687 GenomeReviews:CT486007_GR GO:GO:0005618 GO:GO:0071281 GO:GO:0005730 GO:GO:0005507 EMBL:AC007138 EMBL:AL161493 GO:GO:0070062 GO:GO:0006730 KO:K00789 GO:GO:0006556 UniGene:At.24731 eggNOG:COG0192 HOGENOM:HBG443662 ProtClustDB:PLN02243 GO:GO:0004478 PANTHER:PTHR11964 SUPFAM:SSF55973 TIGRFAMs:TIGR01034 EMBL:M33217 EMBL:AY072327 EMBL:AY094454 EMBL:BT000575 EMBL:Z33778 EMBL:Z29136 IPI:IPI00529348 PIR:JQ0410 RefSeq:NP_001078345.1 RefSeq:NP_192094.1 UniGene:At.24433 UniGene:At.71920 ProteinModelPortal:P17562 SMR:P17562 STRING:P17562 PRIDE:P17562 ProMEX:P17562 EnsemblPlants:AT4G01850.1 EnsemblPlants:AT4G01850.2 GeneID:826987 KEGG:ath:AT4G01850 TAIR:At4g01850 InParanoid:P17562 OMA:ATIDYEK PhylomeDB:P17562 ArrayExpress:P17562 Genevestigator:P17562 GermOnline:AT4G01850 Uniprot:P17562
Root	Isotig07566	62	126	-1.152	1.09E-07	TAIR|locus:2141385 - symbol:SAM-2 "AT4G01850" species:3702 "Arabidopsis thaliana" [GO:0004478 "methionine adenosyltransferase activity" evidence=ISS] [GO:0006556 "S-adenosylmethionine biosynthetic process" evidence=ISS] [GO:0005730 "nucleolus" evidence=IDA] [GO:0005618 "cell wall" evidence=IDA] [GO:0005507 "copper ion binding" evidence=IDA] [GO:0071281 "cellular response to iron ion" evidence=IEP] [GO:0005829 "cytosol" evidence=IDA;RCA] [GO:0070062 "extracellular vesicular exosome" evidence=IDA] [GO:0009506 "plasmodesma" evidence=IDA] InterPro:IPR002133 InterPro:IPR022628 InterPro:IPR022629 InterPro:IPR022630 InterPro:IPR022631 InterPro:IPR022636 Pfam:PF00438 Pfam:PF02772 Pfam:PF02773 PIRSF:PIRSF000497

Tissue	Identification	# of reads (stress)	# of reads (control)	Fold Change	p-value	Description
						PROSITE:PS00376 PROSITE:PS00377 GO:GO:0005829 GO:GO:0009506 GO:GO:0005524 EMBL:CP002687 GenomeReviews:CT486007_GR GO:GO:0005618 GO:GO:0071281 GO:GO:0005730 GO:GO:0005507 EMBL:AC007138 EMBL:AL161493 GO:GO:0070062 GO:GO:0006730 KO:K00789 GO:GO:0006556 UniGene:At.24731 eggNOG:COG0192 HOGENOM:HBG443662 ProtClustDB:PLN02243 GO:GO:0004478 PANTHER:PTHR11964 SUPFAM:SSF55973 TIGRFAMs:TIGR01034 EMBL:M33217 EMBL:AY072327 EMBL:AY094454 EMBL:BT000575 EMBL:Z33778 EMBL:Z29136 IPI:IPI00529348 PIR:JQ0410 RefSeq:NP_001078345.1 RefSeq:NP_192094.1 UniGene:At.24433 UniGene:At.71920 ProteinModelPortal:P17562 SMR:P17562 STRING:P17562 PRIDE:P17562 ProMEX:P17562 EnsemblPlants:AT4G01850.1 EnsemblPlants:AT4G01850.2 GeneID:826987 KEGG:ath:AT4G01850 TAIR:At4g01850 InParanoid:P17562 OMA:ATIDYEK PhylomeDB:P17562 ArrayExpress:P17562 Genevestigator:P17562 GermOnline:AT4G01850 Uniprot:P17562
Root	Isotig07569	5	33	-2.851	5.40E-07	TAIR|locus:2103040 - symbol:APY1 "AT3G04080" species:3702 "Arabidopsis thaliana" [GO:0005516 "calmodulin binding" evidence=ISS;IDA] [GO:0016887 "ATPase activity" evidence=IDA] [GO:0017110 "nucleoside-diphosphatase activity" evidence=IGI;IDA] [GO:0009846 "pollen germination" evidence=IGI] [GO:0005794 "Golgi apparatus" evidence=IDA] InterPro:IPR000407 Pfam:PF01150 PROSITE:PS01238 EMBL:CP002686 GenomeReviews:BA000014_GR GO:GO:0016787 EMBL:AC016829 GO:GO:0009846 eggNOG:COG5371 PANTHER:PTHR11782 OMA:MATSWGA HOGENOM:HBG444677 EMBL:AF093604 EMBL:BT029157 IPI:IPI00522459 RefSeq:NP_187058.1 UniGene:At.16940 ProteinModelPortal:Q9SQG2 SMR:Q9SQG2 STRING:Q9SQG2 PRIDE:Q9SQG2 EnsemblPlants:AT3G04080.1 GeneID:819563 KEGG:ath:AT3G04080 TAIR:At3g04080 InParanoid:Q9SQG2 KO:K14641 PhylomeDB:Q9SQG2 ProtClustDB:CLSN2684264 Genevestigator:Q9SQG2 Uniprot:Q9SQG2
Root	Isotig07570	5	30	-2.714	3.20E-06	TAIR|locus:2103040 - symbol:APY1 "AT3G04080" species:3702 "Arabidopsis thaliana" [GO:0005516 "calmodulin binding" evidence=ISS;IDA] [GO:0016887 "ATPase activity" evidence=IDA] [GO:0017110 "nucleoside-diphosphatase activity" evidence=IGI;IDA] [GO:0009846 "pollen germination" evidence=IGI] [GO:0005794 "Golgi apparatus" evidence=IDA] InterPro:IPR000407 Pfam:PF01150 PROSITE:PS01238 EMBL:CP002686 GenomeReviews:BA000014_GR GO:GO:0016787 EMBL:AC016829 GO:GO:0009846 eggNOG:COG5371 PANTHER:PTHR11782 OMA:MATSWGA HOGENOM:HBG444677 EMBL:AF093604 EMBL:BT029157 IPI:IPI00522459 RefSeq:NP_187058.1 UniGene:At.16940 ProteinModelPortal:Q9SQG2 SMR:Q9SQG2 STRING:Q9SQG2 PRIDE:Q9SQG2 EnsemblPlants:AT3G04080.1 GeneID:819563 KEGG:ath:AT3G04080 TAIR:At3g04080 InParanoid:Q9SQG2 KO:K14641 PhylomeDB:Q9SQG2 ProtClustDB:CLSN2684264 Genevestigator:Q9SQG2 Uniprot:Q9SQG2
Root	Isotig07592	1	13	-3.829	0.000362856	TAIR|locus:2016407 - symbol:AT1G19450 "AT1G19450" species:3702 "Arabidopsis thaliana" [GO:0005351 "sugar:hydrogen symporter activity" evidence=ISS] [GO:0006810 "transport" evidence=IEA] [GO:0015144 "carbohydrate transmembrane transporter activity" evidence=ISS] [GO:0016020 "membrane" evidence=ISS] [GO:0055085 "transmembrane transport" evidence=IEA] [GO:0005773 "vacuole" evidence=IDA] [GO:0005774 "vacuolar membrane" evidence=IDA] InterPro:IPR003663 InterPro:IPR005828 InterPro:IPR005829 Pfam:PF00083 PRINTS:PR00171 PROSITE:PS00216 PROSITE:PS00217 GO:GO:0016021 EMBL:CP002684 GenomeReviews:CT485782_GR GO:GO:0005774 eggNOG:COG0477 InterPro:IPR016196 SUPFAM:SSF103473 GO:GO:0008643 InterPro:IPR020846 PROSITE:PS50850 HOGENOM:HBG744444 GO:GO:0022891 TIGRFAMs:TIGR00879 EMBL:AC025808 EMBL:AY059848 EMBL:AY093274 IPI:IPI00534617 RefSeq:NP_173377.1 UniGene:At.22941 ProteinModelPortal:Q93YP9 IntAct:Q93YP9 PRIDE:Q93YP9 EnsemblPlants:AT1G19450.1 GeneID:838529 KEGG:ath:AT1G19450 TAIR:At1g19450 InParanoid:Q93YP9 OMA:SIGMTIS PhylomeDB:Q93YP9 ProtClustDB:CLSN2682491 ArrayExpress:Q93YP9 Genevestigator:Q93YP9 GermOnline:AT1G19450 Uniprot:Q93YP9
Root	Isotig07593	2	17	-3.216	0.000139706	TAIR|locus:2009323 - symbol:AT1G34580 "AT1G34580" species:3702 "Arabidopsis thaliana" [GO:0005351 "sugar:hydrogen symporter activity" evidence=ISS] [GO:0006810 "transport" evidence=IEA] [GO:0015144 "carbohydrate transmembrane transporter activity" evidence=ISS] [GO:0016020 "membrane" evidence=IEA;ISS] [GO:0016021 "integral to membrane" evidence=IEA] [GO:0055085 "transmembrane transport" evidence=IEA] InterPro:IPR003663 InterPro:IPR005828 InterPro:IPR005829 Pfam:PF00083 PRINTS:PR00171 PROSITE:PS00216 PROSITE:PS00217 GO:GO:0016021 EMBL:CP002684 GenomeReviews:CT485782_GR GO:GO:0015293 eggNOG:COG0477 InterPro:IPR016196 SUPFAM:SSF103473 GO:GO:0008643 InterPro:IPR020846 PROSITE:PS50850 HOGENOM:HBG744444 GO:GO:0022891 TIGRFAMs:TIGR00879 EMBL:AC023279

Tissue	Identification	# of reads (stress)	# of reads (control)	Fold Change	p-value	Description
						EMBL:AJ344335 EMBL:AK118511 EMBL:BT005898 IPI:IPI00529432 PIR:F86469 RefSeq:NP_174718.1 UniGene:At.15065 ProteinModelPortal:Q93Y91 PRIDE:Q93Y91 EnsemblPlants:AT1G34580.1 GeneID:840362 KEGG:ath:AT1G34580 TAIR:At1g34580 InParanoid:Q93Y91 OMA:SMYQVWE PhylomeDB:Q93Y91 ProtClustDB:CLSN2679632 Genevestigator:Q93Y91 GermOnline:AT1G34580 Uniprot:Q93Y91
Root	Isotig07594	2	16	-3.129	0.000262867	TAIR|locus:2009323 - symbol:AT1G34580 "AT1G34580" species:3702 "Arabidopsis thaliana" [GO:0005351 "sugar:hydrogen symporter activity" evidence=ISS] [GO:0006810 "transport" evidence=IEA] [GO:0015144 "carbohydrate transmembrane transporter activity" evidence=ISS] [GO:0016020 "membrane" evidence=IEA;ISS] [GO:0016021 "integral to membrane" evidence=IEA] [GO:0055085 "transmembrane transport" evidence=IEA] InterPro:IPR003663 InterPro:IPR005828 InterPro:IPR005829 Pfam:PF00083 PRINTS:PR00171 PROSITE:PS00216 PROSITE:PS00217 GO:GO:0016021 EMBL:CP002684 GenomeReviews:CT485782_GR GO:GO:0015293 eggNOG:COG0477 InterPro:IPR016196 SUPFAM:SSF103473 GO:GO:0008643 InterPro:IPR020846 PROSITE:PS50850 HOGENOM:HBG744444 GO:GO:0022891 TIGRFAMs:TIGR00879 EMBL:AC023279 EMBL:AJ344335 EMBL:AK118511 EMBL:BT005898 IPI:IPI00529432 PIR:F86469 RefSeq:NP_174718.1 UniGene:At.15065 ProteinModelPortal:Q93Y91 PRIDE:Q93Y91 EnsemblPlants:AT1G34580.1 GeneID:840362 KEGG:ath:AT1G34580 TAIR:At1g34580 InParanoid:Q93Y91 OMA:SMYQVWE PhylomeDB:Q93Y91 ProtClustDB:CLSN2679632 Genevestigator:Q93Y91 GermOnline:AT1G34580 Uniprot:Q93Y91
Root	Isotig07625	13	0	4.571	0.000344357	CGD|CAL0004433 - symbol:orf19.2051 species:5476 "Candida albicans" [GO:0008150 "biological_process" evidence=ND] [GO:0005622 "intracellular" evidence=IEA] [GO:0008270 "zinc ion binding" evidence=IEA] InterPro:IPR007087 InterPro:IPR015880 PROSITE:PS50157 SMART:SM00355 GO:GO:0008270 GO:GO:0005622 EMBL:AACQ01000030 RefSeq:XP_719625.1 GeneID:3638749 KEGG:cal:CaO19.9599 CGD:CAL0072124 Uniprot:Q5ADE8
Root	Isotig07639	6	37	-2.753	1.87E-07	TAIR|locus:2165580 - symbol:AT5G57330 species:3702 "Arabidopsis thaliana" [GO:0003824 "catalytic activity" evidence=IEA] [GO:0004034 "aldose 1-epimerase activity" evidence=ISS] [GO:0006012 "galactose metabolic process" evidence=ISS] [GO:0016853 "isomerase activity" evidence=IEA] [GO:0030246 "carbohydrate binding" evidence=IEA] [GO:0009737 "response to abscisic acid stimulus" evidence=IDA] [GO:0005829 "cytosol" evidence=IDA] InterPro:IPR008183 InterPro:IPR011013 InterPro:IPR014718 Pfam:PF01263 GO:GO:0005829 GO:GO:0009737 EMBL:CP002688 GenomeReviews:BA000015_GR GO:GO:0005975 GO:GO:0016853 GO:GO:0030246 SUPFAM:SSF74650 Gene3D:G3DSA:2.70.98.10 EMBL:AB019233 eggNOG:COG0676 HOGENOM:HBG737737 KO:K01792 InterPro:IPR025532 PIRSF:PIRSF016020 EMBL:AY093119 EMBL:BT003381 IPI:IPI00546569 RefSeq:NP_200543.1 UniGene:At.9593 HSSP:Q9RP27 ProteinModelPortal:Q9LVC5 SMR:Q9LVC5 STRING:Q9LVC5 PRIDE:Q9LVC5 EnsemblPlants:AT5G57330.1 GeneID:835838 KEGG:ath:AT5G57330 TAIR:At5g57330 InParanoid:Q9LVC5 OMA:EYREMVC PhylomeDB:Q9LVC5 ProtClustDB:CLSN2916812 ArrayExpress:Q9LVC5 Genevestigator:Q9LVC5 Uniprot:Q9LVC5
Root	Isotig07640	6	39	-2.829	5.74E-08	TAIR|locus:2165580 - symbol:AT5G57330 species:3702 "Arabidopsis thaliana" [GO:0003824 "catalytic activity" evidence=IEA] [GO:0004034 "aldose 1-epimerase activity" evidence=ISS] [GO:0006012 "galactose metabolic process" evidence=ISS] [GO:0016853 "isomerase activity" evidence=IEA] [GO:0030246 "carbohydrate binding" evidence=IEA] [GO:0009737 "response to abscisic acid stimulus" evidence=IDA] [GO:0005829 "cytosol" evidence=IDA] InterPro:IPR008183 InterPro:IPR011013 InterPro:IPR014718 Pfam:PF01263 GO:GO:0005829 GO:GO:0009737 EMBL:CP002688 GenomeReviews:BA000015_GR GO:GO:0005975 GO:GO:0016853 GO:GO:0030246 SUPFAM:SSF74650 Gene3D:G3DSA:2.70.98.10 EMBL:AB019233 eggNOG:COG0676 HOGENOM:HBG737737 KO:K01792 InterPro:IPR025532 PIRSF:PIRSF016020 EMBL:AY093119 EMBL:BT003381 IPI:IPI00546569 RefSeq:NP_200543.1 UniGene:At.9593 HSSP:Q9RP27 ProteinModelPortal:Q9LVC5 SMR:Q9LVC5 STRING:Q9LVC5 PRIDE:Q9LVC5 EnsemblPlants:AT5G57330.1 GeneID:835838 KEGG:ath:AT5G57330 TAIR:At5g57330 InParanoid:Q9LVC5 OMA:EYREMVC PhylomeDB:Q9LVC5 ProtClustDB:CLSN2916812 ArrayExpress:Q9LVC5 Genevestigator:Q9LVC5 Uniprot:Q9LVC5
Root	Isotig07645	6	22	-2.003	0.00086822	TAIR|locus:2098343 - symbol:AT3G11680 species:3702 "Arabidopsis thaliana" [GO:0010044 "response to aluminum ion" evidence=IEA] InterPro:IPR020966 Pfam:PF11744 GO:GO:0016021 EMBL:CP002686 GenomeReviews:BA000014_GR GO:GO:0010044 GO:GO:0005216 eggNOG:NOG318607 HOGENOM:HBG749623 EMBL:AC009918 IPI:IPI00517606 RefSeq:NP_187774.1 UniGene:At.50172 PRIDE:Q9SRM9 EnsemblPlants:AT3G11680.1 GeneID:820340 KEGG:ath:AT3G11680 TAIR:At3g11680 InParanoid:Q9SRM9 OMA:MIANNIN PhylomeDB:Q9SRM9 ProtClustDB:CLSN2914776 Genevestigator:Q9SRM9 Uniprot:Q9SRM9

Tissue	Identification	# of reads (stress)	# of reads (control)	Fold Change	p-value	Description
Root	Isotig07667	2	44	-4.588	5.52E-12	TAIR|locus:2015021 - symbol:AT1G22170 species:3702 "Arabidopsis thaliana" [GO:0003824 "catalytic activity" evidence=IEA;ISS] [GO:0006096 "glycolysis" evidence=IEA] [GO:0008152 "metabolic process" evidence=IEA;ISS] [GO:0016868 "intramolecular transferase activity, phosphotransferases" evidence=IEA] HAMAP:MF_01039 InterPro:IPR001345 InterPro:IPR005952 PROSITE:PS00175 Pfam:PF00300 EMBL:CP002684 InterPro:IPR013078 SMART:SM00855 GO:GO:0006096 EMBL:AC073942 KO:K01834 PANTHER:PTHR11931 OMA:YWNDVII GO:GO:0016868 HSSP:P00950 EMBL:BT020337 IPI:IPI00547090 PIR:C86354 RefSeq:NP_564161.1 UniGene:At.41618 UniGene:At.43225 ProteinModelPortal:Q9LM13 SMR:Q9LM13 PRIDE:Q9LM13 EnsemblPlants:AT1G22170.1 GeneID:838822 KEGG:ath:AT1G22170 TAIR:At1g22170 InParanoid:Q9LM13 PhylomeDB:Q9LM13 ProtClustDB:CLSN2687909 Genevestigator:Q9LM13 Uniprot:Q9LM13
Root	Isotig07668	2	44	-4.588	5.52E-12	TAIR|locus:2015021 - symbol:AT1G22170 species:3702 "Arabidopsis thaliana" [GO:0003824 "catalytic activity" evidence=IEA;ISS] [GO:0006096 "glycolysis" evidence=IEA] [GO:0008152 "metabolic process" evidence=IEA;ISS] [GO:0016868 "intramolecular transferase activity, phosphotransferases" evidence=IEA] HAMAP:MF_01039 InterPro:IPR001345 InterPro:IPR005952 PROSITE:PS00175 Pfam:PF00300 EMBL:CP002684 InterPro:IPR013078 SMART:SM00855 GO:GO:0006096 EMBL:AC073942 KO:K01834 PANTHER:PTHR11931 OMA:YWNDVII GO:GO:0016868 HSSP:P00950 EMBL:BT020337 IPI:IPI00547090 PIR:C86354 RefSeq:NP_564161.1 UniGene:At.41618 UniGene:At.43225 ProteinModelPortal:Q9LM13 SMR:Q9LM13 PRIDE:Q9LM13 EnsemblPlants:AT1G22170.1 GeneID:838822 KEGG:ath:AT1G22170 TAIR:At1g22170 InParanoid:Q9LM13 PhylomeDB:Q9LM13 ProtClustDB:CLSN2687909 Genevestigator:Q9LM13 Uniprot:Q9LM13
Root	Isotig07673	4	23	-2.653	5.61E-05	TAIR|locus:2170428 - symbol:KASI "3-ketoacyl-acyl carrier protein synthase I" species:3702 "Arabidopsis thaliana" [GO:0003824 "catalytic activity" evidence=IEA] [GO:0004312 "fatty acid synthase activity" evidence=ISS] [GO:0016747 "transferase activity, transferring acyl groups other than amino-acyl groups" evidence=IEA] [GO:0009941 "chloroplast envelope" evidence=IDA] [GO:0009570 "chloroplast stroma" evidence=IDA] [GO:0016020 "membrane" evidence=IDA] [GO:0009507 "chloroplast" evidence=IDA] [GO:0009790 "embryo development" evidence=IMP] [GO:0010020 "chloroplast fission" evidence=IMP] InterPro:IPR000794 InterPro:IPR016038 InterPro:IPR016039 InterPro:IPR017568 Pfam:PF02801 Pfam:PF00109 Prosite:PS00606 GO:GO:0009570 EMBL:CP002688 GenomeReviews:BA000015_GR GO:GO:0016020 GO:GO:0009790 GO:GO:0009941 GO:GO:0006633 GO:GO:0010020 Gene3D:G3DSA:3.40.47.10 SUPFAM:SSF53901 PANTHER:PTHR11712 eggNOG:COG0304 HOGENOM:HBG757733 GO:GO:0004315 InterPro:IPR018201 InterPro:IPR014031 InterPro:IPR014030 TIGRFAMs:TIGR03150 EMBL:AB010698 EMBL:U24177 EMBL:AY037261 EMBL:AY094005 EMBL:AY123979 EMBL:AY087843 IPI:IPI00541557 IPI:IPI00657465 RefSeq:NP_001032018.1 RefSeq:NP_199441.1 UniGene:At.9183 ProteinModelPortal:P52410 SMR:P52410 STRING:P52410 PRIDE:P52410 EnsemblPlants:AT5G46290.1 GeneID:834671 KEGG:ath:AT5G46290 TAIR:At5g46290 InParanoid:P52410 OMA:NAQNTSQ PhylomeDB:P52410 ProtClustDB:CLSN2715653 BioCyc:MetaCyc:MONOMER-14117 ArrayExpress:P52410 Genevestigator:P52410 Uniprot:P52410
Root	Isotig07674	4	23	-2.653	5.61E-05	TAIR|locus:2170428 - symbol:KASI "3-ketoacyl-acyl carrier protein synthase I" species:3702 "Arabidopsis thaliana" [GO:0003824 "catalytic activity" evidence=IEA] [GO:0004312 "fatty acid synthase activity" evidence=ISS] [GO:0016747 "transferase activity, transferring acyl groups other than amino-acyl groups" evidence=IEA] [GO:0009941 "chloroplast envelope" evidence=IDA] [GO:0009570 "chloroplast stroma" evidence=IDA] [GO:0016020 "membrane" evidence=IDA] [GO:0009507 "chloroplast" evidence=IDA] [GO:0009790 "embryo development" evidence=IMP] [GO:0010020 "chloroplast fission" evidence=IMP] InterPro:IPR000794 InterPro:IPR016038 InterPro:IPR016039 InterPro:IPR017568 Pfam:PF02801 Pfam:PF00109 Prosite:PS00606 GO:GO:0009570 EMBL:CP002688 GenomeReviews:BA000015_GR GO:GO:0016020 GO:GO:0009790 GO:GO:0009941 GO:GO:0006633 GO:GO:0010020 Gene3D:G3DSA:3.40.47.10 SUPFAM:SSF53901 PANTHER:PTHR11712 eggNOG:COG0304 HOGENOM:HBG757733 GO:GO:0004315 InterPro:IPR018201 InterPro:IPR014031 InterPro:IPR014030 TIGRFAMs:TIGR03150 EMBL:AB010698 EMBL:U24177 EMBL:AY037261 EMBL:AY094005 EMBL:AY123979 EMBL:AY087843 IPI:IPI00541557 IPI:IPI00657465 RefSeq:NP_001032018.1 RefSeq:NP_199441.1 UniGene:At.9183 ProteinModelPortal:P52410 SMR:P52410 STRING:P52410 PRIDE:P52410 EnsemblPlants:AT5G46290.1 GeneID:834671 KEGG:ath:AT5G46290 TAIR:At5g46290 InParanoid:P52410 OMA:NAQNTSQ PhylomeDB:P52410 ProtClustDB:CLSN2715653 BioCyc:MetaCyc:MONOMER-14117 ArrayExpress:P52410 Genevestigator:P52410 Uniprot:P52410
Root	Isotig07675	2	29	-3.987	6.83E-08	TAIR|locus:2007651 - symbol:TPPD "trehalose-6-phosphate phosphatase D" species:3702 "Arabidopsis thaliana" [GO:0003824 "catalytic activity" evidence=IEA] [GO:0004805 "trehalose-phosphatase activity" evidence=ISS]

Tissue	Identification	# of reads (stress)	# of reads (control)	Fold Change	p-value	Description
						[GO:0005992 "trehalose biosynthetic process" evidence=IEA;ISS] [GO:0008152 "metabolic process" evidence=IEA] InterPro:IPR003337 InterPro:IPR006379 Pfam:PF02358 EMBL:CP002684 GenomeReviews:CT485782_GR InterPro:IPR023214 Gene3D:G3DSA:3.40.50.1000 SUPFAM:SSF56784 GO:GO:0016787 GO:GO:0005992 TIGRFAMs:TIGR01484 eggNOG:COG1877 HOGENOM:HBG686849 OMA:EGANVEN TIGRFAMs:TIGR00685 EMBL:AK176890 IPI:IPI00519798 RefSeq:NP_564464.1 UniGene:At.39452 ProteinModelPortal:Q67XC9 PRIDE:Q67XC9 EnsemblPlants:AT1G35910.1 GeneID:840493 KEGG:ath:AT1G35910 TAIR:At1g35910 InParanoid:Q67XC9 PhylomeDB:Q67XC9 ProtClustDB:CLSN2914984 ArrayExpress:Q67XC9 Genevestigator:Q67XC9 Uniprot:Q67XC9
Root	Isotig07676	2	29	-3.987	6.83E-08	TAIR|locus:2007651 - symbol:TPPD "trehalose-6-phosphate phosphatase D" species:3702 "Arabidopsis thaliana" [GO:0003824 "catalytic activity" evidence=IEA] [GO:0004805 "trehalose-phosphatase activity" evidence=ISS] [GO:0005992 "trehalose biosynthetic process" evidence=IEA;ISS] [GO:0008152 "metabolic process" evidence=IEA] InterPro:IPR003337 InterPro:IPR006379 Pfam:PF02358 EMBL:CP002684 GenomeReviews:CT485782_GR InterPro:IPR023214 Gene3D:G3DSA:3.40.50.1000 SUPFAM:SSF56784 GO:GO:0016787 GO:GO:0005992 TIGRFAMs:TIGR01484 eggNOG:COG1877 HOGENOM:HBG686849 OMA:EGANVEN TIGRFAMs:TIGR00685 EMBL:AK176890 IPI:IPI00519798 RefSeq:NP_564464.1 UniGene:At.39452 ProteinModelPortal:Q67XC9 PRIDE:Q67XC9 EnsemblPlants:AT1G35910.1 GeneID:840493 KEGG:ath:AT1G35910 TAIR:At1g35910 InParanoid:Q67XC9 PhylomeDB:Q67XC9 ProtClustDB:CLSN2914984 ArrayExpress:Q67XC9 Genevestigator:Q67XC9 Uniprot:Q67XC9
Root	Isotig07685	0	32	-6.129	1.84E-09	TAIR|locus:2165897 - symbol:GS2 "AT5G35630" species:3702 "Arabidopsis thaliana" [GO:0004356 "glutamate-ammonia ligase activity" evidence=ISS;IDA;TAS;IPI] [GO:0007568 "aging" evidence=TAS] [GO:0009507 "chloroplast" evidence=IDA;NAS] [GO:0009941 "chloroplast envelope" evidence=IDA] [GO:0005739 "mitochondrion" evidence=IDA] [GO:0009535 "chloroplast thylakoid membrane" evidence=IDA] [GO:0022626 "cytosolic ribosome" evidence=IDA] [GO:0009570 "chloroplast stroma" evidence=IDA] [GO:0019676 "ammonia assimilation cycle" evidence=TAS] [GO:0009579 "thylakoid" evidence=IDA] [GO:0016020 "membrane" evidence=IDA] [GO:0009651 "response to salt stress" evidence=IEP] [GO:0048046 "apoplast" evidence=IDA] [GO:0046686 "response to cadmium ion" evidence=IEP] InterPro:IPR008146 InterPro:IPR008147 InterPro:IPR014746 Pfam:PF00120 Pfam:PF03951 PROSITE:PS00180 PROSITE:PS00181 GO:GO:0005739 GO:GO:0005524 GO:GO:0048046 GO:GO:0009570 GO:GO:0046686 EMBL:CP002688 GenomeReviews:BA000015_GR GO:GO:0007568 GO:GO:0009941 GO:GO:0009651 GO:GO:0009535 GO:GO:0022626 GO:GO:0019676 eggNOG:COG0174 HOGENOM:HBG299709 KO:K01915 GO:GO:0004356 GO:GO:0006542 Gene3D:G3DSA:3.30.590.10 SUPFAM:SSF54368 EMBL:S69727 EMBL:AB015045 EMBL:AB013393 EMBL:AF428319 EMBL:AF428461 EMBL:AY081252 EMBL:AY091114 EMBL:AY122977 EMBL:AY088222 IPI:IPI00534852 PIR:S18600 RefSeq:NP_001031969.1 RefSeq:NP_001078639.1 RefSeq:NP_198413.1 UniGene:At.24218 ProteinModelPortal:Q43127 SMR:Q43127 DIP:DIP-32735N IntAct:Q43127 STRING:Q43127 SWISS-2DPAGE:Q43127 PRIDE:Q43127 EnsemblPlants:AT5G35630.1 EnsemblPlants:AT5G35630.2 EnsemblPlants:AT5G35630.3 GeneID:833535 KEGG:ath:AT5G35630 TAIR:At5g35630 InParanoid:Q43127 OMA:TLDPKPI PhylomeDB:Q43127 ProtClustDB:PLN03036 ArrayExpress:Q43127 Genevestigator:Q43127 GermOnline:AT5G35630 Uniprot:Q43127
Root	Isotig07686	0	29	-5.987	1.01E-08	TAIR|locus:2165897 - symbol:GS2 "AT5G35630" species:3702 "Arabidopsis thaliana" [GO:0004356 "glutamate-ammonia ligase activity" evidence=ISS;IDA;TAS;IPI] [GO:0007568 "aging" evidence=TAS] [GO:0009507 "chloroplast" evidence=IDA;NAS] [GO:0009941 "chloroplast envelope" evidence=IDA] [GO:0005739 "mitochondrion" evidence=IDA] [GO:0009535 "chloroplast thylakoid membrane" evidence=IDA] [GO:0022626 "cytosolic ribosome" evidence=IDA] [GO:0009570 "chloroplast stroma" evidence=IDA] [GO:0019676 "ammonia assimilation cycle" evidence=TAS] [GO:0009579 "thylakoid" evidence=IDA] [GO:0016020 "membrane" evidence=IDA] [GO:0009651 "response to salt stress" evidence=IEP] [GO:0048046 "apoplast" evidence=IDA] [GO:0046686 "response to cadmium ion" evidence=IEP] InterPro:IPR008146 InterPro:IPR008147 InterPro:IPR014746 Pfam:PF00120 Pfam:PF03951 PROSITE:PS00180 PROSITE:PS00181 GO:GO:0005739 GO:GO:0005524 GO:GO:0048046 GO:GO:0009570 GO:GO:0046686 EMBL:CP002688 GenomeReviews:BA000015_GR GO:GO:0007568 GO:GO:0009941 GO:GO:0009651 GO:GO:0009535 GO:GO:0022626 GO:GO:0019676 eggNOG:COG0174 HOGENOM:HBG299709 KO:K01915 GO:GO:0004356 GO:GO:0006542 Gene3D:G3DSA:3.30.590.10 SUPFAM:SSF54368 EMBL:S69727 EMBL:AB015045 EMBL:AB013393 EMBL:AF428319 EMBL:AF428461 EMBL:AY081252 EMBL:AY091114 EMBL:AY122977 EMBL:AY088222 IPI:IPI00534852 PIR:S18600

Tissue	Identification	# of reads (stress)	# of reads (control)	Fold Change	p-value	Description
						RefSeq:NP_001031969.1 RefSeq:NP_001078639.1 RefSeq:NP_198413.1 UniGene:At.24218 ProteinModelPortal:Q43127 SMR:Q43127 DIP:DIP-32735N IntAct:Q43127 STRING:Q43127 SWISS-2DPAGE:Q43127 PRIDE:Q43127 EnsemblPlants:AT5G35630.1 EnsemblPlants:AT5G35630.2 EnsemblPlants:AT5G35630.3 GeneID:833535 KEGG:ath:AT5G35630 TAIR:At5g35630 InParanoid:Q43127 OMA:TLDPKPI PhylomeDB:Q43127 ProtClustDB:PLN03036 ArrayExpress:Q43127 Genevestigator:Q43127 GermOnline:AT5G35630 Uniprot:Q43127
Root	Isotig07703	12	50	-2.188	1.10E-07	TAIR|locus:2028145 - symbol:RFNR2 "AT1G30510" species:3702 "Arabidopsis thaliana" [GO:0016491 "oxidoreductase activity" evidence=ISS] [GO:0042651 "thylakoid membrane" evidence=IEA] [GO:0055114 "oxidation-reduction process" evidence=IEA] [GO:0005507 "copper ion binding" evidence=IDA] InterPro:IPR001433 InterPro:IPR001709 InterPro:IPR012146 InterPro:IPR015701 InterPro:IPR017927 InterPro:IPR017938 Pfam:PF00175 PIRSF:PIRSF000361 PRINTS:PR00371 PROSITE:PS51384 EMBL:CP002684 GenomeReviews:CT485782_GR GO:GO:0009507 GO:GO:0050660 GO:GO:0006810 GO:GO:0050661 GO:GO:0005507 EMBL:AC009917 GO:GO:0022900 GO:GO:0004324 SUPFAM:SSF63380 GO:GO:0015979 GO:GO:0042651 eggNOG:COG0369 PANTHER:PTHR19384:SF1 HOGENOM:HBG353752 KO:K02641 HSSP:P00455 ProtClustDB:PLN03116 EMBL:AF424594 EMBL:AY140017 EMBL:BT008468 EMBL:AY088018 IPI:IPI00525557 IPI:IPI00529741 IPI:IPI00534215 PIR:B86430 RefSeq:NP_564355.1 RefSeq:NP_849734.1 RefSeq:NP_973942.1 UniGene:At.25172 ProteinModelPortal:Q9S9P8 SMR:Q9S9P8 PRIDE:Q9S9P8 EnsemblPlants:AT1G30510.2 GeneID:839930 KEGG:ath:AT1G30510 TAIR:At1g30510 InParanoid:Q9S9P8 OMA:FLCDSKP PhylomeDB:Q9S9P8 ArrayExpress:Q9S9P8 Genevestigator:Q9S9P8 Uniprot:Q9S9P8
Root	Isotig07704	12	49	-2.159	1.86E-07	TAIR|locus:2028145 - symbol:RFNR2 "AT1G30510" species:3702 "Arabidopsis thaliana" [GO:0016491 "oxidoreductase activity" evidence=ISS] [GO:0042651 "thylakoid membrane" evidence=IEA] [GO:0055114 "oxidation-reduction process" evidence=IEA] [GO:0005507 "copper ion binding" evidence=IDA] InterPro:IPR001433 InterPro:IPR001709 InterPro:IPR012146 InterPro:IPR015701 InterPro:IPR017927 InterPro:IPR017938 Pfam:PF00175 PIRSF:PIRSF000361 PRINTS:PR00371 PROSITE:PS51384 EMBL:CP002684 GenomeReviews:CT485782_GR GO:GO:0009507 GO:GO:0050660 GO:GO:0006810 GO:GO:0050661 GO:GO:0005507 EMBL:AC009917 GO:GO:0022900 GO:GO:0004324 SUPFAM:SSF63380 GO:GO:0015979 GO:GO:0042651 eggNOG:COG0369 PANTHER:PTHR19384:SF1 HOGENOM:HBG353752 KO:K02641 HSSP:P00455 ProtClustDB:PLN03116 EMBL:AF424594 EMBL:AY140017 EMBL:BT008468 EMBL:AY088018 IPI:IPI00525557 IPI:IPI00529741 IPI:IPI00534215 PIR:B86430 RefSeq:NP_564355.1 RefSeq:NP_849734.1 RefSeq:NP_973942.1 UniGene:At.25172 ProteinModelPortal:Q9S9P8 SMR:Q9S9P8 PRIDE:Q9S9P8 EnsemblPlants:AT1G30510.2 GeneID:839930 KEGG:ath:AT1G30510 TAIR:At1g30510 InParanoid:Q9S9P8 OMA:FLCDSKP PhylomeDB:Q9S9P8 ArrayExpress:Q9S9P8 Genevestigator:Q9S9P8 Uniprot:Q9S9P8
Root	Isotig07713	175	19	3.074	2.39E-30	ZFIN|ZDB-GENE-070410-9 - symbol:zgc:158463 "zgc:158463" species:7955 "Danio rerio" [GO:0003674 "molecular_function" evidence=ND] [GO:0005575 "cellular_component" evidence=ND] [GO:0008150 "biological_process" evidence=ND] ZFIN:ZDB-GENE-070410-9 EMBL:BX296557 EMBL:BX537263 EMBL:BC129240 IPI:IPI00837201 RefSeq:NP_001091866.1 RefSeq:XP_003200836.1 UniGene:Dr.155369 Ensembl:ENSDART00000102863 Ensembl:ENSDART00000126982 GeneID:100037361 GeneID:100151188 KEGG:dre:100037361 KEGG:dre:100151188 eggNOG:NOG298153 GeneTree:ENSGT00390000016898 InParanoid:A2VD29 OMA:ACHQHAL OrthoDB:EOG4FJ8BF Bgee:A2VD29 Uniprot:A2VD29
Root	Isotig07714	124	12	3.240	5.66E-23	No hit
Root	Isotig07721	31	140	-2.304	4.67E-20	TAIR|locus:2075236 - symbol:MYB48 "AT3G46130" species:3702 "Arabidopsis thaliana" [GO:0003677 "DNA binding" evidence=ISS] [GO:0003700 "sequence-specific DNA binding transcription factor activity" evidence=ISS] [GO:0006355 "regulation of transcription, DNA-dependent" evidence=ISS;TAS] [GO:0009751 "response to salicylic acid stimulus" evidence=IEP] [GO:0005634 "nucleus" evidence=IDA] InterPro:IPR001005 InterPro:IPR009057 Pfam:PF00249 SMART:SM00717 GO:GO:0005634 EMBL:CP002686 GenomeReviews:BA000014_GR GO:GO:0006355 GO:GO:0003677 GO:GO:0006351 GO:GO:0009751 Gene3D:G3DSA:1.10.10.60 SUPFAM:SSF46689 InterPro:IPR017930 PROSITE:PS51294 eggNOG:COG5147 InterPro:IPR015495 PANTHER:PTHR10641 EMBL:AL355775 HOGENOM:HBG750380 EMBL:AF272733 EMBL:AY519594 EMBL:DQ075255 EMBL:DQ075256 EMBL:DQ075257 EMBL:AK176475 IPI:IPI00528378 IPI:IPI00656574 IPI:IPI00759334 IPI:IPI00759367 PIR:T49254 RefSeq:NP_001030816.1 RefSeq:NP_001078249.1 RefSeq:NP_190199.1 UniGene:At.1475 UniGene:At.72744 ProteinModelPortal:Q9LX82 SMR:Q9LX82 STRING:Q9LX82 EnsemblPlants:AT3G46130.1 GeneID:823756 KEGG:ath:AT3G46130 GeneFarm:1132

Tissue	Identification	# of reads (stress)	# of reads (control)	Fold Change	p-value	Description
						TAIR:At3g46130 InParanoid:Q9LX82 OMA:REIDHSA PhylomeDB:Q9LX82 ProtClustDB:CLSN2684805 Genevestigator:Q9LX82 GermOnline:AT3G46130 Uniprot:Q9LX82
Root	Isotig07722	33	137	-2.183	1.68E-18	TAIR|locus:2174324 - symbol:MYB59 "AT5G59780" species:3702 "Arabidopsis thaliana" [GO:0003677 "DNA binding" evidence=ISS] [GO:0006355 "regulation of transcription, DNA-dependent" evidence=ISS] [GO:0003700 "sequence-specific DNA binding transcription factor activity" evidence=ISS] [GO:0009723 "response to ethylene stimulus" evidence=IEP] [GO:0009739 "response to gibberellin stimulus" evidence=IEP] [GO:0009751 "response to salicylic acid stimulus" evidence=IEP] [GO:0009753 "response to jasmonic acid stimulus" evidence=IEP] [GO:0046686 "response to cadmium ion" evidence=IEP] [GO:0005634 "nucleus" evidence=IDA] [GO:0010200 "response to chitin" evidence=IEP] InterPro:IPR001005 InterPro:IPR009057 Pfam:PF00249 SMART:SM00717 GO:GO:0005634 GO:GO:0046686 EMBL:CP002688 GenomeReviews:BA000015_GR GO:GO:0009753 GO:GO:0006355 GO:GO:0003677 GO:GO:0006351 GO:GO:0009723 GO:GO:0009751 Gene3D:G3DSA:1.10.10.60 SUPFAM:SSF46689 GO:GO:0010200 GO:GO:0009739 InterPro:IPR017930 PROSITE:PS51294 eggNOG:COG5147 KO:K09422 InterPro:IPR015495 PANTHER:PTHR10641 EMBL:AB006705 EMBL:AF062894 EMBL:AY519641 EMBL:DQ075252 EMBL:DQ075253 EMBL:DQ075254 EMBL:AK229048 EMBL:Z95781 EMBL:Z95772 IPI:IPI00528791 IPI:IPI00545664 IPI:IPI00548069 IPI:IPI00759289 PIR:T51666 RefSeq:NP_200786.1 RefSeq:NP_851225.1 RefSeq:NP_851226.1 UniGene:At.22428 UniGene:At.70065 ProteinModelPortal:Q4JL84 SMR:Q4JL84 STRING:Q4JL84 PRIDE:Q4JL84 EnsemblPlants:AT5G59780.3 GeneID:836099 KEGG:ath:AT5G59780 GeneFarm:952 TAIR:At5g59780 InParanoid:Q4JL84 OMA:WESSLES PhylomeDB:Q4JL84 ProtClustDB:CLSN2916286 ArrayExpress:Q9FN86 Genevestigator:Q4JL84 GermOnline:AT5G59780 Uniprot:Q4JL84
Root	Isotig07739	22	54	-1.424	4.11E-05	TAIR|locus:2064965 - symbol:EULS3 "AT2G39050" species:3702 "Arabidopsis thaliana" [GO:0008150 "biological_process" evidence=ND] [GO:0005634 "nucleus" evidence=IDA] [GO:0005737 "cytoplasm" evidence=IDA] [GO:0030246 "carbohydrate binding" evidence=IDA] GO:GO:0005634 GO:GO:0005737 EMBL:CP002685 GenomeReviews:CT485783_GR InterPro:IPR000772 SUPFAM:SSF50370 PROSITE:PS50231 GO:GO:0030246 EMBL:AC005770 Pfam:PF14200 EMBL:AF411801 EMBL:AY093795 EMBL:AY087909 IPI:IPI00536641 PIR:E84812 RefSeq:NP_565899.1 UniGene:At.22599 ProteinModelPortal:Q945P1 SMR:Q945P1 PRIDE:Q945P1 EnsemblPlants:AT2G39050.1 GeneID:818491 KEGG:ath:AT2G39050 TAIR:At2g39050 eggNOG:NOG254161 HOGENOM:HBG750017 InParanoid:Q945P1 OMA:GYRTIRM PhylomeDB:Q945P1 ProtClustDB:CLSN2681741 ArrayExpress:Q945P1 Genevestigator:Q945P1 Uniprot:Q945P1
Root	Isotig07740	21	57	-1.570	5.97E-06	TAIR|locus:2064965 - symbol:EULS3 "AT2G39050" species:3702 "Arabidopsis thaliana" [GO:0008150 "biological_process" evidence=ND] [GO:0005634 "nucleus" evidence=IDA] [GO:0005737 "cytoplasm" evidence=IDA] [GO:0030246 "carbohydrate binding" evidence=IDA] GO:GO:0005634 GO:GO:0005737 EMBL:CP002685 GenomeReviews:CT485783_GR InterPro:IPR000772 SUPFAM:SSF50370 PROSITE:PS50231 GO:GO:0030246 EMBL:AC005770 Pfam:PF14200 EMBL:AF411801 EMBL:AY093795 EMBL:AY087909 IPI:IPI00536641 PIR:E84812 RefSeq:NP_565899.1 UniGene:At.22599 ProteinModelPortal:Q945P1 SMR:Q945P1 PRIDE:Q945P1 EnsemblPlants:AT2G39050.1 GeneID:818491 KEGG:ath:AT2G39050 TAIR:At2g39050 eggNOG:NOG254161 HOGENOM:HBG750017 InParanoid:Q945P1 OMA:GYRTIRM PhylomeDB:Q945P1 ProtClustDB:CLSN2681741 ArrayExpress:Q945P1 Genevestigator:Q945P1 Uniprot:Q945P1
Root	Isotig07745	19	52	-1.581	1.37E-05	TAIR|locus:2119251 - symbol:AT4G33420 species:3702 "Arabidopsis thaliana" [GO:0004601 "peroxidase activity" evidence=IEA;ISS] [GO:0006979 "response to oxidative stress" evidence=IEA] [GO:0020037 "heme binding" evidence=IEA] [GO:0055114 "oxidation-reduction process" evidence=IEA] InterPro:IPR000823 InterPro:IPR002016 InterPro:IPR010255 InterPro:IPR019793 InterPro:IPR019794 Pfam:PF00141 PRINTS:PR00458 PRINTS:PR00461 PROSITE:PS00435 PROSITE:PS00436 PROSITE:PS50873 EMBL:CP002687 GenomeReviews:CT486007_GR GO:GO:0005576 GO:GO:0006979 EMBL:AL161583 EMBL:AL035678 GO:GO:0020037 GO:GO:0004601 HOGENOM:HBG597790 SUPFAM:SSF48113 KO:K00430 EMBL:AF451951 EMBL:BT044614 IPI:IPI00539387 PIR:T05993 RefSeq:NP_567919.1 UniGene:At.28650 ProteinModelPortal:Q9SZB9 SMR:Q9SZB9 STRING:Q9SZB9 PeroxiBase:213 PRIDE:Q9SZB9 EnsemblPlants:AT4G33420.1 GeneID:829479 KEGG:ath:AT4G33420 GeneFarm:1879 TAIR:At4g33420 eggNOG:NOG276365 InParanoid:Q9SZB9 ProtClustDB:CLSN2689733 Genevestigator:Q9SZB9 GermOnline:AT4G33420 Uniprot:Q9SZB9
Root	Isotig07747	1	14	-3.936	0.000188601	TAIR|locus:2183379 - symbol:GDH2 "AT5G07440" species:3702 "Arabidopsis thaliana" [GO:0016491 "oxidoreductase activity" evidence=ISS] [GO:0005739 "mitochondrion" evidence=IDA] [GO:0046686 "response to cadmium ion" evidence=IEP] [GO:0005524 "ATP binding" evidence=IDA] [GO:0005774 "vacuolar membrane"

Tissue	Identification	# of reads (stress)	# of reads (control)	Fold Change	p-value	Description
						evidence=IDA] [GO:0009651 "response to salt stress" evidence=IEP] [GO:0004352 "glutamate dehydrogenase (NAD+) activity" evidence=IDA] [GO:0005507 "copper ion binding" evidence=IDA] [GO:0008270 "zinc ion binding" evidence=IDA] [GO:0050897 "cobalt ion binding" evidence=IDA] [GO:0005794 "Golgi apparatus" evidence=RCA] [GO:0004353 "glutamate dehydrogenase [NAD(P)+] activity" evidence=IDA] InterPro:IPR006095 InterPro:IPR006096 InterPro:IPR006097 InterPro:IPR014362 InterPro:IPR016040 Pfam:PF00208 Pfam:PF02812 PIRSF:PIRSF000185 PRINTS:PR00082 PROSITE:PS00074 SMART:SM00839 GO:GO:0005739 GO:GO:0005524 GO:GO:0046686 GO:GO:0005774 EMBL:CP002688 GenomeReviews:BA000015_GR Gene3D:G3DSA:3.40.50.720 GO:GO:0008270 GO:GO:0005507 GO:GO:0006520 GO:GO:0009651 GO:GO:0050897 EMBL:AL163912 eggNOG:COG0334 HOGENOM:HBG590661 KO:K00261 ProtClustDB:PLN02477 GO:GO:0004353 EMBL:U56635 IPI:IPI00543481 PIR:T49883 RefSeq:NP_001119183.1 RefSeq:NP_196361.1 UniGene:At.21712 ProteinModelPortal:Q38946 SMR:Q38946 STRING:Q38946 PRIDE:Q38946 ProMEX:Q38946 EnsemblPlants:AT5G07440.1 EnsemblPlants:AT5G07440.2 GeneID:830635 KEGG:ath:AT5G07440 GeneFarm:2247 TAIR:At5g07440 InParanoid:Q38946 OMA:NTGHTEE PhylomeDB:Q38946 ArrayExpress:Q38946 Genevestigator:Q38946 GermOnline:AT5G07440 GO:GO:0004352 Uniprot:Q38946
Root	Isotig07773	4	126	-5.106	1.81E-32	UNIPROTKB|A8QW53 - symbol:OMT3 "5-pentadecatrienyl resorcinol O-methyltransferase" species:4558 "Sorghum bicolor" [GO:0008171 "O-methyltransferase activity" evidence=IDA] [GO:0008757 "S-adenosylmethionine-dependent methyltransferase activity" evidence=IDA] [GO:0032259 "methylation" evidence=IDA] InterPro:IPR001077 InterPro:IPR012967 InterPro:IPR016461 Pfam:PF00891 Pfam:PF08100 PIRSF:PIRSF005739 InterPro:IPR011991 Gene3D:G3DSA:1.10.10.10 GO:GO:0046983 GO:GO:0008171 GO:GO:0008757 EMBL:EF189708 EMBL:CM000765 RefSeq:XP_002447425.1 UniGene:Sbi.19698 ProteinModelPortal:A8QW53 EnsemblPlants:Sb06g000820.1 GeneID:8080259 KEGG:sbi:SORBI_06g000820 eggNOG:NOG272168 PhylomeDB:A8QW53 ProtClustDB:CLSN2725062 Uniprot:A8QW53
Root	Isotig07774	5	112	-4.614	3.50E-28	UNIPROTKB|A8QW53 - symbol:OMT3 "5-pentadecatrienyl resorcinol O-methyltransferase" species:4558 "Sorghum bicolor" [GO:0008171 "O-methyltransferase activity" evidence=IDA] [GO:0008757 "S-adenosylmethionine-dependent methyltransferase activity" evidence=IDA] [GO:0032259 "methylation" evidence=IDA] InterPro:IPR001077 InterPro:IPR012967 InterPro:IPR016461 Pfam:PF00891 Pfam:PF08100 PIRSF:PIRSF005739 InterPro:IPR011991 Gene3D:G3DSA:1.10.10.10 GO:GO:0046983 GO:GO:0008171 GO:GO:0008757 EMBL:EF189708 EMBL:CM000765 RefSeq:XP_002447425.1 UniGene:Sbi.19698 ProteinModelPortal:A8QW53 EnsemblPlants:Sb06g000820.1 GeneID:8080259 KEGG:sbi:SORBI_06g000820 eggNOG:NOG272168 PhylomeDB:A8QW53 ProtClustDB:CLSN2725062 Uniprot:A8QW53
Root	Isotig07783	3	21	-2.936	4.98E-05	UNIPROTKB|P29717 - symbol:XOG1 "Glucan 1,3-beta-glucosidase" species:237561 "Candida albicans SC5314" [GO:0004338 "glucan exo-1,3-beta-glucosidase activity" evidence=IMP;IDA] [GO:0005576 "extracellular region" evidence=IDA] [GO:0006073 "cellular glucan metabolic process" evidence=IMP] [GO:0009986 "cell surface" evidence=IDA] [GO:0031505 "fungal-type cell wall organization" evidence=IMP] [GO:0031589 "cell-substrate adhesion" evidence=IDA] [GO:0050839 "cell adhesion molecule binding" evidence=IDA] InterPro:IPR001547 InterPro:IPR013781 InterPro:IPR018087 Pfam:PF00150 PROSITE:PS00659 GO:GO:0005576 EMBL:AACQ01000015 GO:GO:0009986 GO:GO:0043169 InterPro:IPR017853 Gene3D:G3DSA:3.20.20.80 SUPFAM:SSF51445 CAZy:GH5 GO:GO:0031505 GO:GO:0050839 GO:GO:0031589 EMBL:AL033497 KO:K01210 GO:GO:0004338 eggNOG:COG2730 GO:GO:0006073 EMBL:X56556 PIR:A47702 PIR:T52149 RefSeq:XP_721488.1 PDB:1CZ1 PDB:1EQC PDB:1EQP PDB:2PB1 PDB:2PBO PDB:2PC8 PDB:2PF0 PDB:3N9K PDB:3O6A PDBsum:1CZ1 PDBsum:1EQC PDBsum:1EQP PDBsum:2PB1 PDBsum:2PBO PDBsum:2PC8 PDBsum:2PF0 PDBsum:3N9K PDBsum:3O6A ProteinModelPortal:P29717 SMR:P29717 STRING:P29717 GeneID:3636837 KEGG:cal:CaO19.2990 PhylomeDB:P29717 PMAP-CutDB:P29717 Uniprot:P29717
Root	Isotig07784	2	20	-3.451	2.08E-05	UNIPROTKB|P29717 - symbol:XOG1 "Glucan 1,3-beta-glucosidase" species:237561 "Candida albicans SC5314" [GO:0004338 "glucan exo-1,3-beta-glucosidase activity" evidence=IMP;IDA] [GO:0005576 "extracellular region" evidence=IDA] [GO:0006073 "cellular glucan metabolic process" evidence=IMP] [GO:0009986 "cell surface" evidence=IDA] [GO:0031505 "fungal-type cell wall organization" evidence=IMP] [GO:0031589 "cell-substrate adhesion" evidence=IDA] [GO:0050839 "cell adhesion molecule binding" evidence=IDA] InterPro:IPR001547 InterPro:IPR013781 InterPro:IPR018087 Pfam:PF00150 PROSITE:PS00659 GO:GO:0005576 EMBL:AACQ01000015 GO:GO:0009986 GO:GO:0043169 InterPro:IPR017853 Gene3D:G3DSA:3.20.20.80 SUPFAM:SSF51445 CAZy:GH5 GO:GO:0031505 GO:GO:0050839 GO:GO:0031589 EMBL:AL033497 KO:K01210 GO:GO:0004338 eggNOG:COG2730 GO:GO:0006073 EMBL:X56556 PIR:A47702 PIR:T52149

Tissue	Identification	# of reads (stress)	# of reads (control)	Fold Change	p-value	Description
						RefSeq:XP_721488.1 PDB:1CZ1 PDB:1EQC PDB:1EQP PDB:2PB1 PDB:2PBO PDB:2PC8 PDB:2PF0 PDB:3N9K PDB:3O6A PDBsum:1CZ1 PDBsum:1EQC PDBsum:1EQP PDBsum:2PB1 PDBsum:2PBO PDBsum:2PC8 PDBsum:2PF0 PDBsum:3N9K PDBsum:3O6A ProteinModelPortal:P29717 SMR:P29717 STRING:P29717 GeneID:3636837 KEGG:cal:CaO19.2990 PhylomeDB:P29717 PMAP-CutDB:P29717 Uniprot:P29717
Root	Isotig07789	2	21	-3.521	1.10E-05	TAIR|locus:2200980 - symbol:PDH-E1 ALPHA "AT1G01090" species:3702 "Arabidopsis thaliana" [GO:0004739 "pyruvate dehydrogenase (acetyl-transferring) activity" evidence=ISS] [GO:0006096 "glycolysis" evidence=IEA] [GO:0008152 "metabolic process" evidence=IEA;ISS] [GO:0055114 "oxidation-reduction process" evidence=IEA] [GO:0009941 "chloroplast envelope" evidence=IDA] [GO:0009507 "chloroplast" evidence=IDA] [GO:0009570 "chloroplast stroma" evidence=IDA] [GO:0005829 "cytosol" evidence=RCA] [GO:0009536 "plastid" evidence=ISS] InterPro:IPR001017 InterPro:IPR017597 Pfam:PF00676 EMBL:CP002684 GO:GO:0009570 GO:GO:0009941 GO:GO:0006096 GO:GO:0004739 eggNOG:COG1071 KO:K00161 TIGRFAMs:TIGR03182 HSSP:P08559 EMBL:U80185 EMBL:AY052721 EMBL:AY063724 EMBL:AK226909 IPI:IPI00525582 RefSeq:NP_171617.1 UniGene:At.20069 ProteinModelPortal:O24457 IntAct:O24457 STRING:O24457 PRIDE:O24457 ProMEX:O24457 EnsemblPlants:AT1G01090.1 GeneID:839429 KEGG:ath:AT1G01090 TAIR:At1g01090 InParanoid:O24457 OMA:SKEHHLL PhylomeDB:O24457 ProtClustDB:PLN02374 Genevestigator:O24457 Uniprot:O24457
Root	Isotig07790	2	21	-3.521	1.10E-05	TAIR|locus:2200980 - symbol:PDH-E1 ALPHA "AT1G01090" species:3702 "Arabidopsis thaliana" [GO:0004739 "pyruvate dehydrogenase (acetyl-transferring) activity" evidence=ISS] [GO:0006096 "glycolysis" evidence=IEA] [GO:0008152 "metabolic process" evidence=IEA;ISS] [GO:0055114 "oxidation-reduction process" evidence=IEA] [GO:0009941 "chloroplast envelope" evidence=IDA] [GO:0009507 "chloroplast" evidence=IDA] [GO:0009570 "chloroplast stroma" evidence=IDA] [GO:0005829 "cytosol" evidence=RCA] [GO:0009536 "plastid" evidence=ISS] InterPro:IPR001017 InterPro:IPR017597 Pfam:PF00676 EMBL:CP002684 GO:GO:0009570 GO:GO:0009941 GO:GO:0006096 GO:GO:0004739 eggNOG:COG1071 KO:K00161 TIGRFAMs:TIGR03182 HSSP:P08559 EMBL:U80185 EMBL:AY052721 EMBL:AY063724 EMBL:AK226909 IPI:IPI00525582 RefSeq:NP_171617.1 UniGene:At.20069 ProteinModelPortal:O24457 IntAct:O24457 STRING:O24457 PRIDE:O24457 ProMEX:O24457 EnsemblPlants:AT1G01090.1 GeneID:839429 KEGG:ath:AT1G01090 TAIR:At1g01090 InParanoid:O24457 OMA:SKEHHLL PhylomeDB:O24457 ProtClustDB:PLN02374 Genevestigator:O24457 Uniprot:O24457
Root	Isotig07811	1	28	-4.936	2.57E-08	TAIR|locus:2082837 - symbol:CYSC1 "AT3G61440" species:3702 "Arabidopsis thaliana" [GO:0004124 "cysteine synthase activity" evidence=ISS;IDA;IMP] [GO:0019344 "cysteine biosynthetic process" evidence=ISS;IDA] [GO:0019499 "cyanide metabolic process" evidence=TAS] [GO:0050017 "L-3-cyanoalanine synthase activity" evidence=IDA] [GO:0051410 "detoxification of nitrogen compound" evidence=TAS] [GO:0005739 "mitochondrion" evidence=IDA] [GO:0009507 "chloroplast" evidence=IDA] [GO:0005507 "copper ion binding" evidence=IDA] [GO:0019500 "cyanide catabolic process" evidence=IMP] [GO:0080147 "root hair cell development" evidence=IMP] InterPro:IPR001216 InterPro:IPR001926 InterPro:IPR005856 InterPro:IPR005859 Pfam:PF00291 PROSITE:PS00901 GO:GO:0005739 GO:GO:0009507 EMBL:CP002686 GenomeReviews:BA000014_GR GO:GO:0030170 EMBL:AL132962 SUPFAM:SSF53686 GO:GO:0016740 GO:GO:0005507 GO:GO:0080147 HOGENOM:HBG748215 GO:GO:0006535 GO:GO:0004124 TIGRFAMs:TIGR01139 TIGRFAMs:TIGR01136 GO:GO:0051410 HSSP:Q9WZD3 EMBL:AY093094 EMBL:AY087208 EMBL:AY128782 EMBL:AB024282 EMBL:AK226606 EMBL:AJ010505 IPI:IPI00534676 PIR:T47936 RefSeq:NP_191703.1 UniGene:At.21833 ProteinModelPortal:Q9S757 SMR:Q9S757 IntAct:Q9S757 STRING:Q9S757 PRIDE:Q9S757 ProMEX:Q9S757 EnsemblPlants:AT3G61440.1 GeneID:825317 KEGG:ath:AT3G61440 TAIR:At3g61440 InParanoid:Q9S757 KO:K13034 OMA:MERDGKG PhylomeDB:Q9S757 ProtClustDB:PLN02556 BRENDA:4.4.1.9 ArrayExpress:Q9S757 Genevestigator:Q9S757 GO:GO:0050017 GO:GO:0019500 Uniprot:Q9S757
Root	Isotig07812	1	37	-5.338	1.07E-10	TAIR|locus:2082837 - symbol:CYSC1 "AT3G61440" species:3702 "Arabidopsis thaliana" [GO:0004124 "cysteine synthase activity" evidence=ISS;IDA;IMP] [GO:0019344 "cysteine biosynthetic process" evidence=ISS;IDA] [GO:0019499 "cyanide metabolic process" evidence=TAS] [GO:0050017 "L-3-cyanoalanine synthase activity" evidence=IDA] [GO:0051410 "detoxification of nitrogen compound" evidence=TAS] [GO:0005739 "mitochondrion" evidence=IDA] [GO:0009507 "chloroplast" evidence=IDA] [GO:0005507 "copper ion binding" evidence=IDA] [GO:0019500 "cyanide catabolic process" evidence=IMP] [GO:0080147 "root hair cell development" evidence=IMP] InterPro:IPR001216 InterPro:IPR001926 InterPro:IPR005856 InterPro:IPR005859 Pfam:PF00291 PROSITE:PS00901 GO:GO:0005739 GO:GO:0009507 EMBL:CP002686 GenomeReviews:BA000014_GR GO:GO:0030170 EMBL:AL132962 SUPFAM:SSF53686 GO:GO:0016740 GO:GO:0005507 GO:GO:0080147 HOGENOM:HBG748215 GO:GO:0006535 GO:GO:0004124 TIGRFAMs:TIGR01139 TIGRFAMs:TIGR01136

Tissue	Identification	# of reads (stress)	# of reads (control)	Fold Change	p-value	Description
						GO:GO:0051410 HSSP:Q9WZD3 EMBL:AY093094 EMBL:AY087208 EMBL:AY128782 EMBL:AB024282 EMBL:AK226606 EMBL:AJ010505 IPI:IPI00534676 PIR:T47936 RefSeq:NP_191703.1 UniGene:At.21833 ProteinModelPortal:Q9S757 SMR:Q9S757 IntAct:Q9S757 STRING:Q9S757 PRIDE:Q9S757 ProMEX:Q9S757 EnsemblPlants:AT3G61440.1 GeneID:825317 KEGG:ath:AT3G61440 TAIR:At3g61440 InParanoid:Q9S757 KO:K13034 OMA:MERDGKG PhylomeDB:Q9S757 ProtClustDB:PLN02556 BRENDA:4.4.1.9 ArrayExpress:Q9S757 Genevestigator:Q9S757 GO:GO:0050017 GO:GO:0019500 Uniprot:Q9S757
Root	Isotig07813	1	18	-4.299	1.41E-05	TAIR|locus:2115065 - symbol:PLP4 "AT4G37050" species:3702 "Arabidopsis thaliana" [GO:0005575 "cellular_component" evidence=ND] [GO:0045735 "nutrient reservoir activity" evidence=ISS] [GO:0004620 "phospholipase activity" evidence=IDA] [GO:0009737 "response to abscisic acid stimulus" evidence=IDA] InterPro:IPR002641 InterPro:IPR016035 Pfam:PF01734 GO:GO:0009737 EMBL:CP002687 GenomeReviews:CT486007_GR GO:GO:0006629 EMBL:Z99707 EMBL:AL161590 UniGene:At.4646 SUPFAM:SSF52151 GO:GO:0004620 eggNOG:COG3621 HSSP:Q8LPW4 HOGENOM:HBG581721 IPI:IPI00546583 PIR:F85437 RefSeq:NP_195422.3 UniGene:At.48948 ProteinModelPortal:O23181 SMR:O23181 PRIDE:O23181 EnsemblPlants:AT4G37050.1 GeneID:829859 KEGG:ath:AT4G37050 TAIR:At4g37050 InParanoid:O23181 PhylomeDB:O23181 ProtClustDB:CLSN2914016 Genevestigator:O23181 Uniprot:O23181
Root	Isotig07814	1	18	-4.299	1.41E-05	TAIR|locus:2115065 - symbol:PLP4 "AT4G37050" species:3702 "Arabidopsis thaliana" [GO:0005575 "cellular_component" evidence=ND] [GO:0045735 "nutrient reservoir activity" evidence=ISS] [GO:0004620 "phospholipase activity" evidence=IDA] [GO:0009737 "response to abscisic acid stimulus" evidence=IDA] InterPro:IPR002641 InterPro:IPR016035 Pfam:PF01734 GO:GO:0009737 EMBL:CP002687 GenomeReviews:CT486007_GR GO:GO:0006629 EMBL:Z99707 EMBL:AL161590 UniGene:At.4646 SUPFAM:SSF52151 GO:GO:0004620 eggNOG:COG3621 HSSP:Q8LPW4 HOGENOM:HBG581721 IPI:IPI00546583 PIR:F85437 RefSeq:NP_195422.3 UniGene:At.48948 ProteinModelPortal:O23181 SMR:O23181 PRIDE:O23181 EnsemblPlants:AT4G37050.1 GeneID:829859 KEGG:ath:AT4G37050 TAIR:At4g37050 InParanoid:O23181 PhylomeDB:O23181 ProtClustDB:CLSN2914016 Genevestigator:O23181 Uniprot:O23181
Root	Isotig07827	6	25	-2.188	0.000173532	TAIR|locus:2135932 - symbol:VEP1 "AT4G24220" species:3702 "Arabidopsis thaliana" [GO:0009611 "response to wounding" evidence=IEP] [GO:0010051 "xylem and phloem pattern formation" evidence=IMP] [GO:0008202 "steroid metabolic process" evidence=IDA] [GO:0035671 "enone reductase activity" evidence=IDA] [GO:0005829 "cytosol" evidence=IDA] InterPro:IPR016040 GO:GO:0005829 EMBL:CP002687 GenomeReviews:CT486007_GR GO:GO:0009611 GO:GO:0000166 Gene3D:G3DSA:3.40.50.720 GO:GO:0008202 EMBL:AL078637 EMBL:AL161561 UniGene:At.32364 UniGene:At.71005 GO:GO:0010051 GO:GO:0035671 HOGENOM:HBG353845 ProtClustDB:CLSN2685402 EMBL:AY062451 EMBL:AY087323 EMBL:BT008479 EMBL:EF579963 IPI:IPI00524911 PIR:T09885 RefSeq:NP_194153.1 ProteinModelPortal:Q9STX2 SMR:Q9STX2 PRIDE:Q9STX2 EnsemblPlants:AT4G24220.1 GeneID:828523 KEGG:ath:AT4G24220 TAIR:At4g24220 InParanoid:Q9STX2 OMA:GLKHYLG PhylomeDB:Q9STX2 ArrayExpress:Q9STX2 Genevestigator:Q9STX2 Uniprot:Q9STX2
Root	Isotig07828	6	25	-2.188	0.000173532	TAIR|locus:2135932 - symbol:VEP1 "AT4G24220" species:3702 "Arabidopsis thaliana" [GO:0009611 "response to wounding" evidence=IEP] [GO:0010051 "xylem and phloem pattern formation" evidence=IMP] [GO:0008202 "steroid metabolic process" evidence=IDA] [GO:0035671 "enone reductase activity" evidence=IDA] [GO:0005829 "cytosol" evidence=IDA] InterPro:IPR016040 GO:GO:0005829 EMBL:CP002687 GenomeReviews:CT486007_GR GO:GO:0009611 GO:GO:0000166 Gene3D:G3DSA:3.40.50.720 GO:GO:0008202 EMBL:AL078637 EMBL:AL161561 UniGene:At.32364 UniGene:At.71005 GO:GO:0010051 GO:GO:0035671 HOGENOM:HBG353845 ProtClustDB:CLSN2685402 EMBL:AY062451 EMBL:AY087323 EMBL:BT008479 EMBL:EF579963 IPI:IPI00524911 PIR:T09885 RefSeq:NP_194153.1 ProteinModelPortal:Q9STX2 SMR:Q9STX2 PRIDE:Q9STX2 EnsemblPlants:AT4G24220.1 GeneID:828523 KEGG:ath:AT4G24220 TAIR:At4g24220 InParanoid:Q9STX2 OMA:GLKHYLG PhylomeDB:Q9STX2 ArrayExpress:Q9STX2 Genevestigator:Q9STX2 Uniprot:Q9STX2
Root	Isotig07831	3	20	-2.866	9.17E-05	TAIR|locus:2167659 - symbol:ERF9 "AT5G44210" species:3702 "Arabidopsis thaliana" [GO:0003700 "sequence-specific DNA binding transcription factor activity" evidence=ISS;TAS] [GO:0003677 "DNA binding" evidence=TAS] [GO:0045892 "negative regulation of transcription, DNA-dependent" evidence=TAS] [GO:0016020 "membrane" evidence=IDA] [GO:0009873 "ethylene mediated signaling pathway" evidence=TAS] InterPro:IPR001471 InterPro:IPR016177 Pfam:PF00847 PRINTS:PR00367 PROSITE:PS51032 SMART:SM00380 GO:GO:0005634 EMBL:CP002688 GenomeReviews:BA000015_GR GO:GO:0006952 GO:GO:0009873 GO:GO:0016020 GO:GO:0003677 GO:GO:0003700 GO:GO:0006351 EMBL:AB005239 KO:K09286 Gene3D:G3DSA:3.30.730.10 SUPFAM:SSF54171 HOGENOM:HBG744062 EMBL:AB047648 EMBL:AY560856 EMBL:BT015876

Tissue	Identification	# of reads (stress)	# of reads (control)	Fold Change	p-value	Description
						EMBL:BT020188 IPI:IPI00535860 RefSeq:NP_199234.1 UniGene:At.30087 ProteinModelPortal:Q9FE67 SMR:Q9FE67 IntAct:Q9FE67 STRING:Q9FE67 EnsemblPlants:AT5G44210.1 GeneID:834444 KEGG:ath:AT5G44210 TAIR:At5g44210 eggNOG:NOG249494 InParanoid:Q9FE67 OMA:FACHRER PhylomeDB:Q9FE67 ProtClustDB:CLSN2916712 ArrayExpress:Q9FE67 Genevestigator:Q9FE67 GermOnline:AT5G44210 Uniprot:Q9FE67
Root	Isotig07832	3	18	-2.714	0.000308932	TAIR|locus:2167659 - symbol:ERF9 "AT5G44210" species:3702 "Arabidopsis thaliana" [GO:0003700 "sequence-specific DNA binding transcription factor activity" evidence=ISS;TAS] [GO:0003677 "DNA binding" evidence=TAS] [GO:0045892 "negative regulation of transcription, DNA-dependent" evidence=TAS] [GO:0016020 "membrane" evidence=IDA] [GO:0009873 "ethylene mediated signaling pathway" evidence=TAS] InterPro:IPR001471 InterPro:IPR016177 Pfam:PF00847 PRINTS:PR00367 PROSITE:PS51032 SMART:SM00380 GO:GO:0005634 EMBL:CP002688 GenomeReviews:BA000015_GR GO:GO:0006952 GO:GO:0009873 GO:GO:0016020 GO:GO:0003677 GO:GO:0003700 GO:GO:0006351 EMBL:AB005239 KO:K09286 Gene3D:G3DSA:3.30.730.10 SUPFAM:SSF54171 HOGENOM:HBG744062 EMBL:AB047648 EMBL:AY560856 EMBL:BT015876 EMBL:BT020188 IPI:IPI00535860 RefSeq:NP_199234.1 UniGene:At.30087 ProteinModelPortal:Q9FE67 SMR:Q9FE67 IntAct:Q9FE67 STRING:Q9FE67 EnsemblPlants:AT5G44210.1 GeneID:834444 KEGG:ath:AT5G44210 TAIR:At5g44210 eggNOG:NOG249494 InParanoid:Q9FE67 OMA:FACHRER PhylomeDB:Q9FE67 ProtClustDB:CLSN2916712 ArrayExpress:Q9FE67 Genevestigator:Q9FE67 GermOnline:AT5G44210 Uniprot:Q9FE67
Root	Isotig07859	6	24	-2.129	0.00029855	TAIR|locus:2134971 - symbol:AT4G24050 species:3702 "Arabidopsis thaliana" [GO:0003824 "catalytic activity" evidence=IEA] [GO:0008152 "metabolic process" evidence=IEA] [GO:0016491 "oxidoreductase activity" evidence=IEA;ISS] [GO:0055114 "oxidation-reduction process" evidence=IEA] InterPro:IPR002198 InterPro:IPR016040 Pfam:PF00106 InterPro:IPR002347 EMBL:CP002687 GenomeReviews:CT486007_GR GO:GO:0000166 Gene3D:G3DSA:3.40.50.720 GO:GO:0016491 eggNOG:COG1028 HOGENOM:HBG750976 PRINTS:PR00081 EMBL:AC002343 EMBL:AL161560 EMBL:AL109619 OMA:TELARNW EMBL:AF439829 EMBL:AY125561 IPI:IPI00517204 PIR:T13447 RefSeq:NP_194136.1 UniGene:At.3661 ProteinModelPortal:O22985 IntAct:O22985 PRIDE:O22985 ProMEX:O22985 EnsemblPlants:AT4G24050.1 GeneID:828505 KEGG:ath:AT4G24050 TAIR:At4g24050 InParanoid:O22985 PhylomeDB:O22985 ProtClustDB:CLSN2679319 ArrayExpress:O22985 Genevestigator:O22985 Uniprot:O22985
Root	Isotig07861	0	34	-6.216	6.02E-10	TAIR|locus:2119642 - symbol:SPHK1 "AT4G21540" species:3702 "Arabidopsis thaliana" [GO:0004143 "diacylglycerol kinase activity" evidence=ISS] [GO:0007205 "activation of protein kinase C activity by G-protein coupled receptor protein signaling pathway" evidence=ISS] [GO:0005773 "vacuole" evidence=IDA] [GO:0017050 "D-erythro-sphingosine kinase activity" evidence=IDA;TAS] [GO:0008481 "sphinganine kinase activity" evidence=IDA] [GO:0009737 "response to abscisic acid stimulus" evidence=IMP] [GO:0009845 "seed germination" evidence=IMP] [GO:0009705 "plant-type vacuole membrane" evidence=IDA] InterPro:IPR001206 Pfam:PF00781 PROSITE:PS50146 SMART:SM00046 GO:GO:0009737 GO:GO:0007205 GO:GO:0009845 GO:GO:0009705 eggNOG:COG1597 GO:GO:0004143 EMBL:AL161555 EMBL:AL022603 GO:GO:0017050 GO:GO:0008481 IPI:IPI01020462 PIR:T05162 ProteinModelPortal:O65419 STRING:O65419 InParanoid:O65419 PhylomeDB:O65419 Genevestigator:O65419 Uniprot:O65419
Root	Isotig07862	0	34	-6.216	6.02E-10	TAIR|locus:2119642 - symbol:SPHK1 "AT4G21540" species:3702 "Arabidopsis thaliana" [GO:0004143 "diacylglycerol kinase activity" evidence=ISS] [GO:0007205 "activation of protein kinase C activity by G-protein coupled receptor protein signaling pathway" evidence=ISS] [GO:0005773 "vacuole" evidence=IDA] [GO:0017050 "D-erythro-sphingosine kinase activity" evidence=IDA;TAS] [GO:0008481 "sphinganine kinase activity" evidence=IDA] [GO:0009737 "response to abscisic acid stimulus" evidence=IMP] [GO:0009845 "seed germination" evidence=IMP] [GO:0009705 "plant-type vacuole membrane" evidence=IDA] InterPro:IPR001206 Pfam:PF00781 PROSITE:PS50146 SMART:SM00046 GO:GO:0009737 GO:GO:0007205 GO:GO:0009845 GO:GO:0009705 eggNOG:COG1597 GO:GO:0004143 EMBL:AL161555 EMBL:AL022603 GO:GO:0017050 GO:GO:0008481 IPI:IPI01020462 PIR:T05162 ProteinModelPortal:O65419 STRING:O65419 InParanoid:O65419 PhylomeDB:O65419 Genevestigator:O65419 Uniprot:O65419
Root	Isotig07871	0	18	-5.299	6.77E-06	TAIR|locus:2183730 - symbol:AT5G10770 "AT5G10770" species:3702 "Arabidopsis thaliana" [GO:0003677 "DNA binding" evidence=ISS] [GO:0004190 "aspartic-type endopeptidase activity" evidence=IEA] [GO:0006508 "proteolysis" evidence=IEA] InterPro:IPR001461 InterPro:IPR009007 Pfam:PF00026 PRINTS:PR00792 EMBL:CP002688 GO:GO:0006508 GO:GO:0004190 InterPro:IPR021109 SUPFAM:SSF50630

Tissue	Identification	# of reads (stress)	# of reads (control)	Fold Change	p-value	Description
						Gene3D:G3DSA:2.40.70.10 PANTHER:PTHR13683 HSSP:P00799 EMBL:AY075656 EMBL:BT001004 IPI:IPI00543783 RefSeq:NP_196638.2 UniGene:At.32336 ProteinModelPortal:Q8S9J6 MEROPS:A01.A15 PRIDE:Q8S9J6 EnsemblPlants:AT5G10770.1 GeneID:830944 KEGG:ath:AT5G10770 TAIR:At5g10770 InParanoid:Q8S9J6 OMA:LEVVYDG PhylomeDB:Q8S9J6 ProtClustDB:CLSN2686424 Genevestigator:Q8S9J6 Uniprot:Q8S9J6
Root	Isotig07881	0	18	-5.299	6.77E-06	UNIPROTKB|B2HIN2 - symbol:fadD26 "Long-chain-fatty-acid--AMP ligase FadD26" species:216594 "Mycobacterium marinum M" [GO:0008610 "lipid biosynthetic process" evidence=ISS] InterPro:IPR000873 Pfam:PF00501 eggNOG:COG0318 HOGENOM:HBG547964 GO:GO:0016874 GO:GO:0008610 GO:GO:0006631 KO:K12426 OMA:PARWIQQ ProtClustDB:PRK05850 EMBL:CP000854 RefSeq:YP_001850081.1 ProteinModelPortal:B2HIN2 STRING:B2HIN2 EnsemblBacteria:EBMYCT00000032275 GeneID:6226036 GenomeReviews:CP000854_GR KEGG:mmi:MMAR_1777 PATRIC:18064429 Uniprot:B2HIN2
Root	Isotig07883	2	52	-4.829	3.94E-14	TAIR|locus:2130329 - symbol:AT4G16260 species:3702 "Arabidopsis thaliana" [GO:0003824 "catalytic activity" evidence=IEA] [GO:0004553 "hydrolase activity, hydrolyzing O-glycosyl compounds" evidence=IEA;ISS] [GO:0043169 "cation binding" evidence=IEA] [GO:0005618 "cell wall" evidence=IDA] [GO:0005774 "vacuolar membrane" evidence=IDA] [GO:0009651 "response to salt stress" evidence=IEP] [GO:0009817 "defense response to fungus, incompatible interaction" evidence=IDA] InterPro:IPR000490 InterPro:IPR013781 Pfam:PF00332 PROSITE:PS00587 EMBL:CP002687 GenomeReviews:CT486007_GR GO:GO:0005774 GO:GO:0005618 GO:GO:0043169 InterPro:IPR017853 Gene3D:G3DSA:3.20.20.80 SUPFAM:SSF51445 GO:GO:0005975 GO:GO:0004553 GO:GO:0009651 GO:GO:0009817 CAZy:GH17 HSSP:P15737 EMBL:AY064130 EMBL:AY143867 IPI:IPI00944240 RefSeq:NP_193361.4 UniGene:At.22570 ProteinModelPortal:Q8VZJ2 SMR:Q8VZJ2 PRIDE:Q8VZJ2 EnsemblPlants:AT4G16260.1 GeneID:827320 KEGG:ath:AT4G16260 TAIR:At4g16260 PhylomeDB:Q8VZJ2 ProtClustDB:CLSN2927419 Genevestigator:Q8VZJ2 Uniprot:Q8VZJ2
Root	Isotig07884	1	44	-5.588	1.69E-12	TAIR|locus:2130329 - symbol:AT4G16260 species:3702 "Arabidopsis thaliana" [GO:0003824 "catalytic activity" evidence=IEA] [GO:0004553 "hydrolase activity, hydrolyzing O-glycosyl compounds" evidence=IEA;ISS] [GO:0043169 "cation binding" evidence=IEA] [GO:0005618 "cell wall" evidence=IDA] [GO:0005774 "vacuolar membrane" evidence=IDA] [GO:0009651 "response to salt stress" evidence=IEP] [GO:0009817 "defense response to fungus, incompatible interaction" evidence=IDA] InterPro:IPR000490 InterPro:IPR013781 Pfam:PF00332 PROSITE:PS00587 EMBL:CP002687 GenomeReviews:CT486007_GR GO:GO:0005774 GO:GO:0005618 GO:GO:0043169 InterPro:IPR017853 Gene3D:G3DSA:3.20.20.80 SUPFAM:SSF51445 GO:GO:0005975 GO:GO:0004553 GO:GO:0009651 GO:GO:0009817 CAZy:GH17 HSSP:P15737 EMBL:AY064130 EMBL:AY143867 IPI:IPI00944240 RefSeq:NP_193361.4 UniGene:At.22570 ProteinModelPortal:Q8VZJ2 SMR:Q8VZJ2 PRIDE:Q8VZJ2 EnsemblPlants:AT4G16260.1 GeneID:827320 KEGG:ath:AT4G16260 TAIR:At4g16260 PhylomeDB:Q8VZJ2 ProtClustDB:CLSN2927419 Genevestigator:Q8VZJ2 Uniprot:Q8VZJ2
Root	Isotig07885	11	33	-1.714	0.000243809	TAIR|locus:2046485 - symbol:ATP3 "gamma subunit of Mt ATP synthase" species:3702 "Arabidopsis thaliana" [GO:0015986 "ATP synthesis coupled proton transport" evidence=IEA;ISS] [GO:0015992 "proton transport" evidence=ISS] [GO:0016020 "membrane" evidence=ISS] [GO:0005739 "mitochondrion" evidence=IDA] [GO:0009507 "chloroplast" evidence=IDA] [GO:0005730 "nucleolus" evidence=IDA] [GO:0005634 "nucleus" evidence=IDA] [GO:0005737 "cytoplasm" evidence=IDA] [GO:0005618 "cell wall" evidence=IDA] [GO:0005753 "mitochondrial proton-transporting ATP synthase complex" evidence=IDA] [GO:0008270 "zinc ion binding" evidence=IDA] [GO:0005794 "Golgi apparatus" evidence=RCA] InterPro:IPR000131 Pfam:PF00231 PRINTS:PR00126 GO:GO:0005618 GO:GO:0009507 EMBL:CP002685 GenomeReviews:CT485783_GR GO:GO:0005730 GO:GO:0008270 EMBL:AC003033 GO:GO:0046933 GO:GO:0046961 GO:GO:0015986 eggNOG:COG0224 HOGENOM:HBG586593 ProtClustDB:CLSN2679503 InterPro:IPR023632 InterPro:IPR023633 PANTHER:PTHR11693 SUPFAM:SSF52943 TIGRFAMs:TIGR01146 PROSITE:PS00153 EMBL:D88374 EMBL:AC002334 EMBL:AY039513 EMBL:AY062627 EMBL:AY102152 EMBL:AY086685 IPI:IPI00526529 PIR:F84740 PIR:T01103 RefSeq:NP_180863.1 UniGene:At.25073 ProteinModelPortal:Q96250 SMR:Q96250 IntAct:Q96250 STRING:Q96250 PRIDE:Q96250 EnsemblPlants:AT2G33040.1 GeneID:817866 KEGG:ath:AT2G33040 GeneFarm:3313 TAIR:At2g33040 InParanoid:Q96250 KO:K02136 OMA:SARRNAM PhylomeDB:Q96250 Genevestigator:Q96250 GermOnline:AT2G33040 Uniprot:Q96250
Root	Isotig07886	11	33	-1.714	0.000243809	TAIR|locus:2046485 - symbol:ATP3 "gamma subunit of Mt ATP synthase" species:3702 "Arabidopsis thaliana" [GO:0015986 "ATP synthesis coupled proton transport" evidence=IEA;ISS] [GO:0015992 "proton transport" evidence=ISS] [GO:0016020 "membrane" evidence=ISS] [GO:0005739 "mitochondrion" evidence=IDA]

Tissue	Identification	# of reads (stress)	# of reads (control)	Fold Change	p-value	Description
						[GO:0009507 "chloroplast" evidence=IDA] [GO:0005730 "nucleolus" evidence=IDA] [GO:0005634 "nucleus" evidence=IDA] [GO:0005737 "cytoplasm" evidence=IDA] [GO:0005618 "cell wall" evidence=IDA] [GO:0005753 "mitochondrial proton-transporting ATP synthase complex" evidence=IDA] [GO:0008270 "zinc ion binding" evidence=IDA] [GO:0005794 "Golgi apparatus" evidence=RCA] InterPro:IPR000131 Pfam:PF00231 PRINTS:PR00126 GO:GO:0005618 GO:GO:0009507 EMBL:CP002685 GenomeReviews:CT485783_GR GO:GO:0005730 GO:GO:0008270 EMBL:AC003033 GO:GO:0046933 GO:GO:0046961 GO:GO:0015986 eggNOG:COG0224 HOGENOM:HBG586593 ProtClustDB:CLSN2679503 InterPro:IPR023632 InterPro:IPR023633 PANTHER:PTHR11693 SUPFAM:SSF52943 TIGRFAMs:TIGR01146 PROSITE:PS00153 EMBL:D88374 EMBL:AC002334 EMBL:AY039513 EMBL:AY062627 EMBL:AY102152 EMBL:AY086685 IPI:IPI00526529 PIR:F84740 PIR:T01103 RefSeq:NP_180863.1 UniGene:At.25073 ProteinModelPortal:Q96250 SMR:Q96250 IntAct:Q96250 STRING:Q96250 PRIDE:Q96250 EnsemblPlants:AT2G33040.1 GeneID:817866 KEGG:ath:AT2G33040 GeneFarm:3313 TAIR:At2g33040 InParanoid:Q96250 KO:K02136 OMA:SARRNAM PhylomeDB:Q96250 Genevestigator:Q96250 GermOnline:AT2G33040 Uniprot:Q96250
Root	Isotig07893	0	21	-5.521	1.09E-06	RGD|727924 - symbol:LOC257642 "rRNA promoter binding protein" species:10116 "Rattus norvegicus" [GO:0003690 "double-stranded DNA binding" evidence=IDA] [GO:0003700 "sequence-specific DNA binding transcription factor activity" evidence=IDA] [GO:0008134 "transcription factor binding" evidence=IDA] [GO:0008283 "cell proliferation" evidence=IDA] [GO:0009303 "rRNA transcription" evidence=IDA] RGD:727924 GO:GO:0008134 GO:GO:0008283 GO:GO:0003700 GO:GO:0003690 GO:GO:0009303 EMBL:U77931 IPI:IPI00195015 RefSeq:NP_671477.1 GeneID:257642 KEGG:rno:257642 NextBio:624170 Genevestigator:Q99JC0 Uniprot:Q99JC0
Root	Isotig07894	0	16	-5.129	2.34E-05	RGD|727924 - symbol:LOC257642 "rRNA promoter binding protein" species:10116 "Rattus norvegicus" [GO:0003690 "double-stranded DNA binding" evidence=IDA] [GO:0003700 "sequence-specific DNA binding transcription factor activity" evidence=IDA] [GO:0008134 "transcription factor binding" evidence=IDA] [GO:0008283 "cell proliferation" evidence=IDA] [GO:0009303 "rRNA transcription" evidence=IDA] RGD:727924 GO:GO:0008134 GO:GO:0008283 GO:GO:0003700 GO:GO:0003690 GO:GO:0009303 EMBL:U77931 IPI:IPI00195015 RefSeq:NP_671477.1 GeneID:257642 KEGG:rno:257642 NextBio:624170 Genevestigator:Q99JC0 Uniprot:Q99JC0
Root	Isotig07897	0	37	-6.338	1.15E-10	TAIR|locus:2135212 - symbol:GGT1 "AT4G39640" species:3702 "Arabidopsis thaliana" [GO:0003840 "gamma-glutamyltransferase activity" evidence=ISS] [GO:0006751 "glutathione catabolic process" evidence=IDA] [GO:0006979 "response to oxidative stress" evidence=IDA] [GO:0016756 "glutathione gamma-glutamylcysteinyltransferase activity" evidence=IDA] [GO:0048046 "apoplast" evidence=IDA] [GO:0009505 "plant-type cell wall" evidence=IDA] [GO:0009506 "plasmodesma" evidence=IDA] InterPro:IPR000101 Pfam:PF01019 PRINTS:PR01210 GO:GO:0009506 EMBL:CP002687 GO:GO:0048046 GO:GO:0006979 GO:GO:0009505 GO:GO:0006751 KO:K00681 GO:GO:0003840 PANTHER:PTHR11686 TIGRFAMs:TIGR00066 GO:GO:0016756 ProtClustDB:PLN02198 EMBL:AY069872 EMBL:BT000495 IPI:IPI00546319 RefSeq:NP_195674.2 RefSeq:NP_974717.1 UniGene:At.21562 ProteinModelPortal:Q8VYW6 SMR:Q8VYW6 STRING:Q8VYW6 MEROPS:T03.008 PRIDE:Q8VYW6 EnsemblPlants:AT4G39640.1 EnsemblPlants:AT4G39640.2 GeneID:830118 KEGG:ath:AT4G39640 TAIR:At4g39640 InParanoid:Q8VYW6 OMA:ANTYQTI PhylomeDB:Q8VYW6 Genevestigator:Q8VYW6 Uniprot:Q8VYW6
Root	Isotig07898	0	21	-5.521	1.09E-06	TAIR|locus:2135212 - symbol:GGT1 "AT4G39640" species:3702 "Arabidopsis thaliana" [GO:0003840 "gamma-glutamyltransferase activity" evidence=ISS] [GO:0006751 "glutathione catabolic process" evidence=IDA] [GO:0006979 "response to oxidative stress" evidence=IDA] [GO:0016756 "glutathione gamma-glutamylcysteinyltransferase activity" evidence=IDA] [GO:0048046 "apoplast" evidence=IDA] [GO:0009505 "plant-type cell wall" evidence=IDA] [GO:0009506 "plasmodesma" evidence=IDA] InterPro:IPR000101 Pfam:PF01019 PRINTS:PR01210 GO:GO:0009506 EMBL:CP002687 GO:GO:0048046 GO:GO:0006979 GO:GO:0009505 GO:GO:0006751 KO:K00681 GO:GO:0003840 PANTHER:PTHR11686 TIGRFAMs:TIGR00066 GO:GO:0016756 ProtClustDB:PLN02198 EMBL:AY069872 EMBL:BT000495 IPI:IPI00546319 RefSeq:NP_195674.2 RefSeq:NP_974717.1 UniGene:At.21562 ProteinModelPortal:Q8VYW6 SMR:Q8VYW6 STRING:Q8VYW6 MEROPS:T03.008 PRIDE:Q8VYW6 EnsemblPlants:AT4G39640.1 EnsemblPlants:AT4G39640.2 GeneID:830118 KEGG:ath:AT4G39640 TAIR:At4g39640 InParanoid:Q8VYW6 OMA:ANTYQTI PhylomeDB:Q8VYW6 Genevestigator:Q8VYW6 Uniprot:Q8VYW6
Root	Isotig07923	2	29	-3.987	6.83E-08	TAIR|locus:2151739 - symbol:GSR 1 "glutamine synthase clone R1" species:3702 "Arabidopsis thaliana" [GO:0004356 "glutamate-ammonia ligase activity" evidence=ISS;IDA;IPI] [GO:0005829 "cytosol" evidence=TAS] [GO:0022626 "cytosolic ribosome" evidence=IDA] [GO:0005618 "cell wall" evidence=IDA] [GO:0042128 "nitrate

Tissue	Identification	# of reads (stress)	# of reads (control)	Fold Change	p-value	Description
						assimilation" evidence=TAS] [GO:0005515 "protein binding" evidence=IPI] [GO:0005507 "copper ion binding" evidence=IDA] [GO:0005886 "plasma membrane" evidence=IDA] [GO:0009506 "plasmodesma" evidence=IDA] InterPro:IPR008146 InterPro:IPR008147 InterPro:IPR014746 Pfam:PF00120 Pfam:PF03951 PROSITE:PS00180 PROSITE:PS00181 GO:GO:0005886 GO:GO:0009506 GO:GO:0005524 EMBL:CP002688 GenomeReviews:BA000015_GR GO:GO:0005618 GO:GO:0005515 GO:GO:0005507 GO:GO:0022626 GO:GO:0042128 GO:GO:0009399 EMBL:AB018107 EMBL:AF419608 EMBL:AF428386 EMBL:AY079113 EMBL:BT000753 EMBL:AK222005 IPI:IPI00519748 PIR:S18601 RefSeq:NP_198576.1 UniGene:At.7003 ProteinModelPortal:Q56WN1 SMR:Q56WN1 IntAct:Q56WN1 STRING:Q56WN1 PRIDE:Q56WN1 EnsemblPlants:AT5G37600.1 GeneID:833738 KEGG:ath:AT5G37600 TAIR:At5g37600 eggNOG:COG0174 HOGENOM:HBG299709 InParanoid:Q56WN1 KO:K01915 OMA:DPYRVTS PhylomeDB:Q56WN1 ProtClustDB:PLN02284 BRENDA:6.3.1.2 ArrayExpress:Q9FHR0 Genevestigator:Q56WN1 GermOnline:AT5G37600 GO:GO:0004356 GO:GO:0006542 Gene3D:G3DSA:3.30.590.10 Gene3D:G3DSA:3.10.20.70 SUPFAM:SSF54368 Uniprot:Q56WN1
Root	Isotig07924	2	29	-3.987	6.83E-08	TAIR|locus:2151739 - symbol:GSR 1 "glutamine synthase clone R1" species:3702 "Arabidopsis thaliana" [GO:0004356 "glutamate-ammonia ligase activity" evidence=ISS;IDA;IPI] [GO:0005829 "cytosol" evidence=TAS] [GO:0022626 "cytosolic ribosome" evidence=IDA] [GO:0005618 "cell wall" evidence=IDA] [GO:0042128 "nitrate assimilation" evidence=TAS] [GO:0005515 "protein binding" evidence=IPI] [GO:0005507 "copper ion binding" evidence=IDA] [GO:0005886 "plasma membrane" evidence=IDA] [GO:0009506 "plasmodesma" evidence=IDA] InterPro:IPR008146 InterPro:IPR008147 InterPro:IPR014746 Pfam:PF00120 Pfam:PF03951 PROSITE:PS00180 PROSITE:PS00181 GO:GO:0005886 GO:GO:0009506 GO:GO:0005524 EMBL:CP002688 GenomeReviews:BA000015_GR GO:GO:0005618 GO:GO:0005515 GO:GO:0005507 GO:GO:0022626 GO:GO:0042128 GO:GO:0009399 EMBL:AB018107 EMBL:AF419608 EMBL:AF428386 EMBL:AY079113 EMBL:BT000753 EMBL:AK222005 IPI:IPI00519748 PIR:S18601 RefSeq:NP_198576.1 UniGene:At.7003 ProteinModelPortal:Q56WN1 SMR:Q56WN1 IntAct:Q56WN1 STRING:Q56WN1 PRIDE:Q56WN1 EnsemblPlants:AT5G37600.1 GeneID:833738 KEGG:ath:AT5G37600 TAIR:At5g37600 eggNOG:COG0174 HOGENOM:HBG299709 InParanoid:Q56WN1 KO:K01915 OMA:DPYRVTS PhylomeDB:Q56WN1 ProtClustDB:PLN02284 BRENDA:6.3.1.2 ArrayExpress:Q9FHR0 Genevestigator:Q56WN1 GermOnline:AT5G37600 GO:GO:0004356 GO:GO:0006542 Gene3D:G3DSA:3.30.590.10 Gene3D:G3DSA:3.10.20.70 SUPFAM:SSF54368 Uniprot:Q56WN1
Root	Isotig07925	0	42	-6.521	7.61E-12	TAIR|locus:2101135 - symbol:BT2 "AT3G48360" species:3702 "Arabidopsis thaliana" [GO:0006355 "regulation of transcription, DNA-dependent" evidence=TAS] [GO:0005515 "protein binding" evidence=IPI] [GO:0005516 "calmodulin binding" evidence=IDA] [GO:0009611 "response to wounding" evidence=IEP] [GO:0009651 "response to salt stress" evidence=IEP] [GO:0009737 "response to abscisic acid stimulus" evidence=IEP] [GO:0009751 "response to salicylic acid stimulus" evidence=IEP] [GO:0042542 "response to hydrogen peroxide" evidence=IEP] [GO:0009734 "auxin mediated signaling pathway" evidence=IMP] [GO:0051973 "positive regulation of telomerase activity" evidence=IGI;IPI] [GO:0005634 "nucleus" evidence=IDA] [GO:0005737 "cytoplasm" evidence=IDA] [GO:0009553 "embryo sac development" evidence=IGI] [GO:0009555 "pollen development" evidence=IGI] [GO:0009733 "response to auxin stimulus" evidence=IEP] [GO:0007623 "circadian rhythm" evidence=IEP] [GO:0009409 "response to cold" evidence=IEP] [GO:0009738 "abscisic acid mediated signaling pathway" evidence=IMP] [GO:0009743 "response to carbohydrate stimulus" evidence=IEP] [GO:0009753 "response to jasmonic acid stimulus" evidence=IEP] [GO:0010167 "response to nitrate" evidence=IEP] [GO:0010182 "sugar mediated signaling pathway" evidence=IMP] [GO:0080134 "regulation of response to stress" evidence=IMP] InterPro:IPR000197 InterPro:IPR000210 InterPro:IPR013069 Pfam:PF00651 Pfam:PF02135 PROSITE:PS50097 PROSITE:PS50134 SMART:SM00225 GO:GO:0005634 GO:GO:0005737 EMBL:CP002686 GenomeReviews:BA000014_GR GO:GO:0009753 GO:GO:0009611 GO:GO:0009738 GO:GO:0009734 GO:GO:0006355 GO:GO:0007623 GO:GO:0008270 GO:GO:0009409 GO:GO:0009651 GO:GO:0005516 GO:GO:0009555 GO:GO:0009751 GO:GO:0010182 InterPro:IPR011333 Gene3D:G3DSA:3.30.710.10 SUPFAM:SSF54695 GO:GO:0051973 GO:GO:0042542 GO:GO:0009553 GO:GO:0003712 GO:GO:0010167 KO:K00517 eggNOG:NOG287613 ProtClustDB:CLSN2687065 GO:GO:0004402 Gene3D:G3DSA:1.20.1020.10 SUPFAM:SSF57933 EMBL:AY316675 EMBL:AL049659 EMBL:AY040015 EMBL:AY079408 EMBL:BT000678 EMBL:AK226596 IPI:IPI00536189 PIR:T06706 RefSeq:NP_566902.1 UniGene:At.21922 UniGene:At.67255 ProteinModelPortal:Q94BN0 SMR:Q94BN0 IntAct:Q94BN0 STRING:Q94BN0 PRIDE:Q94BN0

Tissue	Identification	# of reads (stress)	# of reads (control)	Fold Change	p-value	Description
						EnsemblPlants:AT3G48360.1 GeneID:823994 KEGG:ath:AT3G48360 TAIR:At3g48360 InParanoid:Q94BN0 OMA:RMWQLLR PhylomeDB:Q94BN0 Genevestigator:Q94BN0 GO:GO:0080134 Uniprot:Q94BN0
Root	Isotig07926	0	39	-6.414	3.85E-11	TAIR|locus:2101135 - symbol:BT2 "AT3G48360" species:3702 "Arabidopsis thaliana" [GO:0006355 "regulation of transcription, DNA-dependent" evidence=TAS] [GO:0005515 "protein binding" evidence=IPI] [GO:0005516 "calmodulin binding" evidence=IDA] [GO:0009611 "response to wounding" evidence=IEP] [GO:0009651 "response to salt stress" evidence=IEP] [GO:0009737 "response to abscisic acid stimulus" evidence=IEP] [GO:0009751 "response to salicylic acid stimulus" evidence=IEP] [GO:0042542 "response to hydrogen peroxide" evidence=IEP] [GO:0009734 "auxin mediated signaling pathway" evidence=IMP] [GO:0051973 "positive regulation of telomerase activity" evidence=IGI;IPI] [GO:0005634 "nucleus" evidence=IDA] [GO:0005737 "cytoplasm" evidence=IDA] [GO:0009553 "embryo sac development" evidence=IGI] [GO:0009555 "pollen development" evidence=IGI] [GO:0009733 "response to auxin stimulus" evidence=IEP] [GO:0007623 "circadian rhythm" evidence=IEP] [GO:0009409 "response to cold" evidence=IEP] [GO:0009738 "abscisic acid mediated signaling pathway" evidence=IMP] [GO:0009743 "response to carbohydrate stimulus" evidence=IEP] [GO:0009753 "response to jasmonic acid stimulus" evidence=IEP] [GO:0010167 "response to nitrate" evidence=IEP] [GO:0010182 "sugar mediated signaling pathway" evidence=IMP] [GO:0080134 "regulation of response to stress" evidence=IMP] InterPro:IPR000197 InterPro:IPR000210 InterPro:IPR013069 Pfam:PF00651 Pfam:PF02135 PROSITE:PS50097 PROSITE:PS50134 SMART:SM00225 GO:GO:0005634 GO:GO:0005737 EMBL:CP002686 GenomeReviews:BA000014_GR GO:GO:0009753 GO:GO:0009611 GO:GO:0009738 GO:GO:0009734 GO:GO:0006355 GO:GO:0007623 GO:GO:0008270 GO:GO:0009409 GO:GO:0009651 GO:GO:0005516 GO:GO:0009555 GO:GO:0009751 GO:GO:0010182 InterPro:IPR011333 Gene3D:G3DSA:3.30.710.10 SUPFAM:SSF54695 GO:GO:0051973 GO:GO:0042542 GO:GO:0009553 GO:GO:0003712 GO:GO:0010167 KO:K00517 eggNOG:NOG287613 ProtClustDB:CLSN2687065 GO:GO:0004402 Gene3D:G3DSA:1.20.1020.10 SUPFAM:SSF57933 EMBL:AY316675 EMBL:AL049659 EMBL:AY040015 EMBL:AY079408 EMBL:BT000678 EMBL:AK226596 IPI:IPI00536189 PIR:T06706 RefSeq:NP_566902.1 UniGene:At.21922 UniGene:At.67255 ProteinModelPortal:Q94BN0 SMR:Q94BN0 IntAct:Q94BN0 STRING:Q94BN0 PRIDE:Q94BN0 EnsemblPlants:AT3G48360.1 GeneID:823994 KEGG:ath:AT3G48360 TAIR:At3g48360 InParanoid:Q94BN0 OMA:RMWQLLR PhylomeDB:Q94BN0 Genevestigator:Q94BN0 GO:GO:0080134 Uniprot:Q94BN0
Root	Isotig07929	0	21	-5.521	1.09E-06	No hit
Root	Isotig07930	0	21	-5.521	1.09E-06	No hit
Root	Isotig07947	17	1	3.958	8.09E-05	TAIR|locus:2144905 - symbol:AT5G18780 "AT5G18780" species:3702 "Arabidopsis thaliana" [GO:0003674 "molecular_function" evidence=ND] [GO:0005575 "cellular_component" evidence=ND] [GO:0008150 "biological_process" evidence=ND] InterPro:IPR001810 Pfam:PF00646 PROSITE:PS50181 SMART:SM00256 EMBL:CP002688 GenomeReviews:BA000015_GR SUPFAM:SSF81383 InterPro:IPR006566 SMART:SM00579 EMBL:AC068655 HOGENOM:HBG316570 Pfam:PF08387 EMBL:AK221350 EMBL:AK230408 IPI:IPI00527938 RefSeq:NP_001190332.1 RefSeq:NP_197379.1 UniGene:At.31380 UniGene:At.54909 ProteinModelPortal:Q56YH2 SMR:Q56YH2 STRING:Q56YH2 GeneID:831996 KEGG:ath:AT5G18780 TAIR:At5g18780 eggNOG:NOG253687 InParanoid:Q56YH2 PhylomeDB:Q56YH2 Genevestigator:Q56YH2 Uniprot:Q56YH2
Root	Isotig07963	0	23	-5.653	3.32E-07	No hit
Root	Isotig07964	0	22	-5.588	6.01E-07	No hit
Root	Isotig07971	46	7	2.587	7.68E-08	TAIR|locus:2100449 - symbol:ALDH10A9 "AT3G48170" species:3702 "Arabidopsis thaliana" [GO:0004028 "3-chloroallyl aldehyde dehydrogenase activity" evidence=ISS] [GO:0005777 "peroxisome" evidence=IDA] [GO:0004029 "aldehyde dehydrogenase (NAD) activity" evidence=IDA] [GO:0008802 "betaine-aldehyde dehydrogenase activity" evidence=IDA] [GO:0009414 "response to water deprivation" evidence=IEP] [GO:0009737 "response to abscisic acid stimulus" evidence=IEP] [GO:0055114 "oxidation-reduction process" evidence=IDA] [GO:0005829 "cytosol" evidence=RCA] InterPro:IPR015590 InterPro:IPR016160 InterPro:IPR016161 InterPro:IPR016162 InterPro:IPR016163 Pfam:PF00171 PROSITE:PS00070 PROSITE:PS00687 GO:GO:0005739 GO:GO:0009737 EMBL:CP002686 GenomeReviews:BA000014_GR GO:GO:0005777 eggNOG:COG1012 Gene3D:G3DSA:3.40.309.10 Gene3D:G3DSA:3.40.605.10 SUPFAM:SSF53720 HOGENOM:HBG752218 GO:GO:0009414 HSSP:P05091 GO:GO:0008802 EMBL:AL096856 KO:K00130 ProtClustDB:PLN02467 EMBL:AF370333 EMBL:AY062987 EMBL:Z29888 IPI:IPI00545825 PIR:T13006 RefSeq:NP_190400.1 UniGene:At.1613 ProteinModelPortal:Q9STS1 SMR:Q9STS1 STRING:Q9STS1 PRIDE:Q9STS1 EnsemblPlants:AT3G48170.1 GeneID:823972 KEGG:ath:AT3G48170 TAIR:At3g48170 InParanoid:Q9STS1

Tissue	Identification	# of reads (stress)	# of reads (control)	Fold Change	p-value	Description
						OMA:MEIWREE PhylomeDB:Q9STS1 BioCyc:MetaCyc:AT3G48170-MONOMER ArrayExpress:Q9STS1 Genevestigator:Q9STS1 GermOnline:AT3G48170 Uniprot:Q9STS1
Root	Isotig07997	3	56	-4.351	1.56E-14	TAIR|locus:2119251 - symbol:AT4G33420 species:3702 "Arabidopsis thaliana" [GO:0004601 "peroxidase activity" evidence=IEA;ISS] [GO:0006979 "response to oxidative stress" evidence=IEA] [GO:0020037 "heme binding" evidence=IEA] [GO:0055114 "oxidation-reduction process" evidence=IEA] InterPro:IPR000823 InterPro:IPR002016 InterPro:IPR010255 InterPro:IPR019793 InterPro:IPR019794 Pfam:PF00141 PRINTS:PR00458 PRINTS:PR00461 PROSITE:PS00435 PROSITE:PS00436 PROSITE:PS50873 EMBL:CP002687 GenomeReviews:CT486007_GR GO:GO:0005576 GO:GO:0006979 EMBL:AL161583 EMBL:AL035678 GO:GO:0020037 GO:GO:0004601 HOGENOM:HBG597790 SUPFAM:SSF48113 KO:K00430 EMBL:AF451951 EMBL:BT044614 IPI:IPI00539387 PIR:T05993 RefSeq:NP_567919.1 UniGene:At.28650 ProteinModelPortal:Q9SZB9 SMR:Q9SZB9 STRING:Q9SZB9 PeroxiBase:213 PRIDE:Q9SZB9 EnsemblPlants:AT4G33420.1 GeneID:829479 KEGG:ath:AT4G33420 GeneFarm:1879 TAIR:At4g33420 eggNOG:NOG276365 InParanoid:Q9SZB9 ProtClustDB:CLSN2689733 Genevestigator:Q9SZB9 GermOnline:AT4G33420 Uniprot:Q9SZB9
Root	Isotig07998	5	64	-3.807	2.88E-15	TAIR|locus:2119251 - symbol:AT4G33420 species:3702 "Arabidopsis thaliana" [GO:0004601 "peroxidase activity" evidence=IEA;ISS] [GO:0006979 "response to oxidative stress" evidence=IEA] [GO:0020037 "heme binding" evidence=IEA] [GO:0055114 "oxidation-reduction process" evidence=IEA] InterPro:IPR000823 InterPro:IPR002016 InterPro:IPR010255 InterPro:IPR019793 InterPro:IPR019794 Pfam:PF00141 PRINTS:PR00458 PRINTS:PR00461 PROSITE:PS00435 PROSITE:PS00436 PROSITE:PS50873 EMBL:CP002687 GenomeReviews:CT486007_GR GO:GO:0005576 GO:GO:0006979 EMBL:AL161583 EMBL:AL035678 GO:GO:0020037 GO:GO:0004601 HOGENOM:HBG597790 SUPFAM:SSF48113 KO:K00430 EMBL:AF451951 EMBL:BT044614 IPI:IPI00539387 PIR:T05993 RefSeq:NP_567919.1 UniGene:At.28650 ProteinModelPortal:Q9SZB9 SMR:Q9SZB9 STRING:Q9SZB9 PeroxiBase:213 PRIDE:Q9SZB9 EnsemblPlants:AT4G33420.1 GeneID:829479 KEGG:ath:AT4G33420 GeneFarm:1879 TAIR:At4g33420 eggNOG:NOG276365 InParanoid:Q9SZB9 ProtClustDB:CLSN2689733 Genevestigator:Q9SZB9 GermOnline:AT4G33420 Uniprot:Q9SZB9
Root	Isotig07999	6	52	-3.244	2.28E-11	TAIR|locus:2013001 - symbol:AT1G71695 species:3702 "Arabidopsis thaliana" [GO:0004601 "peroxidase activity" evidence=IEA;ISS] [GO:0006979 "response to oxidative stress" evidence=IEA] [GO:0020037 "heme binding" evidence=IEA] [GO:0055114 "oxidation-reduction process" evidence=IEA] [GO:0005773 "vacuole" evidence=IDA] [GO:0005618 "cell wall" evidence=IDA] [GO:0009505 "plant-type cell wall" evidence=IDA] [GO:0016020 "membrane" evidence=IDA] [GO:0009506 "plasmodesma" evidence=IDA] InterPro:IPR000823 InterPro:IPR002016 InterPro:IPR010255 InterPro:IPR019793 InterPro:IPR019794 Pfam:PF00141 PRINTS:PR00458 PRINTS:PR00461 PROSITE:PS00435 PROSITE:PS00436 PROSITE:PS50873 EMBL:CP002684 GenomeReviews:CT485782_GR GO:GO:0009506 GO:GO:0005773 GO:GO:0005576 GO:GO:0016020 GO:GO:0020037 GO:GO:0009505 EMBL:AC012654 EMBL:AC016163 GO:GO:0004601 GO:GO:0042744 HOGENOM:HBG597790 SUPFAM:SSF48113 KO:K00430 EMBL:X98318 EMBL:X98773 EMBL:AF334732 EMBL:BT000715 EMBL:AY087964 IPI:IPI00538176 PIR:A96739 RefSeq:NP_177313.1 UniGene:At.67041 UniGene:At.94 ProteinModelPortal:Q96520 SMR:Q96520 STRING:Q96520 PeroxiBase:93 PRIDE:Q96520 EnsemblPlants:AT1G71695.1 GeneID:843498 KEGG:ath:AT1G71695 GeneFarm:1474 TAIR:At1g71695 eggNOG:NOG252730 InParanoid:Q96520 OMA:NCSARNT PhylomeDB:Q96520 ProtClustDB:CLSN2679267 ArrayExpress:Q96520 Genevestigator:Q96520 GermOnline:AT1G71695 Uniprot:Q96520
Root	Isotig08000	8	39	-2.414	6.49E-07	TAIR|locus:2013001 - symbol:AT1G71695 species:3702 "Arabidopsis thaliana" [GO:0004601 "peroxidase activity" evidence=IEA;ISS] [GO:0006979 "response to oxidative stress" evidence=IEA] [GO:0020037 "heme binding" evidence=IEA] [GO:0055114 "oxidation-reduction process" evidence=IEA] [GO:0005773 "vacuole" evidence=IDA] [GO:0005618 "cell wall" evidence=IDA] [GO:0009505 "plant-type cell wall" evidence=IDA] [GO:0016020 "membrane" evidence=IDA] [GO:0009506 "plasmodesma" evidence=IDA] InterPro:IPR000823 InterPro:IPR002016 InterPro:IPR010255 InterPro:IPR019793 InterPro:IPR019794 Pfam:PF00141 PRINTS:PR00458 PRINTS:PR00461 PROSITE:PS00435 PROSITE:PS00436 PROSITE:PS50873 EMBL:CP002684 GenomeReviews:CT485782_GR GO:GO:0009506 GO:GO:0005773 GO:GO:0005576 GO:GO:0016020 GO:GO:0020037 GO:GO:0009505 EMBL:AC012654 EMBL:AC016163 GO:GO:0004601 GO:GO:0042744 HOGENOM:HBG597790 SUPFAM:SSF48113 KO:K00430 EMBL:X98318 EMBL:X98773 EMBL:AF334732 EMBL:BT000715 EMBL:AY087964 IPI:IPI00538176 PIR:A96739 RefSeq:NP_177313.1 UniGene:At.67041 UniGene:At.94 ProteinModelPortal:Q96520 SMR:Q96520 STRING:Q96520 PeroxiBase:93 PRIDE:Q96520 EnsemblPlants:AT1G71695.1 GeneID:843498 KEGG:ath:AT1G71695 GeneFarm:1474 TAIR:At1g71695

Tissue	Identification	# of reads (stress)	# of reads (control)	Fold Change	p-value	Description
						eggNOG:NOG252730 InParanoid:Q96520 OMA:NCSARNT PhylomeDB:Q96520 ProtClustDB:CLSN2679267 ArrayExpress:Q96520 Genevestigator:Q96520 GermOnline:AT1G71695 Uniprot:Q96520
Root	Isotig08003	2	14	-2.936	0.000925454	TAIR|locus:2130005 - symbol:AT4G14620 "AT4G14620" species:3702 "Arabidopsis thaliana" [GO:0003674 "molecular_function" evidence=ND] [GO:0008150 "biological_process" evidence=ND] EMBL:CP002687 InterPro:IPR006502 Pfam:PF04720 TIGRFAMs:TIGR01615 EMBL:AY133694 IPI:IPI00536806 RefSeq:NP_193198.2 UniGene:At.33259 PRIDE:Q8L7H9 EnsemblPlants:AT4G14620.1 GeneID:827112 KEGG:ath:AT4G14620 TAIR:At4g14620 InParanoid:Q8L7H9 OMA:EENNDKQ PhylomeDB:Q8L7H9 ProtClustDB:CLSN2918097 Genevestigator:Q8L7H9 Uniprot:Q8L7H9
Root	Isotig08004	2	14	-2.936	0.000925454	TAIR|locus:2084593 - symbol:AT3G22970 "AT3G22970" species:3702 "Arabidopsis thaliana" [GO:0003674 "molecular_function" evidence=ND] [GO:0008150 "biological_process" evidence=ND] EMBL:CP002686 EMBL:AP001300 InterPro:IPR006502 Pfam:PF04720 TIGRFAMs:TIGR01615 EMBL:BT024897 IPI:IPI00544999 RefSeq:NP_188937.1 UniGene:At.43562 UniGene:At.71498 UniGene:At.72007 PRIDE:Q9LIJ8 EnsemblPlants:AT3G22970.1 GeneID:821871 KEGG:ath:AT3G22970 TAIR:At3g22970 InParanoid:Q9LIJ8 OMA:VIIGEER PhylomeDB:Q9LIJ8 ProtClustDB:CLSN2913460 Genevestigator:Q9LIJ8 Uniprot:Q9LIJ8
Root	Isotig08005	15	45	-1.714	1.83E-05	TAIR|locus:2183254 - symbol:AT5G19760 "AT5G19760" species:3702 "Arabidopsis thaliana" [GO:0005743 "mitochondrial inner membrane" evidence=ISS] [GO:0006810 "transport" evidence=ISS] [GO:0006839 "mitochondrial transport" evidence=ISS] [GO:0017077 "oxidative phosphorylation uncoupler activity" evidence=ISS] [GO:0005310 "dicarboxylic acid transmembrane transporter activity" evidence=IDA] [GO:0006835 "dicarboxylic acid transport" evidence=IDA] [GO:0015142 "tricarboxylic acid transmembrane transporter activity" evidence=IDA] [GO:0035674 "tricarboxylic acid transmembrane transport" evidence=IDA] [GO:0009941 "chloroplast envelope" evidence=IDA] [GO:0005739 "mitochondrion" evidence=IDA] [GO:0009507 "chloroplast" evidence=IDA] [GO:0005618 "cell wall" evidence=IDA] [GO:0005774 "vacuolar membrane" evidence=IDA] [GO:0009506 "plasmodesma" evidence=IDA] [GO:0005794 "Golgi apparatus" evidence=RCA] InterPro:IPR002067 PRINTS:PR00926 Pfam:PF00153 GO:GO:0016021 GO:GO:0009506 GO:GO:0005774 EMBL:CP002688 GO:GO:0005618 GO:GO:0005488 GO:GO:0005743 GO:GO:0009941 InterPro:IPR018108 InterPro:IPR023395 Gene3D:G3DSA:1.50.40.10 SUPFAM:SSF103506 PROSITE:PS50920 GO:GO:0005310 KO:K15104 UniGene:At.24504 EMBL:AF360153 EMBL:AY056307 EMBL:AY085901 EMBL:AK226470 EMBL:AJ311780 IPI:IPI00545483 RefSeq:NP_197477.1 UniGene:At.24857 UniGene:At.49038 ProteinModelPortal:Q9C5M0 SMR:Q9C5M0 STRING:Q9C5M0 PRIDE:Q9C5M0 ProMEX:Q9C5M0 DNASU:832096 EnsemblPlants:AT5G19760.1 GeneID:832096 KEGG:ath:AT5G19760 TAIR:At5g19760 InParanoid:Q9C5M0 OMA:LRQAVYT PhylomeDB:Q9C5M0 ProtClustDB:CLSN2687170 ArrayExpress:Q9C5M0 Genevestigator:Q9C5M0 GO:GO:0015142 Uniprot:Q9C5M0
Root	Isotig08006	16	44	-1.588	6.01E-05	TAIR|locus:2183254 - symbol:AT5G19760 "AT5G19760" species:3702 "Arabidopsis thaliana" [GO:0005743 "mitochondrial inner membrane" evidence=ISS] [GO:0006810 "transport" evidence=ISS] [GO:0006839 "mitochondrial transport" evidence=ISS] [GO:0017077 "oxidative phosphorylation uncoupler activity" evidence=ISS] [GO:0005310 "dicarboxylic acid transmembrane transporter activity" evidence=IDA] [GO:0006835 "dicarboxylic acid transport" evidence=IDA] [GO:0015142 "tricarboxylic acid transmembrane transporter activity" evidence=IDA] [GO:0035674 "tricarboxylic acid transmembrane transport" evidence=IDA] [GO:0009941 "chloroplast envelope" evidence=IDA] [GO:0005739 "mitochondrion" evidence=IDA] [GO:0009507 "chloroplast" evidence=IDA] [GO:0005618 "cell wall" evidence=IDA] [GO:0005774 "vacuolar membrane" evidence=IDA] [GO:0009506 "plasmodesma" evidence=IDA] [GO:0005794 "Golgi apparatus" evidence=RCA] InterPro:IPR002067 PRINTS:PR00926 Pfam:PF00153 GO:GO:0016021 GO:GO:0009506 GO:GO:0005774 EMBL:CP002688 GO:GO:0005618 GO:GO:0005488 GO:GO:0005743 GO:GO:0009941 InterPro:IPR018108 InterPro:IPR023395 Gene3D:G3DSA:1.50.40.10 SUPFAM:SSF103506 PROSITE:PS50920 GO:GO:0005310 KO:K15104 UniGene:At.24504 EMBL:AF360153 EMBL:AY056307 EMBL:AY085901 EMBL:AK226470 EMBL:AJ311780 IPI:IPI00545483 RefSeq:NP_197477.1 UniGene:At.24857 UniGene:At.49038 ProteinModelPortal:Q9C5M0 SMR:Q9C5M0 STRING:Q9C5M0 PRIDE:Q9C5M0 ProMEX:Q9C5M0 DNASU:832096 EnsemblPlants:AT5G19760.1 GeneID:832096 KEGG:ath:AT5G19760 TAIR:At5g19760 InParanoid:Q9C5M0 OMA:LRQAVYT PhylomeDB:Q9C5M0 ProtClustDB:CLSN2687170 ArrayExpress:Q9C5M0 Genevestigator:Q9C5M0 GO:GO:0015142 Uniprot:Q9C5M0
Root	Isotig08009	1	14	-3.936	0.000188601	TAIR|locus:2076591 - symbol:AT3G57790 species:3702 "Arabidopsis thaliana" [GO:0004650 "polygalacturonase activity" evidence=IEA;ISS] [GO:0005975 "carbohydrate metabolic process" evidence=IEA;ISS] [GO:0005774

Tissue	Identification	# of reads (stress)	# of reads (control)	Fold Change	p-value	Description
						"vacuolar membrane" evidence=IDA] InterPro:IPR000743 Pfam:PF00295 GO:GO:0005774 GO:GO:0005576 GO:GO:0005975 GO:GO:0004650 InterPro:IPR006626 InterPro:IPR012334 InterPro:IPR011050 Gene3D:G3DSA:2.160.20.10 SMART:SM00710 SUPFAM:SSF51126 GO:GO:0007047 EMBL:AL049660 InterPro:IPR019329 Pfam:PF10183 IPI:IPI00532018 PIR:T06741 ProteinModelPortal:Q9SVZ3 SMR:Q9SVZ3 STRING:Q9SVZ3 PRIDE:Q9SVZ3 TAIR:At3g57790 InParanoid:Q9SVZ3 PhylomeDB:Q9SVZ3 Genevestigator:Q9SVZ3 Uniprot:Q9SVZ3
Root	Isotig08010	1	13	-3.829	0.000362856	TAIR|locus:2076591 - symbol:AT3G57790 species:3702 "Arabidopsis thaliana" [GO:0004650 "polygalacturonase activity" evidence=IEA;ISS] [GO:0005975 "carbohydrate metabolic process" evidence=IEA;ISS] [GO:0005774 "vacuolar membrane" evidence=IDA] InterPro:IPR000743 Pfam:PF00295 GO:GO:0005774 GO:GO:0005576 GO:GO:0005975 GO:GO:0004650 InterPro:IPR006626 InterPro:IPR012334 InterPro:IPR011050 Gene3D:G3DSA:2.160.20.10 SMART:SM00710 SUPFAM:SSF51126 GO:GO:0007047 EMBL:AL049660 InterPro:IPR019329 Pfam:PF10183 IPI:IPI00532018 PIR:T06741 ProteinModelPortal:Q9SVZ3 SMR:Q9SVZ3 STRING:Q9SVZ3 PRIDE:Q9SVZ3 TAIR:At3g57790 InParanoid:Q9SVZ3 PhylomeDB:Q9SVZ3 Genevestigator:Q9SVZ3 Uniprot:Q9SVZ3
Root	Isotig08021	0	52	-6.829	3.96E-14	TAIR|locus:2168912 - symbol:AAP6 "AT5G49630" species:3702 "Arabidopsis thaliana" [GO:0015171 "amino acid transmembrane transporter activity" evidence=ISS] [GO:0016020 "membrane" evidence=ISS] [GO:0015172 "acidic amino acid transmembrane transporter activity" evidence=IDA] [GO:0015175 "neutral amino acid transmembrane transporter activity" evidence=IDA] [GO:0015810 "aspartate transport" evidence=IDA] [GO:0015827 "tryptophan transport" evidence=IDA] [GO:0005886 "plasma membrane" evidence=IDA] GO:GO:0016021 GO:GO:0005886 EMBL:CP002688 GenomeReviews:BA000015_GR GO:GO:0015175 GO:GO:0015827 GO:GO:0015293 InterPro:IPR013057 Pfam:PF01490 HOGENOM:HBG319901 GO:GO:0015172 EMBL:X95736 EMBL:AB025627 EMBL:AK229102 IPI:IPI00517925 PIR:T50691 RefSeq:NP_199774.1 UniGene:At.163 ProteinModelPortal:P92934 TCDB:2.A.18.2.4 EnsemblPlants:AT5G49630.1 GeneID:835025 KEGG:ath:AT5G49630 TAIR:At5g49630 eggNOG:NOG265519 InParanoid:P92934 OMA:FAYAYST PhylomeDB:P92934 ProtClustDB:CLSN2686986 GO:GO:0015810 Uniprot:P92934
Root	Isotig08022	0	50	-6.773	1.11E-13	TAIR|locus:2168912 - symbol:AAP6 "AT5G49630" species:3702 "Arabidopsis thaliana" [GO:0015171 "amino acid transmembrane transporter activity" evidence=ISS] [GO:0016020 "membrane" evidence=ISS] [GO:0015172 "acidic amino acid transmembrane transporter activity" evidence=IDA] [GO:0015175 "neutral amino acid transmembrane transporter activity" evidence=IDA] [GO:0015810 "aspartate transport" evidence=IDA] [GO:0015827 "tryptophan transport" evidence=IDA] [GO:0005886 "plasma membrane" evidence=IDA] GO:GO:0016021 GO:GO:0005886 EMBL:CP002688 GenomeReviews:BA000015_GR GO:GO:0015175 GO:GO:0015827 GO:GO:0015293 InterPro:IPR013057 Pfam:PF01490 HOGENOM:HBG319901 GO:GO:0015172 EMBL:X95736 EMBL:AB025627 EMBL:AK229102 IPI:IPI00517925 PIR:T50691 RefSeq:NP_199774.1 UniGene:At.163 ProteinModelPortal:P92934 TCDB:2.A.18.2.4 EnsemblPlants:AT5G49630.1 GeneID:835025 KEGG:ath:AT5G49630 TAIR:At5g49630 eggNOG:NOG265519 InParanoid:P92934 OMA:FAYAYST PhylomeDB:P92934 ProtClustDB:CLSN2686986 GO:GO:0015810 Uniprot:P92934
Root	Isotig08041	23	5	2.073	0.000873448	TAIR|locus:2092339 - symbol:ERF7 "AT3G20310" species:3702 "Arabidopsis thaliana" [GO:0003700 "sequence-specific DNA binding transcription factor activity" evidence=ISS;TAS] [GO:0006355 "regulation of transcription, DNA-dependent" evidence=TAS] [GO:0003677 "DNA binding" evidence=TAS] [GO:0005634 "nucleus" evidence=IC;IDA] [GO:0005515 "protein binding" evidence=IPI] [GO:0009414 "response to water deprivation" evidence=IMP] [GO:0009737 "response to abscisic acid stimulus" evidence=IMP] [GO:0043565 "sequence-specific DNA binding" evidence=IDA] [GO:0045892 "negative regulation of transcription, DNA-dependent" evidence=IDA] [GO:0009873 "ethylene mediated signaling pathway" evidence=TAS] InterPro:IPR001471 InterPro:IPR016177 Pfam:PF00847 PRINTS:PR00367 PROSITE:PS51032 SMART:SM00380 GO:GO:0009737 GO:GO:0005634 EMBL:CP002686 GenomeReviews:BA000014_GR GO:GO:0045892 GO:GO:0005515 GO:GO:0006952 GO:GO:0009873 GO:GO:0043565 GO:GO:0003700 GO:GO:0006351 GO:GO:0009414 KO:K09286 Gene3D:G3DSA:3.30.730.10 SUPFAM:SSF54171 EMBL:AB024036 HOGENOM:HBG744062 ProtClustDB:CLSN2682907 EMBL:AB032201 EMBL:AY037254 EMBL:AY094001 EMBL:AY085023 EMBL:AK228460 IPI:IPI00537654 RefSeq:NP_188666.1 UniGene:At.20161 ProteinModelPortal:Q9LDE4 SMR:Q9LDE4 IntAct:Q9LDE4 STRING:Q9LDE4 PRIDE:Q9LDE4 EnsemblPlants:AT3G20310.1 GeneID:821575 KEGG:ath:AT3G20310 TAIR:At3g20310 eggNOG:NOG322216 InParanoid:Q9LDE4 OMA:VAPEDCH PhylomeDB:Q9LDE4 ArrayExpress:Q9LDE4 Genevestigator:Q9LDE4 GermOnline:AT3G20310 Uniprot:Q9LDE4

Tissue	Identification	# of reads (stress)	# of reads (control)	Fold Change	p-value	Description
Root	Isotig08043	1	58	-5.987	5.40E-16	TAIR|locus:2202007 - symbol:AT1G12230 "AT1G12230" species:3702 "Arabidopsis thaliana" [GO:0004801 "sedoheptulose-7-phosphate:D-glyceraldehyde-3-phosphate glyceronetransferase activity" evidence=ISS] [GO:0005975 "carbohydrate metabolic process" evidence=IEA] [GO:0008152 "metabolic process" evidence=IEA] [GO:0009507 "chloroplast" evidence=IDA] [GO:0008270 "zinc ion binding" evidence=IDA] [GO:0009570 "chloroplast stroma" evidence=IDA] [GO:0009941 "chloroplast envelope" evidence=IDA] InterPro:IPR001585 InterPro:IPR013785 Pfam:PF00923 EMBL:CP002684 GO:GO:0009570 Gene3D:G3DSA:3.20.20.70 GO:GO:0003824 GO:GO:0005975 GO:GO:0008270 GO:GO:0009941 EMBL:AC022522 PANTHER:PTHR10683 KO:K00616 EMBL:BT002036 EMBL:BT008487 IPI:IPI00518291 PIR:D86257 RefSeq:NP_563900.1 UniGene:At.16219 HSSP:P30148 ProteinModelPortal:Q9FWX0 SMR:Q9FWX0 STRING:Q9FWX0 PRIDE:Q9FWX0 EnsemblPlants:AT1G12230.1 GeneID:837776 KEGG:ath:AT1G12230 TAIR:At1g12230 InParanoid:Q9FWX0 OMA:DWHKAKT PhylomeDB:Q9FWX0 ProtClustDB:CLSN2687776 ArrayExpress:Q9FWX0 Genevestigator:Q9FWX0 Uniprot:Q9FWX0
Root	Isotig08044	1	56	-5.936	1.67E-15	TAIR|locus:2202007 - symbol:AT1G12230 "AT1G12230" species:3702 "Arabidopsis thaliana" [GO:0004801 "sedoheptulose-7-phosphate:D-glyceraldehyde-3-phosphate glyceronetransferase activity" evidence=ISS] [GO:0005975 "carbohydrate metabolic process" evidence=IEA] [GO:0008152 "metabolic process" evidence=IEA] [GO:0009507 "chloroplast" evidence=IDA] [GO:0008270 "zinc ion binding" evidence=IDA] [GO:0009570 "chloroplast stroma" evidence=IDA] [GO:0009941 "chloroplast envelope" evidence=IDA] InterPro:IPR001585 InterPro:IPR013785 Pfam:PF00923 EMBL:CP002684 GO:GO:0009570 Gene3D:G3DSA:3.20.20.70 GO:GO:0003824 GO:GO:0005975 GO:GO:0008270 GO:GO:0009941 EMBL:AC022522 PANTHER:PTHR10683 KO:K00616 EMBL:BT002036 EMBL:BT008487 IPI:IPI00518291 PIR:D86257 RefSeq:NP_563900.1 UniGene:At.16219 HSSP:P30148 ProteinModelPortal:Q9FWX0 SMR:Q9FWX0 STRING:Q9FWX0 PRIDE:Q9FWX0 EnsemblPlants:AT1G12230.1 GeneID:837776 KEGG:ath:AT1G12230 TAIR:At1g12230 InParanoid:Q9FWX0 OMA:DWHKAKT PhylomeDB:Q9FWX0 ProtClustDB:CLSN2687776 ArrayExpress:Q9FWX0 Genevestigator:Q9FWX0 Uniprot:Q9FWX0
Root	Isotig08069	1	24	-4.714	3.11E-07	TAIR|locus:2167225 - symbol:PSY "AT5G17230" species:3702 "Arabidopsis thaliana" [GO:0016117 "carotenoid biosynthetic process" evidence=IMP] [GO:0016767 "geranylgeranyl-diphosphate geranylgeranyltransferase activity" evidence=ISS;TAS] [GO:0046905 "phytoene synthase activity" evidence=TAS] [GO:0009507 "chloroplast" evidence=IDA] InterPro:IPR002060 InterPro:IPR019845 Pfam:PF00494 PROSITE:PS01044 PROSITE:PS01045 EMBL:CP002688 GenomeReviews:BA000015_GR GO:GO:0009507 InterPro:IPR008949 Gene3D:G3DSA:1.10.600.10 SUPFAM:SSF48576 EMBL:AB005238 eggNOG:COG1562 GO:GO:0016767 GO:GO:0046905 EMBL:L25812 EMBL:AF009954 EMBL:BT000450 EMBL:BT002084 EMBL:AY085565 IPI:IPI00527204 RefSeq:NP_001031895.1 RefSeq:NP_197225.1 UniGene:At.23726 ProteinModelPortal:P37271 SMR:P37271 STRING:P37271 PRIDE:P37271 EnsemblPlants:AT5G17230.1 EnsemblPlants:AT5G17230.2 GeneID:831587 KEGG:ath:AT5G17230 TAIR:At5g17230 HOGENOM:HBG693191 InParanoid:P37271 KO:K02291 OMA:ELKLYCY PhylomeDB:P37271 ProtClustDB:PLN02632 BioCyc:ARA:AT5G17230-MONOMER BioCyc:MetaCyc:AT5G17230-MONOMER ArrayExpress:P37271 Genevestigator:P37271 GermOnline:AT5G17230 Uniprot:P37271
Root	Isotig08070	1	20	-4.451	3.92E-06	TAIR|locus:2167225 - symbol:PSY "AT5G17230" species:3702 "Arabidopsis thaliana" [GO:0016117 "carotenoid biosynthetic process" evidence=IMP] [GO:0016767 "geranylgeranyl-diphosphate geranylgeranyltransferase activity" evidence=ISS;TAS] [GO:0046905 "phytoene synthase activity" evidence=TAS] [GO:0009507 "chloroplast" evidence=IDA] InterPro:IPR002060 InterPro:IPR019845 Pfam:PF00494 PROSITE:PS01044 PROSITE:PS01045 EMBL:CP002688 GenomeReviews:BA000015_GR GO:GO:0009507 InterPro:IPR008949 Gene3D:G3DSA:1.10.600.10 SUPFAM:SSF48576 EMBL:AB005238 eggNOG:COG1562 GO:GO:0016767 GO:GO:0046905 EMBL:L25812 EMBL:AF009954 EMBL:BT000450 EMBL:BT002084 EMBL:AY085565 IPI:IPI00527204 RefSeq:NP_001031895.1 RefSeq:NP_197225.1 UniGene:At.23726 ProteinModelPortal:P37271 SMR:P37271 STRING:P37271 PRIDE:P37271 EnsemblPlants:AT5G17230.1 EnsemblPlants:AT5G17230.2 GeneID:831587 KEGG:ath:AT5G17230 TAIR:At5g17230 HOGENOM:HBG693191 InParanoid:P37271 KO:K02291 OMA:ELKLYCY PhylomeDB:P37271 ProtClustDB:PLN02632 BioCyc:ARA:AT5G17230-MONOMER BioCyc:MetaCyc:AT5G17230-MONOMER ArrayExpress:P37271 Genevestigator:P37271 GermOnline:AT5G17230 Uniprot:P37271
Root	Isotig08075	43	121	-1.622	1.44E-11	TAIR|locus:2177386 - symbol:GRF3 "AT5G38480" species:3702 "Arabidopsis thaliana" [GO:0045309 "protein phosphorylated amino acid binding" evidence=TAS] [GO:0005773 "vacuole" evidence=IDA] [GO:0005618 "cell wall"

Tissue	Identification	# of reads (stress)	# of reads (control)	Fold Change	p-value	Description
						evidence=IDA] [GO:0005524 "ATP binding" evidence=IDA] [GO:0005739 "mitochondrion" evidence=IDA] [GO:0005886 "plasma membrane" evidence=IDA] [GO:0009507 "chloroplast" evidence=IDA] [GO:0005829 "cytosol" evidence=IDA] [GO:0009506 "plasmodesma" evidence=IDA] [GO:0005794 "Golgi apparatus" evidence=IDA] InterPro:IPR000308 PIRSF:PIRSF000868 PRINTS:PR00305 eggNOG:COG5040 HOGENOM:HBG611720 GO:GO:0005829 GO:GO:0005739 GO:GO:0005886 GO:GO:0009506 GO:GO:0005524 GO:GO:0019904 InterPro:IPR023409 InterPro:IPR023410 Gene3D:G3DSA:1.20.190.20 PANTHER:PTHR18860 Pfam:PF00244 SMART:SM00101 SUPFAM:SSF48445 PROSITE:PS00796 PROSITE:PS00797 GO:GO:0005773 KO:K06630 EMBL:L09110 EMBL:X74140 EMBL:U09375 EMBL:AB005231 EMBL:AB005248 EMBL:CP002688 EMBL:AF412093 EMBL:AY093976 EMBL:AK226217 EMBL:AK228478 IPI:IPI00538115 PIR:S47969 PIR:S57277 RefSeq:NP_568557.1 UniGene:At.22344 UniGene:At.22949 ProteinModelPortal:P42644 SMR:P42644 IntAct:P42644 STRING:P42644 PRIDE:P42644 EnsemblPlants:AT5G38480.1 GeneID:833836 GenomeReviews:BA000015_GR KEGG:ath:AT5G38480 TAIR:At5g38480 InParanoid:P42644 OMA:KESALIM PhylomeDB:P42644 ProtClustDB:CLSN2689896 ArrayExpress:P42644 Genevestigator:P42644 GO:GO:0005618 GO:GO:0009507 Uniprot:P42644
Root	Isotig08076	43	120	-1.610	2.23E-11	TAIR|locus:2177386 - symbol:GRF3 "AT5G38480" species:3702 "Arabidopsis thaliana" [GO:0045309 "protein phosphorylated amino acid binding" evidence=TAS] [GO:0005773 "vacuole" evidence=IDA] [GO:0005618 "cell wall" evidence=IDA] [GO:0005524 "ATP binding" evidence=IDA] [GO:0005739 "mitochondrion" evidence=IDA] [GO:0005886 "plasma membrane" evidence=IDA] [GO:0009507 "chloroplast" evidence=IDA] [GO:0005829 "cytosol" evidence=IDA] [GO:0009506 "plasmodesma" evidence=IDA] [GO:0005794 "Golgi apparatus" evidence=IDA] InterPro:IPR000308 PIRSF:PIRSF000868 PRINTS:PR00305 eggNOG:COG5040 HOGENOM:HBG611720 GO:GO:0005829 GO:GO:0005739 GO:GO:0005886 GO:GO:0009506 GO:GO:0005524 GO:GO:0019904 InterPro:IPR023409 InterPro:IPR023410 Gene3D:G3DSA:1.20.190.20 PANTHER:PTHR18860 Pfam:PF00244 SMART:SM00101 SUPFAM:SSF48445 PROSITE:PS00796 PROSITE:PS00797 GO:GO:0005773 KO:K06630 EMBL:L09110 EMBL:X74140 EMBL:U09375 EMBL:AB005231 EMBL:AB005248 EMBL:CP002688 EMBL:AF412093 EMBL:AY093976 EMBL:AK226217 EMBL:AK228478 IPI:IPI00538115 PIR:S47969 PIR:S57277 RefSeq:NP_568557.1 UniGene:At.22344 UniGene:At.22949 ProteinModelPortal:P42644 SMR:P42644 IntAct:P42644 STRING:P42644 PRIDE:P42644 EnsemblPlants:AT5G38480.1 GeneID:833836 GenomeReviews:BA000015_GR KEGG:ath:AT5G38480 TAIR:At5g38480 InParanoid:P42644 OMA:KESALIM PhylomeDB:P42644 ProtClustDB:CLSN2689896 ArrayExpress:P42644 Genevestigator:P42644 GO:GO:0005618 GO:GO:0009507 Uniprot:P42644
Root	Isotig08091	21	46	-1.260	0.000570291	TAIR|locus:2075236 - symbol:MYB48 "AT3G46130" species:3702 "Arabidopsis thaliana" [GO:0003677 "DNA binding" evidence=ISS] [GO:0003700 "sequence-specific DNA binding transcription factor activity" evidence=ISS] [GO:0006355 "regulation of transcription, DNA-dependent" evidence=ISS;TAS] [GO:0009751 "response to salicylic acid stimulus" evidence=IEP] [GO:0005634 "nucleus" evidence=IDA] InterPro:IPR001005 InterPro:IPR009057 Pfam:PF00249 SMART:SM00717 GO:GO:0005634 EMBL:CP002686 GenomeReviews:BA000014_GR GO:GO:0006355 GO:GO:0003677 GO:GO:0006351 GO:GO:0009751 Gene3D:G3DSA:1.10.10.60 SUPFAM:SSF46689 InterPro:IPR017930 PROSITE:PS51294 eggNOG:COG5147 InterPro:IPR015495 PANTHER:PTHR10641 EMBL:AL355775 HOGENOM:HBG750380 EMBL:AF272733 EMBL:AY519594 EMBL:DQ075255 EMBL:DQ075256 EMBL:DQ075257 EMBL:AK176475 IPI:IPI00528378 IPI:IPI00656574 IPI:IPI00759334 IPI:IPI00759367 PIR:T49254 RefSeq:NP_001030816.1 RefSeq:NP_001078249.1 RefSeq:NP_190199.1 UniGene:At.1475 UniGene:At.72744 ProteinModelPortal:Q9LX82 SMR:Q9LX82 STRING:Q9LX82 EnsemblPlants:AT3G46130.1 GeneID:823756 KEGG:ath:AT3G46130 GeneFarm:1132 TAIR:At3g46130 InParanoid:Q9LX82 OMA:REIDHSA PhylomeDB:Q9LX82 ProtClustDB:CLSN2684805 Genevestigator:Q9LX82 GermOnline:AT3G46130 Uniprot:Q9LX82
Root	Isotig08092	21	46	-1.260	0.000570291	TAIR|locus:2075236 - symbol:MYB48 "AT3G46130" species:3702 "Arabidopsis thaliana" [GO:0003677 "DNA binding" evidence=ISS] [GO:0003700 "sequence-specific DNA binding transcription factor activity" evidence=ISS] [GO:0006355 "regulation of transcription, DNA-dependent" evidence=ISS;TAS] [GO:0009751 "response to salicylic acid stimulus" evidence=IEP] [GO:0005634 "nucleus" evidence=IDA] InterPro:IPR001005 InterPro:IPR009057 Pfam:PF00249 SMART:SM00717 GO:GO:0005634 EMBL:CP002686 GenomeReviews:BA000014_GR GO:GO:0006355 GO:GO:0003677 GO:GO:0006351 GO:GO:0009751 Gene3D:G3DSA:1.10.10.60 SUPFAM:SSF46689 InterPro:IPR017930 PROSITE:PS51294 eggNOG:COG5147 InterPro:IPR015495 PANTHER:PTHR10641 EMBL:AL355775 HOGENOM:HBG750380 EMBL:AF272733 EMBL:AY519594

Tissue	Identification	# of reads (stress)	# of reads (control)	Fold Change	p-value	Description
						EMBL:DQ075255 EMBL:DQ075256 EMBL:DQ075257 EMBL:AK176475 IPI:IPI00528378 IPI:IPI00656574 IPI:IPI00759334 IPI:IPI00759367 PIR:T49254 RefSeq:NP_001030816.1 RefSeq:NP_001078249.1 RefSeq:NP_190199.1 UniGene:At.1475 UniGene:At.72744 ProteinModelPortal:Q9LX82 SMR:Q9LX82 STRING:Q9LX82 EnsemblPlants:AT3G46130.1 GeneID:823756 KEGG:ath:AT3G46130 GeneFarm:1132 TAIR:At3g46130 InParanoid:Q9LX82 OMA:REIDHSA PhylomeDB:Q9LX82 ProtClustDB:CLSN2684805 Genevestigator:Q9LX82 GermOnline:AT3G46130 Uniprot:Q9LX82
Root	Isotig08111	28	2	3.678	8.04E-07	TAIR|locus:2118929 - symbol:OST1 "AT4G33950" species:3702 "Arabidopsis thaliana" [GO:0016301 "kinase activity" evidence=ISS;IDA] [GO:0009414 "response to water deprivation" evidence=IGI;IMP] [GO:0009737 "response to abscisic acid stimulus" evidence=IDA;IMP] [GO:0010119 "regulation of stomatal movement" evidence=IMP] [GO:0004672 "protein kinase activity" evidence=IDA;TAS] [GO:0009931 "calcium-dependent protein serine/threonine kinase activity" evidence=ISS] [GO:0009738 "abscisic acid mediated signaling pathway" evidence=TAS] [GO:2000377 "regulation of reactive oxygen species metabolic process" evidence=IMP] [GO:0006970 "response to osmotic stress" evidence=IGI;IDA] [GO:0009651 "response to salt stress" evidence=IDA] [GO:0005515 "protein binding" evidence=IPI] [GO:0010118 "stomatal movement" evidence=IMP] [GO:0042742 "defense response to bacterium" evidence=IMP] [GO:0005829 "cytosol" evidence=RCA;IDA] [GO:0005634 "nucleus" evidence=IDA] [GO:0005737 "cytoplasm" evidence=IDA] [GO:0005985 "sucrose metabolic process" evidence=IMP] [GO:0006636 "unsaturated fatty acid biosynthetic process" evidence=IMP] [GO:0019432 "triglyceride biosynthetic process" evidence=IMP] [GO:0040007 "growth" evidence=IMP] [GO:0048366 "leaf development" evidence=IMP] InterPro:IPR000719 InterPro:IPR002290 InterPro:IPR008271 InterPro:IPR011009 InterPro:IPR017441 Pfam:PF00069 PROSITE:PS00107 PROSITE:PS00108 PROSITE:PS50011 SMART:SM00220 GO:GO:0005829 GO:GO:0005524 EMBL:CP002687 GenomeReviews:CT486007_GR GO:GO:0005634 GO:GO:0040007 GO:GO:0042742 GO:GO:0009738 GO:GO:0010119 eggNOG:COG0515 SUPFAM:SSF56112 GO:GO:0004674 HOGENOM:HBG755340 GO:GO:0009651 GO:GO:0009414 GO:GO:0010118 GO:GO:0019432 GO:GO:0048366 GO:GO:0006636 EMBL:AL031032 EMBL:AL161584 GO:GO:0019903 GO:GO:0010359 GO:GO:0005985 GO:GO:2000377 KO:K14498 EMBL:AJ316009 EMBL:AY054624 EMBL:AY081538 IPI:IPI00547757 IPI:IPI00900499 PIR:T05223 RefSeq:NP_567945.1 UniGene:At.2399 PDB:3UC4 PDB:3UDB PDB:3ZUT PDB:3ZUU PDBsum:3UC4 PDBsum:3UDB PDBsum:3ZUT PDBsum:3ZUU ProteinModelPortal:Q940H6 SMR:Q940H6 DIP:DIP-36705N IntAct:Q940H6 MINT:MINT-7260107 STRING:Q940H6 PRIDE:Q940H6 EnsemblPlants:AT4G33950.1 GeneID:829541 KEGG:ath:AT4G33950 TAIR:At4g33950 InParanoid:Q940H6 OMA:QYEEPEQ PhylomeDB:Q940H6 ProtClustDB:CLSN2917660 Genevestigator:Q940H6 Uniprot:Q940H6
Root	Isotig08112	27	2	3.626	1.44E-06	TAIR|locus:2118929 - symbol:OST1 "AT4G33950" species:3702 "Arabidopsis thaliana" [GO:0016301 "kinase activity" evidence=ISS;IDA] [GO:0009414 "response to water deprivation" evidence=IGI;IMP] [GO:0009737 "response to abscisic acid stimulus" evidence=IDA;IMP] [GO:0010119 "regulation of stomatal movement" evidence=IMP] [GO:0004672 "protein kinase activity" evidence=IDA;TAS] [GO:0009931 "calcium-dependent protein serine/threonine kinase activity" evidence=ISS] [GO:0009738 "abscisic acid mediated signaling pathway" evidence=TAS] [GO:2000377 "regulation of reactive oxygen species metabolic process" evidence=IMP] [GO:0006970 "response to osmotic stress" evidence=IGI;IDA] [GO:0009651 "response to salt stress" evidence=IDA] [GO:0005515 "protein binding" evidence=IPI] [GO:0010118 "stomatal movement" evidence=IMP] [GO:0042742 "defense response to bacterium" evidence=IMP] [GO:0005829 "cytosol" evidence=RCA;IDA] [GO:0005634 "nucleus" evidence=IDA] [GO:0005737 "cytoplasm" evidence=IDA] [GO:0005985 "sucrose metabolic process" evidence=IMP] [GO:0006636 "unsaturated fatty acid biosynthetic process" evidence=IMP] [GO:0019432 "triglyceride biosynthetic process" evidence=IMP] [GO:0040007 "growth" evidence=IMP] [GO:0048366 "leaf development" evidence=IMP] InterPro:IPR000719 InterPro:IPR002290 InterPro:IPR008271 InterPro:IPR011009 InterPro:IPR017441 Pfam:PF00069 PROSITE:PS00107 PROSITE:PS00108 PROSITE:PS50011 SMART:SM00220 GO:GO:0005829 GO:GO:0005524 EMBL:CP002687 GenomeReviews:CT486007_GR GO:GO:0005634 GO:GO:0040007 GO:GO:0042742 GO:GO:0009738 GO:GO:0010119 eggNOG:COG0515 SUPFAM:SSF56112 GO:GO:0004674 HOGENOM:HBG755340 GO:GO:0009651 GO:GO:0009414 GO:GO:0010118 GO:GO:0019432 GO:GO:0048366 GO:GO:0006636 EMBL:AL031032 EMBL:AL161584 GO:GO:0019903 GO:GO:0010359 GO:GO:0005985 GO:GO:2000377 KO:K14498 EMBL:AJ316009 EMBL:AY054624 EMBL:AY081538 IPI:IPI00547757 IPI:IPI00900499 PIR:T05223 RefSeq:NP_567945.1 UniGene:At.2399 PDB:3UC4 PDB:3UDB PDB:3ZUT PDB:3ZUU PDBsum:3UC4 PDBsum:3UDB PDBsum:3ZUT PDBsum:3ZUU ProteinModelPortal:Q940H6 SMR:Q940H6 DIP:DIP-36705N IntAct:Q940H6 MINT:MINT-7260107 STRING:Q940H6 PRIDE:Q940H6

Tissue	Identification	# of reads (stress)	# of reads (control)	Fold Change	p-value	Description
						EnsemblPlants:AT4G33950.1 GeneID:829541 KEGG:ath:AT4G33950 TAIR:At4g33950 InParanoid:Q940H6 OMA:QYEEPEQ PhylomeDB:Q940H6 ProtClustDB:CLSN2917660 Genevestigator:Q940H6 Uniprot:Q940H6
Root	Isotig08115	0	39	-6.414	3.85E-11	TAIR|locus:2089423 - symbol:AT3G21360 species:3702 "Arabidopsis thaliana" [GO:0016491 "oxidoreductase activity" evidence=IEA] [GO:0055114 "oxidation-reduction process" evidence=IEA] [GO:0005634 "nucleus" evidence=IDA] InterPro:IPR003819 Pfam:PF02668 GO:GO:0005634 EMBL:CP002686 GenomeReviews:BA000014_GR GO:GO:0046872 GO:GO:0016491 EMBL:AP001305 UniGene:At.47366 UniGene:At.63567 EMBL:AK117772 IPI:IPI00537078 RefSeq:NP_188773.1 PDB:1Y0Z PDB:2Q4A PDBsum:1Y0Z PDBsum:2Q4A ProteinModelPortal:Q9LIG0 SMR:Q9LIG0 PRIDE:Q9LIG0 DNASU:821690 EnsemblPlants:AT3G21360.1 GeneID:821690 KEGG:ath:AT3G21360 TAIR:At3g21360 eggNOG:NOG13343 HOGENOM:HBG592040 InParanoid:Q9LIG0 OMA:NSMVAAY PhylomeDB:Q9LIG0 ProtClustDB:CLSN2685103 Genevestigator:Q9LIG0 GermOnline:AT3G21360 Uniprot:Q9LIG0
Root	Isotig08116	0	40	-6.451	2.24E-11	TAIR|locus:2089423 - symbol:AT3G21360 species:3702 "Arabidopsis thaliana" [GO:0016491 "oxidoreductase activity" evidence=IEA] [GO:0055114 "oxidation-reduction process" evidence=IEA] [GO:0005634 "nucleus" evidence=IDA] InterPro:IPR003819 Pfam:PF02668 GO:GO:0005634 EMBL:CP002686 GenomeReviews:BA000014_GR GO:GO:0046872 GO:GO:0016491 EMBL:AP001305 UniGene:At.47366 UniGene:At.63567 EMBL:AK117772 IPI:IPI00537078 RefSeq:NP_188773.1 PDB:1Y0Z PDB:2Q4A PDBsum:1Y0Z PDBsum:2Q4A ProteinModelPortal:Q9LIG0 SMR:Q9LIG0 PRIDE:Q9LIG0 DNASU:821690 EnsemblPlants:AT3G21360.1 GeneID:821690 KEGG:ath:AT3G21360 TAIR:At3g21360 eggNOG:NOG13343 HOGENOM:HBG592040 InParanoid:Q9LIG0 OMA:NSMVAAY PhylomeDB:Q9LIG0 ProtClustDB:CLSN2685103 Genevestigator:Q9LIG0 GermOnline:AT3G21360 Uniprot:Q9LIG0
Root	Isotig08119	52	16	1.571	3.49E-05	TAIR|locus:2013001 - symbol:AT1G71695 species:3702 "Arabidopsis thaliana" [GO:0004601 "peroxidase activity" evidence=IEA;ISS] [GO:0006979 "response to oxidative stress" evidence=IEA] [GO:0020037 "heme binding" evidence=IEA] [GO:0055114 "oxidation-reduction process" evidence=IEA] [GO:0005773 "vacuole" evidence=IDA] [GO:0005618 "cell wall" evidence=IDA] [GO:0009505 "plant-type cell wall" evidence=IDA] [GO:0016020 "membrane" evidence=IDA] [GO:0009506 "plasmodesma" evidence=IDA] InterPro:IPR000823 InterPro:IPR002016 InterPro:IPR010255 InterPro:IPR019793 InterPro:IPR019794 Pfam:PF00141 PRINTS:PR00458 PRINTS:PR00461 PROSITE:PS00435 PROSITE:PS00436 PROSITE:PS50873 EMBL:CP002684 GenomeReviews:CT485782_GR GO:GO:0009506 GO:GO:0005773 GO:GO:0005576 GO:GO:0016020 GO:GO:0020037 GO:GO:0009505 EMBL:AC012654 EMBL:AC016163 GO:GO:0004601 GO:GO:0042744 HOGENOM:HBG597790 SUPFAM:SSF48113 KO:K00430 EMBL:X98318 EMBL:X98773 EMBL:AF334732 EMBL:BT000715 EMBL:AY087964 IPI:IPI00538176 PIR:A96739 RefSeq:NP_177313.1 UniGene:At.67041 UniGene:At.94 ProteinModelPortal:Q96520 SMR:Q96520 STRING:Q96520 PeroxiBase:93 PRIDE:Q96520 EnsemblPlants:AT1G71695.1 GeneID:843498 KEGG:ath:AT1G71695 GeneFarm:1474 TAIR:At1g71695 eggNOG:NOG252730 InParanoid:Q96520 OMA:NCSARNT PhylomeDB:Q96520 ProtClustDB:CLSN2679267 ArrayExpress:Q96520 Genevestigator:Q96520 GermOnline:AT1G71695 Uniprot:Q96520
Root	Isotig08129	19	0	5.119	1.05E-05	TAIR|locus:2123782 - symbol:GAMMA-VPE "AT4G32940" species:3702 "Arabidopsis thaliana" [GO:0004197 "cysteine-type endopeptidase activity" evidence=ISS;IDA] [GO:0006508 "proteolysis" evidence=ISS] [GO:0000323 "lytic vacuole" evidence=IDA] [GO:0006624 "vacuolar protein processing" evidence=ISS] InterPro:IPR001096 Pfam:PF01650 PIRSF:PIRSF019663 PRINTS:PR00776 EMBL:CP002687 GenomeReviews:CT486007_GR GO:GO:0004197 GO:GO:0006508 EMBL:AL031804 EMBL:AL161582 eggNOG:COG5206 PANTHER:PTHR12000 KO:K01369 HOGENOM:HBG318720 GO:GO:0000323 ProtClustDB:CLSN2682340 EMBL:D61395 EMBL:AF370160 EMBL:AF424619 EMBL:AY059104 EMBL:AY133531 IPI:IPI00537536 PIR:T05302 RefSeq:NP_195020.1 UniGene:At.21567 UniGene:At.48929 UniGene:At.75206 ProteinModelPortal:Q39119 STRING:Q39119 MEROPS:C13.006 PRIDE:Q39119 EnsemblPlants:AT4G32940.1 GeneID:829431 KEGG:ath:AT4G32940 TAIR:At4g32940 InParanoid:Q39119 OMA:ICHAYQL PhylomeDB:Q39119 Genevestigator:Q39119 Uniprot:Q39119
Root	Isotig08130	19	0	5.119	1.05E-05	TAIR|locus:2087625 - symbol:DELTA-VPE "AT3G20210" species:3702 "Arabidopsis thaliana" [GO:0004197 "cysteine-type endopeptidase activity" evidence=ISS;IDA] [GO:0006508 "proteolysis" evidence=ISS] [GO:0006624 "vacuolar protein processing" evidence=ISS] [GO:0005615 "extracellular space" evidence=IDA] [GO:0010214 "seed coat development" evidence=IMP] [GO:0012501 "programmed cell death" evidence=IMP] InterPro:IPR001096 Pfam:PF01650 PIRSF:PIRSF019663 PRINTS:PR00776 EMBL:CP002686 GO:GO:0005615 GO:GO:0004197 GO:GO:0006508 EMBL:AP000383 GO:GO:0010214 GO:GO:0012501 PANTHER:PTHR12000 KO:K01369

Tissue	Identification	# of reads (stress)	# of reads (control)	Fold Change	p-value	Description
						ProtClustDB:CLSN2682340 UniGene:At.26916 EMBL:AY120765 EMBL:BT000949 EMBL:AF521661 EMBL:AB105106 IPI:IPI00543686 RefSeq:NP_188656.1 ProteinModelPortal:Q9LJX8 MEROPS:C13.A01 PRIDE:Q9LJX8 EnsemblPlants:AT3G20210.1 GeneID:821565 KEGG:ath:AT3G20210 TAIR:At3g20210 InParanoid:Q9LJX8 OMA:SWLEDSD PhylomeDB:Q9LJX8 ArrayExpress:Q9LJX8 Genevestigator:Q9LJX8 Uniprot:Q9LJX8
Root	Isotig08133	3	20	-2.866	9.17E-05	TAIR|locus:2173093 - symbol:AT5G51970 species:3702 "Arabidopsis thaliana" [GO:0008270 "zinc ion binding" evidence=IEA] [GO:0016491 "oxidoreductase activity" evidence=IEA] [GO:0055114 "oxidation-reduction process" evidence=IEA] [GO:0005829 "cytosol" evidence=IDA] [GO:0009506 "plasmodesma" evidence=IDA] InterPro:IPR002085 InterPro:IPR002328 InterPro:IPR013149 InterPro:IPR013154 InterPro:IPR016040 Pfam:PF00107 Pfam:PF08240 PROSITE:PS00059 GO:GO:0005829 GO:GO:0009506 EMBL:CP002688 GO:GO:0000166 Gene3D:G3DSA:3.40.50.720 GO:GO:0008270 GO:GO:0016491 InterPro:IPR011032 PANTHER:PTHR11695 SUPFAM:SSF50129 EMBL:AB015478 UniGene:At.29648 UniGene:At.9328 KO:K00008 OMA:FYKHNAN HSSP:O96496 EMBL:AF370161 EMBL:AY133848 EMBL:AK230367 IPI:IPI00526124 RefSeq:NP_200010.1 RefSeq:NP_974925.1 ProteinModelPortal:Q9FJ95 SMR:Q9FJ95 STRING:Q9FJ95 PRIDE:Q9FJ95 EnsemblPlants:AT5G51970.1 EnsemblPlants:AT5G51970.2 GeneID:835272 KEGG:ath:AT5G51970 TAIR:At5g51970 InParanoid:Q9FJ95 PhylomeDB:Q9FJ95 ProtClustDB:PLN02702 Genevestigator:Q9FJ95 Uniprot:Q9FJ95
Root	Isotig08134	2	28	-3.936	1.29E-07	TAIR|locus:2173093 - symbol:AT5G51970 species:3702 "Arabidopsis thaliana" [GO:0008270 "zinc ion binding" evidence=IEA] [GO:0016491 "oxidoreductase activity" evidence=IEA] [GO:0055114 "oxidation-reduction process" evidence=IEA] [GO:0005829 "cytosol" evidence=IDA] [GO:0009506 "plasmodesma" evidence=IDA] InterPro:IPR002085 InterPro:IPR002328 InterPro:IPR013149 InterPro:IPR013154 InterPro:IPR016040 Pfam:PF00107 Pfam:PF08240 PROSITE:PS00059 GO:GO:0005829 GO:GO:0009506 EMBL:CP002688 GO:GO:0000166 Gene3D:G3DSA:3.40.50.720 GO:GO:0008270 GO:GO:0016491 InterPro:IPR011032 PANTHER:PTHR11695 SUPFAM:SSF50129 EMBL:AB015478 UniGene:At.29648 UniGene:At.9328 KO:K00008 OMA:FYKHNAN HSSP:O96496 EMBL:AF370161 EMBL:AY133848 EMBL:AK230367 IPI:IPI00526124 RefSeq:NP_200010.1 RefSeq:NP_974925.1 ProteinModelPortal:Q9FJ95 SMR:Q9FJ95 STRING:Q9FJ95 PRIDE:Q9FJ95 EnsemblPlants:AT5G51970.1 EnsemblPlants:AT5G51970.2 GeneID:835272 KEGG:ath:AT5G51970 TAIR:At5g51970 InParanoid:Q9FJ95 PhylomeDB:Q9FJ95 ProtClustDB:PLN02702 Genevestigator:Q9FJ95 Uniprot:Q9FJ95
Root	Isotig08135	2	18	-3.299	7.42E-05	TAIR|locus:2103172 - symbol:ASD1 "AT3G10740" species:3702 "Arabidopsis thaliana" [GO:0016798 "hydrolase activity, acting on glycosyl bonds" evidence=ISS] [GO:0009044 "xylan 1,4-beta-xylosidase activity" evidence=IDA] [GO:0045493 "xylan catabolic process" evidence=IDA] [GO:0046556 "alpha-N-arabinofuranosidase activity" evidence=IDA] [GO:0005773 "vacuole" evidence=IDA] [GO:0009505 "plant-type cell wall" evidence=IDA] [GO:0048046 "apoplast" evidence=IDA] InterPro:IPR010720 Pfam:PF06964 SMART:SM00813 GO:GO:0048046 GO:GO:0005773 EMBL:CP002686 GenomeReviews:BA000014_GR GO:GO:0045493 InterPro:IPR017853 SUPFAM:SSF51445 GO:GO:0005578 EMBL:AC011708 CAZy:GH51 GO:GO:0046556 GO:GO:0046373 eggNOG:COG3534 GO:GO:0009505 EMBL:AY243509 EMBL:AF372949 EMBL:AY143944 EMBL:AK222175 EMBL:AK220766 IPI:IPI00526411 RefSeq:NP_187685.1 UniGene:At.20271 ProteinModelPortal:Q9SG80 SMR:Q9SG80 STRING:Q9SG80 PRIDE:Q9SG80 EnsemblPlants:AT3G10740.1 GeneID:820243 KEGG:ath:AT3G10740 TAIR:At3g10740 InParanoid:Q9SG80 OMA:LEYLGIG PhylomeDB:Q9SG80 ProtClustDB:CLSN2684036 Genevestigator:Q9SG80 GO:GO:0009044 Uniprot:Q9SG80
Root	Isotig08151	2	15	-3.036	0.000493759	TAIR|locus:2168988 - symbol:TDT "AT5G47560" species:3702 "Arabidopsis thaliana" [GO:0006814 "sodium ion transport" evidence=ISS] [GO:0006835 "dicarboxylic acid transport" evidence=ISS] [GO:0017153 "sodium:dicarboxylate symporter activity" evidence=ISS] [GO:0005773 "vacuole" evidence=IDA] [GO:0015140 "malate transmembrane transporter activity" evidence=IMP;IDA] [GO:0015743 "malate transport" evidence=TAS] [GO:0051453 "regulation of intracellular pH" evidence=TAS] InterPro:IPR001898 Pfam:PF00939 GO:GO:0016021 GO:GO:0005774 EMBL:CP002688 GenomeReviews:BA000015_GR EMBL:AB025628 GO:GO:0006814 GO:GO:0015140 GO:GO:0051453 eggNOG:COG0471 TIGRFAMs:TIGR00785 KO:K14445 HOGENOM:HBG616159 EMBL:AJ223445 EMBL:AB043024 EMBL:AY072145 EMBL:AY133792 EMBL:AY084408 IPI:IPI00534224 RefSeq:NP_199567.1 UniGene:At.21038 UniGene:At.68515 UniGene:At.71618 STRING:Q8LG88 TCDB:2.A.47.1.6 PRIDE:Q8LG88 EnsemblPlants:AT5G47560.1 GeneID:834806 KEGG:ath:AT5G47560 GeneFarm:4980 TAIR:At5g47560 InParanoid:Q8LG88 OMA:TECMPLA PhylomeDB:Q8LG88

Tissue	Identification	# of reads (stress)	# of reads (control)	Fold Change	p-value	Description
						ProtClustDB:CLSN2686388 ArrayExpress:Q9MAW4 Genevestigator:Q8LG88 GermOnline:AT5G47560 Uniprot:Q8LG88
Root	Isotig08152	2	16	-3.129	0.000262867	TAIR|locus:2168988 - symbol:TDT "AT5G47560" species:3702 "Arabidopsis thaliana" [GO:0006814 "sodium ion transport" evidence=ISS] [GO:0006835 "dicarboxylic acid transport" evidence=ISS] [GO:0017153 "sodium:dicarboxylate symporter activity" evidence=ISS] [GO:0005773 "vacuole" evidence=IDA] [GO:0015140 "malate transmembrane transporter activity" evidence=IMP;IDA] [GO:0015743 "malate transport" evidence=TAS] [GO:0051453 "regulation of intracellular pH" evidence=TAS] InterPro:IPR001898 Pfam:PF00939 GO:GO:0016021 GO:GO:0005774 EMBL:CP002688 GenomeReviews:BA000015_GR EMBL:AB025628 GO:GO:0006814 GO:GO:0015140 GO:GO:0051453 eggNOG:COG0471 TIGRFAMs:TIGR00785 KO:K14445 HOGENOM:HBG616159 EMBL:AJ223445 EMBL:AB043024 EMBL:AY072145 EMBL:AY133792 EMBL:AY084408 IPI:IPI00534224 RefSeq:NP_199567.1 UniGene:At.21038 UniGene:At.68515 UniGene:At.71618 STRING:Q8LG88 TCDB:2.A.47.1.6 PRIDE:Q8LG88 EnsemblPlants:AT5G47560.1 GeneID:834806 KEGG:ath:AT5G47560 GeneFarm:4980 TAIR:At5g47560 InParanoid:Q8LG88 OMA:TECMPLA PhylomeDB:Q8LG88 ProtClustDB:CLSN2686388 ArrayExpress:Q9MAW4 Genevestigator:Q8LG88 GermOnline:AT5G47560 Uniprot:Q8LG88
Root	Isotig08173	39	1	5.156	2.63E-10	TAIR|locus:2167418 - symbol:AT5G24130 "AT5G24130" species:3702 "Arabidopsis thaliana" [GO:0003674 "molecular_function" evidence=ND] [GO:0008150 "biological_process" evidence=ND] EMBL:CP002688 GenomeReviews:BA000015_GR EMBL:AB010696 EMBL:BT005307 EMBL:AK118424 IPI:IPI00517115 RefSeq:NP_197801.1 UniGene:At.30976 IntAct:Q9FL61 PRIDE:Q9FL61 EnsemblPlants:AT5G24130.1 GeneID:832478 KEGG:ath:AT5G24130 TAIR:At5g24130 eggNOG:NOG265800 HOGENOM:HBG597859 InParanoid:Q9FL61 OMA:AREIRTY PhylomeDB:Q9FL61 ProtClustDB:CLSN2686316 Genevestigator:Q9FL61 Uniprot:Q9FL61
Root	Isotig08174	39	0	6.156	2.24E-10	TAIR|locus:2167418 - symbol:AT5G24130 "AT5G24130" species:3702 "Arabidopsis thaliana" [GO:0003674 "molecular_function" evidence=ND] [GO:0008150 "biological_process" evidence=ND] EMBL:CP002688 GenomeReviews:BA000015_GR EMBL:AB010696 EMBL:BT005307 EMBL:AK118424 IPI:IPI00517115 RefSeq:NP_197801.1 UniGene:At.30976 IntAct:Q9FL61 PRIDE:Q9FL61 EnsemblPlants:AT5G24130.1 GeneID:832478 KEGG:ath:AT5G24130 TAIR:At5g24130 eggNOG:NOG265800 HOGENOM:HBG597859 InParanoid:Q9FL61 OMA:AREIRTY PhylomeDB:Q9FL61 ProtClustDB:CLSN2686316 Genevestigator:Q9FL61 Uniprot:Q9FL61
Root	Isotig08189	1	14	-3.936	0.000188601	TAIR|locus:2183730 - symbol:AT5G10770 "AT5G10770" species:3702 "Arabidopsis thaliana" [GO:0003677 "DNA binding" evidence=ISS] [GO:0004190 "aspartic-type endopeptidase activity" evidence=IEA] [GO:0006508 "proteolysis" evidence=IEA] InterPro:IPR001461 InterPro:IPR009007 Pfam:PF00026 PRINTS:PR00792 EMBL:CP002688 GO:GO:0006508 GO:GO:0004190 InterPro:IPR021109 SUPFAM:SSF50630 Gene3D:G3DSA:2.40.70.10 PANTHER:PTHR13683 HSSP:P00799 EMBL:AY075656 EMBL:BT001004 IPI:IPI00543783 RefSeq:NP_196638.2 UniGene:At.32336 ProteinModelPortal:Q8S9J6 MEROPS:A01.A15 PRIDE:Q8S9J6 EnsemblPlants:AT5G10770.1 GeneID:830944 KEGG:ath:AT5G10770 TAIR:At5g10770 InParanoid:Q8S9J6 OMA:LEVVYDG PhylomeDB:Q8S9J6 ProtClustDB:CLSN2686424 Genevestigator:Q8S9J6 Uniprot:Q8S9J6
Root	Isotig08213	8	38	-2.377	1.13E-06	UNIPROTKB|Q8I4X0 - symbol:PFL2215w "Actin-1" species:36329 "Plasmodium falciparum 3D7" [GO:0005200 "structural constituent of cytoskeleton" evidence=ISS] [GO:0005884 "actin filament" evidence=ISS] [GO:0007010 "cytoskeleton organization" evidence=ISS] [GO:0070359 "actin polymerization-dependent cell motility involved in migration of symbiont in host" evidence=ISS] InterPro:IPR004001 PROSITE:PS00406 PROSITE:PS00432 InterPro:IPR004000 GO:GO:0005524 GO:GO:0005737 GO:GO:0005200 GO:GO:0005884 HOGENOM:HBG559892 PANTHER:PTHR11937 Pfam:PF00022 PRINTS:PR00190 SMART:SM00268 KO:K10355 OMA:PRHQVRQ InterPro:IPR020902 PROSITE:PS01132 EMBL:AE014188 PIR:S12628 RefSeq:XP_001350847.1 ProteinModelPortal:Q8I4X0 SMR:Q8I4X0 IntAct:Q8I4X0 MINT:MINT-1556031 EnsemblProtists:PFL2215w:mRNA GeneID:811495 KEGG:pfa:PFL2215w EuPathDB:EupathDB:PFL2215w ProtClustDB:PTZ00004 GO:GO:0070359 Uniprot:Q8I4X0
Root	Isotig08214	8	38	-2.377	1.13E-06	TAIR|locus:2099302 - symbol:ACT11 "AT3G12110" species:3702 "Arabidopsis thaliana" [GO:0005200 "structural constituent of cytoskeleton" evidence=ISS] [GO:0030036 "actin cytoskeleton organization" evidence=TAS] [GO:0005739 "mitochondrion" evidence=IDA] [GO:0005886 "plasma membrane" evidence=IDA] [GO:0009570 "chloroplast stroma" evidence=IDA] [GO:0009941 "chloroplast envelope" evidence=IDA] [GO:0009506

Tissue	Identification	# of reads (stress)	# of reads (control)	Fold Change	p-value	Description
						"plasmodesma" evidence=IDA] [GO:0005856 "cytoskeleton" evidence=ISS] InterPro:IPR004001 PROSITE:PS00406 PROSITE:PS00432 InterPro:IPR004000 GO:GO:0005739 GO:GO:0005886 GO:GO:0009506 GO:GO:0005524 GO:GO:0009570 EMBL:CP002686 GenomeReviews:BA000014_GR GO:GO:0005856 EMBL:AC069473 GO:GO:0009941 eggNOG:COG5277 HOGENOM:HBG559892 PANTHER:PTHR11937 Pfam:PF00022 PRINTS:PR00190 SMART:SM00268 KO:K10355 InterPro:IPR020902 PROSITE:PS01132 EMBL:U27981 EMBL:AP002063 EMBL:BT005593 EMBL:AY087740 IPI:IPI00538017 PIR:S68109 RefSeq:NP_187818.1 UniGene:At.28138 ProteinModelPortal:P53496 SMR:P53496 IntAct:P53496 STRING:P53496 PRIDE:P53496 EnsemblPlants:AT3G12110.1 GeneID:820385 KEGG:ath:AT3G12110 TAIR:At3g12110 InParanoid:P53496 OMA:GERFRCP PhylomeDB:P53496 ProtClustDB:CLSN2682469 ArrayExpress:P53496 Genevestigator:P53496 GermOnline:AT3G12110 Uniprot:P53496
Root	Isotig08223	10	29	-1.665	0.000753212	TAIR|locus:2076904 - symbol:GRF7 "AT3G02520" species:3702 "Arabidopsis thaliana" [GO:0005635 "nuclear envelope" evidence=IDA] [GO:0005737 "cytoplasm" evidence=IDA] [GO:0005886 "plasma membrane" evidence=IDA] [GO:0045309 "protein phosphorylated amino acid binding" evidence=TAS] [GO:0009507 "chloroplast" evidence=IDA] [GO:0005829 "cytosol" evidence=RCA] [GO:0009506 "plasmodesma" evidence=IDA] [GO:0005794 "Golgi apparatus" evidence=IDA] [GO:0009570 "chloroplast stroma" evidence=IDA] InterPro:IPR000308 PIRSF:PIRSF000868 PRINTS:PR00305 HOGENOM:HBG611720 GO:GO:0005886 GO:GO:0009506 GO:GO:0019904 InterPro:IPR023409 InterPro:IPR023410 Gene3D:G3DSA:1.20.190.20 PANTHER:PTHR18860 Pfam:PF00244 SMART:SM00101 SUPFAM:SSF48445 PROSITE:PS00796 PROSITE:PS00797 GO:GO:0009507 ProtClustDB:CLSN2688003 EMBL:U60445 EMBL:AF145299 EMBL:AC021640 EMBL:CP002686 EMBL:AY065274 EMBL:AY096526 EMBL:AY087723 IPI:IPI00536399 RefSeq:NP_566174.1 UniGene:At.21863 ProteinModelPortal:Q96300 SMR:Q96300 IntAct:Q96300 STRING:Q96300 PRIDE:Q96300 EnsemblPlants:AT3G02520.1 GeneID:821060 GenomeReviews:BA000014_GR KEGG:ath:AT3G02520 TAIR:At3g02520 InParanoid:Q96300 OMA:KVCQDVL PhylomeDB:Q96300 ArrayExpress:Q96300 Genevestigator:Q96300 GermOnline:AT3G02520 Uniprot:Q96300
Root	Isotig08224	8	28	-1.936	0.000239276	TAIR|locus:2076904 - symbol:GRF7 "AT3G02520" species:3702 "Arabidopsis thaliana" [GO:0005635 "nuclear envelope" evidence=IDA] [GO:0005737 "cytoplasm" evidence=IDA] [GO:0005886 "plasma membrane" evidence=IDA] [GO:0045309 "protein phosphorylated amino acid binding" evidence=TAS] [GO:0009507 "chloroplast" evidence=IDA] [GO:0005829 "cytosol" evidence=RCA] [GO:0009506 "plasmodesma" evidence=IDA] [GO:0005794 "Golgi apparatus" evidence=IDA] [GO:0009570 "chloroplast stroma" evidence=IDA] InterPro:IPR000308 PIRSF:PIRSF000868 PRINTS:PR00305 HOGENOM:HBG611720 GO:GO:0005886 GO:GO:0009506 GO:GO:0019904 InterPro:IPR023409 InterPro:IPR023410 Gene3D:G3DSA:1.20.190.20 PANTHER:PTHR18860 Pfam:PF00244 SMART:SM00101 SUPFAM:SSF48445 PROSITE:PS00796 PROSITE:PS00797 GO:GO:0009507 ProtClustDB:CLSN2688003 EMBL:U60445 EMBL:AF145299 EMBL:AC021640 EMBL:CP002686 EMBL:AY065274 EMBL:AY096526 EMBL:AY087723 IPI:IPI00536399 RefSeq:NP_566174.1 UniGene:At.21863 ProteinModelPortal:Q96300 SMR:Q96300 IntAct:Q96300 STRING:Q96300 PRIDE:Q96300 EnsemblPlants:AT3G02520.1 GeneID:821060 GenomeReviews:BA000014_GR KEGG:ath:AT3G02520 TAIR:At3g02520 InParanoid:Q96300 OMA:KVCQDVL PhylomeDB:Q96300 ArrayExpress:Q96300 Genevestigator:Q96300 GermOnline:AT3G02520 Uniprot:Q96300
Root	Isotig08225	11	45	-2.161	5.75E-07	TAIR|locus:2013001 - symbol:AT1G71695 species:3702 "Arabidopsis thaliana" [GO:0004601 "peroxidase activity" evidence=IEA;ISS] [GO:0006979 "response to oxidative stress" evidence=IEA] [GO:0020037 "heme binding" evidence=IEA] [GO:0055114 "oxidation-reduction process" evidence=IEA] [GO:0005773 "vacuole" evidence=IDA] [GO:0005618 "cell wall" evidence=IDA] [GO:0009505 "plant-type cell wall" evidence=IDA] [GO:0016020 "membrane" evidence=IDA] [GO:0009506 "plasmodesma" evidence=IDA] InterPro:IPR000823 InterPro:IPR002016 InterPro:IPR010255 InterPro:IPR019793 InterPro:IPR019794 Pfam:PF00141 PRINTS:PR00458 PRINTS:PR00461 PROSITE:PS00435 PROSITE:PS00436 PROSITE:PS50873 EMBL:CP002684 GenomeReviews:CT485782_GR GO:GO:0009506 GO:GO:0005773 GO:GO:0005576 GO:GO:0016020 GO:GO:0020037 GO:GO:0009505 EMBL:AC012654 EMBL:AC016163 GO:GO:0004601 GO:GO:0042744 HOGENOM:HBG597790 SUPFAM:SSF48113 KO:K00430 EMBL:X98318 EMBL:X98773 EMBL:AF334732 EMBL:BT000715 EMBL:AY087964 IPI:IPI00538176 PIR:A96739 RefSeq:NP_177313.1 UniGene:At.67041 UniGene:At.94 ProteinModelPortal:Q96520 SMR:Q96520 STRING:Q96520 PeroxiBase:93 PRIDE:Q96520 EnsemblPlants:AT1G71695.1 GeneID:843498 KEGG:ath:AT1G71695 GeneFarm:1474 TAIR:At1g71695

Tissue	Identification	# of reads (stress)	# of reads (control)	Fold Change	p-value	Description
						eggNOG:NOG252730 InParanoid:Q96520 OMA:NCSARNT PhylomeDB:Q96520 ProtClustDB:CLSN2679267 ArrayExpress:Q96520 Genevestigator:Q96520 GermOnline:AT1G71695 Uniprot:Q96520
Root	Isotig08226	11	45	-2.161	5.75E-07	TAIR|locus:2013001 - symbol:AT1G71695 species:3702 "Arabidopsis thaliana" [GO:0004601 "peroxidase activity" evidence=IEA;ISS] [GO:0006979 "response to oxidative stress" evidence=IEA] [GO:0020037 "heme binding" evidence=IEA] [GO:0055114 "oxidation-reduction process" evidence=IEA] [GO:0005773 "vacuole" evidence=IDA] [GO:0005618 "cell wall" evidence=IDA] [GO:0009505 "plant-type cell wall" evidence=IDA] [GO:0016020 "membrane" evidence=IDA] [GO:0009506 "plasmodesma" evidence=IDA] InterPro:IPR000823 InterPro:IPR002016 InterPro:IPR010255 InterPro:IPR019793 InterPro:IPR019794 Pfam:PF00141 PRINTS:PR00458 PRINTS:PR00461 PROSITE:PS00435 PROSITE:PS00436 PROSITE:PS50873 EMBL:CP002684 GenomeReviews:CT485782_GR GO:GO:0009506 GO:GO:0005773 GO:GO:0005576 GO:GO:0016020 GO:GO:0020037 GO:GO:0009505 EMBL:AC012654 EMBL:AC016163 GO:GO:0004601 GO:GO:0042744 HOGENOM:HBG597790 SUPFAM:SSF48113 KO:K00430 EMBL:X98318 EMBL:X98773 EMBL:AF334732 EMBL:BT000715 EMBL:AY087964 IPI:IPI00538176 PIR:A96739 RefSeq:NP_177313.1 UniGene:At.67041 UniGene:At.94 ProteinModelPortal:Q96520 SMR:Q96520 STRING:Q96520 PeroxiBase:93 PRIDE:Q96520 EnsemblPlants:AT1G71695.1 GeneID:843498 KEGG:ath:AT1G71695 GeneFarm:1474 TAIR:At1g71695 eggNOG:NOG252730 InParanoid:Q96520 OMA:NCSARNT PhylomeDB:Q96520 ProtClustDB:CLSN2679267 ArrayExpress:Q96520 Genevestigator:Q96520 GermOnline:AT1G71695 Uniprot:Q96520
Root	Isotig08239	53	14	1.792	4.53E-06	TAIR|locus:2019574 - symbol:AT1G67280 species:3702 "Arabidopsis thaliana" [GO:0004462 "lactoylglutathione lyase activity" evidence=IEA;ISS] [GO:0005975 "carbohydrate metabolic process" evidence=ISS] [GO:0046872 "metal ion binding" evidence=IEA] [GO:0031977 "thylakoid lumen" evidence=IDA] [GO:0009570 "chloroplast stroma" evidence=IDA] [GO:0009579 "thylakoid" evidence=IDA] [GO:0009409 "response to cold" evidence=IEP] [GO:0010319 "stromule" evidence=IDA] [GO:0009507 "chloroplast" evidence=IDA] InterPro:IPR004361 InterPro:IPR018146 PROSITE:PS00934 PROSITE:PS00935 Pfam:PF00903 EMBL:CP002684 GenomeReviews:CT485782_GR GO:GO:0009570 GO:GO:0046872 GO:GO:0010319 GO:GO:0009409 GO:GO:0031977 eggNOG:COG0346 InterPro:IPR004360 EMBL:AC002130 GO:GO:0004462 EMBL:AF419551 EMBL:AY079102 EMBL:AY085148 IPI:IPI00544224 IPI:IPI00846167 PIR:E96696 RefSeq:NP_001077783.1 RefSeq:NP_176896.1 UniGene:At.22738 HSSP:Q59384 ProteinModelPortal:Q8W593 SMR:Q8W593 STRING:Q8W593 PRIDE:Q8W593 EnsemblPlants:AT1G67280.1 GeneID:843048 KEGG:ath:AT1G67280 TAIR:At1g67280 InParanoid:Q8W593 KO:K01759 OMA:GPESSHF PhylomeDB:Q8W593 ProtClustDB:PLN02300 Genevestigator:Q8W593 TIGRFAMs:TIGR00068 Uniprot:Q8W593
Root	Isotig08240	71	16	2.021	8.76E-09	TAIR|locus:2019574 - symbol:AT1G67280 species:3702 "Arabidopsis thaliana" [GO:0004462 "lactoylglutathione lyase activity" evidence=IEA;ISS] [GO:0005975 "carbohydrate metabolic process" evidence=ISS] [GO:0046872 "metal ion binding" evidence=IEA] [GO:0031977 "thylakoid lumen" evidence=IDA] [GO:0009570 "chloroplast stroma" evidence=IDA] [GO:0009579 "thylakoid" evidence=IDA] [GO:0009409 "response to cold" evidence=IEP] [GO:0010319 "stromule" evidence=IDA] [GO:0009507 "chloroplast" evidence=IDA] InterPro:IPR004361 InterPro:IPR018146 PROSITE:PS00934 PROSITE:PS00935 Pfam:PF00903 EMBL:CP002684 GenomeReviews:CT485782_GR GO:GO:0009570 GO:GO:0046872 GO:GO:0010319 GO:GO:0009409 GO:GO:0031977 eggNOG:COG0346 InterPro:IPR004360 EMBL:AC002130 GO:GO:0004462 EMBL:AF419551 EMBL:AY079102 EMBL:AY085148 IPI:IPI00544224 IPI:IPI00846167 PIR:E96696 RefSeq:NP_001077783.1 RefSeq:NP_176896.1 UniGene:At.22738 HSSP:Q59384 ProteinModelPortal:Q8W593 SMR:Q8W593 STRING:Q8W593 PRIDE:Q8W593 EnsemblPlants:AT1G67280.1 GeneID:843048 KEGG:ath:AT1G67280 TAIR:At1g67280 InParanoid:Q8W593 KO:K01759 OMA:GPESSHF PhylomeDB:Q8W593 ProtClustDB:PLN02300 Genevestigator:Q8W593 TIGRFAMs:TIGR00068 Uniprot:Q8W593
Root	Isotig08241	12	33	-1.588	0.000511326	TAIR|locus:2147142 - symbol:JAZ12 "AT5G20900" species:3702 "Arabidopsis thaliana" [GO:0008150 "biological_process" evidence=ND] [GO:0005515 "protein binding" evidence=IPI] InterPro:IPR018467 Pfam:PF09425 GO:GO:0005634 EMBL:CP002688 GenomeReviews:BA000015_GR GO:GO:0005515 GO:GO:0006952 GO:GO:0006355 GO:GO:0006351 EMBL:AF296834 UniGene:At.1134 UniGene:At.68499 InterPro:IPR010399 Pfam:PF06200 SMART:SM00979 PROSITE:PS51320 KO:K13464 EMBL:AF360184 EMBL:AY051013 IPI:IPI00543677 RefSeq:NP_197590.1 DIP:DIP-53279N IntAct:Q9C5K8 STRING:Q9C5K8 PRIDE:Q9C5K8 EnsemblPlants:AT5G20900.1 GeneID:832214 KEGG:ath:AT5G20900 TAIR:At5g20900 eggNOG:NOG316310 InParanoid:Q9C5K8 OMA:PALNRAP PhylomeDB:Q9C5K8 ProtClustDB:CLSN2916811 ArrayExpress:Q9C5K8 Genevestigator:Q9C5K8 Uniprot:Q9C5K8

Tissue	Identification	# of reads (stress)	# of reads (control)	Fold Change	p-value	Description
Root	Isotig08242	12	33	-1.588	0.000511326	TAIR|locus:2147142 - symbol:JAZ12 "AT5G20900" species:3702 "Arabidopsis thaliana" [GO:0008150 "biological_process" evidence=ND] [GO:0005515 "protein binding" evidence=IPI] InterPro:IPR018467 Pfam:PF09425 GO:GO:0005634 EMBL:CP002688 GenomeReviews:BA000015_GR GO:GO:0005515 GO:GO:0006952 GO:GO:0006355 GO:GO:0006351 EMBL:AF296834 UniGene:At.1134 UniGene:At.68499 InterPro:IPR010399 Pfam:PF06200 SMART:SM00979 PROSITE:PS51320 KO:K13464 EMBL:AF360184 EMBL:AY051013 IPI:IPI00543677 RefSeq:NP_197590.1 DIP:DIP-53279N IntAct:Q9C5K8 STRING:Q9C5K8 PRIDE:Q9C5K8 EnsemblPlants:AT5G20900.1 GeneID:832214 KEGG:ath:AT5G20900 TAIR:At5g20900 eggNOG:NOG316310 InParanoid:Q9C5K8 OMA:PALNRAP PhylomeDB:Q9C5K8 ProtClustDB:CLSN2916811 ArrayExpress:Q9C5K8 Genevestigator:Q9C5K8 Uniprot:Q9C5K8
Root	Isotig08253	29	5	2.407	4.20E-05	TAIR|locus:2146178 - symbol:AT5G18400 "AT5G18400" species:3702 "Arabidopsis thaliana" [GO:0008150 "biological_process" evidence=ND] [GO:0005515 "protein binding" evidence=IPI] GO:GO:0005737 EMBL:CP002688 GenomeReviews:BA000015_GR GO:GO:0006915 InterPro:IPR007785 PANTHER:PTHR13273 Pfam:PF05093 HOGENOM:HBG316506 eggNOG:COG5636 EMBL:AC051626 EMBL:AY080855 EMBL:AY123986 EMBL:BT000943 EMBL:BT001035 EMBL:AY088356 IPI:IPI00544195 RefSeq:NP_001078602.1 RefSeq:NP_568363.1 RefSeq:NP_850844.1 UniGene:At.31429 ProteinModelPortal:Q8L7Z3 SMR:Q8L7Z3 IntAct:Q8L7Z3 STRING:Q8L7Z3 PRIDE:Q8L7Z3 EnsemblPlants:AT5G18400.2 EnsemblPlants:AT5G18400.3 GeneID:831958 KEGG:ath:AT5G18400 TAIR:At5g18400 InParanoid:Q8L7Z3 OMA:CGTCPYK PhylomeDB:Q8L7Z3 ProtClustDB:CLSN2689770 Genevestigator:Q8L7Z3 Uniprot:Q8L7Z3
Root	Isotig08254	29	5	2.407	4.20E-05	TAIR|locus:2146178 - symbol:AT5G18400 "AT5G18400" species:3702 "Arabidopsis thaliana" [GO:0008150 "biological_process" evidence=ND] [GO:0005515 "protein binding" evidence=IPI] GO:GO:0005737 EMBL:CP002688 GenomeReviews:BA000015_GR GO:GO:0006915 InterPro:IPR007785 PANTHER:PTHR13273 Pfam:PF05093 HOGENOM:HBG316506 eggNOG:COG5636 EMBL:AC051626 EMBL:AY080855 EMBL:AY123986 EMBL:BT000943 EMBL:BT001035 EMBL:AY088356 IPI:IPI00544195 RefSeq:NP_001078602.1 RefSeq:NP_568363.1 RefSeq:NP_850844.1 UniGene:At.31429 ProteinModelPortal:Q8L7Z3 SMR:Q8L7Z3 IntAct:Q8L7Z3 STRING:Q8L7Z3 PRIDE:Q8L7Z3 EnsemblPlants:AT5G18400.2 EnsemblPlants:AT5G18400.3 GeneID:831958 KEGG:ath:AT5G18400 TAIR:At5g18400 InParanoid:Q8L7Z3 OMA:CGTCPYK PhylomeDB:Q8L7Z3 ProtClustDB:CLSN2689770 Genevestigator:Q8L7Z3 Uniprot:Q8L7Z3
Root	Isotig08291	0	31	-6.083	3.24E-09	TAIR|locus:2013728 - symbol:AT1G29380 "AT1G29380" species:3702 "Arabidopsis thaliana" [GO:0003674 "molecular_function" evidence=ND] [GO:0008150 "biological_process" evidence=ND] EMBL:CP002684 GenomeReviews:CT485782_GR CAZy:CBM43 InterPro:IPR012946 Pfam:PF07983 SMART:SM00768 EMBL:AY648309 EMBL:AY773818 EMBL:AK221467 IPI:IPI00528008 RefSeq:NP_174231.2 UniGene:At.50785 UniGene:At.68810 ProteinModelPortal:Q6DST9 SMR:Q6DST9 STRING:Q6DST9 EnsemblPlants:AT1G29380.1 GeneID:839813 KEGG:ath:AT1G29380 TAIR:At1g29380 eggNOG:NOG246571 HOGENOM:HBG744505 InParanoid:Q6DST9 OMA:WCIAKAN PhylomeDB:Q6DST9 ProtClustDB:CLSN2918796 Genevestigator:Q6DST9 Uniprot:Q6DST9
Root	Isotig08292	0	14	-4.936	8.24E-05	TAIR|locus:2013728 - symbol:AT1G29380 "AT1G29380" species:3702 "Arabidopsis thaliana" [GO:0003674 "molecular_function" evidence=ND] [GO:0008150 "biological_process" evidence=ND] EMBL:CP002684 GenomeReviews:CT485782_GR CAZy:CBM43 InterPro:IPR012946 Pfam:PF07983 SMART:SM00768 EMBL:AY648309 EMBL:AY773818 EMBL:AK221467 IPI:IPI00528008 RefSeq:NP_174231.2 UniGene:At.50785 UniGene:At.68810 ProteinModelPortal:Q6DST9 SMR:Q6DST9 STRING:Q6DST9 EnsemblPlants:AT1G29380.1 GeneID:839813 KEGG:ath:AT1G29380 TAIR:At1g29380 eggNOG:NOG246571 HOGENOM:HBG744505 InParanoid:Q6DST9 OMA:WCIAKAN PhylomeDB:Q6DST9 ProtClustDB:CLSN2918796 Genevestigator:Q6DST9 Uniprot:Q6DST9
Root	Isotig08293	0	18	-5.299	6.77E-06	TAIR|locus:2142105 - symbol:LRX3 "AT4G13340" species:3702 "Arabidopsis thaliana" [GO:0005515 "protein binding" evidence=IPI] [GO:0005199 "structural constituent of cell wall" evidence=ISS] [GO:0005618 "cell wall" evidence=IDA] [GO:0009505 "plant-type cell wall" evidence=IDA] [GO:0009506 "plasmodesma" evidence=IDA] PROSITE:PS51450 GO:GO:0009506 EMBL:CP002687 GenomeReviews:CT486007_GR GO:GO:0005576 GO:GO:0005515 eggNOG:COG4886 HOGENOM:HBG755340 GO:GO:0009505 GO:GO:0007047 EMBL:AL161536 InterPro:IPR013210 Pfam:PF08263 EMBL:AL049608 IPI:IPI00546699 PIR:T06291 RefSeq:NP_193070.1 UniGene:At.48854 ProteinModelPortal:Q9T0K5 SMR:Q9T0K5 PRIDE:Q9T0K5 EnsemblPlants:AT4G13340.1 GeneID:826964 KEGG:ath:AT4G13340 GeneFarm:1493 TAIR:At4g13340 InParanoid:Q9T0K5 OMA:VHYSSPP PhylomeDB:Q9T0K5 ProtClustDB:CLSN2916045 ArrayExpress:Q9T0K5 Genevestigator:Q9T0K5 Uniprot:Q9T0K5

Tissue	Identification	# of reads (stress)	# of reads (control)	Fold Change	p-value	Description
Root	Isotig08294	0	18	-5.299	6.77E-06	TAIR|locus:2142105 - symbol:LRX3 "AT4G13340" species:3702 "Arabidopsis thaliana" [GO:0005515 "protein binding" evidence=IPI] [GO:0005199 "structural constituent of cell wall" evidence=ISS] [GO:0005618 "cell wall" evidence=IDA] [GO:0009505 "plant-type cell wall" evidence=IDA] [GO:0009506 "plasmodesma" evidence=IDA] PROSITE:PS51450 GO:GO:0009506 EMBL:CP002687 GenomeReviews:CT486007_GR GO:GO:0005576 GO:GO:0005515 eggNOG:COG4886 HOGENOM:HBG755340 GO:GO:0009505 GO:GO:0007047 EMBL:AL161536 InterPro:IPR013210 Pfam:PF08263 EMBL:AL049608 IPI:IPI00546699 PIR:T06291 RefSeq:NP_193070.1 UniGene:At.48854 ProteinModelPortal:Q9T0K5 SMR:Q9T0K5 PRIDE:Q9T0K5 EnsemblPlants:AT4G13340.1 GeneID:826964 KEGG:ath:AT4G13340 GeneFarm:1493 TAIR:At4g13340 InParanoid:Q9T0K5 OMA:VHYSSPP PhylomeDB:Q9T0K5 ProtClustDB:CLSN2916045 ArrayExpress:Q9T0K5 Genevestigator:Q9T0K5 Uniprot:Q9T0K5
Root	Isotig08301	0	11	-4.588	0.000569522	TAIR|locus:2019863 - symbol:AT1G79620 "AT1G79620" species:3702 "Arabidopsis thaliana" [GO:0004674 "protein serine/threonine kinase activity" evidence=ISS] [GO:0005524 "ATP binding" evidence=ISS] [GO:0006468 "protein phosphorylation" evidence=IEA;ISS] [GO:0007169 "transmembrane receptor protein tyrosine kinase signaling pathway" evidence=ISS] [GO:0016301 "kinase activity" evidence=ISS] Pfam:PF00560 InterPro:IPR001611 InterPro:IPR000719 InterPro:IPR001245 InterPro:IPR008271 InterPro:IPR011009 InterPro:IPR017441 Pfam:PF07714 PROSITE:PS00107 PROSITE:PS00108 PROSITE:PS50011 GO:GO:0016021 EMBL:CP002684 GenomeReviews:CT485782_GR GO:GO:0005524 eggNOG:COG0515 GO:GO:0004872 SUPFAM:SSF56112 GO:GO:0004674 HSSP:P36897 InterPro:IPR013210 Pfam:PF08263 HOGENOM:HBG316990 EMBL:FJ708684 EMBL:AK117863 IPI:IPI00517320 RefSeq:NP_178080.2 UniGene:At.46098 ProteinModelPortal:Q8GY50 SMR:Q8GY50 PRIDE:Q8GY50 EnsemblPlants:AT1G79620.1 GeneID:844301 KEGG:ath:AT1G79620 TAIR:At1g79620 InParanoid:Q8GY50 OMA:YFIASPY PhylomeDB:Q8GY50 ProtClustDB:CLSN2680668 Genevestigator:Q8GY50 Uniprot:Q8GY50
Root	Isotig08302	0	11	-4.588	0.000569522	TAIR|locus:2019863 - symbol:AT1G79620 "AT1G79620" species:3702 "Arabidopsis thaliana" [GO:0004674 "protein serine/threonine kinase activity" evidence=ISS] [GO:0005524 "ATP binding" evidence=ISS] [GO:0006468 "protein phosphorylation" evidence=IEA;ISS] [GO:0007169 "transmembrane receptor protein tyrosine kinase signaling pathway" evidence=ISS] [GO:0016301 "kinase activity" evidence=ISS] Pfam:PF00560 InterPro:IPR001611 InterPro:IPR000719 InterPro:IPR001245 InterPro:IPR008271 InterPro:IPR011009 InterPro:IPR017441 Pfam:PF07714 PROSITE:PS00107 PROSITE:PS00108 PROSITE:PS50011 GO:GO:0016021 EMBL:CP002684 GenomeReviews:CT485782_GR GO:GO:0005524 eggNOG:COG0515 GO:GO:0004872 SUPFAM:SSF56112 GO:GO:0004674 HSSP:P36897 InterPro:IPR013210 Pfam:PF08263 HOGENOM:HBG316990 EMBL:FJ708684 EMBL:AK117863 IPI:IPI00517320 RefSeq:NP_178080.2 UniGene:At.46098 ProteinModelPortal:Q8GY50 SMR:Q8GY50 PRIDE:Q8GY50 EnsemblPlants:AT1G79620.1 GeneID:844301 KEGG:ath:AT1G79620 TAIR:At1g79620 InParanoid:Q8GY50 OMA:YFIASPY PhylomeDB:Q8GY50 ProtClustDB:CLSN2680668 Genevestigator:Q8GY50 Uniprot:Q8GY50
Root	Isotig08333	1	23	-4.653	5.84E-07	TAIR|locus:2122113 - symbol:XCP1 "xylem cysteine peptidase 1" species:3702 "Arabidopsis thaliana" [GO:0004197 "cysteine-type endopeptidase activity" evidence=IEA] [GO:0006508 "proteolysis" evidence=ISS] [GO:0008234 "cysteine-type peptidase activity" evidence=IEA;ISS] [GO:0000325 "plant-type vacuole" evidence=IDA] [GO:0005634 "nucleus" evidence=IDA] [GO:0010623 "developmental programmed cell death" evidence=IMP] InterPro:IPR000668 InterPro:IPR013128 Pfam:PF00112 PRINTS:PR00705 SMART:SM00645 InterPro:IPR000169 GO:GO:0005886 EMBL:CP002687 GenomeReviews:CT486007_GR GO:GO:0005634 eggNOG:COG4870 HOGENOM:HBG746690 GO:GO:0004197 GO:GO:0006508 InterPro:IPR013201 PANTHER:PTHR12411 Pfam:PF08246 SMART:SM00848 PROSITE:PS00640 PROSITE:PS00139 PROSITE:PS00639 GO:GO:0000325 EMBL:AL161587 EMBL:AL022604 GO:GO:0010623 KO:K01376 EMBL:AF191027 EMBL:AK117394 EMBL:BT005179 IPI:IPI00532220 PIR:T06122 RefSeq:NP_567983.1 UniGene:At.2280 UniGene:At.67622 ProteinModelPortal:O65493 SMR:O65493 STRING:O65493 MEROPS:C01.065 PRIDE:O65493 EnsemblPlants:AT4G35350.1 GeneID:829688 KEGG:ath:AT4G35350 GeneFarm:5033 TAIR:At4g35350 InParanoid:O65493 OMA:FEVFREN PhylomeDB:O65493 ProtClustDB:CLSN2689772 Genevestigator:O65493 Uniprot:O65493
Root	Isotig08334	1	23	-4.653	5.84E-07	TAIR|locus:2122113 - symbol:XCP1 "xylem cysteine peptidase 1" species:3702 "Arabidopsis thaliana" [GO:0004197 "cysteine-type endopeptidase activity" evidence=IEA] [GO:0006508 "proteolysis" evidence=ISS] [GO:0008234 "cysteine-type peptidase activity" evidence=IEA;ISS] [GO:0000325 "plant-type vacuole" evidence=IDA] [GO:0005634 "nucleus" evidence=IDA] [GO:0010623 "developmental programmed cell death" evidence=IMP] InterPro:IPR000668 InterPro:IPR013128 Pfam:PF00112 PRINTS:PR00705 SMART:SM00645

Tissue	Identification	# of reads (stress)	# of reads (control)	Fold Change	p-value	Description
						InterPro:IPR000169 GO:GO:0005886 EMBL:CP002687 GenomeReviews:CT486007_GR GO:GO:0005634 eggNOG:COG4870 HOGENOM:HBG746690 GO:GO:0004197 GO:GO:0006508 InterPro:IPR013201 PANTHER:PTHR12411 Pfam:PF08246 SMART:SM00848 PROSITE:PS00640 PROSITE:PS00139 PROSITE:PS00639 GO:GO:0000325 EMBL:AL161587 EMBL:AL022604 GO:GO:0010623 KO:K01376 EMBL:AF191027 EMBL:AK117394 EMBL:BT005179 IPI:IPI00532220 PIR:T06122 RefSeq:NP_567983.1 UniGene:At.2280 UniGene:At.67622 ProteinModelPortal:O65493 SMR:O65493 STRING:O65493 MEROPS:C01.065 PRIDE:O65493 EnsemblPlants:AT4G35350.1 GeneID:829688 KEGG:ath:AT4G35350 GeneFarm:5033 TAIR:At4g35350 InParanoid:O65493 OMA:FEVFREN PhylomeDB:O65493 ProtClustDB:CLSN2689772 Genevestigator:O65493 Uniprot:O65493
Root	Isotig08353	27	0	5.626	1.24E-07	TAIR|locus:2124784 - symbol:WIN2 "AT4G31750" species:3702 "Arabidopsis thaliana" [GO:0004722 "protein serine/threonine phosphatase activity" evidence=ISS;IDA] [GO:0005886 "plasma membrane" evidence=IDA] [GO:0006470 "protein dephosphorylation" evidence=IDA] [GO:0042742 "defense response to bacterium" evidence=IMP] [GO:0044419 "interspecies interaction between organisms" evidence=IPI] InterPro:IPR000222 InterPro:IPR001932 Pfam:PF00481 PROSITE:PS01032 SMART:SM00331 SMART:SM00332 GO:GO:0005886 EMBL:CP002687 GenomeReviews:CT486007_GR GO:GO:0042742 GO:GO:0044419 GO:GO:0006470 GO:GO:0004722 GO:GO:0046872 eggNOG:COG0631 Gene3D:G3DSA:3.60.40.10 SUPFAM:SSF81606 EMBL:AL161579 EMBL:AL031004 GO:GO:0008287 InterPro:IPR015655 PANTHER:PTHR13832 HSSP:P35813 HOGENOM:HBG747569 EMBL:EU214909 EMBL:AY080658 EMBL:AY133761 IPI:IPI00537408 PIR:T05095 RefSeq:NP_194903.2 UniGene:At.31716 UniGene:At.51039 ProteinModelPortal:Q8RXV3 SMR:Q8RXV3 PRIDE:Q8RXV3 EnsemblPlants:AT4G31750.1 GeneID:829303 KEGG:ath:AT4G31750 TAIR:At4g31750 InParanoid:Q8RXV3 OMA:DIMTFDL PhylomeDB:Q8RXV3 ProtClustDB:CLSN2680208 Genevestigator:Q8RXV3 Uniprot:Q8RXV3
Root	Isotig08354	30	0	5.778	2.46E-08	TAIR|locus:2124784 - symbol:WIN2 "AT4G31750" species:3702 "Arabidopsis thaliana" [GO:0004722 "protein serine/threonine phosphatase activity" evidence=ISS;IDA] [GO:0005886 "plasma membrane" evidence=IDA] [GO:0006470 "protein dephosphorylation" evidence=IDA] [GO:0042742 "defense response to bacterium" evidence=IMP] [GO:0044419 "interspecies interaction between organisms" evidence=IPI] InterPro:IPR000222 InterPro:IPR001932 Pfam:PF00481 PROSITE:PS01032 SMART:SM00331 SMART:SM00332 GO:GO:0005886 EMBL:CP002687 GenomeReviews:CT486007_GR GO:GO:0042742 GO:GO:0044419 GO:GO:0006470 GO:GO:0004722 GO:GO:0046872 eggNOG:COG0631 Gene3D:G3DSA:3.60.40.10 SUPFAM:SSF81606 EMBL:AL161579 EMBL:AL031004 GO:GO:0008287 InterPro:IPR015655 PANTHER:PTHR13832 HSSP:P35813 HOGENOM:HBG747569 EMBL:EU214909 EMBL:AY080658 EMBL:AY133761 IPI:IPI00537408 PIR:T05095 RefSeq:NP_194903.2 UniGene:At.31716 UniGene:At.51039 ProteinModelPortal:Q8RXV3 SMR:Q8RXV3 PRIDE:Q8RXV3 EnsemblPlants:AT4G31750.1 GeneID:829303 KEGG:ath:AT4G31750 TAIR:At4g31750 InParanoid:Q8RXV3 OMA:DIMTFDL PhylomeDB:Q8RXV3 ProtClustDB:CLSN2680208 Genevestigator:Q8RXV3 Uniprot:Q8RXV3
Root	Isotig08367	1	13	-3.829	0.000362856	TAIR|locus:2147117 - symbol:AT5G20950 species:3702 "Arabidopsis thaliana" [GO:0004553 "hydrolase activity, hydrolyzing O-glycosyl compounds" evidence=IEA;ISS] [GO:0005975 "carbohydrate metabolic process" evidence=IEA;ISS] [GO:0005618 "cell wall" evidence=IDA] [GO:0009505 "plant-type cell wall" evidence=IDA] [GO:0016020 "membrane" evidence=IDA] [GO:0005829 "cytosol" evidence=RCA] [GO:0009506 "plasmodesma" evidence=IDA] InterPro:IPR001764 InterPro:IPR002772 Pfam:PF00933 Pfam:PF01915 PRINTS:PR00133 GO:GO:0009506 EMBL:CP002688 GO:GO:0016020 InterPro:IPR017853 SUPFAM:SSF51445 GO:GO:0005975 GO:GO:0004553 GO:GO:0009505 Gene3D:G3DSA:3.40.50.1700 Gene3D:G3DSA:3.20.20.300 SUPFAM:SSF52279 CAZy:GH3 HSSP:Q9XEI3 EMBL:AF462808 EMBL:AY091027 EMBL:AY142679 IPI:IPI00528669 RefSeq:NP_197595.2 RefSeq:NP_851048.1 UniGene:At.23560 ProteinModelPortal:Q8W112 SMR:Q8W112 PRIDE:Q8W112 EnsemblPlants:AT5G20950.1 EnsemblPlants:AT5G20950.2 GeneID:832220 KEGG:ath:AT5G20950 TAIR:At5g20950 InParanoid:Q8W112 OMA:THNGINE PhylomeDB:Q8W112 ProtClustDB:CLSN2690178 ArrayExpress:Q8W112 Genevestigator:Q8W112 Uniprot:Q8W112
Root	Isotig08368	1	13	-3.829	0.000362856	TAIR|locus:2147117 - symbol:AT5G20950 species:3702 "Arabidopsis thaliana" [GO:0004553 "hydrolase activity, hydrolyzing O-glycosyl compounds" evidence=IEA;ISS] [GO:0005975 "carbohydrate metabolic process" evidence=IEA;ISS] [GO:0005618 "cell wall" evidence=IDA] [GO:0009505 "plant-type cell wall" evidence=IDA] [GO:0016020 "membrane" evidence=IDA] [GO:0005829 "cytosol" evidence=RCA] [GO:0009506 "plasmodesma" evidence=IDA] InterPro:IPR001764 InterPro:IPR002772 Pfam:PF00933 Pfam:PF01915 PRINTS:PR00133

Tissue	Identification	# of reads (stress)	# of reads (control)	Fold Change	p-value	Description
						GO:GO:0009506 EMBL:CP002688 GO:GO:0016020 InterPro:IPR017853 SUPFAM:SSF51445 GO:GO:0005975 GO:GO:0004553 GO:GO:0009505 Gene3D:G3DSA:3.40.50.1700 Gene3D:G3DSA:3.20.20.300 SUPFAM:SSF52279 CAZy:GH3 HSSP:Q9XEI3 EMBL:AF462808 EMBL:AY091027 EMBL:AY142679 IPI:IPI00528669 RefSeq:NP_197595.2 RefSeq:NP_851048.1 UniGene:At.23560 ProteinModelPortal:Q8W112 SMR:Q8W112 PRIDE:Q8W112 EnsemblPlants:AT5G20950.1 EnsemblPlants:AT5G20950.2 GeneID:832220 KEGG:ath:AT5G20950 TAIR:At5g20950 InParanoid:Q8W112 OMA:THNGINE PhylomeDB:Q8W112 ProtClustDB:CLSN2690178 ArrayExpress:Q8W112 Genevestigator:Q8W112 Uniprot:Q8W112
Root	Isotig08373	19	0	5.119	1.05E-05	TAIR|locus:2025966 - symbol:E1 ALPHA "pyruvate dehydrogenase complex E1 alpha subunit" species:3702 "Arabidopsis thaliana" [GO:0004739 "pyruvate dehydrogenase (acetyl-transferring) activity" evidence=IEA;ISS] [GO:0008152 "metabolic process" evidence=ISS] [GO:0016624 "oxidoreductase activity, acting on the aldehyde or oxo group of donors, disulfide as acceptor" evidence=IEA] [GO:0005739 "mitochondrion" evidence=IDA] [GO:0046686 "response to cadmium ion" evidence=IEP] [GO:0005634 "nucleus" evidence=IDA] [GO:0005829 "cytosol" evidence=IDA] InterPro:IPR001017 InterPro:IPR017597 Pfam:PF00676 EMBL:CP002684 GenomeReviews:CT485782_GR GO:GO:0005829 GO:GO:0005634 GO:GO:0046686 GO:GO:0005759 EMBL:AC007258 GO:GO:0006096 GO:GO:0004739 eggNOG:COG1071 HOGENOM:HBG753263 EMBL:U21214 EMBL:AF360306 EMBL:BT000974 EMBL:AY087667 IPI:IPI00531318 PIR:B96623 PIR:JC4358 RefSeq:NP_176198.1 UniGene:At.23186 ProteinModelPortal:P52901 SMR:P52901 IntAct:P52901 STRING:P52901 SWISS-2DPAGE:P52901 PRIDE:P52901 EnsemblPlants:AT1G59900.1 GeneID:842284 KEGG:ath:AT1G59900 GeneFarm:4372 TAIR:At1g59900 InParanoid:P52901 KO:K00161 OMA:IVENNRY PhylomeDB:P52901 ProtClustDB:PLN02269 ArrayExpress:P52901 Genevestigator:P52901 GermOnline:AT1G59900 TIGRFAMs:TIGR03182 Uniprot:P52901
Root	Isotig08385	214	0	8.612	5.02E-42	TAIR|locus:2025346 - symbol:LEA14 "AT1G01470" species:3702 "Arabidopsis thaliana" [GO:0003674 "molecular_function" evidence=ND] [GO:0009269 "response to desiccation" evidence=ISS] [GO:0009793 "embryo development ending in seed dormancy" evidence=ISS] [GO:0009644 "response to high light intensity" evidence=IEP] [GO:0009611 "response to wounding" evidence=IEP] [GO:0050832 "defense response to fungus" evidence=IEP] [GO:0005829 "cytosol" evidence=IDA] InterPro:IPR013990 SMART:SM00769 InterPro:IPR004864 EMBL:CP002684 GenomeReviews:CT485782_GR GO:GO:0005829 InterPro:IPR013783 Gene3D:G3DSA:2.60.40.10 EMBL:Y12776 EMBL:AC061957 GO:GO:0009611 GO:GO:0050832 GO:GO:0009644 GO:GO:0009269 EMBL:Y10085 EMBL:AY074855 EMBL:BT015111 EMBL:Z37258 IPI:IPI00525938 PIR:D86145 RefSeq:NP_171654.1 UniGene:At.25113 PDB:1XO8 PDBsum:1XO8 ProteinModelPortal:O03983 SMR:O03983 IntAct:O03983 STRING:O03983 PRIDE:O03983 DNASU:837071 EnsemblPlants:AT1G01470.1 GeneID:837071 KEGG:ath:AT1G01470 TAIR:At1g01470 eggNOG:NOG271766 HOGENOM:HBG320411 InParanoid:O03983 OMA:YSHSIPI PhylomeDB:O03983 ProtClustDB:CLSN2682861 Genevestigator:O03983 GermOnline:AT1G01470 Pfam:PF03168 Uniprot:O03983
Root	Isotig08386	215	0	8.619	3.50E-42	TAIR|locus:2025346 - symbol:LEA14 "AT1G01470" species:3702 "Arabidopsis thaliana" [GO:0003674 "molecular_function" evidence=ND] [GO:0009269 "response to desiccation" evidence=ISS] [GO:0009793 "embryo development ending in seed dormancy" evidence=ISS] [GO:0009644 "response to high light intensity" evidence=IEP] [GO:0009611 "response to wounding" evidence=IEP] [GO:0050832 "defense response to fungus" evidence=IEP] [GO:0005829 "cytosol" evidence=IDA] InterPro:IPR013990 SMART:SM00769 InterPro:IPR004864 EMBL:CP002684 GenomeReviews:CT485782_GR GO:GO:0005829 InterPro:IPR013783 Gene3D:G3DSA:2.60.40.10 EMBL:Y12776 EMBL:AC061957 GO:GO:0009611 GO:GO:0050832 GO:GO:0009644 GO:GO:0009269 EMBL:Y10085 EMBL:AY074855 EMBL:BT015111 EMBL:Z37258 IPI:IPI00525938 PIR:D86145 RefSeq:NP_171654.1 UniGene:At.25113 PDB:1XO8 PDBsum:1XO8 ProteinModelPortal:O03983 SMR:O03983 IntAct:O03983 STRING:O03983 PRIDE:O03983 DNASU:837071 EnsemblPlants:AT1G01470.1 GeneID:837071 KEGG:ath:AT1G01470 TAIR:At1g01470 eggNOG:NOG271766 HOGENOM:HBG320411 InParanoid:O03983 OMA:YSHSIPI PhylomeDB:O03983 ProtClustDB:CLSN2682861 Genevestigator:O03983 GermOnline:AT1G01470 Pfam:PF03168 Uniprot:O03983
Root	Isotig08411	16	2	2.871	0.000812389	No hit
Root	Isotig08412	17	0	4.958	3.30E-05	TAIR|locus:2154930 - symbol:RAB18 "AT5G66400" species:3702 "Arabidopsis thaliana" [GO:0003674 "molecular_function" evidence=ND] [GO:0005575 "cellular_component" evidence=ND] [GO:0009737 "response to abscisic acid stimulus" evidence=IEP] [GO:0009631 "cold acclimation" evidence=IGI] [GO:0009961 "response to 1-aminocyclopropane-1-carboxylic acid" evidence=IEP] [GO:0009414 "response to water deprivation" evidence=IEP]

Tissue	Identification	# of reads (stress)	# of reads (control)	Fold Change	p-value	Description
						[GO:0006950 "response to stress" evidence=IEP] InterPro:IPR000167 Pfam:PF00257 PROSITE:PS00315 PROSITE:PS00823 GO:GO:0009737 EMBL:CP002688 GenomeReviews:BA000015_GR GO:GO:0009414 GO:GO:0009631 EMBL:AB013389 EMBL:X68042 EMBL:L04173 EMBL:AF428458 EMBL:AY093779 EMBL:BT002226 IPI:IPI00540524 PIR:S28021 RefSeq:NP_201441.1 UniGene:At.20221 UniGene:At.74882 STRING:P30185 PRIDE:P30185 EnsemblPlants:AT5G66400.1 GeneID:836772 KEGG:ath:AT5G66400 TAIR:At5g66400 eggNOG:NOG291924 InParanoid:P30185 OMA:MGTHGTT PhylomeDB:P30185 ProtClustDB:CLSN2686697 ArrayExpress:P30185 Genevestigator:P30185 GO:GO:0009961 Uniprot:P30185
Root	Isotig08431	27	4	2.626	3.31E-05	TAIR|locus:2119682 - symbol:AT4G21580 species:3702 "Arabidopsis thaliana" [GO:0003824 "catalytic activity" evidence=IEA] [GO:0008152 "metabolic process" evidence=IEA] [GO:0008270 "zinc ion binding" evidence=IEA;ISS] [GO:0016491 "oxidoreductase activity" evidence=IEA] [GO:0055114 "oxidation-reduction process" evidence=IEA] [GO:0005829 "cytosol" evidence=IDA] InterPro:IPR002085 InterPro:IPR013149 InterPro:IPR013154 InterPro:IPR016040 Pfam:PF00107 Pfam:PF08240 GO:GO:0005829 EMBL:CP002687 GenomeReviews:CT486007_GR GO:GO:0000166 Gene3D:G3DSA:3.40.50.720 GO:GO:0008270 GO:GO:0016491 HOGENOM:HBG753318 InterPro:IPR011032 PANTHER:PTHR11695 SUPFAM:SSF50129 EMBL:AL035527 EMBL:AL161555 EMBL:AL022603 OMA:IPEVWLT InterPro:IPR014189 PANTHER:PTHR11695:SF28 TIGRFAMs:TIGR02824 HSSP:Q8L3C8 EMBL:AF411784 EMBL:AY088237 EMBL:AY124814 IPI:IPI00539765 PIR:T05166 RefSeq:NP_193889.1 UniGene:At.2271 ProteinModelPortal:O65423 SMR:O65423 PRIDE:O65423 EnsemblPlants:AT4G21580.1 GeneID:828243 KEGG:ath:AT4G21580 TAIR:At4g21580 InParanoid:O65423 PhylomeDB:O65423 ProtClustDB:CLSN2685583 ArrayExpress:O65423 Genevestigator:O65423 Uniprot:O65423
Root	Isotig08432	27	4	2.626	3.31E-05	TAIR|locus:2119682 - symbol:AT4G21580 species:3702 "Arabidopsis thaliana" [GO:0003824 "catalytic activity" evidence=IEA] [GO:0008152 "metabolic process" evidence=IEA] [GO:0008270 "zinc ion binding" evidence=IEA;ISS] [GO:0016491 "oxidoreductase activity" evidence=IEA] [GO:0055114 "oxidation-reduction process" evidence=IEA] [GO:0005829 "cytosol" evidence=IDA] InterPro:IPR002085 InterPro:IPR013149 InterPro:IPR013154 InterPro:IPR016040 Pfam:PF00107 Pfam:PF08240 GO:GO:0005829 EMBL:CP002687 GenomeReviews:CT486007_GR GO:GO:0000166 Gene3D:G3DSA:3.40.50.720 GO:GO:0008270 GO:GO:0016491 HOGENOM:HBG753318 InterPro:IPR011032 PANTHER:PTHR11695 SUPFAM:SSF50129 EMBL:AL035527 EMBL:AL161555 EMBL:AL022603 OMA:IPEVWLT InterPro:IPR014189 PANTHER:PTHR11695:SF28 TIGRFAMs:TIGR02824 HSSP:Q8L3C8 EMBL:AF411784 EMBL:AY088237 EMBL:AY124814 IPI:IPI00539765 PIR:T05166 RefSeq:NP_193889.1 UniGene:At.2271 ProteinModelPortal:O65423 SMR:O65423 PRIDE:O65423 EnsemblPlants:AT4G21580.1 GeneID:828243 KEGG:ath:AT4G21580 TAIR:At4g21580 InParanoid:O65423 PhylomeDB:O65423 ProtClustDB:CLSN2685583 ArrayExpress:O65423 Genevestigator:O65423 Uniprot:O65423
Root	Isotig08447	7	85	-3.731	1.62E-19	TAIR|locus:2117939 - symbol:PIP1;5 "AT4G23400" species:3702 "Arabidopsis thaliana" [GO:0006810 "transport" evidence=ISS] [GO:0015250 "water channel activity" evidence=ISS] [GO:0016020 "membrane" evidence=ISS] [GO:0005886 "plasma membrane" evidence=IDA] [GO:0009506 "plasmodesma" evidence=IDA] InterPro:IPR000425 InterPro:IPR012269 InterPro:IPR022357 Pfam:PF00230 PRINTS:PR00783 PROSITE:PS00221 GO:GO:0016021 GO:GO:0005886 GO:GO:0009506 EMBL:CP002687 GenomeReviews:CT486007_GR EMBL:AL161559 GO:GO:0055085 GO:GO:0005215 GO:GO:0006833 EMBL:AL031326 InterPro:IPR023271 Gene3D:G3DSA:1.20.1080.10 SUPFAM:SSF81338 eggNOG:COG0580 HOGENOM:HBG705794 PANTHER:PTHR19139 TIGRFAMs:TIGR00861 KO:K09872 ProtClustDB:CLSN2682869 EMBL:AY059948 EMBL:AY081593 EMBL:AY087945 IPI:IPI00536498 PIR:T05378 RefSeq:NP_194071.1 UniGene:At.2438 ProteinModelPortal:Q8LAA6 SMR:Q8LAA6 IntAct:Q8LAA6 STRING:Q8LAA6 PRIDE:Q8LAA6 EnsemblPlants:AT4G23400.1 GeneID:828439 KEGG:ath:AT4G23400 GeneFarm:4865 TAIR:At4g23400 InParanoid:Q8LAA6 OMA:YDFLLAP PhylomeDB:Q8LAA6 ArrayExpress:Q8LAA6 Genevestigator:Q8LAA6 GermOnline:AT4G23400 Uniprot:Q8LAA6
Root	Isotig08448	7	85	-3.731	1.62E-19	TAIR|locus:2117939 - symbol:PIP1;5 "AT4G23400" species:3702 "Arabidopsis thaliana" [GO:0006810 "transport" evidence=ISS] [GO:0015250 "water channel activity" evidence=ISS] [GO:0016020 "membrane" evidence=ISS] [GO:0005886 "plasma membrane" evidence=IDA] [GO:0009506 "plasmodesma" evidence=IDA] InterPro:IPR000425 InterPro:IPR012269 InterPro:IPR022357 Pfam:PF00230 PRINTS:PR00783 PROSITE:PS00221 GO:GO:0016021 GO:GO:0005886 GO:GO:0009506 EMBL:CP002687 GenomeReviews:CT486007_GR EMBL:AL161559 GO:GO:0055085 GO:GO:0005215 GO:GO:0006833 EMBL:AL031326 InterPro:IPR023271 Gene3D:G3DSA:1.20.1080.10 SUPFAM:SSF81338 eggNOG:COG0580 HOGENOM:HBG705794 PANTHER:PTHR19139 TIGRFAMs:TIGR00861 KO:K09872 ProtClustDB:CLSN2682869 EMBL:AY059948

Tissue	Identification	# of reads (stress)	# of reads (control)	Fold Change	p-value	Description
						EMBL:AY081593 EMBL:AY087945 IPI:IPI00536498 PIR:T05378 RefSeq:NP_194071.1 UniGene:At.2438 ProteinModelPortal:Q8LAA6 SMR:Q8LAA6 IntAct:Q8LAA6 STRING:Q8LAA6 PRIDE:Q8LAA6 EnsemblPlants:AT4G23400.1 GeneID:828439 KEGG:ath:AT4G23400 GeneFarm:4865 TAIR:At4g23400 InParanoid:Q8LAA6 OMA:YDFLLAP PhylomeDB:Q8LAA6 ArrayExpress:Q8LAA6 Genevestigator:Q8LAA6 GermOnline:AT4G23400 Uniprot:Q8LAA6
Root	Isotig08449	0	11	-4.588	0.000569522	No hit
Root	Isotig08450	0	11	-4.588	0.000569522	No hit
Root	Isotig08461	4	25	-2.773	1.71E-05	TAIR|locus:2077502 - symbol:AT3G07720 "AT3G07720" species:3702 "Arabidopsis thaliana" [GO:0003674 "molecular_function" evidence=ND] [GO:0005634 "nucleus" evidence=IDA] [GO:0005737 "cytoplasm" evidence=IDA] [GO:0010043 "response to zinc ion" evidence=IEP] [GO:0005829 "cytosol" evidence=IDA] InterPro:IPR006652 InterPro:IPR015915 Pfam:PF01344 GO:GO:0005829 GO:GO:0005634 EMBL:CP002686 GenomeReviews:BA000014_GR GO:GO:0010043 Gene3D:G3DSA:2.120.10.80 EMBL:AC013483 EMBL:AC009176 HOGENOM:HBG329174 ProtClustDB:CLSN2688154 EMBL:AF324668 EMBL:AF329503 EMBL:AY039559 EMBL:AY113872 EMBL:BT000850 IPI:IPI00543372 RefSeq:NP_566316.1 UniGene:At.18342 ProteinModelPortal:Q9S7W4 SMR:Q9S7W4 IntAct:Q9S7W4 STRING:Q9S7W4 PRIDE:Q9S7W4 ProMEX:Q9S7W4 DNASU:819963 EnsemblPlants:AT3G07720.1 GeneID:819963 KEGG:ath:AT3G07720 TAIR:At3g07720 eggNOG:NOG293724 InParanoid:Q9S7W4 OMA:DDIFFFT PhylomeDB:Q9S7W4 ArrayExpress:Q9S7W4 Genevestigator:Q9S7W4 Uniprot:Q9S7W4
Root	Isotig08462	3	25	-3.188	4.22E-06	TAIR|locus:2077502 - symbol:AT3G07720 "AT3G07720" species:3702 "Arabidopsis thaliana" [GO:0003674 "molecular_function" evidence=ND] [GO:0005634 "nucleus" evidence=IDA] [GO:0005737 "cytoplasm" evidence=IDA] [GO:0010043 "response to zinc ion" evidence=IEP] [GO:0005829 "cytosol" evidence=IDA] InterPro:IPR006652 InterPro:IPR015915 Pfam:PF01344 GO:GO:0005829 GO:GO:0005634 EMBL:CP002686 GenomeReviews:BA000014_GR GO:GO:0010043 Gene3D:G3DSA:2.120.10.80 EMBL:AC013483 EMBL:AC009176 HOGENOM:HBG329174 ProtClustDB:CLSN2688154 EMBL:AF324668 EMBL:AF329503 EMBL:AY039559 EMBL:AY113872 EMBL:BT000850 IPI:IPI00543372 RefSeq:NP_566316.1 UniGene:At.18342 ProteinModelPortal:Q9S7W4 SMR:Q9S7W4 IntAct:Q9S7W4 STRING:Q9S7W4 PRIDE:Q9S7W4 ProMEX:Q9S7W4 DNASU:819963 EnsemblPlants:AT3G07720.1 GeneID:819963 KEGG:ath:AT3G07720 TAIR:At3g07720 eggNOG:NOG293724 InParanoid:Q9S7W4 OMA:DDIFFFT PhylomeDB:Q9S7W4 ArrayExpress:Q9S7W4 Genevestigator:Q9S7W4 Uniprot:Q9S7W4
Root	Isotig08465	23	4	2.395	0.000276124	TAIR|locus:2153117 - symbol:AT5G51570 species:3702 "Arabidopsis thaliana" [GO:0005773 "vacuole" evidence=IDA] [GO:0005774 "vacuolar membrane" evidence=IDA] [GO:0005886 "plasma membrane" evidence=IDA] [GO:0009506 "plasmodesma" evidence=IDA] [GO:0005794 "Golgi apparatus" evidence=IDA] InterPro:IPR001972 InterPro:IPR001107 Pfam:PF01145 GO:GO:0005886 GO:GO:0009506 GO:GO:0005774 EMBL:CP002688 GenomeReviews:BA000015_GR eggNOG:COG0330 PANTHER:PTHR10264 SMART:SM00244 EMBL:AB018109 HOGENOM:HBG519201 ProtClustDB:CLSN2682581 EMBL:BT006203 EMBL:AK227961 IPI:IPI00518030 RefSeq:NP_199970.1 UniGene:At.7811 ProteinModelPortal:Q9FHM7 SMR:Q9FHM7 IntAct:Q9FHM7 STRING:Q9FHM7 PRIDE:Q9FHM7 EnsemblPlants:AT5G51570.1 GeneID:835231 KEGG:ath:AT5G51570 TAIR:At5g51570 InParanoid:Q9FHM7 OMA:SIEHILM PhylomeDB:Q9FHM7 Genevestigator:Q9FHM7 Uniprot:Q9FHM7
Root	Isotig08466	23	4	2.395	0.000276124	TAIR|locus:2153117 - symbol:AT5G51570 species:3702 "Arabidopsis thaliana" [GO:0005773 "vacuole" evidence=IDA] [GO:0005774 "vacuolar membrane" evidence=IDA] [GO:0005886 "plasma membrane" evidence=IDA] [GO:0009506 "plasmodesma" evidence=IDA] [GO:0005794 "Golgi apparatus" evidence=IDA] InterPro:IPR001972 InterPro:IPR001107 Pfam:PF01145 GO:GO:0005886 GO:GO:0009506 GO:GO:0005774 EMBL:CP002688 GenomeReviews:BA000015_GR eggNOG:COG0330 PANTHER:PTHR10264 SMART:SM00244 EMBL:AB018109 HOGENOM:HBG519201 ProtClustDB:CLSN2682581 EMBL:BT006203 EMBL:AK227961 IPI:IPI00518030 RefSeq:NP_199970.1 UniGene:At.7811 ProteinModelPortal:Q9FHM7 SMR:Q9FHM7 IntAct:Q9FHM7 STRING:Q9FHM7 PRIDE:Q9FHM7 EnsemblPlants:AT5G51570.1 GeneID:835231 KEGG:ath:AT5G51570 TAIR:At5g51570 InParanoid:Q9FHM7 OMA:SIEHILM PhylomeDB:Q9FHM7 Genevestigator:Q9FHM7 Uniprot:Q9FHM7
Root	Isotig08467	4	37	-3.338	1.13E-08	No hit
Root	Isotig08468	3	33	-3.588	2.85E-08	No hit

Tissue	Identification	# of reads (stress)	# of reads (control)	Fold Change	p-value	Description
Root	Isotig08501	46	1	5.395	5.60E-12	TAIR|locus:2084598 - symbol:AT3G22850 "AT3G22850" species:3702 "Arabidopsis thaliana" [GO:0003674 "molecular_function" evidence=ND] [GO:0005886 "plasma membrane" evidence=IDA] [GO:0005634 "nucleus" evidence=IDA] [GO:0005829 "cytosol" evidence=IDA] [GO:0009737 "response to abscisic acid stimulus" evidence=IDA] GO:GO:0005829 GO:GO:0005886 GO:GO:0009737 GO:GO:0005634 EMBL:CP002686 GenomeReviews:BA000014_GR EMBL:AP001300 eggNOG:COG0367 InterPro:IPR024286 Pfam:PF12481 HOGENOM:HBG605462 ProtClustDB:CLSN2684172 EMBL:AY072385 EMBL:AY114623 IPI:IPI00535884 RefSeq:NP_188925.1 UniGene:At.28093 UniGene:At.67702 ProteinModelPortal:Q9LIL3 SMR:Q9LIL3 PRIDE:Q9LIL3 ProMEX:Q9LIL3 EnsemblPlants:AT3G22850.1 GeneID:821857 KEGG:ath:AT3G22850 TAIR:At3g22850 InParanoid:Q9LIL3 OMA:EVTIVIE PhylomeDB:Q9LIL3 ArrayExpress:Q9LIL3 Genevestigator:Q9LIL3 Uniprot:Q9LIL3
Root	Isotig08502	47	1	5.426	3.25E-12	TAIR|locus:2084598 - symbol:AT3G22850 "AT3G22850" species:3702 "Arabidopsis thaliana" [GO:0003674 "molecular_function" evidence=ND] [GO:0005886 "plasma membrane" evidence=IDA] [GO:0005634 "nucleus" evidence=IDA] [GO:0005829 "cytosol" evidence=IDA] [GO:0009737 "response to abscisic acid stimulus" evidence=IDA] GO:GO:0005829 GO:GO:0005886 GO:GO:0009737 GO:GO:0005634 EMBL:CP002686 GenomeReviews:BA000014_GR EMBL:AP001300 eggNOG:COG0367 InterPro:IPR024286 Pfam:PF12481 HOGENOM:HBG605462 ProtClustDB:CLSN2684172 EMBL:AY072385 EMBL:AY114623 IPI:IPI00535884 RefSeq:NP_188925.1 UniGene:At.28093 UniGene:At.67702 ProteinModelPortal:Q9LIL3 SMR:Q9LIL3 PRIDE:Q9LIL3 ProMEX:Q9LIL3 EnsemblPlants:AT3G22850.1 GeneID:821857 KEGG:ath:AT3G22850 TAIR:At3g22850 InParanoid:Q9LIL3 OMA:EVTIVIE PhylomeDB:Q9LIL3 ArrayExpress:Q9LIL3 Genevestigator:Q9LIL3 Uniprot:Q9LIL3
Root	Isotig08509	14	0	4.678	0.000190242	TAIR|locus:2129695 - symbol:CYP97B3 ""cytochrome P450, family 97, subfamily B, polypeptide 3"" species:3702 "Arabidopsis thaliana" [GO:0004497 "monooxygenase activity" evidence=IEA] [GO:0005506 "iron ion binding" evidence=IEA] [GO:0009055 "electron carrier activity" evidence=IEA] [GO:0019825 "oxygen binding" evidence=ISS] [GO:0020037 "heme binding" evidence=IEA] [GO:0055114 "oxidation-reduction process" evidence=IEA] [GO:0009570 "chloroplast stroma" evidence=IDA] [GO:0009507 "chloroplast" evidence=IDA] [GO:0009941 "chloroplast envelope" evidence=IDA] InterPro:IPR001128 InterPro:IPR002401 InterPro:IPR017972 Pfam:PF00067 PRINTS:PR00385 PRINTS:PR00463 PROSITE:PS00086 EMBL:CP002687 GenomeReviews:CT486007_GR GO:GO:0009570 GO:GO:0009055 GO:GO:0004497 GO:GO:0031969 EMBL:AL161540 eggNOG:COG2124 GO:GO:0020037 Gene3D:G3DSA:1.10.630.10 SUPFAM:SSF48264 GO:GO:0016117 GO:GO:0016705 EMBL:Z97337 EMBL:AY062675 EMBL:BT002582 IPI:IPI00532091 PIR:H71414 RefSeq:NP_193247.2 UniGene:At.27265 ProteinModelPortal:O23365 SMR:O23365 STRING:O23365 PRIDE:O23365 EnsemblPlants:AT4G15110.1 GeneID:827177 KEGG:ath:AT4G15110 GeneFarm:1346 TAIR:At4g15110 HOGENOM:HBG748119 InParanoid:O23365 OMA:ALMESTV PhylomeDB:O23365 ProtClustDB:CLSN2690929 ArrayExpress:O23365 Genevestigator:O23365 GermOnline:AT4G15110 Uniprot:O23365
Root	Isotig08511	0	12	-4.714	0.000297064	TAIR|locus:2157712 - symbol:CEP1 "cysteine endopeptidase 1" species:3702 "Arabidopsis thaliana" [GO:0004197 "cysteine-type endopeptidase activity" evidence=IEA] [GO:0006508 "proteolysis" evidence=IEA;ISS] [GO:0008234 "cysteine-type peptidase activity" evidence=IEA;ISS] InterPro:IPR000668 InterPro:IPR013128 Pfam:PF00112 PRINTS:PR00705 SMART:SM00645 InterPro:IPR000169 GO:GO:0005783 EMBL:CP002688 GenomeReviews:BA000015_GR eggNOG:COG4870 HOGENOM:HBG746690 GO:GO:0004197 GO:GO:0006508 InterPro:IPR013201 PANTHER:PTHR12411 Pfam:PF08246 SMART:SM00848 PROSITE:PS00640 PROSITE:PS00139 PROSITE:PS00639 PROSITE:PS00014 EMBL:AB024031 EMBL:HM367092 EMBL:AY091087 IPI:IPI00516991 RefSeq:NP_568722.1 UniGene:At.7918 HSSP:O65039 ProteinModelPortal:Q9FGR9 SMR:Q9FGR9 MEROPS:C01.A03 PRIDE:Q9FGR9 EnsemblPlants:AT5G50260.1 GeneID:835091 KEGG:ath:AT5G50260 TAIR:At5g50260 InParanoid:Q9FGR9 KO:K01376 OMA:WELYERW PhylomeDB:Q9FGR9 ProtClustDB:CLSN2689970 ArrayExpress:Q9FGR9 Genevestigator:Q9FGR9 Uniprot:Q9FGR9
Root	Isotig08512	0	12	-4.714	0.000297064	TAIR|locus:2152445 - symbol:SAG12 "AT5G45890" species:3702 "Arabidopsis thaliana" [GO:0006508 "proteolysis" evidence=ISS] [GO:0008234 "cysteine-type peptidase activity" evidence=ISS] [GO:0009723 "response to ethylene stimulus" evidence=IEP] [GO:0007568 "aging" evidence=IEP;TAS] [GO:0010150 "leaf senescence" evidence=TAS] [GO:0010282 "senescence-associated vacuole" evidence=IDA] [GO:0009817 "defense response to fungus, incompatible interaction" evidence=IEP] InterPro:IPR000668 InterPro:IPR013128 Pfam:PF00112 PRINTS:PR00705 SMART:SM00645 InterPro:IPR000169 EMBL:CP002688 GO:GO:0004197 GO:GO:0006508 InterPro:IPR013201 PANTHER:PTHR12411 Pfam:PF08246 SMART:SM00848 PROSITE:PS00640 PROSITE:PS00139

Tissue	Identification	# of reads (stress)	# of reads (control)	Fold Change	p-value	Description
						PROSITE:PS00639 GO:GO:0010150 GO:GO:0009817 EMBL:AB016870 HSSP:O65039 EMBL:AF370131 EMBL:AY040073 IPI:IPI00544181 RefSeq:NP_568651.1 UniGene:At.75256 UniGene:At.7710 ProteinModelPortal:Q9FJ47 SMR:Q9FJ47 IntAct:Q9FJ47 STRING:Q9FJ47 MEROPS:C01.117 PRIDE:Q9FJ47 EnsemblPlants:AT5G45890.1 GeneID:834629 KEGG:ath:AT5G45890 TAIR:At5g45890 InParanoid:Q9FJ47 OMA:NDEQALM PhylomeDB:Q9FJ47 ProtClustDB:CLSN2917735 ArrayExpress:Q9FJ47 Genevestigator:Q9FJ47 GO:GO:0010282 Uniprot:Q9FJ47
Root	Isotig08533	5	31	-2.761	1.77E-06	TAIR|locus:2117939 - symbol:PIP1;5 "AT4G23400" species:3702 "Arabidopsis thaliana" [GO:0006810 "transport" evidence=ISS] [GO:0015250 "water channel activity" evidence=ISS] [GO:0016020 "membrane" evidence=ISS] [GO:0005886 "plasma membrane" evidence=IDA] [GO:0009506 "plasmodesma" evidence=IDA] InterPro:IPR000425 InterPro:IPR012269 InterPro:IPR022357 Pfam:PF00230 PRINTS:PR00783 PROSITE:PS00221 GO:GO:0016021 GO:GO:0005886 GO:GO:0009506 EMBL:CP002687 GenomeReviews:CT486007_GR EMBL:AL161559 GO:GO:0055085 GO:GO:0005215 GO:GO:0006833 EMBL:AL031326 InterPro:IPR023271 Gene3D:G3DSA:1.20.1080.10 SUPFAM:SSF81338 eggNOG:COG0580 HOGENOM:HBG705794 PANTHER:PTHR19139 TIGRFAMs:TIGR00861 KO:K09872 ProtClustDB:CLSN2682869 EMBL:AY059948 EMBL:AY081593 EMBL:AY087945 IPI:IPI00536498 PIR:T05378 RefSeq:NP_194071.1 UniGene:At.2438 ProteinModelPortal:Q8LAA6 SMR:Q8LAA6 IntAct:Q8LAA6 STRING:Q8LAA6 PRIDE:Q8LAA6 EnsemblPlants:AT4G23400.1 GeneID:828439 KEGG:ath:AT4G23400 GeneFarm:4865 TAIR:At4g23400 InParanoid:Q8LAA6 OMA:YDFLLAP PhylomeDB:Q8LAA6 ArrayExpress:Q8LAA6 Genevestigator:Q8LAA6 GermOnline:AT4G23400 Uniprot:Q8LAA6
Root	Isotig08534	5	27	-2.562	1.85E-05	TAIR|locus:2117939 - symbol:PIP1;5 "AT4G23400" species:3702 "Arabidopsis thaliana" [GO:0006810 "transport" evidence=ISS] [GO:0015250 "water channel activity" evidence=ISS] [GO:0016020 "membrane" evidence=ISS] [GO:0005886 "plasma membrane" evidence=IDA] [GO:0009506 "plasmodesma" evidence=IDA] InterPro:IPR000425 InterPro:IPR012269 InterPro:IPR022357 Pfam:PF00230 PRINTS:PR00783 PROSITE:PS00221 GO:GO:0016021 GO:GO:0005886 GO:GO:0009506 EMBL:CP002687 GenomeReviews:CT486007_GR EMBL:AL161559 GO:GO:0055085 GO:GO:0005215 GO:GO:0006833 EMBL:AL031326 InterPro:IPR023271 Gene3D:G3DSA:1.20.1080.10 SUPFAM:SSF81338 eggNOG:COG0580 HOGENOM:HBG705794 PANTHER:PTHR19139 TIGRFAMs:TIGR00861 KO:K09872 ProtClustDB:CLSN2682869 EMBL:AY059948 EMBL:AY081593 EMBL:AY087945 IPI:IPI00536498 PIR:T05378 RefSeq:NP_194071.1 UniGene:At.2438 ProteinModelPortal:Q8LAA6 SMR:Q8LAA6 IntAct:Q8LAA6 STRING:Q8LAA6 PRIDE:Q8LAA6 EnsemblPlants:AT4G23400.1 GeneID:828439 KEGG:ath:AT4G23400 GeneFarm:4865 TAIR:At4g23400 InParanoid:Q8LAA6 OMA:YDFLLAP PhylomeDB:Q8LAA6 ArrayExpress:Q8LAA6 Genevestigator:Q8LAA6 GermOnline:AT4G23400 Uniprot:Q8LAA6
Root	Isotig08539	24	1	4.456	1.32E-06	TAIR|locus:2133945 - symbol:PP2-A1 "AT4G19840" species:3702 "Arabidopsis thaliana" [GO:0005575 "cellular_component" evidence=ND] [GO:0030246 "carbohydrate binding" evidence=ISS] [GO:0009625 "response to insect" evidence=IDA] [GO:0043394 "proteoglycan binding" evidence=IDA] EMBL:CP002687 GenomeReviews:CT486007_GR GO:GO:0009625 EMBL:AL024486 EMBL:AL161551 GO:GO:0043394 HOGENOM:HBG593491 EMBL:AY090355 EMBL:AY122906 EMBL:AY085730 IPI:IPI00545401 PIR:T04765 RefSeq:NP_193719.1 UniGene:At.24681 IntAct:O81865 STRING:O81865 PRIDE:O81865 DNASU:827728 EnsemblPlants:AT4G19840.1 GeneID:827728 KEGG:ath:AT4G19840 TAIR:At4g19840 eggNOG:NOG278951 InParanoid:O81865 OMA:NCFMLFA PhylomeDB:O81865 ProtClustDB:CLSN2915996 ArrayExpress:O81865 Genevestigator:O81865 Uniprot:O81865
Root	Isotig08540	20	1	4.193	1.37E-05	TAIR|locus:2133945 - symbol:PP2-A1 "AT4G19840" species:3702 "Arabidopsis thaliana" [GO:0005575 "cellular_component" evidence=ND] [GO:0030246 "carbohydrate binding" evidence=ISS] [GO:0009625 "response to insect" evidence=IDA] [GO:0043394 "proteoglycan binding" evidence=IDA] EMBL:CP002687 GenomeReviews:CT486007_GR GO:GO:0009625 EMBL:AL024486 EMBL:AL161551 GO:GO:0043394 HOGENOM:HBG593491 EMBL:AY090355 EMBL:AY122906 EMBL:AY085730 IPI:IPI00545401 PIR:T04765 RefSeq:NP_193719.1 UniGene:At.24681 IntAct:O81865 STRING:O81865 PRIDE:O81865 DNASU:827728 EnsemblPlants:AT4G19840.1 GeneID:827728 KEGG:ath:AT4G19840 TAIR:At4g19840 eggNOG:NOG278951 InParanoid:O81865 OMA:NCFMLFA PhylomeDB:O81865 ProtClustDB:CLSN2915996 ArrayExpress:O81865 Genevestigator:O81865 Uniprot:O81865
Root	Isotig08577	19	2	3.119	0.000146225	TAIR|locus:2080585 - symbol:PIP5K4 "AT3G56960" species:3702 "Arabidopsis thaliana" [GO:0016308 "1-phosphatidylinositol-4-phosphate 5-kinase activity" evidence=ISS;IDA] [GO:0005886 "plasma membrane"

Tissue	Identification	# of reads (stress)	# of reads (control)	Fold Change	p-value	Description
						evidence=IDA] [GO:0010118 "stomatal movement" evidence=IMP] [GO:0006897 "endocytosis" evidence=IMP] [GO:0007164 "establishment of tissue polarity" evidence=IMP] [GO:0009860 "pollen tube growth" evidence=IGI;IMP] [GO:0009827 "plant-type cell wall modification" evidence=IMP] [GO:0009846 "pollen germination" evidence=IGI] [GO:0016324 "apical plasma membrane" evidence=IDA] [GO:0044445 "cytosolic part" evidence=IDA] [GO:0046488 "phosphatidylinositol metabolic process" evidence=IDA] [GO:0090406 "pollen tube" evidence=IDA] InterPro:IPR002498 InterPro:IPR017163 Pfam:PF01504 PIRSF:PIRSF037274 PROSITE:PS51455 GO:GO:0005524 EMBL:CP002686 GenomeReviews:BA000014_GR GO:GO:0016324 GO:GO:0006897 GO:GO:0010118 GO:GO:0009860 GO:GO:0044445 eggNOG:COG4642 InterPro:IPR003409 Pfam:PF02493 SMART:SM00698 EMBL:AL138655 GO:GO:0009846 GO:GO:0009827 InterPro:IPR016034 SMART:SM00330 GO:GO:0090406 GO:GO:0016308 GO:GO:0007164 KO:K00889 InterPro:IPR023610 PANTHER:PTHR23086 HOGENOM:HBG588608 IPI:IPI00523818 PIR:T47756 RefSeq:NP_191255.1 UniGene:At.53961 ProteinModelPortal:Q9M1K2 SMR:Q9M1K2 STRING:Q9M1K2 PRIDE:Q9M1K2 ProMEX:Q9M1K2 EnsemblPlants:AT3G56960.1 GeneID:824863 KEGG:ath:AT3G56960 TAIR:At3g56960 InParanoid:Q9M1K2 OMA:SIQYDPT PhylomeDB:Q9M1K2 ProtClustDB:CLSN2683759 Genevestigator:Q9M1K2 GermOnline:AT3G56960 Uniprot:Q9M1K2
Root	Isotig08603	54	16	1.626	1.54E-05	TAIR|locus:2092492 - symbol:CYSB "AT3G12490" species:3702 "Arabidopsis thaliana" [GO:0004869 "cysteine-type endopeptidase inhibitor activity" evidence=ISS;IDA] [GO:0009628 "response to abiotic stimulus" evidence=IMP] [GO:0050897 "cobalt ion binding" evidence=IDA] [GO:0005829 "cytosol" evidence=IDA] InterPro:IPR000010 Pfam:PF00031 SMART:SM00043 GO:GO:0005829 EMBL:CP002686 GenomeReviews:BA000014_GR GO:GO:0005576 GO:GO:0006952 GO:GO:0004869 GO:GO:0050897 EMBL:AP002047 EMBL:AC069474 GO:GO:0009628 InterPro:IPR018073 PROSITE:PS00287 InterPro:IPR020381 ProDom:PD001231 HSSP:P09229 EMBL:AY085950 EMBL:AY065127 EMBL:BT001195 EMBL:AF315737 IPI:IPI00521110 IPI:IPI00657156 RefSeq:NP_566425.1 RefSeq:NP_850570.2 UniGene:At.16910 UniGene:At.48650 ProteinModelPortal:Q8H0X6 SMR:Q8H0X6 STRING:Q8H0X6 PRIDE:Q8H0X6 ProMEX:Q8H0X6 EnsemblPlants:AT3G12490.2 GeneID:820428 KEGG:ath:AT3G12490 TAIR:At3g12490 eggNOG:NOG312503 HOGENOM:HBG317433 InParanoid:Q8H0X6 OMA:FPYELLE PhylomeDB:Q8H0X6 ProtClustDB:CLSN2914970 ArrayExpress:Q8H0X6 Genevestigator:Q8H0X6 Uniprot:Q8H0X6
Root	Isotig08604	53	16	1.599	2.32E-05	TAIR|locus:2092492 - symbol:CYSB "AT3G12490" species:3702 "Arabidopsis thaliana" [GO:0004869 "cysteine-type endopeptidase inhibitor activity" evidence=ISS;IDA] [GO:0009628 "response to abiotic stimulus" evidence=IMP] [GO:0050897 "cobalt ion binding" evidence=IDA] [GO:0005829 "cytosol" evidence=IDA] InterPro:IPR000010 Pfam:PF00031 SMART:SM00043 GO:GO:0005829 EMBL:CP002686 GenomeReviews:BA000014_GR GO:GO:0005576 GO:GO:0006952 GO:GO:0004869 GO:GO:0050897 EMBL:AP002047 EMBL:AC069474 GO:GO:0009628 InterPro:IPR018073 PROSITE:PS00287 InterPro:IPR020381 ProDom:PD001231 HSSP:P09229 EMBL:AY085950 EMBL:AY065127 EMBL:BT001195 EMBL:AF315737 IPI:IPI00521110 IPI:IPI00657156 RefSeq:NP_566425.1 RefSeq:NP_850570.2 UniGene:At.16910 UniGene:At.48650 ProteinModelPortal:Q8H0X6 SMR:Q8H0X6 STRING:Q8H0X6 PRIDE:Q8H0X6 ProMEX:Q8H0X6 EnsemblPlants:AT3G12490.2 GeneID:820428 KEGG:ath:AT3G12490 TAIR:At3g12490 eggNOG:NOG312503 HOGENOM:HBG317433 InParanoid:Q8H0X6 OMA:FPYELLE PhylomeDB:Q8H0X6 ProtClustDB:CLSN2914970 ArrayExpress:Q8H0X6 Genevestigator:Q8H0X6 Uniprot:Q8H0X6
Root	Isotig08619	0	11	-4.588	0.000569522	No hit
Root	Isotig08620	0	11	-4.588	0.000569522	ZFIN|ZDB-GENE-050208-453 - symbol:si:dkeyp-34c12.2 "si:dkeyp-34c12.2" species:7955 "Danio rerio" [GO:0007165 "signal transduction" evidence=IEA] Pfam:PF00018 InterPro:IPR002110 InterPro:IPR000159 InterPro:IPR001452 PROSITE:PS50002 PROSITE:PS50088 PROSITE:PS50200 SMART:SM00248 SMART:SM00314 SMART:SM00326 ZFIN:ZDB-GENE-050208-453 GO:GO:0007165 InterPro:IPR020683 Gene3D:G3DSA:1.25.40.20 Pfam:PF12796 SUPFAM:SSF48403 PROSITE:PS50297 SUPFAM:SSF50044 GeneTree:ENSGT00600000084381 EMBL:BX321898 IPI:IPI00998785 Ensembl:ENSDART00000077117 Bgee:F1QLG0 Uniprot:F1QLG0
Root	Isotig08621	0	17	-5.216	1.26E-05	UNIPROTKB|P83304 - symbol:P83304 "Mannose/glucose-specific lectin" species:185447 "Parkia platycephala" [GO:0000771 "agglutination involved in conjugation" evidence=IDA] [GO:0005536 "glucose binding" evidence=IDA] [GO:0005537 "mannose binding" evidence=IDA] [GO:0005575 "cellular_component" evidence=ND] GO:GO:0005537 GO:GO:0005536 InterPro:IPR001229 Gene3D:G3DSA:2.100.10.30 Pfam:PF01419

Tissue	Identification	# of reads (stress)	# of reads (control)	Fold Change	p-value	Description
						SMART:SM00915 SUPFAM:SSF51101 GO:GO:0000771 PDB:1ZGR PDB:1ZGS PDBsum:1ZGR PDBsum:1ZGS ProteinModelPortal:P83304 SMR:P83304 Uniprot:P83304
Root	Isotig08622	0	17	-5.216	1.26E-05	UNIPROTKB|P83304 - symbol:P83304 "Mannose/glucose-specific lectin" species:185447 "Parkia platycephala" [GO:0000771 "agglutination involved in conjugation" evidence=IDA] [GO:0005536 "glucose binding" evidence=IDA] [GO:0005537 "mannose binding" evidence=IDA] [GO:0005575 "cellular_component" evidence=ND] GO:GO:0005537 GO:GO:0005536 InterPro:IPR001229 Gene3D:G3DSA:2.100.10.30 Pfam:PF01419 SMART:SM00915 SUPFAM:SSF51101 GO:GO:0000771 PDB:1ZGR PDB:1ZGS PDBsum:1ZGR PDBsum:1ZGS ProteinModelPortal:P83304 SMR:P83304 Uniprot:P83304
Root	Isotig08625	14	54	-2.077	9.32E-08	TAIR|locus:2101049 - symbol:CAM7 "calmodulin 7" species:3702 "Arabidopsis thaliana" [GO:0005509 "calcium ion binding" evidence=IEA;ISS] [GO:0005513 "detection of calcium ion" evidence=ISS] [GO:0019722 "calcium-mediated signaling" evidence=IGI] [GO:0005515 "protein binding" evidence=IPI] [GO:0010099 "regulation of photomorphogenesis" evidence=IMP] [GO:0005829 "cytosol" evidence=RCA] InterPro:IPR002048 InterPro:IPR011992 SMART:SM00054 Prosite:PS00018 EMBL:CP002686 GenomeReviews:BA000014_GR GO:GO:0005509 GO:GO:0005515 GO:GO:0019722 InterPro:IPR018247 InterPro:IPR018249 Gene3D:G3DSA:1.10.238.10 PROSITE:PS50222 HOGENOM:HBG746798 KO:K02183 ProtClustDB:CLSN2681765 EMBL:AF178073 EMBL:AL162691 EMBL:AY088477 IPI:IPI00533152 PIR:T47417 RefSeq:NP_189967.1 UniGene:At.23208 ProteinModelPortal:P59220 SMR:P59220 IntAct:P59220 STRING:P59220 EnsemblPlants:AT3G43810.1 GeneID:823492 KEGG:ath:AT3G43810 TAIR:At3g43810 InParanoid:P59220 PhylomeDB:P59220 ArrayExpress:P59220 Genevestigator:P59220 GermOnline:AT3G43810 GO:GO:0010099 Uniprot:P59220
Root	Isotig08626	14	51	-1.994	4.31E-07	TAIR|locus:2101049 - symbol:CAM7 "calmodulin 7" species:3702 "Arabidopsis thaliana" [GO:0005509 "calcium ion binding" evidence=IEA;ISS] [GO:0005513 "detection of calcium ion" evidence=ISS] [GO:0019722 "calcium-mediated signaling" evidence=IGI] [GO:0005515 "protein binding" evidence=IPI] [GO:0010099 "regulation of photomorphogenesis" evidence=IMP] [GO:0005829 "cytosol" evidence=RCA] InterPro:IPR002048 InterPro:IPR011992 SMART:SM00054 Prosite:PS00018 EMBL:CP002686 GenomeReviews:BA000014_GR GO:GO:0005509 GO:GO:0005515 GO:GO:0019722 InterPro:IPR018247 InterPro:IPR018249 Gene3D:G3DSA:1.10.238.10 PROSITE:PS50222 HOGENOM:HBG746798 KO:K02183 ProtClustDB:CLSN2681765 EMBL:AF178073 EMBL:AL162691 EMBL:AY088477 IPI:IPI00533152 PIR:T47417 RefSeq:NP_189967.1 UniGene:At.23208 ProteinModelPortal:P59220 SMR:P59220 IntAct:P59220 STRING:P59220 EnsemblPlants:AT3G43810.1 GeneID:823492 KEGG:ath:AT3G43810 TAIR:At3g43810 InParanoid:P59220 PhylomeDB:P59220 ArrayExpress:P59220 Genevestigator:P59220 GermOnline:AT3G43810 GO:GO:0010099 Uniprot:P59220
Root	Isotig08627	3	20	-2.866	9.17E-05	TAIR|locus:2055572 - symbol:SPX3 "AT2G45130" species:3702 "Arabidopsis thaliana" [GO:0003674 "molecular_function" evidence=ND] [GO:0005575 "cellular_component" evidence=ND] [GO:0016036 "cellular response to phosphate starvation" evidence=IEP] [GO:0080040 "positive regulation of cellular response to phosphate starvation" evidence=IMP] EMBL:CP002685 GenomeReviews:CT485783_GR GO:GO:0016036 InterPro:IPR004331 Pfam:PF03105 PROSITE:PS51382 GO:GO:0080040 EMBL:BT020235 EMBL:BT020499 IPI:IPI00520153 PIR:G84886 RefSeq:NP_182038.1 UniGene:At.50140 EnsemblPlants:AT2G45130.1 GeneID:819120 KEGG:ath:AT2G45130 TAIR:At2g45130 eggNOG:NOG290986 OMA:CGHNDEM PhylomeDB:Q5PP62 ProtClustDB:CLSN2913033 Genevestigator:Q5PP62 Uniprot:Q5PP62
Root	Isotig08635	0	13	-4.829	0.000155955	TAIR|locus:2141762 - symbol:CRK26 "cysteine-rich RLK (RECEPTOR-like protein kinase) 26" species:3702 "Arabidopsis thaliana" [GO:0006468 "protein phosphorylation" evidence=IEA] [GO:0016301 "kinase activity" evidence=ISS] InterPro:IPR000719 InterPro:IPR008271 InterPro:IPR011009 InterPro:IPR017441 Pfam:PF00069 PROSITE:PS00107 PROSITE:PS00108 PROSITE:PS50011 GO:GO:0016021 GO:GO:0005524 EMBL:CP002687 GenomeReviews:CT486007_GR eggNOG:COG0515 GO:GO:0004872 SUPFAM:SSF56112 GO:GO:0004674 HOGENOM:HBG755340 EMBL:AL161594 EMBL:AL035656 InterPro:IPR002902 Pfam:PF01657 PROSITE:PS51473 EMBL:AK228082 IPI:IPI00538329 IPI:IPI00854312 PIR:T06082 RefSeq:NP_195594.1 UniGene:At.66584 ProteinModelPortal:Q9T0J1 SMR:Q9T0J1 PRIDE:Q9T0J1 EnsemblPlants:AT4G38830.1 GeneID:830038 KEGG:ath:AT4G38830 GeneFarm:218 TAIR:At4g38830 InParanoid:Q9T0J1 OMA:HGQFSFK PhylomeDB:Q9T0J1 ProtClustDB:CLSN2915954 ArrayExpress:Q9T0J1 Genevestigator:Q9T0J1 Uniprot:Q9T0J1
Root	Isotig08636	0	13	-4.829	0.000155955	TAIR|locus:2141762 - symbol:CRK26 "cysteine-rich RLK (RECEPTOR-like protein kinase) 26" species:3702 "Arabidopsis thaliana" [GO:0006468 "protein phosphorylation" evidence=IEA] [GO:0016301 "kinase activity"

Tissue	Identification	# of reads (stress)	# of reads (control)	Fold Change	p-value	Description
						evidence=ISS] InterPro:IPR000719 InterPro:IPR008271 InterPro:IPR011009 InterPro:IPR017441 Pfam:PF00069 PROSITE:PS00107 PROSITE:PS00108 PROSITE:PS50011 GO:GO:0016021 GO:GO:0005524 EMBL:CP002687 GenomeReviews:CT486007_GR eggNOG:COG0515 GO:GO:0004872 SUPFAM:SSF56112 GO:GO:0004674 HOGENOM:HBG755340 EMBL:AL161594 EMBL:AL035656 InterPro:IPR002902 Pfam:PF01657 PROSITE:PS51473 EMBL:AK228082 IPI:IPI00538329 IPI:IPI00854312 PIR:T06082 RefSeq:NP_195594.1 UniGene:At.66584 ProteinModelPortal:Q9T0J1 SMR:Q9T0J1 PRIDE:Q9T0J1 EnsemblPlants:AT4G38830.1 GeneID:830038 KEGG:ath:AT4G38830 GeneFarm:218 TAIR:At4g38830 InParanoid:Q9T0J1 OMA:HGQFSFK PhylomeDB:Q9T0J1 ProtClustDB:CLSN2915954 ArrayExpress:Q9T0J1 Genevestigator:Q9T0J1 Uniprot:Q9T0J1
Root	Isotig08647	2	37	-4.338	4.38E-10	No hit
Root	Isotig08648	2	42	-4.521	1.92E-11	No hit
Root	Isotig08651	19	0	5.119	1.05E-05	TAIR|locus:2091762 - symbol:HSFC1 "AT3G24520" species:3702 "Arabidopsis thaliana" [GO:0003677 "DNA binding" evidence=ISS] [GO:0003700 "sequence-specific DNA binding transcription factor activity" evidence=ISS] [GO:0005634 "nucleus" evidence=IEA] [GO:0006355 "regulation of transcription, DNA-dependent" evidence=IEA] InterPro:IPR000232 Pfam:PF00447 PRINTS:PR00056 PROSITE:PS00434 SMART:SM00415 GO:GO:0005634 EMBL:CP002686 GenomeReviews:BA000014_GR GO:GO:0006950 GO:GO:0043565 GO:GO:0003700 GO:GO:0006351 InterPro:IPR011991 Gene3D:G3DSA:1.10.10.10 EMBL:AB020746 eggNOG:COG5169 HSSP:P22121 EMBL:AY056111 EMBL:AY072614 IPI:IPI00548593 RefSeq:NP_189095.1 UniGene:At.25607 UniGene:At.73128 ProteinModelPortal:Q9LV52 SMR:Q9LV52 IntAct:Q9LV52 PRIDE:Q9LV52 EnsemblPlants:AT3G24520.1 GeneID:822047 KEGG:ath:AT3G24520 GeneFarm:4086 TAIR:At3g24520 InParanoid:Q9LV52 OMA:NIARRKH PhylomeDB:Q9LV52 ProtClustDB:CLSN2722272 Genevestigator:Q9LV52 InterPro:IPR009938 Pfam:PF07334 Uniprot:Q9LV52
Root	Isotig08652	19	0	5.119	1.05E-05	TAIR|locus:2091762 - symbol:HSFC1 "AT3G24520" species:3702 "Arabidopsis thaliana" [GO:0003677 "DNA binding" evidence=ISS] [GO:0003700 "sequence-specific DNA binding transcription factor activity" evidence=ISS] [GO:0005634 "nucleus" evidence=IEA] [GO:0006355 "regulation of transcription, DNA-dependent" evidence=IEA] InterPro:IPR000232 Pfam:PF00447 PRINTS:PR00056 PROSITE:PS00434 SMART:SM00415 GO:GO:0005634 EMBL:CP002686 GenomeReviews:BA000014_GR GO:GO:0006950 GO:GO:0043565 GO:GO:0003700 GO:GO:0006351 InterPro:IPR011991 Gene3D:G3DSA:1.10.10.10 EMBL:AB020746 eggNOG:COG5169 HSSP:P22121 EMBL:AY056111 EMBL:AY072614 IPI:IPI00548593 RefSeq:NP_189095.1 UniGene:At.25607 UniGene:At.73128 ProteinModelPortal:Q9LV52 SMR:Q9LV52 IntAct:Q9LV52 PRIDE:Q9LV52 EnsemblPlants:AT3G24520.1 GeneID:822047 KEGG:ath:AT3G24520 GeneFarm:4086 TAIR:At3g24520 InParanoid:Q9LV52 OMA:NIARRKH PhylomeDB:Q9LV52 ProtClustDB:CLSN2722272 Genevestigator:Q9LV52 InterPro:IPR009938 Pfam:PF07334 Uniprot:Q9LV52
Root	Isotig08653	112	0	7.678	7.03E-25	TAIR|locus:2197823 - symbol:AT1G04560 "AT1G04560" species:3702 "Arabidopsis thaliana" [GO:0003674 "molecular_function" evidence=ND] [GO:0008150 "biological_process" evidence=ND] Pfam:PF05512 EMBL:CP002684 GenomeReviews:CT485782_GR EMBL:AC002376 EMBL:AF360343 EMBL:AY051080 IPI:IPI00537314 PIR:B86178 RefSeq:NP_563710.1 UniGene:At.10465 PRIDE:O23029 EnsemblPlants:AT1G04560.1 GeneID:839491 KEGG:ath:AT1G04560 TAIR:At1g04560 eggNOG:NOG327574 HOGENOM:HBG593249 InParanoid:O23029 OMA:VAWAITA PhylomeDB:O23029 ProtClustDB:CLSN2687672 ArrayExpress:O23029 Genevestigator:O23029 InterPro:IPR008390 Uniprot:O23029
Root	Isotig08654	108	0	7.626	3.77E-24	TAIR|locus:2197823 - symbol:AT1G04560 "AT1G04560" species:3702 "Arabidopsis thaliana" [GO:0003674 "molecular_function" evidence=ND] [GO:0008150 "biological_process" evidence=ND] Pfam:PF05512 EMBL:CP002684 GenomeReviews:CT485782_GR EMBL:AC002376 EMBL:AF360343 EMBL:AY051080 IPI:IPI00537314 PIR:B86178 RefSeq:NP_563710.1 UniGene:At.10465 PRIDE:O23029 EnsemblPlants:AT1G04560.1 GeneID:839491 KEGG:ath:AT1G04560 TAIR:At1g04560 eggNOG:NOG327574 HOGENOM:HBG593249 InParanoid:O23029 OMA:VAWAITA PhylomeDB:O23029 ProtClustDB:CLSN2687672 ArrayExpress:O23029 Genevestigator:O23029 InterPro:IPR008390 Uniprot:O23029
Root	Isotig08657	0	14	-4.936	8.24E-05	UNIPROTKB|A8QW53 - symbol:OMT3 "5-pentadecatrienyl resorcinol O-methyltransferase" species:4558 "Sorghum bicolor" [GO:0008171 "O-methyltransferase activity" evidence=IDA] [GO:0008757 "S-adenosylmethionine-dependent methyltransferase activity" evidence=IDA] [GO:0032259 "methylation" evidence=IDA] InterPro:IPR001077 InterPro:IPR012967 InterPro:IPR016461 Pfam:PF00891 Pfam:PF08100 PIRSF:PIRSF005739 InterPro:IPR011991 Gene3D:G3DSA:1.10.10.10 GO:GO:0046983 GO:GO:0008171 GO:GO:0008757 EMBL:EF189708 EMBL:CM000765 RefSeq:XP_002447425.1 UniGene:Sbi.19698 ProteinModelPortal:A8QW53

Tissue	Identification	# of reads (stress)	# of reads (control)	Fold Change	p-value	Description
						EnsemblPlants:Sb06g000820.1 GeneID:8080259 KEGG:sbi:SORBI_06g000820 eggNOG:NOG272168 PhylomeDB:A8QW53 ProtClustDB:CLSN2725062 Uniprot:A8QW53
Root	Isotig08663	4	25	-2.773	1.71E-05	TAIR|locus:2080432 - symbol:AT3G59940 "AT3G59940" species:3702 "Arabidopsis thaliana" [GO:0003674 "molecular_function" evidence=ND] [GO:0005575 "cellular_component" evidence=ND] [GO:0008150 "biological_process" evidence=ND] InterPro:IPR001810 InterPro:IPR006652 Pfam:PF01344 PROSITE:PS50181 SMART:SM00256 SMART:SM00612 GO:GO:0005634 EMBL:CP002686 GenomeReviews:BA000014_GR SUPFAM:SSF81383 InterPro:IPR015916 Gene3D:G3DSA:2.130.10.80 EMBL:AL138647 UniGene:At.1165 UniGene:At.48785 HOGENOM:HBG593750 eggNOG:NOG282025 ProtClustDB:CLSN2685021 EMBL:AY074382 EMBL:AY081325 EMBL:AY091240 EMBL:AY088575 IPI:IPI00534118 PIR:T47818 RefSeq:NP_191553.1 ProteinModelPortal:Q9M1Y1 SMR:Q9M1Y1 IntAct:Q9M1Y1 STRING:Q9M1Y1 PRIDE:Q9M1Y1 EnsemblPlants:AT3G59940.1 GeneID:825164 KEGG:ath:AT3G59940 TAIR:At3g59940 InParanoid:Q9M1Y1 OMA:CFIDSER PhylomeDB:Q9M1Y1 ArrayExpress:Q9M1Y1 Genevestigator:Q9M1Y1 Uniprot:Q9M1Y1
Root	Isotig08664	3	17	-2.631	0.00056383	TAIR|locus:2080432 - symbol:AT3G59940 "AT3G59940" species:3702 "Arabidopsis thaliana" [GO:0003674 "molecular_function" evidence=ND] [GO:0005575 "cellular_component" evidence=ND] [GO:0008150 "biological_process" evidence=ND] InterPro:IPR001810 InterPro:IPR006652 Pfam:PF01344 PROSITE:PS50181 SMART:SM00256 SMART:SM00612 GO:GO:0005634 EMBL:CP002686 GenomeReviews:BA000014_GR SUPFAM:SSF81383 InterPro:IPR015916 Gene3D:G3DSA:2.130.10.80 EMBL:AL138647 UniGene:At.1165 UniGene:At.48785 HOGENOM:HBG593750 eggNOG:NOG282025 ProtClustDB:CLSN2685021 EMBL:AY074382 EMBL:AY081325 EMBL:AY091240 EMBL:AY088575 IPI:IPI00534118 PIR:T47818 RefSeq:NP_191553.1 ProteinModelPortal:Q9M1Y1 SMR:Q9M1Y1 IntAct:Q9M1Y1 STRING:Q9M1Y1 PRIDE:Q9M1Y1 EnsemblPlants:AT3G59940.1 GeneID:825164 KEGG:ath:AT3G59940 TAIR:At3g59940 InParanoid:Q9M1Y1 OMA:CFIDSER PhylomeDB:Q9M1Y1 ArrayExpress:Q9M1Y1 Genevestigator:Q9M1Y1 Uniprot:Q9M1Y1
Root	Isotig08694	12	49	-2.159	1.86E-07	TAIR|locus:2124286 - symbol:CCoAOMT1 "caffeoyl coenzyme A O-methyltransferase 1" species:3702 "Arabidopsis thaliana" [GO:0046686 "response to cadmium ion" evidence=IEP] [GO:0009805 "coumarin biosynthetic process" evidence=IMP] [GO:0042409 "caffeoyl-CoA O-methyltransferase activity" evidence=IDA] [GO:0005829 "cytosol" evidence=TAS] InterPro:IPR002935 Pfam:PF01596 EMBL:CP002687 GenomeReviews:CT486007_GR GO:GO:0046686 GO:GO:0046872 EMBL:AL021961 EMBL:AL161584 GO:GO:0009809 GO:GO:0009805 eggNOG:COG4122 HOGENOM:HBG496808 GO:GO:0042409 PANTHER:PTHR10509 OMA:REKTGHH EMBL:AY057554 EMBL:AY062630 EMBL:AY081457 EMBL:AY143979 EMBL:AY088577 IPI:IPI00521545 PIR:T05431 RefSeq:NP_195131.1 UniGene:At.23175 ProteinModelPortal:O49499 SMR:O49499 STRING:O49499 PRIDE:O49499 EnsemblPlants:AT4G34050.1 GeneID:829551 KEGG:ath:AT4G34050 TAIR:At4g34050 InParanoid:O49499 KO:K00588 PhylomeDB:O49499 ProtClustDB:PLN02589 ArrayExpress:O49499 Genevestigator:O49499 Uniprot:O49499
Root	Isotig08703	6	34	-2.631	1.08E-06	TAIR|locus:2090116 - symbol:AT3G15450 "AT3G15450" species:3702 "Arabidopsis thaliana" [GO:0003674 "molecular_function" evidence=ND] EMBL:CP002686 GenomeReviews:BA000014_GR EMBL:AB022218 EMBL:AC024081 InterPro:IPR024286 Pfam:PF12481 HOGENOM:HBG605462 UniGene:At.48664 UniGene:At.25100 EMBL:AY037223 EMBL:BT000501 IPI:IPI00537203 RefSeq:NP_566513.1 ProteinModelPortal:Q9LE80 IntAct:Q9LE80 STRING:Q9LE80 PRIDE:Q9LE80 ProMEX:Q9LE80 DNASU:820784 EnsemblPlants:AT3G15450.1 GeneID:820784 KEGG:ath:AT3G15450 TAIR:At3g15450 InParanoid:Q9LE80 OMA:EANWALA PhylomeDB:Q9LE80 ProtClustDB:CLSN2917134 Genevestigator:Q9LE80 Uniprot:Q9LE80
Root	Isotig08704	6	35	-2.673	6.02E-07	TAIR|locus:2123994 - symbol:AT4G27450 "AT4G27450" species:3702 "Arabidopsis thaliana" [GO:0008150 "biological_process" evidence=ND] [GO:0005886 "plasma membrane" evidence=IDA] [GO:0005634 "nucleus" evidence=IDA] [GO:0005829 "cytosol" evidence=IDA] [GO:0009506 "plasmodesma" evidence=IDA] [GO:0005515 "protein binding" evidence=IPI] GO:GO:0005829 GO:GO:0005886 GO:GO:0009506 EMBL:CP002687 GO:GO:0005634 GO:GO:0005515 EMBL:AF375452 EMBL:AY045885 EMBL:AY091306 EMBL:AY087420 IPI:IPI00539788 RefSeq:NP_567775.1 UniGene:At.25186 UniGene:At.68765 ProteinModelPortal:Q93V62 IntAct:Q93V62 STRING:Q93V62 MEROPS:C44.A04 PRIDE:Q93V62 ProMEX:Q93V62 DNASU:828854 EnsemblPlants:AT4G27450.1 GeneID:828854 KEGG:ath:AT4G27450 TAIR:At4g27450 eggNOG:NOG245574 InParanoid:Q93V62 OMA:VYTRINS PhylomeDB:Q93V62 ProtClustDB:CLSN2917621 Genevestigator:Q93V62 InterPro:IPR024286 Pfam:PF12481 Uniprot:Q93V62
Root	Isotig08707	0	12	-4.714	0.000297064	TAIR|locus:2129116 - symbol:ERF-1 "AT4G17500" species:3702 "Arabidopsis thaliana" [GO:0003677 "DNA binding" evidence=TAS] [GO:0003700 "sequence-specific DNA binding transcription factor activity"

Tissue	Identification	# of reads (stress)	# of reads (control)	Fold Change	p-value	Description
						evidence=ISS;TAS] [GO:0005643 "nuclear pore" evidence=ISS] [GO:0006355 "regulation of transcription, DNA-dependent" evidence=IDA] [GO:0045893 "positive regulation of transcription, DNA-dependent" evidence=IDA] [GO:0010200 "response to chitin" evidence=IEP] InterPro:IPR001471 InterPro:IPR016177 Pfam:PF00847 PRINTS:PR00367 PROSITE:PS51032 SMART:SM00380 EMBL:CP002687 GenomeReviews:CT486007_GR GO:GO:0005634 GO:GO:0006952 GO:GO:0009873 GO:GO:0003677 GO:GO:0003700 GO:GO:0006351 KO:K09286 Gene3D:G3DSA:3.30.730.10 SUPFAM:SSF54171 GO:GO:0010200 EMBL:Z97343 EMBL:AL161546 HOGENOM:HBG749764 EMBL:AY058174 EMBL:AY062533 EMBL:BT002578 EMBL:AB008103 IPI:IPI00518702 PIR:A85196 PIR:T51988 RefSeq:NP_567530.4 UniGene:At.23185 UniGene:At.68141 PDB:1GCC PDB:2GCC PDB:3GCC PDBsum:1GCC PDBsum:2GCC PDBsum:3GCC ProteinModelPortal:O80337 SMR:O80337 STRING:O80337 PRIDE:O80337 EnsemblPlants:AT4G17500.1 GeneID:827464 KEGG:ath:AT4G17500 TAIR:At4g17500 eggNOG:NOG246525 InParanoid:O80337 PhylomeDB:O80337 ProtClustDB:CLSN2715394 ArrayExpress:O80337 Genevestigator:O80337 GermOnline:AT4G17500 Uniprot:O80337
Root	Isotig08708	0	11	-4.588	0.000569522	TAIR|locus:2129116 - symbol:ERF-1 "AT4G17500" species:3702 "Arabidopsis thaliana" [GO:0003677 "DNA binding" evidence=TAS] [GO:0003700 "sequence-specific DNA binding transcription factor activity" evidence=ISS;TAS] [GO:0005643 "nuclear pore" evidence=ISS] [GO:0006355 "regulation of transcription, DNA-dependent" evidence=IDA] [GO:0045893 "positive regulation of transcription, DNA-dependent" evidence=IDA] [GO:0010200 "response to chitin" evidence=IEP] InterPro:IPR001471 InterPro:IPR016177 Pfam:PF00847 PRINTS:PR00367 PROSITE:PS51032 SMART:SM00380 EMBL:CP002687 GenomeReviews:CT486007_GR GO:GO:0005634 GO:GO:0006952 GO:GO:0009873 GO:GO:0003677 GO:GO:0003700 GO:GO:0006351 KO:K09286 Gene3D:G3DSA:3.30.730.10 SUPFAM:SSF54171 GO:GO:0010200 EMBL:Z97343 EMBL:AL161546 HOGENOM:HBG749764 EMBL:AY058174 EMBL:AY062533 EMBL:BT002578 EMBL:AB008103 IPI:IPI00518702 PIR:A85196 PIR:T51988 RefSeq:NP_567530.4 UniGene:At.23185 UniGene:At.68141 PDB:1GCC PDB:2GCC PDB:3GCC PDBsum:1GCC PDBsum:2GCC PDBsum:3GCC ProteinModelPortal:O80337 SMR:O80337 STRING:O80337 PRIDE:O80337 EnsemblPlants:AT4G17500.1 GeneID:827464 KEGG:ath:AT4G17500 TAIR:At4g17500 eggNOG:NOG246525 InParanoid:O80337 PhylomeDB:O80337 ProtClustDB:CLSN2715394 ArrayExpress:O80337 Genevestigator:O80337 GermOnline:AT4G17500 Uniprot:O80337
Root	Isotig08711	3	28	-3.351	6.51E-07	TAIR|locus:2094977 - symbol:DELTA-TIP "AT3G16240" species:3702 "Arabidopsis thaliana" [GO:0006810 "transport" evidence=ISS] [GO:0015250 "water channel activity" evidence=ISS;IDA] [GO:0016020 "membrane" evidence=ISS] [GO:0009941 "chloroplast envelope" evidence=IDA] [GO:0015204 "urea transmembrane transporter activity" evidence=IGI] [GO:0015840 "urea transport" evidence=IGI] [GO:0009507 "chloroplast" evidence=IDA] [GO:0005886 "plasma membrane" evidence=IDA] [GO:0005773 "vacuole" evidence=IDA] [GO:0015200 "methylammonium transmembrane transporter activity" evidence=IDA] [GO:0051739 "ammonia transmembrane transporter activity" evidence=IGI] [GO:0005618 "cell wall" evidence=IDA] [GO:0009505 "plant-type cell wall" evidence=IDA] [GO:0005774 "vacuolar membrane" evidence=IDA] [GO:0000326 "protein storage vacuole" evidence=IDA] [GO:0042807 "central vacuole" evidence=IDA] [GO:0009506 "plasmodesma" evidence=IDA] [GO:0005794 "Golgi apparatus" evidence=IDA] [GO:0006833 "water transport" evidence=IDA] [GO:0009705 "plant-type vacuole membrane" evidence=IDA] InterPro:IPR000425 InterPro:IPR012269 InterPro:IPR022357 Pfam:PF00230 PRINTS:PR00783 PROSITE:PS00221 GO:GO:0016021 GO:GO:0005886 GO:GO:0009506 EMBL:CP002686 GenomeReviews:BA000014_GR GO:GO:0009941 EMBL:AB023046 GO:GO:0009705 GO:GO:0009505 GO:GO:0015200 GO:GO:0051739 InterPro:IPR023271 Gene3D:G3DSA:1.20.1080.10 SUPFAM:SSF81338 eggNOG:COG0580 HOGENOM:HBG705794 PANTHER:PTHR19139 TIGRFAMs:TIGR00861 GO:GO:0015250 GO:GO:0000326 UniGene:At.32719 KO:K09873 GO:GO:0042807 EMBL:U39485 EMBL:U39486 EMBL:AY065181 EMBL:AY081622 EMBL:AK229954 EMBL:AY085921 EMBL:Z18064 EMBL:Z29043 EMBL:Z29044 IPI:IPI00524268 RefSeq:NP_188245.1 UniGene:At.23571 UniGene:At.6081 UniGene:At.71329 ProteinModelPortal:Q41951 SMR:Q41951 IntAct:Q41951 STRING:Q41951 PRIDE:Q41951 EnsemblPlants:AT3G16240.1 GeneID:820870 KEGG:ath:AT3G16240 GeneFarm:2255 TAIR:At3g16240 InParanoid:Q41951 OMA:MSGNIAF PhylomeDB:Q41951 ProtClustDB:CLSN2713597 ArrayExpress:Q41951 Genevestigator:Q41951 GermOnline:AT3G16240 Uniprot:Q41951
Root	Isotig08731	0	17	-5.216	1.26E-05	TAIR|locus:2010718 - symbol:NAS4 "AT1G56430" species:3702 "Arabidopsis thaliana" [GO:0005575 "cellular_component" evidence=ND] [GO:0030410 "nicotianamine synthase activity" evidence=IGI] [GO:0030418 "nicotianamine biosynthetic process" evidence=IGI] InterPro:IPR004298 Pfam:PF03059 PROSITE:PS51142 EMBL:CP002684 GenomeReviews:CT485782_GR EMBL:AC058785 HOGENOM:HBG594037 KO:K05953

Tissue	Identification	# of reads (stress)	# of reads (control)	Fold Change	p-value	Description
						ProtClustDB:PLN03075 GO:GO:0030410 GO:GO:0030418 EMBL:AY099758 EMBL:AY128890 IPI:IPI00540066 PIR:B96606 RefSeq:NP_176038.1 UniGene:At.66920 ProteinModelPortal:Q9C7X5 SMR:Q9C7X5 IntAct:Q9C7X5 STRING:Q9C7X5 PRIDE:Q9C7X5 DNASU:842096 EnsemblPlants:AT1G56430.1 GeneID:842096 KEGG:ath:AT1G56430 TAIR:At1g56430 eggNOG:NOG305103 InParanoid:Q9C7X5 OMA:SENIQEM PhylomeDB:Q9C7X5 ArrayExpress:Q9C7X5 Genevestigator:Q9C7X5 GermOnline:AT1G56430 Uniprot:Q9C7X5
Root	Isotig08732	0	15	-5.036	4.38E-05	TAIR|locus:2010718 - symbol:NAS4 "AT1G56430" species:3702 "Arabidopsis thaliana" [GO:0005575 "cellular_component" evidence=ND] [GO:0030410 "nicotianamine synthase activity" evidence=IGI] [GO:0030418 "nicotianamine biosynthetic process" evidence=IGI] InterPro:IPR004298 Pfam:PF03059 PROSITE:PS51142 EMBL:CP002684 GenomeReviews:CT485782_GR EMBL:AC058785 HOGENOM:HBG594037 KO:K05953 ProtClustDB:PLN03075 GO:GO:0030410 GO:GO:0030418 EMBL:AY099758 EMBL:AY128890 IPI:IPI00540066 PIR:B96606 RefSeq:NP_176038.1 UniGene:At.66920 ProteinModelPortal:Q9C7X5 SMR:Q9C7X5 IntAct:Q9C7X5 STRING:Q9C7X5 PRIDE:Q9C7X5 DNASU:842096 EnsemblPlants:AT1G56430.1 GeneID:842096 KEGG:ath:AT1G56430 TAIR:At1g56430 eggNOG:NOG305103 InParanoid:Q9C7X5 OMA:SENIQEM PhylomeDB:Q9C7X5 ArrayExpress:Q9C7X5 Genevestigator:Q9C7X5 GermOnline:AT1G56430 Uniprot:Q9C7X5
Root	Isotig08741	0	25	-5.773	1.02E-07	TAIR|locus:2170214 - symbol:AT5G06730 species:3702 "Arabidopsis thaliana" [GO:0004601 "peroxidase activity" evidence=IEA;ISS] [GO:0006979 "response to oxidative stress" evidence=IEA] [GO:0020037 "heme binding" evidence=IEA] [GO:0055114 "oxidation-reduction process" evidence=IEA] [GO:0005774 "vacuolar membrane" evidence=IDA] InterPro:IPR000823 InterPro:IPR002016 InterPro:IPR010255 InterPro:IPR019793 InterPro:IPR019794 Pfam:PF00141 PRINTS:PR00458 PRINTS:PR00461 PROSITE:PS00435 PROSITE:PS00436 PROSITE:PS50873 GO:GO:0005774 EMBL:CP002688 GenomeReviews:BA000015_GR GO:GO:0005576 GO:GO:0020037 GO:GO:0004601 GO:GO:0042744 HOGENOM:HBG597790 SUPFAM:SSF48113 EMBL:AP002032 KO:K00430 eggNOG:NOG310632 ProtClustDB:CLSN2686891 EMBL:AK118827 EMBL:BT008584 EMBL:AY088509 EMBL:Y11794 IPI:IPI00528868 RefSeq:NP_196291.1 UniGene:At.90 ProteinModelPortal:Q9FG34 SMR:Q9FG34 STRING:Q9FG34 PeroxiBase:220 PRIDE:Q9FG34 EnsemblPlants:AT5G06730.1 GeneID:830562 KEGG:ath:AT5G06730 GeneFarm:1908 TAIR:At5g06730 InParanoid:Q9FG34 PhylomeDB:Q9FG34 ArrayExpress:Q9FG34 Genevestigator:Q9FG34 GermOnline:AT5G06730 Uniprot:Q9FG34
Root	Isotig08742	0	24	-5.714	1.84E-07	TAIR|locus:2057180 - symbol:AT2G38380 species:3702 "Arabidopsis thaliana" [GO:0004601 "peroxidase activity" evidence=IEA;ISS] [GO:0020037 "heme binding" evidence=IEA] [GO:0009505 "plant-type cell wall" evidence=IDA] [GO:0009651 "response to salt stress" evidence=IEP] [GO:0010043 "response to zinc ion" evidence=IEP] InterPro:IPR000823 InterPro:IPR002016 InterPro:IPR010255 InterPro:IPR019793 InterPro:IPR019794 Pfam:PF00141 PRINTS:PR00458 PRINTS:PR00461 PROSITE:PS00435 PROSITE:PS00436 PROSITE:PS50873 GO:GO:0005773 EMBL:CP002685 GenomeReviews:CT485783_GR GO:GO:0005576 GO:GO:0010043 GO:GO:0009651 GO:GO:0020037 GO:GO:0009505 EMBL:AC004683 GO:GO:0004601 GO:GO:0042744 HOGENOM:HBG597790 SUPFAM:SSF48113 KO:K00430 EMBL:M58381 EMBL:AF452388 EMBL:AY059106 EMBL:AY035033 IPI:IPI00538455 PIR:JU0458 PIR:T02507 RefSeq:NP_181372.1 UniGene:At.312 ProteinModelPortal:P24102 SMR:P24102 STRING:P24102 PeroxiBase:115 PRIDE:P24102 ProMEX:P24102 EnsemblPlants:AT2G38380.1 GeneID:818419 KEGG:ath:AT2G38380 GeneFarm:1846 TAIR:At2g38380 eggNOG:euNOG17919 InParanoid:P24102 OMA:FIDAMIR PhylomeDB:P24102 ProtClustDB:CLSN2683115 ArrayExpress:P24102 Genevestigator:P24102 GermOnline:AT2G38380 Uniprot:P24102
Root	Isotig08751	24	92	-2.068	3.64E-12	TAIR|locus:2026616 - symbol:APX1 "AT1G07890" species:3702 "Arabidopsis thaliana" [GO:0016688 "L-ascorbate peroxidase activity" evidence=ISS;IMP;TAS] [GO:0000302 "response to reactive oxygen species" evidence=IMP] [GO:0042744 "hydrogen peroxide catabolic process" evidence=IMP] [GO:0009793 "embryo development ending in seed dormancy" evidence=IMP] [GO:0009570 "chloroplast stroma" evidence=IDA] [GO:0005618 "cell wall" evidence=IDA] [GO:0046686 "response to cadmium ion" evidence=IEP] [GO:0005886 "plasma membrane" evidence=IDA] [GO:0009651 "response to salt stress" evidence=IEP] [GO:0009507 "chloroplast" evidence=IDA] [GO:0005829 "cytosol" evidence=IDA;TAS] [GO:0009506 "plasmodesma" evidence=IDA] [GO:0005794 "Golgi apparatus" evidence=IDA] [GO:0009408 "response to heat" evidence=IEP] InterPro:IPR002016 InterPro:IPR002207 InterPro:IPR010255 InterPro:IPR019793 InterPro:IPR019794 Pfam:PF00141 PRINTS:PR00458 PRINTS:PR00459 PROSITE:PS00435 PROSITE:PS00436 PROSITE:PS50873 EMBL:CP002684 GenomeReviews:CT485782_GR GO:GO:0005829 GO:GO:0005886 GO:GO:0009506 GO:GO:0009570 GO:GO:0046686 GO:GO:0005618 GO:GO:0009651 GO:GO:0009793 GO:GO:0009408 GO:GO:0020037 GO:GO:0042744 EMBL:X59600

Tissue	Identification	# of reads (stress)	# of reads (control)	Fold Change	p-value	Description
						EMBL:D14442 EMBL:U63815 EMBL:AC007583 EMBL:AY039879 EMBL:AY056395 EMBL:AY094002 EMBL:AK230096 EMBL:AY086425 IPI:IPI00536287 PIR:D86214 PIR:S20866 RefSeq:NP_001030991.2 RefSeq:NP_001030992.2 RefSeq:NP_001077482.1 RefSeq:NP_001117244.1 RefSeq:NP_172267.1 RefSeq:NP_849607.1 RefSeq:NP_973786.1 UniGene:At.47584 UniGene:At.67008 ProteinModelPortal:Q05431 SMR:Q05431 IntAct:Q05431 STRING:Q05431 PeroxiBase:1890 SWISS-2DPAGE:Q05431 PRIDE:Q05431 ProMEX:Q05431 EnsemblPlants:AT1G07890.1 EnsemblPlants:AT1G07890.2 EnsemblPlants:AT1G07890.3 EnsemblPlants:AT1G07890.4 EnsemblPlants:AT1G07890.5 EnsemblPlants:AT1G07890.7 EnsemblPlants:AT1G07890.8 GeneID:837304 KEGG:ath:AT1G07890 GeneFarm:1942 TAIR:At1g07890 eggNOG:COG0376 HOGENOM:HBG597790 InParanoid:Q05431 KO:K00434 OMA:IAEKNCA PhylomeDB:Q05431 ProtClustDB:PLN02364 ArrayExpress:Q05431 Genevestigator:Q05431 GO:GO:0016688 SUPFAM:SSF48113 Uniprot:Q05431
Root	Isotig08752	19	74	-2.090	3.44E-10	TAIR|locus:2026616 - symbol:APX1 "AT1G07890" species:3702 "Arabidopsis thaliana" [GO:0016688 "L-ascorbate peroxidase activity" evidence=ISS;IMP;TAS] [GO:0000302 "response to reactive oxygen species" evidence=IMP] [GO:0042744 "hydrogen peroxide catabolic process" evidence=IMP] [GO:0009793 "embryo development ending in seed dormancy" evidence=IMP] [GO:0009570 "chloroplast stroma" evidence=IDA] [GO:0005618 "cell wall" evidence=IDA] [GO:0046686 "response to cadmium ion" evidence=IEP] [GO:0005886 "plasma membrane" evidence=IDA] [GO:0009651 "response to salt stress" evidence=IEP] [GO:0009507 "chloroplast" evidence=IDA] [GO:0005829 "cytosol" evidence=IDA;TAS] [GO:0009506 "plasmodesma" evidence=IDA] [GO:0005794 "Golgi apparatus" evidence=IDA] [GO:0009408 "response to heat" evidence=IEP] InterPro:IPR002016 InterPro:IPR002207 InterPro:IPR010255 InterPro:IPR019793 InterPro:IPR019794 Pfam:PF00141 PRINTS:PR00458 PRINTS:PR00459 PROSITE:PS00435 PROSITE:PS00436 PROSITE:PS50873 EMBL:CP002684 GenomeReviews:CT485782_GR GO:GO:0005829 GO:GO:0005886 GO:GO:0009506 GO:GO:0009570 GO:GO:0046686 GO:GO:0005618 GO:GO:0009651 GO:GO:0009793 GO:GO:0009408 GO:GO:0020037 GO:GO:0042744 EMBL:X59600 EMBL:D14442 EMBL:U63815 EMBL:AC007583 EMBL:AY039879 EMBL:AY056395 EMBL:AY094002 EMBL:AK230096 EMBL:AY086425 IPI:IPI00536287 PIR:D86214 PIR:S20866 RefSeq:NP_001030991.2 RefSeq:NP_001030992.2 RefSeq:NP_001077482.1 RefSeq:NP_001117244.1 RefSeq:NP_172267.1 RefSeq:NP_849607.1 RefSeq:NP_973786.1 UniGene:At.47584 UniGene:At.67008 ProteinModelPortal:Q05431 SMR:Q05431 IntAct:Q05431 STRING:Q05431 PeroxiBase:1890 SWISS-2DPAGE:Q05431 PRIDE:Q05431 ProMEX:Q05431 EnsemblPlants:AT1G07890.1 EnsemblPlants:AT1G07890.2 EnsemblPlants:AT1G07890.3 EnsemblPlants:AT1G07890.4 EnsemblPlants:AT1G07890.5 EnsemblPlants:AT1G07890.7 EnsemblPlants:AT1G07890.8 GeneID:837304 KEGG:ath:AT1G07890 GeneFarm:1942 TAIR:At1g07890 eggNOG:COG0376 HOGENOM:HBG597790 InParanoid:Q05431 KO:K00434 OMA:IAEKNCA PhylomeDB:Q05431 ProtClustDB:PLN02364 ArrayExpress:Q05431 Genevestigator:Q05431 GO:GO:0016688 SUPFAM:SSF48113 Uniprot:Q05431
Root	Isotig08763	1	13	-3.829	0.000362856	TAIR|locus:2153509 - symbol:AT5G05250 "AT5G05250" species:3702 "Arabidopsis thaliana" [GO:0003674 "molecular_function" evidence=ND] [GO:0008150 "biological_process" evidence=ND] EMBL:CP002688 GenomeReviews:BA000015_GR EMBL:AB010692 EMBL:AY054291 IPI:IPI00548957 RefSeq:NP_568152.1 UniGene:At.26381 STRING:Q9FLC9 PRIDE:Q9FLC9 EnsemblPlants:AT5G05250.1 GeneID:830407 KEGG:ath:AT5G05250 TAIR:At5g05250 eggNOG:NOG288610 HOGENOM:HBG595030 InParanoid:Q9FLC9 OMA:MAYAKIG PhylomeDB:Q9FLC9 ProtClustDB:CLSN2917568 Genevestigator:Q9FLC9 Uniprot:Q9FLC9
Root	Isotig08764	0	13	-4.829	0.000155955	TAIR|locus:2153509 - symbol:AT5G05250 "AT5G05250" species:3702 "Arabidopsis thaliana" [GO:0003674 "molecular_function" evidence=ND] [GO:0008150 "biological_process" evidence=ND] EMBL:CP002688 GenomeReviews:BA000015_GR EMBL:AB010692 EMBL:AY054291 IPI:IPI00548957 RefSeq:NP_568152.1 UniGene:At.26381 STRING:Q9FLC9 PRIDE:Q9FLC9 EnsemblPlants:AT5G05250.1 GeneID:830407 KEGG:ath:AT5G05250 TAIR:At5g05250 eggNOG:NOG288610 HOGENOM:HBG595030 InParanoid:Q9FLC9 OMA:MAYAKIG PhylomeDB:Q9FLC9 ProtClustDB:CLSN2917568 Genevestigator:Q9FLC9 Uniprot:Q9FLC9
Root	Isotig08775	21	1	4.263	7.63E-06	TAIR|locus:2063130 - symbol:AT2G40390 "AT2G40390" species:3702 "Arabidopsis thaliana" [GO:0003674 "molecular_function" evidence=ND] [GO:0008150 "biological_process" evidence=ND] EMBL:CP002685 GenomeReviews:CT485783_GR EMBL:AY649297 IPI:IPI00535270 RefSeq:NP_181570.2 UniGene:At.37058 PRIDE:Q6DR18 EnsemblPlants:AT2G40390.1 GeneID:818632 KEGG:ath:AT2G40390 TAIR:At2g40390 eggNOG:NOG238598 HOGENOM:HBG591957 InParanoid:Q6DR18 OMA:LAITNWI PhylomeDB:Q6DR18 ProtClustDB:CLSN2683474 Genevestigator:Q6DR18 Uniprot:Q6DR18

Tissue	Identification	# of reads (stress)	# of reads (control)	Fold Change	p-value	Description
Root	Isotig08776	22	0	5.330	1.93E-06	No hit
Root	Isotig08827	16	2	2.871	0.000812389	TAIR|locus:2171417 - symbol:AT5G16550 "AT5G16550" species:3702 "Arabidopsis thaliana" [GO:0003674 "molecular_function" evidence=ND] [GO:0008150 "biological_process" evidence=ND] EMBL:CP002688 EMBL:AB005242 UniGene:At.66763 IPI:IPI00527710 RefSeq:NP_568333.1 PRIDE:Q9FFC9 EnsemblPlants:AT5G16550.1 GeneID:831517 KEGG:ath:AT5G16550 TAIR:At5g16550 InParanoid:Q9FFC9 OMA:REVNIES PhylomeDB:Q9FFC9 ProtClustDB:CLSN2689715 Genevestigator:Q9FFC9 Uniprot:Q9FFC9
Root	Isotig08828	16	2	2.871	0.000812389	TAIR|locus:2171417 - symbol:AT5G16550 "AT5G16550" species:3702 "Arabidopsis thaliana" [GO:0003674 "molecular_function" evidence=ND] [GO:0008150 "biological_process" evidence=ND] EMBL:CP002688 EMBL:AB005242 UniGene:At.66763 IPI:IPI00527710 RefSeq:NP_568333.1 PRIDE:Q9FFC9 EnsemblPlants:AT5G16550.1 GeneID:831517 KEGG:ath:AT5G16550 TAIR:At5g16550 InParanoid:Q9FFC9 OMA:REVNIES PhylomeDB:Q9FFC9 ProtClustDB:CLSN2689715 Genevestigator:Q9FFC9 Uniprot:Q9FFC9
Root	Isotig08843	1	20	-4.451	3.92E-06	TAIR|locus:2141055 - symbol:MYC4 "AT4G17880" species:3702 "Arabidopsis thaliana" [GO:0003677 "DNA binding" evidence=ISS] [GO:0003700 "sequence-specific DNA binding transcription factor activity" evidence=ISS] [GO:0006355 "regulation of transcription, DNA-dependent" evidence=TAS] [GO:0009718 "anthocyanin biosynthetic process" evidence=IMP] [GO:0045893 "positive regulation of transcription, DNA-dependent" evidence=IDA] [GO:0005634 "nucleus" evidence=IDA] [GO:0006952 "defense response" evidence=IMP] [GO:0043425 "bHLH transcription factor binding" evidence=IPI] InterPro:IPR011598 Pfam:PF00010 EMBL:CP002687 GenomeReviews:CT486007_GR GO:GO:0005634 GO:GO:0045893 GO:GO:0006952 GO:GO:0003677 GO:GO:0006351 EMBL:AL161547 EMBL:AL021889 Gene3D:G3DSA:4.10.280.10 SMART:SM00353 SUPFAM:SSF47459 PROSITE:PS50888 InterPro:IPR025610 Pfam:PF14215 GO:GO:0043425 eggNOG:NOG295658 HOGENOM:HBG747745 ProtClustDB:CLSN2686023 GO:GO:0009718 EMBL:AF251689 EMBL:AK221507 IPI:IPI00540927 PIR:T05074 RefSeq:NP_193522.1 UniGene:At.28316 ProteinModelPortal:O49687 SMR:O49687 PRIDE:O49687 EnsemblPlants:AT4G17880.1 GeneID:827511 KEGG:ath:AT4G17880 TAIR:At4g17880 InParanoid:O49687 OMA:SSCERAR PhylomeDB:O49687 ArrayExpress:O49687 Genevestigator:O49687 Uniprot:O49687
Root	Isotig08844	1	22	-4.588	1.10E-06	TAIR|locus:2141055 - symbol:MYC4 "AT4G17880" species:3702 "Arabidopsis thaliana" [GO:0003677 "DNA binding" evidence=ISS] [GO:0003700 "sequence-specific DNA binding transcription factor activity" evidence=ISS] [GO:0006355 "regulation of transcription, DNA-dependent" evidence=TAS] [GO:0009718 "anthocyanin biosynthetic process" evidence=IMP] [GO:0045893 "positive regulation of transcription, DNA-dependent" evidence=IDA] [GO:0005634 "nucleus" evidence=IDA] [GO:0006952 "defense response" evidence=IMP] [GO:0043425 "bHLH transcription factor binding" evidence=IPI] InterPro:IPR011598 Pfam:PF00010 EMBL:CP002687 GenomeReviews:CT486007_GR GO:GO:0005634 GO:GO:0045893 GO:GO:0006952 GO:GO:0003677 GO:GO:0006351 EMBL:AL161547 EMBL:AL021889 Gene3D:G3DSA:4.10.280.10 SMART:SM00353 SUPFAM:SSF47459 PROSITE:PS50888 InterPro:IPR025610 Pfam:PF14215 GO:GO:0043425 eggNOG:NOG295658 HOGENOM:HBG747745 ProtClustDB:CLSN2686023 GO:GO:0009718 EMBL:AF251689 EMBL:AK221507 IPI:IPI00540927 PIR:T05074 RefSeq:NP_193522.1 UniGene:At.28316 ProteinModelPortal:O49687 SMR:O49687 PRIDE:O49687 EnsemblPlants:AT4G17880.1 GeneID:827511 KEGG:ath:AT4G17880 TAIR:At4g17880 InParanoid:O49687 OMA:SSCERAR PhylomeDB:O49687 ArrayExpress:O49687 Genevestigator:O49687 Uniprot:O49687
Root	Isotig08857	1	211	-7.850	9.79E-49	TAIR|locus:2116987 - symbol:TIP1;3 "AT4G01470" species:3702 "Arabidopsis thaliana" [GO:0006810 "transport" evidence=ISS] [GO:0015250 "water channel activity" evidence=ISS;IDA] [GO:0016020 "membrane" evidence=ISS] [GO:0006833 "water transport" evidence=IDA] [GO:0015204 "urea transmembrane transporter activity" evidence=IDA] [GO:0015840 "urea transport" evidence=IDA] InterPro:IPR000425 InterPro:IPR022357 Pfam:PF00230 PRINTS:PR00783 PROSITE:PS00221 GO:GO:0016021 EMBL:CP002687 GenomeReviews:CT486007_GR GO:GO:0005774 GO:GO:0006950 InterPro:IPR023271 Gene3D:G3DSA:1.20.1080.10 SUPFAM:SSF81338 HOGENOM:HBG705794 PANTHER:PTHR19139 GO:GO:0015250 EMBL:AF096370 EMBL:AL161492 GO:GO:0015204 KO:K09873 ProtClustDB:PLN00027 EMBL:DQ446793 EMBL:DQ653172 IPI:IPI00524657 PIR:T01947 RefSeq:NP_192056.1 UniGene:At.65315 ProteinModelPortal:O82598 SMR:O82598 STRING:O82598 TCDB:1.A.8.10.6 PRIDE:O82598 EnsemblPlants:AT4G01470.1 GeneID:828051 KEGG:ath:AT4G01470 GeneFarm:4856 TAIR:At4g01470 eggNOG:NOG312454 InParanoid:O82598 OMA:IGSNGHE PhylomeDB:O82598 ArrayExpress:O82598 Genevestigator:O82598 Uniprot:O82598

Tissue	Identification	# of reads (stress)	# of reads (control)	Fold Change	p-value	Description
Root	Isotig08858	1	204	-7.801	2.22E-47	TAIR|locus:2116987 - symbol:TIP1;3 "AT4G01470" species:3702 "Arabidopsis thaliana" [GO:0006810 "transport" evidence=ISS] [GO:0015250 "water channel activity" evidence=ISS;IDA] [GO:0016020 "membrane" evidence=ISS] [GO:0006833 "water transport" evidence=IDA] [GO:0015204 "urea transmembrane transporter activity" evidence=IDA] [GO:0015840 "urea transport" evidence=IDA] InterPro:IPR000425 InterPro:IPR022357 Pfam:PF00230 PRINTS:PR00783 PROSITE:PS00221 GO:GO:0016021 EMBL:CP002687 GenomeReviews:CT486007_GR GO:GO:0005774 GO:GO:0006950 InterPro:IPR023271 Gene3D:G3DSA:1.20.1080.10 SUPFAM:SSF81338 HOGENOM:HBG705794 PANTHER:PTHR19139 GO:GO:0015250 EMBL:AF096370 EMBL:AL161492 GO:GO:0015204 KO:K09873 ProtClustDB:PLN00027 EMBL:DQ446793 EMBL:DQ653172 IPI:IPI00524657 PIR:T01947 RefSeq:NP_192056.1 UniGene:At.65315 ProteinModelPortal:O82598 SMR:O82598 STRING:O82598 TCDB:1.A.8.10.6 PRIDE:O82598 EnsemblPlants:AT4G01470.1 GeneID:828051 KEGG:ath:AT4G01470 GeneFarm:4856 TAIR:At4g01470 eggNOG:NOG312454 InParanoid:O82598 OMA:IGSNGHE PhylomeDB:O82598 ArrayExpress:O82598 Genevestigator:O82598 Uniprot:O82598
Root	Isotig08877	29	4	2.729	1.12E-05	TAIR|locus:2063104 - symbol:FER4 "AT2G40300" species:3702 "Arabidopsis thaliana" [GO:0006826 "iron ion transport" evidence=IGI;ISS] [GO:0006879 "cellular iron ion homeostasis" evidence=ISS] [GO:0008199 "ferric iron binding" evidence=IEA;ISS] [GO:0016491 "oxidoreductase activity" evidence=IEA] [GO:0046914 "transition metal ion binding" evidence=IEA] [GO:0009507 "chloroplast" evidence=ISS;IDA] [GO:0010039 "response to iron ion" evidence=IEP] [GO:0009941 "chloroplast envelope" evidence=IDA] [GO:0009570 "chloroplast stroma" evidence=IDA] [GO:0000302 "response to reactive oxygen species" evidence=IGI] [GO:0009908 "flower development" evidence=IGI] [GO:0015979 "photosynthesis" evidence=IGI] [GO:0048366 "leaf development" evidence=IGI] [GO:0055072 "iron ion homeostasis" evidence=IGI] [GO:0005739 "mitochondrion" evidence=IDA] [GO:0071214 "cellular response to abiotic stimulus" evidence=IMP] InterPro:IPR001519 InterPro:IPR008331 InterPro:IPR009078 Pfam:PF00210 GO:GO:0005739 GO:GO:0009570 EMBL:CP002685 GenomeReviews:CT485783_GR GO:GO:0009941 GO:GO:0006879 EMBL:AF085279 GO:GO:0010039 eggNOG:COG1528 HOGENOM:HBG750128 GO:GO:0008199 GO:GO:0004322 GO:GO:0006826 InterPro:IPR009040 InterPro:IPR012347 Gene3D:G3DSA:1.20.1260.10 PANTHER:PTHR11431 SUPFAM:SSF47240 PROSITE:PS50905 EMBL:AC007020 UniGene:At.27228 InterPro:IPR014034 PROSITE:PS00540 PROSITE:PS00204 KO:K00522 ProtClustDB:CLSN2683400 EMBL:AJ312191 EMBL:AY062795 EMBL:AY081615 IPI:IPI00534160 PIR:G84827 RefSeq:NP_181559.1 UniGene:At.69019 ProteinModelPortal:Q9S756 SMR:Q9S756 STRING:Q9S756 PRIDE:Q9S756 EnsemblPlants:AT2G40300.1 GeneID:818622 KEGG:ath:AT2G40300 TAIR:At2g40300 InParanoid:Q9S756 OMA:AMAYHFD PhylomeDB:Q9S756 ArrayExpress:Q9S756 Genevestigator:Q9S756 GermOnline:AT2G40300 Uniprot:Q9S756
Root	Isotig08883	1	30	-5.036	7.50E-09	TAIR|locus:2084066 - symbol:PPa4 "AT3G53620" species:3702 "Arabidopsis thaliana" [GO:0004427 "inorganic diphosphatase activity" evidence=IDA] [GO:0008152 "metabolic process" evidence=ISS] [GO:0016020 "membrane" evidence=ISS] [GO:0046686 "response to cadmium ion" evidence=IEP] [GO:0005829 "cytosol" evidence=IDA] HAMAP:MF_00209 InterPro:IPR008162 Pfam:PF00719 PROSITE:PS00387 GO:GO:0005829 GO:GO:0046686 EMBL:CP002686 GO:GO:0000287 EMBL:AL132966 GO:GO:0006796 KO:K01507 GO:GO:0004427 Gene3D:G3DSA:3.90.80.10 PANTHER:PTHR10286 SUPFAM:SSF50324 ProtClustDB:PLN02373 OMA:PNDFNVI HSSP:O59570 EMBL:AK226578 IPI:IPI00524492 PIR:T45902 RefSeq:NP_190930.1 UniGene:At.19820 ProteinModelPortal:Q9LFF9 SMR:Q9LFF9 STRING:Q9LFF9 PRIDE:Q9LFF9 EnsemblPlants:AT3G53620.1 GeneID:824530 KEGG:ath:AT3G53620 TAIR:At3g53620 InParanoid:Q9LFF9 PhylomeDB:Q9LFF9 Genevestigator:Q9LFF9 Uniprot:Q9LFF9
Root	Isotig08884	1	25	-4.773	1.66E-07	TAIR|locus:2084066 - symbol:PPa4 "AT3G53620" species:3702 "Arabidopsis thaliana" [GO:0004427 "inorganic diphosphatase activity" evidence=IDA] [GO:0008152 "metabolic process" evidence=ISS] [GO:0016020 "membrane" evidence=ISS] [GO:0046686 "response to cadmium ion" evidence=IEP] [GO:0005829 "cytosol" evidence=IDA] HAMAP:MF_00209 InterPro:IPR008162 Pfam:PF00719 PROSITE:PS00387 GO:GO:0005829 GO:GO:0046686 EMBL:CP002686 GO:GO:0000287 EMBL:AL132966 GO:GO:0006796 KO:K01507 GO:GO:0004427 Gene3D:G3DSA:3.90.80.10 PANTHER:PTHR10286 SUPFAM:SSF50324 ProtClustDB:PLN02373 OMA:PNDFNVI HSSP:O59570 EMBL:AK226578 IPI:IPI00524492 PIR:T45902 RefSeq:NP_190930.1 UniGene:At.19820 ProteinModelPortal:Q9LFF9 SMR:Q9LFF9 STRING:Q9LFF9 PRIDE:Q9LFF9 EnsemblPlants:AT3G53620.1 GeneID:824530 KEGG:ath:AT3G53620 TAIR:At3g53620 InParanoid:Q9LFF9 PhylomeDB:Q9LFF9 Genevestigator:Q9LFF9 Uniprot:Q9LFF9

Tissue	Identification	# of reads (stress)	# of reads (control)	Fold Change	p-value	Description
Root	Isotig08887	14	1	3.678	0.000484579	No hit
Root	Isotig08888	13	1	3.571	0.000882483	No hit
Root	Isotig08897	0	23	-5.653	3.32E-07	TAIR|locus:2176307 - symbol:CAF1b "AT5G22250" species:3702 "Arabidopsis thaliana" [GO:0004540 "ribonuclease activity" evidence=ISS] [GO:0005634 "nucleus" evidence=IEA] [GO:0009451 "RNA modification" evidence=ISS] [GO:0000289 "nuclear-transcribed mRNA poly(A) tail shortening" evidence=IMP] [GO:0008408 "3'-5' exonuclease activity" evidence=IDA] [GO:0042742 "defense response to bacterium" evidence=IEP] InterPro:IPR006941 InterPro:IPR012337 Pfam:PF04857 GO:GO:0005634 GO:GO:0005737 EMBL:CP002688 GenomeReviews:BA000015_GR GO:GO:0042742 GO:GO:0006355 GO:GO:0046872 GO:GO:0006351 GO:GO:0003723 SUPFAM:SSF53098 eggNOG:COG5228 GO:GO:0004535 HSSP:P39008 HOGENOM:HBG748413 ProtClustDB:CLSN2684465 GO:GO:0000289 EMBL:AB007651 EMBL:AY063985 EMBL:BT001962 IPI:IPI00540783 RefSeq:NP_197617.1 UniGene:At.27479 ProteinModelPortal:Q9FMS6 SMR:Q9FMS6 STRING:Q9FMS6 PRIDE:Q9FMS6 DNASU:832285 EnsemblPlants:AT5G22250.1 GeneID:832285 KEGG:ath:AT5G22250 TAIR:At5g22250 InParanoid:Q9FMS6 OMA:HIMRFCE PhylomeDB:Q9FMS6 ArrayExpress:Q9FMS6 Genevestigator:Q9FMS6 Uniprot:Q9FMS6
Root	Isotig08898	0	24	-5.714	1.84E-07	TAIR|locus:2176307 - symbol:CAF1b "AT5G22250" species:3702 "Arabidopsis thaliana" [GO:0004540 "ribonuclease activity" evidence=ISS] [GO:0005634 "nucleus" evidence=IEA] [GO:0009451 "RNA modification" evidence=ISS] [GO:0000289 "nuclear-transcribed mRNA poly(A) tail shortening" evidence=IMP] [GO:0008408 "3'-5' exonuclease activity" evidence=IDA] [GO:0042742 "defense response to bacterium" evidence=IEP] InterPro:IPR006941 InterPro:IPR012337 Pfam:PF04857 GO:GO:0005634 GO:GO:0005737 EMBL:CP002688 GenomeReviews:BA000015_GR GO:GO:0042742 GO:GO:0006355 GO:GO:0046872 GO:GO:0006351 GO:GO:0003723 SUPFAM:SSF53098 eggNOG:COG5228 GO:GO:0004535 HSSP:P39008 HOGENOM:HBG748413 ProtClustDB:CLSN2684465 GO:GO:0000289 EMBL:AB007651 EMBL:AY063985 EMBL:BT001962 IPI:IPI00540783 RefSeq:NP_197617.1 UniGene:At.27479 ProteinModelPortal:Q9FMS6 SMR:Q9FMS6 STRING:Q9FMS6 PRIDE:Q9FMS6 DNASU:832285 EnsemblPlants:AT5G22250.1 GeneID:832285 KEGG:ath:AT5G22250 TAIR:At5g22250 InParanoid:Q9FMS6 OMA:HIMRFCE PhylomeDB:Q9FMS6 ArrayExpress:Q9FMS6 Genevestigator:Q9FMS6 Uniprot:Q9FMS6
Root	Isotig08904	1	19	-4.377	7.43E-06	TAIR|locus:2079522 - symbol:AT3G62040 species:3702 "Arabidopsis thaliana" [GO:0016787 "hydrolase activity" evidence=IEA;ISS] InterPro:IPR006402 EMBL:CP002686 GenomeReviews:BA000014_GR InterPro:IPR023214 Gene3D:G3DSA:3.40.50.1000 SUPFAM:SSF56784 GO:GO:0016787 eggNOG:COG1011 TIGRFAMs:TIGR01509 EMBL:AL138642 UniGene:At.24742 UniGene:At.48797 InterPro:IPR010237 TIGRFAMs:TIGR01993 EMBL:BT010899 IPI:IPI00520634 PIR:T47996 RefSeq:NP_191763.3 ProteinModelPortal:Q9M262 SMR:Q9M262 PRIDE:Q9M262 EnsemblPlants:AT3G62040.1 GeneID:825377 KEGG:ath:AT3G62040 TAIR:At3g62040 HOGENOM:HBG410732 InParanoid:Q9M262 OMA:HAFNTIC PhylomeDB:Q9M262 ProtClustDB:CLSN2681592 Genevestigator:Q9M262 Uniprot:Q9M262
Root	Isotig08905	0	11	-4.588	0.000569522	TAIR|locus:2170081 - symbol:AT5G50140 "AT5G50140" species:3702 "Arabidopsis thaliana" [GO:0005575 "cellular_component" evidence=ND] [GO:0008150 "biological_process" evidence=ND] InterPro:IPR002110 PROSITE:PS50088 SMART:SM00248 EMBL:CP002688 GenomeReviews:BA000015_GR InterPro:IPR020683 Gene3D:G3DSA:1.25.40.20 Pfam:PF12796 SUPFAM:SSF48403 PROSITE:PS50297 EMBL:AB026650 HSSP:Q60778 eggNOG:KOG0504 IPI:IPI00530282 RefSeq:NP_199825.1 UniGene:At.55468 ProteinModelPortal:Q9FG97 SMR:Q9FG97 PRIDE:Q9FG97 EnsemblPlants:AT5G50140.1 GeneID:835079 KEGG:ath:AT5G50140 TAIR:At5g50140 HOGENOM:HBG750443 InParanoid:Q9FG97 OMA:ISHAGEM PhylomeDB:Q9FG97 Genevestigator:Q9FG97 Uniprot:Q9FG97
Root	Isotig08913	0	13	-4.829	0.000155955	TAIR|locus:2199297 - symbol:ENDO 2 "AT1G68290" species:3702 "Arabidopsis thaliana" [GO:0003676 "nucleic acid binding" evidence=ISS] [GO:0004519 "endonuclease activity" evidence=ISS] [GO:0006308 "DNA catabolic process" evidence=IEA;ISS] [GO:0000014 "single-stranded DNA specific endodeoxyribonuclease activity" evidence=IDA] [GO:0043765 "T/G mismatch-specific endonuclease activity" evidence=IDA] InterPro:IPR003154 InterPro:IPR008947 Pfam:PF02265 EMBL:CP002684 GO:GO:0003676 EMBL:AC016447 GO:GO:0004519 GO:GO:0006308 Gene3D:G3DSA:1.10.575.10 SUPFAM:SSF48537 HSSP:P24289 ProtClustDB:CLSN2681945 IPI:IPI00543025 PIR:E96706 RefSeq:NP_176996.1 UniGene:At.35544 UniGene:At.43330 ProteinModelPortal:Q9C9G4 SMR:Q9C9G4 PRIDE:Q9C9G4 EnsemblPlants:AT1G68290.1 GeneID:843158 KEGG:ath:AT1G68290 TAIR:At1g68290 InParanoid:Q9C9G4 OMA:AACDWAY PhylomeDB:Q9C9G4 Genevestigator:Q9C9G4 Uniprot:Q9C9G4

Tissue	Identification	# of reads (stress)	# of reads (control)	Fold Change	p-value	Description
Root	Isotig08925	1	12	-3.714	0.000699736	TAIR|locus:2025421 - symbol:AT1G01490 "AT1G01490" species:3702 "Arabidopsis thaliana" [GO:0005575 "cellular_component" evidence=ND] [GO:0030001 "metal ion transport" evidence=IEA] [GO:0046872 "metal ion binding" evidence=IEA;ISS] InterPro:IPR006121 Pfam:PF00403 EMBL:CP002684 GO:GO:0046872 GO:GO:0030001 EMBL:AY063805 EMBL:AY091276 EMBL:Y10084 IPI:IPI00534476 RefSeq:NP_001030928.1 RefSeq:NP_171656.1 UniGene:At.27454 ProteinModelPortal:O03982 PRIDE:O03982 EnsemblPlants:AT1G01490.1 EnsemblPlants:AT1G01490.2 GeneID:839440 KEGG:ath:AT1G01490 TAIR:At1g01490 eggNOG:NOG282717 InParanoid:O03982 OMA:YYAQSIE PhylomeDB:O03982 ProtClustDB:CLSN2912783 ArrayExpress:O03982 Genevestigator:O03982 Uniprot:O03982
Root	Isotig08926	1	12	-3.714	0.000699736	TAIR|locus:2025421 - symbol:AT1G01490 "AT1G01490" species:3702 "Arabidopsis thaliana" [GO:0005575 "cellular_component" evidence=ND] [GO:0030001 "metal ion transport" evidence=IEA] [GO:0046872 "metal ion binding" evidence=IEA;ISS] InterPro:IPR006121 Pfam:PF00403 EMBL:CP002684 GO:GO:0046872 GO:GO:0030001 EMBL:AY063805 EMBL:AY091276 EMBL:Y10084 IPI:IPI00534476 RefSeq:NP_001030928.1 RefSeq:NP_171656.1 UniGene:At.27454 ProteinModelPortal:O03982 PRIDE:O03982 EnsemblPlants:AT1G01490.1 EnsemblPlants:AT1G01490.2 GeneID:839440 KEGG:ath:AT1G01490 TAIR:At1g01490 eggNOG:NOG282717 InParanoid:O03982 OMA:YYAQSIE PhylomeDB:O03982 ProtClustDB:CLSN2912783 ArrayExpress:O03982 Genevestigator:O03982 Uniprot:O03982
Root	Isotig08939	105	0	7.585	1.34E-23	TAIR|locus:2061151 - symbol:NRPD1B "AT2G40030" species:3702 "Arabidopsis thaliana" [GO:0003677 "DNA binding" evidence=ISS] [GO:0003899 "DNA-directed RNA polymerase activity" evidence=ISS] [GO:0005634 "nucleus" evidence=ISS;IDA] [GO:0006351 "transcription, DNA-dependent" evidence=ISS] [GO:0000418 "DNA-directed RNA polymerase IV complex" evidence=IPI] [GO:0006306 "DNA methylation" evidence=IMP] [GO:0000419 "DNA-directed RNA polymerase V complex" evidence=IDA;IPI] [GO:0030880 "RNA polymerase complex" evidence=IPI] [GO:0035194 "posttranscriptional gene silencing by RNA" evidence=IMP] [GO:0005515 "protein binding" evidence=IPI] [GO:0005730 "nucleolus" evidence=IDA] [GO:0016604 "nuclear body" evidence=IDA] [GO:0030422 "production of siRNA involved in RNA interference" evidence=IGI;IMP] InterPro:IPR000722 InterPro:IPR006592 InterPro:IPR007066 InterPro:IPR007080 InterPro:IPR007081 InterPro:IPR015801 Pfam:PF00623 Pfam:PF04983 Pfam:PF04997 Pfam:PF04998 SMART:SM00663 EMBL:CP002685 GenomeReviews:CT485783_GR GO:GO:0005515 GO:GO:0005730 GO:GO:0006355 GO:GO:0003677 GO:GO:0005507 GO:GO:0048038 GO:GO:0009308 Gene3D:G3DSA:3.10.450.40 GO:GO:0030422 GO:GO:0006306 GO:GO:0016604 EMBL:AF002109 GO:GO:0003899 eggNOG:COG0086 GO:GO:0000418 InterPro:IPR021602 Pfam:PF11523 EMBL:AY826516 EMBL:AY927744 EMBL:DQ020656 IPI:IPI00537496 PIR:D84824 PIR:E84824 RefSeq:NP_181532.2 UniGene:At.37106 ProteinModelPortal:Q5D869 SMR:Q5D869 DIP:DIP-48678N IntAct:Q5D869 STRING:Q5D869 PRIDE:Q5D869 EnsemblPlants:AT2G40030.1 GeneID:818591 KEGG:ath:AT2G40030 TAIR:At2g40030 InParanoid:Q5D869 OMA:EGHFGYI PhylomeDB:Q5D869 ProtClustDB:CLSN2681712 ArrayExpress:O04206 Genevestigator:Q5D869 GO:GO:0000419 Uniprot:Q5D869
Root	Isotig08940	104	0	7.571	2.05E-23	TAIR|locus:2061151 - symbol:NRPD1B "AT2G40030" species:3702 "Arabidopsis thaliana" [GO:0003677 "DNA binding" evidence=ISS] [GO:0003899 "DNA-directed RNA polymerase activity" evidence=ISS] [GO:0005634 "nucleus" evidence=ISS;IDA] [GO:0006351 "transcription, DNA-dependent" evidence=ISS] [GO:0000418 "DNA-directed RNA polymerase IV complex" evidence=IPI] [GO:0006306 "DNA methylation" evidence=IMP] [GO:0000419 "DNA-directed RNA polymerase V complex" evidence=IDA;IPI] [GO:0030880 "RNA polymerase complex" evidence=IPI] [GO:0035194 "posttranscriptional gene silencing by RNA" evidence=IMP] [GO:0005515 "protein binding" evidence=IPI] [GO:0005730 "nucleolus" evidence=IDA] [GO:0016604 "nuclear body" evidence=IDA] [GO:0030422 "production of siRNA involved in RNA interference" evidence=IGI;IMP] InterPro:IPR000722 InterPro:IPR006592 InterPro:IPR007066 InterPro:IPR007080 InterPro:IPR007081 InterPro:IPR015801 Pfam:PF00623 Pfam:PF04983 Pfam:PF04997 Pfam:PF04998 SMART:SM00663 EMBL:CP002685 GenomeReviews:CT485783_GR GO:GO:0005515 GO:GO:0005730 GO:GO:0006355 GO:GO:0003677 GO:GO:0005507 GO:GO:0048038 GO:GO:0009308 Gene3D:G3DSA:3.10.450.40 GO:GO:0030422 GO:GO:0006306 GO:GO:0016604 EMBL:AF002109 GO:GO:0003899 eggNOG:COG0086 GO:GO:0000418 InterPro:IPR021602 Pfam:PF11523 EMBL:AY826516 EMBL:AY927744 EMBL:DQ020656 IPI:IPI00537496 PIR:D84824 PIR:E84824 RefSeq:NP_181532.2 UniGene:At.37106 ProteinModelPortal:Q5D869 SMR:Q5D869 DIP:DIP-48678N IntAct:Q5D869 STRING:Q5D869 PRIDE:Q5D869 EnsemblPlants:AT2G40030.1 GeneID:818591 KEGG:ath:AT2G40030 TAIR:At2g40030 InParanoid:Q5D869 OMA:EGHFGYI PhylomeDB:Q5D869 ProtClustDB:CLSN2681712 ArrayExpress:O04206 Genevestigator:Q5D869 GO:GO:0000419 Uniprot:Q5D869

Tissue	Identification	# of reads (stress)	# of reads (control)	Fold Change	p-value	Description
[truncated: 1,325,756 more chars]
