# Supplementary material for: Gene expression analysis reveals important pathways for drought response in leaves and roots of a wheat cultivar adapted to rainfed cropping in the Cerrado biome
Source: Genet Mol Biol. 2016 Oct 20;39(4):629–45. doi: 10.1590/1678-4685-GMB-2015-0327 (PMC5127152; doi:10.1590/1678-4685-GMB-2015-0327)
Supplement: Supplementary file 8 [file 1415-4757-gmb-1678-4685-GMB-2015-0327-Suppl08.pdf]

Table S4 - Details of selected candidate genes identified in the wheat cultivar MGS1 Aliança under drought stress.

Tissue	Expression	Gene	Isotig	TAIR locus*[1]	Class
Root	up	DHN1	isotigs 21944, 19917 and 22422	UNIPROTKB|A8CVF3	Dehydrin
Leaf	up	DHN1	isotigs 12334 and 12047	UNIPROTKB|A8CVF3	Dehydrin
Root	down	ERD9	isotigs 18701, 19352, 09429 and 18313	TAIR|locus:2012773 - "AT1G10370"	GST*[2]
Leaf	up	ERD9	isotigs 02218 and 02219	TAIR|locus:2012773 - "AT1G10370"	GST
Root	down	GGT1	isotigs 07897 and 07898	TAIR|locus:2135212 - "AT4G39640"	GST
Root	up and down	ATGSTF13	isotigs 04717, 04718, 04719, 04720, 14807, 14782, 18016, 09789 and 09790	TAIR|locus:2081695 - "AT3G62760"	GST
Root	up	GSH1	isotigs 04750 and 04751	TAIR|locus:2127173 - "AT4G23100"	GST
Root	down	GSTU18	isotig 09430	TAIR|locus:2012758 - "AT1G10360"	GST
Root	up and down	ATGSTF13	isotigs 04117, 04118, 04119, 04120, 14782, 18016, 09789 and 09790	TAIR|locus:2081695 - "AT3G62760"	GST
Root	down	GSTU25	isotig 16756	TAIR|locus:2020312 - "AT1G17180"	GST
Root	down	AT1G65820	isotig 21972	TAIR|locus:2018566 - "AT1G65820"	GST
Root	up	OXP1	isotigs 06621 and 06622	TAIR|locus:2156030 - "AT5G37830"	GST
Root	up	GSTL3	isotig 16495	TAIR|locus:2151326	GST
Leaf	up	GSTL3	isotig 07806	TAIR|locus:2151326	GST
Leaf	up	GR1	isotigs 00610, 00611, 00612, 00613, 00614 and 00615	TAIR|locus:2093691	GST
Leaf	up	AtLEA4-1	isotigs 04918 and 04919	TAIR|locus:2206629 - "AT1G32560"	Late embryogenesis abundant
Root	up	AtLEA4-1	isotigs 22882 and 21093	TAIR|locus:2206629 - "AT1G32560"	Late embryogenesis abundant
Root	up	LEA14	isotigs 08385, 08386 and 20485	TAIR|locus:2025346 - "AT1G01470"	Late embryogenesis abundant
Leaf	up	LEA14	isotigs 09631, 1144 and 11608	TAIR|locus:2025346 - "AT1G01470"	Late embryogenesis abundant
Leaf	up and down	LEA7	isotigs 01765, 01766, 01767 and 00921	TAIR|locus:2035109 - "AT1G52690"	Late embryogenesis abundant
Root	up	LEA7	isotigs 02966, 02968, 05172, 05173 and 05174	TAIR|locus:2035109 - "AT1G52690"	Late embryogenesis abundant
Leaf	up	LEA	isotig 11058	TAIR|locus:2050095 - "AT2G21490"	Late embryogenesis abundant
Leaf	up	LEA4-5	isotig 13697	TAIR|locus:2170239 - "AT5G06760"	Late embryogenesis abundant
Root	up	AT2G46140	isotigs 29594 and 21457	TAIR|locus:2062933 - "AT2G46140"	Late embryogenesis abundant
Root	down	ACC1	isotig 00996	TAIR|locus:2034310 - "AT1G36160"	Late embryogenesis abundant
Root	up	P5CS1	isotigs 11387 and 11306	TAIR|locus:2063907 - "AT2G39800"	Proline biosynthesis
Leaf	up	P5CS1	isotigs 01879, 01880, 018881, 02920 and 02921	TAIR|locus:2063907 - "AT2G39800"	Proline biosynthesis
Leaf	up	P5CS2	isotig 14186	TAIR|locus:2078911	Proline biosynthesis
Leaf	up	ALDH12A1	isotig 04753	TAIR|locus:2154094 - "AT5G62530"	Proline biosynthesis
Root	up	DELTA-OAT	isotig 11923	TAIR|locus:2161398 - "AT5G46180"	Proline biosynthesis
Leaf	up	DELTA-OAT	isotigs 01704, 01705, 01706 and 01707	TAIR|locus:2161398 - "AT5G46180"	Proline biosynthesis
Leaf	down	RCA	isotigs 02002, 02004, 02003, isoitg00923, 00924 and 00925	TAIR|locus:2063922 - "AT2G39730"	Leaf senescence
Root	down	SAG12	isotigs 04483, 04485, 12184, 08512, 14879, 14986 and 15605	TAIR|locus:2152445 - "AT5G45890	Leaf senescence
Root	up	SAG29	isotigs 12741, 07441 and 07442	TAIR|locus:2179867	Leaf senescence
Root	up	LTI65	isotigs 05862 and 05863	TAIR|locus:2156652 - "AT5G52300"	Leaf senescence
Leaf	up	LTI65	isotig 06036	TAIR|locus:2156652 - "AT5G52300"	Leaf senescence
Leaf	up	HAI1	isotig 03931	TAIR|locus:2168449 - "AT5G59220"	Leaf senescence
Root	down	ARF1	isotigs 06705 and 06706	TAIR|locus:2025991 - "AT1G59750"	Leaf senescence
Root	down	WRKY70	isotig 14342	TAIR|locus:2102539 - "AT3G56400"	Leaf senescence
Root	down	OPR1	isotig 15278	TAIR|locus:2030086 - "AT1G76680"	Leaf senescence
Leaf	up	NCED3	isotigs 03234 and 03235	TAIR|locus:2091652 - "AT3G14440"	Abscisic acid biosynthetic process
Root	down	AAO3	isotigs 00384, 00385, 00386 and 00387	TAIR|locus:2045149 - "AT2G27150"	Abscisic acid biosynthetic process
Root and leaf	up	OST1	isotig 03858	TAIR|locus:2118929 - "AT4G33950"	Response to abscisic acid stimulus

*[1] TAIR locus means region corresponding to a single transcription unit in the Arabidopsis genome.

*[2] GST means glutathione S-transferase and others related to glutathione biosynthesis and catabolism.
